# Identification and functional validation of *HLA-C* as a potential gene involved in colorectal cancer in the Korean population

**DOI:** 10.1186/s12864-022-08509-5

**Published:** 2022-04-04

**Authors:** Eun Bi Lim, Ho-Suk Oh, Kang Chang Kim, Moon-Ho Kim, Young Jin Kim, Bong Jo Kim, Chu Won Nho, Yoon Shin Cho

**Affiliations:** 1 Department of Biomedical Science, Hallym University, Chuncheon, Gangwon-do 24252 Republic of Korea; 2 Department of Internal Medicine, GangNeung Asan Hospital, University of Ulsan College of Medicine, Gangneung, Gangwon-do Republic of Korea; 3 Division of Genome Research, Center for Genome Science, National Institute of Health, Chungcheongbuk-do, Republic of Korea; 4 Convergence Research Center for Smart Farm Solution, Korea Institute of Science and Technology, Gangneung, Gangwon-do Republic of Korea

**Keywords:** Colorectal cancer, Exome array association analysis, Functional validation, RNA sequencing

## Abstract

**Background:**

Colorectal cancer (CRC) is the third most common cancer worldwide and is influenced by environmental and genetic factors. Although numerous genetic loci for CRC have been identified, the overall understanding of the genetic factors is yet to be elucidated. We sought to discover new genes involved in CRC applying genetic association analysis and functional study.

**Results:**

We conducted exome array analysis on 194 CRC and 600 control subjects for discovering new candidate CRC genes. Fisher’s exact test detected one exome-wide significant functional locus for CRC on *SMCO1* (*P* < 10^–6^) and two suggestive functional loci on *HLA-C* and *NUTM1* (10^–6^ ≤ *P* < 10^–4^). To evaluate the biological role of three candidate CRC genes, the differential expression of these genes between CRC and non-cancer colorectal cells was analyzed using qRT-PCR and publicly available gene expression data. Of three genes, *HLA-C* consistently revealed the significant down-regulation in CRC cells. In addition, we detected a reduction in cell viability in the *HLA-C* overexpression CRC cell line, implying the functional relevance of *HLA-C* in CRC. To understand the underlying mechanism exerted by *HLA-C* in CRC development, we conducted RNA sequencing analyses of *HLA-C* overexpression CRC cells and non-cancer colorectal cells. Pathway analysis detected that significantly down-regulated genes in *HLA-C* overexpression CRC cells were highly enriched in cancer-related signaling pathways such as JAK/STAT, ErbB, and Hedgehog signaling pathways.

**Conclusions:**

Exome array CRC case–control analysis followed by functional validation demonstrated that *HLA-C* likely exerts its influence on CRC development via cancer-related signaling pathways.

**Supplementary Information:**

The online version contains supplementary material available at 10.1186/s12864-022-08509-5.

## Background

Colorectal cancer (CRC), according to Global Cancer Incidence, Mortality and Prevalence (GLOBOCAN) 2018 (http://gco.iarc.fr), is the third most common cancer and the most prevalent type of malignant tumor in the world. In Korea, CRC has been ranked as the second most common cancer [1]. Both environmental and genetic factors influence CRC onset. It has been reported that about 12–35% of all CRCs are caused by genetic factors [2]. Epidemiological studies show that less than 6% of CRCs can be described as rare high-penetrance variants in CRC susceptibility genes identified to date, such as *APC*, *SMAD4*, *AXIN2*, *BMPR1A*, *POLD1*, *STK11*, *MUTYH,* and DNA mismatch repair genes [3, 4]. To date, 84 unique loci have been identified that are associated with CRC [5, 6]. Most variants mapping to CRC risk loci discovered through genome-wide association studies (GWAS) were found in populations with European ancestry [6–14]. Many genetic differences have been observed in diverse traits between different ethnic groups [15]. Thus, variants found in European ancestry populations show weak or no association with CRC in other ancestry populations [16].

Many studies conducted to discover CRC candidate genes have relied on GWAS. This method is based on the common disease-common variant (CD-CV) hypothesis; however, GWAS’ usefulness in determining the cause of diseases is limited. To overcome this limitation, exome sequencing or exome array analyses are recommended because these methods can identify rare variants located in exons. In this regard, employing exome sequencing or exome array methods in genetic studies likely increases the probability of discovering causal genes for target diseases or traits [17].

**Fig. 1:**
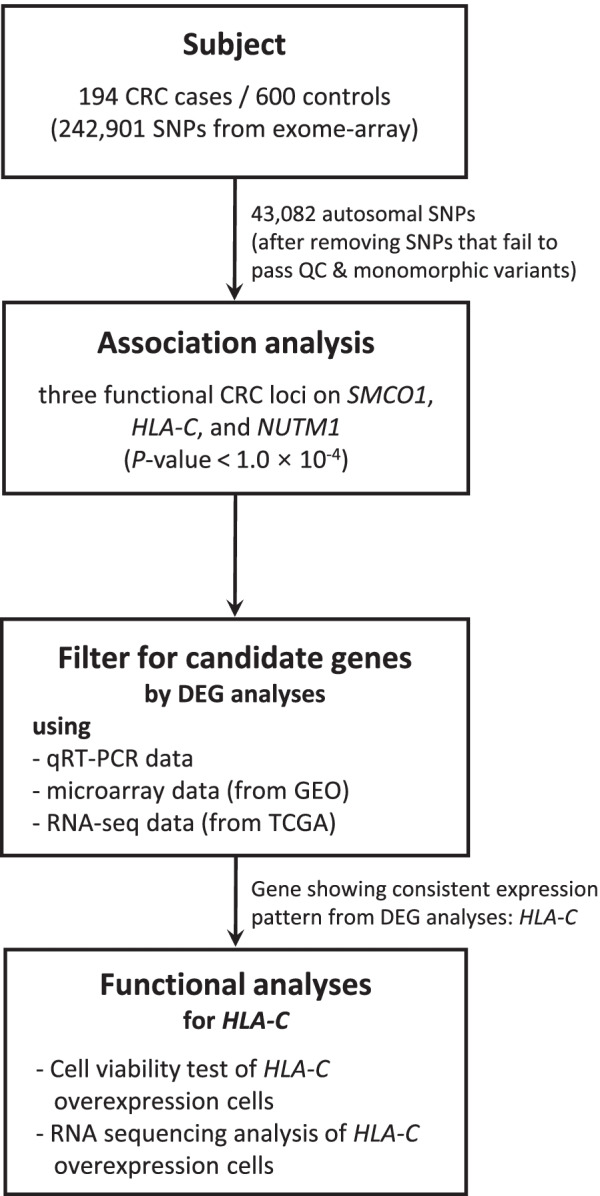
Overall study scheme. Abbreviations: CRC, colorectal cancer; SNP, single nucleotide polymorphism; QC, quality control; DEG, differentially expressed gene; qRT-PCR, quantitative real-time reverse transcription-polymerase chain reaction; GEO, gene expression omnibus

In this study, we conducted a CRC case–control study using an exome array chip (Illumina HumanExome BeadChip) to detect potential new causal genes in a Korean population. In an effort to validate CRC candidate genes identified from Fisher’s exact test of 194 cases and 600 controls, we adopted a functional validation strategy (Fig. 1). First, we compared the expression level of CRC candidate genes between CRC and non-cancer colorectal cells. Then, we generated the stable cell line that overexpresses the CRC candidate gene validated from gene expression analyses to monitor the function exerted by the overexpressed gene. Lastly, we conducted RNA sequencing (RNA-seq) of CRC candidate gene overexpression stable cells to understand the underlying mechanism and role played by the CRC candidate gene.

## Results

### Genetic association analysis of CRC and control subjects

Using genotype data resulting from the Illumina HumanExome BeadChip experiments of 194 CRC and 600 control subjects, we carried out the subject quality control. None of the subjects were excluded based on the exclusion criteria for subjects (sample call rate < 98%, heterozygosity < 25%, outliers from a multi-dimensional scaling (MDS) plot generated via identity by state distance (IBS) calculations, and indication of a cryptic first-degree relative.

Of 242,901 SNPs genotyped by Illumina HumanExome BeadChip, 230,201 autosomal SNPs remained after removing SNPs that failed to pass our inclusion criteria (SNP call rate ≥ 95%, minor allele frequency ≥ 0.001, and Hardy–Weinberg equilibrium *P*-value ≥ 1.0 X 10^–6^). Genotyping results demonstrated that 79.2%, 7.3%, 3.1%, and 10.4% of autosomal SNPs were monomorphic (minor allele frequency, MAF = 0), rare (0 < MAF < 0.01), low frequency (0.01 ≤ MAF < 0.05), and common (0.05 ≤ MAF) variants, respectively (Fig. S1). After further removing monomorphic variants, the remaining 43,082 autosomal SNPs were used for subsequent analyses.

**Fig. 2:**
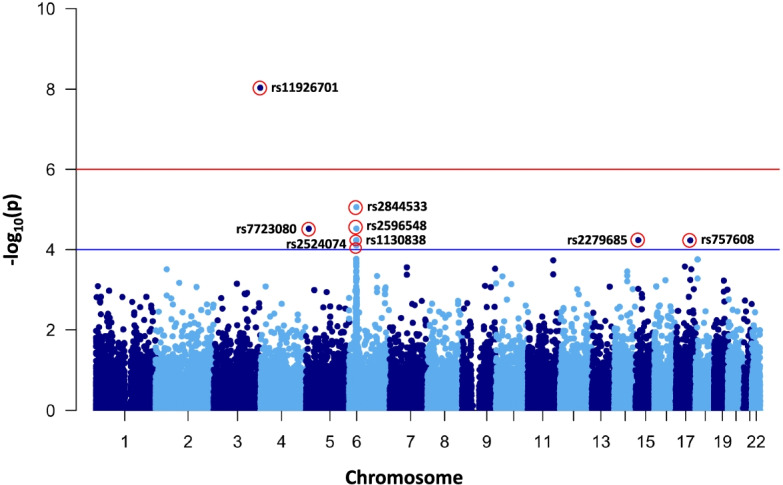
Manhattan plot of colorectal cancer case–control association analysis results. The negative logarithm of the association *P*-value for each SNP distributed in the autosomal genome is represented as a dot. The red line represents the exome-wide significant *P*-value (1.02 X 10^–6^). The green line indicates the suggestive association *P*-value (1.00 X 10^–4^)

Because a substantial portion of remaining autosomal SNPs is rare or low frequency variants, we used Fisher’s exact test in our CRC cases-controls association analysis (Table S1). We detected one exome-wide significant variant (*P* < 1.02 X 10^–6^) for CRC from Fisher’s exact test (Fig. 2 and Table 1). SNP rs11926701 (*P* = 9.23 X 10^–9^) is a missense variant located in *SMCO1* (*Single-Pass Membrane Protein with Coiled-Coli Domains 1*) which is known to promote hepatocyte proliferation and cell growth by regulating the expression of *JUN*, *MYC*, *CCND1,* and *CCNA2*. In addition, we also detected two suggestive functional variants for CRC (1.02 X 10^–6^ < *P* < 1.00 X 10^–4^) in *HLA-C* (*Major Histocompatibility Complex, Class I, C*) and *NUTM1* (*NUT Midline Carcinoma Family Member 1*) (Table 1). The encoded protein of *HLA-C* is a heavy chain receptor of the class I major histocompatibility complex (MHC). It has been known that down-regulation of MHC class I in cancer contributes to the immune evasion of cancer cells, indicating poor prognosis [18]. *NUTM1* is known to play a role in the regulation of proliferation [19]. To support findings from Fisher’s exact test, we attempted the functional validation of three CRC candidate genes in the subsequent analyses (Fig. 1).

**Table 1:** Functional loci for colorectal cancer detected from exome array association analysis

SNP ID	SNP ID	SNP ID	SNP ID	SNP ID	SNP ID	SNP ID	SNP ID
rs11926701	chr3:196,236,401	A	0.03	9.23E-09	6.70 (3.42–13.13)	*SMCO1*	missense variant
rs1130838	chr6:31,237,124	A	0.13	5.81E-05	0.45 (0.29–0.68)	*HLA-C*	missense variant
rs2279685	chr15:34,649,631	A	0.26	5.81E-05	0.56 (0.42–0.75)	*NUTM1*	missense variant

Results of Fisher's exact test showing SNPs with *P*-value < 1.0 × 10^–4^. The SNP ID and chromosomal position (BP) are based on NCBI genome build 37/hg19. Abbreviations are as follows: *SNP* single nucleotide polymorphism, *chr* chromosome, *BP* physical position (base-pair), *MA* minor allele, *MAF* minor allele frequency, *OR* Odd ratio, *CI* confidence interval

### Differential expression of mRNA levels of CRC candidate genes between CRC and non-cancer colorectal cells

**Fig. 3:**
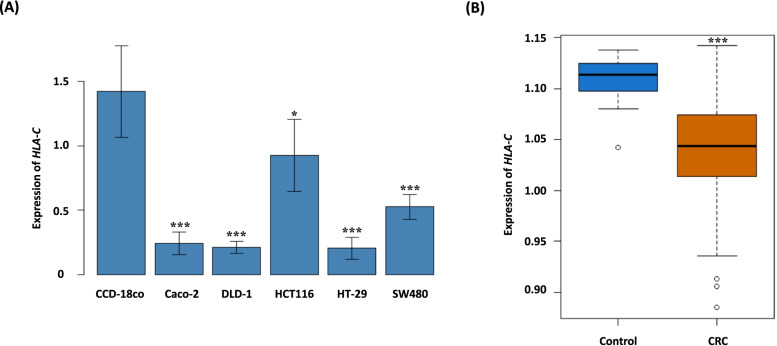
mRNA expression levels of colorectal cancer (CRC) candidate gene, *HLA-C*. **A** qRT-PCR results in the cell-line mRNA expression analyses. qRT-PCR-measured and *ACTB* normalized mRNA expression at the cell level was compared between CRC cells (i.e., Caco-2, DLD-1, HCT116, HT-29, and SW480) and non-cancer colorectal cells (CCD-18co). **B** Gene expression levels detected from online available data (NCBI GEO) on colorectal cancer and normal tissues. mRNA expression microarray data (GSE21510) were normalized with the *B2M* expression level as an internal control and compared between CRC cells and non-cancer colorectal cells. Notes: Group differences were assessed by the Wilcoxon rank-sum test. *P < 0.05, **P < 0.01, ***P < 0.001 vs control

In general, it is believed that the functional cancer-related genes are differentially expressed between cancer cells and normal cells. In this regard, we hypothesized that three candidate genes (such as *SMCO1*, *HLA-C*, and *NUTM1*) for CRC detected in exome-array association analysis are plausibly up- or down-regulated in CRC cells compared to normal colorectal cells. To test this hypothesis, we measured the expression levels of three CRC candidate genes in CRC and non-cancer colorectal cells by conducting qRT-PCR. Of these genes, only *HLA-C* consistently showed the differential expression in different CRC cell lines (such as Caco-2, DLD-1, HCT116, HT-29, and SW480) compared to non-cancer colorectal cell line (CCD-18co) (Fig. S2). The lower expression levels of *HLA-C* were evidently observed in five different CRC cell lines than in non-cancer colorectal cell line (Fig. 3A). Based on these results, we were able to narrow *HLA-C* among three genes to a potential CRC gene for the next round of functional validation analyses.

The differential expression of *HLA-C* in CRC cells was further validated via microarray analysis of 123 colorectal cancer tissue samples and 25 normal colorectal tissue samples available from NCBI GEO datasets (GEO Accession Number: GSE21510) (Fig. 3B). Microarray analysis demonstrated that the expression of *HLA-C* was significantly decreased by about 1.1 fold in CRC tissue samples (Wilcoxon rank sum test *P*-value = 2.83 X 10^–11^). The differential expression pattern of *HLA-C* was also confirmed in TCGA (The Cancer Genome Atlas) RNA sequencing (RNA-seq) datasets of 470 colorectal cancer tissue samples and 42 normal colorectal tissue samples (Wilcoxon rank sum test *P*-value = 1.73 X 10^–6^) (Fig. S2). Taken together, these results strongly imply that *HLA-C* may functionally play a role in the pathobiology of CRC.

### Generation of *HLA-C* overexpression CRC cell line and monitoring cell proliferation

**Fig. 4:**
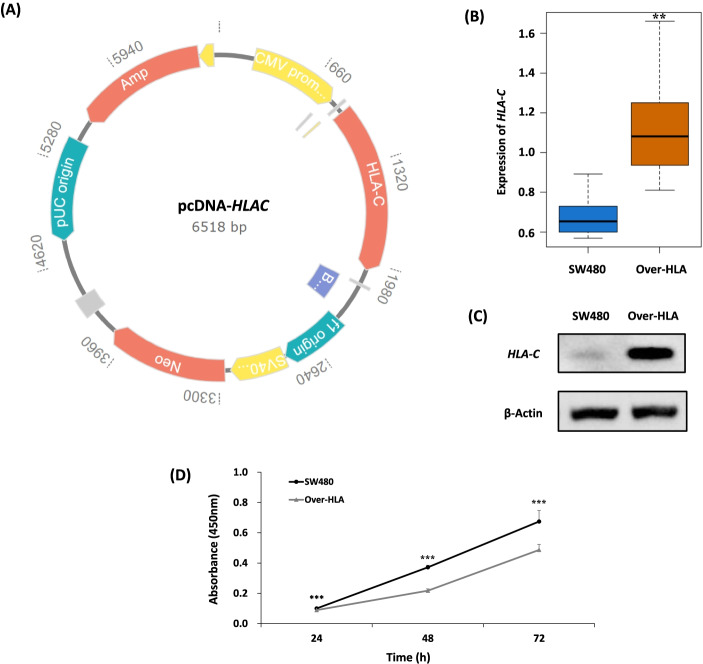
Overexpression of *HLA-C* in colorectal cancer cells (SW480). **A** Map of *HLA-C* overexpression plasmid, pcDNA-*HLAC*. **B** Relative mRNA expression levels of *HLA-C* measured by qRT-PCR in *HLA-C* overexpressing stable cells (Over-HLA) and SW480 cells. qRT-PCR-measured and *ACTB* normalized mRNA expression at the cell level was compared between Over-HLA and SW480 cells. **C** Relative protein levels of HLA-C determined by western blot in Over-HLA and SW480 cells. The blots for HLA-C and β-actin were obtained from duplicated gels using 30 µg of proteins prepared from each Over-HLA and SW480 sample. The full-length blots are presented in a Fig. S3. **D** The viability of Over-HLA and SW480 cells. Absorbance at 450 nm was measured at 24, 48, and 72 h after incubation. Notes: Group differences were assessed by the Wilcoxon rank-sum test. *P < 0.05, **P < 0.01, ***P < 0.001 vs control

Since our study demonstrated that the expression level of *HLA-C* is higher in non-cancer colorectal cells than in CRC cells (Fig. 3), we assumed that the proliferation of CRC cells is decreased by overexpressing *HLA-C* in CRC cells. To test this assumption, we generated an *HLA-C* overexpression stable cell line (Over-HLA) by transfecting an *HLA-C* overexpression plasmid (Fig. 4A) to an SW480 cell line. Increased levels of *HLA-C* expression in Over-HLA cells were detected by qRT-PCR (Fig. 4B) and western blot analyses (Fig. 4C and Fig. S3), in comparison with those in un-transfected SW480 cells. These results demonstrate that the Over-HLA cells overexpress *HLA-C*.

To understand the effect of overexpressed *HLA-C* on CRC cell proliferation, we monitored the cell viability of Over-HLA cells, as well as SW480 cells. Cell viability tests using Ez-cytox demonstrate that *HLA-C* overexpression in cancer cells reduces cell viability (Fig. 4D). This result corresponds to the reduced expression of this gene in CRC cells detected by qRT-PCR test and microarray analysis from online available data on colorectal tumor and normal tissue. Based on these findings, *HLA-C* may be involved in CRC viability by controlling cell proliferation. Furthermore, it is inferred that *HLA-C* overexpression has the potential to protect against cancer or improve prognostic outcome.

### Identification of differentially expressed genes between SW480 and *HLA-C* overexpression stable cells by RNA-seq

To understand the underlying mechanism exerted by *HLA-C* in CRC cell viability, we conducted RNA-seq analysis using SW480 and Over-HLA cells (Fig. S4). The total RNA of SW480 and Over-HLA cells was isolated, and sequencing was conducted using Illumina NextSeq 500. The reads generated from both cell lines were mapped to a reference sequence (GRCh38) and compared to detect differentially expressed genes (DEGs) between SW480 and Over-HLA cells (Table S2). The number of DEGs (adjust *P*-value [padj] < 0.001) was 6,528 (Table S3). Of these DEGs, 3,130 genes were up-regulated, and 3,398 genes were down-regulated (Fig. S5).

### Enrichment of down-regulated genes in *HLA-C* overexpression stable cells in cancer-related signaling pathways

**Fig. 5:**
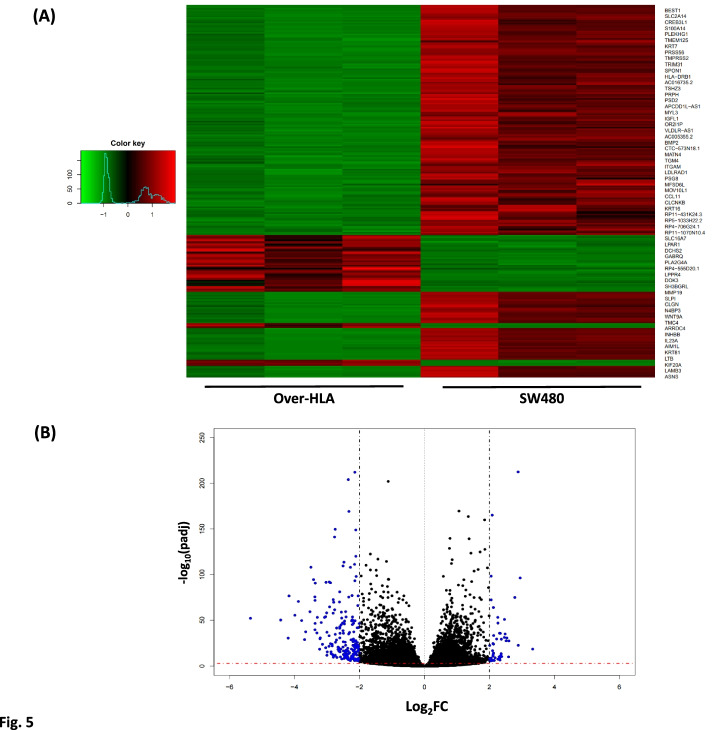
Heatmap (**A**) and volcano plot (**B**) showing differentially expressed genes (DEGs) between *HLA-C* overexpressing stable cells (Over-HLA) and SW480 cells. RNA sequencing was conducted with triplicate samples for each group, Over-HLA and SW480. A total of 248 DEGs, that were fulfilled for selection criteria (fold change ≥ 4 (|log_2_FC|≥ 2) and adjusted *P*-value < 0.001), were included in a heatmap. The blue dots in the volcano plot represent DEGs

To gain insight into genes regulated by the overexpression of *HLA-C*, we performed gene ontology analysis and pathway analysis using DAVID 6.7 (https://david.ncifcrf.gov/). For this purpose, we mainly focused on 248 DEGs that fulfilled DEG selection criteria with padj < 0.001 and fold change (FC) ≥ 4 (or |log_2_FC|≥ 2) (Fig. 5).

Gene ontology analysis demonstrates that many genes down-regulated in Over-HLA cells are highly enriched in cell proliferation-related biological functions (BP). Furthermore, the results of Kyoto Encyclopedia of Genes and Genomes (KEGG) pathway analysis revealed that several down-regulated genes in Over-HLA cells are significantly enriched in cancer-related pathways such as cytokine-cytokine receptor interaction (*P* = 3.80 X 10^–6^), pathways in cancer (*P* = 8.10 X 10^–3^), the JAK/STAT signaling pathway (*P* = 1.40 X 10^–2^), the ErbB signaling pathway (*P* = 2.50 X 10^–2^), Basal cell carcinoma (*P* = 3.30 X 10^–2^) and Hedgehog signaling pathway (*P* = 3.40 X 10^–2^) (Table 2 and Table S4). These results imply that *HLA-C* exerts its influence on CRC development via the down-regulation of genes involved in cancer-related pathways.

**Table 2:** The results of KEGG pathway analysis of 248 DEGs that fulfilled the selection criteria of |log_2_FC|≥ 2 and adjusted *P*-value < 0.001

Group	KEGG pathway term	*P*-value	Genes
**Up-regulated genes**	nd	nd	nd
**Down-regulated genes**	Cytokine-cytokine receptor interaction	3.80E-06	*BMP2, CCL11, CCL5, CSF2, INHBB, IL11, IL2RG, IL20RB, IL23A, IL7R, LTA, LTB, TNFSF15, TNFRSF4, TNFRSF9*
	Hematopoietic cell lineage	4.60E-03	*CSF2, ITGAM, IL11, IL7R, HLA-DRA, HLA-DRB1*
	Asthma	5.70E-03	*CCL11, HLA-DOA, HLA-DRA, HLA-DRB1*
	Pathways in cancer	8.10E-03	*CBLC, BIRC3, BMP2, FGF21, KLK3, LAMB3, LAMC2, MMP9, SHH, WNT7B, WNT9A*
	Jak-STAT signaling pathway	1.40E-02	*CBLC, CSF2, IL11, IL2RG, IL20RB, IL23A, IL7R*
	Type I diabetes mellitus	1.60E-02	*LTA, HLA-DOA, HLA-DRA, HLA-DRB1*
	ErbB signaling pathway	2.50E-02	*CBLC, SHC2, AREG, EREG, HBEGF*
	Basal cell carcinoma	3.30E-02	*BMP2, SHH, WNT7B, WNT9A*
	Hedgehog signaling pathway	3.40E-02	*BMP2, SHH, WNT7B, WNT9A*

Functional annotation is based on KEGG pathway analysis implemented in DAVID 6.7. Abbreviations are as follows: *BP* biological process, *nd* not detected

## Discussion

Most of GWA studies for colorectal cancer (CRC) have been conducted in populations of European ancestry [6–14]. Considering that only a few studies were conducted in Asian ancestry [16, 20] which differ from Europeans in certain features of genetic architecture, our study conducted in the Korean population will help provide additional information to fully understand the genetic basis for CRC susceptibility. Indeed, certain variants influencing differently on traits in different population may be due to the difference in allele frequency, effect size, or linkage disequilibrium (LD) structure [21].

A number of variants for CRC have been discovered by GWAS [6, 16]. However, these variants are not necessarily causal because GWAS largely depends on common variants mostly located in intron or intergenic regions. To obtain information regarding coding variants that are likely low frequency or rare variants, we genotyped 194 CRC cases and 600 controls using an Illumina HumanExome Bead Chip containing 219,621 nonsynonymous SNPs of a total of 242,901 SNPs. We used Fisher’s exact test to detect potential CRC causal genes for this study of low frequency or rare variants (MAF < 0.05).

In our association analysis, we detected three putative functional CRC loci (*P* < 1.0 X 10^–4^) on *SMCO1*, *HLA-C*, and *NUTM1* (Table 1). To test the functional relevance of CRC candidate genes in which functional variants are located (Fig. 2 and Table 1), we adopted biological approaches. The functional validation of GWAS gene candidates has been proposed and applied in the previous studies [22, 23].

Because CRCs are molecularly heterogeneous, we tried to test CRC candidate genes in several different types of CRC cell lines in DEG analysis using qRT-PCR. In this regard, we used five CRC cell lines (such as Caco-2, DLD-1, HCT116, HT-29, and SW480) covering various subtypes. According to the molecular (genomic instability) phenotypes, DLD-1 and HCT116 belong to microsatellite instable phenotype (MSI) as well as the epigenomic CpG island methylator phenotype (CIMP). HT29 belongs to CIMP, while CaCo-2 and SW480 microsatellite stable phenotype (MSS) [24]. These cell lines have long been widely used in CRC research and were available from American Type Culture Collection (ATCC).

From cell line-based qRT-PCR tests as well as DEG analyses using publicly available oligonucleotide microarray (NCBI GEO) and RNA-seq (TCGA) datasets (Fig. 3 and Fig. S2), we chose *HLA-C* as a potential gene functionally influencing CRC. *HLA-C* is a heavy chain receptor of the class I major histocompatibility complex (MHC). MHC molecules are known to control homeostasis in the immune system. Because the down-regulation of MHC class I activity in cancer is known to decrease the expression of tumor-associated antigen (TAA) and prevent cytotoxic T-cells (CTLs) from recognizing and destroying tumors [18], it is postulated that low expression levels of the MHC class may contribute to tumor development, as suggested previously in in vivo immune-tumor interaction [25].

To our knowledge, the involvement of *HLA-C* in CRC development has not been reported. Our exome array analysis detected the suggestive association of variants in *HLA-C* for CRC (Table S1). The minor allele frequency (MAF) of a lead SNP rs1130838 in *HLA-C* locus shows a substantial difference from that of other ethnic groups (Table S5). Here, the MAF of rs1130838 is 0.13 in Korean, while 0.32 in European, 0.33 in South Asian, 0.34 in African, and 0.24 ~ 0.39 in Latin American. In this regard, the allele frequency difference between Korean and other ancestry populations likely account for, in part, why this CRC locus was not detected in previous GWA studies mostly conducted in Europeans. Recently, however, two new associations (rs3131043 and rs9271770) for CRC were identified around the *HLA-C* region from GWAS meta-analysis in the population of European ancestry [6]. SNP rs3131043 and rs9271770 are located 470 kb 3′ and 1.4 Mb 5’, respectively, of *HLA-C*. Considering physical distance and linkage disequilibrium (LD), *HLA-C* is likely an independent CRC locus from these two loci (Table S6).

In our study, the G and A allele frequency of rs1130838 was 94.7% and 5.3% for CRC subjects, respectively, while 85.2% and 14.8% for control subjects **(**Fig. S6**)**. This difference in the allele frequency between the case and the control subjects indicates that the increase of G allele is associated with the increased susceptibility to CRC in our study population.

To gain insight into the functional role of *HLA-C* in CRC, we generated an *HLA-C* overexpression stable cell line (Over-HLA). Consistent to our finding of the down-regulation of *HLA-C* in CRC cells, we observed the reduced cell viability of Over-HLA. We then conducted RNA-seq analysis of Over-HLA cells to understand the underlying mechanism of *HLA-C* in CRC cell viability. RNA-seq is a method of measuring the expression level of RNA by analyzing the sequence of the transcriptome using next-generation sequencing (NGS) [26]. Since transcriptome refers to the set of all RNA molecules present in the cell, transcriptome information is valuable in interpreting the functional elements of the genome and reveals the molecular composition of cells and tissues [27].

RNA-seq analysis of Over-HLA cells and CRC cells demonstrated that several down-regulated genes in Over-HLA cells are significantly enriched in cancer-related signaling pathways (Table 2). Cytokine-cytokine receptor interaction has been reported for inflammation and tumor immunology in CRC [28]. It is believed that chronic inflammation affects the development and progression of cancer.

Down-regulated genes in the increased levels of *HLA-C* may reduce the activity of the Janus kinase/signal transducers and activators of transcription (JAK/STAT) signaling pathway. The JAK/STAT signaling pathway is involved in cell proliferation, differentiation, and migration by mediating cellular responses to several cytokines and growth factors [29, 30]. This pathway transmits information from chemical signals outside the cell to the cell nucleus, activating genes through transcription. The transcription factor known as signal transducer and activator (STAT) is involved in cell proliferation, apoptosis, and differentiation. STAT activation is associated with cancer [31]. Janus tyrosine kinase (JAK) and its phosphorylated target, STAT, are involved in immune regulation as important components of cytokine signal transduction [32].

ErbB signaling pathway activation mediates cell proliferation, migration, and survival [33]. The ErbB family of receptor tyrosine kinases (RTKs) includes an epidermal growth factor receptor (EGFR) that activates the ErbB signaling pathway. Src homology 2 (SHC2) activates cell proliferation though the Ras/Raf/MAPK pathway by son-of-sevenless (SOS) in the tyrosine phosphorylation of EGFR [34]. In our RNA-seq analysis, the downstream signals of *SHC2,* such as *KRAS* (log_2_FC = -0.53, padj = 5.95 X 10^–7^), *NRAS* (log_2_FC = -0.25, padj = 5.90 X 10^–4^), *BRAF* (log_2_FC = -0.68, padj = 3.06 X 10^–9^), and *MAP2K1* (log_2_FC = -0.28, padj = 1.46 X 10^–3^), are down-regulated by *SHC2* down-regulation (Table S4). Therefore, cell viability is thought to be reduced by the down-regulation of EGFR in Over-HLA (log_2_FC = -1.58, padj = 4.02 X 10^–30^).

The Hedgehog signaling pathway also has been reported to be involved in cancer development [35]. One of key components in Hedgehog signaling, SHH is known to inhibit PTCH’s activity, by which activates cancer-related functions such as proliferation, apoptosis suppression and stem cell self-renewal [36]. Therefore, the reduced viability of Over-HLA cells is likely due to the reduced levels of components in the Hedgehog signaling pathway in this study.

## Conclusions

We identified several candidate genes for CRC by genetic association analysis using an exome array chip. *HLA-C* was validated for its relevance to CRC by biological evidences. Indeed, we observed that *HLA-C* overexpression reduces cell viability in CRC cells, in which the expression of *HLA-C* was initially lower than in non-cancer colorectal cells. RNA-seq analysis suggests that overexpressed *HLA-C* reduces cell proliferation and the cell cycle by down-regulating signals in cancer-related pathways such as cytokine-cytokine receptor interaction, the JAK/STAT signaling pathway, the ErbB signaling pathway, and the Hedgehog signaling pathway. Taken together, the evidence from genetic association analysis and functional studies clearly points out that *HLA-C* is a potential gene involved in CRC.

## Materials and methods

### Study subjects, genotyping, and quality control

A total of 194 CRC patients were recruited from the Asan Hospital in Gangneung, Korea. The average age of CRC patients was 66.8 (± 1.5) years. This study was approved by the GangNeung Asan Hospital Institutional Review Board (2012–11-058). Written consent was obtained from study participants. All methods were performed in accordance with the relevant guidelines and regulations.

Genotyping of CRC subjects was performed using an Illumina HumanExome BeadChip (Illumina, San Diego, CA, USA) that includes a total of more than 240,000 exonic variants and can be used to study various common diseases such as type 2 diabetes, cancer, and metabolic diseases [37]. Marker categories included in this Illumina array chip are available elsewhere (http://www.smd.qmul.ac.uk/gc/Services/InfiniumArrays/datasheet_humanexome_beadchips.pdf). Genotype data of 600 controls generated on the same platform were obtained from the Korea Biobank of Korea’s Centers for Disease Control and Prevention. The average age of 600 control subjects was 62.0 (± 10.8) years. Finally, a total of 794 genotype data generated using DNA extracted from blood samples of Koreans were available for CRC case–control association analyses.

Based on genotype data, the exclusion criteria for subjects were as follows: (i) sample call rate < 98%, (ii) heterozygosity < 25%, (iii) outliers from a multi-dimensional scaling (MDS) plot generated via identity by state distance (IBS) calculations, and (iv) close relatives showing calculated average pairwise IBS value higher than that estimated from first-degree relatives of Korean sib-pair samples (> 0.80) [38]. None of the subjects were excluded based on sample quality control.

SNP exclusion criteria were as follows: (i) SNP call rate < 95%, (ii) minor allele frequency < 0.001, and (iii) Hardy–Weinberg equilibrium *P*-value < 1.0 X 10^–6^. For subsequent association analyses, monomorphic variants were further removed, leaving 43,082 autosomal SNPs. Analyses for sample and SNP QC were performed using PLINK 1.9 (http://pngu.mgh.harvard.edu/purcell/plink/) [39].

### Association analyses

To detect CRC candidate loci from the genotype data of 194 CRC and 600 control subjects, Fisher’s exact test were performed using PLINK 1.9. A total of 43,082 autosomal variants were tested for Fisher’s exact test after further excluding monomorphic variants from QC passed SNPs. In this analysis, disease-SNP associations were tested assuming an additive genetic model.

### Cell culture and reagent

Human colorectal cells (CCD-18co) were grown in Alpha + GlutaMAX-l (ThermoFisher Gibco, Waltham, MA, USA), a minimum essential medium, and human colorectal cancer cells HCT116, HT-29, Caco-2, SW480, and DLD-1 were grown in Dulbecco’s modified Eagle’s medium (ThermoFisher Gibco, Waltham, MA, USA) with supplements of 10% heat-inactivated fetal bovine serum (ThermoFisher Gibco, Waltham, MA, USA) and 1% penicillin–streptomycin (ThermoFisher Gibco, Waltham, MA, USA) at 37℃ in a CO_2_ 5% incubation chamber. All cell lines including human normal colon cell (CCD-18co) and human colorectal cancer cells (HCT116, HT-29, Caco-2, SW480, and DLD-1) were originally obtained from ATCC.

### Quantitative real-time reverse transcription-polymerase chain reaction (qRT-PCR)

Total RNA was isolated from human non-cancer colorectal cells (CCD-18co) and human colorectal cancer cells (HCT116, HT-29, Caco-2, SW480, and DLD-1) using a TRizol (Invitrogen) reagent according to the manufacturer’s protocol. cDNA synthesis was performed using Maxime RT PreMix (iNtRON Biotechnology, Seongnam-si, Gyeonggi-do, Republic of Korea) kits. qRT-PCR was performed using an iQTM SYBR Green Supermix (Bio-Rad, Hercules, CA, USA) by CFX Connect Real-Time System (Bio-Rad, Hercules, CA, USA) qPCR machine. The specific primer sequences of each gene are listed in Table S7. The level of mRNA expression of each CRC candidate gene in one cell type was normalized with the *ACTB* (β-actin) expression level as an internal control. The average level of gene expression in eight independent experiments between non-cancer colorectal cells and CRC cells was compared using the Wilcoxon rank-sum test of R software. *P*-values < 0.05 were considered to be statistically significant.

### Identification of DEGs using open access gene expression datasets

The mRNA expression data for CRC DEG analysis were obtained from the NCBI GEO dataset, a publicly available functional genomics data repository. We analyzed mRNA expression levels generated from oligonucleotide microarray analysis of 148 samples (GEO Accession number GSE21510 [40]), including 123 colorectal cancer and 25 normal colorectal tissue samples. After normalizing with the *B2M* expression level as an internal control, the gene expression levels of CRC candidate genes were compared between cancer and control samples by performing the Wilcoxon rank-sum test. We also obtained the RNA-seq data of 470 colorectal cancer tissue samples and 42 normal colorectal tissue samples from TCGA dataset. The mRNA expression levels (represented as HTseq-FPKM values) of CRC candidate genes were compared between cancer and control samples by performing the Wilcoxon rank-sum test.

### Generation of *HLA-C* overexpression CRC cell line

A cloned *HLA-C* coding sequence was purchased from TransOMIC technologies and inserted into pcDNA3.1( +) (Invitrogen, Waltham, MA, USA). The resulting expression plasmid of pcDNA-*HLAC* was transfected with SW480, a colorectal cancer cell line, according to the manufacturer’s suggested protocol using a Lipofectamine 3000 (Thermo Fisher Scientific, Waltham, MA, USA) reagent. After transfection, cells were treated with 1 mg/ml Geneticin (ThermoFisher Gibco, Waltham, MA, USA) for two weeks to identify Geneticin-resistant single clones. The overexpression of *HLA-C* was detected by qRT-PCR and western blot analyses in the *HLA-C* overexpression cells (Over-HLA).

### Western blot analysis

Proteins were extracted from SW480 and the *HLA-C* overexpression cells (Over-HLA) using a Pierce RIPA Buffer (Thermo Fisher Scientific, Waltham, MA, USA) and quantified via a BCA Protein Assay Kit (Thermo Fisher Scientific, Waltham, MA, USA). Thirty µg of protein was loaded on 10% sodium dodecyl sulfate–polyacrylamide gel electrophoresis (SDS-PAGE) gel and transferred to a polyvinylidene difluoride (PVDF) membrane. The membrane was blocked with a TBST (10X TBS, 0.1% Tween 20; Bio-Rad, Hercules, CA, USA) buffer containing 5% skim milk (BD Difco, Franklin Lakes, NJ, USA) for 1 h. Primary antibodies of HLA-C and β-Actin (Abcam, Cambridge, UK) were incubated overnight at 4℃ after washing with TBST buffer. The membrane incubated with Horse Radish Peroxidase (HRP)-conjugated antibodies (Abcam, Cambridge, UK) 1 h at room temperature. After washing the membrane with TBST buffer, Pirece ECL Plus Western Blotting Substrate (Thermo Fisher Scientific, Waltham, MA, USA) reagent was used to detect protein bands using a ChemiDoc MP Imaging System (Bio-Rad, Hercules, CA, USA). Protein bands appear at 45 kDa for HLA-C and 42 kDa for β-Actin.

### Cell viability assay

Cell viability was measured using Ez-cytox (DoGenBio, Guro-gu, Seoul, Republic of Korea) with water-soluble tetrazolium salts (WST). SW480 and Over-HLA cells were seeded with 3 X 10^3^ cells/well in a 96-well culture plate, and cell viability was measured after 24, 48, and 72 h incubation. After treatment with 10 µl/well of Ez-cytox reagent and 1 h incubation, the absorbance at 450 nm was measured using a spectrophotometer (TECAN, Mannedorf, Switzerland). Cell viability analysis is presented as mean ± standard deviation (SD), obtained from eight independent experiments. Statistical analyses were performed using R software. Group differences were assessed by the Wilcoxon rank-sum test. *P*-values < 0.05 were considered to be statistically significant.

### RNA sequencing (RNA-seq)

Total RNA was isolated from SW480 and Over-HLA cells using an RNeasy Mini Kit (QIAGEN, Hilden, Germany) according to the manufacturer’s suggested protocol. The concentration and purity of isolated RNA were determined by optical density measurement (A260 and A260/280, respectively) using a Nanodrop (Thermo Fisher Scientific, Waltham, MA, USA). The RNA-seq library was created using TruSeq RNA Library Prep Kit v2 (Illumina, San Diego, CA, USA) according to the manufacturer’s protocol. RNA-seq was conducted with triplicate samples for each group, Over-HLA and SW480, using an Illumina NextSeq 500 System. The quality of RNA-seq data was measured using FastQC (http://www.bioinformatics.babraham.ac.uk/projects/fastqc) software.

Adaptor trimming and removing sequencing reads with poor quality scores (Q < 30) were performed using the Trim Galore (https://www.encodeproject.org/software/trim_galore/) software. Processed paired end reads were aligned to the human reference genome (hg38) using TopHat2 [41] and Bowtie [42] software. Levels of gene expression in SW480 and *HLA-C* overexpression SW480 cells were measured by HT-seq count [43] software. With HT-seq count data, DEGs between two cell lines were identified by DESeq2 software [44]. The overall RNA-seq process is shown in Fig. S4.

### Pathway analysis for DEGs

For the functional interpretation of DEGs, DAVID 6.7, the Database for Annotation, Visualization, and Integrated Discovery (https://david.ncifcrf.gov/), was used to perform KEGG pathway and gene ontology (GO) enrichment analyses.

## Supplementary Information

**Additional file 1: Fig. S1. **Distribution of minor allele frequencies (MAFs) of genotyping of 794 subjects by Illumina HumanExome BeadChip. **Fig. S2.** The expression levels of three CRC candidate genes such as SMCO1 (A), HLA-C (B), and NUTM1 (C). **Fig. S3.** Full-length blots and gel of Fig. 4 (C). **Fig. S4.** RNA sequencing analysis pipeline. **Fig. S5.** Heatmap of  differentially expressed genes between *HLA-C *overexpressing stable cells (Over-HLA) and SW480 cells. **Fig. S6. **The allele frequency of rs1130838 in CRC and control subjects.

**Additional file 2: Table S1. **Results of Fisher’s exact test for CRC case-control association analysis. A total of 2,068variants were nominally associated with CRC (P-value < 0.05). **Table S2. **The summary statistic of reads produced by RNA sequencing and reads mapped to genome using TopHat2 aligner. **Table S3. **Differentially expressed genes (DEG) between SW480 colorectal cells (SW1, SW2, and SW3) and HLA-C overexpressing stable cells (S1, S2, and S3). **Table S4.** Functional annotation of 248 DEGs that fulfilled the selection criteria of |log2FC| ≥ 2 and adjusted P -value < 0.001. Functional annotation was based on Gene Ontology (GO) and KEGG pathway analyses implemented in DAVID 6.7. **Table S5.** Allele frequency of rs1130838 in several ethnic groups. **Table S6.** Pair-wise LD (r2) between a variant on HLA-C(rs1130838) and each variant (rs3131043 and rs9271770)near HLA-C region reported in Law, P.J. et al. (Nat Commun10, 2154 (2019)). **Table S7.** The primer sequences for qRT-PCR for CRC candidate genes.

## Data Availability

The microarray data for CRC DEG analysis are available in the NCBI Gene Expression Omnibus (GEO) database with the GEO Accession number GSE21510. The RNA-seq data for CRC DEG analysis are available in the Cancer Genome Atlas (TCGA) dataset (https://gdc-hub.s3.us-east-1.amazonaws.com/download/TCGA-COAD.htseq_fpkm-uq.tsv.gz). The RNA-seq data on SW480 and Over-HLA cells are available from the NCBI Short Read Archives (https://www.ncbi.nlm.nih.gov/sra/PRJNA815699). Exome array data are available from National Biobank of Korea (https://nih.go.kr/biobank) upon reasonable request.
