# Supplementary material for: Identification and functional validation of HLA-C as a potential gene involved in colorectal cancer in the Korean population
Source: BMC Genomics. 2022 Apr 4;23:261. doi: 10.1186/s12864-022-08509-5 (PMC8981957; doi:10.1186/s12864-022-08509-5)
Supplement: Supplementary file 1 — Additional file 1: Fig. S1. Distribution of minor allele frequencies (MAFs) of genotyping of 794 subjects by Illumina HumanExome BeadChip. Fig. S2. The expression levels of three CRC candidate genes such as SMCO1 (A), HLA-C (B), and NUTM1 (C). Fig. S3. Full-length blots and gel of Fig. 4 (C). Fig. S4. RNA sequencing analysis pipeline. Fig. S5. Heatmap of differentially expressed genes between HLA-C overexpressing stable cells (Over-HLA) and SW480 cells. Fig. S6. The allele frequency of rs1130838 in CRC and control subjects. [file 12864_2022_8509_MOESM1_ESM.pdf]

## Legends for the supplementary figures

**Fig. S1. Distribution of minor allele frequencies (MAFs) of genotyping of 794 subjects by Illumina HumanExome BeadChip.** After SNP QC, remaining 236,116 autosomal SNPs were categorized according to MAF (such as monomorphic, 0 < MAF < 0.01, 0.01 ≤ MAF <0.05, and 0.05 ≤ MAF).

**Fig. S2. The expression levels of three CRC candidate genes such as *SMCO1* (A), *HLA-C* (B), and *NUTM1* (C)**. Gene expression data were obtained from cell line-based qRT-PCR experiments as well as publicly available oligonucleotide microarray (NCBI GEO) and RNA-seq (TCGA) datasets. In cell line-based qRT-PCR experiments, *GAPDH* was used as an internal control. Group differences were assessed by the Wilcoxon rank-sum test. **P*<0.05, ***P*<0.01, ****P*<0.001 vs control.

**Fig. S3. The full-length blots and gel that were used for Fig. 4 (C).** Equally prepared samples were used in western blot experiments to detect the levels of HLA-C and β-actin in Fig. 4(C).

(A) The full-length SDS-PAGE gel for total lysates of SW480 (lanes 1 and 5) and Over-HLA (lanes 2, 3, 4, 6, 7, and 8). A total of 30μg proteins was loaded in lanes 1, 2, 3, and 4. A total of 70μg proteins was loaded in lanes 5, 6, 7, and 8.

(B) The full-length western blot generated from the duplicate SDS-PAGE gel in Fig. S3(A). The red box area on the blot was cropped for the upper part of Fig. 4(C). The bands in lanes 1 to 8 indicate HLA-C.

(C) The full-length western blot to detect β-Actin bands in SW480 (lanes 1 and 3) and Over-HLA samples (lanes 2 and 4). The red box area on the blot was cropped for the lower part of Fig. 4(C). A total of 30μg proteins was loaded in each lane. The bands in lanes 1 to 4 indicate β-Actin.

**Fig. S4. RNA sequencing analysis pipeline**

**Fig. S5. Heatmap of differentially expressed genes between *HLA-C* overexpressing stable cells (Over-HLA) and SW480 cells.** RNA sequencing was conducted with triplicate samples for each group, Over-HLA and SW480. A total of 6,528 DEGs were detected (adjusted *P*-value < 0.001) by DESeq2 analysis.

**Fig. S6. The allele frequency of rs1130838 in CRC and control subjects.**

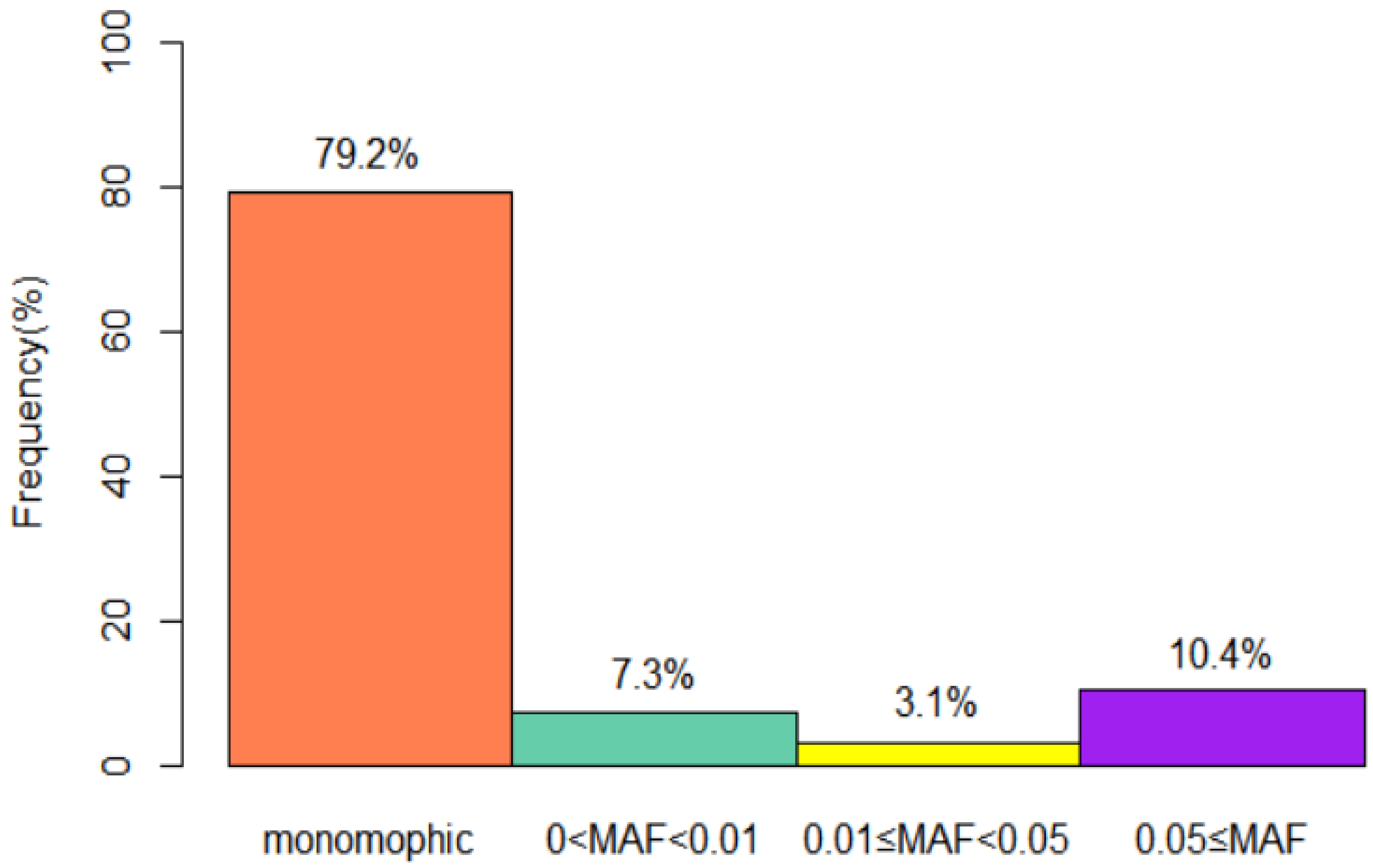


**Fig. S1. Distribution of minor allele frequencies (MAFs) of genotyping of 794 subjects by Illumina HumanExome BeadChip**

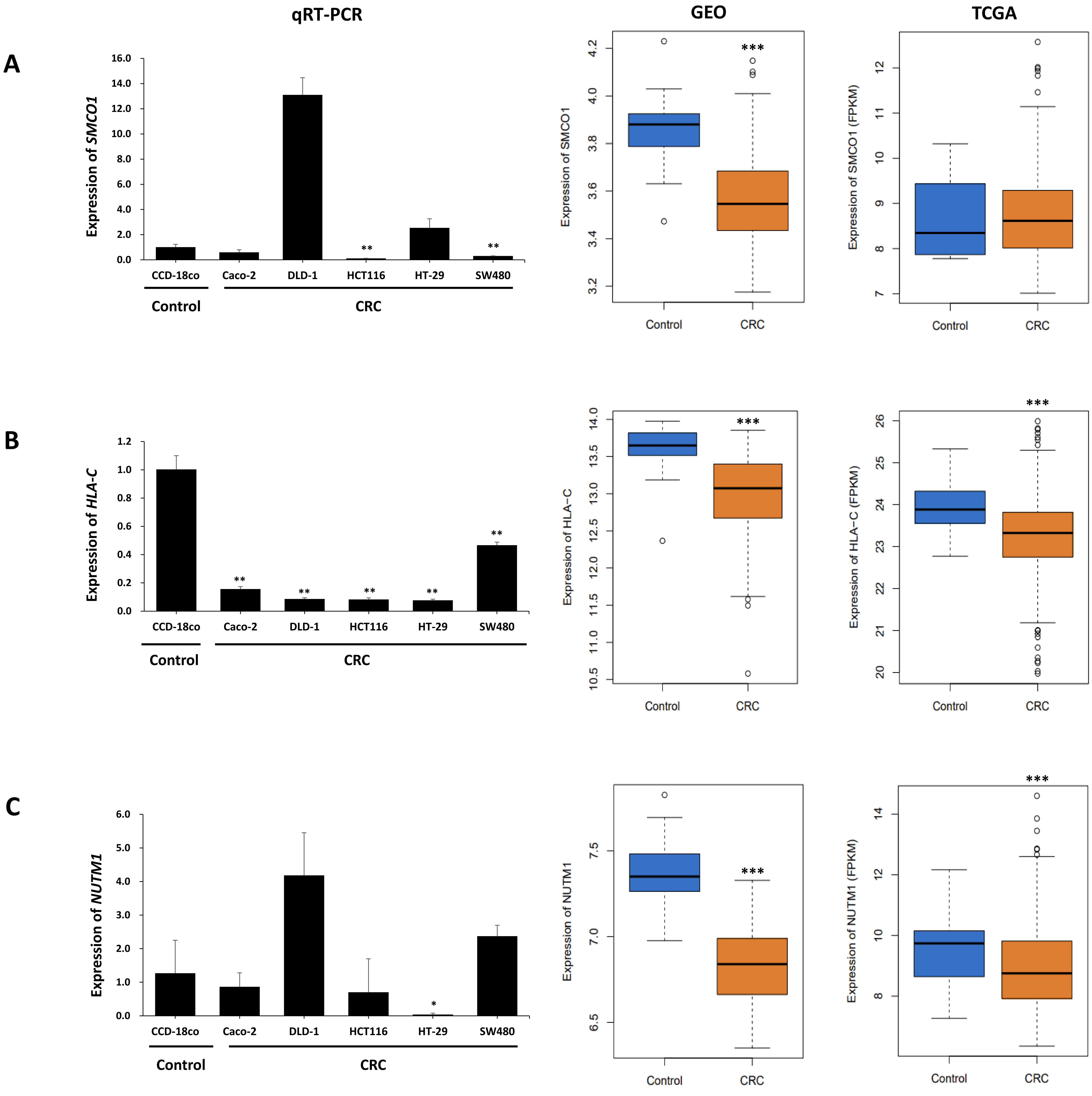


**Fig. S2. The expression levels of three CRC candidate genes such as *SMCO1* (A), *HLA-C* (B), and *NUTM1* (C)**

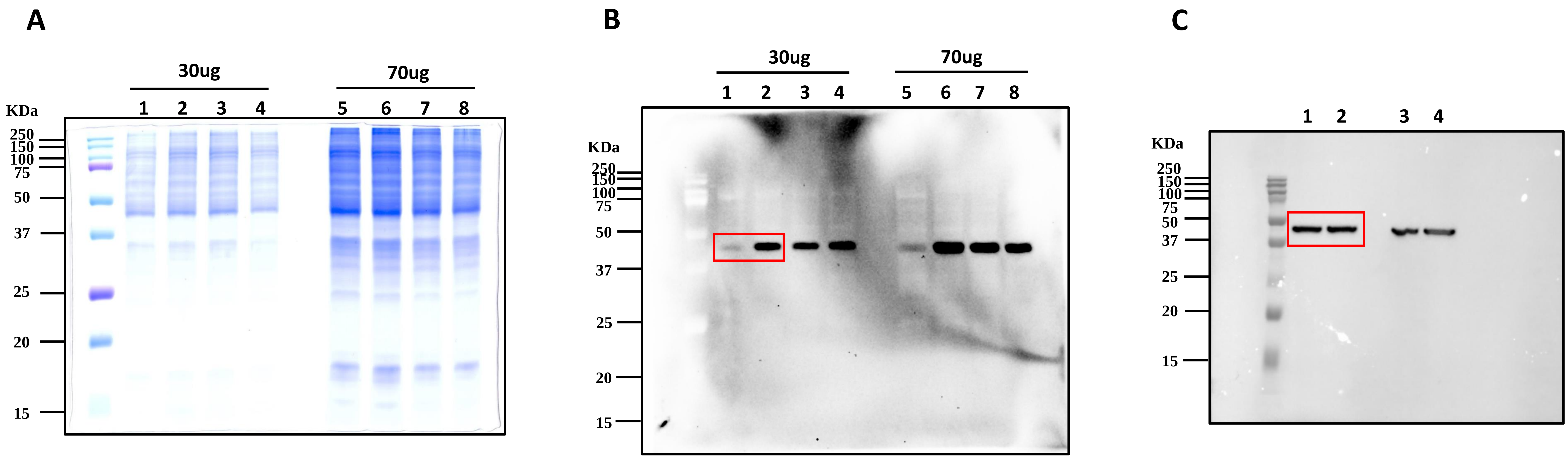


**Fig. S3. Full-length blots and gel of Fig. 4 (C)**

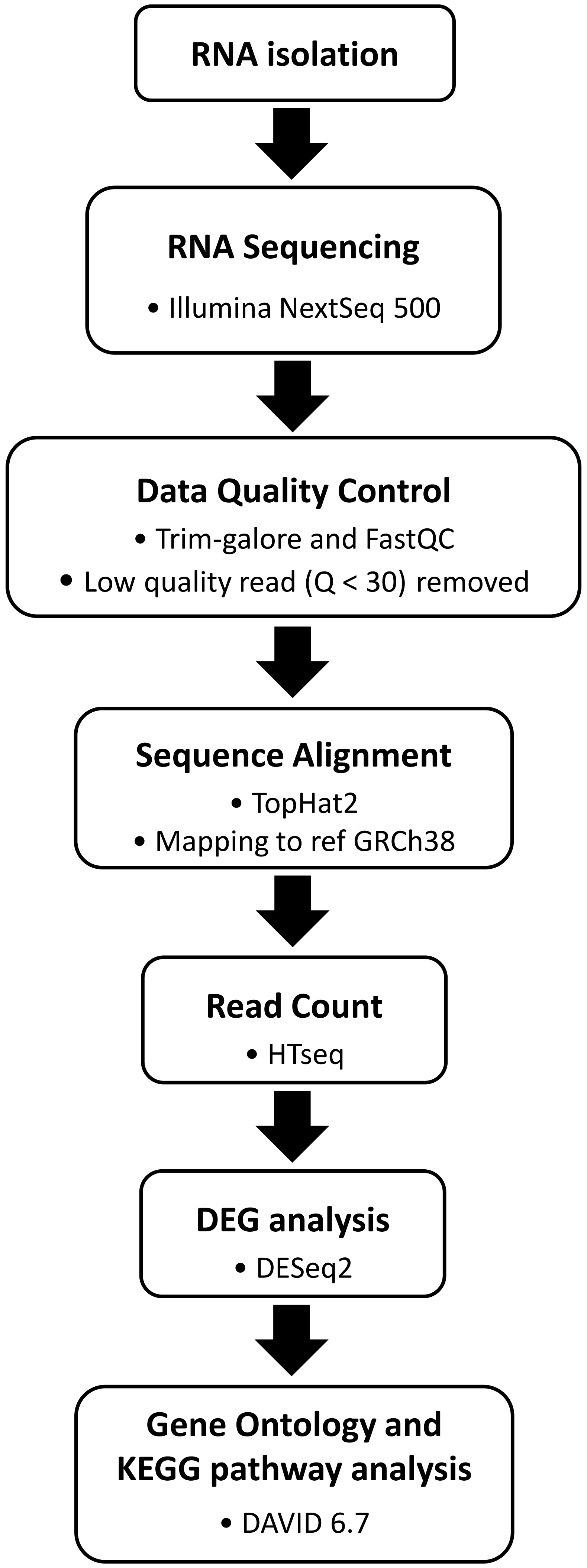


**Fig. S4. RNA sequencing analysis pipeline**

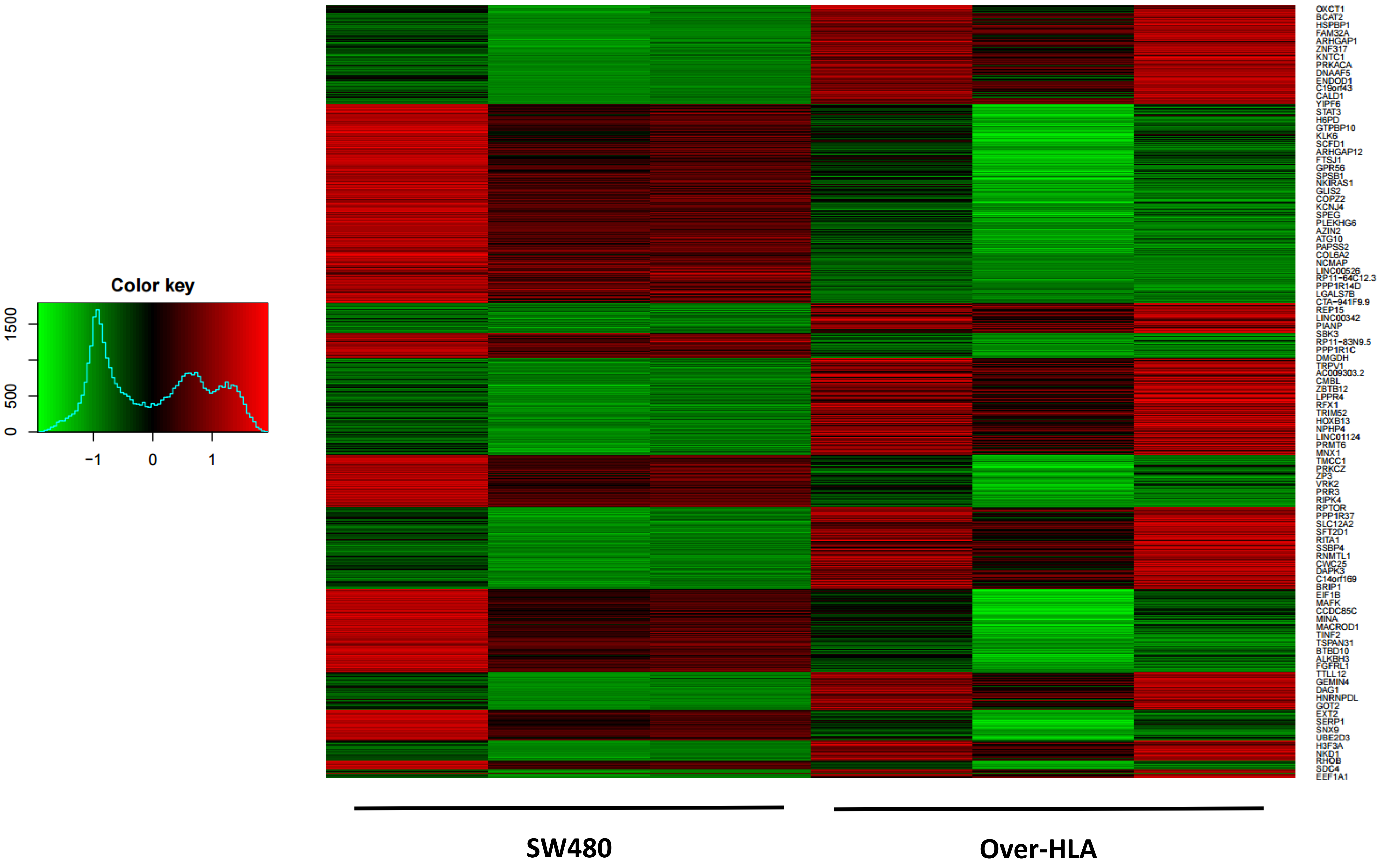


**Fig. S5. Heatmap of differentially expressed genes between *HLA-C* overexpressing stable cells (Over-HLA) and SW480 cells**

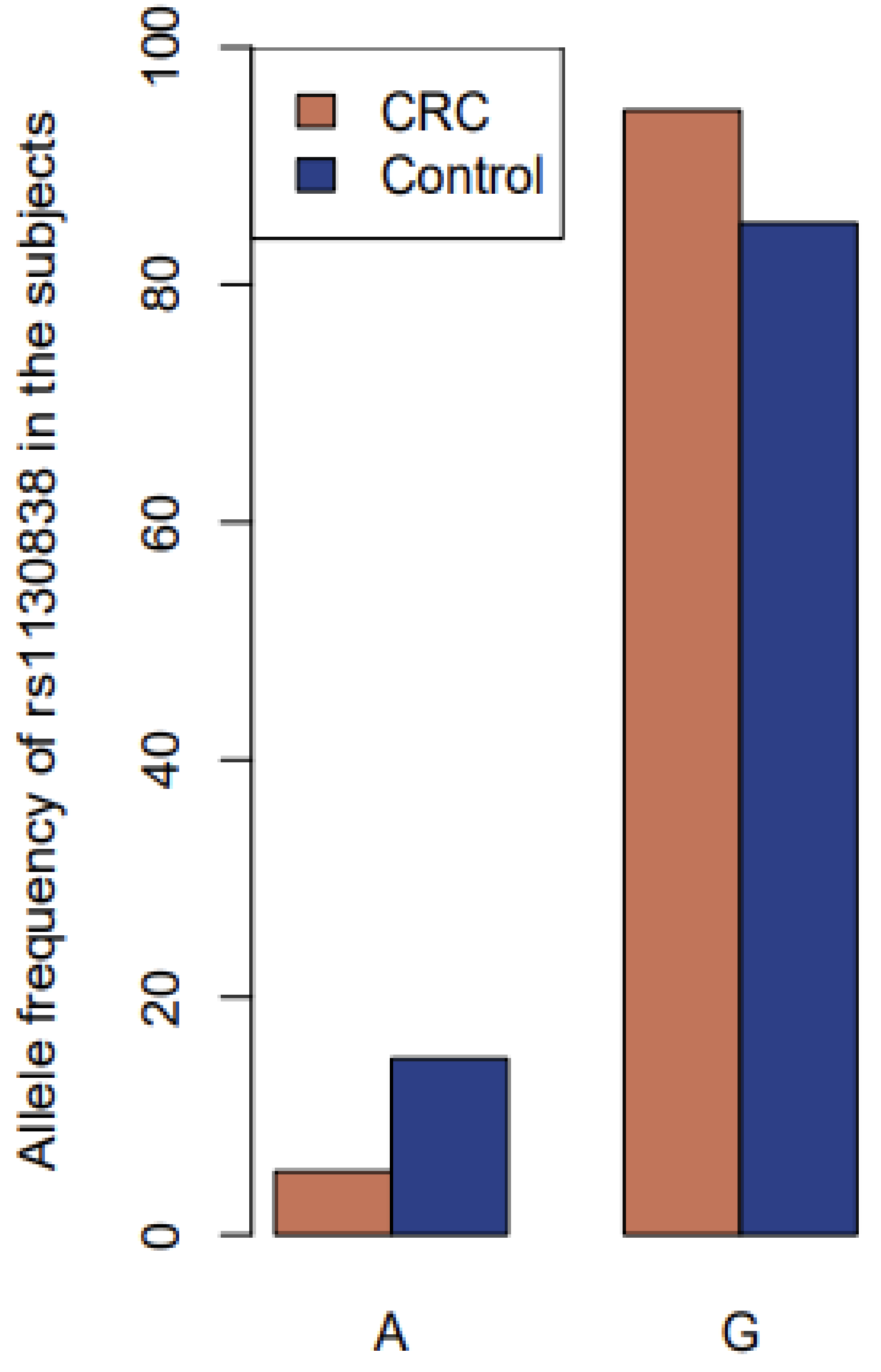


**Fig. S6. The allele frequency of rs1130838 in CRC and control subjects**
