# Supplementary material for: Identification and functional validation of HLA-C as a potential gene involved in colorectal cancer in the Korean population
Source: BMC Genomics. 2022 Apr 4;23:261. doi: 10.1186/s12864-022-08509-5 (PMC8981957; doi:10.1186/s12864-022-08509-5)
Supplement: Supplementary file 2 — Additional file 2: Table S1. Results of Fisher’s exact test for CRC case-control association analysis. A total of 2,068variants were nominally associated with CRC (P-value < 0.05). Table S2. The summary statistic of reads produced by RNA sequencing and reads mapped to genome using TopHat2 aligner. Table S3. Differentially expressed genes (DEG) between SW480 colorectal cells (SW1, SW2, and SW3) and HLA-C overexpressing stable cells (S1, S2, and S3). Table S4. Functional annotation of 248 DEGs that fulfilled the selection criteria of |log2FC| ≥ 2 and adjusted P -value < 0.001. Functional annotation was based on Gene Ontology (GO) and KEGG pathway analyses implemented in DAVID 6.7. Table S5. Allele frequency of rs1130838 in several ethnic groups. Table S6. Pair-wise LD (r2) between a variant on HLA-C(rs1130838) and each variant (rs3131043 and rs9271770)near HLA-C region reported in Law, P.J. et al. (Nat Commun10, 2154 (2019)). Table S7. The primer sequences for qRT-PCR for CRC candidate genes. [file 12864_2022_8509_MOESM2_ESM.pdf]

Legends for the supplementary tables

Table S1. Results of Fisher's exact test for CRC case-control association analysis. A total of 2,068 variants were nominally associated with CRC (*P*-value < 0.05).

Notes: CHR, Chromosome; SNP, SNP ID; BP, Physical position (base-pair); A1, Minor allele name (based on whole sample); F_A, Frequency of this allele in cases; F_U, Frequency of this allele in controls; A2, Major allele name; P, Exact P-value of this test; OR, Estimated odds ratio (for A1); SE, standard error; L95, Lower bound of 95% confidence interval for odds ratio; U95, Upper bound of 95% confidence interval for odds ratio

Table S2. The summary statistics of reads produced by RNA sequencing and reads mapped to genome using TopHat2 aligner.

Table S3. Differentially expressed genes (DEG) between SW480 colorectal cells (SW1, SW2, and SW3) and *HLA-C* overexpressing stable cells (S1, S2, and S3). The cut-off criterion for DEG was adjust *P*-value [padj] < 0.001. Numbers in each sample represent the levels of gene expression that were estimated by RNA sequencing reads. Sequencing reads were counted by HTseq-count software. Differentially expressed genes were detected using DESeq2 program. FC indicates Fold Change. The total gene expression profile resulted from HTseq-count and DESeq2 analyses is available in http://web.hallym.ac.kr/~yooncho/index.html.

Table S4. Functional annotation of 248 DEGs that fulfilled the selection criteria of $|\log_2 FC| \geq 2$ and adjusted *P*-value < 0.001. Functional annotation was based on Gene Ontology (GO) and KEGG pathway analyses implemented in DAVID 6.7.

Notes: Selected DEGs included 45 up- and 203 down-regulated genes. Abbreviations are as follows: BP, biological process; CC, cellular component; MF, molecular function; ND, not detected.

Table S5. Allele frequency of rs1130838 in several ethnic groups.

Notes: The allele frequency of rs1130838 was available in dbSNP database (https://www.ncbi.nlm.nih.gov/snp/). Abbreviation: KRGDB, Korean Reference Genome Database.

Table S6. Pair-wise LD (r^2) between a variant on *HLA-C* (rs1130838) and each variant (rs3131043 and rs9271770) near *HLA-C* region reported in Law, P.J. et al. (Nat Commun 10, 2154 (2019)).

Table S7. The primer sequences for qRT-PCR for CRC candidate genes.

Table S1. Results of Fisher's exact test for CRC case-control association analysis. A total of 2,068 variants were nominally associated with CRC (*P* -value < 0.05).

CHR	SNP	BP	A1	F_A	F_U	A2	P	OR
3	rs11926701	196236401	A	0.06959	0.01104	G	9.23E-09	6.703
6	rs2844533	31350802	G	0.08763	0.1783	A	8.72E-06	0.4425
6	rs2596548	31330546	A	0.0232	0.07917	C	2.98E-05	0.2762
5	rs7723080	11871700	G	0.4149	0.2992	A	3.01E-05	1.662
6	rs1130838	31237124	A	0.07216	0.1483	G	5.81E-05	0.4466
6	rs1131096	31238259	C	0.07216	0.1483	A	5.81E-05	0.4466
15	rs2279685	34649631	A	0.1804	0.2817	G	5.81E-05	0.5614
17	rs757608	59497277	A	0.2062	0.3108	G	5.88E-05	0.5759
6	rs3130542	31232111	A	0.06186	0.1333	G	7.76E-05	0.4286
6	rs2524074	31244021	G	0.06186	0.1325	A	7.79E-05	0.4317
6	rs3132572	30861729	G	0.005155	0.04083	A	0.0001724	0.1217
6	rs2844650	30902533	A	0.005155	0.04083	G	0.0001724	0.1217
6	rs2517451	30914751	G	0.005155	0.04083	A	0.0001724	0.1217
6	rs3130933	31132085	A	0.005155	0.04083	G	0.0001724	0.1217
18	rs2298548	9221883	G	0.06701	0.02417	C	0.000176	2.9
18	rs17498752	9254785	A	0.06701	0.02417	G	0.000176	2.9
18	rs72939232	9255784	A	0.06701	0.02417	C	0.000176	2.9
18	rs3744822	9258537	G	0.06701	0.02417	A	0.000176	2.9
11	rs2513605	106318654	G	0.3325	0.44	A	0.0001845	0.6339
6	rs2523644	31342484	G	0.05729	0.1225	A	0.0001936	0.4353
6	rs2523480	31355702	C	0.007732	0.045	G	0.0002158	0.1654
6	rs3094609	31165566	A	0.04145	0.1	G	0.0002223	0.3892
6	rs2524054	31252396	A	0.03866	0.095	C	0.0002448	0.3831
17	rs201149338	36485071	G	0.03866	0.009167	C	0.0002635	4.347
6	rs2596503	31320810	A	0.09794	0.1733	G	0.0002668	0.5178
7	rs2960999	72193868	G	0.3814	0.4883	A	0.0002761	0.6461
9	rs10858396	138275776	A	0.1649	0.09583	G	0.0002999	1.864
17	rs145353986	63533087	C	0.07474	0.03	G	0.0003083	2.612
2	rs10495928	46353166	G	0.25	0.1658	A	0.0003091	1.677
6	rs2247056	31265490	A	0.03866	0.0925	G	0.0003428	0.3945
6	rs6457374	31272261	G	0.03866	0.0925	A	0.0003428	0.3945
14	exm1112651	73440864	A	0.3943	0.2955	G	0.0003518	1.552
6	rs3095345	30822413	G	0.06186	0.1258	A	0.0003603	0.458
11	rs10895959	106330162	A	0.3634	0.4658	G	0.0004139	0.6547
7	rs35289897	72225895	G	0.386	0.49	A	0.0004228	0.6544
14	rs12931	73440864	A	0.3943	0.2967	G	0.000448	1.544
6	rs2243379	117724462	C	0.5619	0.4592	A	0.0004537	1.51
10	rs7893462	28228865	A	0.4923	0.39	G	0.0004629	1.516
6	rs2073717	31122126	G	0.2758	0.3733	C	0.0004719	0.6392
6	rs3132469	31456567	A	0.007732	0.04167	G	0.0004939	0.1792

18	exm-IND18-9094	9104205	I	0.06443	0.02417	D	0.0005267	2.781
6	rs2076530	32363816	G	0.3737	0.475	A	0.0005271	0.6595
6	rs17885382	32552095	A	0.02618	0.07227	G	0.0005448	0.3451
6	rs2240064	31114573	G	0.2938	0.3908	A	0.0005492	0.6485
6	rs3131012	31115441	A	0.2938	0.3908	G	0.0005492	0.6485
17	rs8068318	59483766	A	0.482	0.3825	G	0.0005664	1.502
16	rs11545690	46652209	C	0.1186	0.0625	A	0.0005787	2.018
19	exm-IND19-4631	41622108	I	0.201	0.29	D	0.0005931	0.616
14	rs997667	73465003	G	0.3789	0.2842	A	0.0006166	1.537
6	rs12526186	30736151	A	0.5052	0.4058	G	0.0006258	1.495
2	rs2271041	99008352	A	0.002577	0.02917	G	0.0006726	0.08601
10	rs9787568	1462457	A	0.4046	0.5042	G	0.0006923	0.6684
3	rs9822460	98073313	G	0.183	0.1142	A	0.0007092	1.738
10	rs1877131	65654554	A	0.451	0.3533	G	0.0007278	1.504
9	rs7048527	95993170	G	0.2062	0.2933	A	0.0007992	0.6257
1	rs41278952	7797503	G	0.03351	0.08167	C	0.0008121	0.3898
16	rs4346186	8305679	A	0.5284	0.43	C	0.0008192	1.485
4	rs358592	21476990	G	0.3866	0.485	A	0.0008281	0.6692
16	rs9927623	8304438	C	0.5284	0.4308	A	0.000832	1.48
6	rs3129987	30766204	A	0.04639	0.1	G	0.000836	0.4378
13	rs7982823	94565251	G	0.1778	0.1108	A	0.0008386	1.735
2	rs202058804	171243679	G	0.01289	0	A	0.0008539	NA
9	rs2226006	120137758	G	0.5722	0.4742	A	0.0008638	1.483
6	rs1471218	153863384	G	0.4845	0.3875	A	0.0008774	1.486
6	rs147836127	117589645	C	0	0.02167	G	0.0009055	0
15	rs57115249	34648935	A	0.1881	0.2717	C	0.0009487	0.6213
6	rs3132499	31207920	G	0.04381	0.095	A	0.000953	0.4365
6	rs7382297	31247067	A	0.04381	0.095	C	0.000953	0.4365
12	rs146035213	71978045	G	0.02062	0.0025	A	0.0009663	8.4
14	rs201880070	96798705	G	0.02062	0.0025	C	0.0009663	8.4
17	rs140140359	74072895	G	0.02062	0.0025	C	0.0009663	8.4
19	rs306457	56499279	G	0.2887	0.3817	C	0.0009945	0.6574
6	rs3734799	151670172	A	0.3763	0.4725	C	0.0009994	0.6735
6	rs3106192	33326250	G	0.2655	0.3567	A	0.001017	0.6519
5	rs1048944	34824555	A	0.3247	0.4183	C	0.00102	0.6687
1	rs2304314	55134585	G	0.04381	0.01419	A	0.001059	3.183
6	rs2298090	26157073	G	0.002577	0.02833	A	0.001062	0.08862
6	rs465223	33359716	G	0.232	0.3192	C	0.001108	0.6442
19	rs3745217	41249853	A	0.01804	0.001667	G	0.001119	11.01
5	rs2290280	86708721	A	0.2062	0.1358	C	0.001144	1.652
6	rs201818972	117589606	G	0.01546	0.0008333	C	0.001147	18.83
3	rs10513137	141143430	A	0.2036	0.2867	G	0.001196	0.6362
3	rs200963433	130289976	A	0.03093	0.0075	G	0.001261	4.223

6	rs3823310	151674326	A	0.3995	0.4942	C	0.00128	0.6809
6	rs6913309	32339840	A	0.1546	0.2312	T	0.001282	0.6082
15	rs4775785	49192326	A	0.5412	0.4467	G	0.001283	1.462
6	rs222577	151660477	C	0.4124	0.5067	A	0.001304	0.6833
3	rs6439508	134388550	A	0.1675	0.2458	G	0.001307	0.6174
12	rs4278572	79391433	G	0.2706	0.3592	A	0.001313	0.662
2	rs10168349	46360907	C	0.2371	0.1625	G	0.001388	1.602
1	rs138061134	20980734	A	0.04124	0.01333	G	0.001499	3.183
17	rs1156287	53076799	G	0.07474	0.03417	A	0.001504	2.284
1	rs11119315	209785206	A	0.2397	0.1658	G	0.001506	1.586
1	rs11582005	109268441	A	0.1314	0.07667	G	0.001519	1.823
1	rs12094678	3111669	G	0.4271	0.5202	A	0.001534	0.6876
6	rs2071295	32038700	A	0.1649	0.2417	G	0.001558	0.6198
15	rs4502159	50468959	C	0.3995	0.3117	A	0.001581	1.469
6	rs4424066	32354428	G	0.3325	0.4225	A	0.001594	0.6808
6	rs3817973	32361111	A	0.3325	0.4225	G	0.001594	0.6808
6	rs9378805	417727	C	0.2474	0.3333	A	0.001604	0.6575
6	rs2239839	33288075	A	0.1546	0.2292	C	0.00161	0.6153
3	rs347134	32439785	G	0.4253	0.5175	A	0.001616	0.6899
2	rs6732438	64735823	G	0.1451	0.2187	A	0.001639	0.6062
6	rs3130501	31136453	A	0.2139	0.2958	G	0.001687	0.6477
6	rs1051488	31322911	G	0.3557	0.4467	A	0.001726	0.6838
20	rs1056720	3784110	A	0.4149	0.3258	G	0.001739	1.467
10	rs201548058	46998873	C	0.02835	0.006667	A	0.001758	4.347
6	rs1265115	31117075	C	0.3789	0.47	A	0.001841	0.6878
21	rs2824721	19670118	G	0.2912	0.3783	A	0.001869	0.6752
8	rs10505477	128407443	A	0.5284	0.4375	G	0.001901	1.44
7	rs4732112	134970088	G	0.4278	0.3392	A	0.001906	1.457
6	rs9267547	31675501	A	0.08763	0.1483	G	0.001936	0.5515
6	rs2071293	32062687	A	0.1649	0.24	G	0.00194	0.6255
1	rs2272629	17593316	A	0.1211	0.1883	G	0.001945	0.594
6	rs3130275	33332137	G	0.1933	0.2717	A	0.001961	0.6424
19	rs388685	44418680	G	0.299	0.3858	C	0.00197	0.6789
19	rs399098	44418824	G	0.299	0.3858	A	0.00197	0.6789
1	rs76075180	150526393	A	0.1289	0.07583	G	0.002011	1.803
6	rs644045	31883957	A	0.1469	0.08917	G	0.002018	1.759
1	rs3750300	17586134	G	0.1237	0.1908	A	0.002074	0.5986
6	rs3095302	31093066	G	0.3608	0.4508	A	0.00213	0.6876
6	rs3095301	31093356	A	0.3608	0.4508	G	0.00213	0.6876
6	rs2073721	31129616	A	0.2165	0.2967	G	0.00214	0.6551
6	rs2073723	31130078	A	0.2165	0.2967	G	0.00214	0.6551
6	rs1065461	31130502	A	0.2165	0.2967	G	0.00214	0.6551
6	rs3130502	31136666	A	0.2165	0.2967	G	0.00214	0.6551

9	rs78653020	20789546	G	0.03866	0.0125	C	0.002161	3.177
3	rs79929179	190999876	A	0.03093	0.008333	G	0.002171	3.798
6	rs6922269	151252985	A	0.03093	0.008333	G	0.002171	3.798
8	rs7017776	19362768	G	0.1082	0.1733	A	0.002238	0.5789
4	rs17013285	88767008	A	0.1881	0.265	G	0.00225	0.6428
6	rs4713447	31162963	A	0.3814	0.4708	G	0.002251	0.6931
7	rs10230714	94540416	G	0.1856	0.1225	A	0.002263	1.632
6	rs456993	33358278	G	0.2216	0.3025	A	0.002269	0.6566
6	rs2844697	30932309	A	0.1495	0.2208	G	0.002273	0.6201
21	rs2073380	47863757	C	0.3814	0.47	A	0.002276	0.6954
12	rs141996636	114395768	A	0.02062	0.003333	G	0.002285	6.295
8	rs6983267	128413305	C	0.5258	0.4358	A	0.002286	1.435
2	rs10210989	214867770	A	0.5309	0.4417	G	0.002324	1.431
6	rs1755047	33431319	C	0.2242	0.305	G	0.002333	0.6586
2	rs2241057	72361960	G	0.1237	0.0725	A	0.002336	1.806
2	rs34885252	241808347	G	0.116	0.06667	A	0.002363	1.837
2	rs34664134	241812461	A	0.116	0.06667	G	0.002363	1.837
6	rs17207867	31938412	A	0.03093	0.0725	C	0.002384	0.4083
6	rs12153855	32074804	G	0.03093	0.0725	A	0.002384	0.4083
6	rs28732176	32097290	A	0.03093	0.0725	G	0.002384	0.4083
6	rs3096696	32122472	A	0.03093	0.0725	C	0.002384	0.4083
6	rs3096697	32134510	A	0.03093	0.0725	G	0.002384	0.4083
6	rs3130284	32140487	G	0.03093	0.0725	A	0.002384	0.4083
6	rs3134947	32145205	A	0.03093	0.0725	G	0.002384	0.4083
6	rs3134946	32145993	C	0.03093	0.0725	G	0.002384	0.4083
6	rs3135405	33311242	G	0.2474	0.33	A	0.002403	0.6675
7	rs1706912	105280270	G	0.3015	0.3875	A	0.002419	0.6824
16	rs9932912	23096952	A	0.02577	0.005833	G	0.002446	4.509
6	rs456261	33258443	A	0.2294	0.31	G	0.002463	0.6625
1	rs4846914	230295691	A	0.1314	0.2	G	0.002468	0.6053
10	rs200905582	125780762	G	0.201	0.135	C	0.002472	1.612
6	rs3131003	31093482	G	0.3608	0.4483	A	0.002591	0.6946
16	rs7190666	19548116	A	0.2139	0.1467	G	0.002595	1.583
5	rs76381196	101631761	G	0.09021	0.0475	A	0.002604	1.988
16	rs3743728	67242276	A	0.007732	0.035	G	0.002606	0.2148
14	exm1132204	105406522	A	0.1443	0.08848	G	0.002651	1.738
9	rs117609609	136029611	A	0.0567	0.02417	G	0.002679	2.427
14	rs6572051	41522215	C	0.0567	0.02417	A	0.002679	2.427
9	rs7024419	102624496	G	0.3222	0.4075	A	0.002711	0.6911
1	rs201005787	184006362	G	0.002577	0.02583	C	0.002725	0.09744
6	rs13195509	26463660	A	0.002577	0.02583	G	0.002725	0.09744
6	rs35555795	26509382	A	0.002577	0.02583	G	0.002725	0.09744
6	rs6923313	31241370	G	0.1366	0.205	A	0.002746	0.6135

5	rs7702331	72551134	G	0.2165	0.1492	A	0.002758	1.576
5	rs250430	139941228	A	0.1443	0.08932	G	0.002765	1.72
6	rs3094188	31142245	C	0.1263	0.1917	A	0.002771	0.6096
6	rs3094208	31090672	A	0.3918	0.48	G	0.002774	0.6977
3	rs2028574	191093175	T	0.518	0.4308	A	0.002785	1.42
3	rs4677728	191093310	G	0.518	0.4308	A	0.002785	1.42
2	rs76224543	240061403	A	0.01546	0.04917	G	0.002807	0.3038
12	rs7956151	14678507	A	0.5412	0.4533	C	0.002846	1.423
1	rs2144300	230294916	A	0.1005	0.1608	G	0.002902	0.5831
9	rs4742314	7124815	G	0.3015	0.3858	A	0.002933	0.6872
5	rs9314069	164877178	A	0.4278	0.3433	C	0.002935	1.43
3	rs191831854	37366667	G	0.01804	0.0025	A	0.002951	7.331
3	rs41298977	133472449	A	0.01804	0.0025	G	0.002951	7.331
14	rs115598823	32561778	A	0.01804	0.0025	G	0.002951	7.331
12	rs3751325	58335626	T	0.2758	0.3583	A	0.003016	0.6819
15	rs8023445	49192791	G	0.1804	0.2542	A	0.00306	0.6459
12	rs7957558	14688644	G	0.02835	0.0075	A	0.003062	3.861
12	rs6488695	14749972	A	0.02835	0.0075	G	0.003062	3.861
20	rs1535339	19820785	G	0.4974	0.4108	A	0.00318	1.419
2	rs291466	191184475	G	0.1985	0.135	A	0.003206	1.586
6	rs2235197	167709702	A	0.2345	0.1667	G	0.003224	1.532
6	rs12198173	32026808	A	0.03093	0.07083	G	0.003274	0.4186
6	rs12211410	32049423	A	0.03093	0.07083	G	0.003274	0.4186
6	rs872071	411064	G	0.2603	0.34	A	0.003284	0.6831
6	rs2239688	32054212	C	0.2371	0.1683	G	0.003295	1.536
17	rs9903137	71119820	A	0.5155	0.4283	G	0.003304	1.42
6	rs3095312	31090837	G	0.3918	0.4783	C	0.003351	0.7024
8	rs10956647	133202844	A	0.5232	0.4367	G	0.003359	1.416
8	rs150489215	10623104	A	0.03351	0.01	G	0.003362	3.432
17	rs2288156	8644854	A	0.2526	0.182	G	0.003376	1.519
6	rs241410	32861651	G	0.5232	0.4375	C	0.003394	1.411
16	rs2070832	71487206	G	0.2423	0.3217	A	0.003401	0.6742
18	rs117498128	42643270	A	0.005155	0.02917	C	0.00344	0.1725
20	rs6102389	40007970	G	0.2964	0.3783	A	0.003456	0.6922
21	rs2827383	23681095	G	0.4098	0.495	A	0.003479	0.7083
6	rs422951	32188383	G	0.1907	0.2642	A	0.003519	0.6565
2	rs11694156	12384394	G	0.01031	0	A	0.003522	NA
3	rs146985269	169802578	G	0.01031	0	A	0.003522	NA
8	rs55991828	48769846	A	0.01031	0	G	0.003522	NA
12	rs138380449	6649710	A	0.01031	0	G	0.003522	NA
10	rs6537616	135428246	A	0.4742	0.3892	G	0.003594	1.416
1	rs2275689	236721660	A	0.3969	0.315	G	0.003604	1.431
17	rs11079339	56270442	G	0.06959	0.1225	A	0.003607	0.5358

2	rs199917984	113069390	G	0.01546	0.001667	A	0.003643	9.408
2	rs147431919	219692747	A	0.01546	0.001667	G	0.003643	9.408
22	rs142215295	30776173	A	0.01546	0.001667	G	0.003643	9.408
16	rs7192086	13061611	A	0.3479	0.2692	T	0.003678	1.449
1	rs35634161	159409757	G	0.1108	0.06333	A	0.003685	1.843
6	rs6917033	42627434	A	0.4794	0.395	G	0.003686	1.41
6	rs3131854	29611885	A	0.1804	0.2525	G	0.003757	0.6517
6	rs2071653	29635928	A	0.1804	0.2525	G	0.003757	0.6517
13	rs9511451	25378476	G	0.1959	0.27	A	0.003764	0.6586
11	rs60912271	121519662	G	0.1675	0.2375	A	0.003823	0.6461
14	rs3949448	86378529	G	0.4407	0.3583	A	0.003869	1.411
16	rs12924729	11187783	A	0.2448	0.1767	G	0.00388	1.511
17	rs35489971	72700943	A	0.1985	0.2717	G	0.003881	0.6638
4	rs3796547	57181995	A	0.2371	0.3133	G	0.003964	0.6811
16	rs12708716	11179873	G	0.2474	0.1783	A	0.003981	1.515
1	rs12047155	50416930	G	0.03351	0.07417	A	0.003984	0.4327
6	rs9263873	31170713	A	0.3918	0.4767	G	0.004035	0.7071
6	rs9267546	31673436	A	0.08763	0.1442	G	0.004077	0.5702
1	rs148324464	20302261	A	0.01289	0.0008333	G	0.00409	15.65
8	rs200547556	63985514	G	0.01289	0.0008333	A	0.00409	15.65
4	rs3733391	156289917	A	0.2629	0.1933	G	0.004132	1.488
8	rs7004633	89760311	G	0.1881	0.26	A	0.004163	0.6596
8	rs9693212	138467144	A	0.433	0.5175	G	0.004192	0.712
1	rs2276405	159043196	A	0.09021	0.04917	G	0.004243	1.917
14	rs2302588	73404752	G	0.1753	0.1175	C	0.004318	1.596
14	rs3815460	73422259	C	0.1753	0.1175	G	0.004318	1.596
12	rs148699934	112130610	A	0.02577	0.006667	G	0.004322	3.942
16	rs2903692	11238783	A	0.2397	0.1725	G	0.004497	1.512
16	rs117687498	10576102	A	0.0232	0.05833	G	0.0045	0.3833
6	rs2239842	33261871	G	0.1521	0.2183	A	0.004529	0.642
16	rs2217332	56969148	A	0.1469	0.09417	G	0.00453	1.657
2	rs964595	150000597	A	0.4871	0.405	G	0.004608	1.395
6	rs3130952	31177915	G	0.0567	0.1033	A	0.004631	0.5216
6	rs4947328	31561747	G	0.3505	0.2733	A	0.00467	1.435
4	rs114085656	40245517	A	0.02062	0.004167	G	0.004686	5.032
5	rs185327147	101795417	A	0.02062	0.004167	G	0.004686	5.032
6	rs55987614	32261790	G	0.02062	0.004167	A	0.004686	5.032
16	rs200760311	16205379	A	0.02062	0.004167	G	0.004686	5.032
1	rs77542170	45797760	G	0.03866	0.01333	A	0.004692	2.976
11	rs10750931	60059810	G	0.1314	0.08083	A	0.004696	1.721
6	rs3094663	31107087	A	0.2165	0.2892	G	0.004777	0.6792
12	rs11062615	3430559	G	0.3273	0.4083	A	0.004832	0.7051
6	rs10885	31604591	A	0.04663	0.09015	G	0.004852	0.4937

6	rs3132451	31582025	C	0.04639	0.09083	G	0.004873	0.4869
6	rs3130070	31591808	G	0.04639	0.09083	A	0.004873	0.4869
6	rs11229	31603770	G	0.04639	0.09083	A	0.004873	0.4869
6	rs3117583	31619576	G	0.04639	0.09083	A	0.004873	0.4869
6	rs3130618	31632134	A	0.04639	0.09083	C	0.004873	0.4869
6	rs2142234	31639129	A	0.04639	0.09083	G	0.004873	0.4869
14	rs12590500	47426637	A	0.1701	0.1133	C	0.004905	1.604
16	rs8047014	69135049	C	0.384	0.3058	A	0.004987	1.415
1	rs545671	57760458	A	0.5438	0.4608	G	0.005	1.395
8	rs6472810	74829717	G	0.2964	0.375	A	0.005011	0.7021
6	rs2853939	31250642	A	0.1134	0.1733	G	0.005047	0.61
6	rs2853933	31254088	A	0.1134	0.1733	G	0.005047	0.61
6	rs2524040	31257625	A	0.1134	0.1733	G	0.005047	0.61
6	rs2524163	31259579	G	0.1134	0.1733	A	0.005047	0.61
6	rs2243868	31261276	A	0.1134	0.1733	G	0.005047	0.61
6	rs3130412	31216262	G	0.4253	0.5075	A	5.05E-03	0.718
12	rs155694	130056480	C	0.4253	0.5075	A	0.005049	0.718
6	rs190573609	137814692	G	0.04124	0.01583	A	0.005052	2.673
7	rs17547816	48311658	A	0.03093	0.009167	G	0.005064	3.45
16	rs8051898	60878008	A	0.384	0.3067	G	0.005066	1.409
16	rs79527462	28631383	G	0.1005	0.0575	C	0.005122	1.832
12	rs7956759	69566855	C	0.3428	0.4242	A	0.005124	0.7081
10	rs7099034	95545378	G	0.1598	0.1058	A	0.005189	1.607
6	rs1540771	466033	A	0.2268	0.3	G	0.005257	0.6844
16	rs1800566	69745145	A	0.4716	0.3908	G	0.005277	1.391
19	rs12977303	44377669	G	0.3067	0.385	A	0.005309	0.7067
6	rs211476	33319596	C	0.3093	0.389	A	0.005332	0.7034
21	rs1006899	15845052	G	0.01804	0.05	A	0.005334	0.3491
12	rs1006737	2345295	A	0.01804	0.04917	G	0.005355	0.3553
6	rs210152	33515520	G	0.1959	0.1358	A	0.005379	1.55
6	rs3117324	33324527	A	0.3093	0.3875	C	0.005383	0.7078
2	rs11424	65496018	G	0.1778	0.2467	C	0.005425	0.6606
6	rs2018501	32856208	A	0.3634	0.4442	G	0.005471	0.7144
3	rs5028811	141845000	C	0.3995	0.3208	A	0.005491	1.408
6	rs211456	33389381	C	0.285	0.362	A	0.005537	0.7023
7	rs3735318	149152906	G	0.2371	0.1717	A	0.005551	1.5
1	rs296547	200892137	G	0.4407	0.3617	A	0.005689	1.391
4	rs2011741	156299620	A	0.3479	0.2733	C	0.005704	1.419
3	rs724016	141105570	G	0.2887	0.3658	A	0.005743	0.7034
6	rs2296748	33745071	G	0.1985	0.2683	A	0.005745	0.6751
19	rs145036301	11624714	G	0.007732	0.0325	A	0.005766	0.232
3	rs17043638	17486643	G	0.3273	0.2542	A	0.005807	1.428
17	rs10512597	72699833	A	0.201	0.2708	G	0.005917	0.6774

13	rs9530435	75993887	A	0.007732	0.03333	G	0.005977	0.226
6	rs2228628	32088854	G	0.1443	0.2067	C	0.006054	0.6475
7	rs3807375	150667210	G	0.1443	0.2067	A	0.006054	0.6475
6	rs3095245	31213902	G	0.4588	0.3792	A	0.006055	1.388
1	rs1041937	236702210	A	0.4149	0.3375	G	0.006087	1.392
3	rs800082	144340205	A	0.4175	0.3392	G	0.006093	1.397
10	rs143874488	35500694	A	0.0232	0.005833	C	0.006106	4.047
11	rs150074663	117050120	C	0.0232	0.005833	G	0.006106	4.047
12	rs117728539	16347327	A	0.0232	0.005833	C	0.006106	4.047
16	rs5736	23200921	A	0.0232	0.005833	G	0.006106	4.047
21	rs4356739	29335827	A	0.3428	0.4225	G	0.006128	0.7129
15	rs7497036	74873679	G	0.4639	0.3842	A	0.006153	1.387
9	rs10813831	32526146	A	0.1053	0.06187	G	0.006171	1.784
1	rs4660939	46944805	A	0.3479	0.4267	G	0.00626	0.717
6	rs2853922	31266190	A	0.1134	0.1708	G	0.006295	0.6208
6	rs2524089	31266522	C	0.1134	0.1708	A	0.006295	0.6208
14	rs977975	83724760	G	0.4253	0.3467	A	0.006321	1.394
8	rs61736163	30702785	G	0.03608	0.07417	A	0.006379	0.4673
20	rs6052095	3803446	G	0.2758	0.3508	A	0.006384	0.7046
6	rs887464	31145920	A	0.4768	0.3967	G	0.006387	1.386
5	rs168726	180276402	G	0.01804	0.003333	A	0.006423	5.493
12	rs142121386	133780554	G	0.01804	0.003333	A	0.006423	5.493
19	rs202071873	16666033	A	0.01804	0.003333	G	0.006423	5.493
19	rs191321109	36530421	A	0.01804	0.003333	G	0.006423	5.493
19	rs141622048	38900728	G	0.01804	0.003333	C	0.006423	5.493
5	rs78068171	179707528	G	0	0.01583	A	0.006468	0
6	rs41543814	31239430	G	0.2294	0.3017	A	6.48E-03	0.6891
6	rs118032130	26056310	C	0.002577	0.0225	G	0.00649	0.1123
9	rs602990	136643994	G	0.002577	0.0225	A	0.00649	0.1123
6	rs2524094	31240041	G	0.1366	0.1975	A	6.49E-03	0.6428
6	rs1050458	31324925	A	0.08763	0.14	G	0.006522	0.59
1	rs61743961	63113968	G	0.08763	0.1408	A	0.006539	0.5859
19	rs200561773	40889743	A	0.03109	0.01	G	0.006551	3.176
16	rs3784962	83757328	G	0.3943	0.3183	A	0.006552	1.394
11	rs498573	128054642	A	0.1263	0.1858	G	0.006604	0.6333
6	rs3749981	32862682	A	0.1134	0.06833	G	0.006609	1.744
8	rs263238	131812033	A	0.366	0.2917	G	0.006613	1.402
6	rs1419881	31130593	G	0.3711	0.4508	A	0.006642	0.7189
7	rs7810629	96989624	G	0.4897	0.4108	A	0.00669	1.376
11	rs113929032	551708	A	0.03093	0.01	G	0.006704	3.16
17	rs200294882	78082587	G	0.03093	0.01	A	0.006704	3.16
2	rs10175058	162276981	A	0.2835	0.3583	G	0.006709	0.7085
18	rs12967709	35921514	G	0.09021	0.05083	A	0.006732	1.851

13	rs1041073	31891746	G	0.1649	0.23	A	0.006757	0.6613
15	rs12913514	60363768	A	0.5	0.4208	G	0.006809	1.376
6	rs2281820	33768897	A	0.09794	0.1517	G	0.00685	0.6073
5	rs4419568	101344133	A	0.5077	0.4275	G	0.006855	1.381
17	rs9635769	33288363	G	0.2062	0.1467	A	0.006859	1.511
19	rs2274896	34712618	A	0.02062	0.0525	G	0.006876	0.3799
2	rs6643	85824251	G	0.2603	0.3342	A	0.006881	0.7012
11	rs901827	68030015	A	0.3299	0.4075	G	0.00698	0.7158
19	rs6791	7712277	A	0.2088	0.1483	G	0.007064	1.515
12	rs10747556	49308284	A	0.4098	0.4883	G	0.007145	0.7275
6	rs2857595	31568469	G	0.433	0.5125	A	0.007184	0.7264
6	rs2523638	31344273	G	0.5593	0.48	A	0.007202	1.375
9	rs1053318	35056961	A	0.04897	0.09083	C	0.007244	0.5154
16	rs58542872	1872922	G	0.04897	0.09083	C	0.007244	0.5154
10	rs10741164	130541770	C	0.03351	0.07083	A	0.00725	0.4547
4	rs2102219	23036522	A	0.299	0.375	G	0.007253	0.7108
6	rs9267673	31883679	A	0.04897	0.09167	G	0.007256	0.5102
2	rs6734849	143743012	A	0.3531	0.28	C	0.007277	1.404
12	rs10747083	133041618	G	0.1314	0.1917	A	0.007284	0.6382
2	rs1348850	178418575	A	0.4588	0.3817	G	0.007366	1.373
6	rs4713505	32105001	A	0.1443	0.2058	C	0.007388	0.6508
2	rs4426527	241817516	G	0.116	0.07083	A	0.007429	1.721
6	rs8111	32083175	A	0.1443	0.205	G	0.00743	0.6541
6	rs9386806	109764222	A	0.05412	0.025	G	0.007434	2.232
8	rs74990397	10470068	A	0.09278	0.05268	G	0.007491	1.839
6	rs3095273	29566369	A	0.01804	0.04833	G	0.007498	0.3618
3	rs13081855	99481539	A	0.04381	0.01833	C	0.007649	2.454
7	rs13232179	151120948	A	0.3582	0.4356	T	0.007649	0.7232
5	rs2304035	168176517	A	0.3582	0.4357	G	0.007654	0.7229
12	rs1878022	108699032	G	0.3093	0.3842	A	0.007677	0.7178
6	rs3130780	30874308	A	0.08505	0.1367	C	0.007679	0.5872
6	rs2253705	30900094	A	0.08505	0.1367	G	0.007679	0.5872
1	rs78489282	160121971	A	0.02835	0.008333	G	0.007711	3.472
10	rs74846600	127555701	A	0.02835	0.008333	G	0.007711	3.472
16	rs1126960	54318172	A	0.02835	0.008333	C	0.007711	3.472
20	rs118040899	10030508	G	0.02835	0.008333	A	0.007711	3.472
22	rs118163237	24919647	A	0.02835	0.008333	G	0.007711	3.472
6	rs4713305	30252836	A	0.2938	0.225	T	0.007731	1.433
6	rs11967	30313268	A	0.2938	0.225	G	0.007731	1.433
11	rs4282961	6554941	A	0.2113	0.1525	C	0.007785	1.489
4	rs10009024	97644876	G	0.1134	0.17	A	0.007809	0.6245
2	rs1881881	123176322	G	0.4871	0.41	A	0.007963	1.367
20	rs965469	3381549	G	0.1366	0.1967	A	0.007966	0.6462

17	rs1052586	45018463	A	0.4784	0.4	G	0.008019	1.376
8	rs11776767	10683929	C	0.201	0.1433	G	0.008077	1.504
11	rs7120118	47286290	A	0.3402	0.2692	G	0.008077	1.4
5	rs57586624	132074695	G	0.2139	0.2817	A	0.008183	0.694
16	rs3213646	20810067	G	0.08763	0.1383	A	0.00822	0.5983
11	rs77873429	7673029	G	0.07216	0.03833	A	0.008241	1.951
11	rs77975665	7673049	G	0.07216	0.03833	C	0.008241	1.951
11	rs61733056	7690873	A	0.07216	0.03833	G	0.008241	1.951
6	rs2294748	29501182	A	0.005155	0.0275	G	0.008254	0.1832
9	rs10746839	90883630	G	0.03093	0.06583	A	0.008264	0.4529
15	rs920915	58688467	G	0.1649	0.2275	C	0.008264	0.6707
1	rs480299	53320274	A	0.1804	0.2458	C	0.008268	0.6753
1	rs2294508	54354658	C	0.2603	0.3317	A	0.008278	0.7091
16	rs4275849	69832105	A	0.5155	0.4375	G	0.008302	1.368
13	rs1336163	45090478	C	0.3711	0.2983	A	0.008319	1.388
13	rs9554742	101596542	G	0.3969	0.4742	A	0.00832	0.7298
3	rs833660	62552794	A	0.2809	0.2142	G	0.008326	1.434
6	rs2523656	31444048	G	0.4404	0.5192	C	0.008409	0.7289
11	rs3802746	68030173	C	0.4098	0.4875	A	0.008424	0.7299
12	rs4760600	49314994	A	0.4124	0.49	G	0.008449	0.7304
9	rs10758029	31163494	A	0.5284	0.4508	C	0.008456	1.365
14	rs2232700	94756450	T	0.3351	0.4108	A	0.008469	0.7226
2	rs283852	79332485	G	0.5335	0.4558	A	0.008481	1.365
19	rs1050457	731144	G	0.5335	0.4558	A	0.008481	1.365
17	rs4790706	4689572	G	0.4098	0.4867	A	0.008488	0.7324
6	rs1265109	31119589	C	0.4124	0.4892	A	0.008519	0.7328
4	rs10050156	187775639	G	0.4278	0.5058	A	0.008522	0.7305
6	rs3130100	33283766	G	0.2655	0.3383	A	0.008522	0.7068
13	rs7327180	57464113	A	0.4356	0.5134	G	0.008538	0.7315
1	rs74853154	12336785	A	0.007732	0.03083	G	0.008575	0.2449
6	rs9384878	113975118	G	0.3376	0.4125	A	0.008612	0.726
10	rs145521001	96117066	A	0.02062	0.005	G	0.008634	4.189
20	rs138598490	3845179	A	0.02062	0.005	G	0.008634	4.189
6	rs211474	33320613	G	0.299	0.3733	A	0.008641	0.7159
3	rs6779930	177707225	C	0.4562	0.38	A	0.008656	1.369
7	rs7778801	13900513	G	0.4562	0.38	A	0.008656	1.369
11	rs3741208	2169774	A	0.1546	0.2158	G	0.008669	0.6646
1	rs118096304	165652343	A	0.01546	0.0025	G	0.008689	6.267
9	rs116055369	125682009	A	0.01546	0.0025	G	0.008689	6.267
11	rs79628679	63885697	G	0.01546	0.0025	A	0.008689	6.267
13	rs115260428	24243081	G	0.01546	0.0025	A	0.008689	6.267
12	rs60284450	113645916	A	0.1186	0.1742	G	0.008703	0.6378
3	rs347685	141807137	C	0.3789	0.3058	A	0.008715	1.384

18	rs569629	7329035	A	0.3789	0.3058	G	0.008715	1.384
7	rs4580937	1478610	A	0.01031	0.035	G	0.008725	0.2872
7	rs61287564	1479659	G	0.01031	0.035	A	0.008725	0.2872
8	rs949493	74527996	G	0.01031	0.035	A	0.008725	0.2872
9	rs2275420	35660901	A	0.01031	0.03583	G	0.008806	0.2803
6	rs469064	33250476	A	0.2448	0.3158	C	0.008814	0.7024
6	rs2395153	32345595	C	0.1856	0.25	G	0.008816	0.6835
5	rs6892901	98694849	A	0.4639	0.3875	C	0.00885	1.368
6	rs2281390	33059669	A	0.06701	0.1125	C	0.00893	0.5666
17	rs11652709	56271094	C	0.06701	0.1133	G	0.008945	0.5619
11	rs3794153	8751889	C	0.4691	0.3933	G	0.009045	1.363
14	rs1760904	20852029	A	0.3582	0.4333	G	0.009153	0.73
10	rs35472668	28228926	A	0.1598	0.2208	G	0.009338	0.671
4	rs788908	73414286	G	0.2294	0.2975	A	0.009357	0.7029
2	rs2943650	227105921	G	0.02835	0.0626	A	0.009387	0.4369
6	rs972275	127391844	C	0.3737	0.45	G	0.009431	0.7293
6	rs2071538	32818678	A	0.04897	0.08917	G	0.009471	0.526
14	rs61754460	31763142	C	0.1727	0.1192	A	0.009501	1.543
14	rs17124581	88826682	G	0.232	0.3008	A	0.009527	0.7019
6	rs1264569	30365320	G	0.02062	0.05083	A	0.009556	0.3931
1	rs10489615	230304988	G	0.1933	0.2583	A	0.009569	0.6879
20	rs6063641	49989435	G	0.4923	0.4167	A	0.009571	1.357
3	rs6780771	59475736	G	0.3814	0.4567	A	0.009641	0.7337
5	rs2278329	38921788	A	0.2577	0.3283	G	0.00965	0.7103
6	rs4714598	42282393	A	0.1134	0.1683	G	0.009662	0.6319
11	rs2167079	47270255	G	0.3376	0.2683	A	0.009668	1.39
9	rs274935	23487566	G	0.4381	0.3633	A	0.009695	1.366
9	rs2778031	90835726	G	0.2577	0.3275	A	0.009695	0.713
6	rs2523554	31331829	G	0.06959	0.1158	A	0.009737	0.5709
6	rs453590	33403422	G	0.2887	0.3608	A	0.009755	0.7188
9	rs7864699	137724371	G	0.2887	0.3608	A	0.009755	0.7188
1	rs6658555	113098534	A	0.04381	0.08333	G	0.009764	0.504
22	rs757660	30793137	A	0.4407	0.3658	G	0.009784	1.366
10	rs1983864	100017453	A	0.3969	0.4725	C	0.009858	0.7347
17	rs35999669	72240177	C	0.1418	0.09333	A	0.009889	1.604
21	rs369908	42767133	G	0.518	0.4417	A	0.009899	1.359
19	rs201231250	18729028	A	0.02835	0.009167	G	0.009923	3.154
22	rs1801198	31011610	G	0.5206	0.445	C	0.009989	1.354
2	rs6753459	191064753	G	0.5258	0.45	A	0.01002	1.355
6	rs2596472	31428967	G	0.09585	0.1475	A	0.01002	0.6127
8	rs751019	27308585	C	0.2938	0.3658	A	0.01002	0.7212
2	rs13306187	21236085	A	0.002577	0.02083	G	0.01008	0.1214
2	rs7594935	235255122	A	0.002577	0.02083	G	0.01008	0.1214

20	rs2426689	55369594	G	0.4407	0.5167	A	0.01015	0.7372
4	rs10518608	133038675	A	0.5567	0.4808	G	0.01016	1.356
20	rs5741804	36952342	A	0.1521	0.2125	G	0.01016	0.6646
17	rs9895661	59456589	G	0.433	0.5083	A	0.0102	0.7386
22	rs79314756	51176734	A	0.06186	0.03167	G	0.0102	2.016
2	rs145948549	228159737	A	0.0232	0.006667	G	0.01022	3.538
15	rs7181742	42643529	G	0.1068	0.1608	A	0.01024	0.6237
13	rs55860328	114897534	A	0.4072	0.335	G	0.01029	1.364
2	rs13023962	1271230	G	0.268	0.3383	A	0.01032	0.7162
11	rs1449627	47290984	A	0.4124	0.3392	C	0.01042	1.367
11	rs326214	47298360	A	0.4124	0.3392	G	0.01042	1.367
2	rs144182857	113820031	A	0.03608	0.01417	G	0.01048	2.605
6	rs2517862	29821937	A	0.05155	0.02417	G	0.01048	2.195
6	rs1349809	67717251	A	0.03608	0.01417	G	0.01048	2.605
6	rs6928399	31195218	A	0.4124	0.34	G	0.01052	1.362
12	rs1141509	14720554	A	0.2448	0.3133	C	0.01053	0.7106
1	rs111933693	1309185	G	0	0.01515	A	0.01057	0
7	rs963323	12410112	G	0.0799	0.1283	A	0.01057	0.5898
1	rs146506266	1322735	A	0	0.015	G	0.01058	0
4	rs13127141	149748096	G	0.4613	0.3875	A	0.01058	1.354
11	rs185836777	76796077	A	0	0.015	G	0.01058	0
11	rs79839754	130340817	C	0	0.015	A	0.01058	0
12	rs1800203	21531247	G	0	0.015	A	0.01058	0
6	rs213202	33232055	G	0.2474	0.3167	A	0.01071	0.7094
6	rs455567	33252115	A	0.2474	0.3167	G	0.01071	0.7094
11	rs2227988	119156193	A	0.01804	0.0475	G	0.01075	0.3684
15	rs75780940	51696856	A	0.1649	0.1142	G	0.01077	1.533
11	rs878874	94588465	G	0.3531	0.2842	A	0.01091	1.375
5	rs4460176	109496960	C	0.3557	0.2858	A	0.01096	1.379
6	rs400547	31702710	A	0.04639	0.085	G	0.01098	0.5237
6	rs707915	31710968	A	0.04639	0.085	T	0.01098	0.5237
6	rs707936	31733650	A	0.04639	0.085	G	0.01098	0.5237
1	rs9804140	223947081	G	0.1443	0.09667	A	0.01099	1.576
14	rs1760903	20852817	G	0.3634	0.4367	A	0.01099	0.7364
2	rs16867245	10059844	A	0.2655	0.2033	G	0.011	1.416
6	rs9267532	31639979	A	0.04639	0.08583	G	0.011	0.5181
8	rs79394014	52366156	A	0.04639	0.08583	G	0.011	0.5181
12	rs4760332	58222672	C	0.1727	0.2342	A	0.011	0.6826
6	rs4708164	75479295	C	0.4794	0.405	A	0.01101	1.353
2	rs6706577	111796833	G	0.3686	0.4433	A	0.01103	0.7329
6	rs6921663	31154620	G	0.4253	0.3525	A	0.01106	1.359
6	rs4122189	31167927	A	0.4253	0.3525	G	0.01106	1.359
2	rs1046356	180810202	A	0.3067	0.2408	G	0.01108	1.394

22	rs13058434	26164413	A	0.3866	0.3158	G	0.01109	1.365
6	rs3106190	33280629	G	0.2526	0.3208	C	0.01113	0.7154
6	rs3106189	33282002	A	0.2526	0.3208	G	0.01113	0.7154
12	rs2292303	102513531	G	0.3582	0.2883	C	0.01114	1.378
5	rs390299	153363334	G	0.1675	0.1158	A	0.01115	1.536
5	rs2578377	153413390	G	0.1675	0.1158	A	0.01115	1.536
6	rs1079991	30377860	A	0.03093	0.06417	G	0.01115	0.4655
4	rs10938397	45182527	G	0.3325	0.2642	A	0.01119	1.387
6	rs3131296	32172993	A	0.03109	0.06594	G	0.01131	0.4545
10	rs35242712	28224075	A	0.1606	0.22	G	0.01134	0.6785
1	rs10918767	167763804	C	0.3918	0.3208	A	0.01137	1.363
17	rs7212197	48335925	A	0.3918	0.3208	G	0.01137	1.363
4	rs17064676	178272700	G	0.06443	0.03333	A	0.01143	1.997
6	rs6906846	31245736	A	0.134	0.1908	G	0.01143	0.6562
1	rs191016322	7863869	A	0.01289	0.001667	G	0.01145	7.82
4	rs144381760	89314687	A	0.01289	0.001667	G	0.01145	7.82
8	rs76824642	23115562	A	0.01289	0.001667	G	0.01145	7.82
9	rs201953164	6007224	A	0.01289	0.001667	C	0.01145	7.82
9	rs140782366	6014071	G	0.01289	0.001667	A	0.01145	7.82
9	rs201291561	138713322	G	0.01289	0.001667	A	0.01145	7.82
16	rs186046750	4484435	C	0.01289	0.001667	G	0.01145	7.82
22	rs200280373	19050750	A	0.01289	0.001667	G	0.01145	7.82
5	rs11955098	159129186	C	0.1804	0.1275	A	0.01152	1.506
12	rs10774978	110341933	G	0.1572	0.1075	A	0.01153	1.549
17	rs391300	2216258	A	0.3376	0.2692	G	0.01163	1.384
16	rs391525	85944439	G	0.2603	0.3292	A	0.01167	0.7172
21	rs1022424	42047446	G	0.4381	0.3658	A	0.01168	1.352
6	rs2249464	30888161	A	0.1778	0.2392	G	0.0117	0.6881
6	rs753725	30890871	A	0.1778	0.2392	G	0.0117	0.6881
6	rs2532934	30894759	G	0.1778	0.2392	A	0.0117	0.6881
1	rs3093059	159685136	G	0.1933	0.1392	A	0.01171	1.482
1	rs11265260	159700039	G	0.1933	0.1392	A	0.01171	1.482
6	rs1571767	155577865	A	0.116	0.07417	T	0.01174	1.638
4	rs10017284	121513215	G	0.2969	0.3675	A	0.01177	0.7267
15	rs17733968	95678494	G	0.2732	0.21	A	0.01177	1.414
4	rs7694926	84580859	A	0.4072	0.4808	G	0.01183	0.7417
3	rs2593321	22122396	A	0.1804	0.1283	G	0.01186	1.495
5	rs6880853	146482041	G	0.2938	0.2291	A	0.01188	1.4
6	rs2229638	33648228	G	0.02577	0.0075	A	0.01188	3.501
15	rs150763255	40913033	A	0.02577	0.0075	G	0.01188	3.501
16	rs186611345	4949345	A	0.02577	0.0075	T	0.01188	3.501
4	rs11556167	152682046	A	0.0232	0.05417	C	0.01196	0.4147
1	rs10493340	63591129	G	0.005155	0.025	A	0.01197	0.2021

3	rs6788567	112437047	C	0.4433	0.5183	A	0.01197	0.74
6	rs211455	33328518	G	0.2964	0.3667	A	0.01203	0.7276
6	rs389883	31947460	C	0.3428	0.2742	A	0.01207	1.381
17	rs9912203	41445145	A	0.3428	0.2742	G	0.01207	1.381
4	rs6554318	56621548	A	0.4407	0.5142	G	0.0121	0.7446
2	rs149596493	152410418	A	0.01804	0.004167	G	0.01222	4.391
3	rs4986984	37053562	A	0.01804	0.004167	G	0.01222	4.391
7	rs55679316	73523347	A	0.01804	0.004167	G	0.01222	4.391
12	rs3809199	12630669	A	0.01804	0.004167	G	0.01222	4.391
12	rs200660845	101740361	A	0.01804	0.004167	G	0.01222	4.391
12	rs5745068	133202740	A	0.01804	0.004167	G	0.01222	4.391
17	rs2292969	80899349	G	0.01804	0.004167	A	0.01222	4.391
20	rs201064818	21494200	A	0.01804	0.004167	C	0.01222	4.391
6	rs2070600	32151443	A	0.2088	0.1533	G	0.01223	1.457
6	rs2395449	33728755	T	0.2191	0.2838	A	0.01224	0.7079
8	rs6981828	130004216	A	0.2423	0.3092	G	0.01229	0.7144
14	rs141696238	102506647	A	0.005155	0.02583	G	0.01229	0.1954
1	rs34868416	161697319	A	0.04897	0.08833	G	0.0123	0.5314
3	rs62264721	126191011	A	0.03351	0.0125	G	0.01237	2.739
19	rs2240932	44103010	A	0.03351	0.0125	G	0.01237	2.739
21	rs201713860	47635212	A	0.03351	0.0125	G	0.01237	2.739
17	rs8073783	51237941	A	0.2603	0.1983	G	0.01249	1.422
12	rs11068997	110383141	A	0.1392	0.0925	G	0.01253	1.586
12	rs2287174	110474070	G	0.1392	0.0925	A	0.01253	1.586
9	rs117748510	20923696	C	0.05412	0.02583	G	0.01266	2.158
7	rs150584875	112424814	A	0.02835	0.06	G	0.01267	0.4571
12	rs16940029	109617728	G	0.05412	0.02588	A	0.0127	2.154
19	rs34415062	59023166	A	0.04381	0.08097	G	0.0127	0.5201
19	rs35350976	59023174	G	0.04381	0.08167	A	0.01277	0.5153
6	rs610913	74155346	A	0.4691	0.3967	C	0.01279	1.344
16	rs3852738	82713551	G	0.007732	0.03	A	0.01284	0.2519
2	rs77546992	168041087	A	0.08505	0.05	G	0.01288	1.766
7	rs12672205	123508328	G	0.08763	0.1358	A	0.0129	0.611
8	rs451034	50808334	G	0.1418	0.1975	A	0.01294	0.6711
3	rs34762726	49689210	A	0.03866	0.07417	G	0.01296	0.502
3	rs9822268	49719729	A	0.03866	0.07417	G	0.01296	0.502
3	rs3197999	49721532	A	0.03866	0.07417	G	0.01296	0.502
6	rs213204	33241076	C	0.25	0.3167	A	0.01298	0.7193
6	rs3130503	31137165	A	0.1881	0.25	G	0.01307	0.6952
4	rs2348164	160341351	A	0.1495	0.1025	C	0.01311	1.539
7	rs197367	36445856	G	0.1881	0.2492	A	0.01313	0.6983
22	rs9617090	50439194	A	0.384	0.315	G	0.01313	1.356
15	rs1471225	29888396	G	0.317	0.3875	A	0.01314	0.7337

9	rs3818764	113276356	C	0.1186	0.1708	G	0.01317	0.6528
6	rs4713506	32113980	A	0.1572	0.215	G	0.01322	0.6811
21	rs2277798	43824106	G	0.4871	0.4142	A	0.01324	1.343
6	rs12660883	30764420	G	0.4278	0.3567	A	0.01331	1.349
10	rs3182535	3200292	G	0.3789	0.4508	A	0.01335	0.743
10	rs4242746	3202065	A	0.3789	0.4508	G	0.01335	0.743
4	rs10937615	5023112	G	0.04897	0.0225	A	0.01338	2.237
4	rs11941467	184190233	A	0.1211	0.07833	C	0.01341	1.622
5	exm-IND5-7620	76173625	D	0.02835	0.01	I	0.01342	2.889
6	rs2071288	32149260	A	0.02835	0.01	G	0.01342	2.889
17	rs117052455	65105513	G	0.02835	0.01	A	0.01342	2.889
22	rs190749586	20761028	G	0.02835	0.01	A	0.01342	2.889
3	rs11924930	195928854	A	0.1314	0.08667	G	0.01356	1.595
16	rs4783718	69547741	G	0.3866	0.4583	A	0.01356	0.7448
1	rs2901785	174104743	G	0.3273	0.3983	A	0.01363	0.735
4	rs2347132	110791413	A	0.2113	0.275	G	0.01364	0.7065
17	rs7208487	37543449	C	0.1753	0.235	A	0.01365	0.6918
6	rs2523578	31328542	G	0.05155	0.09083	A	0.01367	0.544
16	rs2745098	1536535	G	0.5052	0.4325	A	0.01367	1.339
2	rs45523133	31611143	A	0.0799	0.04583	G	0.01374	1.808
14	rs12434716	34269167	G	0.04381	0.01917	C	0.01375	2.345
6	rs10455097	74493432	A	0.5206	0.4475	C	0.01388	1.341
10	rs12411775	28294586	C	0.5206	0.4475	A	0.01388	1.341
6	rs3132496	31208610	C	0.4098	0.4825	A	0.01389	0.7447
1	rs2235302	169580290	A	0.1469	0.2042	G	0.01391	0.6712
6	rs213220	33202640	G	0.1469	0.2042	A	0.01391	0.6712
6	rs2395402	33753673	G	0.1108	0.1625	A	0.01394	0.6424
2	rs10204586	35406207	A	0.4407	0.37	C	0.01398	1.342
8	rs3812447	144671956	A	0.1649	0.1154	G	0.01398	1.514
11	rs1193851	65386206	G	0.1314	0.0875	C	0.01402	1.578
6	rs211449	33333916	C	0.2629	0.33	A	0.01406	0.7241
3	rs709428	109213239	A	0.4253	0.4975	G	0.0141	0.7473
21	rs2834060	34375854	A	0.4175	0.4892	G	0.01411	0.7486
9	rs3217992	22003223	A	0.5387	0.4667	G	0.01417	1.334
1	rs150173822	54562033	A	0.01031	0.0008333	G	0.0142	12.49
1	rs199561187	67507941	G	0.01031	0.0008333	A	0.0142	12.49
1	rs184817147	183190045	A	0.01031	0.0008333	G	0.0142	12.49
2	rs77631323	143959734	G	0.01031	0.0008333	A	0.0142	12.49
3	rs201252242	121350802	A	0.01031	0.0008333	G	0.0142	12.49
4	rs201382853	88414795	G	0.01031	0.0008333	A	0.0142	12.49
6	rs190279776	83898373	A	0.01031	0.0008333	C	0.0142	12.49
7	rs4148725	117307076	A	0.01031	0.0008333	G	0.0142	12.49
12	rs75259413	124298332	G	0.01031	0.0008333	A	0.0142	12.49

13	rs1372085	25008588	A	0.01031	0.0008333	G	0.0142	12.49
13	rs1822135	25008630	A	0.01031	0.0008333	G	0.0142	12.49
13	rs9511259	25009099	A	0.01031	0.0008333	C	0.0142	12.49
14	rs139972787	94394792	A	0.01031	0.0008333	G	0.0142	12.49
15	rs61741253	78825825	A	0.01031	0.0008333	G	0.0142	12.49
16	rs200902675	31367998	A	0.01031	0.0008333	C	0.0142	12.49
16	rs202161040	83998684	A	0.01031	0.0008333	G	0.0142	12.49
17	rs200267938	39041107	A	0.01031	0.0008333	G	0.0142	12.49
18	rs186428920	47367762	G	0.01031	0.0008333	C	0.0142	12.49
19	rs183964084	57985009	C	0.01031	0.0008333	A	0.0142	12.49
20	rs187507776	5579455	A	0.01031	0.0008333	G	0.0142	12.49
13	rs1801243	52548140	C	0.4433	0.515	A	0.01426	0.7499
20	rs143384	34025756	G	0.3119	0.2483	A	0.01433	1.372
10	rs201157692	99332519	A	0.007772	0	G	0.01439	NA
19	rs3745249	10670992	A	0.1495	0.2058	G	0.01439	0.6781
1	rs3737875	203140671	A	0.4046	0.335	G	0.0144	1.349
2	rs2218603	167472743	A	0.4046	0.335	G	0.0144	1.349
11	rs7926933	58978684	A	0.1495	0.2067	G	0.01441	0.6747
14	rs141174151	105930437	A	0.007772	0	G	0.01444	NA
6	rs3130785	30796738	A	0.06701	0.11	G	0.01445	0.5811
1	rs34192549	16464489	A	0.007732	0	G	0.0145	NA
1	rs150541088	41483537	A	0.007732	0	G	0.0145	NA
2	rs141992347	131520034	A	0.007732	0	G	0.0145	NA
2	rs188114756	174047614	G	0.007732	0	A	0.0145	NA
2	rs114861790	234959653	A	0.007732	0	G	0.0145	NA
3	rs150778110	52236528	G	0.007732	0	A	0.0145	NA
5	rs73730745	195323	A	0.007732	0	G	0.0145	NA
5	rs187669648	74865291	G	0.007732	0	A	0.0145	NA
6	rs1202199	20156174	A	0.007732	0	G	0.0145	NA
6	rs148231017	80732137	A	0.007732	0	G	0.0145	NA
6	rs146446780	88321875	A	0.007732	0	G	0.0145	NA
8	rs11538152	17928855	A	0.007732	0	G	0.0145	NA
8	rs182075508	141756950	A	0.007732	0	G	0.0145	NA
10	rs114108618	52008258	G	0.007732	0	A	0.0145	NA
11	rs149901958	66834232	A	0.007732	0	G	0.0145	NA
12	rs78739424	19282743	A	0.007732	0	G	0.0145	NA
13	rs193097418	103514060	G	0.007732	0	A	0.0145	NA
14	rs2295908	24108198	A	0.007732	0	G	0.0145	NA
14	rs192960670	56084687	A	0.007732	0	G	0.0145	NA
16	rs145498856	67322413	A	0.007732	0	G	0.0145	NA
19	rs10417589	55106663	G	0.007732	0	A	0.0145	NA
20	rs144351024	3553472	A	0.007732	0	G	0.0145	NA
22	rs138558620	23492340	A	0.007732	0	G	0.0145	NA

6	rs915660	31143835	G	0.4046	0.3358	C	0.01454	1.344
2	rs137915944	71148389	A	0.007732	0	G	0.01456	NA
1	rs1416840	36888462	G	0.3144	0.2508	A	0.01461	1.37
1	rs61308377	36893968	G	0.3144	0.2508	A	0.01461	1.37
2	rs2303585	109388986	A	0.02062	0.005833	G	0.01462	3.588
6	rs187767438	112421925	A	0.02062	0.005833	G	0.01462	3.588
15	rs140507225	30011116	A	0.02062	0.005833	G	0.01462	3.588
21	rs7354779	45670770	G	0.01804	0.045	A	0.01463	0.3899
13	rs1799944	32911463	G	0.1675	0.1175	A	0.0147	1.511
11	rs143145279	48181561	G	0.02577	0.008333	A	0.01483	3.148
4	rs61733152	143067053	A	0.0799	0.04667	G	0.01485	1.774
10	rs6901	3180227	A	0.2216	0.285	G	0.01487	0.7144
15	rs6494537	66051345	G	0.2216	0.285	A	0.01487	0.7144
20	rs2227891	25262769	A	0.2732	0.3408	G	0.0149	0.727
17	rs16942	41244000	G	0.3454	0.2792	A	0.01494	1.362
17	rs16941	41244435	G	0.3454	0.2792	A	0.01494	1.362
1	rs1046712	169510475	A	0.05412	0.09349	C	0.01508	0.5548
20	rs2228976	25259006	A	0.2758	0.3431	C	0.01509	0.7291
3	rs2005557	49701298	G	0.03608	0.07083	A	0.01512	0.491
22	rs118116676	30934885	A	0.03608	0.07083	G	0.01512	0.491
17	rs799917	41244936	A	0.3454	0.28	G	0.01513	1.357
11	rs17300741	64331462	G	0.08247	0.0475	A	0.01516	1.802
9	rs78649617	140161526	G	0.03351	0.01333	A	0.01517	2.565
15	rs493258	58687880	G	0.2036	0.2658	A	0.01518	0.7061
11	rs10899035	74401763	G	0.3634	0.4333	A	0.01523	0.7465
19	rs999813	18368810	C	0.366	0.4367	A	0.01523	0.7447
4	rs6827214	171089295	G	0.3119	0.38	A	0.01524	0.7394
6	rs3130931	31134888	A	0.25	0.3158	G	0.01535	0.7221
7	rs2072220	21775387	G	0.2268	0.29	A	0.01549	0.7182
13	rs941823	41013977	A	0.09536	0.1425	G	0.01549	0.6343
12	rs9668527	118511726	A	0.07732	0.1225	G	0.01553	0.6003
6	rs2842643	41650736	G	0.2809	0.3475	A	0.01558	0.7336
2	rs3768716	215635794	G	0.2268	0.17	A	0.01568	1.432
5	rs12187614	84088448	A	0.116	0.075	C	0.01568	1.618
3	rs116202356	38103776	A	0.002577	0.01917	G	0.01569	0.1322
14	rs201886389	52520386	A	0.002577	0.01917	G	0.01569	0.1322
3	rs17080284	48641060	G	0.06186	0.0325	C	0.0157	1.963
8	rs2247572	73633028	A	0.1881	0.2475	G	0.01571	0.7046
1	rs79016973	978974	A	0.03866	0.01667	G	0.01573	2.373
1	rs3813188	981942	A	0.03866	0.01667	C	0.01573	2.373
3	rs61737314	129267910	A	0.03866	0.01667	G	0.01573	2.373
6	rs6910311	116478187	A	0.3866	0.3183	G	0.01575	1.35
18	rs4257308	58109190	G	0.06186	0.03255	A	0.01577	1.959

13	rs79111014	43463378	A	0.1366	0.09167	G	0.0158	1.568
16	rs1114931	69076621	A	0.4948	0.4233	G	0.01583	1.334
4	rs2347131	110791361	A	0.2088	0.2708	G	0.01588	0.7103
3	rs9834639	48659009	A	0.01546	0.04083	C	0.01602	0.3689
8	rs11136343	145058986	G	0.08763	0.1333	A	0.01607	0.6243
11	rs4758423	6532519	A	0.2113	0.1575	G	0.0161	1.433
1	rs1411478	180962282	A	0.2887	0.355	G	0.01622	0.7373
6	rs10484813	162093140	G	0.2113	0.2733	A	0.01623	0.7124
8	rs1533308	22669393	A	0.3067	0.2433	G	0.01627	1.376
3	rs35674179	121414217	A	0.0232	0.0525	C	0.01628	0.4286
14	rs2369679	96922752	G	0.0232	0.0525	C	0.01628	0.4286
15	rs12442156	68628636	G	0.4046	0.475	A	0.01629	0.7512
5	rs865093	41061716	A	0.4356	0.3667	G	0.01632	1.333
12	rs703842	58162739	A	0.2603	0.3258	G	0.01635	0.7281
11	rs58186801	6568213	G	0.1985	0.1458	A	0.01637	1.45
4	rs4699215	102343571	A	0.5232	0.4517	G	0.01638	1.332
5	rs1531545	52193287	G	0.5232	0.4517	A	0.01638	1.332
14	rs941591	94756750	A	0.3325	0.4008	G	0.01638	0.7445
16	rs3811363	55362645	A	0.116	0.07583	G	0.01638	1.599
16	rs12918619	81173136	A	0.116	0.07583	G	0.01638	1.599
3	rs4679115	125997198	G	0.3325	0.2675	A	0.0164	1.364
14	rs45567233	25043671	G	0.2294	0.1733	A	0.01644	1.42
17	rs222747	3493200	C	0.5232	0.4525	G	0.01644	1.328
16	rs2745101	1537839	G	0.5284	0.4575	A	0.01648	1.328
6	rs2239518	30865725	A	0.5387	0.4675	G	0.01655	1.33
6	rs2286655	30899746	A	0.5387	0.4675	G	0.01655	1.33
12	rs3825393	109883374	A	0.4433	0.5142	G	0.01661	0.7524
1	rs3806308	20142866	A	0.5464	0.4758	G	0.01664	1.327
14	rs11625796	63372471	G	0.5335	0.4633	A	0.01664	1.325
3	rs890529	140785175	A	0.3376	0.4058	C	0.01668	0.7463
3	rs11715915	49455330	A	0.0567	0.09667	G	0.01669	0.5617
12	rs285584	41946539	A	0.0567	0.09667	G	0.01669	0.5617
19	rs890869	55633043	A	0.4381	0.5083	G	0.01669	0.7542
16	rs11866096	84555319	G	0.1959	0.2558	A	0.01686	0.7086
8	rs28460821	80937871	A	0.06443	0.105	G	0.01687	0.587
21	rs17176933	47660749	C	0.04897	0.02417	A	0.01687	2.079
7	rs79474211	151478406	A	0.08247	0.04917	G	0.01696	1.738
4	rs1063743	1729988	A	0.1108	0.1608	G	0.01702	0.6505
10	rs10741243	132947962	G	0.09021	0.1367	C	0.01703	0.6263
2	rs146933458	149838032	A	0.03866	0.0725	G	0.01704	0.5145
6	rs206762	32970450	G	0.2397	0.3025	A	0.01705	0.7269
2	rs2943641	227093745	A	0.02835	0.05833	G	0.01707	0.471
2	rs2972146	227100698	C	0.02835	0.05833	A	0.01707	0.471

4	rs17680881	1732978	A	0.1108	0.1608	G	0.01709	0.6503
6	rs2228547	116441646	C	0.3119	0.2492	G	0.01709	1.366
10	rs28939689	13178766	A	0.06186	0.03333	G	0.01709	1.912
14	rs17780143	50901768	A	0.06186	0.03333	G	0.01709	1.912
18	rs8305	51820805	G	0.3376	0.2733	A	0.01711	1.355
6	rs79496316	36343668	A	0	0.01333	C	0.01714	0
14	rs2302858	76525675	A	0	0.01333	G	0.01714	0
14	rs146744332	94780768	A	0	0.01333	G	0.01714	0
15	rs60163948	41192719	A	0	0.01333	C	0.01714	0
17	rs201625874	80274081	A	0	0.01333	G	0.01714	0
19	rs200278064	35448919	A	0	0.01333	G	0.01714	0
19	rs201800628	35448945	C	0	0.01333	G	0.01714	0
6	rs2351528	74497009	G	0.3505	0.4192	A	0.01724	0.7478
1	rs6678278	184410702	A	0.1804	0.2392	G	0.01728	0.7003
2	rs35487251	170354138	A	0.01546	0.003333	G	0.0173	4.696
9	rs138346167	128113096	A	0.01546	0.003333	G	0.0173	4.696
9	rs200252232	139905532	A	0.01546	0.003333	C	0.0173	4.696
17	rs2277666	27228118	A	0.01546	0.003333	G	0.0173	4.696
6	rs742132	25607571	G	0.1804	0.2383	A	0.01731	0.7035
6	rs3130014	33312308	A	0.3041	0.3708	G	0.01732	0.7415
4	rs1049544	88415636	C	0.4562	0.3875	G	0.01737	1.326
4	rs13051	88416188	A	0.4562	0.3875	C	0.01737	1.326
8	rs80304851	145058539	G	0.0567	0.02922	A	0.01751	1.997
11	rs1052373	47354787	G	0.4124	0.3442	A	0.01765	1.337
11	rs11540721	111753191	C	0.1005	0.06333	A	0.01767	1.653
16	rs400037	336396	A	0.05155	0.09	G	0.01779	0.5495
7	rs75056037	1486451	A	0.01289	0.0375	G	0.01783	0.3351
7	rs10264952	23775454	A	0.01289	0.0375	G	0.01783	0.3351
7	rs10264967	23775477	A	0.01289	0.0375	C	0.01783	0.3351
17	rs8482	41361960	G	0.3428	0.2792	A	0.01788	1.347
11	rs7480644	6549995	A	0.2191	0.165	G	0.01793	1.42
1	rs1569476	169608917	A	0.1521	0.2075	G	0.01796	0.6849
10	rs11189513	99969568	G	0.317	0.255	A	0.01797	1.356
1	rs6032	169511555	G	0.2526	0.1958	A	0.01806	1.388
1	rs4525	169511734	G	0.2526	0.1958	A	0.01806	1.388
1	rs4524	169511755	G	0.2526	0.1958	A	0.01806	1.388
6	rs3094112	30761734	G	0.09278	0.1383	A	0.01809	0.637
6	rs191549504	33411673	G	0.005155	0.02333	A	0.01811	0.2169
15	rs199864678	42113822	G	0.005155	0.02333	C	0.01811	0.2169
17	rs76067052	66903998	A	0.04381	0.02083	G	0.01811	2.154
17	rs1799966	41223094	G	0.3454	0.2817	A	0.01814	1.345
17	rs6503444	43783370	G	0.1263	0.1775	A	0.01822	0.6698
10	rs11002803	80820431	A	0.2758	0.2158	G	0.01826	1.383

3	rs3732867	141163655	A	0.01031	0.0325	G	0.0183	0.3101
18	rs3748428	10681711	A	0.2268	0.2892	G	0.01832	0.7211
1	rs2642993	247151035	C	0.03093	0.01167	A	0.01838	2.704
1	rs2642992	247151557	A	0.03093	0.01167	G	0.01838	2.704
1	rs2642973	247162658	G	0.03093	0.01167	A	0.01838	2.704
16	rs3829557	1543167	A	0.482	0.4133	G	0.01838	1.32
17	rs34688574	7220881	A	0.03093	0.01167	G	0.01838	2.704
19	rs3218222	10676681	G	0.2526	0.3158	A	0.01846	0.732
19	rs1122955	58946203	A	0.04639	0.0825	G	0.01851	0.541
1	rs145087137	1238583	A	0	0.01417	G	0.01853	0
1	rs17163588	26450009	A	0	0.01417	G	0.01853	0
3	rs147346094	69082833	A	0	0.01417	G	0.01853	0
5	rs1500367	52464757	A	0.09278	0.0575	G	0.01853	1.676
5	rs186497515	149410319	A	0	0.01417	T	0.01853	0
7	rs6964587	91630620	A	0.1546	0.21	C	0.01854	0.6882
7	rs6960867	91712698	G	0.1546	0.21	A	0.01854	0.6882
7	rs1063243	91726927	C	0.1546	0.21	A	0.01854	0.6882
12	rs34513555	110960196	A	0.1211	0.08	G	0.01856	1.585
6	rs2857218	32855340	C	0.4278	0.36	G	0.0187	1.329
5	rs7707660	108519979	A	0.3943	0.4633	G	0.01876	0.7543
1	rs151081025	51826853	G	0.007732	0.02833	A	0.01877	0.2672
12	rs405394	28240440	G	0.3943	0.4633	A	0.01878	0.7541
9	rs72727021	35661943	C	0.007732	0.0275	A	0.0188	0.2756
9	rs45567235	35665153	A	0.007732	0.0275	C	0.0188	0.2756
11	rs75289680	56113648	C	0.007732	0.0275	A	0.0188	0.2756
5	rs8180502	78477017	G	0.5026	0.4333	A	0.01886	1.321
1	rs6131	169580885	A	0.1418	0.195	G	0.01887	0.6818
9	rs6478246	119368245	A	0.3892	0.3225	G	0.01897	1.338
16	rs1040499	1538363	C	0.5026	0.4342	A	0.01901	1.317
6	rs1619936	71598545	G	0.4072	0.4767	A	0.01904	0.7542
19	rs2277921	18375846	A	0.3892	0.3233	G	0.01908	1.333
11	rs11226057	103779025	G	0.5155	0.4458	A	0.01909	1.322
1	rs2890565	7909737	A	0.3015	0.2408	G	0.01911	1.361
11	rs2306029	46893108	G	0.3015	0.2408	A	0.01911	1.361
10	rs4142041	68640951	G	0.4072	0.4758	A	0.01912	0.7567
7	rs3735400	36438709	G	0.2088	0.1558	C	0.01915	1.429
18	rs1057251	33848581	G	0.0567	0.03	A	0.01915	1.944
8	rs11545077	63951237	A	0.2242	0.17	G	0.01918	1.411
7	rs2286845	93623458	G	0.4098	0.4783	A	0.01919	0.7572
13	rs1801244	52544805	C	0.4098	0.4783	G	0.01919	0.7572
17	rs903160	2091765	A	0.3557	0.2917	G	0.01919	1.341
11	rs668581	75390240	G	0.4124	0.4808	A	0.01925	0.7577
8	rs323344	30702525	C	0.04124	0.07583	A	0.01929	0.5242

8	rs323345	30702602	G	0.04124	0.07583	A	0.01929	0.5242
4	rs1033111	20467767	A	0.4227	0.4917	G	0.01932	0.757
16	rs2745103	1538464	G	0.5284	0.4592	A	0.01932	1.319
5	rs12656353	6913626	C	0.4175	0.4858	A	0.01936	0.7586
1	rs77062647	155028450	A	0.02577	0.055	C	0.0194	0.4545
9	rs10967598	2700883	A	0.4459	0.515	C	0.01946	0.7578
16	rs2063506	73793552	G	0.5258	0.4575	A	0.01946	1.315
19	rs7260518	4511955	A	0.05412	0.0925	G	0.01948	0.5614
20	rs6026468	57290347	G	0.5387	0.47	C	0.01949	1.317
1	rs723821	165016712	C	0.4381	0.5067	A	0.01953	0.7593
3	rs6781428	100793911	A	0.4381	0.5067	G	0.01953	0.7593
8	rs6472404	69429259	A	0.1186	0.1683	G	0.01954	0.6645
3	rs6438013	111513720	A	0.4407	0.5092	C	0.01956	0.7596
6	rs1064583	116446576	G	0.3299	0.2675	A	0.01956	1.348
6	rs885950	31140152	C	0.4433	0.375	A	0.01957	1.327
11	rs17880039	67163628	G	0.07732	0.04583	A	0.0196	1.745
14	rs3818186	52509501	G	0.4459	0.5142	A	0.01961	0.7603
17	rs12602912	65870073	G	0.2964	0.3625	A	0.01961	0.7408
19	rs140127889	19747587	A	0.02577	0.009167	G	0.01962	2.86
19	rs201975024	45668158	A	0.02577	0.009167	G	0.01962	2.86
17	rs312893	75423198	A	0.1804	0.1317	C	0.01968	1.452
1	rs17130529	68614320	A	0.04124	0.01833	G	0.01983	2.303
5	rs199678757	127673769	G	0.04124	0.01833	C	0.01983	2.303
3	rs9858542	49701983	A	0.03608	0.06917	G	0.01986	0.5038
8	rs8190955	30565624	A	0.03608	0.06917	G	0.01986	0.5038
6	rs7772436	33991368	G	0.07216	0.04083	A	0.01991	1.827
2	rs2066518	217288388	G	0.1082	0.1567	C	0.01995	0.6534
8	rs4736794	39840234	G	0.3015	0.3675	A	0.02003	0.7431
17	rs4969341	78988362	A	0.451	0.3833	C	0.02004	1.322
8	rs6468230	33982495	A	0.268	0.3317	G	0.02012	0.7379
14	rs56214359	21204213	G	0.4536	0.3858	A	0.02016	1.321
17	rs1800307	78085871	A	0.1469	0.2	G	0.0202	0.6888
6	rs213226	33209310	A	0.1624	0.2175	G	0.0203	0.6974
5	rs11745439	33230034	G	0.3531	0.42	A	0.02031	0.7537
9	rs1226588	103341014	A	0.2423	0.3042	G	0.02036	0.7314
7	rs12700667	25901639	A	0.2294	0.175	G	0.02044	1.403
5	rs4920799	84606528	A	0.4046	0.3392	G	0.02045	1.324
19	rs11539588	6735929	A	0.06443	0.03583	G	0.02045	1.853
3	rs11130296	51812952	A	0.04897	0.085	G	0.02048	0.5543
6	rs16883019	19969638	G	0.3067	0.3717	A	0.02052	0.7479
20	rs143383	34025983	G	0.3093	0.2492	A	0.02052	1.349
21	rs2822544	15653065	G	0.4639	0.3958	A	0.02063	1.321
2	rs3732149	20818764	A	0.1057	0.0675	G	0.02075	1.632

17	rs9913923	1703982	A	0.1057	0.0675	G	0.02075	1.632
1	rs12755088	1558792	A	0.3634	0.4299	G	0.02086	0.7571
18	rs10871565	75698350	A	0.07216	0.04167	G	0.02097	1.789
1	rs117711462	93159927	A	0.01804	0.005	G	0.021	3.656
1	rs191993066	218480973	G	0.01804	0.005	A	0.021	3.656
4	rs61752607	109086280	A	0.01804	0.005	G	0.021	3.656
9	rs61742087	115652463	A	0.01804	0.005	G	0.021	3.656
10	rs117668723	106207474	G	0.01804	0.005	A	0.021	3.656
22	rs5998832	33661960	A	0.01804	0.005	G	0.021	3.656
11	rs2276118	67288594	G	0.4149	0.3483	A	0.02106	1.327
2	rs10498091	221899444	A	0.134	0.0925	G	0.0211	1.518
13	rs9315204	33693837	A	0.183	0.24	G	0.02112	0.7093
1	rs17401966	10385471	G	0.3428	0.28	A	0.02113	1.341
21	rs17182857	47685285	A	0.01804	0.005008	G	0.02115	3.65
21	rs1556314	45811343	C	0.1469	0.1028	A	0.02116	1.502
6	rs2517448	31062667	A	0.2784	0.3425	G	0.02118	0.7405
18	rs7614	51681244	G	0.3737	0.4408	A	0.02122	0.7569
6	rs6457327	31074030	A	0.2784	0.3417	C	0.02123	0.7432
1	rs6674304	116887742	G	0.1598	0.1133	A	0.02124	1.488
5	rs6896969	40424426	C	0.1366	0.1875	A	0.02125	0.6856
5	rs1373692	40431183	C	0.1237	0.1742	A	0.02127	0.6694
5	rs9292777	40437948	A	0.1237	0.1742	G	0.02127	0.6694
8	rs148906742	67809094	A	0.05155	0.02667	G	0.02129	1.984
15	rs2929282	44245931	T	0.05155	0.02667	A	0.02129	1.984
15	rs73398258	49699463	G	0.06443	0.1042	A	0.02131	0.5923
10	rs3189030	115393929	A	0.3763	0.4433	G	0.02133	0.7575
16	rs3751682	89663025	A	0.1373	0.09417	G	0.02141	1.531
16	rs9921148	76013069	G	0.3886	0.4566	A	0.02144	0.7564
13	rs1512651	56772696	G	0.4201	0.3539	A	0.02147	1.322
19	rs140680651	48049175	C	0.05928	0.03167	G	0.02148	1.927
2	rs71425650	180810358	A	0.1675	0.2225	G	0.0215	0.7032
6	rs2736426	31745284	G	0.4175	0.3525	A	0.02157	1.317
11	rs485645	126432979	A	0.201	0.1503	G	0.02161	1.423
6	rs3117035	33086249	A	0.4845	0.4175	G	0.02162	1.311
6	rs3817969	32361388	A	0.1856	0.2425	G	0.02165	0.7117
6	rs41521946	32362703	A	0.1856	0.2425	C	0.02165	0.7117
6	rs28362677	32362741	A	0.1856	0.2425	G	0.02165	0.7117
6	rs28362678	32362745	A	0.1856	0.2425	G	0.02165	0.7117
3	rs13076253	131751775	C	0.116	0.07667	A	0.02169	1.58
19	rs12461753	38202515	A	0.1366	0.09417	G	0.0217	1.522
17	rs7217976	69481277	A	0.3479	0.285	C	0.02175	1.339
3	rs3738000	130947435	A	0.3866	0.4533	T	0.02176	0.76
20	rs2236164	34097353	G	0.2938	0.235	A	0.02176	1.354

22	rs2385714	17285949	G	0.2526	0.197	A	0.02187	1.377
9	rs35142210	36607660	G	0.08247	0.125	A	0.0219	0.6292
6	rs3130349	32147696	A	0.08247	0.1258	G	0.02193	0.6245
17	rs13030	61908556	G	0.3918	0.4583	A	0.02196	0.7612
1	rs9332608	169510118	A	0.1727	0.1258	G	0.02199	1.45
16	rs4781458	13489119	A	0.3969	0.4642	G	0.022	0.7597
8	rs17119525	14372503	G	0.4278	0.3617	A	0.02204	1.32
6	rs76754818	66063428	A	0.03093	0.0125	G	0.02205	2.521
6	rs10872670	151669875	A	0.2861	0.3492	G	0.02205	0.7469
17	rs2034310	72691928	A	0.2861	0.3492	G	0.02205	0.7469
6	rs12664430	33272677	A	0.0232	0.05083	G	0.02206	0.4434
11	rs11568482	62763264	A	0.0232	0.05083	T	0.02206	0.4434
15	rs79161311	94841430	A	0.2294	0.2892	G	0.02207	0.7317
2	rs17175114	180854620	G	0.1701	0.2245	A	0.02213	0.7079
2	rs2216317	205505348	A	0.2526	0.1975	G	0.02213	1.373
11	rs1051006	47306585	G	0.5052	0.4375	A	0.02213	1.312
2	rs1455986	138496511	A	0.3299	0.3958	G	0.02216	0.7514
1	rs319469	118249041	A	0.4072	0.475	G	0.02222	0.7593
2	rs4663845	239039637	A	0.1263	0.08583	G	0.02223	1.539
3	rs7624349	6764063	A	0.4098	0.4775	G	0.02228	0.7598
16	rs16973500	71965196	A	0.2577	0.32	G	0.02231	0.7378
3	rs144633885	139092116	A	0.0232	0.0075	G	0.02238	3.142
6	rs3130991	31087354	A	0.518	0.45	G	0.02238	1.314
9	rs143131480	115973768	A	0.0232	0.0075	G	0.02238	3.142
19	rs187217680	36159524	A	0.0232	0.0075	G	0.02238	3.142
11	rs2239896	118983434	A	0.1366	0.095	G	0.02245	1.507
3	rs61188513	73114003	G	0.2912	0.355	A	0.02247	0.7466
10	rs16928529	72982985	C	0.2912	0.355	A	0.02247	0.7466
16	rs2667661	1544302	A	0.518	0.4508	G	0.02247	1.309
9	rs12686794	5656610	G	0.05155	0.0875	C	0.02251	0.5668
13	rs9531508	84318052	C	0.5232	0.4558	A	0.02253	1.31
8	rs7825269	102706618	G	0.4278	0.4958	A	0.02256	0.7603
3	rs61745795	41759288	G	0.03608	0.01583	A	0.0226	2.327
17	rs11077986	80280865	G	0.5309	0.4633	A	0.0226	1.311
22	rs144284647	21328899	G	0.03608	0.01583	A	0.0226	2.327
9	rs45534637	96021423	A	0.116	0.07763	G	0.02264	1.559
8	rs12541700	139215691	A	0.5464	0.4783	G	0.02267	1.314
12	rs6581950	71043296	A	0.4304	0.4975	G	0.02268	0.7633
12	rs10846247	16062450	G	0.2371	0.1833	A	0.0227	1.385
5	rs39597	96484131	C	0.2938	0.3575	A	0.02273	0.7477
17	rs3186520	72257713	A	0.09794	0.0625	G	0.02274	1.629
6	rs12204275	99138875	A	0.2784	0.22	C	0.02275	1.368
14	rs2275145	35242828	A	0.5464	0.4792	G	0.02275	1.309

19	rs77768804	54573301	A	0.01546	0.04	G	0.02279	0.377
18	rs1149360	13540713	C	0.1495	0.1058	A	0.0228	1.485
16	rs8017	2821573	A	0.3273	0.2658	G	0.02285	1.344
6	rs2284191	32976654	A	0.1289	0.1792	G	0.02294	0.6777
17	rs2012068	2255351	G	0.4381	0.3731	A	0.02297	1.31
6	rs2516698	30232374	A	0.06701	0.1058	G	0.02298	0.6068
17	rs7503034	78184393	A	0.2964	0.36	G	0.02298	0.7489
19	rs2303690	48525507	G	0.1392	0.09667	A	0.0231	1.511
16	rs154659	89667337	G	0.3454	0.4117	A	0.02316	0.754
19	rs8105737	15198631	C	0.3428	0.4075	A	0.02316	0.7584
10	rs1459996	61007534	A	0.1289	0.0875	G	0.0232	1.543
3	rs12633842	152864665	A	0.2139	0.2725	C	0.02323	0.7265
5	rs10051830	110452845	A	0.3943	0.3308	G	0.02327	1.317
19	rs3745540	51535130	A	0.4485	0.3823	G	0.02334	1.314
2	rs149707354	167134775	G	0.04639	0.02333	A	0.02341	2.036
5	rs2228043	55251931	G	0.04639	0.02333	C	0.02341	2.036
19	rs61744742	1255692	A	0.04639	0.02333	G	0.02341	2.036
19	rs2302971	11617079	A	0.1186	0.07917	G	0.02345	1.564
4	rs16998073	81184341	A	0.3969	0.3333	T	0.02349	1.316
2	rs6761276	113832312	G	0.1778	0.13	A	0.02351	1.448
6	rs365936	7920904	C	0.3505	0.4158	A	0.02352	0.7582
3	rs13080594	7403646	G	0.3531	0.4192	A	0.02361	0.7563
6	rs1415534	65316377	A	0.3531	0.4183	G	0.02368	0.7589
20	rs224331	34022387	C	0.3067	0.2471	A	0.02375	1.348
8	rs60050811	145086876	G	0.0544	0.02833	A	0.02376	1.973
7	rs12699839	2370956	G	0.4562	0.39	A	0.0238	1.312
8	rs2271111	25222165	G	0.366	0.3033	A	0.02401	1.326
1	rs77928789	26644537	A	0.05412	0.02833	C	0.02402	1.962
6	rs206768	32961387	G	0.1469	0.1985	A	0.02415	0.6954
7	rs6977576	118092961	A	0.2191	0.2783	G	0.02415	0.7274
2	rs17041850	111850515	A	0.2732	0.3358	G	0.02419	0.7434
3	rs1392224	55474073	A	0.3093	0.3725	G	0.02422	0.7543
6	rs6922865	36094188	C	0.3093	0.3725	A	0.02422	0.7543
11	rs287756	41731763	C	0.366	0.4317	A	0.02432	0.76
7	rs10954253	129434292	G	0.2461	0.3058	A	0.02437	0.741
15	rs9920077	79680749	A	0.4098	0.345	G	0.02438	1.318
7	rs2881758	134628088	A	0.3686	0.4342	C	0.02445	0.7607
1	rs2234266	46646780	G	0.002577	0.0175	A	0.02446	0.1451
5	rs7712091	59823791	A	0.002577	0.0175	G	0.02446	0.1451
5	rs188723514	147024496	A	0.01289	0.0025	C	0.02446	5.209
5	rs201344625	168244406	A	0.01289	0.0025	G	0.02446	5.209
6	rs149965187	148841012	A	0.01289	0.0025	G	0.02446	5.209
7	rs139433641	50596926	G	0.01289	0.0025	C	0.02446	5.209

7	rs77145638	100694953	A	0.002577	0.0175	G	0.02446	0.1451
8	rs17150066	9310511	G	0.3376	0.2767	A	0.02446	1.333
9	rs146243679	35609642	A	0.01289	0.0025	G	0.02446	5.209
9	rs200039443	99250506	A	0.01289	0.0025	G	0.02446	5.209
10	rs184323035	23257325	A	0.01289	0.0025	C	0.02446	5.209
12	rs79889829	9013737	G	0.01289	0.0025	A	0.02446	5.209
12	rs114800468	93148020	A	0.002577	0.0175	T	0.02446	0.1451
12	rs139643676	96379723	A	0.01289	0.0025	G	0.02446	5.209
14	rs202140681	51561405	A	0.01289	0.0025	G	0.02446	5.209
15	rs149008055	89817534	G	0.01289	0.0025	A	0.02446	5.209
15	rs28575545	91811709	A	0.01289	0.0025	G	0.02446	5.209
15	rs202174970	91835729	A	0.01289	0.0025	G	0.02446	5.209
16	rs61733727	74937998	G	0.01289	0.0025	A	0.02446	5.209
19	rs182477622	37618045	A	0.01289	0.0025	G	0.02446	5.209
4	rs17050782	140423134	G	0.3119	0.375	A	0.02447	0.7553
18	rs7506045	11987272	A	0.1804	0.2358	G	0.02449	0.7133
6	rs2745353	127452935	G	0.4691	0.4033	A	0.02452	1.307
22	rs67153574	31061283	A	0.1804	0.1325	G	0.02454	1.441
12	rs7968606	99816842	A	0.1649	0.12	G	0.02455	1.449
8	rs7014346	128424792	A	0.3711	0.3083	G	0.02458	1.324
19	rs111493043	43519270	G	0.06959	0.1093	A	0.02458	0.6092
15	rs61737195	94841691	A	0.2216	0.2808	G	0.02459	0.7292
20	rs11544244	44538271	A	0.002591	0.0175	G	0.0246	0.1458
6	rs1555115	32354520	G	0.09794	0.1425	C	0.02461	0.6533
6	rs12529049	32357715	A	0.09794	0.1425	G	0.02461	0.6533
11	rs10838687	47312892	A	0.4716	0.4058	C	0.02463	1.307
22	rs5751614	23593051	G	0.4716	0.4058	A	0.02463	1.307
5	rs2548145	40134777	G	0.1211	0.1692	A	0.02468	0.6769
12	rs6489844	111385296	G	0.4691	0.4042	A	0.02473	1.302
3	rs10514688	34962669	G	0.1211	0.17	A	0.02476	0.6729
1	rs7516332	62261168	G	0.4149	0.35	A	0.0248	1.317
3	rs189847375	112991397	C	0.002577	0.01833	A	0.02486	0.1384
15	rs3844109	90514894	G	0.02062	0.04583	A	0.0249	0.4383
2	rs2276611	170441001	A	0.1649	0.2183	G	0.02493	0.7072
8	rs2137124	114930114	G	0.3789	0.4442	A	0.02496	0.7633
1	rs2815413	93672723	A	0.02062	0.04667	C	0.02499	0.4301
15	rs1258763	33050423	A	0.04124	0.07333	G	0.02499	0.5435
21	rs1009778	38268859	A	0.3196	0.3842	G	0.02501	0.7529
7	rs17160911	139138950	G	0.1495	0.2008	C	0.02502	0.6994
2	rs147910038	167266312	A	0.01289	0.03583	G	0.02503	0.3513
12	rs3782489	53086645	A	0.1495	0.2017	G	0.02504	0.6958
19	rs35840170	8188812	A	0.183	0.2375	G	0.02515	0.7191
18	rs12604169	59583446	A	0.4845	0.4192	G	0.02526	1.303

20	rs2297345	9543622	G	0.3222	0.3858	A	0.02526	0.7566
6	rs541862	31916951	G	0.1108	0.07333	A	0.02534	1.575
6	rs438999	31928306	G	0.1108	0.07333	A	0.02534	1.575
5	rs10078623	138728483	A	0.04124	0.02	G	0.02535	2.108
13	rs3742289	52603194	C	0.4923	0.4258	A	0.02539	1.307
7	rs7809453	123301940	A	0.3918	0.4575	G	0.02541	0.7637
13	rs1413115	59214304	A	0.3918	0.4575	G	0.02541	0.7637
1	rs6674775	110950439	A	0.3144	0.2558	G	0.02543	1.334
14	rs10133278	65638277	G	0.4948	0.4283	A	0.02547	1.307
15	rs4502156	62383155	A	0.4923	0.4267	G	0.02549	1.303
5	rs11742570	40410584	G	0.1366	0.1867	A	0.0255	0.6893
5	rs6451493	40410935	A	0.1366	0.1867	C	0.0255	0.6893
5	rs1992660	40415067	A	0.1366	0.1867	G	0.0255	0.6893
19	rs8104843	15198292	G	0.4201	0.3567	C	0.02553	1.307
19	rs8108721	15198507	A	0.4201	0.3567	G	0.02553	1.307
5	rs34915710	77125991	G	0.1959	0.1475	A	0.02558	1.408
5	rs1616919	87047416	C	0.3969	0.4625	A	0.0256	0.7648
3	rs11921691	113673125	A	0.4253	0.3608	G	0.0257	1.311
1	rs642961	209989270	A	0.1237	0.1725	G	0.02571	0.6772
12	rs61738699	113589799	A	0.1237	0.1725	G	0.02571	0.6772
2	rs6731289	82892794	A	0.2861	0.3475	C	0.02577	0.7524
3	rs1057278	9876919	A	0.07474	0.045	G	0.02577	1.714
16	rs1362756	51458290	C	0.07474	0.045	G	0.02577	1.714
21	rs2833270	32487354	A	0.07474	0.045	G	0.02577	1.714
1	rs3748597	888659	A	0.03608	0.06667	G	0.0258	0.5241
1	rs3828049	889238	A	0.03608	0.06667	G	0.0258	0.5241
6	rs17270561	25820439	A	0.1005	0.145	C	0.02583	0.6589
19	rs2074078	58213952	A	0.4974	0.4325	C	0.02583	1.299
1	rs1626370	201288955	A	0.2062	0.2625	G	0.02588	0.7297
6	rs969931	29494897	A	0.5077	0.4417	C	0.02588	1.304
6	rs4713380	30785273	G	0.4278	0.3633	A	0.02588	1.31
6	rs3906263	31031904	A	0.183	0.1358	G	0.02602	1.425
18	rs7506909	50620087	G	0.02577	0.05333	A	0.02602	0.4696
16	rs1005190	855717	C	0.5129	0.4475	G	0.02611	1.3
17	rs1130638	37054772	A	0.1572	0.1125	G	0.02611	1.472
16	rs2302607	8729124	A	0.3325	0.3958	G	0.02612	0.7602
6	rs7766874	25589165	A	0.2577	0.3183	G	0.02615	0.7435
12	rs962976	68720627	G	0.4227	0.4883	A	0.02622	0.7671
11	rs2555158	6520164	A	0.25	0.1967	T	0.02632	1.362
12	rs6598163	132325239	A	0.4201	0.485	G	0.02633	0.7693
3	rs2886182	38088279	G	0.5387	0.4725	A	0.02637	1.304
6	rs9505118	7290437	G	0.4381	0.5042	A	0.02637	0.7669
14	rs2101919	52520368	A	0.549	0.4825	G	0.0264	1.305

5	rs1063310	170239112	A	0.232	0.29	C	0.02641	0.7394
3	rs4488809	189356261	A	0.4433	0.5092	G	0.02642	0.7676
9	rs2297879	35662251	G	0.5232	0.4583	A	0.02644	1.297
3	rs7625806	38080952	A	0.5438	0.4783	C	0.02648	1.3
4	rs207681	16770405	C	0.5387	0.4733	A	0.02649	1.299
22	rs6007594	45728370	A	0.5361	0.4708	G	0.02649	1.299
10	rs1219648	123346190	G	0.2938	0.3567	A	0.02652	0.7505
15	rs8024732	42570718	A	0.1134	0.1592	G	0.02656	0.6757
1	rs4128458	201355522	G	0.4433	0.5083	A	0.02657	0.7702
2	rs12711788	114533618	G	0.4356	0.3717	A	0.02657	1.305
4	rs1058992	156274377	A	0.4356	0.3717	G	0.02657	1.305
5	rs395967	38842959	G	0.4433	0.5083	A	0.02657	0.7702
16	rs3909546	68536105	C	0.04897	0.025	A	0.0266	2.008
21	rs2298697	47660867	A	0.04897	0.025	G	0.0266	2.008
6	rs620202	32941910	A	0.1263	0.1742	C	0.02665	0.6854
11	rs7397032	5758062	A	0.3531	0.2925	G	0.02675	1.32
16	rs17291845	55245037	A	0.03608	0.01667	G	0.02684	2.209
17	rs76205959	10219244	A	0.03608	0.01667	G	0.02684	2.209
17	rs1047833	43342141	G	0.1907	0.2458	C	0.02687	0.723
18	rs4797625	12130394	A	0.1907	0.2458	G	0.02687	0.723
22	rs2275984	24622648	G	0.1907	0.2458	A	0.02687	0.723
6	rs2523651	31448154	A	0.1736	0.2275	G	0.02696	0.7132
10	rs4935502	55955444	A	0.134	0.09333	C	0.027	1.503
10	rs2296434	100183570	C	0.1727	0.2267	G	0.027	0.7121
7	rs11773103	86821980	G	0.01031	0.03167	A	0.02705	0.3185
8	rs4872318	25364331	A	0.08247	0.1233	G	0.02708	0.6389
10	rs11266022	42503340	G	0.4407	0.3775	A	0.02714	1.299
20	rs1341024	36932676	G	0.4459	0.3817	C	0.02722	1.304
6	rs6905408	29633775	A	0.1418	0.1917	G	0.02724	0.6966
1	rs13376333	154814353	A	0.005155	0.0225	G	0.02725	0.2251
1	rs145021449	204380127	A	0.005155	0.0225	C	0.02725	0.2251
2	rs192098032	152492791	A	0.005155	0.0225	G	0.02725	0.2251
7	rs77133388	72718278	A	0.005155	0.0225	G	0.02725	0.2251
8	rs115936153	133909927	A	0.005155	0.0225	G	0.02725	0.2251
9	rs3814541	109689752	A	0.1031	0.0675	G	0.02725	1.588
10	rs7913069	105714399	A	0.1031	0.0675	G	0.02725	1.588
14	rs76888297	47426686	C	0.005155	0.0225	G	0.02725	0.2251
16	rs35947557	1842130	A	0.005155	0.0225	G	0.02725	0.2251
1	rs2025236	204783205	A	0.1572	0.2092	G	0.02732	0.7053
14	rs201322620	20344715	G	0.005155	0.02167	A	0.02732	0.234
14	rs943992	21553057	G	0.005155	0.02167	A	0.02732	0.234
15	rs201696797	42133086	C	0.005155	0.02167	A	0.02732	0.234
2	rs2290911	224919	G	0.1572	0.2083	A	0.02737	0.7089

5	rs4958531	151771838	G	0.007732	0.02667	A	0.02737	0.2844
5	rs4958532	151771852	A	0.007732	0.02667	G	0.02737	0.2844
5	rs1895245	151772055	A	0.007732	0.02667	C	0.02737	0.2844
5	rs4958535	151775064	G	0.007732	0.02667	C	0.02737	0.2844
10	rs3088308	81697868	T	0.007732	0.02667	A	0.02737	0.2844
14	rs146022217	96797867	G	0.007732	0.02667	C	0.02737	0.2844
11	rs192501536	3687678	A	0.02835	0.01083	G	0.0274	2.664
17	rs1558876	66364691	G	0.1598	0.115	C	0.02745	1.464
12	rs1859330	113376388	G	0.1933	0.2483	A	0.02747	0.7253
16	rs4788821	71660310	G	0.268	0.3289	A	0.02753	0.7473
19	rs4420638	45422946	G	0.1469	0.1043	A	0.02753	1.478
3	rs2167115	24339734	G	0.4485	0.385	A	0.02758	1.299
2	rs2230515	112755001	G	0.2191	0.1683	A	0.02763	1.386
5	rs27141	64881936	G	0.3557	0.42	A	0.02763	0.7623
13	rs3916958	105782961	A	0.3557	0.42	G	0.02763	0.7623
9	rs10974944	5070831	G	0.1753	0.2283	C	0.02768	0.7182
3	rs1450010	36214197	G	0.5486	0.4817	A	0.02771	1.308
6	rs9494145	135432552	G	0.3582	0.2983	A	0.02771	1.313
1	rs5063	11907648	A	0.1237	0.085	G	0.02781	1.52
10	rs11239786	43312807	A	0.4021	0.3389	G	0.02784	1.312
5	rs42468	64908111	C	0.3067	0.3692	A	0.02785	0.7559
16	rs1035542	72023633	A	0.4562	0.3925	C	0.02798	1.298
19	rs35106910	45284266	C	0.01804	0.04167	A	0.02798	0.4226
20	rs6060369	33907161	G	0.3015	0.245	A	0.028	1.33
1	rs7537296	100203648	A	0.3634	0.3025	G	0.02805	1.316
1	rs200338884	161187847	A	0	0.01167	G	0.02807	0
6	rs146340667	39040708	A	0	0.01167	G	0.02807	0
12	rs34638573	10772876	A	0	0.01167	G	0.02807	0
17	rs3744574	11520833	A	0	0.01167	G	0.02807	0
17	rs149931425	44953783	A	0	0.01167	G	0.02807	0
22	rs145415691	50191485	A	0	0.01167	G	0.02807	0
12	rs10774671	113357193	G	0.1959	0.2517	A	0.02808	0.7243
12	rs1131476	113357209	G	0.1959	0.2517	A	0.02808	0.7243
12	rs1051042	113357237	G	0.1959	0.2517	C	0.02808	0.7243
12	rs2660	113357442	G	0.1959	0.2517	A	0.02808	0.7243
6	rs2517653	30121185	A	0.2784	0.2233	G	0.02809	1.341
7	rs1050331	44808091	A	0.1598	0.2117	C	0.02809	0.7083
8	rs2319936	97871757	A	0.3093	0.3717	G	0.02811	0.757
12	rs795955	78636050	A	0.3093	0.3717	G	0.02811	0.757
2	rs7604639	112751928	A	0.2191	0.1692	G	0.02814	1.378
7	rs72615132	94176476	A	0.01804	0.0425	G	0.02816	0.4139
19	rs2301734	36575645	A	0.1598	0.2108	G	0.02817	0.7119
11	rs10770125	2169014	G	0.3634	0.4267	A	0.02823	0.7671

9	rs10758669	4981602	C	0.2732	0.3342	A	0.02826	0.749
4	rs12507599	36310049	A	0.2202	0.278	G	0.02831	0.7335
12	rs16939904	109520769	G	0.1057	0.1508	A	0.02831	0.6652
2	rs6733011	99465502	G	0.4046	0.3425	A	0.02833	1.305
3	rs16858780	184103903	C	0.4046	0.3425	A	0.02833	1.305
6	rs2233952	31105891	A	0.1134	0.07667	G	0.02833	1.54
8	rs1401430	116274942	G	0.4046	0.3425	A	0.02833	1.305
6	rs394657	32187023	G	0.2191	0.2758	A	0.02839	0.7365
19	rs1345658	35435006	G	0.4665	0.4025	A	0.02849	1.298
1	rs4949198	31681614	C	0.4767	0.4115	A	0.02854	1.303
1	rs139118328	109477470	A	0.03866	0.0175	G	0.02854	2.258
5	rs1801106	52358757	A	0.03866	0.0175	G	0.02854	2.258
19	rs8104533	18377496	G	0.3782	0.4421	C	0.02854	0.7676
19	rs10407022	2249477	C	0.3144	0.3773	A	0.02858	0.757
19	rs705493	38438505	A	0.3067	0.2492	G	0.02858	1.333
3	rs7614116	130368069	A	0.4691	0.405	G	0.02861	1.298
2	rs848642	36782886	G	0.2809	0.2258	A	0.02863	1.339
6	rs2894342	33774394	A	0.1314	0.1792	C	0.02864	0.6933
15	rs2387904	93246962	G	0.3737	0.4383	A	0.02864	0.7646
17	rs10852932	2143460	A	0.3067	0.2496	C	0.02871	1.33
6	rs7745603	27090404	A	0.2603	0.2067	G	0.02884	1.351
16	rs11861974	31765220	A	0.2603	0.2067	G	0.02884	1.351
2	rs150144782	242814702	A	0.0232	0.008333	G	0.02889	2.826
8	rs145821459	1824881	A	0.0232	0.008333	G	0.02889	2.826
6	rs707934	31735631	A	0.05155	0.08667	G	0.02891	0.5727
20	rs6088813	33975181	C	0.3067	0.25	A	0.02893	1.327
9	rs2378383	82039362	G	0.2216	0.1717	A	0.02895	1.374
6	rs511294	31888869	C	0.03351	0.01417	A	0.02896	2.412
22	rs202018920	50655781	A	0.03351	0.01417	G	0.02896	2.412
2	rs2943634	227068080	A	0.05928	0.095	C	0.02897	0.6003
19	rs8110972	18376407	A	0.3789	0.4425	T	0.02898	0.7685
19	rs2277922	18376522	A	0.3789	0.4425	C	0.02898	0.7685
19	rs3746186	18377274	A	0.3789	0.4425	G	0.02898	0.7685
19	rs8103906	18377647	G	0.3789	0.4425	C	0.02898	0.7685
8	rs930557	6302183	C	0.1469	0.1967	G	0.02899	0.7034
9	rs17204533	113132221	A	0.3711	0.31	G	0.02901	1.314
2	rs3731900	220046975	A	0.3376	0.2783	C	0.02905	1.322
10	rs6602878	47646751	G	0.3093	0.2517	A	0.02906	1.331
6	rs6930777	32351566	A	0.03866	0.07	G	0.02907	0.5343
17	rs76896732	72365620	A	0.04381	0.02167	G	0.02909	2.069
11	rs7949651	129832270	A	0.3196	0.3817	G	0.02912	0.761
17	rs1878061	72610089	A	0.09536	0.06167	G	0.02913	1.604
3	rs61758360	105438902	G	0.04897	0.02583	A	0.02924	1.942

6	rs117358940	160482959	A	0.04897	0.02583	G	0.02924	1.942
14	rs2024884	59513299	A	0.384	0.4475	G	0.02925	0.7697
3	rs145154359	124048784	A	0	0.0125	T	0.02926	0
6	rs112779382	27925629	T	0	0.0125	A	0.02926	0
8	rs139723190	75157110	A	0	0.0125	G	0.02926	0
15	rs1455773	93015427	A	0.3866	0.4508	G	0.02926	0.7677
4	rs1812175	145574844	A	0.4845	0.42	G	0.02927	1.298
10	rs17114388	103899871	G	0.2423	0.19	A	0.02927	1.363
11	rs7128017	18319180	A	0.2242	0.1733	C	0.02929	1.378
7	rs1985729	141694805	A	0.3737	0.3125	G	0.02933	1.313
11	rs55920775	60703777	G	0.3222	0.3842	A	0.02937	0.7619
2	rs28651764	42515437	A	0.3866	0.45	G	0.02938	0.7703
4	rs7689420	145568352	A	0.4845	0.4208	G	0.02942	1.294
4	rs7680334	119943328	G	0.1082	0.1533	A	0.02954	0.6703
6	rs594442	93847884	A	0.1082	0.1525	T	0.02957	0.6746
16	rs1135045	23489711	G	0.2835	0.3442	C	0.02959	0.754
1	rs12075	159175354	A	0.1057	0.07	G	0.0296	1.57
16	rs9936815	7161550	A	0.3247	0.3867	G	0.02961	0.7628
17	rs2272606	3657159	A	0.1649	0.2162	G	0.02965	0.7161
10	rs2452505	61005244	G	0.1263	0.0875	C	0.02966	1.507
2	rs699664	85780536	A	0.2835	0.3433	G	0.02968	0.7568
6	rs3131018	31143582	A	0.183	0.2367	C	0.02968	0.7224
1	rs12026893	45788479	A	0.4175	0.3558	G	0.02969	1.298
6	rs7769051	133146796	A	0.06959	0.04167	C	0.0298	1.72
7	rs74327836	122338930	T	0.0232	0.04833	A	0.02981	0.4676
12	rs12227879	102576340	A	0.03351	0.0625	G	0.02985	0.52
5	rs2055388	7527521	C	0.07732	0.1175	A	0.02987	0.6294
5	rs2455230	75932965	C	0.4046	0.4692	G	0.02991	0.769
6	rs3757318	151914113	A	0.2268	0.2833	G	0.02992	0.742
1	rs10888510	152681835	A	0.09794	0.1408	C	0.03005	0.6623
7	rs10271996	29606288	A	0.5103	0.4458	G	0.03015	1.295
14	rs7147576	29046822	G	0.4098	0.4733	A	0.03018	0.7726
6	rs1634722	30979084	G	0.3325	0.395	A	0.03024	0.7629
1	rs10927875	16299312	A	0.01546	0.004167	G	0.03034	3.754
1	exm1792686	32222087	A	0.01546	0.004167	G	0.03034	3.754
1	rs148754480	32222087	A	0.01546	0.004167	G	0.03034	3.754
4	rs76626268	15560840	G	0.01546	0.004167	A	0.03034	3.754
6	rs140125037	32178638	A	0.01546	0.004167	G	0.03034	3.754
7	rs117729990	21856163	A	0.01546	0.004167	G	0.03034	3.754
7	rs78480723	103207075	A	0.01546	0.004167	G	0.03034	3.754
9	rs35568725	19119674	G	0.01546	0.004167	A	0.03034	3.754
15	rs186541436	70960282	A	0.01546	0.004167	G	0.03034	3.754
15	rs61732053	78337303	A	0.01546	0.004167	G	0.03034	3.754

17	rs200662973	10436629	G	0.01546	0.004167	A	0.03034	3.754
19	rs201201843	39907506	A	0.01546	0.004167	G	0.03034	3.754
20	rs142962992	20486079	C	0.01546	0.004167	G	0.03034	3.754
22	rs146133764	19864729	A	0.01546	0.004167	G	0.03034	3.754
8	rs1879184	27311703	G	0.4201	0.4842	A	0.03035	0.7718
1	rs4387163	164995713	A	0.4253	0.49	G	0.03041	0.7701
1	rs4846066	11938050	A	0.5389	0.475	G	0.03042	1.292
2	rs1377956	52883758	A	0.3814	0.3208	C	0.03043	1.305
3	rs710446	186459927	G	0.2294	0.2858	A	0.03043	0.7437
4	rs13129697	9926967	C	0.4175	0.4808	A	0.03044	0.774
6	rs41441749	18791723	A	0.1521	0.1092	G	0.03045	1.463
22	rs2231495	17669306	G	0.1856	0.2383	A	0.03045	0.7282
1	rs16847812	175049379	A	0.317	0.2592	G	0.03048	1.327
6	rs6901761	30790798	A	0.4278	0.3658	G	0.03048	1.296
1	rs1249839	75818341	G	0.2448	0.1933	A	0.03049	1.353
13	rs9533770	44677509	C	0.1366	0.185	A	0.03057	0.697
2	rs4973413	232344556	A	0.4304	0.4942	G	0.03058	0.7735
19	rs2396359	1819125	G	0.4227	0.4858	A	0.03058	0.7749
16	rs9928037	76571199	A	0.4381	0.5025	G	0.03059	0.7721
12	rs2900333	14653867	A	0.3479	0.2883	G	0.03062	1.317
9	rs615474	35043291	C	0.549	0.4842	A	0.03063	1.297
7	rs73195662	105658460	G	0.02835	0.05667	C	0.03064	0.4857
12	rs2306393	68708761	G	0.4253	0.4883	A	0.03067	0.7753
6	rs6901490	124310564	A	0.5309	0.4675	G	0.03072	1.289
4	rs13106227	77418681	G	0.5412	0.4775	A	0.03073	1.291
4	rs4859683	77419073	G	0.5412	0.4775	A	0.03073	1.291
7	rs1055144	25871109	G	0.5361	0.4725	A	0.03073	1.29
17	rs2073866	49738719	C	0.4459	0.5092	A	0.03084	0.7757
19	rs34051133	57325083	A	0.2938	0.355	G	0.03087	0.7559
17	rs12600564	72248512	A	0.09794	0.06333	G	0.03092	1.606
7	rs3735135	40027484	G	0.232	0.2883	A	0.03094	0.7454
1	rs3748022	206669465	A	0.2294	0.1783	G	0.03097	1.371
7	rs35960711	29980471	A	0.1392	0.09917	G	0.03112	1.469
6	rs9398200	109752451	G	0.2474	0.1958	A	0.0312	1.35
6	rs150462171	4126379	A	0.01546	0.0375	G	0.03121	0.4031
6	rs149101394	31918154	G	0.01546	0.0375	A	0.03121	0.4031
9	rs4879929	35815356	G	0.3196	0.2625	A	0.03121	1.32
20	rs6120849	33730387	A	0.01546	0.0375	G	0.03121	0.4031
12	rs118014438	57499258	A	0.03866	0.01833	G	0.03122	2.153
5	rs35361432	54941617	G	0.0567	0.03167	A	0.03131	1.838
6	rs2857597	31585000	A	0.0567	0.03167	T	0.03131	1.838
6	rs1046080	31595882	C	0.0567	0.03167	A	0.03131	1.838
6	rs2272593	31601344	A	0.0567	0.03167	G	0.03131	1.838

6	rs3130617	31627523	G	0.0567	0.03167	A	0.03131	1.838
16	rs11864477	77427769	G	0.0567	0.03167	A	0.03131	1.838
4	rs7666196	27167833	G	0.25	0.1975	A	0.03147	1.354
6	rs379464	32186348	A	0.1289	0.09	G	0.03153	1.496
1	rs7517847	67681669	C	0.4433	0.3817	A	0.03182	1.29
5	rs13156152	169865015	A	0.4433	0.3817	G	0.03182	1.29
15	rs57659670	45398438	G	0.09794	0.06417	A	0.03182	1.583
12	rs2292354	110368201	A	0.2706	0.2175	G	0.03184	1.335
1	rs37458	44290530	G	0.09021	0.1308	A	0.03185	0.6587
21	rs2070425	47836653	A	0.3557	0.4181	G	0.03198	0.7684
10	rs3814596	3207632	G	0.06443	0.0375	A	0.03203	1.768
12	rs61760237	132627365	A	0.06443	0.0375	G	0.03203	1.768
16	rs420259	23634026	G	0.3557	0.4174	A	0.03212	0.7706
17	rs2230553	33269648	G	0.2139	0.2692	C	0.03213	0.7389
12	rs2231909	14577892	T	0.3969	0.3358	A	0.03214	1.302
21	rs2254562	34059352	G	0.3041	0.3642	A	0.03214	0.7631
1	rs150759474	246021875	A	0.02835	0.01167	G	0.03217	2.472
2	rs138888235	97749512	G	0.02835	0.01167	A	0.03217	2.472
4	rs4148304	70460328	A	0.02835	0.01167	G	0.03217	2.472
4	rs1800885	187476360	A	0.02835	0.01167	G	0.03217	2.472
6	rs117513176	109885396	G	0.02835	0.01167	A	0.03217	2.472
11	rs116829295	114393636	A	0.02835	0.01167	G	0.03217	2.472
6	rs2072767	167719436	A	0.299	0.2425	G	0.03219	1.332
8	rs2303995	133175736	G	0.2526	0.2	A	0.0322	1.352
6	rs271118	133236406	G	0.2139	0.2683	A	0.03226	0.742
2	rs2084688	2620146	A	0.2397	0.2967	G	0.03232	0.7474
7	rs7808823	129989878	A	0.3067	0.3675	G	0.03236	0.7614
12	rs76224014	49982238	A	0.04381	0.0225	G	0.03242	1.991
6	rs9261492	30110058	A	0.2732	0.22	G	0.03246	1.333
6	rs9261508	30112704	G	0.2732	0.22	A	0.03246	1.333
16	rs3803641	84270743	A	0.1572	0.2075	G	0.03249	0.7125
19	rs2733743	36050969	G	0.3608	0.4225	A	0.0325	0.7716
10	rs41289305	16979714	G	0.03351	0.015	A	0.03251	2.276
13	rs139495686	37269282	C	0.03351	0.015	A	0.03251	2.276
20	rs149794868	45131121	A	0.03351	0.015	T	0.03251	2.276
17	rs4613097	79170578	A	0.1289	0.09083	G	0.03255	1.481
3	rs9863706	72437413	A	0.2758	0.2217	G	0.03264	1.337
6	rs3749977	29080344	A	0.2345	0.1833	G	0.03265	1.365
1	rs3790479	68948280	C	0.07216	0.1093	A	0.03274	0.6335
12	rs139196998	6167131	A	0.02835	0.01174	G	0.03279	2.455
13	rs9315632	39516627	A	0.299	0.2442	C	0.03285	1.32
16	rs12708923	72020134	G	0.4639	0.4008	A	0.03288	1.294
17	rs2281727	2117945	G	0.3299	0.2721	A	0.03288	1.317

7	rs10254939	10161358	G	0.1031	0.1467	A	0.03292	0.6688
9	rs11789780	90503007	G	0.3634	0.3038	A	0.03294	1.308
17	rs58253413	72588778	A	0.2165	0.1675	G	0.03298	1.373
13	rs348044	80785028	A	0.4639	0.4017	G	0.033	1.289
4	rs11097407	95146135	C	0.1959	0.2492	G	0.03305	0.734
4	rs11722476	95170839	G	0.1959	0.2492	A	0.03305	0.734
6	rs2844463	31615167	A	0.1959	0.25	G	0.03306	0.7308
11	rs10892279	118611781	A	0.1959	0.25	G	0.03306	0.7308
22	rs1894499	32994777	G	0.4093	0.3481	A	0.03311	1.298
13	rs636437	34931307	G	0.09278	0.1342	A	0.03334	0.66
16	rs2397965	55907811	A	0.366	0.3067	G	0.0334	1.305
14	rs935334	76613678	A	0.201	0.1533	G	0.03342	1.389
17	rs1431318	54192523	A	0.201	0.1533	G	0.03342	1.389
4	rs12640613	62256581	A	0.1598	0.2092	G	0.03345	0.7191
19	rs1469023	18378219	A	0.3789	0.4417	G	0.03346	0.7711
6	rs206018	32177880	C	0.2371	0.1858	G	0.0335	1.362
2	rs848534	36690242	G	0.3814	0.4433	A	0.03369	0.7743
2	rs3731607	120125058	A	0.1985	0.2525	G	0.03372	0.733
11	rs79820513	558165	C	0.03608	0.06583	A	0.03377	0.5312
19	rs2302524	44156472	G	0.03608	0.06583	A	0.03377	0.5312
1	rs2794751	236758887	A	0.3325	0.2758	G	0.03381	1.308
1	rs12046117	117751365	A	0.2216	0.2767	G	0.03386	0.7445
12	rs75256744	54448106	G	0.02062	0.045	A	0.03387	0.4468
19	rs2302306	3736239	A	0.02062	0.045	G	0.03387	0.4468
11	rs909116	1941946	A	0.4171	0.3558	G	0.03393	1.295
10	rs11014166	18708798	A	0.09021	0.05833	T	0.03399	1.601
10	rs2370764	32685008	A	0.3892	0.4517	G	0.03399	0.7735
4	rs7677803	156409087	A	0.3222	0.3833	G	0.03402	0.7646
20	rs6060373	33914208	G	0.3067	0.2508	A	0.03403	1.321
2	rs11889862	150697148	A	0.2809	0.3392	G	0.03411	0.7612
1	rs138123146	7724045	G	0.02062	0.006667	A	0.03417	3.137
6	rs448733	33003687	A	0.02062	0.006667	C	0.03417	3.137
7	rs28716840	22202046	A	0.02062	0.006667	C	0.03417	3.137
9	rs76189181	135535118	A	0.02062	0.006667	C	0.03417	3.137
12	rs77767409	54757592	A	0.02062	0.006667	C	0.03417	3.137
22	rs2277838	21377650	A	0.02062	0.006667	G	0.03417	3.137
17	rs11077773	73060073	A	0.4124	0.3525	G	0.03419	1.289
6	rs2072633	31919578	A	0.4897	0.4267	G	0.03421	1.289
1	rs11239930	146560564	G	0.3943	0.4567	A	0.03423	0.7746
8	rs1735169	146067054	G	0.4871	0.425	A	0.03424	1.285
11	rs11605576	64981522	A	0.3351	0.2783	C	0.03424	1.306
11	rs11605632	64981587	A	0.3351	0.2783	G	0.03424	1.306
1	rs2666472	62728784	A	0.1057	0.1483	G	0.03433	0.6784

6	rs204999	32109979	G	0.1057	0.1483	A	0.03433	0.6784
1	rs3917688	169591080	A	0.2835	0.3425	G	0.03439	0.7596
6	rs3132486	31243170	G	0.201	0.2542	A	0.0344	0.7383
1	rs201349073	11889290	A	0.01031	0.001667	G	0.03441	6.24
1	rs9442235	16393357	C	0.01031	0.001667	A	0.03441	6.24
1	rs199897307	35863134	C	0.01031	0.001667	G	0.03441	6.24
1	rs116687780	113120982	A	0.01031	0.001667	C	0.03441	6.24
1	rs200340171	214537946	A	0.01031	0.001667	G	0.03441	6.24
2	rs200562865	228162451	A	0.01031	0.001667	G	0.03441	6.24
3	rs138327769	58140563	G	0.01031	0.001667	C	0.03441	6.24
3	rs114465932	58517084	G	0.01031	0.001667	A	0.03441	6.24
3	rs199780529	151107788	G	0.01031	0.001667	A	0.03441	6.24
4	rs138909144	36152588	A	0.01031	0.001667	G	0.03441	6.24
4	rs201391094	76695831	A	0.01031	0.001667	G	0.03441	6.24
5	rs201752702	98238643	G	0.01031	0.001667	A	0.03441	6.24
7	rs72657364	21781653	G	0.01031	0.001667	A	0.03441	6.24
9	rs139500904	139751640	A	0.01031	0.001667	G	0.03441	6.24
10	rs201955072	30316445	A	0.01031	0.001667	G	0.03441	6.24
10	rs200646223	85961638	A	0.01031	0.001667	G	0.03441	6.24
10	rs148356887	85962796	A	0.01031	0.001667	G	0.03441	6.24
11	rs145680768	18291320	A	0.01031	0.001667	G	0.03441	6.24
11	rs200996605	77734254	A	0.01031	0.001667	G	0.03441	6.24
11	rs143158496	134037976	A	0.01031	0.001667	G	0.03441	6.24
13	rs4770684	25027744	A	0.01031	0.001667	G	0.03441	6.24
16	rs72795864	71483497	G	0.01031	0.001667	C	0.03441	6.24
16	rs200125452	88705398	A	0.01031	0.001667	G	0.03441	6.24
17	rs199775758	8046750	A	0.01031	0.001667	G	0.03441	6.24
17	rs200152929	39672190	A	0.01031	0.001667	C	0.03441	6.24
17	rs145272475	53852185	A	0.01031	0.001667	G	0.03441	6.24
19	rs149763682	14015683	A	0.01031	0.001667	G	0.03441	6.24
19	rs147206412	17394737	A	0.01031	0.001667	C	0.03441	6.24
20	rs188297454	40743919	A	0.01031	0.001667	C	0.03441	6.24
7	rs1133850	6452461	A	0.1108	0.075	G	0.03446	1.537
6	rs3130982	31084075	G	0.2835	0.3417	C	0.0345	0.7624
11	rs4633449	6549779	A	0.2036	0.1558	C	0.0345	1.385
11	rs2344828	6550004	A	0.2036	0.1558	G	0.0345	1.385
14	rs4982419	21623836	A	0.4948	0.4325	G	0.0345	1.285
6	rs4151664	31920873	A	0.03093	0.05833	G	0.03452	0.5152
2	rs200612063	204305568	G	0.01036	0.001678	A	0.03455	6.23
9	rs1417290	7046917	G	0.1469	0.195	A	0.03455	0.7109
6	rs3828855	41900406	A	0.1469	0.1958	G	0.03459	0.7071
16	rs11074889	10633165	A	0.1469	0.1958	G	0.03459	0.7071
5	rs1051489	32400266	G	0.2397	0.1892	A	0.03469	1.351

6	rs241399	32871558	A	0.4046	0.4667	G	0.03469	0.7767
2	rs2293075	220100201	G	0.02577	0.05167	C	0.03471	0.4856
7	rs78598246	141443499	G	0.02577	0.05167	A	0.03471	0.4856
15	rs7172689	81533695	A	0.02577	0.05167	G	0.03471	0.4856
1	rs12723684	77685042	G	0.05928	0.03333	A	0.03483	1.827
3	rs138000709	160143943	G	0.05928	0.03333	A	0.03483	1.827
3	rs1131857	98307696	C	0.2216	0.1725	A	0.03488	1.366
4	rs35402556	163297150	G	0.4098	0.4717	A	0.03489	0.7777
19	rs3745186	17412218	A	0.09536	0.1367	G	0.03494	0.6659
12	rs75238381	105440719	A	0.01289	0.03417	G	0.03497	0.369
6	rs2745400	29488249	A	0.5052	0.4433	G	0.03499	1.282
11	rs2344829	6550309	G	0.2036	0.1567	A	0.03499	1.376
7	rs4646450	99266318	A	0.2036	0.2567	G	0.03507	0.7404
11	rs1566734	48145375	C	0.1649	0.215	A	0.03511	0.7212
11	rs1503185	48146622	A	0.1649	0.215	G	0.03511	0.7212
1	rs500049	228559994	G	0.3325	0.3921	A	0.03516	0.7721
19	rs1054940	55598724	G	0.4278	0.4908	A	0.03521	0.7757
3	rs9819059	11891529	A	0.5206	0.4583	G	0.03525	1.283
3	rs11710345	174826589	A	0.4227	0.4842	G	0.03536	0.78
4	rs1036788	77201487	A	0.4381	0.5008	G	0.03538	0.7772
6	rs9366785	31604011	A	0.07552	0.04591	G	0.0354	1.698
5	rs770189	88088439	G	0.518	0.4566	C	0.03541	1.279
11	rs149402828	8728736	A	0.03866	0.01917	G	0.03544	2.058
19	rs149543099	52380625	A	0.03866	0.01917	G	0.03544	2.058
15	rs12594483	43021986	A	0.2686	0.3267	G	0.03549	0.757
21	rs9980589	34003930	A	0.5206	0.4592	G	0.03549	1.279
4	rs76472753	71275232	A	0.1108	0.07583	G	0.03552	1.519
22	rs760749	50555686	C	0.2423	0.1917	A	0.03552	1.348
6	rs241433	32798795	C	0.4304	0.3692	A	0.03553	1.291
16	rs2112811	25263278	A	0.2577	0.315	G	0.03558	0.7551
2	rs2289235	231738168	G	0.2062	0.1583	A	0.03559	1.381
6	rs2535324	30718035	A	0.4433	0.505	C	0.03561	0.7805
16	rs8043831	17549067	A	0.4433	0.505	G	0.03561	0.7805
7	rs3213633	87476339	C	0.04381	0.07417	A	0.03568	0.572
7	rs118019617	87564388	A	0.04381	0.07417	G	0.03568	0.572
11	rs9888247	40015352	G	0.4304	0.37	A	0.03572	1.287
9	rs61757224	130965795	A	0.06701	0.1033	C	0.03574	0.6232
17	rs2306595	34871721	G	0.1082	0.1517	A	0.03574	0.679
6	rs2021107	30228138	A	0.06701	0.1025	G	0.03586	0.6289
6	rs2844754	30246212	A	0.06701	0.1025	C	0.03586	0.6289
6	rs1048664	30262512	G	0.06701	0.1025	A	0.03586	0.6289
7	rs11764584	99753870	G	0.3402	0.4	A	0.0359	0.7734
7	rs3823646	99757612	G	0.3402	0.4	A	0.0359	0.7734

3	rs267561	37574951	G	0.1675	0.217	A	0.03602	0.726
20	rs3761218	3776175	G	0.2603	0.3175	A	0.03603	0.7565
8	rs1050838	117864217	G	0.2062	0.1592	A	0.03605	1.372
16	rs308925	77769834	A	0.232	0.2875	G	0.03605	0.7485
2	rs798443	7968275	G	0.3605	0.4217	A	0.03606	0.7733
22	rs6000083	36443705	A	0.09278	0.06	G	0.03606	1.602
12	rs1678542	57968715	G	0.2423	0.1925	C	0.03607	1.341
18	exm1376519	13038431	G	0.2423	0.1925	A	0.03607	1.341
18	rs16940057	13038431	G	0.2423	0.1925	A	0.03607	1.341
18	rs3737379	13100451	G	0.2423	0.1925	A	0.03607	1.341
4	rs28672282	40671548	A	0.3479	0.4092	G	0.0364	0.7705
6	rs6941112	31946614	A	0.2629	0.32	G	0.03648	0.7579
20	rs2272955	44184498	G	0.1521	0.2008	A	0.03654	0.7136
10	rs77678145	115377290	G	0.1753	0.1317	A	0.03658	1.401
1	rs3829740	909238	C	0.07732	0.115	G	0.03672	0.6449
14	rs2303345	76156609	A	0.4407	0.3808	G	0.0368	1.281
8	rs17636067	17915126	A	0.04639	0.02417	G	0.03682	1.964
13	rs17773251	37678884	G	0.04639	0.02417	C	0.03682	1.964
8	rs1553412	788912	A	0.2448	0.195	G	0.03687	1.338
19	rs11882217	1709658	A	0.3892	0.3308	G	0.03692	1.289
16	rs249954	23640467	A	0.3557	0.4167	G	0.03702	0.7728
11	rs4909989	11359886	G	0.2655	0.3217	A	0.03708	0.7621
2	rs10930046	163137983	G	0.1108	0.1542	A	0.03716	0.6838
19	rs306496	56477710	A	0.3222	0.2658	G	0.03721	1.313
3	rs9828224	95906561	G	0.3582	0.4192	A	0.03722	0.7735
3	rs11918974	157081324	G	0.451	0.39	A	0.03727	1.285
13	rs3742321	33704065	G	0.1907	0.2425	A	0.03728	0.7362
2	rs61741435	170510651	A	0.1366	0.0975	G	0.03733	1.464
2	rs2070094	215632255	A	0.268	0.3258	G	0.03737	0.7577
17	rs2138852	27703349	G	0.01031	0.02917	A	0.03744	0.3467
6	rs1264570	30365210	G	0.4536	0.3925	A	0.03745	1.285
6	rs3815087	31093587	A	0.3222	0.2667	G	0.03746	1.307
10	rs9420382	87862479	G	0.451	0.3908	A	0.03748	1.281
3	rs11552724	49156473	G	0.03866	0.0675	C	0.03752	0.5556
7	rs2961160	143826603	C	0.1546	0.2033	A	0.03752	0.7167
3	rs4955419	49200692	A	0.03866	0.06833	T	0.03755	0.5483
6	rs8337	29523676	C	0.03866	0.06833	G	0.03755	0.5483
6	rs3129948	32354644	C	0.2474	0.1975	A	0.03767	1.336
6	rs3129954	32365580	A	0.2474	0.1975	G	0.03767	1.336
7	rs17169263	16133713	G	0.4588	0.3973	A	0.03775	1.286
7	rs3735319	149152770	A	0.2113	0.1633	G	0.03777	1.373
6	rs3893538	29924425	G	0.3093	0.3683	A	0.0378	0.7679
15	rs936212	40581543	A	0.3093	0.3683	G	0.0378	0.7679

6	rs2071475	32782387	A	0.2397	0.2942	G	0.03784	0.7564
11	rs110419	8252853	G	0.3093	0.3675	A	0.03792	0.7706
14	rs12883384	47403684	C	0.3093	0.3675	A	0.03792	0.7706
6	rs34816476	31557381	A	0.03351	0.01583	G	0.03794	2.155
10	rs11551267	118424362	A	0.03351	0.01583	G	0.03794	2.155
12	rs73056605	7894056	A	0.2964	0.2425	G	0.03794	1.316
12	rs138916804	70932745	A	0.03351	0.01583	G	0.03794	2.155
14	rs10498514	64899055	C	0.03351	0.01583	A	0.03794	2.155
14	rs2295639	64902116	G	0.03351	0.01583	A	0.03794	2.155
1	rs2992754	18807938	C	0.366	0.4258	A	0.03796	0.7783
16	rs9925415	72007399	A	0.4588	0.3983	G	0.03796	1.28
17	rs61757652	78178916	A	0.1134	0.07833	G	0.03797	1.505
3	rs118154115	12977188	A	0.002577	0.01667	G	0.03804	0.1525
5	rs10067096	156917340	C	0.002577	0.01667	A	0.03804	0.1525
5	rs2287749	156918850	A	0.002577	0.01667	G	0.03804	0.1525
1	rs76187620	162551137	G	0.002577	0.01583	A	0.03812	0.1606
12	rs140958087	11183456	A	0.002577	0.01583	C	0.03812	0.1606
19	rs34577345	12911709	A	0.002577	0.01583	G	0.03812	0.1606
5	rs758459	134872704	G	0.1366	0.09833	A	0.03824	1.451
7	rs3750092	29924072	G	0.2423	0.2983	A	0.03825	0.752
9	rs2075663	101748356	A	0.3119	0.37	G	0.03825	0.7716
17	rs9913317	78247124	A	0.01031	0.03	G	0.03826	0.3368
16	rs904763	71773190	A	0.2165	0.2708	C	0.03829	0.7439
17	rs1231206	2125605	A	0.3273	0.2713	G	0.03831	1.307
17	rs216172	2126504	C	0.3273	0.2713	G	0.03831	1.307
8	rs6992370	28048668	C	0.01804	0.04	A	0.03836	0.4409
22	rs4821469	36616445	G	0.01804	0.04	A	0.03836	0.4409
3	rs183217537	147108757	A	0.01804	0.04083	G	0.03844	0.4316
19	rs80087697	54578105	A	0.01804	0.04083	G	0.03844	0.4316
5	rs10074434	134489182	C	0.3608	0.3038	A	0.03851	1.293
3	rs1801019	124456742	G	0.299	0.245	C	0.03854	1.314
17	rs72818370	34893326	G	0.1134	0.1567	A	0.03858	0.6885
17	rs3803752	56540267	A	0.1753	0.225	G	0.03861	0.7319
20	rs6025351	55650465	A	0.2758	0.3333	G	0.03863	0.7616
9	rs7862326	87864354	A	0.3608	0.3042	G	0.03869	1.291
9	rs17723637	109687403	G	0.1031	0.07	A	0.0387	1.527
11	rs2301574	119063908	A	0.3789	0.4392	G	0.0387	0.7789
15	rs269868	45392075	G	0.1031	0.07	A	0.0387	1.527
3	rs6805320	65127595	C	0.4072	0.3475	A	0.03875	1.29
6	rs17710008	153043035	A	0.09021	0.13	G	0.03875	0.6635
6	rs9395066	45095163	A	0.3634	0.3058	C	0.03876	1.296
17	rs9891146	65988049	G	0.2448	0.3	A	0.03876	0.7565
19	rs10409555	56572832	A	0.3814	0.4425	G	0.03877	0.7769

4	rs7439869	95173779	A	0.1959	0.2475	G	0.03879	0.7406
6	rs1052502	135606565	A	0.0799	0.1175	G	0.03879	0.6522
19	rs12610410	57175578	A	0.1425	0.1886	G	0.03883	0.7146
4	rs2276887	38990575	G	0.09278	0.06167	A	0.03884	1.556
19	rs2287019	46202172	A	0.1418	0.1892	G	0.03886	0.708
12	rs2229373	133683020	A	0.3814	0.4417	G	0.03887	0.7796
7	rs10486567	27976563	G	0.09021	0.1292	A	0.03888	0.6685
3	rs189897	37518545	A	0.06186	0.09667	T	0.03902	0.6161
10	rs78752313	129854414	T	0.06186	0.09667	A	0.03902	0.6161
3	rs9288952	112185025	G	0.3015	0.2475	A	0.03914	1.313
12	rs890693	114484640	A	0.3892	0.45	C	0.03921	0.7787
21	rs3746864	35515333	G	0.03351	0.06167	A	0.03921	0.5275
7	rs4580999	50546261	A	0.3964	0.4564	G	0.03929	0.782
11	rs10902221	802379	G	0.3299	0.275	A	0.03935	1.298
13	rs12146904	52700470	A	0.4871	0.4258	G	0.03946	1.281
6	rs605203	31847012	C	0.116	0.08	A	0.03948	1.509
6	rs486416	31856070	G	0.116	0.08	A	0.03948	1.509
7	rs2373396	88424115	G	0.116	0.08	C	0.03948	1.509
2	rs72786179	29225504	G	0.1985	0.2508	A	0.03954	0.7395
6	rs2251396	31364707	A	0.2565	0.205	G	0.03954	1.338
8	rs4392877	132007359	G	0.3686	0.3108	A	0.03955	1.294
12	rs11049488	28412372	A	0.08505	0.055	G	0.03965	1.597
20	rs6113722	22557099	A	0.1289	0.1742	G	0.03966	0.7014
5	rs2546890	158759900	A	0.3943	0.4542	G	0.03967	0.7825
2	rs75011196	168099724	G	0.05412	0.03	A	0.03969	1.85
5	rs12516652	115811248	A	0.05412	0.03	C	0.03969	1.85
16	rs2966122	81504477	A	0.4897	0.4292	G	0.0397	1.276
3	rs139806311	56628055	G	0.05928	0.035	A	0.03972	1.737
17	rs28903	31336980	C	0.05928	0.035	A	0.03972	1.737
2	rs28399164	231083132	G	0.3995	0.46	A	0.03974	0.7809
10	rs7901695	114754088	G	0.007732	0.025	A	0.03974	0.3039
10	rs4506565	114756041	T	0.007732	0.025	A	0.03974	0.3039
10	rs7903146	114758349	A	0.007732	0.025	G	0.03974	0.3039
15	rs8041035	91496242	A	0.007732	0.025	G	0.03974	0.3039
19	rs114849139	36330456	G	0.007732	0.025	C	0.03974	0.3039
20	rs4911494	33971914	G	0.3041	0.25	A	0.03974	1.311
4	rs6552216	178112004	G	0.1031	0.145	A	0.03984	0.6778
1	rs9697293	981131	G	0.0232	0.0475	A	0.03985	0.4762
6	rs1042149	31082960	A	0.2835	0.3408	G	0.03986	0.7652
6	rs3094212	31085770	G	0.2835	0.3408	A	0.03986	0.7652
14	rs941590	94756794	A	0.2835	0.3408	G	0.03986	0.7652
15	rs3743315	70960582	G	0.02835	0.05417	A	0.03988	0.5095
6	rs2306942	129635800	A	0.1985	0.1525	G	0.03994	1.376

15	rs6493068	43170793	G	0.3995	0.4592	A	0.03996	0.7836
4	rs2639576	145658429	A	0.4072	0.4675	G	0.04011	0.7825
3	rs1042779	52821011	G	0.5026	0.4417	A	0.04014	1.277
11	rs11554663	824428	A	0.2242	0.2783	C	0.04015	0.7494
16	rs74636066	820622	A	0.2242	0.2783	G	0.04015	0.7494
14	rs10498345	39020505	A	0.2784	0.2267	T	0.04018	1.316
6	rs1265155	31143694	A	0.5052	0.4442	G	0.04021	1.277
19	rs13343696	44116956	G	0.1675	0.125	A	0.04021	1.409
1	rs10489177	169762807	C	0.2165	0.1692	A	0.04031	1.357
2	rs138478634	72360331	A	0.02835	0.055	G	0.04035	0.5013
16	rs61587627	1273444	A	0.09536	0.06333	C	0.04036	1.559
1	rs1188697	228468458	A	0.1804	0.23	G	0.04037	0.7369
10	rs41314978	27499979	A	0.07216	0.1083	C	0.04037	0.6402
1	rs61746299	151773851	A	0.2861	0.3425	T	0.0404	0.7693
10	rs3818780	99439673	C	0.2809	0.2283	G	0.04045	1.32
18	rs745894	66542006	A	0.116	0.08083	C	0.04045	1.492
2	rs148994064	209204243	A	0.005155	0.02083	G	0.04048	0.2435
4	rs2319850	169348404	A	0.005155	0.02083	G	0.04048	0.2435
5	rs183679247	141023817	A	0.005155	0.02083	C	0.04048	0.2435
11	rs79267863	5364013	A	0.005155	0.02083	G	0.04048	0.2435
1	rs17110736	94462769	A	0.07216	0.1092	G	0.04058	0.6347
2	rs334044	179102232	A	0.2371	0.1875	G	0.04059	1.347
2	rs4676406	241579108	C	0.06186	0.03583	A	0.04065	1.774
6	rs12525163	152040291	G	0.183	0.1392	A	0.04066	1.385
21	rs11909383	36646280	C	0.5232	0.4625	A	0.04072	1.275
1	rs627928	182551337	A	0.2268	0.2808	C	0.04077	0.7512
1	rs12733856	221551539	C	0.4253	0.3658	A	0.04077	1.283
6	rs6900672	110239704	G	0.2268	0.2808	A	0.04077	0.7512
13	rs9523451	92574422	A	0.2268	0.2808	G	0.04077	0.7512
15	rs11854243	29803473	A	0.5309	0.47	C	0.0408	1.276
4	rs6815580	137539440	G	0.518	0.4583	A	0.04092	1.27
5	rs2006889	38264735	G	0.3763	0.3192	A	0.04092	1.287
8	rs2304787	87568644	C	0.3093	0.255	A	0.04093	1.308
9	rs1183768	135203231	A	0.3093	0.255	G	0.04093	1.308
3	rs9841174	167184878	A	0.4536	0.5142	G	0.04095	0.7844
4	rs1986734	77420784	G	0.5232	0.4633	A	0.04096	1.271
2	rs1058180	191161622	G	0.5438	0.4833	A	0.04097	1.274
6	rs2917862	74521947	A	0.5438	0.4833	G	0.04097	1.274
5	rs74794265	65473481	A	0.007732	0.02583	G	0.04098	0.2938
5	rs13156607	168899987	A	0.007732	0.02583	G	0.04098	0.2938
9	rs12236253	32542801	A	0.007732	0.02583	G	0.04098	0.2938
9	rs3829078	35679251	G	0.007732	0.02583	A	0.04098	0.2938
14	rs143695335	96773164	G	0.007732	0.02583	A	0.04098	0.2938

17	rs2230463	1456318	A	0.007732	0.02583	G	0.04098	0.2938
6	rs806794	26200677	A	0.2036	0.2558	G	0.04099	0.7437
6	rs407161	29513807	A	0.5387	0.4783	G	0.04099	1.273
6	rs429853	32187202	G	0.2036	0.2558	A	0.04099	0.7437
6	rs4713604	32979770	C	0.4253	0.3667	G	0.04099	1.278
6	rs4713605	32985992	A	0.4253	0.3667	T	0.04099	1.278
6	rs1892172	127476516	G	0.5387	0.4783	A	0.04099	1.273
12	rs10771604	30098770	A	0.5361	0.4758	G	0.04099	1.273
1	rs183715673	108138962	G	0.02577	0.01	A	0.04102	2.619
7	rs10235371	50514577	A	0.02577	0.01	G	0.04102	2.619
7	rs17545756	150732812	A	0.02577	0.01	G	0.04102	2.619
12	rs216321	6143984	A	0.1469	0.1933	G	0.04102	0.7185
15	rs2290552	42134763	A	0.02577	0.01	G	0.04102	2.619
20	rs184663098	48729709	C	0.02577	0.01002	G	0.0411	2.615
2	rs1060743	127826533	G	0.4304	0.3708	A	0.04127	1.282
18	rs7227401	21938658	A	0.1418	0.1025	C	0.04129	1.446
8	rs2290416	144657600	A	0.1546	0.1143	G	0.04135	1.418
3	rs3922844	38624253	A	0.1546	0.1142	G	0.04136	1.419
9	rs2282258	139649612	A	0.1186	0.1614	C	0.04142	0.699
1	rs2258470	62728838	A	0.1057	0.1475	G	0.04144	0.6829
7	rs41510047	69867401	G	0.1057	0.1475	A	0.04144	0.6829
1	rs2274531	167042762	A	0.183	0.14	G	0.04147	1.376
1	rs3766871	237778084	A	0.1057	0.0725	G	0.0415	1.512
6	rs2844523	31368588	A	0.06443	0.09917	G	0.04164	0.6256
6	rs3887152	31176335	A	0.3428	0.2876	G	0.04169	1.292
5	rs76469644	176761336	G	0.06959	0.0425	A	0.0417	1.685
7	rs6973420	134618710	A	0.06443	0.09833	G	0.04174	0.6315
8	rs10504826	87690019	A	0.1701	0.1275	G	0.04178	1.403
11	rs2045737	38306585	C	0.2062	0.2575	A	0.0418	0.749
4	rs6849055	163939622	A	0.4356	0.3769	G	0.04187	1.276
1	rs2274329	9009451	G	0.2397	0.1908	C	0.0419	1.337
1	rs58030082	186313108	A	0.07732	0.04917	G	0.04198	1.621
6	rs2151910	150211123	T	0.09536	0.06417	A	0.042	1.537
8	rs4875310	4103130	G	0.3479	0.4067	A	0.04202	0.7785
14	rs3811182	23549785	G	0.3479	0.4067	A	0.04202	0.7785
9	rs17459086	131857687	G	0.08763	0.05667	C	0.04204	1.599
1	rs2298805	159273943	A	0.1186	0.0825	G	0.04205	1.496
3	rs2624901	128677188	A	0.4381	0.3792	G	0.04212	1.277
19	rs73027451	38375666	A	0.1418	0.1033	G	0.04212	1.433
4	rs77524902	17819695	A	0.08763	0.05676	G	0.04213	1.596
19	rs12611158	12186272	A	0.0567	0.08932	G	0.04232	0.6129
5	rs7736233	127031523	A	0.384	0.3275	G	0.04234	1.28
14	rs6571751	21770730	G	0.3866	0.3292	A	0.04235	1.284

8	rs2230801	90784979	G	0.03093	0.01333	A	0.04238	2.362
11	rs148008576	864473	A	0.03093	0.01333	G	0.04238	2.362
12	rs1136125	56115233	A	0.03093	0.01333	G	0.04238	2.362
1	rs3754413	226924196	A	0.0567	0.08917	G	0.04239	0.614
3	rs9866564	175345143	C	0.0567	0.08917	G	0.04239	0.614
15	rs7180279	42643538	G	0.0567	0.08917	A	0.04239	0.614
20	rs6070697	57599402	A	0.09536	0.135	G	0.04242	0.6754
2	rs78140344	202097532	D	0.2629	0.2125	I	0.04248	1.322
17	rs692308	76467946	G	0.1675	0.2167	A	0.0425	0.7276
3	rs2302815	172365832	C	0.09536	0.1358	A	0.04257	0.6706
21	rs2839627	44448718	A	0.09536	0.1358	G	0.04257	0.6706
1	rs12093699	159647988	A	0.04897	0.08083	G	0.04269	0.5855
8	rs1800392	30973957	C	0.3557	0.4142	A	0.04276	0.7808
1	rs2282301	155868625	G	0.2423	0.1933	A	0.04283	1.334
1	rs699	230845794	A	0.2423	0.1933	G	0.04283	1.334
18	rs2027698	13092424	A	0.2423	0.1933	G	0.04283	1.334
19	rs2285745	36590329	A	0.3582	0.4175	G	0.04289	0.7789
3	rs7642497	190207425	A	0.3041	0.3617	C	0.04293	0.7714
4	rs3733616	114277041	A	0.02062	0.0075	G	0.04293	2.786
11	rs4937410	129162711	A	0.3041	0.3617	G	0.04293	0.7714
13	rs58265842	103399837	A	0.02062	0.0075	C	0.04293	2.786
13	rs17592564	103400033	G	0.02062	0.0075	A	0.04293	2.786
14	rs145440362	61510196	A	0.02062	0.0075	C	0.04293	2.786
20	rs138897849	31644401	A	0.02062	0.0075	C	0.04293	2.786
20	rs140765002	31671244	A	0.02062	0.0075	G	0.04293	2.786
22	rs148386625	30661028	G	0.02062	0.0075	A	0.04293	2.786
16	rs8057454	21264441	C	0.1186	0.08333	A	0.04296	1.48
3	rs1515442	172835125	G	0.451	0.3917	A	0.04315	1.276
6	rs210132	33536670	A	0.3067	0.3642	C	0.04329	0.7724
16	rs2294451	847743	A	0.2371	0.29	G	0.04334	0.761
11	rs4944960	74559446	C	0.4536	0.3942	G	0.04335	1.276
7	rs7793993	12417407	G	0.01546	0.03667	A	0.04339	0.4127
12	rs146851850	52995098	G	0.01546	0.03667	A	0.04339	0.4127
17	rs11655512	20851735	G	0.1701	0.2183	A	0.04341	0.7338
19	rs8012	13010520	A	0.1366	0.182	G	0.04343	0.7112
15	rs12101559	42742464	G	0.1366	0.1817	A	0.04345	0.7127
15	rs1648348	50474766	A	0.1366	0.1817	C	0.04345	0.7127
21	rs2073376	47851753	A	0.3531	0.2975	G	0.04352	1.289
10	rs10749138	115370274	A	0.4536	0.395	G	0.04355	1.272
10	rs61850830	63977980	A	0.09794	0.06583	G	0.04364	1.541
6	rs769177	31547611	A	0.03608	0.06417	G	0.04367	0.5459
9	rs4879782	34372873	G	0.03608	0.06417	C	0.04367	0.5459
3	rs9289628	98267289	G	0.3093	0.3658	A	0.04377	0.7762

2	rs2681019	23187504	C	0.3222	0.2675	A	0.04385	1.301
6	rs2857106	32787570	G	0.2397	0.2925	A	0.04397	0.7625
1	rs2378789	183918820	C	0.3995	0.3417	A	0.04404	1.282
11	rs11036814	5344592	C	0.2062	0.1608	A	0.04411	1.355
22	rs2284033	37534034	G	0.3119	0.3683	A	0.04414	0.7772
2	rs7560430	150572567	G	0.3711	0.4292	A	0.04415	0.785
1	rs140144285	15986623	A	0.01289	0.003333	G	0.04417	3.903
1	rs145751121	150471443	A	0.01289	0.003333	G	0.04417	3.903
2	rs149284367	26676222	A	0.01289	0.003333	G	0.04417	3.903
2	rs75335764	216965002	A	0.01289	0.003333	G	0.04417	3.903
4	rs41298245	70346408	G	0.01289	0.003333	A	0.04417	3.903
4	rs145995401	110763764	A	0.01289	0.003333	G	0.04417	3.903
5	rs201956987	195179	A	0.01289	0.003333	G	0.04417	3.903
6	rs2231365	3127644	G	0.01289	0.003333	A	0.04417	3.903
9	rs200270101	119976780	A	0.01289	0.003333	T	0.04417	3.903
9	rs148684713	131107679	A	0.01289	0.003333	G	0.04417	3.903
13	rs199744730	25743803	A	0.01289	0.003333	G	0.04417	3.903
15	rs151021606	99759194	G	0.01289	0.003333	A	0.04417	3.903
16	rs201303478	68309144	A	0.01289	0.003333	G	0.04417	3.903
20	rs201133429	35507556	A	0.01289	0.003333	G	0.04417	3.903
15	rs12899618	71645120	A	0.06959	0.04341	G	0.04419	1.648
6	rs2227955	31778077	C	0.03608	0.0175	A	0.04422	2.102
6	rs4947332	31834197	A	0.03608	0.0175	G	0.04422	2.102
9	rs41302077	4617999	C	0.03608	0.0175	G	0.04422	2.102
9	rs45463394	4625415	G	0.03608	0.0175	A	0.04422	2.102
22	rs140758033	38508172	G	0.03608	0.0175	C	0.04422	2.102
5	rs1279736	180671189	A	0.2732	0.3283	C	0.04427	0.7689
20	rs147239130	33588792	A	0.01289	0.003339	G	0.0444	3.897
11	rs7951829	91178089	G	0.1237	0.1675	A	0.04441	0.7017
3	rs9876345	25423265	G	0.2423	0.2967	A	0.04443	0.758
6	rs2471980	31800868	G	0.3144	0.3708	C	0.04451	0.7782
11	rs61756355	5632018	A	0.0799	0.05083	C	0.04457	1.621
14	rs75345169	31598301	G	0.0799	0.05083	A	0.04457	1.621
18	rs2278154	72103782	G	0.1933	0.2442	A	0.04458	0.7417
1	rs1859728	44447413	C	0.1108	0.1525	G	0.04467	0.6927
18	rs1344883	51908403	G	0.3247	0.2708	A	0.04467	1.295
1	rs4275414	182854200	A	0.2088	0.1625	G	0.04486	1.36
11	rs654723	128586155	C	0.4716	0.4133	A	0.04494	1.267
16	rs935813	63717319	A	0.3866	0.4458	G	0.04494	0.7834
20	rs487656	58790814	G	0.2448	0.2983	A	0.045	0.7626
4	rs76245024	20714522	C	0.04897	0.02667	A	0.04502	1.879
5	rs79390966	118500288	A	0.04897	0.02667	G	0.04502	1.879
13	rs9314973	30332070	C	0.3866	0.445	A	0.04508	0.786

11	rs79043854	56431667	A	0.07732	0.1142	G	0.04519	0.6502
20	rs6115026	25067283	G	0.3196	0.3758	A	0.04523	0.7801
5	rs282414	73236680	G	0.2088	0.1633	A	0.04531	1.352
14	rs35618680	24572812	A	0.05928	0.09197	G	0.04531	0.6221
20	rs56057707	57768743	A	0.2088	0.1633	G	0.04531	1.352
20	rs55786258	57769140	C	0.2088	0.1633	G	0.04531	1.352
1	rs118188721	39802283	C	0.06186	0.0375	G	0.04533	1.692
1	rs7544736	97164343	G	0.1753	0.2242	A	0.04536	0.7355
6	rs1619376	30983326	A	0.1959	0.2458	G	0.04538	0.7473
12	rs3809228	32136750	G	0.1753	0.2233	A	0.04538	0.739
19	rs269912	55481398	A	0.08763	0.05833	G	0.04538	1.55
6	rs3756772	116325142	G	0.2964	0.245	A	0.04542	1.298
3	rs2293203	183017965	A	0.05928	0.0925	T	0.04547	0.6182
6	rs4959505	8869571	G	0.3969	0.4558	A	0.04555	0.7857
5	rs457717	75920972	A	0.3943	0.4525	G	0.0456	0.7878
15	rs7497757	24850224	A	0.3943	0.4525	G	0.0456	0.7878
17	rs8066842	55264833	G	0.3634	0.3083	A	0.04561	1.281
2	rs717366	164731394	G	0.4871	0.4283	A	0.04566	1.268
3	rs59232004	124738107	A	0.4871	0.4283	G	0.04566	1.268
3	rs2981546	124746049	G	0.4871	0.4283	A	0.04566	1.268
5	rs10052657	58407771	A	0.2294	0.1817	C	0.04566	1.341
7	rs6966038	156880398	G	0.02062	0.04333	A	0.04566	0.4648
12	rs142330429	15038703	A	0.02062	0.04333	G	0.04566	0.4648
2	rs75542756	189914122	A	0.05412	0.03167	G	0.04574	1.75
20	rs1803415	1099523	C	0.4021	0.4617	A	0.04576	0.7841
5	rs61734326	121356189	A	0.05155	0.08264	T	0.0458	0.6033
5	rs16876657	78610174	G	0.05155	0.0825	A	0.04584	0.6044
2	rs4671709	66285871	A	0.4046	0.4642	G	0.04587	0.7846
14	rs1377935	25843774	A	0.3995	0.4575	G	0.04592	0.7888
12	rs10746091	107842206	G	0.4046	0.4633	A	0.04596	0.7872
1	rs2642531	203143725	G	0.1418	0.1867	C	0.04598	0.7196
6	rs241405	32865798	A	0.4072	0.4667	G	0.04599	0.7851
6	rs154989	32877173	A	0.4072	0.4667	G	0.04599	0.7851
11	rs10831284	94667964	G	0.02577	0.05	A	0.04603	0.5026
12	rs7297018	80062230	A	0.1108	0.07667	G	0.04605	1.501
21	rs7279142	32410582	A	0.1108	0.07667	G	0.04605	1.501
6	rs4709243	157854806	G	0.2835	0.3392	A	0.04606	0.771
10	rs12250999	88364717	A	0.5	0.4408	G	0.04615	1.268
17	rs1131636	1801189	G	0.5	0.4408	A	0.04615	1.268
5	rs6870619	78996050	G	0.4124	0.4716	A	0.04616	0.7862
6	rs1541267	30103571	G	0.2732	0.2233	A	0.04616	1.307
6	rs1557608	30118582	C	0.2732	0.2233	A	0.04616	1.307
20	rs2281929	62422080	G	0.4948	0.4367	A	0.04623	1.264

6	rs887466	31143511	A	0.5052	0.4457	G	0.04626	1.269
17	rs4143218	6683848	A	0.0799	0.05167	T	0.04626	1.594
2	rs1048135	152139416	A	0.4149	0.4742	G	0.0463	0.7865
13	rs35680839	100511247	A	0.1134	0.155	G	0.0463	0.6973
3	rs2612027	53776678	G	0.4974	0.4392	A	0.04631	1.264
8	rs4732620	27925204	A	0.3299	0.3867	G	0.04636	0.7809
17	rs34818467	56643109	G	0.1778	0.2258	A	0.04636	0.7415
1	rs200952273	23232567	A	0	0.01	T	0.04642	0
1	rs141618798	44075054	A	0	0.01	G	0.04642	0
1	rs182775321	156212567	A	0	0.01	C	0.04642	0
2	rs112050262	108863758	A	0	0.01	G	0.04642	0
2	rs111455491	108875267	G	0	0.01	A	0.04642	0
3	rs59315778	8667325	A	0	0.01	G	0.04642	0
4	rs187602678	57250291	A	0	0.01	G	0.04642	0
4	rs200857997	185580578	C	0	0.01	A	0.04642	0
6	rs10946705	24556936	C	0	0.01	G	0.04642	0
6	rs8192591	32185796	A	0	0.01	G	0.04642	0
6	rs118018352	89888752	A	0	0.01	G	0.04642	0
7	rs184047949	48353853	G	0	0.01	A	0.04642	0
7	rs73727633	149509079	G	0	0.01	A	0.04642	0
10	rs138700544	92503408	C	0	0.01	G	0.04642	0
10	rs112589253	98064363	A	0	0.01	G	0.04642	0
15	rs139788541	90020806	A	0	0.01	G	0.04642	0
16	rs200755220	30665561	G	0	0.01	A	0.04642	0
17	rs138794420	58034638	A	0	0.01	G	0.04642	0
19	rs141198839	56370047	A	0	0.01	G	0.04642	0
20	rs141536016	18377116	A	0	0.01	G	0.04642	0
20	rs6141383	31889141	A	0	0.01	G	0.04642	0
22	rs117353193	31010417	A	0	0.01	G	0.04642	0
21	rs2013275	46334472	A	0.4149	0.4733	G	0.04646	0.7892
14	rs217676	62363763	G	0.5129	0.4533	A	0.04654	1.27
5	rs7718054	150908812	A	0.09067	0.06	G	0.04662	1.562
1	rs7549251	55304970	A	0.4201	0.3625	G	0.04666	1.274
5	rs17245714	34998894	C	0.02577	0.05083	G	0.04667	0.494
15	rs73400524	41148516	A	0.1598	0.2058	G	0.04668	0.7338
4	rs2306985	100516022	G	0.3325	0.2792	C	0.0467	1.286
1	rs11249433	121280613	G	0.0567	0.0325	A	0.04671	1.789
6	rs1999805	152068364	A	0.3015	0.25	G	0.04671	1.295
17	rs150857	3526637	A	0.232	0.1842	G	0.04673	1.338
18	rs142178302	8394505	A	0.007812	0.0008361	G	0.04674	9.409
1	rs116513797	182909488	G	0.03093	0.01417	A	0.04676	2.221
4	rs2498323	3451109	A	0.03093	0.01417	G	0.04676	2.221
10	rs118037636	30318743	A	0.03093	0.01417	G	0.04676	2.221

15	rs3803335	43817225	A	0.03093	0.01417	G	0.04676	2.221
5	rs6897511	118636227	G	0.134	0.0975	A	0.04678	1.433
8	rs2037213	33342018	A	0.433	0.4925	C	0.04678	0.7869
2	rs115549137	27898405	A	0	0.01083	G	0.04679	0
2	rs80328470	54081815	G	0	0.01083	A	0.04679	0
4	rs185899169	47685768	C	0	0.01083	A	0.04679	0
6	rs147878071	38998119	A	0	0.01083	G	0.04679	0
13	rs2070591	78492734	A	0	0.01083	G	0.04679	0
17	rs200313254	3558553	C	0	0.01083	A	0.04679	0
17	rs77630697	19445761	A	0	0.01083	G	0.04679	0
17	rs3744356	33313129	A	0	0.01083	G	0.04679	0
19	rs2302947	49100068	A	0	0.01083	G	0.04679	0
20	rs6028466	38129002	A	0	0.01083	G	0.04679	0
2	rs184614603	239040093	A	0	0.01085	G	0.0468	0
6	rs805301	31618121	C	0.4227	0.4808	A	0.0468	0.7905
6	rs805281	31661489	G	0.4227	0.4808	A	0.0468	0.7905
6	rs805274	31665194	G	0.4227	0.4808	A	0.0468	0.7905
3	rs16839611	98002587	A	0.1933	0.15	G	0.04684	1.358
11	rs12420456	64981837	A	0.3351	0.2808	G	0.04685	1.29
9	rs10465129	115950156	A	0.2758	0.2258	G	0.04693	1.305
16	rs3743599	75646576	C	0.2758	0.2258	A	0.04693	1.305
12	rs254694	115589524	G	0.4227	0.365	A	0.04694	1.274
7	rs1451375	50622712	A	0.5412	0.4817	C	0.04699	1.27
2	rs7588910	27384579	A	0.4278	0.4858	G	0.047	0.7914
1	rs2282316	247875415	G	0.201	0.2517	A	0.04702	0.7482
1	rs116931058	201352291	A	0.02577	0.01083	G	0.04709	2.416
5	rs292182	36954812	A	0.5232	0.465	G	0.04709	1.262
8	rs2306131	99152322	C	0.02577	0.01083	A	0.04709	2.416
11	rs35142528	16812397	A	0.09021	0.1283	G	0.04709	0.6734
14	rs75497728	24568323	A	0.02577	0.01083	G	0.04709	2.416
19	rs76423334	46307742	A	0.02577	0.01083	G	0.04709	2.416
9	rs11243372	133324400	A	0.5412	0.4825	G	0.04711	1.265
11	rs577948	122030190	G	0.5258	0.4675	A	0.04711	1.263
7	rs214455	110843795	G	0.5387	0.48	A	0.04712	1.265
6	rs1265048	31081409	A	0.4433	0.5017	G	0.04715	0.791
9	rs11143193	74853935	A	0.4459	0.5042	G	0.0472	0.7914
11	rs78682406	119206912	A	0.1108	0.0775	C	0.04721	1.484
16	rs9790	3586230	A	0.09067	0.1283	G	0.04723	0.6773
1	rs11265263	159710517	A	0.2113	0.1667	C	0.04724	1.34
3	rs16846616	172835082	A	0.3737	0.3183	G	0.04724	1.278
3	rs1515441	172835290	A	0.3737	0.3183	G	0.04724	1.278
11	rs11031420	31609964	G	0.3737	0.3183	A	0.04724	1.278
4	rs12644284	154154000	G	0.06959	0.105	A	0.04725	0.6375

4	rs763318	12963574	A	0.4536	0.5117	G	0.04733	0.7923
1	rs435776	228494790	A	0.1804	0.2283	G	0.04734	0.7439
4	rs13146355	77412140	A	0.1804	0.2283	G	0.04734	0.7439
1	rs201070239	156500033	A	0.007732	0.0008333	G	0.04744	9.343
1	rs201490397	156756568	A	0.007732	0.0008333	G	0.04744	9.343
1	rs199744208	169773337	G	0.007732	0.0008333	A	0.04744	9.343
1	rs181912115	200635460	C	0.007732	0.0008333	G	0.04744	9.343
1	rs202238030	200635541	A	0.007732	0.0008333	C	0.04744	9.343
2	rs199631736	159389749	A	0.007732	0.0008333	C	0.04744	9.343
2	rs144025747	163228386	A	0.007732	0.0008333	G	0.04744	9.343
2	rs185002960	179427796	A	0.007732	0.0008333	G	0.04744	9.343
2	rs4675743	209087335	C	0.007732	0.0008333	A	0.04744	9.343
3	rs149263615	114018469	A	0.007732	0.0008333	C	0.04744	9.343
3	rs200831051	118865247	C	0.007732	0.0008333	G	0.04744	9.343
3	rs201512803	128181970	G	0.007732	0.0008333	A	0.04744	9.343
4	rs189070828	8584332	A	0.007732	0.0008333	G	0.04744	9.343
4	rs201835999	173930407	G	0.007732	0.0008333	C	0.04744	9.343
5	rs148267849	7869328	A	0.007732	0.0008333	C	0.04744	9.343
5	rs200644004	90074281	G	0.007732	0.0008333	A	0.04744	9.343
5	rs28365994	150632855	T	0.007732	0.0008333	A	0.04744	9.343
6	rs45626535	136913615	A	0.007732	0.0008333	G	0.04744	9.343
6	rs202105909	167343195	A	0.007732	0.0008333	G	0.04744	9.343
7	rs144871193	76058841	A	0.007732	0.0008333	G	0.04744	9.343
7	rs139293638	101923374	A	0.007732	0.0008333	G	0.04744	9.343
7	rs114190729	103197510	A	0.007732	0.0008333	G	0.04744	9.343
7	rs200099984	149557819	A	0.007732	0.0008333	G	0.04744	9.343
7	rs200565750	149558506	A	0.007732	0.0008333	G	0.04744	9.343
8	rs181035264	116426988	A	0.007732	0.0008333	T	0.04744	9.343
8	rs199720565	144997240	A	0.007732	0.0008333	G	0.04744	9.343
9	rs150206751	75420340	A	0.007732	0.0008333	G	0.04744	9.343
9	rs11140241	86383740	G	0.007732	0.0008333	A	0.04744	9.343
9	rs141024100	134165546	A	0.007732	0.0008333	C	0.04744	9.343
10	rs74334349	50873028	A	0.007732	0.0008333	G	0.04744	9.343
10	rs181886504	125521558	A	0.007732	0.0008333	G	0.04744	9.343
10	rs151009069	134622301	A	0.007732	0.0008333	G	0.04744	9.343
11	rs199739008	617452	A	0.007732	0.0008333	G	0.04744	9.343
11	rs201504740	621395	A	0.007732	0.0008333	C	0.04744	9.343
11	rs189440061	69949220	A	0.007732	0.0008333	G	0.04744	9.343
11	rs12222256	102201804	A	0.007732	0.0008333	G	0.04744	9.343
11	rs191029144	124545233	A	0.007732	0.0008333	G	0.04744	9.343
12	rs142370738	6669359	G	0.007732	0.0008333	C	0.04744	9.343
12	rs11539445	32908237	A	0.007732	0.0008333	C	0.04744	9.343
12	rs151223138	123089499	C	0.007732	0.0008333	G	0.04744	9.343

13	rs138014271	25272832	A	0.007732	0.0008333	G	0.04744	9.343
14	rs61732411	22134018	A	0.007732	0.0008333	G	0.04744	9.343
14	rs191597373	74206467	A	0.007732	0.0008333	G	0.04744	9.343
16	rs200498279	12136831	A	0.007732	0.0008333	G	0.04744	9.343
16	rs139478274	89805325	A	0.007732	0.0008333	G	0.04744	9.343
16	rs56369086	89838136	G	0.007732	0.0008333	A	0.04744	9.343
17	rs188469309	694997	A	0.007732	0.0008333	C	0.04744	9.343
17	rs5030739	12899902	A	0.007732	0.0008333	G	0.04744	9.343
17	rs45546232	26821090	A	0.007732	0.0008333	G	0.04744	9.343
17	rs142406693	58740530	A	0.007732	0.0008333	G	0.04744	9.343
17	rs199694023	61622579	G	0.007732	0.0008333	C	0.04744	9.343
17	rs199721470	73919553	A	0.007732	0.0008333	G	0.04744	9.343
17	rs116625217	80363209	A	0.007732	0.0008333	G	0.04744	9.343
18	rs115239581	8720430	G	0.007732	0.0008333	A	0.04744	9.343
19	rs142003967	19337790	A	0.007732	0.0008333	G	0.04744	9.343
19	rs201019662	39362441	C	0.007732	0.0008333	G	0.04744	9.343
19	rs199993010	50905089	G	0.007732	0.0008333	A	0.04744	9.343
19	rs140707289	58421111	A	0.007732	0.0008333	G	0.04744	9.343
20	rs236151	5903517	A	0.007732	0.0008333	G	0.04744	9.343
20	rs75576471	40161851	G	0.007732	0.0008333	C	0.04744	9.343
20	rs138747718	43851115	A	0.007732	0.0008333	G	0.04744	9.343
21	rs78000952	45502870	A	0.007732	0.0008333	G	0.04744	9.343
22	rs184593742	24095337	A	0.007732	0.0008333	G	0.04744	9.343
22	rs200350520	39497300	G	0.007732	0.0008333	A	0.04744	9.343
2	rs4672720	215502000	C	0.3402	0.3975	A	0.04746	0.7815
5	rs9324943	143570841	A	0.4278	0.37	G	0.04748	1.273
16	rs201699835	705658	A	0.007732	0.0008347	G	0.0476	9.327
11	rs7112184	84379740	A	0.3402	0.3967	G	0.04763	0.7843
4	rs11725207	161064449	A	0.1624	0.2092	G	0.04767	0.7329
12	rs483684	24504285	G	0.1624	0.2092	A	0.04767	0.7329
6	rs11155787	151686905	A	0.4304	0.3725	G	0.04775	1.273
15	rs2279683	34649211	A	0.2345	0.1867	C	0.0478	1.335
2	rs191692816	242811915	A	0.02577	0.01091	G	0.04782	2.399
6	rs3130688	31210216	G	0.2938	0.3492	A	0.04783	0.7755
11	rs498872	118477367	A	0.2036	0.2542	G	0.04783	0.7502
22	rs9614653	45767391	A	0.3789	0.3225	G	0.04783	1.281
6	rs6903792	133655858	A	0.433	0.375	G	0.04802	1.273
18	rs9051	2616457	C	0.1495	0.1108	G	0.04804	1.41
5	rs700241	39377132	A	0.04897	0.0275	G	0.04807	1.821
19	rs7255721	8669931	C	0.04897	0.0275	G	0.04807	1.821
3	rs111544243	190573340	G	0.01289	0.03167	C	0.04818	0.3992
7	rs35972858	150500534	A	0.01289	0.03167	G	0.04818	0.3992
9	rs2296871	135173685	A	0.3093	0.2567	G	0.04822	1.297

9	rs543573	135202829	G	0.3093	0.2567	A	0.04822	1.297
3	rs4955418	49200627	A	0.03866	0.06667	G	0.04823	0.563
11	rs11604149	6519642	A	0.2345	0.1875	G	0.04823	1.328
11	rs1695	67352689	G	0.2345	0.1875	A	0.04823	1.328
13	rs7327483	85722171	C	0.3402	0.2867	A	0.04825	1.283
3	rs13068038	49294250	A	0.03866	0.06583	C	0.04826	0.5706
3	rs13077498	49313978	A	0.03866	0.06583	G	0.04826	0.5706
6	rs2515920	31562615	T	0.03866	0.06583	A	0.04826	0.5706
7	rs33917957	116340262	G	0.03866	0.06583	A	0.04826	0.5706
16	rs117575136	67195842	G	0.03866	0.06583	A	0.04826	0.5706
9	rs10817858	100388119	A	0.183	0.2308	C	0.04831	0.7463
15	rs12907128	65621441	C	0.2139	0.1692	A	0.04841	1.337
12	rs1600	14656768	G	0.4356	0.3783	C	0.04843	1.268
14	rs7140949	32913332	A	0.3814	0.3258	G	0.04843	1.276
5	rs4913050	145282109	A	0.06186	0.095	C	0.0485	0.6281
6	rs61758102	30308326	G	0.01289	0.0325	A	0.04861	0.3886
6	rs3129853	32398648	A	0.2062	0.2558	G	0.04873	0.7555
6	rs3129872	32407153	A	0.2062	0.2558	T	0.04873	0.7555
11	rs12223986	64820695	C	0.1314	0.1742	A	0.04873	0.7176
8	rs922053	9250346	A	0.3428	0.2892	G	0.04876	1.282
13	rs12876018	96540204	C	0.3428	0.2892	A	0.04876	1.282
10	rs2590339	60012231	G	0.3531	0.41	A	0.04886	0.7854
6	rs2524044	31256753	C	0.2371	0.1892	A	0.04887	1.332
2	rs7577642	85699750	G	0.1985	0.1542	A	0.04893	1.358
14	rs2121070	76650764	A	0.1985	0.1542	G	0.04893	1.358
17	rs9895373	76121004	A	0.1134	0.07917	G	0.04901	1.488
7	rs4726024	151102867	G	0.3557	0.4133	A	0.04903	0.7835
6	rs2982694	152285687	C	0.3015	0.3575	A	0.04905	0.7759
6	rs2763979	31794592	A	0.3557	0.4125	G	0.04913	0.7862
5	rs258857	173034149	A	0.2668	0.3208	G	0.04935	0.7705
2	rs3213941	70402903	C	0.04639	0.07583	A	0.04936	0.5929
9	rs11103712	138037372	A	0.04639	0.07583	G	0.04936	0.5929
10	rs80323845	58118375	A	0.04639	0.07583	G	0.04936	0.5929
1	rs2186024	192430085	C	0.4433	0.3867	A	0.04941	1.263
19	rs7249858	23415601	G	0.2345	0.2867	A	0.04942	0.7624
2	rs1109758	29240783	G	0.3041	0.36	A	0.04945	0.777
5	rs141811421	79027830	A	0.03608	0.01833	G	0.04947	2.004
6	rs147016715	137482847	C	0.03608	0.01833	A	0.04947	2.004
19	rs148194580	44116662	A	0.03608	0.01833	G	0.04947	2.004
22	rs145373249	51176664	G	0.03608	0.01833	A	0.04947	2.004
6	rs3129858	32400520	A	0.2088	0.2583	G	0.04956	0.7575
14	rs11845134	22782377	A	0.1186	0.16	G	0.04956	0.7061
9	rs12238266	90503734	A	0.2655	0.3183	G	0.04965	0.7739

12	rs1522232	24394372	G	0.2603	0.2117	A	0.04969	1.311
3	rs1003995	187089031	A	0.3634	0.4208	C	0.04975	0.7856
16	rs79030430	17228363	A	0.04639	0.07667	G	0.04979	0.5859
6	rs3094009	31457178	A	0.4485	0.3917	G	0.04982	1.263
4	rs3733557	13601337	A	0.1057	0.1458	G	0.04983	0.6921
1	rs2055491	50852769	A	0.1057	0.145	G	0.04986	0.6967
6	rs682238	63160147	C	0.08247	0.1183	G	0.04989	0.6697
6	rs2941740	152009638	G	0.1521	0.1133	A	0.05004	1.403
3	rs34700313	23893940	A	0.4536	0.3967	G	0.05021	1.263
6	rs2071287	32170433	A	0.3093	0.365	G	0.05024	0.779
16	rs35195	65025718	A	0.1881	0.2358	G	0.05026	0.7509
21	rs425215	43707101	C	0.3505	0.2967	G	0.05026	1.279
21	rs2070426	47850484	G	0.3505	0.2967	C	0.05026	1.279
6	rs1204842	116520348	A	0.4562	0.3992	C	0.0504	1.263
11	rs2298566	130750642	A	0.317	0.265	C	0.05048	1.287
3	rs2567346	197195013	A	0.08247	0.05417	G	0.05061	1.57
20	rs4812831	43018260	A	0.3969	0.3408	G	0.05069	1.273
10	rs1045216	124189197	A	0.2191	0.1742	G	0.05075	1.33
9	rs9695734	96407983	A	0.04381	0.02333	G	0.05076	1.918
6	rs35937022	117114290	G	0.03351	0.06	A	0.0508	0.5431
6	rs28360548	117130544	C	0.03351	0.06	A	0.0508	0.5431
14	rs143920315	104208286	A	0.03351	0.06	C	0.0508	0.5431
17	rs714407	8860493	A	0.3763	0.4333	G	0.05087	0.7889
6	rs2224391	5260936	C	0.2397	0.2908	A	0.05093	0.7687
6	rs719654	32752139	A	0.2397	0.2908	G	0.05093	0.7687
6	rs130068	31116246	A	0.3969	0.3417	G	0.05095	1.268
3	rs9861636	159822667	C	0.1675	0.1275	A	0.05096	1.377
3	rs9825379	141137035	A	0.1701	0.2175	G	0.051	0.7374
12	rs4026608	66394664	A	0.1701	0.2175	G	0.051	0.7374
17	rs2293067	1550106	G	0.2732	0.3272	A	0.051	0.7729
8	rs16879552	32411216	G	0.3995	0.3433	A	0.05105	1.272
10	rs2388556	3191823	A	0.06443	0.04	G	0.0511	1.653
16	rs3812956	88501971	A	0.4665	0.4092	T	0.05111	1.263
6	rs3130055	31497399	G	0.1211	0.1625	A	0.05119	0.7104
6	rs1055388	31501737	G	0.1211	0.1625	A	0.05119	0.7104
20	rs6079391	14306953	A	0.09536	0.1333	C	0.05128	0.6852
6	rs1652507	161082461	G	0.384	0.4417	A	0.05137	0.7881
8	rs9792192	59985792	A	0.2758	0.33	G	0.05157	0.7731
4	rs10021082	100444178	A	0.4021	0.3467	G	0.05163	1.267
20	rs6126344	50407502	A	0.4794	0.4217	C	0.05164	1.263
3	rs646244	120551100	A	0.06443	0.09667	C	0.0517	0.6436
17	rs12603700	18167684	A	0.1856	0.1433	G	0.05174	1.362
12	rs4766310	5022041	G	0.4046	0.3483	A	0.05176	1.272

14	rs12434757	95884323	A	0.3892	0.4458	G	0.05188	0.7919
6	rs210180	33485259	T	0.1263	0.09167	A	0.05189	1.432
17	rs2229304	46629593	C	0.2216	0.1767	A	0.05192	1.327
14	rs1012023	33956209	A	0.4768	0.42	C	0.05198	1.259
17	rs12947658	65903006	G	0.1933	0.2417	A	0.05198	0.7519
3	rs2625954	129246414	A	0.3943	0.4517	G	0.05206	0.7904
10	rs7072268	71099913	G	0.2448	0.2975	A	0.05208	0.7656
12	rs60542959	56660905	A	0.01804	0.0384	C	0.05214	0.4601
7	rs11465293	75442723	A	0.01804	0.03917	G	0.05216	0.4507
6	rs2516472	31394700	G	0.01813	0.03917	A	0.05218	0.4531
6	rs2596460	31417510	G	0.01813	0.03917	A	0.05218	0.4531
6	rs2596480	31425985	A	0.01813	0.03917	G	0.05218	0.4531
11	rs11231181	62434173	A	0.0291	0.05394	G	0.05219	0.5257
2	rs1043160	220037756	G	0.3222	0.2708	A	0.05221	1.28
17	rs220470	3664975	G	0.1546	0.1995	A	0.05222	0.734
19	rs10401454	1009585	G	0.08505	0.1217	C	0.05222	0.6711
3	rs4241472	97983265	C	0.482	0.425	A	0.05223	1.259
6	exm-IND6-3121	31105858	D	0.01804	0.03833	I	0.05223	0.4609
12	rs2066808	56737973	G	0.01804	0.03833	A	0.05223	0.4609
12	rs2066807	56740682	C	0.01804	0.03833	G	0.05223	0.4609
1	rs1308139	151669442	G	0.4845	0.4275	A	0.05235	1.259
19	rs1127888	4454083	A	0.02835	0.0525	G	0.05237	0.5266
21	rs2835566	38427828	G	0.4046	0.35	A	0.05237	1.262
1	rs6699417	89123443	G	0.3969	0.4533	A	0.05244	0.7936
9	rs10739734	131527676	A	0.4871	0.43	C	0.05247	1.259
10	rs986178	124089075	A	0.1856	0.1442	G	0.05256	1.353
17	rs3889402	18167397	A	0.1856	0.1442	C	0.05256	1.353
6	rs373341	42658752	G	0.4098	0.3542	A	0.05262	1.266
6	rs6917450	158496400	G	0.2809	0.3342	A	0.05267	0.7784
11	rs11219726	124466751	G	0.1701	0.13	A	0.0527	1.372
5	rs25640	118811533	A	0.4948	0.4374	G	0.05273	1.26
3	rs17843776	124449406	G	0.01804	0.005833	A	0.05281	3.131
3	rs76736291	167196724	A	0.01804	0.005833	G	0.05281	3.131
4	rs141698377	155219852	T	0.01804	0.005833	A	0.05281	3.131
6	rs201087688	43418591	A	0.01804	0.005833	C	0.05281	3.131
8	rs20576	23058220	C	0.01804	0.005833	A	0.05281	3.131
9	rs142386294	94487020	A	0.01804	0.005833	G	0.05281	3.131
12	rs181241902	58203471	G	0.01804	0.005833	A	0.05281	3.131
16	rs2072086	19516398	A	0.01804	0.005833	G	0.05281	3.131
17	rs186264456	72584730	G	0.01804	0.005833	A	0.05281	3.131
19	rs201923258	50796922	A	0.01804	0.005833	G	0.05281	3.131
20	rs61760191	44444545	A	0.01804	0.005833	C	0.05281	3.131
2	rs10176705	50744774	A	0.09278	0.06333	G	0.05282	1.513

11	rs3758805	20486063	C	0.09278	0.06333	G	0.05282	1.513
11	rs2297781	32955122	G	0.09278	0.06333	A	0.05282	1.513
11	rs59345582	56309889	A	0.09278	0.06333	G	0.05282	1.513
3	rs2593943	108690221	A	0.04124	0.06917	G	0.05285	0.5788
11	rs12793222	554047	A	0.04124	0.06917	G	0.05285	0.5788
11	rs2847655	59865671	G	0.04124	0.06917	A	0.05285	0.5788
15	rs2271437	45556120	C	0.04124	0.06917	A	0.05285	0.5788
3	rs12107252	48691316	G	0.0567	0.0875	A	0.05292	0.6269
17	rs3744075	37074932	A	0.0567	0.0875	G	0.05292	0.6269
19	rs7248200	19744452	A	0.4923	0.4358	G	0.053	1.255
6	rs2078265	131761677	A	0.1546	0.1167	G	0.05307	1.385
4	rs2276961	10022981	A	0.5026	0.445	G	0.05309	1.26
16	rs12928822	11403893	A	0.0232	0.04583	G	0.05309	0.4944
16	rs35363016	76482001	G	0.0232	0.04583	A	0.05309	0.4944
2	rs35038757	219543924	A	0.01031	0.0275	G	0.05313	0.3684
14	rs75211061	77894724	A	0.01031	0.0275	G	0.05313	0.3684
9	rs7035276	17394536	G	0.1753	0.2217	A	0.05315	0.7461
14	rs2275592	50788213	G	0.4175	0.475	A	0.05317	0.7923
6	rs6605534	169722541	G	0.2706	0.2217	A	0.05322	1.303
19	rs7250872	1811603	A	0.4201	0.4775	G	0.05327	0.7927
6	rs9261440	30090820	G	0.272	0.2232	A	0.05332	1.3
6	rs805303	31616366	A	0.4227	0.48	G	0.05337	0.7932
6	rs3130981	31083813	A	0.09794	0.1358	G	0.05338	0.6907
6	rs3130558	31097183	G	0.09794	0.1367	C	0.05349	0.6859
1	rs2250377	201860626	A	0.1418	0.105	G	0.0535	1.408
17	rs4792311	12915009	A	0.05155	0.02917	G	0.05355	1.809
1	rs6588480	53978119	A	0.433	0.4908	G	0.05361	0.7922
11	rs79060400	56468694	G	0.1108	0.15	A	0.05362	0.7063
1	rs150748226	12921252	A	0.03093	0.015	G	0.05363	2.096
19	rs150447853	7083010	A	0.03093	0.015	G	0.05363	2.096
10	rs4554790	128463477	G	0.4304	0.4875	A	0.05364	0.7944
6	rs365488	29517958	A	0.518	0.4608	G	0.05365	1.258
6	rs362536	29526940	G	0.518	0.4608	A	0.05365	1.258
8	rs755683	18560687	A	0.5206	0.4633	G	0.05369	1.258
6	rs9394078	31868938	A	0.1057	0.07333	G	0.05371	1.493
9	rs7025117	98340320	A	0.4253	0.4817	C	0.05371	0.7962
5	rs59596499	150657130	A	0.04897	0.07917	C	0.05374	0.5989
8	rs2741098	6690276	A	0.4278	0.4842	G	0.05382	0.7967
1	rs2499604	238103501	A	0.5412	0.4833	G	0.05387	1.261
1	rs7526034	63586973	C	0.5155	0.4592	A	0.05392	1.253
7	rs36139924	299881	G	0.451	0.5083	A	0.05392	0.7947
2	rs7580515	157558583	A	0.4536	0.5108	G	0.05394	0.795
6	rs2745411	29518442	A	0.4407	0.4975	G	0.05394	0.7959

16	rs2667662	1551082	A	0.5335	0.4766	G	0.05396	1.256
14	rs8007661	92459958	A	0.4562	0.5133	G	0.05397	0.7953
14	rs28564871	51368610	G	0.5232	0.4667	A	0.05399	1.254
2	rs12623234	105819545	G	0.5335	0.4767	A	0.05401	1.256
3	rs11707692	88518442	C	0.5284	0.4717	A	0.05401	1.255
12	rs12227355	93011711	G	0.4433	0.5	A	0.05401	0.7963
2	rs6743376	113832333	C	0.2526	0.3042	A	0.05402	0.7731
1	rs7530465	9068386	A	0.1881	0.1467	G	0.05408	1.348
6	rs6907558	41467860	C	0.3376	0.3933	A	0.05413	0.7862
4	rs962004	74285239	G	0.451	0.5075	A	0.05417	0.7973
7	rs36170987	299850	A	0.451	0.5075	C	0.05417	0.7973
16	rs6563943	83639335	A	0.4227	0.3667	G	0.05418	1.265
17	rs17822735	56620167	A	0.1778	0.225	G	0.05418	0.745
19	rs10084151	57182204	G	0.3711	0.3175	A	0.05437	1.269
2	rs3815875	160710153	C	0.1418	0.1858	A	0.05447	0.7236
6	rs11908	32944746	A	0.1418	0.1858	G	0.05447	0.7236
6	rs206781	32946133	G	0.1418	0.1858	A	0.05447	0.7236
6	rs188245	32955976	G	0.1418	0.1858	A	0.05447	0.7236
6	rs172276	32959770	T	0.1418	0.1858	A	0.05447	0.7236
8	rs2242241	21766881	C	0.04381	0.02417	A	0.05462	1.85
14	rs117362093	61746823	A	0.04381	0.02417	T	0.05462	1.85
16	rs188159138	67862706	G	0.04381	0.02417	C	0.05462	1.85
16	rs2303772	87795580	G	0.04381	0.02417	C	0.05462	1.85
1	rs79791475	180905294	A	0.116	0.08333	G	0.05463	1.443
6	rs2235501	24489691	G	0.116	0.08333	C	0.05463	1.443
3	rs13324142	48669447	A	0.06701	0.1	G	0.05464	0.6464
2	rs10186193	3597974	G	0.3351	0.2825	A	0.05472	1.28
18	rs9949802	45614308	A	0.3351	0.2825	G	0.05472	1.28
6	rs7743761	31336100	A	0.4278	0.3717	C	0.05477	1.264
1	rs12757165	216716537	G	0.3737	0.32	A	0.05481	1.268
6	rs241429	32803840	A	0.4278	0.3725	G	0.05499	1.26
1	rs2294228	26140573	C	0.08505	0.05667	A	0.05502	1.547
6	rs2294763	97512577	G	0.08505	0.05667	A	0.05502	1.547
17	rs8080784	59017025	G	0.1572	0.1192	A	0.05503	1.379
6	rs1448063	127929801	A	0.2577	0.31	G	0.05519	0.7728
2	rs13015447	167377978	A	0.3763	0.3225	C	0.05525	1.267
13	rs9507041	23744379	A	0.1572	0.1194	G	0.0553	1.376
2	rs11164117	95680267	A	0.3376	0.285	G	0.05531	1.279
8	rs4994	37823798	G	0.1134	0.1533	A	0.05533	0.7063
11	rs4633461	7712523	A	0.1134	0.1533	G	0.05533	0.7063
11	rs4519083	7712561	A	0.1134	0.1533	T	0.05533	0.7063
11	rs10893336	125023928	C	0.433	0.3767	A	0.05535	1.264
9	rs3748176	117124731	A	0.433	0.3773	G	0.05542	1.26

1	rs7551175	12009956	A	0.2526	0.205	G	0.05545	1.311
7	rs2892855	17644213	G	0.2964	0.3508	A	0.05547	0.7795
6	rs2273063	39507889	A	0.2294	0.2802	G	0.05548	0.7646
22	rs2106294	31645759	G	0.1134	0.1525	A	0.0555	0.7108
1	rs142698557	57224387	T	0.02062	0.008333	A	0.05567	2.505
3	rs115716405	194080125	A	0.02062	0.008333	G	0.05567	2.505
6	rs149411113	27834790	A	0.02062	0.008333	C	0.05567	2.505
7	rs138429176	88964280	C	0.02062	0.008333	A	0.05567	2.505
8	rs199991427	145617023	G	0.02062	0.008333	A	0.05567	2.505
10	rs145558318	103734984	A	0.02062	0.008333	G	0.05567	2.505
13	rs200679039	25671820	A	0.02062	0.008333	G	0.05567	2.505
18	rs61740078	61387277	A	0.02062	0.008333	G	0.05567	2.505
2	rs17035378	68598955	A	0.3763	0.3233	G	0.0557	1.263
6	rs675531	128040839	G	0.1443	0.1075	A	0.05575	1.4
4	rs2292899	185678110	A	0.4356	0.38	G	0.05578	1.259
10	rs2296441	100144782	A	0.4356	0.38	G	0.05578	1.259
20	rs4809401	62737568	G	0.1314	0.09583	A	0.05582	1.428
6	rs10947091	30747216	G	0.3531	0.4092	A	0.05591	0.7882
2	rs11553746	272203	A	0.1624	0.2075	G	0.05595	0.7404
2	rs79716074	277003	G	0.1624	0.2075	A	0.05595	0.7404
10	rs41301609	45496126	A	0.1186	0.085	G	0.05596	1.448
1	rs910696	30567596	G	0.3814	0.3275	A	0.0561	1.266
6	rs6569992	135452152	A	0.3814	0.3275	G	0.0561	1.266
12	rs2293624	104345384	A	0.1624	0.2067	G	0.05611	0.7441
15	rs1107785	62106862	G	0.1624	0.2067	A	0.05611	0.7441
8	rs11136478	2143418	G	0.4356	0.3808	A	0.05614	1.255
4	rs10008153	185227184	A	0.3557	0.4117	G	0.05618	0.7889
8	rs12549368	98451430	A	0.3557	0.4117	G	0.05618	0.7889
13	rs3813133	115090193	A	0.1753	0.135	G	0.05619	1.362
20	rs3746609	31022469	A	0.1753	0.135	G	0.05619	1.362
13	rs9532117	38363047	A	0.3402	0.2883	G	0.05623	1.273
9	rs16918128	101767303	A	0.03608	0.06281	G	0.05624	0.5585
5	rs10036727	168180081	G	0.3557	0.4108	A	0.05628	0.7916
6	rs3130453	31124849	A	0.4407	0.385	G	0.05629	1.259
4	rs192912733	47939240	A	0.02577	0.01167	G	0.05631	2.241
6	rs113993403	30114897	A	0.02577	0.01167	G	0.05631	2.241
6	rs61735038	30126177	A	0.02577	0.01167	G	0.05631	2.241
6	rs61737424	30126285	A	0.02577	0.01167	G	0.05631	2.241
6	rs61737427	30126303	A	0.02577	0.01167	T	0.05631	2.241
6	rs726599	33121678	G	0.02577	0.01167	A	0.05631	2.241
6	rs2257126	33131734	A	0.02577	0.01167	G	0.05631	2.241
22	rs2076674	41170063	A	0.3015	0.3558	G	0.05636	0.7816
6	rs2894196	31230111	T	0.1933	0.1508	A	0.0564	1.349

9	rs2075662	101748234	A	0.3428	0.29	G	0.05647	1.277
15	rs112100339	98504322	A	0.05928	0.09083	G	0.05648	0.6307
6	rs1264429	30565101	G	0.05928	0.09	A	0.05651	0.6371
4	rs28605080	689743	A	0.4407	0.3858	G	0.05661	1.254
5	rs1805377	82648943	G	0.3093	0.2588	A	0.05669	1.283
9	rs1056899	135139901	A	0.2809	0.2317	G	0.05669	1.296
5	rs16875122	5242218	A	0.2552	0.2083	C	0.05694	1.302
6	rs2743868	15625808	A	0.3093	0.2592	G	0.05696	1.28
6	rs2254847	30933848	A	0.2113	0.1683	T	0.05705	1.324
20	rs2752930	26151507	C	0.3634	0.4183	A	0.05713	0.7937
13	rs9524891	95942561	G	0.2809	0.2325	A	0.05714	1.29
18	rs17083844	68842243	A	0.07732	0.05	G	0.05715	1.592
1	rs1332500	152692074	C	0.1469	0.1908	G	0.05723	0.7302
1	rs873775	152692472	C	0.1469	0.1908	A	0.05723	0.7302
3	rs13094203	112993367	A	0.1753	0.1358	G	0.05723	1.352
6	rs2394427	31019562	A	0.1753	0.1358	G	0.05723	1.352
2	rs16846646	163215423	A	0.09021	0.1275	C	0.05725	0.6785
2	exm-IND2-16292	163217274	D	0.09021	0.1275	I	0.05725	0.6785
4	rs902174	185655171	G	0.3866	0.3333	A	0.0573	1.261
10	rs754720	107026567	G	0.3454	0.2933	A	0.05733	1.271
2	rs2540226	39959060	A	0.232	0.1875	C	0.05735	1.309
10	rs1819658	59913151	A	0.366	0.4208	G	0.05741	0.7944
9	rs1128116	115933980	A	0.007772	0.02345	C	0.05743	0.3262
10	rs149343572	13224970	A	0.007732	0.02333	G	0.05743	0.3262
11	rs78608838	17390443	A	0.007732	0.02333	C	0.05743	0.3262
3	rs9836841	174814920	G	0.3686	0.4242	A	0.05748	0.7924
9	rs10821119	96145271	A	0.1186	0.08583	C	0.0575	1.433
17	rs2158917	69926109	A	0.05155	0.08083	G	0.0575	0.618
19	rs75802152	36259116	A	0.1186	0.08583	G	0.0575	1.433
1	rs3738766	110151395	A	0.2139	0.17	C	0.05762	1.329
5	rs2962330	10481725	G	0.2139	0.17	A	0.05762	1.329
6	rs6911639	32978178	G	0.2139	0.17	A	0.05762	1.329
3	rs9823911	174814738	A	0.3686	0.4233	G	0.05768	0.7951
17	rs2680708	56456120	A	0.3686	0.4233	G	0.05768	0.7951
3	rs11711450	178207097	A	0.4536	0.3983	G	0.05769	1.254
19	rs1811	35434238	A	0.4562	0.4	G	0.0577	1.258
3	rs17057718	57136585	A	0.3711	0.4267	G	0.05772	0.793
4	rs201262483	1017712	A	0.0117	0.03199	G	0.05777	0.3581
19	rs34163641	44118191	A	0.1623	0.1242	G	0.05784	1.367
10	rs947474	6390450	G	0.0799	0.1142	A	0.05786	0.6738
8	rs12541651	37730368	G	0.2577	0.2108	A	0.05793	1.3
10	rs12780223	12689914	G	0.3711	0.4258	A	0.05795	0.7957
10	rs2227310	115489152	G	0.3737	0.4292	C	0.05796	0.7937

14	rs3742591	45433155	G	0.1624	0.1233	C	0.05806	1.378
6	rs2523882	31042217	A	0.4588	0.4033	G	0.05809	1.254
1	rs56174824	228564884	A	0.3943	0.34	G	0.05816	1.264
16	rs4782505	88656638	C	0.3763	0.4317	A	0.0582	0.7943
1	rs189256251	26609233	A	0.002577	0.015	G	0.05827	0.1697
1	rs1541160	169995802	G	0.002577	0.015	A	0.05827	0.1697
10	rs12217419	31784716	A	0.002577	0.015	G	0.05827	0.1697
12	rs41263847	48377898	A	0.002577	0.015	G	0.05827	0.1697
19	rs395136	33106742	A	0.4843	0.4275	G	0.05827	1.258
4	rs3884638	29998461	C	0.3789	0.435	A	0.05835	0.7922
8	rs4873779	49031831	G	0.2139	0.1708	A	0.05835	1.321
11	rs6485702	46898771	G	0.3479	0.2967	A	0.05838	1.265
1	rs1339988	248511674	A	0.3789	0.4341	G	0.05841	0.7953
13	rs4996815	106651661	A	0.1082	0.07667	C	0.05841	1.462
1	rs28377739	248512749	A	0.3789	0.4342	G	0.05843	0.7949
1	rs28545014	248512767	A	0.3789	0.4342	C	0.05843	0.7949
11	rs4237768	6007272	A	0.134	0.09833	G	0.05843	1.419
11	rs4354673	6007613	A	0.134	0.09833	C	0.05843	1.419
1	rs10218696	172823534	G	0.1167	0.08263	A	0.05859	1.466
2	rs12463674	179432185	G	0.007732	0.02417	A	0.05864	0.3146
5	rs2033195	153509596	A	0.007732	0.02417	G	0.05864	0.3146
9	rs146401995	95064042	G	0.007732	0.02417	A	0.05864	0.3146
7	rs12718598	50428445	G	0.3814	0.4367	A	0.05866	0.7955
9	rs16912096	125437725	G	0.09794	0.0675	A	0.0587	1.5
9	rs16912099	125437769	G	0.09794	0.0675	A	0.0587	1.5
9	rs74634130	125438244	G	0.09794	0.0675	A	0.0587	1.5
9	rs79636164	125438378	A	0.09794	0.0675	G	0.0587	1.5
11	rs150905417	67926218	A	0.007732	0.02425	G	0.05875	0.3136
1	rs12094234	54439366	G	0.03093	0.055	A	0.0588	0.5484
18	rs61749943	21529730	A	0.03093	0.055	G	0.0588	0.5484
16	rs4788587	72001136	A	0.3969	0.3433	G	0.05883	1.259
20	rs6034239	1616137	G	0.3969	0.3433	A	0.05883	1.259
12	rs4964469	106949987	A	0.384	0.4392	G	0.05888	0.7961
16	rs3743771	53358439	G	0.2603	0.2133	A	0.05891	1.298
19	rs7252175	46133256	A	0.1211	0.0875	G	0.05891	1.437
14	rs1885097	23549319	G	0.317	0.3708	A	0.05893	0.7875
12	rs1127787	27867727	A	0.1186	0.1583	G	0.059	0.715
1	rs77282993	179363143	A	0.07732	0.05083	C	0.05908	1.565
6	rs1801582	161807855	G	0.03093	0.05583	C	0.0591	0.5397
19	rs190517297	10946843	A	0.005181	0	G	0.05912	NA
2	rs4663607	236450763	A	0.4691	0.4142	G	0.05913	1.25
1	rs11805303	67675516	G	0.4716	0.4167	A	0.05929	1.25
1	rs116854758	150257054	A	0.01546	0.03417	G	0.05931	0.444

6	rs3129157	29141743	A	0.01546	0.03417	G	0.05931	0.444
7	rs10264013	10852392	A	0.3918	0.4475	G	0.05933	0.7952
4	rs1365162	75993726	G	0.2423	0.2925	A	0.05935	0.7734
17	rs147160209	80417913	A	0.01546	0.035	G	0.05937	0.4331
17	rs9303575	49217617	C	0.4742	0.4192	A	0.05944	1.25
6	rs6923504	32428186	G	0.3557	0.3033	C	0.05949	1.268
7	rs860170	122635024	G	0.3943	0.45	A	0.05951	0.7957
8	rs1735192	146045868	A	0.4794	0.4233	G	0.05951	1.254
2	rs7567851	178684720	G	0.134	0.09917	C	0.05952	1.406
2	rs13011089	123828854	A	0.09278	0.13	C	0.05956	0.6844
1	rs144710974	909320	A	0.005155	0	G	0.05958	NA
1	rs201464887	6007194	A	0.005155	0	C	0.05958	NA
1	rs200259274	8075618	G	0.005155	0	A	0.05958	NA
1	rs146738397	24932231	A	0.005155	0	G	0.05958	NA
1	rs200429239	32674915	A	0.005155	0	C	0.05958	NA
1	rs17399810	55119534	A	0.005155	0	G	0.05958	NA
1	rs570218	55139741	A	0.005155	0	G	0.05958	NA
1	rs199845652	55183300	G	0.005155	0	A	0.05958	NA
1	rs201432239	144874752	A	0.005155	0	G	0.05958	NA
1	rs140914817	152329951	A	0.005155	0	G	0.05958	NA
1	rs138411937	159850438	A	0.005155	0	G	0.05958	NA
1	rs139875266	160249363	G	0.005155	0	C	0.05958	NA
1	rs61741479	173915909	G	0.005155	0	C	0.05958	NA
1	rs200640492	222801868	A	0.005155	0	G	0.05958	NA
1	rs147360179	230883297	A	0.005155	0	G	0.05958	NA
1	rs2067481	240070944	A	0.005155	0	G	0.05958	NA
2	rs201073324	17699240	A	0.005155	0	G	0.05958	NA
2	rs142380036	97007457	A	0.005155	0	G	0.05958	NA
2	rs142702703	98351118	G	0.005155	0	A	0.05958	NA
2	rs191576855	103125391	A	0.005155	0	G	0.05958	NA
2	rs75717073	111556617	A	0.005155	0	G	0.05958	NA
2	rs151092449	128408959	A	0.005155	0	G	0.05958	NA
2	rs112581563	145156613	A	0.005155	0	G	0.05958	NA
2	rs201334086	149247495	A	0.005155	0	G	0.05958	NA
2	rs148507801	182409488	A	0.005155	0	G	0.05958	NA
2	rs10185378	206305156	A	0.005155	0	G	0.05958	NA
2	rs192284534	238738099	A	0.005155	0	G	0.05958	NA
3	rs200980879	38038990	G	0.005155	0	A	0.05958	NA
3	rs148183642	39166625	A	0.005155	0	G	0.05958	NA
3	rs77079961	44856499	G	0.005155	0	A	0.05958	NA
3	rs114793043	44867592	G	0.005155	0	C	0.05958	NA
3	rs80007780	44882586	C	0.005155	0	G	0.05958	NA
3	rs17076986	44893288	C	0.005155	0	A	0.05958	NA

3	rs201722129	140997252	A	0.005155	0	G	0.05958	NA
3	rs150165951	195474176	A	0.005155	0	G	0.05958	NA
3	rs34326043	196613101	A	0.005155	0	G	0.05958	NA
4	rs184864770	13602871	G	0.005155	0	A	0.05958	NA
4	rs200345295	76481300	A	0.005155	0	G	0.05958	NA
4	rs200175441	103553318	C	0.005155	0	A	0.05958	NA
5	rs116836100	162040	A	0.005155	0	G	0.05958	NA
5	rs34586473	5303424	A	0.005155	0	G	0.05958	NA
5	rs151157740	72874636	G	0.005155	0	A	0.05958	NA
5	rs61733068	147929809	A	0.005155	0	G	0.05958	NA
5	rs200471156	175926031	A	0.005155	0	G	0.05958	NA
5	rs201799075	177658496	G	0.005155	0	C	0.05958	NA
5	rs201173983	177683382	A	0.005155	0	G	0.05958	NA
6	rs201434867	304646	G	0.005155	0	A	0.05958	NA
6	rs140556836	15504769	A	0.005155	0	G	0.05958	NA
6	rs1047014	19841493	G	0.005155	0	A	0.05958	NA
6	rs362527	29531818	G	0.005155	0	A	0.05958	NA
6	rs2523447	29532739	A	0.005155	0	G	0.05958	NA
6	rs1805056	29600104	A	0.005155	0	G	0.05958	NA
6	rs141551053	35428345	A	0.005155	0	G	0.05958	NA
6	rs147529495	51889764	A	0.005155	0	G	0.05958	NA
6	rs139975520	143828518	G	0.005155	0	A	0.05958	NA
6	rs146845697	168465737	A	0.005155	0	G	0.05958	NA
7	rs202078752	23293854	A	0.005155	0	G	0.05958	NA
7	rs201260783	99261643	A	0.005155	0	T	0.05958	NA
7	rs56006153	143095979	A	0.005155	0	G	0.05958	NA
8	rs332034	8717007	G	0.005155	0	A	0.05958	NA
8	rs111646478	10465962	A	0.005155	0	C	0.05958	NA
8	rs138069770	23054713	A	0.005155	0	G	0.05958	NA
8	rs7814768	24211331	G	0.005155	0	A	0.05958	NA
8	rs200747493	27145622	A	0.005155	0	C	0.05958	NA
8	rs144877578	59544086	G	0.005155	0	A	0.05958	NA
8	rs3907424	81733727	A	0.005155	0	C	0.05958	NA
8	rs200411401	145001032	A	0.005155	0	G	0.05958	NA
9	rs148994877	328163	A	0.005155	0	G	0.05958	NA
9	rs114440343	712681	A	0.005155	0	G	0.05958	NA
9	rs140841790	95400541	C	0.005155	0	A	0.05958	NA
9	rs200570034	123898249	G	0.005155	0	A	0.05958	NA
9	rs908832	139912484	A	0.005155	0	G	0.05958	NA
10	rs199540152	1279752	G	0.005155	0	A	0.05958	NA
10	rs139517333	28225787	A	0.005155	0	G	0.05958	NA
10	rs116628504	50870806	A	0.005155	0	G	0.05958	NA
10	rs201297042	73437293	A	0.005155	0	G	0.05958	NA

10	rs188270898	129183096	A	0.005155	0	G	0.05958	NA
11	rs140700057	6458344	A	0.005155	0	G	0.05958	NA
11	rs150166337	20124873	A	0.005155	0	G	0.05958	NA
11	rs3729799	47355294	A	0.005155	0	G	0.05958	NA
11	rs116848448	57466436	A	0.005155	0	G	0.05958	NA
11	rs79964989	59807978	A	0.005155	0	G	0.05958	NA
11	rs199945827	64880767	A	0.005155	0	G	0.05958	NA
11	rs201845370	70033955	A	0.005155	0	C	0.05958	NA
11	rs201150384	71146443	A	0.005155	0	G	0.05958	NA
11	rs17848367	73717396	A	0.005155	0	G	0.05958	NA
11	exm964668	122669682	A	0.005155	0	G	0.05958	NA
11	rs77812199	123676366	A	0.005155	0	G	0.05958	NA
11	rs150587299	125867185	A	0.005155	0	G	0.05958	NA
11	rs75183025	126142952	A	0.005155	0	G	0.05958	NA
12	rs150922607	56626574	A	0.005155	0	G	0.05958	NA
12	rs139176331	57849921	G	0.005155	0	A	0.05958	NA
12	rs184795006	89917437	A	0.005155	0	G	0.05958	NA
12	rs183923487	124323060	A	0.005155	0	G	0.05958	NA
13	rs138379662	26788304	A	0.005155	0	G	0.05958	NA
14	rs201871015	23530293	A	0.005155	0	G	0.05958	NA
14	rs61741619	23548787	A	0.005155	0	G	0.05958	NA
14	rs80007670	23548793	A	0.005155	0	G	0.05958	NA
14	rs4280164	24771285	A	0.005155	0	G	0.05958	NA
14	rs36004050	63175105	A	0.005155	0	G	0.05958	NA
14	rs28931570	94849388	A	0.005155	0	G	0.05958	NA
15	rs181976061	31339385	A	0.005155	0	G	0.05958	NA
15	rs199850508	44085229	A	0.005155	0	G	0.05958	NA
15	rs200324727	58834122	A	0.005155	0	G	0.05958	NA
15	rs140370405	66220772	A	0.005155	0	G	0.05958	NA
15	rs200437386	68607999	A	0.005155	0	G	0.05958	NA
15	rs185781006	72030142	A	0.005155	0	G	0.05958	NA
15	rs145238866	85341990	A	0.005155	0	G	0.05958	NA
16	rs146373106	113092	A	0.005155	0	G	0.05958	NA
16	rs200113481	240081	A	0.005155	0	G	0.05958	NA
16	rs148475508	682673	A	0.005155	0	G	0.05958	NA
16	rs4984636	1252441	G	0.005155	0	A	0.05958	NA
16	rs8063574	1260481	A	0.005155	0	G	0.05958	NA
16	rs11248860	1272038	G	0.005155	0	A	0.05958	NA
16	rs201327024	1592036	A	0.005155	0	G	0.05958	NA
16	rs148373909	3707257	A	0.005155	0	G	0.05958	NA
16	rs1062706	4487486	A	0.005155	0	G	0.05958	NA
16	rs148868599	10551286	G	0.005155	0	A	0.05958	NA
16	rs192701366	15703380	G	0.005155	0	A	0.05958	NA

16	rs55772253	20352618	A	0.005155	0	C	0.05958	NA
16	rs142000603	20856541	A	0.005155	0	C	0.05958	NA
16	rs79515799	23503040	A	0.005155	0	G	0.05958	NA
16	rs142167857	77327136	A	0.005155	0	G	0.05958	NA
16	rs147451671	85695077	A	0.005155	0	C	0.05958	NA
16	rs56236932	88504204	C	0.005155	0	G	0.05958	NA
16	rs34808360	88675355	A	0.005155	0	G	0.05958	NA
17	rs138543123	3912208	A	0.005155	0	G	0.05958	NA
17	rs200715746	33690693	A	0.005155	0	G	0.05958	NA
17	rs180782754	36002150	A	0.005155	0	G	0.05958	NA
17	rs201729934	39156104	G	0.005155	0	A	0.05958	NA
17	rs9902629	42395568	A	0.005155	0	G	0.05958	NA
17	rs140314190	46136735	A	0.005155	0	G	0.05958	NA
17	rs2304942	55950195	A	0.005155	0	G	0.05958	NA
17	rs150939699	64171222	A	0.005155	0	G	0.05958	NA
17	rs191683466	80680760	A	0.005155	0	C	0.05958	NA
18	rs121918095	29178565	A	0.005155	0	G	0.05958	NA
19	rs148975956	11559428	A	0.005155	0	G	0.05958	NA
19	rs150514783	30164820	A	0.005155	0	G	0.05958	NA
19	rs45532538	46973026	A	0.005155	0	C	0.05958	NA
19	rs202104197	49261455	A	0.005155	0	G	0.05958	NA
19	rs201874615	56736256	G	0.005155	0	A	0.05958	NA
20	rs147232069	1918086	A	0.005155	0	G	0.05958	NA
20	rs146938047	3682094	A	0.005155	0	G	0.05958	NA
20	rs76933768	6064778	A	0.005155	0	G	0.05958	NA
20	rs114396838	20269334	A	0.005155	0	G	0.05958	NA
20	rs201313323	25059497	A	0.005155	0	C	0.05958	NA
20	rs144469725	36938936	G	0.005155	0	A	0.05958	NA
20	rs200604036	60772998	A	0.005155	0	G	0.05958	NA
21	rs200844181	46032204	C	0.005155	0	G	0.05958	NA
22	rs144341537	42564717	C	0.005155	0	G	0.05958	NA
22	rs143916680	44564483	A	0.005155	0	G	0.05958	NA
6	rs929156	30139699	A	0.2603	0.2142	G	0.05962	1.291
6	rs2894181	31174527	G	0.482	0.4258	A	0.05967	1.254
12	rs4236	15035081	G	0.1057	0.1433	A	0.05972	0.7062
7	rs200864007	128484183	A	0.005155	0	C	0.05973	NA
11	rs75988367	381754	A	0.005155	0	G	0.05973	NA
12	rs181999391	9307387	G	0.005155	0	C	0.05973	NA
19	rs192510221	4429603	A	0.005155	0	G	0.05973	NA
5	rs153477	150639409	A	0.2784	0.3308	G	0.05986	0.7802
1	rs6670984	145561594	G	0.2629	0.2158	A	0.05989	1.296
6	rs2857602	31533378	G	0.2784	0.33	A	0.05994	0.7831
5	rs142663787	68411954	A	0.005155	0.01917	G	0.05996	0.2651

10	rs143713985	25861636	A	0.005155	0.01917	G	0.05996	0.2651
11	rs55741856	1277953	A	0.005155	0.01917	G	0.05996	0.2651
14	rs181990876	105420440	A	0.005155	0.01917	G	0.05996	0.2651
1	rs3818562	110300441	G	0.2165	0.2654	A	0.05999	0.7646
19	rs1800437	46181392	G	0.1521	0.195	C	0.06001	0.7403
12	rs1063193	10999780	G	0.2165	0.265	A	0.06004	0.7664
20	rs6025606	56098733	A	0.2165	0.265	G	0.06004	0.7664
8	rs7008165	54431866	C	0.2448	0.295	A	0.06007	0.7749
2	rs1108460	38350007	G	0.06186	0.0925	A	0.06011	0.6469
6	rs2239804	32411523	A	0.4046	0.4608	G	0.06011	0.7952
19	rs7258236	6760974	G	0.06186	0.0925	A	0.06011	0.6469
4	rs28823933	75052592	A	0.1985	0.1567	G	0.06013	1.333
9	rs4978343	110820132	C	0.4897	0.4342	A	0.06023	1.251
8	rs1057091	6500544	A	0.3196	0.27	G	0.06024	1.27
8	rs2722425	40484239	A	0.3196	0.27	G	0.06024	1.27
1	rs17117430	58855299	A	0.4046	0.3517	G	0.06035	1.253
19	rs12151009	46141845	G	0.1211	0.08833	A	0.06039	1.423
3	rs6549236	69592507	A	0.2809	0.3333	G	0.06041	0.7814
3	rs9310736	24350811	A	0.1933	0.24	G	0.06046	0.7588
3	rs2535629	52833219	A	0.4871	0.4325	G	0.06047	1.246
2	rs6737791	242918203	G	0.4974	0.4416	A	0.0605	1.252
1	rs199613	30133502	G	0.3273	0.3808	A	0.06055	0.7911
4	rs324726	76722353	G	0.1108	0.07833	A	0.06055	1.466
10	rs10776612	49735563	A	0.3273	0.3808	G	0.06055	0.7911
7	rs2293583	88423881	G	0.07216	0.105	A	0.06057	0.6627
7	rs9942677	89387363	A	0.4974	0.4417	G	0.06057	1.251
7	rs36104932	16666725	A	0.08247	0.1167	G	0.0606	0.6806
12	rs1464500	24389660	C	0.02577	0.04833	A	0.06066	0.5209
17	rs2521988	7155861	A	0.02577	0.04833	G	0.06066	0.5209
2	rs12615112	107450525	G	0.4175	0.4733	A	0.06075	0.7976
16	rs1424104	79146925	G	0.4948	0.44	A	0.06075	1.247
22	rs9622677	38054262	A	0.5026	0.4467	C	0.06077	1.252
5	rs12188040	157073747	A	0.07216	0.105	C	0.06083	0.663
8	rs12674488	6338306	A	0.1366	0.1783	C	0.06083	0.7289
2	rs1127102	220037666	G	0.3222	0.2725	A	0.06088	1.269
5	rs1031844	134782576	A	0.5026	0.4474	C	0.06092	1.248
15	rs906974	99764859	G	0.5	0.445	A	0.06092	1.247
1	rs2367042	63493164	C	0.2191	0.2683	A	0.06095	0.7649
14	rs7161563	50799126	G	0.4175	0.4725	C	0.06095	0.8003
5	rs12520809	134782510	A	0.5026	0.4475	G	0.06099	1.247
9	rs1185193	135203409	C	0.3247	0.2742	A	0.06102	1.273
10	rs7071768	129903016	G	0.5103	0.4548	A	0.06106	1.249
12	rs1484087	27166575	A	0.5052	0.45	G	0.06107	1.248

6	rs7029	31629953	G	0.4253	0.4808	A	0.06108	0.7989
6	rs7992	31630241	A	0.4253	0.4808	G	0.06108	0.7989
6	rs4569	31637807	A	0.4253	0.4808	G	0.06108	0.7989
1	rs2744835	11395416	A	0.02062	0.04167	G	0.06115	0.4842
11	rs55765473	65055190	G	0.02062	0.04167	A	0.06115	0.4842
9	rs10818504	123860689	G	0.4278	0.4833	A	0.06118	0.7993
1	rs5745325	76269460	A	0.04639	0.02583	G	0.06119	1.835
8	rs16888728	117783975	A	0.04639	0.02583	G	0.06119	1.835
20	rs144253683	61442929	G	0.04639	0.02583	A	0.06119	1.835
1	rs2802796	203440733	C	0.4304	0.4858	A	0.06127	0.7997
1	rs10783050	97037083	A	0.4356	0.4917	G	0.06132	0.7978
5	rs325864	41049397	G	0.4149	0.3608	A	0.06132	1.256
7	rs16874525	28391182	A	0.4356	0.4917	G	0.06132	0.7978
14	rs730532	52518811	G	0.4381	0.4942	A	0.06138	0.7982
6	rs130067	31118511	C	0.3634	0.3117	A	0.06142	1.261
19	rs2241524	55098667	A	0.3634	0.3117	G	0.06142	1.261
1	rs10798302	173987798	A	0.4278	0.4825	G	0.06146	0.802
14	rs17308108	20482919	C	0.1495	0.1133	A	0.06148	1.375
16	rs6498142	11081249	G	0.1495	0.1133	C	0.06148	1.375
2	rs2536217	229977915	G	0.5284	0.4725	A	0.06149	1.251
14	rs12437434	24878370	A	0.2861	0.3383	G	0.06149	0.7837
17	rs13412	39967442	A	0.4381	0.4933	G	0.06153	0.8009
9	rs10738554	19904103	A	0.5335	0.4775	G	0.06154	1.251
1	rs4420115	198365219	A	0.4485	0.5042	G	0.06157	0.7996
2	rs16839962	156925286	T	0.4304	0.485	A	0.06158	0.8024
7	rs79254850	130827699	A	0.0232	0.009167	G	0.06158	2.567
19	rs150885598	50549441	A	0.0232	0.009167	G	0.06158	2.567
1	rs12409352	160000435	T	0.03866	0.065	A	0.06159	0.5785
6	rs3093983	31496925	G	0.03866	0.065	A	0.06159	0.5785
6	rs3115537	31497835	G	0.03866	0.065	C	0.06159	0.5785
6	rs3093978	31498497	C	0.03866	0.065	A	0.06159	0.5785
6	rs3131628	31502767	G	0.03866	0.065	A	0.06159	0.5785
6	rs1129640	31506624	A	0.03866	0.065	G	0.06159	0.5785
4	rs60716049	53757674	A	0.451	0.5067	G	0.0616	0.8
14	rs8019794	26840301	A	0.451	0.5067	G	0.0616	0.8
14	rs4904333	87178343	G	0.4407	0.4958	A	0.06161	0.8013
20	rs6032606	44596207	C	0.4407	0.4958	G	0.06161	0.8013
11	rs11555762	43876698	A	0.2216	0.1775	G	0.06163	1.32
3	rs78962087	70014199	A	0.03866	0.02083	C	0.06166	1.89
12	rs10842975	27915718	A	0.5412	0.4858	T	0.06168	1.249
13	rs4942169	43456753	A	0.4433	0.4983	C	0.06168	0.8016
3	rs16840208	98518377	A	0.03866	0.06417	G	0.0617	0.5865
18	rs2000900	47502338	A	0.03866	0.06417	C	0.0617	0.5865

15	rs2242046	85478729	A	0.08763	0.06	G	0.06171	1.505
1	rs35675666	8021973	A	0.05412	0.08417	C	0.06174	0.6226
5	rs10078391	13811775	G	0.4459	0.5008	A	0.06175	0.802
19	rs17318596	41937095	G	0.4459	0.5008	A	0.06175	0.802
17	exm1303680	20107773	A	0.2423	0.1975	G	0.06182	1.299
17	rs2703805	20107773	A	0.2423	0.1975	G	0.06182	1.299
22	rs744016	27042106	A	0.2938	0.2458	G	0.06183	1.276
6	rs1341278	81038921	C	0.1237	0.09	A	0.06187	1.427
9	rs10761203	96021312	G	0.451	0.5058	A	0.06187	0.8026
17	rs9901755	63600365	G	0.451	0.5058	A	0.06187	0.8026
8	rs10106074	5700776	A	0.366	0.3142	C	0.06191	1.26
18	rs9675785	35427234	A	0.4536	0.5083	G	0.06192	0.803
20	rs3810553	60989365	A	0.4536	0.5083	G	0.06192	0.803
2	rs145367587	186670379	A	0.005155	0.02	G	0.06193	0.2539
3	rs201038046	185184675	G	0.005155	0.02	A	0.06193	0.2539
4	rs76239807	36118703	A	0.005155	0.02	G	0.06193	0.2539
4	rs1656182	37291336	A	0.4175	0.3642	G	0.06197	1.252
5	rs264530	61626262	G	0.4175	0.3642	A	0.06197	1.252
14	rs3168891	36789729	A	0.02073	0.0425	C	0.06203	0.4768
17	rs2727288	61901197	A	0.3376	0.3908	T	0.06209	0.7945
14	rs3829947	93118038	G	0.268	0.2217	A	0.06245	1.286
17	rs2584625	61903445	G	0.3402	0.3933	C	0.06246	0.7953
12	rs78078157	70048824	A	0.03351	0.01667	T	0.06248	2.045
6	rs1367731	32985199	A	0.1521	0.115	G	0.06254	1.38
7	rs12531640	110479535	A	0.1521	0.115	C	0.06254	1.38
6	rs10948197	44967490	G	0.3711	0.3183	A	0.0626	1.264
19	rs41481648	56467375	A	0.1005	0.07	G	0.06262	1.485
6	rs76776064	138754715	A	0.04639	0.075	G	0.06268	0.6
19	rs4808209	19790159	C	0.125	0.1658	A	0.06281	0.7186
17	rs883544	37387413	A	0.2552	0.3058	G	0.06282	0.7775
15	rs8025430	53374026	G	0.3428	0.3958	A	0.06283	0.7961
17	rs4795958	32982929	A	0.3428	0.3958	G	0.06283	0.7961
11	rs507879	104877927	G	0.2448	0.2	A	0.06293	1.297
6	rs9380182	30703625	C	0.06959	0.045	A	0.06297	1.587
16	rs8051241	75622993	A	0.2036	0.1617	C	0.06314	1.326
9	rs385893	4763176	A	0.1778	0.2233	G	0.06318	0.7522
19	rs3815422	44118188	C	0.1778	0.2233	A	0.06318	0.7522
19	rs2302421	44118353	A	0.1778	0.2233	G	0.06318	0.7522
8	rs13269488	8176221	G	0.3454	0.3983	A	0.0632	0.7969
1	rs34545462	9079301	A	0.06186	0.03833	G	0.06323	1.654
7	rs11971880	6661256	G	0.06186	0.03833	C	0.06323	1.654
2	rs12724	231333763	G	0.06443	0.09583	A	0.06352	0.6498
17	rs581157	72613589	C	0.3479	0.4008	A	0.06356	0.7976

19	rs12980833	9362297	A	0.1392	0.1033	G	0.06367	1.403
6	rs7745906	31204008	A	0.2732	0.2258	G	0.06375	1.289
1	rs544172	208164440	A	0.3531	0.4075	G	0.06388	0.7936
8	rs1801195	30999280	C	0.3531	0.4067	A	0.06396	0.7964
1	rs2820312	201869257	A	0.1399	0.1042	G	0.06398	1.399
6	rs130071	31116210	A	0.1134	0.08083	G	0.06399	1.454
18	rs9518	77288806	G	0.1418	0.1833	A	0.064	0.7357
1	rs11118895	222093006	G	0.4356	0.3817	A	0.06403	1.25
7	rs4730250	107207695	G	0.1856	0.1458	A	0.06403	1.335
12	rs1549338	116162616	G	0.3351	0.285	A	0.06403	1.264
14	rs1760897	20876253	G	0.2036	0.1625	A	0.06406	1.318
3	rs13317829	17157586	A	0.1418	0.1842	G	0.06413	0.7317
20	rs181984	57768607	C	0.09794	0.135	G	0.06421	0.6957
12	rs2638953	28534415	G	0.0799	0.05333	C	0.06425	1.541
8	rs1512268	23526463	A	0.3376	0.2867	G	0.06427	1.268
7	rs782513	134195690	G	0.4356	0.3825	A	0.06441	1.246
2	rs118167062	33412036	A	0.01804	0.006667	G	0.06443	2.738
3	rs294636	14899778	C	0.01804	0.006667	A	0.06443	2.738
6	rs201837919	133105087	G	0.01804	0.006667	A	0.06443	2.738
10	rs45545532	44104101	A	0.01804	0.006667	G	0.06443	2.738
11	rs2234281	2424647	A	0.01804	0.006667	G	0.06443	2.738
13	rs145628951	84454655	C	0.01804	0.006667	A	0.06443	2.738
17	rs77852080	6353739	A	0.01804	0.006667	G	0.06443	2.738
19	rs148736944	52468109	G	0.01804	0.006667	A	0.06443	2.738
22	rs147592073	24567805	C	0.01804	0.006667	A	0.06443	2.738
18	rs2230167	3168816	A	0.3041	0.255	G	0.06446	1.277
6	rs7744164	18170524	G	0.4407	0.3867	A	0.06458	1.25
2	rs12582	234749354	A	0.3814	0.3292	G	0.06474	1.257
1	rs17392348	156555764	G	0.04639	0.02667	C	0.06479	1.776
6	rs142920707	159457244	A	0.04639	0.02667	G	0.06479	1.776
10	rs114687140	129901066	A	0.04639	0.02667	C	0.06479	1.776
10	rs79661992	129902368	A	0.04639	0.02667	T	0.06479	1.776
6	rs3117004	33096766	G	0.4433	0.3892	A	0.06484	1.25
6	rs2294479	33098389	C	0.4433	0.3892	G	0.06484	1.25
3	rs1497546	98034526	A	0.05412	0.0325	G	0.06491	1.703
5	rs17139919	115824639	G	0.05412	0.0325	A	0.06491	1.703
7	rs257376	106799997	A	0.4407	0.3875	G	0.06491	1.246
1	rs200661676	33832873	A	0.01031	0.0025	G	0.06502	4.156
1	rs111928995	89473532	G	0.01031	0.0025	C	0.06502	4.156
1	rs140780079	93142719	G	0.01031	0.0025	A	0.06502	4.156
1	rs201907779	155177615	C	0.01031	0.0025	A	0.06502	4.156
2	rs36109304	111415139	G	0.01031	0.0025	A	0.06502	4.156
2	rs35586251	112922697	A	0.01031	0.0025	G	0.06502	4.156

2	rs199709300	136564924	C	0.01031	0.0025	G	0.06502	4.156
2	rs72646859	179451454	A	0.01031	0.0025	G	0.06502	4.156
3	rs201114442	43122470	A	0.01031	0.0025	G	0.06502	4.156
3	rs76863529	69097297	G	0.01031	0.0025	A	0.06502	4.156
3	rs200099928	126137571	A	0.01031	0.0025	G	0.06502	4.156
4	rs10034345	94344042	A	0.01031	0.0025	G	0.06502	4.156
5	rs200314121	10235337	C	0.01031	0.0025	G	0.06502	4.156
6	rs146808129	43017728	A	0.01031	0.0025	C	0.06502	4.156
6	rs200631044	130762915	A	0.01031	0.0025	T	0.06502	4.156
7	rs200111157	30921965	A	0.01031	0.0025	G	0.06502	4.156
8	rs143762729	10464405	C	0.01031	0.0025	G	0.06502	4.156
8	rs75902455	22020112	A	0.01031	0.0025	G	0.06502	4.156
10	rs200572025	73572294	A	0.01031	0.0025	G	0.06502	4.156
10	rs45514002	88485932	A	0.01031	0.0025	G	0.06502	4.156
11	rs200442057	6261502	A	0.01031	0.0025	G	0.06502	4.156
11	rs199522886	35456196	A	0.01031	0.0025	G	0.06502	4.156
11	rs72472248	124310752	A	0.01031	0.0025	T	0.06502	4.156
12	rs17118953	57114869	G	0.01031	0.0025	A	0.06502	4.156
12	rs2287546	108923971	A	0.01031	0.0025	C	0.06502	4.156
13	rs1020228	67376347	A	0.01031	0.0025	C	0.06502	4.156
14	rs76634907	64489478	G	0.01031	0.0025	A	0.06502	4.156
15	rs201602655	27725918	A	0.01031	0.0025	G	0.06502	4.156
16	rs201557156	3108984	C	0.01031	0.0025	G	0.06502	4.156
16	rs200066548	77353801	G	0.01031	0.0025	A	0.06502	4.156
17	rs151011843	77708914	A	0.01031	0.0025	G	0.06502	4.156
18	rs148791504	44584658	A	0.01031	0.0025	G	0.06502	4.156
21	rs146985381	31744473	A	0.01031	0.0025	G	0.06502	4.156
7	rs1859690	99227172	G	0.232	0.2808	A	0.06508	0.7734
15	rs3809530	59499179	G	0.4459	0.3917	A	0.0651	1.25
4	rs2276938	159780244	G	0.1134	0.08167	C	0.06513	1.438
6	rs928822	30275246	C	0.3814	0.33	A	0.06517	1.252
6	rs9391630	29722515	G	0.3067	0.2575	A	0.06524	1.276
16	rs184411457	1841826	A	0.01031	0.002504	T	0.06529	4.149
20	rs75180889	32228315	G	0.01031	0.002504	A	0.06529	4.149
1	rs11210359	74339167	G	0.03351	0.0575	A	0.06531	0.5682
19	rs17608	40225646	A	0.366	0.42	G	0.06532	0.7971
4	rs11730681	106922960	A	0.3428	0.2917	G	0.06554	1.267
8	rs2280851	133089975	A	0.2758	0.2295	G	0.06554	1.278
13	rs1460443	72580624	G	0.3428	0.2917	A	0.06554	1.267
5	rs2560047	153405414	A	0.384	0.3325	C	0.0656	1.252
9	rs199830055	91262347	A	0.183	0.2275	T	0.06563	0.7605
6	rs1362115	30414848	A	0.1443	0.1867	G	0.06567	0.7349
6	rs2077573	30428351	G	0.1443	0.1867	A	0.06567	0.7349

1	rs11580170	15909744	A	0.0567	0.08583	G	0.06573	0.6402
5	rs3749780	140682757	A	0.0567	0.08667	G	0.06576	0.6335
6	rs14362	33052981	A	0.0567	0.08667	C	0.06576	0.6335
22	rs2040533	31679110	C	0.1134	0.1517	G	0.06585	0.7154
2	rs4312532	55812061	G	0.4536	0.3992	A	0.06586	1.25
1	rs2989476	61059259	C	0.4536	0.4	G	0.06607	1.245
12	rs163683	63121060	G	0.3892	0.3367	A	0.06609	1.255
2	rs2240539	242035544	G	0.08763	0.1217	C	0.0661	0.6934
2	rs3796093	242039198	G	0.08763	0.1217	A	0.0661	0.6934
6	rs1634718	30972865	G	0.09021	0.0625	A	0.0661	1.487
6	rs567448	125374466	G	0.2294	0.1858	A	0.06612	1.304
4	rs4698666	18061500	A	0.3737	0.4275	C	0.06613	0.7991
12	rs12424373	11174795	C	0.1289	0.1686	A	0.0662	0.7294
15	rs2279684	34649247	G	0.2526	0.2075	C	0.06622	1.291
5	rs62366821	44875449	G	0.1418	0.1058	A	0.06629	1.395
1	rs3736360	22150120	A	0.1289	0.1683	G	0.06631	0.7309
1	rs2297654	46086436	G	0.06186	0.03917	A	0.06642	1.617
1	rs6698159	110950277	A	0.06186	0.03917	G	0.06642	1.617
2	rs36105240	152566961	G	0.06186	0.03917	A	0.06642	1.617
10	rs2675703	88414569	A	0.06186	0.03917	G	0.06642	1.617
12	rs2638525	53207628	A	0.06186	0.03917	G	0.06642	1.617
6	rs16871612	44145063	G	0.3918	0.3392	A	0.06653	1.255
1	rs7522061	157668390	G	0.3789	0.4333	A	0.06656	0.7976
2	rs1402867	159105196	A	0.3119	0.3633	G	0.06662	0.7941
9	rs9886752	139634495	A	0.2371	0.2858	G	0.0667	0.7766
19	rs2240233	3831525	A	0.4639	0.4098	C	0.06682	1.246
8	rs11997691	74426588	C	0.4588	0.4058	A	0.0669	1.241
19	rs2240235	3831765	A	0.4639	0.41	G	0.06691	1.245
1	rs143466724	183498580	A	0.01289	0.03	G	0.06695	0.4221
9	rs117858678	112963497	A	0.01289	0.03	G	0.06695	0.4221
22	rs6000181	36598081	A	0.04897	0.07667	T	0.06697	0.6201
6	rs9472414	44946506	T	0.3454	0.2958	A	0.067	1.256
10	rs11594445	95083039	A	0.0799	0.05417	G	0.06702	1.516
18	rs3752095	28934681	T	0.06701	0.09833	A	0.06706	0.6586
16	rs11639856	24788645	A	0.07732	0.1108	T	0.06712	0.6723
16	rs11149631	84228770	C	0.1469	0.1883	G	0.06715	0.7422
1	rs2641348	120437884	G	0.01289	0.03083	A	0.0672	0.4103
6	rs9503233	2623930	A	0.01289	0.03083	G	0.0672	0.4103
13	rs61746911	101320979	G	0.01289	0.03083	A	0.0672	0.4103
3	rs144616051	9883709	A	0.02835	0.01333	G	0.06722	2.159
6	rs1611635	29836463	A	0.02835	0.01333	G	0.06722	2.159
16	rs143980531	4952487	C	0.02835	0.01333	A	0.06722	2.159
16	rs74613280	70512510	A	0.02835	0.01333	C	0.06722	2.159

17	rs61758325	32688804	C	0.02835	0.01333	A	0.06722	2.159
19	rs3746013	40363926	C	0.3866	0.4408	G	0.06722	0.7994
9	rs868454	113283645	G	0.3892	0.443	A	0.06731	0.8009
5	rs10941965	22591472	G	0.2732	0.3242	A	0.0674	0.7837
8	rs2294013	124710729	A	0.1907	0.1508	G	0.06742	1.327
2	rs807573	15807637	A	0.3892	0.4433	C	0.06743	0.8
4	rs2412403	54099223	A	0.4716	0.4175	G	0.06749	1.245
9	rs7039618	108457806	A	0.4691	0.4158	G	0.0676	1.241
6	rs261945	30272417	C	0.3505	0.3	G	0.06763	1.259
4	rs1490470	71439354	A	0.4716	0.4183	G	0.06777	1.241
19	rs77897724	12128726	G	0.1289	0.095	A	0.0678	1.409
2	rs10930709	176641602	A	0.3918	0.445	G	0.06783	0.8033
7	rs7791918	97207509	A	0.3943	0.4483	C	0.06784	0.8011
6	rs6931763	30311932	C	0.116	0.1542	A	0.0679	0.7198
6	rs4407723	150716533	T	0.3144	0.2658	A	0.06793	1.267
20	rs6017218	42555737	A	0.4742	0.4208	C	0.06793	1.241
6	rs2844775	30179422	A	0.2577	0.2117	G	0.06794	1.293
6	rs12199580	36270130	A	0.2577	0.2117	C	0.06794	1.293
20	rs6059034	31623170	G	0.2835	0.2367	A	0.06797	1.276
14	rs3783949	81448382	A	0.2758	0.3267	C	0.06801	0.7849
2	rs10931779	198166825	G	0.4794	0.425	A	0.06802	1.246
15	rs2306331	51217361	A	0.4794	0.425	G	0.06802	1.246
9	rs10975003	5213687	G	0.1314	0.1717	A	0.06804	0.7302
11	rs3815045	61669946	A	0.2758	0.3258	G	0.06806	0.7879
6	rs902780	119108363	G	0.3995	0.3475	A	0.06807	1.249
9	rs2001917	94685926	A	0.3995	0.3475	G	0.06807	1.249
3	rs908821	140540718	G	0.4768	0.4233	A	0.06808	1.241
5	rs3806932	110405675	G	0.3505	0.3008	A	0.06813	1.254
9	rs3808782	17340864	A	0.1314	0.1708	G	0.06813	0.7345
2	rs4832242	86317037	C	0.1675	0.2108	G	0.06814	0.7533
2	rs35284483	160761136	G	0.1675	0.2108	A	0.06814	0.7533
6	rs9381994	51483961	A	0.482	0.4275	G	0.06818	1.246
9	rs10814387	3651396	A	0.482	0.4275	C	0.06818	1.246
11	rs1528753	90884339	A	0.3531	0.3025	C	0.06822	1.259
19	rs11879166	13758270	A	0.4794	0.4258	G	0.06823	1.242
22	rs11703684	25144912	A	0.1675	0.2117	G	0.06823	0.7495
5	rs1698063	70809169	G	0.4021	0.3492	A	0.06824	1.253
3	rs999979	170832832	G	0.4845	0.43	A	0.06835	1.246
2	rs2288073	24413298	G	0.1933	0.1525	A	0.06837	1.332
6	rs2250264	30821187	A	0.1933	0.1525	G	0.06837	1.332
20	rs707555	56137895	G	0.2139	0.2608	C	0.06838	0.7712
14	rs3825725	68035879	G	0.2113	0.17	A	0.06843	1.308
11	rs3740868	94113466	A	0.1418	0.1075	C	0.06865	1.371

19	rs306481	56487603	A	0.3531	0.3033	G	0.06868	1.254
15	rs7181230	40360741	G	0.1495	0.1917	A	0.06872	0.7412
1	rs2749097	64127468	C	0.1753	0.1367	G	0.06875	1.342
18	rs566655	7034508	C	0.1753	0.1367	A	0.06875	1.342
5	rs3846599	10255821	G	0.02835	0.05167	A	0.06876	0.5356
12	rs7972187	10244654	G	0.3557	0.305	A	0.06879	1.258
6	rs6911096	152652034	T	0.1031	0.14	A	0.06884	0.7061
12	rs4767631	118311743	G	0.09021	0.1258	A	0.0689	0.6888
5	rs3749741	39202068	G	0.03351	0.0175	A	0.06891	1.946
17	rs73311647	40274891	G	0.03351	0.0175	A	0.06891	1.946
17	rs192357247	40312063	A	0.03351	0.0175	G	0.06891	1.946
20	rs35562811	34542404	G	0.03351	0.0175	C	0.06891	1.946
11	rs1966836	57982229	A	0.1753	0.137	G	0.06892	1.339
11	exm2249671	129784656	A	0.07216	0.0475	G	0.069	1.56
11	rs75558641	129784656	A	0.07216	0.0475	G	0.069	1.56
3	rs12495784	12531421	A	0.05412	0.03333	G	0.06902	1.659
3	rs77899976	12544779	A	0.05412	0.03333	G	0.06902	1.659
12	rs10777974	99776858	C	0.4871	0.4342	A	0.06902	1.238
16	rs72787027	72033801	A	0.05412	0.03333	C	0.06902	1.659
19	rs111846329	50028731	A	0.05412	0.03333	T	0.06902	1.659
7	rs6465353	91779971	C	0.1907	0.2367	A	0.06905	0.7601
13	rs4770433	23903791	G	0.3273	0.38	A	0.06906	0.7939
11	rs11606506	48238549	A	0.2448	0.2942	G	0.06911	0.778
11	rs7130086	48239182	A	0.2448	0.2942	C	0.06911	0.778
11	rs36184181	56467945	C	0.3273	0.3788	A	0.06916	0.7981
3	rs185135517	10050110	A	0.0232	0.01	G	0.06917	2.351
17	rs2228306	80040034	G	0.0232	0.01	A	0.06917	2.351
22	rs6006417	30647657	G	0.0232	0.01	A	0.06917	2.351
14	rs151012539	20389628	A	0.03351	0.01753	G	0.06919	1.943
1	rs12143943	204572071	A	0.2809	0.3317	G	0.06921	0.7873
13	rs11616874	40642658	G	0.2809	0.3317	A	0.06921	0.7873
6	rs1936230	58344955	C	0.4149	0.4683	A	0.06922	0.8053
6	rs3128931	32971708	A	0.3557	0.3058	G	0.06923	1.253
8	rs7463174	37635649	G	0.3557	0.3058	A	0.06923	1.253
17	rs7223054	39574318	A	0.4149	0.4683	G	0.06925	0.8052
15	rs1042078	48702873	G	0.4948	0.4417	A	0.06932	1.238
11	rs988712	27563382	A	0.1598	0.1225	C	0.06933	1.362
5	rs12520799	134782450	T	0.5026	0.4483	A	0.06934	1.243
16	rs2601828	4103871	A	0.5026	0.4483	G	0.06934	1.243
2	rs4643574	315215	A	0.5052	0.4508	G	0.06944	1.243
16	rs6499601	72981230	G	0.5052	0.4508	A	0.06944	1.243
10	rs1051339	91007339	A	0.08247	0.05583	G	0.06945	1.52
1	rs11579366	145562293	C	0.2603	0.215	G	0.06946	1.285

1	rs337161	220914920	A	0.4304	0.4849	C	0.06953	0.8026
22	rs7288600	35262039	A	0.5077	0.4533	G	0.06953	1.244
8	rs1673408	110455321	A	0.4201	0.4733	C	0.06956	0.8061
5	rs273909	131667353	G	0.04124	0.0225	A	0.06962	1.869
5	rs7732591	142125177	G	0.4072	0.3558	A	0.06966	1.244
13	rs9514685	108081712	A	0.4227	0.4758	G	0.0697	0.8065
18	rs8096163	11422601	A	0.5077	0.4542	G	0.06971	1.24
19	rs2241714	41869392	A	0.5103	0.4567	G	0.06978	1.24
10	rs2504011	33137551	G	0.05928	0.08917	A	0.06979	0.6437
16	rs11859822	28995057	G	0.2835	0.3342	A	0.0698	0.7884
3	exm2255767	151154666	A	0.134	0.1742	G	0.06981	0.7338
3	rs2172250	151154666	A	0.134	0.1742	G	0.06981	0.7338
1	rs2298083	183515428	A	0.05928	0.08833	G	0.06986	0.6503
6	rs2894046	30782105	A	0.4278	0.4808	G	0.06997	0.8074
6	rs4713390	30791971	A	0.4278	0.4808	G	0.06997	0.8074
6	rs805290	31648403	A	0.4278	0.4808	G	0.06997	0.8074
4	rs6854600	38219277	A	0.5206	0.4667	G	0.06998	1.241
7	rs34970805	68066373	G	0.4124	0.36	A	0.06999	1.248
10	rs1937664	53657085	G	0.4098	0.3583	A	0.07001	1.243
1	rs7532151	89388944	C	0.4433	0.4975	A	0.07003	0.8043
6	rs1936797	127432657	G	0.4459	0.5	A	0.07008	0.8047
15	rs1432133	27228346	A	0.1186	0.1575	G	0.0701	0.7195
15	rs8033957	40038433	A	0.4304	0.4833	G	0.0701	0.8078
3	rs77247576	42954792	A	0.04639	0.0275	G	0.07014	1.72
1	rs11206378	40049184	G	0.4407	0.4942	A	0.07016	0.8066
3	rs2087017	113842013	A	0.4536	0.5075	G	0.07021	0.8057
8	rs10505605	134195457	A	0.116	0.085	G	0.07023	1.412
2	rs11678886	81098202	A	0.4433	0.4967	G	0.07024	0.807
6	rs1035798	32151222	A	0.1521	0.1942	G	0.07025	0.7443
12	rs1001178	122691045	T	0.0232	0.04417	A	0.07026	0.5139
5	rs1155473	17779133	A	0.5206	0.4675	G	0.07028	1.237
2	rs3637	85549874	A	0.5361	0.4825	G	0.0703	1.239
6	rs9491696	127452639	C	0.5284	0.475	G	0.07031	1.238
16	rs1984470	53250926	A	0.5309	0.4775	G	0.07031	1.239
19	rs2231940	41944237	A	0.4459	0.4992	G	0.07031	0.8073
6	rs76542212	28198122	A	0.01804	0.0375	G	0.07032	0.4716
11	rs2260655	61108974	G	0.01804	0.0375	A	0.07032	0.4716
15	rs145209254	51012201	G	0.01804	0.0375	C	0.07032	0.4716
22	rs7285167	36623920	A	0.01804	0.0375	G	0.07032	0.4716
6	rs2524276	31408265	A	0.01813	0.03756	C	0.07035	0.4732
11	rs7121446	121954657	A	0.06443	0.04083	G	0.07049	1.618
12	rs7959052	53205757	G	0.06443	0.04083	A	0.07049	1.618
1	rs1407477	168863309	G	0.4536	0.5067	A	0.0705	0.8083

1	rs2794763	236749649	G	0.4562	0.5092	A	0.07056	0.8087
11	rs2276064	118529703	G	0.4562	0.5092	A	0.07056	0.8087
14	rs145613562	105268740	A	0.0232	0.01012	G	0.07066	2.323
20	rs12625057	57790436	C	0.2371	0.1942	A	0.07069	1.29
14	rs72663752	20295627	G	0.1778	0.1392	C	0.0707	1.338
6	rs420054	159655084	C	0.3634	0.3133	G	0.07085	1.251
6	rs386360	159660879	C	0.3634	0.3133	A	0.07085	1.251
2	rs2268416	74690039	G	0.1959	0.1561	A	0.07104	1.317
6	rs396660	29646165	A	0.4175	0.3658	G	0.07104	1.243
1	rs187916559	158613136	A	0.01289	0.004167	G	0.07105	3.12
1	rs17847749	204419139	C	0.01289	0.004167	G	0.07105	3.12
1	rs41303261	207867854	A	0.01289	0.004167	G	0.07105	3.12
2	rs148746411	20838254	A	0.01289	0.004167	G	0.07105	3.12
2	rs140112002	101654110	G	0.01289	0.004167	A	0.07105	3.12
3	rs79363433	151162965	A	0.01289	0.004167	C	0.07105	3.12
3	rs35161724	190158168	C	0.01289	0.004167	G	0.07105	3.12
5	rs143439364	32090660	G	0.01289	0.004167	A	0.07105	3.12
6	rs34525648	25914853	A	0.01289	0.004167	G	0.07105	3.12
6	rs16891235	26017542	G	0.01289	0.004167	A	0.07105	3.12
6	rs28382607	28499674	T	0.01289	0.004167	A	0.07105	3.12
7	rs141979671	21826284	A	0.01289	0.004167	G	0.07105	3.12
7	rs199580691	72745715	A	0.01289	0.004167	G	0.07105	3.12
7	rs185594755	91632424	G	0.01289	0.004167	A	0.07105	3.12
8	rs141849061	37691330	C	0.01289	0.004167	A	0.07105	3.12
9	rs149339002	140001194	A	0.01289	0.004167	G	0.07105	3.12
9	rs75319311	140007492	A	0.01289	0.004167	G	0.07105	3.12
11	rs201796252	66616472	A	0.01289	0.004167	C	0.07105	3.12
12	rs138649928	4835967	A	0.01289	0.004167	G	0.07105	3.12
13	rs80073311	24823928	G	0.01289	0.004167	A	0.07105	3.12
13	rs185071949	114149937	A	0.01289	0.004167	G	0.07105	3.12
16	rs201468609	30078860	A	0.01289	0.004167	G	0.07105	3.12
17	rs199759127	2278821	A	0.01289	0.004167	C	0.07105	3.12
17	exm1370435	80543898	A	0.01289	0.004167	G	0.07105	3.12
17	rs142023304	80543898	A	0.01289	0.004167	G	0.07105	3.12
18	rs143519809	43464613	G	0.01289	0.004167	A	0.07105	3.12
19	rs117421142	54756246	G	0.01289	0.004167	A	0.07105	3.12
21	rs148554210	40883608	A	0.01289	0.004167	G	0.07105	3.12
22	rs2277846	41617247	A	0.01289	0.004167	G	0.07105	3.12
2	rs78943740	177193087	A	0.0232	0.045	G	0.07114	0.504
9	rs2270891	2828742	A	0.1443	0.11	C	0.07114	1.365
19	rs2307019	49244220	G	0.1443	0.11	A	0.07114	1.365
5	rs1375466	119999008	A	0.1057	0.1425	G	0.07115	0.711
14	rs7141532	31864485	C	0.1057	0.1425	G	0.07115	0.711

6	rs2501176	159655383	A	0.3634	0.3139	G	0.07117	1.248
6	rs17083435	121662909	G	0.2397	0.1958	A	0.0712	1.295
1	rs1948368	101938477	A	0.2216	0.2683	G	0.07124	0.7765
18	rs2847537	48328760	A	0.2912	0.245	G	0.0713	1.266
8	rs11538389	144295183	A	0.4201	0.3683	C	0.07137	1.242
6	rs4711319	33107461	A	0.366	0.3158	G	0.07138	1.25
6	rs4713610	33107955	C	0.366	0.3158	A	0.07138	1.25
6	rs721844	33123532	A	0.366	0.3158	G	0.07138	1.25
3	rs9826938	115155648	A	0.2938	0.2467	G	0.07155	1.271
6	rs3799977	44837356	C	0.3686	0.3175	A	0.0716	1.255
6	rs2229523	86199233	A	0.3686	0.3175	G	0.0716	1.255
20	rs6118083	8114704	G	0.2655	0.22	A	0.0716	1.281
3	rs79867908	130116684	A	0.1366	0.1758	G	0.07176	0.7416
6	rs885905	30890431	A	0.1546	0.1967	G	0.07176	0.7472
6	rs1864312	49701439	A	0.4253	0.3725	C	0.07178	1.246
11	rs12422149	74883577	A	0.4253	0.3725	G	0.07178	1.246
16	rs6497618	23008602	A	0.3454	0.3975	G	0.07178	0.7996
16	rs7195703	17475645	G	0.03608	0.06	A	0.07184	0.5865
11	rs3802800	20065779	G	0.05155	0.08	C	0.07192	0.625
19	rs12984043	10672493	A	0.1211	0.1592	G	0.072	0.7281
5	rs1473247	158603571	G	0.4253	0.3733	A	0.07202	1.242
5	rs10076782	158604963	A	0.4253	0.3733	G	0.07202	1.242
6	rs8283	32083300	G	0.4253	0.3733	A	0.07202	1.242
1	rs1057027	159002377	C	0.2552	0.3042	A	0.0721	0.7837
1	rs1057028	159002389	A	0.2552	0.3042	T	0.0721	0.7837
2	rs779357	13052225	G	0.2552	0.3042	A	0.0721	0.7837
6	rs6903608	32428285	G	0.3711	0.32	A	0.07214	1.254
11	rs1145415	100470306	G	0.3479	0.4	A	0.07214	0.8004
12	rs11104738	88500847	G	0.2552	0.303	A	0.07226	0.788
3	rs10936599	169492101	G	0.4278	0.3758	A	0.07234	1.242
2	rs1047911	74699778	C	0.1985	0.1583	A	0.07245	1.316
2	rs6726563	126580416	C	0.1985	0.1583	A	0.07245	1.316
3	rs60808057	49085923	A	0.04381	0.07	C	0.07253	0.6088
5	rs2966952	7868030	A	0.2964	0.3467	G	0.07269	0.7939
17	rs2271536	72351406	A	0.2964	0.3467	G	0.07269	0.7939
6	rs995185	29529137	A	0.3299	0.2817	T	0.07274	1.256
12	rs740842	6421495	A	0.3299	0.2817	G	0.07274	1.256
6	rs2355203	108314167	G	0.3531	0.4058	A	0.07278	0.7991
20	rs6039410	9177112	G	0.3505	0.4017	A	0.07279	0.8039
16	rs3826107	75563330	A	0.1813	0.1427	G	0.07294	1.33
13	rs4771946	97062424	G	0.433	0.3808	A	0.07295	1.242
6	rs769051	33473894	A	0.09536	0.06667	C	0.073	1.476
6	rs9296092	33478496	A	0.09536	0.06667	G	0.073	1.476

2	rs117885048	219894330	A	0.04381	0.07083	G	0.07311	0.6011
1	rs11576457	152918556	G	0.08247	0.1158	A	0.07319	0.6861
6	rs987870	33042880	G	0.08247	0.1158	A	0.07319	0.6861
17	rs11552304	79514129	G	0.4356	0.3833	A	0.07325	1.241
3	rs485499	159745863	G	0.08247	0.115	A	0.07327	0.6917
11	rs7118075	22341713	G	0.08247	0.115	A	0.07327	0.6917
1	rs10863790	209988047	C	0.433	0.3817	A	0.07337	1.237
20	rs527158	56888213	A	0.433	0.3817	C	0.07337	1.237
5	rs26008	131008194	A	0.1572	0.1983	G	0.07339	0.754
15	rs11556218	81598269	C	0.1778	0.2208	A	0.07347	0.7632
12	rs3213764	14587301	A	0.4356	0.384	G	0.07348	1.238
22	rs2235339	37966314	A	0.06443	0.04167	G	0.0735	1.584
9	rs72766544	138439809	G	0.1392	0.18	A	0.07352	0.7365
9	rs55695858	138440554	G	0.1392	0.18	C	0.07352	0.7365
9	rs3178137	138440562	A	0.1392	0.18	G	0.07352	0.7365
13	rs368865	113479820	A	0.1392	0.18	G	0.07352	0.7365
5	rs7702057	115727838	A	0.1186	0.0875	C	0.07353	1.403
6	rs4959079	31488879	A	0.0567	0.035	G	0.07353	1.657
15	rs340029	60894965	G	0.1186	0.0875	A	0.07353	1.403
17	rs10852985	54534634	G	0.0567	0.035	A	0.07353	1.657
19	rs10405150	39387919	G	0.1186	0.0875	A	0.07353	1.403
20	rs4911290	31652292	G	0.2216	0.1792	A	0.07353	1.305
18	rs61734344	580623	G	0.1082	0.1442	A	0.07358	0.7206
2	rs2176186	228476140	A	0.4356	0.3842	G	0.07364	1.237
1	rs1430742	68635075	G	0.2448	0.2008	A	0.0737	1.29
1	rs2566755	68635390	G	0.2448	0.2008	A	0.0737	1.29
1	rs714282	68603129	A	0.3608	0.4133	G	0.07374	0.8012
6	rs6569617	129980436	G	0.3608	0.4133	A	0.07374	0.8012
10	rs2236418	26505496	G	0.3015	0.3525	A	0.07374	0.793
5	rs11949657	133869935	C	0.299	0.2525	A	0.07384	1.263
9	rs3827848	137707834	A	0.1649	0.1275	G	0.07384	1.352
16	rs77379438	4625470	C	0.2656	0.3152	G	0.07389	0.7858
1	rs11264743	153941514	A	0.3789	0.3283	G	0.07395	1.248
22	rs2298428	21982892	A	0.3789	0.3283	G	0.07395	1.248
11	rs1000960	79376133	G	0.2294	0.2758	A	0.07405	0.7815
10	rs3127106	115405615	A	0.3351	0.2867	G	0.07409	1.254
2	rs12477430	185802363	A	0.07216	0.1033	G	0.07412	0.6749
4	rs74764772	24810094	A	0.2216	0.18	G	0.07418	1.297
8	rs4961206	87666251	A	0.2216	0.18	C	0.07418	1.297
3	rs1800572	170732328	A	0.04124	0.02333	G	0.07419	1.8
7	rs3757651	111382099	A	0.04124	0.02333	C	0.07419	1.8
19	rs79831308	4359199	A	0.04124	0.02333	G	0.07419	1.8
3	rs2231926	73111809	G	0.3634	0.415	A	0.07422	0.8047

11	rs3751082	67795353	A	0.2706	0.2258	G	0.07432	1.272
2	rs4668246	171225841	G	0.4459	0.3933	A	0.0744	1.241
18	rs1995595	69497922	A	0.366	0.4175	G	0.07455	0.8054
2	rs3769148	174128513	A	0.1804	0.2242	G	0.07469	0.7618
8	rs1347183	108386442	G	0.4459	0.3942	A	0.07469	1.237
6	rs9501626	32400344	A	0.1649	0.1283	C	0.07472	1.342
9	rs11141788	90019633	G	0.3686	0.42	A	0.07487	0.806
2	rs78987180	10053359	G	0.01031	0.02583	A	0.07488	0.3928
2	rs74867758	27715558	G	0.01031	0.02583	A	0.07488	0.3928
4	rs80169463	3318063	A	0.01031	0.02583	G	0.07488	0.3928
4	rs141034810	71497408	A	0.01031	0.02583	T	0.07488	0.3928
6	rs76326644	2623861	C	0.01031	0.02583	G	0.07488	0.3928
9	rs57348436	115932012	A	0.01031	0.02583	C	0.07488	0.3928
1	rs1010294	15708540	G	0.1598	0.2008	A	0.07493	0.7568
6	rs12154178	152251080	A	0.4485	0.3967	C	0.07494	1.237
20	rs3746737	23424638	A	0.3041	0.2567	G	0.07503	1.266
14	rs3829765	58605790	A	0.3402	0.2908	G	0.07506	1.257
2	rs1470414	242077496	A	0.1418	0.1817	G	0.07508	0.744
2	rs3796098	71211815	A	0.2474	0.2037	G	0.0751	1.285
1	rs117505788	6535149	G	0.04897	0.02917	A	0.07513	1.714
20	rs77627768	55020999	A	0.04897	0.02917	G	0.07513	1.714
6	rs1737063	29732590	G	0.3737	0.4258	A	0.07523	0.8046
6	rs2272592	31698352	A	0.1211	0.08917	G	0.07524	1.408
3	rs10513695	171041055	C	0.2732	0.2283	A	0.07533	1.27
5	rs260081	39198142	G	0.3866	0.3358	A	0.07541	1.246
16	rs16940094	23409440	A	0.01031	0.02667	G	0.07544	0.3802
20	rs3752289	37400374	A	0.01031	0.02667	G	0.07544	0.3802
10	rs2281859	105271758	A	0.3737	0.425	G	0.0755	0.8073
5	rs6893679	146542753	A	0.3041	0.2575	G	0.07551	1.26
8	rs2014357	133103290	G	0.2062	0.2517	A	0.07558	0.7723
7	rs73727632	149509064	G	0	0.009182	A	0.07569	0
19	rs9676419	17367435	G	0.2062	0.2508	A	0.07569	0.7758
1	rs2493292	3328659	A	0	0.009167	G	0.07571	0
1	rs149200172	109714505	A	0	0.009167	G	0.07571	0
2	rs201986939	204066437	A	0	0.009167	G	0.07571	0
3	rs148818522	49160340	A	0	0.009167	G	0.07571	0
4	rs202149994	71232520	A	0	0.009167	G	0.07571	0
5	rs121909212	135391459	A	0	0.009167	C	0.07571	0
6	rs117394742	33281576	A	0	0.009167	G	0.07571	0
8	rs7016219	18729817	G	0	0.009167	A	0.07571	0
8	rs7003060	18729818	A	0	0.009167	C	0.07571	0
10	rs114492585	14896189	G	0	0.009167	A	0.07571	0
10	rs140559573	48370864	A	0	0.009167	G	0.07571	0

10	rs3810960	135087305	A	0	0.009167	G	0.07571	0
11	rs58431577	6129756	A	0	0.009167	G	0.07571	0
14	rs150987491	74516891	A	0	0.009167	G	0.07571	0
15	rs75170357	42805628	G	0	0.009167	A	0.07571	0
16	rs78846023	57492176	C	0	0.009167	G	0.07571	0
19	rs78269670	14705574	G	0	0.009167	A	0.07571	0
1	rs145965895	153751311	C	0.02835	0.01417	G	0.07578	2.03
2	rs75758215	140995843	A	0.02835	0.01417	G	0.07578	2.03
5	rs61740602	150646888	G	0.02835	0.01417	A	0.07578	2.03
6	rs356971	29979797	C	0.02835	0.01417	A	0.07578	2.03
6	rs3129012	29988642	A	0.02835	0.01417	G	0.07578	2.03
6	rs1264709	30056476	T	0.02835	0.01417	A	0.07578	2.03
6	rs146437481	160198427	G	0.02835	0.01417	C	0.07578	2.03
9	rs3739871	35045372	A	0.02835	0.01417	G	0.07578	2.03
9	rs34431454	94486693	A	0.1082	0.07833	G	0.07578	1.428
6	rs9373124	135423209	G	0.3866	0.3367	A	0.0758	1.242
6	rs4895441	135426573	G	0.3866	0.3367	A	0.0758	1.242
6	rs9376092	135427144	A	0.3866	0.3367	C	0.0758	1.242
6	rs9402686	135427817	A	0.3866	0.3367	G	0.0758	1.242
6	rs400488	29943035	A	0.183	0.2267	G	0.07596	0.7641
7	rs1806552	6656897	A	0.183	0.2267	G	0.07596	0.7641
18	rs4939883	47167214	A	0.183	0.2267	G	0.07596	0.7641
6	rs2295192	152443761	A	0.03093	0.05333	G	0.07601	0.5665
2	rs7574414	228243905	A	0.1263	0.165	G	0.07606	0.7315
4	rs2358462	149570680	G	0.183	0.2258	A	0.0761	0.7678
12	rs7961581	71663102	G	0.183	0.2258	A	0.0761	0.7678
19	rs198977	51381777	A	0.183	0.2258	G	0.0761	0.7678
13	rs1414319	110446839	G	0.384	0.4367	A	0.0762	0.8043
6	rs534892	31763942	G	0.03093	0.05417	C	0.07625	0.5573
2	rs6710386	65832209	C	0.4588	0.4075	A	0.07629	1.232
9	rs307658	33941759	G	0.1624	0.2033	A	0.07647	0.7595
16	rs6499188	68674788	G	0.1624	0.2033	A	0.07647	0.7595
7	rs662	94937446	A	0.3144	0.3642	G	0.07649	0.8008
7	rs10953541	107244545	A	0.1856	0.1467	G	0.07651	1.326
13	rs116855232	48619855	A	0.09794	0.1325	G	0.07662	0.7108
3	rs11709533	66539628	A	0.09794	0.1333	G	0.07673	0.7057
14	rs3748338	21167576	T	0.2088	0.2533	A	0.07679	0.7776
6	rs3117572	31717692	A	0.1211	0.09	G	0.07682	1.394
6	rs1634717	30972589	A	0.2784	0.2325	C	0.07683	1.273
11	rs10502001	102398593	A	0.2784	0.2325	G	0.07683	1.273
6	rs2074470	29394427	G	0.4691	0.4175	A	0.07704	1.233
9	rs1832741	110241529	A	0.4742	0.4217	G	0.07712	1.237
18	rs17071550	61383354	A	0.1856	0.2292	G	0.07723	0.7664

3	rs2276805	172428782	A	0.3454	0.2975	G	0.07728	1.246
13	rs1571256	48671081	A	0.3969	0.4492	G	0.07731	0.8071
3	rs3817672	195800811	A	0.2062	0.1658	G	0.07743	1.307
3	rs6790837	124732618	G	0.4794	0.4267	A	0.07749	1.237
11	rs75716864	56020049	G	0.09794	0.06917	A	0.07751	1.461
3	rs78680419	124731689	A	0.3969	0.4483	T	0.07756	0.8098
11	rs2271586	3659993	A	0.3119	0.2642	C	0.07757	1.262
19	rs1982074	10668673	G	0.3119	0.2642	A	0.07757	1.262
10	rs2474570	38383757	A	0.4794	0.4275	G	0.0777	1.233
1	rs487230	55541174	A	0.1366	0.1033	G	0.07775	1.373
15	rs2411837	101110302	C	0.1649	0.2067	G	0.07775	0.7583
22	rs1058642	29957570	A	0.1649	0.2067	G	0.07775	0.7583
3	rs73139148	89530956	A	0.06701	0.04333	G	0.07779	1.586
4	rs1949733	8503359	A	0.482	0.43	G	0.07786	1.233
11	rs78972632	431757	A	0.06443	0.09417	G	0.07786	0.6625
7	rs3779536	127233977	A	0.1082	0.07917	C	0.07788	1.412
2	rs16866933	180566678	A	0.4046	0.4567	G	0.07792	0.8086
8	rs2954029	126490972	A	0.4046	0.4567	T	0.07792	0.8086
6	rs1233425	29498497	C	0.4845	0.4325	G	0.078	1.233
16	rs2304492	46697098	A	0.4845	0.4325	G	0.078	1.233
16	rs13336114	54967096	C	0.4072	0.46	A	0.078	0.8064
18	rs7237848	4773110	A	0.3995	0.3492	G	0.078	1.24
6	rs41266821	26247198	G	0.04639	0.0725	A	0.07801	0.6224
8	rs7816436	21650993	G	0.4021	0.4533	A	0.07802	0.8109
9	rs634710	127661645	G	0.4021	0.4533	A	0.07802	0.8109
5	rs381852	54459961	A	0.1134	0.15	G	0.07805	0.7248
3	rs9289324	96929437	G	0.1289	0.1667	A	0.07809	0.7396
1	rs3860340	95276691	A	0.4871	0.435	G	0.07814	1.234
3	rs12053903	38593393	A	0.4871	0.435	G	0.07814	1.234
1	rs1569053	39952822	A	0.3247	0.375	G	0.07824	0.8015
4	rs11737685	9903121	G	0.4046	0.4558	A	0.07824	0.8114
6	rs2294883	32367451	A	0.3247	0.375	T	0.07824	0.8015
3	rs652889	61794054	A	0.4897	0.4375	C	0.07828	1.234
6	rs543650	152110943	A	0.4098	0.4617	C	0.07829	0.8096
12	rs80046313	31106941	C	0.07672	0.1095	A	0.07833	0.6755
6	rs362540	29510325	G	0.4923	0.44	A	0.07841	1.234
20	rs2070317	31622083	G	0.2294	0.1875	A	0.07841	1.29
3	rs4687657	52852538	A	0.4021	0.3517	C	0.07842	1.24
14	rs8014577	73963383	A	0.1469	0.1867	G	0.07856	0.7503
22	rs9616906	51104680	A	0.1521	0.1175	G	0.0786	1.347
3	rs4859259	182664547	A	0.1469	0.1875	G	0.07861	0.7462
19	rs17159388	8320555	A	0.1469	0.1875	G	0.07861	0.7462
6	rs9500256	58308955	A	0.4974	0.445	G	0.07865	1.234

7	rs78008037	14188788	A	0.03608	0.01917	G	0.07869	1.916
14	rs78561092	95670136	A	0.03608	0.01917	G	0.07869	1.916
5	rs3761967	70800538	A	0.4046	0.3542	G	0.07884	1.239
5	rs715748	70806457	A	0.4046	0.3542	G	0.07884	1.239
5	rs1961760	70806649	T	0.4046	0.3542	A	0.07884	1.239
2	rs1385351	193333903	G	0.2835	0.2375	A	0.07887	1.27
2	rs1586249	156136927	A	0.5052	0.4525	C	0.07897	1.235
2	rs6705540	10520520	G	0.4253	0.4775	A	0.07901	0.8096
6	rs11796	31501212	T	0.5	0.4483	A	0.07903	1.23
3	rs61733583	180334458	A	0.07732	0.05167	G	0.07909	1.538
6	rs16870908	32790089	A	0.07732	0.05167	G	0.07909	1.538
7	rs3752368	157931091	A	0.07732	0.05167	G	0.07909	1.538
5	rs12518099	89546109	G	0.5103	0.4575	A	0.07916	1.236
6	rs6557488	156601505	A	0.2448	0.2917	C	0.07922	0.7874
14	rs162654	83778352	A	0.433	0.4858	G	0.07925	0.8082
16	rs7499814	88052161	A	0.433	0.4858	C	0.07925	0.8082
2	rs4666689	186670979	A	0.1005	0.135	G	0.07928	0.716
6	rs2270190	31080586	G	0.1005	0.135	A	0.07928	0.716
4	rs6813195	153520475	A	0.4253	0.4767	G	0.07929	0.8123
2	rs7557452	205926400	G	0.433	0.485	A	0.07936	0.8109
6	rs3806156	32373698	A	0.433	0.485	C	0.07936	0.8109
2	rs10932860	156935758	A	0.5129	0.4608	G	0.07939	1.232
1	rs3200254	21894735	A	0.4278	0.4791	G	0.0794	0.8129
3	rs79308644	133538548	G	0.02577	0.04667	A	0.07941	0.5404
6	rs4122190	31167852	G	0.1366	0.1042	A	0.07943	1.361
17	rs6503870	56659018	A	0.4356	0.4875	G	0.07946	0.8113
6	rs3752430	32832667	A	0.3325	0.3833	G	0.07951	0.8012
4	rs7689609	72083374	A	0.4304	0.4817	G	0.07957	0.8132
11	rs34063493	372157	G	0.5232	0.4708	A	0.07957	1.233
2	rs11556887	231077676	A	0.1108	0.08083	G	0.07962	1.417
5	rs4702813	11742668	G	0.1108	0.08083	A	0.07962	1.417
6	rs9267649	31824828	A	0.1108	0.08083	G	0.07962	1.417
17	rs34367357	78172292	A	0.1108	0.08083	G	0.07962	1.417
11	rs2286614	64068310	A	0.1392	0.105	G	0.07963	1.378
6	rs7752169	136227558	G	0.4433	0.495	A	0.07974	0.8124
9	rs7028052	5703443	A	0.1907	0.2342	G	0.07974	0.7707
6	rs41545814	31382893	G	0.01823	0.007513	A	0.07976	2.453
1	rs78555129	1178482	G	0.232	0.19	A	0.07981	1.288
1	rs12093154	1178925	A	0.232	0.19	G	0.07981	1.288
3	rs2378446	188568652	C	0.232	0.19	A	0.07981	1.288
5	rs7731457	12714599	A	0.5361	0.4842	G	0.07984	1.231
11	rs2276285	76901927	G	0.5361	0.4842	A	0.07984	1.231
2	rs6434831	197078453	A	0.5335	0.4817	G	0.07986	1.231

12	rs2433651	22968378	A	0.5232	0.4717	G	0.07986	1.229
1	rs1015100	232007196	G	0.5258	0.4742	A	0.07987	1.23
3	rs57006145	113165480	C	0.08763	0.06083	A	0.07989	1.483
19	rs62617084	39230852	A	0.08763	0.06083	G	0.07989	1.483
16	rs1035543	71983772	G	0.3351	0.3858	C	0.07994	0.8021
8	rs10957350	66330462	G	0.2552	0.2125	A	0.07997	1.269
1	rs669694	209950760	G	0.1314	0.17	C	0.07999	0.7389
6	rs429608	31930462	A	0.1237	0.0925	G	0.0801	1.385
19	rs12975322	8176640	A	0.4562	0.5075	G	0.0801	0.8141
6	rs1051484	105726036	A	0.2861	0.2408	G	0.08029	1.263
1	rs34287852	41296828	C	0.06842	0.045	A	0.08037	1.559
13	rs1199742	21529256	G	0.4124	0.3625	A	0.08043	1.234
15	rs17514846	91416550	A	0.116	0.1533	C	0.08054	0.7244
5	rs3792646	40955653	C	0.06701	0.04417	A	0.08062	1.554
8	rs4397449	90342186	A	0.06701	0.04417	G	0.08062	1.554
1	rs12139527	201009182	G	0.1392	0.1058	A	0.08063	1.366
3	rs683395	182869818	G	0.1392	0.1058	A	0.08063	1.366
5	rs79378421	179135353	A	0.01546	0.03333	C	0.08069	0.4555
6	rs33941312	31084191	G	0.01546	0.03333	A	0.08069	0.4555
13	rs144650014	67802256	G	0.01546	0.03333	A	0.08069	0.4555
15	rs77028575	84373247	A	0.01546	0.03333	G	0.08069	0.4555
14	rs7158139	102792631	A	0.4149	0.365	G	0.0808	1.234
6	rs9380234	31248568	A	0.2124	0.172	G	0.08083	1.299
12	rs4980859	274647	G	0.3608	0.3125	A	0.08085	1.242
1	rs11163585	83311835	A	0.04124	0.02417	G	0.08089	1.737
5	rs871234	174485598	C	0.04124	0.02417	A	0.08089	1.737
6	rs2307307	24781719	C	0.04124	0.02417	G	0.08089	1.737
6	rs2307306	24781735	A	0.04124	0.02417	G	0.08089	1.737
12	rs17848270	12397266	A	0.04124	0.02417	T	0.08089	1.737
19	rs11671653	10838486	A	0.04124	0.02417	G	0.08089	1.737
5	rs4704896	157194402	G	0.25	0.2967	A	0.08092	0.7903
14	rs169748	54186083	G	0.3196	0.2733	A	0.08095	1.249
1	rs141548164	103444970	T	0.0232	0.01083	A	0.08096	2.168
2	rs57677160	242149010	A	0.0232	0.01083	G	0.08096	2.168
6	rs3117286	29629774	G	0.0232	0.01083	A	0.08096	2.168
6	rs144521033	29637986	A	0.0232	0.01083	G	0.08096	2.168
6	rs184974475	29640416	C	0.0232	0.01083	G	0.08096	2.168
6	rs200537697	29640855	G	0.0232	0.01083	C	0.08096	2.168
6	rs146835830	29641356	G	0.0232	0.01083	A	0.08096	2.168
6	rs201703858	29641413	A	0.0232	0.01083	T	0.08096	2.168
6	rs2747419	29642194	A	0.0232	0.01083	C	0.08096	2.168
6	rs3129051	29650122	A	0.0232	0.01083	T	0.08096	2.168
7	rs186329804	110762920	A	0.0232	0.01083	G	0.08096	2.168

10	rs140638790	25886933	C	0.0232	0.01083	G	0.08096	2.168
10	rs137918654	35485028	A	0.0232	0.01083	C	0.08096	2.168
10	rs114312744	134013974	A	0.0232	0.01083	C	0.08096	2.168
17	rs138195513	79893013	A	0.0232	0.01083	G	0.08096	2.168
21	rs34613278	40720494	A	0.0232	0.01083	G	0.08096	2.168
22	rs184789735	30415938	A	0.0232	0.01083	C	0.08096	2.168
1	rs12078797	219465410	A	0.2887	0.3375	G	0.08097	0.7966
3	rs4685076	14174427	T	0.3402	0.39	A	0.08097	0.8065
2	rs78122518	234408547	A	0.07732	0.0525	G	0.08113	1.512
2	rs79471829	234465572	T	0.07732	0.0525	A	0.08113	1.512
4	rs12504628	145436324	G	0.2887	0.3367	A	0.08117	0.7995
3	rs3821875	48697654	C	0.0567	0.085	G	0.08118	0.6471
4	rs3796780	25278988	G	0.2345	0.1925	A	0.0812	1.285
19	rs2230062	33878977	A	0.07772	0.1092	G	0.08121	0.6877
11	exm2250192	67068859	A	0.1727	0.1358	G	0.08125	1.328
11	rs2298815	67068859	A	0.1727	0.1358	G	0.08125	1.328
17	rs45557233	80041950	A	0.07732	0.1093	G	0.08125	0.6825
2	rs1020064	105897740	A	0.2603	0.2167	C	0.08147	1.272
3	rs373188	120511490	C	0.4201	0.37	A	0.08151	1.234
21	rs12329760	42852497	A	0.4201	0.37	G	0.08151	1.234
1	rs13306766	9097656	A	0.01804	0.0075	T	0.08154	2.431
1	rs144969324	197072871	A	0.01804	0.0075	G	0.08154	2.431
1	rs199851348	223567028	A	0.01804	0.0075	G	0.08154	2.431
2	rs140776586	240960670	G	0.01804	0.0075	A	0.08154	2.431
7	rs202111578	98257814	G	0.01804	0.0075	A	0.08154	2.431
8	rs20575	23059324	G	0.01804	0.0075	C	0.08154	2.431
8	rs17620	23060256	A	0.01804	0.0075	G	0.08154	2.431
8	rs142252012	23225732	A	0.01804	0.0075	G	0.08154	2.431
9	rs61752940	14619085	A	0.01804	0.0075	G	0.08154	2.431
13	rs186364861	48611934	A	0.01804	0.0075	G	0.08154	2.431
14	rs117207261	77735581	G	0.01804	0.0075	C	0.08154	2.431
16	rs201050302	4815871	A	0.01804	0.0075	G	0.08154	2.431
17	rs79007502	33880305	G	0.01804	0.0075	A	0.08154	2.431
19	rs144517416	47572592	A	0.01804	0.0075	G	0.08154	2.431
20	rs75021933	21349138	G	0.01804	0.0075	A	0.08154	2.431
22	rs5753700	31971258	A	0.01804	0.0075	G	0.08154	2.431
22	rs9621295	32007153	A	0.01804	0.0075	G	0.08154	2.431
22	rs16989427	32108475	A	0.01804	0.0075	G	0.08154	2.431
6	rs2071596	31506691	A	0.4227	0.3717	G	0.08159	1.238
17	rs12941884	27284443	G	0.1108	0.08167	A	0.08163	1.402
1	rs143089777	150956469	G	0	0.01	A	0.08175	0
6	rs3177928	32412435	A	0.06701	0.09667	G	0.08188	0.6712
6	rs13209234	32415975	A	0.06701	0.09667	G	0.08188	0.6712

10	rs200241224	102739612	C	0.01804	0.007513	G	0.08189	2.427
20	rs6018027	35990792	G	0.1031	0.1383	A	0.08191	0.716
6	rs4947248	31302025	G	0.134	0.1725	A	0.08195	0.7424
7	rs17165936	12414725	A	0.2887	0.2442	G	0.08195	1.256
16	rs59692769	67214416	A	0.1521	0.1917	G	0.08201	0.7563
19	rs3745379	7963948	A	0.1005	0.07167	G	0.08203	1.448
3	rs200611323	49700471	A	0	0.01	C	0.08204	0
1	rs3795393	153509890	G	0.1727	0.215	A	0.08219	0.7621
5	rs3733709	140181648	G	0.05928	0.0375	A	0.08219	1.617
6	rs115194949	30993440	A	0.05928	0.0375	G	0.08219	1.617
8	rs2931309	62096568	G	0.05928	0.0375	A	0.08219	1.617
14	rs36563	71352648	A	0.1727	0.215	C	0.08219	0.7621
1	rs7414562	14359780	G	0.4253	0.375	A	0.08221	1.233
20	rs2057334	38233163	G	0.2938	0.3425	A	0.08222	0.7987
2	rs1048603	234394487	A	0.1392	0.1067	G	0.08223	1.354
11	rs746463	109995944	G	0.1392	0.1067	A	0.08223	1.354
6	rs1150674	28134368	G	0.09021	0.1233	A	0.08225	0.7048
7	rs3801150	139685695	G	0.1959	0.24	A	0.08225	0.7714
17	rs2304449	5044794	A	0.09021	0.1233	G	0.08225	0.7048
4	rs28642966	47945295	A	0.3479	0.3975	G	0.08226	0.8088
6	rs3131931	30945265	T	0.08763	0.06167	A	0.08228	1.461
17	rs35682745	72240168	A	0.08763	0.06167	G	0.08228	1.461
7	rs13230671	153144983	G	0.366	0.318	A	0.08231	1.238
17	rs114991387	5037281	G	0.09021	0.1227	A	0.08236	0.7089
1	rs41315701	145562087	A	0.1727	0.1367	G	0.08246	1.319
7	rs117711958	48311803	A	0.007732	0.02167	C	0.0825	0.3518
22	rs117546628	23956340	T	0.007732	0.02167	A	0.0825	0.3518
21	rs139612839	47738035	A	0.02062	0.04083	G	0.08253	0.4945
3	rs10936600	169514585	A	0.4278	0.3775	T	0.08254	1.233
6	rs7753890	136516257	G	0.2371	0.195	A	0.08259	1.283
3	rs13100173	50332697	A	0.1186	0.155	G	0.08261	0.7333
15	rs77836884	42986787	A	0.08763	0.06177	G	0.08262	1.459
1	rs187729751	17303630	C	0.01546	0.005	A	0.08268	3.126
3	rs186026958	48265146	A	0.01546	0.005	G	0.08268	3.126
3	rs142494006	127318252	A	0.01546	0.005	G	0.08268	3.126
3	rs117589009	130732909	G	0.01546	0.005	A	0.08268	3.126
6	rs1269854	32070838	G	0.01546	0.005	A	0.08268	3.126
6	rs204894	32093922	A	0.01546	0.005	G	0.08268	3.126
6	rs1057149	32814942	A	0.01546	0.005	G	0.08268	3.126
6	rs41550019	32816772	A	0.01546	0.005	C	0.08268	3.126
8	rs114230243	121267497	G	0.01546	0.005	A	0.08268	3.126
8	rs61740411	145727240	A	0.01546	0.005	G	0.08268	3.126
9	rs200551293	100975481	A	0.01546	0.005	G	0.08268	3.126

10	rs142448790	98742081	A	0.01546	0.005	G	0.08268	3.126
11	rs200464179	124016024	A	0.01546	0.005	G	0.08268	3.126
14	rs61741258	31765126	G	0.01546	0.005	A	0.08268	3.126
15	rs8923	80137560	G	0.01546	0.005	A	0.08268	3.126
15	rs117123311	90185464	G	0.01546	0.005	C	0.08268	3.126
19	rs184799000	46124782	A	0.01546	0.005	C	0.08268	3.126
19	rs114383943	54973821	A	0.01546	0.005	G	0.08268	3.126
20	rs144258253	18163863	A	0.01546	0.005	G	0.08268	3.126
20	rs141888881	31826009	C	0.01546	0.005	G	0.08268	3.126
4	rs11568849	110834537	C	0.04381	0.025	A	0.08269	1.787
11	rs61877970	15170686	A	0.04381	0.025	G	0.08269	1.787
19	rs140441769	39914638	A	0.04381	0.025	C	0.08269	1.787
7	rs61729490	151684256	G	0.1186	0.1544	A	0.08274	0.7365
10	rs3020498	135379710	A	0.1186	0.1544	C	0.08274	0.7365
6	rs9468830	30749712	A	0.3531	0.4033	C	0.08275	0.8074
6	rs4713360	30753146	A	0.3531	0.4033	G	0.08275	0.8074
6	rs6920124	30756593	G	0.3531	0.4033	A	0.08275	0.8074
6	rs3131043	30758466	A	0.3531	0.4033	G	0.08275	0.8074
2	rs59901247	46609572	C	0.1186	0.1558	A	0.08276	0.7286
3	rs9290364	168995182	G	0.3711	0.3217	A	0.08283	1.245
9	rs3808794	17273754	C	0.2912	0.2467	A	0.08288	1.255
14	rs200541919	74035960	A	0.01804	0.00755	G	0.08294	2.415
2	rs13429725	147292648	G	0.4278	0.3783	A	0.08303	1.229
18	rs276933	28618549	C	0.3711	0.3225	A	0.08311	1.24
21	rs2839500	43803167	G	0.3711	0.3225	A	0.08311	1.24
1	rs17367504	11862778	G	0.1263	0.095	A	0.08337	1.377
12	rs1132375	133331537	A	0.2371	0.1958	G	0.08337	1.276
2	rs3749172	241570249	A	0.3299	0.2825	C	0.08346	1.25
2	rs12617311	199632565	A	0.3582	0.4083	G	0.08351	0.8089
9	rs3739523	79319874	A	0.1572	0.1225	C	0.08353	1.336
1	rs11247919	26664968	A	0.007732	0.0225	G	0.08354	0.3385
1	rs74228558	151733327	G	0.007732	0.0225	A	0.08354	0.3385
11	rs12292394	134062764	A	0.007732	0.0225	G	0.08354	0.3385
17	rs2304971	4642633	G	0.007732	0.0225	A	0.08354	0.3385
18	rs2276374	29848306	A	0.007732	0.0225	G	0.08354	0.3385
9	rs10968576	28414339	G	0.1753	0.2175	A	0.0836	0.7645
16	rs11643211	77401545	A	0.433	0.3833	G	0.08363	1.228
6	rs420137	159652931	G	0.3737	0.325	C	0.08366	1.239
6	rs2224660	10105007	C	0.1005	0.0725	A	0.08373	1.43
17	rs722375	33746574	A	0.03351	0.05667	G	0.08375	0.5771
1	rs10913157	171486912	A	0.2655	0.2217	G	0.08379	1.269
15	rs7168069	68624396	A	0.04897	0.07583	C	0.0838	0.6275
2	rs10511	234749258	G	0.3782	0.3292	A	0.08395	1.24

6	rs3129891	32415080	A	0.3015	0.3508	G	0.08398	0.7989
12	rs4763305	13037300	A	0.3015	0.3508	G	0.08398	0.7989
3	rs1355562	122397064	C	0.3634	0.4142	A	0.08406	0.8075
2	rs4851173	99520608	G	0.4407	0.39	A	0.08414	1.233
5	rs4371716	22078584	A	0.1959	0.1567	G	0.08414	1.311
7	rs6961002	71881828	G	0.4407	0.39	A	0.08414	1.233
14	rs1543540	102798055	A	0.3763	0.3275	G	0.0842	1.239
17	rs4968839	67125840	A	0.3634	0.4133	G	0.08423	0.8102
11	rs1939749	62910849	A	0.07732	0.05333	G	0.08427	1.487
11	rs1939748	62911079	C	0.07732	0.05333	G	0.08427	1.487
12	rs7975069	133778796	A	0.07732	0.05333	G	0.08427	1.487
20	rs2424943	31671619	A	0.07732	0.05333	C	0.08427	1.487
5	rs328661	118951870	G	0.4407	0.3908	A	0.08449	1.228
14	rs4466998	65475540	C	0.4407	0.3908	A	0.08449	1.228
9	rs28365557	131830567	A	0.03608	0.02	G	0.08457	1.834
12	rs1688565	89678470	A	0.03608	0.02	G	0.08457	1.834
6	rs2647087	32681049	C	0.3041	0.3525	A	0.08463	0.8028
17	rs9303651	25787234	A	0.3041	0.3525	G	0.08463	0.8028
11	rs79525962	76371418	A	0.0799	0.1108	G	0.08467	0.6966
7	rs3807989	116186241	A	0.3763	0.3283	G	0.08471	1.234
16	rs12598590	64116270	A	0.4459	0.395	G	0.08474	1.232
11	rs2499953	5010905	G	0.2294	0.275	A	0.08484	0.7847
6	rs2239528	31510105	A	0.1211	0.1575	G	0.08487	0.7373
6	rs886390	30334994	A	0.1598	0.1242	C	0.08496	1.341
6	rs970270	30347306	A	0.1598	0.1242	G	0.08496	1.341
12	rs919837	106507724	G	0.1598	0.1242	A	0.08496	1.341
5	rs4296809	44186668	A	0.1778	0.22	C	0.08501	0.7669
6	rs1046089	31602967	A	0.3711	0.4217	G	0.08502	0.8094
12	rs12310307	116335519	C	0.4485	0.3975	A	0.08503	1.232
7	rs886716	26557618	G	0.09021	0.06333	A	0.08509	1.466
11	rs2276113	102196019	G	0.06959	0.04583	A	0.08509	1.557
1	rs10927101	244173872	C	0.3789	0.3308	A	0.08522	1.234
2	rs219553	21577743	A	0.1572	0.1975	G	0.08529	0.758
16	rs1728785	68591230	A	0.1572	0.1975	C	0.08529	0.758
6	rs3129961	32378940	G	0.2423	0.2	A	0.08533	1.279
17	rs783239	72584764	A	0.2423	0.2	G	0.08533	1.279
9	rs913588	7174673	A	0.1572	0.1967	G	0.08542	0.762
10	rs2986014	105194086	A	0.4536	0.4025	G	0.08559	1.232
3	rs6787633	64532476	G	0.268	0.225	C	0.0856	1.261
3	rs6793295	169518455	A	0.299	0.2542	G	0.08563	1.251
6	rs6557316	154093266	A	0.3763	0.4267	G	0.08564	0.8107
18	rs3745051	3729175	A	0.3814	0.3333	G	0.08572	1.233
21	rs2822432	15516948	A	0.05928	0.0875	G	0.08578	0.6571

2	rs1437898	133746630	A	0.384	0.335	C	0.08579	1.238
18	rs2276092	2707619	G	0.3789	0.4292	A	0.08594	0.8113
10	rs7908422	37074863	A	0.4637	0.4125	G	0.08601	1.232
20	rs6108011	7552504	A	0.3015	0.2558	G	0.08601	1.256
11	rs308341	67795299	A	0.2706	0.2267	G	0.08608	1.266
9	rs7863126	31819635	G	0.05959	0.08833	A	0.08617	0.6539
3	rs13084066	78608819	G	0.06959	0.1	A	0.0862	0.6731
7	rs2967917	81056006	A	0.3814	0.4317	G	0.08623	0.8119
16	rs3095627	47276820	C	0.2706	0.227	A	0.08627	1.263
6	rs7765781	161007496	C	0.4588	0.4083	G	0.0863	1.228
12	rs11045689	21201663	A	0.4588	0.4083	G	0.0863	1.228
11	rs11022079	11913585	C	0.1443	0.1108	A	0.08635	1.353
6	rs572280	165798068	A	0.4562	0.4067	C	0.08651	1.224
20	rs1015362	32738612	A	0.1804	0.2217	G	0.08651	0.7729
11	rs1793615	131088026	A	0.384	0.4342	G	0.08652	0.8125
19	rs2651079	35450229	G	0.4613	0.4108	A	0.08653	1.228
4	rs993097	167407097	G	0.3866	0.4375	A	0.08665	0.8103
14	rs8019076	62462766	A	0.1985	0.16	G	0.08668	1.3
14	rs34015250	24026493	G	0.2706	0.2275	A	0.0867	1.26
22	rs5751416	43432826	A	0.3144	0.3633	G	0.08674	0.8037
17	rs77302552	76798435	A	0.04381	0.02583	G	0.08686	1.728
6	rs520692	32188640	G	0.1598	0.2	A	0.08693	0.7607
4	rs4414961	99403784	C	0.2062	0.25	A	0.08697	0.7792
17	rs7406339	22002556	A	0.4665	0.4158	G	0.08698	1.228
3	rs1873351	72650801	A	0.3892	0.4392	G	0.08707	0.8136
2	rs13428812	25492467	G	0.2062	0.2492	A	0.08709	0.7827
5	rs1461237	153238323	G	0.1237	0.16	A	0.08712	0.7412
4	rs6854783	145643079	G	0.4639	0.4142	A	0.08713	1.224
4	rs1492820	145650021	G	0.4639	0.4142	A	0.08713	1.224
6	rs3095250	31208340	G	0.3918	0.4425	A	0.08714	0.8115
20	rs1223271	13296912	A	0.3918	0.4425	G	0.08714	0.8115
2	rs1552280	179644035	G	0.1237	0.1608	A	0.08716	0.7366
13	rs4884881	70849153	A	0.2216	0.1808	G	0.08717	1.29
6	rs139136879	76728524	A	0.0799	0.055	G	0.08718	1.492
10	rs2105450	5115907	C	0.2706	0.3183	A	0.08718	0.7945
16	rs6497671	23536684	A	0.2706	0.3183	G	0.08718	0.7945
9	rs5320	136507473	A	0.1082	0.1425	G	0.08719	0.7305
1	rs9651057	201175274	A	0.4665	0.4167	G	0.08732	1.224
15	rs8033590	88385150	G	0.3918	0.4417	A	0.08734	0.8142
1	rs16831482	160141171	C	0.09021	0.06417	A	0.08736	1.446
6	rs1634725	30982008	T	0.09021	0.06417	A	0.08736	1.446
18	rs2447437	76753768	G	0.09021	0.06417	C	0.08736	1.446
2	rs7603541	69147566	G	0.3943	0.445	A	0.08738	0.812

6	rs3129878	32408735	C	0.3943	0.445	A	0.08738	0.812
18	rs3737374	29432624	G	0.4716	0.4208	A	0.08741	1.229
8	rs7013595	83432030	A	0.3428	0.2958	G	0.0875	1.241
6	rs3131875	29666111	A	0.317	0.365	G	0.08751	0.8075
2	rs13421358	43247227	G	0.3969	0.4475	A	0.08761	0.8125
19	rs7248104	7224431	A	0.3918	0.3433	G	0.08766	1.232
19	rs140363712	1789960	G	0.06959	0.04667	A	0.08772	1.528
6	rs5370	12296255	A	0.2371	0.2825	C	0.08773	0.7894
1	rs4601609	59325626	G	0.1985	0.1608	A	0.08779	1.292
4	rs3747690	48988450	A	0.1985	0.1608	C	0.08779	1.292
3	rs148289202	53153964	A	0.03093	0.01583	G	0.08797	1.984
6	rs2273613	31750517	G	0.03093	0.01583	A	0.08797	1.984
6	rs189701030	116833161	A	0.03093	0.01583	C	0.08797	1.984
10	exm1680169	45878098	A	0.03093	0.01583	G	0.08797	1.984
10	rs150281723	45878098	A	0.03093	0.01583	G	0.08797	1.984
11	rs74985106	71720030	A	0.03093	0.01583	G	0.08797	1.984
12	rs76462677	123344040	A	0.03093	0.01583	G	0.08797	1.984
16	rs199819672	3017966	A	0.03093	0.01583	G	0.08797	1.984
19	rs79890896	53270623	C	0.03093	0.01583	G	0.08797	1.984
20	rs2253427	1552430	A	0.2474	0.205	G	0.08803	1.275
2	rs1117324	21840093	G	0.08247	0.1142	A	0.08805	0.6974
4	rs12507775	74451073	G	0.08247	0.1142	A	0.08805	0.6974
6	rs1007968	55194896	A	0.3196	0.3675	G	0.08806	0.8084
12	rs711101	77982647	A	0.3995	0.4492	C	0.08812	0.8158
10	rs11106	129899778	G	0.5157	0.465	C	0.08825	1.225
11	rs4375446	66910826	A	0.3067	0.2617	G	0.08832	1.248
1	rs147441599	1269432	A	0.03093	0.01589	C	0.08833	1.977
14	rs223124	23992742	C	0.2758	0.2317	A	0.08833	1.263
9	rs6477925	115155264	A	0.4021	0.4517	G	0.08836	0.8163
13	rs9574873	81973439	A	0.4072	0.4583	G	0.08837	0.8119
16	rs13330384	87701468	A	0.482	0.4317	G	0.08837	1.225
1	rs35089879	109461341	A	0.005155	0.0175	G	0.0884	0.2909
2	rs4664950	159201754	G	0.005155	0.0175	A	0.0884	0.2909
3	rs2286786	52547929	G	0.005155	0.0175	C	0.0884	0.2909
4	rs116129895	2746583	A	0.005155	0.0175	C	0.0884	0.2909
6	rs34764696	43112267	A	0.005155	0.0175	G	0.0884	0.2909
11	rs73404785	1718833	C	0.005155	0.0175	A	0.0884	0.2909
7	rs4724399	45522229	G	0.4072	0.4575	A	0.08847	0.8146
20	rs2903808	44505973	A	0.4072	0.4575	G	0.08847	0.8146
13	rs78799989	111367876	G	0.02835	0.04917	C	0.08853	0.5643
4	rs2178603	85090749	G	0.4046	0.4542	A	0.0886	0.8168
12	exm2221241	9243846	A	0.4223	0.4733	G	0.08861	0.8134
5	rs7718708	84452107	A	0.4124	0.4633	C	0.08871	0.8128

9	rs1785506	34017106	G	0.1624	0.2017	A	0.08879	0.7674
3	rs3204024	113666020	C	0.3247	0.3742	G	0.0888	0.8044
12	rs10783528	53088484	G	0.3247	0.3742	A	0.0888	0.8044
5	rs1800197	177419967	A	0.02835	0.05	G	0.08892	0.5544
2	rs780094	27741237	G	0.4923	0.4417	A	0.08895	1.226
4	rs2194926	138232010	A	0.4923	0.4417	C	0.08895	1.226
15	rs7170666	89154966	A	0.4175	0.4683	G	0.08903	0.8137
1	rs4845625	154422067	A	0.4948	0.4442	G	0.08909	1.226
5	rs7724759	96076487	A	0.05155	0.07833	G	0.08911	0.6395
1	rs182169477	19638435	A	0.002577	0.01336	G	0.08916	0.1909
7	rs2243563	6656830	G	0.1856	0.2275	A	0.08918	0.7737
2	rs6717918	233155110	A	0.4897	0.44	G	0.08919	1.221
19	rs4807555	4256919	A	0.4897	0.44	C	0.08919	1.221
1	rs12403266	108185301	C	0.3093	0.2642	G	0.0892	1.247
2	rs1879761	26750769	A	0.3093	0.2642	G	0.0892	1.247
1	rs1131498	169676486	G	0.002577	0.01333	A	0.08921	0.1912
1	rs591650	181384078	C	0.002577	0.01333	A	0.08921	0.1912
2	rs147339619	102625062	G	0.002577	0.01333	A	0.08921	0.1912
3	rs117951912	130346196	A	0.002577	0.01333	G	0.08921	0.1912
4	rs10033029	87685796	C	0.002577	0.01333	A	0.08921	0.1912
4	rs12500797	87692393	A	0.002577	0.01333	G	0.08921	0.1912
5	rs190069099	79026956	A	0.002577	0.01333	G	0.08921	0.1912
5	rs149596574	121488442	A	0.002577	0.01333	G	0.08921	0.1912
6	rs142309377	31130268	A	0.002577	0.01333	G	0.08921	0.1912
10	rs201845881	61819668	C	0.002577	0.01333	G	0.08921	0.1912
11	rs141234111	66392785	G	0.002577	0.01333	A	0.08921	0.1912
12	rs76012715	88440736	A	0.002577	0.01333	G	0.08921	0.1912
15	rs188044698	89392775	A	0.002577	0.01333	G	0.08921	0.1912
16	rs200470984	76509866	A	0.002577	0.01333	G	0.08921	0.1912
19	rs146288460	6147640	G	0.002577	0.01333	A	0.08921	0.1912
8	rs1783174	110539186	A	0.3273	0.3767	G	0.08928	0.8052
6	rs423808	29949123	A	0.2113	0.255	C	0.08929	0.7829
6	rs885948	31140532	A	0.4227	0.4733	G	0.08933	0.8146
8	rs1673407	110460488	A	0.4227	0.4733	C	0.08933	0.8146
9	rs10981899	116478405	C	0.4227	0.4733	A	0.08933	0.8146
1	rs1656022	213527242	G	0.4021	0.3533	A	0.0895	1.231
5	rs4415084	44662515	G	0.5052	0.4542	A	0.08955	1.227
9	rs17611	123769200	G	0.4278	0.4783	A	0.0896	0.8155
20	rs6079395	14327899	A	0.1108	0.1458	G	0.0896	0.73
6	rs2071592	31515340	A	0.5026	0.4525	T	0.08968	1.222
6	rs12055786	153431125	G	0.1443	0.1833	A	0.08977	0.7514
2	rs10933436	233998481	A	0.5077	0.4575	C	0.08983	1.223
11	rs7110779	7657821	C	0.183	0.1458	A	0.08984	1.312

13	rs265654	83726434	A	0.433	0.4833	G	0.08985	0.8163
1	rs77758448	179562792	A	0.1314	0.1	G	0.08987	1.362
4	rs2306949	71339723	A	0.1314	0.1	G	0.08987	1.362
5	rs6892784	112349297	G	0.07216	0.1017	A	0.08987	0.6872
11	rs3017599	61316307	A	0.1314	0.1	G	0.08987	1.362
17	rs2071560	39622385	G	0.1649	0.1292	A	0.08994	1.332
6	rs757259	30115542	A	0.25	0.2083	G	0.08995	1.267
1	rs1553770	176219438	A	0.4356	0.4858	G	0.08996	0.8167
6	rs9264942	31274380	G	0.4356	0.4858	A	0.08996	0.8167
3	rs6445538	52874288	G	0.4021	0.3542	A	0.09	1.226
16	rs444373	66892324	G	0.4021	0.3542	A	0.09	1.226
10	rs8473	129899578	G	0.5155	0.465	A	0.09002	1.224
3	rs2286720	42448471	A	0.2809	0.3283	G	0.09003	0.7992
2	rs1510765	205912403	G	0.4304	0.48	A	0.09006	0.8186
8	rs7009549	70799045	G	0.518	0.4675	A	0.09007	1.224
1	rs4653942	228505204	G	0.4459	0.4967	A	0.09013	0.8155
12	rs1039302	121236258	A	0.2809	0.3275	G	0.09014	0.8022
3	rs2289247	52727257	A	0.5232	0.4725	G	0.09015	1.225
2	rs6543378	106765133	C	0.4407	0.4908	A	0.09017	0.8175
6	rs4432983	96338498	G	0.3325	0.3817	A	0.09022	0.8069
7	rs7802069	24866690	G	0.3325	0.3817	A	0.09022	0.8069
14	rs757572	73132029	G	0.3325	0.3817	A	0.09022	0.8069
16	rs3743979	89180883	G	0.5309	0.48	A	0.09023	1.226
6	rs201995929	168440839	G	0.005155	0.01833	A	0.09026	0.2774
11	rs117135042	57466809	A	0.005155	0.01833	G	0.09026	0.2774
2	rs78190897	84924823	G	0.09278	0.06583	A	0.09028	1.451
4	rs170035	39793856	G	0.4536	0.5042	A	0.09029	0.8165
6	rs3097648	32990970	A	0.05155	0.0325	T	0.09029	1.618
6	rs1074054	113486392	G	0.4536	0.5042	A	0.09029	0.8165
7	rs6464375	153625843	A	0.05155	0.0325	G	0.09029	1.618
18	rs10853323	5036357	A	0.4536	0.5042	G	0.09029	0.8165
7	rs1830035	63726645	G	0.06218	0.04	A	0.09031	1.591
18	rs8085447	57653858	A	0.2036	0.165	G	0.09032	1.294
6	rs396960	32191581	A	0.3325	0.3808	T	0.09034	0.8098
1	rs12742253	85970975	C	0.5129	0.4633	A	0.09036	1.22
11	rs2376558	68851414	A	0.5129	0.4633	G	0.09036	1.22
15	rs28611313	54842584	A	0.2242	0.185	G	0.0904	1.273
22	rs5771069	50435480	A	0.5361	0.4858	G	0.09042	1.223
8	rs10101626	124154697	A	0.2139	0.2575	C	0.09043	0.7847
11	rs4755258	44521492	A	0.4536	0.5033	G	0.0906	0.8192
6	rs379221	29950140	G	0.2139	0.2567	A	0.09066	0.7881
6	rs4711211	30162809	G	0.2526	0.21	A	0.0907	1.271
19	rs35579498	8183871	A	0.1881	0.2292	G	0.09077	0.7795

21	rs2228507	16339570	T	0.1881	0.2292	A	0.09077	0.7795
22	rs7364180	42218856	G	0.3119	0.2675	A	0.09078	1.241
9	rs10980419	113275227	A	0.183	0.1467	G	0.09082	1.303
19	rs34785154	38377773	G	0.4072	0.3592	A	0.09083	1.226
2	rs10177628	17947927	G	0.06186	0.04	A	0.09084	1.582
2	rs57936182	17963098	C	0.06186	0.04	A	0.09084	1.582
5	rs267939	10752315	A	0.1649	0.13	G	0.09084	1.322
11	rs3736508	45975130	G	0.2835	0.33	A	0.09087	0.8034
2	rs16855294	169199695	A	0.3376	0.3867	C	0.09114	0.8085
12	rs79529064	58010132	A	0.03608	0.05917	G	0.09121	0.5952
7	rs17168535	135680562	A	0.4124	0.3633	G	0.09124	1.23
2	rs16866391	179414162	G	0.09794	0.1317	A	0.0913	0.716
11	rs10792436	64219747	G	0.3376	0.3858	A	0.09131	0.8114
1	rs3795737	156697056	G	0.1289	0.1653	A	0.09145	0.7471
12	rs2075260	109696838	G	0.2474	0.2925	A	0.09147	0.7952
11	rs35123761	3681063	A	0.04381	0.06917	G	0.0915	0.6167
14	rs7158731	102792386	G	0.4149	0.3656	A	0.09155	1.231
1	rs6693806	74861767	G	0.1469	0.185	A	0.09157	0.7586
22	rs9625679	29456699	G	0.2165	0.26	A	0.09157	0.7864
12	rs11049125	27916206	A	0.1289	0.165	G	0.09159	0.7486
12	rs11049126	27916224	G	0.1289	0.165	A	0.09159	0.7486
20	rs1000158	36599904	A	0.08505	0.1158	G	0.09163	0.7096
19	rs78161395	14141666	A	0.317	0.2717	C	0.09179	1.244
19	exm-IND19-2371	23927742	D	0.134	0.1017	I	0.09193	1.367
7	rs3815148	106938420	C	0.1856	0.1483	A	0.09195	1.308
16	rs153091	13253956	C	0.1856	0.1483	A	0.09195	1.308
14	rs946918	83472868	A	0.3428	0.3917	C	0.09202	0.8101
7	rs77615410	150883593	A	0.1907	0.2333	G	0.09203	0.7743
12	rs5742692	102799598	G	0.2835	0.24	A	0.09209	1.253
22	rs5762319	28096963	C	0.2887	0.3358	A	0.0921	0.8025
6	rs2244839	31438368	A	0.2513	0.2967	G	0.09222	0.7957
14	rs7145831	45458324	A	0.1649	0.1308	G	0.09228	1.312
2	rs1060384	207041779	G	0.01289	0.02917	A	0.09229	0.4345
2	rs7580332	18540644	G	0.2887	0.335	A	0.0923	0.8055
1	rs28913890	179077415	G	0.01289	0.02833	C	0.09232	0.4477
2	rs142636978	219259458	A	0.01289	0.02833	G	0.09232	0.4477
6	rs4678	30893941	A	0.0232	0.0425	G	0.09233	0.535
6	rs3131787	30899524	G	0.0232	0.0425	A	0.09233	0.535
9	rs35699762	140128644	A	0.07216	0.04833	G	0.09236	1.531
3	rs7615628	98697729	G	0.25	0.295	A	0.09238	0.7966
2	rs840616	188196469	A	0.09278	0.06667	G	0.09242	1.432
16	rs117690401	89293291	A	0.09278	0.06667	G	0.09242	1.432
16	rs118093429	89294798	G	0.09278	0.06667	A	0.09242	1.432

9	rs786990	115416257	A	0.2294	0.1892	G	0.09244	1.276
11	rs158633	30974115	A	0.1057	0.0775	C	0.09249	1.406
16	rs8053136	19767129	C	0.2552	0.2133	A	0.09252	1.263
16	rs7191155	19800213	G	0.2552	0.2133	A	0.09252	1.263
1	rs200612540	983695	A	0.002577	0.01417	G	0.09261	0.1798
2	rs147793515	220431674	A	0.002577	0.01417	G	0.09261	0.1798
4	rs34574349	48088540	A	0.002577	0.01417	G	0.09261	0.1798
10	rs3814166	15255915	A	0.002577	0.01417	G	0.09261	0.1798
11	rs74642146	121495911	A	0.002577	0.01417	G	0.09261	0.1798
13	rs144692979	74420269	A	0.002577	0.01417	G	0.09261	0.1798
14	rs150310098	24976561	G	0.002577	0.01417	C	0.09261	0.1798
19	rs16979912	14910321	G	0.002577	0.01417	A	0.09261	0.1798
19	rs17230134	14910654	G	0.002577	0.01417	A	0.09261	0.1798
19	rs594255	15807984	A	0.002577	0.01417	G	0.09261	0.1798
19	rs11670007	15852363	A	0.002577	0.01417	G	0.09261	0.1798
19	rs199568703	55399719	A	0.002577	0.01417	G	0.09261	0.1798
20	rs151293328	61960994	T	0.002577	0.01417	A	0.09261	0.1798
20	rs200652910	62576034	A	0.002577	0.01417	G	0.09261	0.1798
7	rs2300014	107938242	A	0.2191	0.2633	G	0.09274	0.7848
21	rs13051496	47423509	A	0.09278	0.06678	G	0.09275	1.429
10	rs946185	76423739	A	0.2912	0.3383	G	0.09278	0.8036
17	rs8076604	27438469	G	0.1856	0.1492	A	0.09288	1.3
9	rs79073889	101546410	A	0.04381	0.02667	G	0.09315	1.673
16	rs117953773	72184631	C	0.04381	0.02667	A	0.09315	1.673
17	rs139061476	8736223	A	0.04381	0.02667	G	0.09315	1.673
12	rs2245199	123145864	A	0.3479	0.3958	G	0.09318	0.8144
17	rs6503319	10574159	A	0.3479	0.3958	G	0.09318	0.8144
1	rs12039961	203276546	A	0.0232	0.04333	G	0.09321	0.5243
5	rs6875902	148407893	A	0.0232	0.04333	C	0.09321	0.5243
4	rs448469	139274999	G	0.3196	0.275	A	0.09325	1.238
11	rs297325	16389594	G	0.3196	0.275	A	0.09325	1.238
2	rs7881	75196731	G	0.1933	0.2358	A	0.09334	0.7764
10	rs11187837	96035980	G	0.1701	0.21	A	0.09342	0.7711
5	rs12658202	152979554	A	0.2938	0.34	C	0.09371	0.8076
12	rs7136356	122689181	G	0.2938	0.34	C	0.09371	0.8076
2	rs1561198	85809989	A	0.3531	0.4017	G	0.09372	0.8131
4	rs11932413	56977309	G	0.3531	0.4017	A	0.09372	0.8131
9	rs7022816	1519043	G	0.366	0.3192	A	0.09382	1.231
21	rs469390	42817930	A	0.2088	0.17	G	0.09392	1.288
6	rs3871466	30983683	G	0.232	0.1917	A	0.09396	1.274
6	rs3869096	30984404	C	0.232	0.1917	A	0.09396	1.274
10	rs36121140	27458898	A	0.07474	0.105	C	0.09396	0.6886
6	rs6929796	31522669	A	0.4253	0.3775	G	0.09404	1.22

19	rs335751	30641424	G	0.4253	0.3775	A	0.09404	1.22
15	rs11855415	101875123	T	0.1881	0.1508	A	0.09405	1.305
15	rs2297377	41815649	A	0.3686	0.3208	G	0.09411	1.236
19	rs16980994	16060117	A	0.1005	0.1333	C	0.09414	0.7264
19	rs16980822	16060248	G	0.1005	0.1333	A	0.09414	0.7264
19	rs11880184	16060658	G	0.1005	0.1333	A	0.09414	0.7264
4	rs149571949	41673609	A	0.01804	0.03583	G	0.09415	0.4944
6	rs144847588	31085286	A	0.01804	0.03583	G	0.09415	0.4944
10	rs35349706	78084184	A	0.01804	0.03583	G	0.09415	0.4944
17	rs75655436	5041471	A	0.01804	0.03583	C	0.09415	0.4944
7	rs17165906	12409263	A	0.2887	0.245	C	0.09419	1.251
7	rs17165910	12409327	A	0.2887	0.245	G	0.09419	1.251
7	rs3823844	12409522	G	0.2887	0.245	A	0.09419	1.251
5	rs30489	133481467	A	0.4304	0.3817	G	0.09423	1.224
11	rs11040249	6023640	G	0.05412	0.08	A	0.09433	0.658
12	rs117039796	13211471	A	0.05412	0.08	G	0.09433	0.658
7	rs2714410	11272355	A	0.05412	0.08083	G	0.0944	0.6507
12	rs35767	102875569	A	0.3686	0.3217	G	0.09442	1.231
8	rs6577727	137419150	A	0.433	0.3842	G	0.09457	1.224
4	rs4345220	41639516	G	0.2577	0.2167	A	0.09463	1.255
4	rs3775296	186997767	A	0.2577	0.2167	C	0.09463	1.255
6	rs2306916	152647681	T	0.116	0.1508	A	0.09465	0.7386
4	rs13441	10099340	A	0.4304	0.3825	G	0.09469	1.22
16	rs11648609	1616201	A	0.08763	0.1192	G	0.09481	0.7099
8	rs4144738	130760850	A	0.3247	0.28	G	0.09486	1.237
16	rs383362	79245820	A	0.116	0.1517	C	0.09487	0.7338
1	rs11166240	99871756	G	0.2242	0.2675	A	0.09492	0.7915
2	rs3739097	27360534	A	0.06443	0.0925	G	0.09493	0.6757
15	rs8042919	50878630	A	0.06443	0.0925	G	0.09493	0.6757
20	rs3918392	3655219	G	0.06443	0.0925	A	0.09493	0.6757
19	rs12150963	8160897	G	0.2577	0.3033	C	0.09495	0.7975
10	rs6583826	94347830	G	0.1727	0.2125	A	0.09501	0.7735
9	rs1929539	79582862	C	0.3031	0.35	A	0.0952	0.8078
12	rs2229840	124826462	A	0.08763	0.12	G	0.09522	0.7043
19	rs79297344	38795554	G	0.08763	0.12	A	0.09522	0.7043
17	rs4605213	49244747	G	0.2912	0.2475	C	0.09523	1.249
17	rs6607302	36194230	G	0.1521	0.1908	A	0.09529	0.7604
1	rs1507765	207535246	A	0.3634	0.4117	C	0.09532	0.8158
6	rs1219031	94635123	A	0.3634	0.4117	G	0.09532	0.8158
20	rs6047116	20848968	G	0.3634	0.4117	A	0.09532	0.8158
10	rs650019	79215213	A	0.3015	0.3492	G	0.09535	0.8047
4	rs1799883	120241902	A	0.3015	0.3483	G	0.09541	0.8077
8	rs7015855	13660787	A	0.366	0.415	C	0.09549	0.8137

6	rs1635	28227604	A	0.3737	0.3267	C	0.0956	1.23
6	rs9275524	32675109	A	0.3711	0.325	G	0.0956	1.226
6	rs6932517	32678182	C	0.3711	0.325	G	0.0956	1.226
6	rs9275572	32678999	A	0.3711	0.325	G	0.0956	1.226
1	rs9658588	86048659	A	0.03093	0.01667	G	0.0957	1.883
1	rs484959	110366083	G	0.2113	0.1725	A	0.0957	1.285
3	rs141123012	152058344	G	0.03093	0.01667	A	0.0957	1.883
5	rs149445760	54404085	A	0.03093	0.01667	G	0.0957	1.883
7	rs7781330	30270403	A	0.03093	0.01667	G	0.0957	1.883
8	rs114664885	63942733	G	0.03093	0.01667	A	0.0957	1.883
11	rs75034975	65632242	A	0.03093	0.01667	G	0.0957	1.883
8	rs28705715	39141120	A	0.01804	0.03667	C	0.09574	0.4827
9	rs117582746	139008606	A	0.01804	0.03667	G	0.09574	0.4827
15	rs7167216	91354521	A	0.01804	0.03667	G	0.09574	0.4827
16	rs78964247	71220794	A	0.134	0.1708	C	0.09582	0.7512
16	rs4356470	58529615	G	0.3763	0.3286	A	0.09587	1.233
2	rs1207421	206150594	A	0.2912	0.2483	G	0.09591	1.244
6	rs241404	32865997	G	0.4407	0.3925	A	0.09592	1.22
6	rs241402	32869045	G	0.4407	0.3925	A	0.09592	1.22
6	rs241398	32875450	A	0.4407	0.3925	G	0.09592	1.22
19	rs2285419	36134208	A	0.134	0.17	G	0.096	0.7556
10	rs2295934	84738707	C	0.08247	0.05833	A	0.09604	1.451
1	rs857725	158607935	C	0.3686	0.4167	A	0.09608	0.8171
1	rs857721	158612548	A	0.3686	0.4167	T	0.09608	0.8171
1	rs6664445	32280610	A	0.3737	0.3275	G	0.09615	1.225
19	rs10451497	15203866	A	0.3737	0.3275	G	0.09615	1.225
11	rs1425801	35099975	C	0.3711	0.42	A	0.09618	0.815
4	rs12506517	102642526	G	0.4459	0.3967	A	0.0962	1.224
3	rs2241314	10302056	A	0.2938	0.25	G	0.09626	1.248
14	rs17111394	81523128	G	0.2345	0.195	A	0.09631	1.265
12	rs144092294	56364973	A	0.007772	0.001667	G	0.09633	4.692
16	rs147472574	10996577	A	0.007772	0.001667	G	0.09633	4.692
1	rs192115090	216172258	G	0.02577	0.0125	C	0.09649	2.09
4	rs115046131	89671591	C	0.02577	0.0125	A	0.09649	2.09
6	rs1233493	29458241	G	0.02577	0.0125	A	0.09649	2.09
8	rs78001852	38912091	A	0.02577	0.0125	G	0.09649	2.09
17	rs79976834	10608307	A	0.02577	0.0125	G	0.09649	2.09
17	rs79737146	10628364	A	0.02577	0.0125	G	0.09649	2.09
3	rs6768408	150609866	A	0.3737	0.4225	G	0.09652	0.8156
5	rs10464032	98451600	C	0.2113	0.1733	A	0.09656	1.278
4	rs6836994	169083694	C	0.1753	0.215	A	0.09659	0.7759
8	rs148628207	95800280	A	0.007812	0.001678	G	0.0966	4.685
8	rs141149968	110587910	C	0.007772	0.001669	G	0.09664	4.684

7	rs2171492	129950740	A	0.3067	0.3533	C	0.09668	0.8096
7	rs38855	116358044	A	0.451	0.4017	G	0.09681	1.224
17	rs4968849	67178316	G	0.3737	0.4217	A	0.09681	0.8184
21	rs2832077	30141021	A	0.451	0.4017	G	0.09681	1.224
6	rs2227956	31778272	G	0.1082	0.08	A	0.09683	1.396
5	rs2652098	82808072	A	0.1031	0.1361	G	0.09694	0.7298
7	rs975263	20198440	G	0.1701	0.1358	A	0.09696	1.304
8	rs2458413	105359432	A	0.2294	0.2733	G	0.09702	0.7913
2	rs34505188	228128568	A	0.1031	0.1358	G	0.09708	0.7313
20	rs41418351	23064912	G	0.1031	0.1358	A	0.09708	0.7313
6	rs4959788	3264526	G	0.4536	0.4042	A	0.0971	1.224
19	rs4807298	2565877	G	0.3109	0.3575	A	0.09711	0.8108
12	rs2579028	30707190	A	0.3789	0.3325	G	0.09724	1.225
19	rs3898466	54414637	G	0.201	0.2425	A	0.09726	0.786
1	rs200236217	6293674	A	0.007732	0.001667	T	0.0973	4.668
1	rs74990705	53347210	G	0.007732	0.001667	A	0.0973	4.668
1	rs145376350	112308413	G	0.007732	0.001667	A	0.0973	4.668
1	rs145426547	192128438	A	0.007732	0.001667	G	0.0973	4.668
1	rs201988893	222757615	G	0.007732	0.001667	A	0.0973	4.668
1	rs6669585	245848798	A	0.007732	0.001667	G	0.0973	4.668
2	rs148257995	67631001	G	0.007732	0.001667	A	0.0973	4.668
2	rs199752013	141771224	G	0.007732	0.001667	A	0.0973	4.668
2	rs151034735	170386346	G	0.007732	0.001667	A	0.0973	4.668
2	rs55754655	201526330	G	0.007732	0.001667	A	0.0973	4.668
2	rs201246207	204000661	C	0.007732	0.001667	A	0.0973	4.668
3	rs199549516	13383282	A	0.007732	0.001667	C	0.0973	4.668
3	rs200514916	43389910	A	0.007732	0.001667	G	0.0973	4.668
3	rs201270296	52786165	T	0.007732	0.001667	A	0.0973	4.668
3	rs9868484	108188993	A	0.007732	0.001667	G	0.0973	4.668
3	rs142475278	122345704	A	0.007732	0.001667	G	0.0973	4.668
3	rs186946267	183960671	A	0.007732	0.001667	G	0.0973	4.668
4	rs115630927	103970116	A	0.007732	0.001667	G	0.0973	4.668
5	rs2032892	36677083	C	0.007732	0.001667	G	0.0973	4.668
6	rs116048674	31842531	G	0.007732	0.001667	C	0.0973	4.668
6	rs114957103	31842535	G	0.007732	0.001667	A	0.0973	4.668
6	rs75616932	34802160	G	0.007732	0.001667	A	0.0973	4.668
6	rs151000241	90042914	G	0.007732	0.001667	A	0.0973	4.668
6	rs74325037	107475829	C	0.007732	0.001667	A	0.0973	4.668
6	rs114642748	119252836	A	0.007732	0.001667	G	0.0973	4.668
7	rs187745069	56150988	A	0.007732	0.001667	G	0.0973	4.668
7	rs200733700	110764837	A	0.007732	0.001667	C	0.0973	4.668
7	rs146915016	127014654	A	0.007732	0.001667	G	0.0973	4.668
7	rs201852656	133580445	A	0.007732	0.001667	G	0.0973	4.668

8	rs78400740	39613406	G	0.007732	0.001667	A	0.0973	4.668
8	rs111349599	39695662	A	0.007732	0.001667	G	0.0973	4.668
8	rs143130949	94745597	C	0.007732	0.001667	G	0.0973	4.668
8	rs201776530	139815167	A	0.007732	0.001667	G	0.0973	4.668
9	rs200120504	104449017	A	0.007732	0.001667	G	0.0973	4.668
9	rs146869947	134388669	A	0.007732	0.001667	G	0.0973	4.668
9	rs34024143	136287582	A	0.007732	0.001667	G	0.0973	4.668
10	rs17113412	90588419	G	0.007732	0.001667	A	0.0973	4.668
10	rs141678498	104233495	A	0.007732	0.001667	G	0.0973	4.668
10	rs143409948	113933502	A	0.007732	0.001667	C	0.0973	4.668
11	rs56006697	61081819	G	0.007732	0.001667	A	0.0973	4.668
11	rs607969	64575505	A	0.007732	0.001667	G	0.0973	4.668
11	rs117897496	68748425	A	0.007732	0.001667	G	0.0973	4.668
11	rs199893083	76831801	G	0.007732	0.001667	A	0.0973	4.668
11	rs3812723	78574076	A	0.007732	0.001667	G	0.0973	4.668
11	rs201256460	86662209	A	0.007732	0.001667	G	0.0973	4.668
12	rs201545489	26277654	G	0.007732	0.001667	A	0.0973	4.668
12	rs112334098	55759499	G	0.007732	0.001667	A	0.0973	4.668
13	rs200666130	50279776	A	0.007732	0.001667	T	0.0973	4.668
14	rs17099455	24423007	A	0.007732	0.001667	G	0.0973	4.668
14	rs146717881	64701814	A	0.007732	0.001667	G	0.0973	4.668
14	rs117173425	77751332	G	0.007732	0.001667	A	0.0973	4.668
14	rs45527334	77844265	A	0.007732	0.001667	G	0.0973	4.668
14	rs200769097	91806262	A	0.007732	0.001667	G	0.0973	4.668
15	rs199884674	32928808	A	0.007732	0.001667	G	0.0973	4.668
15	rs34317102	63414083	A	0.007732	0.001667	C	0.0973	4.668
15	rs143695562	66855936	G	0.007732	0.001667	A	0.0973	4.668
15	rs1867219	91503871	A	0.007732	0.001667	C	0.0973	4.668
15	rs8042680	91521337	C	0.007732	0.001667	A	0.0973	4.668
16	rs184493261	836828	A	0.007732	0.001667	G	0.0973	4.668
16	rs34297640	1841499	A	0.007732	0.001667	G	0.0973	4.668
16	rs77254040	3705465	C	0.007732	0.001667	G	0.0973	4.668
16	rs13336804	11001694	G	0.007732	0.001667	A	0.0973	4.668
16	rs182336923	55361523	C	0.007732	0.001667	G	0.0973	4.668
16	rs117141770	67864395	A	0.007732	0.001667	G	0.0973	4.668
16	rs2244899	83984844	A	0.007732	0.001667	C	0.0973	4.668
16	rs2244898	83984850	G	0.007732	0.001667	C	0.0973	4.668
16	rs200076111	88780846	A	0.007732	0.001667	G	0.0973	4.668
17	rs150826013	62228250	G	0.007732	0.001667	A	0.0973	4.668
17	rs201944918	67299017	G	0.007732	0.001667	A	0.0973	4.668
17	rs199888749	67305519	A	0.007732	0.001667	G	0.0973	4.668
18	rs148607670	2925272	A	0.007732	0.001667	G	0.0973	4.668
18	rs2305370	10550121	G	0.007732	0.001667	C	0.0973	4.668

19	rs186519486	9084614	A	0.007732	0.001667	C	0.0973	4.668
19	rs201332435	46273846	T	0.007732	0.001667	A	0.0973	4.668
22	rs199634127	20786125	A	0.007732	0.001667	G	0.0973	4.668
11	rs2957866	3743046	A	0.3093	0.3558	G	0.09731	0.8106
11	exm2249989	55681999	A	0.09536	0.06917	G	0.09745	1.419
11	rs75634103	55681999	A	0.09536	0.06917	G	0.09745	1.419
6	rs45620832	52761651	A	0.03093	0.05175	G	0.09747	0.5848
19	rs10755	40199914	G	0.3789	0.4267	C	0.09752	0.8196
9	rs2292923	139890130	A	0.03093	0.05167	C	0.09759	0.5858
16	rs77170706	88787727	A	0.03093	0.05167	G	0.09759	0.5858
16	rs78574543	88787781	A	0.03093	0.05167	G	0.09759	0.5858
2	rs199500215	183621059	C	0.007732	0.001669	G	0.09761	4.66
5	rs150464684	76734132	A	0.007732	0.001669	G	0.09761	4.66
4	rs3899584	65282211	A	0.1546	0.1208	C	0.09762	1.331
15	rs16943741	86278479	A	0.384	0.4333	G	0.09767	0.8153
3	rs868768	141377532	G	0.2655	0.31	A	0.09772	0.8044
11	rs76779571	94040636	C	0.03093	0.0525	A	0.09775	0.576
12	rs12829856	44191625	A	0.03093	0.0525	G	0.09775	0.576
9	rs879369	100995721	G	0.2371	0.1975	A	0.09776	1.263
20	rs4809324	62318220	G	0.07732	0.1067	A	0.09777	0.7018
3	rs74762169	46492149	A	0.01546	0.005833	G	0.0978	2.677
3	rs3732530	47618953	C	0.384	0.4325	A	0.0978	0.818
3	rs34379671	49737660	A	0.01546	0.005833	G	0.0978	2.677
3	rs114545672	182871776	A	0.01546	0.005833	G	0.0978	2.677
6	rs150345955	31623834	G	0.01546	0.005833	A	0.0978	2.677
6	rs3734559	40359744	A	0.01546	0.005833	G	0.0978	2.677
6	rs10943299	76640803	A	0.01546	0.005833	G	0.0978	2.677
6	rs142951494	151673179	T	0.01546	0.005833	A	0.0978	2.677
11	rs150191942	102464198	A	0.01546	0.005833	G	0.0978	2.677
12	rs2304459	31446744	A	0.01546	0.005833	G	0.0978	2.677
17	rs11653832	5424906	G	0.01546	0.005833	C	0.0978	2.677
19	rs200344400	1244170	G	0.01546	0.005833	A	0.0978	2.677
19	rs77403452	1818484	A	0.01546	0.005833	G	0.0978	2.677
19	rs34264048	39421274	G	0.01546	0.005833	A	0.0978	2.677
19	rs147007310	39423053	G	0.01546	0.005833	C	0.0978	2.677
10	rs4750316	6393260	C	0.07732	0.1075	G	0.09781	0.6957
1	rs860554	201262432	A	0.1366	0.1733	G	0.09795	0.7545
10	rs10822204	65517009	G	0.3866	0.4358	A	0.09795	0.8158
4	rs11726613	101108057	C	0.3866	0.435	A	0.0981	0.8186
16	rs61737709	19475099	A	0.1778	0.2175	C	0.09817	0.7782
6	rs9466453	22735375	A	0.4588	0.4108	G	0.09826	1.216
18	rs1367345	71482490	A	0.4588	0.4108	G	0.09826	1.216
3	rs1523911	812421	G	0.384	0.3375	A	0.0983	1.224

17	rs201455361	4575433	A	0.01546	0.005853	C	0.0983	2.668
21	rs9622	33951068	G	0.268	0.3133	A	0.09831	0.8025
11	rs10839708	7079038	G	0.3376	0.2917	A	0.09837	1.238
7	rs10228313	29575822	A	0.3892	0.4375	G	0.09839	0.8192
4	rs4260585	190008681	A	0.2036	0.2458	G	0.09849	0.7843
5	rs4869419	92567539	G	0.09021	0.1225	A	0.09849	0.7102
4	rs7658300	48115264	A	0.07216	0.05	G	0.09852	1.478
13	rs144848	32906729	C	0.2036	0.245	A	0.09858	0.7879
2	rs2287059	10717806	A	0.1237	0.09333	G	0.0986	1.371
5	rs80241453	160034042	A	0.1237	0.09333	G	0.0986	1.371
9	rs41304943	107299001	A	0.03866	0.06167	C	0.09862	0.6119
12	rs12819160	27850113	T	0.03866	0.06167	A	0.09862	0.6119
16	rs17736370	84256619	A	0.03866	0.06167	G	0.09862	0.6119
16	rs35159887	88787673	A	0.03866	0.06167	G	0.09862	0.6119
17	rs444207	6982444	G	0.3943	0.4432	A	0.09868	0.8178
10	rs7068966	12277992	A	0.3943	0.4433	G	0.09873	0.8175
20	rs17757442	23421101	A	0.299	0.2558	G	0.09886	1.241
6	rs346291	80564836	A	0.1572	0.1953	G	0.09888	0.7685
6	rs3094562	29388306	G	0.4665	0.4183	A	0.0989	1.216
6	rs3189152	32634341	G	0.317	0.3642	A	0.0989	0.8104
7	rs17151293	51973256	G	0.4665	0.4183	A	0.0989	1.216
14	rs10139194	104704722	G	0.3943	0.4425	A	0.09897	0.8203
5	rs958912	160097496	A	0.1082	0.08083	G	0.09906	1.38
11	rs11036815	5344681	A	0.1572	0.195	G	0.09906	0.7701
2	rs1075622	86337930	A	0.2706	0.3167	G	0.09916	0.8006
7	rs4269485	52544356	G	0.4742	0.425	A	0.09921	1.22
10	rs830066	111028887	C	0.4742	0.425	A	0.09921	1.22
6	rs4714261	39539207	A	0.06443	0.0425	G	0.09943	1.552
9	rs10511974	72520270	G	0.4021	0.4508	A	0.09946	0.8191
17	rs4986764	59763347	A	0.268	0.2267	G	0.09947	1.249
22	rs2235171	32650200	A	0.04639	0.02833	G	0.09949	1.668
2	rs1868024	223559089	G	0.1392	0.1075	A	0.0995	1.342
3	rs3732756	150916327	G	0.1392	0.1075	A	0.0995	1.342
11	rs15818	118952173	G	0.1392	0.1075	A	0.0995	1.342
11	rs3740909	125889526	A	0.1392	0.1075	G	0.0995	1.342
6	rs805294	31688217	G	0.3995	0.4475	A	0.09952	0.8213
3	rs6599079	38271881	A	0.03866	0.0625	G	0.09958	0.6032
2	rs6716455	151126403	A	0.1804	0.2208	G	0.09962	0.7767
6	rs7764934	30434566	G	0.4794	0.43	A	0.09962	1.221
3	rs9866141	156950579	A	0.0567	0.08333	G	0.09965	0.6612
6	rs3094225	31113052	G	0.4046	0.4533	A	0.09969	0.8196
10	rs2795918	56177015	A	0.0567	0.0825	G	0.09971	0.6685
1	rs11799990	4541390	C	0.1211	0.1567	A	0.09974	0.7419

6	rs6912077	30739904	A	0.1211	0.1567	G	0.09974	0.7419
11	rs2229456	66328741	C	0.1211	0.1567	A	0.09974	0.7419
5	rs4626335	125897411	A	0.2732	0.3183	G	0.09977	0.8052
16	rs7500034	88039150	A	0.4072	0.4567	G	0.09982	0.8173
2	rs3816183	43015719	A	0.2423	0.2017	G	0.09992	1.266
2	rs10928938	130665114	A	0.3943	0.3467	G	0.1	1.227
4	rs11939157	98228716	G	0.4098	0.4592	A	0.1	0.8178
5	rs10071490	113940187	A	0.482	0.4333	G	0.1	1.217
8	rs2003045	143906396	A	0.3943	0.3467	G	0.1	1.227
12	rs1818930	85391419	C	0.482	0.4333	A	0.1	1.217
22	rs2071762	45258324	A	0.3222	0.3692	G	0.1	0.8122
6	rs1126504	33048457	C	0.1392	0.1758	G	0.1001	0.7578
2	rs10169617	68540378	A	0.3402	0.2958	G	0.1002	1.227
2	rs1582345	171565376	A	0.3428	0.2975	G	0.1002	1.232
3	rs4285007	20772716	G	0.4845	0.4358	A	0.1002	1.217
16	rs11546303	14742400	C	0.1933	0.1575	A	0.1002	1.282
1	rs6662382	158368846	G	0.2371	0.28	A	0.1003	0.7992
2	rs1401386	224713026	C	0.4072	0.455	A	0.1003	0.8228
3	rs3733055	42577733	A	0.03866	0.0225	C	0.1003	1.747
3	rs75414918	193332522	A	0.03866	0.0225	C	0.1003	1.747
6	rs805287	31678730	G	0.4124	0.4608	A	0.1003	0.821
8	rs55793951	48719852	G	0.03866	0.0225	C	0.1003	1.747
9	rs534214	128369224	G	0.3222	0.3683	A	0.1003	0.8151
12	rs3825199	93976954	G	0.3222	0.3683	A	0.1003	0.8151
12	rs77695791	120601014	A	0.03866	0.0225	G	0.1003	1.747
14	rs758109	73958375	A	0.1392	0.1767	G	0.1003	0.7535
15	rs2061822	86122779	A	0.4871	0.4383	G	0.1003	1.217
19	rs243383	4442999	A	0.03866	0.0225	C	0.1003	1.747
3	rs6806444	57475351	A	0.4897	0.4408	C	0.1005	1.217
13	rs1185801	39979675	A	0.4175	0.4667	G	0.1005	0.8192
4	rs2132271	81433856	A	0.3247	0.3717	G	0.1006	0.813
7	rs6967241	12419108	A	0.3247	0.3717	T	0.1006	0.813
9	rs10815798	8235633	G	0.4845	0.4367	A	0.1006	1.213
15	rs7162168	86124946	A	0.4923	0.4433	G	0.1006	1.217
17	rs8076167	69233083	A	0.09794	0.07083	G	0.1006	1.424
14	rs1048755	92548785	A	0.4201	0.4692	G	0.1007	0.8197
10	rs2805910	70856852	G	0.4897	0.4417	A	0.1008	1.213
19	rs7248171	31863085	G	0.3969	0.35	A	0.1008	1.222
6	rs9455313	71770335	A	0.4227	0.4717	G	0.1009	0.8201
7	rs61732005	121653250	A	0.1598	0.1975	G	0.1009	0.7728
10	rs11016076	129911856	C	0.3428	0.2983	G	0.1009	1.227
1	rs10917234	22782602	G	0.4227	0.4708	A	0.1011	0.8229
6	rs2517673	29937242	A	0.183	0.2233	G	0.1011	0.7789

19	rs12978266	11358799	A	0.5155	0.4667	G	0.1011	1.216
19	rs1477334	56003777	G	0.3273	0.3742	A	0.1011	0.8139
17	rs2302189	56584205	A	0.4278	0.4767	C	0.1012	0.821
1	rs2886220	203309752	G	0.3995	0.3525	A	0.1013	1.222
6	rs7763726	159670100	G	0.3067	0.2625	A	0.1013	1.243
12	rs2102294	116991459	A	0.3067	0.2625	G	0.1013	1.243
16	rs187679	52522717	A	0.4253	0.4733	G	0.1013	0.8233
17	rs4986168	43336574	G	0.4304	0.4792	A	0.1013	0.8214
1	rs12754818	79392756	C	0.2397	0.2825	A	0.1014	0.8007
5	rs1320308	76171252	A	0.5052	0.4567	C	0.1014	1.215
6	rs172562	85473758	A	0.433	0.4817	G	0.1014	0.8218
3	rs4855980	76472055	A	0.4278	0.4758	G	0.1015	0.8237
6	rs763026	32691745	A	0.1572	0.1242	G	0.1015	1.316
3	rs4681982	57414071	A	0.4304	0.4783	G	0.1016	0.8242
6	rs9491689	127398595	A	0.4356	0.4842	C	0.1016	0.8222
9	rs1158810	121809519	G	0.5155	0.4666	A	0.1016	1.216
9	rs497632	130475442	C	0.3454	0.3008	A	0.1016	1.226
16	rs2072273	2871452	A	0.0799	0.11	G	0.1016	0.7026
17	rs2058194	62020348	G	0.4407	0.49	A	0.1016	0.8202
18	rs4941232	55654318	G	0.4407	0.49	A	0.1016	0.8202
20	rs1555668	2847377	A	0.0799	0.11	G	0.1016	0.7026
1	rs786906	89271574	G	0.5335	0.4841	A	0.1017	1.219
2	rs3112998	95822447	G	0.5155	0.4667	A	0.1017	1.216
3	rs1357432	156157738	G	0.4381	0.4867	A	0.1017	0.8225
4	rs13150573	173872313	G	0.5155	0.4667	A	0.1017	1.216
6	rs3827784	6642405	G	0.4433	0.4925	A	0.1017	0.8205
9	rs10961780	14898161	C	0.3479	0.3025	A	0.1017	1.23
3	rs1866268	52719398	A	0.5232	0.4742	C	0.1018	1.217
3	rs6617	52740182	G	0.5232	0.4742	C	0.1018	1.217
3	rs1029871	52797634	G	0.5232	0.4742	C	0.1018	1.217
3	rs975121	191897218	G	0.07474	0.05167	A	0.1018	1.483
5	rs79684971	7826853	A	0.07474	0.05167	G	0.1018	1.483
7	rs60805935	21663873	G	0.5206	0.4717	A	0.1018	1.216
9	rs11793637	96415899	G	0.4459	0.495	A	0.1018	0.8209
10	rs7904413	17615647	A	0.4459	0.495	G	0.1018	0.8209
10	rs75028145	116014769	A	0.07474	0.05167	T	0.1018	1.483
11	rs12049869	127718228	G	0.5206	0.4717	A	0.1018	1.216
15	rs12900552	23077857	G	0.4021	0.355	A	0.1018	1.222
17	rs11549830	1690752	G	0.433	0.4808	A	0.1018	0.8246
19	rs10853751	41903220	A	0.4459	0.495	G	0.1018	0.8209
20	rs2093066	31652596	A	0.07474	0.05167	G	0.1018	1.483
20	rs378098	31660543	A	0.07474	0.05167	G	0.1018	1.483
21	rs2825303	20407698	G	0.4485	0.4975	A	0.1018	0.8213

1	rs1165222	55638075	G	0.02062	0.009167	A	0.1019	2.276
2	rs3816281	68607947	C	0.3995	0.3533	A	0.1019	1.218
3	rs143310118	42784452	A	0.02062	0.009167	G	0.1019	2.276
4	rs55755438	39448171	G	0.02062	0.009167	A	0.1019	2.276
5	rs28421695	167881032	T	0.5361	0.4867	A	0.1019	1.219
6	rs61736079	27276861	G	0.02062	0.009167	A	0.1019	2.276
6	rs2746149	29435355	G	0.02062	0.009167	A	0.1019	2.276
6	rs61730667	29455355	A	0.02062	0.009167	T	0.1019	2.276
6	rs148828689	30593528	A	0.02062	0.009167	G	0.1019	2.276
7	rs1554699	144672571	A	0.5309	0.4817	G	0.1019	1.218
8	rs2228979	26481697	A	0.1033	0.075	G	0.1019	1.42
14	rs61976859	31583512	A	0.02062	0.009167	G	0.1019	2.276
16	rs181009434	48212531	A	0.02062	0.009167	G	0.1019	2.276
4	rs13148469	183272312	G	0.4562	0.505	A	0.102	0.8223
5	rs9314177	96028860	G	0.4356	0.4833	A	0.102	0.8249
6	rs9294391	88534879	A	0.5129	0.465	C	0.102	1.211
8	rs754957	143746416	A	0.5361	0.4875	G	0.102	1.215
9	rs10814597	37685362	A	0.4356	0.4833	G	0.102	0.8249
14	rs31434	79416899	G	0.4459	0.4942	A	0.102	0.8236
1	rs3795331	159802683	A	0.1237	0.1592	G	0.1021	0.7458
2	rs399885	79687252	A	0.5258	0.4775	C	0.1021	1.213
3	rs3732895	100712249	A	0.5232	0.475	G	0.1021	1.213
4	rs1980057	145485738	A	0.2824	0.3275	G	0.1021	0.808
6	rs4714910	46232325	A	0.5309	0.4825	G	0.1021	1.214
8	rs3735999	143746069	A	0.5361	0.4875	G	0.1021	1.215
9	rs10756267	11711459	A	0.5155	0.4675	G	0.1021	1.212
12	rs7131744	4496280	A	0.5361	0.4875	G	0.1021	1.215
16	rs7764	22345191	G	0.5155	0.4675	A	0.1021	1.212
18	rs4799088	77573548	A	0.5361	0.4875	G	0.1021	1.215
12	rs2160519	13787846	G	0.2113	0.2529	A	0.1022	0.7915
12	rs3764002	108618630	A	0.451	0.4992	G	0.1022	0.8243
3	rs9853906	97983942	A	0.3505	0.3047	G	0.1023	1.232
10	rs2185913	115410234	G	0.2809	0.3258	A	0.1023	0.8083
1	rs1126407	236700807	T	0.4588	0.5067	A	0.1024	0.8253
1	rs1041935	236700857	A	0.4588	0.5067	G	0.1024	0.8253
5	rs58002603	64764307	G	0.1624	0.2008	A	0.1025	0.7714
17	rs3744332	15341332	A	0.06443	0.04333	G	0.1026	1.52
21	rs2839110	47538960	G	0.06443	0.04333	A	0.1026	1.52
3	rs2290610	3139957	G	0.3351	0.3817	A	0.1027	0.8163
4	rs10518377	121648617	G	0.4046	0.3583	A	0.1027	1.217
10	rs2749647	38549612	A	0.4072	0.36	G	0.1027	1.221
11	rs79004948	55927763	A	0.1082	0.1417	G	0.1027	0.7355
2	rs7596044	167724626	G	0.1598	0.1258	A	0.1031	1.321

4	rs13147758	145460230	G	0.2835	0.3283	A	0.1031	0.8094
2	rs3731594	227660046	A	0.02577	0.045	G	0.1032	0.5614
2	rs78832829	239162038	G	0.02577	0.045	A	0.1032	0.5614
7	rs35827364	73513529	G	0.02577	0.045	C	0.1032	0.5614
17	rs2429387	60767015	G	0.02577	0.045	A	0.1032	0.5614
19	rs10404119	14991963	C	0.4072	0.3608	A	0.1032	1.217
5	rs7715100	149763305	G	0.04897	0.07333	A	0.1034	0.6507
15	rs678892	55632859	C	0.3093	0.2667	A	0.1034	1.231
17	rs8069166	293138	A	0.04897	0.07333	G	0.1034	0.6507
11	rs11220462	126243952	A	0.2876	0.3333	G	0.1035	0.8073
12	rs7300145	80062156	G	0.2139	0.2558	A	0.1035	0.7916
4	rs1051447	49063872	A	0.4098	0.3633	C	0.1036	1.217
7	rs1799999	113518434	C	0.3119	0.2683	A	0.1036	1.236
1	rs117872916	33236845	A	0.1263	0.09667	G	0.1038	1.351
1	rs2745967	208128722	G	0.02577	0.04583	A	0.1038	0.5507
19	rs56248314	17566634	T	0.1263	0.09667	A	0.1038	1.351
19	rs3745725	48967588	A	0.02577	0.04583	G	0.1038	0.5507
20	rs73894084	5903563	A	0.02577	0.04583	G	0.1038	0.5507
17	rs12450550	48456193	G	0.06959	0.0975	A	0.1039	0.6923
8	rs2669425	105395583	G	0.1881	0.2283	A	0.1041	0.7832
17	rs739579	59661552	A	0.4149	0.3675	G	0.1041	1.221
16	rs35259348	72003952	G	0.2474	0.2908	C	0.1042	0.8017
1	rs1436900	217058479	A	0.25	0.2092	G	0.1043	1.26
3	rs1586861	139058496	A	0.1418	0.1108	G	0.1043	1.325
6	rs1265100	31105310	G	0.3119	0.2692	A	0.1043	1.23
19	rs75008691	7809071	A	0.01036	0.003333	G	0.1043	3.131
12	rs4135113	104376693	A	0.1649	0.2042	G	0.1044	0.77
1	rs1124777	204438334	G	0.2216	0.1842	A	0.1045	1.261
4	rs9306981	33762750	A	0.3454	0.3925	G	0.1045	0.8165
4	exm2241199	69434042	A	0.1263	0.1617	G	0.1045	0.7495
4	rs1459157	173739244	G	0.3582	0.3125	A	0.1046	1.228
1	rs12036569	90180529	A	0.01031	0.02417	G	0.1047	0.4206
3	rs9831894	121800487	C	0.2165	0.2583	A	0.1047	0.7933
6	rs6911724	30025179	A	0.2165	0.2583	G	0.1047	0.7933
6	rs7770557	30029109	G	0.2165	0.2583	C	0.1047	0.7933
7	rs56921891	149497421	A	0.01031	0.02417	G	0.1047	0.4206
11	rs145062279	73879583	A	0.01031	0.02417	C	0.1047	0.4206
15	rs78309240	51778412	A	0.01031	0.02417	T	0.1047	0.4206
15	rs5030699	99671795	A	0.01031	0.02417	G	0.1047	0.4206
16	rs118131564	2505490	T	0.01031	0.02417	A	0.1047	0.4206
16	rs117769220	28944282	A	0.01031	0.02417	G	0.1047	0.4206
19	rs2230636	4409756	G	0.01031	0.02417	A	0.1047	0.4206
2	rs10928927	130468366	G	0.2809	0.2383	A	0.1048	1.249

13	rs1332941	41695100	G	0.4175	0.3708	A	0.1048	1.216
16	rs17227424	89804624	C	0.09536	0.1267	G	0.1048	0.7268
19	rs58537897	44500701	A	0.09585	0.1281	G	0.1048	0.7213
3	rs13064954	156854742	A	0.05928	0.08583	G	0.1049	0.6711
9	rs34138827	101767245	A	0.05928	0.08583	G	0.1049	0.6711
1	exm19696	15904318	A	0.05928	0.085	G	0.105	0.6783
1	rs11544523	15904318	A	0.05928	0.085	G	0.105	0.6783
4	rs10013280	147788709	A	0.3582	0.3133	G	0.105	1.223
9	rs10868138	86917301	G	0.05928	0.085	A	0.105	0.6783
9	rs876347	121031029	G	0.201	0.1642	A	0.105	1.281
16	rs9806945	1894961	G	0.2242	0.1858	A	0.105	1.266
1	rs2297792	156011444	G	0.1804	0.145	A	0.1051	1.298
12	rs17224810	68709892	A	0.1804	0.145	G	0.1051	1.298
3	rs28932173	17051450	A	0.08763	0.0625	C	0.1052	1.441
15	rs75021400	42981377	A	0.08763	0.0625	G	0.1052	1.441
19	rs1465789	58946056	A	0.2912	0.3367	G	0.1052	0.8096
7	rs2429582	122202593	G	0.3608	0.315	A	0.1053	1.228
3	rs12496109	12584699	G	0.04639	0.02917	A	0.1054	1.619
6	rs9689337	10747996	G	0.07474	0.0525	A	0.1054	1.458
7	rs6965750	21824122	A	0.07474	0.0525	G	0.1054	1.458
7	rs1532045	129984630	A	0.2912	0.3358	G	0.1054	0.8126
12	rs2731073	48919556	A	0.04639	0.02917	G	0.1054	1.619
18	rs36101975	28956904	A	0.1108	0.1442	G	0.1055	0.7399
1	rs77511432	9118247	C	0.01031	0.003333	A	0.1056	3.115
1	rs138189536	11863038	A	0.01031	0.003333	G	0.1056	3.115
1	rs147421281	25627470	A	0.01031	0.003333	G	0.1056	3.115
1	rs35659744	27687466	A	0.08247	0.1117	C	0.1056	0.7151
1	rs117201204	89486370	A	0.01031	0.003333	G	0.1056	3.115
1	rs143922591	154459120	C	0.01031	0.003333	A	0.1056	3.115
1	rs146346644	179352607	A	0.01031	0.003333	C	0.1056	3.115
1	rs139376570	200943861	A	0.01031	0.003333	G	0.1056	3.115
2	rs80075099	169668122	A	0.01031	0.003333	G	0.1056	3.115
2	rs79776875	185802335	G	0.01031	0.003333	A	0.1056	3.115
2	rs78607324	185802542	C	0.01031	0.003333	A	0.1056	3.115
2	rs145621410	216241271	A	0.01031	0.003333	G	0.1056	3.115
2	rs145708986	234394209	G	0.01031	0.003333	A	0.1056	3.115
3	rs142584671	58520731	A	0.01031	0.003333	G	0.1056	3.115
4	rs148934306	8603180	A	0.01031	0.003333	G	0.1056	3.115
4	rs181299724	57189604	C	0.01031	0.003333	A	0.1056	3.115
4	rs189717933	109677604	A	0.01031	0.003333	G	0.1056	3.115
4	rs201831997	152096094	A	0.01031	0.003333	G	0.1056	3.115
4	rs201558687	156651348	A	0.01031	0.003333	G	0.1056	3.115
5	rs17751013	76736742	A	0.01031	0.003333	G	0.1056	3.115

5	rs78035319	87536663	G	0.01031	0.003333	A	0.1056	3.115
5	rs145965664	150029723	C	0.01031	0.003333	A	0.1056	3.115
5	rs142049559	180166742	A	0.01031	0.003333	G	0.1056	3.115
6	rs2734985	29818662	G	0.1005	0.07333	A	0.1056	1.412
6	rs140038187	35258465	A	0.01031	0.003333	C	0.1056	3.115
6	rs182762857	129691106	A	0.01031	0.003333	G	0.1056	3.115
7	rs200840761	47913546	A	0.01031	0.003333	C	0.1056	3.115
7	rs141723617	117171053	G	0.01031	0.003333	A	0.1056	3.115
7	rs61746066	143632983	A	0.01031	0.003333	G	0.1056	3.115
7	rs201385839	156437221	A	0.01031	0.003333	C	0.1056	3.115
8	rs186188319	53569975	A	0.01031	0.003333	G	0.1056	3.115
9	rs146546123	1056424	A	0.01031	0.003333	C	0.1056	3.115
9	rs200045699	27286353	A	0.01031	0.003333	T	0.1056	3.115
9	rs3213763	132375822	A	0.1005	0.07333	G	0.1056	1.412
10	rs143563006	5937039	C	0.01031	0.003333	G	0.1056	3.115
10	rs200693631	38122023	G	0.01031	0.003333	A	0.1056	3.115
11	rs181657861	68675700	A	0.01031	0.003333	G	0.1056	3.115
11	rs115318063	77937828	A	0.01031	0.003333	G	0.1056	3.115
11	rs117319789	125763902	A	0.01031	0.003333	G	0.1056	3.115
12	rs116626063	48359763	G	0.01031	0.003333	C	0.1056	3.115
12	rs201149022	54339018	A	0.01031	0.003333	G	0.1056	3.115
12	rs199876753	60168591	A	0.01031	0.003333	T	0.1056	3.115
14	rs114213493	24807108	A	0.01031	0.003333	G	0.1056	3.115
14	rs149581331	102482413	A	0.01031	0.003333	G	0.1056	3.115
15	rs34266719	58306448	G	0.01031	0.003333	A	0.1056	3.115
15	rs202074846	76633491	G	0.01031	0.003333	C	0.1056	3.115
15	rs145389125	80460617	A	0.01031	0.003333	G	0.1056	3.115
15	rs191202700	89848621	A	0.01031	0.003333	G	0.1056	3.115
16	rs12444268	20342572	A	0.01031	0.003333	T	0.1056	3.115
16	rs201110794	65022127	G	0.01031	0.003333	A	0.1056	3.115
16	rs199571418	82197733	G	0.01031	0.003333	A	0.1056	3.115
17	rs147936148	62045401	A	0.01031	0.003333	G	0.1056	3.115
17	rs117338850	66250649	G	0.01031	0.003333	A	0.1056	3.115
17	rs200856561	78081415	A	0.01031	0.003333	G	0.1056	3.115
17	rs144638934	80545106	A	0.01031	0.003333	G	0.1056	3.115
19	rs34104245	757887	A	0.01031	0.003333	G	0.1056	3.115
19	rs200939177	1371028	A	0.01031	0.003333	G	0.1056	3.115
19	rs146067833	48519183	A	0.01031	0.003333	G	0.1056	3.115
21	rs145959543	45537786	A	0.01031	0.003333	G	0.1056	3.115
22	rs33918436	32439365	A	0.01031	0.003333	G	0.1056	3.115
22	rs181831287	38323483	A	0.01031	0.003333	G	0.1056	3.115
3	rs6803290	59692168	G	0.1108	0.1433	A	0.1057	0.7449
2	rs1078004	85769711	C	0.3531	0.4	G	0.1058	0.8187

19	rs2303797	42402593	A	0.04639	0.02922	G	0.1058	1.617
11	rs1471446	127274109	C	0.25	0.2108	A	0.1059	1.248
14	rs17197037	21725654	G	0.3634	0.3175	A	0.1059	1.227
1	rs2235371	209964080	A	0.4253	0.3783	G	0.106	1.216
6	rs2243380	117724379	A	0.04124	0.06417	G	0.106	0.6273
10	rs11547671	102822575	C	0.04124	0.06417	A	0.106	0.6273
11	rs143672526	18267021	A	0.01031	0.003339	G	0.106	3.109
12	rs4237805	57249600	A	0.1675	0.2058	G	0.106	0.7764
17	rs75790652	39643934	C	0.1289	0.09833	G	0.106	1.356
22	rs9627183	48284514	A	0.04124	0.06417	G	0.106	0.6273
1	rs12410676	108185309	A	0.2809	0.24	G	0.1061	1.237
2	rs12476047	164317876	G	0.1675	0.2067	A	0.1061	0.7725
5	rs803137	132132647	A	0.1675	0.2067	G	0.1061	0.7725
6	rs2860580	29906691	A	0.2242	0.1867	G	0.1061	1.259
12	rs7298579	24749874	A	0.1675	0.2053	G	0.1061	0.7788
5	rs286154	59926911	G	0.3531	0.3992	A	0.1062	0.8216
17	rs7212944	38122686	A	0.3531	0.3992	G	0.1062	0.8216
3	rs7634981	131530483	G	0.3557	0.4025	A	0.1063	0.8194
6	rs2254287	33143948	C	0.3196	0.2758	G	0.1063	1.233
6	rs1799908	33144243	T	0.3196	0.2758	A	0.1063	1.233
18	rs2162440	35214006	A	0.2835	0.2417	G	0.1064	1.242
18	rs12606417	44104437	G	0.03351	0.05426	A	0.1064	0.6043
2	rs61744510	136107777	A	0.03351	0.055	G	0.1065	0.5956
3	rs17080319	48732480	G	0.04124	0.065	A	0.1067	0.6187
7	rs6964415	46242583	C	0.2268	0.1883	A	0.1067	1.264
16	rs4985556	70694000	A	0.04124	0.065	C	0.1067	0.6187
19	rs330877	6896483	A	0.3582	0.405	G	0.1067	0.8201
1	rs10489849	159196765	A	0.02577	0.01333	G	0.1069	1.958
4	rs75719146	7044006	A	0.02577	0.01333	C	0.1069	1.958
5	rs55970008	66460630	G	0.02577	0.01333	A	0.1069	1.958
6	rs6903433	32842975	A	0.2036	0.1667	G	0.1069	1.278
17	rs61741125	46052883	A	0.02577	0.01333	G	0.1069	1.958
20	rs6011441	61513649	A	0.02577	0.01333	C	0.1069	1.958
21	rs2828520	25143119	G	0.1289	0.1642	A	0.1069	0.7532
21	rs2070417	32638549	A	0.3627	0.4092	C	0.1069	0.8218
6	rs3868082	31207692	A	0.3608	0.4083	G	0.107	0.818
9	rs4877257	86117611	C	0.2552	0.2983	A	0.1071	0.8057
10	rs7897	103922896	C	0.3608	0.4075	A	0.1071	0.8208
6	rs6921636	46080356	G	0.3634	0.4108	A	0.1074	0.8186
11	rs61895095	75298468	C	0.02062	0.03833	A	0.1074	0.5281
12	rs1047699	10999708	A	0.02062	0.03833	G	0.1074	0.5281
15	rs116333487	42977839	G	0.02062	0.03833	C	0.1074	0.5281
15	rs116745790	42980023	A	0.02062	0.03833	G	0.1074	0.5281

18	rs1786648	24462611	A	0.3634	0.41	G	0.1076	0.8215
2	rs3751109	238427194	G	0.08763	0.06333	A	0.1077	1.42
2	rs3751107	238427251	A	0.08763	0.06333	G	0.1077	1.42
21	rs2823850	17877794	A	0.08763	0.06333	G	0.1077	1.42
1	rs10890013	73639751	A	0.1701	0.2092	G	0.1078	0.775
2	rs61746994	218712290	A	0.06701	0.045	G	0.1079	1.524
6	rs2842413	19129805	G	0.4356	0.3892	A	0.1079	1.211
9	rs2814828	90811182	G	0.2577	0.3017	A	0.1079	0.8038
19	rs1078264	12963143	A	0.3015	0.3475	G	0.108	0.8107
7	rs7808249	86983715	A	0.2577	0.3008	G	0.1081	0.807
10	rs2184033	59823894	G	0.3686	0.4158	A	0.1081	0.82
14	rs1958716	20528321	A	0.3686	0.4158	G	0.1081	0.82
4	rs2070631	159642576	A	0.03866	0.02333	G	0.1082	1.683
5	rs4457053	76424949	G	0.03866	0.02333	A	0.1082	1.683
6	rs2523741	30211903	C	0.09794	0.13	G	0.1082	0.7266
12	rs2338104	109895168	G	0.3711	0.3258	C	0.1082	1.221
1	rs12121990	108523670	C	0.2242	0.2667	A	0.1083	0.7949
1	rs3753494	100358103	A	0.03351	0.01833	G	0.1086	1.856
1	rs61729969	118509284	A	0.02062	0.03917	C	0.1086	0.5165
3	rs148960024	20189472	A	0.03351	0.01833	C	0.1086	1.856
4	rs1801208	6302889	A	0.07216	0.1008	G	0.1086	0.6936
5	rs139173233	442675	A	0.02062	0.03917	G	0.1086	0.5165
20	rs3180370	13747441	A	0.07216	0.1008	G	0.1086	0.6936
20	rs2281209	32664926	A	0.07216	0.1008	G	0.1086	0.6936
6	rs2301227	33033814	G	0.07732	0.05417	A	0.1088	1.463
12	rs2958149	57109792	A	0.2603	0.3042	G	0.1088	0.8051
19	rs2229531	11687195	A	0.07732	0.05417	G	0.1088	1.463
19	rs2305799	11687351	A	0.07732	0.05417	G	0.1088	1.463
6	rs9341838	81684572	G	0.3737	0.4208	A	0.1089	0.8212
7	rs75519181	17378908	G	0.01546	0.03167	A	0.1089	0.4803
12	rs1021870	67365344	A	0.3737	0.4208	G	0.1089	0.8212
12	rs56006713	113354384	A	0.01546	0.03167	G	0.1089	0.4803
17	rs11655342	4356375	A	0.1469	0.115	C	0.1089	1.325
17	rs144544474	43924292	A	0.01546	0.03167	G	0.1089	0.4803
7	rs2272330	141727526	A	0.08505	0.1144	C	0.1092	0.7199
15	rs11072506	75052994	A	0.3737	0.42	G	0.1092	0.824
3	rs4687717	53282188	A	0.4485	0.4017	G	0.1094	1.211
6	rs3873342	30957766	G	0.08505	0.1142	A	0.1094	0.7213
6	rs12198448	30992346	G	0.08505	0.1142	A	0.1094	0.7213
6	rs9285421	117842698	G	0.08505	0.1142	A	0.1094	0.7213
11	rs739401	3036324	G	0.3737	0.3292	A	0.1094	1.216
14	rs4982766	23994517	G	0.2887	0.2475	A	0.1094	1.234
15	rs3743171	65916527	A	0.3067	0.3517	T	0.1094	0.8156

2	rs1521882	202949851	G	0.3273	0.2842	A	0.1095	1.226
3	rs2306875	4712413	A	0.3763	0.3308	G	0.1095	1.22
11	rs506028	66468469	G	0.2268	0.2683	A	0.1095	0.7998
12	rs1015443	11061122	A	0.2268	0.2683	G	0.1095	0.7998
12	exm1036637	110841379	A	0.1649	0.1317	G	0.1095	1.303
12	rs75347070	110841379	A	0.1649	0.1317	G	0.1095	1.303
18	rs1962519	3188976	A	0.1727	0.2108	G	0.1095	0.7813
19	rs4830	40197924	G	0.3789	0.4258	A	0.1096	0.8224
20	rs4911249	31214403	A	0.3789	0.4258	G	0.1096	0.8224
14	rs4903031	73019236	G	0.1314	0.1008	A	0.1097	1.35
16	rs11646443	84270476	A	0.116	0.0875	G	0.1097	1.368
6	rs2050189	32339647	G	0.2912	0.2492	A	0.1098	1.238
1	rs6694545	30437268	A	0.1985	0.2392	G	0.1099	0.7876
11	rs57734357	46304338	G	0.1985	0.2392	A	0.1099	0.7876
6	rs3131064	30763893	G	0.06186	0.08833	A	0.11	0.6805
18	rs9944810	56247600	C	0.384	0.4308	A	0.1102	0.8236
4	exm2256327	126240485	A	0.0567	0.0375	G	0.1103	1.543
4	rs144356360	126240485	A	0.0567	0.0375	G	0.1103	1.543
16	rs116991581	68112725	A	0.0567	0.0375	G	0.1103	1.543
13	rs9555810	112181437	G	0.1521	0.1892	C	0.1104	0.7687
2	rs7565527	186667360	C	0.05181	0.0775	A	0.1105	0.6504
8	rs9642880	128718068	A	0.2912	0.25	C	0.1105	1.233
10	rs10999409	72332440	A	0.2912	0.25	G	0.1105	1.233
1	rs4481887	248496863	A	0.1856	0.1508	G	0.1106	1.283
2	rs4852054	240279945	G	0.2294	0.2717	A	0.1106	0.798
5	rs17707947	16877744	A	0.06186	0.08917	G	0.1107	0.6735
6	rs6930572	31428169	G	0.01554	0.0325	A	0.1107	0.47
8	rs117676215	70585325	A	0.06701	0.04583	G	0.1109	1.495
5	rs78363009	41064588	A	0.01546	0.0325	G	0.111	0.4676
6	rs9257857	29394926	A	0.09021	0.065	G	0.111	1.426
6	rs1145712	88021118	G	0.3918	0.4392	A	0.111	0.8225
6	rs76160752	152629631	A	0.01546	0.0325	G	0.111	0.4676
12	rs61941963	48920073	A	0.01546	0.0325	G	0.111	0.4676
12	rs45620632	113407773	A	0.01546	0.0325	G	0.111	0.4676
21	rs8134971	33700426	G	0.3918	0.4392	A	0.111	0.8225
22	rs5903	21133831	A	0.09021	0.065	C	0.111	1.426
22	rs371513	21988599	A	0.01546	0.0325	G	0.111	0.4676
2	rs1992950	200290359	A	0.3814	0.3367	G	0.1111	1.215
5	rs13187289	133849177	C	0.06701	0.04591	G	0.1112	1.493
6	rs1294421	6743149	C	0.1753	0.2142	A	0.1112	0.7797
6	rs204993	32155581	G	0.3943	0.4417	A	0.1112	0.823
2	rs2361502	234698790	G	0.1005	0.1325	A	0.1113	0.7316
6	rs4959786	3264487	A	0.384	0.3383	G	0.1113	1.219

6	rs4959789	3264527	A	0.384	0.3383	G	0.1113	1.219
6	rs3094577	29533870	A	0.1005	0.1325	G	0.1113	0.7316
6	rs1003582	29538403	C	0.1005	0.1325	G	0.1113	0.7316
14	rs17099370	62463129	C	0.4639	0.4175	A	0.1114	1.207
17	rs3809835	6406883	A	0.2629	0.222	G	0.1114	1.25
3	rs843358	183861243	A	0.3969	0.4442	G	0.1115	0.8236
3	rs2362605	190480177	G	0.3943	0.4408	A	0.1115	0.8258
8	rs57041981	10557760	G	0.2938	0.2525	A	0.1115	1.232
10	rs1200821	37559580	A	0.268	0.3117	G	0.1115	0.8088
15	rs2413935	49450944	G	0.3969	0.4442	A	0.1115	0.8236
3	rs4686302	8809222	A	0.2345	0.1958	G	0.1116	1.258
14	rs1950736	52600254	A	0.3351	0.2908	G	0.1116	1.229
1	rs13303010	894573	G	0.232	0.2742	A	0.1117	0.7996
20	rs3752252	59829847	G	0.4716	0.4242	A	0.1118	1.212
3	rs117517364	129195166	A	0.04897	0.03083	C	0.112	1.618
6	rs3129888	32411726	G	0.04897	0.03083	A	0.112	1.618
7	rs2236426	138764233	A	0.4021	0.4492	G	0.112	0.8246
3	rs3907825	186812036	A	0.4716	0.425	G	0.1121	1.208
8	rs3735799	141467960	G	0.4716	0.425	A	0.1121	1.208
13	rs4942486	32953388	A	0.3995	0.4458	G	0.1121	0.8269
7	rs62477301	114924344	G	0.3866	0.3417	A	0.1122	1.214
7	rs6465825	77416439	G	0.1546	0.1917	A	0.1123	0.7715
4	rs10433903	118093137	A	0.4794	0.4317	G	0.1124	1.212
7	rs6964421	6520676	A	0.4021	0.4483	G	0.1124	0.8274
8	rs16939382	77785447	G	0.3376	0.2933	A	0.1124	1.228
8	rs6469804	120044829	G	0.2345	0.1967	A	0.1124	1.252
11	rs10837814	5345128	A	0.3376	0.2933	G	0.1124	1.228
14	rs1132644	20784718	A	0.4794	0.4317	C	0.1124	1.212
1	rs2737619	200107578	G	0.4768	0.43	A	0.1125	1.208
14	rs3825632	44974922	A	0.4794	0.4324	T	0.1125	1.209
1	rs2239819	175375469	C	0.4794	0.4325	A	0.1126	1.208
11	rs2234409	57321811	A	0.3196	0.3642	C	0.1126	0.8201
11	rs886201	124252718	A	0.2964	0.255	G	0.1126	1.231
22	rs4820853	30864610	G	0.4046	0.4508	A	0.1126	0.8282
9	rs10985765	101056122	G	0.2113	0.1742	A	0.1127	1.271
20	rs6057717	31656632	C	0.2113	0.1742	G	0.1127	1.271
2	rs3087386	100055506	G	0.3892	0.3442	A	0.1128	1.214
3	rs13068323	98109640	A	0.1881	0.1533	G	0.1128	1.28
4	rs3796905	7435805	A	0.07474	0.1025	C	0.1128	0.7073
6	rs112276836	29910750	A	0.1778	0.2167	G	0.1128	0.782
6	rs2844502	31445216	G	0.4223	0.47	A	0.1128	0.8243
2	rs75743618	56420296	A	0.08763	0.1175	G	0.1129	0.7214
2	rs4972945	231149097	G	0.08763	0.1175	A	0.1129	0.7214

4	rs6858288	61258122	G	0.3938	0.3483	A	0.1129	1.215
5	rs14251	141019110	A	0.08763	0.1175	C	0.1129	0.7214
14	rs10131193	44266450	A	0.08763	0.1175	C	0.1129	0.7214
19	rs2302182	38827960	G	0.08763	0.1175	A	0.1129	0.7214
20	rs3827103	54824029	A	0.1778	0.2158	G	0.1129	0.7859
2	rs10206850	138420996	A	0.4845	0.4375	G	0.113	1.209
5	rs27650	80376142	A	0.4845	0.4375	G	0.113	1.209
6	rs12662501	31190850	A	0.1314	0.1025	G	0.113	1.325
13	rs2328992	77397401	A	0.3918	0.3458	G	0.113	1.218
20	rs6017667	44352620	A	0.4149	0.4625	G	0.113	0.8243
20	rs6032474	44354372	A	0.4149	0.4625	G	0.113	0.8243
21	rs7279204	47808679	A	0.2655	0.225	G	0.113	1.245
5	rs61739378	53815240	C	0.4871	0.44	G	0.1131	1.209
15	rs2061821	86122654	A	0.4871	0.44	G	0.1131	1.209
15	rs2061824	86123019	G	0.4871	0.44	A	0.1131	1.209
15	rs4075256	86123833	A	0.4871	0.44	G	0.1131	1.209
15	rs4075254	86123988	G	0.4871	0.44	A	0.1131	1.209
15	rs4843074	86124483	G	0.4871	0.44	C	0.1131	1.209
15	rs4843075	86124555	G	0.4871	0.44	A	0.1131	1.209
1	rs926579	210590153	G	0.4149	0.4617	A	0.1132	0.827
3	rs1391050	130149845	C	0.4149	0.4617	A	0.1132	0.827
7	rs2297241	138761034	G	0.4175	0.465	C	0.1132	0.8247
8	rs2738113	6829085	C	0.4175	0.465	A	0.1132	0.8247
10	rs11185790	91372533	A	0.4175	0.465	G	0.1132	0.8247
8	rs13275250	100092857	G	0.4948	0.4472	A	0.1133	1.211
8	rs445114	128323181	G	0.4897	0.4425	A	0.1133	1.209
2	rs780093	27742603	G	0.4871	0.4407	A	0.1134	1.205
3	rs1129183	45869972	A	0.09021	0.06583	G	0.1134	1.407
13	rs1333561	84434888	A	0.4201	0.4675	G	0.1134	0.8252
9	rs79023327	14801769	A	0.1675	0.135	G	0.1135	1.289
10	rs2767434	134622408	A	0.3376	0.295	G	0.1135	1.218
2	rs1529093	177909595	G	0.4201	0.4667	A	0.1136	0.8279
5	rs2269953	137682714	A	0.4871	0.4408	C	0.1136	1.205
6	rs7382112	28665109	A	0.4201	0.4667	G	0.1136	0.8279
8	rs6578193	142505038	A	0.3247	0.37	G	0.1136	0.8189
2	rs6746533	49222572	C	0.4974	0.45	A	0.1137	1.21
2	rs6760896	233172923	A	0.4897	0.4433	G	0.1137	1.205
6	rs7745413	29915469	G	0.443	0.4908	A	0.1137	0.8251
3	rs4855697	108867128	A	0.04381	0.0675	G	0.1138	0.633
4	rs7667636	135741792	A	0.5	0.4525	G	0.1138	1.21
8	rs17335870	101206391	A	0.02835	0.0475	G	0.1139	0.5851
10	rs7094436	8392174	G	0.4278	0.475	A	0.1139	0.8265
2	rs10496651	126667897	A	0.2371	0.1992	G	0.114	1.25

5	rs9293321	82184403	G	0.4253	0.4717	A	0.114	0.8288
6	rs1233495	29444121	A	0.2371	0.1992	G	0.114	1.25
6	rs1233487	29468809	G	0.2371	0.1992	A	0.114	1.25
6	rs3130059	31509284	G	0.5	0.4533	C	0.114	1.206
11	rs10833049	19077765	G	0.1373	0.172	A	0.114	0.7664
14	rs961097	81921468	A	0.5026	0.455	G	0.114	1.21
1	rs1929860	227216775	A	0.5	0.4533	G	0.1141	1.206
2	rs7583383	61500322	G	0.3273	0.3725	A	0.1141	0.8197
4	rs17249857	91826328	A	0.3273	0.3733	G	0.1141	0.8168
5	rs224934	81718588	A	0.3273	0.3733	G	0.1141	0.8168
6	rs2071594	31512720	C	0.5	0.4533	G	0.1141	1.206
8	rs2280560	8560602	A	0.3273	0.3733	G	0.1141	0.8168
10	rs1063535	129902281	A	0.5	0.4533	G	0.1141	1.206
5	rs77732988	41142932	A	0.02835	0.04833	G	0.1142	0.5745
5	rs76528010	41159192	A	0.02835	0.04833	G	0.1142	0.5745
5	rs80108105	41159339	C	0.02835	0.04833	G	0.1142	0.5745
16	rs2287798	25239805	C	0.4278	0.4742	G	0.1142	0.8292
1	rs28362581	110163879	A	0.268	0.2275	G	0.1143	1.243
7	rs848486	77552127	G	0.1572	0.1942	A	0.1143	0.7742
17	rs9303401	56847945	A	0.4381	0.4858	G	0.1143	0.8253
2	rs7594432	25482883	A	0.5103	0.4633	G	0.1144	1.207
2	rs6751715	56363377	G	0.1813	0.22	A	0.1144	0.7854
6	rs1077393	31610529	A	0.4381	0.485	G	0.1144	0.8281
6	rs1052486	31610686	A	0.4381	0.485	G	0.1144	0.8281
6	rs1482394	149217078	G	0.5103	0.4633	A	0.1144	1.207
7	rs215614	32347335	A	0.3969	0.3517	G	0.1144	1.213
7	rs2288275	132263595	G	0.5103	0.4633	A	0.1144	1.207
6	rs2256902	29940372	A	0.1804	0.2192	G	0.1145	0.7843
1	rs4147141	69579252	G	0.4637	0.51	A	0.1146	0.8308
3	rs4462937	57414434	G	0.433	0.4792	A	0.1146	0.83
7	rs7795096	151546589	A	0.3015	0.26	G	0.1146	1.229
8	rs7818553	110380421	G	0.433	0.4792	A	0.1146	0.83
8	rs964307	110413762	G	0.433	0.4792	A	0.1146	0.83
10	rs11203042	90989109	G	0.518	0.4708	A	0.1146	1.208
10	rs2275580	99125949	A	0.4407	0.4875	G	0.1146	0.8284
11	rs1045288	237087	A	0.2139	0.1767	G	0.1146	1.268
11	rs10902112	244106	A	0.2139	0.1767	G	0.1146	1.268
11	rs7107362	244108	A	0.2139	0.1767	G	0.1146	1.268
11	rs7128044	244115	G	0.2139	0.1767	A	0.1146	1.268
11	rs7116130	244129	G	0.2139	0.1767	A	0.1146	1.268
11	rs7128029	244141	G	0.2139	0.1767	A	0.1146	1.268
18	rs125555	47800179	C	0.2139	0.1767	G	0.1146	1.268
15	rs1060939	43816917	A	0.451	0.4983	G	0.1147	0.8271

1	rs7516958	195916112	A	0.4588	0.5058	G	0.1148	0.8281
5	rs12520467	32087583	A	0.03368	0.0192	C	0.1148	1.781
11	rs2276288	76912636	T	0.5309	0.4833	A	0.1148	1.21
13	rs9520691	108658557	G	0.5335	0.4858	A	0.1148	1.21
21	rs4816958	25808101	G	0.4562	0.5033	A	0.1148	0.8278
22	rs4823002	29410104	A	0.5309	0.4833	G	0.1148	1.21
3	rs7632885	140178485	A	0.5129	0.4667	G	0.1149	1.203
6	rs9368699	31802541	G	0.1031	0.0775	A	0.1149	1.368
7	rs2788478	158672619	A	0.3969	0.3525	G	0.1149	1.209
11	rs2271001	19167850	A	0.2784	0.3225	G	0.1149	0.8103
13	rs9567933	48420218	A	0.5335	0.4867	G	0.1149	1.206
16	rs2000999	72108093	A	0.2784	0.3225	G	0.1149	0.8103
3	rs11177	52721305	A	0.518	0.4717	G	0.115	1.204
9	rs7858942	92701618	G	0.5232	0.4767	A	0.115	1.205
20	rs1569767	19261623	G	0.518	0.4717	A	0.115	1.204
8	rs6997563	139039589	A	0.03608	0.0575	G	0.1151	0.6136
10	rs11591817	129904477	A	0.3428	0.3	G	0.1151	1.217
4	rs5018568	186617593	A	0.4588	0.505	G	0.1152	0.8308
5	rs2221443	174465590	G	0.4588	0.505	A	0.1152	0.8308
12	rs2089222	117002658	A	0.3041	0.2617	G	0.1152	1.233
12	rs14024	53069014	A	0.3325	0.3775	G	0.1153	0.8213
14	rs11549465	62207557	A	0.06701	0.04667	G	0.1153	1.467
19	rs2250918	17036087	A	0.06701	0.04667	G	0.1153	1.467
3	rs4857284	93991504	G	0.4639	0.51	A	0.1154	0.8314
6	rs11753382	30039364	A	0.2088	0.2492	C	0.1154	0.7951
6	rs2285805	30042861	C	0.2088	0.2492	G	0.1154	0.7951
6	rs3807033	30043955	A	0.2088	0.2492	G	0.1154	0.7951
1	rs6679393	31770944	G	0.2139	0.1775	A	0.1155	1.261
1	rs146839643	145560094	G	0.03351	0.01917	A	0.1155	1.774
6	rs9296419	43581585	A	0.03351	0.01917	G	0.1155	1.774
19	rs2042200	9646898	A	0.03351	0.01917	G	0.1155	1.774
3	rs3729931	12626516	A	0.0799	0.05667	G	0.1157	1.446
5	rs10045431	158814533	A	0.1186	0.09083	C	0.1157	1.346
6	rs3819721	32804798	A	0.3041	0.2625	G	0.1157	1.228
8	rs4367565	131537955	G	0.3351	0.3808	A	0.1157	0.8192
9	rs41281027	104130469	G	0.0799	0.05667	C	0.1157	1.446
13	rs9513670	87045827	A	0.3351	0.3808	G	0.1157	0.8192
17	rs77585764	76187108	C	0.1186	0.09083	G	0.1157	1.346
1	rs749902	117660754	A	0.04124	0.025	G	0.1158	1.677
1	rs3795504	180905448	A	0.2809	0.3242	C	0.1158	0.8145
6	rs4713069	27128307	C	0.3454	0.3025	A	0.1158	1.216
17	rs139830628	28004744	C	0.04124	0.025	G	0.1158	1.677
19	rs181549336	1819112	G	0.04124	0.025	A	0.1158	1.677

4	rs2168813	81126083	G	0.4021	0.3575	A	0.1159	1.208
9	rs72766543	138439806	A	0.1392	0.1745	G	0.1159	0.7649
1	rs6598919	28432549	G	0.183	0.2217	A	0.1161	0.7864
8	rs2288672	8860620	A	0.183	0.2217	G	0.1161	0.7864
15	rs6496264	98442226	G	0.2423	0.2842	A	0.1161	0.8054
17	rs9909216	67212423	A	0.2423	0.2842	G	0.1161	0.8054
1	rs2291827	22165987	A	0.1392	0.1742	G	0.1162	0.7666
3	rs1850267	8152315	A	0.3376	0.3833	G	0.1162	0.82
10	rs2142991	43341105	G	0.1392	0.1742	A	0.1162	0.7666
1	rs79938119	160141525	G	0.05928	0.03917	A	0.1163	1.546
3	rs13096789	67054649	G	0.05928	0.03917	A	0.1163	1.546
9	rs117332028	140109141	A	0.06959	0.0475	G	0.1163	1.5
12	rs1195569	131225471	A	0.4046	0.36	G	0.1164	1.208
1	rs2229487	22159008	A	0.1392	0.175	C	0.1165	0.7622
15	rs2271725	68609647	A	0.1392	0.175	C	0.1165	0.7622
1	rs79282181	12337888	A	0.02062	0.01	G	0.1166	2.084
1	rs191137543	152085253	C	0.02062	0.01	A	0.1166	2.084
3	rs144022954	52233450	G	0.02062	0.01	A	0.1166	2.084
4	rs12643654	96159817	G	0.1211	0.155	A	0.1166	0.7514
6	rs3129274	33094869	G	0.02062	0.01	A	0.1166	2.084
11	rs1396883	34757447	A	0.1211	0.155	G	0.1166	0.7514
11	rs115240923	85437405	G	0.02062	0.01	A	0.1166	2.084
13	rs74471135	103390386	A	0.02062	0.01	C	0.1166	2.084
13	rs74435572	103392127	T	0.02062	0.01	A	0.1166	2.084
13	rs79592880	103392709	A	0.02062	0.01	T	0.1166	2.084
16	rs151075190	58714002	G	0.02062	0.01	A	0.1166	2.084
4	rs1828591	145480780	G	0.2835	0.3267	A	0.1167	0.8156
4	rs13118928	145486389	G	0.2835	0.3267	A	0.1167	0.8156
7	rs17167553	133848257	A	0.2835	0.3267	C	0.1167	0.8156
4	rs1502736	160607399	A	0.4098	0.3642	G	0.1168	1.212
1	rs897471	22191454	G	0.09021	0.1208	A	0.1169	0.7214
10	rs3740231	26463043	T	0.3402	0.385	A	0.1169	0.8237
2	rs6707475	74710491	G	0.1933	0.1583	A	0.1172	1.274
10	rs2981575	123346116	G	0.3428	0.3883	A	0.1172	0.8215
12	rs12367822	57204160	A	0.2448	0.2867	C	0.1172	0.8068
13	rs1417470	62248573	A	0.2448	0.2867	G	0.1172	0.8068
3	rs2178403	184039666	A	0.4098	0.365	G	0.1173	1.208
4	rs12502866	14790478	A	0.4124	0.3667	G	0.1173	1.212
12	rs187240221	54379117	G	0.04897	0.03167	A	0.1174	1.575
13	rs33949518	96555149	A	0.04897	0.03167	G	0.1174	1.575
1	rs6688451	53086040	G	0.3428	0.3875	A	0.1175	0.8244
2	rs4952710	44524575	G	0.3428	0.3875	A	0.1175	0.8244
5	rs6556993	96946900	A	0.4124	0.3673	C	0.1175	1.209

7	rs2305816	138312122	C	0.2732	0.2333	A	0.1175	1.235
1	rs34141181	40705427	G	0.007732	0.02	C	0.1176	0.3818
3	rs145529151	52568641	G	0.007732	0.02	A	0.1176	0.3818
3	rs77189011	187003786	A	0.007732	0.02	G	0.1176	0.3818
4	rs142651446	6279223	G	0.007732	0.02	A	0.1176	0.3818
4	rs143782841	20597357	G	0.007732	0.02	C	0.1176	0.3818
6	rs9261468	30104480	G	0.2461	0.2069	A	0.1176	1.252
6	rs28362679	32363893	A	0.007732	0.02	G	0.1176	0.3818
9	rs75259523	15744607	A	0.007732	0.02	G	0.1176	0.3818
10	rs188054898	112581547	A	0.007732	0.02	G	0.1176	0.3818
12	rs2229954	49221495	A	0.007732	0.02	G	0.1176	0.3818
12	rs145965687	120241020	A	0.007732	0.02	G	0.1176	0.3818
13	rs148232728	25433204	A	0.007732	0.02	C	0.1176	0.3818
16	rs80149007	84444206	G	0.007732	0.02	C	0.1176	0.3818
6	rs1265110	31119422	A	0.3093	0.2675	G	0.1177	1.226
7	rs875971	65617595	G	0.3093	0.2675	A	0.1177	1.226
18	rs1559806	72108787	G	0.1856	0.2242	A	0.1177	0.7886
19	rs2230539	14580328	G	0.1057	0.1367	A	0.1177	0.7464
6	rs56991168	56537363	A	0.1624	0.1992	G	0.1182	0.7794
19	rs3764650	1046520	C	0.3557	0.3117	A	0.1182	1.219
4	rs74824334	985275	A	0.007732	0.02083	G	0.1183	0.3662
8	rs138257744	144885669	C	0.007812	0.02003	G	0.1183	0.3852
9	rs200390935	139753209	A	0.007732	0.02083	G	0.1183	0.3662
11	rs146588273	72091352	G	0.007732	0.02083	A	0.1183	0.3662
13	rs11619479	88169764	G	0.1546	0.1227	A	0.1183	1.308
14	rs12890644	98113485	A	0.2474	0.29	G	0.1183	0.8049
19	rs7256712	4511525	A	0.007732	0.02083	G	0.1183	0.3662
19	rs1142530	1388538	A	0.2887	0.3314	G	0.1184	0.8187
2	rs744396	224570781	A	0.3479	0.3925	C	0.1185	0.8259
9	rs3780792	136835343	G	0.1418	0.1767	A	0.1185	0.7697
10	rs3810950	50824619	A	0.1933	0.1592	G	0.1185	1.266
6	rs9352613	79424433	G	0.4175	0.3725	A	0.1186	1.208
6	rs41554412	31380194	G	0.1425	0.1783	A	0.1187	0.7656
10	rs870957	45478092	G	0.3557	0.3125	A	0.1187	1.214
12	rs7134594	110000193	A	0.3557	0.3125	G	0.1187	1.214
5	rs7713676	145895246	A	0.0799	0.0575	G	0.1188	1.423
11	rs35199625	74880370	A	0.0799	0.0575	G	0.1188	1.423
3	rs4264750	19339747	G	0.2448	0.207	A	0.1189	1.242
4	rs1014867	126412891	A	0.06959	0.04833	G	0.119	1.473
10	rs2228064	45878050	A	0.06959	0.04833	G	0.119	1.473
11	rs1783596	72946020	G	0.3505	0.395	A	0.119	0.8266
14	rs3803311	105180903	A	0.06959	0.04833	G	0.119	1.473
16	rs11645366	62377153	A	0.06959	0.04833	G	0.119	1.473

1	rs1553921	204438643	C	0.2191	0.1825	A	0.1191	1.257
6	rs2931796	141443540	A	0.2191	0.1825	G	0.1191	1.257
1	rs117805097	24710465	G	0.01554	0.006667	A	0.1192	2.353
2	rs2286963	211060050	C	0.1891	0.2274	A	0.1192	0.7923
4	rs7688496	131254621	A	0.3531	0.3983	G	0.1192	0.8244
5	rs969351	15567629	A	0.3557	0.4015	G	0.1195	0.8228
6	rs757262	30114955	A	0.2474	0.2083	G	0.1195	1.249
6	rs1557611	30118360	A	0.2474	0.2083	G	0.1195	1.249
1	rs11552449	114448389	G	0.3557	0.4017	A	0.1196	0.8223
1	rs12569163	241878547	C	0.1057	0.08	A	0.1196	1.359
6	rs4709845	164900598	A	0.3557	0.4017	G	0.1196	0.8223
9	rs2210327	18109235	A	0.2938	0.3375	T	0.1196	0.8167
15	rs6496047	95378243	G	0.3557	0.4017	A	0.1196	0.8223
18	rs3891458	29848727	A	0.1057	0.08	G	0.1196	1.359
1	rs6677449	56458937	G	0.3608	0.3167	A	0.1197	1.218
6	rs9483788	135435501	G	0.3608	0.3167	A	0.1197	1.218
4	rs34543011	57250285	G	0.2474	0.209	A	0.1198	1.244
2	rs7579899	46537604	G	0.1366	0.1075	A	0.1199	1.314
3	rs1902341	31795570	G	0.2938	0.3367	A	0.1199	0.8198
4	rs3860707	52938243	G	0.3608	0.3175	A	0.1201	1.213
6	rs361525	31543101	A	0.05928	0.04	G	0.1201	1.512
6	rs3093662	31544189	G	0.05928	0.04	A	0.1201	1.512
16	rs34779002	87782396	A	0.05928	0.04	C	0.1201	1.512
12	rs1799516	16425656	A	0.09278	0.1225	T	0.1202	0.7326
15	rs3743398	89398407	A	0.06701	0.09333	G	0.1202	0.6977
8	rs2248609	101608919	G	0.2191	0.1833	A	0.1203	1.25
1	rs61761991	11918444	A	0.01546	0.006667	G	0.1204	2.34
1	rs146575757	16091591	A	0.01546	0.006667	G	0.1204	2.34
1	rs118089857	95307636	G	0.01546	0.006667	A	0.1204	2.34
1	rs12137976	113255212	C	0.4253	0.3808	A	0.1204	1.203
2	rs145510620	219508076	A	0.01546	0.006667	G	0.1204	2.34
3	rs138871762	128628975	A	0.01546	0.006667	C	0.1204	2.34
4	rs9993168	125807868	A	0.3634	0.3192	G	0.1204	1.218
5	rs76243481	54696117	A	0.01546	0.006667	G	0.1204	2.34
5	rs1002807	144528984	A	0.2526	0.2942	G	0.1204	0.8108
5	rs3734053	150921969	A	0.01546	0.006667	G	0.1204	2.34
5	rs3734061	150947751	A	0.01546	0.006667	G	0.1204	2.34
5	rs142612405	170338106	A	0.01546	0.006667	G	0.1204	2.34
6	rs11752262	31431757	G	0.09326	0.1233	A	0.1204	0.7311
7	rs58175273	66410231	G	0.01546	0.006667	A	0.1204	2.34
7	rs35529664	100454534	A	0.01546	0.006667	G	0.1204	2.34
8	rs201373242	17417839	G	0.01546	0.006667	A	0.1204	2.34
8	rs143212880	23002091	A	0.01546	0.006667	G	0.1204	2.34

8	rs1489356	111826826	A	0.3634	0.3192	G	0.1204	1.218
10	rs151027148	24813550	A	0.01546	0.006667	G	0.1204	2.34
10	rs2490741	94594565	A	0.3144	0.2733	G	0.1204	1.219
11	rs199849563	118455310	A	0.01546	0.006667	C	0.1204	2.34
11	rs189783368	119206824	A	0.01546	0.006667	G	0.1204	2.34
12	rs144940565	6753721	A	0.01546	0.006667	G	0.1204	2.34
12	rs708167	27234999	G	0.0232	0.04083	A	0.1204	0.5578
12	rs79585031	29450116	T	0.01546	0.006667	A	0.1204	2.34
13	rs201105747	111164467	A	0.01546	0.006667	G	0.1204	2.34
16	rs188060540	5040828	T	0.01546	0.006667	A	0.1204	2.34
16	rs201954702	23080019	A	0.01546	0.006667	G	0.1204	2.34
16	rs200752015	67919983	A	0.01546	0.006667	G	0.1204	2.34
16	rs200133878	72832153	A	0.01546	0.006667	G	0.1204	2.34
17	rs34733791	5437285	A	0.01546	0.006667	G	0.1204	2.34
17	rs11657747	5445243	A	0.01546	0.006667	G	0.1204	2.34
17	rs142525874	40734767	A	0.01546	0.006667	G	0.1204	2.34
22	rs148001527	24179956	A	0.01546	0.006667	C	0.1204	2.34
1	rs449255	229355000	A	0.4304	0.385	G	0.1206	1.207
6	rs10806425	90926612	A	0.06701	0.09417	C	0.1206	0.6909
8	rs977396	105992696	G	0.1959	0.1617	A	0.1206	1.263
10	rs10886954	123369542	G	0.3608	0.4058	A	0.1206	0.8265
13	rs2352028	92445229	A	0.2191	0.26	G	0.1207	0.7984
5	rs147539548	141021093	C	0.04639	0.07	A	0.1208	0.6463
8	rs201432181	37699794	A	0.01546	0.006678	T	0.1208	2.336
11	rs146341570	71809354	A	0.01554	0.006711	G	0.1208	2.337
21	rs2070418	32638550	A	0.3634	0.4092	G	0.1208	0.8243
1	rs11240573	205810947	A	0.1082	0.1392	G	0.1209	0.7509
7	rs35171886	12400783	A	0.2784	0.2392	G	0.1209	1.227
7	rs80272295	12401021	A	0.2784	0.2392	G	0.1209	1.227
16	rs1364063	69588572	G	0.1082	0.1392	A	0.1209	0.7509
6	rs6911017	148995849	A	0.4304	0.3858	G	0.1211	1.203
2	rs41459549	16169350	G	0.366	0.4117	A	0.1212	0.825
4	rs3796543	57221348	G	0.2474	0.21	A	0.1212	1.237
4	rs34228795	57248716	C	0.2474	0.21	G	0.1212	1.237
15	rs12439183	87058059	A	0.299	0.3425	G	0.1212	0.8187
17	rs52795654	5461671	G	0.01546	0.006689	C	0.1212	2.332
20	rs291671	31950845	G	0.299	0.3425	A	0.1212	0.8187
3	rs56348155	141636249	G	0.4356	0.39	A	0.1213	1.207
10	rs7071247	105257786	A	0.317	0.2758	C	0.1213	1.219
14	rs1958715	20528207	G	0.3705	0.4158	A	0.1213	0.8267
16	rs11641746	605481	A	0.4356	0.39	G	0.1213	1.207
4	rs13121513	92063883	A	0.433	0.3883	G	0.1214	1.203
5	rs11241230	113072475	A	0.2552	0.2967	G	0.1214	0.8121

4	rs1434536	96075965	A	0.366	0.4108	G	0.1215	0.8278
8	rs433689	37127143	G	0.299	0.3417	A	0.1215	0.8217
9	rs6475797	24545513	G	0.1985	0.1633	A	0.1215	1.268
13	rs9554325	28953808	G	0.299	0.3417	A	0.1215	0.8217
6	rs1419673	30096717	G	0.25	0.2117	A	0.1216	1.241
6	rs1419671	30097170	T	0.25	0.2117	A	0.1216	1.241
7	rs10243024	116346603	A	0.3196	0.2775	G	0.1216	1.223
8	rs10098533	105084341	G	0.0567	0.08167	A	0.1216	0.6759
20	rs3746504	44581348	A	0.25	0.2117	G	0.1216	1.241
8	rs7011881	115213656	A	0.1263	0.16	C	0.1218	0.7588
19	rs75388508	40440482	A	0.0567	0.08083	C	0.1218	0.6835
19	rs407731	44418077	G	0.1263	0.16	A	0.1218	0.7588
19	rs388706	44418693	A	0.1263	0.16	G	0.1218	0.7588
2	rs2609101	6185634	C	0.1675	0.2042	A	0.122	0.7844
6	rs2844621	31241537	G	0.1675	0.2042	A	0.122	0.7844
8	rs2403730	133092138	A	0.1675	0.2042	G	0.122	0.7844
4	rs9762089	160630642	A	0.4407	0.395	G	0.1221	1.207
11	rs11038871	46379442	G	0.1675	0.205	A	0.1221	0.7804
6	rs2502295	67794445	C	0.366	0.3233	A	0.1222	1.208
5	rs16899974	34998877	A	0.4407	0.3958	C	0.1224	1.203
12	rs923828	58015494	A	0.1778	0.1442	G	0.1224	1.284
6	rs3130922	31461089	A	0.1933	0.2325	G	0.1225	0.791
10	rs3125734	63958112	A	0.09536	0.07	G	0.1226	1.4
6	rs9320598	98019080	A	0.3737	0.4183	G	0.1227	0.8297
5	rs74512678	39202081	A	0.04124	0.02583	C	0.123	1.622
10	rs3814621	43292346	G	0.04124	0.02583	A	0.123	1.622
16	rs142195552	863422	A	0.04124	0.02583	G	0.123	1.622
1	rs857685	158577109	C	0.3763	0.4208	A	0.1231	0.8303
7	rs935765	37925438	A	0.3222	0.2808	G	0.1231	1.217
8	rs11136067	29305308	G	0.3222	0.2808	A	0.1231	1.217
2	rs77151072	152544903	G	0.06959	0.04917	A	0.1232	1.446
14	rs45587635	92958522	C	0.2242	0.265	A	0.1232	0.8017
15	rs1715919	56756285	C	0.06959	0.04917	A	0.1232	1.446
15	rs5030691	99666979	A	0.06959	0.04917	G	0.1232	1.446
22	rs8139422	50315363	A	0.06959	0.04917	C	0.1232	1.446
1	rs180983516	10166437	A	0.02835	0.015	G	0.1233	1.916
1	rs17410015	101551926	G	0.02835	0.015	A	0.1233	1.916
1	rs142594836	152191809	A	0.02835	0.015	G	0.1233	1.916
1	rs61752348	183086486	C	0.02835	0.015	A	0.1233	1.916
6	rs3873444	32682724	A	0.02835	0.015	G	0.1233	1.916
6	rs28372909	96974255	A	0.02835	0.015	C	0.1233	1.916
7	rs2233997	141478305	C	0.02835	0.015	A	0.1233	1.916
11	rs546381	133760651	A	0.3067	0.35	C	0.1233	0.8216

17	rs74546291	28320302	A	0.02835	0.015	G	0.1233	1.916
17	rs8082521	78302157	A	0.2835	0.2442	C	0.1233	1.225
19	rs200817158	49090527	A	0.02835	0.015	G	0.1233	1.916
21	rs9974333	40871810	A	0.3067	0.35	G	0.1233	0.8216
1	rs3170740	17312743	A	0.3067	0.3495	G	0.1234	0.8234
1	rs12143842	162033890	A	0.2603	0.3017	G	0.1234	0.8147
5	rs353372	40226919	A	0.3814	0.4267	G	0.1235	0.8286
7	rs6467710	137203819	C	0.1778	0.145	G	0.1235	1.275
9	rs2301612	136301982	C	0.201	0.1658	G	0.1236	1.266
15	rs2073440	50534514	C	0.03866	0.06	A	0.1236	0.63
19	rs62122088	35434448	G	0.03866	0.06	A	0.1236	0.63
1	rs6676438	161983089	G	0.451	0.4058	A	0.1237	1.203
4	rs12511068	24896658	A	0.1108	0.1425	G	0.1237	0.75
11	rs960193	56086147	A	0.1108	0.1425	C	0.1237	0.75
19	rs3810265	50223266	A	0.1701	0.2074	G	0.1237	0.7835
4	rs151225636	1738999	A	0	0.007525	G	0.1238	0
16	rs3743853	1877558	A	0.2242	0.1883	G	0.1238	1.246
1	rs11121959	12753203	G	0	0.0075	A	0.1239	0
1	rs184554996	54060127	A	0	0.0075	G	0.1239	0
1	rs10863698	208299199	A	0.3093	0.3533	G	0.1239	0.8195
2	rs63750716	47637371	G	0	0.0075	A	0.1239	0
2	rs151330826	152293370	G	0	0.0075	A	0.1239	0
3	exm2256005	38158122	A	0	0.0075	G	0.1239	0
3	rs115301879	140409919	A	0	0.0075	G	0.1239	0
3	rs140290837	195974246	A	0	0.0075	G	0.1239	0
4	rs78681393	48517084	C	0	0.0075	G	0.1239	0
4	rs725185	186510931	A	0	0.0075	G	0.1239	0
6	rs117882986	30916705	G	0	0.0075	A	0.1239	0
7	rs184462311	129824051	A	0	0.0075	G	0.1239	0
8	rs115263296	25323790	A	0	0.0075	G	0.1239	0
8	rs149565907	30945390	T	0	0.0075	A	0.1239	0
8	rs190676453	124988268	A	0	0.0075	G	0.1239	0
8	rs143727728	144644970	A	0	0.0075	G	0.1239	0
8	rs201030598	144658256	A	0	0.0075	G	0.1239	0
9	rs201611745	139612156	G	0	0.0075	A	0.1239	0
10	rs70937025	97174413	A	0	0.0075	G	0.1239	0
11	rs144302699	3774621	G	0	0.0075	A	0.1239	0
13	rs147120792	50204975	A	0	0.0075	G	0.1239	0
15	rs115124295	45559682	A	0	0.0075	C	0.1239	0
16	rs145539759	49314916	A	0	0.0075	C	0.1239	0
16	rs200621442	71894055	C	0	0.0075	G	0.1239	0
17	rs180851246	3524660	A	0	0.0075	G	0.1239	0
18	rs118089415	7013962	A	0	0.0075	C	0.1239	0

19	rs17216656	856015	A	0	0.0075	G	0.1239	0
19	rs2305770	51961545	A	0	0.0075	G	0.1239	0
20	rs116926921	5935832	G	0	0.0075	A	0.1239	0
20	rs58487183	5935875	A	0	0.0075	G	0.1239	0
20	rs16991591	5937833	G	0	0.0075	A	0.1239	0
20	rs185266382	55093178	A	0	0.0075	G	0.1239	0
21	rs191510940	39947670	A	0	0.0075	G	0.1239	0
21	rs201827340	44492252	A	0	0.0075	G	0.1239	0
22	rs180729981	26868809	C	0	0.0075	G	0.1239	0
22	rs142104315	36892103	C	0	0.0075	A	0.1239	0
22	rs185482276	37480861	A	0	0.0075	G	0.1239	0
1	rs1575938	193792484	A	0.1598	0.1275	G	0.1241	1.301
21	rs77389080	40883671	G	0.3093	0.3525	A	0.1241	0.8225
3	rs1488432	8243768	G	0.2629	0.305	A	0.1242	0.8127
9	rs7046603	125563212	G	0.05155	0.03333	A	0.1242	1.576
2	rs2305835	219130802	G	0.1082	0.0825	A	0.1243	1.35
3	rs114701641	38158122	A	0	0.007513	G	0.1243	0
9	rs117436169	35752124	A	0.1082	0.0825	G	0.1243	1.35
2	rs11687599	137178771	A	0.2629	0.3042	G	0.1244	0.8159
3	rs3772809	124462824	G	0.03093	0.05	A	0.1244	0.6064
7	rs854524	94898811	G	0.2629	0.3042	A	0.1244	0.8159
12	rs2926743	57114100	A	0.2629	0.3042	G	0.1244	0.8159
12	rs12832619	106332840	A	0.3892	0.435	G	0.1244	0.8275
15	rs3814281	83499766	A	0.1289	0.1625	G	0.1244	0.7624
1	rs11542470	25679393	A	0.03093	0.05083	G	0.1245	0.5959
1	rs75443355	226924569	A	0.03093	0.05083	C	0.1245	0.5959
4	rs2703130	77817874	A	0.03093	0.05083	C	0.1245	0.5959
6	rs2256183	31380529	A	0.1289	0.1617	G	0.1245	0.7671
12	rs2532501	6562753	A	0.2552	0.2167	G	0.1245	1.238
12	rs2532500	6562810	A	0.2552	0.2167	G	0.1245	1.238
15	rs1834207	95952841	G	0.4588	0.4133	A	0.1245	1.203
8	rs11782819	10334781	A	0.3119	0.3558	G	0.1246	0.8204
10	rs75316572	127967532	A	0.03866	0.06083	G	0.1246	0.6208
12	rs2238151	112211833	A	0.03866	0.06083	G	0.1246	0.6208
16	rs3024585	27359844	G	0.3119	0.355	A	0.1248	0.8234
17	rs9257	39084504	A	0.2887	0.2483	G	0.1248	1.228
6	rs411337	32077380	A	0.1804	0.1467	G	0.1249	1.281
6	rs204888	32089142	A	0.1804	0.1467	C	0.1249	1.281
6	rs169494	32097876	A	0.1804	0.1467	G	0.1249	1.281
4	rs12651301	47901476	G	0.4588	0.4142	A	0.125	1.199
6	rs200541066	29641397	A	0.01804	0.03417	G	0.1251	0.5194
9	rs74769898	127982839	A	0.01804	0.03417	C	0.1251	0.5194
10	rs74777754	61840324	A	0.01804	0.03417	G	0.1251	0.5194

13	rs17084492	27257004	A	0.01804	0.03417	G	0.1251	0.5194
14	rs76421634	24761467	A	0.01804	0.03417	C	0.1251	0.5194
12	rs3759295	31944985	C	0.1598	0.1283	A	0.1252	1.292
11	rs1156526	7022717	A	0.3299	0.2875	G	0.1253	1.22
20	rs1578411	17178434	G	0.3144	0.3583	A	0.1253	0.8213
1	rs10158354	228548197	A	0.3814	0.3375	G	0.1254	1.21
22	rs8177832	39477566	G	0.08247	0.06	A	0.1254	1.408
1	rs143187236	24432540	A	0.03351	0.02	C	0.1255	1.699
2	rs80120243	29226389	G	0.1985	0.2375	A	0.1255	0.7949
6	rs2516408	31463491	A	0.1985	0.2375	G	0.1255	0.7949
6	rs2246618	31478986	A	0.1985	0.2375	G	0.1255	0.7949
7	rs79413133	107167769	G	0.03351	0.02	A	0.1255	1.699
9	rs10810865	17895105	G	0.03351	0.02	A	0.1255	1.699
10	rs9417256	37976990	A	0.4639	0.4192	G	0.1255	1.199
11	rs2131535	130281488	G	0.1985	0.2375	A	0.1255	0.7949
14	rs118128989	93185129	A	0.03351	0.02	G	0.1255	1.699
15	rs3743247	99670847	A	0.03351	0.02	G	0.1255	1.699
15	rs5030698	99671765	G	0.03351	0.02	C	0.1255	1.699
16	rs34771199	20966291	A	0.03351	0.02	C	0.1255	1.699
16	rs61735055	20975505	G	0.03351	0.02	A	0.1255	1.699
17	rs78272919	39673366	C	0.03351	0.02	G	0.1255	1.699
18	rs76842111	6971872	A	0.03351	0.02	G	0.1255	1.699
9	rs2757858	10480542	G	0.4691	0.4233	A	0.1256	1.204
9	rs4836835	123757412	G	0.3995	0.445	A	0.1256	0.8297
13	rs9554176	28426279	A	0.3995	0.445	G	0.1256	0.8297
7	rs7787378	143701516	A	0.2294	0.27	G	0.1257	0.8048
5	rs11738832	139294677	G	0.4716	0.4258	A	0.1258	1.204
6	rs12523814	123110537	A	0.01289	0.0275	G	0.1258	0.4617
8	rs2233701	26365716	A	0.2294	0.2692	G	0.1258	0.8082
20	rs78274653	3211580	A	0.01289	0.0275	G	0.1258	0.4617
20	rs4814640	17705677	A	0.01289	0.0275	G	0.1258	0.4617
6	rs204896	32064098	A	0.1804	0.1475	G	0.126	1.272
6	rs439844	32072940	A	0.1813	0.1483	G	0.1261	1.272
20	rs1415771	33734493	A	0.268	0.31	G	0.1261	0.8151
2	rs9288684	233968153	A	0.2294	0.1925	G	0.1262	1.249
10	rs2778979	20534333	A	0.4021	0.4467	G	0.1262	0.833
19	rs2111504	32917455	A	0.1263	0.0975	T	0.1262	1.338
6	rs142986308	31112701	A	0.06186	0.04167	G	0.1263	1.516
11	rs7934336	4471473	A	0.06186	0.04167	G	0.1263	1.516
11	rs331537	4471276	A	0.06186	0.04174	G	0.1264	1.514
6	rs2021720	30449244	C	0.4768	0.4317	G	0.1265	1.2
7	rs2302006	75442730	A	0.4098	0.4558	C	0.1265	0.8289
7	rs741535	131491505	G	0.4098	0.4558	A	0.1265	0.8289

4	rs1992414	60960273	C	0.3196	0.3633	A	0.1266	0.823
1	rs11205277	149892872	G	0.2912	0.2517	A	0.1267	1.222
5	rs6889561	129694842	A	0.4124	0.4583	G	0.1267	0.8293
11	rs76215382	47711820	G	0.01804	0.035	A	0.1267	0.5066
15	rs2277848	84639309	A	0.01804	0.035	G	0.1267	0.5066
16	rs8046024	17200685	A	0.01804	0.035	G	0.1267	0.5066
19	rs10419469	37134737	G	0.1108	0.08417	A	0.1267	1.356
4	rs10032626	86808963	G	0.3196	0.3625	A	0.1268	0.826
7	rs820939	109297612	G	0.3196	0.3625	A	0.1268	0.826
17	rs6565484	78848856	G	0.3196	0.3625	A	0.1268	0.826
4	rs726899	94727622	G	0.4845	0.4392	A	0.127	1.2
10	rs10761482	62085337	A	0.232	0.2717	G	0.127	0.8097
12	rs4930979	31648826	G	0.3866	0.3433	A	0.127	1.205
20	rs2800999	51722623	A	0.4098	0.4542	C	0.127	0.8345
3	rs167771	113876275	G	0.1314	0.1642	A	0.1271	0.7705
6	rs765525	51613177	G	0.3351	0.2925	A	0.1271	1.219
4	rs7689639	25575537	G	0.482	0.4375	A	0.1273	1.196
22	rs5748336	19588535	A	0.482	0.4375	G	0.1273	1.196
1	rs7512414	18807536	A	0.04897	0.07167	C	0.1274	0.667
1	rs34976233	18807755	A	0.04897	0.07167	G	0.1274	0.667
1	rs7515150	18808056	G	0.04897	0.07167	C	0.1274	0.667
3	rs62259044	104640080	G	0.4301	0.475	A	0.1274	0.8341
5	rs13165786	83856400	G	0.4897	0.4442	A	0.1274	1.201
6	rs2841307	100756687	A	0.09794	0.1275	G	0.1274	0.743
6	rs3749894	116600453	A	0.04897	0.07167	G	0.1274	0.667
8	rs2926088	50412162	A	0.1521	0.1875	G	0.1274	0.7771
10	rs117568095	105372696	A	0.09845	0.1285	G	0.1274	0.7403
15	rs2280016	42178122	G	0.2603	0.2217	A	0.1274	1.236
16	rs886427	3023605	G	0.183	0.1492	A	0.1274	1.278
19	rs2433724	54545531	G	0.183	0.1492	A	0.1274	1.278
1	rs6668667	1114668	A	0.05928	0.08347	G	0.1275	0.6919
2	rs7572857	65296798	A	0.05928	0.08417	G	0.1275	0.6857
2	rs1366842	185802243	C	0.09794	0.1283	A	0.1275	0.7374
3	rs9864910	36940741	C	0.4845	0.44	A	0.1275	1.196
3	rs10934853	128038373	A	0.4201	0.465	C	0.1275	0.8335
5	rs2270812	157164962	A	0.1753	0.2125	G	0.1275	0.7875
6	rs3129073	29615822	G	0.3351	0.2933	A	0.1275	1.214
8	rs3736001	143762807	A	0.09794	0.1283	G	0.1275	0.7374
12	rs4964518	107466217	A	0.1753	0.2117	G	0.1275	0.7914
8	rs7460625	100133706	A	0.4871	0.4425	C	0.1276	1.197
18	rs7244306	2185385	A	0.4871	0.4425	G	0.1276	1.197
19	rs4740	4236996	A	0.4871	0.4425	G	0.1276	1.197
2	rs11682222	106801256	G	0.4253	0.4708	A	0.1277	0.8316

5	rs6861681	173362458	A	0.05928	0.08333	G	0.1277	0.6932
6	rs6901158	32205942	A	0.05928	0.08333	G	0.1277	0.6932
6	rs1668702	91138902	A	0.4948	0.4492	G	0.1277	1.201
6	rs629648	128018507	G	0.1263	0.09833	A	0.1277	1.325
12	rs10506867	82666115	G	0.4227	0.4675	A	0.1277	0.8339
19	rs1897820	44981883	C	0.4253	0.4708	G	0.1277	0.8316
1	rs11205303	149906413	G	0.2938	0.2542	A	0.1278	1.221
3	rs342733	32481915	G	0.3918	0.3475	A	0.1278	1.209
3	rs9288803	105132008	A	0.3247	0.3683	G	0.1278	0.8247
7	rs2214326	21856151	G	0.2062	0.1708	A	0.1278	1.261
10	rs2311115	89273579	A	0.3918	0.3475	G	0.1278	1.209
11	rs7110338	124449980	A	0.4974	0.4517	C	0.1278	1.202
16	rs8063330	82627952	G	0.4278	0.4733	A	0.1278	0.832
16	rs12051468	84879464	G	0.2062	0.1708	A	0.1278	1.261
4	rs11099864	153893354	G	0.4923	0.4475	A	0.1279	1.197
3	rs161792	151899704	T	0.2732	0.315	A	0.128	0.8174
6	rs2284190	32819517	G	0.232	0.195	A	0.128	1.247
9	rs4977659	24733226	A	0.232	0.195	G	0.128	1.247
10	rs1775919	29173102	G	0.2732	0.315	A	0.128	0.8174
3	rs10513789	182760073	A	0.3918	0.3483	C	0.1281	1.205
4	rs6852435	175300779	A	0.433	0.4783	G	0.1281	0.8328
6	rs909253	31540313	G	0.4974	0.4525	A	0.1281	1.198
6	rs1041981	31540784	A	0.4974	0.4525	C	0.1281	1.198
11	rs494459	118574675	A	0.5026	0.4567	G	0.1281	1.202
3	rs13060964	167217964	G	0.5	0.455	A	0.1282	1.198
4	rs4689801	7624478	G	0.2345	0.2742	A	0.1282	0.8112
12	rs12816355	83733007	A	0.5052	0.4592	G	0.1282	1.202
17	rs1046896	80685533	A	0.4278	0.4725	G	0.1282	0.8348
20	rs530913	927148	G	0.5052	0.4592	A	0.1282	1.202
6	rs1130399	32629755	A	0.3351	0.2942	G	0.1283	1.209
10	rs11200933	85997105	A	0.1443	0.1142	C	0.1284	1.309
11	rs12223229	57313469	G	0.3273	0.3708	C	0.1284	0.8256
22	rs5751933	25082586	C	0.4381	0.4833	A	0.1284	0.8337
6	rs6901250	117114025	A	0.5103	0.465	G	0.1285	1.199
7	rs6950869	17652915	C	0.5103	0.465	A	0.1285	1.199
2	rs1143627	113594387	G	0.4459	0.4917	A	0.1286	0.8319
3	rs72932959	50336292	G	0.5155	0.47	A	0.1286	1.2
3	rs6766549	72064190	A	0.4407	0.4858	G	0.1286	0.834
7	rs4723388	34942820	G	0.2036	0.2417	A	0.1286	0.8023
11	rs1793004	20698929	G	0.5155	0.47	C	0.1286	1.2
11	rs2183848	30394734	G	0.4485	0.4942	A	0.1286	0.8323
17	rs2074222	7129974	G	0.4433	0.4883	A	0.1286	0.8344
19	rs2253563	44983567	G	0.4485	0.4942	C	0.1286	0.8323

1	rs55747232	16461581	A	0.03608	0.02083	G	0.1287	1.759
3	rs11130248	50352200	G	0.518	0.4725	A	0.1287	1.2
3	rs35455589	50355730	C	0.518	0.4725	A	0.1287	1.2
3	rs2251219	52584787	G	0.5206	0.475	A	0.1287	1.2
7	rs11771145	143110762	G	0.4356	0.48	A	0.1287	0.836
8	rs569214	27487790	C	0.4433	0.4883	A	0.1287	0.8343
16	rs34695957	814942	A	0.03608	0.02083	G	0.1287	1.759
6	rs9394021	30893127	G	0.4459	0.4908	A	0.1288	0.8347
6	rs3873334	30896147	G	0.4459	0.4908	A	0.1288	0.8347
6	rs9399840	104076463	A	0.5309	0.485	G	0.1288	1.202
8	rs2301963	1514009	A	0.5284	0.4825	C	0.1288	1.201
12	rs1985825	110054356	A	0.4562	0.5017	G	0.1288	0.8333
14	rs17124909	89311519	A	0.5258	0.48	C	0.1288	1.201
17	rs4485425	73767437	G	0.5335	0.4875	A	0.1288	1.202
2	rs4572540	192666864	A	0.4613	0.5067	G	0.1289	0.8339
4	rs11939575	187627792	G	0.4613	0.5067	A	0.1289	0.8339
6	rs707913	31084787	G	0.2964	0.2567	A	0.1289	1.22
7	rs10278721	143175345	A	0.2758	0.3183	G	0.1289	0.8154
12	rs9971825	31499671	C	0.5077	0.4633	A	0.1289	1.195
19	rs10406222	39542969	A	0.5077	0.4633	G	0.1289	1.195
19	rs3865452	41211056	G	0.4485	0.4933	A	0.1289	0.8351
21	rs2839158	47641794	A	0.1108	0.085	G	0.1289	1.342
9	rs10869943	80164318	G	0.5155	0.4708	A	0.129	1.196
9	rs2773347	100388197	A	0.5335	0.4883	G	0.129	1.198
9	rs886016	136555629	G	0.5129	0.4683	A	0.129	1.195
13	rs8002779	92015977	A	0.5155	0.4708	G	0.129	1.196
1	rs10802990	241907817	A	0.5232	0.4783	G	0.1291	1.197
3	rs12632110	50224225	A	0.518	0.4733	G	0.1291	1.196
5	rs62346827	12911942	G	0.5206	0.4758	A	0.1291	1.196
6	rs6919443	104493098	A	0.5258	0.4808	G	0.1291	1.197
6	rs685449	153344531	A	0.5232	0.4783	T	0.1291	1.197
17	rs2281726	2236404	A	0.5232	0.4783	G	0.1291	1.197
17	rs2125345	73782191	A	0.518	0.4733	G	0.1291	1.196
1	rs117291160	24401961	C	0.05155	0.03417	G	0.1292	1.536
1	rs76337351	152733376	G	0.05155	0.03417	C	0.1292	1.536
2	rs2077079	85895338	C	0.4562	0.5008	A	0.1292	0.8361
7	rs10247620	31683410	G	0.08505	0.06167	A	0.1292	1.414
7	rs78784885	157959965	A	0.05155	0.03417	G	0.1292	1.536
11	rs12792184	124440617	G	0.08505	0.06167	A	0.1292	1.414
11	rs1384510	7855756	G	0.4588	0.5033	A	0.1293	0.8364
17	rs2290400	38066240	G	0.2964	0.2571	A	0.1293	1.217
4	rs1877731	187628398	C	0.4613	0.5058	G	0.1294	0.8367
8	rs1371867	101330209	C	0.4639	0.5083	A	0.1294	0.837

10	rs1801253	115805056	C	0.1778	0.2142	G	0.1294	0.7937
14	rs268805	58038038	A	0.4639	0.5083	C	0.1294	0.837
1	rs111837270	109394593	C	0.005155	0.01583	A	0.1295	0.3221
1	rs111252541	109394609	T	0.005155	0.01583	A	0.1295	0.3221
2	rs144146961	88484958	A	0.005155	0.01583	G	0.1295	0.3221
2	rs4452212	137015991	A	0.005155	0.01583	G	0.1295	0.3221
3	rs151042112	156763370	A	0.005155	0.01583	G	0.1295	0.3221
4	rs34561493	143043397	A	0.005155	0.01583	G	0.1295	0.3221
5	rs184868814	156533787	G	0.005155	0.01583	A	0.1295	0.3221
8	rs75966225	37702012	A	0.005155	0.01583	G	0.1295	0.3221
8	rs3019327	111285009	G	0.3325	0.3765	A	0.1295	0.825
11	rs117398771	124875006	A	0.005155	0.01583	G	0.1295	0.3221
12	rs585664	53088525	A	0.005155	0.01583	G	0.1295	0.3221
12	rs668313	53091676	A	0.005155	0.01583	G	0.1295	0.3221
17	rs34151057	27869597	A	0.005155	0.01583	G	0.1295	0.3221
19	rs201017975	55756951	G	0.005155	0.01583	C	0.1295	0.3221
20	rs148126308	5903646	A	0.005155	0.01583	G	0.1295	0.3221
11	rs62621284	130784396	A	0.1546	0.1892	G	0.1296	0.7841
11	rs61736758	130784694	A	0.1546	0.1892	G	0.1296	0.7841
21	rs6518291	47821588	G	0.2629	0.225	A	0.1296	1.228
2	rs2252641	145801461	A	0.2345	0.1975	G	0.1297	1.245
5	rs1844437	57320954	C	0.134	0.1667	G	0.1297	0.7738
5	rs1592865	86124692	A	0.3325	0.3758	G	0.1297	0.8272
7	rs10270931	12418964	A	0.3325	0.3758	G	0.1297	0.8272
1	rs7543083	47031340	G	0.3995	0.3558	A	0.1298	1.204
2	rs61748245	165476253	T	0.3969	0.3542	A	0.1298	1.2
11	rs11221332	128380974	A	0.06186	0.0425	G	0.1298	1.485
15	rs77531455	33261288	A	0.06186	0.0425	G	0.1298	1.485
6	rs1332844	12889004	G	0.08505	0.1125	A	0.13	0.7333
10	rs7077408	118228763	G	0.2062	0.245	A	0.13	0.8004
1	rs1055851	236381825	C	0.07216	0.09833	G	0.1301	0.7132
6	rs11968400	31804729	A	0.07216	0.09833	G	0.1301	0.7132
6	rs12210887	31815723	A	0.07216	0.09833	C	0.1301	0.7132
12	rs3803064	113173494	G	0.3428	0.3008	A	0.1301	1.212
3	rs11915082	195809139	A	0.1289	0.1	G	0.1303	1.331
6	rs6457452	31795550	A	0.07216	0.09917	G	0.1303	0.7065
20	rs2076559	25187213	G	0.3351	0.3783	A	0.1303	0.828
3	rs62282002	141162128	G	0.2397	0.28	C	0.1304	0.8107
6	rs4428528	32430362	G	0.2397	0.28	C	0.1304	0.8107
6	rs2187823	32439508	A	0.2397	0.28	G	0.1304	0.8107
16	rs7202925	76587202	C	0.2397	0.28	A	0.1304	0.8107
16	rs7184958	76587331	G	0.2397	0.28	A	0.1304	0.8107
8	rs896378	22262321	A	0.2809	0.3233	G	0.1306	0.8176

19	rs45438992	50455180	A	0.3015	0.2608	G	0.1306	1.223
2	rs11681377	65394714	G	0.2397	0.2792	A	0.1307	0.814
10	rs11016071	129901393	G	0.3428	0.3017	A	0.1307	1.207
10	rs10082391	129901825	G	0.3428	0.3017	A	0.1307	1.207
10	rs1050767	129901946	C	0.3428	0.3017	A	0.1307	1.207
10	rs12777740	129902158	A	0.3428	0.3017	G	0.1307	1.207
4	rs16992035	36285777	A	0.04381	0.0275	G	0.1309	1.62
5	rs421379	91275313	A	0.04381	0.0275	G	0.1309	1.62
6	rs12722042	32605309	G	0.09794	0.07358	A	0.1309	1.367
15	rs2073711	65494212	G	0.1865	0.1533	A	0.1309	1.266
17	rs201679806	78444677	A	0	0.008333	C	0.1309	0
18	rs79313911	48335773	G	0.04381	0.0275	A	0.1309	1.62
1	rs79851686	26314810	A	0.005155	0.01667	G	0.131	0.3057
1	rs1407134	32667609	A	0.005155	0.01667	G	0.131	0.3057
1	rs77848158	112029215	A	0.005155	0.01667	G	0.131	0.3057
1	rs149476106	237058835	G	0.005155	0.01667	A	0.131	0.3057
2	rs479661	169721377	G	0.1005	0.13	A	0.131	0.7479
3	rs181750881	8661567	G	0.005155	0.01667	C	0.131	0.3057
3	rs3864087	8864726	A	0.1804	0.2175	G	0.131	0.7919
4	rs17681870	2702115	G	0.005155	0.01667	A	0.131	0.3057
4	rs117028176	54231541	C	0.005155	0.01667	G	0.131	0.3057
4	rs12512639	63722833	A	0.2655	0.2275	G	0.131	1.227
5	rs17156435	103670162	A	0.005155	0.01667	G	0.131	0.3057
6	rs3130250	29625001	A	0.005155	0.01667	G	0.131	0.3057
6	rs2428504	30151370	G	0.1005	0.1308	A	0.131	0.7424
6	rs2284168	30172233	A	0.1005	0.1308	G	0.131	0.7424
6	rs1264691	30193874	A	0.1005	0.1308	G	0.131	0.7424
6	rs192317605	129588259	A	0.005155	0.01667	C	0.131	0.3057
7	rs191064068	149474827	A	0.005155	0.01667	G	0.131	0.3057
9	rs3905000	107657070	A	0.07216	0.05167	G	0.131	1.428
11	rs151260828	58035062	A	0.005155	0.01667	C	0.131	0.3057
11	rs79337234	58126479	A	0.07216	0.05167	G	0.131	1.428
11	rs61754773	102587037	A	0.005155	0.01667	G	0.131	0.3057
12	rs4763862	12814818	G	0.005155	0.01667	A	0.131	0.3057
14	rs183909467	93290938	C	0.005155	0.01667	G	0.131	0.3057
15	rs189675082	42158022	A	0.005155	0.01667	G	0.131	0.3057
19	rs76213282	6755198	A	0.005155	0.01667	G	0.131	0.3057
20	rs3918396	3651765	A	0.005155	0.01667	G	0.131	0.3057
9	rs589292	127818276	A	0.3015	0.2617	G	0.1311	1.218
1	rs199669988	21890590	A	0	0.008333	G	0.1312	0
1	rs191172721	86200556	A	0	0.008333	G	0.1312	0
1	rs145227602	151214944	A	0	0.008333	G	0.1312	0
1	rs141527662	203025635	G	0	0.008333	A	0.1312	0

2	rs188090321	160181398	A	0	0.008333	G	0.1312	0
3	rs146010173	46009324	G	0	0.008333	A	0.1312	0
3	rs140373134	48669776	A	0	0.008333	G	0.1312	0
3	rs3814400	112991514	A	0	0.008333	G	0.1312	0
4	rs730469	5624670	A	0.3402	0.3842	G	0.1312	0.8266
4	rs34905034	39408762	G	0	0.008333	C	0.1312	0
4	rs200759109	48027129	G	0	0.008333	A	0.1312	0
4	rs144453883	110384735	C	0	0.008333	A	0.1312	0
4	rs149307334	184190300	A	0	0.008333	G	0.1312	0
5	rs17641488	154203121	A	0	0.008333	G	0.1312	0
6	rs6939340	22140004	A	0.3402	0.3842	G	0.1312	0.8266
7	rs202206479	56007584	A	0	0.008333	G	0.1312	0
7	rs142390338	81596485	A	0	0.008333	G	0.1312	0
7	rs138887728	108154911	G	0	0.008333	A	0.1312	0
7	rs200595390	150068439	A	0	0.008333	G	0.1312	0
8	rs199959272	30701271	A	0	0.008333	C	0.1312	0
8	rs10111398	83542128	A	0.3402	0.3842	C	0.1312	0.8266
9	rs79471093	19785979	A	0	0.008333	G	0.1312	0
9	rs185388689	131741542	G	0	0.008333	A	0.1312	0
10	rs201284617	94821918	C	0	0.008333	A	0.1312	0
10	rs185016179	134008357	A	0	0.008333	G	0.1312	0
11	rs184842479	619015	G	0	0.008333	C	0.1312	0
11	rs200088893	823799	G	0	0.008333	C	0.1312	0
11	rs190642059	62381175	G	0	0.008333	A	0.1312	0
12	rs55707601	120263003	G	0	0.008333	C	0.1312	0
14	rs75882726	21555574	A	0	0.008333	G	0.1312	0
15	rs187906946	43821686	G	0	0.008333	A	0.1312	0
16	rs191271054	89782976	A	0	0.008333	G	0.1312	0
17	rs150513105	15983784	A	0	0.008333	G	0.1312	0
19	rs200003896	16268287	A	0	0.008333	G	0.1312	0
19	rs200974277	17895706	C	0	0.008333	A	0.1312	0
19	rs191212936	45506208	A	0	0.008333	G	0.1312	0
19	rs201696237	54649769	C	0	0.008333	A	0.1312	0
19	rs28420516	55869892	A	0	0.008333	G	0.1312	0
19	rs12462795	56569629	G	0.3402	0.3842	C	0.1312	0.8266
12	rs2373011	99961349	C	0.4046	0.3617	G	0.1313	1.2
14	rs891297	21026773	A	0.1134	0.08667	G	0.1314	1.348
18	rs1941141	7630982	A	0.2423	0.2822	G	0.1314	0.8131
19	rs1129763	8121369	A	0.1134	0.08667	G	0.1314	1.348
6	rs4397233	98104948	G	0.3402	0.3829	A	0.1315	0.8309
4	rs3733197	102839287	A	0.1572	0.1925	G	0.1316	0.7825
6	rs2295651	24403377	A	0.1469	0.1167	G	0.1317	1.304
19	rs10403021	30079604	A	0.3041	0.2633	G	0.1317	1.223

10	rs10997975	69933921	A	0.2423	0.2817	G	0.1319	0.8154
16	rs113773794	88787610	G	0.04124	0.0625	A	0.1319	0.6452
6	rs7761777	81092467	G	0.3428	0.3858	A	0.132	0.8302
13	rs3803185	50205025	G	0.2113	0.1758	A	0.132	1.256
6	rs34109614	35748931	G	0.3041	0.2642	A	0.1322	1.217
11	rs7949157	92531356	A	0.2371	0.2008	G	0.1322	1.237
1	rs7530560	161210729	A	0.4098	0.3667	G	0.1323	1.199
9	rs73471186	23425236	A	0.1881	0.1542	G	0.1323	1.271
12	rs17249754	90060586	A	0.4098	0.3667	G	0.1323	1.199
22	rs2074739	31533967	A	0.3479	0.3067	G	0.1323	1.206
1	rs200939032	42049603	A	0.02835	0.01583	G	0.1324	1.814
1	rs139751339	55175770	G	0.02835	0.01583	C	0.1324	1.814
2	rs181260849	113736265	C	0.02835	0.01583	A	0.1324	1.814
2	rs76908853	220103491	G	0.02835	0.01583	A	0.1324	1.814
2	rs3752780	232099979	A	0.02835	0.01583	G	0.1324	1.814
11	rs200518616	372138	A	0.02835	0.01583	G	0.1324	1.814
14	rs79425071	96782965	G	0.02835	0.01583	A	0.1324	1.814
17	rs139209450	63531768	A	0.02835	0.01583	G	0.1324	1.814
19	rs3815780	42213944	C	0.02835	0.01583	A	0.1324	1.814
19	rs17847566	49391323	A	0.02835	0.01583	G	0.1324	1.814
20	rs79298157	239804	G	0.02835	0.01583	A	0.1324	1.814
12	rs7298565	109937534	A	0.3505	0.3083	G	0.1325	1.211
16	rs2270286	8722629	C	0.3505	0.3083	G	0.1325	1.211
2	rs1128272	69173570	A	0.2474	0.2871	G	0.1326	0.8161
1	rs2502342	243070467	A	0.04124	0.06333	G	0.1327	0.6361
6	rs9261829	30380245	G	0.04124	0.06333	A	0.1327	0.6361
6	rs6916716	30385235	A	0.04124	0.06333	C	0.1327	0.6361
6	rs9261895	30389323	A	0.04124	0.06333	G	0.1327	0.6361
6	rs9261919	30391723	A	0.04124	0.06333	G	0.1327	0.6361
6	rs9261947	30394628	A	0.04124	0.06333	G	0.1327	0.6361
6	rs12183946	30418533	A	0.04124	0.06333	G	0.1327	0.6361
2	rs11686934	70172116	A	0.3479	0.3917	G	0.1328	0.8288
3	rs3892715	194758010	A	0.3479	0.3917	G	0.1328	0.8288
10	rs2981582	123352317	A	0.2448	0.2858	G	0.1328	0.8101
1	rs2273863	236706278	A	0.1186	0.1508	C	0.1329	0.7572
1	rs2273865	236706300	A	0.1186	0.1508	T	0.1329	0.7572
6	rs7746553	31895973	C	0.1186	0.1508	G	0.1329	0.7572
10	rs2296436	100179851	G	0.1186	0.1508	A	0.1329	0.7572
13	rs7998427	50050620	A	0.1186	0.1508	G	0.1329	0.7572
21	rs2839227	47786524	G	0.2706	0.2317	A	0.133	1.231
4	rs1478091	131790501	G	0.09794	0.07417	A	0.1331	1.355
16	rs79206939	53860052	A	0.02577	0.04333	G	0.1331	0.584
4	rs2960306	2990499	A	0.1469	0.1175	C	0.1332	1.293

1	rs16846206	173516871	G	0.4175	0.3733	C	0.1333	1.203
10	rs2578084	58684269	G	0.4175	0.3733	A	0.1333	1.203
6	rs1063632	31378510	A	0.1134	0.0875	G	0.1335	1.334
5	rs17681908	75991420	A	0.02577	0.04417	G	0.1336	0.5725
6	rs36016898	107088231	A	0.02577	0.04417	G	0.1336	0.5725
11	rs113908376	26743240	A	0.02577	0.04417	G	0.1336	0.5725
12	rs7297245	96374614	G	0.02577	0.04417	A	0.1336	0.5725
7	rs10229603	112628373	G	0.1598	0.195	A	0.1337	0.7851
13	rs7332388	73349359	C	0.2706	0.2325	G	0.1337	1.225
8	rs931001	70091974	G	0.3531	0.3967	A	0.1338	0.8302
1	rs866484	158986477	C	0.2474	0.2883	G	0.1339	0.8115
5	rs786840	9041935	G	0.06186	0.0875	A	0.1339	0.6876
6	rs6926530	30730267	G	0.06186	0.0875	A	0.1339	0.6876
6	rs11757629	30744529	C	0.06186	0.0875	A	0.1339	0.6876
14	rs10134537	24760764	A	0.06186	0.0875	G	0.1339	0.6876
1	rs2230307	100361925	A	0.2139	0.1783	G	0.134	1.254
11	rs7943316	34460472	A	0.2912	0.3333	T	0.134	0.8218
1	rs3795787	228469904	A	0.3557	0.3133	C	0.1341	1.21
7	rs4298438	108258793	G	0.3067	0.2675	A	0.1341	1.211
5	rs6887694	135035711	G	0.3531	0.3958	A	0.1342	0.8331
6	rs204995	32154285	G	0.2912	0.3325	A	0.1342	0.8249
16	rs1381102	63955447	A	0.2912	0.3325	G	0.1342	0.8249
7	rs11238247	53103554	G	0.3093	0.2692	C	0.1343	1.216
10	rs17116471	105840422	G	0.05155	0.07417	A	0.1343	0.6784
14	rs2110552	73355131	A	0.3557	0.3992	G	0.1343	0.8309
14	rs11624704	78786077	C	0.05155	0.07417	A	0.1343	0.6784
15	rs2306022	68628163	A	0.05155	0.07417	G	0.1343	0.6784
1	rs2228567	40773123	C	0.05155	0.075	G	0.1344	0.6703
13	rs4885962	56863954	C	0.2732	0.2342	A	0.1344	1.229
22	rs1128399	19420109	G	0.05155	0.075	A	0.1344	0.6703
18	rs1120787	54983554	A	0.03351	0.05333	C	0.1345	0.6153
19	rs2304167	55527081	G	0.1392	0.1725	A	0.1345	0.7756
6	rs2517723	29915268	G	0.2173	0.182	A	0.1346	1.248
8	rs920829	72977703	A	0.3557	0.3142	G	0.1346	1.205
2	rs55738533	160086654	A	0.4201	0.3775	G	0.1347	1.195
2	rs151314911	163030296	A	0.03608	0.02167	G	0.1347	1.69
3	rs9855082	80359855	A	0.03351	0.0525	G	0.1347	0.6257
3	rs12490319	114511356	A	0.03608	0.02167	G	0.1347	1.69
5	rs77469944	90445889	G	0.03608	0.02167	A	0.1347	1.69
5	rs117381766	149582191	A	0.03608	0.02167	G	0.1347	1.69
6	rs2395253	32715160	A	0.03608	0.02167	G	0.1347	1.69
6	rs206936	34302869	A	0.3582	0.4025	G	0.1347	0.8287
7	rs2302340	30795331	A	0.3582	0.4025	G	0.1347	0.8287

14	rs139619016	75142909	G	0.03608	0.02167	A	0.1347	1.69
19	rs2240234	3831709	A	0.1392	0.1733	G	0.1347	0.7711
4	rs28532673	122664323	A	0.2938	0.3358	G	0.1348	0.8228
15	rs2306933	29421054	A	0.2423	0.205	G	0.1348	1.24
22	rs55989856	39493294	G	0.07474	0.1008	A	0.1349	0.7203
10	rs3737031	135368590	G	0.2732	0.235	A	0.135	1.224
10	rs3747881	135369532	G	0.2732	0.235	A	0.135	1.224
10	rs8181357	135370639	A	0.2732	0.235	C	0.135	1.224
14	rs80200454	92460227	A	0.07474	0.1017	G	0.135	0.7138
14	rs59635749	92472416	C	0.07474	0.1017	G	0.135	0.7138
15	rs8026929	89169703	G	0.03608	0.0217	C	0.135	1.687
17	rs28944173	26093543	G	0.03608	0.0217	A	0.135	1.687
8	rs10109853	39862881	A	0.3093	0.27	G	0.1351	1.211
3	rs4679639	60134540	A	0.3608	0.4042	C	0.1353	0.8322
7	rs143816381	2752322	A	0.03608	0.02174	G	0.1353	1.684
15	rs7181630	29443474	C	0.3608	0.4042	A	0.1353	0.8322
5	rs6886336	70806958	G	0.1314	0.1033	A	0.1355	1.313
7	rs362691	103251161	C	0.1314	0.1033	G	0.1355	1.313
11	rs7945466	96571163	G	0.4253	0.3825	A	0.1355	1.194
6	rs9490809	123869607	G	0.1005	0.07583	C	0.1356	1.362
7	rs12669698	37437969	G	0.3634	0.4075	A	0.1356	0.83
11	rs16928809	2936952	A	0.1005	0.07583	G	0.1356	1.362
21	rs2075876	45709153	A	0.4304	0.3865	G	0.1356	1.2
1	rs1485471	29194901	A	0.08763	0.06417	G	0.1358	1.401
6	rs1084651	161089817	A	0.3634	0.4067	G	0.1358	0.8329
3	rs1137524	47956424	A	0.2526	0.2933	G	0.136	0.8141
5	rs254893	171076227	A	0.116	0.08917	G	0.136	1.34
8	rs7813708	124206324	A	0.2526	0.2933	G	0.136	0.8141
15	rs34949187	89386652	A	0.06443	0.04417	G	0.136	1.49
16	rs2967340	82257551	G	0.3608	0.3192	A	0.136	1.204
18	rs1440827	27378023	G	0.2526	0.2933	A	0.136	0.8141
10	rs2254419	134623959	A	0.3144	0.2738	G	0.1361	1.217
2	rs10495671	18044028	A	0.4304	0.3875	G	0.1362	1.194
2	rs201018300	31758677	A	0.002577	0.01169	G	0.1362	0.2185
3	rs3796360	119134872	A	0.05412	0.03583	G	0.1362	1.54
4	rs4975017	39450229	A	0.366	0.4092	C	0.1362	0.8335
14	rs1760898	20872881	A	0.4304	0.3875	C	0.1362	1.194
14	rs2230500	61924239	A	0.1881	0.225	G	0.1362	0.7982
16	rs7191955	20238586	G	0.4304	0.3875	A	0.1362	1.194
19	rs1610117	9111508	C	0.4304	0.3875	A	0.1362	1.194
22	rs190547628	41077832	A	0.002577	0.01169	G	0.1362	0.2185
1	rs185178501	27219004	A	0.002577	0.01167	G	0.1363	0.2189
1	rs143906646	153333264	A	0.002577	0.01167	G	0.1363	0.2189

1	rs2275481	153958618	G	0.002577	0.01167	A	0.1363	0.2189
1	rs138263364	167973797	G	0.002577	0.01167	A	0.1363	0.2189
2	rs115359066	158390453	A	0.002577	0.01167	G	0.1363	0.2189
2	rs34546065	171371469	A	0.002577	0.01167	G	0.1363	0.2189
2	rs56052422	171375968	A	0.002577	0.01167	G	0.1363	0.2189
3	rs76431673	125690885	A	0.002577	0.01167	G	0.1363	0.2189
3	rs138410529	169835124	G	0.002577	0.01167	A	0.1363	0.2189
3	rs2271822	196865191	A	0.002577	0.01167	G	0.1363	0.2189
5	rs17150488	101774455	G	0.002577	0.01167	A	0.1363	0.2189
5	rs78336563	149500767	A	0.002577	0.01167	G	0.1363	0.2189
6	rs144678437	159398691	G	0.002577	0.01167	A	0.1363	0.2189
7	rs191266281	94540709	C	0.002577	0.01167	A	0.1363	0.2189
7	rs74534379	100683993	A	0.002577	0.01167	C	0.1363	0.2189
8	rs145475640	121061929	A	0.002577	0.01167	G	0.1363	0.2189
8	rs117646413	145619133	A	0.002577	0.01167	G	0.1363	0.2189
9	rs137971910	35555393	A	0.002577	0.01167	G	0.1363	0.2189
11	rs325609	6221249	G	0.002577	0.01167	A	0.1363	0.2189
11	rs200312339	19259598	A	0.002577	0.01167	G	0.1363	0.2189
11	rs199986876	66083511	A	0.002577	0.01167	G	0.1363	0.2189
12	rs183923514	122326793	A	0.002577	0.01167	G	0.1363	0.2189
16	rs147528125	31426259	A	0.002577	0.01167	C	0.1363	0.2189
16	rs145090795	72130820	T	0.002577	0.01167	A	0.1363	0.2189
17	rs190019051	29622668	G	0.002577	0.01167	A	0.1363	0.2189
17	rs118191745	38241000	G	0.002577	0.01167	A	0.1363	0.2189
19	rs16995376	15784377	A	0.002577	0.01167	G	0.1363	0.2189
20	rs114917981	35857025	A	0.002577	0.01167	G	0.1363	0.2189
2	rs4853575	192160875	G	0.3634	0.3208	A	0.1364	1.208
3	rs139176068	111842471	A	0.02356	0.01167	G	0.1364	2.044
20	rs3208008	62326110	C	0.299	0.3408	A	0.1364	0.8248
9	rs28515121	136291594	A	0.1701	0.1383	G	0.1365	1.277
22	rs5762311	28090207	G	0.433	0.39	A	0.1366	1.194
6	rs41559420	32487242	A	0.002618	0.01176	G	0.1367	0.2205
12	rs2856321	11855773	G	0.299	0.34	A	0.1367	0.8279
12	rs1463630	116060432	A	0.3686	0.4117	G	0.1367	0.8342
5	rs860413	35943042	C	0.2165	0.1817	A	0.1368	1.245
6	rs6941421	15089151	G	0.3634	0.3217	A	0.1368	1.204
4	rs79167802	155719189	C	0.1907	0.1583	A	0.1369	1.253
12	rs10860341	98936974	A	0.4381	0.3942	C	0.1369	1.199
12	rs2075387	114282496	A	0.1907	0.1583	G	0.1369	1.253
13	rs1805107	28537317	G	0.1907	0.1583	A	0.1369	1.253
7	rs35272066	97874693	A	0.4356	0.3925	G	0.137	1.194
15	rs11073795	89180778	A	0.4356	0.3925	G	0.137	1.194
1	rs2146315	42050366	A	0.3015	0.3442	G	0.1371	0.8227

1	rs12087666	244334507	G	0.3711	0.4142	A	0.1371	0.8348
8	rs10107472	4190874	A	0.2191	0.2583	G	0.1371	0.8054
11	rs613686	77882393	G	0.2552	0.2958	A	0.1371	0.8154
12	rs4765180	125318772	A	0.2552	0.2958	G	0.1371	0.8154
20	rs2243603	1546911	G	0.2448	0.2083	C	0.1371	1.232
5	rs17157450	104356919	G	0.4407	0.3967	A	0.1373	1.199
6	rs3734804	151939181	A	0.3737	0.4175	G	0.1373	0.8325
7	rs12666072	21901593	A	0.2758	0.2383	G	0.1373	1.217
10	rs7076247	18759629	A	0.2784	0.24	G	0.1376	1.221
11	rs17150664	56237487	A	0.04381	0.02833	G	0.1376	1.571
22	rs136478	32589023	A	0.1314	0.1042	G	0.1376	1.301
22	rs108211	32590449	A	0.1314	0.1042	G	0.1376	1.301
1	rs1334124	106774642	A	0.4433	0.3992	G	0.1377	1.199
16	rs8059366	13351740	G	0.4433	0.3992	A	0.1377	1.199
2	rs11677877	228131169	G	0.1057	0.1358	A	0.1378	0.7517
4	rs2602836	100014805	A	0.1649	0.2	G	0.1378	0.7901
11	rs605734	56237722	T	0.1057	0.1363	A	0.1378	0.7488
13	rs3752472	33629393	A	0.09021	0.1183	G	0.1378	0.7387
2	rs3731631	25359422	A	0.3041	0.3467	G	0.1379	0.8236
10	rs1880676	50824117	A	0.1933	0.16	G	0.1379	1.258
11	rs11023769	15921354	A	0.3041	0.3467	G	0.1379	0.8236
15	rs8451	101109677	A	0.1907	0.2275	C	0.1379	0.8002
15	rs12157	101109794	C	0.1907	0.2275	G	0.1379	0.8002
17	rs10852891	7490810	A	0.1933	0.16	G	0.1379	1.258
1	rs12046765	213743905	G	0.4433	0.4	A	0.138	1.194
6	rs9371533	150210681	G	0.116	0.09	A	0.138	1.327
10	rs2228638	33475282	A	0.116	0.09	G	0.138	1.327
11	rs4144901	108044091	T	0.2165	0.1825	A	0.138	1.238
11	rs2282602	133714522	G	0.3763	0.4192	A	0.138	0.836
19	rs76520998	36230247	C	0.116	0.09	G	0.138	1.327
2	rs191610670	152544878	A	0.0232	0.01167	G	0.1381	2.012
2	rs1440072	223936738	G	0.0232	0.01167	A	0.1381	2.012
4	rs36094464	88533540	T	0.0232	0.01167	A	0.1381	2.012
4	rs201172719	123747947	A	0.0232	0.01167	G	0.1381	2.012
6	rs2855442	33137403	G	0.0232	0.01167	A	0.1381	2.012
6	rs2855425	33144373	G	0.0232	0.01167	A	0.1381	2.012
11	rs171271	126203911	G	0.317	0.2775	A	0.1381	1.208
11	rs117994299	129993553	A	0.0232	0.01167	G	0.1381	2.012
12	rs76634522	13061553	A	0.0232	0.01167	G	0.1381	2.012
16	rs190278786	71509962	A	0.0232	0.01167	G	0.1381	2.012
17	rs140920850	72249389	A	0.0232	0.01167	C	0.1381	2.012
19	rs77942672	19770415	C	0.0232	0.01167	G	0.1381	2.012
20	rs41282984	61512185	G	0.0232	0.01167	C	0.1381	2.012

2	rs2034648	236957822	G	0.1907	0.2267	A	0.1382	0.804
4	rs2085621	56044260	A	0.1907	0.2267	G	0.1382	0.804
13	rs12585626	103392562	A	0.09021	0.1192	C	0.1382	0.7329
15	rs11247226	101120963	G	0.1907	0.2267	A	0.1382	0.804
17	rs9901673	7484101	A	0.1933	0.1603	C	0.1382	1.255
1	rs588098	75716925	G	0.08763	0.065	A	0.1383	1.382
1	rs857705	158687896	A	0.1521	0.1217	G	0.1384	1.295
3	rs16834826	123584488	G	0.4485	0.4042	A	0.1384	1.199
5	rs11435	145442133	A	0.2216	0.2608	G	0.1384	0.807
6	rs423023	32188297	C	0.2216	0.26	G	0.1384	0.8105
7	rs3735615	20180717	G	0.1521	0.1217	C	0.1384	1.295
10	rs3740093	45499009	G	0.1005	0.07667	A	0.1384	1.346
20	exm-IND20-3367	34215235	I	0.1005	0.07667	D	0.1384	1.346
20	rs12481228	34218673	C	0.1005	0.07667	G	0.1384	1.346
22	rs136175	36661566	G	0.1521	0.1217	A	0.1384	1.295
7	rs7789940	75951230	G	0.3814	0.425	A	0.1385	0.8343
7	rs7779014	75975586	A	0.3814	0.425	G	0.1385	0.8343
7	rs17149161	75978229	A	0.3814	0.425	C	0.1385	0.8343
7	rs13438255	155665835	G	0.3814	0.425	A	0.1385	0.8343
2	rs116911250	220337041	A	0.07474	0.05417	G	0.1386	1.411
3	rs9858333	27919338	G	0.4485	0.405	A	0.1386	1.195
4	rs698	100260789	G	0.07474	0.05417	A	0.1386	1.411
4	rs1693482	100263965	A	0.07474	0.05417	G	0.1386	1.411
4	rs1789924	100274286	A	0.07474	0.05417	G	0.1386	1.411
5	rs2291628	127609633	A	0.07474	0.05417	G	0.1386	1.411
3	rs2282751	50291785	G	0.384	0.4283	A	0.1387	0.832
16	rs10852667	2982708	C	0.384	0.4283	A	0.1387	0.832
5	rs6595320	121100484	G	0.384	0.4274	A	0.1388	0.8353
12	rs12146727	7170336	A	0.06443	0.08917	G	0.1388	0.7035
5	rs397287	9463291	C	0.451	0.4075	A	0.1389	1.195
7	rs758944	75953297	A	0.3814	0.4242	C	0.1389	0.8372
4	rs17067488	180280410	G	0.3866	0.4308	A	0.1391	0.8326
7	rs10237193	49836460	C	0.2824	0.2442	A	0.1391	1.218
2	rs10496702	134005569	A	0.1933	0.1608	G	0.1392	1.25
13	rs9578586	23953924	A	0.4485	0.4058	G	0.1392	1.19
1	rs41284333	26517267	G	0.002577	0.0125	A	0.1393	0.2041
1	rs11247866	26517794	G	0.002577	0.0125	A	0.1393	0.2041
1	rs11809207	26521140	A	0.002577	0.0125	G	0.1393	0.2041
1	rs17257155	26526439	G	0.002577	0.0125	A	0.1393	0.2041
1	rs11542623	46746164	A	0.3093	0.3517	G	0.1393	0.8255
1	rs718265	55319902	G	0.4536	0.41	A	0.1393	1.195
4	rs143623936	48533228	G	0.002577	0.0125	A	0.1393	0.2041
6	rs3135461	32680122	G	0.1727	0.1408	A	0.1393	1.273

6	rs36215251	152470773	C	0.002577	0.0125	A	0.1393	0.2041
9	rs4838334	128843105	G	0.4536	0.41	A	0.1393	1.195
11	rs140444692	34502470	C	0.002577	0.0125	G	0.1393	0.2041
11	rs9332838	118373677	G	0.002577	0.0125	C	0.1393	0.2041
11	rs149770693	119050849	A	0.002577	0.0125	G	0.1393	0.2041
14	rs118094356	102675736	A	0.002577	0.0125	G	0.1393	0.2041
16	rs2685127	335373	A	0.002577	0.0125	G	0.1393	0.2041
16	rs144944402	30750212	G	0.002577	0.0125	C	0.1393	0.2041
17	rs2273983	2227988	G	0.002577	0.0125	A	0.1393	0.2041
17	rs202169133	79983359	A	0.002577	0.0125	G	0.1393	0.2041
19	rs150407533	57670570	A	0.002577	0.0125	C	0.1393	0.2041
22	rs3736689	50573036	G	0.002577	0.0125	A	0.1393	0.2041
1	rs7520453	246670481	G	0.06443	0.09	C	0.1394	0.6964
2	rs13392835	6500101	G	0.3892	0.4333	A	0.1394	0.8332
4	rs17026725	98333918	G	0.134	0.1058	A	0.1394	1.308
6	rs452930	94922837	A	0.3892	0.4333	G	0.1394	0.8332
9	rs57728614	129958862	A	0.134	0.1058	C	0.1394	1.308
10	rs2480345	25701341	G	0.06443	0.09	C	0.1394	0.6964
10	rs10901425	127462525	A	0.3222	0.2817	G	0.1394	1.212
16	rs28415940	89294381	A	0.3222	0.2817	G	0.1394	1.212
4	rs1589163	73472941	A	0.3711	0.33	G	0.1395	1.198
4	rs7375925	134384370	G	0.3892	0.4325	A	0.1396	0.836
2	rs1035822	145255778	A	0.07732	0.1042	G	0.1397	0.7207
13	rs534870	80959207	G	0.3918	0.4358	A	0.1397	0.8337
6	rs9261485	30108751	A	0.2474	0.2117	G	0.1398	1.224
20	rs2295490	368905	G	0.2191	0.185	A	0.1399	1.236
1	rs11165846	97819667	C	0.4562	0.4133	G	0.14	1.191
2	rs4952125	30210861	G	0.4562	0.4133	A	0.14	1.191
4	rs1431005	188345368	A	0.3943	0.4383	G	0.14	0.8343
16	rs140871153	315008	G	0.04381	0.065	A	0.1401	0.6591
6	rs2297336	44253765	G	0.3119	0.3533	A	0.1402	0.8294
6	rs324146	44255459	G	0.3119	0.3533	A	0.1402	0.8294
10	rs556165	18266053	A	0.3763	0.3342	C	0.1402	1.202
6	rs9405666	431891	A	0.3969	0.4408	G	0.1403	0.8348
7	rs2075820	30492237	A	0.3943	0.4375	G	0.1403	0.8371
4	rs8396	159630817	G	0.2216	0.1867	A	0.1406	1.241
6	rs2295663	31669295	G	0.1186	0.09167	A	0.1406	1.333
1	rs78202695	241761225	A	0.02062	0.03667	G	0.1407	0.5531
4	rs16869924	10567732	G	0.04381	0.06583	A	0.1407	0.6502
4	rs12504074	159158677	A	0.04381	0.06583	G	0.1407	0.6502
11	rs7232	59940599	T	0.04381	0.06583	A	0.1407	0.6502
12	rs3759251	10954383	T	0.02062	0.03667	A	0.1407	0.5531
12	rs76112700	10954632	A	0.02062	0.03667	C	0.1407	0.5531

12	rs77050900	10954916	G	0.02062	0.03667	A	0.1407	0.5531
12	rs74611066	10959438	A	0.02062	0.03667	G	0.1407	0.5531
12	rs77609577	10961978	A	0.02062	0.03667	G	0.1407	0.5531
22	rs16989291	31971282	G	0.02062	0.03667	A	0.1407	0.5531
9	rs116894068	139753246	A	0.05412	0.03667	G	0.1408	1.503
10	rs34204285	26482157	G	0.05412	0.03667	A	0.1408	1.503
12	rs734856	119656333	A	0.3763	0.335	C	0.1408	1.198
14	rs2232699	94756458	A	0.05412	0.03667	T	0.1408	1.503
20	rs5743509	36940314	A	0.05412	0.03667	G	0.1408	1.503
21	rs239049	17654621	G	0.3763	0.335	A	0.1408	1.198
3	rs1463525	174961387	G	0.3789	0.3367	A	0.1409	1.202
8	rs2978263	30498859	A	0.4021	0.4458	G	0.1409	0.8358
12	rs3213831	9316773	G	0.3995	0.4425	A	0.1409	0.8381
13	rs7995033	25831888	A	0.4691	0.425	G	0.1409	1.195
1	rs2275531	207109116	A	0.2835	0.2458	G	0.141	1.214
2	rs1399645	140147832	A	0.3144	0.3558	G	0.141	0.8303
9	rs2297776	34372931	T	0.1031	0.07833	A	0.141	1.352
13	rs9537793	58331407	A	0.4665	0.4233	G	0.141	1.191
1	rs10923574	119030698	A	0.2268	0.265	C	0.1411	0.8136
4	rs1047642	170663235	G	0.05412	0.07667	A	0.1411	0.6891
4	rs13122912	170676328	G	0.05412	0.0775	A	0.1411	0.6811
10	rs10794720	1156165	A	0.1082	0.1383	G	0.1411	0.7561
12	rs7954976	51681903	A	0.05412	0.07667	G	0.1411	0.6891
15	rs16974569	85399645	A	0.05412	0.07667	G	0.1411	0.6891
22	rs2076212	44322970	A	0.05412	0.07667	C	0.1411	0.6891
6	rs2246638	29949497	A	0.2655	0.3058	G	0.1412	0.8203
6	rs592229	31930441	C	0.4716	0.4275	A	0.1412	1.195
10	rs10882029	93955851	A	0.4021	0.445	G	0.1413	0.8386
3	rs66475108	138678130	G	0.1263	0.1575	A	0.1414	0.7732
8	rs7007913	99399509	G	0.1263	0.1575	A	0.1414	0.7732
9	rs16927557	1325179	A	0.3273	0.2867	G	0.1414	1.211
11	rs10896488	56344260	G	0.1263	0.1575	A	0.1414	0.7732
12	rs10744218	122942185	A	0.317	0.3592	G	0.1414	0.8281
1	rs2275558	164529120	G	0.2655	0.305	A	0.1415	0.8235
6	rs2394734	30046246	A	0.2655	0.305	G	0.1415	0.8235
11	rs540874	66329732	A	0.4768	0.4325	G	0.1416	1.196
19	rs8106130	54598604	C	0.1546	0.1242	A	0.1416	1.29
2	rs10192667	15284746	G	0.4098	0.4533	A	0.1417	0.8373
3	rs2800	143424822	A	0.4098	0.4533	G	0.1417	0.8373
6	rs6921041	136370658	C	0.317	0.3583	A	0.1417	0.8312
6	rs6916394	31464050	G	0.4922	0.4491	A	0.1418	1.189
6	rs9268474	32357165	G	0.1469	0.18	A	0.1418	0.7845
6	rs2076522	32371179	G	0.1469	0.18	C	0.1418	0.7845

6	rs3763316	32376746	A	0.1469	0.18	G	0.1418	0.7845
10	rs1801222	17156151	A	0.1469	0.1808	G	0.1418	0.7801
19	rs1138439	282753	A	0.1469	0.18	G	0.1418	0.7845
6	rs4712990	26413744	A	0.02062	0.0375	C	0.1419	0.5404
6	rs115402675	43970658	T	0.02062	0.0375	A	0.1419	0.5404
11	rs1671064	66327673	G	0.4768	0.4333	A	0.1419	1.192
11	rs1815739	66328095	A	0.4768	0.4333	G	0.1419	1.192
11	rs618838	66328719	A	0.4768	0.4333	G	0.1419	1.192
12	rs7303282	25432626	A	0.3273	0.2875	G	0.1419	1.206
16	rs7199765	27966405	A	0.4768	0.4333	G	0.1419	1.192
16	rs17281813	49750790	G	0.1701	0.2058	A	0.1419	0.7908
20	rs4289250	36921857	A	0.4768	0.4333	G	0.1419	1.192
11	rs2298813	121393684	A	0.1753	0.1433	G	0.142	1.27
12	rs59626664	56636975	C	0.02062	0.03756	G	0.142	0.5394
4	rs17031394	155219662	C	0.09278	0.1217	G	0.1421	0.7383
7	rs3735478	44800176	A	0.09278	0.1217	C	0.1421	0.7383
9	rs647128	138015713	G	0.4149	0.4592	A	0.1421	0.8354
11	rs10793183	76420924	A	0.4794	0.4358	C	0.1421	1.192
12	rs10877331	40084126	A	0.4794	0.4358	G	0.1421	1.192
1	rs11207949	62732421	A	0.4098	0.4525	G	0.1422	0.8401
12	rs11178755	71741064	G	0.268	0.3083	A	0.1422	0.8215
11	rs1626154	72946204	A	0.09021	0.06667	G	0.1423	1.388
11	rs7927064	123261385	G	0.09021	0.06667	A	0.1423	1.388
19	rs58542926	19379549	A	0.09021	0.06667	G	0.1423	1.388
19	rs445925	45415640	A	0.09021	0.06667	G	0.1423	1.388
22	rs7291216	51156078	A	0.4149	0.4583	G	0.1423	0.8382
4	rs3811833	153896358	G	0.4768	0.4342	A	0.1424	1.188
6	rs9388768	130374102	C	0.2294	0.2683	A	0.1424	0.8116
9	rs4877678	84452655	G	0.4768	0.4342	A	0.1424	1.188
16	rs1054645	1270162	G	0.1186	0.0925	A	0.1424	1.32
6	rs6899976	130358428	G	0.2294	0.2675	A	0.1425	0.8151
8	rs2472553	27328511	G	0.4201	0.4642	A	0.1425	0.8363
2	rs10455	172411273	A	0.384	0.3425	G	0.1426	1.197
8	rs2748404	142505578	A	0.4871	0.4433	C	0.1426	1.193
2	rs6708185	217285809	A	0.4201	0.4633	G	0.1427	0.8391
6	rs1355800	67238099	G	0.2526	0.2167	A	0.1428	1.222
13	rs11616593	19729760	G	0.4897	0.4458	A	0.1428	1.193
15	rs10468017	58678512	A	0.2526	0.2167	G	0.1428	1.222
17	rs16522	37316988	G	0.2526	0.2167	A	0.1428	1.222
20	rs236110	5933108	A	0.1546	0.125	C	0.1428	1.28
1	rs3811478	230469505	A	0.4845	0.4417	G	0.1429	1.188
5	rs2043112	38955796	G	0.4923	0.4483	A	0.1429	1.193
5	rs757647	137707315	G	0.4871	0.4441	A	0.1429	1.189

6	rs614570	52319251	G	0.4845	0.4417	A	0.1429	1.188
14	rs8013117	68089430	A	0.4845	0.4417	G	0.1429	1.188
21	rs2835369	37885287	A	0.4845	0.4417	G	0.1429	1.188
6	rs7773481	4604376	G	0.4227	0.4658	A	0.143	0.8395
14	rs12881368	33979974	G	0.4871	0.4442	A	0.143	1.189
7	rs2717351	19019880	A	0.4278	0.4717	G	0.1431	0.8376
6	rs210192	33481204	G	0.1366	0.1083	A	0.1432	1.302
13	rs9548115	38520144	G	0.4974	0.4533	A	0.1432	1.194
19	rs958305	38189440	G	0.3325	0.2917	A	0.1433	1.21
4	rs2346648	63895857	G	0.3892	0.3467	A	0.1435	1.201
12	rs10880568	44196407	A	0.3247	0.3667	G	0.1435	0.8307
16	rs6500744	6113661	G	0.4974	0.4542	A	0.1435	1.19
2	rs955949	76918322	G	0.5052	0.4608	A	0.1436	1.194
6	rs2048327	160863532	G	0.5052	0.4608	A	0.1436	1.194
8	rs4735133	110437384	G	0.4356	0.4792	A	0.1436	0.8388
1	rs1571147	199441521	G	0.4407	0.485	A	0.1437	0.8368
3	rs993691	140120742	G	0.3325	0.2925	A	0.1437	1.205
5	rs1063499	40955561	C	0.4407	0.485	G	0.1437	0.8368
6	rs1633005	29764472	A	0.1031	0.07917	G	0.1437	1.337
10	rs10904481	5415954	G	0.5026	0.4592	A	0.1437	1.19
10	rs4934956	38814815	A	0.5026	0.4592	G	0.1437	1.19
16	rs11075945	72747560	A	0.5026	0.4592	G	0.1437	1.19
18	rs2276199	43568717	G	0.4381	0.4817	A	0.1437	0.8392
2	rs8179206	27720442	G	0.03608	0.0225	A	0.1438	1.626
3	rs3732380	39307562	A	0.03608	0.0225	G	0.1438	1.626
5	rs6880003	107332887	A	0.1727	0.2083	G	0.1438	0.7931
7	rs10488004	90758236	G	0.4433	0.4875	A	0.1438	0.8371
18	rs10438933	29273129	G	0.03608	0.0225	A	0.1438	1.626
20	rs200752	15599531	A	0.03608	0.0225	G	0.1438	1.626
6	rs4713325	30383442	G	0.2912	0.2525	C	0.1439	1.216
9	rs4146960	85627408	G	0.2912	0.2525	A	0.1439	1.216
15	rs25653	90349558	A	0.4459	0.49	G	0.1439	0.8375
9	rs1796993	75315438	A	0.4485	0.4925	G	0.144	0.8378
13	rs7319045	92024574	G	0.5155	0.4717	A	0.144	1.192
3	rs2276774	122646828	G	0.06443	0.04583	A	0.1441	1.434
6	rs6986	30313340	C	0.1495	0.1825	G	0.1441	0.7873
6	rs17411795	66005857	A	0.06443	0.04583	T	0.1441	1.434
7	rs849084	156353029	A	0.4536	0.4975	G	0.1441	0.8385
11	rs77726581	6239890	A	0.06443	0.04583	G	0.1441	1.434
14	rs753361	89574193	G	0.451	0.495	A	0.1441	0.8382
15	rs2472299	75033400	A	0.3273	0.3692	G	0.1441	0.8315
20	rs1127497	44038574	A	0.451	0.495	G	0.1441	0.8382
1	rs2275687	236717926	A	0.5335	0.4892	G	0.1442	1.194

1	rs1126627	236718620	A	0.5335	0.4892	G	0.1442	1.194
1	rs1885533	236719193	A	0.5335	0.4892	G	0.1442	1.194
2	rs6574	71376999	A	0.2732	0.3133	G	0.1442	0.8238
2	rs847622	71860893	A	0.5335	0.4892	G	0.1442	1.194
2	rs6747453	143261959	G	0.4381	0.4808	A	0.1442	0.842
3	rs2931403	19961330	A	0.2732	0.313	G	0.1442	0.8249
5	rs11133637	11087563	A	0.4562	0.5	G	0.1442	0.8389
6	rs2596530	31387373	G	0.1295	0.1617	A	0.1442	0.7717
11	rs12577263	107874113	G	0.4665	0.5108	A	0.1442	0.8373
16	rs2865531	75390316	T	0.4381	0.4808	A	0.1442	0.842
17	rs78943308	34958598	C	0.1289	0.1608	G	0.1442	0.7718
19	rs34440547	15582863	A	0.2732	0.3142	G	0.1442	0.8206
19	rs4517902	29851078	A	0.5232	0.4792	C	0.1442	1.193
20	rs6111221	16629423	G	0.4485	0.4917	A	0.1442	0.8406
20	rs6022758	52425620	G	0.4485	0.4917	A	0.1442	0.8406
2	rs2074955	158977794	G	0.2552	0.2192	A	0.1443	1.22
3	rs79533362	133342223	A	0.03608	0.05583	G	0.1443	0.633
6	rs241424	32804934	G	0.4588	0.5025	A	0.1443	0.8392
10	rs34997494	53822301	G	0.03608	0.05583	A	0.1443	0.633
16	rs7191888	73581058	G	0.0799	0.1067	A	0.1443	0.7272
19	rs141236309	43433687	A	0.03608	0.02254	C	0.1443	1.623
20	rs2269010	17415418	G	0.4588	0.5025	A	0.1443	0.8392
3	rs4518168	97806616	A	0.5155	0.4725	G	0.1444	1.188
7	rs7806224	32273107	A	0.5103	0.4675	G	0.1444	1.187
8	rs6511	101252680	A	0.4639	0.5075	G	0.1444	0.8398
9	rs581080	15305378	C	0.06701	0.09167	G	0.1444	0.7117
10	rs2275047	99439541	C	0.451	0.4942	G	0.1444	0.841
14	rs3751501	23549285	A	0.06701	0.09167	G	0.1444	0.7117
16	rs9924855	60598579	A	0.5284	0.485	G	0.1444	1.19
19	rs12460535	49098750	G	0.5284	0.485	A	0.1444	1.19
20	rs2889732	31676804	A	0.2268	0.1917	C	0.1444	1.237
3	rs4894796	171780763	G	0.5206	0.4775	A	0.1445	1.188
11	rs1905055	56113516	A	0.1108	0.1408	G	0.1445	0.7604
11	rs10896290	56128081	G	0.1108	0.1408	A	0.1445	0.7604
1	rs1891460	205814497	A	0.3351	0.295	G	0.1446	1.204
3	rs6777876	11956598	G	0.09021	0.0675	A	0.1446	1.37
2	rs315952	113890304	A	0.3299	0.3725	G	0.1447	0.8293
3	rs2369955	132693588	C	0.1778	0.1458	A	0.1447	1.267
6	rs1611196	29757995	G	0.1778	0.1458	A	0.1447	1.267
12	rs1564374	58010163	A	0.1778	0.1458	G	0.1447	1.267
12	rs6581895	69842643	G	0.201	0.1675	A	0.1447	1.251
3	rs6776064	196435534	A	0.3299	0.3717	G	0.1448	0.8323
17	rs12451299	48432324	C	0.06701	0.0925	G	0.1448	0.7046

8	rs954295	58306018	C	0.1572	0.1267	A	0.1449	1.286
1	rs525410	183176430	A	0.3943	0.3525	G	0.145	1.196
1	rs192734594	207083135	G	0.01031	0.02333	C	0.145	0.436
7	rs76638693	12689137	A	0.01031	0.02333	G	0.145	0.436
11	rs3740853	94731759	G	0.3943	0.3525	A	0.145	1.196
12	rs201585352	11214634	C	0.01031	0.02333	A	0.145	0.436
12	rs1669413	11338781	C	0.01031	0.02333	A	0.145	0.436
12	rs3829295	77423619	G	0.01031	0.02333	A	0.145	0.436
13	rs2479924	70756313	C	0.3943	0.3525	A	0.145	1.196
19	rs2285412	36109312	A	0.01031	0.02333	C	0.145	0.436
19	rs2285418	36133528	C	0.01031	0.02333	A	0.145	0.436
1	rs10803354	15438990	G	0.2938	0.255	A	0.1451	1.216
17	rs7216389	38069949	G	0.2938	0.255	A	0.1451	1.216
1	rs116333252	175130119	G	0.01031	0.0225	A	0.1452	0.4525
4	rs192169063	89020503	G	0.01031	0.0225	A	0.1452	0.4525
16	rs185235292	336716	A	0.01031	0.0225	G	0.1452	0.4525
16	rs80158709	420079	A	0.01031	0.0225	G	0.1452	0.4525
22	rs118094737	22869528	A	0.01031	0.0225	C	0.1452	0.4525
1	rs3856154	225565015	A	0.2268	0.1925	G	0.1454	1.23
3	rs150644559	52522297	A	0.02835	0.04583	G	0.1454	0.6074
6	rs9262113	30532278	A	0.02835	0.04583	G	0.1454	0.6074
10	rs11556392	99439556	C	0.02835	0.04583	A	0.1454	0.6074
16	rs144651842	27356224	A	0.02835	0.04583	G	0.1454	0.6074
5	rs1022044	174897200	A	0.3376	0.2975	G	0.1455	1.204
6	rs783396	106987370	A	0.03608	0.05667	C	0.1455	0.6232
17	rs917361	10355763	A	0.1366	0.1093	G	0.1455	1.289
19	rs34752754	17766900	A	0.03608	0.05667	G	0.1455	0.6232
2	rs744373	127894615	G	0.3943	0.3533	A	0.1456	1.192
2	rs75671160	131487692	A	0.02835	0.04667	G	0.1456	0.5961
4	rs59591720	15964349	A	0.04639	0.03	C	0.1456	1.573
6	rs130075	31122502	A	0.02835	0.04667	G	0.1456	0.5961
6	rs4839989	99267877	C	0.3943	0.3533	A	0.1456	1.192
13	rs9600179	74440000	G	0.04639	0.03	A	0.1456	1.573
14	rs74913644	105609836	A	0.02835	0.04667	G	0.1456	0.5961
2	rs145283195	1312289	A	0.01546	0.03	C	0.1458	0.5079
3	rs34823813	49749976	A	0.01546	0.03	G	0.1458	0.5079
5	rs201978887	150562697	D	0.1753	0.21	I	0.1458	0.7994
14	rs3803295	105395204	A	0.01546	0.03	G	0.1458	0.5079
22	rs4819756	18912678	A	0.01546	0.03	G	0.1458	0.5079
6	rs12216125	25997458	A	0.09536	0.1242	G	0.1459	0.7436
6	rs6908581	134417322	A	0.3351	0.3775	C	0.1459	0.8309
8	rs12542312	1439795	G	0.1778	0.1467	A	0.1459	1.258
10	rs34552277	37505159	A	0.09536	0.1242	G	0.1459	0.7436

6	rs237025	149721690	G	0.2784	0.3183	A	0.1461	0.826
9	rs301430	4576680	A	0.3351	0.3767	G	0.1461	0.8338
21	rs9981024	43319180	G	0.07732	0.05667	A	0.1461	1.395
1	rs2736831	26357656	C	0.2371	0.2758	A	0.1462	0.816
7	rs12155172	20994491	A	0.2371	0.2758	G	0.1462	0.816
7	rs17520733	70649789	G	0.2306	0.1953	A	0.1462	1.235
20	rs79468308	210225	A	0.1057	0.08083	G	0.1463	1.344
8	rs899979	39991573	C	0.2964	0.2575	A	0.1464	1.215
15	rs1986341	86402162	A	0.2371	0.275	G	0.1464	0.8194
19	rs2722722	44832875	A	0.3402	0.3	G	0.1464	1.203
19	rs4280359	44833851	C	0.3402	0.3	G	0.1464	1.203
19	rs12608979	55568084	A	0.1521	0.1858	G	0.1464	0.7857
4	rs2243682	104059542	A	0.1521	0.185	G	0.1465	0.79
4	rs2615542	104066461	G	0.1521	0.185	A	0.1465	0.79
11	rs948962	76919478	A	0.3376	0.38	C	0.1465	0.8317
6	rs9352006	74238815	A	0.4021	0.36	G	0.1467	1.195
13	rs79053319	25828007	A	0.1211	0.095	C	0.1468	1.313
6	rs9262164	30717389	C	0.1392	0.1108	G	0.1469	1.297
10	rs10882097	82568684	A	0.1314	0.1633	G	0.1469	0.7752
6	rs3793127	32371915	A	0.1314	0.1625	G	0.1471	0.78
13	rs7329480	64749837	G	0.3402	0.3008	A	0.1471	1.198
3	rs1060407	47958037	A	0.2397	0.2783	C	0.1474	0.8174
6	rs12333245	32019769	A	0.1804	0.1483	G	0.1474	1.264
6	rs2269429	32029183	A	0.1804	0.1483	G	0.1474	1.264
6	rs169496	32052983	A	0.1804	0.1483	G	0.1474	1.264
16	rs7203479	48544028	C	0.1804	0.1483	A	0.1474	1.264
1	rs12727642	8046672	A	0.0567	0.08	C	0.1476	0.6913
2	rs2278729	101668857	A	0.0567	0.08	G	0.1476	0.6913
3	rs55683301	169999670	G	0.0567	0.08	A	0.1476	0.6913
1	rs6427384	157508882	G	0.2062	0.2425	A	0.1477	0.8114
3	rs902320	67411103	A	0.2397	0.2775	G	0.1477	0.8208
6	rs11755393	34824636	G	0.4046	0.3633	A	0.1477	1.191
7	rs4722266	23794029	A	0.2062	0.2425	G	0.1477	0.8114
16	rs3136202	14038174	A	0.2062	0.2425	G	0.1477	0.8114
18	rs9967382	61587067	A	0.3428	0.385	G	0.1477	0.8332
18	rs963075	61600384	A	0.3428	0.385	G	0.1477	0.8332
4	rs2048268	58603583	G	0.0567	0.07917	A	0.1478	0.6992
5	rs10042595	72579933	G	0.4072	0.365	A	0.1478	1.195
10	rs1779216	18511082	A	0.4072	0.3654	G	0.1478	1.193
12	rs1860961	5841733	C	0.1572	0.1283	A	0.1478	1.267
12	rs6582630	38743508	G	0.114	0.1442	A	0.1478	0.7637
2	rs2037814	73675669	A	0.2629	0.2258	C	0.1479	1.223
7	rs339065	128371298	A	0.2629	0.2258	G	0.1479	1.223

7	rs11556924	129663496	A	0.0567	0.03833	G	0.1479	1.508
11	rs74484098	65789266	A	0.0567	0.03833	G	0.1479	1.508
19	rs10426537	19338942	A	0.0567	0.03833	G	0.1479	1.508
21	rs2249060	47773177	A	0.2629	0.2258	G	0.1479	1.223
6	rs10456397	31804014	T	0.01546	0.03083	A	0.148	0.4937
6	rs11966200	31837066	A	0.01546	0.03083	G	0.148	0.4937
6	rs117912086	170062481	A	0.01546	0.03083	G	0.148	0.4937
12	rs142507813	10978248	A	0.01546	0.03083	G	0.148	0.4937
17	rs72837329	54872439	G	0.01546	0.03083	A	0.148	0.4937
3	rs2366659	192087751	G	0.04639	0.0675	A	0.1481	0.6721
5	rs2071240	149755744	C	0.04639	0.0675	G	0.1481	0.6721
7	rs3801048	5391702	G	0.04639	0.0675	C	0.1481	0.6721
16	rs3194151	420140	A	0.1134	0.1442	G	0.1481	0.7593
20	rs2427254	60723988	A	0.04639	0.0675	G	0.1481	0.6721
21	rs2833784	33729129	G	0.4098	0.3675	A	0.1483	1.195
12	rs12309142	84346746	A	0.3497	0.3925	G	0.1484	0.8325
4	rs3733250	77192868	A	0.3454	0.3867	G	0.1485	0.8368
6	rs1799912	33132693	A	0.06701	0.0475	G	0.1485	1.44
12	rs8815	7507726	C	0.04639	0.06833	A	0.1485	0.6633
14	rs1380131	54072858	A	0.04639	0.06833	G	0.1485	0.6633
6	rs151021379	32147691	A	0.005181	0.0008333	G	0.1487	6.245
11	rs1050081	130784647	G	0.1546	0.1883	C	0.1487	0.7884
19	rs181506321	53117817	G	0.005181	0.0008333	A	0.1487	6.245
21	rs2839223	47777063	G	0.2629	0.2267	A	0.1487	1.217
22	rs6971	43558926	A	0.03109	0.01756	G	0.1487	1.795
3	rs779710	7511375	G	0.3479	0.39	A	0.1488	0.8346
7	rs7804091	19980396	G	0.1546	0.1875	A	0.1488	0.7927
6	rs79687747	135323891	G	0.03093	0.0175	A	0.1489	1.792
7	rs117593246	134618471	A	0.03093	0.0175	G	0.1489	1.792
12	rs2228587	124815439	G	0.03093	0.0175	A	0.1489	1.792
20	rs3746414	50769379	A	0.08247	0.1092	G	0.1489	0.7335
2	rs1047640	174820750	G	0.3479	0.3075	A	0.149	1.202
2	rs824931	222801699	G	0.2861	0.3258	A	0.149	0.8291
11	rs200353961	113141073	A	0.005181	0.0008347	G	0.149	6.234
4	rs10028610	82318524	A	0.3479	0.3892	G	0.1491	0.8375
15	rs3803354	40856989	G	0.08247	0.1083	A	0.1491	0.7398
6	rs3188482	30032673	G	0.2088	0.245	A	0.1493	0.8131
7	rs1320800	21991532	A	0.2655	0.2283	G	0.1494	1.221
10	rs60731885	99395652	A	0.1804	0.2158	C	0.1495	0.7998
10	rs58533443	119774598	A	0.1804	0.2158	T	0.1495	0.7998
4	rs200142235	2074703	D	0.1237	0.09667	I	0.1496	1.319
6	rs4458716	130152296	A	0.09794	0.1258	G	0.1496	0.7542
1	rs202026791	4832552	A	0.005155	0.0008333	G	0.1497	6.212

1	rs188843029	12640678	A	0.005155	0.0008333	G	0.1497	6.212
1	rs201361255	18023630	A	0.005155	0.0008333	G	0.1497	6.212
1	rs144716849	19564554	A	0.005155	0.0008333	G	0.1497	6.212
1	rs146734508	33361013	A	0.005155	0.0008333	G	0.1497	6.212
1	rs115780313	34254272	G	0.005155	0.0008333	A	0.1497	6.212
1	rs151142512	36235452	A	0.005155	0.0008333	G	0.1497	6.212
1	rs144048339	43308310	A	0.005155	0.0008333	G	0.1497	6.212
1	rs187427261	43893240	A	0.005155	0.0008333	G	0.1497	6.212
1	rs180780655	75107043	G	0.005155	0.0008333	A	0.1497	6.212
1	rs140072705	145561334	A	0.005155	0.0008333	G	0.1497	6.212
1	rs59820789	151542140	A	0.005155	0.0008333	G	0.1497	6.212
1	rs187257317	170007557	G	0.005155	0.0008333	A	0.1497	6.212
1	rs149598381	173797503	G	0.005155	0.0008333	A	0.1497	6.212
1	rs145551734	175048706	T	0.005155	0.0008333	A	0.1497	6.212
1	rs202193200	197070153	A	0.005155	0.0008333	G	0.1497	6.212
1	rs201738898	207133113	A	0.005155	0.0008333	G	0.1497	6.212
2	rs151054868	1687859	A	0.005155	0.0008333	G	0.1497	6.212
2	rs184605839	26702473	A	0.005155	0.0008333	G	0.1497	6.212
2	rs76277684	30863053	A	0.005155	0.0008333	C	0.1497	6.212
2	rs192503385	37439091	A	0.005155	0.0008333	G	0.1497	6.212
2	rs191543274	42990568	A	0.005155	0.0008333	G	0.1497	6.212
2	rs201461683	101644769	G	0.005155	0.0008333	A	0.1497	6.212
2	rs150964994	101654161	A	0.005155	0.0008333	G	0.1497	6.212
2	rs6543242	104757972	G	0.005155	0.0008333	A	0.1497	6.212
2	rs138032670	112990918	G	0.005155	0.0008333	C	0.1497	6.212
2	rs200314889	133489315	A	0.005155	0.0008333	G	0.1497	6.212
2	rs185438286	159196789	A	0.005155	0.0008333	C	0.1497	6.212
2	rs141659304	160604768	A	0.005155	0.0008333	G	0.1497	6.212
2	rs183844833	179431159	G	0.005155	0.0008333	A	0.1497	6.212
2	rs139172299	179613268	C	0.005155	0.0008333	A	0.1497	6.212
2	rs988583	198987935	A	0.005155	0.0008333	C	0.1497	6.212
2	rs140005351	201726566	A	0.005155	0.0008333	G	0.1497	6.212
2	rs10202111	210810644	A	0.005155	0.0008333	G	0.1497	6.212
2	rs200509072	228172468	A	0.005155	0.0008333	G	0.1497	6.212
2	rs199646208	238280909	A	0.005155	0.0008333	G	0.1497	6.212
3	rs139204684	43122527	A	0.005155	0.0008333	G	0.1497	6.212
3	rs184756780	43618504	G	0.005155	0.0008333	A	0.1497	6.212
3	rs9818345	44943088	G	0.005155	0.0008333	A	0.1497	6.212
3	rs192379813	118905614	A	0.005155	0.0008333	G	0.1497	6.212
3	rs201177696	121981074	A	0.005155	0.0008333	G	0.1497	6.212
3	rs201274785	126142479	A	0.005155	0.0008333	C	0.1497	6.212
3	rs199584005	128181491	A	0.005155	0.0008333	G	0.1497	6.212
3	rs35886424	130129322	C	0.09794	0.1267	G	0.1497	0.7486

3	rs76864445	130135644	A	0.09794	0.1267	C	0.1497	0.7486
3	rs141369962	192980885	A	0.005155	0.0008333	G	0.1497	6.212
3	rs200905829	197619527	G	0.005155	0.0008333	A	0.1497	6.212
4	rs139469812	956237	A	0.005155	0.0008333	G	0.1497	6.212
4	rs200994120	1657232	A	0.005155	0.0008333	G	0.1497	6.212
4	rs147295492	71508215	A	0.005155	0.0008333	G	0.1497	6.212
4	rs17000961	76788499	A	0.005155	0.0008333	T	0.1497	6.212
4	rs34155925	76788564	A	0.005155	0.0008333	G	0.1497	6.212
5	rs142752433	34998925	A	0.005155	0.0008333	G	0.1497	6.212
5	rs149043842	35641648	A	0.005155	0.0008333	G	0.1497	6.212
5	rs202138455	76715606	A	0.005155	0.0008333	G	0.1497	6.212
5	rs201800819	90449167	A	0.005155	0.0008333	G	0.1497	6.212
5	rs149823680	134670770	A	0.005155	0.0008333	G	0.1497	6.212
5	rs201055359	141325257	G	0.005155	0.0008333	C	0.1497	6.212
5	rs151002555	141336635	A	0.005155	0.0008333	G	0.1497	6.212
6	rs192246809	25773605	A	0.005155	0.0008333	G	0.1497	6.212
6	rs2517623	30152961	G	0.005155	0.0008333	A	0.1497	6.212
6	rs3179003	31556928	A	0.005155	0.0008333	C	0.1497	6.212
6	rs74993996	32084867	A	0.005155	0.0008333	C	0.1497	6.212
6	rs55827768	32805967	G	0.005155	0.0008333	A	0.1497	6.212
6	rs6923761	39034072	A	0.005155	0.0008333	G	0.1497	6.212
6	rs148120569	43111200	C	0.005155	0.0008333	G	0.1497	6.212
6	rs139571922	106967902	A	0.005155	0.0008333	G	0.1497	6.212
6	rs13194610	117591755	G	0.09794	0.1267	A	0.1497	0.7486
6	rs140385571	139165719	G	0.005155	0.0008333	A	0.1497	6.212
6	rs200154828	161006178	A	0.005155	0.0008333	G	0.1497	6.212
7	rs149438746	7530225	A	0.005155	0.0008333	C	0.1497	6.212
7	rs149025049	47852786	A	0.005155	0.0008333	G	0.1497	6.212
7	rs145480547	69364347	C	0.005155	0.0008333	G	0.1497	6.212
7	rs200110536	84685158	G	0.005155	0.0008333	A	0.1497	6.212
7	rs189083857	91682214	G	0.005155	0.0008333	C	0.1497	6.212
7	rs189374343	94049737	G	0.005155	0.0008333	A	0.1497	6.212
7	rs199992794	99768097	A	0.005155	0.0008333	G	0.1497	6.212
7	rs201639027	100172813	G	0.005155	0.0008333	A	0.1497	6.212
7	rs140244991	122774582	A	0.005155	0.0008333	G	0.1497	6.212
7	rs137989079	142989560	A	0.005155	0.0008333	G	0.1497	6.212
7	rs141479576	150772536	A	0.005155	0.0008333	G	0.1497	6.212
8	rs17665340	363185	G	0.005155	0.0008333	A	0.1497	6.212
8	rs61731551	1900992	A	0.005155	0.0008333	G	0.1497	6.212
8	rs199978883	9610060	A	0.005155	0.0008333	C	0.1497	6.212
8	rs151273238	26627895	A	0.005155	0.0008333	G	0.1497	6.212
8	rs200422473	26721841	G	0.005155	0.0008333	C	0.1497	6.212
8	rs1800391	30938704	A	0.005155	0.0008333	G	0.1497	6.212

8	rs34715198	42977727	A	0.005155	0.0008333	G	0.1497	6.212
8	rs4872971	47800155	G	0.4149	0.3733	A	0.1497	1.191
8	rs114408839	90921899	A	0.005155	0.0008333	T	0.1497	6.212
8	rs191116838	121061823	G	0.005155	0.0008333	C	0.1497	6.212
8	rs190405294	125109516	A	0.005155	0.0008333	G	0.1497	6.212
8	rs192799797	142178261	A	0.005155	0.0008333	G	0.1497	6.212
8	rs200835664	145731257	G	0.005155	0.0008333	C	0.1497	6.212
8	rs61732864	146157256	A	0.005155	0.0008333	G	0.1497	6.212
9	rs3736390	2820046	G	0.005155	0.0008333	C	0.1497	6.212
9	rs185549934	5787566	A	0.005155	0.0008333	G	0.1497	6.212
9	rs57728054	71866119	G	0.005155	0.0008333	A	0.1497	6.212
9	rs78251065	79936495	C	0.005155	0.0008333	A	0.1497	6.212
9	rs141819820	95841847	A	0.005155	0.0008333	G	0.1497	6.212
9	rs34433105	97062295	A	0.005155	0.0008333	G	0.1497	6.212
9	rs201111862	100890454	A	0.005155	0.0008333	G	0.1497	6.212
9	rs202233874	132484575	A	0.005155	0.0008333	G	0.1497	6.212
9	rs7873874	138302726	A	0.005155	0.0008333	C	0.1497	6.212
9	rs28620856	140166981	A	0.005155	0.0008333	G	0.1497	6.212
10	rs138658798	7605243	A	0.005155	0.0008333	G	0.1497	6.212
10	rs141567347	13217560	G	0.005155	0.0008333	C	0.1497	6.212
10	rs140169226	55582205	A	0.005155	0.0008333	C	0.1497	6.212
10	rs201748635	79581223	A	0.005155	0.0008333	G	0.1497	6.212
10	rs150063098	106075100	A	0.005155	0.0008333	G	0.1497	6.212
10	rs117795868	129906577	A	0.005155	0.0008333	G	0.1497	6.212
10	rs147204742	132891531	A	0.005155	0.0008333	G	0.1497	6.212
11	rs184893349	249023	A	0.005155	0.0008333	G	0.1497	6.212
11	rs143908864	541550	A	0.005155	0.0008333	G	0.1497	6.212
11	rs200164387	3832541	A	0.005155	0.0008333	G	0.1497	6.212
11	rs182211570	5153329	G	0.005155	0.0008333	A	0.1497	6.212
11	rs189875301	8014567	A	0.005155	0.0008333	G	0.1497	6.212
11	rs185210904	35243250	G	0.005155	0.0008333	A	0.1497	6.212
11	rs186432117	66816064	A	0.005155	0.0008333	G	0.1497	6.212
11	rs61735578	95571347	C	0.005155	0.0008333	G	0.1497	6.212
11	rs183324237	123624792	C	0.005155	0.0008333	G	0.1497	6.212
12	rs11550887	3147203	A	0.005155	0.0008333	G	0.1497	6.212
12	rs189016395	51639784	G	0.005155	0.0008333	A	0.1497	6.212
12	rs115320022	53004545	C	0.005155	0.0008333	G	0.1497	6.212
12	rs143765948	55886433	A	0.005155	0.0008333	C	0.1497	6.212
12	rs185524052	64895141	A	0.005155	0.0008333	G	0.1497	6.212
12	rs116424321	66704168	A	0.005155	0.0008333	G	0.1497	6.212
12	rs199651099	101381343	A	0.005155	0.0008333	G	0.1497	6.212
12	rs117801489	104408832	G	0.005155	0.0008333	A	0.1497	6.212
12	rs140095224	105445974	A	0.005155	0.0008333	G	0.1497	6.212

12	rs200127576	109281339	A	0.005155	0.0008333	G	0.1497	6.212
12	rs139795134	113837452	C	0.005155	0.0008333	A	0.1497	6.212
12	rs201854291	117383245	A	0.005155	0.0008333	G	0.1497	6.212
12	rs200811312	122441592	A	0.005155	0.0008333	G	0.1497	6.212
12	rs28711796	124008074	G	0.005155	0.0008333	A	0.1497	6.212
13	rs190118929	35751178	A	0.005155	0.0008333	C	0.1497	6.212
13	rs180865044	50594584	A	0.005155	0.0008333	C	0.1497	6.212
13	rs80350829	95118870	A	0.005155	0.0008333	G	0.1497	6.212
13	rs201908744	111932927	G	0.005155	0.0008333	A	0.1497	6.212
14	rs202001645	20923826	A	0.005155	0.0008333	G	0.1497	6.212
14	rs202109789	20978889	A	0.005155	0.0008333	G	0.1497	6.212
14	rs143519313	21167971	G	0.005155	0.0008333	A	0.1497	6.212
14	rs189316749	21360161	A	0.005155	0.0008333	C	0.1497	6.212
14	rs146755923	100129357	A	0.005155	0.0008333	G	0.1497	6.212
14	rs201368458	101201026	A	0.005155	0.0008333	G	0.1497	6.212
14	rs138979378	105642983	A	0.005155	0.0008333	G	0.1497	6.212
14	rs139419197	105847373	A	0.005155	0.0008333	G	0.1497	6.212
15	rs147013385	30556183	A	0.005155	0.0008333	G	0.1497	6.212
15	rs121917739	41011016	A	0.005155	0.0008333	G	0.1497	6.212
15	rs143486742	44087216	A	0.005155	0.0008333	C	0.1497	6.212
15	rs199924625	48431319	A	0.005155	0.0008333	G	0.1497	6.212
15	rs2899472	51516055	A	0.005155	0.0008333	C	0.1497	6.212
15	rs200307684	64455101	A	0.005155	0.0008333	G	0.1497	6.212
15	rs200353973	66610907	G	0.005155	0.0008333	A	0.1497	6.212
15	rs186185335	74490093	G	0.005155	0.0008333	A	0.1497	6.212
15	rs201219073	75632184	G	0.005155	0.0008333	C	0.1497	6.212
15	rs186761656	80855542	A	0.005155	0.0008333	T	0.1497	6.212
15	rs200434957	81593713	C	0.005155	0.0008333	G	0.1497	6.212
16	rs148323035	216090	G	0.005155	0.0008333	A	0.1497	6.212
16	rs191366820	845754	A	0.005155	0.0008333	G	0.1497	6.212
16	rs61741082	845797	C	0.005155	0.0008333	G	0.1497	6.212
16	rs61747731	2817499	A	0.005155	0.0008333	G	0.1497	6.212
16	rs189265602	10788261	G	0.005155	0.0008333	A	0.1497	6.212
16	rs12930355	27720201	A	0.005155	0.0008333	G	0.1497	6.212
16	rs201158872	30735598	A	0.005155	0.0008333	G	0.1497	6.212
16	rs188342896	31096461	A	0.005155	0.0008333	G	0.1497	6.212
16	rs140636792	47683077	C	0.005155	0.0008333	A	0.1497	6.212
17	rs200798233	5286462	A	0.005155	0.0008333	G	0.1497	6.212
17	rs115035111	12655938	A	0.005155	0.0008333	G	0.1497	6.212
17	rs115341498	17722536	A	0.005155	0.0008333	G	0.1497	6.212
17	rs115536974	21081599	C	0.005155	0.0008333	A	0.1497	6.212
17	rs188627683	39644517	A	0.005155	0.0008333	C	0.1497	6.212
17	rs79309130	40341909	A	0.005155	0.0008333	G	0.1497	6.212

17	rs202079779	41568557	C	0.005155	0.0008333	A	0.1497	6.212
17	rs13306776	42335485	A	0.005155	0.0008333	G	0.1497	6.212
17	rs201153167	43313597	G	0.005155	0.0008333	A	0.1497	6.212
17	rs151209912	56440903	A	0.005155	0.0008333	G	0.1497	6.212
17	rs2228305	80042792	A	0.005155	0.0008333	G	0.1497	6.212
17	rs3748811	80676810	A	0.005155	0.0008333	G	0.1497	6.212
18	rs138144679	50866236	A	0.005155	0.0008333	G	0.1497	6.212
18	rs79607526	61234110	A	0.005155	0.0008333	G	0.1497	6.212
19	rs202072324	3825330	A	0.005155	0.0008333	G	0.1497	6.212
19	rs182802964	46394095	A	0.005155	0.0008333	C	0.1497	6.212
19	rs191642980	47880401	A	0.005155	0.0008333	C	0.1497	6.212
19	rs202195426	48789663	C	0.005155	0.0008333	G	0.1497	6.212
19	rs73933937	55175795	A	0.005155	0.0008333	G	0.1497	6.212
19	rs199858730	58967878	A	0.005155	0.0008333	C	0.1497	6.212
20	rs189533691	15210624	G	0.005155	0.0008333	A	0.1497	6.212
20	rs201724904	34091694	A	0.005155	0.0008333	G	0.1497	6.212
20	rs139932041	45354606	A	0.005155	0.0008333	G	0.1497	6.212
21	rs201679623	42866388	C	0.005155	0.0008333	A	0.1497	6.212
21	rs200533753	45064182	G	0.005155	0.0008333	A	0.1497	6.212
21	rs188937098	45220407	A	0.005155	0.0008333	G	0.1497	6.212
22	rs137960357	29454948	A	0.005155	0.0008333	G	0.1497	6.212
22	rs61737784	39218763	A	0.005155	0.0008333	G	0.1497	6.212
22	rs58294273	46708155	A	0.005155	0.0008333	G	0.1497	6.212
22	rs201794808	51065341	A	0.005155	0.0008333	G	0.1497	6.212
1	rs73127483	244980554	G	0.09326	0.07	A	0.1498	1.367
6	rs2844729	30422632	A	0.3505	0.31	G	0.1498	1.201
6	rs2524180	30426130	G	0.3505	0.31	A	0.1498	1.201
14	rs698025	59113430	A	0.09794	0.1269	G	0.1498	0.7471
15	rs12324805	82352200	C	0.3531	0.395	A	0.1498	0.836
18	rs2298546	9255718	A	0.2448	0.2833	G	0.1498	0.8201
21	rs1047978	19169155	G	0.4175	0.375	C	0.1498	1.195
1	rs6030	169498975	G	0.3041	0.265	A	0.1499	1.212
6	rs2295190	152443744	A	0.06959	0.09417	C	0.1499	0.7195
15	rs2614668	86287867	A	0.2887	0.3283	G	0.1499	0.8301
19	rs3746266	18876309	G	0.1849	0.1527	A	0.15	1.259
4	rs191714736	153802307	A	0.005155	0.0008347	G	0.1501	6.202
6	rs45484591	31918468	C	0.005155	0.0008347	A	0.1501	6.202
9	rs145198648	139265456	A	0.005155	0.0008347	G	0.1501	6.202
9	rs144139211	140093696	A	0.005155	0.0008347	G	0.1501	6.202
10	rs116360202	50740626	A	0.005155	0.0008347	G	0.1501	6.202
1	rs140760240	205884138	A	0.01804	0.008333	G	0.1502	2.186
2	rs7565275	234229320	G	0.01804	0.008333	A	0.1502	2.186
3	rs146543428	52939210	G	0.01804	0.008333	A	0.1502	2.186

3	rs147783135	111799851	A	0.01804	0.008333	G	0.1502	2.186
4	rs117523998	148886205	A	0.01804	0.008333	G	0.1502	2.186
5	rs144381958	172750267	A	0.01804	0.008333	G	0.1502	2.186
7	rs56268851	23768759	A	0.01804	0.008333	G	0.1502	2.186
8	rs146190924	101016251	A	0.01804	0.008333	G	0.1502	2.186
9	rs16906079	120474929	G	0.01804	0.008333	A	0.1502	2.186
9	rs115335100	139983339	G	0.01804	0.008333	A	0.1502	2.186
10	rs140510480	127524761	A	0.01804	0.008333	G	0.1502	2.186
11	rs141698114	62291756	G	0.01804	0.008333	A	0.1502	2.186
11	rs150479005	71725397	A	0.01804	0.008333	G	0.1502	2.186
13	rs192811737	67799687	G	0.01804	0.008333	C	0.1502	2.186
13	rs4318070	98960762	A	0.01804	0.008333	G	0.1502	2.186
16	rs150049919	77859153	A	0.01804	0.008333	G	0.1502	2.186
17	rs76753427	27380585	A	0.01804	0.008333	G	0.1502	2.186
17	rs200494869	60768003	A	0.01804	0.008333	G	0.1502	2.186
1	rs6676003	53696784	A	0.3557	0.3983	C	0.1503	0.8338
1	rs202046937	100368318	A	0.01289	0.005	G	0.1503	2.598
1	rs146152769	154247666	A	0.01289	0.005	G	0.1503	2.598
1	rs34379394	167384979	G	0.01289	0.005	A	0.1503	2.598
1	rs141348662	197059211	G	0.01289	0.005	A	0.1503	2.598
1	rs184692250	223178988	G	0.01289	0.005	A	0.1503	2.598
1	rs200397350	226924982	C	0.01289	0.005	G	0.1503	2.598
2	rs143287701	27306780	A	0.01289	0.005	G	0.1503	2.598
2	rs3770739	64780257	G	0.01289	0.005	A	0.1503	2.598
2	rs200980149	85662921	C	0.01289	0.005	G	0.1503	2.598
2	rs61748152	118578770	A	0.01289	0.005	G	0.1503	2.598
2	rs181407252	135745214	G	0.01289	0.005	A	0.1503	2.598
2	rs6759948	158889593	A	0.01289	0.005	G	0.1503	2.598
2	rs79805025	167055438	A	0.01289	0.005	G	0.1503	2.598
2	rs77655188	171675181	A	0.01289	0.005	C	0.1503	2.598
2	rs117088491	232326517	C	0.01289	0.005	A	0.1503	2.598
3	rs139572691	12867010	A	0.01289	0.005	G	0.1503	2.598
3	rs115596050	189706762	A	0.01289	0.005	G	0.1503	2.598
4	rs3765121	20852166	G	0.3557	0.3983	A	0.1503	0.8338
4	rs61740705	169343044	G	0.01289	0.005	A	0.1503	2.598
6	rs2071289	32135136	A	0.01289	0.005	C	0.1503	2.598
6	rs189220440	71235680	G	0.01289	0.005	A	0.1503	2.598
6	rs188391926	99858695	G	0.01289	0.005	A	0.1503	2.598
6	rs139306565	142721674	T	0.01289	0.005	A	0.1503	2.598
7	rs28365085	99245974	G	0.01289	0.005	A	0.1503	2.598
7	rs187667058	122342090	A	0.01289	0.005	G	0.1503	2.598
7	rs78300720	139796783	G	0.01289	0.005	C	0.1503	2.598
7	rs144857453	151483612	A	0.01289	0.005	G	0.1503	2.598

10	rs75234626	81111337	A	0.01289	0.005	G	0.1503	2.598
10	rs143793106	90974776	G	0.01289	0.005	A	0.1503	2.598
12	rs11113318	107814757	G	0.01289	0.005	A	0.1503	2.598
13	rs115158613	38248380	C	0.01289	0.005	A	0.1503	2.598
14	rs75370063	21546635	A	0.01289	0.005	G	0.1503	2.598
14	rs77893724	24842563	C	0.01289	0.005	A	0.1503	2.598
14	rs142838359	50262548	C	0.01289	0.005	A	0.1503	2.598
16	rs10459809	57949224	A	0.01289	0.005	C	0.1503	2.598
19	rs199634123	35550609	G	0.01289	0.005	C	0.1503	2.598
19	rs201657564	46526169	A	0.01289	0.005	C	0.1503	2.598
20	rs147245947	3541462	T	0.01289	0.005	A	0.1503	2.598
3	rs4453795	192093975	G	0.3557	0.3975	A	0.1504	0.8367
6	rs2524198	30421502	A	0.3505	0.3108	G	0.1504	1.197
12	rs10774834	110108063	A	0.3041	0.2658	G	0.1504	1.207
1	rs12726652	107432576	A	0.2912	0.3317	G	0.1505	0.828
2	rs2048726	1426346	A	0.2912	0.3317	C	0.1505	0.828
8	rs1397364	112819969	A	0.2912	0.3314	G	0.1505	0.8291
1	rs3806339	89735135	A	0.1418	0.1133	G	0.1506	1.292
1	rs16834168	152488146	A	0.1418	0.1133	G	0.1506	1.292
1	rs2075740	152975963	A	0.0232	0.0125	G	0.1506	1.876
2	rs34291329	218683139	A	0.0232	0.0125	G	0.1506	1.876
5	rs4143541	5725162	G	0.2113	0.2483	A	0.1506	0.8111
6	rs146534657	138195991	G	0.0232	0.0125	A	0.1506	1.876
12	rs3741803	12483134	A	0.0232	0.0125	T	0.1506	1.876
12	rs12227259	29670504	A	0.1418	0.1133	G	0.1506	1.292
14	rs74807133	31549832	A	0.0232	0.0125	G	0.1506	1.876
15	rs148760039	29346590	A	0.0232	0.0125	G	0.1506	1.876
17	rs63750072	44060859	G	0.0232	0.0125	A	0.1506	1.876
17	rs57367176	67287358	G	0.0232	0.0125	A	0.1506	1.876
20	rs117528644	30366700	G	0.0232	0.0125	A	0.1506	1.876
20	rs150152777	34575330	C	0.0232	0.0125	A	0.1506	1.876
20	rs141071658	36964033	A	0.0232	0.0125	G	0.1506	1.876
21	rs78287602	31654638	A	0.0232	0.0125	G	0.1506	1.876
21	rs12483584	31691792	A	0.0232	0.0125	C	0.1506	1.876
21	rs3804007	31692277	A	0.0232	0.0125	C	0.1506	1.876
21	rs117534873	31709703	A	0.0232	0.0125	G	0.1506	1.876
22	rs11541479	19906511	A	0.0232	0.0125	G	0.1506	1.876
22	rs9626698	47593413	G	0.0232	0.0125	A	0.1506	1.876
6	rs1076832	30419831	A	0.1598	0.1308	C	0.1508	1.263
19	rs4756	59074653	G	0.1572	0.1903	A	0.1509	0.7936
8	rs1866694	62172668	C	0.183	0.1517	A	0.151	1.253
11	rs2712780	74829787	A	0.1572	0.1908	G	0.151	0.791
5	rs17144974	118533499	G	0.1237	0.0975	A	0.1511	1.307

6	rs76463649	28239741	G	0.1237	0.0975	A	0.1511	1.307
6	rs11968814	71776971	A	0.1237	0.0975	G	0.1511	1.307
10	rs10857471	50533776	C	0.1572	0.19	G	0.1511	0.7953
6	rs76604824	76631823	A	0.116	0.1467	G	0.1512	0.7633
10	rs11251469	2766921	A	0.2088	0.175	G	0.1513	1.244
10	rs10788160	123033549	A	0.183	0.2183	G	0.1514	0.8019
5	rs4382127	27252466	G	0.3067	0.2683	A	0.1515	1.206
6	rs3765310	24503597	A	0.1082	0.08333	G	0.1515	1.335
6	rs17850652	88239266	G	0.1082	0.08333	A	0.1515	1.335
8	rs1053079	74893821	G	0.1082	0.08333	A	0.1515	1.335
8	rs1053077	74893850	C	0.1082	0.08333	G	0.1515	1.335
10	rs755793	123310871	G	0.1082	0.08333	A	0.1515	1.335
12	rs3763980	60173356	T	0.3557	0.315	A	0.1515	1.2
10	rs10824885	54863115	C	0.4227	0.3817	A	0.1516	1.186
7	rs2140516	122809234	G	0.3634	0.4058	A	0.1517	0.8358
4	rs1392550	155804083	G	0.4278	0.3858	A	0.1519	1.19
9	rs7847631	29024894	A	0.4278	0.3858	G	0.1519	1.19
22	rs7287160	39573724	A	0.3634	0.405	C	0.1519	0.8387
5	rs17844309	140214995	A	0.0567	0.03917	G	0.1521	1.475
12	rs77621437	47471555	G	0.0567	0.03917	C	0.1521	1.475
4	rs1967397	21254331	G	0.1418	0.1142	A	0.1522	1.282
8	rs4732812	27923388	A	0.1624	0.1325	G	0.1522	1.269
17	rs721957	39137297	G	0.366	0.4083	A	0.1522	0.8364
1	rs3754415	226924642	A	0.2964	0.3367	G	0.1523	0.83
3	rs2290305	9871030	C	0.2088	0.1758	A	0.1523	1.237
3	rs2073495	46480958	C	0.3582	0.3175	G	0.1523	1.2
19	rs1654413	55526359	A	0.1366	0.1686	T	0.1523	0.7801
20	rs2567608	23017082	A	0.4278	0.3867	G	0.1524	1.186
21	rs3746894	43162150	G	0.4278	0.3867	A	0.1524	1.186
3	rs74409632	108021333	A	0.2371	0.2025	G	0.1525	1.224
6	rs2394895	31206979	G	0.1856	0.1533	A	0.1526	1.258
12	rs7296262	129095072	G	0.3686	0.4108	A	0.1526	0.837
1	rs7525223	180917275	A	0.4352	0.3938	G	0.1527	1.186
8	rs10958369	54410208	A	0.433	0.3908	G	0.1527	1.19
4	rs2061571	129052429	A	0.3686	0.41	G	0.1529	0.8399
4	rs12645577	129100643	A	0.3686	0.41	G	0.1529	0.8399
7	rs1049793	150557665	G	0.3686	0.41	C	0.1529	0.8399
9	rs147308483	140110727	A	0.06701	0.04833	G	0.153	1.414
15	rs146403122	72104306	A	0.06701	0.04833	G	0.153	1.414
1	rs2248666	178620601	A	0.4356	0.3933	C	0.1531	1.19
2	rs13002673	26798893	A	0.433	0.3917	T	0.1532	1.186
9	rs7868266	114470138	A	0.03866	0.02417	G	0.1532	1.624
9	rs28551718	114475404	C	0.03866	0.02417	A	0.1532	1.624

9	rs1322257	114480562	G	0.03866	0.02417	A	0.1532	1.624
9	rs10981047	114510473	G	0.03866	0.02417	C	0.1532	1.624
12	rs4760087	130661220	A	0.3711	0.4125	G	0.1533	0.8405
10	rs34473884	133761285	A	0.1598	0.1925	G	0.1534	0.7978
13	rs2224778	46752813	G	0.3711	0.4124	A	0.1534	0.841
14	rs9323945	96781912	A	0.1598	0.1925	G	0.1534	0.7978
3	rs7647075	162282328	C	0.299	0.3383	A	0.1535	0.834
4	rs976002	69343287	G	0.1005	0.1292	A	0.1535	0.7534
13	rs157024	109777503	G	0.1005	0.1292	A	0.1535	0.7534
20	rs979332	3433158	A	0.3608	0.3208	G	0.1535	1.195
4	rs7687921	176859026	T	0.1237	0.09833	A	0.1536	1.295
12	rs9645774	91380633	A	0.4356	0.3942	G	0.1536	1.186
11	rs7481311	27583129	A	0.3119	0.2733	G	0.1537	1.205
16	rs1558561	5061948	G	0.4381	0.3965	A	0.1537	1.187
3	rs10084692	77378207	A	0.2732	0.2358	G	0.1538	1.218
10	rs11200925	85984323	A	0.2732	0.2358	G	0.1538	1.218
2	rs11895564	173339808	A	0.1082	0.08417	G	0.1539	1.321
8	rs7009231	56116863	G	0.3763	0.4183	A	0.1539	0.8389
8	rs2272715	146225280	G	0.3015	0.3417	A	0.1539	0.8319
2	rs12464512	19096394	G	0.05928	0.0825	A	0.154	0.7008
7	rs17145713	72904810	A	0.1263	0.09917	G	0.154	1.313
7	rs3812316	73020337	C	0.1263	0.09917	G	0.154	1.313
12	rs10771357	9305473	A	0.1263	0.09917	G	0.154	1.313
16	rs117821265	721780	G	0.05928	0.0825	A	0.154	0.7008
6	rs6910541	87969480	A	0.1186	0.1483	G	0.1541	0.7723
10	rs79579856	91497912	G	0.3177	0.2784	A	0.1541	1.207
4	rs10518025	68064440	G	0.09278	0.07083	A	0.1542	1.342
8	rs3750228	70980738	G	0.2397	0.205	A	0.1542	1.223
12	rs11060202	129693530	G	0.2397	0.205	A	0.1542	1.223
1	rs6661946	236723108	A	0.1186	0.1492	G	0.1543	0.7672
1	rs79225714	236737983	G	0.1186	0.1492	A	0.1543	0.7672
6	rs7759742	32381736	A	0.3608	0.3217	T	0.1543	1.19
10	rs10764037	19769510	A	0.4407	0.3992	G	0.1543	1.186
16	rs3751821	19547503	A	0.3763	0.4175	G	0.1543	0.8417
19	rs8100856	1796166	G	0.4433	0.4008	A	0.1543	1.19
2	rs4667591	170003432	C	0.3789	0.42	A	0.1547	0.8423
16	rs26856	2260612	A	0.3041	0.345	G	0.1547	0.8297
12	rs12819734	133182818	G	0.4459	0.404	A	0.1548	1.187
15	rs3816533	42134097	G	0.3144	0.2758	A	0.1548	1.204
16	rs258322	89755903	G	0.3814	0.4233	A	0.1548	0.84
12	rs13277	66786091	G	0.09536	0.07167	C	0.1549	1.365
19	rs77072552	55449446	A	0.03866	0.05917	G	0.1549	0.6395
20	rs2235491	45354291	A	0.03866	0.05917	G	0.1549	0.6395

14	rs4078432	48997206	G	0.1881	0.2233	A	0.155	0.8059
14	rs12891095	87384188	G	0.3634	0.3242	A	0.155	1.19
6	rs9351411	65455894	A	0.384	0.4267	G	0.1551	0.8377
12	rs1376251	11138852	G	0.2191	0.2558	A	0.1551	0.816
2	rs1425613	47793217	A	0.3814	0.4225	G	0.1552	0.8429
2	rs55703767	228121101	A	0.07216	0.09667	C	0.1552	0.7268
4	rs2288675	165118652	A	0.1649	0.135	G	0.1552	1.266
10	rs17133693	4875591	G	0.1649	0.135	A	0.1552	1.266
17	rs2277648	10324980	G	0.1649	0.135	A	0.1552	1.266
5	rs1191743	119413303	A	0.3866	0.429	G	0.1553	0.8387
1	rs1474747	159148513	G	0.2732	0.2375	A	0.1554	1.207
2	rs409188	32495471	A	0.1263	0.1	C	0.1554	1.301
10	rs12359281	27353007	G	0.07216	0.0975	A	0.1554	0.7199
4	rs4961	2906707	C	0.3866	0.4283	A	0.1555	0.8412
17	rs4343	61566031	G	0.3866	0.4283	A	0.1555	0.8412
3	rs242519	13232556	A	0.3067	0.3467	G	0.1556	0.8337
10	rs868738	115381747	A	0.2139	0.18	G	0.1556	1.24
11	rs2115763	112051169	A	0.451	0.4092	T	0.1556	1.186
6	rs1050348	112493872	A	0.1624	0.195	G	0.1557	0.8002
11	rs3736228	68201295	A	0.2758	0.2392	G	0.1557	1.211
11	rs10831519	96116444	G	0.3686	0.3283	A	0.1557	1.194
19	rs189664399	51649312	A	0.007895	0.002513	G	0.1557	3.159
3	rs4380451	32329497	A	0.0232	0.03917	G	0.1558	0.5826
4	rs7668988	45638903	G	0.3892	0.4317	A	0.1558	0.8388
4	rs11100719	142354508	G	0.3892	0.4317	A	0.1558	0.8388
12	rs76064926	1965210	A	0.0232	0.03917	G	0.1558	0.5826
20	rs35944046	43629911	G	0.0232	0.03917	A	0.1558	0.5826
1	rs4085613	152550018	A	0.4485	0.4075	C	0.1559	1.182
1	rs20563	183085755	A	0.4485	0.4075	G	0.1559	1.182
2	rs12479352	240435943	G	0.1881	0.1567	A	0.1561	1.247
5	rs7713638	149759096	G	0.04897	0.07083	A	0.1561	0.6754
1	rs11264875	153994647	A	0.3686	0.3292	G	0.1564	1.19
1	rs1056695	156622252	A	0.3686	0.3292	G	0.1564	1.19
16	rs684286	29775947	A	0.02332	0.04	G	0.1564	0.5729
19	rs7188	11275139	C	0.3686	0.3292	A	0.1564	1.19
19	rs3786629	51850597	G	0.3686	0.3292	A	0.1564	1.19
2	rs12471235	142707328	A	0.4588	0.4167	C	0.1565	1.187
6	rs212388	159490436	A	0.3711	0.3308	G	0.1565	1.194
10	rs7831	105205302	C	0.317	0.2792	A	0.1565	1.198
11	rs7927783	103506972	G	0.4588	0.4167	A	0.1565	1.187
16	rs2078478	90130136	G	0.1907	0.2257	A	0.1565	0.8086
18	rs8096353	4153950	A	0.317	0.2792	G	0.1565	1.198
2	rs2302612	102851708	G	0.2216	0.2592	A	0.1566	0.814

5	rs13357554	180630512	C	0.0232	0.04	G	0.1566	0.5699
6	rs415929	32189032	G	0.2216	0.2592	A	0.1566	0.814
12	rs3759299	32137512	G	0.2758	0.24	C	0.1567	1.206
12	rs17101603	54069925	A	0.03093	0.01833	G	0.1567	1.709
15	rs117184817	86822926	G	0.03093	0.01833	A	0.1567	1.709
15	rs79725910	91795124	A	0.03093	0.01833	G	0.1567	1.709
16	rs34832584	72162966	A	0.03093	0.01833	C	0.1567	1.709
17	rs75754003	61619795	A	0.03093	0.01833	G	0.1567	1.709
17	rs1042395	78079669	G	0.3943	0.4358	A	0.1567	0.8428
19	rs117588892	41748818	A	0.03093	0.01833	C	0.1567	1.709
19	rs76428401	57956642	A	0.03093	0.01833	G	0.1567	1.709
1	rs59833900	7278011	G	0.09536	0.0725	A	0.1569	1.349
2	rs4377290	158437683	G	0.2603	0.2983	A	0.1569	0.8277
2	rs55675869	179401740	A	0.09536	0.0725	G	0.1569	1.349
2	rs9967806	219878756	A	0.09536	0.0725	G	0.1569	1.349
17	rs7215209	33464864	G	0.09536	0.0725	A	0.1569	1.349
3	rs11712950	36897812	A	0.4792	0.4367	G	0.157	1.187
4	rs17610219	147824864	G	0.3969	0.4383	A	0.157	0.8433
4	rs10032539	147825919	A	0.3969	0.4383	G	0.157	0.8433
9	rs2230792	111660851	A	0.3711	0.3317	G	0.1571	1.189
8	rs13249908	75014430	G	0.4613	0.42	A	0.1572	1.183
1	rs4423040	109265029	C	0.1211	0.1517	A	0.1574	0.7709
3	rs2053506	19375791	G	0.1211	0.1517	A	0.1574	0.7709
4	rs6829000	117553092	G	0.4021	0.4442	A	0.1574	0.8415
5	rs34594012	177987740	A	0.03093	0.04833	G	0.1574	0.6284
6	rs498114	33671857	A	0.03093	0.04833	C	0.1574	0.6284
10	rs1733743	56847522	C	0.3995	0.4408	A	0.1574	0.8438
12	rs6332	5603632	A	0.4021	0.4442	G	0.1574	0.8415
15	rs2077268	33873751	A	0.03093	0.04833	G	0.1574	0.6284
16	rs28407821	46773999	C	0.4665	0.4242	A	0.1574	1.187
18	rs687080	63112979	G	0.3995	0.4408	A	0.1574	0.8438
2	rs78733601	152511874	C	0.06959	0.05	A	0.1575	1.421
3	rs7611149	192984366	G	0.4639	0.4225	A	0.1575	1.183
14	rs2273946	24458162	C	0.1477	0.1187	G	0.1575	1.286
4	rs3755979	88058340	C	0.1649	0.1983	G	0.1576	0.7984
7	rs3898475	119659037	G	0.2448	0.21	A	0.1576	1.22
9	rs4436218	18323073	A	0.4665	0.425	G	0.1577	1.183
9	rs2230793	111659483	C	0.3737	0.3342	A	0.1577	1.189
1	rs11573590	45292173	A	0.1469	0.1183	G	0.1578	1.283
1	rs11588271	51470242	A	0.08763	0.1142	G	0.1578	0.7452
1	rs2691465	57956702	G	0.1649	0.1992	A	0.1578	0.7943
1	rs1199153	152457624	A	0.1469	0.1183	G	0.1578	1.283
2	rs1990760	163124051	A	0.2139	0.1817	G	0.1578	1.226

2	rs4359646	241834907	A	0.1469	0.1183	G	0.1578	1.283
3	rs6769852	82120191	G	0.1263	0.1008	A	0.1578	1.289
6	rs11756977	56420538	A	0.2629	0.3017	G	0.1578	0.8256
7	rs13233571	72971231	A	0.1263	0.1008	G	0.1578	1.289
17	rs4969481	79993212	A	0.2629	0.3017	G	0.1578	0.8256
19	rs34989303	38817876	A	0.08763	0.1142	G	0.1578	0.7452
2	rs997163	161767905	G	0.3144	0.3542	A	0.1579	0.8364
6	rs4713956	36275458	A	0.4716	0.4292	G	0.1579	1.187
4	rs11548325	7436486	A	0.2242	0.2617	C	0.158	0.8156
6	rs847005	108002078	G	0.4046	0.4458	A	0.158	0.8448
7	rs67047829	64452738	A	0.2242	0.2608	G	0.158	0.8191
10	rs3765524	96058298	A	0.2242	0.2608	G	0.158	0.8191
20	rs397020	1205886	G	0.2629	0.3008	A	0.158	0.8289
1	rs78544048	152129087	G	0.3222	0.2838	A	0.1582	1.199
10	rs10763354	52103707	A	0.4098	0.4517	G	0.1582	0.8429
1	rs6537837	110119732	A	0.2809	0.2442	G	0.1584	1.209
2	rs7597919	24563646	A	0.2809	0.2442	G	0.1584	1.209
6	rs1144708	31710020	A	0.4124	0.455	G	0.1584	0.8406
17	rs1513670	41807331	G	0.4124	0.4542	A	0.1585	0.8434
3	rs1352855	68362436	A	0.4149	0.4575	G	0.1586	0.841
6	rs204994	32154998	A	0.1933	0.2283	G	0.1586	0.8098
9	rs200169387	138836946	A	0.1933	0.2283	C	0.1586	0.8098
15	rs1521484	99511793	C	0.4149	0.4575	G	0.1586	0.841
20	rs6056523	9292176	A	0.4149	0.4575	C	0.1586	0.841
2	rs4832657	16726739	G	0.4098	0.4508	A	0.1587	0.8458
6	rs210968	117622150	C	0.03906	0.025	A	0.1587	1.585
11	rs201237546	130332547	A	0.007772	0.0025	G	0.1587	3.125
1	rs4916245	172206026	A	0.4149	0.4567	G	0.1588	0.8439
3	rs7646238	152328890	A	0.4794	0.4375	G	0.1588	1.184
9	rs4977574	22098574	G	0.4794	0.4375	A	0.1588	1.184
17	rs871841	8216468	A	0.2655	0.3038	G	0.1588	0.8281
4	rs1523504	125318885	A	0.4201	0.4624	G	0.1589	0.8423
7	rs10282214	103681973	A	0.4175	0.46	G	0.1589	0.8415
1	rs76015112	152129094	G	0.3222	0.2846	A	0.159	1.194
14	rs36432	72348825	G	0.4175	0.4592	A	0.159	0.8443
4	rs6533579	112591777	A	0.2655	0.3033	G	0.1591	0.83
19	rs12978757	9062415	A	0.3789	0.3392	C	0.1591	1.188
21	rs243629	19220798	G	0.3789	0.3392	A	0.1591	1.188
3	rs9828064	119446862	A	0.3196	0.36	G	0.1592	0.835
4	rs2276970	106510470	G	0.1469	0.1192	A	0.1593	1.273
10	rs17670074	19705383	C	0.3814	0.3408	A	0.1593	1.193
11	rs1792134	78360169	A	0.4227	0.465	G	0.1593	0.8424
16	rs13926	3724465	G	0.4227	0.465	C	0.1593	0.8424

2	rs6757496	8978676	A	0.3196	0.3592	G	0.1594	0.838
15	rs3809557	96873212	G	0.2268	0.2642	A	0.1594	0.8171
2	rs7570469	79709354	A	0.482	0.4408	G	0.1595	1.18
1	rs4379719	201775746	G	0.4845	0.4433	A	0.1596	1.18
2	rs11691034	168518206	A	0.3814	0.3417	C	0.1597	1.188
5	rs7716600	44875005	A	0.4278	0.47	C	0.1597	0.8432
7	rs6463843	8838717	A	0.4923	0.45	C	0.1597	1.185
13	rs9778	47354101	A	0.3814	0.3417	G	0.1597	1.188
14	rs452036	23865885	A	0.1675	0.1383	G	0.1597	1.253
2	rs10490113	59499347	C	0.04897	0.0325	A	0.1598	1.533
3	rs4345115	167837748	A	0.4253	0.4667	G	0.1598	0.8456
4	rs10001249	1535004	A	0.2835	0.2467	G	0.1598	1.208
5	rs247008	131447104	A	0.4253	0.4667	G	0.1598	0.8456
6	rs2071543	32811629	A	0.2165	0.1842	C	0.1598	1.224
3	rs17181170	87173324	A	0.268	0.3067	G	0.1599	0.8279
4	rs2302212	17818885	G	0.1675	0.2017	A	0.1599	0.7966
6	rs1417352	106899226	G	0.268	0.3067	A	0.1599	0.8279
10	rs359312	17388143	A	0.4897	0.4483	G	0.1599	1.181
13	rs1575891	48198716	A	0.3222	0.3625	G	0.1599	0.8358
13	rs8002725	78188078	G	0.1675	0.2017	A	0.1599	0.7966
18	rs7235971	30941974	G	0.1675	0.2017	A	0.1599	0.7966
3	rs2293370	119219934	A	0.3273	0.2883	G	0.16	1.201
3	rs59508481	153839959	A	0.433	0.475	G	0.16	0.844
7	rs10267413	115327840	G	0.4923	0.4508	A	0.16	1.181
9	rs12375547	127616537	A	0.4278	0.4692	G	0.16	0.846
17	rs2072279	13980350	A	0.433	0.475	G	0.16	0.844
1	rs267733	150958836	G	0.09536	0.07333	A	0.1601	1.332
1	rs182832526	167865921	A	0.007772	0.002513	C	0.1601	3.11
2	rs17655123	220037444	A	0.1443	0.1761	G	0.1601	0.789
6	rs583788	144600884	C	0.5	0.4575	A	0.1601	1.186
9	rs1801968	132580901	G	0.09536	0.07333	C	0.1601	1.332
17	rs1871892	33448818	A	0.09536	0.07333	G	0.1601	1.332
18	rs6508425	23836019	G	0.09536	0.07333	A	0.1601	1.332
1	rs199569261	887967	A	0.007732	0.0025	G	0.1602	3.109
1	rs141809370	1289863	A	0.007732	0.0025	C	0.1602	3.109
1	rs140537393	12726464	C	0.007732	0.0025	A	0.1602	3.109
1	rs145603590	157773757	A	0.007732	0.0025	C	0.1602	3.109
1	rs117326234	205801881	A	0.007732	0.0025	G	0.1602	3.109
1	rs151099610	222803248	A	0.007732	0.0025	G	0.1602	3.109
1	rs41268715	225521101	G	0.007732	0.0025	C	0.1602	3.109
1	rs117876231	226074572	G	0.007732	0.0025	C	0.1602	3.109
2	rs2230517	112786049	A	0.007732	0.0025	G	0.1602	3.109
2	rs184647177	113671389	A	0.007732	0.0025	C	0.1602	3.109

2	rs35732034	163124596	A	0.007732	0.0025	G	0.1602	3.109
2	rs200216092	233712060	A	0.007732	0.0025	C	0.1602	3.109
2	rs148118779	234067017	G	0.007732	0.0025	A	0.1602	3.109
2	rs140729649	238485990	G	0.007732	0.0025	C	0.1602	3.109
3	rs1892814	38045770	G	0.007732	0.0025	C	0.1602	3.109
3	rs117281323	38565618	A	0.007732	0.0025	G	0.1602	3.109
3	rs201758291	52836483	A	0.007732	0.0025	G	0.1602	3.109
3	rs76587478	57108101	A	0.007732	0.0025	G	0.1602	3.109
3	rs6773904	57419497	A	0.4356	0.4775	G	0.1602	0.8444
3	rs202114850	153840104	G	0.007732	0.0025	A	0.1602	3.109
3	rs9877210	192019370	G	0.4356	0.4775	A	0.1602	0.8444
4	rs184242255	8218727	A	0.007732	0.0025	G	0.1602	3.109
4	rs186577808	83839842	C	0.007732	0.0025	A	0.1602	3.109
4	rs76859998	110437762	G	0.007732	0.0025	A	0.1602	3.109
4	rs187060840	186545106	A	0.007732	0.0025	C	0.1602	3.109
5	rs148954387	147207583	G	0.007732	0.0025	A	0.1602	3.109
5	rs12522451	163009137	G	0.268	0.3058	A	0.1602	0.8312
5	rs10037618	179301972	A	0.007732	0.0025	G	0.1602	3.109
6	rs116696494	3106044	A	0.007732	0.0025	T	0.1602	3.109
6	rs9765960	31413000	A	0.1114	0.0875	G	0.1602	1.307
6	rs201901865	34495134	G	0.007732	0.0025	A	0.1602	3.109
6	rs191227648	39512337	A	0.007732	0.0025	G	0.1602	3.109
6	rs147487242	51777251	A	0.007732	0.0025	G	0.1602	3.109
6	rs139636206	84870584	A	0.007732	0.0025	G	0.1602	3.109
6	rs200052023	109775021	A	0.007732	0.0025	G	0.1602	3.109
7	rs181688175	151135194	G	0.007732	0.0025	A	0.1602	3.109
8	rs144953583	124338171	A	0.007732	0.0025	G	0.1602	3.109
8	rs200893203	145007500	A	0.007732	0.0025	G	0.1602	3.109
9	rs146684909	38395983	A	0.007732	0.0025	G	0.1602	3.109
9	rs201578843	103109242	G	0.007732	0.0025	A	0.1602	3.109
9	rs142639501	115962182	G	0.007732	0.0025	A	0.1602	3.109
9	rs199513008	125674231	A	0.007732	0.0025	G	0.1602	3.109
9	rs202036292	138392955	A	0.007732	0.0025	G	0.1602	3.109
10	rs182887196	13375944	G	0.007732	0.0025	A	0.1602	3.109
10	rs117396201	30728042	A	0.007732	0.0025	G	0.1602	3.109
10	rs200309792	70182061	G	0.007732	0.0025	A	0.1602	3.109
10	rs2286734	115389448	A	0.007732	0.0025	C	0.1602	3.109
11	rs12574779	3249219	C	0.007732	0.0025	A	0.1602	3.109
11	rs190441041	5364561	A	0.007732	0.0025	G	0.1602	3.109
11	rs4582927	26728438	G	0.1443	0.1758	A	0.1602	0.7906
11	rs144724685	46564032	A	0.007732	0.0025	G	0.1602	3.109
11	rs150465209	48266695	A	0.007732	0.0025	G	0.1602	3.109
11	rs143337271	56128510	A	0.007732	0.0025	G	0.1602	3.109

11	rs186820568	62415907	A	0.007732	0.0025	G	0.1602	3.109
11	rs144949543	73179451	A	0.007732	0.0025	C	0.1602	3.109
11	rs192278505	77301121	A	0.007732	0.0025	G	0.1602	3.109
11	rs2070661	108043988	A	0.4508	0.4925	G	0.1602	0.8458
11	rs201184697	117076940	A	0.007732	0.0025	G	0.1602	3.109
12	rs200750609	4791379	A	0.007732	0.0025	G	0.1602	3.109
12	rs118150543	27117662	A	0.007732	0.0025	G	0.1602	3.109
12	rs201986662	109041188	G	0.007732	0.0025	A	0.1602	3.109
12	rs143232411	133354262	A	0.007732	0.0025	G	0.1602	3.109
12	rs116985064	133503670	G	0.007732	0.0025	A	0.1602	3.109
14	rs201279659	24573156	A	0.007732	0.0025	G	0.1602	3.109
14	rs78100888	96813521	A	0.007732	0.0025	G	0.1602	3.109
15	rs11638723	45249121	G	0.007732	0.0025	C	0.1602	3.109
15	rs2729835	63433766	G	0.007732	0.0025	A	0.1602	3.109
15	rs141141657	85669541	A	0.007732	0.0025	G	0.1602	3.109
15	rs2677744	91450441	G	0.007732	0.0025	A	0.1602	3.109
15	rs3764738	101605978	T	0.007732	0.0025	A	0.1602	3.109
15	rs3764740	101606290	A	0.007732	0.0025	G	0.1602	3.109
15	rs3752321	101609009	A	0.007732	0.0025	G	0.1602	3.109
16	rs201835041	24582877	A	0.007732	0.0025	G	0.1602	3.109
16	rs77951841	55725912	G	0.007732	0.0025	A	0.1602	3.109
17	rs202193147	4349455	A	0.007732	0.0025	G	0.1602	3.109
17	rs148588034	10401217	A	0.007732	0.0025	G	0.1602	3.109
17	rs140698013	11827206	A	0.007732	0.0025	G	0.1602	3.109
17	rs143989580	16323461	A	0.007732	0.0025	G	0.1602	3.109
17	rs3730017	26109102	A	0.007732	0.0025	G	0.1602	3.109
17	rs201357781	27185772	G	0.007732	0.0025	A	0.1602	3.109
17	rs147293670	40330238	A	0.007732	0.0025	C	0.1602	3.109
17	rs201315770	61315288	G	0.007732	0.0025	A	0.1602	3.109
17	rs115348708	61897716	G	0.007732	0.0025	C	0.1602	3.109
18	rs2289664	25532304	G	0.007732	0.0025	A	0.1602	3.109
18	rs188733917	29867517	A	0.007732	0.0025	G	0.1602	3.109
18	rs8099038	49847408	A	0.268	0.3058	G	0.1602	0.8312
19	rs200838945	8151993	A	0.007732	0.0025	G	0.1602	3.109
19	rs117203028	11040263	A	0.007732	0.0025	G	0.1602	3.109
19	rs201102461	11215926	A	0.007732	0.0025	G	0.1602	3.109
19	rs75257288	33247965	A	0.007732	0.0025	G	0.1602	3.109
19	rs141053619	45314515	A	0.007732	0.0025	G	0.1602	3.109
19	rs200295807	47137864	A	0.007732	0.0025	G	0.1602	3.109
20	rs56342286	377296	A	0.007732	0.0025	G	0.1602	3.109
20	rs183328639	31606510	A	0.007732	0.0025	G	0.1602	3.109
20	rs147472094	37580996	A	0.007732	0.0025	G	0.1602	3.109
2	rs13424243	228102723	C	0.06186	0.085	G	0.1603	0.7098

8	rs9297605	120803637	G	0.4974	0.4558	C	0.1603	1.182
2	rs75447317	242814639	G	0.4407	0.4833	A	0.1604	0.8424
5	rs3730089	67588148	A	0.1237	0.1533	G	0.1604	0.7795
10	rs2504352	35671151	A	0.384	0.3442	G	0.1604	1.188
14	rs767983	27180335	A	0.4381	0.48	C	0.1604	0.8448
2	rs1130866	85893741	A	0.3273	0.2892	G	0.1605	1.196
3	rs7627629	188956297	A	0.4433	0.4858	G	0.1605	0.8427
6	rs1140809	30611676	C	0.5026	0.4608	A	0.1605	1.182
10	rs915188	61198691	G	0.5026	0.4608	A	0.1605	1.182
12	rs7310409	121424861	A	0.5026	0.4608	G	0.1605	1.182
19	rs1233428	44778405	A	0.3273	0.2892	C	0.1605	1.196
5	rs55915030	149003627	A	0.07474	0.1	G	0.1606	0.727
10	rs7069367	84074976	G	0.4459	0.4883	A	0.1606	0.8431
10	rs3781354	105385350	A	0.3866	0.3458	G	0.1606	1.192
13	rs3765534	95815415	A	0.07474	0.1	G	0.1606	0.727
16	rs142522686	2848610	A	0.07474	0.1	G	0.1606	0.727
16	rs58782004	2848986	A	0.07474	0.1	C	0.1606	0.727
20	rs1008953	43980726	A	0.4459	0.4883	G	0.1606	0.8431
8	rs34309584	42186655	A	0.007732	0.002504	G	0.1607	3.104
11	rs78680181	65363063	A	0.007732	0.002504	G	0.1607	3.104
12	rs10842971	9303296	A	0.08247	0.06167	T	0.1607	1.368
16	rs8057551	27434927	A	0.4485	0.4908	G	0.1607	0.8435
5	rs655601	180226673	C	0.1057	0.1342	A	0.1608	0.7625
6	rs1265099	31105413	A	0.4536	0.4958	G	0.1608	0.8441
7	rs66827095	44134391	G	0.4536	0.4958	A	0.1608	0.8441
7	rs10265083	64863392	A	0.2861	0.2487	G	0.1608	1.21
7	rs7782376	116577124	G	0.4536	0.4958	A	0.1608	0.8441
22	rs5746962	17493520	A	0.5129	0.4708	G	0.1608	1.183
6	rs7739323	83667045	A	0.1057	0.1333	G	0.1609	0.768
14	rs12884468	35852486	A	0.4485	0.49	G	0.1609	0.8463
18	rs12606152	64445511	A	0.5206	0.4783	C	0.1609	1.184
20	rs12710319	49382148	A	0.3247	0.3642	G	0.1609	0.8397
21	rs2834442	35690786	A	0.4562	0.4983	T	0.1609	0.8445
3	rs7643736	3767186	G	0.4588	0.5008	A	0.161	0.8448
4	rs546829	74956372	A	0.4613	0.5033	T	0.161	0.8451
5	rs459193	55806751	A	0.5309	0.4883	G	0.161	1.186
6	rs6922743	63967017	A	0.5309	0.4883	G	0.161	1.186
9	rs76547823	34257979	A	0.1933	0.1617	G	0.161	1.243
13	rs1411328	73883426	A	0.4588	0.5008	G	0.161	0.8448
1	rs200084754	109812674	A	0.007732	0.002508	C	0.1611	3.099
3	rs4334660	23176416	A	0.4639	0.5058	G	0.1611	0.8454
3	rs13303	52558008	A	0.4639	0.5058	G	0.1611	0.8454
9	rs773506	93975471	A	0.2861	0.2492	G	0.1611	1.208

12	rs4765819	1563871	A	0.1701	0.14	G	0.1611	1.259
16	rs200675829	1270449	A	0.007732	0.002508	G	0.1611	3.099
2	rs4894234	177896927	C	0.5129	0.4717	A	0.1612	1.179
8	rs10282929	144681777	C	0.5129	0.4717	A	0.1612	1.179
17	rs11078312	2600186	A	0.3273	0.3673	G	0.1613	0.8383
2	rs70940832	234091138	A	0.03866	0.025	G	0.1615	1.568
3	rs180829030	169710839	C	0.03866	0.025	A	0.1615	1.568
5	rs191835914	74646765	C	0.03866	0.025	A	0.1615	1.568
7	rs139757692	143043267	A	0.03866	0.025	G	0.1615	1.568
9	rs1322254	114453925	A	0.03866	0.025	C	0.1615	1.568
10	rs3729496	50821191	A	0.3299	0.2917	C	0.1615	1.196
12	rs78128744	46243406	G	0.03866	0.025	A	0.1615	1.568
21	rs9974927	45821582	C	0.03866	0.025	A	0.1615	1.568
20	rs2235592	39990614	G	0.4665	0.5075	A	0.1616	0.8486
3	rs12634179	16250068	G	0.06959	0.05083	C	0.1618	1.397
3	rs11710077	38657899	A	0.06959	0.05083	T	0.1618	1.397
6	rs2760118	24503590	A	0.2191	0.1867	G	0.1618	1.222
6	rs2278106	94120219	A	0.1289	0.1033	G	0.1618	1.284
6	rs2278107	94120639	G	0.1289	0.1033	A	0.1618	1.284
9	rs1573025	100991430	A	0.2191	0.1867	C	0.1618	1.222
14	rs10146482	75574087	G	0.2191	0.1867	A	0.1618	1.222
14	rs8017642	75590822	G	0.2191	0.1867	A	0.1618	1.222
14	rs910316	75626042	A	0.2191	0.1867	C	0.1618	1.222
16	rs11466023	3299586	A	0.06959	0.05083	G	0.1618	1.397
2	rs6542095	113529183	A	0.2732	0.3125	G	0.162	0.827
9	rs879368	100995758	A	0.2216	0.1878	C	0.162	1.231
12	rs1133690	132626668	A	0.2216	0.1878	G	0.162	1.231
18	rs8098316	20672555	A	0.25	0.2158	C	0.162	1.211
1	rs2297909	200960307	A	0.1985	0.2333	G	0.1621	0.8135
5	rs26232	102596720	A	0.232	0.2692	G	0.1621	0.82
12	rs2694660	80133070	C	0.3299	0.2925	A	0.1624	1.191
2	rs60028731	56420006	A	0.09021	0.1175	T	0.1625	0.7447
7	rs2361519	48287840	A	0.1469	0.1783	G	0.1627	0.7934
4	rs1352714	155243604	A	0.1495	0.1217	G	0.1628	1.269
4	rs12500437	155244475	C	0.1495	0.1217	A	0.1628	1.269
5	rs402710	1320722	A	0.3325	0.2947	G	0.1628	1.192
5	rs10089	127522543	A	0.3943	0.3542	G	0.1628	1.187
6	rs12722039	32605284	A	0.09794	0.075	G	0.1629	1.339
7	rs2072183	44579180	G	0.3918	0.3525	C	0.1629	1.183
11	rs79686423	6977311	A	0.09794	0.075	G	0.1629	1.339
11	rs3740862	94862288	A	0.05928	0.04167	G	0.163	1.449
13	rs9546897	36828237	G	0.3368	0.3767	A	0.163	0.8404
14	rs45584133	20528250	A	0.05928	0.04167	C	0.163	1.449

14	rs45585336	20528682	G	0.05928	0.04167	A	0.163	1.449
12	rs4238010	4118317	G	0.04124	0.06083	A	0.1631	0.664
22	rs2076213	44322922	C	0.04124	0.06083	A	0.1631	0.664
10	rs3758449	26446312	A	0.3351	0.3758	G	0.1632	0.8368
12	rs2271979	94772742	A	0.3351	0.3758	G	0.1632	0.8368
2	rs847194	176958701	C	0.3351	0.2967	A	0.1634	1.195
8	rs35102108	10383138	A	0.2345	0.2717	G	0.1634	0.8214
10	rs12268007	50025446	A	0.1979	0.1658	C	0.1634	1.241
10	rs4086116	96707202	A	0.1263	0.1567	G	0.1634	0.7781
21	rs2823357	16914905	A	0.3351	0.2967	G	0.1634	1.195
5	rs17853861	120021817	A	0.05155	0.0725	C	0.1635	0.6953
9	rs4977638	24055823	A	0.1959	0.1642	C	0.1635	1.24
11	rs4758258	7949350	A	0.1263	0.1558	G	0.1635	0.783
11	rs2304733	12903443	G	0.1263	0.1558	A	0.1635	0.783
3	rs1403069	70274229	G	0.2345	0.2708	A	0.1636	0.8249
4	rs2383393	180664653	A	0.2526	0.2183	G	0.1636	1.21
6	rs2252755	152469188	G	0.2345	0.2708	C	0.1636	0.8249
19	rs11545619	53116643	G	0.05155	0.07333	A	0.1636	0.6868
1	rs2821008	3421897	G	0.2216	0.1892	A	0.1637	1.221
17	rs56716962	4451453	A	0.2784	0.3172	G	0.1639	0.8303
1	rs12742537	63346976	G	0.3995	0.3592	A	0.164	1.187
5	rs1478453	73207372	A	0.1727	0.2067	T	0.164	0.8012
9	rs888219	128929023	A	0.2784	0.3167	C	0.164	0.8323
13	rs7321057	51682014	A	0.3995	0.3592	G	0.164	1.187
6	rs4712056	53989526	G	0.3351	0.2975	A	0.1642	1.19
7	rs2307254	48016353	A	0.1082	0.1367	C	0.1644	0.7668
10	rs10740391	73589418	A	0.3995	0.36	G	0.1645	1.183
4	rs3733413	187629538	A	0.1082	0.1358	G	0.1646	0.7723
14	rs6571820	38221992	A	0.4021	0.3617	G	0.1646	1.187
16	rs3803662	52586341	G	0.4021	0.3617	A	0.1646	1.187
20	rs6515646	2593863	G	0.3402	0.38	A	0.1646	0.8413
2	rs8207	170493863	G	0.01804	0.0325	A	0.1648	0.5469
10	rs2275443	59956041	A	0.2371	0.2734	G	0.1648	0.826
13	rs2762051	50835715	A	0.01804	0.0325	G	0.1648	0.5469
15	rs2271433	85149771	C	0.01804	0.0325	A	0.1648	0.5469
19	rs76009806	51981879	A	0.01804	0.0325	G	0.1648	0.5469
22	rs2239785	36661330	G	0.1521	0.1233	A	0.1649	1.275
4	rs2278542	187171427	A	0.08505	0.06333	C	0.165	1.375
6	rs9322660	102894075	C	0.4046	0.3642	A	0.1651	1.187
1	rs2296225	21031042	G	0.3402	0.3017	A	0.1652	1.194
21	rs2298437	31869286	G	0.3428	0.3825	A	0.1652	0.842
5	rs76854341	15928163	A	0.04897	0.03333	G	0.1653	1.493
5	rs117878510	22078686	C	0.04897	0.03333	G	0.1653	1.493

5	rs115634193	176304670	A	0.04897	0.03333	G	0.1653	1.493
10	rs73302786	50339754	A	0.04897	0.03333	C	0.1653	1.493
10	rs74797959	72462119	A	0.04897	0.03333	G	0.1653	1.493
17	rs117298907	43111558	A	0.04897	0.03333	G	0.1653	1.493
1	rs2992753	18808292	C	0.07732	0.1025	A	0.1657	0.7338
6	rs1063646	31107648	A	0.07732	0.1017	G	0.1657	0.7405
6	rs7757012	31108562	G	0.07732	0.1017	A	0.1657	0.7405
6	rs9263739	31111356	A	0.07732	0.1017	G	0.1657	0.7405
7	rs2074749	107746432	A	0.07732	0.1017	G	0.1657	0.7405
14	rs12896583	92537379	G	0.07732	0.1017	A	0.1657	0.7405
1	rs75124417	176105631	C	0.09794	0.07583	G	0.1659	1.323
6	rs3806157	32373801	C	0.3454	0.385	A	0.1659	0.8427
6	rs176248	32965942	A	0.1314	0.1058	G	0.1659	1.279
6	rs10499052	109885475	A	0.1985	0.1667	G	0.1659	1.238
9	rs2990659	6722212	A	0.3454	0.385	G	0.1659	0.8427
10	rs2478568	18266989	G	0.3402	0.3025	A	0.1659	1.189
4	rs35286445	71384537	A	0.1753	0.2083	G	0.166	0.8075
6	rs2621321	32789480	G	0.2835	0.3217	A	0.166	0.8344
9	rs1329008	32695752	G	0.4072	0.3675	A	0.1661	1.182
9	rs10760706	102723692	G	0.2397	0.2767	A	0.1661	0.8242
5	rs432898	65532505	G	0.3479	0.3883	A	0.1662	0.8405
5	rs2287926	82815408	A	0.2242	0.192	G	0.1662	1.216
17	rs11557467	38028634	A	0.2964	0.2592	C	0.1662	1.204
1	rs13376679	47726087	G	0.007732	0.01833	A	0.1663	0.4172
1	rs6666652	94461717	A	0.007732	0.01833	C	0.1663	0.4172
1	rs117254535	214820570	A	0.007732	0.01833	G	0.1663	0.4172
2	rs76509206	69746142	C	0.007732	0.01833	G	0.1663	0.4172
2	rs143180658	202401019	A	0.007732	0.01833	G	0.1663	0.4172
2	rs1135173	234054873	A	0.007732	0.01833	G	0.1663	0.4172
2	rs114511558	238296273	A	0.007732	0.01833	G	0.1663	0.4172
2	rs3751108	238427223	A	0.007732	0.01833	G	0.1663	0.4172
3	rs190176472	38835449	A	0.007732	0.01833	G	0.1663	0.4172
5	rs6183	42719092	A	0.007732	0.01833	C	0.1663	0.4172
6	rs9342464	66005888	G	0.07216	0.0525	A	0.1663	1.404
7	rs79480470	112102071	G	0.007732	0.01833	C	0.1663	0.4172
9	rs3890182	107647655	A	0.07216	0.0525	G	0.1663	1.404
9	rs3780528	113667277	G	0.007732	0.01833	A	0.1663	0.4172
12	rs1948516	44238723	A	0.07216	0.0525	G	0.1663	1.404
12	rs17860300	51737562	G	0.007732	0.01833	A	0.1663	0.4172
19	rs4528684	14351574	A	0.07216	0.0525	G	0.1663	1.404
20	rs186067627	30530763	A	0.007732	0.01833	G	0.1663	0.4172
1	rs192742085	154991027	A	0.007732	0.01917	G	0.1664	0.3988
2	rs75030364	96993477	A	0.01804	0.03333	C	0.1664	0.5328

3	rs144073270	108568054	A	0.007732	0.01917	G	0.1664	0.3988
3	rs139264479	185252919	A	0.007732	0.01917	G	0.1664	0.3988
5	rs116647383	148989216	A	0.007732	0.01917	G	0.1664	0.3988
6	rs6912437	29541394	A	0.01804	0.03333	G	0.1664	0.5328
6	rs3895681	30585771	G	0.01804	0.03333	C	0.1664	0.5328
7	rs17151692	76950699	A	0.007732	0.01917	G	0.1664	0.3988
10	rs166815	49459610	A	0.007732	0.01917	G	0.1664	0.3988
10	rs77858766	100017532	A	0.007732	0.01917	G	0.1664	0.3988
10	rs11509438	106027059	A	0.007732	0.01917	G	0.1664	0.3988
10	rs117527859	129904094	A	0.007732	0.01917	G	0.1664	0.3988
14	rs146529868	105173653	A	0.01804	0.03333	G	0.1664	0.5328
17	rs146754758	18027845	A	0.007732	0.01917	G	0.1664	0.3988
18	rs147116037	724385	G	0.01804	0.03333	A	0.1664	0.5328
19	rs117038725	19744692	A	0.01804	0.03333	G	0.1664	0.5328
19	rs77428121	52919972	A	0.007732	0.01917	G	0.1664	0.3988
7	rs4148844	150730999	A	0.06443	0.0875	G	0.1665	0.7182
12	rs3088190	14982352	G	0.09278	0.1192	C	0.1665	0.756
13	rs7339187	103397387	C	0.09278	0.1192	A	0.1665	0.756
15	rs3743044	50773787	G	0.06443	0.0875	A	0.1665	0.7182
1	rs10492972	10353112	G	0.4098	0.37	A	0.1666	1.182
11	rs28365927	236091	A	0.09278	0.12	G	0.1667	0.75
15	rs2718926	56551747	G	0.3505	0.3908	A	0.1667	0.8412
22	rs117660317	20761193	A	0.116	0.09083	G	0.1667	1.313
17	rs2872507	38040763	A	0.2964	0.26	G	0.1668	1.199
17	rs2305480	38062196	A	0.2964	0.26	G	0.1668	1.199
17	rs2305479	38062217	A	0.2964	0.26	G	0.1668	1.199
10	rs1052420	5920121	A	0.1753	0.145	G	0.167	1.253
10	exm-IND10-2772	27687535	I	0.3454	0.3067	D	0.167	1.193
12	rs2230283	89916811	A	0.1753	0.145	G	0.167	1.253
13	rs543588	30670985	C	0.2861	0.3242	A	0.167	0.8354
5	rs6596075	131742228	C	0.06443	0.08833	G	0.1671	0.7108
12	rs4760790	71634794	A	0.2062	0.2417	G	0.1671	0.815
14	rs41314525	22102745	A	0.4124	0.3725	C	0.1671	1.182
4	rs3733414	187629770	C	0.2062	0.2408	A	0.1672	0.8188
1	rs2429051	44595113	G	0.3454	0.3072	C	0.1673	1.19
7	rs2120365	121818068	G	0.3531	0.3933	A	0.1673	0.8419
15	rs8039243	60145962	A	0.4149	0.3742	G	0.1673	1.186
1	rs227517	177091092	G	0.2603	0.225	A	0.1674	1.212
16	rs1391904	34548934	A	0.116	0.09129	G	0.1674	1.306
21	rs2833556	33285300	A	0.2423	0.2792	G	0.1674	0.8256
14	rs3742536	52507429	A	0.2268	0.1942	G	0.1676	1.217
9	rs1702956	33192940	G	0.3454	0.3075	A	0.1677	1.188
16	rs1532624	57005479	A	0.2423	0.2783	C	0.1677	0.829

11	rs7930678	56185159	G	0.1108	0.1392	A	0.1679	0.771
11	rs7931261	56185689	G	0.1108	0.1392	A	0.1679	0.771
17	rs9916475	39580559	A	0.3557	0.3958	T	0.1679	0.8425
17	rs9916724	39580660	G	0.3557	0.3958	A	0.1679	0.8425
17	rs2361710	78111877	A	0.3557	0.3958	G	0.1679	0.8425
14	rs234144	98151784	G	0.2887	0.3267	A	0.168	0.8364
1	rs11208997	67560956	G	0.01804	0.009167	A	0.1682	1.986
1	rs859098	95330372	G	0.01804	0.009167	A	0.1682	1.986
1	rs199848085	145115783	A	0.01804	0.009167	G	0.1682	1.986
1	rs149740001	157773646	T	0.01804	0.009167	A	0.1682	1.986
1	rs149508727	212798336	G	0.01804	0.009167	A	0.1682	1.986
3	rs188375397	10116221	A	0.01804	0.009167	G	0.1682	1.986
3	rs187728964	49843379	G	0.01804	0.009167	A	0.1682	1.986
3	rs201024956	49934990	C	0.01804	0.009167	A	0.1682	1.986
3	rs182064884	129305540	G	0.01804	0.009167	A	0.1682	1.986
5	rs73022563	140813	A	0.01804	0.009167	C	0.1682	1.986
5	rs11949577	143197	A	0.01804	0.009167	G	0.1682	1.986
5	rs2303654	77805733	G	0.01804	0.009167	A	0.1682	1.986
5	rs61739270	179302921	A	0.01804	0.009167	G	0.1682	1.986
6	rs9332735	31901773	A	0.01804	0.009167	G	0.1682	1.986
6	rs45476300	31911266	A	0.01804	0.009167	G	0.1682	1.986
6	rs3097644	32945530	G	0.01804	0.009167	C	0.1682	1.986
8	rs1375785	83991231	A	0.1753	0.1458	C	0.1682	1.245
10	rs35746503	70992634	A	0.01804	0.009167	G	0.1682	1.986
12	rs185570590	33579218	G	0.01804	0.009167	C	0.1682	1.986
12	rs5744751	133253995	A	0.1521	0.125	G	0.1682	1.255
16	rs45517295	2130243	A	0.01804	0.009167	G	0.1682	1.986
16	rs200524721	75646683	C	0.01804	0.009167	G	0.1682	1.986
16	rs189466547	84801966	G	0.01804	0.009167	A	0.1682	1.986
17	rs17761023	55182878	A	0.01804	0.009167	G	0.1682	1.986
19	rs147992428	35622368	A	0.01804	0.009167	G	0.1682	1.986
19	rs1613662	55536595	G	0.01804	0.009167	A	0.1682	1.986
20	rs35720349	52193660	A	0.1521	0.125	G	0.1682	1.255
22	rs142942577	22300281	A	0.01804	0.009167	G	0.1682	1.986
2	rs4665855	26700700	G	0.03093	0.01917	A	0.1683	1.633
3	rs789852	194327098	A	0.03093	0.01917	G	0.1683	1.633
19	rs141562312	11979254	A	0.03093	0.01917	G	0.1683	1.633
20	rs73598372	58342349	A	0.03093	0.01917	G	0.1683	1.633
22	rs141288673	32914067	C	0.03093	0.01917	G	0.1683	1.633
1	rs4657583	166652275	A	0.3479	0.31	G	0.1685	1.188
13	rs4506781	71728125	A	0.3479	0.31	G	0.1685	1.188
20	rs11699009	31688241	G	0.2294	0.1958	A	0.1685	1.222
20	rs11696307	31688260	A	0.2294	0.1958	G	0.1685	1.222

11	rs330261	36422792	G	0.03351	0.05167	A	0.1686	0.6363
16	rs4889261	81213378	A	0.03351	0.05167	G	0.1686	0.6363
16	rs13339342	81219187	A	0.03351	0.05167	G	0.1686	0.6363
17	rs11079224	54429257	G	0.4201	0.38	A	0.1686	1.182
17	rs9303029	80408815	A	0.2448	0.2817	G	0.1686	0.8269
19	rs3815746	10079093	A	0.2448	0.2817	G	0.1686	0.8269
20	rs17431878	34502107	A	0.116	0.09167	G	0.1686	1.3
10	rs10999947	73434906	A	0.3015	0.2642	G	0.1687	1.203
1	rs3795785	228469870	A	0.3505	0.3117	G	0.1688	1.192
9	rs3793524	112219474	C	0.4175	0.3783	G	0.1688	1.178
17	rs11078126	13003915	G	0.3505	0.3117	A	0.1688	1.192
19	rs2291516	11508177	A	0.3505	0.3117	G	0.1688	1.192
11	rs11225090	101832608	G	0.2088	0.2433	A	0.1689	0.8204
11	rs11225091	101832670	A	0.2088	0.2433	G	0.1689	0.8204
19	rs1061769	44352666	A	0.2088	0.2433	G	0.1689	0.8204
12	rs10861905	108767333	A	0.2448	0.2808	C	0.169	0.8303
16	rs363156	66792714	A	0.4227	0.3825	G	0.169	1.182
17	rs2074518	33324382	A	0.2448	0.2808	G	0.169	0.8303
20	rs156610	52019450	A	0.2629	0.2275	G	0.169	1.211
12	rs10840729	17653834	G	0.4201	0.3808	A	0.1692	1.178
18	rs12970393	42123551	G	0.4201	0.3808	A	0.1692	1.178
4	rs3750025	88534065	A	0.3505	0.3125	G	0.1693	1.187
6	rs2853977	31379304	A	0.1314	0.1617	T	0.1693	0.7848
12	rs1359082	10163375	A	0.201	0.17	C	0.1694	1.228
1	rs6691768	61791863	G	0.4253	0.385	A	0.1695	1.182
2	rs7603997	24524958	A	0.2294	0.1967	G	0.1695	1.216
5	rs13436090	143534	A	0.01804	0.009213	G	0.1695	1.976
8	rs1429659	16262222	A	0.4253	0.385	G	0.1695	1.182
19	rs888930	4288332	G	0.3634	0.4033	A	0.1695	0.8445
6	rs210196	33507723	G	0.134	0.1083	A	0.1698	1.274
9	rs3780135	36840623	G	0.134	0.1083	A	0.1698	1.274
19	rs3745962	38896244	A	0.1778	0.1475	C	0.1698	1.25
7	rs4725982	150637863	G	0.2938	0.3317	A	0.1699	0.8384
19	rs8106269	40447257	A	0.2474	0.2842	G	0.1699	0.8282
1	rs614486	47138819	A	0.1804	0.2142	C	0.17	0.8077
7	rs191610800	149463007	A	0.01289	0.02588	G	0.17	0.4915
5	rs117027344	477479	A	0.01289	0.02583	G	0.1701	0.4923
7	rs6965332	149462621	G	0.01289	0.02583	A	0.1701	0.4923
8	rs117839538	143740311	C	0.01289	0.02583	A	0.1701	0.4923
8	rs34288113	143747389	A	0.01289	0.02583	G	0.1701	0.4923
9	rs28550932	91262387	A	0.1804	0.2133	G	0.1701	0.8117
9	rs2250494	100919318	A	0.4253	0.3858	C	0.1701	1.178
10	rs75862308	5781967	C	0.01289	0.02583	A	0.1701	0.4923

12	rs76626801	19427764	G	0.01289	0.02583	A	0.1701	0.4923
12	rs77598867	19427766	G	0.01289	0.02583	A	0.1701	0.4923
14	rs80142487	105963700	A	0.1546	0.1867	G	0.1701	0.797
15	rs143790069	39883738	A	0.01289	0.02583	G	0.1701	0.4923
17	rs144506499	34097183	A	0.01289	0.02583	G	0.1701	0.4923
17	rs140876567	38643384	A	0.01289	0.02583	C	0.1701	0.4923
17	rs28365947	72869050	A	0.01289	0.02583	C	0.1701	0.4923
1	rs2275344	109325118	A	0.06186	0.04333	G	0.1702	1.456
8	rs16936455	70724754	G	0.2979	0.3367	A	0.1702	0.8361
8	rs201070741	144998444	A	0.02577	0.04174	G	0.1702	0.6074
15	rs4778752	80404656	G	0.06186	0.04333	A	0.1702	1.456
17	rs9895866	48895156	A	0.06186	0.04333	G	0.1702	1.456
19	rs8100114	52627349	A	0.1546	0.1858	G	0.1702	0.8014
4	rs67437265	71888240	A	0.07216	0.05333	G	0.1703	1.381
6	rs549652	33686103	A	0.02577	0.04167	G	0.1703	0.6085
12	rs35444	115552437	G	0.2113	0.2467	A	0.1703	0.8184
14	rs7157940	94563193	A	0.02577	0.04167	G	0.1703	0.6085
16	rs235636	29790864	G	0.02577	0.04167	A	0.1703	0.6085
16	rs235638	29791721	C	0.02577	0.04167	G	0.1703	0.6085
17	rs4789879	77081788	A	0.02577	0.04167	G	0.1703	0.6085
19	rs79573242	10961219	G	0.07216	0.05333	C	0.1703	1.381
1	rs1492899	172615395	A	0.4304	0.39	G	0.1704	1.182
6	rs658087	33665020	T	0.02591	0.04181	A	0.1704	0.6096
19	rs641738	54676763	A	0.232	0.1983	G	0.1704	1.221
2	rs11550347	120980428	G	0.3557	0.3167	C	0.1705	1.191
7	rs2108225	107453103	A	0.4278	0.3883	G	0.1705	1.178
11	rs11225089	101832590	A	0.2113	0.2458	C	0.1705	0.8221
22	rs2228314	42276742	G	0.2655	0.23	C	0.1705	1.21
7	rs17165995	12433347	A	0.2036	0.1717	G	0.1706	1.234
1	rs34976864	32149721	G	0.02577	0.0425	C	0.1707	0.596
5	rs7732714	149006726	C	0.0799	0.105	A	0.1707	0.7402
6	rs2844586	31318024	A	0.02577	0.0425	G	0.1707	0.596
7	rs17852167	149129829	A	0.0799	0.105	G	0.1707	0.7402
8	rs56184717	25735330	A	0.0799	0.105	G	0.1707	0.7402
10	rs55859130	97445357	A	0.02577	0.0425	C	0.1707	0.596
14	rs2899849	36789775	A	0.02577	0.0425	T	0.1707	0.596
14	rs10141024	94391699	G	0.3711	0.4117	A	0.1707	0.8434
20	rs6017342	43065028	A	0.0799	0.1042	C	0.1708	0.7468
1	rs2568958	72765116	G	0.05412	0.07583	A	0.1709	0.6973
1	rs2815752	72812440	G	0.05412	0.07583	A	0.1709	0.6973
4	rs17390445	34893855	A	0.05412	0.07583	G	0.1709	0.6973
10	rs74146946	91163743	G	0.05412	0.07583	A	0.1709	0.6973
10	rs807037	102824349	C	0.4304	0.3908	G	0.1709	1.178

16	rs2292329	84031884	G	0.05412	0.07583	C	0.1709	0.6973
2	rs4832524	18113623	A	0.04124	0.02667	G	0.171	1.57
2	rs77419653	179613264	A	0.04124	0.02667	G	0.171	1.57
9	rs902488	117106522	G	0.05412	0.075	A	0.171	0.7057
9	rs10982174	117110180	A	0.05412	0.075	G	0.171	0.7057
11	rs117056378	107312315	A	0.04124	0.02667	G	0.171	1.57
16	rs220379	3339435	C	0.04124	0.02667	G	0.171	1.57
18	rs4331426	20190795	G	0.04124	0.02667	A	0.171	1.57
8	rs4871930	24691440	G	0.232	0.1992	A	0.1713	1.214
10	rs2448347	62545142	G	0.3582	0.3192	A	0.1714	1.191
14	rs8005745	62185295	A	0.134	0.1641	T	0.1714	0.7882
1	rs75269200	55144466	G	0.1186	0.09333	A	0.1715	1.307
17	rs12949907	27224799	G	0.1546	0.1275	A	0.1715	1.252
1	rs953597	241946766	C	0.3763	0.4167	A	0.1716	0.8446
2	rs7562325	182399097	A	0.3763	0.4167	G	0.1716	0.8446
5	rs2560306	121488203	A	0.1005	0.07833	G	0.1716	1.315
6	rs1029295	32856482	G	0.1005	0.07833	A	0.1716	1.315
18	rs12607841	6986227	A	0.1005	0.07833	G	0.1716	1.315
3	rs9840077	69206654	C	0.299	0.3367	A	0.1717	0.8403
17	rs745400	2266799	G	0.2655	0.2312	A	0.1717	1.202
20	rs6051339	2709659	A	0.1186	0.09349	G	0.1717	1.304
2	rs17005441	70504247	C	0.02577	0.01417	G	0.1718	1.841
2	rs2303603	71827854	A	0.02577	0.01417	G	0.1718	1.841
2	rs35794776	109408159	A	0.02577	0.01417	C	0.1718	1.841
4	rs4689388	6270056	G	0.02577	0.01417	A	0.1718	1.841
4	rs143318227	186548084	A	0.02577	0.01417	G	0.1718	1.841
6	rs2857605	31524851	G	0.1134	0.1425	A	0.1718	0.7697
7	rs78073668	87369109	A	0.02577	0.01417	C	0.1718	1.841
8	rs8192874	59409373	G	0.02577	0.01417	A	0.1718	1.841
10	rs145397956	15883429	T	0.02577	0.01417	A	0.1718	1.841
11	rs2301726	60687275	A	0.02577	0.01417	G	0.1718	1.841
15	rs139450496	33260993	A	0.02577	0.01417	G	0.1718	1.841
19	rs78991146	55994413	A	0.02577	0.01417	G	0.1718	1.841
5	rs3734166	137665323	G	0.4407	0.3998	A	0.1719	1.183
1	rs3213190	236141174	A	0.4381	0.3983	G	0.172	1.178
4	rs79858408	48493237	G	0.3763	0.4158	A	0.172	0.8475
8	rs2648875	129072161	G	0.3763	0.4158	A	0.172	0.8475
10	rs3781264	96070375	G	0.183	0.2167	A	0.172	0.8098
13	rs9547621	37178919	A	0.4407	0.4	C	0.172	1.182
13	rs9515185	110959356	C	0.3031	0.3411	G	0.172	0.84
19	rs1816412	24058196	A	0.4407	0.4	G	0.172	1.182
2	rs6741892	38916970	T	0.183	0.2158	A	0.1721	0.8137
4	rs4596205	31012310	G	0.183	0.2158	A	0.1721	0.8137

3	rs13098911	46235201	A	0.08763	0.06583	G	0.1722	1.363
6	rs1547668	33775446	A	0.04381	0.06333	G	0.1722	0.6777
8	rs9297577	119179057	G	0.134	0.1642	A	0.1722	0.788
12	rs3205421	102108345	G	0.3015	0.3408	A	0.1722	0.835
13	rs4941435	43196187	G	0.3015	0.34	A	0.1722	0.8381
15	rs10518765	54680632	C	0.04381	0.06333	A	0.1722	0.6777
9	rs7861820	108936674	A	0.2345	0.2008	G	0.1723	1.219
1	rs11206407	55119144	A	0.4407	0.4008	G	0.1724	1.178
10	rs10828317	22839628	G	0.4433	0.4025	A	0.1724	1.182
12	rs2711698	47416587	G	0.3789	0.4183	A	0.1724	0.8481
2	rs17268785	56592083	G	0.1572	0.1892	A	0.1725	0.7996
12	rs74659822	55886379	A	0.06701	0.09	G	0.1725	0.7262
13	rs7998781	49138910	A	0.134	0.1633	G	0.1725	0.7928
16	rs3764340	78466437	G	0.06701	0.09	C	0.1725	0.7262
19	rs2972588	8953922	G	0.06701	0.09	A	0.1725	0.7262
19	rs1035442	8961981	C	0.06701	0.09	G	0.1725	0.7262
16	rs7185307	12046899	G	0.4433	0.4033	A	0.1727	1.178
1	rs10797854	183106739	G	0.4482	0.4083	A	0.1728	1.177
5	rs532964	78340286	A	0.268	0.2333	G	0.1728	1.203
11	rs11219544	124135215	C	0.04381	0.06417	A	0.1728	0.6683
14	rs7147132	96577787	G	0.4459	0.405	A	0.1728	1.182
2	rs1527598	34843930	A	0.384	0.4242	G	0.1729	0.8463
6	rs1264327	30850582	A	0.2062	0.1742	G	0.1729	1.232
15	rs8182086	85341859	A	0.384	0.424	G	0.1729	0.8468
2	rs11558741	64124722	A	0.06701	0.09083	C	0.173	0.7189
2	rs6541927	124605930	G	0.01289	0.02667	A	0.173	0.4765
11	rs12291062	76895792	C	0.3041	0.3433	A	0.173	0.8359
12	rs2227277	124821416	A	0.01289	0.02667	G	0.173	0.4765
14	rs9944035	64447776	G	0.06701	0.09083	A	0.173	0.7189
19	rs12608960	6755155	G	0.06701	0.09083	A	0.173	0.7189
19	rs35578165	35613689	A	0.01289	0.02667	G	0.173	0.4765
1	rs6703791	117122130	A	0.4459	0.4058	G	0.1731	1.178
6	rs1322512	152947953	C	0.3041	0.3425	A	0.1731	0.839
17	rs2248821	2268311	G	0.268	0.2337	A	0.1732	1.201
2	rs79762465	186654867	G	0.4485	0.4083	C	0.1734	1.178
6	rs35229355	155929801	A	0.3634	0.325	G	0.1734	1.186
2	rs17031297	43927538	A	0.05155	0.035	C	0.1735	1.498
3	rs80311637	64536648	A	0.05155	0.035	G	0.1735	1.498
3	rs9284813	87152169	G	0.3093	0.2733	A	0.1735	1.19
12	rs149972273	5842040	A	0.05155	0.035	G	0.1735	1.498
1	rs4112788	152551276	A	0.4459	0.4067	G	0.1737	1.174
6	rs2253907	31336870	A	0.4459	0.4067	G	0.1737	1.174
6	rs204900	32056580	C	0.1804	0.1508	A	0.1737	1.239

1	rs12077871	40773150	A	0.1366	0.1108	G	0.1738	1.269
16	rs2302677	53682949	A	0.1366	0.1108	G	0.1738	1.269
17	rs807122	51235062	G	0.2165	0.2508	A	0.1738	0.8253
6	rs505274	32294992	A	0.4485	0.4092	G	0.174	1.174
11	rs2276305	113803104	A	0.1856	0.2183	G	0.1741	0.8157
13	rs4885200	75084120	G	0.399	0.4392	A	0.1742	0.8477
3	rs455863	9956279	A	0.1031	0.08	G	0.1746	1.322
3	rs708567	9960070	A	0.1031	0.08	G	0.1746	1.322
3	rs13314993	33015469	A	0.4536	0.4142	C	0.1746	1.174
8	rs10089461	134225068	A	0.4536	0.4142	G	0.1746	1.174
11	rs12277422	92623877	A	0.3093	0.3483	G	0.1746	0.8377
2	rs2889267	139666757	G	0.2577	0.295	A	0.1747	0.8298
5	rs3749736	147040822	A	0.08763	0.06667	G	0.1747	1.345
6	rs302968	25127176	A	0.1572	0.13	G	0.1747	1.248
3	rs6783318	162123179	G	0.09794	0.1242	A	0.1748	0.7658
7	rs3917503	94945453	G	0.3093	0.3475	A	0.1748	0.8408
11	rs12146487	64026639	A	0.1031	0.08013	G	0.1748	1.319
17	rs820196	73627539	G	0.366	0.3283	A	0.1748	1.181
19	rs12459008	53454789	A	0.3093	0.3475	T	0.1748	0.8408
2	rs12472674	80177744	G	0.3686	0.33	A	0.1749	1.185
6	rs7776426	130152520	C	0.09794	0.125	A	0.1749	0.76
8	rs61756425	8999039	A	0.07474	0.055	C	0.1749	1.388
8	rs2613266	53387502	C	0.3686	0.33	A	0.1749	1.185
12	rs4765623	125320850	A	0.3995	0.44	G	0.1749	0.8467
14	rs2289622	96771959	A	0.1598	0.1917	G	0.1749	0.8021
16	rs1875394	13090833	C	0.3995	0.44	A	0.1749	0.8467
18	rs11664063	6985270	A	0.116	0.1442	G	0.1749	0.7788
4	rs11734372	41673604	G	0.2371	0.2042	A	0.175	1.212
4	rs1996023	45164637	A	0.3144	0.2775	C	0.175	1.194
4	rs344141	77660731	G	0.2371	0.2042	C	0.175	1.212
6	rs12174433	30445476	A	0.2371	0.2042	G	0.175	1.212
1	rs2228084	45479709	G	0.116	0.145	A	0.1751	0.7736
6	rs29232	29611431	G	0.4588	0.4192	A	0.1751	1.175
6	rs4947019	110090049	G	0.2191	0.255	C	0.1751	0.8196
5	rs328694	118969960	G	0.4613	0.4215	A	0.1752	1.175
3	rs2070488	38442490	A	0.2706	0.2367	G	0.1753	1.197
5	rs359457	173279842	A	0.4639	0.4233	G	0.1753	1.179
16	rs757189	17988303	G	0.4639	0.4233	A	0.1753	1.179
2	rs11903403	207824384	C	0.3119	0.3508	A	0.1754	0.8386
8	rs10958476	57095808	G	0.1366	0.1658	A	0.1754	0.7958
9	rs2230794	111659439	G	0.183	0.1525	A	0.1754	1.245
16	rs247897	84476200	A	0.3119	0.3508	T	0.1754	0.8386
21	rs220129	43531632	A	0.183	0.1525	G	0.1754	1.245

1	rs11249215	25297184	G	0.4665	0.4258	A	0.1756	1.179
2	rs13002993	131037447	A	0.4046	0.445	G	0.1756	0.8477
2	rs13420911	207820276	A	0.3119	0.35	G	0.1756	0.8416
6	rs943080	43826627	G	0.3119	0.35	A	0.1756	0.8416
6	rs10948132	44310854	G	0.4665	0.4258	A	0.1756	1.179
7	rs2028028	112318992	A	0.08247	0.1075	G	0.1756	0.7463
12	rs305396	73022080	A	0.4046	0.445	C	0.1756	0.8477
16	rs26857	2260567	G	0.3119	0.3497	A	0.1756	0.8426
17	rs2879097	36889559	A	0.08247	0.1075	G	0.1756	0.7463
4	rs7677913	8586596	A	0.4639	0.4242	G	0.1757	1.175
4	rs2051788	105450529	A	0.3144	0.2783	G	0.1757	1.189
6	rs969694	146394655	G	0.4691	0.4283	A	0.1758	1.179
12	rs7980126	86716229	C	0.2603	0.2975	A	0.1758	0.831
16	rs2292954	89613123	G	0.1392	0.1125	A	0.1759	1.275
2	rs12620556	159196771	G	0.2088	0.1775	A	0.1761	1.223
14	rs10138997	45606287	A	0.1598	0.1317	G	0.1761	1.254
15	rs12593397	42166500	G	0.317	0.28	A	0.1761	1.194
19	rs3746101	2050823	A	0.2088	0.1775	C	0.1761	1.223
19	rs3810291	47569003	A	0.1881	0.2208	G	0.1761	0.8177
1	rs11811919	91916880	A	0.4098	0.45	G	0.1762	0.8486
18	rs8083346	62998775	C	0.4098	0.45	A	0.1762	0.8486
1	rs6424388	70930788	G	0.4742	0.4333	A	0.1763	1.179
4	rs6828560	43328707	G	0.3144	0.3525	A	0.1764	0.8425
10	rs4075501	134738232	G	0.4716	0.4317	A	0.1764	1.175
19	rs8101048	34996252	A	0.4249	0.4657	G	0.1764	0.8475
2	rs3749147	27851918	A	0.2642	0.3008	G	0.1765	0.8347
7	rs2527866	157090296	C	0.4124	0.4525	A	0.1765	0.8491
8	rs6558702	2909992	G	0.1598	0.1319	A	0.1765	1.252
12	rs10845279	11149711	C	0.2216	0.2567	A	0.1765	0.8247
12	rs10845280	11149720	A	0.2216	0.2567	G	0.1765	0.8247
12	rs10845281	11149769	A	0.2216	0.2567	G	0.1765	0.8247
12	rs12226919	11150033	C	0.2216	0.2567	A	0.1765	0.8247
12	rs12226920	11150046	C	0.2216	0.2567	A	0.1765	0.8247
3	rs11720267	129677673	A	0.4149	0.455	G	0.1767	0.8495
6	rs3763341	32979020	A	0.2732	0.2392	G	0.1767	1.196
8	rs10105774	102836613	G	0.2732	0.2392	A	0.1767	1.196
2	rs2909111	206911228	A	0.4175	0.4583	G	0.1768	0.8471
6	rs2239530	30152115	C	0.2397	0.2067	G	0.1768	1.21
14	rs6573250	59790813	A	0.4768	0.4367	G	0.1768	1.176
6	rs9260988	29962515	G	0.2629	0.3	A	0.177	0.8322
6	rs6905157	29971548	G	0.2629	0.3	A	0.177	0.8322
6	rs6926792	29985849	A	0.2629	0.3	G	0.177	0.8322
6	rs6919617	29991699	G	0.2629	0.3	A	0.177	0.8322

6	rs3869068	30004052	A	0.2629	0.3	G	0.177	0.8322
6	rs9261292	30038830	G	0.2629	0.3	C	0.177	0.8322
6	rs2074479	30041009	G	0.2629	0.3	A	0.177	0.8322
6	rs3807035	30044827	A	0.2629	0.3	G	0.177	0.8322
18	rs7232000	76533765	G	0.317	0.3558	A	0.177	0.8403
5	rs1829883	98781103	A	0.3763	0.3375	G	0.1771	1.184
11	rs10128711	18632984	G	0.4201	0.4608	A	0.1771	0.8476
1	rs2274702	205353492	C	0.1031	0.08083	A	0.1772	1.307
2	rs28739019	228493211	A	0.2629	0.2992	G	0.1772	0.8355
2	rs3811515	228883029	C	0.2629	0.2992	A	0.1772	0.8355
4	rs4691896	164085425	A	0.1624	0.1942	G	0.1772	0.8045
14	rs1299013	90965383	G	0.2629	0.2992	A	0.1772	0.8355
17	rs35789723	79612621	A	0.1031	0.08083	G	0.1772	1.307
2	rs10804178	210849283	G	0.4201	0.46	A	0.1773	0.8504
3	rs902321	67411166	G	0.4201	0.46	A	0.1773	0.8504
1	rs2232816	171753039	A	0.4768	0.4375	G	0.1774	1.172
10	rs2242206	61414011	C	0.4768	0.4375	A	0.1774	1.172
20	rs760998	36845738	A	0.4768	0.4375	G	0.1774	1.172
3	rs7610033	15344254	A	0.4227	0.4625	G	0.1775	0.8509
18	rs4499304	13001512	G	0.4794	0.44	C	0.1775	1.172
6	rs735860	53123118	G	0.4871	0.4467	A	0.1776	1.177
10	rs10881994	93668408	A	0.2113	0.1795	G	0.1776	1.225
16	rs3811348	69782855	A	0.1907	0.2236	G	0.1776	0.8182
21	rs2835991	39194878	A	0.4871	0.4467	G	0.1776	1.177
4	rs1389169	28669712	G	0.3196	0.3583	A	0.1777	0.8411
5	rs830230	165402097	G	0.4948	0.4541	A	0.1779	1.178
11	rs66918959	82444021	A	0.4304	0.4708	C	0.1779	0.8493
16	rs13339649	23098422	G	0.1598	0.1325	A	0.1779	1.245
20	rs1122174	33110846	A	0.2423	0.2083	G	0.1779	1.215
1	rs1046331	14143003	A	0.2242	0.26	G	0.178	0.8226
1	rs1316871	66339048	A	0.3196	0.3575	G	0.178	0.8441
6	rs2523674	31436789	A	0.5052	0.465	G	0.178	1.175
6	rs1135056	70961833	G	0.2242	0.26	A	0.178	0.8226
7	rs765114	25316682	A	0.183	0.1542	G	0.178	1.229
8	rs10109439	139307961	A	0.4871	0.4475	G	0.178	1.173
11	rs5104	116692334	G	0.3196	0.3575	A	0.178	0.8441
13	rs9532152	38635214	A	0.4871	0.4475	G	0.178	1.173
16	rs34883368	56500132	A	0.1211	0.09667	G	0.178	1.288
19	rs2240154	1003172	A	0.4897	0.4499	G	0.178	1.173
1	rs41270041	161161284	G	0.0567	0.07833	C	0.1781	0.7072
6	rs9380351	33129746	A	0.4404	0.48	G	0.1781	0.8526
11	rs4754743	101114519	A	0.0567	0.07833	G	0.1781	0.7072
12	rs7313402	108602970	C	0.4897	0.45	A	0.1781	1.173

13	rs2755237	41109429	C	0.2655	0.3025	A	0.1781	0.8333
4	rs147109187	71024346	A	0.01295	0.005833	C	0.1782	2.237
6	rs2523535	31336250	G	0.1392	0.17	A	0.1782	0.7894
8	rs7018455	72180634	A	0.4923	0.4525	G	0.1782	1.173
8	rs1055130	120847188	G	0.4948	0.4549	A	0.1782	1.174
14	rs147724387	24458201	C	0.01295	0.005833	G	0.1782	2.237
14	rs8007846	66262963	A	0.1392	0.17	G	0.1782	0.7894
1	rs36013429	161139738	A	0.0567	0.0775	G	0.1783	0.7155
5	rs1009686	129863694	A	0.4356	0.4758	C	0.1783	0.8501
6	rs791183	160610124	G	0.4304	0.47	A	0.1783	0.8521
10	rs1133837	50901938	A	0.1186	0.1467	G	0.1783	0.7826
10	rs7095325	129913960	A	0.3222	0.285	T	0.1783	1.192
16	rs17232910	89839766	G	0.1186	0.1467	C	0.1783	0.7826
19	rs447802	49116359	A	0.3196	0.2838	G	0.1783	1.185
20	rs1020572	12659751	A	0.4304	0.47	C	0.1783	0.8521
1	rs139345631	1322736	A	0.04124	0.0275	G	0.1784	1.521
3	rs56260729	195594950	A	0.04124	0.0275	G	0.1784	1.521
4	rs117059276	520908	A	0.04124	0.0275	C	0.1784	1.521
5	rs2544600	79733079	A	0.04124	0.0275	G	0.1784	1.521
5	rs259028	79734297	A	0.04124	0.0275	G	0.1784	1.521
5	rs168939	79735858	A	0.04124	0.0275	G	0.1784	1.521
5	rs249038	79745469	A	0.04124	0.0275	G	0.1784	1.521
5	rs17135859	112996654	G	0.04124	0.0275	A	0.1784	1.521
10	rs35118262	43600607	A	0.04124	0.0275	C	0.1784	1.521
11	rs147029675	82880258	A	0.04124	0.0275	C	0.1784	1.521
15	rs12905964	84179115	G	0.4948	0.455	A	0.1784	1.173
17	rs137983973	7490231	G	0.04124	0.0275	C	0.1784	1.521
19	rs12972670	14952238	G	0.04124	0.0275	A	0.1784	1.521
19	rs2304103	33467413	A	0.04124	0.0275	G	0.1784	1.521
19	rs138808565	43772010	A	0.04124	0.0275	C	0.1784	1.521
22	rs16987136	29534621	G	0.04124	0.0275	A	0.1784	1.521
1	rs4650280	75166623	A	0.3222	0.3608	G	0.1785	0.8419
2	rs2646265	238257013	A	0.05155	0.03583	G	0.1785	1.462
4	rs28661939	119219909	A	0.06959	0.0925	C	0.1785	0.7338
6	rs1810126	160872151	A	0.5026	0.4617	G	0.1785	1.178
7	rs6979493	53216005	A	0.4974	0.4575	G	0.1785	1.174
14	rs4903576	77844869	G	0.05155	0.03583	A	0.1785	1.462
14	rs2193595	77845121	G	0.05155	0.03583	A	0.1785	1.462
2	rs3791679	56096892	A	0.2423	0.2092	G	0.1786	1.209
2	rs10186572	141795269	A	0.07474	0.05583	C	0.1786	1.366
7	rs10226193	116292034	A	0.2423	0.2092	G	0.1786	1.209
8	rs74894413	25220572	A	0.07474	0.05583	T	0.1786	1.366
1	rs4655134	23198619	A	0.4459	0.4867	C	0.1787	0.8487

4	rs6847947	23123307	G	0.4356	0.475	A	0.1787	0.8529
3	rs9872536	55453536	A	0.1005	0.1269	C	0.1788	0.769
8	rs10107630	130603635	A	0.4485	0.4892	G	0.1788	0.8491
1	rs2275303	232539219	A	0.4381	0.4775	G	0.1789	0.8533
4	rs3733182	5448429	G	0.451	0.4917	A	0.1789	0.8494
5	rs2082412	158717789	A	0.5103	0.47	G	0.1789	1.175
5	rs13165201	111538602	C	0.3814	0.3433	A	0.179	1.179
5	rs978012	150667016	A	0.5232	0.4825	G	0.179	1.177
6	rs6298	78172992	A	0.4536	0.4942	G	0.179	0.8498
7	rs4298437	103625877	A	0.06186	0.045	G	0.179	1.399
9	rs6149	2635545	A	0.06186	0.045	G	0.179	1.399
9	rs11789412	127589808	G	0.3814	0.3433	A	0.179	1.179
17	rs90951	6981397	A	0.5232	0.4825	G	0.179	1.177
20	rs6063966	52193088	A	0.06186	0.045	G	0.179	1.399
3	rs12488457	130116696	C	0.1856	0.1558	A	0.1791	1.234
6	rs3132506	31176226	G	0.1856	0.1558	A	0.1791	1.234
10	rs2588941	63588911	A	0.4588	0.4992	G	0.1791	0.8504
13	rs17086609	28929711	A	0.4588	0.4992	G	0.1791	0.8504
13	rs9547952	38138689	A	0.09021	0.06833	G	0.1791	1.352
17	rs1135688	73827205	G	0.4562	0.4967	A	0.1791	0.8501
22	rs2006771	31998612	A	0.1856	0.1558	G	0.1791	1.234
5	rs349475	19440168	A	0.3247	0.3633	G	0.1792	0.8427
6	rs2239701	32805049	A	0.4613	0.5017	G	0.1792	0.8508
19	rs167479	11526765	A	0.4613	0.5017	C	0.1792	0.8508
9	rs3808795	17273731	G	0.3247	0.2875	A	0.1793	1.192
9	rs1333048	22125347	C	0.5077	0.4683	A	0.1793	1.171
21	rs9981468	18741831	A	0.5052	0.4658	G	0.1793	1.171
21	rs7279297	43227915	A	0.4665	0.5067	G	0.1793	0.8514
2	rs10929910	14537835	A	0.5206	0.4808	G	0.1794	1.173
10	rs3740015	12131081	A	0.5103	0.4708	C	0.1794	1.171
10	rs3814219	105647095	A	0.1624	0.1342	G	0.1794	1.251
10	rs7098436	124043629	A	0.518	0.4783	G	0.1794	1.172
1	rs183122334	68564392	C	0.01289	0.005833	G	0.1795	2.225
2	rs150546965	54080522	A	0.01289	0.005833	G	0.1795	2.225
2	rs55767983	128088018	G	0.01289	0.005833	A	0.1795	2.225
3	rs117543659	46010113	C	0.01289	0.005833	A	0.1795	2.225
3	rs76048775	100962744	C	0.01289	0.005833	A	0.1795	2.225
6	rs201384398	132938872	A	0.01289	0.005833	G	0.1795	2.225
7	rs201309922	149520478	A	0.01289	0.005833	G	0.1795	2.225
8	rs34346032	8235504	A	0.03608	0.05417	G	0.1795	0.6536
8	rs199731969	96272735	G	0.01289	0.005833	A	0.1795	2.225
8	rs75811370	105001628	G	0.01289	0.005833	A	0.1795	2.225
8	rs201322968	144641997	G	0.01289	0.005833	A	0.1795	2.225

9	rs138967351	1056975	C	0.01289	0.005833	G	0.1795	2.225
9	rs4130590	130107964	G	0.4588	0.4983	A	0.1795	0.8533
9	rs189319790	140007915	A	0.01289	0.005833	G	0.1795	2.225
10	rs3740296	48381942	A	0.01289	0.005833	G	0.1795	2.225
10	rs150283160	95107386	C	0.01289	0.005833	G	0.1795	2.225
11	rs184037935	6589961	G	0.01289	0.005833	A	0.1795	2.225
11	rs60969594	67203442	A	0.01289	0.005833	G	0.1795	2.225
11	rs199946639	119044199	A	0.01289	0.005833	G	0.1795	2.225
11	rs147651078	119058712	A	0.01289	0.005833	G	0.1795	2.225
11	rs2509062	134182375	G	0.01289	0.005833	A	0.1795	2.225
12	rs17755373	999638	A	0.01289	0.005833	G	0.1795	2.225
12	rs4987207	1023218	A	0.01289	0.005833	C	0.1795	2.225
12	rs185754854	93870771	A	0.01289	0.005833	G	0.1795	2.225
13	rs9318554	25020863	G	0.01289	0.005833	A	0.1795	2.225
13	rs9536041	52951802	G	0.01289	0.005833	A	0.1795	2.225
13	rs9536062	52971718	C	0.01289	0.005833	G	0.1795	2.225
13	rs35975899	53035665	A	0.01289	0.005833	T	0.1795	2.225
13	rs200369995	108861099	G	0.01289	0.005833	A	0.1795	2.225
14	rs200587171	104000888	A	0.01289	0.005833	G	0.1795	2.225
15	rs149167939	89847143	A	0.01289	0.005833	G	0.1795	2.225
16	rs200699325	1569934	A	0.01289	0.005833	G	0.1795	2.225
16	rs4149796	3025774	A	0.01289	0.005833	G	0.1795	2.225
16	rs184466930	19452146	C	0.01289	0.005833	G	0.1795	2.225
17	rs16942615	4875628	G	0.03608	0.05417	A	0.1795	0.6536
18	rs200431802	76886315	A	0.01289	0.005833	G	0.1795	2.225
19	rs79277136	1005070	A	0.01289	0.005833	G	0.1795	2.225
19	rs34259399	35757427	A	0.1649	0.1967	G	0.1795	0.8069
19	rs201727290	57888679	G	0.01289	0.005833	A	0.1795	2.225
21	rs146310440	43524018	A	0.01289	0.005833	G	0.1795	2.225
21	rs190664941	47552350	G	0.01289	0.005833	A	0.1795	2.225
22	rs6004901	26423124	G	0.01289	0.005833	C	0.1795	2.225
22	rs7284177	26423477	A	0.01289	0.005833	G	0.1795	2.225
22	rs61737776	29706927	G	0.01289	0.005833	C	0.1795	2.225
22	rs118087710	46930733	A	0.01289	0.005833	G	0.1795	2.225
1	rs6940	159024668	T	0.1933	0.2267	A	0.1796	0.8175
2	rs2704633	18637078	G	0.4613	0.5008	A	0.1796	0.8536
9	rs1832313	38615698	A	0.4639	0.5033	G	0.1796	0.8539
10	rs11812479	9975098	G	0.3247	0.3625	A	0.1796	0.8457
15	rs10906982	84568158	A	0.1933	0.2267	T	0.1796	0.8175
1	rs12069239	16577908	G	0.1649	0.1975	C	0.1797	0.8026
1	rs16855642	180235728	A	0.03351	0.02083	G	0.1797	1.629
1	rs150080259	207076321	C	0.03351	0.02083	A	0.1797	1.629
9	rs79607078	33944586	A	0.03351	0.02083	G	0.1797	1.629

14	rs114526346	20404076	A	0.03351	0.02083	T	0.1797	1.629
19	rs2124920	55872591	A	0.03351	0.02083	G	0.1797	1.629
3	rs7637099	33138549	A	0.4665	0.5058	G	0.1798	0.8542
6	rs3218097	41905275	A	0.2423	0.21	G	0.1798	1.203
15	rs11636318	81625385	G	0.4665	0.5058	A	0.1798	0.8542
3	rs9813316	174708659	G	0.3273	0.3658	A	0.1799	0.8435
19	rs7253312	38622123	A	0.3273	0.3658	G	0.1799	0.8435
17	rs201569320	48655644	A	0.01289	0.005843	G	0.18	2.221
5	rs1077613	173003451	A	0.2706	0.308	G	0.1802	0.8335
6	rs200484	27775674	G	0.1057	0.0825	A	0.1802	1.314
5	rs12523287	38220774	G	0.2706	0.3075	A	0.1803	0.8356
6	rs1890997	40546850	A	0.2706	0.3075	G	0.1803	0.8356
5	rs17855844	141019569	C	0.08505	0.11	G	0.1804	0.7521
11	rs1509170	26500273	G	0.3273	0.29	A	0.1804	1.191
18	rs17782413	61471523	A	0.08505	0.1104	G	0.1804	0.7493
6	rs9472138	43811762	A	0.1057	0.08264	G	0.1805	1.312
2	rs3821033	219507302	A	0.1211	0.0975	G	0.1806	1.276
10	rs11196400	115393948	A	0.1856	0.1567	G	0.1806	1.227
3	rs1450314	35316680	A	0.3299	0.3683	G	0.1807	0.8443
4	rs10213340	19147496	A	0.3299	0.3683	G	0.1807	0.8443
6	rs9394891	42220117	A	0.3299	0.3683	C	0.1807	0.8443
9	rs7040024	845516	C	0.1418	0.1717	A	0.1807	0.797
18	rs12604483	53855525	T	0.1418	0.1717	A	0.1807	0.797
9	rs75660101	116136473	A	0.03608	0.055	C	0.1808	0.6432
2	rs6433472	175143309	G	0.3273	0.2908	A	0.1809	1.186
15	rs12440298	78927589	C	0.1418	0.1725	A	0.1809	0.7923
18	rs1893523	43481075	A	0.3273	0.2908	G	0.1809	1.186
6	rs2518103	49616512	G	0.2294	0.2642	A	0.181	0.8291
7	rs1527482	80427530	A	0.04639	0.06583	G	0.181	0.6903
10	rs17116350	105793750	G	0.1624	0.135	A	0.181	1.242
18	rs3826593	56204747	G	0.1624	0.135	A	0.181	1.242
1	rs760644	171509327	G	0.08505	0.1108	A	0.1811	0.7458
17	rs2292064	7217463	A	0.3866	0.3492	G	0.1811	1.175
2	rs13016400	148563890	A	0.3325	0.3717	G	0.1813	0.842
2	rs2627043	179582537	C	0.3325	0.3717	A	0.1813	0.842
2	rs1813160	71360625	A	0.2732	0.3108	C	0.1814	0.8334
17	rs117633958	27959264	A	0.04639	0.06667	G	0.1814	0.6811
2	rs3791675	56111309	G	0.2448	0.2125	A	0.1815	1.202
16	rs8058922	74695079	C	0.2448	0.2125	A	0.1815	1.202
1	rs60920266	236749730	A	0.1211	0.1492	G	0.1816	0.7862
6	rs1321311	36622900	A	0.1211	0.1492	C	0.1816	0.7862
13	rs1360308	104967115	A	0.3918	0.3533	G	0.1816	1.179
6	rs9470361	36623379	A	0.1211	0.15	G	0.1817	0.781

10	rs525455	13103285	A	0.3892	0.3517	G	0.1817	1.175
10	rs2893923	65261184	A	0.3351	0.3742	G	0.1819	0.8428
15	rs7178634	41634587	G	0.2139	0.1833	A	0.1819	1.212
1	rs146234576	203652352	A	0.02577	0.015	C	0.1822	1.737
2	rs115940994	149247210	G	0.02577	0.015	A	0.1822	1.737
6	rs241457	32901421	C	0.02577	0.015	A	0.1822	1.737
6	rs45467798	35426132	A	0.02577	0.015	G	0.1822	1.737
7	rs41281099	117864712	C	0.02577	0.015	A	0.1822	1.737
8	rs2304789	87567193	A	0.02577	0.015	G	0.1822	1.737
10	rs117647164	64974017	G	0.02577	0.015	A	0.1822	1.737
11	rs17106909	107286943	A	0.2835	0.2492	G	0.1822	1.192
13	rs144938364	25479714	A	0.02577	0.015	G	0.1822	1.737
14	rs57904317	103576526	A	0.02577	0.015	G	0.1822	1.737
16	rs113956264	1997004	A	0.02577	0.015	G	0.1822	1.737
16	rs144183945	31049298	G	0.02577	0.015	A	0.1822	1.737
17	rs146918776	39659245	G	0.02577	0.015	A	0.1822	1.737
18	rs145920224	59167623	A	0.02577	0.015	G	0.1822	1.737
6	rs2523990	30077229	G	0.3918	0.3542	A	0.1823	1.174
18	rs9955521	4083561	C	0.2758	0.3133	A	0.1825	0.8345
6	rs6922302	28295310	C	0.1237	0.09917	G	0.1826	1.282
14	rs3784038	74514674	T	0.1237	0.09917	A	0.1826	1.282
5	rs45625534	109153084	G	0.1057	0.08333	A	0.1827	1.3
6	rs35183513	26370748	A	0.02062	0.03506	G	0.1827	0.5794
4	rs78478598	5527214	C	0.02062	0.035	G	0.1828	0.5805
4	rs77708442	153896160	A	0.02062	0.035	G	0.1828	0.5805
16	rs75909132	90095690	A	0.02062	0.035	G	0.1828	0.5805
20	rs77538589	50408673	A	0.02062	0.035	G	0.1828	0.5805
2	rs7570219	42329329	G	0.3402	0.3792	A	0.1832	0.8443
6	rs4148871	32803316	A	0.1881	0.1592	G	0.1832	1.224
17	rs544198	37319029	C	0.2474	0.215	A	0.1832	1.2
17	rs657672	37319065	A	0.2474	0.215	G	0.1832	1.2
17	rs657723	37319103	G	0.2474	0.215	A	0.1832	1.2
1	rs76232257	155911517	A	0.07732	0.0575	C	0.1833	1.374
2	rs2709363	208520043	G	0.3995	0.3608	A	0.1834	1.178
6	rs2071286	32179896	A	0.1443	0.1742	G	0.1834	0.7998
19	rs8102380	10801185	G	0.2784	0.3158	A	0.1835	0.8355
6	rs4959167	2145136	G	0.1443	0.1175	A	0.1836	1.267
7	rs6962176	148164406	A	0.3325	0.2967	G	0.1837	1.181
17	rs2641256	5126674	G	0.2345	0.2696	A	0.1837	0.83
20	rs6032538	44417675	G	0.3428	0.3817	C	0.1838	0.845
1	rs3024505	206939904	A	0.02062	0.03583	G	0.1839	0.5665
1	rs3024493	206943968	A	0.02062	0.03583	C	0.1839	0.5665
3	rs652026	99442809	G	0.3351	0.2983	A	0.1839	1.185

3	rs9880647	122306920	G	0.3351	0.2983	A	0.1839	1.185
4	rs6847454	126238924	T	0.2345	0.2692	A	0.1839	0.8319
6	rs2275289	105573420	A	0.02062	0.03583	G	0.1839	0.5665
11	rs77534773	113222931	G	0.02062	0.03583	A	0.1839	0.5665
15	rs2271286	42185482	A	0.02062	0.03583	G	0.1839	0.5665
17	rs2303015	46929908	G	0.02062	0.03583	A	0.1839	0.5665
22	rs2076101	39445554	G	0.2345	0.2692	A	0.1839	0.8319
1	rs148929083	32205180	A	0.06443	0.04667	T	0.184	1.407
2	rs1729674	61389737	C	0.3428	0.3808	A	0.1841	0.848
4	rs7442317	29903052	A	0.3428	0.3808	G	0.1841	0.848
6	rs444921	31932177	A	0.1649	0.1375	G	0.1841	1.239
2	rs17848149	170097707	C	0.02835	0.04417	A	0.1842	0.6314
16	rs783762	71007809	A	0.07216	0.09516	G	0.1842	0.7396
6	rs1633021	29746869	G	0.1062	0.08417	A	0.1843	1.293
13	rs9603422	39343822	A	0.1907	0.1608	G	0.1843	1.23
15	rs4411464	63995423	A	0.07216	0.095	G	0.1843	0.7409
1	rs7414489	64165243	A	0.4021	0.3642	C	0.1845	1.174
7	rs1802437	44874113	G	0.4021	0.3642	A	0.1845	1.174
11	rs1785016	115515326	A	0.4046	0.3658	G	0.1846	1.178
13	rs1078421	39679678	G	0.4046	0.3658	A	0.1846	1.178
17	rs550510	46926615	G	0.2809	0.3175	A	0.1846	0.8398
12	rs2072355	4737715	G	0.3454	0.3833	A	0.1847	0.8487
4	rs2980089	4267298	A	0.3376	0.3008	G	0.1848	1.185
11	rs11229457	58207203	A	0.2191	0.1875	G	0.1848	1.216
14	rs3742778	75538217	C	0.2191	0.1875	A	0.1848	1.216
5	rs10055723	128578098	A	0.1237	0.1517	G	0.1849	0.7897
14	rs8005462	97555659	A	0.25	0.2175	G	0.1849	1.199
15	rs4775041	58674695	G	0.25	0.2175	C	0.1849	1.199
16	rs12599339	820888	G	0.3479	0.3871	A	0.1849	0.8448
5	rs2526247	128301885	C	0.05928	0.08083	A	0.185	0.7165
6	rs2076535	32339511	A	0.05928	0.08083	G	0.185	0.7165
6	rs41289932	116784850	G	0.05928	0.08083	C	0.185	0.7165
16	rs151222	20674492	G	0.201	0.235	C	0.185	0.8191
2	rs72648257	179411011	G	0.09021	0.07	A	0.1851	1.317
2	rs3731745	179431594	G	0.09021	0.07	A	0.1851	1.317
2	rs56018860	179433221	G	0.09021	0.07	A	0.1851	1.317
2	rs3813246	179433580	G	0.09021	0.07	A	0.1851	1.317
2	rs3813245	179434139	G	0.09021	0.07	A	0.1851	1.317
2	rs2288570	179444289	G	0.09021	0.07	A	0.1851	1.317
7	rs2392465	36800555	G	0.4046	0.3667	A	0.1851	1.174
9	rs865686	110888478	C	0.09021	0.07	A	0.1851	1.317
11	rs3751055	9069046	A	0.201	0.2342	G	0.1851	0.8229
12	rs10219555	62928633	A	0.3479	0.3867	G	0.1851	0.8464

12	rs61754235	78225299	A	0.09021	0.07	G	0.1851	1.317
12	rs2681492	90013089	G	0.4046	0.3667	A	0.1851	1.174
17	rs10782008	78305871	A	0.4072	0.3683	G	0.1851	1.178
19	rs2230216	863220	G	0.1237	0.1	C	0.1851	1.271
19	rs12983010	39229089	G	0.1237	0.1	A	0.1851	1.271
10	rs71535732	21104613	G	0.08763	0.1125	A	0.1852	0.7577
4	rs7678436	17797966	A	0.2912	0.2558	G	0.1854	1.195
12	rs1520223	102726338	G	0.2912	0.2558	A	0.1854	1.195
14	rs11622475	104509076	A	0.2371	0.2717	G	0.1854	0.8333
14	rs1951550	44319485	G	0.2835	0.3208	A	0.1855	0.8376
21	rs9306160	45107562	A	0.2526	0.2192	G	0.1855	1.204
1	rs61742508	3410644	G	0.1082	0.085	A	0.1857	1.307
6	rs2301527	168445769	A	0.1082	0.085	G	0.1857	1.307
9	rs1360517	13007129	A	0.08763	0.1133	G	0.1857	0.7514
15	rs11073288	38360209	A	0.3505	0.3892	G	0.1857	0.8471
21	rs34115287	47635176	G	0.1082	0.085	A	0.1857	1.307
4	rs4148254	115544713	A	0.1684	0.14	G	0.1859	1.244
2	rs12469459	242716380	A	0.3505	0.3883	T	0.186	0.8501
11	rs10466659	58190112	G	0.2191	0.1883	A	0.186	1.209
13	rs7332268	100597589	G	0.3505	0.3883	A	0.186	0.8501
18	rs11083271	25926059	A	0.3505	0.3883	G	0.186	0.8501
20	rs2245898	44238741	C	0.3505	0.3883	A	0.186	0.8501
3	rs28594654	140406947	A	0.4098	0.3717	G	0.1861	1.174
3	rs9876490	140407260	A	0.4098	0.3717	C	0.1861	1.174
6	rs7765379	32680928	C	0.1443	0.1187	A	0.1861	1.252
5	rs16777	4874950	G	0.1469	0.1775	A	0.1862	0.798
9	rs3739915	135277130	A	0.1727	0.2042	C	0.1862	0.8136
1	rs10801922	117529458	G	0.4124	0.3733	A	0.1863	1.178
3	rs7641556	22350158	G	0.4124	0.3733	A	0.1863	1.178
11	rs7941588	48367073	A	0.1907	0.1619	C	0.1863	1.22
1	rs4515815	20020994	A	0.2526	0.22	G	0.1865	1.198
4	rs10031777	48498290	G	0.3575	0.3958	A	0.1865	0.8493
4	rs4696354	153690842	A	0.2397	0.275	G	0.1865	0.8311
19	rs60213124	48495304	A	0.2526	0.22	G	0.1865	1.198
1	rs10923191	117531911	A	0.4124	0.3742	G	0.1866	1.174
8	rs4236946	55914515	A	0.05412	0.0375	G	0.1866	1.469
12	rs1012315	97639144	A	0.2861	0.3225	G	0.1866	0.8418
14	rs78110192	64540823	G	0.05412	0.0375	A	0.1866	1.469
18	rs7236477	28902322	G	0.05412	0.0375	A	0.1866	1.469
19	rs59912467	1223125	G	0.05412	0.0375	C	0.1866	1.469
22	rs432869	21408430	G	0.05412	0.0375	A	0.1866	1.469
1	rs41313272	67558756	A	0.07732	0.05833	G	0.1867	1.353
1	rs72933970	67561090	G	0.07732	0.05833	A	0.1867	1.353

1	rs1192415	92077097	G	0.1057	0.1325	A	0.1868	0.7736
2	rs12623569	214794743	C	0.3557	0.3942	A	0.1868	0.8484
9	rs10978001	990076	A	0.1057	0.1325	G	0.1868	0.7736
1	rs28599722	248569369	G	0.2036	0.2367	A	0.1869	0.8246
9	rs9969804	95429120	A	0.1933	0.1633	C	0.1869	1.227
1	rs789360	232491315	C	0.4149	0.3767	A	0.1871	1.174
14	rs1309353	102894593	G	0.4149	0.3767	A	0.1871	1.174
7	rs6968385	110047471	G	0.3582	0.3975	A	0.1873	0.8461
17	rs11078928	38064469	G	0.2938	0.2592	A	0.1873	1.189
17	rs9905639	79614154	A	0.2938	0.2592	G	0.1873	1.189
1	rs832156	201175227	C	0.2887	0.3258	A	0.1874	0.8396
1	rs1341631	201175715	C	0.2887	0.3258	A	0.1874	0.8396
1	rs10753895	201176018	A	0.2887	0.3258	G	0.1874	0.8396
5	rs45074	126993249	G	0.3582	0.3967	C	0.1874	0.8491
5	rs248709	126993263	T	0.3582	0.3967	A	0.1874	0.8491
16	rs1869348	81142257	A	0.2887	0.3258	G	0.1874	0.8396
3	rs1353613	130114082	G	0.4175	0.3792	C	0.1876	1.174
17	rs7210026	79612382	A	0.2938	0.2596	G	0.1878	1.187
18	rs9965848	13261896	A	0.3608	0.4	G	0.1878	0.8468
22	rs2272837	50582626	A	0.3608	0.4	G	0.1878	0.8468
1	rs142885686	47138637	A	0.04381	0.02917	G	0.1879	1.525
4	rs6848883	156787340	G	0.04381	0.02917	A	0.1879	1.525
6	rs7758736	32758394	A	0.04381	0.02917	G	0.1879	1.525
8	rs62526872	85800261	A	0.2964	0.2608	G	0.188	1.194
17	rs8067378	38051348	G	0.2964	0.2608	A	0.188	1.194
4	rs45539933	141489068	A	0.09278	0.07167	G	0.1881	1.325
6	rs1055569	31440082	A	0.2927	0.33	G	0.1881	0.8404
6	rs2516440	31440497	A	0.2927	0.33	G	0.1881	0.8404
9	rs1889899	26789940	G	0.1263	0.155	A	0.1881	0.788
11	rs4127353	58125620	A	0.2216	0.1908	G	0.1881	1.207
12	rs61736594	65563712	A	0.1933	0.1642	G	0.1882	1.22
16	rs3213422	72042682	A	0.2423	0.2767	C	0.1882	0.8359
20	exm1524812	8770822	A	0.1933	0.1642	G	0.1882	1.22
20	rs2294597	8770822	A	0.1933	0.1642	G	0.1882	1.22
22	rs8137714	32871383	C	0.1263	0.1542	A	0.1882	0.793
1	rs148007015	186330810	G	0.005155	0.01417	A	0.1883	0.3606
1	rs180909883	241757947	A	0.005155	0.01417	G	0.1883	0.3606
4	rs6828922	68618451	A	0.005155	0.01417	G	0.1883	0.3606
6	rs147630987	46661477	A	0.005155	0.01417	G	0.1883	0.3606
6	rs811925	106547372	C	0.005155	0.01417	G	0.1883	0.3606
6	rs1190788	131520655	A	0.005155	0.01417	G	0.1883	0.3606
7	rs75133741	128482959	A	0.005155	0.01417	G	0.1883	0.3606
9	exm2259280	2054672	C	0.005155	0.01417	G	0.1883	0.3606

9	rs138404604	2054672	C	0.005155	0.01417	G	0.1883	0.3606
9	rs140024771	123927222	A	0.005155	0.01417	C	0.1883	0.3606
10	rs501764	8093034	C	0.005155	0.01417	A	0.1883	0.3606
10	rs145504188	108469093	G	0.005155	0.01417	A	0.1883	0.3606
11	rs61889489	32852167	A	0.005155	0.01417	G	0.1883	0.3606
11	rs1064608	47640429	G	0.3454	0.3092	C	0.1883	1.179
11	rs116483940	64055562	A	0.005155	0.01417	G	0.1883	0.3606
11	rs35089741	67816463	G	0.005155	0.01417	C	0.1883	0.3606
11	rs201811328	118486833	A	0.005155	0.01417	G	0.1883	0.3606
12	rs139761201	7084432	G	0.005155	0.01417	A	0.1883	0.3606
12	rs143225893	7091899	A	0.005155	0.01417	G	0.1883	0.3606
12	rs187675334	121476597	A	0.005155	0.01417	G	0.1883	0.3606
14	rs199704293	64483247	A	0.005155	0.01417	C	0.1883	0.3606
14	rs142398813	95088683	G	0.005155	0.01417	A	0.1883	0.3606
14	rs200982434	105361204	A	0.005155	0.01417	C	0.1883	0.3606
16	rs118055386	18864949	G	0.005155	0.01417	A	0.1883	0.3606
1	rs7542955	238716872	A	0.2912	0.3283	C	0.1884	0.8406
15	rs1374092	72808428	C	0.1753	0.2075	A	0.1884	0.8116
19	rs16999012	22522950	G	0.2912	0.3283	A	0.1884	0.8406
18	rs6104	61570529	G	0.3634	0.4017	C	0.1885	0.8504
6	rs2844452	31882024	A	0.2964	0.2617	G	0.1886	1.189
12	rs9593	109994870	A	0.3479	0.3108	T	0.1886	1.183
13	rs4771948	97087892	C	0.4227	0.3842	A	0.1886	1.174
2	rs11899555	241827798	G	0.1469	0.1208	A	0.1888	1.253
3	rs57657464	112666718	A	0.06443	0.0475	G	0.1889	1.381
6	rs2273709	43184132	C	0.366	0.405	A	0.1889	0.848
6	rs4870044	151901409	G	0.2577	0.2242	A	0.1889	1.202
7	rs1533956	57460667	G	0.4253	0.3867	A	0.189	1.174
20	rs2070325	31673846	A	0.2242	0.1925	G	0.189	1.212
21	rs3746893	43161877	G	0.4253	0.3867	A	0.189	1.174
1	rs116935123	155896514	A	0.005155	0.015	G	0.1891	0.3402
1	rs145850954	178427541	A	0.005155	0.015	G	0.1891	0.3402
2	rs2272428	62107466	G	0.005155	0.015	A	0.1891	0.3402
2	rs12612365	71576412	G	0.005155	0.015	A	0.1891	0.3402
3	rs2279289	66431078	C	0.005155	0.015	G	0.1891	0.3402
6	rs79785688	53767477	C	0.005155	0.015	G	0.1891	0.3402
6	rs6907580	117150008	A	0.005155	0.015	G	0.1891	0.3402
6	rs2229079	117683821	A	0.005155	0.015	G	0.1891	0.3402
7	rs78004519	151860023	G	0.005155	0.015	A	0.1891	0.3402
8	rs139165843	27922169	A	0.005155	0.015	G	0.1891	0.3402
9	rs141549766	116929987	A	0.005155	0.015	G	0.1891	0.3402
9	rs1236913	125133479	A	0.005155	0.015	G	0.1891	0.3402
11	rs201812719	66325618	A	0.005155	0.015	C	0.1891	0.3402

11	rs181358688	67186686	A	0.005155	0.015	G	0.1891	0.3402
13	rs144806444	42303746	A	0.005155	0.015	G	0.1891	0.3402
15	rs150818024	85191826	A	0.005155	0.015	G	0.1891	0.3402
15	rs138813434	89449879	G	0.005155	0.015	A	0.1891	0.3402
16	rs202082300	1143614	C	0.005155	0.015	G	0.1891	0.3402
16	rs146591751	27786313	A	0.005155	0.015	G	0.1891	0.3402
17	rs3803784	72832650	A	0.005155	0.015	G	0.1891	0.3402
19	rs192095216	19823593	C	0.005155	0.015	A	0.1891	0.3402
20	rs3764690	45130338	G	0.005155	0.015	A	0.1891	0.3402
18	rs12185369	40655271	G	0.4227	0.385	A	0.1892	1.17
17	rs743686	39637244	G	0.2938	0.3308	A	0.1894	0.8415
6	rs3129304	32973743	G	0.04897	0.06833	A	0.1895	0.702
7	rs2240466	72856269	A	0.1263	0.1025	G	0.1895	1.266
7	rs1178979	72856430	G	0.1263	0.1025	A	0.1895	1.266
11	rs2298707	93431887	C	0.04897	0.06833	G	0.1895	0.702
11	rs3802773	93432400	A	0.04897	0.06833	G	0.1895	0.702
11	rs3802774	93432454	C	0.04897	0.06833	A	0.1895	0.702
11	rs80145928	93432858	C	0.04897	0.06833	A	0.1895	0.702
11	rs78230233	93433024	C	0.04897	0.06833	A	0.1895	0.702
11	rs77922978	93461895	G	0.04897	0.06833	A	0.1895	0.702
16	rs143198507	55905560	A	0.04897	0.06833	G	0.1895	0.702
17	rs68124408	72588217	A	0.04897	0.06833	G	0.1895	0.702
1	rs11588857	204587047	A	0.2938	0.33	G	0.1896	0.8447
22	rs1633399	19183787	G	0.04897	0.06917	A	0.1897	0.693
22	rs807547	19195680	G	0.04897	0.06917	A	0.1897	0.693
22	rs712952	19197949	A	0.04897	0.06917	G	0.1897	0.693
2	rs2288072	24390517	G	0.2577	0.225	A	0.1898	1.196
1	rs999765	22923873	G	0.07474	0.0975	C	0.19	0.7477
2	rs28505970	1168781	A	0.07474	0.0975	C	0.19	0.7477
6	rs3819299	31322367	C	0.1263	0.1027	A	0.19	1.263
6	rs73749738	83900923	T	0.07474	0.0975	A	0.19	0.7477
9	rs129933	136538149	G	0.07474	0.0975	A	0.19	0.7477
11	rs11600896	56058535	A	0.07474	0.09833	G	0.19	0.7407
14	rs78610683	65197534	A	0.03351	0.02167	G	0.19	1.565
14	rs145013864	65198157	A	0.03351	0.02167	G	0.19	1.565
14	rs28757011	75506696	A	0.03351	0.02167	G	0.19	1.565
15	rs2229749	63937209	C	0.07474	0.09833	G	0.19	0.7407
18	rs6566752	71327754	G	0.4278	0.39	A	0.19	1.17
19	rs151089997	57037037	G	0.03351	0.02167	A	0.19	1.565
20	rs75146235	13251388	C	0.03351	0.02167	G	0.19	1.565
3	rs7627615	183818416	G	0.2098	0.2433	A	0.1901	0.8258
6	rs9262636	31025848	G	0.2242	0.1933	A	0.1901	1.206
14	rs1043831	24909362	A	0.2242	0.1933	G	0.1901	1.206

16	rs28584228	76486581	G	0.2242	0.1933	A	0.1901	1.206
1	rs6679793	157514097	A	0.09021	0.1158	G	0.1902	0.7568
19	rs117753855	53056465	A	0.09021	0.1158	G	0.1902	0.7568
13	rs1590305	38035189	A	0.433	0.3942	G	0.1904	1.174
1	rs12568050	182026813	G	0.2603	0.2267	C	0.1905	1.201
1	rs12564283	182027022	A	0.2603	0.2267	G	0.1905	1.201
3	rs9290663	178429939	T	0.1778	0.21	A	0.1905	0.8137
9	rs11243403	134351343	A	0.1082	0.135	G	0.1906	0.7778
10	rs7076938	115789375	G	0.1778	0.2092	A	0.1906	0.8178
2	rs16867274	10126416	G	0.433	0.395	A	0.1908	1.17
4	rs7668923	85401169	A	0.433	0.395	G	0.1908	1.17
5	rs416574	180626927	G	0.3763	0.415	A	0.1908	0.8504
15	rs2596209	33677731	G	0.433	0.395	A	0.1908	1.17
15	rs12915209	46040457	C	0.433	0.395	A	0.1908	1.17
17	rs570145	18284262	A	0.2603	0.227	G	0.1908	1.198
21	rs9636639	39972727	A	0.3763	0.415	C	0.1908	0.8504
2	exm212840	96952833	G	0.2268	0.195	A	0.191	1.211
2	rs3171927	96952833	G	0.2268	0.195	A	0.191	1.211
14	rs11625819	24910973	A	0.2268	0.195	C	0.191	1.211
3	rs1675953	194373832	A	0.1495	0.1225	G	0.1911	1.259
9	rs7868230	103598789	A	0.3015	0.2667	C	0.1911	1.187
9	rs79134766	127561723	A	0.03866	0.0575	G	0.1911	0.6592
14	rs12147950	37989270	A	0.3763	0.4142	G	0.1911	0.8534
17	rs2074890	15554561	A	0.1495	0.1225	C	0.1911	1.259
17	rs887241	19645938	A	0.03866	0.0575	C	0.1911	0.6592
1	rs3850625	201016296	A	0.05412	0.03833	G	0.1912	1.435
2	rs2592394	176991779	G	0.4356	0.3975	A	0.1912	1.17
3	rs1047855	40468896	A	0.3789	0.4175	G	0.1912	0.851
4	rs12508837	129043204	C	0.3789	0.4175	G	0.1912	0.851
6	rs2240066	31114335	G	0.05412	0.03833	A	0.1912	1.435
11	rs2509010	102562700	A	0.1108	0.0875	G	0.1912	1.3
18	rs1879352	2498054	G	0.05412	0.03833	A	0.1912	1.435
19	rs76218394	46526272	A	0.05412	0.03833	G	0.1912	1.435
1	rs474474	34238283	G	0.2603	0.2275	A	0.1913	1.195
3	rs2718790	180932119	C	0.1289	0.1575	A	0.1913	0.7913
5	rs931555	35803577	A	0.2603	0.2275	G	0.1913	1.195
7	rs9088	150501516	A	0.1289	0.1567	G	0.1914	0.7963
20	rs6050791	25672987	A	0.0799	0.06	G	0.1914	1.36
8	rs1992045	58840924	A	0.1289	0.1042	G	0.1915	1.272
15	exm-IND15-4187	44089415	I	0.09278	0.0725	D	0.1915	1.308
19	rs1051738	10577843	A	0.09278	0.0725	C	0.1915	1.308
10	rs10825659	57650954	G	0.3789	0.4167	A	0.1916	0.8539
1	rs11240594	205896235	G	0.3814	0.42	A	0.1917	0.8516

10	rs7088318	22852948	C	0.4407	0.4017	A	0.1917	1.174
18	rs502337	13157966	A	0.4407	0.4023	C	0.1918	1.171
2	rs9869	159663599	A	0.2268	0.1958	G	0.192	1.205
6	rs2504063	152090707	G	0.2268	0.1958	A	0.192	1.205
11	rs2241571	129794950	G	0.4407	0.4025	A	0.192	1.17
21	rs409782	15596772	A	0.3015	0.2675	C	0.192	1.182
2	rs10171464	139211959	A	0.384	0.4225	G	0.1921	0.8521
3	rs2070492	38357817	A	0.1727	0.1444	G	0.1921	1.237
9	rs1999589	127634429	A	0.3814	0.4192	C	0.1921	0.8545
15	rs4776472	70006873	G	0.3015	0.3392	A	0.1921	0.8412
7	rs7797400	80812398	A	0.4433	0.405	G	0.1924	1.17
5	rs17324782	164306736	G	0.1495	0.1233	A	0.1925	1.249
11	rs12146493	65547333	A	0.3582	0.3217	G	0.1925	1.177
11	rs10765737	95155770	A	0.3866	0.4258	G	0.1925	0.8498
15	rs1030986	66850362	A	0.1495	0.1233	G	0.1925	1.249
4	rs710841	82149831	A	0.1804	0.2125	G	0.1926	0.8158
6	rs7752591	151947068	A	0.384	0.4217	G	0.1926	0.8551
9	rs28429833	91262522	A	0.1804	0.2125	T	0.1926	0.8158
22	rs7291048	35480467	A	0.06186	0.08417	G	0.1926	0.7174
1	rs1921	949608	A	0.1804	0.2117	G	0.1927	0.8198
1	rs20558	183094547	A	0.4459	0.4075	G	0.1927	1.17
3	rs4974437	127183098	G	0.4459	0.4075	A	0.1927	1.17
17	rs3744215	72838649	A	0.4534	0.4147	C	0.1927	1.171
2	rs4927635	1320143	A	0.3892	0.4283	G	0.1928	0.8503
12	rs1798192	123200768	A	0.3892	0.4283	C	0.1928	0.8503
1	rs199675	77547831	A	0.3892	0.4275	G	0.193	0.8532
2	rs2381140	142077946	A	0.3041	0.3408	G	0.193	0.8452
9	rs1053959	114359624	C	0.3892	0.4275	A	0.193	0.8532
17	rs2251660	34252537	C	0.2294	0.1975	A	0.193	1.209
19	rs8104890	52519608	A	0.3041	0.3417	G	0.193	0.8421
6	rs547261	32282033	A	0.4485	0.41	G	0.1931	1.17
6	rs531094	32286102	A	0.4485	0.41	T	0.1931	1.17
6	rs9405090	32298372	G	0.4485	0.41	A	0.1931	1.17
6	rs742582	32299715	A	0.4485	0.41	G	0.1931	1.17
6	rs1033500	32307382	A	0.4485	0.41	G	0.1931	1.17
6	rs2143465	32309352	G	0.4485	0.41	A	0.1931	1.17
6	rs9268368	32333955	G	0.4485	0.41	A	0.1931	1.17
6	rs2073046	32336074	A	0.4485	0.41	G	0.1931	1.17
7	rs6960270	151680072	A	0.1985	0.1692	G	0.1931	1.216
7	rs35682935	151680175	A	0.1985	0.1692	G	0.1931	1.216
16	rs763014	675680	A	0.3099	0.347	G	0.1931	0.8451
3	rs1803155	151545601	G	0.3918	0.4308	A	0.1932	0.8509
15	rs2679623	70208373	G	0.3918	0.4308	A	0.1932	0.8509

17	rs4796604	39890876	A	0.3041	0.27	G	0.1932	1.182
4	rs3775290	187004217	A	0.3582	0.3225	C	0.1933	1.173
6	rs9365785	165262895	A	0.3608	0.3242	G	0.1933	1.177
8	rs10481151	124831834	G	0.3608	0.3242	A	0.1933	1.177
12	rs7296261	7309199	A	0.4459	0.4083	G	0.1933	1.166
22	rs5762326	28106402	A	0.3608	0.3242	G	0.1933	1.177
1	rs190983971	43812255	G	0.01546	0.02833	A	0.1934	0.5387
2	rs13420690	215235867	A	0.3918	0.43	C	0.1934	0.8538
2	rs1131293	242046785	C	0.451	0.4125	A	0.1934	1.17
6	rs61572689	30656509	A	0.01546	0.02833	G	0.1934	0.5387
16	rs195990	23946385	C	0.451	0.4125	A	0.1934	1.17
18	rs35428499	52258548	G	0.01546	0.02833	A	0.1934	0.5387
6	rs2894043	30668488	G	0.2139	0.2475	C	0.1935	0.8274
13	rs1585440	66481815	A	0.3099	0.275	C	0.1935	1.184
17	rs1042393	78079597	A	0.3943	0.4333	G	0.1936	0.8514
6	rs3094097	30633875	G	0.2139	0.2467	A	0.1938	0.8311
13	rs2274285	46103935	A	0.2139	0.2467	G	0.1938	0.8311
13	rs7987165	77145054	A	0.3943	0.4325	C	0.1938	0.8543
18	rs12957087	31328519	G	0.3067	0.3442	A	0.1938	0.843
3	rs1656922	186443018	G	0.3067	0.3433	A	0.1939	0.8461
14	rs28396248	31790886	C	0.1546	0.185	A	0.1939	0.8059
1	rs4950322	146854836	A	0.06701	0.04917	G	0.194	1.389
2	rs115095929	26678082	C	0.06701	0.04917	A	0.194	1.389
3	rs76563279	167035354	A	0.06701	0.04917	G	0.194	1.389
5	rs17101463	143467421	A	0.06701	0.04917	C	0.194	1.389
6	rs1343075	121339179	A	0.451	0.4133	G	0.194	1.166
12	rs10842994	27965150	A	0.2294	0.1983	G	0.194	1.203
16	rs1742446	1918125	A	0.2294	0.1983	C	0.194	1.203
10	rs3810947	50821243	G	0.3634	0.3267	A	0.1941	1.177
6	rs547077	32289318	G	0.4536	0.4158	A	0.1942	1.166
6	rs6929776	32303511	A	0.4536	0.4158	G	0.1942	1.166
7	rs3913932	86144698	A	0.4536	0.4158	G	0.1942	1.166
10	rs1780436	34257612	A	0.2565	0.2925	G	0.1942	0.8344
1	rs4845618	154400015	C	0.4588	0.42	A	0.1944	1.171
2	rs16986953	19942473	A	0.4588	0.42	G	0.1944	1.171
4	rs2973275	37592128	A	0.09278	0.1175	G	0.1944	0.7681
15	rs3743211	75503147	G	0.201	0.1708	A	0.1944	1.221
2	rs6547243	79761016	A	0.3995	0.4375	G	0.1946	0.8553
3	rs793440	99643176	A	0.1495	0.1242	G	0.1946	1.24
10	rs2231420	5567142	G	0.04381	0.03	C	0.1946	1.482
11	rs2074238	2484803	A	0.04381	0.03	G	0.1946	1.482
18	rs1145315	51688955	G	0.3995	0.4375	A	0.1946	0.8553
18	rs17069506	59821843	G	0.0799	0.06083	A	0.1946	1.341

4	rs7699006	89397091	A	0.183	0.215	G	0.1947	0.8178
11	rs200659526	65487793	G	0.09278	0.1183	A	0.1947	0.762
16	rs17855750	28515228	C	0.09278	0.1183	A	0.1947	0.762
17	rs9894429	79596811	G	0.3093	0.2742	A	0.1947	1.185
4	rs61753649	153874666	A	0.1108	0.1383	G	0.1948	0.7764
7	rs2303929	150761314	A	0.3093	0.3458	G	0.1948	0.847
1	rs11485177	222640209	A	0.3634	0.3275	C	0.1949	1.172
11	rs11025466	20301930	G	0.3634	0.3275	A	0.1949	1.172
17	rs58337165	1684605	A	0.1753	0.1467	C	0.1949	1.236
3	rs2244708	129291751	G	0.4639	0.425	A	0.195	1.171
6	rs7770592	29395747	A	0.2165	0.25	C	0.1952	0.8289
6	rs151719	32903900	G	0.2165	0.25	A	0.1952	0.8289
5	rs157496	32087374	G	0.4639	0.4258	A	0.1953	1.167
6	rs2116263	33025493	A	0.4639	0.4258	G	0.1953	1.167
7	rs6463841	8797321	A	0.4046	0.4425	G	0.1953	0.8563
8	rs1550210	85121515	G	0.4046	0.4425	A	0.1953	0.8563
18	rs11661542	20223695	A	0.4046	0.4425	C	0.1953	0.8563
3	rs2540078	3156811	G	0.4665	0.4282	A	0.1954	1.168
1	rs11360	156629037	G	0.4691	0.43	A	0.1955	1.171
1	rs79523790	203472112	G	0.201	0.1717	A	0.1955	1.214
2	rs17848880	73676008	G	0.04749	0.0325	A	0.1955	1.484
3	rs1131199	112059768	G	0.2165	0.2492	C	0.1955	0.8326
6	rs9491140	124691237	A	0.2165	0.2492	G	0.1955	0.8326
8	rs1209879	146076708	A	0.2165	0.2492	G	0.1955	0.8326
12	rs7977734	58301062	G	0.07732	0.1008	A	0.1955	0.7473
12	rs1290030	76212584	A	0.4691	0.43	G	0.1955	1.171
16	rs61747738	2854494	G	0.07732	0.1008	A	0.1955	0.7473
1	rs117454252	43917083	A	0.01546	0.02917	G	0.1956	0.5228
1	rs35687416	47834209	A	0.07732	0.1	C	0.1956	0.7542
1	rs10911390	183616884	A	0.01546	0.02917	G	0.1956	0.5228
1	rs10911391	183617094	A	0.01546	0.02917	G	0.1956	0.5228
1	rs10911392	183617311	T	0.01546	0.02917	A	0.1956	0.5228
1	rs16861394	183617693	A	0.01546	0.02917	G	0.1956	0.5228
1	rs1418150	223438771	A	0.07732	0.1	G	0.1956	0.7542
2	rs6740034	182036900	A	0.3119	0.3483	G	0.1956	0.8478
6	rs150850606	27925076	G	0.01546	0.02917	C	0.1956	0.5228
9	rs17613	139840560	A	0.01546	0.02917	C	0.1956	0.5228
10	rs7919006	76808760	A	0.366	0.33	G	0.1956	1.172
11	rs7950811	92651002	A	0.01546	0.02917	C	0.1956	0.5228
13	rs9548509	39430314	G	0.01546	0.02917	A	0.1956	0.5228
15	rs2622530	100572954	G	0.01546	0.02917	A	0.1956	0.5228
16	rs139279676	50324561	A	0.01546	0.02917	G	0.1956	0.5228
16	rs11640912	77359919	A	0.07732	0.1	T	0.1956	0.7542

19	rs440677	11285390	A	0.3119	0.3483	G	0.1956	0.8478
1	rs7535396	97016984	A	0.2577	0.2925	G	0.1957	0.8399
11	rs1466492	109199040	A	0.3686	0.3317	G	0.1957	1.176
15	rs1614300	42271067	C	0.4072	0.445	A	0.1957	0.8568
22	rs183455182	38130827	A	0.02083	0.01083	T	0.1957	1.943
13	rs9538523	34318605	G	0.4716	0.4325	A	0.1958	1.171
20	rs6016373	39154095	A	0.4691	0.4308	G	0.1958	1.167
1	rs7525142	240742905	A	0.4124	0.4508	C	0.1959	0.8548
2	rs2444263	152311570	G	0.232	0.2008	A	0.1959	1.202
2	rs2123465	152320118	G	0.232	0.2008	A	0.1959	1.202
2	rs1065177	152331418	C	0.232	0.2008	G	0.1959	1.202
2	rs722943	163892135	A	0.4124	0.4508	G	0.1959	0.8548
3	rs832038	97741821	G	0.4124	0.4508	A	0.1959	0.8548
1	rs16855164	180010911	G	0.268	0.235	A	0.196	1.192
3	rs7626857	33926125	G	0.4716	0.4333	A	0.196	1.167
11	rs643423	119043656	G	0.1521	0.1258	A	0.196	1.246
21	rs2277800	43824136	A	0.1521	0.1258	G	0.196	1.246
22	rs136176	36661646	G	0.1521	0.1258	A	0.196	1.246
2	rs7567398	21685076	G	0.4149	0.4533	A	0.1962	0.8553
18	rs1243039	61498396	G	0.4149	0.4533	A	0.1962	0.8553
3	rs4511910	58590284	A	0.4691	0.4316	C	0.1963	1.164
3	rs651907	101600724	A	0.4175	0.4567	G	0.1963	0.8529
5	rs6865063	28635825	G	0.4124	0.45	A	0.1964	0.8577
6	rs3129029	29662443	C	0.1572	0.1875	A	0.1964	0.8084
10	rs10857472	50534599	A	0.1572	0.1875	G	0.1964	0.8084
11	rs2165832	60107105	A	0.4124	0.45	G	0.1964	0.8577
5	rs10065575	86714840	A	0.4175	0.4558	G	0.1965	0.8557
7	rs1859041	25687828	G	0.3711	0.3342	A	0.1965	1.176
11	rs11024739	18645843	A	0.4201	0.4592	C	0.1965	0.8533
14	rs7160912	71236728	A	0.4201	0.4592	C	0.1965	0.8533
17	rs883541	66449122	G	0.4175	0.4558	A	0.1965	0.8557
18	rs3745060	55021725	A	0.4201	0.4592	C	0.1965	0.8533
6	rs4639381	30766765	G	0.4227	0.4615	C	0.1966	0.8542
19	rs12609004	55568085	A	0.1572	0.1867	G	0.1966	0.8128
9	rs1058446	100823135	C	0.1856	0.2175	G	0.1967	0.8197
10	rs11200524	124036328	A	0.1856	0.2175	G	0.1967	0.8197
14	rs8192917	25102160	G	0.1856	0.2175	A	0.1967	0.8197
17	rs11657804	67210992	G	0.1856	0.2175	A	0.1967	0.8197
2	rs6749689	20396122	G	0.4227	0.4617	A	0.1968	0.8537
9	rs10519	101831995	A	0.2191	0.2525	G	0.1968	0.8305
2	rs79465176	86364629	C	0.02062	0.01083	A	0.1969	1.922
2	rs12621278	173311553	G	0.2191	0.2533	A	0.1969	0.8268
3	rs9853551	149889584	G	0.482	0.4433	A	0.1969	1.168

4	rs192415191	1369892	A	0.02062	0.01083	G	0.1969	1.922
4	rs141239137	74310774	C	0.02062	0.01083	G	0.1969	1.922
5	rs151145750	13719123	A	0.02062	0.01083	G	0.1969	1.922
5	rs118026202	13911508	A	0.02062	0.01083	G	0.1969	1.922
5	rs116995413	13919366	G	0.02062	0.01083	C	0.1969	1.922
6	rs3129809	30335621	G	0.02062	0.01083	A	0.1969	1.922
6	rs3132625	30347720	G	0.02062	0.01083	A	0.1969	1.922
6	rs3130116	30365448	G	0.02062	0.01083	A	0.1969	1.922
8	rs1805794	90990479	C	0.482	0.4433	G	0.1969	1.168
8	rs75858201	103308010	G	0.02062	0.01083	A	0.1969	1.922
10	rs2804054	10160039	A	0.2603	0.295	G	0.1969	0.841
10	rs10748579	94090498	A	0.482	0.4433	G	0.1969	1.168
11	rs78209054	34474721	A	0.02062	0.01083	G	0.1969	1.922
12	rs12230341	15689951	G	0.2603	0.295	A	0.1969	0.841
12	rs141402989	56628681	A	0.02062	0.01083	G	0.1969	1.922
12	rs1949536	89490667	A	0.482	0.4433	G	0.1969	1.168
12	rs11065772	109617865	A	0.2603	0.295	G	0.1969	0.841
15	rs146535143	42128423	A	0.02062	0.01083	G	0.1969	1.922
17	rs116015543	9631994	G	0.02062	0.01083	A	0.1969	1.922
17	rs2302669	65214779	G	0.02062	0.01083	A	0.1969	1.922
18	rs200885371	76753882	A	0.02062	0.01083	G	0.1969	1.922
19	rs149921319	44097343	A	0.02062	0.01083	G	0.1969	1.922
20	rs149862820	45021740	G	0.02062	0.01083	A	0.1969	1.922
1	rs1128400	29542637	A	0.4768	0.4392	G	0.197	1.164
1	rs1333727	97681342	A	0.4227	0.4608	G	0.197	0.8566
7	rs2371208	82337859	A	0.4253	0.4642	C	0.197	0.8542
10	rs2293277	95279506	T	0.4768	0.4392	A	0.197	1.164
10	rs845085	125217896	A	0.4227	0.4608	G	0.197	0.8566
12	rs1678560	25666033	A	0.317	0.3539	G	0.197	0.8473
17	rs8073039	57521208	C	0.3144	0.2792	A	0.197	1.184
17	rs1558878	66364804	G	0.2345	0.2025	A	0.197	1.207
1	rs17723260	155058663	A	0.02577	0.01583	G	0.1972	1.644
2	rs4461296	50100835	G	0.4794	0.4417	A	0.1972	1.164
2	rs1046976	234255548	G	0.02577	0.01583	A	0.1972	1.644
3	rs55898544	10242070	G	0.02577	0.01583	A	0.1972	1.644
4	rs13305979	2993963	G	0.02577	0.01583	C	0.1972	1.644
6	rs11965214	30647005	C	0.02577	0.01583	G	0.1972	1.644
6	rs76636517	46659440	A	0.02577	0.01583	C	0.1972	1.644
6	rs1327283	51248309	A	0.4278	0.4667	G	0.1972	0.8546
6	rs79728438	56362247	A	0.02577	0.01583	G	0.1972	1.644
6	rs150766204	131894479	A	0.02577	0.01583	G	0.1972	1.644
7	rs1922084	156042560	A	0.02577	0.01583	G	0.1972	1.644
10	rs200816990	15617571	A	0.02577	0.01583	C	0.1972	1.644

15	rs62001448	41865525	A	0.02577	0.01583	C	0.1972	1.644
19	rs76629753	17662616	A	0.02577	0.01583	G	0.1972	1.644
19	rs10409124	47226434	A	0.02577	0.01583	G	0.1972	1.644
6	rs9267536	31651194	C	0.03093	0.0475	A	0.1973	0.64
5	rs2914908	117982046	G	0.4304	0.4692	A	0.1974	0.855
9	rs10818918	126860510	A	0.4304	0.4692	G	0.1974	0.855
14	rs2273171	31381351	G	0.4304	0.4692	A	0.1974	0.855
3	rs4432626	1791236	A	0.4278	0.4658	G	0.1975	0.8574
5	rs1651478	3606284	A	0.4278	0.4658	G	0.1975	0.8574
11	rs6357	2188238	A	0.03093	0.04667	G	0.1975	0.652
14	rs17122388	50789476	A	0.03093	0.04667	G	0.1975	0.652
16	rs2071915	426432	G	0.3212	0.3581	A	0.1975	0.8484
17	rs226441	34033072	A	0.4845	0.4467	G	0.1975	1.164
17	rs8065903	48629458	A	0.03093	0.04667	G	0.1975	0.652
1	rs4649175	24406647	A	0.4923	0.4533	G	0.1976	1.169
1	rs4320729	24409191	A	0.4923	0.4533	G	0.1976	1.169
1	rs6427850	200672410	A	0.5052	0.4667	C	0.1976	1.167
3	rs978979	56533016	G	0.134	0.1617	A	0.1977	0.8025
7	rs1800000	113518498	A	0.134	0.1617	C	0.1977	0.8025
5	rs3756669	35965970	C	0.3737	0.3375	A	0.1978	1.171
5	rs9686661	55861786	A	0.09536	0.075	G	0.1978	1.3
5	rs11167756	141059868	G	0.4304	0.4683	A	0.1978	0.8578
6	rs610604	138199417	C	0.09536	0.075	A	0.1978	1.3
12	rs10770658	20586821	G	0.4974	0.4583	A	0.1978	1.17
20	rs17725255	18079131	G	0.1778	0.1492	A	0.1978	1.234
2	rs1919128	27801759	A	0.4923	0.4542	G	0.1979	1.165
2	rs13022873	27815510	A	0.4923	0.4542	C	0.1979	1.165
5	rs17132261	110008214	A	0.2629	0.2983	G	0.1979	0.8388
6	rs4323302	21081206	G	0.4923	0.4542	A	0.1979	1.165
6	rs1042044	39041502	A	0.4923	0.4542	C	0.1979	1.165
12	rs7299095	118896304	A	0.4974	0.4583	G	0.1979	1.17
12	rs1183910	121420807	A	0.4974	0.4583	G	0.1979	1.17
16	rs60438349	10434389	G	0.2642	0.2992	A	0.1979	0.8414
4	rs1880024	8454639	A	0.4974	0.4591	G	0.198	1.166
6	rs77152992	21065449	A	0.1134	0.14	G	0.198	0.7857
6	rs9262628	31023148	A	0.5	0.4608	G	0.198	1.17
6	rs2517523	31026434	G	0.5	0.4608	A	0.198	1.17
6	rs2517516	31028920	G	0.5	0.4608	A	0.198	1.17
6	rs2844641	31037794	G	0.5	0.4608	A	0.198	1.17
6	rs2523888	31040720	C	0.5	0.4608	A	0.198	1.17
6	rs2249231	31043232	A	0.5	0.4608	G	0.198	1.17
8	rs4921637	19218766	G	0.1134	0.14	A	0.198	0.7857
11	rs3802813	126162468	A	0.1134	0.14	G	0.198	0.7857

16	rs8050354	53649168	G	0.5	0.4608	A	0.198	1.17
11	rs2272094	122738261	G	0.4948	0.4567	A	0.1981	1.165
12	rs1169076	122429484	A	0.4948	0.4567	G	0.1981	1.165
16	rs1801257	57689385	C	0.1521	0.1267	G	0.1981	1.236
19	rs2288523	55702910	A	0.2629	0.2975	C	0.1981	0.8422
2	rs11124754	40876113	G	0.4433	0.4825	A	0.1982	0.8541
3	rs10433406	124192674	C	0.3196	0.3558	A	0.1982	0.8503
5	rs2548612	53815495	C	0.4356	0.4733	A	0.1982	0.8586
8	rs12546751	57752823	G	0.4974	0.4592	A	0.1982	1.166
19	rs731839	33899065	A	0.4356	0.4733	G	0.1982	0.8586
20	rs2423846	14901776	G	0.4433	0.4825	A	0.1982	0.8541
6	rs6941772	31048916	G	0.5	0.4617	C	0.1983	1.166
6	rs9263473	31050179	G	0.5	0.4617	A	0.1983	1.166
6	rs2517471	31052098	G	0.5	0.4617	C	0.1983	1.166
12	rs1169081	122405912	A	0.5	0.4617	C	0.1983	1.166
17	rs9895895	71503636	G	0.5	0.4617	A	0.1983	1.166
9	rs10746620	82812849	A	0.4459	0.4841	G	0.1984	0.8574
10	rs8354	104436641	A	0.5026	0.4642	G	0.1984	1.166
11	rs10791961	56143434	G	0.1134	0.1408	C	0.1984	0.7803
1	rs4233414	167021041	G	0.4459	0.4842	A	0.1985	0.8573
2	rs441584	76240260	G	0.4459	0.4842	A	0.1985	0.8573
4	rs1109501	71329490	A	0.4459	0.4842	G	0.1985	0.8573
5	rs1129770	79086883	A	0.06443	0.08583	G	0.1985	0.7335
6	rs194609	82467107	A	0.5077	0.4692	G	0.1985	1.167
8	rs11775334	10071620	A	0.451	0.49	G	0.1985	0.8551
10	rs3740447	72307101	A	0.4459	0.4842	G	0.1985	0.8573
11	rs4638289	18285774	A	0.2216	0.2558	T	0.1985	0.8283
1	rs148707018	228033667	G	0.01031	0.02167	C	0.1986	0.4704
3	rs10866033	60280743	G	0.01031	0.02167	A	0.1986	0.4704
6	rs147166240	35430691	A	0.01031	0.02167	G	0.1986	0.4704
7	rs148703211	7491996	C	0.01031	0.02167	G	0.1986	0.4704
8	rs77359862	145154282	A	0.01031	0.02167	G	0.1986	0.4704
9	rs1984216	8230433	A	0.5103	0.4717	G	0.1986	1.167
11	rs140514299	62293913	C	0.01031	0.02167	A	0.1986	0.4704
12	rs117936881	10978607	G	0.01031	0.02167	A	0.1986	0.4704
14	rs149669464	51219249	A	0.01031	0.02167	G	0.1986	0.4704
16	rs150670945	1536149	A	0.01031	0.02167	G	0.1986	0.4704
16	rs1345889	71361886	C	0.4485	0.4867	A	0.1986	0.8576
19	rs74491133	11258570	A	0.01031	0.02167	G	0.1986	0.4704
20	rs80113248	18142462	G	0.01031	0.02167	A	0.1986	0.4704
22	rs2281192	46719120	A	0.01031	0.02167	G	0.1986	0.4704
22	rs130129	49096694	G	0.4536	0.4925	A	0.1986	0.8555
1	rs2479409	55504650	A	0.3222	0.3592	G	0.1987	0.848

2	rs4369034	140494388	G	0.3222	0.3592	A	0.1987	0.848
18	rs1970671	52863108	G	0.4407	0.4783	C	0.1987	0.8594
3	rs13317287	21399012	A	0.317	0.2825	G	0.1988	1.179
6	rs243125	48631508	A	0.5284	0.4892	G	0.1988	1.17
10	rs4747755	31888431	A	0.5232	0.4842	G	0.1988	1.169
18	rs12607385	47777937	A	0.4588	0.4975	G	0.1988	0.8561
2	rs2161036	186659359	C	0.4639	0.5025	A	0.1989	0.8568
3	rs1458007	88484631	A	0.4665	0.505	G	0.1989	0.8571
6	rs2517455	31059900	G	0.5	0.4625	A	0.1989	1.162
6	rs2517453	31060115	A	0.5	0.4625	G	0.1989	1.162
6	rs1064190	31075115	A	0.5	0.4625	C	0.1989	1.162
6	rs1265063	31077938	G	0.5	0.4625	A	0.1989	1.162
6	rs1265054	31079643	G	0.5	0.4625	A	0.1989	1.162
6	rs2233966	31080859	G	0.5026	0.465	A	0.1989	1.162
9	rs3808869	34622389	A	0.5	0.4625	C	0.1989	1.162
9	rs10817896	119232655	A	0.4639	0.5025	G	0.1989	0.8568
10	rs6480305	69879709	G	0.5026	0.465	A	0.1989	1.162
11	rs12808199	39327959	A	0.4665	0.505	G	0.1989	0.8571
15	rs7164258	81661346	A	0.4665	0.505	G	0.1989	0.8571
1	rs2012235	68912755	A	0.5284	0.49	G	0.199	1.166
1	rs2274064	183542387	A	0.3222	0.3583	G	0.199	0.8511
2	rs6740609	79855622	G	0.3222	0.3583	A	0.199	0.8511
4	rs7671167	89883979	G	0.5077	0.47	A	0.199	1.163
6	rs1233399	29539482	A	0.2371	0.205	G	0.199	1.205
6	rs10945919	164186677	G	0.3222	0.3583	A	0.199	0.8511
7	rs4130913	84256576	G	0.3222	0.3583	A	0.199	0.8511
7	rs259139	89623846	A	0.2371	0.205	C	0.199	1.205
7	rs10488172	133335176	C	0.5077	0.47	A	0.199	1.163
12	rs7969300	111993712	G	0.5077	0.47	A	0.199	1.163
18	rs931982	45110833	A	0.2732	0.24	G	0.199	1.19
6	rs1634731	30955681	G	0.09536	0.1208	A	0.1991	0.767
6	rs4715631	56417545	A	0.2655	0.3008	G	0.1991	0.8399
7	rs72601626	47917087	A	0.06443	0.08667	G	0.1991	0.7258
8	rs1708789	29247126	A	0.5206	0.4825	G	0.1991	1.165
14	rs8009041	37190198	G	0.5232	0.485	A	0.1991	1.165
19	rs2188736	58213773	C	0.09536	0.1208	G	0.1991	0.767
6	rs2286656	30899571	A	0.3789	0.3425	G	0.1992	1.171
1	rs2232041	111494194	A	0.0567	0.04	G	0.1993	1.443
2	rs17229201	186656956	A	0.4639	0.5017	G	0.1993	0.8596
2	rs7605884	186667121	A	0.4639	0.5017	G	0.1993	0.8596
2	rs16827154	186670780	T	0.4639	0.5017	A	0.1993	0.8596
4	rs2271777	139964455	A	0.08247	0.0625	G	0.1994	1.348
4	rs17291045	161506897	A	0.08247	0.0625	G	0.1994	1.348

7	rs4732385	139185449	A	0.4665	0.5042	C	0.1994	0.8599
18	rs11081859	31926289	G	0.2655	0.3	A	0.1994	0.8433
11	rs12574749	32405355	C	0.3814	0.3442	A	0.1995	1.175
1	rs4074436	154531910	G	0.1546	0.1283	A	0.1996	1.242
1	rs4651156	183837032	A	0.1546	0.1283	G	0.1996	1.242
1	rs10917459	19806692	C	0.2371	0.2058	A	0.1998	1.199
1	rs4603	151374025	G	0.3247	0.3608	A	0.1998	0.8519
11	rs3206824	11986061	A	0.2371	0.2058	G	0.1998	1.199
12	rs2071024	56351128	A	0.0232	0.0375	C	0.1999	0.6095
15	rs12898794	74884447	A	0.0232	0.0375	G	0.1999	0.6095
19	rs16975963	38325536	G	0.2732	0.2408	C	0.2001	1.185
3	rs59021909	130285929	A	0.134	0.1092	G	0.2002	1.263
4	rs11941589	140115744	G	0.384	0.3467	A	0.2002	1.175
9	rs13295305	91978397	A	0.1546	0.1288	G	0.2003	1.238
18	rs12963653	23872235	G	0.04124	0.05917	A	0.2003	0.6839
6	rs3734312	76660397	G	0.2758	0.2425	A	0.2005	1.189
5	rs79464236	90087161	G	0.0232	0.03833	C	0.2006	0.5957
5	rs80145956	162945269	A	0.0232	0.0384	G	0.2006	0.5947
19	rs146799017	14749131	A	0.0232	0.03833	G	0.2006	0.5957
2	rs415793	160182302	A	0.1366	0.1642	G	0.2007	0.8055
5	rs11957313	169950394	A	0.1907	0.2225	G	0.2007	0.8235
7	rs6461515	20778646	G	0.1366	0.1642	A	0.2007	0.8055
1	rs2271885	110921715	C	0.1804	0.1517	A	0.2008	1.231
2	rs4676049	109635257	A	0.0799	0.1033	G	0.2008	0.7535
11	rs34050666	73814438	C	0.0799	0.1033	G	0.2008	0.7535
1	rs267738	150940625	C	0.09794	0.07667	A	0.2009	1.308
1	rs267734	150951477	G	0.09794	0.07667	A	0.2009	1.308
2	rs4416248	22455521	G	0.09794	0.07667	A	0.2009	1.308
12	rs1800194	57567762	A	0.3299	0.3667	G	0.2009	0.8503
13	rs117984168	103389158	A	0.09794	0.07667	T	0.2009	1.308
1	rs12411176	110766466	A	0.0799	0.1025	G	0.201	0.7603
2	rs825287	222524920	G	0.3222	0.2875	A	0.201	1.178
9	rs2842483	78906150	G	0.0799	0.1025	A	0.201	0.7603
9	rs2842480	78910241	C	0.0799	0.1025	A	0.201	0.7603
9	rs2643325	78910273	A	0.0799	0.1025	G	0.201	0.7603
11	rs3814747	65788072	G	0.0799	0.1025	A	0.201	0.7603
13	rs9528116	60932642	A	0.3222	0.2875	G	0.201	1.178
16	rs11150610	31334236	C	0.1907	0.2217	A	0.201	0.8275
1	rs2294514	54432019	A	0.04124	0.06	G	0.2011	0.6738
22	rs131794	50971752	A	0.04124	0.06	C	0.2011	0.6738
2	rs146258586	131520942	A	0.03608	0.02333	G	0.2013	1.567
2	rs12470505	219908369	C	0.1624	0.1925	A	0.2013	0.8131
3	rs3772219	56771251	C	0.3866	0.35	A	0.2013	1.17

6	rs2229699	31548856	A	0.03608	0.02333	G	0.2013	1.567
6	rs113689068	47681711	A	0.03608	0.02333	C	0.2013	1.567
6	rs4870093	152652184	A	0.03608	0.02333	G	0.2013	1.567
7	rs10270308	105671267	G	0.3866	0.35	A	0.2013	1.17
14	rs941920	91739081	A	0.3866	0.35	G	0.2013	1.17
22	rs11913840	18912677	A	0.03608	0.02333	G	0.2013	1.567
6	rs362949	146652354	G	0.1546	0.1292	A	0.2015	1.233
8	rs4738101	71676339	A	0.2268	0.2608	G	0.2015	0.8313
18	rs2282542	13056682	A	0.2268	0.26	G	0.2015	0.8349
18	rs7228940	13068109	A	0.2268	0.26	G	0.2015	0.8349
6	rs408359	32141883	A	0.2088	0.1783	G	0.2016	1.216
7	rs58295063	151240486	G	0.2088	0.1783	A	0.2016	1.216
22	rs6000174	36587279	A	0.1624	0.1917	G	0.2016	0.8175
22	rs2227167	36587486	A	0.1624	0.1917	G	0.2016	0.8175
1	rs2239560	9304731	A	0.116	0.0925	G	0.2017	1.287
11	rs7126805	828916	G	0.2706	0.305	A	0.2018	0.8454
12	rs1551120	80614851	G	0.2706	0.305	A	0.2018	0.8454
22	rs2018393	40803186	A	0.3892	0.3525	C	0.2019	1.17
18	rs2249769	32834186	C	0.2784	0.245	A	0.202	1.189
12	rs79897879	52685213	C	0.134	0.11	A	0.2022	1.252
2	rs992159	206391545	A	0.08247	0.06333	C	0.2023	1.329
5	rs11952064	173637603	A	0.3351	0.3725	C	0.2023	0.8488
2	exm-IND2-70914	71060822	D	0.02835	0.01667	I	0.2026	1.721
2	rs79378995	103090268	G	0.02835	0.01667	A	0.2026	1.721
2	rs144957722	122125373	A	0.02835	0.01667	C	0.2026	1.721
3	rs73202271	129130018	A	0.02835	0.01667	G	0.2026	1.721
6	rs2517896	29800142	A	0.02835	0.01667	G	0.2026	1.721
6	rs2735018	29804409	C	0.02835	0.01667	G	0.2026	1.721
8	rs1600249	11359638	C	0.3273	0.2917	A	0.2026	1.182
12	rs34778348	40757328	A	0.02835	0.01667	G	0.2026	1.721
12	rs1569019	131576191	A	0.02835	0.01667	C	0.2026	1.721
19	rs72558007	7762440	A	0.02835	0.01667	G	0.2026	1.721
21	rs189737519	43833520	A	0.02835	0.01667	C	0.2026	1.721
10	rs2768713	14813860	G	0.134	0.1102	A	0.2027	1.25
6	rs9261535	30127323	A	0.2397	0.2092	G	0.2029	1.192
10	rs2274223	96066341	G	0.2294	0.2625	A	0.2031	0.8363
22	rs12484684	17589794	A	0.3943	0.3575	C	0.2032	1.17
5	rs692843	176255646	G	0.09794	0.1225	A	0.2033	0.7777
4	rs12513290	141317068	A	0.2809	0.2475	G	0.2034	1.188
5	rs29674	163260	A	0.1166	0.0938	C	0.2034	1.275
6	rs184204164	63990101	A	0.0567	0.04083	G	0.2034	1.412
19	rs56194853	8154984	A	0.1804	0.1533	G	0.2035	1.215
1	rs12407896	57933955	A	0.116	0.09333	G	0.2036	1.274

5	rs889014	172984114	A	0.116	0.09333	G	0.2036	1.274
6	rs4947339	28916252	G	0.3402	0.3775	A	0.2036	0.8503
19	rs3815905	52857623	A	0.06959	0.05167	G	0.2036	1.373
12	rs216013	2729632	G	0.2758	0.3117	A	0.2037	0.841
12	rs2730829	41831174	A	0.2758	0.3108	G	0.2037	0.8443
15	rs700519	51507968	A	0.1392	0.1683	G	0.2037	0.7988
12	rs7314355	60493222	C	0.3969	0.36	A	0.2038	1.17
14	rs709932	94849201	A	0.1649	0.195	G	0.2038	0.8154
21	rs11909074	35820324	A	0.3943	0.3583	G	0.2038	1.166
6	rs62399430	30993866	T	0.09794	0.0775	A	0.2039	1.292
6	rs61731441	88125542	A	0.09794	0.0775	G	0.2039	1.292
2	rs3769969	162091932	A	0.04639	0.03167	G	0.204	1.488
6	rs2301226	33034596	A	0.3273	0.2933	G	0.204	1.172
9	rs7027110	109599046	A	0.2088	0.18	G	0.204	1.202
9	rs16916091	114184259	C	0.04639	0.03167	G	0.204	1.488
3	rs937878	96472739	G	0.3299	0.295	A	0.2041	1.177
3	rs2291498	56658871	G	0.3995	0.3623	A	0.2043	1.171
1	rs11161436	85206477	A	0.232	0.2658	G	0.2045	0.8341
4	rs9996636	170628524	G	0.1959	0.2279	A	0.2045	0.8253
5	rs4700448	61161048	A	0.3428	0.3792	G	0.2045	0.854
12	rs150694299	22208133	A	0	0.005882	T	0.2045	0
17	rs9902398	1686410	A	0.1959	0.2279	G	0.2045	0.8253
3	rs1497312	130125116	G	0.183	0.155	C	0.2046	1.221
6	rs2844677	30955359	A	0.183	0.155	G	0.2046	1.221
16	rs12443741	59475044	A	0.1959	0.2275	G	0.2046	0.8271
17	rs7946	17409560	A	0.232	0.265	G	0.2046	0.8377
3	rs111934125	56650054	G	0.3995	0.3631	A	0.2047	1.167
4	rs10520163	170626552	A	0.1959	0.2283	G	0.2047	0.8232
19	rs3764537	52619065	G	0.1959	0.2283	A	0.2047	0.8232
5	rs6882076	156390297	A	0.2448	0.2129	G	0.2048	1.199
11	rs35968518	107299623	C	0.2835	0.25	A	0.2048	1.187
13	rs7318731	22709670	A	0.2835	0.25	C	0.2048	1.187
11	rs200271333	77386245	A	0	0.005853	G	0.2049	0
2	rs12622896	138030234	A	0.06701	0.08833	G	0.205	0.7413
4	rs4521339	25915957	A	0.06701	0.08833	G	0.205	0.7413
6	rs3129996	30651587	A	0	0.005843	C	0.205	0
8	rs149225584	30537086	A	0	0.005843	G	0.205	0
12	rs645510	118106433	A	0.4021	0.365	G	0.205	1.17
19	rs5491	10385540	T	0.06701	0.08833	A	0.205	0.7413
20	rs199671123	25457541	A	0	0.005843	G	0.205	0
1	rs201876924	16373080	A	0	0.005833	C	0.2051	0
1	rs6087	43803863	A	0	0.005833	G	0.2051	0
1	rs70937045	44084819	A	0	0.005833	G	0.2051	0

1	rs17567	51826921	G	0	0.005833	A	0.2051	0
1	rs111296687	146737700	C	0	0.005833	A	0.2051	0
1	rs141970914	154247435	A	0	0.005833	G	0.2051	0
1	rs201477597	214820634	A	0	0.005833	G	0.2051	0
1	rs142356826	225332296	G	0	0.005833	C	0.2051	0
1	rs28603121	228461615	A	0	0.005833	G	0.2051	0
1	rs202098111	228602665	A	0	0.005833	G	0.2051	0
1	rs11537737	245018909	A	0	0.005833	G	0.2051	0
1	rs200231160	248004745	C	0	0.005833	G	0.2051	0
2	rs149894156	1241712	A	0	0.005833	G	0.2051	0
2	rs202110907	73718004	A	0	0.005833	G	0.2051	0
2	rs117622212	74746771	G	0	0.005833	A	0.2051	0
2	rs201550126	210887751	G	0	0.005833	A	0.2051	0
2	rs201415961	219221890	A	0	0.005833	T	0.2051	0
3	rs11711229	2954866	A	0.3325	0.2975	C	0.2051	1.176
3	rs200900937	40192643	A	0	0.005833	G	0.2051	0
3	rs199591994	50211665	A	0	0.005833	C	0.2051	0
3	rs138230109	50324215	A	0	0.005833	G	0.2051	0
3	rs201680751	111923150	G	0	0.005833	A	0.2051	0
3	rs56195264	119203154	A	0	0.005833	G	0.2051	0
3	rs11539377	119219573	G	0	0.005833	A	0.2051	0
3	rs57168946	119236104	A	0	0.005833	G	0.2051	0
3	rs116186804	121410365	G	0	0.005833	A	0.2051	0
3	rs201254023	186024765	G	0	0.005833	A	0.2051	0
4	rs191105001	79428614	G	0	0.005833	A	0.2051	0
4	rs114769117	104065609	G	0	0.005833	A	0.2051	0
4	rs74991481	123868545	A	0	0.005833	C	0.2051	0
4	rs2567241	141323162	A	0	0.005833	C	0.2051	0
4	rs185641600	144618119	A	0	0.005833	G	0.2051	0
4	rs114112493	178274840	A	0	0.005833	G	0.2051	0
4	rs188825200	187521158	A	0	0.005833	G	0.2051	0
5	rs142797820	133702147	G	0	0.005833	A	0.2051	0
5	rs142480526	140966709	A	0	0.005833	G	0.2051	0
5	rs186029710	148406139	A	0	0.005833	G	0.2051	0
5	rs72805941	149784658	G	0	0.005833	A	0.2051	0
6	rs145084016	21000533	A	0	0.005833	G	0.2051	0
6	rs145685809	30671206	G	0	0.005833	A	0.2051	0
6	rs6916357	30833826	A	0	0.005833	G	0.2051	0
6	rs138097862	30916645	A	0	0.005833	G	0.2051	0
6	rs115600609	30997667	G	0	0.005833	A	0.2051	0
6	rs2516312	31539435	G	0	0.005833	A	0.2051	0
6	rs28400004	31734385	A	0	0.005833	G	0.2051	0
6	rs7769537	31741981	A	0	0.005833	G	0.2051	0

6	rs151103262	36993613	A	0	0.005833	G	0.2051	0
6	rs200657227	41062230	C	0	0.005833	A	0.2051	0
6	rs140000057	110763731	A	0	0.005833	G	0.2051	0
6	rs200639804	132938401	G	0	0.005833	A	0.2051	0
7	rs148913962	45148814	A	0	0.005833	G	0.2051	0
7	rs148629439	48335451	G	0	0.005833	A	0.2051	0
7	rs201001953	77261719	A	0	0.005833	G	0.2051	0
7	rs199577930	91739445	A	0	0.005833	G	0.2051	0
7	rs150466149	99017660	A	0	0.005833	G	0.2051	0
7	rs201844799	107430114	A	0	0.005833	G	0.2051	0
7	rs58369703	149519649	G	0	0.005833	C	0.2051	0
8	rs191182708	1993158	A	0	0.005833	G	0.2051	0
8	rs61733705	17510766	G	0	0.005833	C	0.2051	0
8	rs61748836	17541943	C	0	0.005833	A	0.2051	0
8	rs200784274	24197017	A	0	0.005833	G	0.2051	0
8	rs151044076	48883929	A	0	0.005833	G	0.2051	0
8	rs78251408	54742024	G	0	0.005833	A	0.2051	0
8	rs193072874	81888847	A	0	0.005833	G	0.2051	0
8	rs7825375	125990008	A	0	0.005833	G	0.2051	0
8	rs115877910	133980143	G	0	0.005833	A	0.2051	0
9	rs74959855	95142121	A	0	0.005833	G	0.2051	0
9	rs145551982	131371201	A	0	0.005833	G	0.2051	0
10	rs199997598	7773942	A	0	0.005833	G	0.2051	0
10	rs61730102	27366407	A	0	0.005833	C	0.2051	0
10	rs182132442	96731876	A	0	0.005833	C	0.2051	0
10	rs186551854	121689160	G	0	0.005833	A	0.2051	0
10	rs185020377	134038000	A	0	0.005833	G	0.2051	0
11	rs140890951	5758012	G	0	0.005833	A	0.2051	0
11	rs7951904	6412931	G	0	0.005833	A	0.2051	0
11	rs139967004	9850942	T	0	0.005833	A	0.2051	0
11	rs139251036	66272198	A	0	0.005833	G	0.2051	0
11	rs143822485	89896199	A	0	0.005833	C	0.2051	0
11	rs7113004	90562153	G	0	0.005833	A	0.2051	0
11	rs55870064	108168053	G	0	0.005833	A	0.2051	0
11	rs117077516	130003541	A	0	0.005833	C	0.2051	0
12	rs2970818	4606168	A	0	0.005833	T	0.2051	0
12	rs11609202	14959470	G	0	0.005833	A	0.2051	0
12	rs147130460	25679137	G	0	0.005833	A	0.2051	0
12	rs199940769	48111969	A	0	0.005833	T	0.2051	0
12	rs142554052	105467708	A	0	0.005833	G	0.2051	0
13	rs199614400	38144735	A	0	0.005833	G	0.2051	0
14	rs116961275	33292345	A	0	0.005833	G	0.2051	0
14	rs148266372	36147197	G	0	0.005833	A	0.2051	0

15	rs25397	48936888	A	0	0.005833	G	0.2051	0
15	rs137967275	49033797	A	0	0.005833	C	0.2051	0
15	rs140688864	49284490	A	0	0.005833	G	0.2051	0
15	rs141749560	68606130	A	0	0.005833	G	0.2051	0
15	rs117208397	71329621	A	0	0.005833	G	0.2051	0
15	rs181620994	83790737	C	0	0.005833	G	0.2051	0
15	rs142254313	89424828	A	0	0.005833	G	0.2051	0
15	rs75536206	91489936	A	0	0.005833	G	0.2051	0
16	rs200569447	29830928	A	0	0.005833	G	0.2051	0
16	rs140017746	55907857	A	0	0.005833	G	0.2051	0
16	rs61746433	77245139	G	0	0.005833	A	0.2051	0
17	rs188772907	11672509	A	0	0.005833	G	0.2051	0
17	rs121912749	42337869	A	0	0.005833	G	0.2051	0
17	rs146371375	76120658	A	0	0.005833	G	0.2051	0
18	rs55674018	56191239	C	0	0.005833	G	0.2051	0
18	rs114579767	77679290	A	0	0.005833	G	0.2051	0
19	rs202078277	21300054	A	0	0.005833	G	0.2051	0
19	rs199577709	36357242	A	0	0.005833	C	0.2051	0
19	rs200281362	40889694	A	0	0.005833	G	0.2051	0
19	rs143978380	43702355	G	0	0.005833	C	0.2051	0
19	rs76914433	45381836	A	0	0.005833	C	0.2051	0
19	rs143724166	50967760	A	0	0.005833	G	0.2051	0
20	rs151164700	40049349	A	0	0.005833	G	0.2051	0
20	rs149450567	61542195	A	0	0.005833	G	0.2051	0
21	rs201854024	19726136	G	0	0.005833	A	0.2051	0
21	rs182565117	47660803	G	0	0.005833	A	0.2051	0
22	rs143972918	47095300	A	0	0.005833	G	0.2051	0
5	rs1501908	156398169	G	0.2448	0.2133	C	0.2053	1.196
1	rs11538340	161172233	A	0.06701	0.08917	C	0.2055	0.7337
5	rs7724813	180374523	A	0.06701	0.08917	G	0.2055	0.7337
6	rs4248148	30742134	A	0.1186	0.1458	G	0.2055	0.7878
6	rs9388766	130354855	A	0.1186	0.1458	G	0.2055	0.7878
1	rs16824588	1900232	G	0.4021	0.3658	A	0.2056	1.166
1	rs12126589	105447979	G	0.3479	0.385	A	0.2056	0.8524
1	rs7537938	173408212	G	0.3479	0.385	A	0.2056	0.8524
2	exm186378	36810586	G	0.4046	0.3675	A	0.2056	1.17
2	rs14291	36810586	G	0.4046	0.3675	A	0.2056	1.17
4	rs1587563	78734563	G	0.3479	0.385	A	0.2056	0.8524
11	rs505802	64357072	A	0.2448	0.2137	G	0.2056	1.193
17	rs8079495	70317988	G	0.4021	0.3658	A	0.2056	1.166
19	rs4804381	9056989	C	0.3479	0.385	G	0.2056	0.8524
19	rs7255165	20133846	A	0.05412	0.07346	G	0.2057	0.7218
5	rs12657663	134076812	A	0.3351	0.2997	G	0.2059	1.178

6	rs10499194	138002637	A	0.05412	0.07333	G	0.2059	0.7231
12	rs9262	88440676	C	0.2809	0.3158	G	0.2059	0.8463
5	rs401681	1322087	A	0.3325	0.2983	G	0.206	1.171
6	rs64036	29527267	A	0.1675	0.1975	C	0.2061	0.8177
6	rs6915183	166706169	A	0.08247	0.1058	G	0.2061	0.7594
6	rs630379	31922254	A	0.2835	0.2513	C	0.2062	1.179
8	rs822318	16437261	A	0.4072	0.37	G	0.2062	1.17
11	rs7128311	20562519	G	0.4072	0.37	A	0.2062	1.17
12	rs300489	78485994	A	0.1366	0.1125	G	0.2063	1.248
16	rs13336595	57996960	G	0.183	0.1558	A	0.2063	1.213
16	rs7194136	81180988	G	0.1366	0.1125	C	0.2063	1.248
6	rs2273017	32337630	G	0.3505	0.3867	A	0.2065	0.856
22	rs3747076	20759672	A	0.4072	0.3708	G	0.2067	1.166
1	rs6541085	10285709	G	0.4098	0.3725	A	0.2068	1.17
11	rs13817	65628334	A	0.2835	0.3192	G	0.2068	0.8441
15	rs4350528	94163700	A	0.2835	0.3183	G	0.2069	0.8473
7	rs10224537	21640405	A	0.2861	0.2533	G	0.207	1.181
1	rs201888830	906512	A	0.002577	0.01002	G	0.2071	0.2554
12	rs3741486	133384864	A	0.2474	0.2158	G	0.2071	1.194
13	rs75071921	42795514	G	0.002577	0.01002	A	0.2071	0.2554
9	rs7021524	10928314	G	0.4098	0.3733	A	0.2072	1.165
1	rs200182409	984947	A	0.002577	0.01	G	0.2073	0.2558
1	rs139744148	85035778	C	0.002577	0.01	A	0.2073	0.2558
1	rs141932545	153750665	A	0.002577	0.01	G	0.2073	0.2558
1	rs188107197	185969311	A	0.002577	0.01	T	0.2073	0.2558
2	rs142952986	228758555	A	0.002577	0.01	G	0.2073	0.2558
2	rs116238578	238283646	A	0.002577	0.01	G	0.2073	0.2558
3	rs201136314	13399853	A	0.002577	0.01	C	0.2073	0.2558
3	rs201026294	44776070	G	0.002577	0.01	A	0.2073	0.2558
3	rs138367039	132257069	A	0.002577	0.01	G	0.2073	0.2558
3	rs146981118	197597101	A	0.002577	0.01	T	0.2073	0.2558
3	rs114780577	197687252	C	0.002577	0.01	A	0.2073	0.2558
4	rs200587747	90035314	A	0.002577	0.01	G	0.2073	0.2558
5	rs200609148	44809338	G	0.002577	0.01	A	0.2073	0.2558
5	rs16878472	80409407	C	0.002577	0.01	A	0.2073	0.2558
5	rs118132132	96130769	C	0.002577	0.01	A	0.2073	0.2558
5	rs139484838	169028397	C	0.002577	0.01	A	0.2073	0.2558
6	rs201992278	42571338	A	0.002577	0.01	C	0.2073	0.2558
6	rs139339332	50807934	A	0.002577	0.01	G	0.2073	0.2558
6	rs139555370	51712738	A	0.002577	0.01	G	0.2073	0.2558
7	rs186526946	48318613	G	0.002577	0.01	A	0.2073	0.2558
7	rs142471703	100211255	A	0.002577	0.01	G	0.2073	0.2558
7	rs75646720	112415316	A	0.002577	0.01	G	0.2073	0.2558

7	rs148680931	143792742	G	0.002577	0.01	A	0.2073	0.2558
8	rs150427959	144991773	A	0.002577	0.01	G	0.2073	0.2558
9	rs41283630	101552584	A	0.002577	0.01	G	0.2073	0.2558
9	rs142193455	130488607	A	0.002577	0.01	G	0.2073	0.2558
10	rs2293239	50947852	G	0.002577	0.01	A	0.2073	0.2558
10	rs10884071	106731970	A	0.002577	0.01	G	0.2073	0.2558
11	rs148440178	121016781	A	0.002577	0.01	G	0.2073	0.2558
12	rs138151883	120569132	A	0.002577	0.01	G	0.2073	0.2558
13	rs354772	58158127	G	0.002577	0.01	A	0.2073	0.2558
13	rs117665810	109535415	C	0.002577	0.01	G	0.2073	0.2558
15	rs202012478	43818732	C	0.002577	0.01	G	0.2073	0.2558
16	rs117384853	2814074	A	0.002577	0.01	C	0.2073	0.2558
17	rs199632792	7681731	C	0.002577	0.01	A	0.2073	0.2558
17	rs147945685	26919336	A	0.002577	0.01	G	0.2073	0.2558
18	rs11661691	46770186	C	0.002577	0.01	A	0.2073	0.2558
18	rs9967417	46959500	C	0.002577	0.01	G	0.2073	0.2558
20	rs192330235	31023613	A	0.002577	0.01	T	0.2073	0.2558
21	rs8788	34897113	G	0.002577	0.01	A	0.2073	0.2558
21	rs75581470	47541500	A	0.002577	0.01	C	0.2073	0.2558
13	rs9529775	71136789	G	0.3557	0.3925	A	0.2074	0.8544
19	rs306507	56459342	G	0.1856	0.1575	A	0.2074	1.219
2	rs9013	143798189	G	0.2371	0.27	A	0.2076	0.8403
19	rs3746207	58233008	A	0.1005	0.125	G	0.2077	0.7822
19	rs2288904	10742170	A	0.2861	0.3217	G	0.2078	0.845
6	rs537160	31916400	A	0.3376	0.3033	G	0.2079	1.171
9	rs10739459	118869883	A	0.3402	0.305	G	0.2081	1.175
1	rs41309639	233122127	A	0.2474	0.2167	G	0.2082	1.189
6	rs2854028	33179689	A	0.2474	0.2167	G	0.2082	1.189
17	rs9911005	15412809	A	0.25	0.2179	G	0.2084	1.197
1	rs12569218	209034761	G	0.3608	0.3983	A	0.2085	0.8527
1	rs34001279	162367103	A	0.1186	0.09583	C	0.2086	1.269
12	rs3759301	32135441	A	0.1186	0.09583	G	0.2086	1.269
12	rs77028313	88547213	G	0.1186	0.09583	A	0.2086	1.269
20	rs237422	5753579	A	0.1701	0.2008	G	0.2086	0.8156
10	rs2068888	94839642	G	0.2397	0.2733	A	0.2088	0.8381
11	rs12793173	34834204	G	0.1211	0.1475	A	0.2088	0.7966
6	rs3132680	30073195	A	0.25	0.2183	C	0.2089	1.193
1	rs28548017	1887019	A	0.1856	0.1583	G	0.209	1.211
11	rs55975541	64597201	A	0.1856	0.1583	G	0.209	1.211
15	rs20543	101910550	G	0.2912	0.2575	A	0.209	1.185
6	rs366676	88717766	A	0.2397	0.2725	G	0.2091	0.8416
11	rs183698487	113860274	A	0.002632	0.01087	G	0.2091	0.2401
2	rs13021401	20688519	G	0.4201	0.3833	A	0.2092	1.165

3	rs151188219	66433793	A	0.002577	0.01083	G	0.2093	0.2359
3	rs184333551	142030465	A	0.002577	0.01083	G	0.2093	0.2359
5	rs199625658	108714557	A	0.002577	0.01083	G	0.2093	0.2359
5	rs143589506	126860562	A	0.002577	0.01083	G	0.2093	0.2359
6	rs144789373	33693264	A	0.002577	0.01083	G	0.2093	0.2359
7	rs114209249	48065498	A	0.002577	0.01083	G	0.2093	0.2359
7	rs146387132	82595219	A	0.002577	0.01083	G	0.2093	0.2359
7	rs146523163	151046195	C	0.002577	0.01083	A	0.2093	0.2359
10	rs79865214	94396989	C	0.002577	0.01083	G	0.2093	0.2359
10	rs118110471	121685689	A	0.002577	0.01083	G	0.2093	0.2359
11	rs114544023	5322249	G	0.002577	0.01083	C	0.2093	0.2359
11	rs7940646	10669228	A	0.002577	0.01083	G	0.2093	0.2359
11	rs2298447	65629960	A	0.002577	0.01083	G	0.2093	0.2359
11	rs34052882	108040484	A	0.002577	0.01083	C	0.2093	0.2359
11	rs61907183	123777502	A	0.002577	0.01083	G	0.2093	0.2359
12	rs139969601	6753525	A	0.002577	0.01083	G	0.2093	0.2359
12	rs145493619	42866206	A	0.002577	0.01083	G	0.2093	0.2359
12	rs199922925	130926892	C	0.002577	0.01083	G	0.2093	0.2359
13	rs190136021	42442510	A	0.002577	0.01083	G	0.2093	0.2359
13	rs148334149	99076833	A	0.002577	0.01083	G	0.2093	0.2359
15	rs141828250	43237642	A	0.002577	0.01083	G	0.2093	0.2359
15	rs150307169	69728061	A	0.002577	0.01083	G	0.2093	0.2359
17	rs138254713	48246530	A	0.002577	0.01083	G	0.2093	0.2359
18	rs142859678	61471522	A	0.002577	0.01083	G	0.2093	0.2359
18	rs148947974	61654210	A	0.002577	0.01083	G	0.2093	0.2359
20	rs201985967	34204198	A	0.002577	0.01083	T	0.2093	0.2359
2	rs6726919	23174479	A	0.4175	0.3817	G	0.2094	1.161
5	rs139145814	148727918	A	0.002577	0.01085	G	0.2094	0.2355
8	rs317952	85328171	C	0.4175	0.3817	A	0.2094	1.161
4	rs1789891	100250419	A	0.0567	0.04167	C	0.2096	1.383
5	rs3822530	75906923	A	0.0567	0.04167	C	0.2096	1.383
8	rs7460090	57194163	G	0.0567	0.04167	A	0.2096	1.383
19	exm-IND19-5799	53304469	D	0.0567	0.04167	I	0.2096	1.383
7	rs2240090	51096974	A	0.366	0.4025	G	0.2097	0.8569
10	rs7910550	86633900	G	0.2912	0.2583	A	0.2097	1.18
17	rs7216729	71970337	A	0.366	0.4025	C	0.2097	0.8569
5	rs61743146	109756436	A	0.03351	0.05	G	0.2098	0.6587
12	rs117088942	56755652	A	0.03351	0.05	G	0.2098	0.6587
19	rs41275782	51172526	G	0.08505	0.06583	C	0.2098	1.319
1	rs2275237	150785811	A	0.04639	0.0325	G	0.2099	1.448
1	exm165131	245849729	A	0.1005	0.08	G	0.2099	1.285
1	rs3820246	245849729	A	0.1005	0.08	G	0.2099	1.285
2	rs2302694	170088351	A	0.1005	0.08	G	0.2099	1.285

8	rs4434604	67064613	G	0.1005	0.08	A	0.2099	1.285
9	rs512110	79320640	G	0.04639	0.0325	A	0.2099	1.448
9	rs517634	79321276	A	0.04639	0.0325	G	0.2099	1.448
9	rs680775	79322674	A	0.04639	0.0325	G	0.2099	1.448
11	rs2468844	18267027	G	0.1005	0.08	A	0.2099	1.285
14	rs10483853	73756299	A	0.2912	0.3267	G	0.2099	0.847
19	rs17685098	2396611	G	0.4201	0.3842	A	0.2099	1.161
20	rs4358188	36946848	A	0.25	0.2192	G	0.2099	1.188
22	rs144665682	50303575	A	0.04639	0.0325	G	0.2099	1.448
9	rs141463239	97062283	A	0.02073	0.01167	G	0.21	1.793
7	rs10953315	100648029	G	0.1881	0.16	A	0.2101	1.217
3	rs1972977	151463421	C	0.3686	0.405	A	0.2103	0.8575
7	rs4132601	50470604	C	0.1392	0.115	A	0.2103	1.244
14	rs4902176	63174960	A	0.1392	0.115	G	0.2103	1.244
19	rs78320454	4294807	A	0.1392	0.115	G	0.2103	1.244
1	rs3814300	152275640	A	0.03608	0.02417	G	0.2104	1.512
5	rs758037	145393364	G	0.2938	0.26	A	0.2104	1.184
8	rs118172137	142367014	A	0.03608	0.02417	G	0.2104	1.512
14	rs202025710	59931280	A	0.03608	0.02417	G	0.2104	1.512
17	rs8081653	10667638	C	0.03608	0.02417	A	0.2104	1.512
19	rs116900177	39390785	A	0.03608	0.02417	G	0.2104	1.512
2	rs2280718	54587596	G	0.2423	0.275	A	0.2105	0.8429
8	rs7839934	144941181	C	0.2423	0.275	G	0.2105	0.8429
15	rs28756768	43531411	C	0.2423	0.275	G	0.2105	0.8429
10	rs2270182	115392919	T	0.2165	0.1875	A	0.2106	1.197
11	rs3817334	47650993	A	0.3454	0.3108	G	0.2106	1.17
11	rs10838738	47663049	G	0.3454	0.3108	A	0.2106	1.17
7	rs3800952	99757954	A	0.04381	0.0625	G	0.2107	0.6873
21	rs56680307	42749081	G	0.04381	0.0625	A	0.2107	0.6873
1	rs1464461	237921596	A	0.3711	0.4075	G	0.2108	0.8581
6	rs2305089	166579270	A	0.2938	0.3292	G	0.2108	0.8479
10	rs2295878	123996934	A	0.1727	0.2025	G	0.2108	0.822
10	rs2901286	122900623	A	0.1727	0.2033	C	0.2109	0.8178
13	rs561072	33704651	A	0.4304	0.3933	G	0.2111	1.166
17	rs2301354	39643646	A	0.2938	0.3283	G	0.2111	0.8511
1	rs138266774	247464573	A	0.02062	0.01167	G	0.2112	1.783
2	rs149628174	242695306	A	0.02062	0.01167	G	0.2112	1.783
4	rs151072018	54257655	G	0.02062	0.01167	A	0.2112	1.783
4	rs146168958	146073711	G	0.02062	0.01167	A	0.2112	1.783
6	rs739784	30098135	C	0.02062	0.01167	G	0.2112	1.783
7	rs151075297	75187016	A	0.02062	0.01167	G	0.2112	1.783
8	rs74996838	11708479	G	0.02062	0.01167	A	0.2112	1.783
10	rs2669130	5982577	G	0.08505	0.1083	A	0.2112	0.7651

10	rs186197369	43326351	C	0.02062	0.01167	G	0.2112	1.783
11	rs151042558	7949294	A	0.02062	0.01167	G	0.2112	1.783
13	rs201483142	109610116	A	0.02062	0.01167	G	0.2112	1.783
16	rs140370418	2811962	A	0.02062	0.01167	G	0.2112	1.783
17	rs143989935	6722546	A	0.02062	0.01167	G	0.2112	1.783
17	rs1380657	48598785	G	0.08505	0.1083	A	0.2112	0.7651
17	rs139139850	76198639	A	0.02062	0.01167	G	0.2112	1.783
18	rs61743415	44559844	A	0.02062	0.01167	G	0.2112	1.783
18	rs72921303	44560038	A	0.02062	0.01167	C	0.2112	1.783
18	rs72921305	44560123	A	0.02062	0.01167	G	0.2112	1.783
1	rs140817021	6534182	G	0	0.006667	A	0.2113	0
1	rs189399027	28071229	G	0	0.006667	A	0.2113	0
1	rs143956189	47717505	G	0	0.006667	A	0.2113	0
1	rs112795811	100379160	A	0	0.006667	G	0.2113	0
1	rs189870832	162731077	A	0	0.006667	G	0.2113	0
1	rs200531164	247420287	A	0	0.006667	T	0.2113	0
1	rs140972140	248551324	A	0	0.006667	G	0.2113	0
2	rs75602337	20132090	G	0	0.006667	A	0.2113	0
2	rs143542688	27459256	A	0	0.006667	T	0.2113	0
2	rs6750486	35526918	A	0.1186	0.09667	G	0.2113	1.257
2	rs199581012	40655858	A	0	0.006667	G	0.2113	0
2	rs201796380	47357383	A	0	0.006667	G	0.2113	0
2	rs76921212	85554287	G	0	0.006667	A	0.2113	0
2	rs201374872	95954264	G	0	0.006667	A	0.2113	0
2	rs8192507	120129886	A	0.02835	0.0175	G	0.2113	1.638
2	rs117608083	163144647	G	0	0.006667	A	0.2113	0
2	rs6704644	234406655	A	0.02835	0.0175	C	0.2113	1.638
3	rs143133121	58502943	A	0	0.006667	G	0.2113	0
3	rs145653627	180321090	C	0	0.006667	A	0.2113	0
4	rs1801214	6303022	G	0.02835	0.0175	A	0.2113	1.638
4	exm2241327	80328142	A	0	0.006667	G	0.2113	0
4	rs186261533	88231477	G	0	0.006667	A	0.2113	0
4	rs75753256	134071430	C	0	0.006667	A	0.2113	0
4	rs2175563	141320021	A	0	0.006667	G	0.2113	0
5	rs35080306	74017590	G	0.02835	0.0175	C	0.2113	1.638
5	rs16899484	81608521	A	0	0.006667	C	0.2113	0
5	rs17138681	115358137	A	0.02835	0.0175	G	0.2113	1.638
6	rs144676135	4995505	G	0	0.006667	A	0.2113	0
6	rs3025623	29607747	A	0	0.006667	G	0.2113	0
6	exm-IND6-2974(	29638516	D	0	0.006667	I	0.2113	0
6	rs61729757	29760462	G	0	0.006667	A	0.2113	0
6	rs9468626	29769246	A	0	0.006667	G	0.2113	0
6	rs2057726	30335350	G	0	0.006667	A	0.2113	0

6	rs564533	74072937	G	0.02835	0.0175	C	0.2113	1.638
6	rs202244416	136565919	A	0	0.006667	G	0.2113	0
6	rs147983194	136599714	A	0	0.006667	G	0.2113	0
7	rs192974657	47342658	A	0	0.006667	G	0.2113	0
7	rs201720579	73635063	G	0	0.006667	A	0.2113	0
7	rs114003896	103234329	C	0	0.006667	A	0.2113	0
7	rs75191113	151859288	A	0.02835	0.0175	C	0.2113	1.638
8	rs147306385	42552742	A	0	0.006667	G	0.2113	0
8	rs199611064	144803934	A	0	0.006667	G	0.2113	0
8	rs138538445	145580008	A	0	0.006667	G	0.2113	0
9	rs143382624	2719083	G	0	0.006667	C	0.2113	0
9	rs142986969	34017115	A	0	0.006667	G	0.2113	0
9	rs199476092	98229479	G	0	0.006667	A	0.2113	0
9	rs75516981	100201542	A	0	0.006667	G	0.2113	0
9	rs117071184	109691690	A	0	0.006667	G	0.2113	0
10	rs185249748	98744019	A	0	0.006667	G	0.2113	0
10	rs146176839	104130573	A	0	0.006667	G	0.2113	0
11	rs80159720	6942555	G	0	0.006667	A	0.2113	0
11	rs114062454	62037726	G	0	0.006667	C	0.2113	0
11	rs200684568	65618258	A	0	0.006667	G	0.2113	0
11	rs139441816	102272889	A	0	0.006667	G	0.2113	0
12	rs187271032	10007062	G	0	0.006667	A	0.2113	0
12	rs2306852	27089540	G	0	0.006667	A	0.2113	0
12	rs150946604	56213186	G	0	0.006667	A	0.2113	0
12	rs115614701	93134648	A	0	0.006667	G	0.2113	0
12	rs141857063	94976125	A	0	0.006667	G	0.2113	0
12	rs183059673	96371782	A	0	0.006667	G	0.2113	0
14	rs193095681	24768291	A	0	0.006667	G	0.2113	0
14	rs190070568	33293244	G	0	0.006667	A	0.2113	0
15	rs138185152	86800251	C	0	0.006667	A	0.2113	0
15	rs199904269	91424255	G	0	0.006667	A	0.2113	0
16	rs139329616	56918023	A	0	0.006667	G	0.2113	0
16	rs192739719	71319715	A	0	0.006667	G	0.2113	0
16	rs185611053	71570978	A	0	0.006667	G	0.2113	0
16	rs35370634	72184566	A	0.02835	0.0175	G	0.2113	1.638
17	rs202197762	39092808	C	0	0.006667	A	0.2113	0
17	rs200780085	43924028	G	0	0.006667	A	0.2113	0
17	rs200195383	45299073	C	0	0.006667	A	0.2113	0
19	rs11544610	8430886	A	0.02835	0.0175	G	0.2113	1.638
19	rs143006157	13226154	A	0	0.006667	G	0.2113	0
19	rs4808907	19136541	G	0.1186	0.09667	A	0.2113	1.257
19	rs10420608	35617920	A	0	0.006667	G	0.2113	0
19	rs141415697	38190092	G	0.02835	0.0175	A	0.2113	1.638

19	rs182097373	38853095	G	0	0.006667	A	0.2113	0
19	rs181607989	58597580	A	0	0.006667	G	0.2113	0
20	rs144748129	33584465	A	0	0.006667	G	0.2113	0
22	rs115199082	19960533	A	0	0.006667	G	0.2113	0
22	rs141868181	46759949	A	0	0.006667	G	0.2113	0
2	rs185632404	136564939	A	0	0.006667	G	0.2115	0
7	rs6945843	65734783	A	0.3479	0.3133	G	0.2115	1.169
12	rs3741883	106460938	C	0.3763	0.4133	G	0.2115	0.8563
4	rs7694491	95290250	A	0.2448	0.2783	G	0.2116	0.8407
15	rs1197682	42171483	A	0.2526	0.2217	G	0.2116	1.187
18	rs616114	29797921	A	0.2448	0.2783	G	0.2116	0.8407
7	rs41350445	73455561	A	0.02835	0.01753	G	0.2117	1.635
11	rs192806153	68704415	A	0	0.006734	G	0.2117	0
14	rs17563	54417522	G	0.2461	0.2792	A	0.2117	0.843
11	rs376152	16883854	G	0.1031	0.1283	A	0.2118	0.7807
7	rs1419391	126924753	A	0.3505	0.315	G	0.2119	1.174
11	rs3019708	96535365	G	0.3763	0.4125	A	0.2119	0.8593
19	exm1439563	16601194	G	0.3505	0.315	A	0.2119	1.174
19	rs3810198	16601194	G	0.3505	0.315	A	0.2119	1.174
3	rs7612209	177596989	A	0.433	0.3967	G	0.212	1.161
9	rs1043836	116136198	A	0.433	0.3967	G	0.212	1.161
14	rs11628722	94931105	A	0.4356	0.3983	G	0.212	1.166
19	rs7252988	48800338	G	0.2448	0.2775	A	0.212	0.8442
20	rs6138482	25059442	G	0.3789	0.4158	A	0.212	0.8569
20	rs34515071	56034988	G	0.433	0.3967	A	0.212	1.161
2	rs4667010	185944619	G	0.1469	0.1758	A	0.2121	0.8072
7	rs10244051	15063833	A	0.2964	0.3308	C	0.2121	0.852
11	rs11041115	6976996	A	0.1237	0.1508	G	0.2123	0.7948
5	rs7735138	37239240	C	0.4381	0.4008	A	0.2124	1.166
14	rs1051388	61745417	A	0.3789	0.415	G	0.2124	0.8598
5	rs1131769	138857919	A	0.1418	0.1167	G	0.2126	1.251
11	rs12273892	64480930	A	0.2191	0.19	T	0.2127	1.196
16	rs2923147	57762401	G	0.384	0.4215	A	0.2127	0.8555
1	rs61851487	225373072	A	0.299	0.3342	G	0.2128	0.8498
11	exm1712590	48266736	G	0.1907	0.1625	C	0.2128	1.215
11	rs16905753	48285906	A	0.1907	0.1625	G	0.2128	1.215
19	rs2258557	58596449	A	0.1907	0.1625	G	0.2128	1.215
12	rs2228226	57865821	G	0.3814	0.4175	C	0.2129	0.8604
1	rs2229857	154573967	A	0.3005	0.2667	G	0.213	1.181
3	rs295322	141326602	G	0.2474	0.2808	A	0.213	0.8419
17	rs9907151	42744238	C	0.07216	0.05417	A	0.213	1.358
6	rs449643	31936679	A	0.1649	0.1383	G	0.2131	1.23
6	rs4711312	32814659	G	0.1649	0.1383	A	0.2131	1.23

6	rs1135216	32814975	G	0.1649	0.1383	A	0.2131	1.23
7	exm2258251	16640491	A	0.1031	0.08167	G	0.2131	1.293
7	rs541797	16640491	A	0.1031	0.08167	G	0.2131	1.293
9	rs10983238	119333683	C	0.1649	0.1383	G	0.2131	1.23
10	rs7088737	113672010	G	0.299	0.3333	A	0.2131	0.8529
20	rs450739	1210647	G	0.1753	0.205	A	0.2131	0.8241
7	rs4078096	131358281	A	0.4407	0.4042	G	0.2132	1.162
16	rs6498169	11249329	A	0.4407	0.4042	G	0.2132	1.162
10	rs10761779	65274927	G	0.3866	0.4242	A	0.2133	0.8556
20	rs4911287	31627291	A	0.2552	0.2242	G	0.2133	1.186
2	rs3771350	242110786	G	0.3866	0.4233	A	0.2134	0.8585
3	rs6438869	124728626	A	0.384	0.42	G	0.2134	0.8609
6	rs2072915	33162082	T	0.2964	0.2642	A	0.2134	1.173
9	rs10512248	98259703	C	0.2964	0.2642	A	0.2134	1.173
2	rs11542478	38938	C	0.0567	0.07667	A	0.2135	0.7239
6	rs9373523	147701133	A	0.4433	0.4067	C	0.2135	1.162
8	rs3735972	87588198	G	0.0567	0.07667	A	0.2135	0.7239
14	rs17776811	90066451	C	0.1211	0.09833	A	0.2135	1.264
1	rs950210	225569241	A	0.2216	0.1917	C	0.2137	1.201
11	rs4939208	58126305	A	0.2216	0.1917	T	0.2137	1.201
15	rs425829	66270361	A	0.3557	0.32	G	0.2137	1.173
19	rs8100718	45016116	G	0.3557	0.32	A	0.2137	1.173
5	rs6896087	139375932	A	0.3892	0.4258	G	0.2138	0.8591
14	rs243284	33713054	A	0.3892	0.4258	G	0.2138	0.8591
15	rs2703580	57353034	A	0.3892	0.4258	G	0.2138	0.8591
6	rs1225746	10955408	G	0.2088	0.24	A	0.2139	0.8355
3	rs1056522	126261345	A	0.2577	0.2258	G	0.2141	1.19
5	rs9790916	118506685	A	0.3918	0.4283	G	0.2142	0.8596
6	rs9276810	32810443	A	0.4433	0.4075	G	0.2142	1.158
11	rs2270993	48145166	A	0.1907	0.1633	G	0.2142	1.207
11	rs11039571	48207259	A	0.1907	0.1633	G	0.2142	1.207
14	rs7146473	79451465	G	0.3918	0.4283	A	0.2142	0.8596
17	rs3809876	39959433	G	0.3943	0.4313	A	0.2142	0.8584
9	rs1935338	74039166	G	0.4485	0.4117	A	0.2143	1.162
13	rs4770394	23692978	G	0.25	0.2842	A	0.2144	0.8397
4	rs1394845	150712923	G	0.4459	0.41	A	0.2145	1.158
11	rs10791078	130417573	A	0.4459	0.41	G	0.2145	1.158
2	rs4971516	20903015	G	0.1649	0.1392	A	0.2146	1.222
2	rs7586970	188343497	G	0.08763	0.0675	A	0.2146	1.327
4	rs3733617	114278277	A	0.1649	0.1392	G	0.2146	1.222
6	rs3129158	29141772	A	0.4016	0.4379	G	0.2146	0.8612
6	rs441576	33008546	G	0.451	0.4142	A	0.2146	1.162
11	rs1792351	92316799	C	0.3041	0.3389	A	0.2147	0.8525

2	rs55972894	78104290	G	0.3969	0.4342	A	0.2148	0.8577
2	rs2288210	152422076	G	0.2216	0.1925	C	0.2148	1.195
7	rs3750103	29519929	G	0.1495	0.1783	A	0.2148	0.8098
10	rs2070845	91066075	G	0.1495	0.1783	A	0.2148	0.8098
13	rs7318267	98896776	G	0.3557	0.3217	A	0.215	1.164
5	rs2287870	52371089	G	0.01804	0.03083	A	0.2151	0.5775
6	rs6569930	134461880	C	0.01804	0.03083	A	0.2151	0.5775
11	rs80293525	92087959	A	0.01804	0.03083	G	0.2151	0.5775
14	rs2180792	39650234	A	0.01804	0.03083	T	0.2151	0.5775
16	rs143004911	88804365	A	0.01804	0.03083	G	0.2151	0.5775
1	rs6685064	1211292	G	0.451	0.415	A	0.2152	1.158
3	rs323623	151978186	A	0.4021	0.4392	C	0.2155	0.8587
4	rs17724993	6598184	A	0.3067	0.3425	G	0.2155	0.8492
4	rs3775948	9995182	C	0.4021	0.4392	G	0.2155	0.8587
6	rs3123101	159185563	T	0.1933	0.165	A	0.2155	1.213
10	rs6537579	50034833	A	0.1933	0.165	G	0.2155	1.213
14	rs4243749	104849219	G	0.2113	0.2433	A	0.2155	0.8333
17	rs2279054	74287594	G	0.3995	0.4358	A	0.2155	0.8611
8	rs6578185	142458053	G	0.3067	0.3417	A	0.2156	0.8524
8	rs3739310	12878807	A	0.2526	0.2867	C	0.2157	0.8409
9	rs7023244	14819370	A	0.1263	0.1533	C	0.2157	0.7981
1	rs3795251	76397972	G	0.3608	0.3258	A	0.2158	1.168
6	rs3129945	32342537	A	0.3608	0.3258	G	0.2158	1.168
2	rs1260326	27730940	G	0.4613	0.4242	A	0.2159	1.163
7	rs10952179	9770307	G	0.4046	0.4417	A	0.2159	0.8592
9	rs10757957	30476239	A	0.4613	0.4242	G	0.2159	1.163
10	rs3013936	49201964	A	0.2539	0.2867	G	0.2159	0.8467
12	rs73159540	129559421	A	0.1057	0.1308	G	0.2159	0.7849
20	rs726256	17456843	G	0.4046	0.4417	A	0.2159	0.8592
4	rs7677363	186631388	A	0.4588	0.4225	G	0.216	1.159
3	rs6439371	132610752	G	0.3041	0.2708	A	0.2161	1.177
6	rs3757370	88231226	G	0.1057	0.13	A	0.2161	0.7907
8	rs1048101	26628028	A	0.1057	0.13	G	0.2161	0.7907
10	rs1320448	105846074	A	0.1211	0.09917	G	0.2161	1.252
13	rs9523352	92188512	A	0.3041	0.2708	G	0.2161	1.177
1	rs2230678	29475341	A	0.1675	0.1408	G	0.2164	1.228
1	rs2230677	29475394	C	0.1675	0.1408	G	0.2164	1.228
3	rs9864051	41725005	G	0.1675	0.1408	A	0.2164	1.228
11	rs3817198	1909006	G	0.1675	0.1408	A	0.2164	1.228
22	rs148922297	44022345	A	0.02577	0.0409	G	0.2164	0.6203
7	rs10250876	34497585	A	0.4098	0.4467	G	0.2165	0.8601
18	rs8096775	9782448	G	0.4098	0.4467	A	0.2165	0.8601
6	rs74946910	36262153	A	0.1443	0.1192	G	0.2166	1.247

11	rs1051992	6340706	A	0.3608	0.3267	G	0.2166	1.164
14	rs1060570	73138189	C	0.4072	0.4433	A	0.2166	0.8626
6	rs56307355	43581755	G	0.01804	0.03167	A	0.2167	0.5618
6	rs9488991	116951678	C	0.01804	0.03167	A	0.2167	0.5618
12	rs1024582	2402246	A	0.01804	0.03167	G	0.2167	0.5618
19	rs12459238	16593359	A	0.01804	0.03167	G	0.2167	0.5618
1	rs1065761	203186093	G	0.08763	0.1117	C	0.2168	0.7641
1	rs2477599	236413230	A	0.08763	0.1117	T	0.2168	0.7641
21	rs2837960	42511918	C	0.1933	0.1658	A	0.2168	1.205
3	rs6766244	147121751	A	0.07216	0.055	G	0.2169	1.336
1	rs10752633	159896294	A	0.2552	0.2883	G	0.217	0.8455
10	rs590937	43149991	G	0.4098	0.4458	A	0.217	0.863
10	rs3763763	124013604	A	0.2552	0.2883	C	0.217	0.8455
8	rs9325872	20480271	G	0.08763	0.06833	A	0.2171	1.309
8	rs11545078	63938764	A	0.08763	0.06833	G	0.2171	1.309
3	rs34788938	46759010	C	0.2139	0.2458	A	0.2172	0.8348
5	rs10077875	128722696	G	0.4149	0.4517	A	0.2172	0.861
21	rs686364	31587793	G	0.4149	0.4517	A	0.2172	0.861
1	rs1801133	11856378	A	0.4175	0.455	G	0.2173	0.8586
6	rs4520040	104432165	A	0.4175	0.455	G	0.2173	0.8586
15	rs13180	78789488	A	0.4716	0.435	G	0.2173	1.159
20	rs12625565	39986085	A	0.4124	0.4483	C	0.2173	0.8635
6	rs1983891	41536427	A	0.3119	0.3467	G	0.2174	0.8541
8	rs67958403	124828616	A	0.3634	0.3292	G	0.2174	1.163
1	rs7520258	236313127	G	0.1804	0.2108	A	0.2175	0.8239
2	rs3820700	73716810	A	0.2629	0.2308	G	0.2175	1.188
10	rs7096356	89912106	C	0.2139	0.245	A	0.2175	0.8386
12	rs2255336	10532326	A	0.1804	0.2108	G	0.2175	0.8239
13	rs1218307	79417832	A	0.366	0.3308	G	0.2175	1.168
18	rs498703	10478759	G	0.4742	0.4375	A	0.2175	1.16
2	rs3771333	234749722	C	0.07216	0.09417	A	0.2177	0.7482
10	rs4622160	29550249	A	0.07216	0.09417	G	0.2177	0.7482
2	rs700651	198631714	G	0.4201	0.4567	A	0.2178	0.8619
6	rs2856726	32666721	A	0.4201	0.4567	T	0.2178	0.8619
14	rs11157729	50583083	A	0.4716	0.4358	C	0.2179	1.156
18	rs1377016	44063598	G	0.2268	0.1967	A	0.2179	1.198
21	rs7283807	22539376	A	0.4716	0.4358	G	0.2179	1.156
1	rs6427504	160124875	G	0.3144	0.3497	A	0.218	0.8527
3	rs11712165	119118796	C	0.4227	0.4592	A	0.218	0.8624
6	rs3005511	74605806	A	0.4253	0.4625	G	0.218	0.8599
9	rs6475467	20638248	A	0.4227	0.4592	G	0.218	0.8624
15	rs4775261	60630902	A	0.4742	0.4383	C	0.2181	1.156
15	rs743581	74328141	A	0.4742	0.4383	C	0.2181	1.156

16	rs837763	88853729	G	0.3144	0.35	A	0.2181	0.8518
17	rs17711371	51529139	G	0.3144	0.35	A	0.2181	0.8518
1	rs11578336	166958710	C	0.2577	0.2917	A	0.2182	0.8433
4	rs316452	73657570	A	0.3144	0.3492	G	0.2182	0.8549
4	rs3775291	187004074	A	0.2577	0.2917	G	0.2182	0.8433
6	rs17194460	30131546	G	0.3067	0.2742	A	0.2182	1.171
7	rs35332062	73012042	A	0.1237	0.1008	G	0.2182	1.259
12	rs7968890	24990707	A	0.366	0.3317	G	0.2182	1.163
13	rs2806592	79152524	G	0.1237	0.1008	A	0.2182	1.259
20	rs6088792	33909784	A	0.1237	0.1008	G	0.2182	1.259
21	rs2827150	23323887	G	0.3144	0.3492	A	0.2182	0.8549
1	rs4920310	18563889	G	0.4278	0.465	A	0.2183	0.8603
1	rs2640464	29519778	A	0.4768	0.4408	C	0.2183	1.156
12	rs1347570	29604392	C	0.4278	0.465	G	0.2183	0.8603
1	rs1349566	195923203	A	0.4304	0.4675	C	0.2185	0.8607
4	rs6447368	44682465	G	0.482	0.4458	A	0.2186	1.156
6	rs34068461	80228535	C	0.2629	0.2321	A	0.2186	1.18
7	rs2074000	20685484	C	0.4278	0.4642	A	0.2186	0.8632
11	rs6265	27679916	A	0.482	0.4458	G	0.2186	1.156
7	rs2159570	123850419	A	0.433	0.47	G	0.2187	0.8611
8	rs1367972	62003453	G	0.433	0.47	A	0.2187	0.8611
9	rs541805	25934932	A	0.433	0.47	G	0.2187	0.8611
17	rs2003968	2266812	A	0.2655	0.2332	G	0.2187	1.188
1	rs4908482	7877488	G	0.4845	0.4483	A	0.2188	1.157
4	rs7693478	166981852	A	0.4923	0.455	G	0.2188	1.161
6	rs572012	168096647	C	0.4923	0.455	A	0.2188	1.161
8	rs12677741	52529097	A	0.4304	0.4667	G	0.2188	0.8636
17	rs242557	44019712	G	0.4845	0.4483	A	0.2188	1.157
18	rs1424401	53640779	A	0.4304	0.4667	G	0.2188	0.8636
20	rs6134919	13562481	G	0.4923	0.455	A	0.2188	1.161
2	rs1427407	60718043	A	0.2268	0.1975	C	0.2189	1.192
6	rs3094727	29672529	A	0.2165	0.2483	G	0.2189	0.8364
6	rs2524272	29682400	G	0.2165	0.2483	A	0.2189	0.8364
6	rs2327800	137508437	A	0.4356	0.4725	G	0.2189	0.8615
10	rs10761659	64445564	A	0.2165	0.2483	G	0.2189	0.8364
12	rs10843206	28722756	A	0.317	0.3525	G	0.2189	0.8526
17	rs557491	67267317	A	0.317	0.3525	G	0.2189	0.8526
5	rs10064163	111979706	C	0.4897	0.4533	A	0.219	1.157
6	rs1042153	33048663	A	0.1289	0.1558	G	0.219	0.8013
12	rs242018	3788145	A	0.1289	0.1558	G	0.219	0.8013
12	rs242017	3788222	A	0.1289	0.1558	G	0.219	0.8013
6	rs6933050	31343632	G	0.3711	0.3358	A	0.2191	1.167
12	rs17836273	3789452	A	0.1289	0.155	G	0.2191	0.8065

19	rs4606855	14769339	C	0.1289	0.155	G	0.2191	0.8065
19	rs1078373	31801130	A	0.4637	0.5008	G	0.2191	0.8619
3	rs13462	170584249	A	0.04897	0.03417	C	0.2192	1.456
6	rs9767441	169496144	C	0.4923	0.4558	A	0.2192	1.157
8	rs75318724	2033446	A	0.04897	0.03417	G	0.2192	1.456
17	rs80084721	73759113	A	0.04897	0.03417	G	0.2192	1.456
5	rs57392066	158258461	G	0.4356	0.4717	A	0.2193	0.8644
10	rs2153136	57584038	A	0.4356	0.4717	G	0.2193	0.8644
2	rs7587059	236021090	A	0.4459	0.4833	G	0.2194	0.8601
5	rs1966590	24779565	A	0.1959	0.1683	G	0.2194	1.203
20	rs3761161	10110237	A	0.4433	0.48	G	0.2194	0.8627
9	rs2209440	17736342	C	0.4381	0.4742	A	0.2195	0.8648
10	rs180722	117728741	A	0.4485	0.4858	G	0.2195	0.8605
11	rs2513116	106972340	G	0.4485	0.4858	A	0.2195	0.8605
1	rs7512898	200672906	C	0.5026	0.4658	A	0.2196	1.159
4	rs2247341	1701317	G	0.2603	0.2933	A	0.2196	0.8478
4	rs2280099	90035549	G	0.1701	0.1433	A	0.2196	1.225
6	rs6911143	86221771	A	0.451	0.4883	G	0.2196	0.8609
7	rs2686817	47968927	C	0.4459	0.4825	A	0.2196	0.863
4	rs9312345	187091644	A	0.04639	0.06417	C	0.2197	0.7095
13	rs7328282	99554955	A	0.3196	0.355	G	0.2197	0.8534
3	rs4685147	14424656	G	0.3737	0.3383	A	0.2198	1.167
3	rs2686268	71145830	A	0.4562	0.4933	C	0.2198	0.8615
11	rs672113	119386594	A	0.4407	0.4767	G	0.2198	0.8652
17	rs1563632	18220770	G	0.4588	0.4958	A	0.2198	0.8619
1	rs1369023	98993058	A	0.451	0.4875	G	0.2199	0.8637
2	rs12611515	133541575	A	0.5129	0.4758	G	0.2199	1.16
8	rs3108919	101841546	A	0.5155	0.4783	G	0.2199	1.16
14	rs1154155	23002684	C	0.5155	0.4783	A	0.2199	1.16
14	rs2295847	51202311	C	0.4588	0.4958	G	0.2199	0.8619
18	rs4797585	11911143	G	0.518	0.4808	A	0.2199	1.161
20	rs856427	51251485	A	0.451	0.4875	G	0.2199	0.8637
22	rs2241230	20784050	T	0.5129	0.4758	A	0.2199	1.16
1	rs41264632	153276449	G	0.1082	0.1333	A	0.22	0.789
3	rs9858491	76992952	G	0.4716	0.5092	A	0.22	0.8605
6	rs9381468	46625274	G	0.5232	0.4858	A	0.22	1.161
17	rs9754	34261831	G	0.2294	0.1992	A	0.22	1.197
19	rs1469087	58572979	A	0.5206	0.4833	G	0.22	1.161
3	rs78245253	128204693	G	0.04639	0.065	C	0.2201	0.6998
10	rs2399786	12215180	G	0.4665	0.5033	A	0.2201	0.8628
10	rs1864345	49420008	G	0.04639	0.065	A	0.2201	0.6998
12	rs7953274	87901028	A	0.4639	0.5008	G	0.2201	0.8625
17	rs9303521	43805194	A	0.5	0.4642	C	0.2201	1.154

1	rs927743	59368947	C	0.5077	0.4717	A	0.2202	1.155
1	rs6687419	240869087	A	0.4562	0.4925	G	0.2202	0.8644
6	rs2132683	99672014	A	0.5077	0.4717	G	0.2202	1.155
8	rs1383914	26723049	A	0.5103	0.4742	G	0.2202	1.156
11	rs900145	13293905	G	0.4562	0.4925	A	0.2202	0.8644
12	rs1896312	115346424	G	0.5258	0.4892	A	0.2202	1.158
21	rs73364974	41917406	C	0.1082	0.1325	A	0.2202	0.7947
1	rs2797685	7879063	G	0.518	0.4817	A	0.2203	1.157
3	rs2872373	78107128	C	0.5129	0.4767	A	0.2203	1.156
5	rs9112	121518309	A	0.5129	0.4767	G	0.2203	1.156
6	rs3127328	166572045	A	0.06053	0.045	G	0.2203	1.367
17	rs8080167	13949828	A	0.5155	0.4792	G	0.2203	1.156
20	rs396373	42291567	G	0.5206	0.4842	A	0.2203	1.157
1	rs6690903	228057761	A	0.3119	0.2792	G	0.2205	1.17
2	rs1008002	5847893	G	0.4639	0.5	A	0.2205	0.8654
5	rs2369754	99156362	A	0.4639	0.5	C	0.2205	0.8654
8	rs13277113	11349186	G	0.3119	0.2792	A	0.2205	1.17
9	rs10756461	13185149	A	0.4639	0.5	G	0.2205	0.8654
22	rs11553143	50754512	A	0.1469	0.1217	G	0.2205	1.243
3	rs1274958	39184959	G	0.2191	0.2508	A	0.2206	0.8379
6	rs3798751	10942964	G	0.2191	0.2508	A	0.2206	0.8379
22	rs7290832	27328787	G	0.3763	0.3408	A	0.2206	1.167
4	exm2256408	152609784	A	0.05928	0.04417	G	0.2208	1.364
4	rs75029272	152609784	A	0.05928	0.04417	G	0.2208	1.364
7	rs42524	94043239	G	0.05928	0.04417	C	0.2208	1.364
11	rs2276054	124006972	A	0.05928	0.04417	C	0.2208	1.364
11	rs2276053	124006993	A	0.05928	0.04417	G	0.2208	1.364
15	rs12440300	69548338	G	0.05928	0.04417	A	0.2208	1.364
1	rs3820129	147091972	A	0.0599	0.07983	G	0.2209	0.7344
17	rs17854914	4721376	G	0.08763	0.06917	A	0.2209	1.293
21	rs145732243	45707457	A	0.01554	0.0075	G	0.2209	2.089
1	rs2820289	201777250	A	0.2294	0.2	G	0.221	1.191
4	rs2515960	2951804	G	0.2294	0.2	A	0.221	1.191
12	rs2698081	119294242	A	0.3808	0.345	G	0.221	1.168
21	rs2284472	32515535	A	0.3144	0.2808	G	0.221	1.175
1	rs1541185	20141528	A	0.3763	0.3417	G	0.2211	1.162
6	rs9295930	30849822	A	0.3763	0.3417	G	0.2211	1.162
14	rs11845640	33291494	A	0.3247	0.36	G	0.2213	0.855
6	rs2240803	30920957	A	0.3789	0.3433	G	0.2214	1.167
1	rs146746341	86289373	A	0.03866	0.02583	C	0.2215	1.516
6	rs41270461	32065023	A	0.03866	0.02583	G	0.2215	1.516
7	rs61740421	92027080	A	0.03866	0.02583	G	0.2215	1.516
7	rs17159640	112086333	T	0.1057	0.085	A	0.2215	1.272

10	rs151204356	85961608	A	0.03866	0.02583	G	0.2215	1.516
11	rs546359	109697903	G	0.1057	0.085	A	0.2215	1.272
12	rs77759698	54756528	G	0.03866	0.02583	A	0.2215	1.516
14	rs11539752	25101589	G	0.1856	0.2161	C	0.2215	0.8266
14	rs140408138	50583096	G	0.03866	0.02583	A	0.2215	1.516
15	rs80281762	69238660	A	0.03866	0.02583	C	0.2215	1.516
17	rs117932646	72308307	A	0.03866	0.02583	G	0.2215	1.516
1	rs76862302	33245687	G	0.01546	0.0075	A	0.2216	2.079
1	rs143462459	35260527	A	0.01546	0.0075	G	0.2216	2.079
1	rs139429089	90179843	G	0.01546	0.0075	A	0.2216	2.079
2	rs190151140	74750039	G	0.01546	0.0075	A	0.2216	2.079
2	rs3811039	113496520	T	0.01546	0.0075	A	0.2216	2.079
3	rs150518151	37163011	G	0.01546	0.0075	A	0.2216	2.079
3	rs2306983	73113180	A	0.01546	0.0075	G	0.2216	2.079
3	rs7373165	197701913	A	0.01546	0.0075	C	0.2216	2.079
4	rs7442295	9966380	G	0.01546	0.0075	A	0.2216	2.079
4	rs117576731	22390049	A	0.01546	0.0075	G	0.2216	2.079
4	rs4149416	70596385	C	0.01546	0.0075	A	0.2216	2.079
4	rs144689051	187539275	G	0.01546	0.0075	A	0.2216	2.079
7	rs45578433	116436092	A	0.01546	0.0075	G	0.2216	2.079
8	rs144350008	100796564	A	0.01546	0.0075	G	0.2216	2.079
8	rs145333587	120608229	A	0.01546	0.0075	G	0.2216	2.079
8	rs115902639	133945848	A	0.01546	0.0075	G	0.2216	2.079
9	rs188070090	79955345	A	0.01546	0.0075	G	0.2216	2.079
10	rs192099405	97023725	A	0.01546	0.0075	G	0.2216	2.079
11	rs145584690	2924589	A	0.01546	0.0075	G	0.2216	2.079
11	rs200608651	65744142	A	0.01546	0.0075	G	0.2216	2.079
11	rs116693665	123756104	C	0.01546	0.0075	A	0.2216	2.079
12	rs1345092	105536238	T	0.01546	0.0075	A	0.2216	2.079
14	rs17127374	91948043	G	0.01546	0.0075	A	0.2216	2.079
17	rs143966312	34431360	A	0.01546	0.0075	G	0.2216	2.079
17	rs142267571	39777047	A	0.01546	0.0075	G	0.2216	2.079
17	rs200840955	80043274	A	0.01546	0.0075	G	0.2216	2.079
19	rs143506499	37618047	A	0.01546	0.0075	G	0.2216	2.079
20	rs200310862	35243680	G	0.01546	0.0075	C	0.2216	2.079
20	rs2233884	43836261	G	0.01546	0.0075	A	0.2216	2.079
3	rs117692440	50327845	G	0.03608	0.0525	C	0.2217	0.6756
7	rs12540583	102760511	C	0.3144	0.2817	A	0.2217	1.17
8	rs7463937	72597645	A	0.3247	0.3592	G	0.2217	0.8581
9	rs2280843	131585069	A	0.1856	0.2158	G	0.2217	0.8278
12	rs10507047	95604290	G	0.3144	0.2817	A	0.2217	1.17
14	rs2369049	96171851	G	0.03608	0.0525	A	0.2217	0.6756
5	rs11241095	110439509	G	0.268	0.2371	A	0.2218	1.179

19	rs2911006	48182946	A	0.03866	0.02588	G	0.2218	1.514
5	rs3748042	70751781	G	0.01546	0.007513	A	0.2219	2.075
5	rs32462	179328228	A	0.1985	0.1708	G	0.2219	1.202
6	rs4706980	83838673	A	0.1985	0.1708	G	0.2219	1.202
11	rs11544721	68777323	G	0.2668	0.3	A	0.2219	0.8492
12	rs145334570	21011481	A	0.01546	0.007513	C	0.2219	2.075
19	rs1423051	9057408	A	0.1469	0.1225	G	0.2219	1.234
19	rs1895364	9057687	A	0.1469	0.1225	G	0.2219	1.234
1	rs4233367	161163037	A	0.07474	0.05667	G	0.2221	1.345
4	rs6845304	88280502	A	0.3273	0.3625	G	0.2221	0.8557
6	rs9349205	41925159	A	0.232	0.2017	G	0.2221	1.196
12	rs2769481	95796054	A	0.3814	0.3458	G	0.2221	1.166
13	rs9536591	54581087	A	0.3814	0.3458	C	0.2221	1.166
16	rs2279348	89350038	A	0.2216	0.2538	G	0.2221	0.8374
16	rs11648852	87493737	G	0.2216	0.2533	A	0.2223	0.8393
2	rs2305355	98736225	A	0.1314	0.1575	G	0.2224	0.8095
12	rs1351394	66351826	A	0.1314	0.1575	G	0.2224	0.8095
16	rs2059238	78258810	A	0.1314	0.1575	C	0.2224	0.8095
19	rs3826736	7571030	T	0.317	0.2842	A	0.2228	1.169
2	rs17720303	71163173	A	0.2706	0.2392	G	0.2229	1.18
7	rs77721383	82784832	G	0.2706	0.2392	C	0.2229	1.18
7	rs10265	139026152	C	0.2706	0.2392	A	0.2229	1.18
11	rs1883916	33578510	G	0.3299	0.3648	A	0.2229	0.8573
6	rs619203	117622184	G	0.1572	0.185	C	0.223	0.8218
6	rs529156	117622188	C	0.1572	0.185	A	0.223	0.8218
6	rs529038	117622233	A	0.1572	0.185	G	0.223	0.8218
1	rs76782506	12320753	A	0.03608	0.05333	G	0.2231	0.6644
2	rs74727069	15555762	C	0.03608	0.05333	G	0.2231	0.6644
11	rs2905495	60841281	C	0.03608	0.05333	A	0.2231	0.6644
16	rs1304163	31737373	G	0.268	0.3017	A	0.2231	0.8477
19	rs11882256	9060541	G	0.384	0.3492	A	0.2232	1.162
20	rs2423467	9741876	A	0.384	0.3492	G	0.2232	1.162
14	rs3742926	33014869	A	0.201	0.1725	G	0.2233	1.207
14	rs17099240	33015082	G	0.201	0.1725	A	0.2233	1.207
16	rs1134760	67964203	G	0.201	0.1728	A	0.2235	1.205
6	rs9444039	83850049	A	0.07474	0.09667	T	0.2236	0.7549
15	rs76902501	81665021	A	0.07474	0.09583	T	0.2236	0.7621
16	rs4785686	89587871	A	0.3325	0.3675	C	0.2236	0.8572
17	rs3213690	66246416	A	0.1881	0.2183	G	0.2238	0.8297
5	rs10035539	125443261	G	0.1108	0.1358	A	0.2239	0.7929
18	rs2243803	42956672	T	0.2345	0.2042	A	0.2241	1.194
1	rs60950503	197111650	A	0.02835	0.01833	G	0.2242	1.562
3	rs140173974	112546020	A	0.02835	0.01833	G	0.2242	1.562

4	rs76996680	109084821	A	0.02835	0.01833	G	0.2242	1.562
10	rs17116635	105945842	A	0.1469	0.1233	G	0.2242	1.224
17	rs117134739	61556429	G	0.02835	0.01833	A	0.2242	1.562
22	rs148258530	46654664	A	0.02835	0.01833	C	0.2242	1.562
1	rs2229496	152882610	A	0.3351	0.3708	G	0.2243	0.8549
19	rs329966	53070617	G	0.3351	0.3708	A	0.2243	0.8549
1	rs10908292	36764770	G	0.04897	0.035	A	0.2246	1.42
7	rs3087615	102952123	A	0.04897	0.035	C	0.2246	1.42
10	rs113108393	115668149	G	0.04897	0.035	A	0.2246	1.42
11	rs2292290	26586855	G	0.04897	0.035	A	0.2246	1.42
11	rs3782089	65336819	A	0.04897	0.035	G	0.2246	1.42
5	rs10060874	100582655	G	0.3866	0.3525	A	0.2247	1.158
20	rs2249317	1532595	A	0.2345	0.205	G	0.2249	1.188
1	rs2889809	218950403	G	0.3222	0.2892	A	0.225	1.168
7	rs4917152	48506566	G	0.3222	0.2892	A	0.225	1.168
11	rs10750270	124294703	A	0.3402	0.3758	G	0.2253	0.8563
2	rs1908252	225484736	A	0.2732	0.3067	G	0.2255	0.8498
12	rs2926747	57109931	T	0.2732	0.3067	A	0.2255	0.8498
12	rs2958127	57114307	A	0.2732	0.3067	T	0.2255	0.8498
18	rs56913743	13095591	A	0.2268	0.2583	G	0.2255	0.8422
1	rs2986742	6550376	G	0.3247	0.2908	A	0.2256	1.173
2	rs698761	44547574	A	0.3402	0.3758	G	0.2257	0.8563
3	rs17801309	89521664	A	0.07474	0.0575	G	0.2257	1.324
19	rs814501	41038574	A	0.07474	0.0575	G	0.2257	1.324
22	rs76099251	19220971	A	0.07474	0.0575	C	0.2257	1.324
2	rs4673981	216129158	C	0.3402	0.375	A	0.2258	0.8594
6	rs629849	160494409	A	0.1753	0.1483	G	0.2258	1.22
2	rs1514680	1396971	G	0.3918	0.3575	A	0.2259	1.158
6	rs4715166	13216058	G	0.2732	0.3058	A	0.2259	0.8532
10	rs4142048	70044031	G	0.3943	0.3592	A	0.2259	1.162
11	rs2512526	77921527	C	0.3918	0.3575	G	0.2259	1.158
10	rs12358575	122962934	A	0.2758	0.2442	G	0.226	1.179
11	rs17612126	68705674	A	0.3247	0.2917	C	0.2261	1.168
20	rs2250889	44642406	C	0.3247	0.2917	G	0.2261	1.168
6	rs204992	32156908	A	0.1907	0.22	G	0.2262	0.8356
5	rs2973558	73163965	A	0.09278	0.1167	C	0.2263	0.7744
15	rs1197665	42143533	C	0.3969	0.3614	A	0.2263	1.163
17	rs17704679	64023624	G	0.09278	0.1167	A	0.2263	0.7744
16	rs77463028	3613637	A	0.06186	0.04583	G	0.2264	1.373
19	rs59595912	50360989	G	0.06186	0.04583	A	0.2264	1.373
20	rs34323943	52192637	G	0.06186	0.04583	A	0.2264	1.373
1	rs3828024	240694114	A	0.3969	0.3617	G	0.2265	1.162
6	rs2859112	32698013	G	0.3943	0.36	A	0.2265	1.157

17	rs197922	45008570	A	0.3969	0.3617	G	0.2265	1.162
19	rs1061680	55143452	A	0.3428	0.3775	G	0.2266	0.8601
8	rs6991991	81271432	G	0.3454	0.3813	A	0.2268	0.8561
1	rs1635517	242012033	G	0.2371	0.2075	A	0.2269	1.187
6	rs2021368	30429383	A	0.2306	0.2625	G	0.2269	0.8419
2	rs6712932	105837598	G	0.2758	0.245	A	0.227	1.173
6	rs6900311	103225137	A	0.3454	0.3808	C	0.227	0.8577
10	rs7902158	83723457	A	0.2758	0.245	G	0.227	1.173
2	rs7601049	98844674	C	0.2294	0.2608	G	0.2271	0.8435
2	rs11889349	98853173	G	0.2294	0.2608	A	0.2271	0.8435
5	rs339445	13944512	A	0.1082	0.0875	C	0.2271	1.266
12	rs9805130	121715395	A	0.3273	0.2942	G	0.2271	1.168
17	rs2378871	59638769	C	0.1082	0.0875	A	0.2271	1.266
19	rs1122608	11163601	A	0.1082	0.0875	C	0.2271	1.266
19	rs11878666	9058624	A	0.3454	0.38	G	0.2273	0.8608
1	rs3747617	159912899	A	0.1289	0.1058	G	0.2275	1.25
16	rs11150643	31842848	A	0.2784	0.3119	C	0.2276	0.8511
3	rs79979130	52551010	A	0.01289	0.02417	G	0.2277	0.5271
5	rs141100793	43295881	C	0.01289	0.02417	G	0.2277	0.5271
6	rs3778630	31091328	A	0.09021	0.07167	G	0.2277	1.284
6	rs200978040	32945701	A	0.01289	0.02417	C	0.2277	0.5271
6	rs79753406	35201057	G	0.01289	0.02417	A	0.2277	0.5271
7	rs75029939	149482764	A	0.01289	0.02417	G	0.2277	0.5271
10	rs10508517	16997891	A	0.3479	0.3833	G	0.2277	0.8584
11	rs10838375	5602928	C	0.01289	0.02417	G	0.2277	0.5271
11	rs138418543	15170743	A	0.01289	0.02417	G	0.2277	0.5271
11	rs61995733	117279712	A	0.01289	0.02417	G	0.2277	0.5271
14	rs114411077	91666162	A	0.09021	0.07167	G	0.2277	1.284
16	rs138146407	30123523	A	0.01289	0.02417	C	0.2277	0.5271
19	rs12609150	9066874	A	0.3479	0.3833	C	0.2277	0.8584
19	rs769449	45410002	A	0.09021	0.07167	G	0.2277	1.284
1	rs561042	89652071	G	0.4021	0.3667	A	0.2278	1.161
1	rs1142890	89652087	A	0.4021	0.3667	G	0.2278	1.161
1	rs2070065	214811244	G	0.1134	0.1383	C	0.2278	0.7967
1	rs2666839	214816224	A	0.1134	0.1383	G	0.2278	0.7967
1	rs3748693	214818223	T	0.1134	0.1383	A	0.2278	0.7967
6	rs7767455	121642851	A	0.1134	0.1383	G	0.2278	0.7967
6	rs3734795	151674593	A	0.1134	0.1383	C	0.2278	0.7967
7	rs1860521	38331504	A	0.1134	0.1383	G	0.2278	0.7967
16	rs12446956	73501536	G	0.2784	0.3117	A	0.2278	0.8519
1	rs12127588	198595506	A	0.1624	0.1908	G	0.2279	0.8219
2	rs1052483	219934348	A	0.1624	0.1908	C	0.2279	0.8219
2	rs6724465	219943846	A	0.1624	0.1908	G	0.2279	0.8219

15	rs7183263	84573041	A	0.1933	0.2233	C	0.2279	0.8333
15	rs11259936	84580582	A	0.1933	0.2233	C	0.2279	0.8333
15	rs4842838	84582124	C	0.1933	0.2233	A	0.2279	0.8333
13	rs1927667	110115817	A	0.1933	0.2242	G	0.2281	0.8293
1	rs11590090	113313563	A	0.1134	0.1392	C	0.2282	0.7912
9	exm-IND9-13765	138516347	D	0.2062	0.1775	I	0.2282	1.204
11	rs12272403	56143977	G	0.1134	0.1392	A	0.2282	0.7912
6	rs9483751	135128858	G	0.4046	0.3692	A	0.2284	1.161
1	rs35273427	120436751	G	0.01289	0.02517	A	0.2287	0.5055
14	rs7157792	77353103	G	0.232	0.2633	A	0.2287	0.8449
6	rs11970254	67649216	A	0.2397	0.21	C	0.2288	1.186
17	rs3785879	43985636	A	0.4046	0.37	C	0.2288	1.157
3	rs3772173	170078232	G	0.1366	0.1625	A	0.2289	0.8154
6	rs1264323	30855907	G	0.3531	0.3883	A	0.2289	0.8597
9	rs16911681	97522829	A	0.04897	0.06667	G	0.2289	0.7209
1	rs12402583	41306571	A	0.2809	0.2492	G	0.229	1.177
1	rs1065457	158324425	A	0.4072	0.3717	G	0.229	1.161
2	rs7591849	160112881	A	0.2809	0.2492	C	0.229	1.177
4	rs12646270	90857479	A	0.2809	0.2492	G	0.229	1.177
12	rs2681472	90008959	G	0.4072	0.3717	A	0.229	1.161
19	rs344560	6665020	A	0.06186	0.0825	G	0.229	0.7333
3	rs4857855	128260550	A	0.3299	0.2975	G	0.2291	1.163
4	rs2227902	57797100	A	0.04897	0.0675	C	0.2291	0.7113
11	rs1800172	2869129	A	0.04897	0.0675	G	0.2291	0.7113
16	rs148234688	55905571	A	0.04897	0.0675	C	0.2291	0.7113
17	rs117612144	18025140	A	0.06186	0.08264	C	0.2291	0.7319
4	rs2536512	24801315	A	0.3325	0.2992	G	0.2292	1.167
11	rs1378739	4945196	A	0.3325	0.2992	C	0.2292	1.167
8	rs532841	12957475	A	0.3531	0.3875	G	0.2293	0.8627
1	rs1800205	40557033	G	0.1521	0.1275	A	0.2294	1.227
3	rs77982452	64580068	A	0.07732	0.09917	G	0.2294	0.7612
19	rs3745764	37854580	A	0.1521	0.1275	G	0.2294	1.227
1	rs11161747	86591837	G	0.4098	0.3742	A	0.2295	1.161
10	rs2380205	5886734	A	0.07732	0.09833	G	0.2295	0.7684
14	rs2781377	64560092	A	0.07732	0.09833	G	0.2295	0.7684
16	rs11117432	86019271	A	0.07732	0.09833	G	0.2295	0.7684
19	rs2304208	50157946	G	0.07732	0.09833	A	0.2295	0.7684
2	rs77624405	219257795	A	0.03866	0.02667	G	0.2296	1.468
4	rs666088	20495135	G	0.03866	0.02667	A	0.2296	1.468
7	rs34219043	29160604	A	0.03866	0.02667	G	0.2296	1.468
8	rs78140118	144130643	A	0.03866	0.02667	G	0.2296	1.468
11	rs75844061	63057846	G	0.03866	0.02667	A	0.2296	1.468
13	rs70961707	41134096	A	0.03866	0.02667	G	0.2296	1.468

15	rs138712718	41961113	C	0.03866	0.02667	A	0.2296	1.468
16	rs17611866	3367229	G	0.03866	0.02667	A	0.2296	1.468
19	rs1054486	12774208	C	0.03866	0.02667	G	0.2296	1.468
19	rs77422116	42861046	G	0.03866	0.02667	C	0.2296	1.468
19	rs75954299	42937953	C	0.03866	0.02667	A	0.2296	1.468
2	rs6739772	241840711	A	0.2835	0.3175	G	0.2299	0.8506
4	rs150260	16777175	G	0.4098	0.375	A	0.2299	1.157
6	rs2301436	167437988	A	0.4098	0.375	G	0.2299	1.157
2	rs10189857	60713235	A	0.2397	0.2108	G	0.23	1.18
4	rs12715977	177495414	C	0.1959	0.2267	A	0.23	0.8311
7	rs4729645	100675713	A	0.1778	0.1517	G	0.23	1.21
9	rs13291830	140277826	A	0.3557	0.39	G	0.23	0.8634
21	rs1735151	41123301	A	0.2835	0.3167	G	0.23	0.8538
4	rs11096955	38776107	A	0.4124	0.3767	C	0.2301	1.161
4	rs11096957	38776491	A	0.4124	0.3767	C	0.2301	1.161
4	rs17064420	178128842	A	0.4124	0.3767	G	0.2301	1.161
19	rs2722723	44832992	G	0.3325	0.3	C	0.2301	1.162
1	rs1344	147119273	A	0.3582	0.3933	G	0.2302	0.861
2	rs934945	239155053	A	0.3351	0.3017	G	0.2303	1.166
4	rs1522073	17170579	A	0.2345	0.2658	C	0.2303	0.8462
14	rs223116	23977010	A	0.3351	0.3017	G	0.2303	1.166
4	rs4691895	164048199	G	0.1649	0.1933	C	0.2304	0.8242
6	rs9386758	109197379	G	0.2835	0.2517	A	0.2304	1.177
10	rs11203032	90964614	A	0.1649	0.1933	C	0.2304	0.8242
2	rs838543	234432017	G	0.1108	0.08917	A	0.2305	1.273
1	rs12755827	189924896	G	0.3582	0.3925	A	0.2306	0.864
2	rs11898505	54684557	A	0.1649	0.1942	G	0.2307	0.8198
2	rs7601000	242610773	T	0.1649	0.1942	A	0.2307	0.8198
3	rs13326165	52532118	A	0.01289	0.025	G	0.2307	0.5091
4	rs34860182	6925237	C	0.01289	0.025	G	0.2307	0.5091
6	rs11575907	32782112	A	0.01289	0.025	G	0.2307	0.5091
9	rs10761054	107379895	A	0.3608	0.3967	C	0.2307	0.8586
10	rs79826323	5788833	C	0.01289	0.025	G	0.2307	0.5091
11	rs118136428	34938249	C	0.01289	0.025	A	0.2307	0.5091
13	rs139160172	112722387	A	0.01289	0.025	G	0.2307	0.5091
14	rs4981683	29579407	G	0.3608	0.3967	A	0.2307	0.8586
17	rs3826358	64225484	G	0.01289	0.025	A	0.2307	0.5091
19	rs140414099	15163018	G	0.01289	0.025	A	0.2307	0.5091
2	rs12714251	29218411	A	0.3608	0.3958	G	0.2308	0.8616
2	rs117843818	110053570	A	0.07732	0.05917	G	0.2308	1.333
3	rs6785067	186364046	A	0.07732	0.05917	G	0.2308	1.333
6	rs3800381	35209353	A	0.07732	0.05917	G	0.2308	1.333
14	rs7159841	47863835	G	0.3608	0.3958	A	0.2308	0.8616

14	rs175080	75513828	A	0.2062	0.1792	G	0.2308	1.19
17	rs35397826	66270082	G	0.2062	0.1792	A	0.2308	1.19
19	rs2287813	10084292	G	0.3608	0.3958	C	0.2308	0.8616
20	rs147670144	61945439	C	0.01289	0.02504	G	0.2308	0.5083
22	rs8135665	38476276	A	0.09536	0.1192	G	0.2309	0.7792
4	rs10033900	110659067	G	0.2861	0.32	A	0.231	0.8515
9	rs10106	130576075	A	0.3351	0.3025	G	0.231	1.162
2	rs4664229	158386938	G	0.09278	0.07333	A	0.2311	1.292
3	rs75744986	126132999	A	0.09278	0.07333	C	0.2311	1.292
13	rs9569690	58004699	A	0.2861	0.3192	G	0.2311	0.8548
6	rs2057068	41681787	G	0.4124	0.3783	A	0.2312	1.153
16	rs12598474	88501034	C	0.4124	0.3783	G	0.2312	1.153
1	rs72637949	43906169	A	0.02062	0.0125	G	0.2313	1.663
1	rs77900924	93602439	G	0.02062	0.0125	A	0.2313	1.663
2	rs146175795	27721143	A	0.02062	0.0125	G	0.2313	1.663
2	rs10181042	61224259	A	0.02835	0.04333	G	0.2313	0.6442
4	rs11937432	2233709	G	0.02835	0.04333	A	0.2313	0.6442
4	rs75621037	39271653	G	0.02062	0.0125	A	0.2313	1.663
4	rs2114039	55092626	G	0.2835	0.2525	A	0.2313	1.171
6	rs116541124	7402881	G	0.02062	0.0125	A	0.2313	1.663
7	rs142231743	143792747	A	0.02062	0.0125	G	0.2313	1.663
8	rs17833992	121298156	G	0.02835	0.04333	C	0.2313	0.6442
10	rs3765571	97386497	A	0.02835	0.04333	C	0.2313	0.6442
10	rs145837135	108459112	A	0.02062	0.0125	G	0.2313	1.663
10	rs200171942	116048897	A	0.02062	0.0125	G	0.2313	1.663
10	rs73391295	134672609	A	0.02062	0.0125	G	0.2313	1.663
11	rs34364959	2432666	A	0.02835	0.04348	G	0.2313	0.6419
11	rs35525096	18108512	G	0.02062	0.0125	A	0.2313	1.663
12	rs142354668	117691485	G	0.02062	0.0125	A	0.2313	1.663
15	rs117557829	49048668	A	0.02062	0.0125	T	0.2313	1.663
18	rs3794942	72343181	G	0.02062	0.0125	A	0.2313	1.663
19	rs11666735	55396913	A	0.02835	0.04333	G	0.2313	0.6442
20	rs2231839	44171347	C	0.02062	0.0125	A	0.2313	1.663
1	rs7518979	205812912	G	0.3634	0.3983	A	0.2314	0.8622
1	rs12045447	220344453	G	0.02835	0.0425	A	0.2314	0.6574
5	rs138642837	129520338	A	0.02835	0.0425	G	0.2314	0.6574
6	rs2857101	32794676	G	0.3634	0.3983	A	0.2314	0.8622
6	rs881858	43806609	G	0.1521	0.1283	A	0.2314	1.218
12	rs74727297	133272470	A	0.1521	0.1283	C	0.2314	1.218
15	rs10152591	70048157	C	0.02835	0.0425	A	0.2314	0.6574
16	rs1421085	53800954	G	0.1521	0.1283	A	0.2314	1.218
16	rs1558902	53803574	T	0.1521	0.1283	A	0.2314	1.218
17	rs2271534	72350402	A	0.02835	0.0425	G	0.2314	0.6574

21	rs2827312	23631676	A	0.1114	0.09	C	0.2314	1.268
6	rs2234951	52617738	A	0.1392	0.1664	G	0.2315	0.81
19	rs4805825	33414420	A	0.4175	0.3825	G	0.2315	1.157
1	rs6658356	3280253	A	0.06186	0.04667	G	0.2316	1.347
3	rs11128141	69779740	A	0.06186	0.04667	C	0.2316	1.347
6	rs7771335	29448038	G	0.1392	0.1658	A	0.2316	0.8133
6	rs10484547	29452774	C	0.1392	0.1658	G	0.2316	0.8133
6	rs2066951	29454489	C	0.1392	0.1658	A	0.2316	0.8133
9	rs117143733	36169894	G	0.116	0.1408	A	0.2316	0.8004
9	rs59873903	139235526	A	0.2835	0.2529	C	0.2316	1.169
11	rs4505038	45934697	C	0.1392	0.1658	A	0.2316	0.8133
6	rs9471447	40921160	A	0.366	0.4008	G	0.2319	0.8629
12	rs3765044	85438499	A	0.2423	0.2133	G	0.2319	1.179
12	rs17012533	85466723	A	0.2423	0.2133	G	0.2319	1.179
17	rs3744554	10346781	A	0.1328	0.1093	G	0.2319	1.247
1	rs3747965	94342564	C	0.4201	0.385	A	0.232	1.157
10	rs3814226	97920062	A	0.4201	0.385	G	0.232	1.157
2	rs6547705	87044316	G	0.007732	0.0175	A	0.2321	0.4375
2	rs36127492	234066969	G	0.007732	0.0175	C	0.2321	0.4375
3	rs116921702	39228905	A	0.007732	0.0175	G	0.2321	0.4375
4	rs148635969	72316967	A	0.007732	0.0175	G	0.2321	0.4375
5	rs201839213	147661742	A	0.007732	0.0175	C	0.2321	0.4375
6	rs9374435	97681784	G	0.007732	0.0175	A	0.2321	0.4375
7	rs141350740	94996741	G	0.007732	0.0175	A	0.2321	0.4375
7	rs4725617	143097100	A	0.007732	0.0175	G	0.2321	0.4375
9	rs2270889	2811557	A	0.007732	0.0175	G	0.2321	0.4375
11	rs149721746	123810502	G	0.007732	0.0175	A	0.2321	0.4375
12	rs16914640	9822387	G	0.007732	0.0175	C	0.2321	0.4375
13	rs9543104	73307527	A	0.007732	0.0175	G	0.2321	0.4375
14	rs138585435	74409395	A	0.007732	0.0175	G	0.2321	0.4375
16	rs181115838	30976665	G	0.007732	0.0175	A	0.2321	0.4375
17	rs117221419	73832723	C	0.007732	0.0175	A	0.2321	0.4375
3	rs2929349	19975582	G	0.2887	0.3217	A	0.2322	0.8557
17	rs4578702	70441787	A	0.4275	0.3917	C	0.2323	1.16
7	rs6952251	122547130	A	0.4227	0.3875	C	0.2325	1.157
1	rs1058885	110466466	A	0.4201	0.3858	G	0.2326	1.153
4	rs11734306	188973194	C	0.1108	0.09	A	0.2326	1.26
9	rs16909898	98231008	G	0.1108	0.09	A	0.2326	1.26
9	rs75989782	99580834	C	0.1108	0.09	A	0.2326	1.26
19	rs61574510	52520607	A	0.4201	0.3858	G	0.2326	1.153
10	rs2981579	123337335	A	0.3711	0.4067	G	0.2328	0.8611
12	rs61926450	89653020	G	0.3711	0.4067	A	0.2328	0.8611
1	rs12410869	150856153	A	0.4253	0.39	C	0.233	1.157

9	rs4626664	9261737	A	0.4253	0.39	G	0.233	1.157
20	rs6015112	56450431	A	0.1546	0.13	G	0.233	1.224
7	rs2072164	143657427	A	0.1684	0.1962	G	0.2331	0.8298
11	rs7130251	55979266	G	0.2912	0.325	A	0.2331	0.8534
13	rs3843679	21517919	A	0.4227	0.3883	G	0.2331	1.153
21	rs2094881	42848835	G	0.2397	0.2717	A	0.2331	0.8452
1	rs5746026	12253062	A	0.03866	0.055	G	0.2332	0.691
22	rs2269961	30860830	A	0.3428	0.3092	G	0.2333	1.165
19	rs2074789	3613346	A	0.2912	0.3239	G	0.2334	0.8578
8	rs10448108	4809686	A	0.3737	0.4083	G	0.2336	0.8646
1	rs374450	12305286	A	0.2448	0.2158	G	0.2337	1.178
5	rs816736	154271948	G	0.05155	0.03667	A	0.2337	1.428
6	rs7385	76425594	A	0.201	0.2312	C	0.2337	0.8366
6	rs35989216	151789709	G	0.05155	0.03667	A	0.2337	1.428
8	rs7833870	100791156	G	0.05155	0.03667	A	0.2337	1.428
11	rs1965207	6913243	G	0.05155	0.03667	A	0.2337	1.428
15	rs3742985	44158549	G	0.05155	0.03667	A	0.2337	1.428
21	rs1209950	40173528	A	0.05155	0.03667	G	0.2337	1.428
12	rs167769	57503775	A	0.201	0.2317	G	0.2338	0.8345
1	rs3122713	212799870	A	0.4304	0.395	G	0.2339	1.157
2	rs7917	201768238	A	0.01036	0.004174	G	0.2339	2.498
3	rs13066103	72590900	G	0.4304	0.395	A	0.2339	1.157
6	rs9258208	29704545	A	0.201	0.2308	G	0.2339	0.8384
22	rs17854874	24468386	A	0.2113	0.1833	G	0.2339	1.194
1	rs12741973	10638604	A	0.2938	0.3275	G	0.2341	0.8543
16	rs11640794	88494976	A	0.1314	0.1092	C	0.2341	1.235
17	rs4924935	18753870	G	0.1314	0.1092	A	0.2341	1.235
6	rs130072	31112484	A	0.07732	0.06	G	0.2342	1.313
6	rs11540822	31118898	T	0.07732	0.06	A	0.2342	1.313
6	rs2073724	31129707	A	0.07732	0.06	G	0.2342	1.313
14	rs17646457	86089315	A	0.07732	0.06	G	0.2342	1.313
18	rs1426310	28898294	G	0.07732	0.06	A	0.2342	1.313
10	rs1935	64927823	C	0.3789	0.4142	G	0.2343	0.8628
2	rs6542746	108605836	A	0.2938	0.3267	G	0.2344	0.8576
2	rs6437262	242470544	G	0.4304	0.3958	A	0.2344	1.153
4	rs36097019	152201053	A	0.2938	0.3267	G	0.2344	0.8576
4	rs13126069	152212486	A	0.2938	0.3267	G	0.2344	0.8576
16	rs2967334	82273152	G	0.4304	0.3958	A	0.2344	1.153
1	rs145116277	986963	A	0.01031	0.004167	G	0.2345	2.49
1	rs148658404	33999455	A	0.01031	0.004167	G	0.2345	2.49
1	rs35352891	45797401	A	0.01031	0.004167	G	0.2345	2.49
1	rs202171165	110282909	G	0.01031	0.004167	A	0.2345	2.49
1	rs199998827	183920187	G	0.01031	0.004167	A	0.2345	2.49

1	rs139658488	201177415	A	0.01031	0.004167	G	0.2345	2.49
2	rs16867256	10101468	G	0.01031	0.004167	A	0.2345	2.49
2	rs142759452	25022589	A	0.01031	0.004167	C	0.2345	2.49
2	rs113621793	184025845	G	0.01031	0.004167	A	0.2345	2.49
2	rs201567759	231120239	A	0.01031	0.004167	G	0.2345	2.49
2	rs114751344	242035520	G	0.01031	0.004167	C	0.2345	2.49
3	rs151205891	108396420	A	0.01031	0.004167	G	0.2345	2.49
3	rs35156360	123451932	A	0.01031	0.004167	G	0.2345	2.49
3	rs76174573	183521887	A	0.01031	0.004167	C	0.2345	2.49
3	rs140350328	186512517	A	0.01031	0.004167	G	0.2345	2.49
4	rs75022435	20706388	G	0.01031	0.004167	A	0.2345	2.49
4	rs74492021	20709441	A	0.01031	0.004167	G	0.2345	2.49
4	rs73224221	64961101	C	0.01031	0.004167	A	0.2345	2.49
4	rs117615730	153896380	A	0.01031	0.004167	G	0.2345	2.49
5	rs3733689	140572156	A	0.01031	0.004167	G	0.2345	2.49
6	rs3130050	31618761	G	0.01031	0.004167	A	0.2345	2.49
6	rs145866895	35208974	A	0.01031	0.004167	G	0.2345	2.49
6	rs138701301	101215022	G	0.01031	0.004167	A	0.2345	2.49
6	rs115151149	111280027	A	0.01031	0.004167	G	0.2345	2.49
6	rs200320051	139233939	G	0.01031	0.004167	A	0.2345	2.49
6	rs139937718	161026078	A	0.01031	0.004167	G	0.2345	2.49
6	rs188414778	170627191	G	0.01031	0.004167	A	0.2345	2.49
7	rs139311512	12376689	G	0.01031	0.004167	A	0.2345	2.49
7	rs150761907	47870905	A	0.01031	0.004167	C	0.2345	2.49
7	rs199983945	131082101	G	0.01031	0.004167	A	0.2345	2.49
8	rs185916745	104438333	G	0.01031	0.004167	A	0.2345	2.49
8	rs139817759	145135175	A	0.01031	0.004167	G	0.2345	2.49
9	rs143671666	37304309	A	0.01031	0.004167	G	0.2345	2.49
9	rs200014240	130941557	A	0.01031	0.004167	G	0.2345	2.49
9	rs184547222	130941691	C	0.01031	0.004167	A	0.2345	2.49
10	rs201351111	118427770	A	0.01031	0.004167	G	0.2345	2.49
11	rs200292193	19954772	A	0.01031	0.004167	G	0.2345	2.49
11	rs201136391	64361124	A	0.01031	0.004167	G	0.2345	2.49
11	rs190635740	112064292	A	0.01031	0.004167	G	0.2345	2.49
11	rs149084200	119291594	A	0.01031	0.004167	G	0.2345	2.49
11	rs147530219	126162698	A	0.01031	0.004167	G	0.2345	2.49
12	rs28930670	46320944	A	0.01031	0.004167	G	0.2345	2.49
12	rs184816447	109613826	G	0.01031	0.004167	A	0.2345	2.49
14	rs200366546	23344800	G	0.01031	0.004167	A	0.2345	2.49
14	rs202171930	45633721	A	0.01031	0.004167	G	0.2345	2.49
14	rs12881869	50923249	A	0.01031	0.004167	G	0.2345	2.49
15	rs199549867	45434195	A	0.01031	0.004167	T	0.2345	2.49
16	rs74869205	14234477	A	0.01031	0.004167	G	0.2345	2.49

16	rs184459807	27752087	C	0.01031	0.004167	G	0.2345	2.49
17	rs149945889	48184448	A	0.01031	0.004167	G	0.2345	2.49
18	rs139280277	50929215	A	0.01031	0.004167	G	0.2345	2.49
19	rs7247284	12921186	G	0.01031	0.004167	A	0.2345	2.49
22	rs142715413	38541604	A	0.01031	0.004167	C	0.2345	2.49
22	rs143994992	51064489	A	0.01031	0.004167	G	0.2345	2.49
1	rs5744174	223284528	G	0.1804	0.155	A	0.2346	1.2
5	rs634501	180218668	A	0.433	0.3982	G	0.2346	1.154
11	rs7570	66610645	C	0.1804	0.155	G	0.2346	1.2
16	rs2301770	20802172	A	0.1804	0.155	G	0.2346	1.2
1	rs141394572	18149645	A	0.01031	0.004174	G	0.2347	2.485
1	rs151153383	44685926	A	0.01031	0.004174	G	0.2347	2.485
6	rs16894945	34824107	C	0.2912	0.2592	A	0.2347	1.175
10	rs11013093	18518934	A	0.2912	0.2592	G	0.2347	1.175
17	rs34379910	79612633	G	0.2912	0.2592	A	0.2347	1.175
19	rs118046282	15567047	A	0.01031	0.004174	G	0.2347	2.485
19	rs36070283	18876258	A	0.01031	0.004174	G	0.2347	2.485
4	rs6852535	123478716	A	0.3454	0.3125	G	0.2348	1.161
6	rs1591830	150651900	G	0.3814	0.4167	A	0.2348	0.8633
4	rs3214045	56874517	C	0.1546	0.1308	A	0.2349	1.215
5	rs2234233	9629529	A	0.1546	0.1308	G	0.2349	1.215
8	rs11991695	119691074	C	0.1546	0.1308	A	0.2349	1.215
2	rs72648953	179593503	A	0.01031	0.004181	C	0.235	2.481
10	rs61751507	101829514	A	0.03141	0.02	G	0.2351	1.589
3	rs544043	146186189	C	0.3814	0.4158	A	0.2352	0.8663
7	rs7789768	65938980	A	0.3479	0.3142	C	0.2352	1.165
2	rs13387042	217905832	A	0.0799	0.1008	G	0.2353	0.7743
2	rs192246394	218682785	A	0.01031	0.004188	G	0.2353	2.477
2	rs140202838	241439537	A	0.02062	0.03333	G	0.2353	0.6105
2	rs141615023	241530242	A	0.01031	0.004188	G	0.2353	2.477
2	rs117755329	242716387	A	0.02062	0.03333	G	0.2353	0.6105
4	rs1051881	122737965	G	0.02062	0.03333	C	0.2353	0.6105
6	rs2153157	10897488	G	0.3454	0.313	A	0.2353	1.158
8	rs75295956	142500345	A	0.2113	0.1842	G	0.2353	1.187
11	rs150237829	9005491	A	0.02062	0.03333	G	0.2353	0.6105
11	rs2276295	62402335	A	0.02062	0.03333	G	0.2353	0.6105
19	rs143660467	40433397	A	0.02062	0.03333	C	0.2353	0.6105
2	rs11123953	103593540	G	0.09794	0.1208	A	0.2354	0.79
4	rs6535830	152900692	G	0.2964	0.3292	A	0.2354	0.8585
8	rs2272714	146225112	A	0.2964	0.3292	G	0.2354	0.8585
10	rs4751995	118397884	G	0.2912	0.26	A	0.2354	1.17
10	rs10885997	118397971	G	0.2912	0.26	A	0.2354	1.17
14	rs1953233	65573162	G	0.2912	0.26	A	0.2354	1.17

16	rs34665498	337691	A	0.09794	0.1208	G	0.2354	0.79
19	rs3745298	49658367	A	0.2964	0.3292	G	0.2354	0.8585
22	rs9614402	44645332	A	0.09794	0.1208	G	0.2354	0.79
8	rs36041430	24199218	A	0.09794	0.1217	C	0.2355	0.7838
14	rs17128572	93263982	C	0.1701	0.1992	G	0.2355	0.8242
22	rs2272843	50599466	A	0.1701	0.1992	C	0.2355	0.8242
9	rs10901333	133927878	G	0.4381	0.4033	A	0.2356	1.154
10	rs75363119	68857397	C	0.1186	0.1442	A	0.2356	0.7985
11	rs2847476	114191016	G	0.4381	0.4033	A	0.2356	1.154
1	rs4949526	30432219	A	0.2036	0.2342	G	0.2357	0.8361
10	rs1885434	115350597	A	0.3479	0.315	G	0.2357	1.16
3	rs16827497	130134492	G	0.183	0.1567	A	0.2358	1.206
13	rs9548119	38531581	A	0.2036	0.2333	T	0.2358	0.84
20	rs2425163	34432670	G	0.183	0.1567	A	0.2358	1.206
11	rs1356428	114576626	A	0.299	0.3322	T	0.236	0.8572
1	rs11211199	46287869	A	0.3505	0.3167	G	0.2361	1.165
7	rs2191349	15064309	C	0.299	0.3325	A	0.2361	0.8561
17	rs1003645	34340284	G	0.3892	0.425	A	0.2361	0.862
22	rs2238743	19049602	C	0.3866	0.4208	A	0.2362	0.8674
2	rs11886868	60720246	A	0.02062	0.03417	G	0.2363	0.5951
2	rs10172646	60720757	A	0.02062	0.03417	G	0.2363	0.5951
3	rs56890907	196673831	G	0.02062	0.03417	A	0.2363	0.5951
6	rs749005	6283666	A	0.02062	0.03417	C	0.2363	0.5951
7	rs12112370	157880211	G	0.02083	0.03333	A	0.2363	0.617
8	rs78122695	146156921	C	0.02062	0.03417	A	0.2363	0.5951
14	rs138689053	64519978	G	0.02062	0.03417	A	0.2363	0.5951
14	rs116914994	74971769	A	0.02062	0.03417	G	0.2363	0.5951
8	rs2979531	30383013	A	0.4433	0.4083	G	0.2364	1.154
19	rs1045361	10692000	A	0.299	0.3317	G	0.2364	0.8594
6	rs1116221	30071330	A	0.134	0.1108	G	0.2365	1.242
8	rs2923419	42466416	G	0.134	0.1108	A	0.2365	1.242
16	rs2070203	70303580	A	0.3918	0.4275	G	0.2365	0.8625
3	rs6439334	131740655	A	0.3505	0.3175	G	0.2366	1.16
1	rs2307130	100316589	A	0.4459	0.4108	G	0.2367	1.154
11	rs7927138	7727886	A	0.4407	0.4067	G	0.2367	1.15
17	rs4561518	51901643	G	0.2938	0.2625	A	0.2367	1.169
17	rs4561519	51901703	C	0.2938	0.2625	G	0.2367	1.169
19	rs157580	45395266	A	0.4459	0.4108	G	0.2367	1.154
3	rs2255703	129293256	G	0.4485	0.4133	A	0.2371	1.154
3	rs266717	186530484	A	0.3943	0.4292	G	0.2371	0.866
10	rs524295	97348734	A	0.3943	0.4292	G	0.2371	0.866
17	rs2270379	55356209	G	0.4485	0.4133	A	0.2371	1.154
1	rs3789699	95363001	G	0.06443	0.04833	A	0.2372	1.356

5	rs146701102	172396450	A	0.02062	0.03463	G	0.2372	0.5869
4	rs11724609	14745825	A	0.4459	0.4117	G	0.2373	1.15
6	rs9383583	150212003	G	0.4459	0.4117	A	0.2373	1.15
9	rs3739609	36169598	G	0.3969	0.4325	A	0.2373	0.8635
15	rs11072930	80910857	A	0.3015	0.3342	G	0.2374	0.8602
15	rs1655455	94858686	G	0.451	0.4158	A	0.2374	1.154
19	rs4801478	57909872	G	0.183	0.1575	A	0.2374	1.198
3	rs4684746	10966588	G	0.3531	0.32	A	0.2375	1.16
4	rs4690798	165926586	G	0.3531	0.32	A	0.2375	1.16
8	rs10505630	135796966	G	0.3531	0.32	A	0.2375	1.16
1	rs6699528	95140173	C	0.2062	0.2367	A	0.2376	0.8378
1	rs3768593	183212548	C	0.4485	0.4142	A	0.2377	1.15
2	rs55999472	135744469	G	0.0232	0.01333	A	0.2377	1.757
2	rs28497362	231248261	A	0.1443	0.1708	G	0.2377	0.8187
3	rs3814404	113003262	A	0.0232	0.01333	G	0.2377	1.757
5	rs184201084	86675592	A	0.0232	0.01333	C	0.2377	1.757
6	rs9475487	55825851	G	0.0232	0.01333	A	0.2377	1.757
7	rs849141	28185091	A	0.0232	0.01333	G	0.2377	1.757
7	rs1708299	28189946	A	0.0232	0.01333	G	0.2377	1.757
7	rs56128139	37934146	G	0.1443	0.1708	A	0.2377	0.8187
8	rs2436654	75840531	A	0.1443	0.1708	G	0.2377	0.8187
8	rs28399585	95893915	G	0.0232	0.01333	A	0.2377	1.757
8	rs138171489	100155375	G	0.0232	0.01333	A	0.2377	1.757
12	rs10842760	26772311	A	0.4485	0.4142	G	0.2377	1.15
14	rs148686027	50244662	G	0.0232	0.01333	A	0.2377	1.757
17	rs11538868	45904420	A	0.0232	0.01333	G	0.2377	1.757
1	rs2275902	79357360	G	0.1727	0.2008	C	0.2378	0.8306
2	rs830998	170133086	A	0.05155	0.06917	C	0.2378	0.7314
3	rs35692900	142548560	G	0.05155	0.07	A	0.2378	0.722
9	rs10867752	84015037	A	0.05155	0.06917	G	0.2378	0.7314
12	rs57971665	80878317	A	0.05155	0.06917	G	0.2378	0.7314
12	rs12579151	80900381	G	0.05155	0.06917	A	0.2378	0.7314
14	rs210381	54107791	G	0.2474	0.2783	A	0.2378	0.8524
14	rs7159300	72883874	A	0.1727	0.2017	G	0.2378	0.8263
17	rs2291447	43212963	A	0.1727	0.2008	C	0.2378	0.8306
1	rs201343862	1277184	A	0.0232	0.01336	G	0.2379	1.754
2	rs17217716	47630353	A	0.03093	0.02	G	0.2379	1.564
6	rs116672033	7229974	C	0.03093	0.02	A	0.2379	1.564
6	rs13191258	30978717	A	0.03093	0.02	G	0.2379	1.564
10	rs4548513	68040325	A	0.3995	0.4342	G	0.2379	0.867
10	rs11200385	123842523	A	0.1134	0.0925	G	0.2379	1.255
11	exm2249501	10019879	A	0.03093	0.02	G	0.2379	1.564
11	rs16907355	10019879	A	0.03093	0.02	G	0.2379	1.564

11	rs78765998	47289906	A	0.03093	0.02	T	0.2379	1.564
12	rs3741466	117465264	A	0.1134	0.0925	G	0.2379	1.255
14	rs146869637	62541988	G	0.03093	0.02	C	0.2379	1.564
15	rs2291166	30008977	C	0.03093	0.02	A	0.2379	1.564
16	rs59706474	840599	A	0.03093	0.02	G	0.2379	1.564
19	rs75670627	47968998	A	0.03093	0.02	G	0.2379	1.564
5	rs509237	112550247	A	0.2526	0.2225	G	0.238	1.181
14	rs11627059	100087364	A	0.3041	0.3383	G	0.238	0.8547
20	rs2426983	59777873	C	0.451	0.4167	A	0.238	1.15
7	rs2228410	36763672	G	0.5	0.465	A	0.2381	1.151
4	rs1430934	116804645	G	0.4536	0.4192	A	0.2383	1.15
6	rs117171552	29364508	G	0.03093	0.02003	A	0.2383	1.561
2	rs2244492	179554305	A	0.134	0.1117	G	0.2384	1.231
9	rs137897809	35674208	G	0.134	0.1117	A	0.2384	1.231
9	rs77984049	35674347	T	0.134	0.1117	A	0.2384	1.231
11	rs11222798	131665483	G	0.4046	0.44	A	0.2384	0.865
19	rs3760776	5839746	A	0.134	0.1117	G	0.2384	1.231
2	rs4407201	130522894	A	0.05155	0.0375	G	0.2385	1.395
5	rs117524265	127800515	G	0.05155	0.0375	A	0.2385	1.395
11	rs11229411	58170342	A	0.2165	0.1883	G	0.2385	1.191
12	rs114167123	125450253	A	0.03109	0.02017	G	0.2385	1.559
14	rs11844594	77843814	G	0.05155	0.0375	A	0.2385	1.395
14	rs36099812	93708759	C	0.05155	0.0375	A	0.2385	1.395
1	rs4129267	154426264	A	0.4072	0.4425	G	0.2387	0.8655
1	rs2228145	154426970	C	0.4072	0.4425	A	0.2387	0.8655
4	rs4336169	145174382	G	0.4072	0.4425	A	0.2387	0.8655
4	rs12502935	177113779	A	0.4072	0.4425	G	0.2387	0.8655
21	rs2832007	30106926	A	0.4046	0.4392	G	0.2387	0.8679
2	rs3100583	232944860	G	0.25	0.2825	A	0.2388	0.8466
3	rs2166799	196547538	A	0.25	0.2825	G	0.2388	0.8466
1	rs111819661	1222958	A	0.0232	0.01347	G	0.2389	1.739
1	rs9662783	199238445	G	0.3067	0.34	A	0.2389	0.8587
17	rs11077994	80404326	C	0.3067	0.34	A	0.2389	0.8587
1	rs12085319	11029478	A	0.4639	0.4283	C	0.239	1.155
11	rs1351696	48382471	A	0.1856	0.1594	G	0.239	1.201
19	rs7246856	57956828	A	0.2526	0.2233	C	0.239	1.175
1	rs904589	1354515	G	0.1211	0.1467	C	0.2392	0.8019
19	rs12151338	44933947	A	0.3557	0.3233	G	0.2392	1.155
17	rs1105353	71426656	A	0.0799	0.06167	C	0.2393	1.321
1	rs1034268	42898843	A	0.4639	0.4292	G	0.2394	1.151
6	rs2076487	29523859	C	0.2088	0.2392	G	0.2394	0.8393
6	rs2076486	29523872	G	0.2088	0.2392	A	0.2394	0.8393
6	rs2076484	29524003	G	0.2088	0.2392	A	0.2394	0.8393

6	rs2272991	29527599	G	0.2088	0.2392	A	0.2394	0.8393
8	rs1058720	42044954	G	0.4639	0.4292	A	0.2394	1.151
8	rs1364705	120224806	A	0.4639	0.4292	G	0.2394	1.151
18	rs8093631	42134697	A	0.4639	0.4292	G	0.2394	1.151
3	rs4855056	181638250	A	0.4665	0.4317	G	0.2396	1.151
6	rs9389917	141707045	G	0.4691	0.4333	A	0.2396	1.155
7	rs6968828	27223563	C	0.4665	0.4317	A	0.2396	1.151
19	rs321937	52869278	C	0.4665	0.4317	G	0.2396	1.151
8	rs2460335	12874138	A	0.4149	0.45	G	0.2397	0.8669
2	rs6729801	183584755	A	0.2552	0.225	G	0.2398	1.18
4	rs2301790	6600012	A	0.3093	0.3433	G	0.2398	0.8564
6	rs9257890	29486908	A	0.2165	0.1892	G	0.2398	1.184
12	rs1129649	6948468	G	0.3093	0.3433	A	0.2398	0.8564
14	rs9646133	71096344	C	0.3093	0.3433	A	0.2398	0.8564
15	rs1048943	75012985	G	0.2552	0.225	A	0.2398	1.18
2	rs10179028	140566297	A	0.4175	0.4533	G	0.2399	0.8644
4	rs10461336	176391372	A	0.1005	0.1233	G	0.2399	0.7943
8	rs7829965	24207438	A	0.1005	0.1233	G	0.2399	0.7943
16	rs2444217	4038387	G	0.4175	0.4533	A	0.2399	0.8644
19	rs2288515	55713535	A	0.4175	0.4533	G	0.2399	0.8644
6	rs402219	160789296	G	0.4201	0.4558	A	0.2401	0.8648
10	rs10733970	58773025	A	0.01562	0.008333	G	0.2401	1.889
22	rs9616915	51117580	G	0.1598	0.135	A	0.2401	1.219
1	rs1133891	75214441	A	0.2526	0.2842	G	0.2402	0.8513
1	rs2276404	158979950	G	0.2526	0.2842	A	0.2402	0.8513
7	rs1858948	20686111	A	0.2526	0.285	C	0.2402	0.8478
8	rs502882	12879099	G	0.2526	0.2842	A	0.2402	0.8513
10	rs7909670	12918764	A	0.4691	0.4348	G	0.2402	1.149
13	rs9318731	25287214	A	0.4149	0.4492	G	0.2402	0.8698
13	rs1359215	37877935	A	0.4149	0.4492	G	0.2402	0.8698
17	rs2293180	25909816	A	0.2526	0.285	G	0.2402	0.8478
10	rs2049587	21616815	A	0.4201	0.455	G	0.2403	0.8677
12	rs2887885	55077399	A	0.4742	0.4392	G	0.2403	1.152
12	rs317689	69727190	G	0.3015	0.2696	A	0.2403	1.17
9	rs321739	28755357	A	0.4227	0.4583	G	0.2404	0.8653
12	rs2075322	31249861	G	0.1598	0.1352	C	0.2404	1.216
1	rs2230679	29475648	A	0.4768	0.4417	C	0.2405	1.152
6	rs1884260	91988031	G	0.4691	0.435	A	0.2405	1.148
7	rs757911	41994837	G	0.4768	0.4417	A	0.2405	1.152
16	rs1800775	56995236	C	0.4768	0.4417	A	0.2405	1.152
19	rs8110452	998126	G	0.4716	0.4374	A	0.2405	1.148
21	rs34191159	30339120	A	0.4716	0.4374	C	0.2405	1.148
3	rs2232350	53899178	G	0.1469	0.1733	A	0.2406	0.8213

6	rs7770407	53447470	G	0.4253	0.4608	A	0.2406	0.8657
8	rs1112331	8534192	A	0.4253	0.4608	C	0.2406	0.8657
14	rs1061040	23242828	A	0.08247	0.1042	G	0.2406	0.773
2	rs2312147	58222928	A	0.2552	0.2258	G	0.2407	1.174
2	rs6735786	103770238	G	0.3119	0.3458	A	0.2407	0.8572
10	rs1329650	93348120	C	0.2552	0.2258	A	0.2407	1.174
17	rs8072888	11490257	G	0.4742	0.4399	A	0.2407	1.148
21	rs61735768	30339234	G	0.4716	0.4375	A	0.2407	1.148
1	rs1553000	187278646	A	0.3119	0.345	G	0.2408	0.8604
9	rs8999	135277341	A	0.3119	0.345	C	0.2408	0.8604
17	rs11654604	34077232	A	0.1366	0.1133	G	0.2408	1.238
3	rs9310592	1982593	A	0.4278	0.4633	G	0.2409	0.8661
4	rs10516523	106625263	A	0.08247	0.1033	C	0.2409	0.78
6	rs2394401	30724430	G	0.08247	0.1033	A	0.2409	0.78
6	rs35517174	167343199	A	0.09536	0.07667	G	0.2409	1.27
9	rs2296957	134401335	G	0.09536	0.07667	A	0.2409	1.27
19	rs17160348	8326866	A	0.09536	0.07667	G	0.2409	1.27
19	rs17160349	8326944	A	0.09536	0.07667	G	0.2409	1.27
7	rs4719989	29872395	C	0.482	0.4467	A	0.241	1.153
3	rs7629490	87241497	A	0.4768	0.4425	G	0.2411	1.148
18	rs2164772	4554426	G	0.4304	0.4658	A	0.2411	0.8665
5	rs3729740	38496637	A	0.2113	0.2417	G	0.2412	0.8409
9	rs4546744	139694521	C	0.2113	0.2417	A	0.2412	0.8409
21	rs1051266	46957794	G	0.4278	0.4625	A	0.2412	0.869
1	rs2275697	205027737	A	0.4871	0.4517	G	0.2413	1.153
1	rs10754697	240453628	A	0.5156	0.4808	G	0.2413	1.149
3	rs2317212	97328284	C	0.433	0.4683	A	0.2413	0.8669
3	rs2229362	187446211	A	0.116	0.09417	G	0.2413	1.262
5	rs62347066	13147070	A	0.433	0.4683	G	0.2413	0.8669
11	rs4938303	116584987	G	0.4794	0.445	A	0.2413	1.148
21	rs162350	44865282	G	0.433	0.4683	A	0.2413	0.8669
6	rs154978	32899296	G	0.4304	0.465	A	0.2414	0.8694
7	rs3173833	150491084	A	0.3634	0.3308	C	0.2414	1.155
9	rs945386	139693596	G	0.2113	0.2408	A	0.2414	0.8447
17	rs1035050	47564012	A	0.2113	0.2408	G	0.2414	0.8447
17	rs6501358	67992704	G	0.2113	0.2408	A	0.2414	0.8447
19	rs200303476	3753816	A	0.01554	0.008333	G	0.2414	1.879
1	rs17021090	213781198	A	0.4897	0.4542	G	0.2415	1.153
4	rs2301796	6594947	A	0.3144	0.3483	C	0.2415	0.858
5	rs6451180	35118848	G	0.2577	0.2275	A	0.2415	1.179
7	rs3779234	35709842	A	0.2577	0.2275	G	0.2415	1.179
3	rs34624208	75552718	G	0.4381	0.4733	A	0.2416	0.8678
15	rs6078	58833993	A	0.3015	0.2708	G	0.2416	1.162

15	rs9652490	77963887	G	0.2552	0.2867	A	0.2416	0.8524
20	rs6051520	351944	C	0.2552	0.2867	A	0.2416	0.8524
1	rs704326	181759657	A	0.433	0.4675	G	0.2417	0.8698
6	rs16893917	28269407	A	0.2688	0.2387	G	0.2417	1.173
9	rs2297002	88651370	G	0.4381	0.4733	A	0.2417	0.8677
16	rs3743590	11930337	A	0.3634	0.3308	C	0.2417	1.155
19	rs2302188	42085873	A	0.4381	0.4733	G	0.2417	0.8677
3	rs2305397	12857493	A	0.1598	0.1358	G	0.2418	1.21
11	rs10750836	68815523	G	0.4948	0.4592	A	0.2418	1.154
13	rs1012053	42653437	A	0.4948	0.4592	C	0.2418	1.154
3	rs11543283	125786852	A	0.2191	0.1917	G	0.2419	1.183
3	rs13062144	166522847	A	0.2191	0.1917	G	0.2419	1.183
6	rs7772982	29341007	G	0.2191	0.1917	A	0.2419	1.183
11	rs12279895	58169996	G	0.2191	0.1917	A	0.2419	1.183
11	rs11229410	58170291	G	0.2191	0.1917	A	0.2419	1.183
11	rs12280114	58170374	G	0.2191	0.1917	A	0.2419	1.183
11	rs11229413	58170738	G	0.2191	0.1917	A	0.2419	1.183
16	rs2719710	10769958	A	0.2191	0.1917	G	0.2419	1.183
3	rs4390941	95795276	A	0.4948	0.4599	C	0.242	1.15
11	rs10500991	24849566	A	0.06443	0.04917	G	0.242	1.332
11	rs76353556	65361309	G	0.06443	0.04917	A	0.242	1.332
12	rs11175593	40601940	A	0.06443	0.04917	G	0.242	1.332
20	rs208817	37492316	G	0.4923	0.4575	A	0.242	1.15
8	rs2442572	6454559	C	0.4433	0.4783	A	0.2421	0.8684
16	rs75559202	78458915	G	0.06701	0.08667	C	0.2422	0.7569
6	rs2252816	97058574	A	0.5026	0.4674	G	0.2423	1.151
10	rs2395289	77284374	G	0.451	0.4867	A	0.2423	0.8666
14	rs1135641	23299135	C	0.4459	0.4808	A	0.2423	0.8688
1	rs668556	39914363	C	0.317	0.3508	G	0.2424	0.8588
1	rs9865	46827456	G	0.317	0.3508	A	0.2424	0.8588
2	rs2227982	242793433	A	0.4407	0.475	G	0.2424	0.871
4	rs4698797	110773067	G	0.4485	0.4833	A	0.2424	0.8692
6	rs6926543	111935682	A	0.4407	0.475	C	0.2424	0.871
8	rs1160555	13401972	A	0.4407	0.475	G	0.2424	0.871
21	rs2838622	46086377	A	0.5026	0.4675	G	0.2424	1.151
1	rs7514972	231262876	G	0.4562	0.4917	A	0.2425	0.8673
6	rs3869109	31184196	G	0.5077	0.4725	A	0.2425	1.151
10	rs1801231	17024503	G	0.4562	0.4917	A	0.2425	0.8673
12	rs1135816	9750669	A	0.5129	0.4775	G	0.2425	1.152
1	rs2297881	10397567	G	0.06701	0.0875	A	0.2426	0.749
5	rs32447	15117438	G	0.3686	0.335	A	0.2426	1.159
6	rs6457721	33107751	C	0.451	0.4858	A	0.2426	0.8695
6	rs721394	33117818	A	0.451	0.4858	G	0.2426	0.8695

9	rs4962153	136323754	A	0.06701	0.0875	G	0.2426	0.749
11	rs2420525	34297211	A	0.4433	0.4775	G	0.2426	0.8713
16	rs1805010	27356203	A	0.451	0.4858	G	0.2426	0.8695
16	rs10871290	74472696	G	0.3686	0.335	A	0.2426	1.159
17	rs12948945	75038439	A	0.5103	0.475	C	0.2426	1.152
18	rs2576037	44585420	G	0.4588	0.4942	A	0.2426	0.8676
19	rs1059369	18497141	A	0.317	0.35	T	0.2426	0.862
1	rs11209552	70297993	A	0.5155	0.48	G	0.2427	1.152
1	rs5745549	76378502	A	0.01546	0.008333	G	0.2427	1.869
1	rs118010078	197070906	A	0.01546	0.008333	G	0.2427	1.869
1	rs151244742	220338086	G	0.01546	0.008333	A	0.2427	1.869
1	rs78828796	225592364	A	0.01546	0.008333	G	0.2427	1.869
1	rs138506576	235897950	C	0.01546	0.008333	A	0.2427	1.869
1	rs3768067	235973750	G	0.01546	0.008333	A	0.2427	1.869
2	rs148827158	79253283	C	0.01546	0.008333	A	0.2427	1.869
2	rs45507693	113890284	A	0.01546	0.008333	G	0.2427	1.869
2	rs2304705	143715265	A	0.01546	0.008333	G	0.2427	1.869
2	rs77826191	152548874	A	0.01546	0.008333	T	0.2427	1.869
2	rs143360352	230456445	A	0.01546	0.008333	G	0.2427	1.869
2	rs147943274	242509693	A	0.01546	0.008333	G	0.2427	1.869
3	rs1375403	159073317	G	0.518	0.4825	A	0.2427	1.153
4	rs4643790	154709854	A	0.01546	0.008333	G	0.2427	1.869
4	rs117270501	175413228	G	0.01546	0.008333	A	0.2427	1.869
5	rs78818665	43700321	G	0.01546	0.008333	A	0.2427	1.869
6	rs118021320	96988480	A	0.01546	0.008333	G	0.2427	1.869
6	rs636252	117157774	A	0.5155	0.48	G	0.2427	1.152
7	rs200555224	15405216	A	0.01546	0.008333	G	0.2427	1.869
7	rs75882660	82595427	G	0.01546	0.008333	A	0.2427	1.869
7	rs149822831	99170144	G	0.01546	0.008333	A	0.2427	1.869
8	rs74596676	21982991	G	0.01546	0.008333	A	0.2427	1.869
8	rs73549523	21984765	A	0.01546	0.008333	G	0.2427	1.869
9	rs201821837	139925446	A	0.01546	0.008333	G	0.2427	1.869
10	rs143186590	26513539	G	0.01546	0.008333	A	0.2427	1.869
10	rs117515566	28224128	A	0.01546	0.008333	C	0.2427	1.869
10	rs147221652	28343067	G	0.01546	0.008333	A	0.2427	1.869
10	rs4359141	99554180	A	0.5155	0.48	G	0.2427	1.152
12	rs117016551	1005486	A	0.01546	0.008333	G	0.2427	1.869
12	rs144560690	53233704	A	0.01546	0.008333	G	0.2427	1.869
12	rs145269194	69140381	A	0.01546	0.008333	G	0.2427	1.869
13	rs79109564	114535698	A	0.01546	0.008333	G	0.2427	1.869
14	rs4982051	20869163	A	0.01546	0.008333	G	0.2427	1.869
14	rs928108	57170474	G	0.5155	0.48	A	0.2427	1.152
17	rs117892584	1579300	A	0.01546	0.008333	G	0.2427	1.869

17	rs200275094	35946626	A	0.01546	0.008333	G	0.2427	1.869
17	rs151176313	72278080	A	0.01546	0.008333	G	0.2427	1.869
19	rs28359762	2269728	A	0.01546	0.008333	G	0.2427	1.869
19	rs16979670	9452879	A	0.01546	0.008333	C	0.2427	1.869
19	rs147456906	9453655	A	0.01546	0.008333	G	0.2427	1.869
19	rs185662873	52537751	A	0.01546	0.008333	C	0.2427	1.869
19	rs146368839	54301639	A	0.01546	0.008333	G	0.2427	1.869
3	rs6775476	106941033	G	0.5206	0.485	A	0.2428	1.153
5	rs7728043	5897694	G	0.5232	0.4875	A	0.2428	1.154
5	rs379707	39119723	A	0.4974	0.4633	C	0.2428	1.146
6	rs2501720	90899561	G	0.5206	0.485	A	0.2428	1.153
8	rs1106334	71012811	A	0.5258	0.49	G	0.2428	1.154
9	rs7048618	109132446	A	0.2577	0.29	G	0.2428	0.8501
9	rs7874142	137704782	G	0.4665	0.5017	A	0.2428	0.8686
11	rs1873059	45540603	A	0.5258	0.49	G	0.2428	1.154
1	rs12045440	19765518	C	0.2577	0.2892	A	0.2429	0.8535
2	rs4668909	15607842	G	0.2216	0.1933	A	0.2429	1.188
2	rs13029846	15674686	G	0.2216	0.1933	A	0.2429	1.188
2	rs56201324	182840114	G	0.4691	0.5042	A	0.2429	0.8689
2	rs28381984	228135631	A	0.5026	0.4683	G	0.2429	1.147
7	rs1880738	48285485	G	0.4562	0.4908	A	0.2429	0.8702
7	rs7789541	150177295	A	0.4562	0.4908	G	0.2429	0.8702
17	rs9908587	63090555	A	0.4588	0.4933	G	0.2429	0.8706
2	rs2042995	179558366	A	0.4588	0.4933	G	0.243	0.8705
3	exm2255837	172046861	A	0.5129	0.4783	G	0.243	1.148
3	rs2270568	172046861	A	0.5129	0.4783	G	0.243	1.148
4	rs2070025	155511824	G	0.09794	0.07789	A	0.243	1.285
5	rs10066478	164370690	A	0.4588	0.4933	G	0.243	0.8705
6	rs1265759	32322393	G	0.4588	0.4933	A	0.243	0.8705
6	rs2395150	32326045	G	0.4588	0.4933	A	0.243	0.8705
6	rs3129923	32333195	A	0.4588	0.4933	G	0.243	0.8705
6	rs2295837	110064928	A	0.2139	0.2442	T	0.243	0.8424
8	rs4876662	117556270	G	0.5232	0.4883	A	0.243	1.15
15	rs1820357	61020808	C	0.5206	0.4858	A	0.243	1.149
15	rs7164592	73276931	A	0.5232	0.4883	C	0.243	1.15
21	rs2833031	32090908	G	0.5077	0.4733	A	0.243	1.148
4	rs2709828	152355268	A	0.3196	0.3533	G	0.2432	0.8596
5	rs2896103	13764419	A	0.116	0.095	G	0.2432	1.25
12	rs10849033	4425122	G	0.4639	0.4983	A	0.2432	0.8712
13	rs12015	43597865	G	0.116	0.095	A	0.2432	1.25
14	rs1045644	31355096	C	0.3686	0.3358	G	0.2432	1.154
17	rs74006007	79163758	A	0.116	0.095	G	0.2432	1.25
1	rs1340117	208830611	C	0.2603	0.23	A	0.2433	1.178

5	rs292998	58032485	G	0.2139	0.2433	A	0.2433	0.8462
6	rs2116264	32984788	G	0.2603	0.23	A	0.2433	1.178
6	rs4714759	44115169	A	0.2139	0.2433	G	0.2433	0.8462
3	rs3617	52833805	C	0.4716	0.5058	A	0.2435	0.8721
5	rs244903	167913510	G	0.1624	0.1375	A	0.2435	1.216
6	rs78648104	50683009	G	0.1495	0.1767	A	0.2435	0.8191
10	rs17417407	95931087	A	0.1624	0.1375	C	0.2435	1.216
14	rs1045002	55818517	T	0.3196	0.3525	A	0.2435	0.8628
14	rs3742569	55818706	G	0.3196	0.3525	A	0.2435	0.8628
15	rs4533267	100786271	A	0.1624	0.1375	G	0.2435	1.216
21	rs11700636	42896828	G	0.4716	0.5058	A	0.2435	0.8721
5	rs616235	76128521	G	0.1624	0.1377	A	0.2438	1.214
6	rs2647012	32664458	A	0.2603	0.2308	G	0.244	1.173
6	rs2268657	39020542	A	0.3067	0.2758	G	0.244	1.161
20	rs4925386	60921044	A	0.2603	0.2308	G	0.244	1.173
1	rs687848	39919234	G	0.3222	0.3558	A	0.2441	0.8604
11	rs2226633	132320394	A	0.2216	0.1942	G	0.2441	1.182
17	rs1126690	78091405	G	0.3222	0.3558	A	0.2441	0.8604
1	rs6027	169483561	G	0.04124	0.0575	A	0.2442	0.705
11	rs2241002	60886913	A	0.04124	0.0575	G	0.2442	0.705
2	rs4667596	170070172	A	0.09794	0.07833	G	0.2443	1.277
17	rs931196	33288882	A	0.09794	0.07833	C	0.2443	1.277
17	rs2072446	47587819	A	0.09794	0.07833	G	0.2443	1.277
1	rs587404	39908506	A	0.3222	0.355	G	0.2444	0.8635
1	rs998532	117617705	A	0.3093	0.2775	G	0.2444	1.166
3	rs9841544	75202547	G	0.3222	0.355	A	0.2444	0.8635
12	rs10745954	103483094	A	0.1804	0.2083	G	0.2448	0.8365
17	rs887230	32904586	G	0.2242	0.1958	A	0.2451	1.187
2	rs454305	70736219	G	0.3093	0.2783	A	0.2452	1.161
17	rs6504712	49446154	G	0.3093	0.2783	A	0.2452	1.161
1	rs2228564	40773149	A	0.329	0.362	G	0.2454	0.8641
4	rs6838769	190915650	G	0.329	0.362	A	0.2454	0.8641
6	rs1043618	31783507	G	0.2629	0.295	C	0.2454	0.8523
1	rs785467	46521559	T	0.3119	0.28	A	0.2456	1.165
2	exm177311	25064193	G	0.3119	0.28	A	0.2456	1.165
2	rs2241759	25064193	G	0.3119	0.28	A	0.2456	1.165
2	rs3796033	218713282	A	0.2629	0.2942	G	0.2456	0.8557
1	rs80127542	169391135	A	0.05155	0.03833	G	0.2457	1.363
10	rs2395528	80100861	A	0.2629	0.2333	G	0.2457	1.172
11	rs2943511	1272245	A	0.05412	0.07215	G	0.2457	0.7359
6	rs3025643	29569955	A	0.08505	0.1067	G	0.246	0.7785
12	rs114378235	51639899	G	0.116	0.09583	A	0.246	1.238
6	rs7744001	32626086	A	0.3273	0.36	G	0.2461	0.865

6	rs7761731	46563817	A	0.3273	0.36	T	0.2461	0.865
8	rs4735246	94895433	A	0.3273	0.36	G	0.2461	0.865
9	rs2418135	113901309	G	0.3273	0.36	A	0.2461	0.865
6	rs1322219	7539954	A	0.3789	0.3458	G	0.2462	1.154
6	rs937057	46977458	G	0.2242	0.1967	A	0.2462	1.181
2	rs10497844	200813126	G	0.1521	0.1792	A	0.2463	0.8216
1	rs2275866	201351421	A	0.05412	0.07167	T	0.2464	0.7412
12	rs12424086	66364509	G	0.05412	0.07167	A	0.2464	0.7412
12	rs11836131	99586180	A	0.1521	0.1783	G	0.2464	0.8263
19	rs2562473	21720803	A	0.05412	0.07167	G	0.2464	0.7412
12	rs9788233	3806147	G	0.2191	0.2492	A	0.2465	0.8453
12	rs5020531	11338957	A	0.2191	0.2492	G	0.2465	0.8453
12	rs35969491	11339020	T	0.2191	0.2492	A	0.2465	0.8453
9	rs35969327	27168571	G	0.08505	0.1075	A	0.2466	0.7718
19	rs25487	44055726	A	0.2655	0.235	G	0.2466	1.176
20	rs6020624	49214156	A	0.2655	0.235	G	0.2466	1.176
4	rs61758879	86852097	A	0.1392	0.1167	C	0.2467	1.224
8	rs3750208	144621035	A	0.1392	0.1167	G	0.2467	1.224
8	rs3750206	144621091	A	0.1392	0.1167	T	0.2467	1.224
17	rs61740308	5042715	A	0.1392	0.1167	G	0.2467	1.224
17	rs61745111	5042837	A	0.1392	0.1167	G	0.2467	1.224
20	rs2295764	31025163	A	0.3144	0.2825	G	0.2468	1.165
3	rs6795970	38766675	A	0.1649	0.14	G	0.2469	1.213
14	rs1303	94844843	C	0.2191	0.2483	A	0.2469	0.8491
16	rs4347621	51683901	G	0.1649	0.14	A	0.2469	1.213
16	rs9930506	53830465	G	0.1933	0.1667	A	0.2469	1.198
9	rs520198	124390320	G	0.3325	0.3667	A	0.2472	0.8603
19	rs401502	18180413	C	0.3325	0.3667	G	0.2472	0.8603
19	rs375947	18180451	G	0.3325	0.3667	A	0.2472	0.8603
22	rs80575	36539754	A	0.3325	0.3667	C	0.2472	0.8603
4	rs17013181	88732763	G	0.2655	0.2358	A	0.2473	1.171
6	rs2394180	29805199	G	0.3325	0.3658	A	0.2473	0.8634
12	rs17124220	50369305	A	0.09794	0.07917	G	0.2473	1.263
14	rs17122693	51084235	G	0.09794	0.07917	A	0.2473	1.263
15	rs1356410	42434837	A	0.09794	0.07917	G	0.2473	1.263
17	rs12453124	38938316	A	0.2268	0.1983	G	0.2473	1.186
1	rs7535306	152883680	A	0.3368	0.3708	G	0.2475	0.8616
11	rs6682	870446	G	0.3144	0.2833	A	0.2475	1.16
1	rs7550381	156495001	A	0.384	0.3508	G	0.2476	1.154
1	rs35737297	217604654	G	0.06701	0.05083	A	0.2476	1.341
3	rs112115496	98503792	G	0.04124	0.02917	A	0.2476	1.432
3	rs28489284	98512540	A	0.04124	0.02917	G	0.2476	1.432
6	rs62385118	4996543	A	0.04124	0.02917	G	0.2476	1.432

10	rs3006843	26636925	C	0.384	0.3508	A	0.2476	1.154
10	rs10887548	87772933	G	0.384	0.3508	A	0.2476	1.154
10	rs17112809	99225846	A	0.04124	0.02917	G	0.2476	1.432
12	rs150140900	7457072	A	0.04124	0.02917	G	0.2476	1.432
17	rs3795244	30692396	A	0.04124	0.02917	C	0.2476	1.432
20	rs116876380	61444523	A	0.04124	0.02917	G	0.2476	1.432
5	rs257742	15853255	A	0.3351	0.3692	C	0.2479	0.861
19	rs1127307	14589378	A	0.3351	0.3692	G	0.2479	0.861
19	rs12979328	49424381	A	0.3351	0.3692	C	0.2479	0.861
6	rs4869782	150303241	A	0.268	0.2992	G	0.2481	0.8579
14	rs1954231	47428100	G	0.1933	0.1675	A	0.2482	1.191
14	rs8015875	101533288	A	0.1933	0.1675	G	0.2482	1.191
1	rs273259	79093818	A	0.2268	0.1992	G	0.2483	1.179
8	rs7830613	9649769	A	0.384	0.3517	G	0.2484	1.149
9	rs10817479	116085423	A	0.1649	0.1408	G	0.2484	1.205
16	rs3743601	75590092	G	0.2655	0.2367	C	0.2485	1.166
6	rs9381472	46658905	G	0.3376	0.3717	C	0.2487	0.8617
19	rs2279008	17283303	G	0.3376	0.3717	A	0.2487	0.8617
18	rs12454733	68108228	G	0.3376	0.3708	A	0.2488	0.8648
21	rs2226548	31802768	A	0.3376	0.3706	G	0.2488	0.8656
3	rs28365795	122259640	G	0.06959	0.08917	A	0.2489	0.764
14	rs61744857	21546354	A	0.06959	0.08917	G	0.2489	0.764
3	rs2118170	100594640	A	0.3866	0.3542	G	0.249	1.149
9	rs1105223	126128211	G	0.3892	0.3558	A	0.249	1.153
14	rs179075	30792163	G	0.3866	0.3542	A	0.249	1.149
2	rs35941588	160673519	A	0.03093	0.02083	G	0.2491	1.5
2	rs76355956	239157708	A	0.0232	0.01417	G	0.2491	1.652
3	rs3732978	9027262	A	0.03093	0.02083	G	0.2491	1.5
3	rs79604232	57494251	G	0.03093	0.02083	A	0.2491	1.5
3	rs145814348	66431968	A	0.03093	0.02083	G	0.2491	1.5
4	rs11549516	3526645	A	0.0232	0.01417	G	0.2491	1.652
4	rs315675	25363901	A	0.1546	0.1817	T	0.2491	0.824
5	rs25901	16673975	G	0.1554	0.1825	C	0.2491	0.8244
5	rs150188040	70754475	A	0.0232	0.01417	T	0.2491	1.652
5	rs141398694	70849136	C	0.0232	0.01417	A	0.2491	1.652
5	rs2053028	150901613	A	0.03093	0.02083	G	0.2491	1.5
6	rs2789066	121100137	A	0.0232	0.01417	G	0.2491	1.652
7	rs74959149	104752798	A	0.03093	0.02083	G	0.2491	1.5
10	rs7905537	34231275	C	0.2706	0.3025	A	0.2491	0.8555
10	rs11191737	105344451	A	0.1546	0.1817	G	0.2491	0.824
11	rs186237230	102661435	G	0.03093	0.02083	A	0.2491	1.5
12	rs147735206	20893259	G	0.0232	0.01417	A	0.2491	1.652
14	rs77170608	102964008	A	0.03093	0.02083	G	0.2491	1.5

15	rs2739804	25494501	G	0.0232	0.01417	A	0.2491	1.652
17	rs73250854	8243598	A	0.03093	0.02083	G	0.2491	1.5
1	rs75161007	186141181	C	0.06959	0.09	G	0.2492	0.7562
7	rs11978267	50466304	G	0.1418	0.1183	A	0.2492	1.231
10	rs2289337	122649482	G	0.06959	0.09	A	0.2492	0.7562
11	rs2508490	120099679	A	0.1418	0.1183	G	0.2492	1.231
12	rs58920821	55863537	A	0.06959	0.09	G	0.2492	0.7562
19	rs2336573	8367709	A	0.06959	0.09	G	0.2492	0.7562
17	rs72483216	33771996	A	0.2294	0.2008	G	0.2494	1.184
4	rs7657799	105375423	C	0.1289	0.1542	A	0.2496	0.8116
6	rs9469220	32658310	G	0.3918	0.3583	A	0.2496	1.153
7	rs6965444	63588181	G	0.3402	0.3733	A	0.2496	0.8655
10	rs2475298	121679013	A	0.1959	0.1692	G	0.2496	1.196
12	rs10778886	82701485	C	0.2294	0.2012	A	0.2496	1.182
13	rs754100	26538455	A	0.3918	0.3583	G	0.2496	1.153
18	rs1484873	43206985	A	0.1959	0.1692	G	0.2496	1.196
1	rs35874116	19181393	G	0.1289	0.1533	A	0.2497	0.8168
2	rs7558074	99992822	C	0.3892	0.3567	A	0.2497	1.149
14	rs2154294	42655275	A	0.1289	0.1533	G	0.2497	0.8168
20	rs13038095	46425576	A	0.03093	0.02087	C	0.2497	1.497
7	rs10499859	80258810	G	0.3196	0.2883	A	0.2498	1.159
11	rs523104	104869708	G	0.2242	0.255	C	0.2499	0.8444
5	rs2241568	77784433	G	0.268	0.2392	A	0.25	1.165
5	rs2241567	77784438	T	0.268	0.2392	A	0.25	1.165
4	rs989902	87706506	C	0.3428	0.3767	A	0.2501	0.8631
22	rs1139793	19868218	A	0.3428	0.3767	G	0.2501	0.8631
2	rs79443131	137988626	A	0.08247	0.065	G	0.2503	1.293
6	rs9461799	32689529	A	0.3943	0.3608	G	0.2503	1.153
11	rs1369322	38409443	G	0.3428	0.3758	A	0.2503	0.8662
11	rs12287912	122675193	A	0.3943	0.3608	C	0.2503	1.153
12	rs11170177	52966428	G	0.2732	0.305	C	0.2503	0.8565
13	rs17760491	97167152	A	0.08247	0.065	C	0.2503	1.293
14	rs8007286	45081126	A	0.2732	0.305	G	0.2503	0.8565
2	rs10199521	2519513	A	0.3222	0.29	G	0.2504	1.164
1	rs200339924	196871607	A	0.005181	0.001667	G	0.2505	3.12
19	rs58123634	15569378	C	0.005181	0.001667	G	0.2505	3.12
8	rs13439780	56344186	A	0.1418	0.1192	G	0.2508	1.221
11	rs10892063	116896155	C	0.1959	0.17	A	0.2508	1.189
13	rs7336995	95178317	A	0.3454	0.3792	C	0.2508	0.8638
16	rs11075812	70937582	A	0.1418	0.1192	G	0.2508	1.221
6	rs139000140	71233822	G	0.005181	0.001669	A	0.251	3.115
14	rs200818825	23744820	A	0.1186	0.09833	T	0.251	1.233
15	rs2297773	79755604	G	0.1186	0.09833	A	0.251	1.233

18	rs3744900	33779705	A	0.1186	0.09833	G	0.251	1.233
19	rs504280	21477379	A	0.08763	0.1092	G	0.2512	0.7838
19	rs4803448	41734560	G	0.3479	0.3817	A	0.2514	0.8645
4	rs362272	3234980	A	0.3969	0.3642	G	0.2515	1.149
16	rs480727	88872229	A	0.232	0.2033	G	0.2515	1.183
19	rs13346368	48198675	G	0.2758	0.3075	A	0.2515	0.8575
1	rs324420	46870761	A	0.1675	0.1433	C	0.2516	1.203
6	rs683369	160551204	C	0.1675	0.1433	G	0.2516	1.203
9	rs2269700	118163563	G	0.1675	0.1433	A	0.2516	1.203
12	rs2429467	47186770	A	0.2706	0.2417	G	0.2516	1.164
6	rs61736085	27277365	A	0.05412	0.04	G	0.2518	1.373
7	rs2074654	45217015	G	0.08763	0.11	A	0.2518	0.7771
12	rs3751262	12618690	A	0.1572	0.1842	G	0.2518	0.8264
21	rs417708	42717662	A	0.05412	0.04	G	0.2518	1.373
5	rs3106500	104222478	A	0.2758	0.3067	C	0.2519	0.8609
1	rs505151	55529187	G	0.06701	0.05167	A	0.252	1.318
5	rs7447381	123401074	A	0.3247	0.2933	G	0.252	1.159
6	rs4947324	31528130	A	0.06701	0.05167	G	0.252	1.318
12	rs34162417	101336201	G	0.06701	0.05167	C	0.252	1.318
17	rs16943991	58121453	A	0.06701	0.05167	G	0.252	1.318
20	rs6036107	22403287	A	0.3247	0.2933	G	0.252	1.159
1	rs113288277	979748	A	0.005155	0.001667	T	0.2521	3.104
1	rs118105080	981345	A	0.005155	0.001667	G	0.2521	3.104
1	rs201113424	16101302	G	0.005155	0.001667	C	0.2521	3.104
1	rs141954697	42045818	A	0.005155	0.001667	G	0.2521	3.104
1	rs146175246	44463398	A	0.005155	0.001667	G	0.2521	3.104
1	rs56051744	45811119	G	0.005155	0.001667	A	0.2521	3.104
1	rs144896315	47279176	A	0.005155	0.001667	G	0.2521	3.104
1	rs147829805	86512531	C	0.005155	0.001667	A	0.2521	3.104
1	rs200389969	116227961	G	0.005155	0.001667	A	0.2521	3.104
1	rs200985071	118481173	A	0.005155	0.001667	G	0.2521	3.104
1	rs199998182	144917636	A	0.005155	0.001667	G	0.2521	3.104
1	rs201955039	150049795	A	0.005155	0.001667	C	0.2521	3.104
1	rs201743876	156263837	G	0.005155	0.001667	A	0.2521	3.104
1	rs200756276	156646306	A	0.005155	0.001667	C	0.2521	3.104
1	rs55892037	156846256	G	0.005155	0.001667	A	0.2521	3.104
1	rs75584817	178777231	C	0.005155	0.001667	A	0.2521	3.104
1	rs79183244	186099134	G	0.005155	0.001667	A	0.2521	3.104
1	rs184883943	196973817	A	0.005155	0.001667	G	0.2521	3.104
1	rs117583891	202245609	A	0.005155	0.001667	G	0.2521	3.104
1	rs188651046	202270005	G	0.005155	0.001667	A	0.2521	3.104
1	rs140703266	207317935	G	0.005155	0.001667	A	0.2521	3.104
1	rs199541826	222700317	C	0.005155	0.001667	G	0.2521	3.104

2	rs35966666	58316786	G	0.005155	0.001667	A	0.2521	3.104
2	rs146959744	61297612	A	0.005155	0.001667	G	0.2521	3.104
2	rs146563500	73457287	A	0.005155	0.001667	G	0.2521	3.104
2	rs145200665	86707449	A	0.005155	0.001667	G	0.2521	3.104
2	rs143372458	97508139	A	0.005155	0.001667	G	0.2521	3.104
2	rs148616345	99012373	A	0.005155	0.001667	G	0.2521	3.104
2	rs189963968	128878832	A	0.005155	0.001667	G	0.2521	3.104
2	rs151112282	135756499	A	0.005155	0.001667	G	0.2521	3.104
2	rs150095386	172309675	A	0.005155	0.001667	C	0.2521	3.104
2	rs181618376	175337832	A	0.005155	0.001667	G	0.2521	3.104
2	rs4894028	179403750	A	0.005155	0.001667	G	0.2521	3.104
2	rs62621236	179408713	G	0.005155	0.001667	A	0.2521	3.104
2	rs747122	179414318	A	0.005155	0.001667	G	0.2521	3.104
2	rs202040332	179425453	C	0.005155	0.001667	G	0.2521	3.104
2	rs10164753	179438866	A	0.005155	0.001667	G	0.2521	3.104
2	rs4893852	179571448	G	0.005155	0.001667	A	0.2521	3.104
2	rs13398235	179578704	A	0.005155	0.001667	G	0.2521	3.104
2	rs11888217	179593352	A	0.005155	0.001667	G	0.2521	3.104
2	rs200792058	179599565	A	0.005155	0.001667	G	0.2521	3.104
2	rs4893853	179632496	G	0.005155	0.001667	A	0.2521	3.104
2	rs150123071	204009369	G	0.005155	0.001667	A	0.2521	3.104
2	rs150197749	205986404	A	0.005155	0.001667	G	0.2521	3.104
2	rs147173240	206617688	A	0.005155	0.001667	G	0.2521	3.104
2	rs184981869	206659759	A	0.005155	0.001667	C	0.2521	3.104
2	rs182044267	211306091	A	0.005155	0.001667	G	0.2521	3.104
2	rs2292549	219128506	G	0.005155	0.001667	A	0.2521	3.104
2	rs79141637	219549910	G	0.005155	0.001667	A	0.2521	3.104
2	rs201044232	241569703	A	0.005155	0.001667	G	0.2521	3.104
3	rs118038433	48683635	A	0.005155	0.001667	G	0.2521	3.104
3	rs148917929	49459647	A	0.005155	0.001667	C	0.2521	3.104
3	exm2056570	51386327	A	0.005155	0.001667	G	0.2521	3.104
3	rs139420522	51386327	A	0.005155	0.001667	G	0.2521	3.104
3	rs199775265	53905393	G	0.005155	0.001667	C	0.2521	3.104
3	rs73076451	57432311	A	0.005155	0.001667	G	0.2521	3.104
3	rs149188165	64666977	T	0.005155	0.001667	A	0.2521	3.104
3	rs80220955	97806944	G	0.005155	0.001667	A	0.2521	3.104
3	rs185937975	97852274	A	0.005155	0.001667	G	0.2521	3.104
3	rs143049331	111298010	A	0.005155	0.001667	G	0.2521	3.104
3	rs141422904	131181605	A	0.005155	0.001667	G	0.2521	3.104
3	rs34111099	133098806	A	0.005155	0.001667	G	0.2521	3.104
3	rs147665692	145809661	A	0.005155	0.001667	G	0.2521	3.104
3	rs75523528	189711924	A	0.005155	0.001667	T	0.2521	3.104
3	rs199691918	195243723	A	0.005155	0.001667	C	0.2521	3.104

4	rs202104460	6302695	A	0.005155	0.001667	G	0.2521	3.104
4	rs147974629	6302831	A	0.005155	0.001667	G	0.2521	3.104
4	rs138386873	6600070	A	0.005155	0.001667	G	0.2521	3.104
4	rs142390648	8293200	A	0.005155	0.001667	G	0.2521	3.104
4	rs149183264	36069767	A	0.005155	0.001667	G	0.2521	3.104
4	rs79036998	46043120	C	0.005155	0.001667	G	0.2521	3.104
4	rs192553789	106157539	A	0.005155	0.001667	G	0.2521	3.104
4	rs202061241	187209733	A	0.005155	0.001667	G	0.2521	3.104
4	rs201363601	187540610	A	0.005155	0.001667	G	0.2521	3.104
5	rs188173439	33455801	G	0.005155	0.001667	A	0.2521	3.104
5	rs186911216	65008798	A	0.005155	0.001667	G	0.2521	3.104
5	rs145422621	68490521	A	0.005155	0.001667	G	0.2521	3.104
5	rs199702305	131326566	A	0.005155	0.001667	G	0.2521	3.104
5	rs150637009	149219653	A	0.005155	0.001667	G	0.2521	3.104
5	rs80162387	149515232	A	0.005155	0.001667	G	0.2521	3.104
5	rs185600013	150514023	A	0.005155	0.001667	G	0.2521	3.104
5	rs141803746	168093596	A	0.005155	0.001667	G	0.2521	3.104
5	rs200255935	172755187	G	0.005155	0.001667	C	0.2521	3.104
6	rs17300141	3012778	A	0.005155	0.001667	G	0.2521	3.104
6	rs11964779	31711624	C	0.005155	0.001667	A	0.2521	3.104
6	rs11967206	31732679	A	0.005155	0.001667	G	0.2521	3.104
6	rs147409390	31743842	A	0.005155	0.001667	G	0.2521	3.104
6	rs34360259	31778869	G	0.005155	0.001667	A	0.2521	3.104
6	rs36038685	31929737	A	0.005155	0.001667	G	0.2521	3.104
6	rs111605439	35745305	A	0.005155	0.001667	G	0.2521	3.104
6	rs199815717	37427378	A	0.005155	0.001667	G	0.2521	3.104
6	rs138455961	42796753	G	0.005155	0.001667	A	0.2521	3.104
6	rs6905288	43758873	G	0.2732	0.2433	A	0.2521	1.169
6	rs193274454	45870832	A	0.005155	0.001667	G	0.2521	3.104
6	rs45504096	52768506	A	0.005155	0.001667	G	0.2521	3.104
6	rs200340875	74524749	A	0.005155	0.001667	G	0.2521	3.104
6	rs181848883	82935295	A	0.005155	0.001667	G	0.2521	3.104
6	rs78964329	84925583	G	0.005155	0.001667	A	0.2521	3.104
6	rs11964202	122766138	A	0.005155	0.001667	G	0.2521	3.104
6	rs202166497	168465667	A	0.005155	0.001667	G	0.2521	3.104
7	rs199799310	21847580	A	0.005155	0.001667	G	0.2521	3.104
7	rs200891942	21948010	G	0.005155	0.001667	A	0.2521	3.104
7	rs190136958	37136290	A	0.005155	0.001667	G	0.2521	3.104
7	rs181233090	48045605	A	0.005155	0.001667	G	0.2521	3.104
7	rs78244461	55269456	A	0.005155	0.001667	G	0.2521	3.104
7	rs139583955	87214970	A	0.005155	0.001667	C	0.2521	3.104
7	rs183670544	97921977	A	0.005155	0.001667	G	0.2521	3.104
7	rs140881970	100685760	A	0.005155	0.001667	G	0.2521	3.104

7	rs121909034	117243663	A	0.005155	0.001667	G	0.2521	3.104
7	rs75645485	138874088	A	0.005155	0.001667	G	0.2521	3.104
7	rs13229174	143632833	G	0.005155	0.001667	A	0.2521	3.104
7	rs13235235	143633157	G	0.005155	0.001667	A	0.2521	3.104
8	rs141804459	16012620	A	0.005155	0.001667	G	0.2521	3.104
8	rs200353170	20028625	A	0.005155	0.001667	G	0.2521	3.104
8	rs200101638	24358350	A	0.005155	0.001667	G	0.2521	3.104
8	rs117934093	100887797	T	0.005155	0.001667	A	0.2521	3.104
8	rs1388959	113470948	A	0.3505	0.3842	G	0.2521	0.8651
8	rs6993813	120052238	A	0.3995	0.3667	G	0.2521	1.149
8	rs143026820	144698305	A	0.005155	0.001667	G	0.2521	3.104
8	rs138829800	145770775	A	0.005155	0.001667	G	0.2521	3.104
9	rs188377589	4719182	A	0.005155	0.001667	C	0.2521	3.104
9	rs180950847	6421069	A	0.005155	0.001667	G	0.2521	3.104
9	rs143617101	33366663	G	0.005155	0.001667	A	0.2521	3.104
9	rs149501146	35740009	A	0.005155	0.001667	G	0.2521	3.104
9	rs201747635	71538291	G	0.005155	0.001667	A	0.2521	3.104
9	rs189443180	71852884	A	0.005155	0.001667	G	0.2521	3.104
9	rs2274922	77390991	G	0.005155	0.001667	A	0.2521	3.104
9	rs200867550	94486156	A	0.005155	0.001667	G	0.2521	3.104
9	rs145020127	116241775	C	0.005155	0.001667	G	0.2521	3.104
9	rs75986214	125273904	A	0.005155	0.001667	G	0.2521	3.104
9	rs201284198	130263306	A	0.005155	0.001667	G	0.2521	3.104
10	rs149804621	1245930	A	0.005155	0.001667	G	0.2521	3.104
10	rs150824509	18834927	A	0.005155	0.001667	G	0.2521	3.104
10	rs202201583	37431020	A	0.005155	0.001667	G	0.2521	3.104
10	rs184026653	56287583	G	0.005155	0.001667	A	0.2521	3.104
10	rs144459930	64936086	G	0.005155	0.001667	A	0.2521	3.104
10	rs139681358	79566672	A	0.005155	0.001667	G	0.2521	3.104
10	rs140549288	91099466	C	0.005155	0.001667	G	0.2521	3.104
10	rs142573385	101610475	A	0.005155	0.001667	G	0.2521	3.104
10	rs3750690	117075031	A	0.005155	0.001667	T	0.2521	3.104
11	rs144034443	617571	A	0.005155	0.001667	G	0.2521	3.104
11	rs201134228	21581854	A	0.005155	0.001667	G	0.2521	3.104
11	rs74858686	40136434	A	0.005155	0.001667	G	0.2521	3.104
11	rs182417012	56431736	A	0.005155	0.001667	C	0.2521	3.104
11	rs200014909	58275106	A	0.005155	0.001667	G	0.2521	3.104
11	rs200423971	62186622	A	0.005155	0.001667	G	0.2521	3.104
11	rs186663136	62445052	A	0.005155	0.001667	G	0.2521	3.104
11	rs200209283	66358352	A	0.005155	0.001667	G	0.2521	3.104
11	rs115700798	87908411	A	0.005155	0.001667	G	0.2521	3.104
11	rs523604	118755738	G	0.3505	0.3842	A	0.2521	0.8651
11	rs144732457	124551336	G	0.005155	0.001667	A	0.2521	3.104

11	rs201491308	125546370	A	0.005155	0.001667	G	0.2521	3.104
12	rs192185341	42745717	C	0.005155	0.001667	G	0.2521	3.104
12	rs147582637	51589851	A	0.005155	0.001667	G	0.2521	3.104
12	rs28382854	57910354	C	0.005155	0.001667	A	0.2521	3.104
12	rs144198119	75683520	A	0.005155	0.001667	G	0.2521	3.104
12	rs116062676	123424824	G	0.005155	0.001667	C	0.2521	3.104
12	rs192445871	123950400	A	0.005155	0.001667	G	0.2521	3.104
12	rs10773189	126171662	A	0.3505	0.3842	G	0.2521	0.8651
12	rs201330179	133311055	A	0.005155	0.001667	G	0.2521	3.104
13	rs187575622	48986450	G	0.005155	0.001667	A	0.2521	3.104
13	rs111983452	50056926	A	0.005155	0.001667	G	0.2521	3.104
13	rs201755795	78320753	A	0.005155	0.001667	G	0.2521	3.104
14	rs143406902	20612565	A	0.005155	0.001667	G	0.2521	3.104
14	rs201409522	24885925	A	0.005155	0.001667	G	0.2521	3.104
14	rs138926495	56114823	C	0.005155	0.001667	A	0.2521	3.104
14	rs202057128	59112510	G	0.005155	0.001667	A	0.2521	3.104
14	rs61744208	67759282	A	0.005155	0.001667	C	0.2521	3.104
14	rs139586208	90770487	A	0.005155	0.001667	G	0.2521	3.104
14	rs61993318	101347482	A	0.005155	0.001667	G	0.2521	3.104
15	rs187392024	40846212	A	0.005155	0.001667	G	0.2521	3.104
15	rs140543634	42129061	A	0.005155	0.001667	G	0.2521	3.104
15	rs200049880	43622197	G	0.005155	0.001667	A	0.2521	3.104
15	rs185665930	44864940	G	0.005155	0.001667	A	0.2521	3.104
15	rs200785525	45386877	A	0.005155	0.001667	G	0.2521	3.104
15	rs142762839	51250834	A	0.005155	0.001667	C	0.2521	3.104
15	rs201427665	63618488	A	0.005155	0.001667	G	0.2521	3.104
15	rs201090788	65703546	A	0.005155	0.001667	C	0.2521	3.104
15	rs147142448	70961435	A	0.005155	0.001667	G	0.2521	3.104
15	rs55918015	75044489	A	0.005155	0.001667	G	0.2521	3.104
15	rs148893322	81229192	A	0.005155	0.001667	G	0.2521	3.104
15	rs191789336	91310239	A	0.005155	0.001667	G	0.2521	3.104
16	rs192598225	2049831	C	0.005155	0.001667	G	0.2521	3.104
16	rs145251229	2331461	A	0.005155	0.001667	G	0.2521	3.104
16	rs146862274	3299718	A	0.005155	0.001667	G	0.2521	3.104
16	rs143873904	20410421	A	0.005155	0.001667	G	0.2521	3.104
16	rs2228248	27472772	A	0.005155	0.001667	G	0.2521	3.104
16	rs12919017	27474910	G	0.005155	0.001667	A	0.2521	3.104
16	rs151056688	31213040	A	0.005155	0.001667	G	0.2521	3.104
16	rs9934891	85144023	A	0.005155	0.001667	G	0.2521	3.104
16	rs201245608	88705607	G	0.005155	0.001667	C	0.2521	3.104
16	exm1269680	89169017	A	0.005155	0.001667	G	0.2521	3.104
16	rs150374081	89169017	A	0.005155	0.001667	G	0.2521	3.104
16	rs200606123	89715864	A	0.005155	0.001667	G	0.2521	3.104

17	rs151266248	8273108	A	0.005155	0.001667	G	0.2521	3.104
17	rs149166851	9086248	A	0.005155	0.001667	G	0.2521	3.104
17	rs201405375	17700557	A	0.005155	0.001667	G	0.2521	3.104
17	rs117072920	18149978	G	0.005155	0.001667	A	0.2521	3.104
17	rs79370191	27958628	G	0.005155	0.001667	A	0.2521	3.104
17	rs146557148	31075961	A	0.005155	0.001667	G	0.2521	3.104
17	rs142883160	33497222	T	0.005155	0.001667	A	0.2521	3.104
17	rs115744817	34182738	A	0.005155	0.001667	G	0.2521	3.104
17	rs202067640	35297628	A	0.005155	0.001667	G	0.2521	3.104
17	rs140590253	43924163	G	0.005155	0.001667	A	0.2521	3.104
17	rs146454487	48538186	A	0.005155	0.001667	G	0.2521	3.104
17	rs74404893	67170885	C	0.005155	0.001667	G	0.2521	3.104
17	rs143203816	73733712	A	0.005155	0.001667	C	0.2521	3.104
17	rs199643693	78188523	A	0.005155	0.001667	C	0.2521	3.104
18	rs116291960	8825584	A	0.005155	0.001667	G	0.2521	3.104
18	rs200120172	77895286	A	0.005155	0.001667	C	0.2521	3.104
18	rs113879497	77895740	G	0.005155	0.001667	A	0.2521	3.104
19	rs148427350	1043739	A	0.005155	0.001667	G	0.2521	3.104
19	rs142573667	1064171	A	0.005155	0.001667	C	0.2521	3.104
19	rs142805889	2877641	G	0.005155	0.001667	A	0.2521	3.104
19	rs145110523	3648677	A	0.005155	0.001667	G	0.2521	3.104
19	rs80050321	5711966	A	0.005155	0.001667	G	0.2521	3.104
19	rs72486387	9074073	A	0.3505	0.3842	G	0.2521	0.8651
19	rs73011014	9077581	A	0.3505	0.3842	C	0.2521	0.8651
19	rs34351170	11559907	G	0.005155	0.001667	A	0.2521	3.104
19	rs140648132	12298811	C	0.005155	0.001667	G	0.2521	3.104
19	rs78444677	12461510	A	0.005155	0.001667	G	0.2521	3.104
19	rs181476648	12766542	A	0.005155	0.001667	C	0.2521	3.104
19	rs147251319	13065125	A	0.005155	0.001667	G	0.2521	3.104
19	rs150851599	14938690	A	0.005155	0.001667	G	0.2521	3.104
19	rs143735207	15164583	A	0.005155	0.001667	G	0.2521	3.104
19	rs144064099	15465976	A	0.005155	0.001667	G	0.2521	3.104
19	rs117610912	16060267	A	0.005155	0.001667	G	0.2521	3.104
19	rs189485804	16687007	A	0.005155	0.001667	G	0.2521	3.104
19	rs115826747	18120784	A	0.005155	0.001667	G	0.2521	3.104
19	rs139622811	18726857	A	0.005155	0.001667	G	0.2521	3.104
19	rs200835025	36211118	A	0.005155	0.001667	G	0.2521	3.104
19	rs117872433	41522646	A	0.005155	0.001667	G	0.2521	3.104
19	rs1800028	49206817	A	0.005155	0.001667	G	0.2521	3.104
19	rs114957460	51512575	A	0.005155	0.001667	G	0.2521	3.104
19	rs150872882	55485879	G	0.005155	0.001667	A	0.2521	3.104
19	rs72490759	57868180	A	0.005155	0.001667	C	0.2521	3.104
19	rs199844677	59021241	A	0.005155	0.001667	G	0.2521	3.104

20	rs147664383	2465216	A	0.005155	0.001667	T	0.2521	3.104
20	rs652633	10030188	A	0.005155	0.001667	T	0.2521	3.104
20	rs524625	10030452	A	0.005155	0.001667	G	0.2521	3.104
20	rs17807673	18513372	A	0.005155	0.001667	G	0.2521	3.104
20	rs199888621	25457375	A	0.005155	0.001667	G	0.2521	3.104
20	rs8115854	35766337	G	0.005155	0.001667	A	0.2521	3.104
20	rs6031882	35809783	G	0.005155	0.001667	A	0.2521	3.104
20	rs148496920	49446886	A	0.005155	0.001667	G	0.2521	3.104
20	rs188447984	51871432	A	0.005155	0.001667	G	0.2521	3.104
20	rs201407701	62577807	A	0.005155	0.001667	G	0.2521	3.104
21	rs181536872	30419535	G	0.005155	0.001667	A	0.2521	3.104
21	rs201454422	38117485	A	0.005155	0.001667	G	0.2521	3.104
21	rs185333589	43281687	C	0.005155	0.001667	G	0.2521	3.104
21	rs183264392	45732896	A	0.005155	0.001667	G	0.2521	3.104
21	rs199508525	47571485	A	0.005155	0.001667	G	0.2521	3.104
22	rs189185729	17600393	A	0.005155	0.001667	G	0.2521	3.104
22	rs116694036	19363188	A	0.005155	0.001667	G	0.2521	3.104
22	rs175174	20127554	A	0.3505	0.3842	G	0.2521	0.8651
22	rs861854	21988524	A	0.005155	0.001667	G	0.2521	3.104
22	rs5749118	30927975	G	0.005155	0.001667	A	0.2521	3.104
22	rs188312885	31484930	A	0.005155	0.001667	G	0.2521	3.104
2	rs2005771	242066156	A	0.1985	0.1717	G	0.2523	1.195
4	rs7679852	56683846	A	0.1985	0.1717	G	0.2523	1.195
19	rs56405542	9060572	A	0.3505	0.3833	G	0.2524	0.8682
19	rs1007160	33353061	A	0.232	0.2042	C	0.2524	1.177
1	rs202201982	62456019	A	0.005155	0.001669	G	0.2526	3.098
2	rs201114978	234652193	C	0.005155	0.001669	G	0.2526	3.098
18	rs181589519	29795177	A	0.005155	0.001669	G	0.2526	3.098
20	rs151052399	3684023	G	0.005155	0.001669	A	0.2526	3.098
5	rs6420094	176817636	G	0.2784	0.31	A	0.2527	0.8585
9	rs4978584	117186712	A	0.4021	0.3692	G	0.2527	1.149
2	rs11686212	3392295	A	0.3575	0.3917	G	0.2529	0.8643
14	rs3212254	24805463	A	0.1082	0.1308	C	0.253	0.8064
17	rs7208693	56357818	A	0.1082	0.1308	C	0.253	0.8064
5	rs2547961	98058939	A	0.3273	0.2958	C	0.2531	1.158
8	rs201278290	144998336	A	0.005155	0.001672	G	0.2531	3.093
10	rs2185724	30316208	G	0.2732	0.2442	A	0.2531	1.164
14	rs145939433	73989385	A	0.005155	0.001672	G	0.2531	3.093
14	rs201934446	104639442	A	0.005155	0.001672	G	0.2531	3.093
16	rs201767825	921203	G	0.005155	0.001672	A	0.2531	3.093
17	rs961	8378932	G	0.3531	0.3858	A	0.2531	0.8688
2	rs2165738	24692809	G	0.4046	0.3717	C	0.2532	1.149
2	rs6725776	43783141	G	0.2294	0.2592	A	0.2532	0.8509

5	rs4235486	60603696	G	0.1314	0.1558	A	0.2532	0.8198
16	rs34444956	81185412	A	0.2294	0.26	G	0.2532	0.8472
16	rs433598	20680206	G	0.1443	0.1208	A	0.2533	1.227
1	rs12139511	59147926	A	0.4072	0.3733	G	0.2534	1.153
1	rs2789443	164733504	G	0.1907	0.2192	A	0.2534	0.8396
1	rs7532704	217971980	G	0.4072	0.3733	A	0.2534	1.153
2	rs61732269	97355026	G	0.1005	0.08167	A	0.2534	1.257
2	rs17367118	123641611	A	0.1005	0.08167	G	0.2534	1.257
3	rs293833	191179193	G	0.4072	0.3733	A	0.2534	1.153
19	rs4804378	9046876	A	0.3557	0.389	C	0.2534	0.8671
19	rs56334501	9048320	A	0.3557	0.3892	G	0.2534	0.8664
19	rs56307190	9049910	A	0.3557	0.3892	G	0.2534	0.8664
21	rs57646126	30331935	A	0.4072	0.3733	G	0.2534	1.153
1	rs735943	242030151	A	0.2345	0.2058	G	0.2536	1.182
2	rs191428830	241659323	A	0.005155	0.001675	G	0.2536	3.088
1	rs1361193	211383129	A	0.2809	0.3133	G	0.2538	0.8562
6	rs6570507	142679572	A	0.4072	0.3742	G	0.2538	1.149
8	rs17581368	21662445	C	0.3557	0.3883	A	0.2538	0.8695
9	rs7852039	39103743	A	0.2809	0.3133	G	0.2538	0.8562
20	rs3746471	36841914	A	0.4072	0.3742	G	0.2538	1.149
7	rs74498833	100850908	A	0.1005	0.0818	G	0.2539	1.254
6	rs3789783	143429476	G	0.3582	0.3917	A	0.254	0.867
11	rs2863174	45264277	A	0.3273	0.2967	G	0.254	1.154
19	rs143659792	14862462	A	0.0232	0.03589	G	0.254	0.6378
20	rs2207418	11174903	A	0.4098	0.3758	G	0.254	1.153
2	rs2303834	179469715	G	0.01546	0.02667	A	0.2541	0.5733
3	rs35923425	169569432	C	0.01546	0.02667	G	0.2541	0.5733
6	rs9370867	16145325	A	0.01546	0.02667	G	0.2541	0.5733
6	rs56027330	25850845	A	0.01546	0.02667	G	0.2541	0.5733
6	rs17177632	29408090	G	0.01546	0.02667	A	0.2541	0.5733
6	rs17177674	29408555	A	0.01546	0.02667	C	0.2541	0.5733
6	rs7768854	29428831	C	0.01546	0.02667	A	0.2541	0.5733
6	rs3132581	30913458	A	0.0232	0.03583	G	0.2541	0.639
6	rs3129900	32305979	C	0.01546	0.02667	A	0.2541	0.5733
6	rs3132963	32320153	G	0.01546	0.02667	A	0.2541	0.5733
6	rs3129934	32336187	A	0.01546	0.02667	G	0.2541	0.5733
10	rs184216138	30315856	G	0.01546	0.02667	A	0.2541	0.5733
11	rs10501570	84417846	G	0.0232	0.03583	A	0.2541	0.639
12	exm1761304	15739836	A	0.01546	0.02667	G	0.2541	0.5733
12	rs77872564	15739836	A	0.01546	0.02667	G	0.2541	0.5733
13	rs17279819	21732175	A	0.01546	0.02667	C	0.2541	0.5733
13	rs2236364	42875044	C	0.01546	0.02667	G	0.2541	0.5733
14	rs34645221	24785092	A	0.01546	0.02667	C	0.2541	0.5733

14	rs77910307	47566114	G	0.01546	0.02667	A	0.2541	0.5733
16	rs117325492	71718424	A	0.01546	0.02667	G	0.2541	0.5733
22	rs3177243	24179922	G	0.01546	0.02667	C	0.2541	0.5733
10	rs445739	55296717	G	0.3299	0.2983	A	0.2542	1.158
1	rs11576830	161068836	C	0.4098	0.3767	A	0.2543	1.149
6	rs3094631	30267748	G	0.0567	0.075	A	0.2543	0.7413
6	rs3094065	30282332	A	0.0567	0.075	G	0.2543	0.7413
6	rs3129838	30306553	G	0.0567	0.075	A	0.2543	0.7413
1	rs12410563	247006051	G	0.1598	0.187	C	0.2544	0.827
6	rs3129938	32336495	A	0.01554	0.02667	T	0.2544	0.5763
1	rs3850531	19566382	G	0.08247	0.06583	A	0.2546	1.275
5	rs10071758	89643399	A	0.2758	0.2467	G	0.2546	1.163
7	rs33922584	56125757	G	0.08247	0.06583	A	0.2546	1.275
9	rs1851722	107361642	A	0.3608	0.3942	G	0.2546	0.8677
13	rs2210007	35126337	G	0.08247	0.06583	A	0.2546	1.275
20	rs11700112	9547018	G	0.2758	0.2467	C	0.2546	1.163
4	rs11932636	119302960	A	0.1598	0.1858	G	0.2547	0.8332
6	rs1766848	656555	A	0.0232	0.03667	C	0.2547	0.6239
9	rs75241638	90502058	G	0.0232	0.03667	C	0.2547	0.6239
11	rs76022391	101833768	A	0.0232	0.03667	G	0.2547	0.6239
14	rs2073342	21360216	C	0.232	0.2625	G	0.2547	0.8485
16	rs4784227	52599188	A	0.232	0.2625	G	0.2547	0.8485
19	rs799193	11891003	G	0.0232	0.03667	A	0.2547	0.6239
11	rs12791318	17393644	G	0.1701	0.1458	A	0.2548	1.201
9	rs4836891	125273574	A	0.232	0.2617	G	0.2549	0.8522
16	rs255049	68013471	G	0.201	0.1742	A	0.2549	1.193
17	rs11552708	7462555	A	0.4149	0.3815	G	0.2549	1.15
13	rs12857479	31531009	A	0.2835	0.315	G	0.255	0.8605
6	rs1341268	105643136	G	0.4098	0.3775	A	0.2551	1.145
1	rs501163	110078434	G	0.3325	0.3008	A	0.2552	1.158
3	rs7637449	56628031	A	0.04381	0.06083	G	0.2553	0.7074
3	rs9835332	56667682	C	0.04381	0.06083	G	0.2553	0.7074
6	rs2273668	109323519	A	0.04381	0.06083	C	0.2553	0.7074
19	rs78052167	52887603	A	0.04381	0.06083	G	0.2553	0.7074
9	rs10760563	131303522	A	0.4149	0.3817	G	0.2554	1.149
10	rs11187895	96106240	C	0.1933	0.2217	G	0.2554	0.8414
13	rs7325843	41837155	A	0.4149	0.3817	G	0.2554	1.149
16	rs4889241	81157324	G	0.07216	0.09167	A	0.2554	0.7707
3	rs16863403	151164174	C	0.08505	0.06667	A	0.2555	1.301
8	rs1903595	14389481	G	0.08505	0.06667	A	0.2555	1.301
9	rs12001918	86518796	G	0.07216	0.0925	A	0.2555	0.7631
10	rs9422897	127296145	A	0.07216	0.0925	C	0.2555	0.7631
20	rs2070856	23731412	A	0.08505	0.06667	G	0.2555	1.301

22	rs35047625	32000930	A	0.08505	0.06667	G	0.2555	1.301
15	rs3829462	58853079	C	0.07216	0.09265	A	0.2556	0.7617
6	rs2071474	32782582	A	0.366	0.4	G	0.2557	0.8659
2	rs2341459	44768202	G	0.366	0.3992	A	0.2558	0.8689
6	rs2621338	32776583	A	0.366	0.3992	G	0.2558	0.8689
3	rs1444812	122756601	C	0.1211	0.1008	A	0.2559	1.229
6	rs9364602	161974956	G	0.4175	0.3842	A	0.2559	1.149
13	rs495680	33703656	A	0.4175	0.3842	G	0.2559	1.149
17	rs9892334	35307070	A	0.201	0.175	G	0.2559	1.186
20	rs2245220	4705718	A	0.3325	0.3017	G	0.256	1.153
8	rs12675375	21984945	A	0.4245	0.3917	G	0.2561	1.146
1	rs3088232	92445257	G	0.01546	0.0275	C	0.2562	0.5554
1	rs199842829	159890155	A	0.01546	0.0275	G	0.2562	0.5554
6	rs7765791	29410093	G	0.01546	0.0275	A	0.2562	0.5554
6	rs6456951	29426203	A	0.01546	0.0275	T	0.2562	0.5554
6	rs7751705	29441618	A	0.01546	0.0275	G	0.2562	0.5554
7	rs972283	130466854	A	0.3351	0.3033	G	0.2562	1.157
7	rs140463378	139575408	A	0.01546	0.0275	G	0.2562	0.5554
10	rs34042320	37421199	A	0.01546	0.0275	G	0.2562	0.5554
12	rs984303	15750391	G	0.01546	0.0275	A	0.2562	0.5554
12	rs146881270	124846833	C	0.01546	0.0275	A	0.2562	0.5554
14	rs77323631	59112221	A	0.01546	0.0275	G	0.2562	0.5554
16	rs74344827	71610276	A	0.01546	0.0275	G	0.2562	0.5554
2	rs1041973	102955468	A	0.134	0.1592	C	0.2563	0.8176
3	rs35319679	12962074	A	0.134	0.1586	G	0.2563	0.8211
2	rs10198230	140750641	A	0.4201	0.3867	C	0.2564	1.149
6	rs9373985	97063522	G	0.3686	0.4017	C	0.2564	0.8695
7	rs17472556	33139147	A	0.4201	0.3867	G	0.2564	1.149
7	rs797820	83609039	G	0.09021	0.1117	A	0.2564	0.7888
11	rs558813	129722553	A	0.09021	0.1117	G	0.2564	0.7888
12	rs2277339	57146069	C	0.2371	0.2092	A	0.2564	1.175
6	rs3128927	33074288	A	0.134	0.1583	G	0.2565	0.8227
9	rs10972341	35141705	G	0.2345	0.2642	A	0.2565	0.8535
1	rs75306471	54605320	C	0.4175	0.385	G	0.2566	1.145
1	rs4655852	88035857	G	0.4175	0.385	A	0.2566	1.145
20	rs881118	5903388	C	0.1727	0.1475	A	0.2567	1.206
3	rs2291089	124515636	A	0.1108	0.1342	G	0.2568	0.8043
16	rs2241414	58314433	G	0.1108	0.1342	A	0.2568	0.8043
16	rs1052276	58314598	A	0.1108	0.1342	G	0.2568	0.8043
11	rs12362209	85584303	G	0.1031	0.08333	A	0.2569	1.264
17	rs1801690	64208285	C	0.1031	0.08333	G	0.2569	1.264
2	rs888012	202561571	G	0.4201	0.3875	A	0.257	1.145
6	rs7764856	32680640	T	0.4201	0.3875	A	0.257	1.145

8	rs1063582	23167353	A	0.2887	0.3208	C	0.257	0.859
8	rs17196271	121984358	G	0.2887	0.3208	A	0.257	0.859
15	rs933769	96052742	G	0.4201	0.3875	A	0.257	1.145
19	rs8112263	43344686	G	0.3711	0.4042	A	0.257	0.87
20	rs11960	17600357	A	0.3351	0.3042	G	0.257	1.153
2	rs17852959	219900068	A	0.1624	0.1892	G	0.2571	0.8309
7	rs1637696	34979905	A	0.1443	0.1225	C	0.2571	1.208
9	rs2622266	116420246	G	0.1443	0.1225	A	0.2571	1.208
10	rs12766217	78122729	A	0.1443	0.1225	C	0.2571	1.208
12	rs61960670	133768553	T	0.1443	0.1225	A	0.2571	1.208
19	rs302453	56390237	A	0.1443	0.1225	T	0.2571	1.208
20	rs2024714	60212494	G	0.2887	0.32	A	0.2572	0.8623
1	rs17856201	212965095	A	0.1108	0.135	G	0.2573	0.7986
19	rs2074858	57723007	C	0.3376	0.3058	G	0.2573	1.157
19	rs917340	57723013	A	0.3376	0.3058	G	0.2573	1.157
2	rs10929808	12568996	G	0.4253	0.3917	A	0.2574	1.149
3	rs2276742	58107156	A	0.01613	0.009182	G	0.2574	1.769
11	rs10898392	85164751	C	0.4253	0.3917	A	0.2574	1.149
15	rs28620278	56959028	A	0.4253	0.3917	G	0.2574	1.149
7	rs7802007	124638034	G	0.3737	0.4067	A	0.2576	0.8706
8	rs10111520	139749799	A	0.06959	0.05333	C	0.2576	1.328
11	exm2250378	86267678	A	0.06959	0.05333	G	0.2576	1.328
11	rs4943943	86267678	A	0.06959	0.05333	G	0.2576	1.328
12	rs1471997	48723595	A	0.2371	0.2675	G	0.2578	0.8511
20	rs6010620	62309839	G	0.2371	0.2675	A	0.2578	0.8511
14	rs36017951	81743565	G	0.08505	0.0675	A	0.258	1.284
4	rs9996317	113867271	A	0.2912	0.3224	G	0.2581	0.8634
5	rs254658	86470998	C	0.3402	0.308	A	0.2581	1.158
10	rs2135720	55755491	A	0.4973	0.4633	G	0.2581	1.146
19	rs56408444	17017878	A	0.2912	0.3233	G	0.2581	0.8599
1	rs142425806	155217739	A	0.04381	0.03083	G	0.2582	1.44
2	rs2363468	209190330	A	0.04381	0.03083	G	0.2582	1.44
2	rs893254	209190519	A	0.04381	0.03083	T	0.2582	1.44
2	rs893253	209190528	G	0.04381	0.03083	C	0.2582	1.44
2	rs1529979	209191082	C	0.04381	0.03083	A	0.2582	1.44
4	rs117401196	54424055	G	0.04381	0.03083	C	0.2582	1.44
7	rs7787531	129023597	G	0.04381	0.03083	A	0.2582	1.44
9	rs3739553	129984107	A	0.1237	0.1025	G	0.2582	1.236
17	rs2280490	9676134	A	0.1237	0.1025	G	0.2582	1.236
19	rs11559188	10221642	G	0.04381	0.03083	A	0.2582	1.44
1	rs13306581	183536358	A	0.05412	0.04083	G	0.2583	1.344
6	rs1619379	29785235	A	0.2397	0.2117	G	0.2583	1.174
8	rs28608091	36767024	G	0.3402	0.3083	A	0.2583	1.157

11	rs4929949	8604593	G	0.3789	0.4125	A	0.2583	0.8687
12	rs1847461	91077508	A	0.3789	0.4125	G	0.2583	0.8687
17	rs2014055	60741917	A	0.05412	0.04083	G	0.2583	1.344
21	rs2032287	18008449	G	0.3789	0.4125	A	0.2583	0.8687
2	rs10199914	239896861	A	0.4304	0.3967	G	0.2584	1.149
16	rs7359397	28885659	A	0.1469	0.1242	G	0.2587	1.215
6	rs6906021	32626311	G	0.3814	0.415	A	0.2588	0.8693
2	rs77081203	15698746	C	0.03351	0.04833	G	0.2589	0.6826
4	rs4596219	175617314	G	0.3402	0.3092	A	0.2589	1.152
7	rs12536223	2472555	A	0.03351	0.04833	G	0.2589	0.6826
11	rs10769908	8484089	G	0.3814	0.4142	A	0.2592	0.8723
2	rs1545133	113309473	A	0.3428	0.3108	G	0.2593	1.156
9	rs1851725	107360826	A	0.384	0.4175	G	0.2593	0.8698
9	rs1523678	107360851	A	0.384	0.4175	G	0.2593	0.8698
11	rs7110736	113561054	A	0.2397	0.27	G	0.2594	0.8524
17	rs1048649	3564378	A	0.2397	0.2125	G	0.2595	1.168
9	rs10818503	123850770	G	0.1985	0.2275	A	0.2596	0.8407
4	rs10021843	119407189	A	0.4381	0.4042	G	0.2597	1.15
9	rs7029145	27882653	A	0.384	0.4167	G	0.2597	0.8728
4	rs4835002	146218651	A	0.3866	0.42	G	0.2598	0.8703
20	rs1111032	43378770	A	0.3866	0.42	G	0.2598	0.8703
1	rs4845898	12339619	A	0.1649	0.1925	T	0.2599	0.8286
1	rs61746559	48694594	A	0.1366	0.1608	G	0.2599	0.8255
6	rs1805018	46679303	G	0.1649	0.1925	A	0.2599	0.8286
14	rs1152571	60647891	G	0.1649	0.1925	A	0.2599	0.8286
15	rs4923705	36293605	G	0.4381	0.405	A	0.26	1.146
17	rs2740354	632905	G	0.3892	0.4232	A	0.26	0.8684
21	rs1053312	47423389	A	0.2036	0.1783	G	0.26	1.178
19	rs35717293	21912755	A	0.2964	0.3283	G	0.2601	0.8617
12	rs7963799	22313448	A	0.3892	0.4225	G	0.2603	0.8709
13	rs2296984	99457431	C	0.2861	0.2567	A	0.2603	1.161
17	rs3213755	39197499	A	0.2861	0.2567	G	0.2603	1.161
8	rs897051	131027841	A	0.4407	0.4075	G	0.2604	1.146
12	rs7972509	114713515	A	0.4407	0.4075	G	0.2604	1.146
15	rs16962252	36290481	A	0.4407	0.4075	G	0.2604	1.146
19	rs2216662	9057721	G	0.4407	0.4075	A	0.2604	1.146
1	rs12566027	73281986	G	0.2964	0.3275	A	0.2605	0.865
2	rs6705471	191634597	A	0.2964	0.3275	G	0.2605	0.865
8	rs8178235	48697705	A	0.03351	0.04917	G	0.2606	0.6704
15	rs12900132	100537494	A	0.3918	0.4258	G	0.2606	0.8684
16	rs1004041	1271890	C	0.1237	0.1033	A	0.2606	1.225
20	rs6579255	34219496	G	0.1237	0.1033	A	0.2606	1.225
2	rs888096	37603801	G	0.3454	0.3142	A	0.2608	1.152

3	rs9812284	68102663	G	0.4433	0.41	A	0.2608	1.146
4	rs4695357	48199195	C	0.4433	0.41	A	0.2608	1.146
5	rs37370	35039486	G	0.4711	0.4375	A	0.2608	1.145
7	rs2390080	19337064	G	0.3892	0.4217	A	0.2608	0.8739
9	rs2274924	77376647	G	0.3454	0.3142	A	0.2608	1.152
4	rs34627298	76881233	A	0.1134	0.1367	C	0.2609	0.808
4	rs34193982	178262784	G	0.1134	0.1367	A	0.2609	0.808
2	rs12623297	29226512	A	0.1753	0.1508	G	0.261	1.196
7	rs11533869	63754787	A	0.1134	0.1361	G	0.261	0.8122
10	rs4387287	105677897	A	0.1753	0.1508	C	0.261	1.196
1	rs10752881	182973491	A	0.4407	0.4083	G	0.2611	1.142
6	rs480092	31764899	G	0.2423	0.2717	A	0.2612	0.8572
10	rs2797992	95959384	A	0.299	0.3308	G	0.2612	0.8626
14	rs2582513	105414790	G	0.3943	0.4275	A	0.2612	0.8719
7	rs57020142	137778542	A	0.3969	0.4308	G	0.2614	0.8694
10	rs3750587	134626293	A	0.07474	0.09432	C	0.2615	0.7756
14	rs4905081	94088587	A	0.4433	0.4108	G	0.2615	1.142
19	rs1366245	52660279	G	0.4485	0.415	A	0.2615	1.146
3	rs4858817	48416756	A	0.3969	0.43	G	0.2616	0.8724
3	rs6442117	48419897	G	0.3969	0.43	A	0.2616	0.8724
6	rs7759272	29684994	G	0.201	0.23	A	0.2616	0.8424
6	rs9258157	29688099	T	0.201	0.23	A	0.2616	0.8424
6	rs2076183	29691303	A	0.201	0.23	G	0.2616	0.8424
6	rs3734814	29694680	C	0.201	0.23	A	0.2616	0.8424
6	rs2272874	29696245	G	0.201	0.23	A	0.2616	0.8424
1	rs35533335	32280369	A	0.06959	0.05417	G	0.2617	1.306
1	rs11119014	208391098	G	0.09278	0.115	A	0.2617	0.7871
3	rs9840993	123457893	G	0.06959	0.05417	A	0.2617	1.306
6	rs73717741	4874759	C	0.06959	0.05417	G	0.2617	1.306
6	rs3811102	146125793	T	0.2887	0.2592	A	0.2617	1.16
6	rs9373475	146126419	A	0.2887	0.2592	G	0.2617	1.16
12	rs11060869	130897323	A	0.06959	0.05417	G	0.2617	1.306
15	rs76735592	81654624	A	0.07474	0.095	G	0.2617	0.7695
17	rs3744255	7637789	A	0.06959	0.05417	G	0.2617	1.306
22	rs5770651	50077531	G	0.456	0.4225	A	0.2617	1.146
3	rs33759	45276370	G	0.3479	0.3167	A	0.2618	1.151
14	rs3825549	39716808	G	0.07474	0.09516	A	0.2618	0.7681
6	rs3873386	31273745	G	0.451	0.4175	A	0.2619	1.146
7	rs10227687	38741626	A	0.3995	0.4325	G	0.262	0.8729
12	rs11106216	76672829	G	0.08505	0.06833	A	0.262	1.267
5	rs4466137	82985739	A	0.2448	0.2167	C	0.2621	1.172
12	rs2708421	40593403	G	0.4485	0.4158	A	0.2621	1.142
4	rs11734603	32268382	G	0.3505	0.3183	A	0.2622	1.156

5	rs11134742	171764665	A	0.4536	0.42	G	0.2622	1.146
10	rs2918142	132992902	A	0.4536	0.42	G	0.2622	1.146
12	rs10846979	126284098	G	0.3015	0.3333	A	0.2622	0.8635
14	rs10145325	78705184	A	0.3505	0.3183	C	0.2622	1.156
17	rs9913145	56389732	G	0.1675	0.1942	A	0.2622	0.8352
1	rs189570035	31189689	A	0.03351	0.0225	G	0.2623	1.506
2	rs1432295	61066666	G	0.03351	0.0225	A	0.2623	1.506
3	rs56369596	130437265	G	0.03351	0.0225	C	0.2623	1.506
4	rs72938299	144532644	G	0.03351	0.0225	A	0.2623	1.506
4	rs17017968	144542213	G	0.03351	0.0225	A	0.2623	1.506
4	rs7679078	144546704	G	0.03351	0.0225	A	0.2623	1.506
5	rs143444882	10261748	A	0.03351	0.0225	G	0.2623	1.506
13	rs12863903	96638651	A	0.03351	0.0225	G	0.2623	1.506
14	rs8016621	22002461	A	0.03351	0.0225	G	0.2623	1.506
15	rs143022130	43276168	A	0.03351	0.0225	G	0.2623	1.506
16	rs80346348	67685694	A	0.03351	0.0225	G	0.2623	1.506
22	rs146769197	24567910	A	0.03351	0.0225	G	0.2623	1.506
2	rs2710684	17814030	G	0.2062	0.1808	A	0.2624	1.177
3	rs6781846	33786741	C	0.4021	0.435	A	0.2624	0.8734
4	rs7675987	54011526	G	0.2062	0.1808	A	0.2624	1.177
4	rs7697556	73515313	A	0.4021	0.435	G	0.2624	0.8734
6	rs9356703	19665871	A	0.4021	0.435	G	0.2624	0.8734
6	rs9501057	31106516	A	0.2062	0.1808	G	0.2624	1.177
8	rs4735980	1018332	G	0.451	0.4183	A	0.2624	1.142
10	rs4881086	3155698	A	0.2062	0.1808	G	0.2624	1.177
10	rs7083165	20516587	G	0.4046	0.4383	A	0.2625	0.8709
19	rs2547067	9070837	G	0.1495	0.1267	A	0.2625	1.212
2	rs4665730	25055774	G	0.4562	0.4225	A	0.2626	1.147
3	rs10934211	112696331	A	0.3505	0.3192	G	0.2627	1.151
11	rs1939810	105886989	G	0.4536	0.4208	A	0.2627	1.143
11	rs2305830	117266312	G	0.2927	0.2629	C	0.2627	1.16
16	rs28417933	89293271	A	0.4536	0.4208	C	0.2627	1.143
19	rs58824375	55673145	A	0.2448	0.2742	G	0.2627	0.8584
22	rs5998267	32554985	G	0.4536	0.4208	A	0.2627	1.143
20	rs9346	42826350	C	0.4046	0.4375	A	0.2628	0.8738
2	rs6738028	111949327	G	0.4588	0.425	C	0.2629	1.147
6	rs1233492	29458477	G	0.1753	0.1517	A	0.2629	1.189
6	rs1233490	29461914	G	0.1753	0.1517	A	0.2629	1.189
6	rs2746147	29466608	A	0.1753	0.1517	G	0.2629	1.189
6	rs1233486	29469652	A	0.1753	0.1517	G	0.2629	1.189
6	rs1233482	29475682	A	0.1753	0.1517	C	0.2629	1.189
11	rs12806437	6942695	A	0.1753	0.1517	G	0.2629	1.189
12	rs3759171	72307616	A	0.1057	0.08583	G	0.2629	1.258

19	rs11554159	18285944	A	0.1263	0.105	G	0.263	1.232
8	rs7812779	127830777	G	0.3041	0.3358	A	0.2631	0.8643
10	rs2286735	115389366	A	0.2912	0.2617	G	0.2631	1.159
1	rs4074196	1611995	A	0.4613	0.4275	G	0.2632	1.147
2	rs1028144	99279354	A	0.4072	0.44	G	0.2632	0.8743
3	rs3796164	123453061	G	0.4072	0.44	A	0.2632	0.8743
6	rs2240070	30071110	A	0.2448	0.2175	G	0.2632	1.166
6	rs4713429	31021017	C	0.2448	0.2175	G	0.2632	1.166
7	rs11559146	6621277	A	0.2448	0.2175	G	0.2632	1.166
13	rs7335534	34710780	A	0.4072	0.44	G	0.2632	0.8743
5	rs4282331	30881510	A	0.4124	0.4458	C	0.2635	0.8723
10	rs2281880	104269217	A	0.2036	0.2325	G	0.2635	0.844
13	rs911973	109438084	A	0.2036	0.2325	T	0.2635	0.844
19	rs4884	45810035	G	0.2036	0.2325	A	0.2635	0.844
1	rs12044926	151501841	G	0.2036	0.2317	C	0.2636	0.8479
3	rs75554452	131323142	G	0.4098	0.4425	A	0.2636	0.8748
5	rs337847	78259888	G	0.3041	0.335	A	0.2636	0.8676
8	rs2464592	118183551	G	0.2036	0.2317	A	0.2636	0.8479
9	rs1063192	22003367	G	0.2036	0.2317	A	0.2636	0.8479
1	rs1475064	73882478	A	0.2047	0.2325	G	0.2637	0.8495
6	rs1466749	170347127	A	0.4639	0.4308	G	0.2638	1.143
2	rs2304402	11774324	A	0.2938	0.2633	G	0.2639	1.164
2	rs16853306	168099796	G	0.2938	0.2633	A	0.2639	1.164
16	rs11150624	31476458	A	0.4149	0.4483	G	0.2639	0.8727
6	rs2844529	31353593	A	0.4124	0.445	G	0.264	0.8752
6	rs4816	150114745	G	0.2474	0.2192	A	0.264	1.171
17	rs4792801	16845467	A	0.4124	0.4449	G	0.264	0.8755
1	exm11606	9030964	G	0.4665	0.4333	A	0.2641	1.143
1	rs3737665	9030964	G	0.4665	0.4333	A	0.2641	1.143
6	rs1126533	33037640	A	0.4665	0.4333	G	0.2641	1.143
11	rs1912143	24544761	G	0.4665	0.4333	A	0.2641	1.143
17	rs8070414	63428940	C	0.4175	0.4517	A	0.2641	0.8702
18	rs34458979	41148471	G	0.4665	0.4333	A	0.2641	1.143
21	rs17001239	27002377	A	0.1778	0.1533	G	0.2641	1.194
4	rs1518485	73470972	G	0.4175	0.4508	A	0.2642	0.8732
6	rs2844513	31388214	G	0.4793	0.4458	A	0.2643	1.144
10	rs2165468	3516105	A	0.4691	0.4358	C	0.2643	1.144
2	rs3924521	45995181	G	0.0567	0.0425	A	0.2644	1.354
12	rs710415	7023123	G	0.4149	0.4475	A	0.2644	0.8757
14	rs143477571	76905712	G	0.0567	0.0425	A	0.2644	1.354
1	rs34160967	6635306	A	0.3557	0.3242	G	0.2645	1.151
1	rs6658576	56035952	A	0.4716	0.4383	G	0.2645	1.144
2	rs16853330	168106246	A	0.2938	0.2642	G	0.2645	1.159

6	rs1736926	29692562	A	0.04381	0.03167	G	0.2645	1.401
6	rs1610613	29710963	A	0.04381	0.03167	G	0.2645	1.401
6	rs733841	29717980	A	0.04381	0.03167	G	0.2645	1.401
6	rs1737079	29724745	G	0.04381	0.03167	A	0.2645	1.401
6	rs1610628	29728209	C	0.04381	0.03167	A	0.2645	1.401
6	rs1625636	29732302	A	0.04381	0.03167	G	0.2645	1.401
12	rs770087	89744773	C	0.04381	0.03167	A	0.2645	1.401
12	rs138805411	95456367	G	0.04381	0.03167	A	0.2645	1.401
18	rs470490	63031236	C	0.4201	0.4533	A	0.2645	0.8736
22	rs1894473	30221201	A	0.4201	0.4533	G	0.2645	0.8736
6	rs2274658	46801071	A	0.1495	0.1275	C	0.2646	1.203
6	rs7770628	161018174	G	0.1495	0.1275	A	0.2646	1.203
12	rs78905764	133306710	A	0.1495	0.1275	G	0.2646	1.203
16	rs17817449	53813367	C	0.1495	0.1275	A	0.2646	1.203
19	rs7508222	9059808	A	0.1495	0.1275	G	0.2646	1.203
19	rs2547063	9062544	A	0.1495	0.1275	C	0.2646	1.203
1	rs10888501	152537954	A	0.4175	0.45	G	0.2647	0.8761
18	rs2685428	69487009	A	0.2088	0.1833	G	0.2647	1.175
2	rs2075252	170010985	G	0.4227	0.4558	A	0.2648	0.874
4	rs6824106	178801018	A	0.4742	0.4408	G	0.2648	1.144
5	rs3749671	154346296	A	0.04639	0.0625	G	0.2648	0.7297
6	rs1536500	33723383	A	0.04639	0.0625	G	0.2648	0.7297
9	rs3088235	116132334	A	0.116	0.1392	G	0.2648	0.8115
9	rs17519205	132720770	G	0.116	0.1392	A	0.2648	0.8115
13	rs7986001	114830132	A	0.4253	0.4592	G	0.2648	0.8715
15	rs2934442	57762793	G	0.04639	0.0625	A	0.2648	0.7297
16	rs257636	58757683	A	0.4742	0.4408	G	0.2648	1.144
18	rs1074554	36902638	C	0.4253	0.4592	A	0.2648	0.8715
19	rs4805298	29396344	A	0.4227	0.4558	G	0.2648	0.874
11	rs118052160	58190415	A	0.04381	0.03172	G	0.2649	1.399
12	rs2730753	119171974	G	0.3093	0.3417	A	0.265	0.8628
15	rs62022931	57029988	A	0.3093	0.3417	G	0.265	0.8628
7	rs11768654	141286885	A	0.4253	0.4583	G	0.2651	0.8744
18	rs2249810	28734749	A	0.3093	0.3408	G	0.2651	0.866
6	rs28421666	32592737	G	0.04639	0.06333	A	0.2652	0.7195
8	rs6996401	123348238	A	0.25	0.28	G	0.2652	0.8571
16	rs201072221	75563732	A	0.04639	0.06333	G	0.2652	0.7195
1	rs2782643	43886494	A	0.1057	0.08667	G	0.2653	1.245
2	rs4907240	97228039	A	0.4742	0.4417	G	0.2653	1.14
4	rs2089990	93300188	A	0.3557	0.325	C	0.2653	1.146
2	rs6544972	47454527	A	0.2062	0.235	G	0.2654	0.8455
2	rs724568	67942480	C	0.2062	0.235	A	0.2654	0.8455
8	rs285799	106118299	G	0.482	0.4483	A	0.2654	1.145

18	rs8092429	73990475	G	0.4278	0.4608	A	0.2654	0.8749
2	rs4849105	111591331	A	0.4768	0.4442	G	0.2655	1.14
10	rs3765101	3197856	C	0.2938	0.265	G	0.2655	1.154
12	rs11056390	15332867	G	0.4456	0.4791	A	0.2655	0.8738
18	rs2289520	61160320	G	0.2062	0.2342	C	0.2655	0.8495
21	rs1475591	32440540	A	0.08763	0.07	G	0.2655	1.276
6	rs1406957	109954252	G	0.2113	0.185	A	0.2656	1.181
4	rs6552496	182293083	C	0.4794	0.4467	A	0.2657	1.141
15	rs2573652	100514614	A	0.4794	0.4467	G	0.2657	1.141
2	rs138165082	37296064	A	0.0232	0.015	C	0.2658	1.559
2	rs992822	186654592	A	0.4688	0.5017	G	0.2658	0.8765
3	rs6775745	131813900	A	0.1778	0.1542	G	0.2658	1.187
3	rs12695036	194338423	G	0.1778	0.1542	A	0.2658	1.187
5	rs145194026	39377375	A	0.0232	0.015	G	0.2658	1.559
6	rs146992026	13620487	A	0.0232	0.015	G	0.2658	1.559
6	rs240736	75848181	G	0.1778	0.1542	A	0.2658	1.187
6	rs188337991	138638442	G	0.0232	0.015	A	0.2658	1.559
8	rs374957	90837630	A	0.25	0.2217	G	0.2658	1.17
9	rs3750427	35548479	A	0.0232	0.015	G	0.2658	1.559
9	rs148626718	95378176	A	0.0232	0.015	G	0.2658	1.559
11	rs78826049	10648029	A	0.0232	0.015	C	0.2658	1.559
12	rs187856936	108145411	A	0.0232	0.015	G	0.2658	1.559
13	rs7333181	112221297	A	0.0232	0.015	G	0.2658	1.559
15	rs116682844	44107202	A	0.0232	0.015	C	0.2658	1.559
16	rs141675661	4790503	A	0.0232	0.015	G	0.2658	1.559
16	rs188574195	67682157	A	0.0232	0.015	G	0.2658	1.559
19	rs200504060	15290031	A	0.0232	0.015	G	0.2658	1.559
19	rs892055	38912764	A	0.4897	0.4558	G	0.2658	1.146
22	rs80100118	50277352	C	0.0232	0.015	A	0.2658	1.559
22	rs5771107	50658698	A	0.25	0.2217	T	0.2658	1.17
2	rs7578326	227020653	G	0.1418	0.1667	A	0.2659	0.8258
11	rs1216183	100226883	A	0.3119	0.3442	T	0.2659	0.8636
11	rs6590022	123814478	G	0.1418	0.1667	A	0.2659	0.8258
12	rs1622965	30427049	G	0.1418	0.1667	A	0.2659	0.8258
4	rs12644356	66232761	C	0.4845	0.4517	A	0.266	1.141
6	rs931312	155451352	A	0.3119	0.3433	G	0.266	0.8668
11	rs2640779	108381247	G	0.3119	0.3433	A	0.266	0.8668
2	rs4148211	44071743	A	0.1418	0.1675	G	0.2661	0.8209
4	rs1550057	142117424	A	0.3582	0.3275	G	0.2661	1.146
4	rs2332027	171672038	G	0.3582	0.3275	A	0.2661	1.146
7	rs3763505	48128850	G	0.4871	0.4542	A	0.2661	1.141
10	rs2766053	129123033	G	0.4871	0.4542	A	0.2661	1.141
2	rs12615742	37995727	G	0.4356	0.4683	A	0.2662	0.876

2	rs6712720	60281608	G	0.3608	0.3292	C	0.2662	1.15
8	rs7824377	72978593	G	0.4948	0.4608	A	0.2662	1.146
8	rs323346	30703431	G	0.09536	0.1167	A	0.2663	0.7981
8	rs323347	30706224	G	0.09536	0.1167	A	0.2663	0.7981
8	rs11136341	145043543	G	0.09536	0.1167	A	0.2663	0.7981
9	rs755109	100696203	G	0.1521	0.1292	A	0.2663	1.209
6	rs3130685	31206206	G	0.4459	0.48	A	0.2664	0.8717
6	rs1034240	97093037	A	0.4433	0.4767	G	0.2664	0.8743
1	rs3795524	214813782	G	0.09536	0.1175	A	0.2665	0.7917
1	rs3795523	214813941	C	0.09536	0.1175	G	0.2665	0.7917
1	rs3795522	214814125	A	0.09536	0.1175	G	0.2665	0.7917
1	rs3795519	214814733	C	0.09536	0.1175	A	0.2665	0.7917
1	rs12067133	214814995	A	0.09536	0.1175	G	0.2665	0.7917
1	rs3795517	214815916	G	0.09536	0.1175	A	0.2665	0.7917
1	rs3795514	214816297	G	0.09536	0.1175	A	0.2665	0.7917
1	rs3748692	214818215	A	0.09536	0.1175	G	0.2665	0.7917
1	rs3790646	214818846	G	0.09536	0.1175	A	0.2665	0.7917
1	rs3790647	214818945	C	0.09536	0.1175	A	0.2665	0.7917
5	rs835063	14989346	A	0.4459	0.4792	G	0.2665	0.8746
7	rs2708913	47979922	A	0.4459	0.4792	G	0.2665	0.8746
10	rs10904086	431161	G	0.4948	0.4617	A	0.2665	1.142
10	rs4750568	15008493	C	0.4459	0.4792	A	0.2665	0.8746
1	rs7552670	203768328	G	0.2526	0.2833	A	0.2666	0.8548
1	rs7540041	203769532	A	0.2526	0.2833	G	0.2666	0.8548
7	rs218966	11022230	A	0.451	0.485	G	0.2666	0.8724
8	rs608052	12879539	A	0.2526	0.2833	G	0.2666	0.8548
2	rs882924	60299010	C	0.4407	0.4733	A	0.2667	0.8768
17	rs8074150	65768543	G	0.5	0.4667	A	0.2667	1.143
20	rs17857295	3838441	G	0.4485	0.4817	C	0.2667	0.875
1	rs2494287	203207520	A	0.2964	0.2675	G	0.2668	1.154
3	rs57326399	38768300	G	0.451	0.4841	A	0.2668	0.8754
11	rs10792837	85905715	G	0.3144	0.3467	A	0.2668	0.8644
19	rs525420	7936208	A	0.5026	0.4692	G	0.2668	1.143
20	rs6066384	36071598	G	0.3144	0.3467	A	0.2668	0.8644
1	rs195204	115734091	G	0.4433	0.4758	A	0.2669	0.8772
4	rs6823706	63448482	G	0.5077	0.4742	A	0.2669	1.144
4	rs764205	110790911	T	0.451	0.4842	A	0.2669	0.8753
5	rs1919519	136816816	G	0.5077	0.4742	A	0.2669	1.144
5	rs3213094	158750769	A	0.5052	0.4717	G	0.2669	1.143
8	rs4738252	73379450	A	0.5077	0.4742	G	0.2669	1.144
15	rs560191	43767774	C	0.4433	0.4758	G	0.2669	0.8772
15	rs12900805	90174824	A	0.3608	0.33	G	0.2669	1.146
1	rs2295283	12082926	G	0.5129	0.4792	A	0.267	1.144

1	rs2358996	114515717	A	0.5129	0.4792	G	0.267	1.144
6	rs2747457	29656417	C	0.3634	0.3317	A	0.267	1.15
3	rs10935120	134233092	A	0.1804	0.1558	G	0.2671	1.192
5	rs1801394	7870973	G	0.299	0.2692	A	0.2671	1.158
7	rs740250	28996944	A	0.1804	0.1558	G	0.2671	1.192
7	rs10260404	154210798	G	0.1804	0.1558	A	0.2671	1.192
9	rs683866	79322068	A	0.4459	0.4783	C	0.2671	0.8775
10	rs7919322	15003278	A	0.4459	0.4783	G	0.2671	0.8775
10	rs7342028	114479262	C	0.5206	0.4867	A	0.2671	1.146
11	rs10741838	20447419	G	0.4459	0.4783	A	0.2671	0.8775
11	rs7121633	34032330	A	0.4639	0.4975	G	0.2671	0.8741
12	rs1852450	16397734	C	0.5232	0.4892	A	0.2671	1.146
12	rs3915247	16400613	G	0.5232	0.4892	A	0.2671	1.146
1	rs12740374	109817590	A	0.07216	0.05583	C	0.2672	1.315
1	rs7527925	225534219	A	0.4974	0.465	G	0.2672	1.139
1	rs7535953	225534348	G	0.4974	0.465	A	0.2672	1.139
4	rs4292399	116490478	G	0.4974	0.465	A	0.2672	1.139
6	rs2071544	32806121	A	0.4562	0.4892	G	0.2672	0.876
6	rs9322225	150184882	G	0.2139	0.1871	C	0.2672	1.182
7	rs1861141	154060445	A	0.07216	0.05583	G	0.2672	1.315
11	rs10839616	6790028	G	0.1727	0.1992	C	0.2672	0.8393
16	rs1079349	8873493	G	0.4562	0.4892	A	0.2672	0.876
16	rs12599795	55906063	G	0.4562	0.4892	A	0.2672	0.876
3	rs4374540	83546019	C	0.5	0.4675	A	0.2673	1.139
5	rs160632	96503523	A	0.4716	0.505	G	0.2673	0.875
9	rs10991359	107456763	G	0.4742	0.5075	A	0.2673	0.8753
10	rs6601764	3862542	A	0.2088	0.2375	G	0.2673	0.8471
11	rs494791	82996986	A	0.5026	0.47	C	0.2673	1.139
20	rs6075544	19598493	A	0.4588	0.4917	G	0.2673	0.8764
1	rs7533906	80134123	A	0.5232	0.49	G	0.2674	1.142
5	rs6876143	17380270	G	0.5103	0.4775	A	0.2674	1.14
6	rs1150739	30031345	A	0.5052	0.4725	G	0.2674	1.14
6	rs2844472	31589676	G	0.5206	0.4875	A	0.2674	1.142
8	rs4739525	37445639	G	0.5103	0.4775	A	0.2674	1.14
8	rs6998760	87076520	C	0.5129	0.48	A	0.2674	1.141
10	rs11598592	123043389	G	0.5155	0.4825	A	0.2674	1.141
11	rs224630	32087108	A	0.4613	0.4942	G	0.2674	0.8767
14	rs2378858	31863750	A	0.5206	0.4875	G	0.2674	1.142
22	rs138777	35711098	A	0.5103	0.4775	G	0.2674	1.14
6	rs2502601	158517308	G	0.4639	0.4967	A	0.2676	0.877
14	rs74062730	70991650	A	0.4639	0.4967	C	0.2676	0.877
10	rs4897783	133608237	A	0.4665	0.4992	G	0.2677	0.8773
11	rs11605546	94261280	A	0.1289	0.1075	G	0.2677	1.228

12	rs11831360	10215654	G	0.1289	0.1075	C	0.2677	1.228
13	rs1747109	45630008	G	0.317	0.3492	A	0.2677	0.8652
1	rs6695185	79299411	G	0.4691	0.5017	A	0.2678	0.8776
2	rs17228441	186627943	A	0.4716	0.5042	G	0.2678	0.8779
2	rs60029104	186658056	G	0.4716	0.5042	A	0.2678	0.8779
2	rs10490391	186658438	G	0.4716	0.5042	A	0.2678	0.8779
2	rs10490392	186658565	C	0.4716	0.5042	A	0.2678	0.8779
2	rs10931200	186664963	C	0.4716	0.5042	A	0.2678	0.8779
2	rs11695215	186665824	A	0.4716	0.5042	G	0.2678	0.8779
2	rs17826534	186671357	G	0.4716	0.5042	A	0.2678	0.8779
2	rs1862066	186671912	G	0.4716	0.5042	A	0.2678	0.8779
5	rs27324	52550580	G	0.4691	0.5017	A	0.2678	0.8776
6	rs9268645	32408527	C	0.4716	0.5042	G	0.2678	0.8779
7	rs62490396	140080087	G	0.4716	0.5042	C	0.2678	0.8779
20	rs11274	42826379	A	0.4691	0.5017	G	0.2678	0.8776
4	rs2725236	88919106	G	0.366	0.3342	A	0.2679	1.15
9	rs613391	22670715	G	0.317	0.3483	C	0.2679	0.8683
11	rs117805838	93844989	A	0.07732	0.09667	G	0.2679	0.7831
20	rs2076026	43942676	G	0.366	0.3344	A	0.2679	1.149
10	rs3824700	26355906	A	0.2552	0.2858	G	0.268	0.8559
22	rs68178377	50752150	A	0.2139	0.1875	G	0.268	1.179
11	rs7104098	42458745	G	0.2552	0.2846	A	0.2681	0.8609
18	rs12966340	23584044	G	0.299	0.27	A	0.2681	1.153
3	rs4596126	13659897	C	0.1521	0.13	A	0.2682	1.2
6	rs6930977	30435288	G	0.1521	0.13	A	0.2682	1.2
11	rs4245191	119052826	C	0.1521	0.13	A	0.2682	1.2
12	rs2166807	32134638	A	0.1521	0.13	G	0.2682	1.2
16	rs8050136	53816275	A	0.1521	0.13	C	0.2682	1.2
16	rs9939609	53820527	A	0.1521	0.13	T	0.2682	1.2
19	rs7507593	2888242	A	0.3015	0.2717	G	0.2684	1.157
1	rs12094334	201176108	A	0.366	0.335	G	0.2685	1.146
1	rs12063867	201176968	G	0.366	0.335	A	0.2685	1.146
1	rs7551098	201177278	A	0.366	0.335	G	0.2685	1.146
1	rs12070918	201181217	G	0.366	0.335	A	0.2685	1.146
10	rs1900500	118448078	A	0.3196	0.3517	G	0.2685	0.8659
1	rs7551538	201177538	A	0.3686	0.3367	C	0.2687	1.15
3	rs1371924	143250070	G	0.1186	0.1417	A	0.2687	0.8149
11	rs6591561	60070176	G	0.3686	0.3367	A	0.2687	1.15
5	rs2269287	83360520	A	0.1082	0.08833	G	0.2688	1.253
6	rs205982	164421002	A	0.1082	0.08833	G	0.2688	1.253
3	rs4680534	159698945	G	0.1443	0.1692	A	0.2689	0.8284
6	rs9368624	30135977	A	0.3057	0.2752	G	0.2689	1.16
11	rs1129782	2943671	G	0.1186	0.1425	C	0.2689	0.8094

12	rs2075033	644337	A	0.1443	0.1692	G	0.2689	0.8284
12	rs2230281	89917518	A	0.1443	0.1692	G	0.2689	0.8284
12	rs1132780	121691096	A	0.2113	0.24	G	0.2691	0.8486
16	rs12927214	57957216	G	0.01036	0.005	A	0.2692	2.084
22	rs114778719	45312455	A	0.01036	0.005	G	0.2692	2.084
1	rs78303930	156640385	C	0.01031	0.02	G	0.2693	0.5104
1	rs2793086	231899704	C	0.01031	0.02	A	0.2693	0.5104
1	rs56229136	231906773	G	0.01031	0.02	C	0.2693	0.5104
3	rs148849582	49313893	A	0.01031	0.02	G	0.2693	0.5104
3	rs7635839	191667777	A	0.01031	0.02	G	0.2693	0.5104
4	rs3822227	13601443	A	0.01031	0.02	G	0.2693	0.5104
5	rs34916955	96512854	A	0.01031	0.02	G	0.2693	0.5104
6	rs34643516	144780437	A	0.01031	0.02	G	0.2693	0.5104
9	rs2237857	35076755	A	0.01031	0.02	G	0.2693	0.5104
10	rs200560789	24959317	G	0.01031	0.02	A	0.2693	0.5104
10	rs11187583	95441272	A	0.01031	0.02	G	0.2693	0.5104
12	rs1545843	84564068	A	0.3686	0.3375	G	0.2693	1.146
13	rs548097	75776268	C	0.01031	0.02	A	0.2693	0.5104
14	rs1042704	23312594	A	0.01031	0.02	G	0.2693	0.5104
15	rs8033476	49952940	G	0.2139	0.1883	A	0.2693	1.173
15	rs149100994	83932408	G	0.01031	0.02	A	0.2693	0.5104
17	rs4581	64210757	C	0.2139	0.1883	A	0.2693	1.173
17	rs79354765	73897344	A	0.01031	0.02	G	0.2693	0.5104
17	rs149105732	73917372	G	0.01031	0.02	A	0.2693	0.5104
19	rs145973159	52249695	G	0.01031	0.02	A	0.2693	0.5104
1	rs34625170	117503940	A	0.3222	0.3542	G	0.2694	0.8667
6	rs2395730	39784365	C	0.3222	0.3542	A	0.2694	0.8667
3	rs61743461	33195309	G	0.2552	0.2267	A	0.2695	1.169
5	rs6451953	21437412	C	0.3711	0.3392	A	0.2695	1.15
5	rs10475598	173648438	A	0.2552	0.2267	G	0.2695	1.169
2	rs2683825	142109239	A	0.1753	0.2017	C	0.2696	0.8412
4	rs4698804	110940045	G	0.1753	0.2025	A	0.2697	0.8369
14	rs144163059	20444162	A	0.01036	0.005008	G	0.2697	2.08
7	rs35897481	30831088	A	0.1289	0.1083	G	0.2698	1.218
4	rs886532	5527102	A	0.06186	0.08	C	0.2699	0.7582
15	rs201865787	89760506	A	0.01554	0.009182	G	0.2699	1.704
17	rs74372573	79181034	A	0.01053	0.0201	G	0.27	0.5186
6	rs2523756	29820717	G	0.3711	0.34	A	0.2701	1.146
6	rs1611699	29827753	T	0.3711	0.34	A	0.2701	1.146
6	rs1611732	29831008	A	0.3711	0.34	G	0.2701	1.146
8	rs2053931	102013597	A	0.3711	0.34	G	0.2701	1.146
4	rs28489116	187542755	G	0.3247	0.3567	A	0.2702	0.8674
6	rs2074469	29407970	G	0.2165	0.19	A	0.2703	1.178

6	rs9257863	29425584	C	0.2165	0.19	A	0.2703	1.178
6	rs1345228	29432390	G	0.2165	0.19	A	0.2703	1.178
6	rs734960	29475142	A	0.2165	0.19	G	0.2703	1.178
7	rs445	92408370	A	0.3737	0.3417	G	0.2703	1.15
11	rs1032939	60531346	A	0.3737	0.3417	C	0.2703	1.15
3	rs10510837	60289842	A	0.0567	0.04333	G	0.2704	1.327
1	rs41305876	57254697	A	0.01031	0.005	G	0.2706	2.073
1	rs147851396	186094772	A	0.01031	0.005	G	0.2706	2.073
1	rs117147433	228432047	A	0.06186	0.08083	C	0.2706	0.7497
2	rs186563338	1652377	A	0.01031	0.005	G	0.2706	2.073
2	rs6707600	24521766	G	0.01031	0.005	A	0.2706	2.073
2	rs2272466	26802230	A	0.01031	0.005	T	0.2706	2.073
2	rs141714250	27352794	A	0.01031	0.005	G	0.2706	2.073
2	rs57257569	43520160	C	0.01031	0.005	G	0.2706	2.073
2	rs2278206	99172244	G	0.3041	0.275	A	0.2706	1.152
2	rs144499152	207635935	G	0.01031	0.005	A	0.2706	2.073
2	rs16859382	219693856	G	0.01031	0.005	A	0.2706	2.073
2	rs201590115	232389975	A	0.01031	0.005	C	0.2706	2.073
3	rs151099815	5255207	A	0.01031	0.005	G	0.2706	2.073
3	rs116965256	16358693	A	0.01031	0.005	C	0.2706	2.073
3	rs193072142	44489264	A	0.01031	0.005	G	0.2706	2.073
4	rs7673955	12440385	A	0.01031	0.005	G	0.2706	2.073
4	rs79402270	88583405	A	0.06186	0.08083	C	0.2706	0.7497
6	rs201931731	69728298	G	0.01031	0.005	A	0.2706	2.073
6	rs146188768	96974210	A	0.01031	0.005	G	0.2706	2.073
6	rs77703807	166572039	A	0.01031	0.005	G	0.2706	2.073
7	rs2691543	77964788	G	0.01031	0.005	A	0.2706	2.073
7	rs201123128	142983743	G	0.01031	0.005	A	0.2706	2.073
8	rs150053915	82713734	G	0.01031	0.005	A	0.2706	2.073
9	rs200377723	117186681	A	0.01031	0.005	G	0.2706	2.073
10	rs144456901	48414516	A	0.01031	0.005	T	0.2706	2.073
10	rs148949699	99349802	A	0.01031	0.005	G	0.2706	2.073
11	rs183069692	62301444	A	0.01031	0.005	G	0.2706	2.073
11	rs17847835	67266466	G	0.01031	0.005	C	0.2706	2.073
11	rs76191655	71906438	A	0.01031	0.005	G	0.2706	2.073
11	rs12146610	102988504	T	0.01031	0.005	A	0.2706	2.073
11	rs78031342	129722549	A	0.01031	0.005	G	0.2706	2.073
12	rs79165229	55641811	A	0.01031	0.005	G	0.2706	2.073
12	rs7965277	80900348	C	0.06186	0.08083	A	0.2706	0.7497
12	rs4300440	109633316	G	0.2603	0.2908	A	0.2706	0.8581
12	rs188735557	118588826	T	0.01031	0.005	A	0.2706	2.073
14	rs140363428	59821916	A	0.01031	0.005	G	0.2706	2.073
15	rs8041675	37342602	G	0.2603	0.2908	A	0.2706	0.8581

15	rs114649602	52230365	T	0.01031	0.005	A	0.2706	2.073
15	rs184326854	102191929	A	0.01031	0.005	G	0.2706	2.073
16	rs201250928	20976150	G	0.01031	0.005	A	0.2706	2.073
17	rs12450112	12205234	A	0.2603	0.2908	G	0.2706	0.8581
17	rs150994435	73888900	A	0.01031	0.005	T	0.2706	2.073
19	rs200879429	4323195	A	0.01031	0.005	G	0.2706	2.073
19	rs201855801	15220100	A	0.01031	0.005	G	0.2706	2.073
19	rs146402867	32845370	A	0.01031	0.005	G	0.2706	2.073
19	rs184705974	51582741	A	0.01031	0.005	G	0.2706	2.073
19	rs200612878	51770638	G	0.01031	0.005	A	0.2706	2.073
19	rs17854523	54387497	A	0.01031	0.005	G	0.2706	2.073
20	rs199581355	44756839	G	0.01031	0.005	A	0.2706	2.073
22	rs201416955	29881766	A	0.01031	0.005	G	0.2706	2.073
22	rs151071851	31924729	G	0.01031	0.005	A	0.2706	2.073
22	rs183406976	32019741	A	0.01031	0.005	G	0.2706	2.073
22	rs143392071	44328930	G	0.01031	0.005	A	0.2706	2.073
1	rs138692279	60466808	A	0.005155	0.01333	G	0.2708	0.3834
1	rs1146320	119016240	C	0.2603	0.29	A	0.2708	0.8616
1	rs116959874	158605725	A	0.005155	0.01333	G	0.2708	0.3834
1	rs115968198	161130579	A	0.005155	0.01333	G	0.2708	0.3834
1	rs2504057	179687259	G	0.3737	0.3425	A	0.2708	1.146
1	rs116879071	186275857	A	0.005155	0.01333	G	0.2708	0.3834
1	rs190061594	225341473	G	0.005155	0.01333	A	0.2708	0.3834
2	rs151203280	55445061	A	0.005155	0.01333	G	0.2708	0.3834
2	rs201369797	240984709	A	0.005155	0.01333	G	0.2708	0.3834
3	rs12637996	49049391	A	0.005155	0.01333	G	0.2708	0.3834
4	rs144030215	6081975	A	0.005155	0.01333	G	0.2708	0.3834
4	rs145872263	38776355	G	0.005155	0.01333	A	0.2708	0.3834
6	rs17419851	24749413	A	0.005155	0.01333	G	0.2708	0.3834
6	rs149645105	97609932	A	0.005155	0.01333	G	0.2708	0.3834
6	rs218867	121398535	G	0.3737	0.3425	A	0.2708	1.146
8	rs187998469	10465286	A	0.005155	0.01333	G	0.2708	0.3834
8	rs143580028	53062395	G	0.005155	0.01333	A	0.2708	0.3834
9	rs117881664	14770787	C	0.005155	0.01333	A	0.2708	0.3834
9	rs188943134	15474122	A	0.005155	0.01333	G	0.2708	0.3834
9	rs144710877	99413954	C	0.005155	0.01333	A	0.2708	0.3834
9	rs150077107	139635287	A	0.005155	0.01333	G	0.2708	0.3834
10	rs116840175	118364914	A	0.005155	0.01333	G	0.2708	0.3834
11	rs115707133	57144356	A	0.005155	0.01333	C	0.2708	0.3834
11	rs148437608	60617426	A	0.005155	0.01333	G	0.2708	0.3834
11	rs80358282	86665923	A	0.005155	0.01333	G	0.2708	0.3834
11	rs187196207	103062861	G	0.005155	0.01333	A	0.2708	0.3834
12	rs151134407	666923	A	0.005155	0.01333	G	0.2708	0.3834

14	rs114668642	81329198	A	0.005155	0.01333	G	0.2708	0.3834
16	rs138948625	20809773	G	0.005155	0.01333	A	0.2708	0.3834
19	rs77168423	1399937	A	0.005155	0.01336	G	0.2708	0.3828
19	rs142760960	8181682	A	0.005155	0.01333	G	0.2708	0.3834
19	rs61738876	58213826	A	0.005155	0.01333	G	0.2708	0.3834
20	rs138278466	2560676	G	0.005155	0.01333	A	0.2708	0.3834
20	rs117489377	3624844	G	0.005155	0.01333	A	0.2708	0.3834
22	rs117291545	36894133	G	0.005155	0.01333	C	0.2708	0.3834
3	rs1673607	138289221	G	0.07216	0.05667	A	0.271	1.295
11	rs55990541	8159857	A	0.07216	0.05667	G	0.271	1.295
14	rs973968	62654698	G	0.07216	0.05667	A	0.271	1.295
14	rs8018360	92987358	A	0.2139	0.2425	G	0.271	0.8501
19	rs3752232	1043748	G	0.1082	0.08917	A	0.271	1.24
19	rs3764647	1044712	G	0.1082	0.08917	A	0.271	1.24
1	rs75680976	155006141	C	0.01031	0.005008	G	0.2711	2.069
2	rs115380913	231048312	G	0.005155	0.0125	A	0.2711	0.4093
3	rs145385804	8590329	A	0.005155	0.0125	G	0.2711	0.4093
3	rs1511412	138713704	A	0.005155	0.0125	G	0.2711	0.4093
4	rs201057296	2933799	A	0.005155	0.0125	G	0.2711	0.4093
4	rs149865710	6302756	G	0.01031	0.005008	C	0.2711	2.069
4	rs74981230	71509672	C	0.005155	0.0125	A	0.2711	0.4093
4	rs893971	89203670	G	0.005155	0.0125	A	0.2711	0.4093
4	rs142621179	147724769	A	0.005155	0.0125	G	0.2711	0.4093
4	rs201305369	174235242	G	0.005155	0.0125	A	0.2711	0.4093
5	rs73815153	180432564	A	0.005155	0.0125	C	0.2711	0.4093
5	rs7726604	180432662	G	0.005155	0.0125	C	0.2711	0.4093
5	rs59220426	180432747	C	0.005155	0.0125	G	0.2711	0.4093
5	rs3806869	180485353	A	0.005155	0.0125	G	0.2711	0.4093
6	rs72821581	10756728	G	0.005155	0.0125	A	0.2711	0.4093
6	rs3130786	30807948	G	0.3763	0.3442	A	0.2711	1.15
6	rs117448081	32185857	G	0.005155	0.0125	C	0.2711	0.4093
6	rs117069761	41309788	A	0.005155	0.0125	G	0.2711	0.4093
6	rs2095381	155454811	A	0.005155	0.0125	G	0.2711	0.4093
7	rs802568	145959243	C	0.005155	0.0125	A	0.2711	0.4093
7	rs181262306	151668119	A	0.005155	0.0125	G	0.2711	0.4093
9	rs75645675	2718802	G	0.005155	0.0125	A	0.2711	0.4093
11	rs76159120	66191310	C	0.005155	0.0125	A	0.2711	0.4093
11	rs144055440	124742934	A	0.005155	0.0125	C	0.2711	0.4093
12	rs189251687	99020453	G	0.005155	0.0125	A	0.2711	0.4093
12	rs149064276	122398520	A	0.005155	0.0125	G	0.2711	0.4093
17	rs35901087	45916018	G	0.005155	0.0125	C	0.2711	0.4093
17	rs691652	76491127	A	0.005155	0.0125	G	0.2711	0.4093
18	rs116292140	2890649	A	0.005155	0.0125	G	0.2711	0.4093

19	rs75377748	3193375	C	0.005155	0.0125	A	0.2711	0.4093
19	rs145629439	48807255	A	0.005155	0.0125	G	0.2711	0.4093
22	rs146127576	37457585	A	0.005155	0.0125	G	0.2711	0.4093
22	rs147247708	45689118	A	0.005155	0.0125	G	0.2711	0.4093
5	rs148324440	109190841	A	0.005291	0.01333	G	0.2714	0.3936
17	rs142620798	73888898	A	0.01031	0.005017	G	0.2715	2.066
7	rs1202283	87082292	A	0.3763	0.345	G	0.2716	1.145
16	rs3751800	86566042	A	0.183	0.1592	G	0.2716	1.183
17	rs3744637	27030713	A	0.02577	0.03833	G	0.2716	0.6637
1	rs17368528	9324213	A	0.03608	0.05083	G	0.2717	0.699
4	rs138883861	69342021	A	0.03608	0.05083	G	0.2717	0.699
11	rs34009635	102584176	G	0.03608	0.05083	A	0.2717	0.699
15	rs79004308	42640302	A	0.03608	0.05083	T	0.2717	0.699
1	rs75658797	202287754	A	0.02577	0.03917	G	0.2718	0.649
2	rs1505229	77589901	G	0.3299	0.3617	A	0.2718	0.8689
2	rs7594727	97489870	A	0.1546	0.1325	C	0.2718	1.198
3	rs8147	186301703	G	0.1546	0.1325	A	0.2718	1.198
6	rs424232	32208324	A	0.3299	0.3617	G	0.2718	0.8689
15	rs2236722	51534995	G	0.02577	0.03917	A	0.2718	0.649
17	rs138527765	71357828	A	0.02577	0.03917	G	0.2718	0.649
21	rs71321355	32253629	A	0.02577	0.03917	G	0.2718	0.649
1	rs3795800	228505727	A	0.3789	0.3467	G	0.2719	1.15
6	rs2859100	32699479	G	0.3789	0.3467	C	0.2719	1.15
7	rs2195669	49435299	G	0.3789	0.3467	A	0.2719	1.15
5	rs16870720	41042204	A	0.2577	0.23	T	0.272	1.162
10	rs146819332	43882723	A	0.03351	0.02333	G	0.272	1.451
10	rs588517	83153246	A	0.03351	0.02333	G	0.272	1.451
12	rs17848835	109692053	A	0.03351	0.02333	G	0.272	1.451
15	rs188590145	53901720	G	0.03351	0.02333	A	0.272	1.451
17	rs1990236	11865462	A	0.1469	0.1717	G	0.272	0.8309
19	rs7507442	53278953	G	0.1469	0.1717	A	0.272	0.8309
7	rs56333627	21939032	A	0.1469	0.1725	C	0.2721	0.8261
3	rs12491921	106288950	G	0.3093	0.2792	A	0.2722	1.156
12	rs10771894	32134943	G	0.3789	0.3475	A	0.2723	1.145
2	rs17744093	29294754	C	0.1211	0.1442	G	0.2725	0.8182
6	rs2242668	31773821	A	0.1211	0.1442	G	0.2725	0.8182
10	rs4919641	104217503	A	0.2191	0.1925	C	0.2725	1.177
16	rs2228479	89985940	A	0.1211	0.1442	G	0.2725	0.8182
18	rs35033671	10759840	A	0.1314	0.1102	C	0.2725	1.222
22	rs9607469	37919267	A	0.1211	0.1442	G	0.2725	0.8182
6	rs2523721	30166266	A	0.1211	0.145	G	0.2726	0.8127
6	rs885912	30174633	A	0.1211	0.145	C	0.2726	0.8127
6	rs9345464	95032230	G	0.3325	0.3642	A	0.2726	0.8696

17	rs4889730	21717727	C	0.3325	0.3642	A	0.2726	0.8696
12	rs74641138	56848079	A	0.09021	0.0725	G	0.2727	1.268
17	rs1133326	2940759	A	0.1211	0.1452	G	0.2727	0.8111
19	rs1172822	55819845	A	0.09021	0.0725	G	0.2727	1.268
19	rs2286550	38861362	G	0.1856	0.1608	A	0.2729	1.189
2	rs117162487	240969182	A	0.03608	0.05167	G	0.273	0.6871
6	rs1611133	29809382	A	0.1571	0.135	G	0.273	1.194
8	rs896854	95960511	A	0.3093	0.28	G	0.273	1.151
13	rs1034200	23228691	A	0.3814	0.35	C	0.273	1.145
2	rs9808377	179421694	A	0.3325	0.3633	G	0.2731	0.8728
2	rs3829746	179427536	A	0.3325	0.3633	G	0.2731	0.8728
2	rs13306198	21260084	A	0.03608	0.05175	G	0.2732	0.6859
1	rs146128753	153750682	A	0.01031	0.02083	G	0.2733	0.4896
2	rs76455076	204022478	A	0.01031	0.02083	C	0.2733	0.4896
3	rs2289272	10413526	A	0.01031	0.02083	G	0.2733	0.4896
5	rs143692635	139938337	A	0.01031	0.02083	G	0.2733	0.4896
8	rs2230009	30921935	A	0.01031	0.02083	G	0.2733	0.4896
9	rs148438754	140446879	G	0.01031	0.02083	A	0.2733	0.4896
10	rs41284134	91066446	C	0.01031	0.02083	G	0.2733	0.4896
10	rs181201567	105762362	G	0.01031	0.02083	C	0.2733	0.4896
15	rs117804335	33261185	A	0.01031	0.02083	G	0.2733	0.4896
16	rs74988150	597764	A	0.01031	0.02083	G	0.2733	0.4896
16	rs115337501	57764895	A	0.01031	0.02083	G	0.2733	0.4896
5	rs4703129	97888736	C	0.3351	0.3675	A	0.2734	0.8672
11	rs607301	79683501	A	0.3351	0.3675	G	0.2734	0.8672
9	rs1327837	77283889	G	0.3119	0.2817	A	0.2735	1.156
11	rs584427	113103996	A	0.3119	0.2817	C	0.2735	1.156
14	rs141927799	70925102	A	0.01031	0.02087	G	0.2735	0.4888
19	rs8103847	30909447	A	0.2655	0.295	C	0.2735	0.8637
6	rs1548349	168459845	G	0.1572	0.1342	C	0.2736	1.204
12	rs2172873	103125690	A	0.0799	0.1	G	0.2736	0.7815
12	rs62644541	133780851	C	0.1572	0.1342	G	0.2736	1.204
6	rs6901900	132012886	C	0.384	0.3525	A	0.2737	1.145
15	rs11072532	75499861	G	0.2191	0.1933	A	0.2737	1.17
20	rs753381	39797465	A	0.2191	0.1933	G	0.2737	1.17
20	rs932472	51199343	G	0.2191	0.1933	A	0.2737	1.17
16	rs9930984	77353973	C	0.0799	0.09917	A	0.2739	0.7888
13	rs1765769	113529257	A	0.04897	0.06533	G	0.2741	0.7367
4	rs4699052	104137790	A	0.3376	0.3692	G	0.2742	0.871
9	rs13292136	81952128	A	0.1314	0.1108	G	0.2742	1.214
21	rs2832873	31812772	A	0.3376	0.3692	C	0.2742	0.871
3	rs1549979	85460131	G	0.1856	0.1617	A	0.2744	1.182
7	rs2293362	47408443	G	0.1856	0.1617	C	0.2744	1.182

16	rs255052	68024995	A	0.1856	0.1617	G	0.2744	1.182
1	rs11206510	55496039	G	0.04897	0.065	A	0.2745	0.7407
2	rs3770740	64779509	A	0.268	0.2983	G	0.2745	0.8613
3	rs7610607	24904904	G	0.2191	0.2475	A	0.2745	0.8529
10	rs1733724	54223977	A	0.04897	0.065	G	0.2745	0.7407
11	rs610037	65546857	C	0.384	0.3533	A	0.2745	1.141
17	rs16973424	66904001	C	0.04897	0.065	A	0.2745	0.7407
20	rs117961856	1235040	A	0.04897	0.065	G	0.2745	0.7407
1	rs710235	42202714	G	0.2629	0.2342	A	0.2747	1.166
3	rs13320571	43334020	G	0.04897	0.06583	A	0.2747	0.7306
7	rs1194182	80231504	C	0.3144	0.2842	G	0.2747	1.155
9	rs561970	79318677	A	0.04639	0.03333	G	0.2747	1.411
9	rs620552	79318998	A	0.04639	0.03333	G	0.2747	1.411
17	rs8074751	63642542	G	0.04639	0.03333	A	0.2747	1.411
17	rs1800309	78087041	A	0.268	0.2975	G	0.2748	0.8647
1	rs12033518	115797624	G	0.3892	0.3573	A	0.2749	1.146
3	rs1795145	116269592	G	0.3402	0.3717	A	0.275	0.8717
3	rs3816527	157155314	C	0.1495	0.1742	A	0.275	0.8334
1	rs1207935	60211466	A	0.1572	0.135	G	0.2753	1.195
5	rs32579	149210848	A	0.3144	0.285	G	0.2753	1.151
4	rs10516430	99337881	A	0.2629	0.235	G	0.2754	1.161
9	rs2475335	10260263	G	0.2629	0.235	A	0.2754	1.161
15	rs3751655	90771750	A	0.2629	0.235	C	0.2754	1.161
6	rs1226475	46976812	G	0.2228	0.1967	A	0.2755	1.171
10	rs10885556	116102242	G	0.1881	0.1633	A	0.2757	1.187
18	rs62086326	14106039	G	0.1891	0.1644	A	0.2757	1.185
11	rs7481951	22271870	T	0.1005	0.1225	A	0.2758	0.8005
11	rs2273117	33604885	A	0.3892	0.3583	G	0.2758	1.141
13	rs9319321	27415673	A	0.3892	0.3583	T	0.2758	1.141
6	rs1875845	80196848	A	0.317	0.2867	G	0.2759	1.155
6	rs3752536	130031215	A	0.317	0.2867	G	0.2759	1.155
7	rs1449592	96410250	A	0.3454	0.3775	G	0.2762	0.8699
15	rs2871865	99194896	G	0.09021	0.07333	C	0.2762	1.253
1	rs1494373	220929356	G	0.2216	0.25	A	0.2763	0.8543
7	rs59985563	138603282	G	0.1237	0.1475	A	0.2763	0.816
16	rs1453324	81190613	A	0.2216	0.25	G	0.2763	0.8543
3	rs3853735	168308799	A	0.3943	0.3625	G	0.2764	1.145
1	rs608339	89652078	T	0.399	0.3667	A	0.2765	1.147
1	rs2070150	161761276	C	0.3943	0.3629	G	0.2765	1.143
2	rs11677099	55254165	A	0.05928	0.045	T	0.2765	1.337
7	rs117142233	20201458	A	0.07474	0.05833	G	0.2765	1.304
7	exm624676	72093938	A	0.07474	0.05833	G	0.2765	1.304
13	rs9546785	36801415	A	0.3454	0.3767	G	0.2765	0.873

20	rs140977227	23401975	G	0.07474	0.05833	A	0.2765	1.304
11	rs7945565	45878992	G	0.183	0.21	A	0.2766	0.8426
21	rs11553518	47423040	G	0.1881	0.1642	A	0.2769	1.18
1	rs1470261	146694922	A	0.2655	0.2375	G	0.277	1.16
2	rs16853305	168099775	G	0.2655	0.2375	C	0.277	1.16
6	rs3819409	135525396	A	0.2732	0.3033	C	0.277	0.8633
2	rs1801262	182543455	A	0.06443	0.0825	G	0.2773	0.7659
6	rs1580345	32837696	C	0.3196	0.2896	A	0.2773	1.152
1	rs2609383	223116472	A	0.2732	0.3025	G	0.2774	0.8667
1	rs2789975	223116474	A	0.2732	0.3025	C	0.2774	0.8667
9	rs1751798	93941796	G	0.2732	0.3025	A	0.2774	0.8667
2	rs9789477	51723107	G	0.3505	0.3823	A	0.2775	0.872
12	rs10783975	59871710	A	0.3505	0.3823	G	0.2775	0.872
5	rs10059955	105425352	A	0.3505	0.3825	G	0.2776	0.8713
8	rs11995824	120012700	C	0.3969	0.3658	G	0.2776	1.141
19	rs1862458	9069792	A	0.3505	0.3825	G	0.2776	0.8713
19	rs1862460	9069892	C	0.3505	0.3825	A	0.2776	0.8713
3	rs73005814	150530065	A	0.3995	0.3675	G	0.2777	1.145
11	rs74522421	51411510	A	0.2277	0.2015	G	0.2777	1.169
4	rs4691644	156380807	C	0.3505	0.3817	A	0.2779	0.8743
12	rs11168459	48596241	G	0.2242	0.2525	A	0.278	0.8557
12	rs17122812	48597053	G	0.2242	0.2525	A	0.278	0.8557
19	rs7260180	45028169	A	0.2242	0.1983	G	0.278	1.168
1	rs2275654	234529446	G	0.01804	0.02917	A	0.2781	0.6115
2	rs2072590	177042633	A	0.2242	0.2533	C	0.2781	0.8519
2	rs78964459	237300650	A	0.01804	0.02917	G	0.2781	0.6115
5	rs146525982	86564477	G	0.01804	0.02917	A	0.2781	0.6115
6	rs2858880	32704884	A	0.01804	0.02917	T	0.2781	0.6115
11	rs3763853	62473044	A	0.01804	0.02917	G	0.2781	0.6115
12	rs7297130	113659260	G	0.2242	0.2533	A	0.2781	0.8519
19	rs73509026	12059467	G	0.01804	0.02917	C	0.2781	0.6115
19	rs3745672	12146370	G	0.01804	0.02917	A	0.2781	0.6115
19	rs34971363	54313707	G	0.01804	0.02917	C	0.2781	0.6115
2	rs2139118	35952305	G	0.3995	0.3683	A	0.2782	1.141
5	rs2431363	75948650	G	0.2655	0.2383	A	0.2782	1.155
5	rs949974	123853591	A	0.2758	0.3058	C	0.2782	0.8643
13	rs314530	89921892	A	0.2655	0.2383	C	0.2782	1.155
19	rs3733829	41310571	G	0.3531	0.385	A	0.2783	0.8719
1	rs2798109	49002721	A	0.2758	0.305	C	0.2786	0.8677
15	rs3743143	40750740	G	0.134	0.1133	A	0.2786	1.211
19	rs1035938	48183771	A	0.2758	0.305	G	0.2786	0.8677
15	rs146054360	78635889	A	0.005848	0.01523	G	0.2787	0.3804
3	rs6777976	16327909	A	0.1598	0.1375	G	0.2788	1.193

6	rs2071476	32825379	G	0.4021	0.3708	A	0.2788	1.141
17	rs35612698	39884065	A	0.1598	0.1375	C	0.2788	1.193
21	rs74369337	15524921	A	0.3222	0.2925	G	0.2788	1.15
1	rs2811893	59162148	G	0.4046	0.3725	A	0.2789	1.145
9	rs4741510	15591372	T	0.4046	0.3725	A	0.2789	1.145
1	rs2304778	9994845	A	0.01804	0.01	G	0.279	1.819
1	rs200715189	151535130	A	0.01804	0.01	G	0.279	1.819
1	rs185953741	236720535	A	0.01804	0.01	G	0.279	1.819
2	rs201718270	85576653	G	0.01804	0.01	A	0.279	1.819
2	rs143502096	168100993	A	0.01804	0.01	G	0.279	1.819
3	rs200143538	9515062	G	0.01804	0.01	A	0.279	1.819
3	rs35625434	10084828	A	0.01804	0.01	G	0.279	1.819
3	rs149125003	10088285	C	0.01804	0.01	A	0.279	1.819
3	rs185298481	127318207	C	0.01804	0.01	G	0.279	1.819
4	rs143947747	15591190	G	0.01804	0.01	C	0.279	1.819
4	rs7671281	71509086	G	0.01804	0.01	A	0.279	1.819
4	rs3796704	71509431	A	0.01804	0.01	G	0.279	1.819
5	rs62000998	653145	G	0.01804	0.01	C	0.279	1.819
5	rs75134564	41862758	A	0.01804	0.01	G	0.279	1.819
5	rs115472345	127514316	A	0.01804	0.01	G	0.279	1.819
6	rs973233	33140009	A	0.01804	0.01	C	0.279	1.819
6	rs213212	33185918	C	0.01804	0.01	A	0.279	1.819
7	rs1568773	1248880	G	0.01804	0.01	A	0.279	1.819
8	rs57699806	27362587	A	0.01804	0.01	G	0.279	1.819
9	rs11793196	34500821	A	0.01804	0.01	G	0.279	1.819
9	rs117349601	108097888	A	0.01804	0.01	G	0.279	1.819
10	rs199694570	45472899	A	0.01804	0.01	G	0.279	1.819
12	rs10743347	20154110	G	0.01804	0.01	A	0.279	1.819
13	rs11571653	32910842	G	0.01804	0.01	A	0.279	1.819
13	rs17592438	103385534	A	0.01804	0.01	G	0.279	1.819
13	rs117878269	114325855	A	0.01804	0.01	G	0.279	1.819
15	rs191257274	90904421	G	0.01804	0.01	A	0.279	1.819
17	rs190850127	3343611	C	0.01804	0.01	A	0.279	1.819
17	rs200432202	7192869	C	0.01804	0.01	G	0.279	1.819
17	rs182345537	46128778	A	0.01804	0.01	C	0.279	1.819
19	rs140843177	52033031	A	0.01804	0.01	G	0.279	1.819
19	rs17305269	54578328	G	0.01804	0.01	A	0.279	1.819
19	rs35336528	54968038	G	0.01804	0.01	A	0.279	1.819
20	rs201552233	33447262	G	0.01804	0.01	A	0.279	1.819
21	rs199543737	42813714	A	0.01804	0.01	G	0.279	1.819
22	rs148203655	24717411	A	0.01804	0.01	G	0.279	1.819
15	rs1376785	35112710	G	0.2268	0.2	A	0.2792	1.173
16	rs191685707	2160358	A	0.01804	0.01002	G	0.2792	1.816

17	rs3809875	1703543	C	0.2268	0.2	G	0.2792	1.173
8	rs975730	129316014	G	0.2784	0.3092	A	0.2794	0.8619
12	rs17860364	51723499	G	0.08247	0.1025	A	0.2794	0.7871
12	rs17860287	51739648	C	0.08247	0.1025	G	0.2794	0.7871
20	rs1055246	52561469	G	0.08247	0.1025	A	0.2794	0.7871
1	rs763821	15668276	A	0.01804	0.03	G	0.2795	0.5941
1	rs147586507	39782063	A	0.01804	0.03	G	0.2795	0.5941
2	rs138660785	99342785	A	0.01804	0.03	G	0.2795	0.5941
4	rs149434359	184205482	A	0.01804	0.03	G	0.2795	0.5941
5	rs3985058	111756424	C	0.01804	0.03	A	0.2795	0.5941
10	rs75004071	5804509	A	0.01804	0.03	G	0.2795	0.5941
11	rs11031357	31349808	G	0.01804	0.03	A	0.2795	0.5941
15	rs141726041	40916632	A	0.01804	0.03	T	0.2795	0.5941
15	rs12902421	72161403	G	0.01804	0.03	A	0.2795	0.5941
15	rs34815962	72462255	A	0.01804	0.03	G	0.2795	0.5941
15	rs1266483	91462406	A	0.01804	0.03	G	0.2795	0.5941
16	rs3096381	71318001	A	0.01804	0.03	C	0.2795	0.5941
16	rs11545954	71411636	A	0.01804	0.03	G	0.2795	0.5941
6	rs592121	70984436	G	0.3582	0.39	A	0.2796	0.8731
12	rs4760998	127918882	A	0.3582	0.39	C	0.2796	0.8731
14	rs58668399	99983489	A	0.01804	0.01005	G	0.2796	1.81
3	rs4679621	59324733	G	0.08247	0.1017	A	0.2797	0.7943
11	rs592128	87597790	G	0.268	0.2408	A	0.2798	1.154
2	rs7603514	206836612	A	0.1263	0.1492	G	0.2799	0.8245
3	rs112632845	197432000	A	0.07474	0.05917	G	0.2799	1.285
6	rs2844580	31333303	G	0.1263	0.1492	A	0.2799	0.8245
11	rs1965209	6913290	G	0.1263	0.15	A	0.2799	0.8191
10	rs2769214	32515093	G	0.3582	0.3892	A	0.28	0.8762
17	rs9898164	38928014	G	0.2268	0.2008	A	0.2801	1.167
10	rs10887063	123844503	A	0.1134	0.09333	G	0.2802	1.243
12	rs4761815	51656651	A	0.3608	0.3925	C	0.2802	0.8737
3	rs55785561	40231383	A	0.2706	0.2425	G	0.2803	1.159
7	rs732577	12932049	A	0.2706	0.2425	C	0.2803	1.159
3	rs2272022	112063850	A	0.1031	0.1242	C	0.2804	0.8108
6	rs2763982	31872551	G	0.4072	0.3765	C	0.2804	1.138
12	rs11547907	108096769	C	0.04639	0.03417	G	0.2804	1.375
13	rs62619824	25823441	A	0.04639	0.03417	G	0.2804	1.375
16	rs7187227	632283	A	0.04639	0.03417	G	0.2804	1.375
19	rs117465739	3831781	A	0.04639	0.03417	G	0.2804	1.375
19	rs6603095	7411780	G	0.1031	0.1242	A	0.2804	0.8108
4	rs2323070	21627809	A	0.2809	0.3117	G	0.2805	0.8628
20	rs6032259	44141331	G	0.2809	0.3105	A	0.2806	0.8675
2	rs12623642	26676395	C	0.3608	0.3917	A	0.2807	0.8768

5	rs162036	7885959	G	0.1546	0.1792	A	0.2808	0.8381
6	rs439812	29616607	G	0.3247	0.2958	A	0.2808	1.145
6	rs2075799	31778529	A	0.1546	0.1792	G	0.2808	0.8381
8	rs4871137	121868551	C	0.3634	0.395	A	0.2808	0.8743
11	rs495487	79031671	A	0.3247	0.2958	G	0.2808	1.145
3	rs9830791	57091575	G	0.4098	0.379	A	0.2809	1.138
3	rs1606434	145955736	G	0.4124	0.3808	A	0.281	1.141
5	rs9329079	178936722	A	0.3273	0.2975	G	0.281	1.149
6	rs624851	65301504	A	0.3273	0.2975	G	0.281	1.149
9	rs2228093	38396002	A	0.4124	0.3808	G	0.281	1.141
6	rs241448	32796685	G	0.3679	0.399	A	0.2812	0.8766
1	rs2378792	183979248	G	0.366	0.3983	A	0.2813	0.8719
2	rs741006	210558162	A	0.2706	0.2433	G	0.2813	1.154
6	rs241447	32796751	G	0.366	0.3983	A	0.2813	0.8719
8	rs16914781	51287482	G	0.1881	0.2142	A	0.2813	0.8503
3	rs1112438	39152345	A	0.2294	0.2575	G	0.2814	0.8583
7	rs12701859	41072834	A	0.366	0.3975	G	0.2814	0.8749
20	rs1555120	42696580	A	0.366	0.3975	C	0.2814	0.8749
1	rs699738	117311147	A	0.2419	0.2718	C	0.2815	0.855
3	rs16843947	101238259	A	0.4124	0.3817	G	0.2817	1.137
8	rs2062377	120007420	A	0.2732	0.245	T	0.2818	1.158
11	rs11828782	6789929	A	0.2732	0.245	C	0.2818	1.158
12	rs1185244	66725160	G	0.4175	0.3856	A	0.2819	1.142
4	rs6532600	97215545	G	0.3686	0.4	A	0.282	0.8755
4	rs9993805	117800441	G	0.3686	0.4	A	0.282	0.8755
10	rs4752642	123846288	A	0.1134	0.09417	G	0.282	1.23
3	rs4619816	51248198	A	0.4175	0.3858	G	0.2821	1.141
4	rs7692791	55980239	G	0.4175	0.3858	A	0.2821	1.141
5	rs12697058	67335548	G	0.4175	0.3858	A	0.2821	1.141
6	rs1615962	29745726	A	0.1624	0.14	G	0.2821	1.191
15	rs17677991	42032383	G	0.1624	0.14	C	0.2821	1.191
10	rs61866610	95326676	A	0.4201	0.3881	G	0.2824	1.142
14	rs12437266	21526079	G	0.3711	0.4033	A	0.2824	0.8731
1	rs78861150	36932272	A	0.02577	0.01667	C	0.2825	1.561
1	rs139619405	43305713	A	0.02577	0.01667	G	0.2825	1.561
4	rs3821945	6304118	A	0.02577	0.01667	G	0.2825	1.561
10	rs145330425	102743599	G	0.02577	0.01667	A	0.2825	1.561
10	rs75800852	104678841	A	0.02577	0.01667	G	0.2825	1.561
13	rs41306656	41508049	G	0.02577	0.01667	A	0.2825	1.561
16	rs78086885	2220641	A	0.02577	0.01667	G	0.2825	1.561
19	rs70937099	42907064	G	0.02577	0.01667	A	0.2825	1.561
22	rs189043542	46712229	A	0.02577	0.01667	G	0.2825	1.561
7	rs712701	127251188	C	0.4175	0.3867	A	0.2827	1.137

7	rs6959212	38128326	A	0.2861	0.3167	G	0.2828	0.8647
7	rs2522833	82453708	A	0.2861	0.3167	C	0.2828	0.8647
6	rs746647	31114182	G	0.1366	0.1158	A	0.2829	1.208
6	rs1265112	31118019	G	0.1366	0.1158	A	0.2829	1.208
17	rs41298712	78396004	G	0.1366	0.1158	A	0.2829	1.208
6	rs2858884	32700083	C	0.09278	0.07583	A	0.283	1.246
6	rs636864	149525606	A	0.09278	0.07583	G	0.283	1.246
19	rs4801959	53669273	A	0.09278	0.07583	G	0.283	1.246
1	rs156651	45051656	G	0.4227	0.3908	A	0.2831	1.141
6	rs9268877	32431147	A	0.4227	0.3908	G	0.2831	1.141
6	rs5026743	32439964	C	0.4227	0.3908	A	0.2831	1.141
12	rs11170353	53369160	G	0.4227	0.3908	A	0.2831	1.141
6	rs2621367	32768721	A	0.3737	0.405	G	0.2832	0.8766
20	rs872808	61878950	A	0.3737	0.405	C	0.2832	0.8766
17	rs76527190	183725	G	0.1289	0.1525	A	0.2834	0.8221
19	rs12150889	33355055	G	0.232	0.205	A	0.2834	1.171
5	rs11749269	54019772	G	0.3763	0.4083	A	0.2835	0.8742
5	rs444527	54410099	A	0.1289	0.1517	G	0.2835	0.8274
6	rs13193063	49479750	G	0.1289	0.1517	A	0.2835	0.8274
6	rs9453108	65620858	G	0.3763	0.4083	A	0.2835	0.8742
6	rs9294631	65622463	G	0.3763	0.4083	A	0.2835	0.8742
11	rs10895991	64972286	G	0.1289	0.1517	C	0.2835	0.8274
12	rs8756	66359752	C	0.1289	0.1517	A	0.2835	0.8274
2	rs7581432	32395315	A	0.4227	0.3917	G	0.2836	1.137
4	rs7660807	71388551	G	0.1572	0.1817	A	0.2836	0.8403
2	rs6735179	1777150	A	0.4225	0.4548	G	0.2838	0.877
8	rs2954026	126484526	A	0.2887	0.3189	C	0.2838	0.8668
12	rs61729278	80933609	G	0.05155	0.06833	A	0.2838	0.741
12	rs7975340	80935411	G	0.05155	0.06833	C	0.2838	0.741
1	rs1780324	21821757	A	0.3325	0.3033	G	0.2839	1.144
3	rs12638540	32488534	G	0.03866	0.05333	A	0.2839	0.7138
5	rs116897210	149375110	A	0.03866	0.05333	G	0.2839	0.7138
11	rs11037840	44069719	A	0.05155	0.0675	G	0.2839	0.7508
17	rs2305213	72769739	G	0.03866	0.05333	A	0.2839	0.7138
1	rs2992650	221147712	A	0.4278	0.3958	G	0.284	1.141
7	rs35928055	23737891	G	0.1649	0.1417	A	0.284	1.197
20	rs6017949	45269556	A	0.1649	0.1417	G	0.284	1.197
17	rs3848445	14294021	G	0.2887	0.318	A	0.2841	0.8702
3	rs818818	38350519	G	0.2758	0.2483	A	0.2843	1.153
9	rs2073935	136340200	A	0.06701	0.08514	C	0.2843	0.7717
11	rs10836954	4945229	A	0.232	0.2058	G	0.2843	1.165
14	rs4904947	92972162	A	0.232	0.2058	G	0.2843	1.165
14	rs10438246	105410183	G	0.3789	0.41	A	0.2843	0.8777

16	rs1395585	7594958	A	0.232	0.2058	G	0.2843	1.165
19	rs2163808	33418175	A	0.2758	0.2483	C	0.2843	1.153
6	rs10947088	30709830	G	0.06701	0.085	A	0.2844	0.7732
6	rs17719	30711031	A	0.06701	0.085	G	0.2844	0.7732
6	rs4327730	30780936	A	0.06701	0.085	G	0.2844	0.7732
3	rs11130097	46620614	G	0.3814	0.4133	A	0.2845	0.8753
3	rs4646750	125826003	G	0.03608	0.025	A	0.2845	1.46
4	rs2034938	169989993	G	0.4278	0.3967	A	0.2845	1.137
5	rs4921537	158366263	G	0.4304	0.3983	A	0.2845	1.141
7	rs146797353	91646406	A	0.03608	0.025	G	0.2845	1.46
7	rs357394	138049387	G	0.03608	0.025	C	0.2845	1.46
14	rs61534804	68041061	A	0.03608	0.025	G	0.2845	1.46
17	rs55996424	78293189	A	0.4304	0.3983	T	0.2845	1.141
19	rs5030878	52250216	A	0.03608	0.025	G	0.2845	1.46
8	rs1390938	20036713	A	0.2345	0.2633	G	0.2846	0.8571
14	rs1127066	23371055	G	0.2345	0.2625	C	0.2847	0.8608
1	rs11161887	76386305	A	0.3351	0.3058	G	0.2849	1.144
1	rs3753566	186316436	A	0.03866	0.05417	G	0.2849	0.7022
4	rs1562975	109408608	A	0.03866	0.05417	G	0.2849	0.7022
12	rs619381	10954258	A	0.1057	0.1267	G	0.2849	0.8147
14	rs2278607	105420215	G	0.2912	0.3217	A	0.2849	0.8665
14	rs78154277	105614734	A	0.03866	0.05417	G	0.2849	0.7022
15	rs16973457	82563991	A	0.2784	0.25	G	0.2849	1.157
19	rs138148015	7569091	C	0.03866	0.05417	G	0.2849	0.7022
19	rs117355762	38901578	A	0.03866	0.05417	G	0.2849	0.7022
1	rs7517810	172853460	G	0.1134	0.095	A	0.285	1.218
2	rs17183814	166152389	A	0.08505	0.105	G	0.285	0.7924
3	rs2290477	113374633	A	0.08505	0.105	C	0.285	0.7924
6	rs1044498	132172368	C	0.1134	0.095	A	0.285	1.218
7	rs886469	144152054	A	0.1134	0.095	G	0.285	1.218
10	rs2994684	31366271	A	0.08505	0.105	G	0.285	0.7924
12	rs74416240	110342598	A	0.08505	0.105	G	0.285	0.7924
14	rs4900384	98498951	A	0.433	0.4008	G	0.285	1.141
17	rs61741523	76525759	C	0.08505	0.105	G	0.285	0.7924
1	rs4233050	24419496	G	0.3376	0.3075	A	0.2852	1.148
10	rs1999240	26463130	C	0.2912	0.3208	A	0.2852	0.8698
14	rs3912200	56594745	G	0.3376	0.3075	A	0.2852	1.148
19	rs62136856	47573527	G	0.1933	0.22	A	0.2853	0.8495
3	rs2639801	4662497	A	0.433	0.4017	G	0.2854	1.138
9	rs7036568	114468966	C	0.07732	0.06083	A	0.2854	1.294
17	rs2074158	40257163	G	0.1392	0.1175	A	0.2854	1.214
17	rs34038065	72860653	A	0.07732	0.06083	G	0.2854	1.294
18	rs3809975	56204489	G	0.1392	0.1175	C	0.2854	1.214

21	rs75635009	43291618	A	0.07732	0.06083	G	0.2854	1.294
7	rs11977526	46008110	G	0.1933	0.2208	A	0.2855	0.8454
11	rs2584918	62559385	G	0.1649	0.1425	A	0.2855	1.189
19	rs3829647	52216563	A	0.3866	0.4183	C	0.2855	0.8763
4	rs117922332	22394239	C	0.08505	0.1058	A	0.2856	0.7854
9	rs16932300	14801738	C	0.08505	0.1058	G	0.2856	0.7854
1	rs12566888	156869047	A	0.4356	0.4042	C	0.2858	1.138
7	rs1107592	2041432	A	0.3866	0.4175	G	0.2859	0.8793
1	rs603991	229956480	G	0.3892	0.4208	A	0.286	0.8768
12	rs11544238	57870155	C	0.3892	0.4208	A	0.286	0.8768
6	rs9277348	33048538	G	0.4381	0.4067	A	0.2862	1.138
11	rs1033543	33667333	A	0.2371	0.265	G	0.2863	0.8621
19	rs7254346	17958777	G	0.2345	0.2083	A	0.2863	1.164
1	rs9253	37958813	C	0.1598	0.1842	G	0.2864	0.8425
5	rs16869392	90350998	G	0.1598	0.1842	A	0.2864	0.8425
6	rs6920606	32997674	A	0.3918	0.4233	G	0.2864	0.8774
11	rs2903566	57982896	C	0.2809	0.2525	A	0.2864	1.157
11	rs1793169	118070264	G	0.3918	0.4233	A	0.2864	0.8774
12	rs3847687	131525053	A	0.1598	0.1842	G	0.2864	0.8425
10	rs11259403	6572225	G	0.3892	0.42	A	0.2865	0.8798
12	rs11109986	100063310	A	0.3892	0.42	G	0.2865	0.8798
6	rs1559876	32215769	C	0.4407	0.4092	G	0.2866	1.138
6	rs4713518	32257337	G	0.4407	0.4092	A	0.2866	1.138
6	rs9469300	32860752	A	0.09536	0.0775	G	0.2866	1.255
4	rs1473222	143488095	G	0.1314	0.155	A	0.2868	0.825
11	rs1138800	93862493	A	0.3402	0.3108	C	0.2868	1.143
2	rs1734422	10932207	A	0.4381	0.4075	C	0.2869	1.134
4	rs3108796	179482360	A	0.4381	0.4075	G	0.2869	1.134
10	rs1551067	76787128	A	0.4433	0.4117	G	0.2869	1.138
14	rs8012090	86778000	A	0.4433	0.4117	G	0.2869	1.138
9	rs28412870	112078548	G	0.1392	0.1183	A	0.287	1.205
15	rs2289181	49090175	A	0.1392	0.1183	G	0.287	1.205
3	rs9870680	7529555	G	0.2964	0.3267	A	0.2871	0.8683
3	rs6784322	48422235	T	0.3969	0.4292	A	0.2871	0.8754
12	rs2072357	4735894	A	0.2964	0.3267	G	0.2871	0.8683
1	rs1707336	46493460	A	0.3428	0.3125	C	0.2872	1.147
6	rs537757	32268501	A	0.4407	0.41	T	0.2872	1.134
8	rs11996455	95853708	G	0.3428	0.3125	A	0.2872	1.147
2	rs10460460	121349814	G	0.4459	0.4142	A	0.2873	1.138
2	rs192798269	241404342	A	0.02835	0.04111	G	0.2873	0.6806
5	rs1502050	13779743	G	0.116	0.09667	A	0.2873	1.226
8	rs3735754	27516955	A	0.116	0.09667	G	0.2873	1.226
1	rs1532815	165180089	T	0.2964	0.3258	A	0.2874	0.8716

7	rs34518648	23728916	A	0.1675	0.1442	G	0.2874	1.195
10	rs531676	99637578	G	0.2964	0.3258	A	0.2874	0.8716
2	rs7602460	182261869	G	0.1959	0.2225	A	0.2875	0.8512
11	rs7480010	42246718	G	0.1959	0.2225	A	0.2875	0.8512
14	rs11160713	103600756	G	0.3995	0.4317	A	0.2875	0.8759
17	rs2279052	74287204	C	0.3995	0.4317	G	0.2875	0.8759
17	rs2279053	74287389	A	0.3995	0.4317	G	0.2875	0.8759
17	rs6501880	74289705	A	0.3995	0.4317	G	0.2875	0.8759
1	rs2647116	219009835	A	0.2345	0.2092	G	0.2876	1.158
3	rs11708014	183074900	G	0.4433	0.4125	A	0.2876	1.134
14	rs7146427	66407339	G	0.4433	0.4125	A	0.2876	1.134
15	rs4144691	84539619	C	0.1959	0.2233	G	0.2876	0.8471
11	rs11227664	66807561	C	0.02835	0.04174	A	0.2878	0.6699
12	rs17034433	104124018	C	0.02835	0.04167	A	0.2878	0.6711
16	rs9928448	30072530	G	0.3428	0.3133	A	0.2878	1.143
19	rs35025963	8191184	A	0.3428	0.3133	G	0.2878	1.143
20	rs74638057	31024207	A	0.02835	0.04167	G	0.2878	0.6711
22	rs9623117	40452119	G	0.02835	0.04167	A	0.2878	0.6711
22	rs2018458	40526472	A	0.02835	0.04167	C	0.2878	0.6711
6	rs41293883	31474820	A	0.06186	0.0475	G	0.2879	1.322
14	rs1009170	92636713	A	0.4021	0.4342	T	0.2879	0.8763
1	rs1628172	144912153	A	0.02835	0.04083	G	0.288	0.6854
9	rs147162442	5361758	G	0.02835	0.04083	C	0.288	0.6854
13	rs1801426	32972884	G	0.02835	0.04083	A	0.288	0.6854
1	rs3789044	204589101	A	0.299	0.3292	G	0.2881	0.8691
5	rs13162716	108040166	A	0.3454	0.315	G	0.2882	1.147
10	rs359307	17342489	A	0.4046	0.4367	G	0.2883	0.8768
12	rs7955162	38711658	G	0.4046	0.4367	A	0.2883	0.8768
15	rs12904234	78779384	G	0.4046	0.4367	A	0.2883	0.8768
16	rs8050871	71509796	G	0.451	0.42	C	0.2885	1.135
13	rs4525349	94803335	G	0.4562	0.4242	A	0.2887	1.139
17	rs33989543	7324788	A	0.1675	0.145	G	0.2887	1.187
2	rs10198396	37742142	A	0.4536	0.4225	G	0.2888	1.135
4	rs10004516	111398208	A	0.1624	0.1875	G	0.2889	0.84
2	rs3739038	238672703	G	0.4098	0.4417	C	0.289	0.8777
4	rs17067123	180048362	A	0.4588	0.4267	G	0.289	1.139
15	rs2290272	85447431	A	0.4072	0.4383	G	0.289	0.8802
19	rs11881700	52538428	G	0.1082	0.13	A	0.289	0.8124
22	rs132700	36587704	G	0.1082	0.13	A	0.289	0.8124
14	rs8014204	75322794	G	0.4562	0.425	A	0.2891	1.135
17	rs712270	18057167	T	0.1082	0.1294	A	0.2891	0.8168
20	rs2273535	54961541	A	0.3479	0.3175	T	0.2892	1.147
3	rs13072883	194994754	A	0.1082	0.1292	G	0.2893	0.8184

22	rs132628	36541825	G	0.1082	0.1292	A	0.2893	0.8184
3	rs1561072	181752619	G	0.201	0.1761	A	0.2894	1.177
11	rs7115014	102804933	A	0.4098	0.4408	G	0.2894	0.8807
11	rs10895391	103158278	A	0.2861	0.2575	G	0.2894	1.155
2	rs4453725	69659126	A	0.2371	0.2117	T	0.2895	1.158
6	rs3823418	31100942	A	0.2371	0.2117	G	0.2895	1.158
6	rs707926	31748820	A	0.2423	0.27	G	0.2895	0.8644
9	rs2586356	11660139	A	0.1985	0.225	C	0.2895	0.8528
1	rs209729	22835677	A	0.09536	0.07833	G	0.2896	1.24
1	rs10874924	95966161	A	0.4639	0.4317	G	0.2896	1.139
8	rs4909444	139701209	A	0.1985	0.2258	C	0.2896	0.8487
15	rs11634397	80432222	G	0.09536	0.07833	A	0.2896	1.24
2	rs4953954	132915518	G	0.4149	0.4467	A	0.2897	0.8786
15	rs4984390	94939508	A	0.3479	0.3183	G	0.2897	1.143
3	rs2251788	54604188	G	0.4639	0.4325	A	0.2899	1.136
6	rs2581	32974401	A	0.4665	0.4342	C	0.2899	1.14
11	rs7939451	9961079	G	0.4639	0.4325	A	0.2899	1.136
11	rs7949313	99512311	G	0.4639	0.4325	A	0.2899	1.136
1	rs4537545	154418879	A	0.4175	0.4492	G	0.29	0.8791
5	rs7715811	13769974	A	0.116	0.0975	G	0.2901	1.214
6	rs41269255	27277051	A	0.116	0.0975	G	0.2901	1.214
7	rs77058871	899820	A	0.116	0.0975	C	0.2901	1.214
12	exm1031949	104645405	A	0.116	0.0975	G	0.2901	1.214
12	rs7975161	104645405	A	0.116	0.0975	G	0.2901	1.214
12	rs4275659	123447928	A	0.3041	0.3342	G	0.2901	0.8708
16	rs59292608	20471450	A	0.3041	0.3342	G	0.2901	0.8708
17	rs74899363	8700778	A	0.116	0.0975	G	0.2901	1.214
18	rs7227022	10705744	A	0.4301	0.4624	G	0.2901	0.8771
19	rs7258368	12256912	A	0.2861	0.2583	G	0.2901	1.15
1	rs6426446	4955862	A	0.4665	0.435	G	0.2902	1.136
4	rs3796528	57798174	C	0.2397	0.2133	G	0.2902	1.162
6	rs2233434	44232920	G	0.2397	0.2133	A	0.2902	1.162
9	rs1969763	89874893	A	0.4201	0.4525	C	0.2902	0.8765
1	rs2298110	20233051	G	0.04897	0.03583	A	0.2903	1.385
1	rs41308154	247420421	A	0.4201	0.4517	G	0.2903	0.8795
7	rs79377093	103179594	A	0.04897	0.03583	G	0.2903	1.385
9	rs41272237	26995787	A	0.04897	0.03583	G	0.2903	1.385
10	rs7077074	116539304	A	0.4201	0.4517	G	0.2903	0.8795
11	rs11545200	65319751	A	0.04897	0.03583	G	0.2903	1.385
1	rs3737002	207760773	A	0.4227	0.455	G	0.2904	0.877
6	rs2239517	30865115	G	0.4227	0.455	A	0.2904	0.877
6	rs8408	30867666	A	0.4227	0.455	G	0.2904	0.877
6	rs9468845	30869593	G	0.4227	0.455	A	0.2904	0.877

6	rs3909130	30874165	A	0.4227	0.455	G	0.2904	0.877
6	rs916920	30877202	A	0.4227	0.455	G	0.2904	0.877
7	rs3793306	29314083	A	0.4691	0.4375	C	0.2904	1.136
9	rs7852296	126352218	A	0.08763	0.1075	G	0.2904	0.7974
1	rs466639	165394882	A	0.134	0.1567	G	0.2905	0.8331
6	rs2071285	32180431	A	0.134	0.1567	T	0.2905	0.8331
4	rs17037102	107845794	A	0.4227	0.4542	G	0.2906	0.8799
5	rs250853	132754003	A	0.4253	0.4574	G	0.2906	0.8776
6	rs3129816	30341068	G	0.3505	0.3208	A	0.2906	1.142
11	rs3825020	60184191	A	0.4227	0.4542	G	0.2906	0.8799
17	rs3760454	30222002	A	0.3505	0.3208	G	0.2906	1.142
11	rs4909951	8959545	C	0.2448	0.2733	A	0.2907	0.862
12	rs4763825	12578786	A	0.2448	0.2733	G	0.2907	0.862
19	rs3848618	55673654	G	0.2448	0.2733	A	0.2907	0.862
3	rs6438191	113955320	G	0.4665	0.4358	A	0.2908	1.132
14	rs10083386	75347585	C	0.4665	0.4358	A	0.2908	1.132
2	rs6728586	88837444	G	0.2887	0.26	A	0.2909	1.155
4	rs2291822	167020644	G	0.4742	0.4425	A	0.2909	1.136
6	rs2647050	32669767	G	0.4253	0.4567	A	0.2909	0.8803
6	rs2856718	32670255	A	0.4253	0.4567	G	0.2909	0.8803
19	rs1045747	18288898	G	0.08763	0.1083	A	0.2909	0.7905
7	rs10950380	1843966	A	0.4691	0.4383	G	0.291	1.132
7	rs7811547	93423576	C	0.4691	0.4383	A	0.291	1.132
19	rs10410631	58058739	A	0.4691	0.4383	G	0.291	1.132
4	rs2280205	9909923	A	0.3067	0.3367	G	0.2911	0.8716
5	rs4527649	2104302	A	0.4768	0.445	G	0.2911	1.137
5	rs12517906	180170819	A	0.1418	0.1208	G	0.2911	1.202
11	rs2306168	74907582	A	0.2448	0.2725	G	0.2911	0.8656
21	rs968714	45553596	A	0.2448	0.2725	G	0.2911	0.8656
11	rs10837055	38845174	C	0.4716	0.4408	A	0.2912	1.132
17	rs1126667	6902760	A	0.4278	0.4591	G	0.2912	0.881
20	rs6102059	39228784	G	0.4716	0.4408	A	0.2912	1.132
4	rs2160043	15499251	A	0.4794	0.4475	G	0.2913	1.137
5	rs3734091	82500734	A	0.06959	0.0875	C	0.2913	0.78
6	rs1264419	30576781	C	0.4794	0.4475	G	0.2913	1.137
6	rs9277946	33194717	A	0.06959	0.0875	G	0.2913	0.78
10	rs1904589	72195439	A	0.06959	0.0875	G	0.2913	0.78
11	rs61746574	68840399	A	0.06959	0.0875	G	0.2913	0.78
12	rs7971073	55794976	A	0.06959	0.0875	G	0.2913	0.78
13	rs9533090	42951449	A	0.06959	0.0875	G	0.2913	0.78
6	rs6901686	29715721	A	0.2021	0.2287	G	0.2914	0.854
6	rs154977	32900018	G	0.2397	0.2142	C	0.2914	1.157
6	rs9492393	130154667	G	0.2397	0.2142	A	0.2914	1.157

11	rs2090192	1401890	A	0.4742	0.4433	G	0.2914	1.133
17	rs2589133	78793476	A	0.433	0.465	G	0.2914	0.8786
2	rs6711770	160692761	G	0.3531	0.3233	A	0.2915	1.142
3	rs1683758	31090454	A	0.3505	0.3217	G	0.2915	1.138
8	rs10102598	48089751	A	0.3505	0.3217	G	0.2915	1.138
15	rs683676	40088761	A	0.482	0.45	G	0.2915	1.137
6	rs2072894	29713036	A	0.201	0.2283	G	0.2916	0.8503
11	rs3829241	68855363	A	0.201	0.2275	G	0.2916	0.8544
17	rs4790555	3968064	C	0.06959	0.08833	A	0.2916	0.7719
3	rs4680879	30193577	A	0.433	0.4641	G	0.2917	0.8818
8	rs9297291	100825521	C	0.4356	0.4675	A	0.2917	0.879
15	rs1878326	89450587	A	0.4845	0.4525	C	0.2917	1.137
22	rs6007344	45255637	C	0.4794	0.4483	G	0.2918	1.133
1	rs572235	212651499	A	0.1701	0.1475	G	0.2919	1.185
2	rs2240543	242066789	G	0.201	0.1775	A	0.2919	1.166
4	rs362331	3215835	G	0.482	0.4508	A	0.2919	1.133
6	rs3130663	30666594	G	0.07068	0.08917	A	0.2919	0.7769
6	rs901363	159178345	G	0.201	0.1775	A	0.2919	1.166
10	rs2279335	81164521	G	0.201	0.1775	A	0.2919	1.166
19	rs8113506	13519929	A	0.482	0.4508	C	0.2919	1.133
1	rs1697421	21823292	A	0.4356	0.4667	G	0.292	0.8819
1	rs6658304	89803882	G	0.4663	0.4983	A	0.2921	0.8796
4	rs10027817	69829222	A	0.2474	0.2758	G	0.2922	0.8631
6	rs2844745	30343703	G	0.2423	0.2158	A	0.2922	1.162
6	rs2516688	30364524	A	0.2423	0.2158	G	0.2922	1.162
9	rs1887221	26565913	C	0.4871	0.4558	A	0.2922	1.134
19	rs3745297	49658209	C	0.2474	0.2758	A	0.2922	0.8631
3	rs2292245	62189189	A	0.2912	0.2625	G	0.2923	1.154
8	rs1347779	105805344	A	0.3531	0.3242	G	0.2923	1.138
12	rs11114620	81173501	G	0.4897	0.4583	A	0.2923	1.134
15	rs2602141	43724646	C	0.4433	0.475	A	0.2923	0.8801
1	rs3890745	2553624	G	0.4923	0.4608	A	0.2924	1.134
4	rs764497	24981689	A	0.4485	0.4808	T	0.2924	0.8779
7	rs3857841	125224602	A	0.3557	0.3258	G	0.2924	1.142
1	rs4332350	26610891	C	0.4459	0.4775	A	0.2925	0.8805
3	rs13064905	32031615	A	0.513	0.4817	C	0.2925	1.133
4	rs1045080	121957051	G	0.1186	0.09917	A	0.2925	1.222
5	rs4701732	6454662	A	0.4459	0.4775	G	0.2925	0.8805
9	rs10738760	2691186	A	0.4407	0.4717	G	0.2925	0.8827
10	rs11593766	115457264	C	0.1186	0.09917	A	0.2925	1.222
18	rs1943338	59244189	G	0.4407	0.4717	A	0.2925	0.8827
2	rs10497377	172926226	A	0.4485	0.48	C	0.2926	0.8808
5	rs155808	140346712	A	0.451	0.4833	G	0.2926	0.8783

19	rs5167	45448465	A	0.4948	0.4633	C	0.2926	1.135
20	rs6012504	47262549	A	0.05412	0.07083	G	0.2926	0.7506
1	rs6667999	225533684	A	0.4974	0.4658	G	0.2927	1.135
3	rs2035254	105809730	G	0.2036	0.1792	A	0.2927	1.171
5	rs41469	9629813	A	0.4974	0.4658	G	0.2927	1.135
2	rs836237	231406680	G	0.451	0.4825	A	0.2928	0.8812
2	rs7607759	241536126	G	0.05412	0.07	A	0.2928	0.7602
3	rs12632942	38764998	G	0.4562	0.4883	A	0.2928	0.8789
3	rs6773138	124826408	G	0.05412	0.07	A	0.2928	0.7602
4	rs6836368	130751286	G	0.4562	0.4883	A	0.2928	0.8789
6	rs3763349	32808232	G	0.5026	0.4708	A	0.2928	1.136
7	rs7808424	117067822	C	0.451	0.4825	A	0.2928	0.8812
15	rs689647	43762196	A	0.4433	0.4742	G	0.2928	0.8831
16	rs1058158	88781614	G	0.05412	0.07	C	0.2928	0.7602
1	rs1801265	98348885	G	0.06186	0.04833	A	0.2929	1.298
3	rs6783439	95154073	G	0.4588	0.4908	A	0.2929	0.8793
3	rs4572767	95176677	A	0.4588	0.4908	C	0.2929	0.8793
6	rs2295006	39024225	A	0.06186	0.04833	G	0.2929	1.298
6	rs1805075	160505207	G	0.3119	0.3425	A	0.2929	0.87
10	rs3748229	98469507	A	0.2912	0.2633	C	0.2929	1.15
1	rs183441903	150236264	A	0.03608	0.02583	G	0.293	1.412
2	rs117260874	102019083	A	0.03608	0.02583	G	0.293	1.412
2	rs7591163	228715375	G	0.4613	0.4933	A	0.293	0.8796
4	rs17062055	176721793	A	0.5077	0.4758	G	0.293	1.136
7	rs7808146	124332986	A	0.4613	0.4933	G	0.293	0.8796
7	rs6966462	154157438	C	0.5206	0.4883	A	0.293	1.138
9	rs3739721	104188842	C	0.03608	0.02583	G	0.293	1.412
10	rs35924554	101716112	G	0.03608	0.02583	C	0.293	1.412
12	rs45607836	113401212	A	0.03608	0.02583	C	0.293	1.412
12	rs112818862	129190266	A	0.03608	0.02583	G	0.293	1.412
14	rs2235967	68249499	A	0.03608	0.02583	G	0.293	1.412
17	rs13894	7529902	A	0.03608	0.02583	G	0.293	1.412
17	rs3764400	46123932	G	0.4459	0.4767	A	0.293	0.8834
19	rs452767	5995450	G	0.4459	0.4767	A	0.293	0.8834
19	rs2075803	51628529	A	0.4459	0.4767	G	0.293	0.8834
20	rs1132274	17596155	C	0.5103	0.4783	A	0.293	1.137
2	rs6886	85622059	A	0.4639	0.4958	G	0.2931	0.8799
4	rs10025456	10140259	A	0.4562	0.4875	G	0.2931	0.8819
6	rs2507964	31458157	A	0.5206	0.4883	G	0.2931	1.138
6	rs2844477	31578772	G	0.5206	0.4883	A	0.2931	1.138
7	rs4722067	21901566	A	0.5206	0.4883	G	0.2931	1.138
9	rs3793577	23737627	G	0.5206	0.4883	A	0.2931	1.138
9	rs1927321	121066417	C	0.4562	0.4875	A	0.2931	0.8819

11	rs2276102	87013438	A	0.5206	0.4883	G	0.2931	1.138
11	rs953778	64066999	A	0.4691	0.5008	G	0.2932	0.8806
15	rs1200345	41819716	A	0.3557	0.3267	G	0.2932	1.138
5	rs10067052	32941161	G	0.4716	0.5033	A	0.2933	0.8809
11	rs1350445	91952154	G	0.5026	0.4717	A	0.2933	1.132
11	rs6590322	128206410	G	0.4716	0.5033	A	0.2933	0.8809
15	rs634990	35006073	G	0.4716	0.5033	A	0.2933	0.8809
18	rs11873555	23413944	A	0.4588	0.49	G	0.2933	0.8822
22	rs2235321	37462926	A	0.5077	0.4766	G	0.2933	1.133
3	rs13087612	32031622	A	0.5129	0.4817	G	0.2934	1.133
5	rs13182512	78573790	A	0.5129	0.4817	T	0.2934	1.133
5	rs1584717	101671415	G	0.518	0.4867	C	0.2934	1.134
6	rs7744845	99956560	G	0.5052	0.4742	A	0.2934	1.132
6	rs9346918	162920212	A	0.5129	0.4817	G	0.2934	1.133
7	rs2285738	25181912	A	0.518	0.4867	G	0.2934	1.134
10	rs7082870	92799087	A	0.5206	0.4892	G	0.2934	1.134
11	rs10840285	9690013	C	0.5077	0.4767	A	0.2934	1.132
11	rs10790342	119630418	G	0.518	0.4867	A	0.2934	1.134
11	rs4937920	134465744	G	0.4613	0.4925	A	0.2934	0.8825
15	rs3742950	24922230	G	0.5052	0.4742	C	0.2934	1.132
17	rs1078485	77894206	A	0.5232	0.4917	G	0.2934	1.134
18	rs4941203	61163084	A	0.4613	0.4925	C	0.2934	0.8825
20	rs2235587	1115919	G	0.5155	0.4842	A	0.2934	1.133
22	rs2072550	21386019	G	0.518	0.4867	A	0.2934	1.134
4	rs1129998	151719263	G	0.1366	0.16	A	0.2935	0.8306
5	rs148346330	176025287	A	0.03608	0.02588	G	0.2935	1.409
7	rs4720015	30795436	A	0.1366	0.16	C	0.2935	0.8306
14	rs1000203	40896108	G	0.1418	0.1217	A	0.2935	1.192
16	rs12934922	81301694	A	0.1418	0.1217	T	0.2935	1.192
1	rs2229291	53676401	C	0.2036	0.23	A	0.2936	0.8559
5	rs2270915	32786389	G	0.2036	0.2308	A	0.2936	0.8519
11	rs10831496	88557991	A	0.2036	0.23	G	0.2936	0.8559
2	rs3762487	135139506	A	0.4716	0.5025	G	0.2937	0.8838
12	rs2958154	57065713	G	0.25	0.2783	A	0.2937	0.8643
4	rs2301795	6596360	A	0.3144	0.345	G	0.2938	0.8708
15	rs17636733	25912339	G	0.1108	0.1333	A	0.2938	0.8101
20	rs4810865	47328358	G	0.1108	0.1333	A	0.2938	0.8101
2	rs11901202	37041439	A	0.1727	0.1492	G	0.2939	1.191
3	rs2304456	186445052	C	0.1727	0.1492	A	0.2939	1.191
8	rs35883156	57078933	A	0.0799	0.06333	C	0.2939	1.284
8	rs7833986	57100149	A	0.0799	0.06333	G	0.2939	1.284
8	rs13273123	57100791	G	0.0799	0.06333	A	0.2939	1.284
11	rs3802904	124789755	A	0.0799	0.06333	G	0.2939	1.284

16	rs330150	21051209	G	0.1727	0.1492	C	0.2939	1.191
19	rs1433083	52393984	A	0.0799	0.06333	G	0.2939	1.284
1	rs1213510	72098811	A	0.2912	0.2642	G	0.294	1.145
6	rs2442752	31351764	G	0.3144	0.3442	A	0.294	0.874
7	rs4947385	51963775	A	0.2912	0.2642	G	0.294	1.145
13	rs7326068	21209512	G	0.3144	0.3442	A	0.294	0.874
20	rs41309371	61443716	A	0.03608	0.02592	G	0.294	1.407
11	rs4057749	124294236	G	0.1675	0.1925	A	0.2941	0.8442
3	rs1799864	46399208	A	0.2938	0.2658	G	0.2943	1.149
15	rs7119	77777632	A	0.1675	0.1933	G	0.2943	0.8396
1	rs145091069	34330321	A	0.01804	0.01083	G	0.2947	1.678
1	rs78982044	152282495	A	0.01804	0.01083	G	0.2947	1.678
1	rs1452632	215154120	A	0.317	0.3475	C	0.2947	0.8715
2	rs200617980	220412696	A	0.01804	0.01083	C	0.2947	1.678
7	rs202169962	4823070	G	0.01804	0.01083	A	0.2947	1.678
7	rs146649877	19748587	A	0.01804	0.01083	G	0.2947	1.678
7	rs149938024	150034456	A	0.01804	0.01083	G	0.2947	1.678
8	rs201204920	21964944	A	0.01804	0.01083	C	0.2947	1.678
9	rs1572299	121346417	G	0.01804	0.01083	A	0.2947	1.678
9	rs11789399	121359286	A	0.01804	0.01083	G	0.2947	1.678
10	rs3758464	79785825	C	0.01804	0.01083	A	0.2947	1.678
10	rs7899453	79799959	A	0.01804	0.01083	C	0.2947	1.678
11	rs34978242	108382207	G	0.01804	0.01083	A	0.2947	1.678
11	rs114496868	108382375	A	0.01804	0.01083	G	0.2947	1.678
11	rs145397072	111953419	C	0.01804	0.01083	A	0.2947	1.678
11	rs35551906	123753958	A	0.01804	0.01083	G	0.2947	1.678
12	rs118133397	55641331	A	0.01804	0.01083	T	0.2947	1.678
12	rs150964423	129559063	A	0.01804	0.01083	C	0.2947	1.678
13	rs79733377	37441448	A	0.01804	0.01083	C	0.2947	1.678
14	rs144418311	69791444	A	0.01804	0.01083	G	0.2947	1.678
14	rs144881047	81307087	A	0.01804	0.01083	G	0.2947	1.678
16	rs41292396	28997537	A	0.01804	0.01083	G	0.2947	1.678
16	rs62619985	75646423	G	0.01804	0.01083	A	0.2947	1.678
16	rs60664312	75657221	A	0.01804	0.01083	C	0.2947	1.678
20	rs143811282	17950588	C	0.01804	0.01083	G	0.2947	1.678
21	rs117272030	37442634	A	0.01804	0.01083	C	0.2947	1.678
15	rs3803530	90176073	A	0.3608	0.3317	C	0.2948	1.138
2	rs1900833	168583981	A	0.317	0.3467	G	0.2949	0.8747
9	rs929589	122899590	A	0.3634	0.3333	G	0.2949	1.142
12	rs1126605	7242204	A	0.3634	0.3333	G	0.2949	1.142
15	rs12050778	76126371	C	0.3634	0.3333	A	0.2949	1.142
3	rs1811510	5775231	A	0.1727	0.15	C	0.2951	1.183
9	rs62540100	27414808	G	0.2526	0.2808	A	0.2951	0.8654

11	rs2508489	120099658	G	0.1443	0.1233	A	0.2951	1.199
18	rs623053	33779855	G	0.1186	0.1	A	0.2951	1.211
1	rs2249265	117559726	A	0.2062	0.1817	G	0.2952	1.17
2	rs77687733	131520178	G	0.2062	0.1817	C	0.2952	1.17
6	rs2240061	31114848	G	0.2062	0.1817	A	0.2952	1.17
6	rs2073716	31122997	C	0.2062	0.1817	G	0.2952	1.17
6	rs61742784	44255387	A	0.2448	0.2192	G	0.2952	1.155
1	rs10916668	20233086	A	0.04897	0.03667	G	0.2953	1.353
1	rs143331871	38357078	G	0.04897	0.03667	A	0.2953	1.353
1	rs12084919	224491502	A	0.04897	0.03667	G	0.2953	1.353
10	rs3858340	121436286	A	0.2938	0.2667	G	0.2953	1.144
14	rs77860620	58949390	A	0.04897	0.03667	G	0.2953	1.353
22	rs362090	35463249	G	0.2062	0.2333	A	0.2955	0.8534
7	rs76440115	100675128	A	0.1736	0.1511	G	0.2956	1.18
13	rs1831443	19861674	A	0.3634	0.3342	G	0.2956	1.137
2	rs17010210	30985977	A	0.09021	0.11	G	0.2957	0.8022
6	rs35772543	96053922	A	0.09021	0.11	T	0.2957	0.8022
7	rs73168398	100695138	A	0.09021	0.11	G	0.2957	0.8022
13	rs16973313	109496813	G	0.09021	0.11	A	0.2957	0.8022
5	rs7731657	129943319	G	0.366	0.3358	A	0.2958	1.142
10	rs16931938	76803567	C	0.366	0.3358	A	0.2958	1.142
18	rs12454365	54396215	G	0.3196	0.3492	A	0.2958	0.8755
19	rs200797032	8609201	G	0.01804	0.01089	A	0.2959	1.669
10	rs210280	43089965	G	0.09021	0.1108	A	0.2961	0.7954
1	rs3138105	158263011	G	0.04124	0.05667	A	0.2962	0.716
6	rs4148249	30558190	A	0.04124	0.05667	C	0.2962	0.716
1	rs1933182	109999838	A	0.02577	0.0175	C	0.2965	1.485
2	rs6736609	171260787	G	0.3222	0.3525	A	0.2965	0.873
7	rs11525066	78175977	A	0.02577	0.0175	G	0.2965	1.485
7	rs3750105	94294682	A	0.2552	0.2833	C	0.2965	0.8665
7	rs314370	100453208	G	0.02577	0.0175	A	0.2965	1.485
7	rs2072165	143657473	G	0.1701	0.1957	A	0.2965	0.8427
8	rs74196658	38852950	G	0.02577	0.0175	A	0.2965	1.485
9	rs199901947	136198734	C	0.02577	0.0175	G	0.2965	1.485
10	rs7918793	27497191	A	0.02577	0.0175	G	0.2965	1.485
11	rs188780113	64678544	A	0.02577	0.0175	G	0.2965	1.485
12	rs11062745	3724583	G	0.02577	0.0175	A	0.2965	1.485
22	rs115076367	33778028	G	0.02577	0.0175	A	0.2965	1.485
18	rs12607406	8439366	A	0.3686	0.3383	G	0.2966	1.141
2	rs874816	121264471	G	0.2062	0.1825	A	0.2967	1.163
4	rs28647489	187531022	G	0.3222	0.3517	A	0.2967	0.8762
7	rs56204700	97821855	G	0.3222	0.3517	A	0.2967	0.8762
9	rs56108623	98638306	A	0.2062	0.1825	G	0.2967	1.163

22	rs5766113	45251423	G	0.3222	0.3517	A	0.2967	0.8762
1	rs1174657	183616926	G	0.1392	0.1625	A	0.2968	0.8333
1	rs16856202	232155151	C	0.1701	0.1958	A	0.2968	0.8417
6	rs11966360	26316982	G	0.0799	0.06417	A	0.2968	1.266
6	rs61740375	56919443	G	0.0799	0.06417	A	0.2968	1.266
6	rs1040461	57055354	A	0.0799	0.06417	G	0.2968	1.266
8	rs7386474	142359550	G	0.1701	0.1958	A	0.2968	0.8417
12	rs4251545	44180295	A	0.0799	0.06417	G	0.2968	1.266
13	rs1609848	106266101	A	0.0799	0.06417	G	0.2968	1.266
14	rs8009159	99182559	A	0.0799	0.06417	G	0.2968	1.266
10	rs1048347	124096061	A	0.299	0.2708	C	0.2969	1.148
2	rs3755321	234621825	G	0.1727	0.1508	A	0.297	1.175
1	rs1174658	183617105	G	0.1392	0.1633	A	0.2971	0.8282
7	rs12718597	50428428	A	0.1392	0.1633	C	0.2971	0.8282
1	rs4970862	145421477	A	0.3686	0.3392	G	0.2972	1.137
4	rs13129469	83991529	A	0.3247	0.355	C	0.2973	0.8738
12	rs1129167	122812503	C	0.3247	0.355	G	0.2973	0.8738
1	rs3795789	228476366	A	0.3711	0.3408	C	0.2974	1.141
6	rs2397060	51611470	G	0.1443	0.1242	A	0.2974	1.19
7	rs12537658	16119502	C	0.3711	0.3408	A	0.2974	1.141
9	rs12352313	730054	G	0.1134	0.135	A	0.2974	0.8196
12	rs12579350	5797101	A	0.1443	0.1242	G	0.2974	1.19
1	rs7554119	68965439	C	0.2088	0.235	A	0.2976	0.8589
9	rs2274324	37731007	A	0.2088	0.1842	G	0.2976	1.169
18	rs2002842	76409597	C	0.2088	0.1842	A	0.2976	1.169
21	rs11088859	22689344	A	0.2088	0.1842	G	0.2976	1.169
2	rs13385731	33701890	G	0.1134	0.1358	A	0.2978	0.8137
3	rs41272321	132338346	C	0.1134	0.1358	A	0.2978	0.8137
11	rs3751057	9051475	G	0.1134	0.1358	C	0.2978	0.8137
19	rs79338777	51857738	A	0.1134	0.1358	G	0.2978	0.8137
4	rs1594468	141387438	A	0.299	0.2717	G	0.2979	1.143
5	rs4704585	79028472	G	0.2577	0.2867	A	0.2979	0.864
17	rs15563	47005193	A	0.2577	0.2858	G	0.2979	0.8675
17	rs2291725	47039132	A	0.2577	0.2858	G	0.2979	0.8675
2	rs7562790	36673555	A	0.3711	0.3417	C	0.298	1.137
2	rs7567285	196663997	G	0.3711	0.3417	A	0.298	1.137
4	rs4558821	157030680	C	0.3711	0.3417	A	0.298	1.137
6	rs9268507	32377539	A	0.3711	0.3417	G	0.298	1.137
7	rs10229215	7171465	G	0.3711	0.3417	A	0.298	1.137
10	rs3817405	20506418	A	0.3711	0.3417	G	0.298	1.137
10	rs2484180	27692279	A	0.3711	0.3417	C	0.298	1.137
11	rs4414223	130776524	A	0.3711	0.3417	G	0.298	1.137
1	rs10913933	180124061	C	0.07216	0.09	G	0.2981	0.7864

1	rs2272757	881627	G	0.3273	0.3575	A	0.2982	0.8745
1	rs4656197	169823718	A	0.3273	0.3575	G	0.2982	0.8745
6	rs10484561	32665420	C	0.07216	0.09083	A	0.2982	0.7785
10	rs2359536	20899608	G	0.07216	0.09083	A	0.2982	0.7785
10	rs2792980	131200443	A	0.1753	0.1525	G	0.2982	1.181
14	rs2239557	74551970	G	0.1753	0.1525	C	0.2982	1.181
17	rs9891699	8157310	G	0.3737	0.3433	A	0.2982	1.141
17	rs2074286	39464046	C	0.3273	0.3575	G	0.2982	0.8745
17	rs2074284	39464736	G	0.3273	0.3575	C	0.2982	0.8745
22	rs1057353	45683304	G	0.3015	0.2733	C	0.2983	1.148
4	rs4333130	80949829	G	0.06443	0.05	A	0.2989	1.309
5	rs4621553	113030164	G	0.06443	0.05	A	0.2989	1.309
4	rs4235151	47504161	C	0.3299	0.36	A	0.299	0.8752
6	rs449242	33496714	C	0.1469	0.1258	A	0.2991	1.196
2	rs4669131	7232478	A	0.2603	0.2892	G	0.2992	0.8651
2	rs1876518	65608909	A	0.1727	0.1975	G	0.2992	0.8481
4	rs6817572	151303318	G	0.2603	0.2892	A	0.2992	0.8651
10	rs2254067	5781628	A	0.1727	0.1975	C	0.2992	0.8481
10	rs2797491	5788608	C	0.1727	0.1975	G	0.2992	0.8481
2	rs2305160	101591304	A	0.1727	0.1983	G	0.2993	0.8437
6	rs6905949	30140525	G	0.1727	0.1983	A	0.2993	0.8437
6	rs9261582	30196980	G	0.1727	0.1983	A	0.2993	0.8437
19	rs3816032	33467515	G	0.2603	0.2883	A	0.2993	0.8686
8	rs13263453	18729496	A	0.3299	0.3592	G	0.2994	0.8784
14	rs1950500	24830850	A	0.3299	0.3592	G	0.2994	0.8784
3	rs2289746	105455955	G	0.3041	0.2758	A	0.2995	1.147
6	rs7192	32411646	A	0.3342	0.3642	C	0.2995	0.8764
6	rs2621377	32763110	G	0.3041	0.2758	A	0.2995	1.147
1	rs10889092	58915024	G	0.2526	0.2258	A	0.2996	1.158
6	rs9380030	27925827	G	0.1005	0.0825	A	0.2996	1.243
20	rs2231616	44005936	A	0.2526	0.2258	G	0.2996	1.158
6	rs683208	32937901	G	0.3325	0.3623	A	0.2998	0.8768
10	rs117330938	104119139	G	0.02062	0.03172	A	0.2998	0.6427
1	rs140750531	17567149	A	0.02062	0.03167	G	0.2999	0.6438
1	rs36108216	161970046	G	0.02062	0.03167	A	0.2999	0.6438
3	rs2605419	123414429	G	0.02062	0.03167	A	0.2999	0.6438
4	rs61155675	187086562	A	0.02062	0.03167	C	0.2999	0.6438
9	rs142855762	119249734	A	0.02062	0.03167	G	0.2999	0.6438
11	rs2341432	5602438	A	0.02062	0.03167	T	0.2999	0.6438
11	rs2341434	5602615	A	0.02062	0.03167	G	0.2999	0.6438
12	rs61736010	122247853	A	0.02062	0.03167	G	0.2999	0.6438
15	rs77809780	43512995	A	0.02062	0.03167	G	0.2999	0.6438
15	rs2106673	91452595	A	0.02062	0.03167	G	0.2999	0.6438

19	rs3745650	12774532	A	0.02062	0.03167	C	0.2999	0.6438
6	rs1436870	81136945	C	0.1753	0.1533	A	0.3	1.173
6	rs25683	160196343	G	0.2113	0.1867	A	0.3	1.168
7	rs17364464	22514053	G	0.1211	0.1025	A	0.3	1.207
12	rs2723880	119588962	A	0.1211	0.1025	G	0.3	1.207
16	rs2904880	28944396	C	0.1211	0.1025	G	0.3	1.207
20	rs3790174	19261606	G	0.1211	0.1025	A	0.3	1.207
1	rs6699312	171805322	A	0.3789	0.3492	G	0.3002	1.137
3	rs11705970	66714410	A	0.3789	0.3492	G	0.3002	1.137
9	rs1780159	15157977	G	0.1005	0.08278	A	0.3002	1.238
11	rs1493954	12315915	A	0.3325	0.3617	G	0.3002	0.8791
22	rs2072123	19026613	G	0.3325	0.3617	A	0.3002	0.8791
11	rs7937317	123813795	G	0.1418	0.1658	A	0.3003	0.8308
11	rs6590021	123814356	A	0.1418	0.1658	G	0.3003	0.8308
19	rs12104233	1310821	G	0.3041	0.2767	A	0.3004	1.143
2	rs10489990	71062648	A	0.2629	0.2917	G	0.3005	0.8661
3	rs6804162	108639384	G	0.2526	0.2267	A	0.3005	1.153
6	rs2153960	108988184	G	0.2196	0.2475	A	0.3006	0.8555
16	rs201708128	71019225	A	0.0567	0.07299	G	0.3006	0.7635
10	rs868471	44210600	G	0.2629	0.2908	A	0.3007	0.8696
2	rs143123053	152543967	G	0.02062	0.03255	A	0.3008	0.6256
2	rs34371548	239098594	G	0.02062	0.0325	A	0.3008	0.6267
5	rs4975709	1877280	C	0.3067	0.2783	A	0.3008	1.147
6	rs117061525	38754694	G	0.02062	0.0325	A	0.3008	0.6267
9	rs76796290	139751649	A	0.02062	0.0325	G	0.3008	0.6267
12	rs36023382	122277891	A	0.02062	0.0325	C	0.3008	0.6267
15	rs3759901	72511415	A	0.02062	0.0325	G	0.3008	0.6267
21	rs78244022	41151203	A	0.02062	0.0325	G	0.3008	0.6267
6	rs3830076	32096244	A	0.09278	0.1125	G	0.3009	0.8068
14	rs7158173	65645250	G	0.3814	0.3517	A	0.3009	1.137
17	rs4790333	2262703	G	0.2526	0.227	A	0.301	1.15
4	rs10049756	28872833	A	0.0567	0.07333	G	0.3011	0.7596
11	rs4397868	133566985	G	0.0567	0.07333	A	0.3011	0.7596
14	rs2228541	94776221	A	0.0567	0.07333	C	0.3011	0.7596
16	rs12917698	80335572	A	0.0567	0.07333	G	0.3011	0.7596
19	rs77836061	9297286	A	0.0567	0.07333	T	0.3011	0.7596
1	rs56168609	228471379	G	0.1469	0.1267	C	0.3012	1.187
1	rs61825301	228504591	A	0.1469	0.1267	C	0.3012	1.187
1	rs3795801	228505739	A	0.1469	0.1267	G	0.3012	1.187
2	rs2271683	189873737	G	0.0799	0.065	A	0.3012	1.249
3	rs16845107	113127991	A	0.1778	0.155	C	0.3012	1.179
5	rs2853676	1288547	A	0.1778	0.155	G	0.3012	1.179
8	rs6558052	22273687	G	0.1778	0.155	A	0.3012	1.179

19	rs76935241	56329447	A	0.0799	0.065	G	0.3012	1.249
20	rs451571	5923204	G	0.1778	0.155	A	0.3012	1.179
3	rs60149583	197624444	G	0.2113	0.1875	A	0.3013	1.161
4	rs1870477	166605228	G	0.2113	0.1875	A	0.3013	1.161
8	rs2306825	18656805	A	0.3376	0.3675	G	0.3014	0.8773
10	rs4751674	116139029	A	0.2139	0.24	G	0.3014	0.8617
12	rs4931655	33058062	G	0.2552	0.2283	A	0.3014	1.158
17	rs8066636	18761109	A	0.3376	0.3673	G	0.3014	0.8781
7	rs10262505	149484590	G	0.01289	0.02261	A	0.3015	0.5643
1	rs145602591	32126672	A	0.01289	0.02254	G	0.3016	0.5662
20	rs364585	12962718	A	0.384	0.3542	G	0.3016	1.137
1	rs3737703	15813843	A	0.01289	0.0225	G	0.3017	0.5672
1	rs6127	169566313	G	0.01289	0.0225	A	0.3017	0.5672
1	rs151004649	203192360	A	0.01289	0.0225	C	0.3017	0.5672
2	rs3769838	231076191	G	0.01289	0.0225	A	0.3017	0.5672
4	rs2306242	843720	G	0.116	0.1383	A	0.3017	0.8172
4	rs4698412	15737348	A	0.3814	0.3525	G	0.3017	1.133
6	rs79862727	564041	A	0.01289	0.0225	G	0.3017	0.5672
9	rs141877075	129642663	A	0.01289	0.0225	C	0.3017	0.5672
11	rs144528596	5686870	A	0.01289	0.0225	T	0.3017	0.5672
11	rs56200889	72408055	C	0.1753	0.2	G	0.3017	0.85
15	rs11073922	90814664	A	0.01289	0.0225	G	0.3017	0.5672
17	rs76119062	1673228	G	0.01289	0.0225	C	0.3017	0.5672
17	rs897453	17425631	A	0.116	0.1383	G	0.3017	0.8172
19	rs2234373	36207077	A	0.01289	0.0225	G	0.3017	0.5672
11	rs116779179	619767	A	0.01295	0.02258	G	0.3018	0.5682
17	rs7226363	15234895	A	0.2655	0.2942	G	0.3018	0.8672
5	rs6884959	157102185	G	0.3402	0.3708	A	0.302	0.8748
7	rs2269970	107002806	A	0.3402	0.3708	G	0.302	0.8748
9	rs200620036	79635474	A	0.01295	0.0225	G	0.302	0.5701
1	rs12134363	101711964	C	0.3093	0.2808	A	0.3021	1.147
1	rs6688782	201181994	G	0.1005	0.08333	A	0.3022	1.229
2	rs17838437	89130009	A	0.3402	0.37	C	0.3022	0.878
6	rs35471617	56033094	A	0.0567	0.07417	G	0.3022	0.7504
12	rs10743938	11183451	A	0.1005	0.08333	T	0.3022	1.229
12	rs1355696	17424512	G	0.3402	0.37	A	0.3022	0.878
12	rs61757741	27844824	A	0.0567	0.07417	G	0.3022	0.7504
17	rs1127440	7221385	G	0.0567	0.07417	A	0.3022	0.7504
19	rs1596797	9088017	A	0.2139	0.1892	C	0.3024	1.166
7	rs17145738	72982874	A	0.1237	0.1042	G	0.3025	1.214
21	rs2236695	43221797	G	0.1237	0.1042	C	0.3025	1.214
4	rs17270628	76254408	A	0.3428	0.3733	G	0.3027	0.8755
5	rs3756668	67596088	A	0.3428	0.3733	G	0.3027	0.8755

6	rs17072442	111543412	C	0.04897	0.0375	A	0.3027	1.322
8	rs7834383	13273477	A	0.04897	0.0375	C	0.3027	1.322
9	rs17694549	27009100	G	0.04897	0.0375	A	0.3027	1.322
17	rs45443898	26816259	A	0.04897	0.0375	G	0.3027	1.322
4	rs34369149	17309436	G	0.3093	0.2817	A	0.3028	1.142
10	rs7913176	115644040	A	0.3093	0.2817	G	0.3028	1.142
3	rs2285372	129304797	A	0.1495	0.1283	G	0.3029	1.194
3	rs2301572	129305460	A	0.1495	0.1283	G	0.3029	1.194
4	rs17030434	154703596	G	0.3428	0.3725	A	0.3029	0.8786
6	rs4713346	30631063	A	0.03093	0.04417	G	0.3029	0.6907
6	rs3129907	32323745	G	0.03093	0.04417	A	0.3029	0.6907
6	rs3129931	32335516	G	0.03093	0.04417	A	0.3029	0.6907
6	rs3129941	32337686	A	0.03093	0.04417	G	0.3029	0.6907
8	rs34823600	2017399	A	0.03093	0.04417	G	0.3029	0.6907
10	rs2275272	97388162	A	0.03093	0.04417	G	0.3029	0.6907
11	rs142487000	118518751	A	0.03093	0.04417	G	0.3029	0.6907
11	rs147473566	118532376	G	0.03093	0.04417	A	0.3029	0.6907
15	rs3867498	25078787	A	0.03093	0.04417	G	0.3029	0.6907
16	rs61753635	71660404	A	0.03093	0.04417	G	0.3029	0.6907
2	rs12988934	182323665	A	0.2165	0.2433	G	0.303	0.8592
14	rs10151658	64612858	C	0.3866	0.3575	A	0.303	1.133
16	rs666636	56601720	A	0.2165	0.2433	G	0.303	0.8592
16	rs1493892	77545082	C	0.3892	0.3592	A	0.303	1.137
21	rs9306111	46077946	A	0.3892	0.3592	G	0.303	1.137
14	rs1952790	38342676	A	0.268	0.2967	G	0.3031	0.8682
1	rs285480	165403903	G	0.1443	0.1683	A	0.3033	0.8334
1	rs10779261	216595306	G	0.1443	0.1683	A	0.3033	0.8334
2	rs10202624	42515388	A	0.1443	0.1683	G	0.3033	0.8334
1	rs3795809	228525008	A	0.06443	0.05083	G	0.3034	1.286
2	rs35608243	174131392	G	0.06443	0.05083	A	0.3034	1.286
4	rs60816800	7435058	A	0.06443	0.05083	G	0.3034	1.286
4	rs61738677	7435486	A	0.06443	0.05083	C	0.3034	1.286
4	rs6850206	7435721	A	0.06443	0.05083	G	0.3034	1.286
2	rs2288569	179458591	A	0.3454	0.3758	G	0.3035	0.8761
1	rs12037173	70355981	G	0.2139	0.19	A	0.3036	1.16
10	rs7070980	89855608	A	0.2139	0.19	G	0.3036	1.16
17	rs8079702	68190826	G	0.3892	0.36	A	0.3036	1.133
3	rs388483	50615539	A	0.3918	0.3623	G	0.304	1.134
10	rs2244967	50224766	G	0.2577	0.2317	A	0.304	1.152
3	rs9809384	111981878	A	0.1778	0.2033	G	0.3041	0.8475
3	rs9809404	111981924	A	0.1778	0.2033	G	0.3041	0.8475
6	rs4335016	104217420	A	0.1778	0.2025	G	0.3041	0.8519
10	rs1079610	88422116	G	0.1778	0.2025	A	0.3041	0.8519

4	rs1455311	79964587	G	0.1804	0.1575	A	0.3042	1.177
9	rs144452859	97062138	A	0.03886	0.0275	G	0.3042	1.43
14	rs1953358	56295580	G	0.3479	0.3783	A	0.3042	0.8768
5	rs1394650	8837390	A	0.3918	0.3625	G	0.3043	1.133
6	rs7774158	33007752	A	0.3943	0.3642	C	0.3043	1.137
14	rs3625	75904660	A	0.3918	0.3625	G	0.3043	1.133
15	rs2398162	96830550	A	0.3918	0.3625	G	0.3043	1.133
22	rs7289099	43155392	G	0.3918	0.3625	A	0.3043	1.133
9	rs55654273	86474115	G	0.07474	0.09265	A	0.3044	0.7911
8	rs16920543	57311502	A	0.3144	0.2858	G	0.3045	1.146
1	rs199856800	23760011	C	0.01289	0.02333	G	0.3046	0.5464
1	rs1801160	97770920	A	0.01289	0.02333	G	0.3046	0.5464
1	rs11805972	201355653	A	0.01289	0.02333	C	0.3046	0.5464
1	rs12088790	201355761	G	0.01289	0.02333	A	0.3046	0.5464
2	rs72900086	186672527	T	0.01289	0.02333	A	0.3046	0.5464
2	rs35781413	190925045	A	0.01289	0.02333	G	0.3046	0.5464
3	rs11920719	170834559	C	0.07474	0.0925	A	0.3046	0.7925
5	rs76268053	118451873	C	0.01289	0.02333	A	0.3046	0.5464
5	rs112912947	118485238	C	0.01289	0.02333	G	0.3046	0.5464
8	rs117666725	20038445	A	0.01289	0.02333	G	0.3046	0.5464
11	rs149280308	93440001	A	0.01289	0.02333	G	0.3046	0.5464
12	rs2302516	7842587	G	0.01289	0.02333	C	0.3046	0.5464
14	rs2273607	101198426	A	0.07474	0.09333	G	0.3046	0.7847
16	rs6497759	24801737	A	0.07474	0.09333	G	0.3046	0.7847
16	rs3803716	24802325	A	0.07474	0.09333	G	0.3046	0.7847
17	rs201776800	7359930	G	0.01289	0.02333	A	0.3046	0.5464
18	rs12386117	8798185	A	0.01289	0.02333	G	0.3046	0.5464
11	rs4517489	36896281	A	0.2706	0.2983	G	0.3047	0.8726
1	rs2274067	231376806	C	0.08247	0.06667	G	0.3048	1.258
3	rs2062583	56966246	C	0.08247	0.06667	A	0.3048	1.258
6	rs45515398	43251118	A	0.08247	0.06667	G	0.3048	1.258
9	rs557352	79324741	G	0.3505	0.3808	A	0.3048	0.8774
12	rs2669010	77009060	A	0.3505	0.3808	G	0.3048	0.8774
15	rs261334	58726744	G	0.3505	0.3808	C	0.3048	0.8774
16	rs11074656	24888646	G	0.08247	0.06667	A	0.3048	1.258
18	rs3744921	29867688	G	0.08247	0.06667	A	0.3048	1.258
19	rs10414177	13336617	G	0.3505	0.3808	A	0.3048	0.8774
1	rs1135983	161761312	A	0.3943	0.365	G	0.3049	1.133
1	rs7530997	161810151	A	0.3943	0.365	G	0.3049	1.133
2	rs10188217	61217542	G	0.03093	0.045	A	0.3049	0.6773
6	rs2233969	31080432	G	0.3943	0.365	A	0.3049	1.133
8	rs6992851	2717835	A	0.3943	0.365	G	0.3049	1.133
9	rs12554086	82807503	A	0.03093	0.045	G	0.3049	0.6773

11	rs10838525	5701001	A	0.03093	0.045	G	0.3049	0.6773
11	rs2298484	118509676	A	0.03093	0.045	G	0.3049	0.6773
11	rs117826294	118516453	A	0.03093	0.045	G	0.3049	0.6773
1	rs117113675	3425722	A	0.03866	0.0275	G	0.305	1.422
4	rs62623564	140058849	A	0.03866	0.0275	T	0.305	1.422
6	rs9321659	100116092	A	0.2603	0.2333	G	0.305	1.156
7	rs17172694	46437154	A	0.03866	0.0275	C	0.305	1.422
13	rs7993921	90001033	A	0.2603	0.2333	G	0.305	1.156
14	rs729421	24736961	A	0.3969	0.3667	G	0.305	1.137
16	rs72810507	55905676	A	0.03866	0.0275	G	0.305	1.422
16	rs2303853	84474484	A	0.03866	0.0275	G	0.305	1.422
19	rs34626245	33372814	A	0.03866	0.0275	G	0.305	1.422
4	rs1533317	35410631	A	0.3505	0.38	G	0.3052	0.8805
9	rs530978	79324179	A	0.3505	0.38	G	0.3052	0.8805
6	rs2451731	26624822	A	0.3531	0.3833	C	0.3055	0.8781
6	rs2844498	31476854	G	0.3531	0.3833	A	0.3055	0.8781
6	rs9346277	70146474	A	0.3531	0.3833	G	0.3055	0.8781
9	rs1108580	136505114	G	0.1186	0.1408	A	0.3056	0.8205
9	rs7868651	95555939	A	0.317	0.2883	C	0.3057	1.146
9	rs1131773	95840256	G	0.1804	0.1583	A	0.3058	1.17
16	rs3829533	86575737	A	0.1804	0.1583	G	0.3058	1.17
5	rs12658464	121489122	G	0.3531	0.3825	A	0.3059	0.8812
12	rs2216228	22365835	G	0.2165	0.1925	A	0.3059	1.159
2	rs10203995	39165054	G	0.2732	0.3008	A	0.306	0.8736
6	rs61746206	32065113	A	0.08333	0.06761	G	0.306	1.254
2	rs3115327	114954162	G	0.09536	0.1158	A	0.3061	0.8046
14	rs11848785	72057355	G	0.1031	0.08514	A	0.3061	1.235
3	rs10513205	143200129	G	0.4021	0.3717	A	0.3062	1.137
7	rs10242744	11579793	A	0.2644	0.2387	G	0.3062	1.146
21	rs11558819	45656774	A	0.1469	0.17	G	0.3062	0.8408
9	rs340892	6098662	A	0.317	0.2892	C	0.3063	1.141
1	rs192636495	204159701	A	0.04381	0.05833	C	0.3064	0.7397
7	rs314339	100391581	G	0.04381	0.05833	A	0.3064	0.7397
12	rs902049	87287786	A	0.4021	0.3723	G	0.3064	1.134
16	rs66463064	81180995	G	0.1804	0.2058	A	0.3064	0.8493
16	exm-IND16-829[illegible]	84438556	D	0.04381	0.05833	I	0.3064	0.7397
17	rs61740658	78337584	G	0.04381	0.05833	A	0.3064	0.7397
2	rs7598922	39082344	A	0.1521	0.1308	G	0.3067	1.191
15	rs75560163	42434336	C	0.1521	0.1308	G	0.3067	1.191
1	rs3100865	2806107	A	0.3582	0.3883	C	0.3068	0.8793
17	rs8074015	78306280	G	0.4046	0.3742	A	0.3068	1.137
1	rs2230061	150727539	A	0.2758	0.3042	G	0.3069	0.8711
4	rs6843082	111718067	A	0.2758	0.3042	G	0.3069	0.8711

5	rs17876032	176830627	A	0.2758	0.3042	G	0.3069	0.8711
6	rs2255221	31431691	A	0.04381	0.05917	C	0.3069	0.7286
6	rs1585891	32736722	G	0.3196	0.2908	A	0.3069	1.145
6	rs6457661	32737494	G	0.3196	0.2908	A	0.3069	1.145
10	rs2228526	50678717	G	0.04381	0.05917	A	0.3069	0.7286
10	rs1248634	79579222	A	0.3196	0.2908	G	0.3069	1.145
16	rs7194871	81232564	A	0.04381	0.05917	C	0.3069	0.7286
22	rs2298375	24106448	A	0.04381	0.05917	G	0.3069	0.7286
22	rs228942	37524619	A	0.3196	0.2908	C	0.3069	1.145
11	rs12574508	9853777	G	0.2191	0.1942	C	0.307	1.164
19	rs862708	57867599	G	0.183	0.16	A	0.3072	1.176
7	rs854555	94930391	C	0.3582	0.3875	A	0.3073	0.8824
21	rs458076	28025911	A	0.4072	0.3767	G	0.3074	1.137
11	rs666194	65159382	G	0.3196	0.2917	A	0.3075	1.141
14	rs1009203	76062806	A	0.3196	0.2917	C	0.3075	1.141
2	rs2248489	222438968	G	0.3608	0.39	A	0.308	0.883
21	rs2284958	44088631	G	0.3634	0.3942	A	0.308	0.8774
4	rs8340	57204777	C	0.2191	0.195	A	0.3081	1.158
5	rs1295686	131995843	A	0.2784	0.3075	G	0.3081	0.8686
11	rs4627097	125452313	G	0.2784	0.3075	A	0.3081	0.8686
1	rs10493389	66310865	G	0.1031	0.08583	A	0.3082	1.224
2	rs4352210	37750980	G	0.4098	0.38	A	0.3083	1.133
7	rs1637498	87599027	G	0.4098	0.38	A	0.3083	1.133
11	rs16937251	19955194	G	0.4098	0.38	C	0.3083	1.133
1	rs987495	79095581	G	0.2242	0.2517	A	0.3084	0.8595
6	rs2523897	30993958	A	0.2242	0.2517	G	0.3084	0.8595
13	rs9516806	97662381	A	0.3196	0.2925	G	0.3085	1.136
1	rs3738136	20972111	A	0.3222	0.2942	G	0.3086	1.14
4	rs6837753	128079327	G	0.366	0.3967	A	0.3086	0.878
6	rs9362636	90026797	G	0.1521	0.1317	A	0.3086	1.183
7	rs17132289	48428715	T	0.1521	0.1317	A	0.3086	1.183
11	rs1784302	118940957	C	0.1521	0.1317	G	0.3086	1.183
11	rs1177562	118949331	A	0.1521	0.1317	G	0.3086	1.183
17	rs35400274	4803711	A	0.1521	0.1317	G	0.3086	1.183
1	rs2936051	222803204	G	0.366	0.3958	A	0.3087	0.881
8	rs7464769	23007793	A	0.366	0.3958	G	0.3087	0.881
13	rs17278868	21707094	G	0.366	0.3958	A	0.3087	0.881
4	rs13148903	187257576	G	0.08247	0.0675	A	0.3089	1.242
7	rs3765187	80286003	A	0.08247	0.0675	G	0.3089	1.242
12	rs7962426	10415847	A	0.183	0.2075	C	0.3089	0.8554
14	rs2286653	93673655	A	0.08247	0.0675	G	0.3089	1.242
7	rs7776970	2752487	A	0.1521	0.1319	G	0.309	1.18
10	rs177045	71321279	A	0.4098	0.3808	G	0.3091	1.129

2	rs1061305	152346979	G	0.2809	0.31	A	0.3092	0.8696
2	rs7589368	194189678	G	0.3686	0.3992	A	0.3092	0.8786
3	rs993804	25112171	G	0.183	0.1611	A	0.3092	1.166
11	rs33952257	44089352	A	0.2809	0.31	G	0.3092	0.8696
20	rs1406961	61895920	A	0.05928	0.07583	C	0.3092	0.7679
1	rs2298014	228362682	A	0.06701	0.0525	G	0.3093	1.296
7	rs11767557	143109139	G	0.1495	0.1725	A	0.3093	0.8431
3	rs6438424	117574822	A	0.4149	0.385	C	0.3094	1.133
6	rs2064318	35477025	G	0.1211	0.1433	C	0.3094	0.8238
6	rs8192646	132938842	A	0.1263	0.1075	G	0.3094	1.2
7	rs17852421	1595068	A	0.1211	0.1433	G	0.3094	0.8238
8	rs4875320	4164013	A	0.1211	0.1433	T	0.3094	0.8238
10	rs1000797	129652787	A	0.1263	0.1075	G	0.3094	1.2
6	rs2252711	29626321	G	0.3222	0.295	A	0.3095	1.136
8	rs1529316	3828138	G	0.2216	0.197	A	0.3095	1.161
5	rs3733890	78421959	A	0.3247	0.2967	G	0.3097	1.14
8	rs1514626	132765741	G	0.3711	0.4008	A	0.3099	0.8822
19	rs28714142	11771482	C	0.4175	0.3875	A	0.3099	1.133
6	rs2142672	16197194	G	0.2268	0.2542	A	0.3101	0.8608
9	rs7043162	114641291	C	0.2268	0.2542	A	0.3101	0.8608
15	rs11858145	33359574	C	0.1856	0.1625	A	0.3101	1.174
3	rs9290245	100612453	A	0.4197	0.3907	C	0.3102	1.128
6	rs9263726	31106499	A	0.05928	0.07667	G	0.3102	0.7589
5	rs20541	131995964	A	0.2835	0.3125	G	0.3104	0.8705
7	rs6952566	131894868	C	0.4201	0.39	A	0.3104	1.133
17	rs1136201	37879588	G	0.1546	0.1333	A	0.3104	1.189
18	rs1893217	12809340	G	0.1546	0.1333	A	0.3104	1.189
19	rs10413455	58131623	A	0.1546	0.1333	G	0.3104	1.189
20	rs7267828	18239970	G	0.4201	0.39	A	0.3104	1.133
3	rs34690639	172589869	C	0.2835	0.3117	A	0.3105	0.8739
4	rs2276904	1349029	A	0.3737	0.4033	G	0.3105	0.8827
6	rs406238	6172302	A	0.4175	0.3883	C	0.3105	1.129
15	rs3803412	66618342	G	0.2835	0.3117	A	0.3105	0.8739
15	rs3865014	69561518	G	0.3273	0.2988	A	0.3105	1.142
17	rs3785599	64969815	A	0.3737	0.4033	G	0.3105	0.8827
6	rs17062449	102198569	A	0.09794	0.1175	C	0.3108	0.8154
11	rs5015541	6070462	G	0.3273	0.2992	A	0.3108	1.14
17	rs7217319	38924	A	0.07732	0.09599	G	0.3108	0.7892
17	rs2292971	80863857	G	0.3273	0.2992	A	0.3108	1.14
20	rs6125931	48795413	G	0.3763	0.4067	A	0.3108	0.8802
1	rs10864382	9095610	A	0.4227	0.3925	G	0.3109	1.133
1	rs61740234	200880702	A	0.07732	0.095	G	0.3109	0.7983
6	rs10947723	38222018	A	0.4227	0.3925	G	0.3109	1.133

7	rs273957	137600690	G	0.07732	0.095	A	0.3109	0.7983
10	rs34957097	73046617	G	0.07732	0.095	A	0.3109	0.7983
10	rs7069294	73046625	A	0.07732	0.095	G	0.3109	0.7983
13	rs1576562	87950318	G	0.4227	0.3925	A	0.3109	1.133
19	rs717225	42393286	G	0.1856	0.2108	A	0.311	0.8529
1	rs6428492	89467787	G	0.3789	0.4092	A	0.3113	0.8808
1	rs55877187	151230929	A	0.3789	0.4092	C	0.3113	0.8808
2	rs330585	151000108	A	0.3789	0.4092	G	0.3113	0.8808
19	rs10411210	33532300	A	0.1856	0.21	G	0.3113	0.8571
9	rs2131355	71128144	G	0.4253	0.395	A	0.3114	1.133
12	rs542736	299715	G	0.4253	0.395	A	0.3114	1.133
13	rs9562362	42465713	A	0.2242	0.1992	G	0.3114	1.162
1	rs2274328	9009444	C	0.4227	0.3933	A	0.3115	1.129
4	rs2302310	122250734	A	0.4227	0.3933	G	0.3115	1.129
16	rs4787806	26054257	A	0.268	0.2425	G	0.3117	1.144
19	rs7259041	18123738	G	0.2294	0.2567	A	0.3117	0.8621
7	rs2240362	149513077	G	0.2706	0.2437	C	0.3118	1.151
16	rs9921748	81181783	G	0.2294	0.2558	A	0.3118	0.8658
2	rs17036104	108922036	C	0.1057	0.0875	A	0.3119	1.232
10	rs2062988	12143105	C	0.3299	0.3017	G	0.3119	1.14
21	rs378108	40469520	G	0.3814	0.4117	A	0.3119	0.8813
8	rs2073618	119964052	C	0.2706	0.2441	G	0.312	1.149
1	rs192219497	33957267	G	0.02835	0.01917	A	0.3121	1.493
2	rs28930676	105959592	A	0.02835	0.01917	G	0.3121	1.493
5	rs35397	33951116	A	0.02835	0.01917	C	0.3121	1.493
6	rs3130609	32989521	A	0.02835	0.01917	G	0.3121	1.493
6	rs138721292	36931386	A	0.02835	0.01917	G	0.3121	1.493
9	rs189601606	126777726	A	0.02835	0.01917	G	0.3121	1.493
10	rs141881176	124600764	G	0.02835	0.01917	A	0.3121	1.493
13	rs114184584	31858836	A	0.02835	0.01917	T	0.3121	1.493
1	rs2816316	192536813	C	0.2706	0.2442	A	0.3122	1.149
8	rs7817444	54559770	A	0.4304	0.3998	G	0.3122	1.134
10	rs1900004	70000881	A	0.4278	0.3983	G	0.3123	1.129
1	rs28362497	208061142	A	0.03866	0.02833	C	0.3124	1.379
2	rs144829356	170177382	A	0.03866	0.02833	G	0.3124	1.379
2	rs1143674	182374534	A	0.384	0.4142	G	0.3124	0.8818
2	rs2293066	196726471	A	0.03866	0.02833	G	0.3124	1.379
2	rs13394720	234502121	G	0.03866	0.02833	A	0.3124	1.379
3	rs1282549	107175486	A	0.03866	0.02833	G	0.3124	1.379
4	rs1024323	3006043	A	0.2242	0.2	G	0.3124	1.156
8	rs2979182	8325448	G	0.384	0.4142	A	0.3124	0.8818
9	rs140120230	139233113	A	0.03866	0.02833	G	0.3124	1.379
12	rs61733179	6980482	A	0.03866	0.02833	G	0.3124	1.379

14	rs80237479	50092598	G	0.03866	0.02833	A	0.3124	1.379
16	rs12931267	89818732	G	0.03866	0.02833	C	0.3124	1.379
6	rs17688097	27279774	A	0.08505	0.06917	G	0.3125	1.251
11	rs7131178	93462607	T	0.2902	0.318	A	0.3126	0.8765
12	rs1639122	6711147	A	0.3866	0.4172	C	0.3126	0.8803
17	rs11871801	40570772	C	0.1295	0.11	A	0.3126	1.204
17	rs6259	7536527	A	0.1546	0.1344	G	0.3127	1.178
4	rs1128864	77033590	G	0.433	0.4025	A	0.3128	1.134
8	rs11780596	124023538	C	0.4304	0.4008	A	0.3128	1.13
10	rs3858145	70011838	G	0.4304	0.4008	A	0.3128	1.13
1	rs200264075	150444959	A	0.002577	0.008347	G	0.3129	0.307
1	rs1771487	228402121	A	0.1881	0.165	G	0.3129	1.173
1	rs7527165	234524045	A	0.3866	0.4167	G	0.3129	0.8824
11	rs12291017	18158993	G	0.03866	0.02838	A	0.3129	1.377
12	rs188656178	10959252	G	0.002577	0.008347	A	0.3129	0.307
12	rs200080744	49434910	T	0.002577	0.008347	A	0.3129	0.307
14	rs3742642	60591199	A	0.1881	0.165	T	0.3129	1.173
1	rs143264287	33361163	A	0.002577	0.008333	G	0.3131	0.3075
1	rs201360481	33625445	A	0.002577	0.008333	G	0.3131	0.3075
1	rs10159302	57805550	C	0.002577	0.008333	A	0.3131	0.3075
1	rs191538411	62574133	G	0.002577	0.008333	A	0.3131	0.3075
1	rs1047325	153534027	A	0.002577	0.008333	G	0.3131	0.3075
1	rs41267592	179437659	A	0.002577	0.008333	G	0.3131	0.3075
1	rs139797888	201351787	A	0.002577	0.008333	C	0.3131	0.3075
2	rs144915302	26750770	A	0.002577	0.008333	G	0.3131	0.3075
2	rs999890	209190632	C	0.002577	0.008333	A	0.3131	0.3075
2	rs201104642	219894893	A	0.002577	0.008333	T	0.3131	0.3075
3	rs139026854	108220564	G	0.002577	0.008333	A	0.3131	0.3075
3	rs3772836	124535635	A	0.1237	0.145	C	0.3131	0.8325
3	rs143309901	134968200	A	0.002577	0.008333	G	0.3131	0.3075
4	rs138840649	2632766	A	0.002577	0.008333	G	0.3131	0.3075
4	rs139525740	2664692	A	0.002577	0.008333	G	0.3131	0.3075
4	rs28436183	76811175	A	0.002577	0.008333	C	0.3131	0.3075
4	rs202155622	76882438	A	0.002577	0.008333	G	0.3131	0.3075
4	rs150755521	151935743	A	0.002577	0.008333	G	0.3131	0.3075
6	rs142074612	106967260	G	0.002577	0.008333	A	0.3131	0.3075
6	rs147798585	116784682	G	0.002577	0.008333	C	0.3131	0.3075
6	rs142395441	116821709	A	0.002577	0.008333	G	0.3131	0.3075
6	rs140082458	131490312	G	0.002577	0.008333	A	0.3131	0.3075
7	rs149038179	878584	G	0.002577	0.008333	C	0.3131	0.3075
7	rs147656962	2405952	A	0.002577	0.008333	G	0.3131	0.3075
7	rs112568753	51096830	C	0.002577	0.008333	G	0.3131	0.3075
7	rs146220939	51096913	A	0.002577	0.008333	G	0.3131	0.3075

7	rs11768465	100198386	A	0.002577	0.008333	G	0.3131	0.3075
7	rs149190269	140064221	A	0.002577	0.008333	G	0.3131	0.3075
8	rs192256508	53571536	G	0.002577	0.008333	A	0.3131	0.3075
9	rs201074293	2718659	C	0.002577	0.008333	A	0.3131	0.3075
10	rs190798694	91099603	G	0.002577	0.008333	C	0.3131	0.3075
10	rs199925121	127477495	A	0.002577	0.008333	G	0.3131	0.3075
10	rs78371050	127522465	C	0.002577	0.008333	A	0.3131	0.3075
11	rs186519914	4389257	A	0.002577	0.008333	G	0.3131	0.3075
11	rs138329346	11941964	A	0.002577	0.008333	G	0.3131	0.3075
11	rs147522958	64331790	A	0.002577	0.008333	G	0.3131	0.3075
11	rs614367	69328764	A	0.002577	0.008333	G	0.3131	0.3075
11	rs17848372	73716960	A	0.002577	0.008333	G	0.3131	0.3075
11	rs200686731	118498804	A	0.002577	0.008333	G	0.3131	0.3075
12	rs149404188	7548943	G	0.002577	0.008333	A	0.3131	0.3075
12	rs1546308	21176135	A	0.002577	0.008333	G	0.3131	0.3075
12	rs12229654	111414461	C	0.1237	0.1458	A	0.3131	0.8269
12	rs73164912	132562126	A	0.002577	0.008333	G	0.3131	0.3075
13	rs3764522	115091019	C	0.002577	0.008333	G	0.3131	0.3075
14	rs2234637	21469268	A	0.002577	0.008333	G	0.3131	0.3075
14	rs201911072	23415738	A	0.002577	0.008333	G	0.3131	0.3075
14	rs185135187	38724656	A	0.002577	0.008333	G	0.3131	0.3075
14	rs117748213	92470181	A	0.002577	0.008333	G	0.3131	0.3075
14	rs140821122	93397654	A	0.002577	0.008333	C	0.3131	0.3075
14	rs199648972	105964213	A	0.002577	0.008333	G	0.3131	0.3075
15	rs1800401	28260053	A	0.002577	0.008333	G	0.3131	0.3075
15	rs187937579	45707953	G	0.002577	0.008333	A	0.3131	0.3075
15	rs139180466	63097809	A	0.002577	0.008333	G	0.3131	0.3075
15	rs201477273	89864088	A	0.002577	0.008333	G	0.3131	0.3075
16	rs185254286	847877	G	0.002577	0.008333	A	0.3131	0.3075
16	rs201553871	57957231	C	0.002577	0.008333	G	0.3131	0.3075
16	rs35572355	68867247	A	0.002577	0.008333	G	0.3131	0.3075
16	rs140240513	77369732	A	0.002577	0.008333	G	0.3131	0.3075
16	rs147920041	84229883	A	0.002577	0.008333	G	0.3131	0.3075
17	rs140376049	436105	G	0.002577	0.008333	A	0.3131	0.3075
17	rs150008607	10302192	A	0.002577	0.008333	G	0.3131	0.3075
17	rs150351713	10310061	A	0.002577	0.008333	G	0.3131	0.3075
17	rs70937018	17723019	C	0.002577	0.008333	G	0.3131	0.3075
17	rs117556970	18891631	A	0.002577	0.008333	G	0.3131	0.3075
17	rs115534630	39727808	A	0.002577	0.008333	G	0.3131	0.3075
17	rs114337127	47579431	A	0.002577	0.008333	G	0.3131	0.3075
17	rs146684394	72338038	A	0.002577	0.008333	G	0.3131	0.3075
17	rs138029774	78346531	A	0.002577	0.008333	C	0.3131	0.3075
17	rs41299812	78397337	A	0.002577	0.008333	G	0.3131	0.3075

18	rs17659179	47511113	A	0.002577	0.008333	C	0.3131	0.3075
18	rs145324633	51818379	G	0.002577	0.008333	A	0.3131	0.3075
19	rs45572632	18576691	A	0.002577	0.008333	G	0.3131	0.3075
19	rs145753923	33119653	A	0.002577	0.008333	G	0.3131	0.3075
20	rs185740474	9376217	C	0.002577	0.008333	A	0.3131	0.3075
20	rs2275807	35152722	A	0.002577	0.008333	G	0.3131	0.3075
20	rs750077	61526220	G	0.002577	0.008333	C	0.3131	0.3075
21	rs190773441	34951831	A	0.002577	0.008333	C	0.3131	0.3075
22	rs141421532	19026556	A	0.002577	0.008333	G	0.3131	0.3075
22	rs138317708	24217368	A	0.002577	0.008333	G	0.3131	0.3075
22	rs137794	44079680	A	0.002577	0.008333	G	0.3131	0.3075
1	rs1514175	74991644	G	0.1881	0.2133	A	0.3133	0.8546
2	rs2101865	241093031	A	0.4304	0.4015	C	0.3133	1.126
4	rs641041	159884753	G	0.3892	0.4192	A	0.3133	0.8829
11	rs4758042	8129296	A	0.3866	0.4158	C	0.3133	0.8854
2	rs6542670	4050728	G	0.232	0.2592	A	0.3134	0.8633
15	rs7175248	100048722	C	0.232	0.2592	A	0.3134	0.8633
1	rs10462020	7880683	C	0.002577	0.009167	A	0.3135	0.2793
1	rs10462021	7897133	G	0.002577	0.009167	A	0.3135	0.2793
1	rs183852988	110301709	A	0.002577	0.009167	G	0.3135	0.2793
1	rs187465892	153921054	A	0.002577	0.009167	G	0.3135	0.2793
1	rs114050151	205027790	C	0.002577	0.009167	A	0.3135	0.2793
1	rs17416074	205042840	A	0.002577	0.009167	G	0.3135	0.2793
1	rs16855059	205053150	G	0.002577	0.009167	A	0.3135	0.2793
1	rs200458426	212617723	G	0.002577	0.009167	A	0.3135	0.2793
1	rs145808681	226924291	A	0.002577	0.009167	G	0.3135	0.2793
2	rs190968346	1544394	A	0.002577	0.009167	G	0.3135	0.2793
2	rs116518028	37586736	A	0.002577	0.009167	G	0.3135	0.2793
2	rs116094901	73928414	A	0.002577	0.009182	G	0.3135	0.2788
2	rs117022217	170505822	G	0.002577	0.009167	A	0.3135	0.2793
3	rs11914332	111760812	C	0.002577	0.009167	G	0.3135	0.2793
3	rs61740263	184801094	A	0.06701	0.05333	G	0.3135	1.275
4	rs34994651	8228943	A	0.002577	0.009167	G	0.3135	0.2793
4	rs149405506	148827791	A	0.002577	0.009167	C	0.3135	0.2793
5	rs201553806	65083952	C	0.002577	0.009167	A	0.3135	0.2793
5	rs183754401	74442748	A	0.002577	0.009167	G	0.3135	0.2793
5	rs199854306	94857860	G	0.002577	0.009167	A	0.3135	0.2793
5	rs10491334	110772404	A	0.06701	0.05333	G	0.3135	1.275
5	rs1560058	111519740	A	0.002577	0.009167	G	0.3135	0.2793
5	rs890757	111545670	G	0.002577	0.009167	C	0.3135	0.2793
5	rs151160787	140744997	A	0.002577	0.009167	G	0.3135	0.2793
5	rs79602188	178410090	A	0.002577	0.009167	G	0.3135	0.2793
6	rs2844503	31442731	T	0.3938	0.4233	A	0.3135	0.8849

6	rs1042136	33048628	C	0.06701	0.05333	A	0.3135	1.275
6	rs186444567	71003892	A	0.002577	0.009167	T	0.3135	0.2793
7	rs142034862	150554112	G	0.002577	0.009167	C	0.3135	0.2793
7	rs114837285	150746097	A	0.002577	0.009167	G	0.3135	0.2793
8	rs2292927	139838912	A	0.232	0.2583	G	0.3135	0.8671
9	rs56118985	5044432	A	0.002577	0.009167	G	0.3135	0.2793
9	rs138893846	6984169	G	0.002577	0.009167	C	0.3135	0.2793
9	rs190763692	14770803	G	0.002577	0.009167	A	0.3135	0.2793
9	rs142154818	74315561	A	0.002577	0.009167	G	0.3135	0.2793
9	rs3737311	111692144	A	0.002577	0.009167	G	0.3135	0.2793
9	rs75396640	140138702	C	0.002577	0.009167	G	0.3135	0.2793
10	rs182978198	76788267	G	0.002577	0.009167	A	0.3135	0.2793
11	rs74878556	61106544	G	0.002577	0.009167	A	0.3135	0.2793
12	rs78482853	7021983	A	0.002577	0.009167	G	0.3135	0.2793
12	rs939876	65252118	G	0.002577	0.009167	A	0.3135	0.2793
12	rs141338855	66725100	A	0.002577	0.009167	G	0.3135	0.2793
12	rs183845640	124968240	G	0.002577	0.009197	A	0.3135	0.2784
13	rs72474224	20763612	A	0.002577	0.009167	G	0.3135	0.2793
13	rs201344804	75861080	A	0.002577	0.009167	G	0.3135	0.2793
14	rs200690091	59931005	A	0.002577	0.009167	G	0.3135	0.2793
14	rs142378431	91792293	G	0.002577	0.009167	C	0.3135	0.2793
15	rs55876255	41797170	A	0.002577	0.009167	G	0.3135	0.2793
15	rs141858745	48559801	A	0.002577	0.009167	G	0.3135	0.2793
15	rs56015306	85382313	A	0.002577	0.009167	G	0.3135	0.2793
16	rs186487775	4908664	A	0.002577	0.009167	G	0.3135	0.2793
17	rs201753350	7579705	A	0.002577	0.009167	G	0.3135	0.2793
17	rs150213949	34324849	A	0.002577	0.009167	G	0.3135	0.2793
19	rs199569320	8808822	A	0.002577	0.009167	C	0.3135	0.2793
19	rs182087961	14748936	A	0.002577	0.009167	G	0.3135	0.2793
19	rs147972842	51738468	G	0.002577	0.009167	A	0.3135	0.2793
19	rs142282393	56463924	A	0.002577	0.009167	C	0.3135	0.2793
21	rs12483377	46931109	A	0.002577	0.009167	G	0.3135	0.2793
4	rs1054090	2943361	G	0.2268	0.2017	A	0.3136	1.161
19	rs891174	2389872	C	0.4356	0.4058	G	0.3136	1.13
1	rs697842	226813061	G	0.3918	0.4225	A	0.3137	0.8804
8	rs2954033	126493746	A	0.2912	0.32	G	0.3137	0.8732
15	rs8035957	38838264	A	0.3918	0.4225	G	0.3137	0.8804
4	rs950063	126431919	G	0.2732	0.2467	A	0.3138	1.148
5	rs10478508	120508561	A	0.3892	0.4183	G	0.3138	0.8859
12	rs14121	95694394	G	0.2912	0.3192	A	0.3139	0.8765
6	rs16897557	27279632	A	0.06701	0.05342	C	0.314	1.273
11	rs4755454	32903263	G	0.4381	0.4083	A	0.314	1.13
12	rs10784750	69424967	A	0.4381	0.4083	G	0.314	1.13

12	rs7138906	91277503	A	0.4381	0.4083	G	0.314	1.13
17	rs7406910	46688256	A	0.1572	0.1358	G	0.314	1.187
17	rs78023288	76420085	G	0.1289	0.11	A	0.314	1.197
1	rs563939	5347155	G	0.3943	0.425	A	0.3141	0.8809
13	rs9565152	75915261	C	0.1057	0.08833	A	0.3141	1.219
17	rs7220988	44109474	A	0.1057	0.08833	G	0.3141	1.219
7	rs801524	18706918	G	0.4356	0.4067	A	0.3143	1.126
13	rs7990216	25900838	G	0.4356	0.4067	A	0.3143	1.126
10	rs7071471	51503335	A	0.3969	0.4273	G	0.3144	0.8822
12	rs1897639	82346908	G	0.4407	0.4108	A	0.3144	1.13
22	rs4823006	29451671	A	0.4407	0.4108	G	0.3144	1.13
10	rs10466126	135186806	A	0.06701	0.05351	G	0.3145	1.27
9	rs2153769	75706106	G	0.3969	0.4267	A	0.3147	0.8843
12	rs1800378	6172202	A	0.3351	0.3075	G	0.3147	1.135
1	rs3007729	18795255	A	0.3995	0.43	G	0.3149	0.8818
1	rs2171584	92241776	A	0.2938	0.3217	G	0.315	0.8774
3	rs10511089	84481068	A	0.2345	0.2617	C	0.315	0.8645
6	rs2074488	31240431	A	0.2758	0.2487	C	0.315	1.15
7	rs10244620	113315926	A	0.3376	0.3092	G	0.315	1.139
11	rs7930940	60525786	A	0.2345	0.2617	G	0.315	0.8645
11	rs644799	95564259	G	0.3376	0.3092	A	0.315	1.139
17	rs321612	33881631	A	0.2345	0.2617	G	0.315	0.8645
17	rs321613	33881718	G	0.2345	0.2617	A	0.315	0.8645
13	rs12859773	101453470	G	0.4459	0.4158	A	0.3151	1.13
2	rs6542522	120078778	G	0.3995	0.4292	A	0.3152	0.8848
6	rs35771565	29012067	A	0.4021	0.4325	G	0.3153	0.8823
8	rs4317609	117467008	A	0.4021	0.4325	G	0.3153	0.8823
11	rs4509745	7712471	G	0.4433	0.4142	A	0.3154	1.126
15	rs2277552	69339787	A	0.1907	0.2158	G	0.3155	0.8562
7	rs6958498	107834613	C	0.1005	0.12	G	0.3156	0.8195
1	rs35269416	1900186	G	0.1572	0.1367	A	0.3157	1.178
6	rs3130151	33115024	A	0.4459	0.4167	G	0.3157	1.127
6	rs6901953	123699042	G	0.1572	0.1367	A	0.3157	1.178
8	rs7459793	125755750	A	0.4459	0.4167	G	0.3157	1.127
10	rs6583810	94116233	A	0.4046	0.435	G	0.3157	0.8828
17	rs2286806	53485186	G	0.4459	0.4167	A	0.3157	1.127
14	rs229587	65263300	A	0.2964	0.325	G	0.3158	0.8749
17	rs197932	44974347	G	0.2964	0.325	A	0.3158	0.8749
2	rs4954379	138332365	A	0.3402	0.3117	G	0.316	1.139
6	rs2274089	25488583	A	0.05155	0.04	G	0.316	1.304
6	rs4708204	76682252	A	0.4485	0.4192	G	0.316	1.127
11	rs7123133	95712897	A	0.05155	0.04	C	0.316	1.304
11	rs1290018	123545176	A	0.4485	0.4192	G	0.316	1.127

20	rs139946022	31680288	A	0.05155	0.04	G	0.316	1.304
21	exm-IND21-4241	43543115	I	0.05155	0.04	D	0.316	1.304
6	rs6457121	29963065	A	0.4536	0.4233	G	0.3161	1.131
7	rs1558557	8308993	G	0.4536	0.4233	A	0.3161	1.131
17	rs3930821	20697797	A	0.4536	0.4233	C	0.3161	1.131
3	rs13087528	82280224	G	0.5	0.4691	A	0.3163	1.132
4	rs3733418	165878335	A	0.451	0.4217	G	0.3163	1.127
6	rs2240619	29975453	C	0.4562	0.4257	A	0.3163	1.132
6	rs510579	165713961	C	0.2758	0.25	A	0.3163	1.142
7	rs2037677	137327157	A	0.08505	0.07	G	0.3163	1.235
10	rs17107368	82369219	A	0.451	0.4217	T	0.3163	1.127
10	rs10490924	124214448	A	0.451	0.4217	C	0.3163	1.127
12	rs3742049	120954490	A	0.08505	0.07	G	0.3163	1.235
15	rs2304580	41029667	A	0.2758	0.25	G	0.3163	1.142
16	rs30208	14428853	G	0.451	0.4217	A	0.3163	1.127
4	rs12500918	153669008	A	0.4098	0.44	C	0.3165	0.8837
6	rs6457126	29983039	G	0.4562	0.4258	A	0.3165	1.131
6	rs3869070	30023868	G	0.4562	0.4258	A	0.3165	1.131
13	rs9522407	89637081	A	0.4562	0.4258	G	0.3165	1.131
1	rs3737972	203766964	G	0.3402	0.3125	A	0.3166	1.134
3	rs753331	38348743	C	0.2371	0.2642	A	0.3166	0.8658
4	rs1450500	93479275	G	0.4536	0.4242	A	0.3166	1.127
6	rs2076185	11723636	A	0.4536	0.4242	G	0.3166	1.127
8	rs2726990	27766880	G	0.4536	0.4242	A	0.3166	1.127
12	rs1385374	129300694	A	0.2294	0.205	G	0.3166	1.154
6	rs9367358	49485266	C	0.4124	0.4424	A	0.3167	0.8845
1	rs3738178	220971339	A	0.0799	0.09833	T	0.3168	0.7962
1	rs1805087	237048500	G	0.1263	0.1483	A	0.3168	0.8299
2	rs1820965	10059770	C	0.4588	0.4283	A	0.3168	1.131
4	rs28622301	90170118	G	0.1263	0.1483	A	0.3168	0.8299
4	rs6814242	186959365	G	0.4588	0.4283	A	0.3168	1.131
19	rs17271272	46543566	A	0.0799	0.09833	G	0.3168	0.7962
1	rs11211247	46476587	A	0.01804	0.01167	C	0.3169	1.556
1	rs11552561	47653037	A	0.03351	0.04674	G	0.3169	0.707
1	rs140022518	109377140	A	0.01804	0.01167	C	0.3169	1.556
1	rs11537583	157805650	A	0.1907	0.1683	T	0.3169	1.164
1	rs2252483	182333131	G	0.4562	0.4267	A	0.3169	1.127
1	rs200168581	197890684	A	0.01804	0.01167	G	0.3169	1.556
1	rs41271527	232941215	G	0.01804	0.01167	A	0.3169	1.556
3	rs33955793	12544829	A	0.01804	0.01167	G	0.3169	1.556
3	rs140444427	64666931	A	0.01804	0.01167	G	0.3169	1.556
4	rs116952023	5755529	C	0.01804	0.01167	A	0.3169	1.556
4	rs117303916	152212475	G	0.01804	0.01167	A	0.3169	1.556

5	rs17421627	87847586	C	0.01804	0.01167	A	0.3169	1.556
5	rs76224953	131007358	A	0.01804	0.01167	G	0.3169	1.556
6	rs763469	33004387	A	0.04639	0.06083	G	0.3169	0.7511
6	rs75133930	34826672	G	0.01804	0.01167	A	0.3169	1.556
6	rs3828741	87725674	A	0.01804	0.01167	G	0.3169	1.556
6	rs138160934	110778060	G	0.01804	0.01167	C	0.3169	1.556
8	rs16892767	120577150	A	0.01804	0.01167	G	0.3169	1.556
8	rs77613350	139712355	A	0.01804	0.01167	G	0.3169	1.556
9	rs140132974	138589474	A	0.01804	0.01167	G	0.3169	1.556
11	rs150176794	66360206	A	0.01804	0.01167	C	0.3169	1.556
11	rs12804616	111102839	A	0.2784	0.2517	G	0.3169	1.147
12	rs79109432	72022747	T	0.01804	0.01167	A	0.3169	1.556
12	rs77466370	95604074	G	0.01804	0.01167	A	0.3169	1.556
13	rs201543678	108922556	A	0.01804	0.01167	G	0.3169	1.556
15	rs28399430	75012894	C	0.01804	0.01167	G	0.3169	1.556
17	rs17566772	39023381	A	0.01804	0.01167	G	0.3169	1.556
17	rs7212483	39041052	A	0.01804	0.01167	G	0.3169	1.556
19	rs3848635	2410332	G	0.4562	0.4267	A	0.3169	1.127
19	rs7257762	3281586	A	0.1907	0.1683	G	0.3169	1.164
19	rs147272153	44006318	A	0.01804	0.01167	G	0.3169	1.556
19	rs183892712	49652888	A	0.01804	0.01167	C	0.3169	1.556
19	rs2074059	57985460	A	0.2784	0.2517	T	0.3169	1.147
19	rs2074060	57985566	G	0.2784	0.2517	C	0.3169	1.147
20	rs142953196	32685262	A	0.03368	0.04698	G	0.3169	0.707
20	rs115303435	62326159	A	0.03351	0.04674	G	0.3169	0.707
1	rs41273179	19235138	A	0.03351	0.04667	G	0.317	0.7082
15	rs2241268	86278309	A	0.03351	0.04667	G	0.317	0.7082
17	rs79254649	34304605	A	0.03351	0.04667	C	0.317	0.7082
17	rs61739953	46877063	A	0.03351	0.04667	G	0.317	0.7082
17	rs142677234	48776886	G	0.03351	0.04667	A	0.317	0.7082
22	rs135052	43843065	G	0.03351	0.04667	A	0.317	0.7082
1	rs2230301	220197625	C	0.0799	0.0975	A	0.3171	0.8038
6	rs1355931	68705526	G	0.06186	0.07833	A	0.3171	0.7758
8	rs12680546	136871739	G	0.0799	0.0975	A	0.3171	0.8038
10	rs11544712	118387282	A	0.4613	0.4308	G	0.3171	1.131
11	rs75384502	94861862	G	0.06186	0.07833	A	0.3171	0.7758
2	rs4672596	211648806	G	0.299	0.3267	A	0.3172	0.8791
3	rs2270781	113015660	A	0.4124	0.4417	G	0.3172	0.8871
6	rs3130604	32985052	G	0.04639	0.06167	A	0.3172	0.7402
6	rs943463	33719877	A	0.04639	0.06167	G	0.3172	0.7402
8	rs35522859	17749317	C	0.04639	0.06167	G	0.3172	0.7402
9	rs670205	29595350	G	0.4124	0.4417	A	0.3172	0.8871
9	rs2073817	136559460	A	0.4588	0.4292	G	0.3172	1.127

14	rs10872871	33386759	A	0.4149	0.445	G	0.3172	0.8846
3	rs9837834	7142227	G	0.4639	0.4333	A	0.3174	1.132
3	rs11924144	185845583	C	0.4613	0.4317	A	0.3174	1.128
10	rs3793917	124219275	C	0.4613	0.4317	G	0.3174	1.128
10	rs11200638	124220544	A	0.4613	0.4317	G	0.3174	1.128
20	rs6137297	21218023	G	0.4639	0.4333	A	0.3174	1.132
1	rs11264625	157128705	A	0.1598	0.1383	G	0.3175	1.185
1	rs6427356	157130566	A	0.1598	0.1383	G	0.3175	1.185
3	rs6800541	38774832	G	0.1598	0.1383	A	0.3175	1.185
2	rs6736922	219903258	C	0.4149	0.4442	A	0.3176	0.8876
2	rs4674354	219903723	A	0.4149	0.4442	G	0.3176	0.8876
5	rs10474347	90566498	A	0.4201	0.4508	G	0.3176	0.8827
6	rs10946999	29571609	A	0.2784	0.2525	C	0.3177	1.142
6	rs6909253	30055643	C	0.4639	0.4342	A	0.3177	1.128
11	rs3812738	28129982	A	0.4639	0.4342	C	0.3177	1.128
12	rs7297754	63443521	A	0.4639	0.4342	G	0.3177	1.128
13	rs912007	114007887	A	0.2784	0.2525	G	0.3177	1.142
7	rs9640663	77247821	G	0.1082	0.09	A	0.3178	1.227
11	rs2846707	102576358	A	0.1082	0.09	G	0.3178	1.227
11	rs11225389	102576468	A	0.1082	0.09	C	0.3178	1.227
11	rs3814762	119216504	A	0.1933	0.2192	G	0.3178	0.8537
12	rs1462978	89204842	G	0.1082	0.09	A	0.3178	1.227
17	rs434473	6904934	G	0.4201	0.4499	A	0.3178	0.8857
17	rs61744544	76522785	A	0.06186	0.07917	G	0.3178	0.7669
19	rs10408163	47597102	G	0.1933	0.2192	A	0.3178	0.8537
5	rs34241005	125923632	A	0.1572	0.1808	G	0.3179	0.845
6	rs2857697	31585219	G	0.4227	0.4533	A	0.3179	0.8829
6	rs2857694	31587870	T	0.4227	0.4533	A	0.3179	0.8829
6	rs6934603	87994015	A	0.4665	0.4367	G	0.3179	1.128
8	rs3739238	142222445	A	0.1572	0.1808	G	0.3179	0.845
13	rs9573096	73649333	A	0.3015	0.33	G	0.3179	0.8766
18	rs2271733	56940307	A	0.1572	0.1808	C	0.3179	0.845
21	rs1297102	15384814	A	0.1572	0.1808	G	0.3179	0.845
1	rs684371	60579518	A	0.4175	0.4467	G	0.318	0.888
4	rs4253325	187178473	A	0.3454	0.3167	G	0.318	1.138
1	rs4630153	63881552	G	0.1572	0.18	A	0.3181	0.8498
1	rs2228693	66509014	A	0.4227	0.4525	C	0.3181	0.8859
5	rs11556045	73985215	G	0.1572	0.18	A	0.3181	0.8498
1	rs10917809	161701810	A	0.3472	0.3192	G	0.3182	1.134
2	rs11563140	52010562	G	0.4253	0.4558	A	0.3182	0.8833
1	rs4654925	20227723	C	0.2809	0.2542	G	0.3184	1.146
2	rs1446575	177047801	A	0.2397	0.2658	C	0.3184	0.8707
6	rs7756521	30848253	A	0.4253	0.455	G	0.3184	0.8863

6	rs4618569	30855251	G	0.4253	0.455	A	0.3184	0.8863
6	rs3117231	33074908	G	0.4716	0.4417	A	0.3184	1.128
6	rs210170	33525680	G	0.4253	0.455	A	0.3184	0.8863
19	rs17570	33878837	A	0.1933	0.17	G	0.3184	1.17
22	rs14136	51048721	A	0.1933	0.17	G	0.3184	1.17
6	rs2285801	30209493	G	0.3454	0.3175	A	0.3185	1.134
9	rs1975197	8846955	A	0.3454	0.3175	G	0.3185	1.134
9	rs11790577	34397545	G	0.4278	0.4583	A	0.3185	0.8837
16	rs4077410	29998200	G	0.3454	0.3175	A	0.3185	1.134
17	rs34562254	16842991	A	0.3454	0.3175	G	0.3185	1.134
1	rs1535872	64615775	G	0.4742	0.4442	A	0.3186	1.129
5	rs17054522	156770049	C	0.03351	0.0475	G	0.3186	0.6952
6	rs142919790	87968265	C	0.03351	0.0475	G	0.3186	0.6952
6	rs9362415	87968565	A	0.4665	0.4375	G	0.3186	1.124
6	rs9353479	88058596	A	0.03351	0.0475	G	0.3186	0.6952
11	rs74562598	55587748	A	0.03351	0.0475	G	0.3186	0.6952
13	rs9518946	103831756	G	0.4665	0.4375	A	0.3186	1.124
15	rs7173743	79141784	G	0.4742	0.4442	A	0.3186	1.129
19	rs3745953	38852357	C	0.03351	0.0475	A	0.3186	0.6952
20	rs6074678	14031392	A	0.4742	0.4442	G	0.3186	1.129
7	rs4490737	34416608	A	0.4304	0.4608	G	0.3187	0.8841
14	rs2775254	20528528	G	0.4278	0.4575	A	0.3187	0.8867
19	rs3764645	1042809	G	0.4278	0.4575	A	0.3187	0.8867
9	rs10869758	78965376	G	0.4768	0.4467	A	0.3188	1.129
2	rs741326	71058835	A	0.3041	0.3325	G	0.3189	0.8774
16	rs2502726	70972595	G	0.4794	0.4491	A	0.3189	1.13
1	rs545809	24690764	A	0.433	0.4633	T	0.319	0.8845
1	rs10888561	153410822	C	0.433	0.4633	A	0.319	0.8845
1	rs72704099	161697072	C	0.3479	0.3192	G	0.319	1.138
19	rs2571051	44979443	G	0.433	0.4633	A	0.319	0.8845
9	rs13298768	130489743	G	0.2809	0.255	A	0.3192	1.141
12	rs3741628	49048738	C	0.2809	0.255	A	0.3192	1.141
1	rs3736930	198505830	A	0.06959	0.055	G	0.3193	1.285
7	rs6952624	20960488	A	0.4768	0.4475	G	0.3193	1.125
8	rs2294015	124696867	A	0.4768	0.4475	G	0.3193	1.125
2	rs4849167	113940839	C	0.3041	0.3317	G	0.3194	0.8807
6	rs2596473	31430799	C	0.456	0.4867	A	0.3194	0.884
7	rs17166351	128597804	G	0.4845	0.4542	A	0.3194	1.13
8	rs2252399	52951973	A	0.4381	0.4683	C	0.3194	0.8853
15	rs12440440	34041896	A	0.3041	0.3317	G	0.3194	0.8807
5	rs6879065	172737199	G	0.4794	0.45	A	0.3195	1.125
6	rs743399	31749804	A	0.2423	0.2688	G	0.3196	0.8698
7	rs6466677	118571442	C	0.4871	0.4567	A	0.3196	1.13

8	rs17466684	27452847	A	0.1933	0.1708	G	0.3196	1.163
18	rs7235585	9478794	G	0.4407	0.4708	A	0.3196	0.8856
3	rs6785504	152672779	A	0.4897	0.4592	C	0.3197	1.13
8	rs59331088	74005131	G	0.2423	0.2692	A	0.3197	0.8681
9	rs1180117	72897440	G	0.2435	0.27	A	0.3197	0.8704
11	rs2276036	66033430	A	0.2423	0.2692	G	0.3197	0.8681
20	rs944895	60887581	G	0.2423	0.2692	A	0.3197	0.8681
21	rs2823109	16537728	C	0.4897	0.4592	A	0.3197	1.13
8	rs4236772	105185621	G	0.4381	0.4675	A	0.3198	0.8882
17	rs3760318	29247715	A	0.1959	0.2208	G	0.3198	0.8595
19	rs2303108	47589895	G	0.1959	0.2208	A	0.3198	0.8595
1	rs56222536	216258213	G	0.0232	0.03417	A	0.3199	0.6713
7	rs1526520	11779718	A	0.4923	0.4617	G	0.3199	1.131
7	rs34587586	129938598	A	0.3067	0.3358	C	0.3199	0.8749
9	rs76974239	34256634	A	0.0232	0.03417	G	0.3199	0.6713
11	rs74787566	124744033	A	0.0232	0.03417	G	0.3199	0.6713
17	rs7219146	14450777	A	0.4897	0.4599	G	0.3199	1.127
17	rs3736238	27208326	A	0.0232	0.03417	G	0.3199	0.6713
21	rs1805127	35821821	A	0.2835	0.2567	G	0.3199	1.146
22	rs11704009	19840123	G	0.0232	0.03417	A	0.3199	0.6713
3	rs9990174	11040439	A	0.232	0.2083	C	0.32	1.148
3	rs1372072	16955259	G	0.4897	0.46	A	0.32	1.126
4	rs6839940	184242959	C	0.2423	0.2683	G	0.32	0.8718
7	rs2073793	92764419	G	0.08763	0.07167	A	0.32	1.244
13	rs9556688	97835876	A	0.4459	0.4758	G	0.32	0.8864
3	rs6785912	135288025	G	0.4407	0.47	A	0.3201	0.8886
1	rs10797437	2539006	G	0.4948	0.465	A	0.3202	1.127
1	rs10797440	2541269	G	0.4948	0.465	A	0.3202	1.127
1	rs3748735	208390469	A	0.1031	0.1233	G	0.3202	0.817
2	rs1837495	198921201	G	0.4485	0.4783	A	0.3202	0.8867
8	rs4734653	103663814	A	0.1031	0.1233	G	0.3202	0.817
10	rs2742234	43612609	A	0.4948	0.465	G	0.3202	1.127
12	rs7978999	102368065	A	0.4485	0.4783	G	0.3202	0.8867
13	rs1335787	64585809	A	0.451	0.4817	G	0.3202	0.8841
1	rs76625876	6528468	A	0.007732	0.01583	G	0.3203	0.4843
1	rs61740145	6531124	G	0.007732	0.01583	A	0.3203	0.4843
1	rs192833530	24140734	G	0.007732	0.01583	A	0.3203	0.4843
1	rs151324745	26664907	C	0.0232	0.035	A	0.3203	0.6547
1	rs7547485	145021126	A	0.007732	0.01583	G	0.3203	0.4843
2	rs79064914	113343886	A	0.007732	0.01583	G	0.3203	0.4843
2	rs78446341	160690656	A	0.007732	0.01583	G	0.3203	0.4843
2	rs191482653	179422112	A	0.007732	0.01583	G	0.3203	0.4843
2	rs184529696	210559239	A	0.007732	0.01583	G	0.3203	0.4843

2	rs1574192	241308505	A	0.007732	0.01583	G	0.3203	0.4843
3	rs2303857	32578505	G	0.0232	0.035	A	0.3203	0.6547
3	rs143290812	130368098	A	0.0232	0.035	G	0.3203	0.6547
3	rs2306374	138119952	G	0.007732	0.01583	A	0.3203	0.4843
5	rs2303743	38493874	G	0.0232	0.035	A	0.3203	0.6547
5	rs6875401	62456005	G	0.007732	0.01583	A	0.3203	0.4843
5	rs261001	169335129	A	0.4433	0.4725	G	0.3203	0.889
6	rs138620921	33172479	A	0.007732	0.01583	C	0.3203	0.4843
6	rs199838681	33694588	A	0.007732	0.01583	G	0.3203	0.4843
6	rs143979330	51944763	A	0.007732	0.01583	G	0.3203	0.4843
7	rs6460939	12406989	C	0.4536	0.4842	G	0.3203	0.8845
8	rs2272720	2021421	A	0.3479	0.3208	C	0.3203	1.13
8	rs117958007	103564056	A	0.007732	0.01583	G	0.3203	0.4843
10	rs145152071	15146020	G	0.007732	0.01583	A	0.3203	0.4843
10	rs3740168	16948390	C	0.1031	0.1225	G	0.3203	0.8234
10	rs2615876	117665860	A	0.007732	0.01583	G	0.3203	0.4843
11	rs490592	116298367	A	0.0232	0.035	C	0.3203	0.6547
12	rs138634363	31944576	A	0.007732	0.01583	G	0.3203	0.4843
12	rs4767884	120661977	A	0.007732	0.01583	G	0.3203	0.4843
13	rs4884216	83963488	A	0.1031	0.1225	G	0.3203	0.8234
15	rs275183	39444966	A	0.1031	0.1225	G	0.3203	0.8234
16	rs117066066	3339413	C	0.007732	0.01583	G	0.3203	0.4843
16	rs150082808	69973023	G	0.007732	0.01583	A	0.3203	0.4843
17	rs12150518	15406197	A	0.007732	0.01583	T	0.3203	0.4843
17	rs142471902	76568872	C	0.0232	0.03506	A	0.3203	0.6536
19	rs527245	55434502	G	0.4433	0.4725	A	0.3203	0.889
19	rs3745904	55493970	G	0.007732	0.01583	C	0.3203	0.4843
22	rs143775693	30688611	C	0.007732	0.01583	G	0.3203	0.4843
4	rs1441433	102209360	A	0.5	0.47	C	0.3204	1.128
6	rs2016588	159425707	A	0.5	0.47	G	0.3204	1.128
10	rs1211188	86296621	A	0.4562	0.4867	G	0.3204	0.8848
21	rs461769	24474928	A	0.4562	0.4867	C	0.3204	0.8848
6	rs9377619	104593867	G	0.1289	0.15	A	0.3205	0.8383
6	rs575131	128979455	A	0.4536	0.4833	C	0.3205	0.8874
11	rs10896053	65579670	A	0.4588	0.4892	G	0.3205	0.8852
1	rs2201841	67694202	A	0.2835	0.2575	G	0.3206	1.141
2	rs34577613	152527608	G	0.4562	0.4858	A	0.3207	0.8878
3	rs1001817	183995341	A	0.4562	0.4858	G	0.3207	0.8878
6	rs7751158	89162999	A	0.5129	0.4825	G	0.3207	1.129
6	rs2807278	131809920	G	0.1598	0.1833	A	0.3207	0.8472
8	rs2439322	32520472	G	0.5129	0.4825	A	0.3207	1.129
11	rs2126709	123595927	A	0.1598	0.1833	G	0.3207	0.8472
15	rs3812907	97327393	A	0.1598	0.1833	G	0.3207	0.8472

17	rs9896398	76503593	G	0.4923	0.4633	A	0.3207	1.123
1	rs3766160	15808872	G	0.4588	0.4883	A	0.3208	0.8881
5	rs26001	11303908	A	0.5155	0.485	G	0.3208	1.13
6	rs2259571	31583827	C	0.518	0.4875	A	0.3208	1.13
6	rs805262	31628733	A	0.518	0.4875	G	0.3208	1.13
9	rs3761847	123690239	G	0.4691	0.4992	A	0.3208	0.8864
10	rs11014595	25822949	C	0.4768	0.5075	A	0.3208	0.8844
11	rs3741280	117403235	C	0.4691	0.4992	A	0.3208	0.8864
14	rs2250718	100380948	A	0.4588	0.4883	G	0.3208	0.8881
17	rs7220955	76700063	A	0.518	0.4875	G	0.3208	1.13
19	rs329746	53539860	A	0.5155	0.485	G	0.3208	1.13
22	rs1049534	29908072	A	0.518	0.4875	G	0.3208	1.13
2	rs12469465	188800394	A	0.4974	0.4683	G	0.3209	1.124
3	rs6762208	185331165	A	0.3093	0.3383	C	0.3209	0.8757
4	rs2703129	77818548	G	0.1598	0.1825	A	0.3209	0.8519
6	rs10946113	158584691	C	0.4716	0.5017	A	0.3209	0.8868
9	rs1333049	22125503	G	0.4948	0.4658	C	0.3209	1.123
9	rs1984582	24477128	G	0.5206	0.4908	A	0.3209	1.127
17	rs77630516	34195739	G	0.1598	0.1825	A	0.3209	0.8519
19	rs890871	55672055	G	0.2448	0.2722	A	0.3209	0.8669
6	rs6899309	32915823	G	0.134	0.1142	A	0.321	1.201
8	rs1871686	109571545	G	0.5026	0.4733	A	0.321	1.124
13	rs9544789	78994226	G	0.5026	0.4733	A	0.321	1.124
6	rs2255625	30940705	G	0.1959	0.1725	A	0.3211	1.169
6	rs2517439	30942929	A	0.1959	0.1725	G	0.3211	1.169
6	rs2844682	30946148	A	0.1959	0.1725	G	0.3211	1.169
6	rs2517424	30949996	T	0.1959	0.1725	A	0.3211	1.169
10	rs10903387	1207809	G	0.5103	0.4808	A	0.3211	1.125
10	rs2182169	100148004	A	0.4639	0.4933	G	0.3211	0.8888
11	rs12807582	39327563	C	0.518	0.4883	A	0.3211	1.126
15	rs1042630	89402051	A	0.5103	0.4808	G	0.3211	1.125
16	rs7499886	66413195	G	0.5077	0.4783	A	0.3211	1.125
19	rs17628	39926509	A	0.518	0.4883	C	0.3211	1.126
3	rs11917356	130110550	A	0.3531	0.325	G	0.3212	1.134
3	rs10937405	189383183	A	0.3531	0.325	G	0.3212	1.134
6	rs3132643	30340145	G	0.3505	0.3233	A	0.3212	1.129
6	rs3132629	30346093	C	0.3505	0.3233	A	0.3212	1.129
6	rs3130133	30350087	A	0.3505	0.3233	C	0.3212	1.129
6	rs3130122	30352527	G	0.3505	0.3233	A	0.3212	1.129
6	rs3130144	30358522	A	0.3505	0.3233	G	0.3212	1.129
6	rs3094698	30361193	A	0.3505	0.3233	G	0.3212	1.129
6	rs3130109	30362751	A	0.3505	0.3233	C	0.3212	1.129
9	rs753033	115676986	A	0.2448	0.2717	G	0.3212	0.8693

21	rs2020945	45528919	G	0.2448	0.2717	A	0.3212	0.8693
6	rs3905495	31265539	G	0.4691	0.4983	A	0.3213	0.8894
17	rs231678	3214181	A	0.4691	0.4983	C	0.3213	0.8894
13	rs9300734	102959556	G	0.4716	0.5008	A	0.3214	0.8897
8	rs4733951	38369831	A	0.2448	0.2708	C	0.3215	0.8729
21	rs1131999	45506819	G	0.2448	0.2708	A	0.3215	0.8729
5	rs349128	141080897	G	0.3119	0.3408	A	0.3218	0.8765
1	rs7528153	108307727	A	0.3119	0.34	T	0.3219	0.8797
7	rs1147504	121653398	G	0.3119	0.34	A	0.3219	0.8797
14	rs8022091	70655751	A	0.3557	0.3275	C	0.3221	1.133
5	rs2277027	156932376	C	0.1959	0.1733	A	0.3222	1.162
5	rs1422795	156936364	G	0.1959	0.1733	A	0.3222	1.162
1	rs12750774	154516477	A	0.05412	0.04167	G	0.3225	1.316
3	rs2687729	127895226	G	0.08247	0.1008	A	0.3227	0.8016
4	rs74643640	123113428	G	0.08247	0.1008	A	0.3227	0.8016
4	rs2306369	123268859	G	0.08247	0.1008	A	0.3227	0.8016
11	rs3751066	8947283	C	0.2474	0.2742	G	0.3227	0.8704
12	rs11171511	55894216	A	0.08247	0.1008	G	0.3227	0.8016
12	rs11835716	55945258	G	0.08247	0.1008	A	0.3227	0.8016
1	rs11164337	102389733	G	0.2887	0.2617	A	0.3228	1.145
15	rs10873636	26888978	A	0.134	0.115	C	0.3228	1.191
1	rs475508	112385547	G	0.3557	0.3283	A	0.3229	1.129
5	rs200723635	109190938	G	0.3582	0.33	A	0.323	1.133
6	rs1480382	32740895	A	0.3582	0.33	G	0.323	1.133
17	rs6065	4836381	A	0.1082	0.09167	G	0.323	1.203
16	rs2345080	6602397	A	0.06959	0.05583	G	0.3231	1.265
19	rs2365725	55672470	G	0.06959	0.05583	A	0.3231	1.265
7	rs7804414	29424644	G	0.2887	0.2625	A	0.3234	1.14
11	rs12279261	113106455	G	0.08763	0.0725	A	0.3235	1.229
14	rs7492332	58497641	A	0.317	0.3458	G	0.3236	0.878
10	rs1801224	17147521	C	0.3582	0.3308	A	0.3237	1.129
3	rs75748256	195934276	G	0.04124	0.03	A	0.3238	1.391
4	rs6536541	161767814	G	0.1314	0.1533	A	0.3238	0.8356
9	rs2066479	98997810	A	0.2371	0.2133	G	0.3238	1.146
12	rs1458175	41965861	A	0.1314	0.1533	C	0.3238	0.8356
12	rs7312075	85432040	A	0.2371	0.2133	T	0.3238	1.146
15	rs149974542	52504009	A	0.04124	0.03	G	0.3238	1.391
16	rs3747582	4511926	A	0.2371	0.2133	G	0.3238	1.146
19	rs117192179	57803539	A	0.04124	0.03	G	0.3238	1.391
20	rs117729108	33591013	C	0.04124	0.03	G	0.3238	1.391
21	rs61735836	47572887	A	0.1314	0.1533	G	0.3238	0.8356
1	rs562556	55524237	G	0.02835	0.02	A	0.3239	1.43
1	rs3795783	228467711	A	0.3608	0.3325	G	0.3239	1.133

6	rs2395165	32388144	G	0.02835	0.02	A	0.3239	1.43
8	rs76070333	95416309	A	0.02835	0.02	T	0.3239	1.43
12	rs148108557	53215816	A	0.02835	0.02	C	0.3239	1.43
16	rs2305658	4802874	A	0.02835	0.02	G	0.3239	1.43
19	rs4252572	55873726	A	0.02835	0.02	G	0.3239	1.43
2	rs3795958	26667130	G	0.201	0.2267	A	0.324	0.8584
10	rs874556	70992664	G	0.201	0.2258	A	0.324	0.8625
12	rs11063529	657404	A	0.1985	0.1753	G	0.324	1.165
4	rs10517133	45364914	C	0.25	0.2767	G	0.3242	0.8715
6	rs176095	32158319	G	0.25	0.2767	A	0.3242	0.8715
7	rs2288967	132102811	C	0.25	0.2775	A	0.3242	0.8679
1	rs74685771	989207	C	0.02835	0.02003	G	0.3244	1.427
2	rs11888559	204165171	A	0.1649	0.1433	G	0.3244	1.181
12	rs7966162	77154974	A	0.3608	0.3333	G	0.3245	1.129
17	rs3826496	3564073	G	0.2397	0.215	A	0.3246	1.151
17	rs760274	3564084	G	0.2397	0.215	A	0.3246	1.151
2	rs1056836	38298203	C	0.1057	0.126	G	0.3247	0.8193
6	rs1264352	30789647	C	0.06443	0.08083	G	0.3247	0.7831
19	rs2569491	51584916	A	0.06443	0.08083	G	0.3247	0.7831
19	rs35287116	51584951	G	0.06443	0.08083	A	0.3247	0.7831
12	rs7298766	661656	G	0.1985	0.1758	A	0.3248	1.16
4	rs2119788	174447389	A	0.3238	0.3525	G	0.325	0.8797
5	rs75714066	112889370	G	0.06443	0.08167	C	0.3252	0.7744
22	rs13053175	37613309	A	0.06443	0.08167	G	0.3252	0.7744
1	rs4319261	24421950	G	0.3634	0.3358	C	0.3253	1.129
9	rs4149268	107647220	A	0.3634	0.3358	G	0.3253	1.129
1	rs5938	28476862	A	0.1108	0.09333	C	0.3254	1.211
6	rs2281023	27277665	A	0.1366	0.1167	G	0.3254	1.198
20	rs13036385	31671209	G	0.1366	0.1167	C	0.3254	1.198
7	rs2640	6066450	G	0.366	0.3375	A	0.3255	1.133
3	rs140693	129155451	A	0.3679	0.34	G	0.3257	1.13
1	rs3795263	2938989	A	0.1649	0.1442	G	0.3258	1.173
1	rs143437991	22176542	A	0.007732	0.01667	G	0.3258	0.4597
1	rs118092780	110582038	A	0.007732	0.01667	G	0.3258	0.4597
1	rs146222009	151633309	G	0.007732	0.01667	A	0.3258	0.4597
2	rs76774368	160604514	A	0.007732	0.01667	G	0.3258	0.4597
2	rs17827158	160676350	G	0.007732	0.01667	A	0.3258	0.4597
6	rs79274660	36982699	T	0.007732	0.01667	A	0.3258	0.4597
7	rs56100904	47927744	A	0.007732	0.01667	G	0.3258	0.4597
7	rs7785206	87021023	G	0.007732	0.01667	C	0.3258	0.4597
9	rs10739614	125330125	C	0.007732	0.01667	G	0.3258	0.4597
11	rs201855427	19256365	G	0.007732	0.01667	A	0.3258	0.4597
11	rs4935969	126296589	G	0.1649	0.1442	A	0.3258	1.173

12	rs149647403	14976571	A	0.007732	0.01667	C	0.3258	0.4597
12	rs148598781	50501810	G	0.007732	0.01667	A	0.3258	0.4597
12	rs17106884	70048829	G	0.007732	0.01667	A	0.3258	0.4597
12	rs117417637	101738482	A	0.007732	0.01667	G	0.3258	0.4597
15	rs76282169	41796599	A	0.007732	0.01667	G	0.3258	0.4597
16	rs75986066	89790048	G	0.007732	0.01667	C	0.3258	0.4597
17	rs201824198	44076928	G	0.007732	0.01667	A	0.3258	0.4597
17	rs143082560	79914822	A	0.007732	0.01667	G	0.3258	0.4597
18	rs76525142	77171164	A	0.007732	0.01667	G	0.3258	0.4597
1	rs4658359	89847372	C	0.2036	0.2292	A	0.326	0.86
1	rs4658360	89847411	G	0.2036	0.2292	A	0.326	0.86
6	rs2239705	31513402	A	0.1649	0.1883	G	0.3261	0.8513
7	rs73692834	47851623	A	0.1649	0.1883	G	0.3261	0.8513
7	rs2290386	47852837	A	0.1649	0.1883	G	0.3261	0.8513
19	rs9304655	46646841	G	0.3247	0.3533	A	0.3262	0.8802
1	rs184327209	46768970	A	0.01289	0.006667	C	0.3264	1.945
1	rs74518578	100155425	G	0.01289	0.006667	A	0.3264	1.945
1	rs141950039	228294567	A	0.01289	0.006667	G	0.3264	1.945
1	rs116617673	229771835	A	0.01289	0.006667	G	0.3264	1.945
2	rs76654748	1243512	G	0.01289	0.006667	C	0.3264	1.945
2	rs34476437	37599863	A	0.01289	0.006667	G	0.3264	1.945
2	rs138143232	71429659	T	0.01289	0.006667	A	0.3264	1.945
2	rs138159056	116594083	A	0.01289	0.006667	C	0.3264	1.945
2	rs201572288	178562138	T	0.01289	0.006667	A	0.3264	1.945
2	rs182035689	204111603	A	0.01289	0.006667	C	0.3264	1.945
2	rs188718238	234449382	A	0.01289	0.006667	G	0.3264	1.945
3	exm2050736	38598067	G	0.01289	0.006667	A	0.3264	1.945
3	rs200556220	38598067	G	0.01289	0.006667	A	0.3264	1.945
3	rs143469719	49836736	A	0.01289	0.006667	T	0.3264	1.945
4	rs734553	9923004	C	0.01289	0.006667	A	0.3264	1.945
4	rs6855911	9935910	G	0.01289	0.006667	A	0.3264	1.945
4	rs139258002	10446610	G	0.01289	0.006667	A	0.3264	1.945
4	rs11099273	135121066	A	0.01289	0.006667	C	0.3264	1.945
4	rs10009368	135121601	G	0.01289	0.006667	A	0.3264	1.945
4	rs199734613	186096969	G	0.01289	0.006667	C	0.3264	1.945
5	rs201995478	65350441	G	0.01289	0.006667	A	0.3264	1.945
5	rs3733712	140167220	C	0.01289	0.006667	A	0.3264	1.945
6	rs61732793	34826921	G	0.01289	0.006667	C	0.3264	1.945
6	rs143517301	35260345	A	0.01289	0.006667	G	0.3264	1.945
6	rs12207746	66042231	C	0.01289	0.006667	A	0.3264	1.945
6	rs3734305	138752241	A	0.01289	0.006667	G	0.3264	1.945
6	rs117412760	155761166	A	0.01289	0.006667	G	0.3264	1.945
7	rs186578702	20198546	A	0.01289	0.006667	T	0.3264	1.945

7	rs143559107	37903050	A	0.01289	0.006667	G	0.3264	1.945
7	rs80244589	50742212	A	0.01289	0.006667	G	0.3264	1.945
7	rs143387495	51096640	G	0.01289	0.006667	C	0.3264	1.945
7	rs182572283	128357021	A	0.01289	0.006667	G	0.3264	1.945
8	rs79324124	100865854	A	0.01289	0.006667	C	0.3264	1.945
8	rs188859975	118831994	A	0.01289	0.006667	G	0.3264	1.945
9	rs200111837	32784432	A	0.01289	0.006667	G	0.3264	1.945
9	rs200841827	100194348	G	0.01289	0.006667	A	0.3264	1.945
9	rs145638335	104238966	A	0.01289	0.006667	G	0.3264	1.945
11	rs12364724	57081146	C	0.01289	0.006667	G	0.3264	1.945
11	rs117253311	59573896	A	0.01289	0.006667	G	0.3264	1.945
11	rs148324299	93913355	G	0.01289	0.006667	A	0.3264	1.945
12	rs150532648	992587	G	0.01289	0.006667	A	0.3264	1.945
12	rs147834091	12982358	G	0.01289	0.006667	A	0.3264	1.945
13	rs77613105	27255207	A	0.01289	0.006667	G	0.3264	1.945
14	rs145600912	24680670	A	0.01289	0.006667	G	0.3264	1.945
14	rs61742122	91252650	A	0.01289	0.006667	G	0.3264	1.945
16	rs150122367	58743401	G	0.01289	0.006667	A	0.3264	1.945
18	rs34811964	61390515	G	0.01289	0.006667	A	0.3264	1.945
19	rs202232423	30165063	G	0.01289	0.006667	A	0.3264	1.945
19	rs139908769	48639372	A	0.01289	0.006667	G	0.3264	1.945
20	rs138744086	2778834	A	0.01289	0.006667	G	0.3264	1.945
20	rs192056427	30407433	A	0.01289	0.006667	C	0.3264	1.945
20	rs148496595	49508479	C	0.01289	0.006667	G	0.3264	1.945
22	rs79294358	26159232	A	0.01289	0.006667	G	0.3264	1.945
2	rs908545	113389232	A	0.2423	0.2175	G	0.3265	1.15
5	rs10513155	13788886	A	0.1649	0.1875	C	0.3265	0.856
7	rs12113288	75920815	A	0.3247	0.3525	G	0.3265	0.8834
11	rs150803613	65792968	A	0.05412	0.0423	G	0.3265	1.295
19	rs3745516	50926742	G	0.1649	0.1875	A	0.3265	0.856
20	rs4809330	62349586	G	0.3247	0.3525	A	0.3265	0.8834
4	rs74986565	187538271	T	0.01289	0.006678	A	0.3266	1.942
2	rs3731879	219920547	A	0.04897	0.06333	G	0.3269	0.7615
3	rs33915638	19574945	G	0.04897	0.06333	A	0.3269	0.7615
4	rs1280098	187538942	A	0.3686	0.3408	C	0.3269	1.129
17	rs116808485	18584142	G	0.01289	0.006689	A	0.3269	1.939
2	rs10172023	152476028	G	0.2552	0.2825	C	0.327	0.87
16	rs75357093	71220702	G	0.04897	0.06417	A	0.327	0.751
16	rs7205673	81232275	G	0.04897	0.06417	A	0.327	0.751
20	rs4339026	31671599	G	0.1366	0.1175	A	0.327	1.188
1	rs1801159	97981395	G	0.2552	0.2817	A	0.3271	0.8736
4	rs13142173	33953964	G	0.3273	0.3558	A	0.3271	0.8809
1	rs75981854	152081108	A	0.09021	0.07417	G	0.3272	1.238

2	rs6737027	29070805	A	0.09021	0.07417	G	0.3272	1.238
2	rs3211098	228194481	A	0.2938	0.2683	C	0.3272	1.134
4	rs78613619	89385130	C	0.09021	0.07417	G	0.3272	1.238
11	rs11234454	85579304	A	0.3711	0.3425	C	0.3272	1.133
1	rs78469326	47904000	A	0.201	0.1783	G	0.3273	1.159
1	rs6664618	66714584	C	0.134	0.1558	A	0.3273	0.8384
6	rs7742369	34165721	G	0.201	0.1783	A	0.3273	1.159
6	rs2854050	32185605	A	0.134	0.155	G	0.3275	0.8437
15	rs4779584	32994756	G	0.134	0.155	A	0.3275	0.8437
19	rs2277998	7831628	A	0.134	0.155	G	0.3275	0.8437
6	rs7773149	32706042	A	0.2423	0.2183	G	0.3276	1.145
6	rs9276431	32712247	A	0.2423	0.2183	G	0.3276	1.145
17	rs12944821	35956391	C	0.2423	0.2183	G	0.3276	1.145
3	rs16832203	161102719	A	0.1675	0.1458	G	0.3278	1.179
6	rs10046257	32854697	A	0.1675	0.1458	G	0.3278	1.179
7	rs12672119	30831181	A	0.1675	0.1458	G	0.3278	1.179
17	rs9908389	39137104	G	0.1675	0.1458	A	0.3278	1.179
17	rs721958	39137154	C	0.1675	0.1458	G	0.3278	1.179
17	rs1510068	39140221	A	0.1675	0.1458	G	0.3278	1.179
17	rs1510069	39140417	G	0.1675	0.1458	A	0.3278	1.179
17	rs75462245	76795805	C	0.1675	0.1458	G	0.3278	1.179
22	rs9466	38273749	G	0.1675	0.1458	A	0.3278	1.179
15	rs3809529	59499228	G	0.3737	0.345	A	0.3279	1.133
17	rs11546630	79639550	A	0.2964	0.2705	G	0.3279	1.136
1	rs17672135	240445596	G	0.1108	0.09417	A	0.3284	1.199
4	rs10011926	111026927	G	0.2577	0.285	A	0.3284	0.8711
5	rs4639193	79030045	A	0.2577	0.285	G	0.3284	0.8711
6	rs3132667	30191587	G	0.3737	0.3458	A	0.3284	1.129
10	rs34110351	50341954	G	0.2448	0.22	A	0.3284	1.15
10	rs11001034	76180335	G	0.2577	0.2842	A	0.3285	0.8747
1	rs76017814	55074683	A	0.05412	0.0425	C	0.3287	1.289
2	rs112962843	239049928	A	0.05412	0.0425	G	0.3287	1.289
5	rs705441	180278393	G	0.05412	0.0425	C	0.3287	1.289
6	rs7753116	10661262	G	0.05412	0.0425	A	0.3287	1.289
19	rs891088	7184762	G	0.1675	0.1908	A	0.3287	0.8533
22	rs148739949	45740462	C	0.05412	0.0425	G	0.3287	1.289
1	rs646776	109818530	G	0.07216	0.0575	A	0.3288	1.275
1	rs602633	109821511	A	0.07216	0.0575	C	0.3288	1.275
8	rs11544484	144406705	A	0.07216	0.0575	G	0.3288	1.275
10	rs2229603	129249662	G	0.07216	0.0575	A	0.3288	1.275
11	rs2726355	99455061	G	0.1675	0.1903	A	0.3288	0.8561
22	rs76578729	50684747	A	0.1675	0.1917	G	0.3289	0.8487
1	rs17107806	53532535	C	0.1675	0.1467	G	0.329	1.171

7	rs35495590	23728931	G	0.1675	0.1467	A	0.329	1.171
11	rs2245803	102495998	A	0.1675	0.1467	C	0.329	1.171
20	rs742710	5904028	A	0.1675	0.1467	G	0.329	1.171
22	rs4838865	50664612	A	0.1082	0.1283	G	0.329	0.8245
15	rs2277849	84639350	A	0.3763	0.3483	G	0.3291	1.129
5	rs415407	55414956	C	0.3325	0.36	A	0.3292	0.8855
6	rs129653	32931536	G	0.3325	0.36	A	0.3292	0.8855
12	rs11170389	53451849	C	0.1082	0.1275	G	0.3293	0.8307
13	rs9570576	62000820	A	0.2448	0.2208	G	0.3294	1.144
13	rs7331342	27188563	G	0.3351	0.3642	A	0.3295	0.8798
11	rs1498486	5461991	A	0.1392	0.1192	C	0.3297	1.195
13	rs2031640	23930055	A	0.1392	0.1192	T	0.3297	1.195
20	rs1047972	54961463	A	0.1392	0.1192	G	0.3297	1.195
6	rs79221470	121602785	T	0.2036	0.1808	A	0.3298	1.158
6	rs79040695	121625945	G	0.2036	0.1808	A	0.3298	1.158
7	rs3102817	68544052	A	0.3376	0.3666	C	0.3298	0.8807
1	rs2297950	203194186	A	0.2603	0.2867	G	0.3299	0.8757
16	rs11077227	7857969	A	0.2088	0.2342	G	0.3299	0.8629
16	rs188966111	88791441	A	0.01546	0.02517	G	0.3299	0.6084
20	rs680379	12969400	A	0.3789	0.3508	G	0.3299	1.129
3	rs710521	189645933	G	0.2088	0.2333	A	0.33	0.8669
4	rs11734944	75681217	A	0.3763	0.3492	G	0.33	1.125
6	rs34511704	26545696	A	0.01546	0.02508	G	0.33	0.6105
6	rs1141321	49412433	A	0.2088	0.2333	G	0.33	0.8669
11	rs13448	46695414	A	0.3763	0.3492	G	0.33	1.125
1	rs12130333	63191777	A	0.01546	0.025	G	0.3302	0.6126
1	rs16852767	163313568	C	0.01546	0.025	A	0.3302	0.6126
3	rs13060192	52378540	C	0.01546	0.025	G	0.3302	0.6126
3	rs61734640	52390789	A	0.01546	0.025	G	0.3302	0.6126
3	rs419752	52429665	A	0.01546	0.025	G	0.3302	0.6126
3	rs141784567	53263112	G	0.01546	0.025	A	0.3302	0.6126
4	rs117758185	88415038	G	0.01546	0.025	A	0.3302	0.6126
7	rs2252586	54978924	A	0.01546	0.025	G	0.3302	0.6126
8	rs114645573	25319653	A	0.01546	0.025	G	0.3302	0.6126
11	rs2305985	47772545	G	0.01546	0.025	A	0.3302	0.6126
15	rs34484871	63982745	A	0.01546	0.025	C	0.3302	0.6126
15	rs35978932	64025223	G	0.01546	0.025	A	0.3302	0.6126
17	rs77092590	6704182	A	0.01546	0.025	C	0.3302	0.6126
1	rs13306575	183532437	A	0.04124	0.03083	G	0.3303	1.352
2	rs117012322	233345474	A	0.03608	0.04917	C	0.3303	0.7239
5	rs78822243	71524160	C	0.03608	0.04917	G	0.3303	0.7239
9	rs7847413	107267352	G	0.3376	0.3658	A	0.3303	0.8836
9	rs57561786	130941134	A	0.04124	0.03083	G	0.3303	1.352

10	rs3750575	75138691	A	0.03608	0.04917	G	0.3303	0.7239
11	rs16933844	5664684	C	0.04124	0.03083	A	0.3303	1.352
12	rs17790558	6946718	A	0.04124	0.03083	G	0.3303	1.352
12	rs2270581	54911426	A	0.04124	0.03083	G	0.3303	1.352
12	rs7958163	114275242	C	0.3376	0.3658	A	0.3303	0.8836
14	rs17277025	20502044	A	0.04124	0.03083	C	0.3303	1.352
14	rs17121605	50074122	T	0.04124	0.03083	A	0.3303	1.352
14	rs7148147	50074520	A	0.04124	0.03083	G	0.3303	1.352
16	rs201914539	69364857	A	0.04124	0.03083	G	0.3303	1.352
19	rs35762773	38817359	A	0.03608	0.04917	G	0.3303	0.7239
20	rs310654	62172269	A	0.04124	0.03083	C	0.3303	1.352
20	rs310655	62172281	A	0.04124	0.03083	G	0.3303	1.352
20	rs404613	62173925	C	0.04124	0.03083	A	0.3303	1.352
10	rs4750511	14391273	G	0.3814	0.3531	A	0.3304	1.13
1	rs28365868	46049849	G	0.04124	0.03088	C	0.3308	1.35
3	rs6810039	33194990	C	0.1134	0.09583	A	0.3308	1.207
10	rs11004439	56423968	C	0.1134	0.09583	A	0.3308	1.207
13	rs9567280	44411432	G	0.1134	0.09583	A	0.3308	1.207
19	rs10411314	45728059	G	0.1134	0.09583	A	0.3308	1.207
4	rs4588	72618323	A	0.3015	0.2758	C	0.331	1.133
12	rs261902	32476727	A	0.1701	0.1483	G	0.3311	1.177
15	rs2304824	78466127	G	0.2629	0.29	A	0.3311	0.8732
22	rs4680	19951271	A	0.2629	0.29	G	0.3311	0.8732
6	rs1811197	31327660	A	0.03627	0.05	G	0.3312	0.7151
22	rs1001021	26403599	A	0.2474	0.2233	G	0.3312	1.143
4	rs2289433	47839929	G	0.384	0.3558	A	0.3313	1.129
18	rs3809977	56203898	A	0.2036	0.1817	C	0.3313	1.152
1	rs1009831	100771636	G	0.3814	0.3542	A	0.3314	1.125
1	rs3806366	163115321	G	0.3041	0.2775	A	0.3314	1.138
2	rs73082223	227973892	G	0.3041	0.2775	A	0.3314	1.138
6	rs362522	29545208	A	0.3041	0.2775	G	0.3314	1.138
6	rs2517449	30919701	G	0.3041	0.2775	A	0.3314	1.138
15	rs1026732	68095085	G	0.3041	0.2775	A	0.3314	1.138
1	rs3828054	151512895	G	0.03608	0.05	A	0.3316	0.7112
1	rs10919071	169099483	G	0.03608	0.05	A	0.3316	0.7112
2	rs77810069	29274947	C	0.03608	0.05	A	0.3316	0.7112
3	rs3762697	108754214	G	0.03608	0.05	A	0.3316	0.7112
9	rs61742100	130279279	G	0.03608	0.05	A	0.3316	0.7112
14	rs2228035	20871973	G	0.03608	0.05	A	0.3316	0.7112
5	rs6880381	157095798	G	0.3428	0.3717	A	0.3317	0.8818
1	rs1874045	34071525	G	0.3428	0.3708	A	0.3319	0.8849
5	rs2234942	86708529	A	0.01554	0.02583	C	0.3319	0.5954
1	rs2270976	18023690	A	0.06701	0.08333	G	0.332	0.7901

6	rs7744914	30348247	A	0.06701	0.08333	G	0.332	0.7901
6	rs3127573	160681393	G	0.06701	0.08333	A	0.332	0.7901
16	rs11989	4390358	A	0.06701	0.08333	C	0.332	0.7901
6	rs697739	16742033	G	0.25	0.225	A	0.3321	1.148
2	rs7592657	26755397	G	0.01546	0.02583	A	0.3322	0.5923
2	rs6543095	102412586	G	0.01546	0.02583	A	0.3322	0.5923
3	rs144659891	42733420	A	0.01546	0.02583	G	0.3322	0.5923
3	rs61729488	49067904	G	0.01546	0.02583	A	0.3322	0.5923
5	rs79392300	74998606	A	0.01546	0.02583	G	0.3322	0.5923
6	rs35331811	24848372	A	0.01546	0.02583	G	0.3322	0.5923
6	rs16894898	29395057	G	0.01546	0.02583	A	0.3322	0.5923
6	rs9469578	33706479	A	0.01546	0.02583	G	0.3322	0.5923
7	rs77743549	30791903	C	0.01546	0.02583	A	0.3322	0.5923
8	rs11985241	52321686	A	0.3041	0.2783	G	0.3322	1.133
9	rs190658661	20988418	A	0.01546	0.02583	G	0.3322	0.5923
9	rs138981588	35556365	A	0.01546	0.02583	G	0.3322	0.5923
9	rs76261117	112705060	A	0.01546	0.02583	T	0.3322	0.5923
11	rs55752830	124947396	G	0.1701	0.1492	A	0.3322	1.169
12	rs1153188	55098996	A	0.01546	0.02583	T	0.3322	0.5923
15	rs151252589	55632944	C	0.01546	0.02583	A	0.3322	0.5923
19	rs8102258	12186921	G	0.01546	0.02583	A	0.3322	0.5923
19	rs9419	39322087	C	0.3041	0.2783	A	0.3322	1.133
21	rs61741846	38538018	G	0.01546	0.02583	A	0.3322	0.5923
21	rs61736685	38568259	A	0.01546	0.02583	G	0.3322	0.5923
22	rs3747052	19118992	G	0.01546	0.02583	A	0.3322	0.5923
22	rs132653	36556823	C	0.01546	0.02583	A	0.3322	0.5923
22	rs117749664	45221398	A	0.01546	0.02583	G	0.3322	0.5923
1	rs75853003	3680333	A	0.07216	0.05833	G	0.3323	1.256
5	rs17418283	94154588	G	0.07216	0.05833	A	0.3323	1.256
5	rs7732671	149212243	C	0.07216	0.05833	G	0.3323	1.256
5	rs17572019	149212471	A	0.07216	0.05833	G	0.3323	1.256
12	rs79711087	80655832	A	0.07216	0.05833	T	0.3323	1.256
13	rs7322971	50127719	G	0.2062	0.1833	A	0.3323	1.157
16	rs10083789	23080634	A	0.07216	0.05833	C	0.3323	1.256
17	rs3744807	79890818	A	0.07216	0.05833	G	0.3323	1.256
11	rs10768895	42885153	G	0.3454	0.3742	A	0.3324	0.8824
11	rs6856	62458275	G	0.06701	0.08417	A	0.3324	0.7815
19	rs12983375	16831060	A	0.3454	0.3742	G	0.3324	0.8824
5	rs26279	80168937	G	0.2655	0.2917	A	0.3326	0.8777
8	rs1436013	58778118	C	0.2655	0.2917	A	0.3326	0.8777
1	rs12402711	41879081	A	0.3067	0.28	G	0.3327	1.138
1	rs7547370	191590362	G	0.3866	0.3592	A	0.3327	1.125
6	rs647744	145406142	C	0.3866	0.3592	A	0.3327	1.125

17	rs5020078	9697973	A	0.3067	0.2805	G	0.3329	1.135
17	rs7216445	35984410	A	0.3479	0.3767	G	0.3331	0.883
1	rs6702384	233023947	G	0.3918	0.3633	A	0.3333	1.129
4	rs6818012	65712811	G	0.3918	0.3633	A	0.3333	1.129
14	rs7144658	38061742	G	0.1108	0.1308	A	0.3333	0.828
18	rs7240405	47159090	A	0.1108	0.1308	G	0.3333	0.828
6	rs485502	32943345	A	0.3479	0.3758	G	0.3334	0.8862
6	rs1147857	148841752	A	0.3067	0.2808	G	0.3334	1.133
4	rs2290846	151199080	A	0.2139	0.2392	G	0.3336	0.8657
16	rs78180055	19548670	A	0.1134	0.09667	G	0.3336	1.195
19	rs4806163	36004106	A	0.1392	0.1208	G	0.3336	1.176
2	rs7600163	194888500	G	0.3505	0.3792	A	0.3338	0.8837
6	rs3734797	151672812	A	0.1108	0.1317	G	0.3338	0.822
16	rs193911	49520603	G	0.1108	0.1317	A	0.3338	0.822
2	rs934734	65595586	G	0.1727	0.1967	A	0.3339	0.8526
2	rs3213790	160294842	G	0.08763	0.1058	A	0.3339	0.8115
6	rs2274459	33762242	A	0.08763	0.1058	G	0.3339	0.8115
8	rs16872085	105957540	G	0.1727	0.1967	A	0.3339	0.8526
11	rs12574273	12315256	C	0.08763	0.1058	A	0.3339	0.8115
14	rs2268983	69408697	G	0.1392	0.1608	A	0.3339	0.8436
15	rs8024644	73052840	G	0.2139	0.2383	A	0.3339	0.8697
18	rs4799915	34928639	G	0.3093	0.2825	A	0.3339	1.137
4	rs356312	68219296	G	0.2526	0.2279	A	0.3342	1.145
9	rs2296949	134385436	A	0.09278	0.07667	G	0.3342	1.232
15	rs28384991	91295110	A	0.09278	0.07667	G	0.3342	1.232
17	rs1979370	72308319	G	0.1701	0.15	A	0.3342	1.161
16	rs4785763	90066936	A	0.1392	0.1617	C	0.3343	0.8384
9	rs7874348	127566378	A	0.08763	0.1067	G	0.3344	0.8044
6	rs86567	32976759	C	0.3531	0.3815	A	0.3345	0.885
12	rs7296248	103077198	A	0.3531	0.3817	G	0.3345	0.8843
17	rs7210616	72937605	G	0.3943	0.3667	A	0.3345	1.125
1	rs7546413	199274467	A	0.3093	0.2833	G	0.3346	1.133
2	rs2288563	179499530	G	0.116	0.09766	A	0.3346	1.212
17	rs12449858	76121318	A	0.3093	0.2833	G	0.3346	1.133
8	rs2645424	11684463	G	0.2088	0.1858	A	0.3347	1.156
9	rs368297	10063148	G	0.2088	0.1858	A	0.3347	1.156
2	rs2562832	179581835	A	0.2526	0.2283	C	0.3348	1.142
2	rs2286323	242036797	G	0.2526	0.2283	A	0.3348	1.142
9	rs1800435	116153891	C	0.0567	0.04417	G	0.335	1.301
7	rs713317	91054340	G	0.3995	0.3708	A	0.3352	1.129
12	rs7138792	127484300	G	0.3995	0.3708	A	0.3352	1.129
16	rs12447804	58075282	A	0.3557	0.3842	G	0.3352	0.8849
20	rs6077510	9288522	A	0.3995	0.3708	G	0.3352	1.129

7	rs2590599	118402674	C	0.2706	0.2967	A	0.3353	0.8796
2	rs17031056	43797619	A	0.2165	0.2417	G	0.3355	0.8671
3	rs1059502	148562310	A	0.2165	0.2417	G	0.3355	0.8671
6	rs3734316	37597129	G	0.2165	0.2417	A	0.3355	0.8671
6	rs3736352	54254589	C	0.2165	0.2417	A	0.3355	0.8671
13	rs1062087	75884216	G	0.2165	0.2417	A	0.3355	0.8671
3	rs6765213	163233510	G	0.3557	0.3833	A	0.3356	0.888
12	rs2228224	57865321	A	0.3557	0.3833	G	0.3356	0.888
2	rs2627037	179606538	A	0.2552	0.23	G	0.3357	1.147
6	rs3094694	30451904	G	0.2552	0.23	A	0.3357	1.147
6	rs2856717	32670308	A	0.2552	0.23	G	0.3357	1.147
11	rs1176744	113803028	C	0.2552	0.23	A	0.3357	1.147
2	rs3951216	160711045	C	0.3119	0.2858	G	0.3358	1.132
2	rs3732083	207041053	G	0.3119	0.2858	A	0.3358	1.132
4	rs2611818	56910860	G	0.2165	0.2408	A	0.3358	0.871
15	rs7170534	27812011	A	0.3119	0.2858	G	0.3358	1.132
16	rs8049603	23067260	A	0.2165	0.2408	C	0.3358	0.871
7	rs17854363	143053723	A	0.4021	0.3733	G	0.3359	1.129
6	rs2523685	31426256	G	0.2746	0.3017	C	0.336	0.8764
6	rs453658	29519848	G	0.2732	0.3	A	0.3362	0.8771
1	rs11265343	160144895	G	0.4021	0.3742	A	0.3363	1.125
10	rs2797501	5804531	G	0.1753	0.1983	A	0.3364	0.8589
10	rs11593576	81015896	A	0.1753	0.1983	G	0.3364	0.8589
14	rs4906593	37603194	A	0.05155	0.06667	G	0.3364	0.7609
4	rs11726022	188170467	A	0.2552	0.2308	G	0.3365	1.141
6	rs484730	10368708	G	0.4046	0.3758	A	0.3365	1.129
10	rs7076156	64415184	A	0.05155	0.06583	G	0.3365	0.7712
4	rs2903150	99027184	G	0.2732	0.2992	A	0.3366	0.8806
4	rs17558193	99049593	G	0.2732	0.2992	A	0.3366	0.8806
9	rs1123125	71893554	G	0.4046	0.3767	A	0.3368	1.125
12	rs708224	32436409	G	0.3608	0.3883	A	0.337	0.8892
18	rs2236700	13826391	G	0.3608	0.3883	C	0.337	0.8892
1	rs1543369	54907935	A	0.3634	0.3925	C	0.3371	0.8835
10	rs12355784	65121565	A	0.3634	0.3925	C	0.3371	0.8835
12	rs2731038	45417666	A	0.1418	0.1633	G	0.3372	0.8461
15	rs35114543	32347464	G	0.1727	0.1525	A	0.3372	1.16
4	rs1134648	107249244	G	0.2191	0.2442	C	0.3373	0.8684
11	rs2237878	2766282	A	0.2191	0.2442	G	0.3373	0.8684
20	rs11086243	46779235	A	0.2191	0.2442	G	0.3373	0.8684
12	rs140655819	335631	A	0.03109	0.02167	G	0.3374	1.449
12	rs12368048	52284668	A	0.1418	0.1642	C	0.3374	0.8409
12	rs7778	133498358	C	0.4072	0.3792	A	0.3374	1.125
2	rs3796110	107459974	A	0.1134	0.1333	G	0.3375	0.8314

4	rs4370153	122683007	G	0.1418	0.1233	A	0.3375	1.174
7	rs4717229	64451699	A	0.1134	0.1333	G	0.3375	0.8314
7	rs4618579	64451963	A	0.1134	0.1333	G	0.3375	0.8314
7	rs6460219	64453136	G	0.1134	0.1333	A	0.3375	0.8314
11	rs1453547	59189912	A	0.1418	0.1233	G	0.3375	1.174
11	rs11018874	89875437	A	0.2758	0.3025	G	0.3375	0.878
1	rs6678540	24421474	A	0.317	0.29	G	0.3376	1.136
5	rs12519269	26383363	A	0.317	0.29	G	0.3376	1.136
7	rs878644	13492428	A	0.3634	0.3908	G	0.3376	0.8897
1	rs4839047	116383333	A	0.366	0.395	C	0.3377	0.8841
2	rs2111349	83094402	G	0.2191	0.2433	A	0.3377	0.8723
3	rs3792594	33877626	A	0.366	0.395	G	0.3377	0.8841
4	rs7690538	75202049	A	0.366	0.395	G	0.3377	0.8841
7	rs2066853	17379110	A	0.366	0.395	G	0.3377	0.8841
12	rs77474006	248071	C	0.09278	0.07763	G	0.3377	1.215
15	rs25651	90335788	A	0.2191	0.2433	G	0.3377	0.8723
15	rs8192297	90344352	G	0.2191	0.2433	A	0.3377	0.8723
16	rs666647	56601722	A	0.2191	0.2433	G	0.3377	0.8723
20	rs80158178	44047974	A	0.04145	0.03167	G	0.3377	1.322
22	rs361557	18611223	A	0.366	0.395	G	0.3377	0.8841
1	rs34961909	23415438	A	0.02062	0.01333	G	0.3378	1.558
1	rs78637376	34003176	A	0.02062	0.01333	G	0.3378	1.558
1	rs12025510	145414790	C	0.02062	0.01333	G	0.3378	1.558
2	rs145912850	20234122	A	0.02062	0.01333	C	0.3378	1.558
2	rs199883809	119739738	A	0.02062	0.01333	G	0.3378	1.558
2	rs150990193	165552167	G	0.02062	0.01333	A	0.3378	1.558
3	rs2292661	122869110	A	0.02062	0.01333	G	0.3378	1.558
4	rs7683838	47525054	G	0.02062	0.01333	A	0.3378	1.558
5	rs35848863	176721119	A	0.02062	0.01333	G	0.3378	1.558
5	rs34165241	176721151	G	0.02062	0.01333	A	0.3378	1.558
5	rs78247455	176722005	A	0.02062	0.01333	G	0.3378	1.558
6	rs116888866	7585411	A	0.02062	0.01333	G	0.3378	1.558
7	rs919533	150873664	A	0.02062	0.01333	C	0.3378	1.558
8	rs8191666	11643693	A	0.02062	0.01333	C	0.3378	1.558
8	rs148527079	144940959	A	0.02062	0.01333	G	0.3378	1.558
9	rs142902473	74489863	A	0.02062	0.01333	G	0.3378	1.558
9	rs34115159	113563231	A	0.02062	0.01333	G	0.3378	1.558
10	rs6482626	27702725	G	0.02062	0.01333	A	0.3378	1.558
11	rs530965	79065101	A	0.366	0.3942	G	0.3378	0.8872
12	rs116851432	20774317	A	0.02062	0.01333	T	0.3378	1.558
12	rs412978	27641473	A	0.366	0.3942	G	0.3378	0.8872
12	rs149553969	57870740	A	0.02062	0.01333	C	0.3378	1.558
12	rs3802989	57872448	A	0.02062	0.01333	G	0.3378	1.558

14	rs138151018	45658156	A	0.02062	0.01333	G	0.3378	1.558
14	rs139074103	74411257	A	0.02062	0.01333	G	0.3378	1.558
14	rs1051340	92441066	A	0.366	0.3942	G	0.3378	0.8872
15	rs185361903	60297656	A	0.02062	0.01333	C	0.3378	1.558
15	rs115087152	86123049	A	0.02062	0.01333	C	0.3378	1.558
16	rs146749118	20975817	G	0.02062	0.01333	A	0.3378	1.558
17	rs117204968	48784934	G	0.02062	0.01333	A	0.3378	1.558
18	rs76572574	21353474	G	0.02062	0.01333	C	0.3378	1.558
20	rs55980737	50048687	A	0.02062	0.01333	G	0.3378	1.558
22	rs36070348	19808874	A	0.02062	0.01333	G	0.3378	1.558
5	rs4580814	1113244	A	0.2758	0.3017	C	0.3379	0.8815
5	rs6235	95728898	C	0.2758	0.3017	G	0.3379	0.8815
5	rs6234	95728974	C	0.2758	0.3017	G	0.3379	0.8815
1	rs12139550	12921132	G	0.07474	0.06	A	0.338	1.266
3	rs61742251	197403844	G	0.07474	0.06	A	0.338	1.266
7	rs62489646	150873754	A	0.07474	0.06	G	0.338	1.266
4	rs3733591	9922130	G	0.317	0.2908	A	0.3381	1.132
6	rs117692893	24596112	T	0.02577	0.03673	A	0.3381	0.6938
1	rs28568406	158687163	A	0.3686	0.3975	G	0.3382	0.8847
1	rs857703	158687224	A	0.3686	0.3975	G	0.3382	0.8847
11	rs7926767	123777536	G	0.2577	0.2333	A	0.3382	1.141
16	rs16940202	86014241	G	0.2577	0.2333	A	0.3382	1.141
18	rs9951150	52821124	G	0.3686	0.3975	A	0.3382	0.8847
1	rs12073549	17720545	A	0.02577	0.03667	G	0.3383	0.695
2	rs2290427	33505139	A	0.02577	0.0375	C	0.3383	0.679
3	rs78202770	124281844	A	0.02577	0.03667	C	0.3383	0.695
4	rs61745597	113544993	A	0.02577	0.03667	C	0.3383	0.695
10	rs16924863	24832283	C	0.02577	0.0375	G	0.3383	0.679
11	rs6578533	4825010	A	0.02577	0.0375	T	0.3383	0.679
11	rs7941731	4825225	G	0.02577	0.0375	A	0.3383	0.679
11	rs77057293	57177579	A	0.02577	0.03667	G	0.3383	0.695
14	rs17128116	55458040	G	0.02577	0.03667	A	0.3383	0.695
17	rs3744718	5462805	A	0.02577	0.0375	G	0.3383	0.679
17	rs16946335	60788696	A	0.02577	0.0375	G	0.3383	0.679
18	rs146950918	908295	A	0.02577	0.03667	G	0.3383	0.695
1	rs4908923	6614535	G	0.1753	0.1542	A	0.3384	1.166
3	rs34307082	193336676	A	0.03093	0.02167	G	0.3384	1.441
6	rs3096702	32192331	A	0.1753	0.1542	G	0.3384	1.166
9	rs2230287	124065224	A	0.1753	0.1542	G	0.3384	1.166
14	rs201799144	73958558	A	0.03093	0.02167	G	0.3384	1.441
16	rs3213511	112775	A	0.03093	0.02167	G	0.3384	1.441
16	rs79631587	89789073	A	0.03093	0.02167	G	0.3384	1.441
20	rs3748433	34090519	A	0.1753	0.1542	G	0.3384	1.166

19	rs1325158	55226402	G	0.4124	0.3842	A	0.3385	1.125
3	rs817515	117740707	G	0.4098	0.3825	A	0.3387	1.121
7	rs3735684	1024855	A	0.03093	0.0217	G	0.3387	1.439
7	rs80328282	21631070	A	0.03093	0.0217	G	0.3387	1.439
9	rs10968457	2838470	A	0.116	0.09917	G	0.3387	1.192
12	rs1129646	6946247	A	0.2784	0.3058	C	0.3387	0.8755
14	rs11625921	60923783	G	0.116	0.09917	A	0.3387	1.192
15	rs2959832	76402624	A	0.4098	0.3825	G	0.3387	1.121
17	rs9908032	20108184	C	0.116	0.09917	G	0.3387	1.192
18	rs11662608	62726012	C	0.4098	0.3825	A	0.3387	1.121
12	rs76543640	121174938	A	0.1778	0.2008	G	0.3388	0.8607
4	rs74511500	187524464	A	0.06959	0.08583	G	0.339	0.7966
5	rs272893	131663062	G	0.4149	0.3867	A	0.339	1.125
5	rs274546	131699867	G	0.4149	0.3867	A	0.339	1.125
16	rs1805011	27373872	C	0.06959	0.08583	A	0.339	0.7966
16	rs1805015	27374180	G	0.06959	0.08583	A	0.339	0.7966
9	exm2259179	131846957	G	0.2216	0.2467	A	0.3391	0.8697
9	rs11541836	131846957	G	0.2216	0.2467	A	0.3391	0.8697
1	rs10903122	25303576	A	0.3196	0.2933	G	0.3392	1.132
3	rs4684883	12861586	G	0.1443	0.125	A	0.3392	1.181
8	rs448705	17694702	G	0.1443	0.125	A	0.3392	1.181
11	rs11222114	130340962	G	0.3196	0.2933	A	0.3392	1.132
14	rs2295524	91671124	A	0.1443	0.125	G	0.3392	1.181
5	rs3213837	65321311	A	0.06959	0.08667	G	0.3393	0.7882
6	rs2596542	31366595	A	0.3756	0.4033	G	0.3393	0.8901
7	rs3750010	4901191	G	0.09021	0.1083	A	0.3393	0.8161
7	rs1524058	38136277	A	0.3737	0.4025	G	0.3393	0.8858
12	rs3761863	40758652	G	0.3737	0.4025	A	0.3393	0.8858
15	rs73403546	42442823	C	0.09021	0.1083	G	0.3393	0.8161
16	rs13333991	863498	C	0.06959	0.08667	G	0.3393	0.7882
19	rs404299	36349752	A	0.06959	0.08667	G	0.3393	0.7882
2	rs13427905	72073715	A	0.2139	0.1908	G	0.3394	1.154
19	rs4807145	1821637	A	0.2139	0.1908	G	0.3394	1.154
11	rs1453541	59225221	G	0.2577	0.2342	A	0.3395	1.136
6	rs9391682	30357291	C	0.4149	0.3875	A	0.3396	1.121
15	rs4777857	93925327	A	0.3737	0.4017	G	0.3396	0.8889
1	rs3748695	214819328	A	0.09021	0.1092	G	0.3397	0.8091
1	rs12058704	214819587	G	0.09021	0.1092	A	0.3397	0.8091
1	rs3748696	214819980	A	0.09021	0.1092	G	0.3397	0.8091
3	rs4682119	112546306	A	0.09021	0.1092	C	0.3397	0.8091
9	rs79340881	100092918	C	0.09021	0.1092	G	0.3397	0.8091
9	rs2306093	100133973	A	0.09021	0.1092	G	0.3397	0.8091
1	rs10798269	173309713	A	0.2809	0.3083	G	0.3399	0.8764

2	rs10208207	169074972	G	0.2603	0.2358	A	0.3399	1.14
4	rs930625	5163466	A	0.2809	0.3083	G	0.3399	0.8764
6	rs2516697	30232514	A	0.3763	0.405	G	0.3399	0.8863
6	rs2517552	31007590	G	0.3763	0.405	A	0.3399	0.8863
8	rs753778	142228909	A	0.4175	0.39	G	0.3401	1.121
12	rs4293214	23923719	G	0.4175	0.39	A	0.3401	1.121
8	rs10088262	124765702	A	0.3196	0.2942	G	0.3402	1.127
1	rs880633	203152801	G	0.3789	0.4075	A	0.3404	0.8869
6	rs4713438	31146846	A	0.1443	0.1658	G	0.3404	0.8485
7	rs4339527	68860360	G	0.3222	0.2958	A	0.3404	1.131
17	rs11080150	29629326	A	0.3789	0.4075	G	0.3404	0.8869
19	rs740495	1124835	G	0.3789	0.4075	A	0.3404	0.8869
19	rs2288481	49878196	A	0.3222	0.2958	G	0.3404	1.131
1	rs2805053	162781787	G	0.4227	0.3942	A	0.3405	1.125
3	rs11709625	66823157	A	0.1443	0.1667	C	0.3405	0.8434
11	rs3802842	111171709	C	0.4227	0.3942	A	0.3405	1.125
18	rs2889405	6854453	A	0.4227	0.3942	C	0.3405	1.125
1	rs3795595	202092360	G	0.2139	0.1917	A	0.3406	1.148
6	rs9357728	13436619	A	0.0567	0.045	G	0.3406	1.276
9	rs2296067	6984236	A	0.4201	0.3925	G	0.3406	1.121
9	rs872665	113261483	A	0.2139	0.1917	G	0.3406	1.148
11	rs34097396	4593411	A	0.0567	0.045	G	0.3406	1.276
20	rs11551768	17950545	A	0.0567	0.045	T	0.3406	1.276
1	rs4845490	152849299	A	0.3789	0.4067	G	0.3407	0.8899
5	rs832582	56177743	G	0.3789	0.4067	A	0.3407	0.8899
7	rs10499559	22109459	A	0.3789	0.4067	G	0.3407	0.8899
5	rs4895291	120254126	A	0.3222	0.2963	G	0.3408	1.129
1	rs2759281	204866365	A	0.2242	0.2492	G	0.3409	0.871
2	rs77063376	178936561	A	0.09536	0.07917	G	0.3409	1.226
2	rs12993533	193203397	G	0.09536	0.07917	A	0.3409	1.226
6	rs2071554	32784676	A	0.09536	0.07917	G	0.3409	1.226
7	rs1767742	86625854	C	0.2242	0.25	A	0.3409	0.8671
9	rs77621374	88937854	A	0.3814	0.41	T	0.3409	0.8874
14	rs3742935	105405599	G	0.3814	0.41	C	0.3409	0.8874
14	rs2819419	105406238	C	0.3814	0.41	A	0.3409	0.8874
14	rs4465542	105407798	G	0.3814	0.41	A	0.3409	0.8874
21	rs226794	28302355	G	0.4227	0.395	A	0.341	1.121
2	rs13014858	115192890	G	0.1804	0.2042	A	0.3411	0.858
1	rs17356233	146740502	G	0.07474	0.06083	C	0.3412	1.247
1	rs1055935	152975941	G	0.3238	0.2983	C	0.3412	1.126
2	rs11541017	71351487	A	0.07474	0.06083	G	0.3412	1.247
5	rs6867983	55854153	A	0.04381	0.0325	G	0.3412	1.364
5	rs17376456	93557702	G	0.07474	0.06083	A	0.3412	1.247

8	rs61733696	17612518	G	0.04381	0.0325	A	0.3412	1.364
11	rs11024769	18735465	A	0.04381	0.0325	G	0.3412	1.364
18	rs74414891	6890538	A	0.04381	0.0325	C	0.3412	1.364
19	rs17851959	6187680	A	0.04381	0.0325	G	0.3412	1.364
19	rs17851960	6187686	A	0.04381	0.0325	G	0.3412	1.364
20	rs7260921	32251396	G	0.04381	0.0325	A	0.3412	1.364
5	rs10074258	106982560	A	0.3814	0.4092	G	0.3413	0.8905
11	rs7936512	5776287	G	0.1443	0.1258	A	0.3413	1.172
11	rs7394584	5776470	A	0.1443	0.1258	C	0.3413	1.172
11	rs586088	74203308	T	0.3222	0.2967	A	0.3413	1.127
17	rs4968104	649505	T	0.1443	0.1258	A	0.3413	1.172
1	rs4446959	159357684	G	0.3247	0.2983	A	0.3414	1.131
1	rs12059562	206665052	A	0.384	0.4125	G	0.3414	0.8879
8	rs444772	55539057	A	0.384	0.4125	G	0.3414	0.8879
11	rs9651740	77746118	A	0.3247	0.2983	G	0.3414	1.131
5	rs1805074	78324352	G	0.1778	0.1567	A	0.3415	1.164
5	rs1805073	78326750	G	0.1778	0.1567	C	0.3415	1.164
6	rs1335035	102408680	A	0.4253	0.3975	G	0.3415	1.121
11	rs2512988	86414027	G	0.4278	0.3992	A	0.3415	1.126
4	rs7666785	144060464	A	0.116	0.1358	G	0.3416	0.8347
11	rs10896818	58491921	A	0.2629	0.2383	T	0.3416	1.14
6	rs387608	31941557	A	0.2165	0.1933	G	0.3417	1.153
6	rs389512	31947594	C	0.2165	0.1933	G	0.3417	1.153
12	rs2634041	53045626	A	0.2165	0.1933	G	0.3417	1.153
3	rs12638212	108159977	G	0.384	0.4117	A	0.3418	0.891
4	rs4860406	62136915	A	0.384	0.4117	G	0.3418	0.891
10	rs7900909	66448472	G	0.384	0.4117	A	0.3418	0.891
17	rs1057040	76799795	A	0.384	0.4117	G	0.3418	0.891
2	rs7565124	20261685	A	0.116	0.1367	G	0.3419	0.8288
8	rs33977775	53852871	T	0.116	0.1367	A	0.3419	0.8288
9	rs7032296	86713990	G	0.3866	0.415	A	0.3419	0.8884
6	rs61730668	29455465	A	0.1178	0.1383	G	0.342	0.8318
7	rs6962772	99081730	G	0.2861	0.3133	A	0.3422	0.8782
2	rs12999397	13652731	G	0.3892	0.4175	A	0.3424	0.8889
6	rs6930033	29323905	C	0.3866	0.4142	A	0.3424	0.8915
6	rs1044303	131148737	G	0.3866	0.4142	A	0.3424	0.8915
6	rs2147457	168810725	G	0.3892	0.4175	A	0.3424	0.8889
8	rs35497596	21956108	G	0.433	0.4042	A	0.3424	1.126
11	rs2298553	60265002	A	0.3892	0.4175	G	0.3424	0.8889
15	rs4984241	63623463	G	0.3892	0.4175	A	0.3424	0.8889
19	rs35612307	14875388	A	0.228	0.2538	G	0.3424	0.8684
19	rs10412613	52712828	A	0.3273	0.3008	G	0.3425	1.131
5	rs13157656	40964852	C	0.2268	0.2517	A	0.3426	0.8722

11	rs537916	125617606	A	0.2268	0.2525	G	0.3426	0.8684
22	rs2073201	25294369	A	0.2268	0.2525	G	0.3426	0.8684
1	rs476141	244176424	A	0.2629	0.2392	C	0.3427	1.135
2	rs6756597	237149941	G	0.433	0.405	A	0.3427	1.122
8	rs7824557	11104111	G	0.433	0.405	A	0.3427	1.122
8	rs1056148	144358381	G	0.2655	0.2404	A	0.3427	1.142
9	rs7856369	22768513	G	0.433	0.405	A	0.3427	1.122
9	rs657454	32534851	A	0.2629	0.2392	G	0.3427	1.135
10	rs10826172	60118681	G	0.3918	0.4207	A	0.3427	0.8869
1	rs77751812	16069071	A	0.2165	0.1942	G	0.3428	1.147
1	rs117105149	167095468	C	0.03866	0.05167	A	0.3428	0.7381
16	rs61745756	839600	A	0	0.004181	G	0.3428	0
1	rs2279681	201861016	C	0.3892	0.4167	G	0.3429	0.892
14	rs7158437	35134328	A	0.3918	0.42	G	0.3429	0.8894
2	rs1800440	38298139	G	0	0.004174	A	0.343	0
12	rs186646374	77202823	G	0	0.004174	A	0.343	0
17	rs187972762	14248937	G	0	0.004174	A	0.343	0
16	rs58468753	66527192	A	0.1778	0.1575	G	0.3431	1.157
1	rs149124387	12025561	A	0	0.004167	G	0.3432	0
1	rs145819522	17672566	C	0	0.004167	G	0.3432	0
1	rs116209107	17913964	G	0	0.004167	A	0.3432	0
1	rs28374389	19175846	G	0	0.004167	A	0.3432	0
1	rs17163868	26648381	A	0	0.004167	G	0.3432	0
1	rs200785272	44482799	A	0	0.004167	G	0.3432	0
1	rs189650822	44685747	G	0	0.004167	C	0.3432	0
1	rs200400903	51753878	A	0	0.004167	G	0.3432	0
1	rs77267196	54332025	A	0	0.004167	G	0.3432	0
1	rs201826477	63064458	G	0	0.004167	A	0.3432	0
1	rs62642564	94466624	A	0	0.004167	G	0.3432	0
1	rs199768177	108185345	G	0	0.004167	A	0.3432	0
1	rs138466584	109394412	A	0	0.004167	G	0.3432	0
1	rs17229382	117568217	A	0	0.004167	G	0.3432	0
1	rs192289549	119575758	A	0	0.004167	G	0.3432	0
1	rs181428774	120307179	A	0	0.004167	C	0.3432	0
1	rs112651497	144916644	A	0	0.004167	G	0.3432	0
1	rs142294146	153317814	A	0	0.004167	G	0.3432	0
1	rs180708431	156206248	A	0	0.004167	G	0.3432	0
1	rs142700943	156496297	G	0	0.004167	A	0.3432	0
1	rs149238952	156536300	A	0	0.004167	G	0.3432	0
1	rs188054107	156708315	A	0	0.004167	G	0.3432	0
1	rs150875349	167024212	A	0	0.004167	C	0.3432	0
1	rs200822402	169347635	A	0	0.004167	G	0.3432	0
1	rs114531453	173551091	G	0	0.004167	A	0.3432	0

1	rs12568823	183502945	A	0	0.004167	C	0.3432	0
1	rs201243236	185130019	G	0	0.004167	A	0.3432	0
1	rs56219555	203472822	A	0	0.004167	G	0.3432	0
1	rs199535586	207195632	G	0	0.004167	A	0.3432	0
1	rs75868118	207196598	A	0	0.004167	G	0.3432	0
1	rs10888255	248004687	C	0	0.004167	G	0.3432	0
1	rs10888256	248004775	G	0	0.004167	A	0.3432	0
1	rs4607924	248004848	G	0	0.004167	C	0.3432	0
1	rs10888257	248004877	G	0	0.004167	A	0.3432	0
1	rs139029646	249150577	G	0	0.004167	A	0.3432	0
2	rs28913014	1520672	A	0	0.004167	G	0.3432	0
2	rs201258434	27529103	A	0	0.004167	G	0.3432	0
2	rs184249075	29294264	A	0	0.004167	G	0.3432	0
2	rs199935818	70387882	A	0	0.004167	G	0.3432	0
2	rs183869493	97019131	G	0	0.004167	A	0.3432	0
2	rs12989701	127887985	A	0	0.004167	C	0.3432	0
2	rs199849486	135745024	A	0	0.004167	G	0.3432	0
2	rs184341388	168101906	A	0	0.004167	C	0.3432	0
2	rs2335704	205864832	C	0	0.004167	A	0.3432	0
2	rs184388902	224824363	G	0	0.004167	A	0.3432	0
3	rs117441424	9969881	A	0	0.004167	G	0.3432	0
3	rs150753845	15062438	G	0	0.004167	A	0.3432	0
3	rs117564813	42956454	C	0	0.004167	A	0.3432	0
3	rs147919436	48616675	A	0	0.004167	G	0.3432	0
3	rs145068043	48623145	A	0	0.004167	G	0.3432	0
3	rs187641562	48638451	G	0	0.004167	A	0.3432	0
3	rs117433616	48789660	G	0	0.004167	A	0.3432	0
3	rs149647295	50231244	A	0	0.004167	G	0.3432	0
3	rs2231928	73111973	G	0.3943	0.4233	A	0.3432	0.8869
3	rs201292837	77671576	G	0	0.004167	A	0.3432	0
3	rs139467007	110852851	G	0	0.004167	A	0.3432	0
3	rs76884941	111201785	C	0	0.004167	A	0.3432	0
3	rs56238310	111233239	A	0	0.004167	C	0.3432	0
3	rs199861750	119176890	G	0	0.004167	A	0.3432	0
3	rs12637267	129120534	A	0	0.004167	G	0.3432	0
3	rs115756407	140407014	G	0	0.004167	A	0.3432	0
3	rs201758122	182789102	A	0	0.004167	G	0.3432	0
3	rs76438938	186461524	A	0	0.004167	G	0.3432	0
3	rs139813132	196434643	G	0	0.004167	C	0.3432	0
3	rs147719730	196435539	G	0	0.004167	A	0.3432	0
4	rs139066088	1729959	A	0	0.004167	G	0.3432	0
4	rs363125	3189547	A	0	0.004167	C	0.3432	0
4	rs147134533	39064241	A	0	0.004167	G	0.3432	0

4	rs138364911	39245910	G	0	0.004167	A	0.3432	0
4	rs199867239	39515754	G	0	0.004167	A	0.3432	0
4	rs180820099	72635115	A	0	0.004167	G	0.3432	0
4	rs186418626	85654590	G	0	0.004167	A	0.3432	0
4	rs187207325	91389444	G	0	0.004167	A	0.3432	0
4	rs2276973	104577382	G	0	0.004167	A	0.3432	0
4	rs61757395	106552167	A	0	0.004167	C	0.3432	0
4	rs142944337	108866639	C	0	0.004167	G	0.3432	0
4	rs139260545	126411082	G	0	0.004167	A	0.3432	0
4	rs199652168	187584654	G	0	0.004167	A	0.3432	0
5	rs201280643	10262696	G	0	0.004167	C	0.3432	0
5	rs115004914	13900321	A	0	0.004167	C	0.3432	0
5	rs199637361	38493801	A	0	0.004167	G	0.3432	0
5	rs192602074	54327231	A	0	0.004167	G	0.3432	0
5	rs150017263	82834437	A	0	0.004167	G	0.3432	0
5	rs200847506	131303680	A	0	0.004167	G	0.3432	0
5	rs147426159	131877734	C	0	0.004167	A	0.3432	0
5	rs148555720	135390452	A	0	0.004167	G	0.3432	0
5	rs144347026	139189261	G	0	0.004167	A	0.3432	0
5	rs190820379	139921839	A	0	0.004167	G	0.3432	0
5	rs202102338	150885550	A	0	0.004167	G	0.3432	0
5	rs188526149	160061418	G	0	0.004167	A	0.3432	0
5	rs145873210	169469096	A	0	0.004167	G	0.3432	0
6	rs10485172	4069133	G	0	0.004167	A	0.3432	0
6	rs3177253	4133855	A	0	0.004167	G	0.3432	0
6	rs11755724	7118990	G	0	0.004167	A	0.3432	0
6	rs4585612	7153152	A	0	0.004167	G	0.3432	0
6	rs3198005	32813214	A	0	0.004167	G	0.3432	0
6	rs149016705	38820545	A	0	0.004167	G	0.3432	0
6	rs191386841	109700851	G	0	0.004167	C	0.3432	0
6	rs17847050	132181533	G	0	0.004167	A	0.3432	0
6	rs200437948	142691874	T	0	0.004167	A	0.3432	0
6	rs200700475	155761270	G	0	0.004167	A	0.3432	0
6	rs147847428	158454680	A	0	0.004167	G	0.3432	0
6	rs56092260	161807897	A	0	0.004167	G	0.3432	0
7	rs10256972	1039003	A	0.3943	0.4233	C	0.3432	0.8869
7	rs3213641	25266423	A	0	0.004167	G	0.3432	0
7	rs149042169	33407445	A	0	0.004167	G	0.3432	0
7	rs145850475	45011343	A	0	0.004167	T	0.3432	0
7	rs200906390	47408900	A	0	0.004167	G	0.3432	0
7	rs17471501	101200765	A	0	0.004167	G	0.3432	0
7	rs145765083	101916711	A	0	0.004167	G	0.3432	0
7	rs114385222	102524110	A	0	0.004167	G	0.3432	0

7	rs145309509	121716610	A	0	0.004167	G	0.3432	0
7	rs200274325	134719061	A	0	0.004167	G	0.3432	0
7	rs148450860	134943185	A	0	0.004167	G	0.3432	0
7	rs141884347	149520553	A	0	0.004167	G	0.3432	0
7	rs75056573	150034065	A	0	0.004167	G	0.3432	0
7	rs201579252	150707257	A	0	0.004167	G	0.3432	0
8	rs149935963	1998956	A	0	0.004167	G	0.3432	0
8	rs147933019	17570762	A	0	0.004167	C	0.3432	0
8	rs151100700	17942229	A	0	0.004167	G	0.3432	0
8	rs202137427	39694686	A	0	0.004167	G	0.3432	0
8	rs1818	56686224	A	0	0.004167	G	0.3432	0
8	rs10100659	56708701	G	0	0.004167	A	0.3432	0
8	rs10109493	56711713	G	0	0.004167	A	0.3432	0
8	rs7823773	56723557	C	0	0.004167	A	0.3432	0
8	rs62525422	86021994	A	0	0.004167	G	0.3432	0
8	rs62620995	105367264	A	0	0.004167	G	0.3432	0
8	rs11573906	119945260	A	0	0.004167	G	0.3432	0
8	rs114665145	121220474	A	0	0.004167	G	0.3432	0
8	rs143408022	125989375	A	0	0.004167	G	0.3432	0
8	rs145933616	143832508	A	0	0.004167	G	0.3432	0
8	rs202175941	144993053	A	0	0.004167	G	0.3432	0
9	rs75679360	35563361	A	0	0.004167	G	0.3432	0
9	rs138364612	85640699	A	0	0.004167	G	0.3432	0
9	rs201603947	116049104	A	0	0.004167	G	0.3432	0
9	rs17819466	117819436	A	0	0.004167	G	0.3432	0
9	rs141510612	124094754	A	0	0.004167	G	0.3432	0
9	rs146953746	127287147	G	0	0.004167	A	0.3432	0
9	rs118142639	132636945	A	0	0.004167	G	0.3432	0
9	rs61751543	139401233	A	0	0.004167	G	0.3432	0
9	rs968733	139981627	A	0	0.004167	G	0.3432	0
10	rs34821949	5498724	A	0	0.004167	G	0.3432	0
10	rs199951450	29821560	C	0	0.004167	A	0.3432	0
10	rs139186815	62648240	A	0	0.004167	G	0.3432	0
10	rs188784527	100503697	A	0	0.004167	T	0.3432	0
10	rs148004684	101639978	A	0	0.004167	G	0.3432	0
10	rs202011870	105371375	C	0	0.004167	A	0.3432	0
10	rs149089631	123724863	A	0	0.004167	G	0.3432	0
11	rs201592902	198547	A	0	0.004167	G	0.3432	0
11	rs202188897	1021245	A	0	0.004167	G	0.3432	0
11	rs201891529	1086422	A	0	0.004167	G	0.3432	0
11	rs78500033	16847973	A	0	0.004167	G	0.3432	0
11	rs143204089	18313529	A	0	0.004167	G	0.3432	0
11	exm2249736	18568542	T	0	0.004167	A	0.3432	0

11	rs34242612	18568542	T	0	0.004167	A	0.3432	0
11	rs61752314	18600283	G	0	0.004167	A	0.3432	0
11	rs199902250	47201041	A	0	0.004167	G	0.3432	0
11	rs201490859	62445871	A	0	0.004167	G	0.3432	0
11	rs76718173	62911224	A	0	0.004167	G	0.3432	0
11	rs35386215	65650978	C	0	0.004167	G	0.3432	0
11	rs148948642	66299152	G	0	0.004167	A	0.3432	0
11	rs200325719	78380625	A	0	0.004167	G	0.3432	0
11	rs1047964	117156893	C	0	0.004167	G	0.3432	0
11	rs140133210	118982297	A	0	0.004167	G	0.3432	0
11	rs200700939	119058356	A	0	0.004167	C	0.3432	0
11	rs140506777	122827205	T	0	0.004167	A	0.3432	0
11	rs139133124	122944456	A	0	0.004167	G	0.3432	0
11	rs61747005	124412838	A	0	0.004167	G	0.3432	0
11	rs117548122	124508502	A	0	0.004167	G	0.3432	0
12	rs61731141	6464944	A	0	0.004167	G	0.3432	0
12	rs74060111	8325226	C	0	0.004167	A	0.3432	0
12	rs140433964	13061554	A	0	0.004167	G	0.3432	0
12	rs140585847	42778747	A	0	0.004167	G	0.3432	0
12	rs145979946	44148486	A	0	0.004167	G	0.3432	0
12	rs200408293	53715187	C	0	0.004167	G	0.3432	0
12	rs7978197	69326547	A	0	0.004167	G	0.3432	0
12	rs139349075	77427693	G	0	0.004167	A	0.3432	0
12	rs150841760	102141003	A	0	0.004167	G	0.3432	0
12	rs202114208	119591376	C	0	0.004167	A	0.3432	0
12	rs76176317	124258725	A	0	0.004167	G	0.3432	0
13	rs7990167	26789411	T	0	0.004167	A	0.3432	0
13	rs145975088	43528113	C	0	0.004167	A	0.3432	0
14	rs79804050	53331183	A	0	0.004167	G	0.3432	0
14	rs146668840	76088479	A	0	0.004167	G	0.3432	0
14	rs201330148	96904193	A	0	0.004167	G	0.3432	0
15	rs144282125	34102850	G	0	0.004167	A	0.3432	0
15	rs146553005	40914216	G	0	0.004167	A	0.3432	0
15	rs117842035	42978426	A	0	0.004167	T	0.3432	0
15	rs184505235	56258734	A	0	0.004167	G	0.3432	0
15	rs151097746	57731443	G	0	0.004167	C	0.3432	0
15	rs190718928	70960331	A	0	0.004167	G	0.3432	0
15	rs35796837	75043593	A	0	0.004167	G	0.3432	0
15	rs181914721	84488608	A	0	0.004167	G	0.3432	0
15	rs201680379	85659278	G	0	0.004167	A	0.3432	0
15	rs141435604	90137671	G	0	0.004167	A	0.3432	0
15	rs7171194	90784250	A	0.2655	0.2408	G	0.3432	1.139
15	rs143774359	98513207	G	0	0.004167	C	0.3432	0

15	rs200815939	101169796	G	0	0.004167	A	0.3432	0
16	rs141366917	560734	A	0	0.004167	G	0.3432	0
16	rs185197463	3334030	A	0	0.004167	C	0.3432	0
16	rs114065872	14340885	A	0	0.004167	C	0.3432	0
16	rs150366923	27221796	A	0	0.004167	G	0.3432	0
16	rs200337123	30939801	A	0	0.004167	G	0.3432	0
16	rs201449358	57921800	A	0	0.004167	G	0.3432	0
16	rs200632318	69602397	A	0	0.004167	C	0.3432	0
16	rs200017298	70986520	A	0	0.004167	C	0.3432	0
16	rs202143365	88705543	A	0	0.004167	G	0.3432	0
16	rs868044	90102833	A	0	0.004167	G	0.3432	0
17	rs149901678	3638200	G	0	0.004167	A	0.3432	0
17	rs187609896	4936443	C	0	0.004167	G	0.3432	0
17	rs200392931	6941768	A	0	0.004167	C	0.3432	0
17	rs199946589	7707605	A	0	0.004167	G	0.3432	0
17	rs202198533	10436864	A	0	0.004167	G	0.3432	0
17	rs200517965	17698166	A	0	0.004167	T	0.3432	0
17	rs201112044	21217491	A	0	0.004167	C	0.3432	0
17	rs117752697	27028457	A	0	0.004167	G	0.3432	0
17	rs141608844	33464838	A	0	0.004167	G	0.3432	0
17	rs3803860	33679616	G	0	0.004167	A	0.3432	0
17	rs140842796	34074883	A	0	0.004167	G	0.3432	0
17	rs115602554	42225482	G	0	0.004167	A	0.3432	0
17	rs141898450	48606118	A	0	0.004167	G	0.3432	0
17	rs140093645	59668229	C	0	0.004167	G	0.3432	0
17	rs200376492	67121109	G	0	0.004167	A	0.3432	0
17	rs59690629	76503781	A	0	0.004167	G	0.3432	0
17	rs2589156	78820374	A	0	0.004167	G	0.3432	0
18	rs143946098	21743216	G	0	0.004167	A	0.3432	0
18	rs146549538	44560693	G	0	0.004167	A	0.3432	0
19	rs28626600	830820	A	0	0.004167	G	0.3432	0
19	rs192694824	1056431	A	0	0.004167	G	0.3432	0
19	rs144505131	2877719	A	0	0.004167	G	0.3432	0
19	rs200535011	2933929	C	0	0.004167	A	0.3432	0
19	rs200047802	4446617	A	0	0.004167	G	0.3432	0
19	rs200541526	6710651	A	0	0.004167	G	0.3432	0
19	rs187868330	6755219	A	0	0.004167	G	0.3432	0
19	rs146702874	7509332	A	0	0.004167	G	0.3432	0
19	rs201631789	7998749	C	0	0.004167	A	0.3432	0
19	rs200758108	10403815	A	0	0.004167	G	0.3432	0
19	rs200101413	12979885	A	0	0.004167	G	0.3432	0
19	rs150479603	18727858	A	0	0.004167	G	0.3432	0
19	rs144897155	19037115	A	0	0.004167	G	0.3432	0

19	rs1781872	21477403	C	0	0.004167	A	0.3432	0
19	rs201012525	32902198	G	0	0.004167	A	0.3432	0
19	rs199915749	43999748	A	0	0.004167	G	0.3432	0
19	rs200691935	46299329	T	0	0.004167	A	0.3432	0
19	rs199797698	51633253	G	0	0.004167	A	0.3432	0
19	rs75142551	52220394	A	0	0.004167	G	0.3432	0
19	rs117157582	52938429	G	0	0.004167	C	0.3432	0
19	rs144870963	53057722	G	0	0.004167	A	0.3432	0
19	rs200957236	58565052	A	0	0.004167	G	0.3432	0
20	rs8120693	14307024	A	0	0.004167	G	0.3432	0
20	rs151139830	61444918	G	0	0.004167	A	0.3432	0
20	rs146347952	62839237	A	0	0.004167	G	0.3432	0
21	rs115649519	26979768	A	0	0.004167	C	0.3432	0
21	rs11553519	47404302	A	0	0.004167	G	0.3432	0
22	rs146819542	19839517	A	0	0.004167	G	0.3432	0
22	rs116710238	19963271	C	0	0.004167	A	0.3432	0
22	rs141896929	24891453	A	0	0.004167	G	0.3432	0
22	rs201392026	31011643	A	0	0.004167	G	0.3432	0
22	rs61739193	35463225	A	0	0.004167	G	0.3432	0
22	rs140970741	38061816	A	0	0.004167	G	0.3432	0
22	rs200068984	38483189	G	0	0.004167	A	0.3432	0
22	rs200777988	46751377	C	0	0.004167	G	0.3432	0
6	rs3736781	26505362	G	0.2887	0.3158	A	0.3433	0.879
2	rs1052500	120060082	G	0.3943	0.4225	A	0.3434	0.8899
9	rs1929425	12367988	A	0.3918	0.4192	G	0.3434	0.8925
10	rs11000889	75902177	A	0.2887	0.315	G	0.3434	0.8824
12	rs527118	371410	G	0.2887	0.315	A	0.3434	0.8824
12	rs7315917	3250797	G	0.2887	0.315	A	0.3434	0.8824
5	rs2329435	40998851	G	0.4381	0.41	A	0.3435	1.122
5	rs2279587	52214581	A	0.183	0.2067	G	0.3435	0.8598
5	rs12520591	52229745	C	0.183	0.2067	A	0.3435	0.8598
11	rs73481226	60184277	C	0.183	0.2067	A	0.3435	0.8598
16	rs72628271	81193321	A	0.183	0.2067	G	0.3435	0.8598
6	rs6903448	76173832	A	0.183	0.2058	G	0.3436	0.8642
2	rs34715748	68882661	G	0.09536	0.08	A	0.3437	1.212
18	rs77169818	74980601	A	0.09536	0.08	T	0.3437	1.212
1	rs1934709	102968036	G	0.03866	0.0525	A	0.3438	0.7258
5	rs201489289	177035576	A	0	0.004167	G	0.3438	0
6	rs9378305	1703290	A	0.3969	0.425	G	0.3438	0.8904
7	rs869127	101091470	A	0.03866	0.0525	G	0.3438	0.7258
7	rs149217752	142920042	A	0	0.004167	G	0.3438	0
17	rs2301739	5308377	G	0.3969	0.425	A	0.3438	0.8904
2	rs918949	218674697	A	0.4407	0.4125	G	0.3439	1.122

3	rs10933973	108634973	A	0.2191	0.1958	C	0.3439	1.152
7	rs2722372	37890267	A	0.2191	0.1958	G	0.3439	1.152
6	rs909728	29719561	A	0.4381	0.4108	G	0.3442	1.118
6	rs9351290	92576162	A	0.2294	0.255	G	0.3443	0.8696
10	rs117193245	38404188	C	0.09278	0.1112	G	0.3443	0.8174
1	rs507181	74939570	C	0.2912	0.3183	A	0.3444	0.8799
3	rs4679323	126737268	A	0.4021	0.4307	C	0.3444	0.8887
6	rs78862138	33382818	A	0	0.004167	G	0.3444	0
8	rs1351424	136176400	C	0.2294	0.2538	A	0.3444	0.8754
11	rs2250417	112085316	G	0.1804	0.1592	A	0.3444	1.163
16	rs3751801	86566039	A	0.1804	0.1592	G	0.3444	1.163
2	rs12712508	36771309	A	0.4021	0.4308	G	0.3445	0.8883
3	rs2654694	192312270	A	0.4021	0.4308	G	0.3445	0.8883
15	rs1038094	74012437	A	0.4021	0.4308	G	0.3445	0.8883
1	rs678962	172189889	C	0.3325	0.3058	A	0.3446	1.13
7	rs10274334	47925331	C	0.2912	0.3175	G	0.3446	0.8833
11	rs141638912	129993558	A	0	0.004181	G	0.3446	0
4	rs3911610	180472174	G	0.4433	0.4158	A	0.3448	1.119
6	rs9379479	23191842	A	0.4046	0.4333	G	0.3448	0.8888
10	rs77473776	27702624	A	0.3342	0.3082	C	0.3448	1.127
14	rs11626651	102183359	A	0.4433	0.4158	G	0.3448	1.119
15	rs1871017	27890491	C	0.268	0.2433	A	0.3448	1.139
15	rs79501973	90167658	A	0.09278	0.1117	G	0.3448	0.8136
3	rs6795735	64705365	G	0.2191	0.1967	A	0.345	1.146
9	rs12156449	83686072	A	0.4485	0.42	G	0.345	1.123
19	rs61748441	48342689	A	0	0.004167	G	0.345	0
2	rs1044710	211297355	G	0.4459	0.4183	A	0.3451	1.119
5	rs244669	133416258	G	0.4459	0.4183	A	0.3451	1.119
5	rs35883638	170095415	A	0.4046	0.4325	G	0.3451	0.8918
9	rs2986671	113192655	C	0.4459	0.4183	A	0.3451	1.119
18	rs2542151	12779947	C	0.1469	0.1283	A	0.3451	1.17
19	rs12608849	8160334	A	0.4459	0.4183	G	0.3451	1.119
11	rs7946749	44373635	G	0.05412	0.06917	A	0.3454	0.7701
16	rs1803382	88783100	G	0.05412	0.06917	A	0.3454	0.7701
17	rs4239020	80176641	G	0.05412	0.06917	A	0.3454	0.7701
21	rs7510381	46852824	A	0.4098	0.4382	G	0.3455	0.8901
9	rs7034200	4289050	A	0.4072	0.435	C	0.3456	0.8923
15	rs3803403	85383145	G	0.1186	0.1383	C	0.3456	0.8378
6	rs2804918	13584583	G	0.3351	0.3083	A	0.3457	1.13
6	rs12193504	31820805	A	0.07216	0.08848	G	0.3457	0.8013
6	rs1512116	80598166	A	0.2938	0.32	G	0.3457	0.8841
13	rs9550987	24167505	A	0.2938	0.32	T	0.3457	0.8841
18	rs2010834	44560875	C	0.3351	0.3083	A	0.3457	1.13

19	rs2228615	10403368	A	0.3351	0.3083	G	0.3457	1.13
19	rs10500264	33750314	A	0.05412	0.06833	G	0.3457	0.7802
1	rs190864802	20067396	A	0	0.005	G	0.3458	0
1	rs201994378	32834055	A	0	0.005008	G	0.3458	0
1	rs184222741	43748701	A	0	0.005	G	0.3458	0
1	rs17849102	44063613	A	0	0.005	G	0.3458	0
1	rs41269097	45475886	A	0	0.005	G	0.3458	0
1	rs200038150	45476368	C	0	0.005	G	0.3458	0
1	rs140035988	64104470	A	0	0.005	G	0.3458	0
1	rs188557928	100964928	C	0	0.005	G	0.3458	0
1	rs142603483	117568514	A	0	0.005	G	0.3458	0
1	rs140487163	144871837	A	0	0.005	C	0.3458	0
1	rs79228057	153283134	A	0	0.005	G	0.3458	0
1	rs199792419	156882331	C	0	0.005	A	0.3458	0
1	rs200687438	179965908	T	0	0.005	A	0.3458	0
2	rs202096955	10829982	C	0	0.005	G	0.3458	0
2	rs150030853	15319147	A	0	0.005	G	0.3458	0
2	rs201420303	27851963	A	0	0.005	G	0.3458	0
2	rs117051248	71209218	A	0	0.005	G	0.3458	0
2	rs192499639	73677983	A	0	0.005	C	0.3458	0
2	rs77000353	74077556	A	0	0.005	C	0.3458	0
2	rs202055795	74717206	A	0	0.005	G	0.3458	0
2	rs138317624	148716374	G	0	0.005	A	0.3458	0
2	rs147318949	150432319	C	0	0.005	A	0.3458	0
2	rs143822500	170113674	G	0	0.005	A	0.3458	0
2	rs200214298	211444453	A	0	0.005	G	0.3458	0
2	rs200851225	228881697	C	0	0.005	G	0.3458	0
2	rs149783296	240954276	G	0	0.005	A	0.3458	0
2	rs117389170	241465683	A	0	0.005	G	0.3458	0
3	rs141816989	47453372	A	0	0.005	G	0.3458	0
3	rs112544934	66457866	A	0	0.005	G	0.3458	0
3	rs145806877	66502031	A	0	0.005	G	0.3458	0
3	rs28362487	99567870	C	0	0.005	G	0.3458	0
3	rs2931761	112190137	C	0	0.005	A	0.3458	0
3	rs79791762	137849888	A	0	0.005	G	0.3458	0
3	rs3796241	150690476	A	0	0.005	T	0.3458	0
3	rs200013345	167750553	A	0	0.005	G	0.3458	0
3	rs193059575	186024707	A	0	0.005	C	0.3458	0
4	rs3755954	994452	A	0.1186	0.1392	G	0.3458	0.832
4	rs62272670	3475166	A	0	0.005	G	0.3458	0
4	rs28544093	104182954	G	0	0.005	A	0.3458	0
4	rs4444841	127412444	A	0	0.005	G	0.3458	0
4	rs116782984	141832504	G	0	0.005	A	0.3458	0

4	rs192063486	152054218	T	0	0.005	A	0.3458	0
4	exm2067772	154625259	G	0	0.005	A	0.3458	0
4	rs144038898	154625259	G	0	0.005	A	0.3458	0
4	rs114032464	166981211	A	0	0.005	G	0.3458	0
4	rs74560263	186366181	A	0	0.005	G	0.3458	0
5	rs140524514	55204174	A	0	0.005	G	0.3458	0
5	rs200584417	72173169	A	0	0.005	G	0.3458	0
5	rs28942073	74014629	A	0	0.005	G	0.3458	0
5	rs190580387	79029369	A	0	0.005	G	0.3458	0
5	rs138316719	79375779	A	0	0.005	C	0.3458	0
5	rs141467927	79734726	G	0	0.005	A	0.3458	0
5	rs148338096	89814764	A	0	0.005	G	0.3458	0
5	rs17544552	89979518	A	0	0.005	G	0.3458	0
5	rs200017020	131924538	G	0	0.005	A	0.3458	0
5	rs33972313	138715502	A	0	0.005	G	0.3458	0
5	rs186370335	140953385	A	0	0.005	G	0.3458	0
5	rs200109165	145537108	A	0	0.005	G	0.3458	0
5	rs144941254	148996132	A	0	0.005	G	0.3458	0
5	rs180898690	156184733	C	0	0.005	G	0.3458	0
5	rs146949812	157073657	G	0	0.005	A	0.3458	0
6	rs200564301	26056085	C	0	0.005	A	0.3458	0
6	rs58554303	28499567	G	0	0.005	A	0.3458	0
6	rs3130253	29634012	A	0	0.005	G	0.3458	0
6	rs9261387	30061361	A	0	0.005	G	0.3458	0
6	rs2395029	31431780	C	0	0.005	A	0.3458	0
6	rs28399976	31725978	G	0	0.005	C	0.3458	0
6	rs201153896	34985311	A	0	0.005	C	0.3458	0
6	rs185218985	43748545	A	0	0.005	G	0.3458	0
6	rs146320190	44103073	A	0	0.005	G	0.3458	0
6	rs147656266	105771566	A	0	0.005	G	0.3458	0
6	rs77221231	152697940	C	0	0.005	A	0.3458	0
6	rs146406000	155579035	A	0	0.005	G	0.3458	0
6	rs142642959	170155481	A	0	0.005	G	0.3458	0
7	rs113614660	2629671	A	0	0.005	G	0.3458	0
7	rs200692522	6193698	G	0	0.005	A	0.3458	0
7	rs201087247	6449550	A	0	0.005	G	0.3458	0
7	rs192409840	23004127	A	0	0.005	G	0.3458	0
7	rs190612258	27211786	A	0	0.005	G	0.3458	0
7	rs201645679	30868352	A	0	0.005	G	0.3458	0
7	rs17131915	47933494	A	0	0.005	G	0.3458	0
7	rs149702058	149557928	G	0	0.005	A	0.3458	0
8	rs150631675	21996040	A	0	0.005	G	0.3458	0
8	rs118019452	27779717	A	0	0.005	C	0.3458	0

8	rs193281437	39626981	G	0	0.005	A	0.3458	0
8	rs199908344	48771430	A	0	0.005	G	0.3458	0
8	rs2303460	53073986	A	0	0.005	G	0.3458	0
8	rs141145196	144398191	A	0	0.005	G	0.3458	0
8	rs200374705	144998234	A	0	0.005	G	0.3458	0
9	rs78641305	5300439	C	0	0.005	G	0.3458	0
9	rs138812386	34249882	A	0	0.005	G	0.3458	0
9	rs149771118	34618720	G	0	0.005	C	0.3458	0
9	rs202213081	73152208	A	0	0.005	C	0.3458	0
9	rs188339598	84231031	A	0	0.005	G	0.3458	0
9	rs200805854	94486667	A	0	0.005	C	0.3458	0
9	rs41276200	96436037	A	0	0.005	G	0.3458	0
9	rs116686341	103046760	G	0	0.005	A	0.3458	0
9	rs201629680	131709203	G	0	0.005	A	0.3458	0
9	rs11243731	135202993	A	0	0.005	G	0.3458	0
9	rs137877337	139298607	A	0	0.005	G	0.3458	0
9	rs181795958	140002963	A	0	0.005	G	0.3458	0
10	rs191787640	16990479	A	0	0.005	G	0.3458	0
10	rs142995301	18292207	A	0	0.005	G	0.3458	0
10	rs149602031	44052728	G	0	0.005	A	0.3458	0
10	rs149298698	95113644	A	0	0.005	T	0.3458	0
10	rs57321698	124793921	G	0	0.005	A	0.3458	0
11	rs146230036	4622323	G	0	0.005	A	0.3458	0
11	rs190765812	4674016	C	0	0.005	A	0.3458	0
11	rs186983128	7071027	A	0	0.005	G	0.3458	0
11	rs115188826	7818137	A	0	0.005051	G	0.3458	0
11	rs190566144	19167805	A	0	0.005	G	0.3458	0
11	rs140934116	34163339	A	0	0.005	G	0.3458	0
11	rs181230846	57581818	G	0	0.005	A	0.3458	0
11	rs2276427	62010944	G	0	0.005	A	0.3458	0
11	rs202173749	107524930	A	0	0.005	C	0.3458	0
11	rs190953225	110451725	A	0	0.005	G	0.3458	0
11	rs12797755	120809460	C	0	0.005	A	0.3458	0
11	rs185989409	130784838	A	0	0.005	G	0.3458	0
12	rs187022621	644394	A	0	0.005	G	0.3458	0
12	rs181667117	4461781	A	0	0.005	G	0.3458	0
12	rs34196984	7092665	G	0	0.005	A	0.3458	0
12	rs146924988	21652503	G	0	0.005	A	0.3458	0
12	rs149097385	31820650	G	0	0.005	A	0.3458	0
12	rs187461001	47472156	G	0	0.005	C	0.3458	0
12	rs202167830	49421648	A	0	0.005	G	0.3458	0
12	rs201244168	52565270	A	0	0.005	G	0.3458	0
12	rs184808867	53164891	C	0	0.005	A	0.3458	0

12	rs180748572	58125149	A	0	0.005	G	0.3458	0
12	rs59453864	122691541	A	0	0.005	G	0.3458	0
12	rs192638383	124409745	A	0	0.005	G	0.3458	0
12	rs200875323	133353269	A	0	0.005	G	0.3458	0
14	rs140761202	50262640	A	0	0.005	G	0.3458	0
14	rs201677383	71052475	A	0	0.005	G	0.3458	0
14	rs200945035	94844968	G	0	0.005	A	0.3458	0
14	rs200831020	96797857	A	0	0.005	G	0.3458	0
14	rs3742937	105353305	A	0	0.005	C	0.3458	0
15	rs147204964	38591665	A	0	0.005	G	0.3458	0
15	rs201079226	42193088	A	0	0.005	T	0.3458	0
15	rs149919213	45059853	A	0	0.005	G	0.3458	0
15	rs180757384	62316036	A	0	0.005	G	0.3458	0
15	rs140723056	63128147	G	0	0.005	C	0.3458	0
16	rs140898293	11824509	G	0	0.005	A	0.3458	0
16	rs202239509	68026874	A	0	0.005	G	0.3458	0
16	rs193102233	68265182	C	0	0.005	A	0.3458	0
16	rs140304498	72173288	A	0	0.005	G	0.3458	0
17	rs200231675	1944781	G	0	0.005	C	0.3458	0
17	rs148199774	3301421	A	0	0.005	G	0.3458	0
17	rs200883298	4348459	G	0	0.005	C	0.3458	0
17	rs140082251	4647532	A	0	0.005	G	0.3458	0
17	rs201354502	7758532	C	0	0.005	A	0.3458	0
17	rs140873918	10398298	A	0	0.005	C	0.3458	0
17	rs181887565	27442042	C	0	0.005	G	0.3458	0
17	rs12943866	33772689	G	0	0.005008	A	0.3458	0
17	rs200203531	45773594	A	0	0.005	G	0.3458	0
17	rs201739796	75488774	G	0	0.005	C	0.3458	0
18	rs17664267	40808798	A	0	0.005	C	0.3458	0
18	rs12457996	40872273	G	0	0.005	A	0.3458	0
18	rs189077012	48439270	A	0	0.005	G	0.3458	0
19	rs202131921	3525882	A	0	0.005	G	0.3458	0
19	rs200193168	4298169	A	0	0.005	G	0.3458	0
19	rs113286748	7743869	A	0	0.005	G	0.3458	0
19	rs188490696	10818225	G	0	0.005	A	0.3458	0
19	rs140692918	15052581	G	0	0.005	A	0.3458	0
19	rs199782025	15651439	A	0	0.005	G	0.3458	0
19	rs200765474	48953925	A	0	0.005	G	0.3458	0
19	rs149419497	49949912	A	0	0.005	G	0.3458	0
19	rs57256087	50384677	A	0	0.005	G	0.3458	0
19	rs8108074	52130925	A	0	0.005	G	0.3458	0
19	rs181252364	55870804	A	0	0.005	G	0.3458	0
19	rs75471108	56467001	A	0	0.005	G	0.3458	0

20	rs199706843	3204031	A	0	0.005	G	0.3458	0
20	rs200985651	3846479	A	0	0.005	G	0.3458	0
20	rs144300885	49203789	A	0	0.005	G	0.3458	0
20	rs190219512	58560057	A	0	0.005	G	0.3458	0
20	rs199926959	62197178	A	0	0.005017	C	0.3458	0
20	rs200473388	62200017	A	0	0.005	G	0.3458	0
21	rs16987932	32567583	C	0	0.005	G	0.3458	0
21	rs145947009	45833864	A	0	0.005	G	0.3458	0
22	rs77130927	29121019	A	0	0.005	G	0.3458	0
22	rs112311672	50686403	A	0	0.005	G	0.3458	0
10	rs6586145	90433345	T	0.07216	0.08833	A	0.3459	0.8027
14	rs2763529	103654939	A	0.07216	0.08833	C	0.3459	0.8027
15	rs4362360	86940622	A	0.07216	0.08833	G	0.3459	0.8027
16	rs1424087	74264261	G	0.4562	0.4275	A	0.3459	1.123
17	rs200409284	61987638	G	0	0.005025	C	0.3459	0
19	rs148314941	58850058	A	0	0.005034	G	0.3459	0
1	rs2305549	75055419	G	0.232	0.2567	A	0.346	0.8747
3	rs74737650	155200562	A	0.07216	0.08917	G	0.346	0.7945
6	rs13118	31827286	A	0.07216	0.08917	T	0.346	0.7945
13	rs1868463	39266484	A	0.232	0.2567	G	0.346	0.8747
14	rs17090921	94933695	A	0.4098	0.4375	G	0.346	0.8927
9	rs357564	98209594	G	0.4562	0.4282	A	0.3462	1.12
1	rs10921318	193104827	A	0.4149	0.4433	G	0.3463	0.8906
17	rs4968281	44950122	G	0.4562	0.4283	A	0.3463	1.12
7	rs822552	148650634	C	0.2706	0.2458	G	0.3464	1.138
4	rs7685686	3207142	G	0.4613	0.4325	A	0.3465	1.124
10	rs2295774	102265847	C	0.1495	0.1708	A	0.3465	0.8531
10	rs3793706	102269085	A	0.1495	0.1708	C	0.3465	0.8531
10	rs3763695	102269206	G	0.1495	0.1708	A	0.3465	0.8531
11	rs10458896	28057957	G	0.4613	0.4325	A	0.3465	1.124
3	rs9825091	56796026	G	0.4175	0.4458	A	0.3466	0.891
6	rs166327	30002881	A	0.4588	0.4308	G	0.3466	1.12
10	rs2296610	21157621	A	0.1495	0.1717	C	0.3466	0.8481
3	rs2278911	108298260	G	0.4149	0.4425	A	0.3467	0.8936
9	rs2297089	74305098	A	0.07732	0.0625	G	0.3467	1.257
11	rs4561213	24678819	C	0.4149	0.4425	A	0.3467	0.8936
15	rs7257	80191343	A	0.3376	0.3108	G	0.3467	1.13
2	rs4369876	167129256	A	0.05928	0.04667	C	0.3468	1.287
6	rs2251765	30330341	A	0.05928	0.04667	C	0.3468	1.287
6	rs2158285	30331027	G	0.05928	0.04667	A	0.3468	1.287
6	rs2516683	30348090	G	0.05928	0.04667	A	0.3468	1.287
6	rs2187978	30362961	A	0.05928	0.04667	G	0.3468	1.287
8	rs13248774	53291161	A	0.2964	0.3225	G	0.3468	0.8849

8	rs7815788	57179020	A	0.05928	0.04667	G	0.3468	1.287
10	rs78881625	21455373	G	0.2964	0.3225	A	0.3468	0.8849
13	rs116981543	25487096	G	0.05928	0.04667	A	0.3468	1.287
13	rs41286126	76395464	A	0.05928	0.04667	G	0.3468	1.287
16	rs33932559	89986025	G	0.05928	0.04667	A	0.3468	1.287
17	rs4280292	13704274	G	0.4639	0.435	A	0.3468	1.124
19	rs75029862	12763007	A	0.05928	0.04667	G	0.3468	1.287
2	rs75663378	219900320	C	0.04381	0.03333	A	0.3469	1.329
4	rs78000298	123664254	A	0.04381	0.03333	G	0.3469	1.329
12	rs3803002	123444422	A	0.04381	0.03333	C	0.3469	1.329
14	rs3742728	77951124	G	0.04381	0.03333	A	0.3469	1.329
18	exm1390145	59942691	G	0.04381	0.03333	A	0.3469	1.329
18	rs9948159	59942691	G	0.04381	0.03333	A	0.3469	1.329
21	rs9978445	25356564	A	0.04381	0.03333	C	0.3469	1.329
4	rs4449432	131167056	G	0.4201	0.4483	A	0.347	0.8914
2	rs6435238	205274728	C	0.4639	0.4358	A	0.3471	1.12
2	rs16824283	228855866	G	0.2216	0.1992	C	0.3471	1.145
6	rs1727638	72139572	G	0.4175	0.445	A	0.3471	0.894
15	rs4924618	42371752	A	0.4175	0.445	T	0.3471	0.894
6	rs434102	42666061	A	0.183	0.1617	G	0.3473	1.161
20	rs236152	5903848	C	0.4227	0.4508	G	0.3473	0.8918
22	rs4819925	17446991	G	0.183	0.1617	A	0.3473	1.161
6	rs6913283	64613740	G	0.4253	0.4542	A	0.3474	0.8893
12	rs12831974	72724034	G	0.4665	0.4383	A	0.3474	1.12
15	rs10851797	69381742	G	0.4253	0.4542	A	0.3474	0.8893
6	rs41560824	31379043	G	0.01302	0.007513	A	0.3475	1.743
7	rs17143187	20666235	G	0.4201	0.4475	C	0.3475	0.8944
9	rs11243419	134422439	A	0.4201	0.4475	G	0.3475	0.8944
16	rs173539	56988044	A	0.2345	0.26	G	0.3475	0.8721
18	rs9949868	29727583	G	0.5052	0.4765	A	0.3475	1.122
2	rs6728818	238244963	A	0.4253	0.4533	G	0.3476	0.8922
4	rs345513	79240063	G	0.4253	0.4533	A	0.3476	0.8922
4	rs10519613	142654084	A	0.4378	0.4667	C	0.3476	0.8901
6	rs4551215	33025700	A	0.4691	0.4408	G	0.3476	1.121
6	rs1062481	33037611	A	0.4691	0.4408	G	0.3476	1.121
9	rs10812610	27533984	C	0.299	0.3258	A	0.3476	0.8824
14	rs3742717	93118668	A	0.4691	0.4408	G	0.3476	1.121
15	rs12702	44093927	G	0.299	0.3258	A	0.3476	0.8824
8	rs7842575	142461694	G	0.3402	0.3133	A	0.3477	1.13
19	rs11668530	52001485	A	0.2345	0.2592	G	0.3477	0.8758
6	rs3130190	33061690	G	0.4716	0.4433	A	0.3478	1.121
6	rs2395314	33062673	A	0.4716	0.4433	C	0.3478	1.121
10	rs10882993	99504630	C	0.4639	0.4367	A	0.3478	1.116

2	rs10199656	148138082	A	0.4304	0.4592	G	0.3479	0.8901
3	rs818817	38350543	A	0.2732	0.2483	G	0.3479	1.138
5	rs1159045	145953954	A	0.4278	0.4558	G	0.3479	0.8926
6	rs4498353	52272574	A	0.299	0.325	G	0.3479	0.8857
7	rs315860	33926351	A	0.4304	0.4592	G	0.3479	0.8901
9	rs3816094	124222033	G	0.4304	0.4592	A	0.3479	0.8901
16	rs344365	1974795	A	0.4278	0.4558	G	0.3479	0.8926
11	rs560096	68678962	A	0.433	0.4617	G	0.3481	0.8904
2	rs2253680	170403030	G	0.4768	0.4483	A	0.3482	1.121
6	rs3117226	33057659	A	0.4691	0.4417	G	0.3482	1.117
7	rs1624161	78562474	A	0.4691	0.4417	G	0.3482	1.117
14	rs4899947	88769467	A	0.4691	0.4417	G	0.3482	1.117
1	rs3819946	75175886	A	0.1881	0.2108	G	0.3483	0.8674
8	rs10112981	114965243	G	0.3402	0.3142	A	0.3483	1.126
17	rs11079346	56383744	G	0.1881	0.2108	A	0.3483	0.8674
2	rs4416261	69569930	A	0.4716	0.4442	G	0.3484	1.117
2	rs3739124	131127651	G	0.03093	0.0225	A	0.3484	1.387
2	rs186211637	219887984	A	0.03093	0.0225	G	0.3484	1.387
6	rs3128917	33059996	C	0.4716	0.4442	A	0.3484	1.117
6	rs9398652	122146034	A	0.4716	0.4442	C	0.3484	1.117
8	rs150115838	99101813	G	0.03093	0.0225	A	0.3484	1.387
8	rs11992195	103573042	A	0.03093	0.0225	G	0.3484	1.387
8	rs11995900	103573086	G	0.03093	0.0225	A	0.3484	1.387
9	rs76430730	138712896	A	0.03093	0.0225	G	0.3484	1.387
10	rs184626206	76995102	A	0.03093	0.0225	C	0.3484	1.387
10	rs4751185	132056415	A	0.1211	0.1042	G	0.3484	1.185
15	rs147962000	44858159	G	0.03093	0.0225	A	0.3484	1.387
15	rs16956375	72190417	C	0.03093	0.0225	G	0.3484	1.387
19	rs80283711	33321587	A	0.03093	0.0225	G	0.3484	1.387
22	rs2071883	44286950	G	0.4356	0.4642	A	0.3484	0.8908
2	rs732609	1499927	C	0.4742	0.4467	A	0.3485	1.117
1	rs10915891	224048645	G	0.482	0.4533	A	0.3486	1.122
8	rs17058207	27767231	G	0.3015	0.3283	C	0.3486	0.8832
9	rs12338899	107457725	A	0.482	0.4533	G	0.3486	1.122
5	rs4703849	81106956	A	0.4768	0.4492	G	0.3487	1.118
16	rs61744916	67578257	A	0.01295	0.007513	G	0.3488	1.734
12	rs7136702	50880216	G	0.4433	0.4717	A	0.349	0.892
1	rs3748816	2526746	G	0.4897	0.4608	A	0.3491	1.123
6	rs4896495	140197079	G	0.4845	0.4567	A	0.3491	1.118
7	rs6459717	156732717	G	0.4897	0.4608	A	0.3491	1.123
8	rs2294066	2046700	A	0.2371	0.2625	G	0.3491	0.8732
4	rs6814310	140455528	C	0.4459	0.4742	A	0.3492	0.8923
10	rs2147896	100148176	A	0.4459	0.4742	G	0.3492	0.8923

10	rs4409764	101284237	A	0.4407	0.4683	C	0.3492	0.8946
10	rs10883365	101287764	G	0.4407	0.4683	A	0.3492	0.8946
10	rs6584283	101290301	A	0.4407	0.4683	G	0.3492	0.8946
10	rs11190140	101291593	A	0.4407	0.4683	G	0.3492	0.8946
11	rs11030016	27487992	A	0.4459	0.4742	G	0.3492	0.8923
17	rs892657	52381288	A	0.4407	0.4683	G	0.3492	0.8946
4	rs6826460	149605653	G	0.2371	0.2617	A	0.3493	0.877
8	rs2882217	61955772	A	0.4871	0.4592	G	0.3493	1.119
9	rs10757278	22124477	G	0.4923	0.4633	A	0.3493	1.123
9	rs473325	133394635	A	0.4923	0.4633	G	0.3493	1.123
15	rs351220	74489153	G	0.4923	0.4633	A	0.3493	1.123
22	rs5770917	51017353	G	0.2242	0.2017	A	0.3493	1.144
11	rs7112513	117037361	A	0.451	0.48	G	0.3494	0.8901
11	rs4936367	117042377	G	0.451	0.48	A	0.3494	0.8901
12	rs1990313	4736086	G	0.4485	0.4767	A	0.3494	0.8927
15	rs2290344	55619796	A	0.4897	0.4617	G	0.3494	1.119
1	rs2072748	26097731	A	0.4536	0.4825	G	0.3495	0.8904
2	rs6717575	64371569	G	0.1211	0.1408	A	0.3495	0.8408
5	rs7727656	25760679	C	0.4536	0.4825	A	0.3495	0.8904
14	rs8572	23236524	G	0.1521	0.1742	A	0.3495	0.8503
19	rs2074071	58003580	A	0.2758	0.2508	G	0.3495	1.137
20	rs874468	18968197	A	0.4923	0.4642	C	0.3495	1.119
22	rs7575	19423250	A	0.1211	0.1408	G	0.3495	0.8408
1	rs142180334	32635611	A	0.01289	0.0075	G	0.3496	1.728
1	rs17119280	59799161	A	0.01289	0.0075	G	0.3496	1.728
1	rs181187833	151774034	C	0.01289	0.0075	G	0.3496	1.728
1	rs140303758	156626789	G	0.01289	0.0075	A	0.3496	1.728
2	rs58453991	26533808	A	0.01289	0.0075	G	0.3496	1.728
2	rs150597431	42578409	G	0.01289	0.0075	A	0.3496	1.728
2	rs77260934	61575266	G	0.01289	0.0075	A	0.3496	1.728
2	rs1517182	72735313	A	0.01289	0.0075	C	0.3496	1.728
2	rs56349597	135738850	A	0.01289	0.0075	G	0.3496	1.728
2	rs2271703	233839369	G	0.01289	0.0075	A	0.3496	1.728
4	rs16890979	9922167	A	0.01289	0.0075	G	0.3496	1.728
4	rs75386686	122068316	C	0.01289	0.0075	A	0.3496	1.728
4	rs10000692	122078317	G	0.01289	0.0075	A	0.3496	1.728
5	rs13360277	176370402	G	0.01289	0.0075	A	0.3496	1.728
6	rs2517614	30163955	A	0.1211	0.1417	G	0.3496	0.8351
6	rs2517611	30169327	G	0.1211	0.1417	A	0.3496	0.8351
6	rs199819170	43017147	G	0.01289	0.0075	A	0.3496	1.728
6	rs144106341	51656139	C	0.01289	0.0075	A	0.3496	1.728
6	rs185347145	80197173	A	0.01289	0.0075	G	0.3496	1.728
6	rs201458750	116442098	C	0.01289	0.0075	G	0.3496	1.728

7	rs218965	11022564	G	0.4562	0.485	A	0.3496	0.8908
7	rs150722853	94897945	A	0.01289	0.0075	G	0.3496	1.728
7	rs144415744	100479385	A	0.01289	0.0075	C	0.3496	1.728
8	rs11546144	145161823	G	0.01289	0.0075	A	0.3496	1.728
9	rs34832656	732536	G	0.01289	0.0075	A	0.3496	1.728
9	rs114774740	114325415	G	0.01289	0.0075	A	0.3496	1.728
9	rs192587937	114464401	A	0.01289	0.0075	G	0.3496	1.728
10	rs77672823	30336710	A	0.01289	0.0075	T	0.3496	1.728
11	rs2071348	5264146	C	0.1211	0.1417	A	0.3496	0.8351
11	rs188024258	18105213	A	0.01289	0.0075	C	0.3496	1.728
11	rs146021011	110451011	A	0.01289	0.0075	G	0.3496	1.728
12	rs143033072	666917	C	0.01289	0.0075	A	0.3496	1.728
12	rs185965842	110385123	A	0.01289	0.0075	C	0.3496	1.728
12	rs201018883	122437729	C	0.01289	0.0075	G	0.3496	1.728
13	rs138181145	50594532	G	0.01289	0.0075	A	0.3496	1.728
14	rs199618384	20404495	A	0.01289	0.0075	G	0.3496	1.728
14	rs2296928	61442756	C	0.01289	0.0075	A	0.3496	1.728
15	rs202079449	54614271	A	0.01289	0.0075	C	0.3496	1.728
16	rs79546825	14738316	G	0.01289	0.0075	A	0.3496	1.728
16	rs145161932	75646568	G	0.01289	0.0075	A	0.3496	1.728
16	rs140817689	78198138	A	0.01289	0.0075	C	0.3496	1.728
17	rs3027238	8135061	A	0.01289	0.0075	G	0.3496	1.728
17	rs2703806	20108239	T	0.01289	0.0075	A	0.3496	1.728
17	rs2120282	20209474	G	0.01289	0.0075	A	0.3496	1.728
17	rs78242165	48601096	A	0.01289	0.0075	G	0.3496	1.728
17	rs140060241	48762173	A	0.01289	0.0075	G	0.3496	1.728
17	rs17760296	54615617	C	0.01289	0.0075	A	0.3496	1.728
17	rs149907018	62041992	G	0.01289	0.0075	A	0.3496	1.728
17	rs192561539	65732851	A	0.01289	0.0075	G	0.3496	1.728
17	rs188382333	67129926	C	0.01289	0.0075	A	0.3496	1.728
17	rs190646470	67183892	A	0.01289	0.0075	G	0.3496	1.728
17	rs139545118	67303010	A	0.01289	0.0075	C	0.3496	1.728
18	rs200996196	6949209	A	0.01289	0.0075	G	0.3496	1.728
19	rs117645581	7685310	A	0.01289	0.0075	G	0.3496	1.728
19	rs117868611	35612132	G	0.01289	0.0075	A	0.3496	1.728
19	rs11555891	44223113	A	0.1211	0.1417	G	0.3496	0.8351
19	rs186054339	45851252	A	0.01289	0.0075	C	0.3496	1.728
19	rs1054770	49878115	A	0.3454	0.3186	G	0.3496	1.129
19	rs142455549	50086931	G	0.01289	0.0075	C	0.3496	1.728
19	rs138586360	54695642	A	0.01289	0.0075	G	0.3496	1.728
19	rs191636423	56466574	G	0.01289	0.0075	A	0.3496	1.728
20	rs11570019	3785507	A	0.01289	0.0075	G	0.3496	1.728
22	rs9621461	32871365	A	0.07732	0.06333	G	0.3496	1.239

22	rs185758992	38481311	A	0.01289	0.0075	C	0.3496	1.728
22	rs201522786	39639934	A	0.01289	0.0075	C	0.3496	1.728
3	rs1869405	61446281	A	0.4588	0.4875	G	0.3498	0.8911
6	rs6905389	30319930	A	0.09536	0.1142	C	0.3498	0.8179
7	rs17157663	8998976	A	0.09536	0.1142	G	0.3498	0.8179
7	rs6464094	150414572	G	0.4588	0.4875	A	0.3498	0.8911
13	rs7318072	40146225	A	0.5	0.4717	G	0.3498	1.12
14	rs12885391	63098431	A	0.5026	0.4742	G	0.3498	1.12
19	rs7254359	17239566	A	0.5	0.4717	G	0.3498	1.12
20	rs6074755	14530220	A	0.4588	0.4875	C	0.3498	0.8911
6	rs1206153	97546036	G	0.4562	0.4842	A	0.3499	0.8937
8	rs1264202	102227923	A	0.4639	0.4925	C	0.3499	0.8917
8	rs12680655	135637337	G	0.4485	0.4758	C	0.3499	0.8957
11	rs2244621	64026219	A	0.4639	0.4925	G	0.3499	0.8917
18	rs4891159	74101941	A	0.4485	0.4758	G	0.3499	0.8957
19	rs7260548	32677182	A	0.4562	0.4842	G	0.3499	0.8937
3	rs2272522	361508	A	0.3041	0.33	G	0.35	0.8873
3	rs9851851	132609907	A	0.5103	0.4817	G	0.35	1.121
4	rs34611001	5830296	A	0.01289	0.007513	G	0.35	1.725
4	rs370655	74903134	A	0.5103	0.4817	G	0.35	1.121
6	rs9355447	164340663	G	0.3041	0.33	A	0.35	0.8873
13	rs7985274	28299780	G	0.5129	0.4842	A	0.35	1.122
19	rs2240157	1004740	A	0.5155	0.4866	G	0.35	1.122
3	rs6764190	153258272	G	0.5155	0.4867	A	0.3501	1.122
5	rs380441	25696285	A	0.4588	0.4867	C	0.3501	0.8941
6	rs9468925	31258837	A	0.518	0.4892	G	0.3501	1.122
8	rs1595406	55630615	A	0.4588	0.4867	G	0.3501	0.8941
8	rs13280444	139165273	G	0.4923	0.465	A	0.3501	1.115
9	rs7849626	74956781	C	0.4923	0.465	A	0.3501	1.115
14	rs12050260	23761094	A	0.5206	0.4917	G	0.3501	1.123
19	rs3795003	57641799	A	0.4923	0.465	G	0.3501	1.115
1	rs631357	20992819	C	0.4974	0.47	G	0.3502	1.116
1	rs580682	34208399	C	0.4613	0.4892	A	0.3502	0.8944
2	rs2304678	27353507	G	0.1907	0.2142	C	0.3502	0.8647
2	rs4241264	75107048	A	0.4974	0.47	C	0.3502	1.116
4	rs717615	10104670	G	0.4613	0.4892	A	0.3502	0.8944
6	rs1327264	51169536	C	0.4974	0.47	A	0.3502	1.116
9	rs11243897	135618283	G	0.4948	0.4675	A	0.3502	1.116
11	rs11229651	58602309	G	0.4742	0.5025	A	0.3502	0.893
11	rs1917448	103498150	A	0.4613	0.4892	G	0.3502	0.8944
14	rs9658655	93398031	G	0.1907	0.2142	C	0.3502	0.8647
19	rs714106	42083849	C	0.4613	0.4892	A	0.3502	0.8944
22	rs178264	21327589	A	0.4974	0.47	G	0.3502	1.116

22	rs4838815	50713409	G	0.4948	0.4675	A	0.3502	1.116
1	rs4654748	21786068	A	0.4639	0.4917	G	0.3503	0.8947
1	rs1930760	57007539	A	0.4639	0.4917	G	0.3503	0.8947
7	rs2391276	26385004	A	0.4639	0.4917	C	0.3503	0.8947
8	rs10448011	50010896	A	0.5	0.4725	C	0.3503	1.116
8	rs7006364	67199420	A	0.5026	0.475	G	0.3503	1.117
8	rs1318196	144620342	G	0.2758	0.2517	A	0.3503	1.132
10	rs2210969	109033022	G	0.4768	0.505	A	0.3503	0.8933
11	rs10791132	131117445	A	0.5206	0.4925	G	0.3503	1.119
15	rs9920881	35961254	A	0.4639	0.4917	G	0.3503	0.8947
15	rs6496858	92261849	A	0.518	0.49	G	0.3503	1.119
20	rs448960	56531928	A	0.5052	0.4775	G	0.3503	1.117
7	rs1367590	136979849	A	0.5155	0.4875	G	0.3504	1.118
11	rs9630182	13620172	A	0.4665	0.4942	G	0.3504	0.895
13	rs9301584	90209302	A	0.4665	0.4942	G	0.3504	0.895
4	rs1484145	80217741	G	0.1907	0.2133	A	0.3505	0.869
22	rs5764698	45749983	A	0.3067	0.3342	C	0.3506	0.8814
3	rs1982821	177793107	C	0.2397	0.265	A	0.3507	0.8744
12	rs11044004	18435452	A	0.2397	0.265	G	0.3507	0.8744
14	rs7150931	93138041	A	0.4716	0.4992	G	0.3507	0.8957
3	rs2287208	179162316	A	0.4742	0.5017	C	0.3508	0.896
9	rs1549314	127910307	A	0.4742	0.5017	C	0.3508	0.896
2	rs2304681	27315252	A	0.2397	0.2642	G	0.3509	0.8781
2	rs1131375	27323385	A	0.2397	0.2642	G	0.3509	0.8781
19	rs874628	18304700	G	0.1237	0.1058	A	0.351	1.193
2	rs2053163	190827023	G	0.3479	0.3217	A	0.3511	1.125
3	rs2302819	184922294	A	0.1856	0.165	G	0.3515	1.153
3	rs6774273	143969565	A	0.3093	0.3358	G	0.3516	0.8855
10	rs2574951	52043987	A	0.3093	0.3367	G	0.3516	0.8822
6	rs10947338	32708598	T	0.05928	0.0475	A	0.3519	1.264
2	rs4072149	233508677	G	0.3505	0.3242	A	0.352	1.125
11	rs11037445	5462255	C	0.3505	0.3242	G	0.352	1.125
18	rs2156099	70501767	A	0.3505	0.3242	G	0.352	1.125
16	rs3785100	67997920	G	0.1856	0.1653	A	0.3521	1.151
18	rs12103986	56205262	C	0.2423	0.2675	A	0.3523	0.8755
11	rs6421571	118743772	A	0.1521	0.1333	G	0.3524	1.166
17	rs6501384	68291133	A	0.1546	0.1767	G	0.3524	0.8525
1	rs606002	22915753	G	0.2423	0.2667	A	0.3525	0.8793
3	rs118036548	146167089	T	0.07474	0.09083	A	0.3525	0.8085
6	rs34598813	36269725	G	0.07474	0.09083	A	0.3525	0.8085
7	rs7783606	98790669	G	0.07474	0.09083	C	0.3525	0.8085
8	rs12548737	8560536	A	0.07474	0.09167	G	0.3525	0.8005
16	rs12921846	6910385	T	0.3119	0.3392	A	0.3525	0.883

17	rs2289629	27959903	A	0.07474	0.09083	G	0.3525	0.8085
1	rs8480	151733335	C	0.3119	0.3383	A	0.3526	0.8863
1	rs1058405	161751741	G	0.3119	0.3383	A	0.3526	0.8863
4	rs4859571	76857309	A	0.3119	0.3383	G	0.3526	0.8863
4	rs4859572	76857388	A	0.3119	0.3383	G	0.3526	0.8863
5	rs6872806	41336050	A	0.3505	0.325	G	0.3528	1.121
14	rs3742801	74759006	A	0.1933	0.2158	G	0.3528	0.8706
6	rs390659	42666164	G	0.1881	0.1667	C	0.353	1.159
4	rs4234798	7219933	A	0.2809	0.2567	C	0.3532	1.131
9	rs3814113	16915021	G	0.2809	0.2567	A	0.3532	1.131
1	rs2274976	11850927	A	0.1005	0.08417	G	0.3537	1.216
3	rs2306857	112727184	T	0.3557	0.3292	A	0.3537	1.125
16	rs9928668	81209378	A	0.1005	0.08417	G	0.3537	1.216
20	rs3746581	43559199	A	0.02073	0.01417	G	0.3537	1.473
16	rs7193297	72993831	C	0.2448	0.27	A	0.3538	0.8766
1	rs1277207	109395105	G	0.0567	0.07167	A	0.354	0.7786
2	rs3087403	100058870	A	0.07732	0.06417	G	0.354	1.222
6	rs1741780	64081606	A	0.0567	0.07167	C	0.354	0.7786
12	rs10860392	99498187	G	0.0567	0.07167	A	0.354	0.7786
19	rs73048066	46664561	G	0.07732	0.06417	A	0.354	1.222
21	rs2071152	45503121	A	0.2448	0.2692	G	0.3541	0.8803
6	rs1633041	29733223	A	0.1546	0.135	G	0.3542	1.172
6	rs1611208	29759885	A	0.1546	0.135	G	0.3542	1.172
6	rs1633009	29763087	A	0.1546	0.135	T	0.3542	1.172
6	rs3918140	32948462	A	0.1546	0.135	G	0.3542	1.172
8	rs12546065	139356716	G	0.317	0.3442	A	0.3543	0.8845
1	rs1547832	151477193	G	0.1959	0.2192	A	0.3545	0.8678
14	rs7155490	52937255	A	0.317	0.3433	G	0.3545	0.8877
1	rs6033	169521853	G	0.04124	0.05417	A	0.3546	0.751
4	rs6841925	43485769	G	0.09794	0.1158	A	0.3546	0.8287
6	rs11751545	32041043	C	0.04124	0.05417	A	0.3546	0.751
6	rs756138	33755711	C	0.04124	0.05417	G	0.3546	0.751
6	rs17707331	35960390	A	0.04124	0.05417	G	0.3546	0.751
6	rs17713154	35967772	G	0.04124	0.05417	A	0.3546	0.751
10	rs6602175	17142526	C	0.3582	0.3317	A	0.3546	1.125
12	rs4519134	48808927	G	0.3582	0.3317	A	0.3546	1.125
16	rs2303790	57017292	G	0.04124	0.05417	A	0.3546	0.751
18	rs4383230	26862435	A	0.2835	0.2592	G	0.3546	1.131
19	rs34488963	6430106	A	0.09794	0.1167	G	0.3546	0.822
8	rs2582454	137817574	G	0.2294	0.2075	A	0.3548	1.137
15	rs8027765	89169858	A	0.2294	0.2075	G	0.3548	1.137
18	rs8085804	40288293	G	0.2294	0.2075	A	0.3548	1.137
11	rs75482179	18303597	A	0.02835	0.04	G	0.3549	0.7003

12	rs117911920	106786858	C	0.02835	0.04	A	0.3549	0.7003
12	rs2306536	133423662	A	0.02835	0.04	G	0.3549	0.7003
17	rs117262744	7131010	G	0.02835	0.04	A	0.3549	0.7003
2	rs117768861	172180842	A	0.0567	0.0725	G	0.3551	0.769
1	rs3748800	44058143	A	0.02835	0.03917	G	0.3552	0.7158
5	rs55940513	112769840	G	0.02835	0.03917	C	0.3552	0.7158
5	rs17844444	140532165	A	0.02835	0.03917	G	0.3552	0.7158
5	rs3733694	140558528	G	0.02835	0.03917	A	0.3552	0.7158
5	rs17096961	140559849	A	0.02835	0.03917	G	0.3552	0.7158
5	rs3756323	140579358	G	0.02835	0.03917	A	0.3552	0.7158
10	rs75514089	5772920	T	0.02835	0.03917	A	0.3552	0.7158
10	rs76183460	5789418	C	0.02835	0.03917	A	0.3552	0.7158
10	rs11593253	5790729	A	0.02835	0.03917	G	0.3552	0.7158
19	rs80351552	38876688	A	0.02835	0.03917	G	0.3552	0.7158
19	rs146483189	38877168	G	0.02835	0.03917	A	0.3552	0.7158
6	rs7738221	6908368	A	0.3582	0.3325	C	0.3553	1.121
15	rs2292288	99672722	A	0.3582	0.3325	G	0.3553	1.121
1	rs12127469	71849037	A	0.232	0.2092	C	0.3554	1.142
4	rs3860715	1659162	A	0.04124	0.055	G	0.3554	0.739
10	rs3739998	30316072	C	0.1572	0.1783	G	0.3554	0.8595
11	rs35982635	64802388	C	0.04124	0.055	A	0.3554	0.739
11	rs540261	118947634	G	0.232	0.2092	A	0.3554	1.142
17	rs999796	29159404	C	0.1572	0.1783	G	0.3554	0.8595
17	rs61359568	78298926	A	0.04124	0.055	G	0.3554	0.739
1	rs148169601	908390	A	0.02062	0.01417	G	0.3555	1.465
1	rs188024983	2252827	A	0.02062	0.01417	G	0.3555	1.465
2	rs3749081	98382991	C	0.02062	0.01417	G	0.3555	1.465
2	rs77802032	131103405	G	0.02062	0.01417	A	0.3555	1.465
3	rs6767237	183976241	G	0.02062	0.01417	A	0.3555	1.465
4	rs1800561	15826558	A	0.02062	0.01417	G	0.3555	1.465
5	rs144607594	79952348	A	0.02062	0.01417	G	0.3555	1.465
11	rs11604232	6585032	A	0.02062	0.01417	G	0.3555	1.465
11	rs140147916	6588944	A	0.02062	0.01417	G	0.3555	1.465
12	rs74691643	64485153	A	0.02062	0.01417	G	0.3555	1.465
12	rs148804891	100433407	C	0.02062	0.01417	A	0.3555	1.465
13	rs80359065	32937526	A	0.02062	0.01417	C	0.3555	1.465
13	rs75187049	114303822	A	0.02062	0.01417	G	0.3555	1.465
14	rs1046428	77794283	A	0.02062	0.01417	G	0.3555	1.465
15	rs2652834	63396867	A	0.02062	0.01417	G	0.3555	1.465
16	rs143952603	112659	A	0.02062	0.01417	G	0.3555	1.465
16	rs144648182	72110589	A	0.02062	0.01417	G	0.3555	1.465
19	rs200013279	1360692	A	0.02062	0.01417	G	0.3555	1.465
19	rs186722296	1790763	A	0.02062	0.01417	G	0.3555	1.465

1	rs1134586	40980559	A	0.01804	0.0275	G	0.3556	0.6497
1	rs12405001	242428735	C	0.01804	0.0275	G	0.3556	0.6497
2	rs138217960	152423951	A	0.01804	0.0275	G	0.3556	0.6497
5	rs3752704	139907810	G	0.01804	0.0275	A	0.3556	0.6497
9	rs75765336	136029301	A	0.01804	0.0275	G	0.3556	0.6497
10	rs3781408	126715692	A	0.01804	0.0275	G	0.3556	0.6497
1	rs164898	91569689	G	0.1263	0.1083	A	0.3557	1.19
5	rs2288466	26988328	A	0.1907	0.1692	G	0.3557	1.157
5	rs2288467	26988424	G	0.1907	0.1692	A	0.3557	1.157
11	rs4926	57381989	A	0.1263	0.1083	G	0.3557	1.19
15	rs34988193	65235776	G	0.1263	0.1083	A	0.3557	1.19
17	rs4794665	54850329	A	0.1907	0.1692	G	0.3557	1.157
6	rs1632987	29772549	G	0.1546	0.1358	C	0.3559	1.164
16	rs925331	84012104	G	0.1546	0.1358	A	0.3559	1.164
6	rs2857114	32779996	G	0.2861	0.2617	A	0.356	1.131
15	rs6496074	95511837	G	0.2861	0.2617	A	0.356	1.131
2	rs61746887	133541422	A	0.1005	0.085	T	0.3561	1.203
2	rs17868387	234854552	G	0.1005	0.085	A	0.3561	1.203
9	rs3827816	117848197	A	0.3222	0.3492	G	0.3561	0.8859
16	rs76033196	81187635	A	0.1005	0.085	C	0.3561	1.203
16	rs77318108	81187709	T	0.1005	0.085	A	0.3561	1.203
19	rs16983353	52496149	A	0.3222	0.3492	G	0.3561	0.8859
19	rs1978717	52497250	A	0.3222	0.3492	G	0.3561	0.8859
19	rs10500311	52497943	A	0.3222	0.3492	G	0.3561	0.8859
2	rs1056104	39008949	A	0.3222	0.3483	G	0.3564	0.8892
4	rs730741	168610140	A	0.1985	0.2225	G	0.3567	0.8652
14	rs2236338	25100282	G	0.1985	0.2217	A	0.3567	0.8694
2	rs80028181	71357884	A	0.01804	0.02833	C	0.3568	0.6301
3	rs2665390	156397749	G	0.01804	0.02833	A	0.3568	0.6301
5	rs3110234	38486065	A	0.01804	0.02833	G	0.3568	0.6301
6	rs2239697	32782897	A	0.01804	0.02833	G	0.3568	0.6301
12	rs75025746	22814023	G	0.01804	0.02833	A	0.3568	0.6301
19	rs427127	53086032	A	0.01804	0.02833	G	0.3568	0.6301
1	rs2232016	107599918	A	0.1907	0.17	G	0.3569	1.151
6	rs10498792	51666631	G	0.1907	0.17	A	0.3569	1.151
12	rs7306523	53393964	G	0.3634	0.3375	A	0.3569	1.121
6	rs2523734	30129676	C	0.1263	0.1467	A	0.357	0.841
15	rs4842860	83680287	A	0.3247	0.3517	G	0.357	0.8866
19	rs4805975	34556604	G	0.3247	0.3517	A	0.357	0.8866
2	rs11902811	68729992	G	0.04639	0.035	A	0.3573	1.341
7	rs7807131	95001132	A	0.04639	0.035	G	0.3573	1.341
9	rs3739757	13219593	A	0.04639	0.035	G	0.3573	1.341
9	rs59249347	140330497	A	0.04639	0.035	G	0.3573	1.341

13	rs7323	28009031	G	0.04639	0.035	C	0.3573	1.341
13	rs3751336	29855847	A	0.04639	0.035	G	0.3573	1.341
19	rs11085120	5270931	G	0.3247	0.3508	A	0.3573	0.8899
1	rs3091242	25674785	A	0.366	0.34	G	0.3577	1.121
2	rs2366894	32713706	T	0.366	0.34	A	0.3577	1.121
7	rs6945306	23757162	C	0.366	0.34	G	0.3577	1.121
10	rs2296613	21074455	G	0.3273	0.3542	A	0.3578	0.8873
11	rs55987642	67200812	A	0.0799	0.06583	G	0.3578	1.232
19	rs34052647	42911769	A	0.0799	0.06583	G	0.3578	1.232
9	rs7037849	139973820	G	0.3686	0.3417	A	0.3579	1.125
14	rs1243647	21024619	A	0.3686	0.3417	G	0.3579	1.125
14	rs12590446	21024786	A	0.3686	0.3417	G	0.3579	1.125
6	rs10944433	89868128	G	0.06186	0.04917	A	0.358	1.275
11	rs11539762	69063510	A	0.1598	0.1817	G	0.358	0.8567
11	rs11228610	69063728	G	0.1598	0.1817	C	0.358	0.8567
22	rs61995891	43936117	A	0.06186	0.04917	G	0.358	1.275
4	rs2229940	46995366	A	0.3273	0.3533	C	0.3581	0.8906
8	rs7830618	100926037	G	0.1598	0.1808	A	0.3582	0.8615
10	rs11192036	106152111	A	0.2526	0.2775	T	0.3582	0.8798
14	rs2236264	93199080	A	0.1598	0.1808	G	0.3582	0.8615
14	rs4647899	33293531	A	0.3796	0.3525	T	0.3584	1.124
6	rs210139	33543409	A	0.3711	0.3442	C	0.3587	1.125
6	rs12200969	89926966	G	0.07732	0.09417	A	0.3587	0.8061
7	rs6962756	123256427	G	0.2345	0.2125	A	0.3587	1.135
9	rs965897	77175017	C	0.2345	0.2125	A	0.3587	1.135
14	rs2877832	27800177	A	0.07732	0.09417	G	0.3587	0.8061
14	rs3742836	73220020	G	0.201	0.2242	A	0.3587	0.8708
17	rs3091315	32593665	A	0.3711	0.3442	G	0.3587	1.125
10	rs3088142	76854564	G	0.07732	0.09333	A	0.3589	0.814
4	rs7672688	113001776	A	0.1572	0.1383	G	0.3594	1.162
9	rs25692	74332970	A	0.1005	0.1183	G	0.3594	0.8326
12	rs7969931	25242595	G	0.1005	0.1192	C	0.3594	0.826
1	rs1497828	217527024	G	0.1933	0.1725	C	0.3595	1.149
6	rs6918464	3032804	A	0.3737	0.3467	G	0.3595	1.125
15	rs2125623	31829707	A	0.3737	0.3467	G	0.3595	1.125
19	rs61743859	51983673	A	0.101	0.12	G	0.3597	0.8242
2	rs17007544	29295866	G	0.2912	0.2675	A	0.3598	1.125
2	rs7595103	78075244	G	0.1031	0.08667	A	0.3598	1.211
19	rs2682585	44081288	A	0.1031	0.08667	G	0.3598	1.211
1	rs11806429	6550505	A	0.2938	0.2692	G	0.3601	1.13
13	rs4942848	50141345	G	0.3351	0.3625	A	0.3602	0.8861
19	rs11575934	18186618	G	0.3351	0.3625	A	0.3602	0.8861
13	rs17078601	23906983	G	0.1289	0.1108	C	0.3603	1.187

16	rs8166	90025640	C	0.1289	0.1486	G	0.3605	0.8477
12	rs2584021	71029733	A	0.3763	0.35	G	0.3606	1.12
12	rs2074356	112645401	A	0.1289	0.1492	G	0.3606	0.8438
7	rs35912436	12334083	G	0.1289	0.1483	A	0.3607	0.8493
5	rs40986	135276314	G	0.2397	0.2162	A	0.3608	1.143
8	rs10109414	23751151	A	0.2036	0.2267	G	0.3608	0.8723
1	rs150966402	43892987	A	0.01031	0.01833	G	0.3609	0.5578
1	rs151193009	55509585	A	0.01031	0.01833	G	0.3609	0.5578
1	rs560389	92647223	A	0.01031	0.01833	G	0.3609	0.5578
2	rs188500710	69206066	A	0.01031	0.01833	G	0.3609	0.5578
2	rs117870995	113950108	A	0.01031	0.01833	G	0.3609	0.5578
3	rs146292440	44884627	A	0.01031	0.01833	G	0.3609	0.5578
3	rs78565722	50099500	G	0.01031	0.01833	A	0.3609	0.5578
3	rs139593877	151164013	A	0.01031	0.01833	G	0.3609	0.5578
3	rs141667917	169578448	T	0.01031	0.01833	A	0.3609	0.5578
5	rs139626065	145895270	A	0.01031	0.01833	G	0.3609	0.5578
6	rs4576240	24596478	A	0.01031	0.01833	C	0.3609	0.5578
6	rs116943808	117866721	A	0.01031	0.01833	T	0.3609	0.5578
7	rs42235	92248076	A	0.01031	0.01833	G	0.3609	0.5578
8	rs150886774	144732181	A	0.01031	0.01833	G	0.3609	0.5578
9	rs147046649	20933030	C	0.01031	0.01833	A	0.3609	0.5578
9	rs75769247	125391653	A	0.01031	0.01833	C	0.3609	0.5578
12	rs11613495	4554630	G	0.01031	0.01833	A	0.3609	0.5578
12	rs58801491	122260549	G	0.01031	0.01833	A	0.3609	0.5578
14	rs74357167	23346025	A	0.01031	0.01833	G	0.3609	0.5578
14	rs8010888	105833679	G	0.01031	0.01833	A	0.3609	0.5578
15	rs140893433	100272109	G	0.01031	0.01833	A	0.3609	0.5578
16	rs117078057	81077664	A	0.01031	0.01833	G	0.3609	0.5578
18	rs139560400	44593458	C	0.01031	0.01833	A	0.3609	0.5578
19	rs141830432	6467619	A	0.01031	0.01833	C	0.3609	0.5578
8	rs310558	51412591	G	0.1624	0.1833	A	0.361	0.8635
12	rs11180311	75360228	G	0.3376	0.365	A	0.361	0.8868
19	rs10407640	34766588	A	0.3376	0.365	G	0.361	0.8868
22	rs6009824	50086373	A	0.1624	0.1833	G	0.361	0.8635
1	rs6426616	20490518	A	0.2577	0.2825	G	0.3611	0.8819
6	rs7194	32412480	G	0.3376	0.3642	A	0.3611	0.89
6	rs2227139	32413459	G	0.3376	0.3642	A	0.3611	0.89
8	rs2618476	11352541	A	0.2938	0.27	G	0.3611	1.125
19	rs4147918	1058176	G	0.1598	0.14	A	0.3613	1.168
1	rs10800098	165409095	A	0.3763	0.3508	G	0.3615	1.116
2	rs3739014	101638888	G	0.3402	0.3675	A	0.3617	0.8874
1	rs696698	75065441	A	0.0799	0.06667	G	0.3618	1.216
7	rs2289366	45113121	A	0.01047	0.01833	G	0.3618	0.5666

9	rs11144688	78542286	A	0.0799	0.06667	G	0.3618	1.216
2	rs746924	101624471	G	0.3402	0.3667	A	0.3619	0.8906
3	rs17366568	186570453	A	0.03351	0.02417	G	0.3619	1.4
5	rs66812017	104952574	A	0.3402	0.3664	G	0.3619	0.8915
11	rs4353250	18323517	A	0.03351	0.02417	G	0.3619	1.4
11	rs11230562	60776186	A	0.03351	0.02417	G	0.3619	1.4
12	rs11546871	132401566	A	0.03351	0.02417	G	0.3619	1.4
17	rs13306168	4544904	G	0.03351	0.02417	A	0.3619	1.4
20	rs142639450	61451333	A	0.03351	0.02417	G	0.3619	1.4
1	rs6426219	247259684	A	0.3814	0.355	G	0.3621	1.12
8	rs42490	90778513	A	0.3814	0.355	G	0.3621	1.12
11	rs4298923	58190136	G	0.3814	0.355	A	0.3621	1.12
13	rs576674	33554302	G	0.1959	0.175	A	0.3621	1.148
14	rs861547	104155317	A	0.3814	0.355	G	0.3621	1.12
1	rs1886686	67390468	C	0.1289	0.1117	G	0.3622	1.177
17	rs11538161	1371393	A	0.1289	0.1117	G	0.3622	1.177
1	rs41265234	173772330	G	0.04639	0.03583	C	0.3623	1.309
1	rs144437187	186099099	A	0.04639	0.03583	G	0.3623	1.309
2	rs35002937	68753240	A	0.04639	0.03583	T	0.3623	1.309
13	rs9558562	106124937	G	0.04639	0.03583	A	0.3623	1.309
15	rs690347	41105926	A	0.05928	0.07417	C	0.3623	0.7866
3	rs7619322	151171439	A	0.2603	0.2858	C	0.3624	0.8793
12	rs11058659	126940768	G	0.2603	0.2858	A	0.3624	0.8793
17	rs46521	46988529	A	0.2603	0.2858	G	0.3624	0.8793
17	rs46522	46988597	G	0.2603	0.2858	A	0.3624	0.8793
5	rs10043775	147805120	G	0.1959	0.1753	A	0.3626	1.146
9	rs117352600	117186678	A	0.06186	0.05	G	0.3626	1.253
16	rs1895557	61022169	A	0.3428	0.3692	G	0.3626	0.8913
2	rs472161	30930165	A	0.384	0.3575	G	0.3627	1.12
6	rs2524005	29899677	A	0.2062	0.23	G	0.3627	0.8696
6	rs2068204	33058718	A	0.384	0.3575	G	0.3627	1.12
17	rs4393623	18775900	G	0.3428	0.3689	A	0.3627	0.8921
1	rs1136164	57002627	A	0.1598	0.1408	G	0.3628	1.16
4	rs1379659	20620683	G	0.1598	0.1408	A	0.3628	1.16
5	rs12522248	156479426	G	0.1598	0.1408	A	0.3628	1.16
6	rs4412248	33072415	G	0.3814	0.3558	C	0.3628	1.116
8	rs10503253	4180844	A	0.299	0.2742	C	0.3628	1.129
11	rs974819	103660567	G	0.3814	0.3558	A	0.3628	1.116
2	rs4671124	65495283	A	0.05928	0.075	G	0.3632	0.7772
2	rs61739715	71649966	G	0.05928	0.075	A	0.3632	0.7772
4	rs324689	76730195	G	0.05928	0.075	A	0.3632	0.7772
6	rs2301271	32725193	A	0.2423	0.2192	G	0.3632	1.139
11	rs544864	58978940	A	0.3454	0.3725	G	0.3632	0.8887

11	rs487989	65064690	A	0.05928	0.075	G	0.3632	0.7772
1	rs3795677	240492734	A	0.3866	0.36	G	0.3634	1.12
2	rs41464348	33527299	A	0.3866	0.36	G	0.3634	1.12
6	rs61738275	31380198	A	0.1658	0.1875	G	0.3634	0.8613
6	rs11551421	33053609	A	0.384	0.3583	G	0.3634	1.116
12	rs7973756	102064518	G	0.3866	0.36	A	0.3634	1.12
16	rs1127920	5097947	G	0.1649	0.1867	A	0.3634	0.8607
19	rs12460876	33356891	A	0.299	0.275	G	0.3636	1.124
17	rs9891572	2428508	A	0.2629	0.2883	G	0.3637	0.8803
1	rs2271933	32092525	G	0.2629	0.2875	A	0.3639	0.8839
6	rs664370	36393816	A	0.3479	0.375	G	0.3639	0.8893
9	rs17370391	98319969	G	0.1031	0.1217	A	0.364	0.8298
11	rs10741747	18586122	G	0.3015	0.2767	A	0.364	1.129
2	rs1106639	242690675	A	0.1031	0.1208	G	0.3641	0.8363
9	rs34498078	25677953	A	0.1314	0.1517	G	0.3641	0.8465
16	rs1105066	88504850	C	0.3892	0.3625	G	0.3641	1.12
17	rs2587507	77790135	G	0.3479	0.3742	A	0.3641	0.8925
18	rs61735987	34156497	G	0.1314	0.1517	A	0.3641	0.8465
20	rs746192	61391977	C	0.1031	0.1208	A	0.3641	0.8363
7	rs7776725	121033121	G	0.1314	0.1508	A	0.3643	0.852
6	rs4267943	49439805	A	0.2088	0.2325	G	0.3646	0.871
10	rs772394	35980025	G	0.2088	0.2325	A	0.3646	0.871
7	rs2525743	120565264	A	0.3918	0.365	G	0.3647	1.121
14	rs2232059	69922552	G	0.1624	0.1425	A	0.3647	1.166
19	rs25667	50967640	A	0.1624	0.1425	G	0.3647	1.166
1	rs145561033	167654716	A	0.01031	0.01917	C	0.3648	0.5331
1	rs2738755	221057646	A	0.0799	0.09667	G	0.3648	0.8115
2	rs143800457	74594519	G	0.01031	0.01917	A	0.3648	0.5331
2	rs10928302	147372024	A	0.2088	0.2317	G	0.3648	0.875
2	rs776488	165890733	C	0.01031	0.01917	A	0.3648	0.5331
2	rs141506076	219206292	A	0.01031	0.01917	G	0.3648	0.5331
3	rs11719086	122418750	A	0.0799	0.09667	G	0.3648	0.8115
3	rs13093808	122419083	A	0.0799	0.09667	C	0.3648	0.8115
3	rs9816226	185834499	T	0.01031	0.01917	A	0.3648	0.5331
4	rs144951440	6302757	G	0.01031	0.01917	A	0.3648	0.5331
5	rs3733782	7868188	T	0.01031	0.01917	A	0.3648	0.5331
6	rs148547958	56426246	G	0.01031	0.01917	A	0.3648	0.5331
6	rs17847328	144263274	G	0.01031	0.01917	A	0.3648	0.5331
7	rs77743816	76019476	A	0.01031	0.01917	G	0.3648	0.5331
7	rs113857788	117304834	G	0.01031	0.01917	C	0.3648	0.5331
8	rs16916207	52325767	C	0.3015	0.2775	A	0.3648	1.124
9	rs150594929	35107455	G	0.01031	0.01917	A	0.3648	0.5331
9	rs41306498	117139280	G	0.01031	0.01917	A	0.3648	0.5331

10	rs2228065	45920506	A	0.01031	0.01917	G	0.3648	0.5331
10	rs74537149	116068193	A	0.01031	0.01917	G	0.3648	0.5331
11	rs117594890	4790921	A	0.01031	0.01917	G	0.3648	0.5331
12	rs145371745	2061956	A	0.01031	0.01917	G	0.3648	0.5331
13	rs1333026	66120784	A	0.0799	0.09667	G	0.3648	0.8115
16	rs145721165	67578623	A	0.01031	0.01917	G	0.3648	0.5331
18	rs7232237	31324934	A	0.0799	0.09667	G	0.3648	0.8115
18	rs16956461	50797444	G	0.01031	0.01917	A	0.3648	0.5331
21	rs148576261	31933451	A	0.01031	0.01917	G	0.3648	0.5331
1	rs1288621	53946644	G	0.3505	0.3767	A	0.3649	0.8931
3	rs9841329	38687803	G	0.3505	0.3767	A	0.3649	0.8931
5	rs2431352	75923294	A	0.0799	0.09583	C	0.365	0.8193
6	rs11970154	30919878	C	0.0799	0.09583	G	0.365	0.8193
9	rs28655161	114831896	A	0.0799	0.09583	G	0.365	0.8193
15	rs28744617	42981022	G	0.0799	0.09583	A	0.365	0.8193
11	rs12049823	74109166	G	0.2655	0.29	A	0.3652	0.8848
12	rs10876993	58062667	A	0.2655	0.29	G	0.3652	0.8848
5	rs1031389	93918392	G	0.3918	0.3658	A	0.3653	1.116
10	rs2442904	25994745	A	0.3531	0.38	G	0.3653	0.8905
15	rs11070883	52483565	G	0.3918	0.3658	A	0.3653	1.116
9	rs613444	13682827	G	0.3943	0.3675	A	0.3654	1.121
11	rs3741056	107992346	C	0.3531	0.3792	G	0.3656	0.8937
19	rs118046250	31039146	G	0.08247	0.06833	A	0.3656	1.226
20	rs926487	25666737	A	0.08247	0.06833	G	0.3656	1.226
1	rs6018	169511878	C	0.04381	0.05676	A	0.3657	0.7615
19	rs2227278	36206050	C	0.1057	0.08917	A	0.3657	1.207
1	rs75687095	1847886	A	0.04381	0.05667	G	0.3658	0.7628
1	rs144200280	17431434	A	0.04381	0.05667	G	0.3658	0.7628
6	rs117024916	30993419	G	0.04381	0.05667	A	0.3658	0.7628
6	rs28360986	30994248	G	0.04381	0.05667	C	0.3658	0.7628
8	rs117397164	21996546	A	0.04381	0.05667	G	0.3658	0.7628
20	rs79534510	25263834	G	0.04381	0.05667	C	0.3658	0.7628
5	rs6891871	95357625	A	0.3557	0.3823	G	0.3659	0.8919
12	exm1016213	57859431	A	0.3557	0.3823	G	0.3659	0.8919
1	rs6587467	248550976	A	0.3969	0.37	C	0.366	1.121
4	rs6853596	181460127	G	0.3969	0.37	A	0.366	1.121
6	rs4713600	32822858	A	0.3969	0.37	C	0.366	1.121
6	rs7767288	32830013	A	0.3969	0.37	G	0.366	1.121
6	rs6938130	32831247	T	0.3969	0.37	A	0.366	1.121
6	rs287928	157150145	A	0.3969	0.37	G	0.366	1.121
11	rs2017434	5536852	G	0.3969	0.37	A	0.366	1.121
12	rs2228225	57859431	A	0.3557	0.3825	G	0.366	0.8911
17	rs2032844	46847364	A	0.1675	0.1892	C	0.366	0.8626

11	rs10743108	9537904	C	0.3041	0.28	G	0.3661	1.124
12	exm2251115	6167196	G	0.1624	0.1433	A	0.3661	1.159
12	rs1800379	6167196	G	0.1624	0.1433	A	0.3661	1.159
1	rs17426456	117113552	G	0.04381	0.0575	A	0.3663	0.7511
2	rs10496288	83292565	G	0.1675	0.19	A	0.3663	0.8579
3	rs2228000	14199887	A	0.268	0.2933	G	0.3663	0.8822
6	rs4418214	31391401	G	0.04404	0.05667	A	0.3663	0.7669
8	rs16902094	128320346	G	0.268	0.2933	A	0.3663	0.8822
9	rs1535480	7925381	A	0.268	0.2933	C	0.3663	0.8822
10	rs2228529	50667105	G	0.04381	0.0575	A	0.3663	0.7511
12	rs1373549	46485838	A	0.268	0.2933	G	0.3663	0.8822
14	rs12436220	47950171	G	0.3557	0.3817	A	0.3663	0.8943
16	rs16967494	15820863	A	0.3557	0.3817	G	0.3663	0.8943
16	rs12931472	16281007	G	0.1985	0.1783	A	0.3663	1.141
6	rs9449444	82900811	A	0.2124	0.2353	G	0.3664	0.8764
6	rs2535294	31062133	G	0.3969	0.3708	A	0.3665	1.117
19	rs8102476	38735613	G	0.3969	0.3708	A	0.3665	1.117
3	rs2340917	14175262	A	0.3995	0.3725	G	0.3666	1.121
3	rs16843645	135745911	A	0.1314	0.1142	G	0.3666	1.174
3	rs13075089	190578566	G	0.3582	0.385	A	0.3666	0.8917
4	rs2236786	1719294	G	0.1314	0.1142	A	0.3666	1.174
4	rs798766	1734239	A	0.1314	0.1142	G	0.3666	1.174
6	rs2245961	29944004	C	0.3582	0.385	A	0.3666	0.8917
7	rs7808531	5106222	A	0.3995	0.3725	G	0.3666	1.121
4	rs2288255	79786856	A	0.2113	0.2342	G	0.3667	0.8764
2	rs3749006	168115710	C	0.2474	0.2242	A	0.3669	1.138
19	rs3745833	56693620	G	0.2474	0.2242	C	0.3669	1.138
6	rs2844542	31347274	C	0.3582	0.3842	G	0.367	0.8949
10	rs10761731	65027610	T	0.3608	0.3875	A	0.3673	0.8923
19	rs8101643	21384253	G	0.3608	0.3875	A	0.3673	0.8923
19	rs2356437	44352665	G	0.3608	0.3875	A	0.3673	0.8923
19	rs630283	50745408	G	0.3608	0.3875	A	0.3673	0.8923
20	rs113352451	34797455	A	0.008021	0.003344	G	0.3674	2.41
6	rs3128955	33021192	A	0.134	0.1542	T	0.3676	0.8491
1	rs10458561	70921173	A	0.1057	0.09	G	0.3678	1.195
3	rs9832653	178585678	G	0.4046	0.3775	A	0.3678	1.121
5	rs31244	75594743	A	0.1057	0.09	G	0.3678	1.195
12	rs7294902	112354531	G	0.2474	0.225	A	0.3678	1.132
12	rs3752630	112375990	C	0.2474	0.225	A	0.3678	1.132
7	rs712735	129171989	A	0.3634	0.39	G	0.3679	0.8929
6	rs1265078	31112602	C	0.1328	0.1158	G	0.3681	1.169
2	rs1729413	201086663	C	0.4046	0.3783	A	0.3682	1.117
1	rs10067	111957311	G	0.366	0.3933	C	0.3684	0.8903

2	rs1050354	170493103	A	0.366	0.3933	T	0.3684	0.8903
6	rs2473517	139428758	A	0.2139	0.2375	G	0.3684	0.8737
9	rs10758593	4292083	A	0.366	0.3933	G	0.3684	0.8903
10	rs1329231	19209184	G	0.366	0.3933	A	0.3684	0.8903
19	rs4805885	33906123	G	0.366	0.3933	A	0.3684	0.8903
21	rs3761342	33694224	C	0.2139	0.2375	G	0.3684	0.8737
2	rs6730905	173341828	G	0.366	0.3925	A	0.3685	0.8934
9	rs2492626	29939556	A	0.366	0.3925	G	0.3685	0.8934
15	rs2412504	40558064	G	0.1057	0.1242	A	0.3685	0.8334
15	rs3743135	40558482	G	0.1057	0.1242	A	0.3685	0.8334
1	rs4149963	242035382	A	0.1057	0.1233	G	0.3687	0.8399
2	rs2952381	164263699	G	0.25	0.2267	A	0.3687	1.137
3	rs74823804	63976489	A	0.06443	0.05167	G	0.3687	1.264
4	rs3749574	151207127	A	0.2139	0.2367	G	0.3687	0.8777
6	rs3817928	142750516	G	0.1057	0.1233	A	0.3687	0.8399
8	rs1346044	31024654	G	0.1057	0.1233	A	0.3687	0.8399
8	rs7009183	96731898	A	0.1057	0.1233	G	0.3687	0.8399
8	rs142539426	110413752	A	0.06443	0.05167	T	0.3687	1.264
15	rs2304380	33855138	G	0.06443	0.05167	A	0.3687	1.264
4	rs4371677	57516896	A	0.3686	0.3958	G	0.369	0.8909
5	rs35391	33955673	G	0.4046	0.3792	A	0.369	1.113
6	rs3748069	142767633	G	0.4046	0.3792	A	0.369	1.113
10	rs11197825	118551705	A	0.3686	0.3958	C	0.369	0.8909
19	rs2659053	51340794	G	0.4046	0.3792	A	0.369	1.113
22	rs2269529	36684354	A	0.3686	0.3958	G	0.369	0.8909
8	rs1350058	63874975	A	0.3686	0.395	G	0.3691	0.894
4	rs1827951	117151215	A	0.134	0.1158	G	0.3692	1.181
6	rs3757261	13599303	A	0.134	0.1158	G	0.3692	1.181
11	rs12286721	47701528	C	0.2732	0.2975	A	0.3692	0.8876
18	rs671871	6977844	G	0.134	0.1158	A	0.3692	1.181
22	rs6010164	50515270	G	0.134	0.1158	A	0.3692	1.181
16	rs77682584	839269	A	0.1649	0.1458	G	0.3693	1.157
16	rs1231122	3293888	A	0.4098	0.3833	G	0.3693	1.117
6	rs2228396	32797809	A	0.08247	0.06917	G	0.3694	1.21
6	rs3734292	112430669	A	0.08247	0.06917	G	0.3694	1.21
22	rs2272830	39907941	C	0.08247	0.06917	G	0.3694	1.21
6	rs78292651	74104285	C	0.2036	0.1825	A	0.3696	1.145
7	rs17132399	2472707	G	0.25	0.2275	C	0.3696	1.132
10	rs4536103	71332204	G	0.3119	0.2875	A	0.3696	1.123
18	rs1115562	30114188	C	0.3119	0.2875	A	0.3696	1.123
8	rs968381	2048831	G	0.3711	0.3975	A	0.3697	0.8945
1	rs970973	7202190	G	0.4124	0.3858	A	0.3698	1.117
4	rs4623048	41833487	A	0.04639	0.03667	G	0.3698	1.278

6	rs2523567	31330015	C	0.04639	0.03667	G	0.3698	1.278
15	rs34355056	65703625	G	0.04639	0.03667	C	0.3698	1.278
1	rs11204697	150658971	A	0.2758	0.3008	G	0.3701	0.885
1	rs1336900	150679033	A	0.2758	0.3008	G	0.3701	0.885
2	rs13392800	38759797	A	0.2758	0.3008	C	0.3701	0.885
17	rs3760460	1538302	A	0.06186	0.07679	C	0.3701	0.7926
6	rs2170185	32849639	G	0.3144	0.2892	A	0.3702	1.127
9	rs2277168	95400370	A	0.06186	0.07667	G	0.3702	0.7941
13	rs1305088	29278450	A	0.3144	0.2892	G	0.3702	1.127
17	rs4790250	1538546	G	0.06186	0.07667	A	0.3702	0.7941
1	rs6686842	41530871	A	0.3737	0.4	G	0.3703	0.8951
7	rs66755489	47874630	A	0.03093	0.0425	G	0.3703	0.719
12	rs77729665	93171826	A	0.03093	0.0425	G	0.3703	0.719
13	rs9561329	94011169	G	0.3737	0.4	A	0.3703	0.8951
14	rs12586317	35682172	G	0.4149	0.3883	A	0.3703	1.117
21	rs2276231	38437951	C	0.03093	0.0425	A	0.3703	0.719
5	rs17844514	140567973	A	0.03351	0.025	G	0.3705	1.352
6	rs362513	29528318	G	0.2758	0.3	A	0.3705	0.8885
6	rs12201676	89732101	G	0.03351	0.025	A	0.3705	1.352
7	rs2697180	121046807	C	0.2526	0.2292	A	0.3705	1.137
14	rs77129887	68265046	G	0.03351	0.025	A	0.3705	1.352
17	rs77919366	38978462	A	0.03351	0.025	G	0.3705	1.352
17	rs9899862	72249229	A	0.03351	0.025	C	0.3705	1.352
1	rs7513590	5170712	G	0.08247	0.09917	A	0.3707	0.8165
3	rs323871	51990605	C	0.08247	0.09917	G	0.3707	0.8165
4	rs17085113	55787043	G	0.3763	0.4033	A	0.3707	0.8925
6	rs2517538	31013541	G	0.3763	0.4033	C	0.3707	0.8925
7	rs12538505	133136872	A	0.3763	0.4033	G	0.3707	0.8925
8	rs35647122	19218745	G	0.08247	0.09917	A	0.3707	0.8165
18	rs4800148	20724328	G	0.3144	0.29	A	0.3707	1.123
1	rs4494160	109268573	G	0.2539	0.2308	A	0.3708	1.134
5	rs10479488	177396392	G	0.4149	0.3892	A	0.3709	1.113
6	rs8192575	32166384	G	0.1366	0.1569	C	0.3709	0.85
19	rs2303729	41111069	A	0.3763	0.4025	G	0.3709	0.8956
2	rs78076311	239039970	A	0.03351	0.02504	G	0.371	1.35
3	rs4618222	164586522	A	0.2036	0.1833	G	0.371	1.139
5	rs4867440	32403346	G	0.1057	0.09083	A	0.371	1.183
13	rs4771122	28020180	G	0.2036	0.1833	A	0.371	1.139
14	rs10483776	65914867	G	0.1057	0.09083	A	0.371	1.183
19	rs147391627	44500475	C	0.007772	0.003333	A	0.371	2.342
1	rs983332	88132380	A	0.2784	0.3033	C	0.3713	0.8859
6	rs1592404	29358676	C	0.1727	0.195	G	0.3713	0.8617
6	rs9405124	29368813	G	0.1727	0.195	A	0.3713	0.8617

8	rs2100347	64499604	G	0.2526	0.23	A	0.3713	1.131
15	rs8036796	38118873	G	0.2784	0.3033	A	0.3713	0.8859
15	rs2305707	51569410	G	0.2784	0.3042	A	0.3713	0.8824
19	rs4808863	18980172	A	0.2526	0.23	G	0.3713	1.131
19	rs10419198	50038017	A	0.2526	0.23	G	0.3713	1.131
3	rs9657904	105586714	A	0.4175	0.3917	G	0.3714	1.113
12	rs11610206	47639526	G	0.1675	0.1475	A	0.3714	1.163
13	rs141399003	52726781	G	0.007772	0.003344	A	0.3714	2.334
17	rs227787	3324810	G	0.4175	0.3917	A	0.3714	1.113
17	rs8064733	39135084	G	0.1675	0.1475	A	0.3714	1.163
6	rs2517550	31008368	G	0.3789	0.405	A	0.3715	0.8961
6	rs10943716	81253073	G	0.3789	0.405	A	0.3715	0.8961
10	rs12778533	57105244	G	0.322	0.2975	A	0.3715	1.121
1	rs7535752	8395560	A	0.007732	0.003333	C	0.3717	2.33
1	rs201994917	20073053	A	0.007732	0.003333	C	0.3717	2.33
1	rs41264113	20141405	A	0.007732	0.003333	G	0.3717	2.33
1	rs6657616	26527951	A	0.007732	0.003333	G	0.3717	2.33
1	rs45500401	47278169	A	0.007732	0.003333	G	0.3717	2.33
1	rs75421517	50572019	C	0.007732	0.003333	A	0.3717	2.33
1	rs117523576	109359702	A	0.007732	0.003333	G	0.3717	2.33
1	rs35393723	159505101	A	0.007732	0.003333	G	0.3717	2.33
1	rs202163820	229601256	G	0.007732	0.003333	A	0.3717	2.33
2	rs201642875	23980848	A	0.007732	0.003333	G	0.3717	2.33
2	rs141559644	95976152	A	0.007732	0.003333	G	0.3717	2.33
2	rs189205115	113309560	A	0.007732	0.003333	G	0.3717	2.33
2	rs13419085	179702437	G	0.007732	0.003333	A	0.3717	2.33
2	rs149823722	219411735	A	0.007732	0.003333	G	0.3717	2.33
2	rs146820102	239257480	A	0.007732	0.003333	G	0.3717	2.33
3	rs200787099	57131719	A	0.007732	0.003333	G	0.3717	2.33
3	rs117103025	113755549	G	0.007732	0.003333	A	0.3717	2.33
4	rs180792819	76452375	G	0.007732	0.003333	A	0.3717	2.33
4	rs2661805	173231379	G	0.3814	0.4083	A	0.3717	0.8935
4	rs141463006	186515050	A	0.007732	0.003333	C	0.3717	2.33
5	rs112632478	21802485	A	0.007732	0.003333	C	0.3717	2.33
5	rs76751505	40852977	A	0.007732	0.003333	G	0.3717	2.33
5	rs143336043	43508741	A	0.007732	0.003333	G	0.3717	2.33
5	rs201914751	68715804	A	0.007732	0.003333	G	0.3717	2.33
5	rs140434940	96076976	C	0.007732	0.003333	A	0.3717	2.33
5	rs151300194	108714529	A	0.007732	0.003333	G	0.3717	2.33
5	rs118080183	132197363	G	0.007732	0.003333	C	0.3717	2.33
5	rs200451693	141337254	C	0.007732	0.003333	G	0.3717	2.33
5	rs149015090	154282717	A	0.007732	0.003333	G	0.3717	2.33
5	rs17551608	167835539	A	0.007732	0.003333	G	0.3717	2.33

6	rs202152797	348155	A	0.007732	0.003333	G	0.3717	2.33
6	rs149410050	31929115	C	0.007732	0.003333	G	0.3717	2.33
6	rs182024970	73934044	G	0.007732	0.003333	A	0.3717	2.33
6	rs140603319	117128107	G	0.007732	0.003333	A	0.3717	2.33
6	rs140583847	130476007	G	0.007732	0.003333	A	0.3717	2.33
6	rs189384233	133035012	A	0.007732	0.003333	G	0.3717	2.33
7	rs149937937	2962364	G	0.007732	0.003333	A	0.3717	2.33
7	rs140427575	7412716	G	0.007732	0.003333	A	0.3717	2.33
7	exm2258357	45743321	A	0.007732	0.003333	G	0.3717	2.33
7	rs61735492	45743321	A	0.007732	0.003333	G	0.3717	2.33
7	rs121908362	107350577	G	0.007732	0.003333	A	0.3717	2.33
7	rs76521701	111644163	A	0.007732	0.003333	G	0.3717	2.33
7	rs104894031	128415610	A	0.007732	0.003333	G	0.3717	2.33
7	rs199910387	142564807	G	0.007732	0.003333	C	0.3717	2.33
8	rs117348493	41790969	A	0.007732	0.003333	C	0.3717	2.33
8	rs115580154	42275346	A	0.007732	0.003333	T	0.3717	2.33
8	rs149857905	42837779	A	0.007732	0.003333	G	0.3717	2.33
8	rs202001245	43002177	A	0.007732	0.003333	G	0.3717	2.33
8	rs139920573	77764484	G	0.007732	0.003333	A	0.3717	2.33
9	rs148372841	5892552	G	0.007732	0.003333	C	0.3717	2.33
9	rs143643420	17298289	A	0.007732	0.003333	G	0.3717	2.33
9	rs141121202	35093077	A	0.007732	0.003333	G	0.3717	2.33
9	rs144396411	71833267	A	0.007732	0.003333	G	0.3717	2.33
9	rs142380496	72833310	G	0.007732	0.003333	A	0.3717	2.33
9	rs200261885	117838677	A	0.007732	0.003333	G	0.3717	2.33
9	rs118203357	135801087	A	0.007732	0.003333	G	0.3717	2.33
9	rs200735402	139265120	A	0.007732	0.003333	G	0.3717	2.33
10	rs117525057	33502357	A	0.007732	0.003333	G	0.3717	2.33
10	rs144879420	127520029	A	0.007732	0.003333	G	0.3717	2.33
11	rs199775374	57886450	A	0.007732	0.003333	C	0.3717	2.33
11	rs199632362	58920603	A	0.007732	0.003333	T	0.3717	2.33
11	rs149751735	118404150	A	0.007732	0.003333	G	0.3717	2.33
12	rs202054008	2022196	A	0.007732	0.003333	G	0.3717	2.33
12	rs180999307	8687371	G	0.007732	0.003333	A	0.3717	2.33
12	rs138533375	51773409	A	0.007732	0.003333	G	0.3717	2.33
12	rs147424169	70963641	A	0.007732	0.003333	C	0.3717	2.33
12	rs75373025	123022996	G	0.007732	0.003333	A	0.3717	2.33
12	rs145597018	124341723	A	0.007732	0.003333	G	0.3717	2.33
12	rs145509540	133351809	A	0.007732	0.003333	G	0.3717	2.33
13	rs150309559	23907106	G	0.007732	0.003333	A	0.3717	2.33
13	rs200061738	42793364	G	0.007732	0.003333	A	0.3717	2.33
13	rs200574898	114150029	C	0.007732	0.003333	G	0.3717	2.33
14	rs142233028	20502389	T	0.007732	0.003333	A	0.3717	2.33

15	rs8034735	42162157	G	0.007732	0.003333	A	0.3717	2.33
15	rs192268080	43017376	A	0.007732	0.003333	G	0.3717	2.33
15	rs75737947	50168665	G	0.007732	0.003333	A	0.3717	2.33
15	rs28399427	75014921	G	0.007732	0.003333	C	0.3717	2.33
16	rs150670530	460365	A	0.007732	0.003333	G	0.3717	2.33
16	rs199784503	9858774	G	0.007732	0.003333	A	0.3717	2.33
16	rs114811657	14341011	A	0.007732	0.003333	G	0.3717	2.33
16	rs138480762	22126672	A	0.007732	0.003333	G	0.3717	2.33
16	rs144797498	28513376	A	0.007732	0.003333	C	0.3717	2.33
16	rs116093741	68846047	G	0.007732	0.003333	A	0.3717	2.33
16	rs1805007	89986117	A	0.007732	0.003333	G	0.3717	2.33
17	rs142095753	4058111	G	0.007732	0.003333	A	0.3717	2.33
17	rs190479497	27011891	G	0.007732	0.003333	A	0.3717	2.33
17	rs56026142	33445587	A	0.007732	0.003333	G	0.3717	2.33
17	rs117180332	43922922	A	0.007732	0.003333	G	0.3717	2.33
17	rs200264312	46628142	A	0.007732	0.003333	G	0.3717	2.33
17	rs148765149	63532439	A	0.007732	0.003333	G	0.3717	2.33
17	rs200588963	77918853	A	0.007732	0.003333	G	0.3717	2.33
19	rs184642529	6535064	A	0.007732	0.003333	G	0.3717	2.33
19	rs141998898	14829284	G	0.007732	0.003333	A	0.3717	2.33
19	rs148810844	58352661	T	0.007732	0.003333	A	0.3717	2.33
20	rs191392262	33588858	C	0.007732	0.003333	A	0.3717	2.33
20	rs142613680	44352696	A	0.007732	0.003333	G	0.3717	2.33
21	rs202170105	47863848	A	0.007732	0.003333	G	0.3717	2.33
22	rs76452330	19950329	A	0.007732	0.003333	G	0.3717	2.33
22	rs141822648	21384206	G	0.007732	0.003333	A	0.3717	2.33
22	rs200651736	24483436	A	0.007732	0.003333	G	0.3717	2.33
22	rs199774854	24581769	C	0.007732	0.003333	G	0.3717	2.33
22	rs6008794	46787694	G	0.007732	0.003333	A	0.3717	2.33
1	rs35296149	99156634	A	0.4201	0.3942	T	0.3718	1.113
7	rs10949838	73810096	A	0.4227	0.3958	G	0.3718	1.117
1	rs185769891	39827080	A	0.007732	0.003339	G	0.3719	2.326
15	rs200023487	55485030	G	0.007772	0.003356	A	0.3719	2.326
16	rs75025148	709271	A	0.007732	0.003344	G	0.3721	2.322
8	rs446227	55541450	A	0.384	0.4108	G	0.3722	0.894
14	rs7158003	46919042	G	0.384	0.4107	A	0.3722	0.8946
1	rs117890305	34554603	G	0.03093	0.04333	C	0.3723	0.7046
1	rs2819770	237911004	A	0.03093	0.04333	G	0.3723	0.7046
4	rs7664238	29755511	A	0.2552	0.2317	C	0.3723	1.136
4	rs79921527	76525532	A	0.03093	0.04333	G	0.3723	0.7046
5	rs1966265	176516631	G	0.4227	0.3967	A	0.3723	1.114
6	rs3132958	32297901	A	0.03093	0.04333	G	0.3723	0.7046
6	rs3132959	32298942	G	0.03093	0.04333	A	0.3723	0.7046

6	rs2182659	33751767	A	0.03093	0.04333	G	0.3723	0.7046
6	rs751727	33764158	A	0.03093	0.04333	G	0.3723	0.7046
7	rs35039803	94294982	A	0.03093	0.04333	G	0.3723	0.7046
10	rs7916697	69991853	A	0.4253	0.3983	G	0.3723	1.118
11	rs55909156	13032511	C	0.03093	0.04333	G	0.3723	0.7046
15	rs3759880	45048651	A	0.03093	0.04333	G	0.3723	0.7046
16	rs59025904	20432605	G	0.03093	0.04333	A	0.3723	0.7046
21	rs2070430	47982363	G	0.4227	0.3967	A	0.3723	1.114
14	rs62619918	20248768	G	0.007732	0.00335	C	0.3724	2.318
4	rs13133548	89740128	G	0.2809	0.3058	A	0.3725	0.8868
7	rs12531488	144892086	A	0.1675	0.1483	G	0.3725	1.155
8	rs3808564	26438546	A	0.2809	0.3067	G	0.3725	0.8833
9	rs10977204	8561542	G	0.2809	0.3067	A	0.3725	0.8833
12	rs7135131	75144471	G	0.2809	0.3058	A	0.3725	0.8868
16	rs17684500	10783113	G	0.1675	0.1483	A	0.3725	1.155
18	rs1791235	29126670	A	0.2809	0.3067	G	0.3725	0.8833
19	rs3764633	33439187	A	0.1675	0.1483	G	0.3725	1.155
1	rs200774891	17296833	A	0.007732	0.003356	G	0.3726	2.314
14	rs12878420	52336682	A	0.4253	0.3992	C	0.3727	1.114
1	rs12408481	84112571	A	0.06443	0.0525	G	0.3728	1.243
1	rs3748665	222713621	A	0.06443	0.0525	G	0.3728	1.243
3	rs7621875	82789651	G	0.06443	0.0525	A	0.3728	1.243
4	rs2272739	4276475	A	0.1082	0.1267	C	0.3728	0.8369
8	rs1037934	14256574	A	0.1082	0.1267	G	0.3728	0.8369
10	rs34587013	88696622	G	0.06443	0.0525	C	0.3728	1.243
22	rs1892722	25145471	A	0.317	0.2933	G	0.3728	1.118
3	rs2227294	46902129	C	0.3892	0.4165	A	0.373	0.8925
1	rs11558606	230814668	G	0.08505	0.07083	A	0.3731	1.219
2	rs150129946	200790050	G	0.08505	0.07083	A	0.3731	1.219
3	rs9847153	147359950	C	0.3866	0.4125	A	0.3731	0.8976
5	rs6182	42718928	A	0.08505	0.07083	C	0.3731	1.219
5	rs6184	42719344	A	0.08505	0.07083	C	0.3731	1.219
8	rs7832021	33462084	A	0.1082	0.1258	G	0.3731	0.8433
10	rs2228528	50732280	A	0.3866	0.4125	G	0.3731	0.8976
11	rs74359608	9225326	G	0.08505	0.07083	A	0.3731	1.219
16	rs78344060	84227713	A	0.08505	0.07083	G	0.3731	1.219
5	rs6861942	77812808	A	0.4304	0.4033	G	0.3732	1.118
10	rs2398245	10570880	G	0.4304	0.4033	A	0.3732	1.118
11	rs2276122	117965530	G	0.3892	0.4158	A	0.3732	0.895
2	rs12467509	146913332	C	0.2062	0.1858	A	0.3734	1.138
4	rs7690457	17805379	A	0.1082	0.0925	G	0.3734	1.191
17	rs220479	3657175	G	0.3918	0.4189	A	0.3734	0.8935
6	rs9267873	32199352	G	0.1366	0.1183	A	0.3735	1.179

6	rs61740334	170047902	A	0.1366	0.1183	T	0.3735	1.179
11	rs2303973	14264916	G	0.2835	0.3092	A	0.3736	0.8842
19	rs146395464	55889178	A	0.007732	0.003378	G	0.3736	2.299
1	rs96067	36571920	A	0.3892	0.415	G	0.3737	0.8981
1	rs771205	150975108	A	0.1753	0.1967	G	0.3737	0.868
5	rs10475878	167790204	G	0.3918	0.4182	A	0.3737	0.896
11	rs11212024	106947600	A	0.1753	0.1975	G	0.3737	0.8634
12	rs11066587	113979656	C	0.1753	0.1967	G	0.3737	0.868
13	rs5009567	85394807	A	0.3918	0.4183	G	0.3737	0.8955
16	rs6420424	81242102	G	0.1753	0.1975	A	0.3737	0.8634
16	rs6564838	81242194	A	0.1753	0.1975	G	0.3737	0.8634
19	rs11538683	991129	A	0.1753	0.1975	G	0.3737	0.8634
11	rs10767971	32895664	G	0.433	0.4067	A	0.3739	1.114
16	rs9935059	22237118	G	0.3196	0.2958	A	0.3739	1.118
19	rs3810206	17450016	G	0.2216	0.245	A	0.3739	0.8776
6	rs2523608	31322559	G	0.3943	0.4217	A	0.374	0.893
12	rs2731032	45477074	G	0.2216	0.2458	A	0.374	0.8736
1	rs61743209	7797534	A	0.0232	0.01583	G	0.3741	1.476
1	rs3811028	157545384	G	0.0232	0.01583	A	0.3741	1.476
2	rs145137974	42580450	A	0.0232	0.01583	C	0.3741	1.476
2	rs148723232	68521040	G	0.0232	0.01583	A	0.3741	1.476
2	rs76979001	215880278	A	0.0232	0.01583	G	0.3741	1.476
5	rs140981366	94818236	A	0.0232	0.01583	G	0.3741	1.476
6	rs2256266	29632318	A	0.3222	0.2975	G	0.3741	1.122
6	rs2857766	29634003	G	0.3222	0.2975	C	0.3741	1.122
6	rs9276601	32734304	G	0.0232	0.01583	A	0.3741	1.476
6	rs4435957	135307309	C	0.0232	0.01583	G	0.3741	1.476
8	rs200832762	121290743	G	0.0232	0.01583	A	0.3741	1.476
10	rs117992169	105184858	A	0.0232	0.01583	G	0.3741	1.476
12	rs117275340	40013392	C	0.0232	0.01583	G	0.3741	1.476
13	rs148908916	76432070	A	0.0232	0.01583	G	0.3741	1.476
14	rs187901859	24730946	A	0.0232	0.01583	G	0.3741	1.476
14	rs138067674	64935983	A	0.0232	0.01583	G	0.3741	1.476
15	rs7164132	40675155	A	0.0232	0.01583	C	0.3741	1.476
15	rs7169404	40675443	A	0.0232	0.01583	C	0.3741	1.476
15	rs7169262	40675493	A	0.0232	0.01583	G	0.3741	1.476
18	rs59422275	43534824	G	0.0232	0.01583	A	0.3741	1.476
18	rs141888762	52596055	G	0.0232	0.01583	A	0.3741	1.476
19	rs138021555	9453161	A	0.0232	0.01583	G	0.3741	1.476
19	rs2302411	44501145	G	0.0232	0.01583	A	0.3741	1.476
19	rs146000520	51840579	A	0.0232	0.01583	G	0.3741	1.476
3	rs6444895	169801658	G	0.3943	0.4208	A	0.3742	0.896
5	rs72635619	79026370	C	0.2577	0.2346	A	0.3742	1.133

17	rs34293811	76060866	C	0.2216	0.2442	G	0.3742	0.8815
22	rs36082900	45132687	A	0.1392	0.1592	G	0.3742	0.8541
3	rs6796037	67657497	A	0.4356	0.4092	C	0.3743	1.114
18	rs2445439	48870202	G	0.4356	0.4092	A	0.3743	1.114
10	rs41308663	95276742	C	0.1675	0.1492	G	0.3744	1.148
13	rs2329188	82104522	G	0.1675	0.1492	A	0.3744	1.148
17	rs61738821	79096115	A	0.0232	0.01586	G	0.3744	1.474
1	rs945508	156907081	A	0.1392	0.16	G	0.3746	0.8488
6	rs9478712	156858484	A	0.3969	0.4233	G	0.3746	0.8965
7	rs10488360	4411209	A	0.3969	0.4233	G	0.3746	0.8965
7	rs213950	117199533	A	0.3969	0.4233	G	0.3746	0.8965
10	rs10883100	100219374	A	0.433	0.4075	T	0.3746	1.11
17	rs56129846	74932934	A	0.4381	0.4117	C	0.3747	1.114
2	rs7567607	43638185	A	0.2861	0.3117	G	0.3748	0.885
20	rs6013382	50702633	A	0.2861	0.3117	G	0.3748	0.885
1	rs6687840	159342439	A	0.3222	0.2983	G	0.3749	1.118
2	rs673951	220473355	G	0.2861	0.3108	A	0.3749	0.8885
15	rs10520045	36313965	A	0.2861	0.3108	C	0.3749	0.8885
21	rs2836754	40291740	G	0.2861	0.3108	A	0.3749	0.8885
3	rs6794	45052775	G	0.1366	0.1192	C	0.375	1.169
2	rs2303920	10126330	A	0.3247	0.3	G	0.3751	1.122
10	rs10761725	64974537	A	0.3995	0.4258	T	0.3751	0.897
11	rs365605	2920666	A	0.3995	0.4258	G	0.3751	0.897
16	rs4889071	80071458	G	0.4407	0.4142	A	0.3751	1.115
13	rs4143768	23911820	G	0.4433	0.4165	A	0.3753	1.115
13	rs11556093	25876011	G	0.4381	0.4125	A	0.3753	1.111
15	rs7175404	94036688	G	0.4021	0.4292	A	0.3753	0.8944
16	rs10514585	83284338	A	0.4381	0.4125	G	0.3753	1.111
20	rs200881	1771233	G	0.4381	0.4125	A	0.3753	1.111
20	rs5743523	36954724	A	0.4381	0.4125	G	0.3753	1.111
10	rs4747574	7755623	A	0.4433	0.4167	C	0.3754	1.115
12	rs2228500	48526712	A	0.2242	0.2475	G	0.3756	0.8788
1	rs2989946	227178948	G	0.2242	0.2483	A	0.3757	0.8749
5	rs6452051	22109314	G	0.4407	0.415	A	0.3757	1.111
9	rs10989589	104433235	A	0.2088	0.1883	G	0.3757	1.137
10	rs6584859	109894731	A	0.2088	0.1883	G	0.3757	1.137
11	rs4622263	22173372	G	0.2242	0.2483	A	0.3757	0.8749
16	rs2075520	21222672	A	0.4407	0.415	C	0.3757	1.111
18	rs7234309	10752666	A	0.4046	0.4317	G	0.3757	0.8948
5	rs72635618	79025085	G	0.2577	0.2358	A	0.3759	1.125
10	rs6480855	79026227	A	0.4485	0.4215	G	0.376	1.116
20	rs6090457	62200576	G	0.4072	0.4342	A	0.376	0.8953
7	rs6975024	44231886	G	0.1778	0.2	A	0.3761	0.8652

10	rs7349	31817905	A	0.1778	0.2	G	0.3761	0.8652
11	rs2155378	132274000	A	0.4485	0.4217	G	0.3761	1.115
15	rs2229116	33905410	G	0.1778	0.1992	A	0.3761	0.8697
3	rs11720982	17193348	A	0.3273	0.3025	C	0.3762	1.122
1	rs4970625	39221925	C	0.451	0.4242	A	0.3764	1.115
2	rs9678851	27887034	A	0.1082	0.09333	C	0.3764	1.179
2	rs260687	109578855	A	0.08505	0.1017	G	0.3764	0.8214
2	rs260690	109579738	A	0.08505	0.1017	C	0.3764	0.8214
5	rs10038113	25902342	A	0.4098	0.4367	G	0.3764	0.8957
7	rs17147735	100367573	G	0.04639	0.05917	A	0.3764	0.7736
7	rs77145942	112407371	A	0.04639	0.05917	G	0.3764	0.7736
8	rs20571	86351997	A	0.4098	0.4367	G	0.3764	0.8957
18	rs1574039	50253740	G	0.451	0.4242	A	0.3764	1.115
19	rs2075650	45395619	G	0.1082	0.09333	A	0.3764	1.179
19	rs75864168	56473458	A	0.04639	0.05917	G	0.3764	0.7736
1	rs2273285	12010469	A	0.08505	0.07167	C	0.3766	1.204
3	rs286588	6245005	A	0.4485	0.4225	G	0.3766	1.111
19	rs35413309	33167837	A	0.08505	0.07167	G	0.3766	1.204
2	rs3762568	207631461	A	0.04897	0.03833	G	0.3767	1.292
6	rs2230926	138196066	C	0.04639	0.06	A	0.3767	0.7622
7	rs196586	36320821	G	0.04639	0.06	A	0.3767	0.7622
9	rs11539737	139946061	G	0.04897	0.03833	A	0.3767	1.292
4	rs6846243	11053537	A	0.4536	0.4267	G	0.3768	1.116
6	rs399604	32975014	G	0.4536	0.4267	A	0.3768	1.116
2	rs4254535	69198388	G	0.3273	0.3033	A	0.377	1.118
6	rs9393728	26509330	G	0.2912	0.3167	C	0.377	0.8867
6	rs1003979	33114171	A	0.08505	0.1025	G	0.377	0.8139
7	rs6466460	113294567	A	0.3273	0.3033	G	0.377	1.118
12	rs60238091	121221518	A	0.08505	0.1025	G	0.377	0.8139
2	rs1564973	240423994	G	0.4149	0.4417	A	0.3771	0.8966
5	rs2645329	178421770	A	0.4149	0.4417	C	0.3771	0.8966
12	rs1609860	104048454	A	0.1108	0.1292	C	0.3771	0.8403
17	rs2272011	1542190	G	0.1108	0.1292	A	0.3771	0.8403
18	rs7239368	31645379	G	0.4149	0.4417	A	0.3771	0.8966
2	rs6747972	68070225	A	0.4124	0.4383	G	0.3772	0.8992
5	rs933688	90762748	G	0.4536	0.4275	A	0.3772	1.112
6	rs10502	90039670	G	0.2912	0.3158	C	0.3772	0.8901
11	rs7947230	8646670	C	0.2912	0.3158	A	0.3772	0.8901
11	rs7935453	8646763	C	0.2912	0.3158	G	0.3772	0.8901
11	rs2155174	87664122	A	0.4536	0.4275	G	0.3772	1.112
14	rs6571975	40388314	G	0.2912	0.3158	A	0.3772	0.8901
22	rs132852	45560641	A	0.4124	0.4383	G	0.3772	0.8992
1	rs4437851	68428049	G	0.1366	0.12	A	0.3774	1.16

2	rs1441234	41488391	A	0.4588	0.4317	G	0.3774	1.116
6	rs2756381	89360837	C	0.1366	0.12	A	0.3774	1.16
9	rs34155071	140327663	A	0.2268	0.2508	G	0.3774	0.8761
14	rs17792748	22022117	G	0.4588	0.4317	A	0.3774	1.116
14	rs1958686	38299266	A	0.1366	0.12	G	0.3774	1.16
4	rs1801058	3039150	G	0.4175	0.4442	A	0.3775	0.897
5	rs30688	128975230	A	0.4175	0.4442	G	0.3775	0.897
7	rs2158836	107580839	A	0.1701	0.1517	G	0.3775	1.146
11	rs2119510	70969719	A	0.4149	0.4408	G	0.3775	0.8996
18	rs8099090	71106985	A	0.4149	0.4408	G	0.3775	0.8996
21	rs2407582	21373901	G	0.1418	0.1617	A	0.3775	0.8565
4	rs4234717	6020190	G	0.4275	0.4542	A	0.3776	0.8973
6	rs2294478	33098966	C	0.1108	0.13	A	0.3776	0.8341
3	rs11719997	63560623	G	0.4588	0.4325	A	0.3777	1.112
11	rs10836265	34637669	A	0.4588	0.4325	G	0.3777	1.112
19	rs2967526	36904463	A	0.4201	0.4467	G	0.3778	0.8974
2	rs270979	161694980	G	0.4175	0.4433	A	0.3779	0.9001
3	rs6766692	195308171	G	0.4175	0.4433	A	0.3779	0.9001
7	rs74366495	19184732	A	0.2113	0.1908	C	0.3779	1.136
12	rs10963	7281317	A	0.4175	0.4433	G	0.3779	0.9001
12	rs10777317	91980374	A	0.4175	0.4433	G	0.3779	0.9001
12	rs4486692	91996056	A	0.4175	0.4433	C	0.3779	0.9001
14	rs11158685	68042574	G	0.4175	0.4433	A	0.3779	0.9001
15	rs1800414	28197037	A	0.4175	0.4433	G	0.3779	0.9001
17	rs7212298	11025242	G	0.2113	0.1908	A	0.3779	1.136
21	rs62223052	40708702	C	0.4175	0.4433	A	0.3779	0.9001
6	rs2157605	30454076	G	0.2629	0.24	A	0.378	1.129
10	rs791587	6088699	A	0.3299	0.3058	G	0.378	1.117
16	rs1054644	1270111	A	0.02062	0.03005	G	0.378	0.6795
19	rs4801635	56372705	G	0.3299	0.3058	A	0.378	1.117
20	rs910122	5903323	G	0.4227	0.45	A	0.378	0.8948
7	rs12704649	92674917	G	0.4227	0.4492	A	0.3781	0.8979
10	rs1227049	73434888	G	0.2938	0.3192	C	0.3781	0.8875
15	rs7496668	100821576	G	0.4227	0.4492	A	0.3781	0.8979
2	rs2258175	173686798	A	0.4253	0.4525	G	0.3782	0.8953
6	rs4713358	30716117	A	0.02062	0.03	C	0.3782	0.6807
7	rs10230913	79643444	A	0.4639	0.4375	G	0.3782	1.113
8	rs4388421	10474042	C	0.4253	0.4525	A	0.3782	0.8953
8	rs13273355	13424583	G	0.02062	0.03	A	0.3782	0.6807
13	rs148399850	52511626	A	0.02062	0.03	G	0.3782	0.6807
15	rs6493352	31234064	A	0.02062	0.03	G	0.3782	0.6807
19	rs116617171	36342568	A	0.02062	0.03	G	0.3782	0.6807
1	rs476157	108088171	G	0.2938	0.3183	A	0.3783	0.8909

3	rs6786229	177744450	G	0.3325	0.3075	A	0.3783	1.122
2	rs2167530	134393846	A	0.4253	0.4517	G	0.3784	0.8983
2	rs13015491	228398422	G	0.06443	0.08	A	0.3784	0.792
4	rs10856927	98596032	G	0.4253	0.4517	A	0.3784	0.8983
9	rs76334696	34514426	A	0.06443	0.08	C	0.3784	0.792
10	rs210238	43014780	A	0.301	0.3255	G	0.3784	0.8925
5	rs11750203	180597660	G	0.4278	0.455	A	0.3785	0.8957
1	rs6426749	22711473	G	0.1813	0.2025	C	0.3786	0.8724
6	rs417812	32974081	A	0.4613	0.4358	G	0.3786	1.109
12	rs6488674	14488914	C	0.4613	0.4358	A	0.3786	1.109
1	rs3738283	201458071	A	0.06701	0.05417	T	0.3787	1.254
3	rs61629992	130360527	A	0.06701	0.05417	G	0.3787	1.254
5	rs3812054	126732427	A	0.06701	0.05417	G	0.3787	1.254
8	rs16937689	72517499	A	0.4304	0.4575	G	0.3787	0.8961
3	rs59722850	113146130	A	0.4794	0.4527	G	0.3788	1.113
15	exm1150082	41186990	A	0.1108	0.095	G	0.3788	1.187
15	rs16971177	41186990	A	0.1108	0.095	G	0.3788	1.187
19	rs75444444	9006749	A	0.1108	0.095	G	0.3788	1.187
1	rs76529463	179051508	A	0.02062	0.03083	G	0.3789	0.6617
1	rs79881900	206325259	A	0.02062	0.03083	G	0.3789	0.6617
2	rs76384189	102031351	C	0.02062	0.03083	G	0.3789	0.6617
3	rs2276717	170201230	A	0.02062	0.03083	G	0.3789	0.6617
3	rs17129219	196735687	A	0.02062	0.03083	G	0.3789	0.6617
4	rs1803183	122735142	G	0.02062	0.03083	A	0.3789	0.6617
5	rs61744047	13708284	A	0.02062	0.03083	G	0.3789	0.6617
12	rs12424429	85266484	A	0.02062	0.03083	G	0.3789	0.6617
15	rs7171797	43527871	A	0.02062	0.03083	G	0.3789	0.6617
15	rs2044502	85163998	G	0.02062	0.03083	C	0.3789	0.6617
4	rs1024020	131137761	G	0.4304	0.4567	A	0.379	0.8991
6	rs6941356	87967636	G	0.1392	0.1217	A	0.379	1.167
7	rs1801368	1976457	A	0.4742	0.4474	G	0.379	1.114
8	rs1454292	4926465	G	0.3325	0.3083	A	0.379	1.117
10	rs3802526	27459746	G	0.2139	0.1925	C	0.379	1.142
10	rs2292584	50186415	A	0.2629	0.2408	G	0.379	1.124
12	rs6539524	80935345	G	0.2294	0.2533	A	0.379	0.8773
13	rs1372000	73369642	G	0.2629	0.2408	A	0.379	1.124
16	rs12929546	20981162	G	0.2139	0.1925	A	0.379	1.142
19	rs2228137	7763248	A	0.06701	0.05426	G	0.379	1.252
20	rs146578812	61467883	A	0.01295	0.008333	G	0.379	1.562
1	rs2275477	36886117	A	0.2294	0.2525	G	0.3791	0.8812
1	rs34409118	36898067	G	0.2294	0.2525	A	0.3791	0.8812
7	rs7796495	43267757	A	0.2964	0.3217	G	0.3791	0.8883
14	rs862034	74990746	A	0.2294	0.2525	G	0.3791	0.8812

2	rs1549579	160668661	C	0.4356	0.4625	A	0.3792	0.8968
2	rs7605378	200676926	C	0.433	0.4591	A	0.3792	0.8997
5	rs11750538	16769273	G	0.4356	0.4625	A	0.3792	0.8968
6	rs6910017	84971363	A	0.4691	0.4433	G	0.3792	1.109
17	rs9303277	37976469	A	0.3351	0.31	G	0.3793	1.122
2	rs4664325	161281691	G	0.2964	0.3208	A	0.3794	0.8917
10	rs7097617	77504287	G	0.4716	0.4458	A	0.3794	1.11
12	rs11114086	109286399	A	0.4716	0.4458	G	0.3794	1.11
5	rs11959574	32286948	A	0.4381	0.465	G	0.3795	0.8972
1	rs6698109	185171869	G	0.2655	0.2425	A	0.3796	1.129
9	rs3739881	34724241	C	0.482	0.455	A	0.3796	1.114
10	rs10904392	5068113	C	0.4356	0.4617	A	0.3796	0.8998
10	rs1780179	26997889	A	0.4356	0.4617	G	0.3796	0.8998
10	rs241	78444456	C	0.482	0.455	A	0.3796	1.114
6	rs2284178	31432125	G	0.4585	0.4858	A	0.3797	0.8963
12	rs269806	65393186	A	0.4768	0.4508	G	0.3797	1.11
18	rs7239022	6646853	A	0.4768	0.4508	G	0.3797	1.11
18	rs7234352	60739233	G	0.4407	0.4675	A	0.3797	0.8976
10	rs4406737	90759724	A	0.4381	0.4642	G	0.3798	0.9002
4	rs4864124	133529403	G	0.3351	0.3108	A	0.3799	1.117
5	rs7714485	92515301	A	0.3351	0.3108	C	0.3799	1.117
6	rs3117016	33095516	A	0.4794	0.4533	G	0.3799	1.11
1	rs9427726	202034271	G	0.482	0.4558	A	0.38	1.111
1	rs2306120	226352498	C	0.482	0.4558	A	0.38	1.111
2	rs6715339	92027973	A	0.482	0.4558	G	0.38	1.111
6	rs1265095	31106643	G	0.4459	0.4725	A	0.38	0.8985
17	rs1714987	35743010	C	0.482	0.4558	G	0.38	1.111
6	rs4706636	77566201	A	0.4459	0.4725	G	0.3801	0.8983
9	rs6475273	18950787	G	0.4459	0.4725	A	0.3801	0.8983
11	rs3740955	36595600	A	0.2139	0.1933	G	0.3801	1.135
19	rs3745974	33698291	G	0.4897	0.4625	A	0.3801	1.115
22	rs11090122	43308475	G	0.2139	0.1933	A	0.3801	1.135
2	rs289872	152065857	A	0.08763	0.07333	G	0.3803	1.214
11	rs118160252	117988105	A	0.08763	0.07333	G	0.3803	1.214
13	rs9509086	20767957	C	0.4433	0.4692	A	0.3803	0.901
16	rs16971436	72992763	C	0.08763	0.07333	A	0.3803	1.214
18	rs11081909	33083546	G	0.4871	0.4608	A	0.3803	1.111
18	rs28564177	72866264	G	0.4485	0.475	A	0.3803	0.8987
19	rs1044250	8436164	A	0.08763	0.07333	G	0.3803	1.214
21	rs228104	43985955	A	0.4485	0.475	G	0.3803	0.8987
1	rs12402973	9074861	A	0.1727	0.1542	G	0.3804	1.145
5	rs4551093	162652827	A	0.451	0.4775	G	0.3804	0.899
8	rs11989122	118827839	A	0.451	0.4775	G	0.3804	0.899

9	rs4606122	74186310	G	0.451	0.4775	A	0.3804	0.899
16	rs3104767	52624738	C	0.1727	0.1542	A	0.3804	1.145
19	rs58237134	4294623	A	0.1727	0.1542	C	0.3804	1.145
5	rs1910795	26809915	G	0.4562	0.4833	A	0.3805	0.8967
8	rs4260880	69020496	G	0.4923	0.4658	A	0.3805	1.112
10	rs1242976	9238010	G	0.4923	0.4658	A	0.3805	1.112
2	rs1045910	55771161	A	0.4536	0.48	G	0.3806	0.8994
2	rs7577925	134040519	A	0.2655	0.2433	G	0.3806	1.124
2	rs7575052	222618435	G	0.1443	0.1642	A	0.3806	0.8588
3	rs9289122	118867047	C	0.4536	0.48	G	0.3806	0.8994
13	rs716655	75629027	G	0.4588	0.4858	A	0.3806	0.8971
18	rs1943392	54976972	G	0.4948	0.4683	A	0.3806	1.112
3	rs1676232	117234839	A	0.232	0.2558	G	0.3807	0.8785
4	rs6819167	175871449	A	0.5	0.4733	G	0.3807	1.113
17	rs17690195	10219113	A	0.183	0.2045	G	0.3807	0.8712
20	rs4809959	52785859	G	0.4613	0.4883	A	0.3807	0.8974
1	rs200213253	909006	A	0.01289	0.008333	G	0.3808	1.554
1	rs115823881	6631157	G	0.01289	0.008333	A	0.3808	1.554
1	rs61746706	6634901	C	0.01289	0.008333	A	0.3808	1.554
1	rs114597256	6636662	G	0.01289	0.008333	A	0.3808	1.554
1	rs181142071	18152977	G	0.01289	0.008333	A	0.3808	1.554
1	rs151081875	21054485	A	0.01289	0.008333	C	0.3808	1.554
1	rs77101686	46874246	A	0.01289	0.008333	G	0.3808	1.554
1	rs77724956	46877284	C	0.01289	0.008333	A	0.3808	1.554
1	rs183838419	51860059	A	0.01289	0.008333	G	0.3808	1.554
1	rs76465406	53980431	A	0.01289	0.008333	G	0.3808	1.554
1	rs143080179	154227639	G	0.01289	0.008333	A	0.3808	1.554
1	rs3737521	158653225	A	0.01289	0.008333	G	0.3808	1.554
2	rs7577483	29274730	A	0.1443	0.165	C	0.3808	0.8536
2	rs138062318	64779452	A	0.01289	0.008333	G	0.3808	1.554
2	rs2302620	102842467	G	0.183	0.205	A	0.3808	0.8686
2	rs2272058	120388412	G	0.01289	0.008333	A	0.3808	1.554
2	rs17304212	179325735	G	0.01289	0.008333	C	0.3808	1.554
2	rs141615048	180815329	A	0.01289	0.008333	G	0.3808	1.554
2	rs181295117	218712674	A	0.01289	0.008333	T	0.3808	1.554
3	rs142408226	9908888	A	0.01289	0.008333	G	0.3808	1.554
3	rs2240859	42787469	G	0.4485	0.4742	A	0.3808	0.9017
4	rs138982106	40127852	G	0.01289	0.008333	A	0.3808	1.554
4	rs28416473	53731802	A	0.01289	0.008333	G	0.3808	1.554
4	rs201164541	70823320	A	0.01289	0.008333	G	0.3808	1.554
5	rs77036027	35779419	A	0.01289	0.008333	G	0.3808	1.554
5	rs183910847	43175096	A	0.01289	0.008333	T	0.3808	1.554
5	rs148902887	54442491	T	0.01289	0.008333	A	0.3808	1.554

5	rs3815895	74970024	G	0.4562	0.4825	A	0.3808	0.8997
5	rs184693277	168093595	A	0.01289	0.008333	G	0.3808	1.554
7	rs149662771	20782621	G	0.01289	0.008333	A	0.3808	1.554
8	rs114868912	11614584	A	0.01289	0.008333	G	0.3808	1.554
8	rs115099192	11615875	A	0.01289	0.008333	C	0.3808	1.554
9	rs186662317	13176152	A	0.01289	0.008333	G	0.3808	1.554
9	rs146710785	21077640	A	0.01289	0.008333	G	0.3808	1.554
9	rs200165112	33295206	A	0.01289	0.008333	G	0.3808	1.554
9	rs116922187	74365261	A	0.01289	0.008333	C	0.3808	1.554
10	rs117017856	105762945	G	0.01289	0.008333	A	0.3808	1.554
11	rs17884773	64977890	A	0.01289	0.008333	G	0.3808	1.554
11	rs1712790	114621469	G	0.4639	0.4908	A	0.3808	0.8977
13	rs200595109	42876668	C	0.01289	0.008333	A	0.3808	1.554
14	rs151124111	24029155	A	0.01289	0.008333	G	0.3808	1.554
14	rs28756992	75513826	G	0.01289	0.008333	A	0.3808	1.554
14	rs28756990	75514138	A	0.01289	0.008333	C	0.3808	1.554
14	rs202149884	94756542	C	0.01289	0.008333	A	0.3808	1.554
15	rs2458236	45446156	G	0.4639	0.4908	A	0.3808	0.8977
15	rs2445751	51696003	G	0.4639	0.4908	A	0.3808	0.8977
15	rs146162352	51993318	G	0.01289	0.008333	A	0.3808	1.554
15	rs147946196	80454614	A	0.01289	0.008333	G	0.3808	1.554
16	rs150910102	2347859	C	0.01289	0.008333	G	0.3808	1.554
17	rs200040856	73914244	A	0.01289	0.008333	G	0.3808	1.554
18	rs1840440	23257186	G	0.5026	0.4758	A	0.3808	1.113
18	rs149617776	29126558	A	0.01289	0.008333	G	0.3808	1.554
18	rs11082414	42529996	G	0.01289	0.008333	C	0.3808	1.554
18	rs199848449	50683844	C	0.01289	0.008333	A	0.3808	1.554
22	rs142214633	17447138	C	0.01289	0.008333	G	0.3808	1.554
1	rs4072677	206879122	C	0.3376	0.3133	A	0.3809	1.117
3	rs1449444	145790803	C	0.4588	0.485	A	0.3809	0.9
6	rs9342637	62759028	A	0.183	0.2042	C	0.3809	0.873
7	rs4326334	52996013	G	0.183	0.2042	A	0.3809	0.873
13	rs9567771	31587270	C	0.5052	0.4783	A	0.3809	1.113
14	rs7144509	53023607	A	0.5077	0.4808	G	0.3809	1.114
14	rs12892024	91450698	G	0.5052	0.4783	A	0.3809	1.113
15	rs6495451	80164053	G	0.4665	0.4933	A	0.3809	0.898
16	rs9921812	81181821	G	0.1451	0.1644	A	0.3809	0.8623
17	rs7209812	27536988	A	0.5052	0.4783	G	0.3809	1.113
17	rs9896104	30011493	A	0.3376	0.3133	C	0.3809	1.117
1	rs6671166	154093825	A	0.5129	0.4858	G	0.381	1.114
1	rs7553207	218370200	A	0.4691	0.4958	G	0.381	0.8983
2	rs11687765	82325531	C	0.4948	0.4691	A	0.381	1.109
4	rs6844095	59630002	A	0.451	0.4767	G	0.381	0.902

8	rs11167136	143310815	G	0.518	0.4908	A	0.381	1.115
11	rs10128713	26770926	C	0.451	0.4767	A	0.381	0.902
12	rs10745488	77771135	A	0.451	0.4767	G	0.381	0.902
19	rs2288419	55693244	G	0.451	0.4767	A	0.381	0.902
21	rs9305406	31386243	A	0.518	0.4908	G	0.381	1.115
1	rs2294740	170928671	G	0.4613	0.4875	A	0.3811	0.9004
3	rs4682576	110083511	G	0.4742	0.5008	A	0.3811	0.899
10	rs7087634	118171016	G	0.4716	0.4983	A	0.3811	0.8987
1	rs33943686	169951997	G	0.5	0.4742	A	0.3812	1.109
3	rs184502	168196092	G	0.2165	0.195	A	0.3812	1.141
7	rs7793970	70736168	G	0.4794	0.5058	A	0.3812	0.8996
8	rs1128397	21550800	A	0.4639	0.49	T	0.3812	0.9007
8	rs1124	22021517	A	0.3015	0.3267	G	0.3812	0.8899
9	rs10491654	102139527	G	0.3015	0.3267	A	0.3812	0.8899
12	rs10777027	87723347	A	0.4948	0.4692	G	0.3812	1.108
12	rs17852561	122396395	A	0.4948	0.4692	G	0.3812	1.108
15	rs11071428	59614841	A	0.2165	0.195	G	0.3812	1.141
20	rs4815025	21142523	C	0.4974	0.4717	G	0.3812	1.109
21	rs2837225	41165462	A	0.5	0.4742	C	0.3812	1.109
1	rs709682	19565338	A	0.1392	0.1225	G	0.3813	1.158
1	rs360645	115427218	G	0.5155	0.4892	A	0.3813	1.111
4	rs28385534	18736797	G	0.5103	0.4842	A	0.3813	1.11
5	rs13155377	157528319	A	0.5103	0.4842	C	0.3813	1.11
6	rs1884478	85588632	G	0.5129	0.4867	A	0.3813	1.111
7	rs4532523	153970767	G	0.5103	0.4842	A	0.3813	1.11
9	rs10757049	19281501	A	0.518	0.4917	G	0.3813	1.111
10	rs740363	118575606	A	0.1134	0.1317	G	0.3813	0.8435
11	rs1139971	44640268	A	0.5026	0.4767	G	0.3813	1.109
11	rs658006	64664774	G	0.5155	0.4892	A	0.3813	1.111
11	rs1052030	76853783	A	0.5052	0.4792	G	0.3813	1.11
12	rs7133914	40702911	A	0.1392	0.1225	G	0.3813	1.158
12	rs1057190	44148259	G	0.1392	0.1225	A	0.3813	1.158
14	rs3784116	68851441	G	0.5155	0.4892	A	0.3813	1.111
15	rs8031046	81625028	A	0.1134	0.1317	G	0.3813	0.8435
22	rs165815	19959473	A	0.518	0.4917	G	0.3813	1.111
7	rs1029396	117024820	C	0.4691	0.495	A	0.3814	0.9013
12	rs4402352	108323947	A	0.3015	0.3258	C	0.3815	0.8933
13	rs211252	33584957	A	0.4716	0.4975	G	0.3815	0.9017
15	rs899609	94570578	G	0.3015	0.3258	A	0.3815	0.8933
19	rs3826942	1360575	A	0.4716	0.4975	G	0.3815	0.9017
1	rs1138294	27688633	G	0.4742	0.5	A	0.3816	0.902
2	rs3739040	238672290	A	0.1108	0.09583	G	0.3816	1.176
2	rs3739039	238672691	G	0.1108	0.09583	A	0.3816	1.176

3	rs61751634	25646268	A	0.1753	0.1558	G	0.3817	1.151
6	rs9493698	134013272	G	0.1753	0.1558	A	0.3817	1.151
9	rs2297105	37020622	C	0.4768	0.5025	A	0.3817	0.9023
15	rs2730081	84327771	G	0.4768	0.5025	A	0.3817	0.9023
17	rs1136287	1673276	A	0.4768	0.5025	G	0.3817	0.9023
19	rs3752233	1045173	A	0.1753	0.1558	G	0.3817	1.151
6	rs1046943	109783941	G	0.4794	0.505	A	0.3818	0.9026
3	rs6801634	4354697	A	0.08763	0.1042	G	0.3819	0.826
17	rs202232792	4926868	A	0.01289	0.008361	G	0.382	1.548
19	rs151250046	7811372	A	0.01289	0.008361	G	0.382	1.548
1	rs7524907	81867479	G	0.268	0.2458	A	0.3821	1.123
8	rs1047266	22900701	A	0.2345	0.2583	G	0.3823	0.8797
11	rs12291120	80132486	A	0.2165	0.1958	G	0.3823	1.135
15	rs1037575	35740582	G	0.2345	0.2575	A	0.3824	0.8835
4	rs78205702	113469473	A	0.06701	0.055	G	0.3826	1.234
7	rs1527263	76991935	A	0.06701	0.055	G	0.3826	1.234
8	rs6474359	41549194	G	0.06701	0.055	A	0.3826	1.234
11	rs76301014	123676388	A	0.06701	0.055	G	0.3826	1.234
15	rs59550885	43571958	A	0.06701	0.055	C	0.3826	1.234
6	rs3130655	30823710	T	0.3428	0.3183	A	0.3828	1.117
6	rs3095354	30836111	G	0.3428	0.3183	A	0.3828	1.117
6	rs3130796	30840950	A	0.3428	0.3183	G	0.3828	1.117
6	rs7754788	131505531	G	0.3428	0.3183	A	0.3828	1.117
8	rs1134975	17412140	C	0.3428	0.3183	G	0.3828	1.117
9	rs1112863	123122187	G	0.3428	0.3183	A	0.3828	1.117
2	rs1260327	27711893	G	0.1418	0.1242	A	0.383	1.165
1	rs77387570	145561110	A	0.03608	0.02667	C	0.3831	1.366
5	rs184318860	132200108	C	0.03608	0.02667	A	0.3831	1.366
7	rs55976638	149529970	A	0.03608	0.02667	C	0.3831	1.366
8	rs117189614	2024280	A	0.03608	0.02667	G	0.3831	1.366
10	rs11594560	128974220	A	0.03608	0.02667	G	0.3831	1.366
2	rs1126497	47601106	A	0.1856	0.2067	G	0.3832	0.8746
2	rs2287345	54035508	G	0.1856	0.2067	C	0.3832	0.8746
3	rs822354	186480206	A	0.1856	0.2067	G	0.3832	0.8746
4	rs2279027	37903756	A	0.3454	0.32	G	0.3832	1.121
5	rs310501	82889910	G	0.1856	0.2067	A	0.3832	0.8746
8	rs17690844	17611959	C	0.1856	0.2067	A	0.3832	0.8746
9	rs61759822	140147273	A	0.3067	0.3317	G	0.3832	0.8914
10	rs1062465	91497631	A	0.3067	0.3317	T	0.3832	0.8914
10	rs1886997	91498254	G	0.3067	0.3317	A	0.3832	0.8914
14	rs7152747	20960168	G	0.3067	0.3317	A	0.3832	0.8914
7	rs61764067	33380556	A	0.03608	0.02671	G	0.3833	1.364
3	rs6772209	175647165	A	0.04897	0.03917	G	0.3834	1.263

4	rs12505765	159544076	A	0.04897	0.03917	C	0.3834	1.263
9	rs16937883	20098711	G	0.04897	0.03917	A	0.3834	1.263
12	rs36146434	133803551	G	0.1418	0.1244	C	0.3834	1.163
16	rs11466024	3299468	A	0.04897	0.03917	G	0.3834	1.263
19	rs73053944	49930295	C	0.04897	0.03917	G	0.3834	1.263
20	rs6029526	39672618	T	0.2191	0.1975	A	0.3834	1.14
2	rs76878531	99279812	A	0.08763	0.07417	C	0.3836	1.199
2	rs922984	179615887	A	0.2706	0.2483	G	0.3836	1.123
6	rs3135338	32401217	G	0.2706	0.2483	A	0.3836	1.123
6	rs3135335	32401845	G	0.2706	0.2483	C	0.3836	1.123
6	rs2395178	32405362	G	0.2706	0.2483	C	0.3836	1.123
19	rs4801177	57089050	G	0.3067	0.3308	A	0.3836	0.8948
1	rs823128	205713378	G	0.1469	0.1667	A	0.3837	0.861
14	rs4261431	60004844	G	0.1469	0.1675	A	0.3838	0.8559
22	rs426938	21354970	C	0.1469	0.1675	G	0.3838	0.8559
14	rs75475362	60581906	G	0.1134	0.0975	A	0.384	1.184
15	rs1670227	99670518	G	0.1134	0.0975	A	0.384	1.184
1	exm2230882	117156651	A	0.005155	0.01167	C	0.3841	0.4389
1	rs183111883	178426850	G	0.005155	0.01167	A	0.3841	0.4389
1	rs76432158	186057353	A	0.005155	0.01167	T	0.3841	0.4389
2	rs141225250	109098854	G	0.005155	0.01167	A	0.3841	0.4389
2	rs11240785	110458618	A	0.005155	0.01167	G	0.3841	0.4389
3	rs144518762	99509813	A	0.005155	0.01167	G	0.3841	0.4389
3	rs11545169	184020542	A	0.005155	0.01167	C	0.3841	0.4389
5	rs73191279	103096646	G	0.005155	0.01167	A	0.3841	0.4389
5	rs188700714	139251346	A	0.005155	0.01167	G	0.3841	0.4389
6	rs201867336	109766660	A	0.005155	0.01167	G	0.3841	0.4389
6	rs2297852	131277575	A	0.005155	0.01167	C	0.3841	0.4389
7	rs7799181	146048830	G	0.005155	0.01167	A	0.3841	0.4389
9	rs74671532	34290230	G	0.005155	0.01167	A	0.3841	0.4389
10	rs34097979	89503310	A	0.005155	0.01167	G	0.3841	0.4389
10	rs140444928	97791754	A	0.005155	0.01167	G	0.3841	0.4389
11	rs76274604	7060977	A	0.005155	0.01167	T	0.3841	0.4389
11	rs188442678	65408874	A	0.005155	0.01167	G	0.3841	0.4389
11	rs183462348	75572808	A	0.005155	0.01167	G	0.3841	0.4389
12	exm1766909	2973887	A	0.005155	0.01167	C	0.3841	0.4389
12	rs143532828	2973887	A	0.005155	0.01167	G	0.3841	0.4389
12	rs150572401	29786124	A	0.005155	0.01167	G	0.3841	0.4389
12	rs201002553	133682198	A	0.005155	0.01167	G	0.3841	0.4389
13	rs17314986	23824818	A	0.005155	0.01167	G	0.3841	0.4389
13	rs9583500	111121620	A	0.005155	0.01167	G	0.3841	0.4389
13	rs41306688	114078558	C	0.005155	0.01167	A	0.3841	0.4389
14	rs200783456	24901687	A	0.005155	0.01167	C	0.3841	0.4389

14	rs74581978	52520782	T	0.005155	0.01167	A	0.3841	0.4389
14	rs181093232	61482634	T	0.005155	0.01167	A	0.3841	0.4389
14	rs200152990	64676760	A	0.005155	0.01167	G	0.3841	0.4389
17	rs142719069	72584887	T	0.005155	0.01167	A	0.3841	0.4389
19	rs200972486	4359190	A	0.005155	0.01167	G	0.3841	0.4389
19	rs117661715	7696419	A	0.08763	0.07429	G	0.3841	1.197
19	rs17616661	11303554	G	0.005155	0.01167	A	0.3841	0.4389
19	rs755237	11304404	A	0.005155	0.01167	G	0.3841	0.4389
19	rs148268672	48523018	G	0.005155	0.01167	C	0.3841	0.4389
19	rs147613429	51918617	G	0.005155	0.01167	A	0.3841	0.4389
22	rs186458188	31338196	A	0.005155	0.01167	C	0.3841	0.4389
3	rs7609893	138154174	G	0.3093	0.335	A	0.3842	0.8888
8	rs1986357	10316236	G	0.3093	0.3342	A	0.3842	0.8922
10	rs2277257	73082563	G	0.3093	0.335	A	0.3842	0.8888
10	rs12572012	91487649	A	0.3093	0.335	T	0.3842	0.8888
12	rs2304630	30881809	G	0.3093	0.3342	A	0.3842	0.8922
16	rs16973716	72156842	C	0.3093	0.335	A	0.3842	0.8888
3	rs9860730	64701146	A	0.2191	0.1983	G	0.3844	1.134
5	rs6451173	34840841	G	0.2191	0.1983	A	0.3844	1.134
2	rs1442763	117473622	A	0.3479	0.3233	G	0.3846	1.117
1	rs2272920	169799880	A	0.03351	0.045	C	0.3847	0.7357
7	rs12540919	24756951	A	0.1778	0.1583	G	0.3847	1.15
14	rs1760921	20818131	G	0.1778	0.1583	A	0.3847	1.15
6	rs7768484	29855978	A	0.06701	0.08208	C	0.3849	0.8032
5	rs4634319	27418887	G	0.1418	0.125	A	0.3851	1.156
6	rs2844654	30838688	C	0.2732	0.2508	A	0.3851	1.123
6	rs2582	32974551	A	0.2732	0.2508	C	0.3851	1.123
6	rs1321744	85891744	A	0.06701	0.08167	G	0.3851	0.8076
8	rs2231524	141525327	G	0.06701	0.08167	A	0.3851	0.8076
11	rs578331	97894832	A	0.1418	0.125	G	0.3851	1.156
12	rs202023163	14976419	G	0.06701	0.08167	A	0.3851	0.8076
9	rs2149547	37990801	G	0.3119	0.3367	A	0.3852	0.8929
22	rs5751901	24992266	G	0.3119	0.3367	A	0.3852	0.8929
21	rs13047599	34926260	G	0.1881	0.21	A	0.3853	0.8718
6	rs9367630	55148103	A	0.3479	0.3242	G	0.3854	1.112
11	rs3213706	22881002	G	0.3505	0.3258	A	0.3854	1.117
19	rs3815745	10085054	A	0.2397	0.2633	G	0.3854	0.8819
21	rs2300395	36245198	A	0.116	0.1342	G	0.3854	0.8467
1	rs145815386	26691310	C	0.06701	0.0825	G	0.3855	0.7988
2	rs13429458	43638838	C	0.1881	0.2092	A	0.3855	0.8762
5	rs2637496	124743267	A	0.1881	0.2092	G	0.3855	0.8762
11	rs61759818	7721974	A	0.06701	0.0825	G	0.3855	0.7988
11	rs12289558	7727872	G	0.06701	0.0825	A	0.3855	0.7988

1	rs11249024	24527095	A	0.2397	0.2625	G	0.3856	0.8857
2	rs1545216	130095123	G	0.3144	0.3389	A	0.3861	0.8947
9	rs10780291	81725579	G	0.3144	0.3392	A	0.3861	0.8936
5	rs786841	9041924	A	0.03351	0.04583	G	0.3862	0.7217
6	rs3130041	30536133	G	0.3505	0.3267	A	0.3862	1.112
11	rs10750250	123777497	G	0.1778	0.1592	A	0.3862	1.143
11	rs7942047	123777986	A	0.1778	0.1592	G	0.3862	1.143
16	rs11556528	2050192	A	0.03351	0.04583	C	0.3862	0.7217
21	rs8134582	22861778	G	0.03351	0.04583	A	0.3862	0.7217
22	rs116892729	21235389	G	0.03351	0.04583	A	0.3862	0.7217
19	rs4801958	53667804	G	0.3531	0.3283	A	0.3863	1.117
19	rs12460170	53669492	A	0.3531	0.3283	G	0.3863	1.117
6	rs7453920	32730012	A	0.2216	0.2008	G	0.3865	1.133
6	rs2051549	32730086	G	0.2216	0.2008	A	0.3865	1.133
7	rs60783739	100385532	C	0.04897	0.06167	A	0.3865	0.7835
19	rs4803799	45655486	C	0.04897	0.06167	G	0.3865	0.7835
1	rs2282284	157648543	G	0.04897	0.0625	A	0.3866	0.7724
3	rs78560646	12776291	A	0.04897	0.0625	C	0.3866	0.7724
4	rs3792687	42509101	A	0.04897	0.0625	G	0.3866	0.7724
14	rs35949016	66136163	A	0.1134	0.09833	C	0.3866	1.173
17	rs1064245	644986	C	0.1443	0.1267	A	0.3868	1.163
1	rs11185362	104561488	G	0.2423	0.2658	A	0.387	0.883
10	rs2231687	101473218	A	0.2423	0.2658	G	0.387	0.883
3	rs1353776	134077470	G	0.09021	0.07583	C	0.3873	1.208
7	rs7787167	31789186	G	0.09021	0.07583	A	0.3873	1.208
10	rs805657	105762591	A	0.09021	0.07583	G	0.3873	1.208
12	rs7296418	123457619	G	0.3212	0.3462	A	0.3874	0.894
12	rs2291483	48723784	A	0.1804	0.1608	G	0.3876	1.149
14	rs1367580	45644589	A	0.1804	0.1608	C	0.3876	1.149
8	rs1129424	22926313	G	0.09021	0.1075	A	0.3877	0.8232
10	rs3812694	72015573	C	0.09021	0.1075	A	0.3877	0.8232
2	rs56411706	219895548	A	0.1907	0.2117	C	0.3878	0.8777
7	rs76100363	47879049	A	0.1907	0.2117	G	0.3878	0.8777
14	rs4644	55604935	A	0.1907	0.2117	C	0.3878	0.8777
20	rs66673706	18045793	G	0.3557	0.3317	A	0.3879	1.112
3	rs2606736	11400249	G	0.3582	0.3333	A	0.388	1.116
4	rs12647859	91230579	A	0.2784	0.2558	G	0.388	1.122
6	rs1757171	37487044	G	0.3196	0.3442	A	0.388	0.895
14	rs45621632	71137844	A	0.2784	0.2558	C	0.388	1.122
20	rs6087577	32955423	A	0.3196	0.3442	G	0.388	0.895
7	rs34242818	100230864	G	0.06959	0.05667	C	0.3883	1.245
11	rs11826236	8132301	A	0.06959	0.05667	G	0.3883	1.245
17	rs8074277	80789628	G	0.06959	0.05667	A	0.3883	1.245

1	rs12562058	203192355	A	0.0232	0.01667	C	0.3884	1.401
2	rs118109635	169801131	A	0.0232	0.01667	G	0.3884	1.401
2	rs17856918	187692983	G	0.0232	0.01667	A	0.3884	1.401
2	rs10195758	187693155	T	0.0232	0.01667	A	0.3884	1.401
2	rs10164815	187697883	G	0.0232	0.01667	A	0.3884	1.401
2	rs201306518	202900299	G	0.0232	0.01667	A	0.3884	1.401
2	rs117674897	210887734	G	0.0232	0.01667	A	0.3884	1.401
3	rs3733085	48699599	G	0.0232	0.01667	C	0.3884	1.401
4	rs2276444	74363387	A	0.0232	0.01667	T	0.3884	1.401
4	rs76491994	126372975	C	0.0232	0.01667	A	0.3884	1.401
4	rs142450830	141461350	C	0.0232	0.01667	A	0.3884	1.401
5	rs139387249	68616301	G	0.0232	0.01667	A	0.3884	1.401
6	rs138350475	42712809	G	0.0232	0.01667	A	0.3884	1.401
7	rs7786641	128520441	C	0.0232	0.01667	G	0.3884	1.401
9	rs16931745	117553158	T	0.0232	0.01667	A	0.3884	1.401
16	rs77497016	2215883	G	0.0232	0.01667	A	0.3884	1.401
16	rs75816088	50347917	A	0.0232	0.01667	G	0.3884	1.401
18	rs12606658	30969588	A	0.0232	0.01667	G	0.3884	1.401
19	rs11541192	49377424	A	0.0232	0.01667	G	0.3884	1.401
19	rs80095365	49860521	A	0.0232	0.01667	C	0.3884	1.401
19	rs2240038	57955729	A	0.0232	0.01667	G	0.3884	1.401
1	rs34188717	152191696	C	0.06959	0.05676	G	0.3886	1.243
11	rs239259	64981853	A	0.3222	0.3475	C	0.3886	0.8924
6	rs1573649	32731258	G	0.3582	0.3342	A	0.3887	1.112
6	rs6902723	32731960	G	0.3582	0.3342	A	0.3887	1.112
6	rs9276598	32733987	G	0.3582	0.3342	A	0.3887	1.112
12	rs7963027	108894909	G	0.2809	0.2575	A	0.3887	1.127
1	rs1009806	19500551	A	0.3608	0.3358	G	0.3888	1.116
3	rs12490035	70397985	A	0.2448	0.2675	C	0.3888	0.8879
5	rs9293471	85960197	G	0.2448	0.2675	A	0.3888	0.8879
5	rs10079250	149450132	G	0.3608	0.3358	A	0.3888	1.116
6	rs1579219	30224305	A	0.3608	0.3358	G	0.3888	1.116
7	rs4726020	140603652	G	0.1443	0.1275	A	0.3889	1.154
9	rs476612	79300111	C	0.1443	0.1275	A	0.3889	1.154
6	rs219950	12165363	A	0.1804	0.1617	G	0.389	1.141
20	rs6076122	23731494	A	0.116	0.1	G	0.3891	1.181
1	rs10923472	118565953	A	0.1186	0.1367	G	0.3893	0.8497
16	rs289723	57080528	A	0.1186	0.1367	C	0.3893	0.8497
1	rs10889677	67725120	C	0.2809	0.2583	A	0.3894	1.122
2	rs6719451	238336802	G	0.2809	0.2583	A	0.3894	1.122
2	rs6731972	26151206	G	0.1186	0.1375	A	0.3896	0.8437
15	rs3803405	85383640	A	0.1186	0.1375	G	0.3896	0.8437
17	rs12453883	12656111	G	0.1933	0.215	A	0.3896	0.8749

22	rs137673	39737669	A	0.1186	0.1375	C	0.3896	0.8437
12	rs11066280	112817783	A	0.1521	0.1725	T	0.3897	0.8603
15	rs17555920	25953198	A	0.1521	0.1717	G	0.3897	0.8653
17	rs117651561	34093663	A	0.1521	0.1725	G	0.3897	0.8603
22	rs968451	39670851	C	0.1521	0.1725	A	0.3897	0.8603
1	rs1812738	72410812	A	0.3247	0.3492	G	0.3898	0.8964
2	rs243021	60584819	G	0.3247	0.3492	A	0.3898	0.8964
9	rs3750450	111945049	A	0.1933	0.2142	C	0.39	0.8792
12	rs7487904	53624954	G	0.2474	0.2708	C	0.39	0.8851
2	rs45547640	31569660	C	0.05155	0.04083	A	0.3901	1.277
10	rs117713471	101993090	A	0.05155	0.04083	T	0.3901	1.277
15	rs2290558	42168334	G	0.05155	0.04083	C	0.3901	1.277
15	rs117238468	79217691	A	0.05155	0.04083	G	0.3901	1.277
1	rs680328	32438234	G	0.3634	0.3392	A	0.3902	1.112
6	rs2107203	29669830	A	0.3634	0.3392	G	0.3902	1.112
6	rs3129055	29670261	G	0.3634	0.3392	A	0.3902	1.112
6	rs3131865	29672165	G	0.3634	0.3392	C	0.3902	1.112
7	rs61747373	156743264	A	0.3634	0.3392	C	0.3902	1.112
12	rs11611647	4333919	G	0.2474	0.27	A	0.3903	0.8889
17	rs8178847	64216815	A	0.09021	0.07667	G	0.3903	1.194
17	rs52797880	64216854	G	0.09021	0.07667	A	0.3903	1.194
18	rs16942421	24156425	A	0.09021	0.07667	C	0.3903	1.194
12	rs2048719	43581323	A	0.3273	0.3525	G	0.3904	0.8938
13	rs2260335	114175032	A	0.183	0.1633	G	0.3904	1.147
17	rs2270716	71380087	C	0.183	0.1633	G	0.3904	1.147
20	rs2315008	62343956	C	0.3273	0.3525	A	0.3904	0.8938
1	rs140221000	26688125	G	0.03608	0.0275	A	0.3905	1.324
1	rs3768249	85028969	A	0.03608	0.0275	C	0.3905	1.324
3	rs9838238	98600385	G	0.03608	0.0275	A	0.3905	1.324
6	rs142752446	96996133	A	0.03608	0.0275	G	0.3905	1.324
17	rs145809917	65120111	G	0.03608	0.0275	C	0.3905	1.324
19	rs10410649	57840547	G	0.03608	0.0275	A	0.3905	1.324
1	rs12584	19413261	T	0.2268	0.2058	A	0.3906	1.132
1	rs6703267	112018657	A	0.1469	0.1292	G	0.3906	1.161
2	rs17039212	49955497	A	0.2268	0.2058	C	0.3906	1.132
8	rs11579	142205132	G	0.3273	0.3517	A	0.3907	0.8971
11	rs11823828	5906203	C	0.366	0.3414	A	0.3907	1.114
20	rs6083505	2488753	G	0.3273	0.3517	A	0.3907	0.8971
22	rs107017	23912723	A	0.3273	0.3517	G	0.3907	0.8971
4	rs13116679	102199215	A	0.366	0.3417	G	0.391	1.112
6	rs1233389	29552483	A	0.366	0.3417	C	0.391	1.112
11	rs11823842	5906291	G	0.366	0.3417	A	0.391	1.112
10	rs1013153	119229866	G	0.3686	0.3433	A	0.3912	1.116

17	rs3895874	47047868	G	0.2513	0.2755	A	0.3913	0.8828
2	rs1425046	160374182	A	0.2861	0.2625	C	0.3915	1.126
2	rs61197218	237209197	A	0.2861	0.2625	C	0.3915	1.126
4	rs1339	154631563	G	0.2861	0.2625	A	0.3915	1.126
6	rs2180341	127600630	G	0.2861	0.2625	A	0.3915	1.126
9	rs10120210	95284982	A	0.2861	0.2625	C	0.3915	1.126
3	rs11129795	38589163	A	0.116	0.1008	G	0.3916	1.17
3	rs61744881	183954203	A	0.06959	0.0575	G	0.3918	1.226
7	rs12671170	20193979	C	0.06959	0.0575	A	0.3918	1.226
7	rs441051	94054000	A	0.06959	0.0575	G	0.3918	1.226
10	rs36072133	95162056	G	0.06959	0.0575	C	0.3918	1.226
11	rs16930685	37870981	G	0.183	0.1642	A	0.3918	1.14
15	rs60154727	43579621	A	0.06959	0.0575	G	0.3918	1.226
15	rs74752435	101718950	G	0.06959	0.0575	A	0.3918	1.226
16	rs9921518	54494424	A	0.06959	0.0575	G	0.3918	1.226
5	rs1445898	35910529	G	0.1959	0.2183	A	0.3919	0.8721
2	rs13392783	115198886	G	0.3325	0.3575	A	0.392	0.8951
1	rs202093389	38192814	A	0.01546	0.009167	G	0.3921	1.698
1	rs199883053	152191577	A	0.01546	0.009167	G	0.3921	1.698
1	rs6587649	152192978	A	0.01546	0.009167	G	0.3921	1.698
1	rs3795564	204924020	A	0.01546	0.009167	G	0.3921	1.698
2	rs183773608	20818889	C	0.01546	0.009167	A	0.3921	1.698
2	rs116177854	144966285	A	0.01546	0.009167	C	0.3921	1.698
2	rs17826666	186678633	A	0.01546	0.009167	G	0.3921	1.698
2	rs61732371	225244352	A	0.01546	0.009167	G	0.3921	1.698
3	rs201713841	39226928	A	0.01546	0.009167	G	0.3921	1.698
3	rs79668755	170198680	A	0.01546	0.009167	C	0.3921	1.698
4	rs117478149	3009536	A	0.01546	0.009167	G	0.3921	1.698
4	rs147434354	6925790	T	0.01546	0.009167	A	0.3921	1.698
4	rs150598937	47408896	A	0.01546	0.009167	G	0.3921	1.698
4	rs182531866	68610309	A	0.01546	0.009167	G	0.3921	1.698
4	rs115442639	104101539	A	0.01546	0.009167	T	0.3921	1.698
6	rs199618122	26508197	A	0.01546	0.009167	G	0.3921	1.698
6	rs11538263	31601735	A	0.01546	0.009167	G	0.3921	1.698
7	rs192640482	64167308	G	0.01546	0.009167	A	0.3921	1.698
7	rs12669450	93516148	A	0.01546	0.009167	G	0.3921	1.698
8	rs79487342	11702705	A	0.01546	0.009167	G	0.3921	1.698
8	rs58080661	21978578	G	0.01546	0.009167	A	0.3921	1.698
8	rs2721876	133528075	A	0.2861	0.2633	G	0.3921	1.121
9	rs118034043	138839708	A	0.01546	0.009167	G	0.3921	1.698
10	rs142643091	50098651	A	0.01546	0.009167	G	0.3921	1.698
11	rs61729748	5020933	A	0.01546	0.009167	T	0.3921	1.698
11	rs200976094	6499372	A	0.01546	0.009167	G	0.3921	1.698

11	rs60702727	6503335	A	0.01546	0.009167	C	0.3921	1.698
11	rs61742524	94038811	C	0.01546	0.009167	G	0.3921	1.698
11	rs79887053	117869979	A	0.01546	0.009167	G	0.3921	1.698
11	rs116878385	118920540	A	0.01546	0.009167	G	0.3921	1.698
11	rs76415576	118942344	A	0.01546	0.009167	G	0.3921	1.698
11	rs138802175	125548192	A	0.01546	0.009167	G	0.3921	1.698
16	rs145004672	12798724	A	0.01546	0.009167	G	0.3921	1.698
17	rs3027205	8012511	G	0.01546	0.009167	A	0.3921	1.698
17	rs77688767	39724592	G	0.01546	0.009167	A	0.3921	1.698
17	rs78570050	74288853	C	0.01546	0.009167	A	0.3921	1.698
19	rs78029270	10221218	A	0.01546	0.009167	G	0.3921	1.698
20	rs74420259	239936	A	0.01546	0.009167	G	0.3921	1.698
20	rs139700575	18395092	A	0.01546	0.009167	G	0.3921	1.698
6	rs2229784	33136310	A	0.06959	0.08417	C	0.3922	0.8138
10	rs10885122	113042093	A	0.06959	0.08417	C	0.3922	0.8138
19	rs446014	36348078	C	0.06959	0.08417	A	0.3922	0.8138
4	rs7692808	86641149	G	0.06959	0.085	A	0.3924	0.8051
4	rs7660702	86651464	A	0.06959	0.085	G	0.3924	0.8051
12	rs925368	110390979	G	0.06959	0.085	A	0.3924	0.8051
15	rs55905564	76017452	A	0.09278	0.1092	G	0.3925	0.8346
19	rs2230752	9492370	A	0.3711	0.3467	T	0.3925	1.112
11	rs1443548	20623042	A	0.1546	0.175	G	0.3926	0.8624
13	rs1000022	100461219	G	0.1546	0.175	A	0.3926	0.8624
16	rs7188856	5075633	A	0.1546	0.175	G	0.3926	0.8624
8	rs2293925	144392368	G	0.2294	0.2083	A	0.3927	1.131
10	rs7081076	97174537	A	0.09278	0.11	C	0.3928	0.8275
19	rs8113704	36387881	G	0.09278	0.11	A	0.3928	0.8275
16	rs2111119	53671754	A	0.2526	0.2758	G	0.3929	0.8872
4	rs4456958	104215332	G	0.3351	0.3592	A	0.3932	0.899
11	rs1470899	83986071	A	0.3737	0.3492	G	0.3932	1.112
8	rs3133711	101011612	A	0.1211	0.14	G	0.3934	0.8467
16	rs16946429	77775672	G	0.1211	0.14	A	0.3934	0.8467
3	rs1768208	39523003	G	0.3376	0.3633	A	0.3935	0.8932
3	rs4679392	124646594	G	0.2887	0.2658	A	0.3935	1.121
6	rs3094549	29355148	C	0.3376	0.3633	A	0.3935	0.8932
4	rs4599440	61966273	G	0.3376	0.3625	A	0.3936	0.8964
6	rs3799896	90577984	A	0.09278	0.07833	G	0.394	1.203
8	rs2228505	59508134	G	0.1985	0.2208	A	0.394	0.8736
11	rs11042937	10745394	C	0.1985	0.2208	A	0.394	0.8736
15	rs1319869	99212485	C	0.09278	0.07833	A	0.394	1.203
19	rs10408510	38376485	T	0.09278	0.07833	A	0.394	1.203
19	rs10422056	38378539	G	0.09278	0.07833	C	0.394	1.203
21	rs2823400	17001054	G	0.1985	0.2208	A	0.394	0.8736

4	rs2522426	108621483	G	0.1186	0.1025	A	0.3941	1.178
5	rs13173930	41048533	A	0.1186	0.1025	G	0.3941	1.178
8	rs959409	128920127	G	0.1186	0.1025	A	0.3941	1.178
11	rs55944888	123847473	G	0.1186	0.1025	A	0.3941	1.178
2	rs3754644	237327841	G	0.1495	0.1317	A	0.3943	1.159
3	rs1574455	171509135	A	0.3402	0.3658	G	0.3943	0.8938
5	rs6862273	7272401	A	0.3402	0.3658	G	0.3943	0.8938
9	rs1023000	132662786	G	0.2552	0.2783	A	0.3943	0.8882
10	rs11539866	16858993	G	0.1495	0.1317	A	0.3943	1.159
12	rs2111049	11827629	G	0.1495	0.1317	A	0.3943	1.159
16	rs2290182	66614008	G	0.3402	0.3658	A	0.3943	0.8938
10	rs13088	50315893	G	0.1856	0.1667	A	0.3944	1.139
1	rs1622208	46498375	A	0.2887	0.2667	G	0.3945	1.116
1	rs6663047	77865140	G	0.3789	0.3542	A	0.3946	1.112
6	rs3734854	31078836	A	0.232	0.2108	G	0.3946	1.13
6	rs2233984	31079264	A	0.232	0.2108	G	0.3946	1.13
11	rs2553780	35076479	A	0.3789	0.3542	G	0.3946	1.112
3	rs145422476	9885205	G	0.01289	0.02083	A	0.395	0.6136
3	rs147962041	186917405	A	0.01289	0.02083	C	0.395	0.6136
4	rs146768859	186456560	A	0.01289	0.02083	G	0.395	0.6136
6	rs2076489	29576437	G	0.01289	0.02083	A	0.395	0.6136
6	rs7738815	33020387	C	0.01289	0.02083	G	0.395	0.6136
6	rs56149740	150005515	A	0.01289	0.02083	C	0.395	0.6136
10	rs74878516	49400746	A	0.01289	0.02083	G	0.395	0.6136
10	rs29001311	99227333	C	0.01289	0.02083	A	0.395	0.6136
11	rs145740483	12244998	A	0.01289	0.02083	G	0.395	0.6136
11	rs117320886	76979586	C	0.01289	0.02083	A	0.395	0.6136
20	rs2229473	36760894	A	0.01289	0.02083	C	0.395	0.6136
22	rs200021644	19751849	A	0.01289	0.02083	G	0.395	0.6136
6	rs4840086	100208438	G	0.3428	0.3675	A	0.3951	0.8977
10	rs12217617	50533237	A	0.3789	0.355	G	0.3953	1.108
14	rs8017161	103563195	G	0.3814	0.3567	A	0.3953	1.112
17	rs9910051	29161202	T	0.1572	0.1775	A	0.3954	0.8644
17	rs3816780	29161358	A	0.1572	0.1775	G	0.3954	0.8644
17	rs17826219	29161845	A	0.1572	0.1775	G	0.3954	0.8644
17	rs3764421	29167653	C	0.1572	0.1775	A	0.3954	0.8644
17	rs11657270	29214387	G	0.1572	0.1775	A	0.3954	0.8644
17	rs2433	29226228	G	0.1572	0.1775	A	0.3954	0.8644
10	rs805708	105824333	G	0.2912	0.2692	A	0.3958	1.116
1	rs5368	169696946	A	0.232	0.2117	G	0.3959	1.125
6	rs2517409	30964393	G	0.1881	0.1683	A	0.3959	1.145
6	rs2523915	30973358	T	0.1881	0.1683	A	0.3959	1.145
6	rs2523921	30975090	G	0.1881	0.1683	A	0.3959	1.145

6	rs2308557	31239417	A	0.1881	0.1683	G	0.3959	1.145
5	rs26722	33963870	A	0.3814	0.3575	G	0.396	1.108
6	rs2429657	30471520	G	0.05155	0.06511	A	0.396	0.7804
8	rs3748140	8999019	A	0.05155	0.065	G	0.396	0.7818
1	rs835574	120463230	A	0.05155	0.06417	G	0.3961	0.7926
2	rs10166913	29295389	A	0.2938	0.2708	G	0.3961	1.12
6	rs1057373	32813279	A	0.1495	0.1325	C	0.3961	1.151
18	rs2000813	47093864	A	0.2938	0.2708	G	0.3961	1.12
19	rs3745651	12692001	A	0.05155	0.06417	G	0.3961	0.7926
2	rs2229345	158290914	C	0.05155	0.04167	G	0.3962	1.25
6	rs73439094	10694725	G	0.05155	0.04167	A	0.3962	1.25
9	rs77442027	5920649	A	0.05155	0.04167	G	0.3962	1.25
11	rs4963245	62886800	G	0.05155	0.04167	C	0.3962	1.25
14	rs148747129	77294703	A	0.05155	0.04167	G	0.3962	1.25
19	rs141732375	51845070	A	0.05155	0.04167	T	0.3962	1.25
15	rs12442923	42375978	A	0.1186	0.1033	G	0.3964	1.167
17	rs3744558	10357915	A	0.1186	0.1033	G	0.3964	1.167
5	rs3734110	13701536	G	0.384	0.36	A	0.3966	1.108
7	rs869332	102632247	A	0.3479	0.3725	G	0.3966	0.8989
4	rs13147559	17524570	C	0.09278	0.07917	G	0.3968	1.19
2	rs4247303	85554080	G	0.1237	0.1417	A	0.397	0.8554
6	rs2076299	7580958	G	0.2938	0.2717	A	0.397	1.115
6	rs13196885	36632688	A	0.2603	0.2842	G	0.397	0.8865
18	rs9320001	59814324	G	0.1237	0.1417	C	0.397	0.8554
1	rs1342401	96366808	G	0.2603	0.2833	A	0.3971	0.8901
4	rs17013182	88732874	G	0.1237	0.1425	A	0.3971	0.8495
4	rs12508222	126370647	A	0.1237	0.1425	G	0.3971	0.8495
5	rs2273234	149907602	G	0.1237	0.1425	A	0.3971	0.8495
6	rs3131783	30932068	A	0.1237	0.1425	G	0.3971	0.8495
9	rs1755289	17938351	A	0.3505	0.3758	G	0.3971	0.8963
12	rs839131	86548197	A	0.3505	0.3758	G	0.3971	0.8963
1	rs2641962	34038214	G	0.3866	0.3625	A	0.3972	1.108
2	rs2278586	42990101	G	0.3892	0.3642	C	0.3973	1.112
6	rs1042337	32904981	G	0.3892	0.3642	A	0.3973	1.112
7	rs11760434	130894081	G	0.3505	0.375	A	0.3974	0.8995
14	rs12147141	91790566	G	0.3505	0.375	A	0.3974	0.8995
1	rs75432354	15599025	A	0.09536	0.1117	G	0.3975	0.8386
2	rs2271038	98928757	A	0.07216	0.05917	G	0.3975	1.237
2	rs80272723	238280476	A	0.09536	0.1117	G	0.3975	0.8386
10	rs3747879	135197680	A	0.07216	0.05917	G	0.3975	1.237
16	rs2745097	1536499	A	0.09536	0.1117	C	0.3975	0.8386
1	rs117780546	6215695	A	0.01289	0.02167	G	0.3977	0.5895
1	rs2228605	109884672	A	0.01289	0.02167	C	0.3977	0.5895

1	rs143078977	152277614	A	0.01289	0.02167	G	0.3977	0.5895
2	rs1979038	149210898	A	0.01289	0.02167	G	0.3977	0.5895
6	rs142917604	29644668	A	0.01289	0.02167	G	0.3977	0.5895
6	rs142979264	30043000	A	0.01289	0.02167	G	0.3977	0.5895
7	rs147912535	21609889	C	0.01289	0.02167	A	0.3977	0.5895
11	rs10769086	5602968	G	0.01289	0.02167	A	0.3977	0.5895
11	rs199812058	63072226	A	0.01289	0.02167	C	0.3977	0.5895
11	rs4482039	124121162	A	0.01289	0.02167	G	0.3977	0.5895
13	rs59267170	43463359	A	0.01289	0.02167	G	0.3977	0.5895
15	rs148513655	41797721	A	0.01289	0.02167	C	0.3977	0.5895
17	rs140963535	7847814	A	0.01289	0.02167	G	0.3977	0.5895
20	rs117280420	18122912	G	0.01289	0.02167	C	0.3977	0.5895
1	rs340874	214159256	G	0.3531	0.3783	A	0.3978	0.8969
5	rs10941112	34004707	A	0.3531	0.3783	G	0.3978	0.8969
11	rs1080015	2511527	G	0.3892	0.365	A	0.3978	1.108
15	rs146699267	75183789	C	0.01289	0.0217	A	0.3978	0.5885
18	rs2032729	32917644	G	0.3531	0.3783	A	0.3978	0.8969
4	rs75301950	110773108	A	0.1521	0.1342	G	0.3979	1.157
5	rs4975616	1315660	G	0.1521	0.1342	A	0.3979	1.157
5	rs1823068	58676049	G	0.2036	0.225	A	0.3979	0.8806
6	rs73789362	168708501	A	0.03608	0.04774	C	0.3979	0.7467
13	rs9576175	37679268	C	0.2036	0.225	A	0.3979	0.8806
19	rs12462372	56373462	A	0.2036	0.2258	G	0.3979	0.8764
21	rs220126	43531008	A	0.1521	0.1342	G	0.3979	1.157
1	rs3737564	74929170	G	0.03608	0.0475	A	0.3981	0.7506
2	rs10496176	70173629	G	0.3531	0.3775	A	0.3981	0.9001
3	rs55657005	130442259	G	0.03608	0.0475	A	0.3981	0.7506
5	rs3810869	163234	A	0.03608	0.0475	G	0.3981	0.7506
6	rs79931308	168708887	G	0.03608	0.0475	A	0.3981	0.7506
6	rs117378794	168709728	A	0.03608	0.0475	G	0.3981	0.7506
7	rs6957768	156554830	G	0.1598	0.18	A	0.3981	0.8664
2	rs1143676	182395345	G	0.1598	0.1792	A	0.3984	0.8713
2	rs11897119	66772000	G	0.2371	0.2158	A	0.3985	1.129
1	rs472498	100598866	G	0.0232	0.0325	A	0.3986	0.7069
1	rs687513	100598867	G	0.0232	0.0325	A	0.3986	0.7069
1	rs57960694	196963213	A	0.0232	0.0325	G	0.3986	0.7069
1	rs6000	197019858	C	0.0232	0.0325	A	0.3986	0.7069
1	rs6003	197031021	G	0.0232	0.0325	A	0.3986	0.7069
1	rs61249253	197072560	G	0.0232	0.0325	A	0.3986	0.7069
1	rs77138363	197072802	A	0.0232	0.0325	G	0.3986	0.7069
1	rs62624968	197073318	A	0.0232	0.0325	G	0.3986	0.7069
1	rs112113370	197112375	A	0.0232	0.0325	C	0.3986	0.7069
1	rs77736715	197112477	A	0.0232	0.0325	G	0.3986	0.7069

1	rs113777932	197112538	C	0.0232	0.0325	A	0.3986	0.7069
2	rs35858730	17998095	A	0.0232	0.0325	G	0.3986	0.7069
3	rs117209107	49568275	A	0.0232	0.0325	G	0.3986	0.7069
6	rs12198986	7720059	A	0.1907	0.1708	G	0.3986	1.144
12	rs61760910	123798209	G	0.0232	0.0325	A	0.3986	0.7069
14	rs11549467	62207575	A	0.0232	0.0325	G	0.3986	0.7069
17	rs76982026	73235099	A	0.0232	0.0325	G	0.3986	0.7069
19	rs147675790	46393885	A	0.0232	0.0325	G	0.3986	0.7069
22	rs146205533	26688840	A	0.0232	0.0325	G	0.3986	0.7069
11	rs1293035	62246596	C	0.299	0.2758	A	0.3987	1.12
1	rs10494751	197168625	A	0.0232	0.03333	C	0.3988	0.6887
4	rs2575625	109636348	G	0.3557	0.38	A	0.3988	0.9006
10	rs1799939	43610119	A	0.07216	0.08681	G	0.3988	0.8182
11	rs17744245	85448679	A	0.0232	0.03333	G	0.3988	0.6887
12	rs34719836	122217559	A	0.0232	0.03333	G	0.3988	0.6887
19	rs202050948	14499629	A	0.0232	0.03333	G	0.3988	0.6887
19	rs144157950	38976294	A	0.0232	0.03333	G	0.3988	0.6887
4	rs4647930	1018705	A	0.07216	0.08667	C	0.399	0.8197
6	rs2295275	42224546	T	0.07216	0.08667	A	0.399	0.8197
19	rs3745470	50156329	G	0.07216	0.0875	A	0.3991	0.8111
3	rs75893579	42794123	C	0.03608	0.04833	G	0.3993	0.737
4	rs4956145	108931039	A	0.03608	0.04833	G	0.3993	0.737
14	rs1152522	57948380	A	0.03608	0.04833	G	0.3993	0.737
18	rs2680406	25048913	A	0.3582	0.3825	G	0.3995	0.9012
19	rs11879168	12384580	G	0.299	0.2767	A	0.3995	1.115
19	rs8101881	33364628	A	0.299	0.2767	G	0.3995	1.115
20	rs6136489	1923734	A	0.299	0.2767	C	0.3995	1.115
2	rs60086424	52390119	A	0.3969	0.3725	G	0.3996	1.109
3	rs9821143	14724413	A	0.2655	0.2892	G	0.3996	0.8884
8	rs4582599	66079805	G	0.3969	0.3725	A	0.3996	1.109
11	rs17146964	65249145	G	0.1521	0.135	A	0.3996	1.149
6	rs3812163	7725760	A	0.1907	0.1717	T	0.3997	1.137
20	rs3818222	31812923	G	0.1907	0.1717	A	0.3997	1.137
15	rs62007840	78570918	G	0.3995	0.3742	A	0.3998	1.113
5	rs4479806	26723783	A	0.2062	0.2275	C	0.3999	0.882
6	rs1577917	86691940	A	0.3015	0.2783	G	0.3999	1.119
12	rs2228315	109017898	A	0.3015	0.2783	G	0.3999	1.119
15	rs4489954	68072075	C	0.3015	0.2783	A	0.3999	1.119
2	rs16866406	179457147	A	0.3608	0.385	G	0.4002	0.9018
4	rs10006864	171963233	A	0.3634	0.3883	G	0.4004	0.8992
2	rs13004520	234854540	C	0.09536	0.08083	G	0.4005	1.199
3	rs1131356	58109162	G	0.09536	0.08083	A	0.4005	1.199
5	rs163895	106879502	C	0.07216	0.06	A	0.4006	1.219

9	rs10989064	103111965	G	0.07216	0.06	A	0.4006	1.219
10	rs10825269	55955610	A	0.07216	0.06	G	0.4006	1.219
14	rs1127392	74388908	G	0.07216	0.06	A	0.4006	1.219
20	rs41274714	35128751	A	0.07216	0.06	G	0.4006	1.219
1	rs12060255	109377070	A	0.1263	0.145	C	0.4007	0.8523
6	rs214447	9401979	A	0.3015	0.2792	G	0.4007	1.115
7	rs864745	28180556	G	0.3015	0.2792	A	0.4007	1.115
7	rs849134	28196222	G	0.3015	0.2792	A	0.4007	1.115
8	rs1052704	52232487	A	0.1263	0.1442	C	0.4007	0.8581
8	rs3110127	60130296	G	0.4021	0.3775	A	0.4007	1.109
12	rs17287293	24770878	G	0.1263	0.145	A	0.4007	0.8523
8	rs13261938	138325276	A	0.1624	0.1825	G	0.4008	0.8683
11	rs77336780	5461834	G	0.1624	0.1825	C	0.4008	0.8683
3	rs1805124	38645420	G	0.1211	0.1058	A	0.401	1.164
9	rs9792640	20035279	A	0.1211	0.1058	G	0.401	1.164
1	rs1796942	238270039	A	0.1624	0.1817	G	0.4011	0.8732
5	rs2257505	56207123	T	0.268	0.2908	A	0.4011	0.8929
12	rs9658625	53497402	G	0.1624	0.1817	A	0.4011	0.8732
1	rs10873876	76772328	A	0.3041	0.2808	G	0.4012	1.119
1	rs2806864	117469781	C	0.1933	0.1733	G	0.4012	1.143
16	rs2294619	1814440	G	0.3041	0.2808	A	0.4012	1.119
1	rs3795578	204112984	A	0.4046	0.38	G	0.4013	1.109
3	rs9876781	48487338	A	0.3686	0.3942	G	0.4015	0.8971
5	rs3822357	141244356	A	0.1546	0.1367	G	0.4015	1.156
6	rs1736959	29782470	A	0.1546	0.1367	G	0.4015	1.156
7	rs9639168	13978809	G	0.3686	0.3942	A	0.4015	0.8971
12	rs192798	27666258	A	0.3686	0.3942	G	0.4015	0.8971
12	rs60927391	132626425	A	0.1546	0.1367	G	0.4015	1.156
16	rs3096380	71319646	G	0.1546	0.1367	A	0.4015	1.156
2	rs7598069	98761391	G	0.3686	0.3933	A	0.4016	0.9002
3	rs3827496	115340900	A	0.4072	0.3825	G	0.4018	1.109
3	rs4682090	111897540	A	0.4046	0.3808	C	0.402	1.105
8	rs11135703	23021346	G	0.3711	0.3967	A	0.4021	0.8976
17	rs4795690	30625205	A	0.3711	0.3967	G	0.4021	0.8976
20	rs2071056	32265513	G	0.3711	0.3967	A	0.4021	0.8976
3	rs3796129	196199204	A	0.1933	0.1742	C	0.4022	1.136
6	rs3807031	30033884	A	0.2423	0.2208	C	0.4022	1.128
6	rs2501968	49459978	A	0.3711	0.3958	G	0.4022	0.9008
11	rs10899489	78095373	A	0.3711	0.3958	C	0.4022	0.9008
12	rs3847680	29639186	A	0.2423	0.2208	G	0.4022	1.128
3	rs17806888	67416322	G	0.03866	0.02917	A	0.4023	1.339
3	rs35494829	67426281	G	0.03866	0.02917	A	0.4023	1.339
5	rs117093563	94785958	A	0.03866	0.02917	G	0.4023	1.339

6	rs4711531	38250780	G	0.4098	0.385	A	0.4023	1.109
6	rs61754935	139564117	C	0.03866	0.02917	G	0.4023	1.339
8	rs76659506	22138953	G	0.03866	0.02917	C	0.4023	1.339
9	rs75361513	107288586	G	0.03866	0.02917	A	0.4023	1.339
13	rs2232641	108861645	G	0.03866	0.02917	A	0.4023	1.339
22	rs17003397	24035129	A	0.03866	0.02917	G	0.4023	1.339
1	rs2501279	22368342	A	0.3067	0.2833	G	0.4024	1.119
6	rs6904320	32312082	A	0.09794	0.1142	G	0.4024	0.8424
14	rs7153703	51919822	G	0.09794	0.1142	A	0.4024	0.8424
6	rs12175588	109894773	A	0.2706	0.2933	T	0.4025	0.8938
3	rs1126477	46501268	A	0.3737	0.3992	G	0.4026	0.8982
8	rs667859	2820745	C	0.3737	0.3992	G	0.4026	0.8982
17	rs8080966	18220674	A	0.09794	0.1152	G	0.4026	0.834
17	exm1301275	18221134	A	0.09794	0.1152	G	0.4026	0.834
17	rs1563631	18221134	A	0.09794	0.1152	G	0.4026	0.834
16	rs12930629	24088428	A	0.05412	0.04333	G	0.4027	1.263
16	rs75913929	81187665	A	0.05412	0.04333	G	0.4027	1.263
17	rs140947135	39871804	G	0.05412	0.04333	A	0.4027	1.263
7	rs7804157	144277770	A	0.3737	0.3983	C	0.4028	0.9013
10	rs58692969	71164595	G	0.05412	0.04341	A	0.403	1.261
12	rs7308720	40657700	G	0.1546	0.1375	C	0.403	1.147
16	rs2240140	2812939	A	0.3067	0.2842	C	0.403	1.114
19	rs10416265	33605300	A	0.1546	0.1375	G	0.403	1.147
3	rs2305398	12856856	G	0.2423	0.2217	A	0.4032	1.123
3	rs3732678	12859004	C	0.2423	0.2217	A	0.4032	1.123
4	rs7698239	151368816	A	0.3763	0.4017	G	0.4032	0.8987
11	rs2373115	78091150	A	0.3763	0.4017	C	0.4032	0.8987
15	rs11636580	38872179	G	0.3763	0.4017	A	0.4032	0.8987
6	rs9381174	42399074	A	0.4149	0.39	G	0.4033	1.109
2	rs7584345	67718290	G	0.3763	0.4008	A	0.4034	0.9018
4	rs6817934	157344783	A	0.4124	0.3883	C	0.4034	1.105
5	rs160560	3153253	A	0.2732	0.2967	G	0.4034	0.8911
6	rs3020644	31894626	A	0.4124	0.3883	G	0.4034	1.105
9	rs2298312	35044493	A	0.3763	0.4008	T	0.4034	0.9018
11	rs226115	110664089	A	0.3763	0.4008	C	0.4034	0.9018
14	rs1884537	101251989	A	0.3763	0.4008	G	0.4034	0.9018
19	rs2871987	47175528	G	0.3763	0.4008	A	0.4034	0.9018
5	rs2438652	10239261	G	0.1649	0.185	A	0.4035	0.8702
9	rs10965235	22115105	A	0.1649	0.185	C	0.4035	0.8702
17	rs4969391	79089590	G	0.1649	0.185	A	0.4035	0.8702
1	rs4639796	197126649	G	0.3093	0.2858	A	0.4036	1.119
8	rs2272613	1873540	A	0.3093	0.2858	G	0.4036	1.119
13	rs626277	72347696	A	0.1237	0.1075	C	0.4036	1.172

6	rs1997660	28269663	A	0.3789	0.4042	G	0.4037	0.8992
1	rs617169	88745673	A	0.4175	0.3925	G	0.4038	1.109
4	rs10026185	158390203	A	0.1959	0.1758	G	0.4038	1.142
6	rs2780226	34199092	G	0.1959	0.1758	A	0.4038	1.142
8	rs3816208	97614625	A	0.2113	0.2325	G	0.4038	0.8846
1	rs874881	17660499	C	0.3789	0.4033	G	0.404	0.9023
4	rs56292881	58694609	G	0.3814	0.4067	A	0.4042	0.8997
6	rs3748045	36198577	C	0.3093	0.2867	G	0.4042	1.114
7	rs10238440	14092993	G	0.3093	0.2867	A	0.4042	1.114
1	rs10789332	38734768	A	0.4227	0.3975	G	0.4048	1.11
1	rs12735613	118883973	A	0.3119	0.2883	G	0.4048	1.119
19	rs4805576	28258027	A	0.3119	0.2883	C	0.4048	1.119
19	rs2159324	45695738	G	0.3119	0.2883	A	0.4048	1.119
21	rs2829910	27168049	A	0.3886	0.4133	C	0.4048	0.9021
6	rs9497606	146993445	G	0.1572	0.1392	A	0.4049	1.154
9	rs10761129	94486321	G	0.05412	0.06761	A	0.4049	0.7891
15	rs12594837	42977290	A	0.05412	0.0675	G	0.4049	0.7905
15	rs28680600	42979105	G	0.05412	0.0675	A	0.4049	0.7905
15	rs16957055	42980390	A	0.05412	0.0675	G	0.4049	0.7905
15	rs16957061	42984182	G	0.05412	0.0675	A	0.4049	0.7905
15	rs16957063	42984288	G	0.05412	0.06761	A	0.4049	0.7891
17	rs7209435	61712964	G	0.05412	0.0675	A	0.4049	0.7905
1	rs141489383	241798684	G	0.02577	0.01833	A	0.4051	1.417
2	rs139296152	98737874	A	0.02577	0.01833	C	0.4051	1.417
2	rs56211316	135744356	A	0.02577	0.01833	G	0.4051	1.417
6	rs17068451	139576736	A	0.02577	0.01833	G	0.4051	1.417
7	rs3815682	36324325	A	0.02577	0.01833	C	0.4051	1.417
8	rs77653001	53586746	A	0.02577	0.01833	G	0.4051	1.417
10	rs142187025	106125594	A	0.02577	0.01833	G	0.4051	1.417
10	rs35697338	120928705	A	0.02577	0.01833	G	0.4051	1.417
11	rs79102077	34118137	G	0.02577	0.01833	A	0.4051	1.417
11	rs61897432	47431703	G	0.02577	0.01833	A	0.4051	1.417
15	rs28381099	48623812	A	0.02577	0.01833	G	0.4051	1.417
19	rs7260605	41947635	G	0.384	0.4083	A	0.4051	0.9033
19	rs3745226	42583753	A	0.02577	0.01833	G	0.4051	1.417
4	rs1567047	126372742	A	0.4253	0.4	G	0.4052	1.11
6	rs707938	31729359	G	0.3866	0.4117	A	0.4052	0.9007
6	rs12191891	69631411	A	0.4253	0.4	G	0.4052	1.11
6	rs3816665	129571330	A	0.05412	0.06667	G	0.4052	0.8011
11	rs6483617	19854088	A	0.4227	0.3983	G	0.4052	1.106
15	rs2228510	63970456	G	0.05412	0.06667	A	0.4052	0.8011
17	rs12449568	54430155	G	0.4227	0.3983	A	0.4052	1.106
1	rs11264300	155019710	A	0.07474	0.08917	C	0.4055	0.8252

2	rs2228202	70037739	C	0.07474	0.08917	G	0.4055	0.8252
9	rs3739922	135203530	C	0.2139	0.2358	A	0.4055	0.8818
11	rs7111341	2213166	A	0.07474	0.09	G	0.4055	0.8168
11	rs34123504	118771747	G	0.07474	0.08917	A	0.4055	0.8252
11	rs34752197	118772516	G	0.07474	0.08917	A	0.4055	0.8252
11	rs78780803	118772825	G	0.07474	0.08917	A	0.4055	0.8252
12	rs11172782	59259628	G	0.07474	0.09015	A	0.4055	0.8153
15	rs7176508	70018990	A	0.2139	0.2358	G	0.4055	0.8818
19	rs75766177	36674533	A	0.07474	0.09	C	0.4055	0.8168
6	rs2342767	150209717	C	0.1237	0.1083	G	0.4056	1.162
6	rs9383921	150210685	G	0.1237	0.1083	A	0.4056	1.162
10	rs3810964	135000159	A	0.4253	0.4008	G	0.4056	1.106
14	rs7155279	92485881	A	0.3866	0.4108	C	0.4056	0.9038
11	rs2618202	127169960	G	0.4278	0.4025	A	0.4057	1.11
18	rs16951095	7042911	A	0.2139	0.235	G	0.4057	0.8859
2	rs2288100	31178818	A	0.1675	0.1875	G	0.4061	0.872
8	exm2258692	141461266	A	0.4304	0.405	G	0.4061	1.11
8	rs3735801	141461266	A	0.4304	0.405	G	0.4061	1.11
11	rs1317826	46387868	G	0.1675	0.1875	A	0.4061	0.872
18	rs9945476	9786265	A	0.1675	0.1875	G	0.4061	0.872
1	rs74702216	120168654	C	0.07474	0.06167	A	0.4062	1.229
3	rs6799767	176030096	A	0.3918	0.4167	C	0.4062	0.9017
9	rs3849120	114858776	A	0.3918	0.4167	G	0.4062	0.9017
11	rs7111400	99611634	A	0.3918	0.4167	G	0.4062	0.9017
19	rs59539774	549667	A	0.07474	0.06167	G	0.4062	1.229
4	rs10018622	39135564	G	0.4304	0.4058	A	0.4064	1.106
6	rs3132662	30215592	A	0.3144	0.2917	G	0.4064	1.114
6	rs2844767	30222256	A	0.3144	0.2917	G	0.4064	1.114
12	rs11170631	54041192	G	0.4304	0.4058	A	0.4064	1.106
13	rs4770661	24963371	G	0.4036	0.4282	A	0.4064	0.9038
20	rs6067640	49709374	A	0.4304	0.4058	G	0.4064	1.106
22	rs137425	33591130	G	0.3144	0.2917	A	0.4064	1.114
1	rs61310274	179562740	C	0.3943	0.4192	G	0.4066	0.9022
2	rs6734118	37559355	C	0.3943	0.4192	A	0.4066	0.9022
3	rs11719741	39214312	G	0.3943	0.4192	A	0.4066	0.9022
4	rs340639	87924979	A	0.3918	0.4158	G	0.4066	0.9048
6	rs303648	39699399	C	0.3943	0.4192	A	0.4066	0.9022
7	rs12531723	104326251	C	0.3918	0.4158	A	0.4066	0.9048
10	rs1001065	92090566	G	0.3918	0.4158	A	0.4066	0.9048
16	rs7193955	48122582	G	0.09794	0.08333	A	0.4067	1.194
18	rs1893963	28649042	G	0.09794	0.08333	A	0.4067	1.194
17	rs9890560	31579872	G	0.3969	0.4225	A	0.4069	0.8996
7	rs2227264	141490238	C	0.2809	0.305	A	0.4071	0.8902

2	rs12472172	46023916	A	0.4356	0.4108	G	0.4072	1.107
3	rs33969824	42679777	C	0.1005	0.1175	A	0.4072	0.8393
3	rs10514718	61413814	G	0.1005	0.1167	C	0.4072	0.8461
6	rs2747454	29655113	A	0.1005	0.1167	G	0.4072	0.8461
12	rs2384550	115352731	A	0.1005	0.1167	G	0.4072	0.8461
15	rs2279997	81440818	A	0.1985	0.1792	G	0.4072	1.134
17	rs7207403	47210506	A	0.1005	0.1167	C	0.4072	0.8461
7	rs13246460	73249165	T	0.3995	0.425	A	0.4073	0.9
7	rs13232463	73249299	C	0.3995	0.425	G	0.4073	0.9
12	rs10771427	28605426	A	0.3995	0.425	G	0.4073	0.9
13	rs8000776	74474851	G	0.2165	0.2383	A	0.4073	0.8831
16	rs1713480	12758966	A	0.3995	0.425	G	0.4073	0.9
2	rs2314398	97413488	G	0.3144	0.2925	C	0.4074	1.109
6	rs1150735	30045199	A	0.3144	0.2925	G	0.4074	1.109
6	rs61745666	132938817	A	0.127	0.1117	G	0.4074	1.157
8	rs4961252	142104944	A	0.3144	0.2925	G	0.4074	1.109
5	rs4835768	137441767	A	0.433	0.4092	G	0.4075	1.103
5	rs13178677	153867221	G	0.317	0.2942	A	0.4075	1.114
7	rs13241921	73254812	A	0.3995	0.4242	G	0.4075	0.9031
1	rs3795686	26582091	A	0.2165	0.2375	G	0.4076	0.8871
1	rs1126728	64097432	A	0.25	0.2283	G	0.4076	1.127
2	rs2742347	179600648	A	0.25	0.2283	G	0.4076	1.127
2	rs1883085	179604160	C	0.25	0.2283	A	0.4076	1.127
2	rs2562829	179604366	C	0.25	0.2283	A	0.4076	1.127
9	rs4574	127177161	G	0.2165	0.2375	A	0.4076	0.8871
1	rs3754334	16451767	A	0.1314	0.15	G	0.4077	0.8576
6	rs3094104	30609629	G	0.1314	0.15	A	0.4077	0.8576
4	rs4242004	57953278	C	0.4356	0.4117	A	0.4078	1.103
1	rs3806164	3380057	A	0.1314	0.1492	G	0.4079	0.8632
3	rs7624585	57723143	A	0.4407	0.4158	G	0.4079	1.107
12	rs4764039	14064461	A	0.4021	0.4265	G	0.4079	0.904
1	rs650616	20978058	A	0.4021	0.4267	G	0.408	0.9036
2	rs2404991	128321770	G	0.4021	0.4267	A	0.408	0.9036
3	rs2279830	147105790	G	0.443	0.419	A	0.408	1.103
2	rs1344706	185778428	A	0.4046	0.43	C	0.4081	0.9009
1	rs687050	54718770	G	0.2835	0.3067	A	0.4082	0.8946
1	rs9783065	58913654	A	0.05412	0.04417	G	0.4082	1.238
1	rs75743857	150935173	A	0.01546	0.01	G	0.4082	1.555
1	rs186602563	152191526	C	0.01546	0.01	G	0.4082	1.555
2	rs188718007	24245720	C	0.01546	0.01	G	0.4082	1.555
2	rs35073634	29449820	A	0.01546	0.01	G	0.4082	1.555
2	rs12478601	43721508	A	0.2835	0.3075	G	0.4082	0.8911
2	rs117965097	46739715	A	0.01546	0.01	G	0.4082	1.555

2	rs181228139	170382097	G	0.01546	0.01	A	0.4082	1.555
2	rs114322958	238275918	A	0.01546	0.01	G	0.4082	1.555
3	rs146036715	77595544	G	0.01546	0.01	A	0.4082	1.555
3	rs117872197	132415614	G	0.01546	0.01	A	0.4082	1.555
4	rs140040693	76896921	G	0.01546	0.01	A	0.4082	1.555
4	rs76757102	120057709	C	0.05412	0.04417	A	0.4082	1.238
4	rs35199409	130023884	A	0.05412	0.04417	C	0.4082	1.238
5	rs201139565	55479405	G	0.01546	0.01	C	0.4082	1.555
5	rs112846276	63257002	A	0.01546	0.01	G	0.4082	1.555
5	rs61978570	109954164	A	0.01546	0.01	G	0.4082	1.555
5	rs184792648	160115051	A	0.01546	0.01	G	0.4082	1.555
6	rs2027937	31116502	A	0.05412	0.04417	G	0.4082	1.238
6	rs13204965	127167072	C	0.01546	0.01	A	0.4082	1.555
6	rs145576242	145156939	G	0.01546	0.01	A	0.4082	1.555
6	rs438657	159948959	G	0.05412	0.04417	A	0.4082	1.238
7	rs7810009	56344705	G	0.2835	0.3075	A	0.4082	0.8911
7	rs117305226	123254647	A	0.01546	0.01	G	0.4082	1.555
9	rs180760026	107379712	A	0.01546	0.01	C	0.4082	1.555
9	rs139706626	123220809	C	0.01546	0.01	G	0.4082	1.555
9	rs140786088	130263351	A	0.01546	0.01	G	0.4082	1.555
9	rs56231911	139100889	A	0.01546	0.01	G	0.4082	1.555
10	rs57900755	71164692	G	0.05412	0.04417	A	0.4082	1.238
10	rs79030584	71168766	C	0.05412	0.04417	G	0.4082	1.238
11	rs116665491	213397	A	0.01546	0.01	G	0.4082	1.555
11	rs142554154	1027177	A	0.01546	0.01	G	0.4082	1.555
11	rs3748261	96117529	G	0.01546	0.01	A	0.4082	1.555
11	rs186780639	121008699	A	0.01546	0.01	G	0.4082	1.555
14	rs946616	51387782	A	0.01546	0.01	G	0.4082	1.555
15	rs146571920	40939221	C	0.01546	0.01	A	0.4082	1.555
16	rs199649626	1449417	G	0.01546	0.01	A	0.4082	1.555
16	rs28484999	80660388	A	0.01546	0.01	G	0.4082	1.555
19	rs76546241	16514662	A	0.01546	0.01	G	0.4082	1.555
19	rs79552175	37038682	G	0.01546	0.01	A	0.4082	1.555
19	rs448921	44500761	G	0.01546	0.01	A	0.4082	1.555
19	rs1671152	55526345	A	0.01546	0.01	C	0.4082	1.555
20	rs114335521	20071529	G	0.01546	0.01	A	0.4082	1.555
22	rs34096096	24226584	A	0.01546	0.01	T	0.4082	1.555
22	rs60882514	24226950	G	0.01546	0.01	A	0.4082	1.555
22	rs5759167	43500212	A	0.2835	0.3067	C	0.4082	0.8946
4	rs4145944	47593283	C	0.1598	0.1417	G	0.4083	1.152
4	rs3816873	100504664	G	0.1598	0.1417	A	0.4083	1.152
11	rs622961	94154902	G	0.4046	0.4292	A	0.4084	0.904
17	rs1019238	54278715	G	0.4046	0.4292	A	0.4084	0.904

3	rs7652177	171969077	G	0.4072	0.4325	C	0.4085	0.9014
4	rs13114300	83403563	A	0.4072	0.4325	C	0.4085	0.9014
22	rs79246959	50879373	C	0.01546	0.01002	G	0.4085	1.552
6	rs6454568	87500445	A	0.3196	0.2967	G	0.4086	1.114
1	rs117525287	10381887	G	0.03866	0.03	A	0.4087	1.3
1	rs78916782	236195728	A	0.03866	0.03	G	0.4087	1.3
2	rs141810453	167330355	A	0.03866	0.03	G	0.4087	1.3
6	rs12205331	34898455	A	0.03866	0.03	G	0.4087	1.3
6	rs9464337	55925801	A	0.03866	0.03	C	0.4087	1.3
12	rs74582459	9004388	A	0.03866	0.03	G	0.4087	1.3
12	rs10862089	80699475	A	0.03866	0.03	C	0.4087	1.3
15	rs8034191	78806023	G	0.03866	0.03	A	0.4087	1.3
16	rs74656628	820215	A	0.03866	0.03	T	0.4087	1.3
1	rs2500106	183808474	C	0.4098	0.435	A	0.4089	0.9018
21	rs2837352	41363778	A	0.4485	0.4233	G	0.4089	1.108
6	rs80303690	17292048	A	0.07474	0.0625	G	0.409	1.212
2	rs6710838	223044442	A	0.09794	0.08417	G	0.4091	1.181
3	rs6808138	161392576	G	0.2191	0.2408	C	0.4091	0.8843
9	rs73567906	125239796	G	0.09794	0.08417	C	0.4091	1.181
10	rs4933504	91067595	C	0.09794	0.08417	G	0.4091	1.181
22	rs4821112	21964761	A	0.4459	0.4217	G	0.4091	1.104
11	rs687782	95766185	G	0.4098	0.4342	A	0.4092	0.9049
14	rs28583900	94933640	A	0.4124	0.4375	T	0.4092	0.9023
16	rs8059194	68749663	G	0.4098	0.4342	A	0.4092	0.9049
20	rs3810487	62196033	G	0.4124	0.4375	A	0.4092	0.9023
3	rs182418	60020000	A	0.2861	0.31	G	0.4093	0.8919
5	rs6892205	147475386	G	0.451	0.4258	A	0.4093	1.108
11	rs2618088	76026791	G	0.451	0.4258	A	0.4093	1.108
14	rs6574433	78786159	A	0.2861	0.31	G	0.4093	0.8919
16	rs4889244	81173193	A	0.2526	0.2308	G	0.4093	1.126
22	rs181359	21928641	A	0.451	0.4258	G	0.4093	1.108
22	rs181362	21932068	A	0.451	0.4258	G	0.4093	1.108
22	rs5754217	21939675	A	0.451	0.4258	C	0.4093	1.108
1	rs2786152	111857154	G	0.4485	0.4242	A	0.4094	1.104
1	rs2275253	111861841	G	0.4485	0.4242	A	0.4094	1.104
2	rs2287631	207603234	A	0.4485	0.4242	C	0.4094	1.104
17	rs11867190	5261220	A	0.4149	0.4399	G	0.4095	0.9031
2	rs830964	170156880	A	0.4536	0.4283	G	0.4096	1.108
6	rs75747353	121638666	A	0.201	0.1817	G	0.4096	1.133
6	rs75540618	121638682	A	0.201	0.1817	G	0.4096	1.133
7	rs2072443	150490376	G	0.4536	0.4283	A	0.4096	1.108
7	rs842681	158639395	A	0.4124	0.4367	G	0.4096	0.9053
11	rs4757987	5906205	A	0.4124	0.4367	G	0.4096	0.9053

11	rs497714	128643553	C	0.4149	0.44	A	0.4096	0.9027
17	rs3760269	66289041	A	0.4149	0.44	C	0.4096	0.9027
1	rs11260579	1222267	C	0.451	0.4267	G	0.4097	1.104
2	rs10192856	718744	A	0.451	0.4267	G	0.4097	1.104
2	rs12472079	132298468	C	0.3222	0.2992	A	0.4097	1.113
4	rs2333233	176538654	A	0.451	0.4267	G	0.4097	1.104
6	rs6910232	10902439	A	0.451	0.4267	G	0.4097	1.104
13	rs1887752	29670346	G	0.3222	0.2992	A	0.4097	1.113
19	rs11883343	58117399	A	0.1598	0.1425	G	0.4097	1.144
1	rs2256721	111862952	C	0.4536	0.4292	A	0.4099	1.104
20	rs6067967	50466232	G	0.4562	0.4308	A	0.4099	1.108
1	rs2076349	209800230	G	0.4201	0.445	A	0.4102	0.9035
7	rs11971702	140120546	A	0.4588	0.4333	G	0.4102	1.108
8	rs799889	117250895	A	0.4588	0.4333	C	0.4102	1.108
6	rs200843890	136913602	G	0.007812	0.004167	A	0.4103	1.882
3	rs9854207	27614316	C	0.4227	0.4483	A	0.4104	0.9009
3	rs2139181	181020115	A	0.4175	0.4417	G	0.4104	0.9062
9	rs10756072	10635939	G	0.4227	0.4483	A	0.4104	0.9009
2	rs7589293	81583557	G	0.4588	0.4342	A	0.4105	1.105
6	rs3077	33033022	A	0.4588	0.4342	G	0.4105	1.105
6	rs1042434	33036505	C	0.4588	0.4342	G	0.4105	1.105
6	rs1126543	33037419	G	0.4588	0.4342	A	0.4105	1.105
6	rs1126542	33037424	A	0.4588	0.4342	G	0.4105	1.105
6	rs10214910	33037675	C	0.4588	0.4342	A	0.4105	1.105
12	rs1494505	65810174	G	0.4588	0.4342	A	0.4105	1.105
22	rs6008118	47683805	A	0.4588	0.4342	G	0.4105	1.105
9	rs140486340	130279255	A	0.03866	0.05	G	0.4106	0.7641
11	rs61749195	71943961	A	0.03866	0.05	C	0.4106	0.7641
1	rs193150310	236718052	A	0.007812	0.004174	T	0.4107	1.879
8	rs4419794	87229739	A	0.3247	0.3017	G	0.4107	1.113
14	rs2238272	72467631	C	0.4613	0.4367	A	0.4107	1.105
18	rs7235371	33674808	A	0.4201	0.4442	G	0.4107	0.9066
1	rs2020862	171168584	G	0.4639	0.4392	A	0.4109	1.105
1	rs4551624	200151824	A	0.2216	0.2433	G	0.4109	0.8855
2	rs7421394	14756349	G	0.4639	0.4392	A	0.4109	1.105
7	rs55666134	21723431	A	0.134	0.1525	G	0.4111	0.8601
8	rs10504543	73778642	A	0.201	0.1825	G	0.4111	1.127
10	rs2170132	50013402	A	0.134	0.1525	G	0.4111	0.8601
11	rs73493606	76751166	A	0.201	0.1825	G	0.4111	1.127
1	rs4654773	22252262	A	0.4665	0.4417	G	0.4112	1.105
3	rs2131877	194858374	G	0.4304	0.4558	A	0.4112	0.9021
7	rs1130499	157931144	G	0.4665	0.4417	A	0.4112	1.105
13	rs7323558	37250048	A	0.4304	0.4558	G	0.4112	0.9021

22	rs133501	48912283	G	0.4278	0.4525	A	0.4112	0.9047
1	rs4118325	107577832	A	0.134	0.1517	G	0.4113	0.8656
4	rs2710833	169409958	A	0.134	0.1517	G	0.4113	0.8656
5	rs2431697	159879978	G	0.1727	0.1933	A	0.4113	0.8709
5	rs251253	172480336	A	0.134	0.1517	G	0.4113	0.8656
6	rs66462731	64498055	T	0.1727	0.1933	A	0.4113	0.8709
10	rs3793771	102222957	G	0.134	0.1517	C	0.4113	0.8656
10	rs2298075	102247408	A	0.134	0.1517	C	0.4113	0.8656
3	rs6599132	41039907	A	0.4639	0.4399	G	0.4114	1.102
6	rs1933873	120989799	G	0.4691	0.4442	A	0.4114	1.106
5	rs4869272	95539448	G	0.2912	0.315	A	0.4115	0.8936
7	rs80187893	36657926	A	0.03866	0.05083	G	0.4115	0.7509
10	rs17112598	99016088	G	0.03866	0.05083	A	0.4115	0.7509
17	rs907092	37922259	A	0.3247	0.3025	G	0.4115	1.109
20	rs1884862	23532718	G	0.433	0.4583	A	0.4115	0.9025
2	rs13385952	41531398	G	0.1624	0.1442	A	0.4116	1.151
2	rs68148245	65777628	G	0.4716	0.4467	A	0.4116	1.106
11	rs117625538	76183722	G	0.007772	0.004167	A	0.4116	1.872
18	rs2717128	74987632	G	0.1624	0.1442	A	0.4116	1.151
1	rs3738286	201386916	A	0.1031	0.12	G	0.4117	0.8429
7	rs12670798	21607352	G	0.4356	0.4608	A	0.4117	0.9029
7	rs11978249	121942621	A	0.433	0.4575	G	0.4117	0.9055
11	rs17301182	102991197	A	0.1031	0.12	G	0.4117	0.8429
12	rs1348582	20531756	G	0.2912	0.3139	A	0.4117	0.8983
15	rs74505897	89401379	A	0.07732	0.0925	G	0.4117	0.8221
17	rs2074877	10223714	G	0.433	0.4574	A	0.4117	0.9058
17	rs67568091	60440732	A	0.4356	0.4608	G	0.4117	0.9029
19	rs62115071	51984656	A	0.1031	0.12	G	0.4117	0.8429
20	rs2295564	23546639	A	0.433	0.4575	C	0.4117	0.9055
1	rs609320	25717365	C	0.3273	0.3042	G	0.4118	1.113
10	rs149199305	29783896	C	0.4381	0.4633	G	0.4119	0.9032
2	rs3813243	179434516	A	0.2036	0.1842	G	0.412	1.133
2	rs2293076	220100209	A	0.007772	0.004174	G	0.412	1.869
4	rs34670941	79447763	G	0.2036	0.1842	A	0.412	1.133
5	rs889312	56031884	A	0.4768	0.4517	C	0.412	1.106
6	rs3094576	29516242	A	0.2036	0.1842	C	0.412	1.133
12	rs11068428	117693817	A	0.4768	0.4517	G	0.412	1.106
16	rs1859088	71206836	A	0.2036	0.1842	G	0.412	1.133
2	rs12463513	59644842	A	0.4716	0.4475	G	0.4121	1.102
18	rs3744998	43497710	A	0.4716	0.4475	G	0.4121	1.102
6	rs1233387	29555864	A	0.4768	0.4524	G	0.4122	1.103
6	rs743923	35980121	A	0.07772	0.09333	G	0.4122	0.8186
12	rs2650000	121388962	C	0.4407	0.4658	A	0.4122	0.9036

6	rs440169	32213788	G	0.4381	0.4625	A	0.4123	0.9063
8	rs28432935	77764207	A	0.4381	0.4625	G	0.4123	0.9063
9	rs736253	93072009	G	0.4381	0.4625	A	0.4123	0.9063
16	rs72787857	63120000	A	0.482	0.4567	G	0.4123	1.107
3	rs2642926	27615419	G	0.4768	0.4525	A	0.4124	1.103
12	rs2111902	109278747	C	0.4768	0.4525	A	0.4124	1.103
16	rs17856580	27246617	G	0.4433	0.4683	A	0.4124	0.904
20	rs2211350	40483420	A	0.4433	0.4683	G	0.4124	0.904
2	rs1054565	10050960	A	0.4845	0.4592	G	0.4125	1.107
3	rs6798928	151579873	T	0.4794	0.455	A	0.4125	1.103
3	rs2271791	192994543	G	0.3273	0.305	A	0.4125	1.109
5	rs2199161	71410356	C	0.4407	0.465	G	0.4125	0.9066
8	rs4737009	41630405	G	0.4845	0.4592	A	0.4125	1.107
8	rs4335161	136770912	G	0.2938	0.3175	A	0.4125	0.8944
10	rs3793805	51049027	G	0.4407	0.465	A	0.4125	0.9066
11	rs1793174	118074337	A	0.2938	0.3175	G	0.4125	0.8944
7	rs34207852	152402621	A	0.4871	0.4617	G	0.4126	1.107
11	rs236918	117091609	C	0.4459	0.4708	G	0.4126	0.9043
13	rs17070284	79359424	A	0.1289	0.1125	G	0.4126	1.167
6	rs7739434	29930619	G	0.2242	0.2467	A	0.4127	0.8827
6	rs2523708	31451279	G	0.4637	0.4883	A	0.4127	0.9061
12	rs2251979	104253514	G	0.2242	0.2467	A	0.4127	0.8827
15	rs2469202	77282884	G	0.4485	0.4733	A	0.4127	0.9047
20	rs2072910	9365303	G	0.2938	0.3167	A	0.4127	0.8978
1	rs1543102	227418130	G	0.4433	0.4675	A	0.4128	0.907
3	rs16888	157375087	A	0.4433	0.4675	C	0.4128	0.907
5	rs11743823	168164744	G	0.4845	0.46	A	0.4128	1.103
6	rs4712523	20657564	G	0.4897	0.4642	A	0.4128	1.108
10	rs7087280	91275580	A	0.4897	0.4642	G	0.4128	1.108
20	rs2236178	21142813	A	0.4897	0.4642	G	0.4128	1.108
1	rs200980839	10166271	A	0.007732	0.004167	G	0.4129	1.862
1	rs149866732	14108437	A	0.007732	0.004167	G	0.4129	1.862
1	rs191318271	17559438	A	0.007732	0.004167	G	0.4129	1.862
1	rs188918935	17748785	G	0.007732	0.004167	A	0.4129	1.862
1	rs148310921	33361173	A	0.007732	0.004167	G	0.4129	1.862
1	rs57378215	54059857	G	0.007732	0.004167	A	0.4129	1.862
1	rs180858237	55474231	A	0.007732	0.004167	G	0.4129	1.862
1	rs74882029	120497840	A	0.007732	0.004167	T	0.4129	1.862
1	rs2274618	145535884	A	0.007732	0.004167	G	0.4129	1.862
1	rs186255422	155451832	G	0.007732	0.004167	A	0.4129	1.862
1	rs200621017	156502788	A	0.007732	0.004167	G	0.4129	1.862
1	rs116754841	159409798	T	0.007732	0.004167	A	0.4129	1.862
1	rs199649451	160394930	A	0.007732	0.004167	G	0.4129	1.862

1	rs79517167	166892001	A	0.007732	0.004167	G	0.4129	1.862
1	rs114052315	197398616	A	0.007732	0.004167	G	0.4129	1.862
1	rs28450935	206302435	G	0.007732	0.004167	A	0.4129	1.862
1	rs200319163	228363090	C	0.007732	0.004167	A	0.4129	1.862
2	rs142847271	3504619	A	0.007732	0.004167	G	0.4129	1.862
2	rs7561273	24247514	A	0.1624	0.145	G	0.4129	1.143
2	rs201295238	24477250	G	0.007732	0.004167	A	0.4129	1.862
2	rs142177494	32475369	G	0.007732	0.004167	A	0.4129	1.862
2	rs117416357	38201217	G	0.007732	0.004167	A	0.4129	1.862
2	rs201315315	62066748	G	0.007732	0.004167	A	0.4129	1.862
2	rs192569396	69723185	G	0.007732	0.004167	A	0.4129	1.862
2	rs143215007	70082038	G	0.007732	0.004167	A	0.4129	1.862
2	rs201974100	119603999	A	0.007732	0.004167	C	0.4129	1.862
2	rs148375672	120362492	A	0.007732	0.004167	G	0.4129	1.862
2	rs117266463	131797874	A	0.007732	0.004167	G	0.4129	1.862
2	rs200253659	158272332	G	0.007732	0.004167	A	0.4129	1.862
2	rs200947131	197586271	A	0.007732	0.004167	G	0.4129	1.862
2	rs143196218	220412402	A	0.007732	0.004167	G	0.4129	1.862
2	rs146905561	233392983	A	0.007732	0.004167	C	0.4129	1.862
2	rs150681136	241402836	A	0.007732	0.004167	G	0.4129	1.862
3	rs148330866	19559510	G	0.007732	0.004167	C	0.4129	1.862
3	rs78863115	19974891	A	0.007732	0.004167	T	0.4129	1.862
3	rs181153514	47040466	A	0.007732	0.004167	C	0.4129	1.862
3	rs201462120	47702865	A	0.007732	0.004167	G	0.4129	1.862
3	rs182302391	127703051	G	0.007732	0.004167	A	0.4129	1.862
3	rs142933486	138382857	A	0.007732	0.004167	C	0.4129	1.862
3	rs146851567	183822699	A	0.007732	0.004167	G	0.4129	1.862
4	rs36086854	2460546	A	0.007732	0.004167	G	0.4129	1.862
4	rs56359290	66231683	A	0.007732	0.004167	T	0.4129	1.862
4	rs147000473	87691080	A	0.007732	0.004167	G	0.4129	1.862
5	rs201188055	63509716	A	0.007732	0.004167	C	0.4129	1.862
5	rs1071598	78181423	A	0.007732	0.004167	G	0.4129	1.862
6	rs200438724	24436901	G	0.007732	0.004167	A	0.4129	1.862
6	rs181680390	26452274	T	0.007732	0.004167	A	0.4129	1.862
6	rs147592442	36762419	A	0.007732	0.004167	G	0.4129	1.862
6	rs147357473	41304139	A	0.007732	0.004167	G	0.4129	1.862
6	rs6935403	72456393	G	0.451	0.4758	A	0.4129	0.9051
6	rs115931523	90405706	A	0.007732	0.004167	G	0.4129	1.862
6	rs112671344	99893693	G	0.007732	0.004167	A	0.4129	1.862
6	rs112157723	99893698	A	0.007732	0.004167	C	0.4129	1.862
6	rs189281869	99894012	A	0.007732	0.004167	T	0.4129	1.862
6	rs181478757	109197433	A	0.007732	0.004167	G	0.4129	1.862
6	rs183117869	111628730	A	0.007732	0.004167	G	0.4129	1.862

6	rs138509817	152631649	G	0.007732	0.004167	A	0.4129	1.862
7	rs188935423	878560	A	0.007732	0.004167	G	0.4129	1.862
7	rs113573154	48520686	A	0.007732	0.004167	C	0.4129	1.862
7	rs45476795	87056178	A	0.007732	0.004167	C	0.4129	1.862
7	rs146486358	101848448	A	0.007732	0.004167	G	0.4129	1.862
8	rs199982834	21956804	A	0.007732	0.004167	C	0.4129	1.862
8	rs200325494	27277553	A	0.007732	0.004167	C	0.4129	1.862
8	rs77688035	66539611	G	0.007732	0.004167	A	0.4129	1.862
8	rs827528	104312432	G	0.007732	0.004167	A	0.4129	1.862
9	rs56345770	137806260	A	0.007732	0.004167	G	0.4129	1.862
10	rs61733228	50165258	A	0.007732	0.004167	G	0.4129	1.862
11	rs147309967	4703776	A	0.007732	0.004167	C	0.4129	1.862
11	rs140930907	5221885	C	0.007732	0.004167	A	0.4129	1.862
11	rs201140744	6233097	C	0.007732	0.004167	A	0.4129	1.862
11	rs190405500	57077444	G	0.007732	0.004167	A	0.4129	1.862
11	rs1042838	100933412	A	0.007732	0.004167	C	0.4129	1.862
11	rs188113889	103128425	A	0.007732	0.004167	G	0.4129	1.862
12	rs201930455	129360559	A	0.007732	0.004167	G	0.4129	1.862
13	rs143304315	78146273	A	0.007732	0.004167	G	0.4129	1.862
13	rs1218282	79455832	G	0.007732	0.004167	A	0.4129	1.862
13	rs149464166	88327997	A	0.007732	0.004167	C	0.4129	1.862
14	rs146638221	45431760	C	0.007732	0.004167	A	0.4129	1.862
14	rs79082734	61442445	A	0.007732	0.004167	G	0.4129	1.862
14	rs148266566	72138332	G	0.007732	0.004167	A	0.4129	1.862
14	rs140462715	72138346	A	0.007732	0.004167	T	0.4129	1.862
14	rs28365054	90429858	A	0.007732	0.004167	G	0.4129	1.862
14	rs201962564	95923590	A	0.007732	0.004167	C	0.4129	1.862
14	rs12898111	101668606	G	0.4536	0.4792	A	0.4129	0.9024
15	rs77843912	74627499	C	0.007732	0.004167	G	0.4129	1.862
15	rs147387691	90171613	A	0.007732	0.004167	G	0.4129	1.862
16	rs77567924	2511194	A	0.007732	0.004167	G	0.4129	1.862
16	rs200969701	14743700	A	0.007732	0.004167	G	0.4129	1.862
16	rs185496208	15702351	A	0.007732	0.004167	T	0.4129	1.862
16	rs138333984	30715397	A	0.007732	0.004167	G	0.4129	1.862
16	rs11646512	54120952	A	0.4871	0.4625	G	0.4129	1.104
16	rs140682724	84256130	A	0.007732	0.004167	G	0.4129	1.862
16	rs114255772	89592774	G	0.007732	0.004167	A	0.4129	1.862
17	rs149143405	7637795	A	0.007732	0.004167	G	0.4129	1.862
17	rs145772552	36868162	C	0.007732	0.004167	G	0.4129	1.862
17	rs2290863	48606194	A	0.007732	0.004167	G	0.4129	1.862
17	rs202026402	55065622	A	0.007732	0.004167	G	0.4129	1.862
17	rs117065924	76121927	A	0.007732	0.004167	G	0.4129	1.862
18	rs142471510	321773	A	0.007732	0.004167	G	0.4129	1.862

18	rs3848519	55247336	A	0.007732	0.004167	C	0.4129	1.862
19	rs200208099	14580776	A	0.007732	0.004167	G	0.4129	1.862
19	rs61734899	15580694	A	0.007732	0.004167	G	0.4129	1.862
19	rs2290647	35506729	A	0.2552	0.235	G	0.4129	1.115
19	rs192426883	51273886	C	0.007732	0.004167	G	0.4129	1.862
19	rs137892890	52380624	G	0.007732	0.004167	C	0.4129	1.862
19	rs141703645	56436357	G	0.007732	0.004167	C	0.4129	1.862
19	rs145003090	57326839	G	0.007732	0.004167	C	0.4129	1.862
20	rs61742029	3016327	A	0.007732	0.004167	G	0.4129	1.862
20	rs186687743	33330534	G	0.007732	0.004167	A	0.4129	1.862
20	rs201134866	52570062	A	0.007732	0.004167	G	0.4129	1.862
22	rs146907080	24717843	G	0.007732	0.004167	A	0.4129	1.862
22	rs1053593	35660875	C	0.4871	0.4625	A	0.4129	1.104
22	rs16845	37326443	G	0.007732	0.004167	C	0.4129	1.862
22	rs17812699	37578652	A	0.007732	0.004167	G	0.4129	1.862
2	rs1982235	176047906	C	0.4897	0.465	A	0.413	1.104
6	rs7759827	67494388	G	0.4562	0.4817	A	0.413	0.9027
15	rs1865486	62864393	C	0.4897	0.465	A	0.413	1.104
19	rs7252434	3739673	G	0.4459	0.47	A	0.413	0.9074
21	rs67886296	16209949	C	0.4897	0.465	A	0.413	1.104
1	rs10218698	76870939	G	0.4923	0.4675	A	0.4131	1.104
6	rs9295474	20652717	G	0.4923	0.4675	C	0.4131	1.104
14	rs7143039	98486883	G	0.4923	0.4675	A	0.4131	1.104
1	rs12121540	85666156	A	0.4588	0.4842	C	0.4132	0.9031
3	rs4682479	112974641	A	0.4588	0.4842	G	0.4132	0.9031
6	rs2736171	31595487	A	0.4485	0.4725	G	0.4132	0.9077
4	rs2118922	178167074	G	0.5	0.475	A	0.4133	1.105
6	rs2734335	31893944	G	0.2577	0.2367	A	0.4133	1.12
1	rs2133173	67243024	A	0.07474	0.06333	C	0.4134	1.195
1	rs7535476	153514241	C	0.0567	0.07	A	0.4134	0.7986
1	rs4925663	247614617	A	0.4639	0.4892	G	0.4134	0.9037
5	rs6897932	35874575	A	0.2036	0.185	G	0.4134	1.126
6	rs561930	74073531	G	0.4588	0.4833	C	0.4134	0.9061
11	rs35844388	71701763	A	0.07474	0.06333	G	0.4134	1.195
12	rs79716906	102117581	G	0.07474	0.06333	A	0.4134	1.195
17	rs11652075	78178893	G	0.4588	0.4833	A	0.4134	0.9061
1	rs2297810	47280859	A	0.3299	0.3075	G	0.4135	1.109
1	rs2782948	208391254	A	0.5052	0.48	G	0.4135	1.106
3	rs9438	154018887	G	0.451	0.475	C	0.4135	0.9081
5	rs28362541	79032666	G	0.4613	0.4858	A	0.4135	0.9065
5	rs28362542	79032711	G	0.4613	0.4858	A	0.4135	0.9065
6	rs6927567	116950734	A	0.3299	0.3075	G	0.4135	1.109
6	rs6916087	130665389	G	0.5052	0.48	A	0.4135	1.106

7	rs4727586	103706936	G	0.5103	0.485	A	0.4135	1.107
8	rs56203327	60775976	A	0.5052	0.48	G	0.4135	1.106
8	rs10956483	130572110	G	0.4691	0.4942	C	0.4135	0.9044
13	rs798274	33045639	A	0.5052	0.48	G	0.4135	1.106
16	rs9937453	22155629	A	0.5077	0.4825	G	0.4135	1.106
18	rs2850542	48403560	C	0.4665	0.4917	A	0.4135	0.904
2	rs28368764	242815059	G	0.518	0.4925	A	0.4136	1.108
3	rs2718812	133399702	A	0.4613	0.4858	G	0.4136	0.9064
3	rs9290883	188605426	A	0.4716	0.4967	G	0.4136	0.9047
5	rs2278239	79033306	A	0.4613	0.4858	C	0.4136	0.9064
5	rs3828611	79034662	C	0.4613	0.4858	G	0.4136	0.9064
7	rs11978304	119313222	A	0.518	0.4925	G	0.4136	1.108
8	rs10780175	6218289	A	0.518	0.4925	G	0.4136	1.108
8	rs35646721	65175053	G	0.4897	0.4658	A	0.4136	1.1
10	rs4363506	129274503	A	0.5155	0.49	G	0.4136	1.107
11	rs7930185	25098938	C	0.1753	0.195	G	0.4136	0.8772
13	rs4772094	99194125	G	0.4716	0.4967	A	0.4136	0.9047
14	rs4981950	32501255	G	0.2964	0.32	A	0.4136	0.8951
15	rs491567	53946593	C	0.518	0.4925	A	0.4136	1.108
18	rs8089027	10597874	A	0.5129	0.4875	G	0.4136	1.107
20	rs3209183	55108617	C	0.4613	0.4858	A	0.4136	0.9064
1	rs6685906	118504265	A	0.4768	0.5017	C	0.4137	0.9053
2	rs6706868	3384825	G	0.4948	0.4708	A	0.4137	1.101
7	rs17136118	102495249	G	0.1753	0.1958	A	0.4137	0.8726
8	rs2114372	53796903	A	0.4639	0.4883	G	0.4137	0.9067
14	rs11704	102808655	G	0.4639	0.4883	C	0.4137	0.9067
15	rs17648128	51689772	A	0.4639	0.4883	G	0.4137	0.9067
19	rs10411735	33444707	G	0.4768	0.5017	A	0.4137	0.9053
1	rs16826658	22485871	A	0.5026	0.4783	C	0.4138	1.102
3	rs6791703	46569721	C	0.2964	0.3192	A	0.4138	0.8986
3	rs3821508	133293618	A	0.2964	0.3192	G	0.4138	0.8986
5	rs1502844	101859317	A	0.5026	0.4783	G	0.4138	1.102
5	rs161557	143200053	A	0.4974	0.4733	G	0.4138	1.101
6	rs2227125	116721656	G	0.4974	0.4733	A	0.4138	1.101
6	rs4896780	145517407	C	0.4665	0.4908	A	0.4138	0.9071
10	rs10748869	83776091	G	0.5155	0.4908	A	0.4138	1.104
11	rs117374975	71507152	C	0.1624	0.1455	A	0.4138	1.139
18	rs7233515	44585955	G	0.4794	0.5042	A	0.4138	0.9056
19	rs2125579	44792701	A	0.5155	0.4908	C	0.4138	1.104
3	rs952365	104981251	A	0.5052	0.4808	G	0.4139	1.102
6	rs2239808	36446975	C	0.5077	0.4833	G	0.4139	1.103
7	rs324987	34821428	A	0.5129	0.4883	G	0.4139	1.103
7	rs4718163	64364381	A	0.5052	0.4808	C	0.4139	1.102

8	rs2294008	143761931	A	0.5103	0.4858	G	0.4139	1.103
9	rs10119431	7759331	G	0.4691	0.4933	A	0.4139	0.9074
12	rs12423651	133295353	G	0.5103	0.4858	A	0.4139	1.103
18	rs1379805	20494552	G	0.5129	0.4883	A	0.4139	1.103
3	rs9870636	81346834	G	0.4716	0.4958	A	0.414	0.9077
19	rs966384	4538599	G	0.4716	0.4958	A	0.414	0.9077
6	rs6568433	106829537	A	0.4742	0.4983	G	0.4141	0.908
10	rs11009601	34237166	G	0.4742	0.4983	A	0.4141	0.908
7	rs77973466	130368464	A	0.1289	0.1133	G	0.4143	1.157
8	rs17337252	53586706	A	0.2062	0.1867	G	0.4143	1.132
9	rs2274159	117166246	A	0.4794	0.5033	G	0.4143	0.9086
1	rs12023067	220964843	C	0.0567	0.07083	G	0.4145	0.7885
6	rs3800324	28264681	A	0.2577	0.2375	G	0.4145	1.115
6	rs11753326	30925985	G	0.0567	0.07083	A	0.4145	0.7885
7	rs4730335	108455345	A	0.0567	0.07083	G	0.4145	0.7885
10	rs4919199	100044136	G	0.0567	0.04583	A	0.4145	1.251
11	rs1255161	95494313	A	0.0567	0.07083	G	0.4145	0.7885
13	rs1886512	74520186	A	0.2577	0.2375	T	0.4145	1.115
10	rs2297991	113913222	A	0.299	0.3225	G	0.4146	0.8959
11	rs57344881	1856344	A	0.007732	0.004195	C	0.4146	1.85
11	rs728341	133037027	A	0.299	0.3225	C	0.4146	0.8959
7	rs28555173	100681172	G	0.2062	0.187	A	0.4147	1.129
9	rs10818991	98367717	G	0.1366	0.1542	A	0.4147	0.868
4	rs13143848	100443720	A	0.1649	0.1467	G	0.4148	1.149
12	rs526680	10069302	A	0.3351	0.3117	G	0.4148	1.113
14	rs1051695	33293122	A	0.2603	0.2392	G	0.4149	1.12
5	rs35755888	52013727	C	0.1005	0.08667	A	0.415	1.178
1	rs3935295	202129895	A	0.1649	0.1475	G	0.416	1.142
4	rs13133174	105061061	A	0.2294	0.2508	C	0.416	0.889
7	rs730497	44223721	A	0.1778	0.1983	G	0.416	0.8743
8	rs4961102	89640705	A	0.1778	0.1983	G	0.416	0.8743
15	rs8030587	42981806	A	0.2294	0.2517	G	0.416	0.8851
20	rs3936192	47246077	G	0.1649	0.1475	C	0.416	1.142
2	rs10195705	80664439	A	0.1057	0.1225	C	0.4162	0.8464
19	rs2261988	4910889	A	0.1057	0.1225	C	0.4162	0.8464
17	rs12150338	1634104	A	0.1057	0.1217	G	0.4164	0.853
8	rs2062375	119977792	C	0.2629	0.2417	G	0.4165	1.119
2	rs4668077	169439848	A	0.3041	0.3275	G	0.4166	0.8974
16	rs1049970	66432423	A	0.2088	0.1892	G	0.4166	1.131
14	rs8022629	94935108	A	0.3402	0.3167	G	0.4167	1.113
1	rs2481665	62594677	G	0.02577	0.01917	A	0.4168	1.354
1	rs2282298	152484245	A	0.02577	0.01917	G	0.4168	1.354
3	rs9843344	13670536	A	0.02577	0.01917	G	0.4168	1.354

5	rs55661460	158747334	A	0.02577	0.01917	G	0.4168	1.354
10	rs11254363	17130693	G	0.02577	0.01917	A	0.4168	1.354
12	rs35000511	48359676	A	0.02577	0.01917	C	0.4168	1.354
12	rs12721427	48368541	A	0.02577	0.01917	G	0.4168	1.354
16	rs200760725	321200	A	0.02577	0.01917	G	0.4168	1.354
16	rs144344219	11272497	A	0.02577	0.01917	G	0.4168	1.354
17	rs190811861	76480966	G	0.02577	0.01917	A	0.4168	1.354
18	rs72481820	67534642	A	0.02577	0.01917	G	0.4168	1.354
19	rs145435433	8188820	A	0.02577	0.01917	G	0.4168	1.354
19	rs10410169	9047267	A	0.02577	0.01917	G	0.4168	1.354
19	rs10417358	9048035	C	0.02577	0.01917	G	0.4168	1.354
19	rs10417600	9048170	A	0.02577	0.01917	G	0.4168	1.354
19	rs12461007	9049149	A	0.02577	0.01917	G	0.4168	1.354
19	rs10410136	9056941	G	0.02577	0.01917	A	0.4168	1.354
19	rs12982113	9057750	T	0.02577	0.01917	A	0.4168	1.354
19	rs12984471	9057896	G	0.02577	0.01917	C	0.4168	1.354
21	rs146664036	34951753	G	0.02577	0.01917	A	0.4168	1.354
3	rs74811185	150931203	A	0.02577	0.03583	G	0.4169	0.7118
4	rs7658637	83499313	A	0.02577	0.03583	G	0.4169	0.7118
5	rs75953884	141036099	A	0.02577	0.03589	G	0.4169	0.7106
11	rs11603869	6541225	G	0.02577	0.03583	C	0.4169	0.7118
11	rs150145390	111896242	A	0.02577	0.03583	G	0.4169	0.7118
13	rs2274827	99356584	A	0.02577	0.03583	G	0.4169	0.7118
16	rs113527563	89348038	G	0.2088	0.1895	C	0.4169	1.129
5	rs2270912	1409836	A	0.02577	0.035	G	0.417	0.7294
8	rs76147813	133634908	A	0.02577	0.035	C	0.417	0.7294
12	rs118092776	103306579	A	0.02577	0.035	G	0.417	0.7294
18	rs17059264	74069122	G	0.3041	0.3267	A	0.417	0.9008
20	rs17440813	56735759	G	0.02577	0.035	A	0.417	0.7294
7	rs2233580	127253550	A	0.07732	0.065	G	0.4171	1.205
8	rs3739261	134239770	G	0.07732	0.065	A	0.4171	1.205
5	rs10069393	50352533	G	0.3402	0.3175	A	0.4172	1.108
6	rs1264344	30800577	A	0.3402	0.3175	G	0.4172	1.108
16	rs137894790	89349519	A	0.02577	0.0192	G	0.4173	1.352
3	rs6809394	156862145	G	0.3067	0.33	A	0.4176	0.8982
13	rs7336332	28058404	G	0.232	0.2542	A	0.4176	0.8862
1	rs11210805	43652442	C	0.232	0.2533	G	0.4177	0.8901
4	rs61747885	140640703	A	0.1392	0.1575	C	0.4177	0.8648
6	rs3095352	30805921	A	0.3402	0.318	G	0.4177	1.106
6	rs6933400	30907176	A	0.0799	0.095	G	0.4177	0.8272
7	rs1134956	89938680	A	0.232	0.2533	G	0.4177	0.8901
10	rs2072276	117849353	G	0.1392	0.1575	A	0.4177	0.8648
13	rs3803230	111119396	G	0.0799	0.095	C	0.4177	0.8272

1	rs79308175	19775375	A	0.1649	0.1483	G	0.4179	1.134
1	rs12129861	145725689	A	0.1649	0.1483	G	0.4179	1.134
3	rs33985936	38936134	A	0.0799	0.09417	G	0.4179	0.8353
3	rs1025341	155548882	A	0.0799	0.09417	C	0.4179	0.8353
9	rs7851696	137779091	A	0.1649	0.1483	C	0.4179	1.134
6	rs943072	43795968	C	0.1392	0.1583	A	0.418	0.8594
9	rs17535963	73150873	C	0.1392	0.1583	A	0.418	0.8594
9	rs41287373	73150918	A	0.1392	0.1583	G	0.418	0.8594
12	rs2230911	121615131	C	0.1392	0.1583	G	0.418	0.8594
21	rs2825730	21048041	G	0.3067	0.3292	A	0.418	0.9016
1	rs508448	29181525	A	0.2655	0.2442	G	0.4181	1.119
1	rs13375867	100575933	G	0.3428	0.32	A	0.4181	1.108
6	rs13199398	15713820	A	0.2655	0.2442	G	0.4181	1.119
11	rs552966	71788217	A	0.2655	0.2442	C	0.4181	1.119
19	rs1545620	17303774	A	0.2655	0.2442	C	0.4181	1.119
2	rs72819488	95947099	A	0.1804	0.2008	G	0.4183	0.8759
17	rs4796555	6683215	G	0.3093	0.3331	A	0.4184	0.8966
14	rs11622794	21109726	A	0.3093	0.3325	G	0.4186	0.8989
15	rs11259964	83745534	A	0.3093	0.3333	G	0.4186	0.8955
17	rs4562	7163739	A	0.3454	0.3222	G	0.4187	1.11
1	rs78399057	152326843	A	0.2113	0.1917	G	0.4189	1.13
19	rs3761097	36290977	A	0.2113	0.1917	G	0.4189	1.13
11	rs60832895	5462105	G	0.3428	0.3208	A	0.419	1.104
11	rs16930998	5462702	A	0.3428	0.3208	G	0.419	1.104
8	rs3739336	67341938	A	0.1675	0.15	C	0.4191	1.14
4	rs3813832	69203080	G	0.2345	0.2567	A	0.4192	0.8874
5	rs2973930	72622740	G	0.2345	0.2567	A	0.4192	0.8874
6	rs2523765	29817106	A	0.3119	0.335	T	0.4195	0.8996
6	rs12205291	56274973	A	0.3119	0.3358	G	0.4195	0.8962
11	rs1374570	57799371	G	0.3119	0.3358	C	0.4195	0.8962
1	rs11581921	43215930	A	0.04124	0.03167	G	0.4198	1.315
1	rs1655519	55119515	C	0.3454	0.3233	A	0.4198	1.104
1	rs211718	76106675	A	0.04124	0.03167	G	0.4198	1.315
7	rs55939427	6820508	A	0.04124	0.03167	C	0.4198	1.315
9	rs116483202	33467469	C	0.04124	0.03167	G	0.4198	1.315
9	rs10978931	110346728	G	0.3454	0.3233	A	0.4198	1.104
11	rs4756448	38590019	G	0.3454	0.3233	A	0.4198	1.104
11	rs117450252	57798798	A	0.04124	0.03167	T	0.4198	1.315
12	rs1994090	40428561	C	0.04124	0.03167	A	0.4198	1.315
17	rs1542752	72938100	A	0.04124	0.03167	G	0.4198	1.315
10	rs10904908	17260290	A	0.3479	0.325	G	0.4199	1.108
1	rs11207933	62676091	A	0.2113	0.1925	C	0.42	1.124
6	rs1612904	32669018	C	0.2113	0.1925	A	0.42	1.124

8	rs2738047	6728298	A	0.04124	0.03172	G	0.42	1.313
1	rs3753565	186316488	A	0.1082	0.125	G	0.4205	0.8497
4	rs11934907	168965733	C	0.3144	0.3375	A	0.4205	0.9003
7	rs62621204	149543269	A	0.268	0.2475	G	0.4205	1.113
9	rs16909677	96860722	A	0.1031	0.08917	T	0.4206	1.174
12	rs77422261	122413196	G	0.183	0.2033	A	0.4206	0.8775
19	rs10846	14582468	G	0.183	0.2033	A	0.4206	0.8775
19	rs34562867	36230767	G	0.1031	0.08917	A	0.4206	1.174
19	rs919804	48869178	A	0.1031	0.08917	G	0.4206	1.174
1	rs7524102	22698447	G	0.183	0.2025	A	0.4207	0.8821
6	rs4122198	29745593	G	0.2396	0.2608	A	0.4207	0.8929
13	rs4772970	109089409	G	0.3505	0.3275	A	0.4207	1.108
1	rs3010877	12779618	A	0.1418	0.16	G	0.4208	0.8671
3	rs7638321	78865306	C	0.2371	0.2592	A	0.4208	0.8885
3	rs9290264	164793758	A	0.1082	0.1242	C	0.4208	0.8562
4	rs10010188	68534392	A	0.1418	0.16	G	0.4208	0.8671
7	rs10949808	156048649	C	0.1082	0.1242	A	0.4208	0.8562
13	rs3897926	28562605	C	0.2371	0.2592	A	0.4208	0.8885
20	rs2145270	6621685	G	0.1082	0.1242	A	0.4208	0.8562
20	rs1741344	4101800	G	0.1675	0.1508	A	0.4209	1.133
2	rs13016342	133540605	A	0.07732	0.06583	C	0.4211	1.189
2	rs7585334	179620951	G	0.2706	0.2492	A	0.4211	1.118
4	rs11568943	110883121	A	0.2139	0.1942	G	0.4211	1.129
6	rs75424888	139094906	A	0.07732	0.06583	G	0.4211	1.189
11	rs8176785	20805286	G	0.134	0.1175	A	0.4211	1.162
9	rs2966332	134183379	G	0.3505	0.3283	A	0.4212	1.104
4	rs13115251	46039478	A	0.317	0.34	G	0.4214	0.901
4	rs2172802	62453209	A	0.317	0.34	G	0.4214	0.901
12	rs557881	319125	G	0.3505	0.3283	A	0.4215	1.104
6	rs2844795	30073847	G	0.3531	0.33	A	0.4216	1.108
6	rs7758128	32345283	A	0.05928	0.0725	C	0.4216	0.8061
6	rs3763308	32374640	A	0.05928	0.0725	G	0.4216	0.8061
6	rs3804505	52303361	A	0.05928	0.0725	G	0.4216	0.8061
3	rs61736141	14798976	A	0.1701	0.1525	G	0.4221	1.139
4	rs12498340	57211352	G	0.2139	0.195	A	0.4221	1.123
6	rs2187689	32852647	G	0.1701	0.1525	A	0.4221	1.139
10	rs3814165	15256194	A	0.1701	0.1525	G	0.4221	1.139
4	rs7693131	42652004	A	0.3196	0.3433	G	0.4222	0.8984
15	rs16963151	50279662	G	0.3196	0.3433	A	0.4222	0.8984
17	rs6504218	62408299	A	0.3196	0.3433	G	0.4222	0.8984
1	exm121943	169513583	A	0.04124	0.0525	C	0.4223	0.7762
1	rs6037	169513583	A	0.04124	0.0525	C	0.4223	0.7762
2	rs17692792	138375972	A	0.04124	0.0525	T	0.4223	0.7762

4	rs3749591	120214030	C	0.3531	0.3308	A	0.4223	1.104
6	rs3130654	30823075	C	0.2397	0.2617	G	0.4223	0.8895
18	rs4940404	56204671	A	0.2397	0.2617	T	0.4223	0.8895
18	rs3809973	56204932	C	0.2397	0.2617	A	0.4223	0.8895
18	rs3809970	56204991	A	0.2397	0.2617	G	0.4223	0.8895
19	rs11556992	38800176	A	0.2397	0.2617	G	0.4223	0.8895
11	rs952700	99585736	A	0.3557	0.3325	G	0.4224	1.108
2	rs12691830	133541454	A	0.05928	0.07333	G	0.4225	0.7963
17	rs12943505	8243469	G	0.05928	0.07333	A	0.4225	0.7963
20	rs17854409	61491494	G	0.05928	0.07333	A	0.4225	0.7963
6	rs1233386	29558190	A	0.2732	0.2517	G	0.4226	1.118
6	rs1233384	29559295	A	0.2732	0.2517	G	0.4226	1.118
6	rs1264333	30844314	G	0.2732	0.2517	A	0.4226	1.118
8	rs17057523	27528446	G	0.2732	0.2517	A	0.4226	1.118
11	rs4753069	92590448	A	0.2732	0.2517	G	0.4226	1.118
11	rs61995873	100863319	C	0.1856	0.206	A	0.4226	0.878
2	rs28364731	42557334	A	0.04124	0.05333	G	0.423	0.7634
3	rs41261344	38616876	A	0.04124	0.05333	G	0.423	0.7634
6	rs17200983	31675283	A	0.04124	0.05333	C	0.423	0.7634
6	rs5030798	31747510	A	0.04124	0.05333	G	0.423	0.7634
6	rs117127493	31837009	C	0.04124	0.05333	G	0.423	0.7634
22	rs11568188	50502491	G	0.134	0.1185	A	0.423	1.151
15	rs202237336	49030822	G	0.002632	0.0008333	C	0.4233	3.164
6	rs2239709	31507447	A	0.08247	0.0975	G	0.4235	0.832
16	rs11642873	85991705	C	0.08247	0.0975	A	0.4235	0.832
17	rs2279961	4689313	G	0.08247	0.0975	C	0.4235	0.832
8	rs1480347	20347457	A	0.1701	0.1533	G	0.4238	1.132
12	rs10770612	20230639	G	0.3247	0.3478	A	0.4238	0.9017
17	rs4792394	13684917	C	0.3582	0.3358	A	0.4238	1.104
2	rs75972274	98408918	A	0.1443	0.1625	G	0.4239	0.8693
8	rs11136344	145059425	G	0.1443	0.1625	A	0.4239	0.8693
11	rs569108	59863104	G	0.1443	0.1625	A	0.4239	0.8693
3	rs704959	39180263	G	0.2423	0.2633	A	0.424	0.8944
4	rs13114026	520853	A	0.2423	0.2633	G	0.424	0.8944
8	rs78202224	145535881	A	0.04145	0.0325	C	0.4241	1.287
11	rs12790125	55322539	A	0.1443	0.1633	G	0.4241	0.864
11	rs12800642	55339676	A	0.1443	0.1633	C	0.4241	0.864
18	rs789063	1006470	G	0.2758	0.2542	A	0.4241	1.117
1	rs4846033	11788564	A	0.01546	0.02333	G	0.4242	0.6574
2	rs77642505	231077517	C	0.01546	0.02333	A	0.4242	0.6574
2	rs116794756	238289977	G	0.01546	0.02333	A	0.4242	0.6574
3	rs2362904	58112488	G	0.01546	0.02333	A	0.4242	0.6574
3	rs12632456	58118555	G	0.01546	0.02333	A	0.4242	0.6574

3	rs114485048	194373592	A	0.01546	0.02333	G	0.4242	0.6574
4	rs7697680	55245541	G	0.08247	0.09833	A	0.4242	0.8242
4	rs17361055	152994226	A	0.01546	0.02333	G	0.4242	0.6574
5	rs141645096	150102470	A	0.01546	0.02333	G	0.4242	0.6574
5	rs140911228	156589901	A	0.01546	0.02333	G	0.4242	0.6574
6	rs75675396	49701456	C	0.01546	0.02333	G	0.4242	0.6574
9	rs147826159	96428334	A	0.01546	0.02333	G	0.4242	0.6574
9	rs7020782	119106881	C	0.2165	0.1975	A	0.4242	1.123
14	rs4027405	64498037	G	0.08247	0.09833	A	0.4242	0.8242
14	rs1256531	65747759	G	0.2165	0.1975	A	0.4242	1.123
16	rs141827032	1537872	G	0.01546	0.02333	C	0.4242	0.6574
16	rs145777693	47497874	A	0.01546	0.02333	G	0.4242	0.6574
16	rs113808967	57761248	A	0.01546	0.02333	G	0.4242	0.6574
19	rs8107776	16842052	A	0.2165	0.1975	C	0.4242	1.123
21	rs150224037	37618346	A	0.01546	0.02333	G	0.4242	0.6574
3	rs7636401	86249401	A	0.3608	0.3383	G	0.4246	1.104
11	rs10896271	56113575	A	0.3273	0.3508	G	0.4247	0.9004
11	rs10896272	56113593	C	0.3273	0.3508	A	0.4247	0.9004
17	rs11868946	7673605	G	0.2165	0.1978	A	0.4247	1.12
3	rs1620217	102240202	A	0.2758	0.255	G	0.4248	1.112
6	rs1233374	29608694	G	0.0799	0.0675	A	0.4248	1.2
9	rs117863779	20885204	G	0.0799	0.0675	A	0.4248	1.2
10	rs10883841	104934709	G	0.0799	0.0675	A	0.4248	1.2
11	rs12290702	99415903	C	0.3273	0.35	A	0.425	0.9037
16	rs3112612	52635164	G	0.1727	0.155	A	0.425	1.138
2	rs12476147	185800905	T	0.1108	0.1283	A	0.4252	0.8466
6	rs7769992	144038185	A	0.1108	0.1283	G	0.4252	0.8466
6	rs9479297	152658142	A	0.1108	0.1283	G	0.4252	0.8466
7	rs13233513	158590740	A	0.1108	0.1283	T	0.4252	0.8466
11	rs17301750	103027234	G	0.1108	0.1283	A	0.4252	0.8466
1	rs11802875	163313539	A	0.04124	0.0325	G	0.4253	1.28
1	rs4418639	197359999	G	0.3634	0.3408	A	0.4253	1.104
1	rs961490	227219076	A	0.04124	0.0325	G	0.4253	1.28
3	rs75314175	196675319	A	0.04124	0.0325	G	0.4253	1.28
4	rs2291182	71201648	C	0.1881	0.2075	A	0.4253	0.8851
5	rs4957048	583442	A	0.04124	0.0325	G	0.4253	1.28
8	rs7837675	36615252	C	0.04124	0.0325	A	0.4253	1.28
8	rs6651252	129567181	G	0.04124	0.0325	A	0.4253	1.28
19	rs117045684	3822179	A	0.04124	0.0325	C	0.4253	1.28
20	rs2064949	19808654	G	0.1881	0.2075	A	0.4253	0.8851
21	rs2824495	19169218	G	0.3634	0.3408	C	0.4253	1.104
7	rs3800855	157985149	C	0.2191	0.1992	G	0.4254	1.128
2	rs7577851	69723710	A	0.2784	0.2567	G	0.4255	1.117

12	rs2291260	133357412	G	0.2784	0.2567	A	0.4255	1.117
4	rs17037864	159894391	A	0.366	0.3425	G	0.4256	1.108
11	rs7114672	5989415	G	0.3073	0.2853	A	0.4256	1.111
1	rs6692219	179989584	G	0.01546	0.02417	C	0.4259	0.6342
2	rs955592	85595818	A	0.01546	0.02417	G	0.4259	0.6342
9	rs3802490	115652927	C	0.01546	0.02417	A	0.4259	0.6342
10	rs149958745	124390741	A	0.01546	0.02417	G	0.4259	0.6342
11	rs11570112	47355475	C	0.01546	0.02417	G	0.4259	0.6342
11	rs41302407	128709940	G	0.01546	0.02417	A	0.4259	0.6342
15	rs149503501	90167382	C	0.01546	0.02417	G	0.4259	0.6342
1	rs2287537	197482265	A	0.366	0.3433	G	0.4261	1.104
7	rs184853658	64439605	A	0.002604	0.0008333	G	0.4262	3.131
1	rs3789529	6153525	A	0.3325	0.3558	G	0.4263	0.9017
4	rs1448808	111445587	A	0.3325	0.3558	G	0.4263	0.9017
4	rs2636761	142067235	A	0.2191	0.2	G	0.4263	1.122
11	rs3018474	77215400	A	0.2191	0.2	C	0.4263	1.122
15	rs8187758	85448875	A	0.3325	0.3556	C	0.4263	0.9026
17	rs2906966	15331517	C	0.3325	0.3558	A	0.4263	0.9017
3	rs596953	172102173	A	0.3686	0.345	G	0.4264	1.108
4	rs7669724	21649840	G	0.1366	0.1208	A	0.4266	1.151
12	rs6487679	9371332	G	0.1366	0.1208	A	0.4266	1.151
14	rs2282032	90758891	A	0.1366	0.1208	C	0.4266	1.151
1	rs145994637	17666309	A	0.002604	0.0008347	G	0.4267	3.125
3	rs13963	45077123	G	0.1727	0.1558	A	0.4267	1.131
4	rs661301	2306053	A	0.2784	0.2579	G	0.4267	1.11
5	rs11957820	74350090	A	0.3325	0.355	G	0.4267	0.9049
8	rs3736032	23423697	A	0.1727	0.1558	G	0.4267	1.131
10	rs7908745	45953767	G	0.1727	0.1558	A	0.4267	1.131
10	rs11239550	46024729	G	0.1727	0.1558	A	0.4267	1.131
16	rs2941265	15235820	G	0.1727	0.1558	A	0.4267	1.131
6	rs2074477	30132035	A	0.2809	0.2592	G	0.4269	1.117
6	rs9380156	30141042	G	0.2809	0.2592	A	0.4269	1.117
6	rs2857174	32754768	A	0.2809	0.2592	G	0.4269	1.117
1	rs3738346	35227466	C	0.1469	0.165	A	0.427	0.8715
12	rs8793	66546100	A	0.1469	0.165	G	0.427	0.8715
20	rs1877751	57967906	A	0.1469	0.165	G	0.427	0.8715
5	rs12188863	101086200	A	0.2474	0.2683	G	0.4271	0.8965
7	rs76490935	150028098	G	0.1469	0.1658	A	0.4271	0.8662
11	rs201124957	47464258	A	0.002604	0.0008361	G	0.4271	3.12
16	rs3213758	53639438	A	0.2474	0.2683	G	0.4271	0.8965
6	rs9362662	90296588	A	0.3351	0.3575	G	0.4275	0.9056
14	rs34852276	65428165	A	0.3711	0.3483	G	0.4275	1.104
18	rs2278161	72176083	G	0.3351	0.3575	A	0.4275	0.9056

2	rs34993780	234681059	C	0.002591	0.0008333	A	0.4276	3.114
3	rs181743300	190147482	A	0.002591	0.0008333	C	0.4276	3.114
11	rs201892175	47183044	G	0.002591	0.0008333	C	0.4276	3.114
12	rs7307331	118509191	G	0.2809	0.26	A	0.4276	1.112
15	rs201424548	42438400	A	0.002591	0.0008333	G	0.4276	3.114
15	rs2414418	55809660	G	0.2809	0.26	A	0.4276	1.112
6	rs1567	126249914	C	0.1753	0.1575	A	0.4279	1.137
4	rs114727657	89671058	A	0.002591	0.0008347	G	0.4281	3.109
2	rs2115591	207527840	A	0.2216	0.2025	C	0.4283	1.121
3	rs7645375	44283808	C	0.2216	0.2025	A	0.4283	1.121
3	rs15967	158320703	A	0.2835	0.2617	C	0.4283	1.116
11	rs1240773	116520527	A	0.2216	0.2025	C	0.4283	1.121
13	rs16946160	92203813	A	0.2216	0.2025	G	0.4283	1.121
11	rs34573569	55681130	A	0.0799	0.06833	T	0.4285	1.184
11	rs61749302	55681566	A	0.0799	0.06833	G	0.4285	1.184
11	rs4367963	55703728	G	0.0799	0.06833	A	0.4285	1.184
11	rs35607186	55761528	G	0.0799	0.06833	A	0.4285	1.184
13	rs17267292	93323146	G	0.0799	0.06833	A	0.4285	1.184
16	rs117800841	86544651	G	0.0799	0.06833	A	0.4285	1.184
4	rs6839946	99076132	C	0.3402	0.3642	A	0.4286	0.9003
6	rs201032669	43623417	A	0.002591	0.0008361	G	0.4286	3.104
9	rs13294245	35826038	G	0.3402	0.3642	A	0.4286	0.9003
9	rs13294256	35826058	G	0.3402	0.3642	A	0.4286	0.9003
20	rs283272	53532627	G	0.3402	0.3642	A	0.4286	0.9003
4	rs3736455	187122319	C	0.3402	0.3633	A	0.4287	0.9035
12	rs6487138	20905250	G	0.3402	0.3633	A	0.4287	0.9035
11	rs1436109	112991618	A	0.1366	0.1217	C	0.4288	1.142
8	rs3747531	16012648	G	0.3763	0.3533	C	0.4289	1.104
11	rs11040920	6579106	A	0.2835	0.2625	C	0.4289	1.112
22	rs5759612	23503170	A	0.3763	0.3533	G	0.4289	1.104
20	rs13831	57475191	A	0.3737	0.3517	G	0.429	1.1
1	rs149268246	982968	A	0.002577	0.0008333	G	0.4291	3.098
1	rs200847553	1116195	A	0.002577	0.0008333	G	0.4291	3.098
1	rs149976444	3753079	A	0.002577	0.0008333	G	0.4291	3.098
1	rs201841976	7870606	A	0.002577	0.0008333	G	0.4291	3.098
1	rs141023619	12172056	A	0.002577	0.0008333	G	0.4291	3.098
1	rs200236202	12725971	C	0.002577	0.0008333	G	0.4291	3.098
1	rs2303193	15794011	G	0.002577	0.0008333	A	0.4291	3.098
1	rs1748020	17668609	A	0.002577	0.0008333	G	0.4291	3.098
1	rs35446243	24406535	A	0.002577	0.0008333	G	0.4291	3.098
1	rs142139501	25880437	A	0.002577	0.0008333	G	0.4291	3.098
1	rs12049489	26648684	G	0.002577	0.0008333	A	0.4291	3.098
1	rs200056806	31189701	A	0.002577	0.0008333	G	0.4291	3.098

1	rs182611312	34158543	A	0.002577	0.0008333	G	0.4291	3.098
1	rs74315319	35250901	A	0.002577	0.0008333	G	0.4291	3.098
1	rs183345423	39945581	A	0.002577	0.0008333	G	0.4291	3.098
1	rs200854120	47013413	A	0.002577	0.0008333	G	0.4291	3.098
1	rs201729184	52215832	G	0.002577	0.0008333	A	0.4291	3.098
1	rs182750927	52703709	G	0.002577	0.0008333	A	0.4291	3.098
1	rs145316839	53553767	A	0.002577	0.0008333	G	0.4291	3.098
1	rs116623976	53569237	A	0.002577	0.0008333	G	0.4291	3.098
1	rs200937198	54348914	A	0.002577	0.0008333	C	0.4291	3.098
1	rs35123335	54433353	A	0.002577	0.0008333	C	0.4291	3.098
1	rs188072572	62732409	A	0.002577	0.0008333	G	0.4291	3.098
1	rs201379753	68897243	A	0.002577	0.0008333	G	0.4291	3.098
1	rs202103118	75596410	G	0.002577	0.0008333	A	0.4291	3.098
1	rs201336358	76384659	T	0.002577	0.0008333	A	0.4291	3.098
1	rs142253670	94473808	A	0.002577	0.0008333	G	0.4291	3.098
1	rs192842970	103444283	A	0.002577	0.0008333	G	0.4291	3.098
1	rs12567377	109808789	A	0.002577	0.0008333	G	0.4291	3.098
1	rs147655719	109812321	A	0.002577	0.0008333	G	0.4291	3.098
1	rs183826417	109823488	A	0.002577	0.0008333	G	0.4291	3.098
1	rs113130058	110260040	G	0.002577	0.0008333	A	0.4291	3.098
1	rs147205230	111490850	A	0.002577	0.0008333	C	0.4291	3.098
1	rs200546876	111781528	G	0.002577	0.0008333	A	0.4291	3.098
1	rs62642559	112045886	A	0.002577	0.0008333	G	0.4291	3.098
1	rs72563166	145440941	A	0.002577	0.0008333	G	0.4291	3.098
1	rs60042414	151263026	A	0.002577	0.0008333	G	0.4291	3.098
1	rs145132244	152193528	A	0.002577	0.0008333	G	0.4291	3.098
1	rs200016086	152328956	T	0.002577	0.0008333	A	0.4291	3.098
1	rs150219758	153313010	A	0.002577	0.0008333	G	0.4291	3.098
1	rs8191984	154938215	A	0.002577	0.0008333	G	0.4291	3.098
1	rs6427128	155026942	A	0.002577	0.0008333	C	0.4291	3.098
1	rs11589479	155033308	A	0.002577	0.0008333	G	0.4291	3.098
1	rs73002716	156713631	A	0.002577	0.0008333	G	0.4291	3.098
1	rs201283301	158324173	G	0.002577	0.0008333	A	0.4291	3.098
1	rs141645736	158326349	A	0.002577	0.0008333	C	0.4291	3.098
1	rs200029238	159904340	A	0.002577	0.0008333	G	0.4291	3.098
1	rs148729808	161132827	A	0.002577	0.0008333	G	0.4291	3.098
1	rs71634903	161681869	G	0.002577	0.0008333	A	0.4291	3.098
1	rs188895908	167096241	A	0.002577	0.0008333	G	0.4291	3.098
1	rs150946852	168066183	G	0.002577	0.0008333	A	0.4291	3.098
1	rs191775270	176564490	A	0.002577	0.0008333	G	0.4291	3.098
1	rs137974907	180044254	G	0.002577	0.0008333	A	0.4291	3.098
1	rs199562661	182026303	A	0.002577	0.0008333	C	0.4291	3.098
1	rs35704242	184764824	A	0.002577	0.0008333	G	0.4291	3.098

1	rs2274367	198677342	A	0.002577	0.0008333	G	0.4291	3.098
1	rs11584383	200935866	G	0.002577	0.0008333	A	0.4291	3.098
1	rs115025356	201352206	A	0.002577	0.0008333	G	0.4291	3.098
1	rs150517332	201356059	A	0.002577	0.0008333	G	0.4291	3.098
1	rs140642471	201843969	G	0.002577	0.0008333	A	0.4291	3.098
1	rs191933826	203154356	A	0.002577	0.0008333	G	0.4291	3.098
1	exm143506	206665036	A	0.002577	0.0008333	G	0.4291	3.098
1	rs182449382	207196454	A	0.002577	0.0008333	G	0.4291	3.098
1	rs34228541	210577901	G	0.002577	0.0008333	A	0.4291	3.098
1	rs200475275	217784321	A	0.002577	0.0008333	C	0.4291	3.098
1	rs147261047	231829700	A	0.002577	0.0008333	G	0.4291	3.098
1	rs117142664	236143150	C	0.002577	0.0008333	A	0.4291	3.098
1	rs150140015	238048809	A	0.002577	0.0008333	G	0.4291	3.098
1	rs28361506	248028136	A	0.002577	0.0008333	G	0.4291	3.098
2	rs202069415	3200775	A	0.002577	0.0008333	G	0.4291	3.098
2	rs182343732	26178526	A	0.002577	0.0008333	G	0.4291	3.098
2	rs200654171	26702166	G	0.002577	0.0008333	A	0.4291	3.098
2	rs200873278	27305584	A	0.002577	0.0008333	G	0.4291	3.098
2	rs77321087	32865434	A	0.002577	0.0008333	G	0.4291	3.098
2	rs13388394	33764176	G	0.002577	0.0008333	A	0.4291	3.098
2	rs143481916	39262577	A	0.002577	0.0008333	G	0.4291	3.098
2	rs34372645	42281312	A	0.002577	0.0008333	G	0.4291	3.098
2	rs201437414	42544584	A	0.002577	0.0008333	G	0.4291	3.098
2	rs4299376	44072576	C	0.002577	0.0008333	A	0.4291	3.098
2	rs6544713	44073881	A	0.002577	0.0008333	G	0.4291	3.098
2	rs4245791	44074431	G	0.002577	0.0008333	A	0.4291	3.098
2	rs6544718	44104925	A	0.002577	0.0008333	G	0.4291	3.098
2	rs145230776	44549897	A	0.002577	0.0008333	G	0.4291	3.098
2	rs142793585	45829107	A	0.002577	0.0008333	G	0.4291	3.098
2	rs74990336	46313438	A	0.002577	0.0008333	G	0.4291	3.098
2	rs149274310	47601060	A	0.002577	0.0008333	G	0.4291	3.098
2	rs78645734	54197829	A	0.002577	0.0008333	C	0.4291	3.098
2	rs201168258	65237843	A	0.002577	0.0008333	G	0.4291	3.098
2	rs145839401	69049787	A	0.002577	0.0008333	G	0.4291	3.098
2	rs143860688	70187890	A	0.002577	0.0008333	G	0.4291	3.098
2	rs149386446	71778842	A	0.002577	0.0008333	G	0.4291	3.098
2	rs189387085	73927826	A	0.002577	0.0008333	G	0.4291	3.098
2	rs186098243	74074562	C	0.002577	0.0008333	G	0.4291	3.098
2	rs180686118	98543946	A	0.002577	0.0008333	G	0.4291	3.098
2	rs13151	101898412	C	0.002577	0.0008333	G	0.4291	3.098
2	rs201793571	109546656	A	0.002577	0.0008333	G	0.4291	3.098
2	rs200635929	111691199	A	0.002577	0.0008333	G	0.4291	3.098
2	rs148442092	121708869	A	0.002577	0.0008333	G	0.4291	3.098

2	rs141573066	121748117	A	0.002577	0.0008333	G	0.4291	3.098
2	rs76476478	131127678	G	0.002577	0.0008333	A	0.4291	3.098
2	rs147613962	144525724	A	0.002577	0.0008333	G	0.4291	3.098
2	rs7559772	159166069	A	0.002577	0.0008333	G	0.4291	3.098
2	rs200624920	167134816	G	0.002577	0.0008333	C	0.4291	3.098
2	rs201385307	168100553	G	0.002577	0.0008333	A	0.4291	3.098
2	rs199566956	168115314	C	0.002577	0.0008333	A	0.4291	3.098
2	rs200916432	170148844	G	0.002577	0.0008333	C	0.4291	3.098
2	rs114420548	170493567	G	0.002577	0.0008333	C	0.4291	3.098
2	rs181918774	175213587	G	0.002577	0.0008333	A	0.4291	3.098
2	rs189421632	178592768	G	0.002577	0.0008333	A	0.4291	3.098
2	rs6433711	178936614	G	0.002577	0.0008333	A	0.4291	3.098
2	rs146831363	179248772	A	0.002577	0.0008333	G	0.4291	3.098
2	rs200321239	179545032	A	0.002577	0.0008333	G	0.4291	3.098
2	rs140037014	183582868	A	0.002577	0.0008333	C	0.4291	3.098
2	rs202188720	196851900	A	0.002577	0.0008333	G	0.4291	3.098
2	rs201002323	197737745	G	0.002577	0.0008333	A	0.4291	3.098
2	rs191446880	202688397	A	0.002577	0.0008333	C	0.4291	3.098
2	rs139395839	207620015	A	0.002577	0.0008333	G	0.4291	3.098
2	rs61752198	214160817	A	0.002577	0.0008333	G	0.4291	3.098
2	rs61752199	214354811	A	0.002577	0.0008333	G	0.4291	3.098
2	rs188997281	216211627	G	0.002577	0.0008333	A	0.4291	3.098
2	rs201988343	216861089	A	0.002577	0.0008333	G	0.4291	3.098
2	rs142015527	216965258	A	0.002577	0.0008333	G	0.4291	3.098
2	rs61746065	218682497	A	0.002577	0.0008333	G	0.4291	3.098
2	rs201975910	219103463	A	0.002577	0.0008333	G	0.4291	3.098
2	rs201910426	219251364	A	0.002577	0.0008333	G	0.4291	3.098
2	rs138326187	219603432	G	0.002577	0.0008333	C	0.4291	3.098
2	rs114268182	220089803	A	0.002577	0.0008333	G	0.4291	3.098
2	rs201865832	220116831	G	0.002577	0.0008333	A	0.4291	3.098
2	rs185822333	220497018	A	0.002577	0.0008333	G	0.4291	3.098
2	rs141548793	223423135	A	0.002577	0.0008333	G	0.4291	3.098
2	rs139596776	223497945	A	0.002577	0.0008333	G	0.4291	3.098
2	rs147605304	227779064	A	0.002577	0.0008333	G	0.4291	3.098
2	rs146333019	228389571	C	0.002577	0.0008333	G	0.4291	3.098
2	rs200837058	230744732	A	0.002577	0.0008333	G	0.4291	3.098
2	rs13397985	231091223	C	0.002577	0.0008333	A	0.4291	3.098
2	rs7423615	231116874	A	0.002577	0.0008333	G	0.4291	3.098
2	rs143514956	233321769	A	0.002577	0.0008333	G	0.4291	3.098
2	rs181492639	234621848	A	0.002577	0.0008333	C	0.4291	3.098
2	rs183802414	234627737	A	0.002577	0.0008333	G	0.4291	3.098
2	rs188786181	238002837	G	0.002577	0.0008333	A	0.4291	3.098
2	rs142851023	238249550	A	0.002577	0.0008333	G	0.4291	3.098

3	rs185053200	10146208	T	0.002577	0.0008333	A	0.4291	3.098
3	rs202117175	10280469	A	0.002577	0.0008333	G	0.4291	3.098
3	rs34020239	12616498	G	0.002577	0.0008333	A	0.4291	3.098
3	rs187013550	15260008	G	0.002577	0.0008333	A	0.4291	3.098
3	rs200908564	17052311	C	0.002577	0.0008333	G	0.4291	3.098
3	rs143411747	37476419	G	0.002577	0.0008333	A	0.4291	3.098
3	rs149921874	38042947	A	0.002577	0.0008333	C	0.4291	3.098
3	rs201025253	38760270	A	0.002577	0.0008333	C	0.4291	3.098
3	rs145034699	39226336	A	0.002577	0.0008333	G	0.4291	3.098
3	rs200112152	39230110	A	0.002577	0.0008333	G	0.4291	3.098
3	rs180834056	44611558	G	0.002577	0.0008333	A	0.4291	3.098
3	rs200700185	46245548	A	0.002577	0.0008333	G	0.4291	3.098
3	rs199525645	47958224	A	0.002577	0.0008333	C	0.4291	3.098
3	rs144196027	49548127	C	0.002577	0.0008333	G	0.4291	3.098
3	rs201733026	49749942	A	0.002577	0.0008333	G	0.4291	3.098
3	rs117758999	49751251	A	0.002577	0.0008333	G	0.4291	3.098
3	rs202219889	49924777	A	0.002577	0.0008333	G	0.4291	3.098
3	rs117368814	51746780	A	0.002577	0.0008333	G	0.4291	3.098
3	rs145603944	52269066	C	0.002577	0.0008333	A	0.4291	3.098
3	rs199810521	52384025	C	0.002577	0.0008333	G	0.4291	3.098
3	rs138560279	52400828	G	0.002577	0.0008333	A	0.4291	3.098
3	rs140460972	52818446	A	0.002577	0.0008333	G	0.4291	3.098
3	rs114804033	58097527	A	0.002577	0.0008333	G	0.4291	3.098
3	rs61730544	88189580	C	0.002577	0.0008333	A	0.4291	3.098
3	rs36040433	97983253	A	0.002577	0.0008333	G	0.4291	3.098
3	rs11921054	98304403	A	0.002577	0.0008333	G	0.4291	3.098
3	rs116508263	100566500	A	0.002577	0.0008333	G	0.4291	3.098
3	rs114399573	111812214	G	0.002577	0.0008333	A	0.4291	3.098
3	rs146790452	118865479	A	0.002577	0.0008333	G	0.4291	3.098
3	rs141778533	119379264	A	0.002577	0.0008333	G	0.4291	3.098
3	rs188225112	119886621	A	0.002577	0.0008333	C	0.4291	3.098
3	rs184446231	119886974	A	0.002577	0.0008333	C	0.4291	3.098
3	rs186768873	120941860	G	0.002577	0.0008333	A	0.4291	3.098
3	rs149775261	123010034	A	0.002577	0.0008333	G	0.4291	3.098
3	rs61736433	124515496	A	0.002577	0.0008333	G	0.4291	3.098
3	rs201937228	127394885	A	0.002577	0.0008333	G	0.4291	3.098
3	rs61750883	130150704	G	0.002577	0.0008333	A	0.4291	3.098
3	rs35409692	130889683	A	0.002577	0.0008333	G	0.4291	3.098
3	rs200768861	165504085	G	0.002577	0.0008333	C	0.4291	3.098
3	rs11920090	170717521	A	0.002577	0.0008333	T	0.4291	3.098
3	rs149460434	170724956	A	0.002577	0.0008333	G	0.4291	3.098
3	rs5400	170732300	A	0.002577	0.0008333	G	0.4291	3.098
3	rs139752448	179298481	G	0.002577	0.0008333	A	0.4291	3.098

3	rs200016332	184008139	A	0.002577	0.0008333	G	0.4291	3.098
3	rs200305407	196386993	A	0.002577	0.0008333	G	0.4291	3.098
3	rs146006052	197430430	A	0.002577	0.0008333	G	0.4291	3.098
4	rs149742374	843765	A	0.002577	0.0008333	G	0.4291	3.098
4	rs188608480	4276484	G	0.002577	0.0008333	A	0.4291	3.098
4	rs139532629	10446892	G	0.002577	0.0008333	A	0.4291	3.098
4	rs190503741	10567709	G	0.002577	0.0008333	A	0.4291	3.098
4	rs180745710	13602603	A	0.002577	0.0008333	G	0.4291	3.098
4	rs118191855	13604644	A	0.002577	0.0008333	G	0.4291	3.098
4	rs62641703	17586746	A	0.002577	0.0008333	G	0.4291	3.098
4	rs201588937	38910316	A	0.002577	0.0008333	G	0.4291	3.098
4	rs201340555	41983886	A	0.002577	0.0008333	G	0.4291	3.098
4	rs140041720	55961103	A	0.002577	0.0008333	G	0.4291	3.098
4	rs35603373	55964373	A	0.002577	0.0008333	G	0.4291	3.098
4	rs201719457	55984888	A	0.002577	0.0008333	G	0.4291	3.098
4	rs55710198	66467566	C	0.002577	0.0008333	A	0.4291	3.098
4	rs138912167	68566823	A	0.002577	0.0008333	C	0.4291	3.098
4	rs139061539	68784777	A	0.002577	0.0008333	G	0.4291	3.098
4	rs143470126	70592820	A	0.002577	0.0008333	G	0.4291	3.098
4	rs149937358	71472409	A	0.002577	0.0008333	G	0.4291	3.098
4	rs201687667	71654677	A	0.002577	0.0008333	C	0.4291	3.098
4	rs186119175	76530796	A	0.002577	0.0008333	C	0.4291	3.098
4	rs61730247	77184996	A	0.002577	0.0008333	G	0.4291	3.098
4	rs141598796	83839374	A	0.002577	0.0008333	C	0.4291	3.098
4	rs144584495	110608696	A	0.002577	0.0008333	G	0.4291	3.098
4	rs147085897	113353620	A	0.002577	0.0008333	G	0.4291	3.098
4	rs188598288	113360884	A	0.002577	0.0008333	C	0.4291	3.098
4	rs183643524	115769485	A	0.002577	0.0008333	G	0.4291	3.098
4	rs35206443	124177315	A	0.002577	0.0008333	C	0.4291	3.098
4	rs115535556	141832316	A	0.002577	0.0008333	G	0.4291	3.098
4	rs199940140	143043366	A	0.002577	0.0008333	G	0.4291	3.098
4	rs200992768	151504191	A	0.002577	0.0008333	G	0.4291	3.098
4	rs75005416	152583764	C	0.002577	0.0008333	G	0.4291	3.098
4	rs189772026	153268198	A	0.002577	0.0008333	G	0.4291	3.098
4	rs62323857	154625398	A	0.002577	0.0008333	G	0.4291	3.098
4	rs1337	154631587	C	0.002577	0.0008333	G	0.4291	3.098
4	rs10517577	154644537	G	0.002577	0.0008333	A	0.4291	3.098
4	rs182327626	155253645	G	0.002577	0.0008333	A	0.4291	3.098
4	rs201570514	155256049	G	0.002577	0.0008333	A	0.4291	3.098
4	rs146201163	169292905	A	0.002577	0.0008333	G	0.4291	3.098
4	rs188714503	185678376	G	0.002577	0.0008333	A	0.4291	3.098
4	rs183979417	186318347	G	0.002577	0.0008333	A	0.4291	3.098
4	rs181526716	187549766	G	0.002577	0.0008333	C	0.4291	3.098

5	rs191669572	492130	A	0.002577	0.0008333	G	0.4291	3.098
5	rs138889059	9227033	A	0.002577	0.0008333	G	0.4291	3.098
5	rs149788485	32087891	A	0.002577	0.0008333	G	0.4291	3.098
5	rs10941412	38919158	A	0.002577	0.0008333	G	0.4291	3.098
5	rs201809503	39202924	A	0.002577	0.0008333	G	0.4291	3.098
5	rs145168670	54423155	A	0.002577	0.0008333	G	0.4291	3.098
5	rs150401044	56777839	A	0.002577	0.0008333	G	0.4291	3.098
5	rs147688707	61690420	G	0.002577	0.0008333	A	0.4291	3.098
5	rs146230664	68419076	A	0.002577	0.0008333	G	0.4291	3.098
5	rs191127702	70805429	C	0.002577	0.0008333	A	0.4291	3.098
5	rs192605828	71654202	A	0.002577	0.0008333	T	0.4291	3.098
5	rs199641855	72469286	A	0.002577	0.0008333	C	0.4291	3.098
5	rs188040167	73197089	A	0.002577	0.0008333	G	0.4291	3.098
5	rs192034423	79815529	A	0.002577	0.0008333	C	0.4291	3.098
5	rs61733389	82836912	A	0.002577	0.0008333	G	0.4291	3.098
5	rs201444192	82837184	G	0.002577	0.0008333	A	0.4291	3.098
5	rs13355469	83136574	A	0.002577	0.0008333	G	0.4291	3.098
5	rs190681125	95128819	C	0.002577	0.0008333	G	0.4291	3.098
5	rs75452273	96320902	A	0.002577	0.0008333	G	0.4291	3.098
5	rs201239691	109117167	A	0.002577	0.0008333	G	0.4291	3.098
5	rs55822801	112920108	C	0.002577	0.0008333	G	0.4291	3.098
5	rs451195	122359640	G	0.002577	0.0008333	A	0.4291	3.098
5	rs145687686	131607096	A	0.002577	0.0008333	G	0.4291	3.098
5	rs202009458	134223478	G	0.002577	0.0008333	A	0.4291	3.098
5	rs17107315	147207678	G	0.002577	0.0008333	A	0.4291	3.098
5	rs41338945	149456995	A	0.002577	0.0008333	C	0.4291	3.098
5	rs140044970	150701661	A	0.002577	0.0008333	G	0.4291	3.098
5	rs189126703	156479540	G	0.002577	0.0008333	A	0.4291	3.098
5	rs200842328	159992512	A	0.002577	0.0008333	G	0.4291	3.098
5	rs79136198	162911142	C	0.002577	0.0008333	A	0.4291	3.098
5	rs74729929	173035291	A	0.002577	0.0008333	G	0.4291	3.098
5	rs35018750	176011984	A	0.002577	0.0008333	G	0.4291	3.098
5	rs144556523	176310795	A	0.002577	0.0008333	G	0.4291	3.098
5	rs61749653	176316559	A	0.002577	0.0008333	G	0.4291	3.098
5	rs2230939	176831085	A	0.002577	0.0008333	G	0.4291	3.098
5	rs202061203	178413934	A	0.002577	0.0008333	G	0.4291	3.098
5	rs200806292	178562980	A	0.002577	0.0008333	C	0.4291	3.098
5	rs143664576	179264734	A	0.002577	0.0008333	G	0.4291	3.098
5	rs17617270	180582043	A	0.002577	0.0008333	G	0.4291	3.098
6	rs2231366	3129280	A	0.002577	0.0008333	G	0.4291	3.098
6	rs139625876	7231022	G	0.002577	0.0008333	A	0.4291	3.098
6	rs199850738	7373782	G	0.002577	0.0008333	A	0.4291	3.098
6	rs202232360	7581366	G	0.002577	0.0008333	A	0.4291	3.098

6	rs62621438	10702799	A	0.002577	0.0008333	G	0.4291	3.098
6	rs567083	10775600	A	0.002577	0.0008333	G	0.4291	3.098
6	rs55714642	11104469	A	0.002577	0.0008333	G	0.4291	3.098
6	rs147510333	12124633	A	0.002577	0.0008333	G	0.4291	3.098
6	rs16885	16306751	A	0.002577	0.0008333	G	0.4291	3.098
6	rs143314495	24436910	G	0.002577	0.0008333	A	0.4291	3.098
6	rs2817191	24564540	G	0.002577	0.0008333	A	0.4291	3.098
6	rs2744550	24564544	G	0.002577	0.0008333	A	0.4291	3.098
6	rs2744559	24576631	A	0.002577	0.0008333	G	0.4291	3.098
6	rs200727863	25811886	A	0.002577	0.0008333	G	0.4291	3.098
6	rs188397577	26017510	G	0.002577	0.0008333	A	0.4291	3.098
6	rs200077598	28251925	A	0.002577	0.0008333	G	0.4291	3.098
6	exm2257715	28297368	A	0.002577	0.0008333	G	0.4291	3.098
6	rs41269297	28297368	A	0.002577	0.0008333	G	0.4291	3.098
6	rs28986467	30675605	C	0.002577	0.0008333	A	0.4291	3.098
6	rs7742033	31085226	A	0.002577	0.0008333	G	0.4291	3.098
6	rs115591494	31599370	G	0.002577	0.0008333	C	0.4291	3.098
6	rs35595439	31605016	G	0.002577	0.0008333	A	0.4291	3.098
6	rs145921744	31646986	A	0.002577	0.0008333	G	0.4291	3.098
6	rs915653	31657047	G	0.002577	0.0008333	A	0.4291	3.098
6	rs9469058	31788050	A	0.002577	0.0008333	T	0.4291	3.098
6	rs9469063	31813576	C	0.002577	0.0008333	G	0.4291	3.098
6	rs147774384	31842575	A	0.002577	0.0008333	G	0.4291	3.098
6	rs9404991	32169288	G	0.002577	0.0008333	A	0.4291	3.098
6	rs9276	33053577	A	0.002577	0.0008333	G	0.4291	3.098
6	rs11965763	33076682	G	0.002577	0.0008333	A	0.4291	3.098
6	rs201179101	33148754	A	0.002577	0.0008333	G	0.4291	3.098
6	rs6907034	33252661	A	0.002577	0.0008333	G	0.4291	3.098
6	rs6900824	33302666	A	0.002577	0.0008333	T	0.4291	3.098
6	rs142313923	33371778	A	0.002577	0.0008333	C	0.4291	3.098
6	rs2228265	35253974	A	0.002577	0.0008333	G	0.4291	3.098
6	rs199713807	35554808	A	0.002577	0.0008333	G	0.4291	3.098
6	rs187241615	35716618	G	0.002577	0.0008333	A	0.4291	3.098
6	rs201419269	36343662	A	0.002577	0.0008333	G	0.4291	3.098
6	rs150277350	41126713	A	0.002577	0.0008333	G	0.4291	3.098
6	rs149152275	43012583	A	0.002577	0.0008333	G	0.4291	3.098
6	rs142657432	46661730	A	0.002577	0.0008333	G	0.4291	3.098
6	rs78333594	46851296	A	0.002577	0.0008333	G	0.4291	3.098
6	rs147190552	52144204	A	0.002577	0.0008333	G	0.4291	3.098
6	rs145364903	52546658	A	0.002577	0.0008333	G	0.4291	3.098
6	rs35862137	88240570	A	0.002577	0.0008333	G	0.4291	3.098
6	rs4140446	90448092	G	0.002577	0.0008333	A	0.4291	3.098
6	rs149506107	109871399	C	0.002577	0.0008333	A	0.4291	3.098

6	rs183827596	110759924	A	0.002577	0.0008333	T	0.4291	3.098
6	rs138153075	112486397	A	0.002577	0.0008333	G	0.4291	3.098
6	rs193203831	116879214	G	0.002577	0.0008333	A	0.4291	3.098
6	rs146135522	117050829	A	0.002577	0.0008333	G	0.4291	3.098
6	rs118009892	117114050	A	0.002577	0.0008333	G	0.4291	3.098
6	rs192842064	123122552	A	0.002577	0.0008333	G	0.4291	3.098
6	rs143638361	129828772	A	0.002577	0.0008333	G	0.4291	3.098
6	rs199509529	133014219	A	0.002577	0.0008333	G	0.4291	3.098
6	rs45624336	133015227	A	0.002577	0.0008333	G	0.4291	3.098
6	rs9321453	134773554	A	0.002577	0.0008333	G	0.4291	3.098
6	rs73774527	135306521	G	0.002577	0.0008333	A	0.4291	3.098
6	rs141597237	136599846	A	0.002577	0.0008333	G	0.4291	3.098
6	rs116499914	143823102	A	0.002577	0.0008333	G	0.4291	3.098
6	rs142207202	147680320	A	0.002577	0.0008333	G	0.4291	3.098
6	rs117534985	148711301	A	0.002577	0.0008333	G	0.4291	3.098
6	rs184093459	150211242	G	0.002577	0.0008333	A	0.4291	3.098
6	rs141729078	151670449	A	0.002577	0.0008333	G	0.4291	3.098
6	rs200135486	152536152	A	0.002577	0.0008333	G	0.4291	3.098
6	rs138487081	158900939	A	0.002577	0.0008333	G	0.4291	3.098
6	rs140821356	159398620	A	0.002577	0.0008333	G	0.4291	3.098
6	rs10455840	159401898	A	0.002577	0.0008333	G	0.4291	3.098
6	rs138993417	160328586	A	0.002577	0.0008333	G	0.4291	3.098
6	rs200398362	160963839	G	0.002577	0.0008333	A	0.4291	3.098
6	rs4252120	161143608	G	0.002577	0.0008333	A	0.4291	3.098
6	rs113290085	168443330	G	0.002577	0.0008333	A	0.4291	3.098
7	rs148035334	2748349	A	0.002577	0.0008333	G	0.4291	3.098
7	rs138116831	4050627	A	0.002577	0.0008333	G	0.4291	3.098
7	rs182694738	4820928	A	0.002577	0.0008333	G	0.4291	3.098
7	rs149672683	21469700	G	0.002577	0.0008333	A	0.4291	3.098
7	rs10252720	31618008	A	0.002577	0.0008333	G	0.4291	3.098
7	rs201502354	45003685	A	0.002577	0.0008333	G	0.4291	3.098
7	rs201196054	73011028	A	0.002577	0.0008333	G	0.4291	3.098
7	rs199567908	73245868	A	0.002577	0.0008333	G	0.4291	3.098
7	rs144099562	77265144	A	0.002577	0.0008333	G	0.4291	3.098
7	rs200694475	87020962	G	0.002577	0.0008333	C	0.4291	3.098
7	rs58238559	87082273	G	0.002577	0.0008333	A	0.4291	3.098
7	rs117676704	95442510	C	0.002577	0.0008333	G	0.4291	3.098
7	rs80338722	95813588	A	0.002577	0.0008333	G	0.4291	3.098
7	rs201287330	99221731	G	0.002577	0.0008333	A	0.4291	3.098
7	rs67784355	99359829	A	0.002577	0.0008333	G	0.4291	3.098
7	rs191137766	99778269	A	0.002577	0.0008333	C	0.4291	3.098
7	rs184812195	100229438	A	0.002577	0.0008333	G	0.4291	3.098
7	rs117406702	100346094	A	0.002577	0.0008333	G	0.4291	3.098

7	rs199517803	100682565	C	0.002577	0.0008333	A	0.4291	3.098
7	rs149830940	100685775	A	0.002577	0.0008333	C	0.4291	3.098
7	rs139945142	112424037	A	0.002577	0.0008333	G	0.4291	3.098
7	rs200462759	117368252	A	0.002577	0.0008333	G	0.4291	3.098
7	rs185248756	117407169	G	0.002577	0.0008333	A	0.4291	3.098
7	rs202057188	121733133	A	0.002577	0.0008333	T	0.4291	3.098
7	rs148407750	127892124	G	0.002577	0.0008333	A	0.4291	3.098
7	rs145413607	127999459	A	0.002577	0.0008333	G	0.4291	3.098
7	rs11983205	128531765	A	0.002577	0.0008333	G	0.4291	3.098
7	rs141924548	138732434	A	0.002577	0.0008333	G	0.4291	3.098
7	rs200011142	151878431	A	0.002577	0.0008333	G	0.4291	3.098
7	rs199796552	151884872	G	0.002577	0.0008333	A	0.4291	3.098
8	rs201454449	2999992	T	0.002577	0.0008333	A	0.4291	3.098
8	rs182635679	6669303	A	0.002577	0.0008333	C	0.4291	3.098
8	rs202162624	11421448	A	0.002577	0.0008333	G	0.4291	3.098
8	rs3947	11702375	A	0.002577	0.0008333	G	0.4291	3.098
8	rs138663166	23186030	A	0.002577	0.0008333	G	0.4291	3.098
8	rs200105783	28573810	A	0.002577	0.0008333	G	0.4291	3.098
8	rs145215857	30694962	C	0.002577	0.0008333	A	0.4291	3.098
8	rs144081400	35579908	A	0.002577	0.0008333	G	0.4291	3.098
8	rs191212056	52359659	A	0.002577	0.0008333	G	0.4291	3.098
8	rs7816287	103981314	G	0.002577	0.0008333	A	0.4291	3.098
8	rs118154190	104240224	A	0.002577	0.0008333	G	0.4291	3.098
8	rs35843564	106813586	A	0.002577	0.0008333	G	0.4291	3.098
8	rs185843292	109241340	G	0.002577	0.0008333	A	0.4291	3.098
8	rs199557734	110412464	A	0.002577	0.0008333	G	0.4291	3.098
8	rs199934029	122641109	A	0.002577	0.0008333	G	0.4291	3.098
8	rs115981067	133935617	A	0.002577	0.0008333	G	0.4291	3.098
8	rs56230101	133981760	G	0.002577	0.0008333	A	0.4291	3.098
8	rs201280992	134107420	A	0.002577	0.0008333	G	0.4291	3.098
8	rs146684203	135649838	A	0.002577	0.0008333	G	0.4291	3.098
8	rs200414597	144403532	G	0.002577	0.0008333	A	0.4291	3.098
8	rs192975604	144919856	A	0.002577	0.0008333	G	0.4291	3.098
9	rs3209441	286491	A	0.002577	0.0008333	G	0.4291	3.098
9	rs114872818	2643934	G	0.002577	0.0008333	C	0.4291	3.098
9	rs2169285	6280786	A	0.002577	0.0008333	G	0.4291	3.098
9	rs200642807	14842560	A	0.002577	0.0008333	G	0.4291	3.098
9	rs41269003	19060197	A	0.002577	0.0008333	T	0.4291	3.098
9	rs144642460	33466673	A	0.002577	0.0008333	G	0.4291	3.098
9	rs200064089	35045064	C	0.002577	0.0008333	G	0.4291	3.098
9	rs138357804	36222965	A	0.002577	0.0008333	G	0.4291	3.098
9	rs202063130	71628213	A	0.002577	0.0008333	G	0.4291	3.098
9	rs139432893	79117391	T	0.002577	0.0008333	A	0.4291	3.098

9	rs113761190	84606251	C	0.002577	0.0008333	A	0.4291	3.098
9	rs77416773	100890484	A	0.002577	0.0008333	G	0.4291	3.098
9	rs199985270	101052937	A	0.002577	0.0008333	G	0.4291	3.098
9	rs45455902	104171523	G	0.002577	0.0008333	A	0.4291	3.098
9	rs200509488	110068823	A	0.002577	0.0008333	G	0.4291	3.098
9	rs141324198	111665136	A	0.002577	0.0008333	G	0.4291	3.098
9	rs148086697	115651904	G	0.002577	0.0008333	A	0.4291	3.098
9	rs189249722	117848470	A	0.002577	0.0008333	G	0.4291	3.098
9	rs56188613	123782408	A	0.002577	0.0008333	C	0.4291	3.098
9	rs144581550	125551738	A	0.002577	0.0008333	G	0.4291	3.098
9	rs140089266	129102906	A	0.002577	0.0008333	G	0.4291	3.098
9	rs142148319	132377811	A	0.002577	0.0008333	G	0.4291	3.098
9	rs200121474	133943558	A	0.002577	0.0008333	G	0.4291	3.098
9	rs113975085	133952674	A	0.002577	0.0008333	G	0.4291	3.098
9	rs148728567	135273601	A	0.002577	0.0008333	G	0.4291	3.098
9	rs143189775	137809629	A	0.002577	0.0008333	G	0.4291	3.098
9	rs76349765	138516088	G	0.002577	0.0008333	A	0.4291	3.098
9	rs199943004	139277766	A	0.002577	0.0008333	G	0.4291	3.098
9	rs201808826	139299121	A	0.002577	0.0008333	G	0.4291	3.098
9	rs200623737	140174327	A	0.002577	0.0008333	G	0.4291	3.098
10	rs115840214	3199703	A	0.002577	0.0008333	G	0.4291	3.098
10	rs147954390	5791895	A	0.002577	0.0008333	G	0.4291	3.098
10	rs200352408	5959469	A	0.002577	0.0008333	G	0.4291	3.098
10	rs116378282	29811564	A	0.002577	0.0008333	G	0.4291	3.098
10	rs140481567	45941076	C	0.002577	0.0008333	A	0.4291	3.098
10	rs149209475	70863740	A	0.002577	0.0008333	G	0.4291	3.098
10	rs1111335	71018660	A	0.002577	0.0008333	G	0.4291	3.098
10	rs147264354	71391507	A	0.002577	0.0008333	G	0.4291	3.098
10	rs199799516	71647247	A	0.002577	0.0008333	G	0.4291	3.098
10	rs115220270	72298932	A	0.002577	0.0008333	G	0.4291	3.098
10	rs150484386	79742529	A	0.002577	0.0008333	G	0.4291	3.098
10	rs201263662	93221951	G	0.002577	0.0008333	A	0.4291	3.098
10	rs200549010	96081787	A	0.002577	0.0008333	G	0.4291	3.098
10	rs17383738	97772358	G	0.002577	0.0008333	A	0.4291	3.098
10	rs147702836	98095688	A	0.002577	0.0008333	G	0.4291	3.098
10	rs143401252	105138047	C	0.002577	0.0008333	G	0.4291	3.098
10	rs12782946	115922492	A	0.002577	0.0008333	G	0.4291	3.098
10	rs79009215	116045796	A	0.002577	0.0008333	G	0.4291	3.098
10	rs10490923	124214251	A	0.002577	0.0008333	G	0.4291	3.098
10	rs117762702	129914272	A	0.002577	0.0008333	G	0.4291	3.098
11	rs35482931	610724	A	0.002577	0.0008333	G	0.4291	3.098
11	rs202193629	831549	C	0.002577	0.0008333	G	0.4291	3.098
11	rs201593546	1254356	A	0.002577	0.0008333	G	0.4291	3.098

11	rs57724831	3691024	C	0.002577	0.0008333	A	0.4291	3.098
11	rs149144644	5602461	C	0.002577	0.0008333	A	0.4291	3.098
11	rs140094304	6898789	A	0.002577	0.0008333	G	0.4291	3.098
11	rs202106934	8959255	G	0.002577	0.0008333	C	0.4291	3.098
11	rs144109761	9459831	A	0.002577	0.0008333	G	0.4291	3.098
11	rs114382440	15247313	A	0.002577	0.0008333	G	0.4291	3.098
11	rs150856594	18566292	A	0.002577	0.0008333	G	0.4291	3.098
11	rs3026404	31804978	C	0.002577	0.0008333	G	0.4291	3.098
11	rs140188192	34129779	A	0.002577	0.0008333	G	0.4291	3.098
11	rs201179105	35513671	A	0.002577	0.0008333	C	0.4291	3.098
11	rs200557252	47861581	G	0.002577	0.0008333	A	0.4291	3.098
11	rs201618586	48142818	A	0.002577	0.0008333	G	0.4291	3.098
11	rs140161944	48285558	A	0.002577	0.0008333	G	0.4291	3.098
11	rs185143469	48285846	G	0.002577	0.0008333	A	0.4291	3.098
11	rs148311440	50003290	A	0.002577	0.0008333	G	0.4291	3.098
11	rs140532711	59857166	A	0.002577	0.0008333	G	0.4291	3.098
11	rs141543926	60152690	C	0.002577	0.0008333	G	0.4291	3.098
11	rs34209302	60892605	A	0.002577	0.0008333	G	0.4291	3.098
11	rs145029610	63066472	A	0.002577	0.0008333	G	0.4291	3.098
11	rs201804022	63149670	A	0.002577	0.0008333	C	0.4291	3.098
11	rs146903011	63585359	C	0.002577	0.0008333	G	0.4291	3.098
11	rs200059663	64122747	A	0.002577	0.0008333	G	0.4291	3.098
11	rs146240427	64985107	A	0.002577	0.0008333	G	0.4291	3.098
11	rs200119718	70013464	A	0.002577	0.0008333	G	0.4291	3.098
11	rs151173629	71734115	A	0.002577	0.0008333	G	0.4291	3.098
11	rs70940819	71946912	G	0.002577	0.0008333	A	0.4291	3.098
11	rs34841082	73915481	G	0.002577	0.0008333	A	0.4291	3.098
11	rs200207753	76910709	A	0.002577	0.0008333	G	0.4291	3.098
11	rs141677387	83641480	A	0.002577	0.0008333	G	0.4291	3.098
11	exm2250396	88911169	A	0.002577	0.0008333	G	0.4291	3.098
11	rs148710429	88911169	A	0.002577	0.0008333	G	0.4291	3.098
11	rs200763217	92534418	A	0.002577	0.0008333	G	0.4291	3.098
11	rs77315074	101765654	A	0.002577	0.0008333	G	0.4291	3.098
11	rs147267769	102643604	A	0.002577	0.0008333	C	0.4291	3.098
11	rs192172142	111784570	A	0.002577	0.0008333	G	0.4291	3.098
11	rs180358	116599392	A	0.002577	0.0008333	G	0.4291	3.098
11	rs139099960	116633322	A	0.002577	0.0008333	G	0.4291	3.098
11	rs201007236	123777296	A	0.002577	0.0008333	G	0.4291	3.098
11	rs201358968	123887117	A	0.002577	0.0008333	G	0.4291	3.098
11	rs79210019	126139156	A	0.002577	0.0008333	C	0.4291	3.098
11	rs200789628	126333139	A	0.002577	0.0008333	C	0.4291	3.098
11	rs141934398	134240289	A	0.002577	0.0008333	G	0.4291	3.098
12	rs10735005	1995403	A	0.002577	0.0008333	G	0.4291	3.098

12	rs202035339	6623492	A	0.002577	0.0008333	G	0.4291	3.098
12	rs149811742	7045127	G	0.002577	0.0008333	A	0.4291	3.098
12	rs185520750	7476127	A	0.002577	0.0008333	C	0.4291	3.098
12	rs61584783	7477100	A	0.002577	0.0008333	G	0.4291	3.098
12	rs188675162	9875338	G	0.002577	0.0008333	A	0.4291	3.098
12	rs145803876	10163422	A	0.002577	0.0008333	G	0.4291	3.098
12	rs189582628	10167986	G	0.002577	0.0008333	A	0.4291	3.098
12	rs139727620	12870998	C	0.002577	0.0008333	G	0.4291	3.098
12	rs117330813	18891318	A	0.002577	0.0008333	G	0.4291	3.098
12	rs200994482	21377774	A	0.002577	0.0008333	G	0.4291	3.098
12	rs138278747	21639508	A	0.002577	0.0008333	G	0.4291	3.098
12	rs181888536	39761807	G	0.002577	0.0008333	A	0.4291	3.098
12	rs56312115	44161927	G	0.002577	0.0008333	A	0.4291	3.098
12	rs118033428	46244712	A	0.002577	0.0008333	C	0.4291	3.098
12	rs75531856	48596546	A	0.002577	0.0008333	G	0.4291	3.098
12	rs17823193	48596890	A	0.002577	0.0008333	G	0.4291	3.098
12	rs2732441	48723324	C	0.002577	0.0008333	G	0.4291	3.098
12	rs75937132	49430947	G	0.002577	0.0008333	A	0.4291	3.098
12	rs117761535	50357924	A	0.002577	0.0008333	G	0.4291	3.098
12	rs200914206	51739602	A	0.002577	0.0008333	G	0.4291	3.098
12	rs192947508	52711539	A	0.002577	0.0008333	G	0.4291	3.098
12	rs186113671	58204613	A	0.002577	0.0008333	G	0.4291	3.098
12	rs76408141	66605329	A	0.002577	0.0008333	C	0.4291	3.098
12	rs12580996	67699856	G	0.002577	0.0008333	A	0.4291	3.098
12	rs201628210	95645827	G	0.002577	0.0008333	C	0.4291	3.098
12	rs142475910	105515947	C	0.002577	0.0008333	A	0.4291	3.098
12	rs7300972	109017264	G	0.002577	0.0008333	A	0.4291	3.098
12	rs61729674	109017266	A	0.002577	0.0008333	G	0.4291	3.098
12	exm1034255	109017560	A	0.002577	0.0008333	G	0.4291	3.098
12	rs8179116	109025427	A	0.002577	0.0008333	G	0.4291	3.098
12	rs117096112	109221123	A	0.002577	0.0008333	G	0.4291	3.098
12	rs3184504	111884608	A	0.002577	0.0008333	G	0.4291	3.098
12	rs10774625	111910219	A	0.002577	0.0008333	G	0.4291	3.098
12	rs653178	112007756	G	0.002577	0.0008333	A	0.4291	3.098
12	rs141477684	112699118	A	0.002577	0.0008333	G	0.4291	3.098
12	rs1293767	113425154	C	0.08505	0.1	G	0.4291	0.8366
12	rs200347609	113871944	A	0.002577	0.0008333	G	0.4291	3.098
12	rs138430777	120214173	G	0.002577	0.0008333	A	0.4291	3.098
12	rs200245091	120572361	A	0.002577	0.0008333	G	0.4291	3.098
12	rs28360447	121600238	A	0.002577	0.0008333	G	0.4291	3.098
12	rs200180376	123481962	A	0.002577	0.0008333	G	0.4291	3.098
12	rs143052054	124289423	A	0.002577	0.0008333	C	0.4291	3.098
12	rs147653672	132238932	A	0.002577	0.0008333	G	0.4291	3.098

12	rs140505420	132238953	A	0.002577	0.0008333	G	0.4291	3.098
12	rs12423664	133069894	A	0.002577	0.0008333	G	0.4291	3.098
13	rs191372140	22084195	A	0.002577	0.0008333	G	0.4291	3.098
13	rs17369571	22487625	A	0.002577	0.0008333	G	0.4291	3.098
13	rs143197066	31233231	A	0.002577	0.0008333	G	0.4291	3.098
13	rs56191579	32914623	A	0.002577	0.0008333	C	0.4291	3.098
13	rs192704075	36878734	A	0.002577	0.0008333	G	0.4291	3.098
13	rs200094541	50265556	A	0.002577	0.0008333	C	0.4291	3.098
13	rs149821147	75887003	G	0.002577	0.0008333	A	0.4291	3.098
13	rs41281664	103396628	A	0.002577	0.0008333	G	0.4291	3.098
14	rs181753558	20482440	A	0.002577	0.0008333	G	0.4291	3.098
14	rs17114309	21108965	A	0.002577	0.0008333	G	0.4291	3.098
14	rs115891437	23236598	G	0.002577	0.0008333	A	0.4291	3.098
14	rs141730904	24507114	A	0.002577	0.0008333	C	0.4291	3.098
14	rs201803784	45657020	A	0.002577	0.0008333	G	0.4291	3.098
14	rs145204580	51057682	A	0.002577	0.0008333	C	0.4291	3.098
14	rs144461334	52493997	A	0.002577	0.0008333	G	0.4291	3.098
14	rs140026883	59014522	A	0.002577	0.0008333	G	0.4291	3.098
14	exm1109025	66096297	A	0.002577	0.0008333	G	0.4291	3.098
14	rs35137471	66096297	A	0.002577	0.0008333	G	0.4291	3.098
14	rs138742665	69792727	A	0.002577	0.0008333	G	0.4291	3.098
14	rs147946137	73137890	A	0.002577	0.0008333	G	0.4291	3.098
14	rs76172717	74978010	C	0.002577	0.0008333	G	0.4291	3.098
14	rs114347531	75558058	A	0.002577	0.0008333	G	0.4291	3.098
14	rs186804111	77944602	A	0.002577	0.0008333	G	0.4291	3.098
14	rs1805078	88450770	A	0.002577	0.0008333	G	0.4291	3.098
14	rs114142372	91779956	A	0.002577	0.0008333	G	0.4291	3.098
14	rs139055497	95085651	A	0.002577	0.0008333	G	0.4291	3.098
14	rs34137879	105965102	A	0.002577	0.0008333	G	0.4291	3.098
15	rs1448484	28283441	G	0.002577	0.0008333	A	0.4291	3.098
15	rs144119621	41508002	C	0.002577	0.0008333	A	0.4291	3.098
15	rs116848967	48512855	A	0.002577	0.0008333	G	0.4291	3.098
15	rs201118855	51507935	G	0.002577	0.0008333	A	0.4291	3.098
15	rs180954449	51696692	A	0.002577	0.0008333	G	0.4291	3.098
15	rs199588777	65214156	A	0.002577	0.0008333	C	0.4291	3.098
15	rs191444296	68497632	A	0.002577	0.0008333	G	0.4291	3.098
15	rs45442501	75015036	A	0.002577	0.0008333	G	0.4291	3.098
15	rs200789139	75042198	G	0.002577	0.0008333	A	0.4291	3.098
15	rs1989	83781631	A	0.002577	0.0008333	G	0.4291	3.098
15	rs201410488	85198599	G	0.002577	0.0008333	A	0.4291	3.098
15	rs200413336	86312617	G	0.002577	0.0008333	A	0.4291	3.098
15	rs201274525	89182820	C	0.002577	0.0008333	G	0.4291	3.098
15	rs142991295	89736497	A	0.002577	0.0008333	G	0.4291	3.098

15	rs200018187	90129090	A	0.002577	0.0008333	G	0.4291	3.098
15	rs146924321	101775405	A	0.002577	0.0008333	G	0.4291	3.098
15	rs199625817	101938651	A	0.002577	0.0008333	G	0.4291	3.098
15	rs200649259	102346619	G	0.002577	0.0008333	A	0.4291	3.098
16	rs146067716	772978	A	0.002577	0.0008333	G	0.4291	3.098
16	rs142369196	1869210	A	0.002577	0.0008333	G	0.4291	3.098
16	rs199702402	2030055	G	0.002577	0.0008333	A	0.4291	3.098
16	rs185364182	3139190	A	0.002577	0.0008333	C	0.4291	3.098
16	rs201495311	3139999	A	0.002577	0.0008333	C	0.4291	3.098
16	rs199775931	4254629	A	0.002577	0.0008333	G	0.4291	3.098
16	rs78074706	4755108	A	0.002577	0.0008333	G	0.4291	3.098
16	rs199905767	5061153	A	0.002577	0.0008333	C	0.4291	3.098
16	rs142791436	8890443	A	0.002577	0.0008333	G	0.4291	3.098
16	rs200415151	15729838	G	0.002577	0.0008333	A	0.4291	3.098
16	rs77634238	21123315	A	0.002577	0.0008333	G	0.4291	3.098
16	rs61744122	22157582	A	0.002577	0.0008333	C	0.4291	3.098
16	rs72770407	24800897	A	0.002577	0.0008333	G	0.4291	3.098
16	rs72770408	24801468	C	0.002577	0.0008333	G	0.4291	3.098
16	rs138308875	27078611	A	0.002577	0.0008333	G	0.4291	3.098
16	rs200237368	27499714	C	0.002577	0.0008333	G	0.4291	3.098
16	rs117350233	28905848	A	0.002577	0.0008333	G	0.4291	3.098
16	rs201622634	29853160	A	0.002577	0.0008333	G	0.4291	3.098
16	rs17855606	31142271	G	0.002577	0.0008333	A	0.4291	3.098
16	rs59889488	31391887	A	0.002577	0.0008333	G	0.4291	3.098
16	rs200235612	31418934	G	0.002577	0.0008333	A	0.4291	3.098
16	rs146149363	48210877	A	0.002577	0.0008333	G	0.4291	3.098
16	rs202220919	50342686	A	0.002577	0.0008333	C	0.4291	3.098
16	rs149394680	57764957	A	0.002577	0.0008333	G	0.4291	3.098
16	rs140803819	66946471	A	0.002577	0.0008333	G	0.4291	3.098
16	rs201203484	67324822	A	0.002577	0.0008333	C	0.4291	3.098
16	rs199747574	69350175	A	0.002577	0.0008333	G	0.4291	3.098
16	rs199749272	69729182	A	0.002577	0.0008333	C	0.4291	3.098
16	rs149553279	70503178	A	0.002577	0.0008333	C	0.4291	3.098
16	rs200083914	70515032	A	0.002577	0.0008333	G	0.4291	3.098
16	rs201571332	70897063	A	0.002577	0.0008333	G	0.4291	3.098
16	rs200979879	70993639	A	0.002577	0.0008333	G	0.4291	3.098
16	rs116978477	71668552	A	0.002577	0.0008333	G	0.4291	3.098
16	rs145888651	88779790	A	0.002577	0.0008333	G	0.4291	3.098
16	rs200779411	88781475	A	0.002577	0.0008333	G	0.4291	3.098
16	rs191519947	88909215	G	0.002577	0.0008333	A	0.4291	3.098
16	rs200088367	89293520	G	0.002577	0.0008333	A	0.4291	3.098
16	rs201473247	90128558	A	0.002577	0.0008333	G	0.4291	3.098
17	exm1878310	1961223	A	0.002577	0.0008333	G	0.4291	3.098

17	rs199689510	1961223	A	0.002577	0.0008333	G	0.4291	3.098
17	rs201868792	3119840	A	0.002577	0.0008333	G	0.4291	3.098
17	rs188168113	3421956	A	0.002577	0.0008333	G	0.4291	3.098
17	rs56224301	3628076	A	0.002577	0.0008333	G	0.4291	3.098
17	rs150892365	3977620	A	0.002577	0.0008333	G	0.4291	3.098
17	rs149528100	4337384	A	0.002577	0.0008333	G	0.4291	3.098
17	rs9436	4574751	A	0.002577	0.0008333	T	0.4291	3.098
17	rs201480696	7948257	A	0.002577	0.0008333	C	0.4291	3.098
17	rs200806151	7950661	A	0.002577	0.0008333	T	0.4291	3.098
17	rs61521510	10348150	A	0.002577	0.0008333	G	0.4291	3.098
17	rs142427652	11896106	A	0.002577	0.0008333	G	0.4291	3.098
17	rs141020107	17924535	A	0.002577	0.0008333	C	0.4291	3.098
17	rs199587101	18052221	G	0.002577	0.0008333	A	0.4291	3.098
17	rs185227361	18220133	G	0.002577	0.0008333	A	0.4291	3.098
17	rs148686581	26657491	C	0.002577	0.0008333	A	0.4291	3.098
17	rs201828647	27182063	A	0.002577	0.0008333	G	0.4291	3.098
17	rs180817786	29632309	G	0.002577	0.0008333	A	0.4291	3.098
17	rs200269504	31322699	A	0.002577	0.0008333	G	0.4291	3.098
17	rs147497902	33491181	G	0.002577	0.0008333	A	0.4291	3.098
17	rs139899726	33768368	A	0.002577	0.0008333	G	0.4291	3.098
17	rs200046706	34171583	G	0.002577	0.0008333	A	0.4291	3.098
17	rs200136431	36485693	A	0.002577	0.0008333	G	0.4291	3.098
17	rs189784680	38430059	A	0.002577	0.0008333	C	0.4291	3.098
17	rs200843429	39635169	A	0.002577	0.0008333	G	0.4291	3.098
17	rs192328036	39635214	A	0.002577	0.0008333	G	0.4291	3.098
17	rs34353668	40120702	G	0.002577	0.0008333	A	0.4291	3.098
17	rs200095635	40341894	A	0.002577	0.0008333	G	0.4291	3.098
17	rs138005262	42155892	A	0.002577	0.0008333	G	0.4291	3.098
17	rs147019251	48174864	G	0.002577	0.0008333	A	0.4291	3.098
17	rs202105387	48539903	C	0.002577	0.0008333	A	0.4291	3.098
17	rs201461737	49245648	A	0.002577	0.0008333	G	0.4291	3.098
17	rs185658468	58235051	A	0.002577	0.0008333	T	0.4291	3.098
17	rs61747443	61899452	G	0.002577	0.0008333	A	0.4291	3.098
17	rs200082532	65734425	G	0.002577	0.0008333	A	0.4291	3.098
17	rs192451785	71233128	G	0.002577	0.0008333	A	0.4291	3.098
17	rs35467001	71380062	A	0.002577	0.0008333	G	0.4291	3.098
17	rs201256089	71397190	A	0.002577	0.0008333	G	0.4291	3.098
17	rs140270094	73497583	A	0.002577	0.0008333	C	0.4291	3.098
17	rs78367086	73560601	A	0.002577	0.0008333	G	0.4291	3.098
17	rs11651675	76134237	A	0.002577	0.0008333	G	0.4291	3.098
17	rs186768969	76528637	A	0.002577	0.0008333	G	0.4291	3.098
17	rs143523371	78078931	A	0.002577	0.0008333	G	0.4291	3.098
17	rs183141669	79093324	A	0.002577	0.0008333	G	0.4291	3.098

17	rs200375025	79428143	A	0.002577	0.0008333	G	0.4291	3.098
17	rs148878409	80706786	A	0.002577	0.0008333	C	0.4291	3.098
18	rs142044547	20573213	G	0.002577	0.0008333	A	0.4291	3.098
18	rs146716628	29793475	A	0.002577	0.0008333	G	0.4291	3.098
18	rs201460958	32844108	A	0.002577	0.0008333	G	0.4291	3.098
18	rs144644128	33793426	G	0.002577	0.0008333	A	0.4291	3.098
18	rs36086089	44143153	A	0.002577	0.0008333	G	0.4291	3.098
18	rs138848935	47778134	A	0.002577	0.0008333	G	0.4291	3.098
18	rs202107348	54363541	G	0.002577	0.0008333	C	0.4291	3.098
18	rs184467284	56203365	G	0.002577	0.0008333	A	0.4291	3.098
18	rs141329024	56414794	G	0.002577	0.0008333	A	0.4291	3.098
18	rs144167768	59483248	A	0.002577	0.0008333	G	0.4291	3.098
18	rs7238987	71928150	A	0.08505	0.1	G	0.4291	0.8366
18	rs201810396	72226583	G	0.002577	0.0008333	A	0.4291	3.098
19	rs201725343	643333	A	0.002577	0.0008333	G	0.4291	3.098
19	rs61738004	875245	A	0.002577	0.0008333	G	0.4291	3.098
19	rs200726568	2191238	A	0.002577	0.0008333	G	0.4291	3.098
19	rs199674173	2852503	A	0.002577	0.0008333	G	0.4291	3.098
19	rs200032828	3807217	A	0.002577	0.0008333	G	0.4291	3.098
19	rs200345987	4171872	A	0.002577	0.0008333	C	0.4291	3.098
19	rs150835577	4428739	A	0.002577	0.0008333	G	0.4291	3.098
19	rs201365136	5131539	A	0.002577	0.0008333	G	0.4291	3.098
19	rs149195870	6333498	A	0.002577	0.0008333	G	0.4291	3.098
19	rs138460722	8438643	A	0.002577	0.0008333	G	0.4291	3.098
19	rs61734967	8973559	G	0.002577	0.0008333	A	0.4291	3.098
19	rs187293598	9006664	A	0.002577	0.0008333	G	0.4291	3.098
19	rs7249989	10097073	A	0.002577	0.0008333	G	0.4291	3.098
19	rs147221323	13318811	A	0.002577	0.0008333	G	0.4291	3.098
19	rs4239634	17746947	A	0.002577	0.0008333	G	0.4291	3.098
19	rs199625640	18709278	A	0.002577	0.0008333	G	0.4291	3.098
19	rs116791423	19675769	A	0.002577	0.0008333	C	0.4291	3.098
19	rs182604067	35828903	A	0.002577	0.0008333	G	0.4291	3.098
19	rs199824317	39371790	A	0.002577	0.0008333	G	0.4291	3.098
19	rs144382583	40886586	A	0.002577	0.0008333	G	0.4291	3.098
19	rs202065092	40900984	C	0.002577	0.0008333	A	0.4291	3.098
19	rs34826503	41518244	A	0.002577	0.0008333	G	0.4291	3.098
19	rs239942	44377357	G	0.002577	0.0008333	A	0.4291	3.098
19	rs76483217	44377614	A	0.002577	0.0008333	G	0.4291	3.098
19	rs142426196	44611383	A	0.002577	0.0008333	G	0.4291	3.098
19	rs144032518	44611656	A	0.002577	0.0008333	G	0.4291	3.098
19	rs200818596	45540799	G	0.002577	0.0008333	A	0.4291	3.098
19	rs150205438	46376202	C	0.002577	0.0008333	G	0.4291	3.098
19	rs1800030	49207095	A	0.002577	0.0008333	G	0.4291	3.098

19	rs200962284	51453297	G	0.002577	0.0008333	A	0.4291	3.098
19	rs184499009	53645528	C	0.002577	0.0008333	G	0.4291	3.098
19	rs142185185	54695789	C	0.002577	0.0008333	A	0.4291	3.098
19	rs193018967	55378173	C	0.002577	0.0008333	A	0.4291	3.098
19	rs201327656	56203144	A	0.002577	0.0008333	G	0.4291	3.098
19	rs199861211	57646716	A	0.002577	0.0008333	G	0.4291	3.098
19	rs186801815	57868457	G	0.002577	0.0008333	A	0.4291	3.098
19	rs199955971	58755399	A	0.002577	0.0008333	G	0.4291	3.098
19	rs200679930	58921357	A	0.002577	0.0008333	G	0.4291	3.098
20	rs199507287	3760323	A	0.002577	0.0008333	G	0.4291	3.098
20	rs150798461	17475569	G	0.002577	0.0008333	A	0.4291	3.098
20	rs200476717	18470608	A	0.002577	0.0008333	G	0.4291	3.098
20	rs379538	25481622	G	0.002577	0.0008333	A	0.4291	3.098
20	rs34482966	30385267	A	0.002577	0.0008333	G	0.4291	3.098
20	rs199912788	36694484	A	0.002577	0.0008333	G	0.4291	3.098
20	rs199943093	47851538	A	0.002577	0.0008333	G	0.4291	3.098
20	rs140863452	49227439	A	0.002577	0.0008333	G	0.4291	3.098
20	rs146782561	52198285	A	0.002577	0.0008333	G	0.4291	3.098
20	rs115260488	52786155	A	0.002577	0.0008333	G	0.4291	3.098
20	rs200019171	60991235	A	0.002577	0.0008333	G	0.4291	3.098
20	rs115976084	61525165	A	0.002577	0.0008333	G	0.4291	3.098
20	rs201141635	62190688	A	0.002577	0.0008333	G	0.4291	3.098
21	rs144443158	35147404	C	0.002577	0.0008333	G	0.4291	3.098
21	rs144738332	37619914	G	0.002577	0.0008333	C	0.4291	3.098
21	rs146658168	43412853	A	0.002577	0.0008333	G	0.4291	3.098
21	rs200792922	43829717	A	0.002577	0.0008333	G	0.4291	3.098
21	rs141352481	45678440	A	0.002577	0.0008333	G	0.4291	3.098
21	rs77759997	45825855	A	0.002577	0.0008333	G	0.4291	3.098
21	rs138384392	46117123	A	0.002577	0.0008333	G	0.4291	3.098
21	rs113169531	47552423	A	0.002577	0.0008333	G	0.4291	3.098
21	rs146189038	47775425	A	0.002577	0.0008333	G	0.4291	3.098
21	rs200629608	47969809	A	0.002577	0.0008333	G	0.4291	3.098
22	rs148317214	19125772	A	0.002577	0.0008333	G	0.4291	3.098
22	exm1585000	19220066	A	0.002577	0.0008333	G	0.4291	3.098
22	rs148779081	19483552	A	0.002577	0.0008333	G	0.4291	3.098
22	rs200986651	19504398	G	0.002577	0.0008333	A	0.4291	3.098
22	rs139920129	19965501	A	0.002577	0.0008333	G	0.4291	3.098
22	rs201488040	21988422	A	0.002577	0.0008333	G	0.4291	3.098
22	rs200749476	28378445	G	0.002577	0.0008333	A	0.4291	3.098
22	rs200628910	29737551	G	0.002577	0.0008333	A	0.4291	3.098
22	rs200693435	32007224	A	0.002577	0.0008333	C	0.4291	3.098
22	rs199849700	32009612	A	0.002577	0.0008333	G	0.4291	3.098
22	rs199554283	35779124	A	0.002577	0.0008333	C	0.4291	3.098

22	rs143532979	37261015	A	0.002577	0.0008333	G	0.4291	3.098
22	rs145073875	37492731	A	0.002577	0.0008333	G	0.4291	3.098
22	rs138108680	37962756	A	0.002577	0.0008333	G	0.4291	3.098
22	rs201794535	39146966	A	0.002577	0.0008333	G	0.4291	3.098
22	rs202034135	40802115	A	0.002577	0.0008333	G	0.4291	3.098
22	rs200656934	43926741	G	0.002577	0.0008333	A	0.4291	3.098
22	rs146945151	45204240	G	0.002577	0.0008333	A	0.4291	3.098
22	rs73176189	45255688	A	0.002577	0.0008333	G	0.4291	3.098
22	rs200519592	51143462	A	0.002577	0.0008333	G	0.4291	3.098
5	rs6898138	160343115	G	0.3763	0.3539	A	0.4293	1.101
13	rs2483424	39359708	G	0.3428	0.3667	A	0.4293	0.9009
17	rs3744644	1538628	G	0.06186	0.07513	C	0.4293	0.8117
17	exm1296274	12656111	G	0.1933	0.2137	A	0.4293	0.8817
2	rs3739011	101624384	G	0.06186	0.075	A	0.4294	0.8132
3	rs6785339	196054439	A	0.06186	0.075	C	0.4294	0.8132
6	rs72502540	31000005	T	0.06186	0.075	A	0.4294	0.8132
20	rs3810574	23345589	G	0.06186	0.075	A	0.4294	0.8132
3	rs201868429	184428747	A	0.002577	0.0008347	G	0.4295	3.093
6	rs802734	128278798	G	0.1753	0.1583	A	0.4295	1.13
7	rs143150225	80292448	A	0.002577	0.0008347	G	0.4295	3.093
7	rs200318159	134853622	A	0.002577	0.0008347	G	0.4295	3.093
9	rs201030657	139277793	A	0.002577	0.0008347	G	0.4295	3.093
11	rs10794324	428489	A	0.002577	0.0008347	G	0.4295	3.093
13	rs143452573	42264331	A	0.002577	0.0008347	G	0.4295	3.093
16	rs141101134	3063683	A	0.002577	0.0008347	C	0.4295	3.093
17	rs139395451	42019010	A	0.002577	0.0008347	G	0.4295	3.093
17	rs201890212	48183455	A	0.002577	0.0008347	G	0.4295	3.093
19	rs200774437	2271924	A	0.002577	0.0008347	C	0.4295	3.093
1	rs6691117	207782931	G	0.2242	0.2042	A	0.4296	1.127
2	rs766432	60719970	C	0.2242	0.2042	A	0.4296	1.127
3	rs2306272	66434643	A	0.2526	0.2742	G	0.4296	0.8946
4	rs66538112	10447168	G	0.2242	0.2042	C	0.4296	1.127
10	rs56206226	50531549	A	0.3789	0.3558	C	0.4296	1.104
10	rs55780106	50531606	A	0.3789	0.3558	C	0.4296	1.104
10	rs45554335	50531972	C	0.3789	0.3558	A	0.4296	1.104
1	rs1150258	207074905	G	0.1933	0.2125	A	0.4297	0.888
3	rs7632426	122296647	A	0.1933	0.2125	G	0.4297	0.888
4	rs60640167	36317965	G	0.08505	0.1008	A	0.4297	0.8289
7	rs6943153	50791579	A	0.2526	0.275	G	0.4297	0.8909
8	rs323375	30731171	G	0.1933	0.2125	A	0.4297	0.888
12	rs11276	14993439	A	0.08505	0.1008	G	0.4297	0.8289
21	rs9306171	45458778	A	0.2526	0.275	G	0.4297	0.8909
17	exm1315128	36485693	A	0.002604	0.0008446	G	0.4299	3.089

1	rs187082229	185175856	A	0.002577	0.0008361	C	0.43	3.088
6	rs2273962	41318438	A	0.1495	0.1683	G	0.43	0.8683
9	rs4076795	90501448	C	0.1495	0.1675	A	0.43	0.8735
10	rs72559710	135342034	A	0.002577	0.0008361	G	0.43	3.088
16	rs2071979	624114	A	0.3454	0.3692	G	0.43	0.9015
16	rs200611878	847871	A	0.002577	0.0008361	G	0.43	3.088
16	rs199513661	920844	A	0.002577	0.0008361	G	0.43	3.088
1	rs12037377	246714520	A	0.05928	0.04917	G	0.4301	1.219
5	rs154001	127685135	G	0.05928	0.04917	A	0.4301	1.219
13	rs3910433	26793644	G	0.05928	0.04917	A	0.4301	1.219
1	rs277665	6814758	G	0.3454	0.3683	A	0.4302	0.9047
1	rs3738115	180165699	C	0.2861	0.265	A	0.4302	1.111
3	rs4682506	113586776	G	0.3454	0.3683	A	0.4302	0.9047
6	rs9277934	33153528	A	0.2861	0.265	G	0.4302	1.111
10	rs4747647	8006798	A	0.2861	0.265	G	0.4302	1.111
10	rs77961654	114925369	A	0.2861	0.265	C	0.4302	1.111
11	rs757081	17351683	G	0.3814	0.3583	C	0.4302	1.104
16	rs1045277	633125	A	0.3454	0.3683	G	0.4302	0.9047
6	rs3132672	30175847	C	0.3789	0.3567	A	0.4303	1.1
6	rs3130383	30179026	C	0.3789	0.3567	A	0.4303	1.1
21	rs2826891	22910116	A	0.2242	0.205	G	0.4303	1.121
6	rs2032567	112457390	G	0.1392	0.1233	A	0.4304	1.149
11	rs4075289	830670	C	0.08247	0.0694	A	0.4304	1.205
6	rs200928800	42893226	A	0.002577	0.0008375	G	0.4305	3.083
22	rs201519448	50752105	A	0.002577	0.0008375	G	0.4305	3.083
6	rs209474	32924584	G	0.3479	0.3717	A	0.4307	0.9021
6	rs206786	32935179	C	0.3479	0.3717	A	0.4307	0.9021
6	rs9493627	133789728	A	0.3479	0.3717	G	0.4307	0.9021
14	rs2236316	51224417	G	0.1778	0.16	C	0.4308	1.136
1	rs142471602	24447657	G	0.01546	0.01083	A	0.4309	1.434
1	rs146782110	151107140	C	0.01546	0.01083	A	0.4309	1.434
1	rs200633919	161696663	A	0.01546	0.01083	G	0.4309	1.434
1	rs75832955	235597525	A	0.01546	0.01083	G	0.4309	1.434
2	rs149731356	58392880	G	0.01546	0.01083	A	0.4309	1.434
2	rs13407355	71740953	G	0.01546	0.01083	C	0.4309	1.434
2	rs75780727	238280366	A	0.01546	0.01083	G	0.4309	1.434
3	rs143182914	44489059	G	0.01546	0.01083	A	0.4309	1.434
3	rs182965524	50391017	G	0.01546	0.01083	A	0.4309	1.434
3	rs55993015	134898754	A	0.01546	0.01083	G	0.4309	1.434
3	rs9815702	163735214	A	0.3479	0.3708	G	0.4309	0.9053
4	rs144839123	6711295	G	0.01546	0.01083	A	0.4309	1.434
4	rs137874988	78871021	G	0.01546	0.01083	C	0.4309	1.434
5	rs9292342	29806888	A	0.3814	0.3592	G	0.4309	1.1

5	rs56337909	66460595	A	0.01546	0.01083	G	0.4309	1.434
5	rs77758389	175740782	A	0.01546	0.01083	G	0.4309	1.434
6	rs140966197	35765001	A	0.01546	0.01083	G	0.4309	1.434
6	rs78446650	54025539	G	0.01546	0.01083	A	0.4309	1.434
6	rs12662753	97533027	G	0.01546	0.01083	A	0.4309	1.434
6	rs118003870	138752051	C	0.01546	0.01083	G	0.4309	1.434
6	rs55704622	161512446	A	0.01546	0.01083	T	0.4309	1.434
7	rs4723010	30791848	A	0.01546	0.01083	G	0.4309	1.434
8	rs7465584	124987478	G	0.3814	0.3592	A	0.4309	1.1
9	exm2259351	36669341	G	0.01546	0.01083	A	0.4309	1.434
9	rs56176668	36669341	G	0.01546	0.01083	A	0.4309	1.434
10	rs41277370	21134282	G	0.01546	0.01083	C	0.4309	1.434
10	rs4025981	21139389	G	0.01546	0.01083	A	0.4309	1.434
10	rs116844906	24790335	G	0.01546	0.01083	A	0.4309	1.434
10	rs59446434	124325512	A	0.01546	0.01083	G	0.4309	1.434
11	rs77344525	2424105	G	0.01546	0.01083	A	0.4309	1.434
11	rs147597453	6567826	A	0.01546	0.01083	G	0.4309	1.434
11	exm2250347	8251900	A	0.01546	0.01083	G	0.4309	1.434
11	rs138302396	8251900	A	0.01546	0.01083	G	0.4309	1.434
11	rs200873091	129742837	C	0.01546	0.01083	G	0.4309	1.434
12	rs4454801	88414188	G	0.01546	0.01083	A	0.4309	1.434
12	rs149169269	130934799	G	0.01546	0.01083	A	0.4309	1.434
13	rs56255799	103510736	A	0.01546	0.01083	G	0.4309	1.434
13	rs4150313	103513951	G	0.01546	0.01083	A	0.4309	1.434
14	rs28711516	23876267	A	0.01546	0.01083	G	0.4309	1.434
14	rs141215765	75537429	C	0.01546	0.01083	G	0.4309	1.434
14	rs3813536	81744381	A	0.01546	0.01083	G	0.4309	1.434
14	rs200884137	103576369	A	0.01546	0.01083	G	0.4309	1.434
15	rs114777682	86087092	A	0.01546	0.01083	G	0.4309	1.434
15	rs139042772	89390520	C	0.01546	0.01083	G	0.4309	1.434
17	rs9903170	53785504	A	0.3479	0.3708	G	0.4309	0.9053
19	rs200531149	10416617	A	0.01546	0.01083	C	0.4309	1.434
20	rs1291212	62340115	G	0.01546	0.01083	C	0.4309	1.434
20	rs1151625	62369997	A	0.01546	0.01083	G	0.4309	1.434
1	rs76343542	46976131	A	0.002577	0.0008389	G	0.431	3.078
6	rs9349379	12903957	A	0.2552	0.2767	G	0.431	0.8956
6	rs577493	165933673	A	0.2552	0.2775	G	0.431	0.8919
11	rs2853083	56756664	G	0.2552	0.2775	A	0.431	0.8919
11	rs10769699	6588228	A	0.2861	0.2658	G	0.4312	1.107
17	rs2072153	47390014	G	0.3505	0.3742	C	0.4314	0.9027
3	rs9843405	86637887	C	0.2887	0.2675	A	0.4315	1.111
10	rs1509963	64570411	A	0.384	0.3617	G	0.4315	1.1
11	rs4244812	65480768	A	0.384	0.3617	G	0.4315	1.1

12	rs2268116	13870080	G	0.384	0.3617	A	0.4315	1.1
17	rs7209129	20919504	A	0.2887	0.2675	G	0.4315	1.111
4	rs35609075	34924357	G	0.3505	0.3733	A	0.4316	0.9059
11	rs4939490	60793651	G	0.3505	0.3733	C	0.4316	0.9059
16	rs3743749	77323235	G	0.2242	0.2058	C	0.4317	1.115
10	rs12768894	14974905	G	0.1082	0.09432	A	0.4318	1.166
1	rs200373846	219347282	A	0.02835	0.02083	G	0.4319	1.371
2	rs139125264	37471114	A	0.02835	0.02083	C	0.4319	1.371
4	rs17618262	39449062	G	0.02835	0.02083	A	0.4319	1.371
6	rs202045843	84896316	A	0.02835	0.02083	G	0.4319	1.371
8	rs2446927	101075806	A	0.02835	0.02083	C	0.4319	1.371
9	rs116537880	33463255	A	0.02835	0.02083	G	0.4319	1.371
11	rs11229277	57970980	G	0.02835	0.02083	A	0.4319	1.371
12	rs79164944	100048951	G	0.02835	0.02083	A	0.4319	1.371
15	rs184218886	72283488	A	0.02835	0.02083	G	0.4319	1.371
16	rs138459146	50326666	A	0.02835	0.02083	G	0.4319	1.371
17	rs138284687	67187340	G	0.02835	0.02083	A	0.4319	1.371
22	rs145952310	24087185	G	0.02835	0.02083	A	0.4319	1.371
22	rs75073100	24451504	A	0.02835	0.02083	C	0.4319	1.371
1	rs7524527	225495697	G	0.3531	0.3767	A	0.432	0.9033
5	rs4479825	73527328	A	0.3531	0.3767	C	0.432	0.9033
5	rs930072	36666071	A	0.3866	0.3642	G	0.4321	1.1
10	rs706779	6098824	G	0.3866	0.3642	A	0.4321	1.1
1	rs1043424	20977000	C	0.3892	0.3658	A	0.4322	1.104
1	rs17128866	86591141	G	0.1778	0.1608	A	0.4322	1.129
18	rs17187262	21402298	A	0.08247	0.07	C	0.4322	1.194
16	rs723875	26886022	G	0.3531	0.3758	A	0.4323	0.9065
19	rs2240744	5455735	A	0.3531	0.3758	G	0.4323	0.9065
12	rs4760270	59708827	A	0.2577	0.28	G	0.4324	0.8929
12	rs7963521	73828956	G	0.2577	0.2792	A	0.4324	0.8966
3	rs11720452	21248642	A	0.1392	0.1242	G	0.4325	1.14
4	rs4401444	82620131	A	0.2887	0.2683	C	0.4325	1.106
8	rs11993776	106813518	C	0.1392	0.1242	G	0.4325	1.14
10	rs2275060	72289778	A	0.2887	0.2683	G	0.4325	1.106
19	rs1064395	19361735	A	0.1392	0.1242	G	0.4325	1.14
20	rs6062343	62695931	G	0.2887	0.2683	A	0.4325	1.106
12	rs10848683	2791130	G	0.3892	0.3667	A	0.4327	1.1
14	rs1045922	21250124	A	0.3892	0.3667	G	0.4327	1.1
2	rs72624659	87405001	A	0.3918	0.3687	G	0.4328	1.103
3	rs9311120	35926289	A	0.116	0.1325	C	0.4328	0.859
7	rs146786889	76144473	A	0.002577	0.0008446	G	0.4328	3.057
9	rs277588	34820094	A	0.3918	0.3683	G	0.4328	1.105
9	rs1359590	37729786	A	0.116	0.1325	G	0.4328	0.859

6	rs2074483	30076755	A	0.1521	0.17	G	0.4329	0.8756
1	rs694214	18149510	A	0.3557	0.3783	C	0.433	0.907
3	rs4607103	64711904	A	0.3557	0.3783	G	0.433	0.907
15	rs12909704	77450964	A	0.116	0.1333	G	0.4331	0.8528
1	rs2236387	36557619	A	0.3918	0.3692	G	0.4333	1.101
1	rs148135241	216373416	C	0.04381	0.055	A	0.4333	0.7873
3	rs2054989	56530709	A	0.3582	0.3817	G	0.4333	0.9044
6	rs140770834	32032628	G	0.04381	0.055	C	0.4333	0.7873
8	rs3808553	104337367	A	0.3918	0.3692	C	0.4333	1.101
18	rs8099549	19826742	A	0.3918	0.3692	G	0.4333	1.101
9	rs2274158	117166206	A	0.3943	0.3708	C	0.4334	1.105
18	rs2305027	334742	A	0.3943	0.3708	G	0.4334	1.105
1	rs78000272	85666118	A	0.02835	0.03833	G	0.4335	0.732
2	rs3815849	218713469	A	0.02835	0.03833	G	0.4335	0.732
4	rs1804080	89625427	G	0.1985	0.2183	C	0.4335	0.8864
4	rs34585936	96091414	A	0.02835	0.03833	G	0.4335	0.732
7	rs10259584	100675512	A	0.02835	0.03833	G	0.4335	0.732
8	rs2705293	138918945	G	0.02835	0.03833	A	0.4335	0.732
11	rs77833427	64678098	A	0.04124	0.03333	G	0.4335	1.247
13	rs61729904	52365354	A	0.02835	0.03833	C	0.4335	0.732
16	rs4787483	29885447	G	0.04124	0.03333	A	0.4335	1.247
18	rs1787771	55768995	G	0.02835	0.03833	A	0.4335	0.732
19	rs3810449	36530913	A	0.1985	0.2183	G	0.4335	0.8864
19	rs3218773	50902164	A	0.04124	0.03333	G	0.4335	1.247
4	rs11940551	27162478	C	0.2268	0.2083	A	0.4336	1.115
11	rs712022	22843155	A	0.1985	0.2192	T	0.4336	0.8821
16	rs61747555	71885423	G	0.2268	0.2083	A	0.4336	1.115
19	rs506463	37357289	A	0.2268	0.2083	G	0.4336	1.115
2	rs1052161	73828538	G	0.2912	0.2708	A	0.4337	1.106
3	rs6788448	193209178	G	0.3582	0.3808	A	0.4337	0.9076
5	rs1530496	13931340	G	0.2912	0.2708	A	0.4337	1.106
12	rs6486781	122318985	C	0.2603	0.2825	A	0.4337	0.8938
14	rs2236225	64908845	A	0.2912	0.2708	G	0.4337	1.106
6	rs2235233	27279852	G	0.3943	0.3717	A	0.4338	1.101
6	rs7759666	30569489	A	0.04381	0.05583	G	0.4338	0.7749
7	rs17145468	73814702	A	0.04381	0.05583	C	0.4338	0.7749
2	rs16867321	181362379	A	0.3969	0.3733	G	0.434	1.105
7	rs731257	12669251	A	0.3608	0.3842	G	0.434	0.9049
7	rs17168761	15503133	G	0.3969	0.3733	A	0.434	1.105
7	rs1012036	52472450	A	0.3608	0.3842	G	0.434	0.9049
8	rs1451139	58471927	A	0.3608	0.3842	G	0.434	0.9049
16	rs12921392	77320605	G	0.3969	0.3733	A	0.434	1.105
5	rs152528	142017860	A	0.2294	0.21	G	0.4342	1.12

14	rs12879377	69795199	A	0.1418	0.1258	G	0.4342	1.147
16	rs4889294	81303759	G	0.1418	0.1258	A	0.4342	1.147
19	rs2304220	39971432	C	0.1082	0.095	G	0.4342	1.156
20	rs6033098	11289485	G	0.1082	0.095	A	0.4342	1.156
1	rs3828102	19632332	A	0.3969	0.3742	G	0.4344	1.101
6	rs9393941	29045632	G	0.3608	0.3833	A	0.4344	0.9081
6	rs3129109	29084232	G	0.3608	0.3833	A	0.4344	0.9081
6	rs209473	32922908	C	0.3608	0.3833	A	0.4344	0.9081
11	rs308757	88158865	G	0.3969	0.3742	A	0.4344	1.101
1	rs17124318	63480730	G	0.08763	0.1025	C	0.4345	0.841
6	rs9277965	33256397	A	0.08763	0.1025	G	0.4345	0.841
10	rs10510108	124096160	G	0.08763	0.1025	A	0.4345	0.841
15	rs3817428	89415247	C	0.08763	0.1025	G	0.4345	0.841
1	rs652785	57340727	A	0.3995	0.3758	C	0.4346	1.105
10	rs2234970	102116311	C	0.3995	0.3758	A	0.4346	1.105
11	rs643788	118967758	A	0.3634	0.3867	G	0.4346	0.9055
12	rs10846448	17028884	A	0.3634	0.3867	C	0.4346	0.9055
14	rs2526913	73120986	A	0.3634	0.3867	G	0.4346	0.9055
19	rs2304256	10475652	A	0.3995	0.3758	C	0.4346	1.105
2	rs76801084	80101372	A	0.1804	0.1633	G	0.4349	1.128
16	rs12448227	68068627	G	0.1804	0.1633	A	0.4349	1.128
16	rs2073852	72992667	G	0.1804	0.1633	C	0.4349	1.128
20	rs2990505	14066276	A	0.1804	0.1633	G	0.4349	1.128
2	rs4670696	37599963	C	0.3634	0.3858	A	0.435	0.9087
11	rs10501293	43086094	C	0.08763	0.1033	A	0.435	0.8334
13	rs9564167	64969902	G	0.3634	0.3858	A	0.435	0.9087
14	rs1885315	85915440	A	0.3634	0.3858	C	0.435	0.9087
19	rs3745345	17113147	G	0.08763	0.1033	A	0.435	0.8334
4	rs6827803	175060620	G	0.366	0.39	A	0.4351	0.9029
10	rs11199065	121437329	G	0.1108	0.09599	A	0.4351	1.174
16	rs2925979	81534790	A	0.366	0.39	G	0.4351	0.9029
17	rs532648	26864302	C	0.2629	0.2842	G	0.4351	0.8984
17	rs2074404	44865439	A	0.366	0.39	C	0.4351	0.9029
4	rs2282679	72608383	C	0.2964	0.275	A	0.4353	1.111
16	rs4889240	81156522	G	0.2964	0.275	A	0.4353	1.111
3	rs2293232	195497143	A	0.201	0.2217	G	0.4355	0.8835
7	rs17712293	48311563	C	0.201	0.2217	A	0.4355	0.8835
9	rs1330199	27760946	C	0.08247	0.07083	A	0.4356	1.179
11	rs17324812	4791111	G	0.08247	0.07083	A	0.4356	1.179
14	rs17102999	75513534	A	0.08247	0.07083	G	0.4356	1.179
20	rs35666277	25456698	A	0.08247	0.07083	G	0.4356	1.179
5	rs257747	15817966	A	0.3686	0.3925	C	0.4357	0.9034
9	rs7858199	132436381	A	0.366	0.3883	G	0.4357	0.9092

2	rs17224367	47656972	A	0.04381	0.03417	G	0.4358	1.295
2	rs2276635	220087441	G	0.04381	0.03417	A	0.4358	1.295
3	rs2271502	13384748	G	0.04381	0.03417	A	0.4358	1.295
5	rs1469832	24322935	G	0.3686	0.3917	A	0.4358	0.9066
6	rs116476753	72889549	A	0.04381	0.03417	G	0.4358	1.295
10	rs76591097	105215147	C	0.04381	0.03417	A	0.4358	1.295
19	rs30461	39789115	G	0.04381	0.03417	A	0.4358	1.295
1	rs11203367	17657616	A	0.3776	0.4002	G	0.436	0.9094
2	rs11896521	238283472	C	0.06186	0.05083	A	0.436	1.231
2	rs113155945	238287357	A	0.06186	0.05083	G	0.436	1.231
6	rs6457144	30063368	A	0.4046	0.3817	T	0.436	1.101
8	rs36027551	105459614	A	0.06186	0.05083	G	0.436	1.231
9	rs3429	27062721	A	0.06186	0.05083	G	0.436	1.231
18	rs8091003	28919779	C	0.06186	0.05083	A	0.436	1.231
18	rs8091117	28919794	A	0.06186	0.05083	C	0.436	1.231
18	rs16961689	28919884	C	0.06186	0.05083	A	0.436	1.231
18	rs61730306	28919911	G	0.06186	0.05083	A	0.436	1.231
19	rs67102109	12154799	C	0.06186	0.05083	G	0.436	1.231
19	rs17719289	35832823	G	0.06186	0.05083	A	0.436	1.231
21	rs7275734	42293410	A	0.2964	0.2758	C	0.4361	1.106
17	rs4796693	39884583	G	0.1418	0.1267	A	0.4362	1.139
8	rs4587320	52757270	A	0.3711	0.395	G	0.4363	0.9039
16	rs8049284	80718879	G	0.2655	0.2875	A	0.4363	0.8957
19	rs890935	41089390	C	0.007732	0.01433	G	0.4363	0.5358
22	rs28502153	17469049	A	0.3711	0.395	C	0.4363	0.9039
2	rs6722390	70176616	G	0.2655	0.2867	A	0.4364	0.8993
2	rs766674	137312731	A	0.3711	0.3942	C	0.4364	0.9071
3	rs13076266	87431686	A	0.2655	0.2867	G	0.4364	0.8993
1	rs2840532	2327815	A	0.4072	0.3842	G	0.4365	1.101
1	rs291096	207110936	G	0.4072	0.3842	A	0.4365	1.101
2	rs12618769	99239931	A	0.1108	0.09667	G	0.4365	1.165
2	rs2303832	179472292	A	0.1108	0.09667	T	0.4365	1.165
19	rs35582075	52620269	A	0.1108	0.09667	G	0.4365	1.165
1	rs77696779	39951243	A	0.007732	0.01417	G	0.4366	0.5422
1	rs41296184	113657233	C	0.007732	0.01417	A	0.4366	0.5422
1	rs141831783	207642151	A	0.007732	0.01417	G	0.4366	0.5422
1	rs35073925	226027630	G	0.007732	0.01417	A	0.4366	0.5422
3	rs144249115	14799143	G	0.007732	0.01417	A	0.4366	0.5422
3	rs202061995	42727387	C	0.007732	0.01417	G	0.4366	0.5422
4	rs201412127	337598	G	0.007732	0.01417	A	0.4366	0.5422
4	rs2577055	95444255	G	0.1186	0.135	A	0.4366	0.8618
4	rs1800884	187455426	A	0.007732	0.01417	G	0.4366	0.5422
5	rs17286891	140552774	A	0.007732	0.01417	G	0.4366	0.5422

5	rs41298406	160757907	A	0.007732	0.01417	G	0.4366	0.5422
6	rs6457590	32421798	A	0.1186	0.135	C	0.4366	0.8618
6	rs146866401	100838544	A	0.007732	0.01417	G	0.4366	0.5422
7	rs4728142	128573967	A	0.1186	0.135	G	0.4366	0.8618
8	rs199804539	1808221	A	0.007732	0.01417	C	0.4366	0.5422
9	rs144651672	131767727	G	0.007732	0.01417	A	0.4366	0.5422
10	rs145865648	75673417	A	0.007732	0.01417	G	0.4366	0.5422
10	rs141776380	129913632	A	0.007732	0.01417	C	0.4366	0.5422
11	rs2276263	111680441	A	0.007732	0.01417	G	0.4366	0.5422
11	rs138863361	118451973	A	0.007732	0.01417	C	0.4366	0.5422
15	rs76663839	65209705	A	0.007732	0.01417	G	0.4366	0.5422
15	rs117772298	89388867	A	0.007732	0.01417	G	0.4366	0.5422
16	rs35599524	4790446	A	0.007732	0.01417	G	0.4366	0.5422
16	rs34389205	74709610	A	0.007732	0.01417	G	0.4366	0.5422
17	rs189267287	8701497	C	0.007732	0.01417	G	0.4366	0.5422
17	rs142794787	27425172	A	0.007732	0.01417	G	0.4366	0.5422
20	rs141795719	34117097	A	0.007732	0.01417	G	0.4366	0.5422
4	rs2389890	120575619	A	0.007772	0.01417	C	0.4367	0.5451
12	rs7136716	7691134	G	0.4046	0.3825	A	0.4367	1.097
6	rs2844700	30912900	A	0.06443	0.0775	G	0.4368	0.8198
6	rs2429557	30928317	A	0.06443	0.0775	T	0.4368	0.8198
11	rs499037	59480952	A	0.06443	0.0775	G	0.4368	0.8198
15	rs8023524	43020983	A	0.06443	0.0775	G	0.4368	0.8198
2	rs13420028	133188106	C	0.1186	0.1358	A	0.4369	0.8557
6	rs2239802	32411846	C	0.1186	0.1358	G	0.4369	0.8557
21	rs2014300	36357861	A	0.1186	0.1358	G	0.4369	0.8557
20	rs3746804	744415	A	0.4098	0.3867	G	0.437	1.101
22	rs470072	32263131	G	0.3737	0.3967	A	0.437	0.9076
14	rs2273800	100847707	G	0.4072	0.385	A	0.4372	1.097
1	rs58037016	159283494	G	0.3763	0.4	A	0.4373	0.905
5	rs13170390	35709184	C	0.232	0.2133	G	0.4373	1.114
6	rs9275495	32673574	T	0.1108	0.09699	A	0.4373	1.16
11	rs12365651	70523993	G	0.299	0.2783	A	0.4373	1.106
22	rs4507196	40849704	A	0.3763	0.4	C	0.4373	0.905
10	rs7920896	123846490	A	0.06443	0.07833	G	0.4374	0.8103
17	rs2016126	72784932	A	0.4171	0.3933	G	0.4374	1.104
6	rs853684	28294550	A	0.3763	0.3992	G	0.4375	0.9081
10	rs16920386	66551141	C	0.2036	0.2242	A	0.4375	0.8848
11	rs6591595	60291413	G	0.268	0.29	A	0.4375	0.8966
11	rs11237145	76954812	G	0.2036	0.2233	A	0.4375	0.8891
11	rs11237146	76954833	A	0.2036	0.2233	G	0.4375	0.8891
20	rs6035851	2186970	G	0.268	0.29	A	0.4375	0.8966
1	rs7532317	104736156	A	0.4098	0.3875	C	0.4376	1.097

9	rs1831370	125315557	A	0.4098	0.3875	G	0.4376	1.097
8	rs10866911	144654249	A	0.268	0.2892	G	0.4377	0.9002
9	rs2073478	38396065	A	0.3015	0.28	C	0.4378	1.11
10	rs10828833	25888180	A	0.3015	0.28	G	0.4378	1.11
19	rs10406453	54974318	G	0.4124	0.3898	A	0.4378	1.098
17	rs12604020	18923180	A	0.1443	0.1283	G	0.4379	1.146
2	rs11891861	49156580	A	0.4149	0.3917	C	0.438	1.102
2	rs2279105	96993757	A	0.2345	0.215	G	0.438	1.119
2	rs11695180	115848781	G	0.4149	0.3917	A	0.438	1.102
3	rs1700936	24327558	A	0.2345	0.215	G	0.438	1.119
6	rs1810472	33083121	G	0.4149	0.3917	A	0.438	1.102
2	rs12475304	56760994	A	0.3953	0.4183	G	0.4384	0.9089
3	rs3806633	54959123	A	0.007979	0.01417	G	0.4384	0.5597
18	rs628419	12013384	G	0.1572	0.1758	A	0.4384	0.8744
1	rs242056	4772717	A	0.3015	0.2808	G	0.4385	1.106
1	rs4907103	85316840	C	0.4175	0.3942	A	0.4385	1.102
2	rs289904	152085643	G	0.4149	0.3925	A	0.4385	1.098
4	rs2289043	96106322	A	0.4175	0.3942	G	0.4385	1.102
7	rs1635852	28189411	G	0.3015	0.2808	A	0.4385	1.106
7	rs260368	46463342	C	0.4149	0.3925	A	0.4385	1.098
12	rs10444502	118353871	A	0.3015	0.2808	C	0.4385	1.106
16	rs4270172	55157679	A	0.3015	0.2808	G	0.4385	1.106
17	rs56058441	79668135	A	0.3015	0.2808	C	0.4385	1.106
19	rs1130426	51850290	A	0.3015	0.2808	G	0.4385	1.106
14	rs2284735	81553786	G	0.3814	0.4042	A	0.4386	0.9091
1	rs58275168	234097138	A	0.2706	0.2925	G	0.4388	0.8974
8	rs6999234	124440174	A	0.384	0.4075	G	0.4389	0.9065
8	rs7014678	124448736	A	0.384	0.4075	G	0.4389	0.9065
8	rs6470147	124448804	A	0.384	0.4075	T	0.4389	0.9065
8	rs3824250	124449466	G	0.384	0.4075	A	0.4389	0.9065
8	rs13265801	124460546	A	0.384	0.4075	G	0.4389	0.9065
11	rs10501429	78279790	A	0.384	0.4075	C	0.4389	0.9065
15	rs1982473	48254702	A	0.1856	0.1675	G	0.4389	1.132
5	rs4705752	112366678	A	0.2706	0.2917	G	0.439	0.9011
16	rs1528601	51098427	G	0.2706	0.2917	C	0.439	0.9011
1	rs9661417	232385051	A	0.2345	0.2158	G	0.4391	1.113
6	rs7454108	32681483	G	0.1108	0.0975	A	0.4391	1.154
16	rs68004739	84020685	A	0.1108	0.0975	C	0.4391	1.154
3	rs358733	155485302	G	0.08505	0.0725	A	0.4393	1.189
4	rs1354680	113918577	A	0.3866	0.41	G	0.4393	0.9069
10	rs35796382	63450379	A	0.08505	0.0725	G	0.4393	1.189
9	rs7030820	107267304	A	0.4201	0.3975	G	0.4394	1.098
15	rs4924595	42287606	G	0.2062	0.2258	A	0.4394	0.8904

19	rs77971861	901507	G	0.2062	0.2267	A	0.4394	0.8862
4	rs7669418	154513627	G	0.3041	0.2833	A	0.4396	1.105
4	rs17370297	154514965	G	0.3041	0.2833	A	0.4396	1.105
9	rs10759216	109950798	G	0.4227	0.3998	A	0.4396	1.099
16	rs11558533	67865937	G	0.09021	0.105	A	0.4397	0.8451
8	rs2977852	41488652	C	0.3892	0.4125	A	0.4398	0.9074
15	rs55637254	42171655	A	0.008287	0.0151	C	0.4399	0.545
2	rs12472911	142228509	G	0.1856	0.1683	A	0.4401	1.126
6	rs1042391	16290761	A	0.09021	0.1058	T	0.4401	0.8377
6	rs2274136	17633061	G	0.09021	0.1058	A	0.4401	0.8377
7	rs78680793	2395043	G	0.06186	0.05167	C	0.4401	1.21
8	rs2270641	20038466	C	0.3067	0.285	A	0.4401	1.11
11	rs6592622	75756145	G	0.3067	0.285	A	0.4401	1.11
12	rs479499	10137557	A	0.09021	0.1058	C	0.4401	0.8377
16	rs2292027	58201628	G	0.06186	0.05167	A	0.4401	1.21
18	rs34302455	28919913	A	0.06186	0.05167	G	0.4401	1.21
22	rs5746697	19230365	G	0.06186	0.05167	A	0.4401	1.21
22	rs3747059	19263214	A	0.06186	0.05167	G	0.4401	1.21
5	rs4704434	76890568	A	0.4253	0.4025	G	0.4402	1.098
6	rs7747960	152791474	A	0.3892	0.4117	C	0.4402	0.9106
9	rs3739740	107533159	G	0.2371	0.2179	A	0.4402	1.116
21	rs915876	45376207	G	0.3892	0.4117	A	0.4402	0.9106
7	rs6971091	128363287	A	0.1211	0.1375	G	0.4404	0.8646
14	rs74887188	88431969	G	0.1211	0.1375	A	0.4404	0.8646
1	rs146109722	205549933	A	0.04381	0.035	G	0.4406	1.263
2	rs3738918	187511466	G	0.04381	0.035	A	0.4406	1.263
18	rs2852003	28612229	A	0.04381	0.035	C	0.4406	1.263
19	rs778805	5832209	G	0.4278	0.405	A	0.4406	1.099
5	rs637450	72419456	C	0.3918	0.4142	G	0.4407	0.911
6	rs2621416	32741868	G	0.3943	0.4172	A	0.4407	0.9094
6	rs2621366	32768980	A	0.3918	0.4142	C	0.4407	0.911
6	rs2145544	66853636	A	0.4304	0.4067	G	0.4407	1.103
7	rs1456893	50269672	A	0.3943	0.4175	G	0.4407	0.9084
7	rs1456896	50304461	A	0.3943	0.4175	G	0.4407	0.9084
9	rs12684476	137777132	A	0.04381	0.03506	G	0.441	1.261
12	rs12821922	1778292	G	0.4304	0.4075	A	0.441	1.099
7	rs6978637	80385987	A	0.3969	0.4208	G	0.4411	0.9057
7	rs2075078	150389856	G	0.1598	0.1783	A	0.4411	0.8763
1	rs3736963	208207839	A	0.2758	0.2975	G	0.4412	0.8992
2	rs1432523	183023484	A	0.3943	0.4167	G	0.4412	0.9115
6	rs2853961	31231989	A	0.2758	0.2975	G	0.4412	0.8992
15	rs7182648	57731573	A	0.2088	0.2292	G	0.4412	0.8875
18	rs9952705	31763797	G	0.3943	0.4167	A	0.4412	0.9115

11	rs77206991	123894317	A	0.1598	0.1775	C	0.4413	0.8813
11	rs75994039	123900694	G	0.1598	0.1775	A	0.4413	0.8813
11	rs75240316	123900916	A	0.1598	0.1775	G	0.4413	0.8813
17	rs76299620	45447802	A	0.1598	0.1775	G	0.4413	0.8813
18	rs2729409	28141688	A	0.2088	0.2283	G	0.4413	0.8917
11	rs11212364	97574984	G	0.1134	0.09917	A	0.4415	1.162
4	rs73205493	2460571	G	0.4304	0.4083	A	0.4416	1.095
14	rs7151042	87924278	G	0.1881	0.17	A	0.4416	1.131
1	rs200330080	16529016	A	0.007732	0.015	G	0.4417	0.5117
1	rs3218625	186643541	A	0.02835	0.02167	G	0.4417	1.317
1	rs139441918	203741217	G	0.02835	0.02167	A	0.4417	1.317
1	rs6604611	216655251	A	0.2397	0.22	C	0.4417	1.118
2	rs117616494	97009908	G	0.02835	0.02167	A	0.4417	1.317
2	rs141973562	120387515	A	0.02835	0.02167	C	0.4417	1.317
2	rs17544587	170502401	A	0.007732	0.015	G	0.4417	0.5117
2	rs36051007	179545859	A	0.007732	0.015	G	0.4417	0.5117
2	rs11684227	201540533	G	0.4356	0.4125	A	0.4417	1.099
2	rs35350960	234669619	A	0.007732	0.015	C	0.4417	0.5117
4	rs111678678	106158215	A	0.007732	0.015	G	0.4417	0.5117
4	rs138508820	189061099	A	0.007732	0.015	C	0.4417	0.5117
5	rs72707016	665148	G	0.02835	0.02167	A	0.4417	1.317
5	rs1799904	95764963	A	0.007732	0.015	G	0.4417	0.5117
5	rs73288874	134537897	A	0.007732	0.015	G	0.4417	0.5117
5	rs117222015	178634641	A	0.02835	0.02167	G	0.4417	1.317
6	rs61748600	38729511	G	0.007732	0.015	A	0.4417	0.5117
6	rs61748601	38746176	A	0.007732	0.015	G	0.4417	0.5117
6	rs139503277	64394450	A	0.007732	0.015	G	0.4417	0.5117
10	rs41298373	7622009	A	0.007732	0.015	G	0.4417	0.5117
10	rs78201384	17153023	A	0.02835	0.02167	G	0.4417	1.317
10	rs118166857	105797517	C	0.007732	0.015	A	0.4417	0.5117
11	rs78455795	68822264	A	0.02835	0.02167	G	0.4417	1.317
11	rs1802608	71850722	A	0.007732	0.015	G	0.4417	0.5117
11	rs117485712	113105857	A	0.02835	0.02167	G	0.4417	1.317
11	rs138333675	113236975	A	0.007732	0.015	G	0.4417	0.5117
12	rs143518266	25314094	G	0.007732	0.015	C	0.4417	0.5117
13	rs77886481	39266397	A	0.02835	0.02167	G	0.4417	1.317
13	rs9577273	114107733	A	0.007732	0.015	G	0.4417	0.5117
14	rs114521420	96922766	G	0.02835	0.02167	A	0.4417	1.317
17	rs56119997	73733520	G	0.02835	0.02167	A	0.4417	1.317
18	rs79592897	5396258	A	0.007732	0.015	G	0.4417	0.5117
19	rs182361580	460883	A	0.007732	0.015	G	0.4417	0.5117
19	rs147310621	2278936	A	0.007732	0.015	G	0.4417	0.5117
19	rs12459916	46318286	A	0.007732	0.015	G	0.4417	0.5117

20	rs74643370	25457559	G	0.007732	0.015	A	0.4417	0.5117
17	rs2074409	35898342	C	0.443	0.4199	A	0.4418	1.099
9	rs3124299	137619195	A	0.4021	0.4258	G	0.4419	0.9066
1	rs926268	171151991	A	0.433	0.4108	C	0.442	1.095
13	rs654804	53401505	G	0.433	0.4108	A	0.442	1.095
21	rs2183573	40574305	A	0.433	0.4108	G	0.442	1.095
3	rs3732913	188181560	G	0.4021	0.425	A	0.4421	0.9097
7	rs757702	44686497	A	0.4021	0.425	G	0.4421	0.9097
14	rs1958980	30312091	A	0.4021	0.425	G	0.4421	0.9097
4	rs10517480	60748829	T	0.4046	0.4283	A	0.4422	0.9071
12	rs408260	92772307	A	0.4356	0.4133	G	0.4423	1.095
6	rs2092204	14093556	G	0.3119	0.29	A	0.4424	1.11
6	rs11961569	32864681	C	0.08505	0.07333	A	0.4425	1.175
6	rs10080898	32869882	C	0.08505	0.07333	A	0.4425	1.175
6	rs1585894	32875323	A	0.08505	0.07333	C	0.4425	1.175
6	rs154984	32880419	A	0.08505	0.07333	G	0.4425	1.175
15	rs4932380	91499986	A	0.4046	0.4275	C	0.4425	0.9102
19	rs8102137	30296853	G	0.08505	0.07333	A	0.4425	1.175
20	rs146288604	25457055	A	0.08505	0.07333	G	0.4425	1.175
1	rs1142057	24706292	G	0.4381	0.4158	A	0.4426	1.095
2	rs1059524	132181483	C	0.2397	0.2208	A	0.4426	1.112
2	rs7602307	187838940	A	0.4072	0.4308	G	0.4426	0.9075
10	rs3808907	25242922	G	0.04381	0.03529	A	0.4426	1.252
1	rs61730407	247769752	G	0.4433	0.42	A	0.4427	1.1
5	rs1092913	10467702	G	0.2784	0.2992	A	0.4427	0.9036
5	rs7714670	73072354	G	0.2784	0.2992	A	0.4427	0.9036
5	rs2112347	75015242	A	0.4433	0.42	C	0.4427	1.1
5	rs10277	179264731	A	0.1881	0.1708	G	0.4427	1.125
13	rs2297322	99376181	A	0.4459	0.4227	G	0.4427	1.099
15	rs12443471	89679515	A	0.4433	0.42	G	0.4427	1.1
3	rs7633531	28828525	A	0.4072	0.43	C	0.4429	0.9106
17	rs2551799	9200911	A	0.4072	0.43	G	0.4429	0.9106
6	rs9368151	20115177	C	0.4098	0.4333	A	0.443	0.908
12	rs11835818	122494809	G	0.4098	0.4333	A	0.443	0.908
14	rs28618118	94933473	A	0.4098	0.4333	C	0.443	0.908
1	rs11583447	176521349	C	0.4459	0.4225	A	0.4431	1.1
1	rs10873830	87803200	G	0.2113	0.2308	A	0.4432	0.8929
12	rs10772420	11174276	A	0.2113	0.2308	G	0.4432	0.8929
2	rs10204355	12149887	G	0.4124	0.4358	A	0.4433	0.9084
5	rs6868223	33636594	G	0.4098	0.4325	A	0.4433	0.911
6	rs1632988	29772395	A	0.1469	0.1317	G	0.4433	1.136
11	rs501139	83267970	A	0.4098	0.4325	G	0.4433	0.911
12	rs917855	21630146	A	0.4124	0.4358	G	0.4433	0.9084

16	rs10852325	10880077	A	0.4124	0.4358	G	0.4433	0.9084
5	rs2307111	75003678	A	0.4459	0.4233	G	0.4435	1.096
6	rs6928101	73533851	A	0.4459	0.4233	G	0.4435	1.096
7	rs11763932	47607478	A	0.4459	0.4233	G	0.4435	1.096
17	rs4595846	26051702	C	0.451	0.4273	A	0.4435	1.101
17	rs9893385	66818335	G	0.4459	0.4233	A	0.4435	1.096
17	rs2271535	72350412	A	0.4459	0.4233	G	0.4435	1.096
20	rs2232074	6065729	G	0.2809	0.3025	A	0.4435	0.9008
12	rs13377999	84875392	G	0.4485	0.4257	A	0.4436	1.097
6	rs707928	31742590	G	0.4124	0.435	A	0.4437	0.9115
3	rs75615379	27444657	G	0.04639	0.05833	A	0.4439	0.7853
6	rs720465	31125777	A	0.1134	0.1	C	0.4439	1.151
6	rs3998159	32682019	C	0.1134	0.1	A	0.4439	1.151
9	rs61758536	35810245	A	0.04639	0.05833	G	0.4439	0.7853
10	rs2631681	93032943	A	0.04639	0.05833	G	0.4439	0.7853
17	rs11657054	4463023	G	0.1134	0.1	A	0.4439	1.151
1	rs10926012	240065644	G	0.4149	0.4375	A	0.444	0.9119
2	rs10496150	67729255	A	0.4175	0.4408	C	0.444	0.9092
6	rs2394255	29949821	A	0.4536	0.43	G	0.444	1.1
10	rs79871698	47087609	A	0.04688	0.05789	G	0.444	0.8004
10	rs3123078	51524971	G	0.4175	0.4408	A	0.444	0.9092
10	rs2273697	101563815	A	0.06701	0.08	G	0.444	0.826
11	rs491973	65727301	A	0.4149	0.4375	G	0.444	0.9119
15	rs8038713	34276989	A	0.4149	0.4375	C	0.444	0.9119
18	rs3744825	42532606	A	0.3144	0.2933	G	0.444	1.105
4	rs6817648	29339030	A	0.451	0.4283	G	0.4441	1.097
14	rs7161192	64637147	C	0.451	0.4283	A	0.4441	1.097
2	rs3731958	144899559	C	0.1237	0.1408	A	0.4442	0.8613
3	rs9832396	79354187	A	0.1237	0.1408	G	0.4442	0.8613
7	rs61729612	83037731	G	0.1237	0.1408	C	0.4442	0.8613
6	rs3013169	64842960	A	0.4562	0.4325	C	0.4443	1.101
10	rs2358866	20501874	A	0.4536	0.4308	G	0.4443	1.097
10	rs7071684	48369749	A	0.4562	0.4325	G	0.4443	1.101
12	rs10506525	65783378	A	0.4562	0.4325	G	0.4443	1.101
19	rs10415230	20488845	G	0.4555	0.4791	A	0.4443	0.9095
3	rs1138454	17208267	G	0.06701	0.08083	A	0.4444	0.8167
5	rs13165478	153869040	A	0.3144	0.2938	G	0.4444	1.102
7	rs2390205	19999873	G	0.4175	0.44	A	0.4444	0.9123
8	rs2914862	107180333	G	0.06701	0.08083	A	0.4444	0.8167
10	rs2275799	115409840	A	0.2423	0.2233	G	0.4444	1.112
12	rs2241221	14959391	G	0.06701	0.08083	A	0.4444	0.8167
12	rs7304054	14976063	G	0.06701	0.08083	A	0.4444	0.8167
12	rs7307438	14976178	A	0.06701	0.08083	T	0.4444	0.8167

12	rs1056320	31605044	A	0.2423	0.2233	C	0.4444	1.112
6	rs2064317	35477032	G	0.4588	0.435	A	0.4445	1.101
14	rs1131877	103342049	G	0.4588	0.435	A	0.4445	1.101
4	rs937788	76846755	G	0.4227	0.4458	A	0.4446	0.91
4	rs6845322	157884105	G	0.4562	0.4333	A	0.4446	1.097
15	rs28653195	95851885	C	0.4227	0.4458	A	0.4446	0.91
3	rs4241552	88374655	A	0.4253	0.4492	C	0.4448	0.9074
10	rs12571751	80942631	G	0.4253	0.4492	A	0.4448	0.9074
11	rs3740861	94862577	A	0.09278	0.1075	C	0.4448	0.8491
16	rs337285	1961674	G	0.4253	0.4492	C	0.4448	0.9074
3	rs3732858	43097765	A	0.09326	0.1085	G	0.4449	0.845
11	rs3758911	107197640	G	0.2139	0.2342	A	0.4449	0.89
16	rs8055325	1962132	G	0.4253	0.4482	A	0.4449	0.9111
1	rs11264581	156883493	A	0.4613	0.4383	G	0.445	1.097
3	rs4373074	84688248	A	0.4613	0.4383	G	0.445	1.097
6	rs1042174	33037626	C	0.4613	0.4388	G	0.445	1.096
10	rs7096169	104618695	G	0.4613	0.4383	A	0.445	1.097
19	rs12986413	2170954	A	0.4253	0.4483	T	0.445	0.9104
1	rs10864004	213009469	A	0.4278	0.4517	G	0.4451	0.9078
1	rs2810008	242687390	A	0.09278	0.1083	G	0.4451	0.8418
3	rs6414248	110882326	A	0.317	0.2958	C	0.4451	1.105
3	rs448378	169100899	G	0.09278	0.1083	A	0.4451	0.8418
4	rs1517552	82046984	A	0.4227	0.445	C	0.4451	0.9131
5	rs2230524	66478729	G	0.2139	0.2333	A	0.4451	0.8941
6	rs9502893	1340189	G	0.317	0.2958	A	0.4451	1.105
6	rs3129904	32310396	A	0.09278	0.1083	G	0.4451	0.8418
6	rs2273426	70733547	C	0.317	0.2958	G	0.4451	1.105
7	rs2290228	128388648	A	0.2139	0.2333	G	0.4451	0.8941
8	rs2290472	98943598	A	0.09278	0.1083	G	0.4451	0.8418
11	rs3751095	8435077	A	0.2139	0.2333	G	0.4451	0.8941
11	rs3751096	8435078	C	0.2139	0.2333	A	0.4451	0.8941
12	rs10774021	349298	A	0.2139	0.2333	G	0.4451	0.8941
12	rs2578475	4007324	A	0.317	0.2958	C	0.4451	1.105
12	rs11171466	55846538	G	0.09278	0.1083	C	0.4451	0.8418
5	rs13358260	78845711	G	0.09278	0.1085	A	0.4452	0.8402
9	rs2073924	136037742	G	0.4278	0.4508	A	0.4452	0.9108
1	rs10737680	196679455	C	0.4639	0.4408	A	0.4453	1.098
9	rs7871736	124650611	G	0.4304	0.4542	A	0.4453	0.9082
4	rs4470624	20039702	G	0.4304	0.4533	A	0.4455	0.9112
6	rs12110693	122158270	A	0.4665	0.4433	G	0.4455	1.098
11	rs7927378	114233785	G	0.4665	0.4433	A	0.4455	1.098
19	rs3764609	46823702	G	0.433	0.4567	A	0.4456	0.9086
2	rs1534422	12640741	G	0.2861	0.3083	A	0.4457	0.8989

7	rs2538918	88311428	G	0.2861	0.3083	A	0.4457	0.8989
8	rs7825567	63449366	A	0.4613	0.4392	G	0.4457	1.094
14	rs992745	27810095	G	0.2861	0.3083	A	0.4457	0.8989
3	rs9865242	112642568	G	0.2861	0.3075	C	0.4458	0.9024
3	rs325798	151921374	G	0.433	0.4558	A	0.4458	0.9116
4	rs898500	42639186	G	0.2861	0.3075	A	0.4458	0.9024
1	rs6677604	196686918	A	0.06443	0.05333	G	0.4459	1.222
6	rs902393	168463624	A	0.06443	0.05333	G	0.4459	1.222
7	rs2293507	70228020	A	0.06443	0.05333	C	0.4459	1.222
7	rs1916667	85004375	G	0.4716	0.4483	A	0.4459	1.098
14	rs36014869	75555305	A	0.06443	0.05333	C	0.4459	1.222
6	rs2894239	32215796	A	0.4381	0.4617	C	0.446	0.9093
6	rs3115573	32218843	A	0.4381	0.4617	G	0.446	0.9093
11	rs11227673	66832528	G	0.4665	0.4442	A	0.446	1.094
12	rs10784277	62381413	A	0.4381	0.4617	G	0.446	0.9093
13	rs2476722	32755834	A	0.4381	0.4617	G	0.446	0.9093
2	rs3828323	160808075	A	0.3196	0.2983	G	0.4461	1.105
6	rs9295206	163326692	G	0.4742	0.4508	A	0.4461	1.099
8	rs40457	90823687	G	0.2448	0.2258	A	0.4461	1.111
9	rs7044529	137568051	A	0.08763	0.075	G	0.4461	1.185
12	rs7310011	43822171	A	0.08763	0.075	G	0.4461	1.185
12	rs7302446	43828141	T	0.08763	0.075	A	0.4461	1.185
19	rs2305777	14038791	A	0.08763	0.075	G	0.4461	1.185
19	rs3752365	38230720	G	0.08763	0.075	C	0.4461	1.185
6	rs9406335	169600942	G	0.4407	0.4642	A	0.4462	0.9097
14	rs1051858	58831142	A	0.4691	0.4467	G	0.4462	1.094
22	rs5768001	48198112	G	0.4691	0.4467	A	0.4462	1.094
1	rs2793813	179158614	C	0.4716	0.4492	A	0.4463	1.095
4	rs10938367	44832274	G	0.4716	0.4492	A	0.4463	1.095
4	rs17400095	78205171	G	0.4768	0.4533	A	0.4463	1.099
6	rs9271366	32586854	G	0.116	0.1017	A	0.4464	1.159
9	rs439467	10102903	A	0.4433	0.4667	G	0.4464	0.9101
9	rs6560608	80252414	G	0.4611	0.4841	A	0.4464	0.9121
10	rs2154028	115411614	A	0.116	0.1017	G	0.4464	1.159
11	rs12576239	2502319	A	0.116	0.1017	G	0.4464	1.159
13	rs6492665	94184165	A	0.4433	0.4667	G	0.4464	0.9101
6	rs4712524	20657865	G	0.4742	0.4517	A	0.4465	1.095
6	rs7754840	20661250	C	0.4742	0.4517	G	0.4465	1.095
2	rs4674438	220643119	G	0.4768	0.4542	A	0.4466	1.095
3	rs690037	16395668	A	0.4459	0.4692	G	0.4466	0.9104
4	rs2171755	26503960	A	0.4768	0.4542	G	0.4466	1.095
4	rs10001384	134860496	A	0.4407	0.4633	G	0.4466	0.9127
6	rs730223	100676593	A	0.4768	0.4542	G	0.4466	1.095

11	rs2276348	122955402	A	0.1933	0.175	G	0.4466	1.13
12	rs2608405	32296621	G	0.482	0.4583	A	0.4466	1.1
12	rs7980521	56535251	G	0.4459	0.4692	A	0.4466	0.9104
2	rs2583556	85331686	A	0.2165	0.2367	G	0.4467	0.8912
4	rs10030475	70807771	A	0.2165	0.2367	G	0.4467	0.8912
22	rs2413338	35663523	G	0.4845	0.4608	A	0.4467	1.1
2	rs6716724	11510948	A	0.2887	0.3108	G	0.4468	0.8997
2	rs7583048	153891830	A	0.4485	0.4717	G	0.4468	0.9108
12	rs159685	29727218	A	0.4485	0.4717	C	0.4468	0.9108
13	rs188096	103705044	A	0.2887	0.3108	C	0.4468	0.8997
4	rs12650513	61560936	G	0.482	0.4592	A	0.4469	1.096
9	rs10781471	80515456	C	0.482	0.4592	A	0.4469	1.096
17	rs4071641	76157266	A	0.2887	0.31	G	0.4469	0.9032
1	rs17645	26646730	G	0.451	0.4742	A	0.447	0.9111
4	rs11542133	39000305	G	0.4536	0.4775	A	0.447	0.9084
4	rs227368	103611845	G	0.451	0.4742	A	0.447	0.9111
12	rs2074533	48134695	G	0.4536	0.4775	A	0.447	0.9084
2	rs1821421	133964894	A	0.4459	0.4683	G	0.4471	0.9135
6	rs4895548	139134754	A	0.4871	0.4642	G	0.4471	1.096
16	rs8049607	11691753	G	0.4871	0.4642	A	0.4471	1.096
19	rs966591	7083629	A	0.4871	0.4642	G	0.4471	1.096
14	rs4905326	95931254	G	0.4897	0.4667	A	0.4472	1.097
4	rs6825911	111381638	G	0.4562	0.4792	A	0.4473	0.9118
5	rs10514995	65739439	G	0.4485	0.4708	A	0.4473	0.9138
14	rs6573781	68045935	G	0.4588	0.4825	A	0.4473	0.9091
1	rs2609348	223194033	A	0.4613	0.485	G	0.4474	0.9094
1	rs913426	42806191	A	0.5	0.4767	G	0.4475	1.098
1	rs2485652	44595410	G	0.4639	0.4875	A	0.4475	0.9098
5	rs169082	169074056	G	0.5	0.4767	A	0.4475	1.098
12	rs516016	119901095	G	0.5	0.4767	A	0.4475	1.098
15	rs16939752	45444133	G	0.5	0.4767	A	0.4475	1.098
2	rs4662834	129478842	A	0.4665	0.49	G	0.4476	0.9101
2	rs3771895	152224339	G	0.4613	0.4842	A	0.4476	0.9125
2	rs1569175	201021954	A	0.1933	0.1758	G	0.4476	1.123
2	rs2230115	219509618	C	0.1933	0.1758	A	0.4476	1.123
6	rs3128935	32972404	G	0.1933	0.1758	A	0.4476	1.123
9	rs7022714	110950572	A	0.5052	0.4817	G	0.4476	1.099
11	rs11023787	15952294	A	0.5026	0.4792	G	0.4476	1.098
12	rs7974993	27329738	A	0.4665	0.49	C	0.4476	0.9101
14	rs4906217	103007318	A	0.5026	0.4792	G	0.4476	1.098
17	rs2430514	69676169	A	0.4665	0.49	C	0.4476	0.9101
20	rs4608	35865054	A	0.5026	0.4792	G	0.4476	1.098
1	rs1827293	21795388	G	0.4691	0.4925	A	0.4477	0.9104

1	rs2740168	226020988	A	0.5103	0.4867	G	0.4477	1.099
1	rs6664332	248128929	G	0.5077	0.4842	A	0.4477	1.099
2	rs79399438	179325816	A	0.1263	0.1433	G	0.4477	0.8639
3	rs1476184	2302790	A	0.4691	0.4925	G	0.4477	0.9104
3	rs1494225	22466842	A	0.4716	0.495	G	0.4477	0.9107
3	rs6767666	118840453	G	0.4536	0.4758	A	0.4477	0.9145
6	rs910050	32315654	C	0.4536	0.4758	G	0.4477	0.9145
7	rs7313	29070209	A	0.1263	0.1433	G	0.4477	0.8639
7	rs324981	34818113	A	0.5129	0.4892	T	0.4477	1.1
9	rs7859081	5346352	G	0.5129	0.4892	A	0.4477	1.1
10	rs1891111	124606165	A	0.5077	0.4842	G	0.4477	1.099
11	rs11228759	56508541	A	0.1263	0.1433	G	0.4477	0.8639
11	rs11228763	56511160	G	0.1263	0.1433	A	0.4477	0.8639
15	rs3743248	99670989	A	0.2474	0.2283	G	0.4477	1.111
16	rs4967857	64886822	A	0.5103	0.4867	G	0.4477	1.099
22	rs1962222	32971900	G	0.4536	0.4758	A	0.4477	0.9145
1	rs2275363	21940555	A	0.4742	0.4975	G	0.4478	0.911
2	rs1014286	103149100	A	0.4768	0.5	G	0.4478	0.9113
6	rs6904400	11477493	A	0.4742	0.4975	G	0.4478	0.911
6	rs2517532	31018407	A	0.4768	0.5	G	0.4478	0.9113
14	rs4254249	28457813	G	0.4897	0.4675	A	0.4478	1.093
1	rs4072247	223716508	A	0.04381	0.03583	G	0.4479	1.233
2	rs1445136	18893327	G	0.4665	0.4892	A	0.4479	0.9131
3	rs11712780	51416368	A	0.3222	0.3017	G	0.4479	1.1
3	rs704529	58036651	G	0.4794	0.5025	A	0.4479	0.9116
3	rs6444847	168904516	A	0.4665	0.4892	G	0.4479	0.9131
5	rs16876062	78190471	A	0.04381	0.03583	G	0.4479	1.233
5	rs13182402	125918148	G	0.04381	0.03583	A	0.4479	1.233
6	rs9368758	33138021	G	0.482	0.505	A	0.4479	0.9119
7	rs6962352	75189471	A	0.4923	0.47	G	0.4479	1.093
8	rs4593549	23396732	G	0.4794	0.5025	A	0.4479	0.9116
9	rs1138545	117835899	A	0.04381	0.03583	G	0.4479	1.233
14	rs8017377	24883887	A	0.04381	0.03583	G	0.4479	1.233
14	rs12433177	70462361	G	0.4923	0.47	A	0.4479	1.093
15	rs1528472	55321128	A	0.482	0.505	C	0.4479	0.9119
16	rs11864573	19421858	A	0.4948	0.4725	G	0.4479	1.094
4	rs13122930	142650413	G	0.5026	0.48	A	0.448	1.095
5	rs30827	149293949	A	0.5103	0.4875	G	0.448	1.096
6	rs9473582	49488908	A	0.2912	0.3125	C	0.448	0.904
8	rs7832232	38469303	G	0.5026	0.48	A	0.448	1.095
8	rs28703878	79417222	A	0.5129	0.49	G	0.448	1.096
8	rs10103003	118853829	A	0.5052	0.4825	G	0.448	1.095
10	rs10788350	81765502	G	0.5155	0.4925	A	0.448	1.096

11	rs7941030	122522375	G	0.2912	0.3125	A	0.448	0.904
13	rs160816	93751957	G	0.5129	0.49	A	0.448	1.096
4	rs356219	90637601	A	0.4716	0.4942	G	0.4481	0.9138
1	rs11121804	11766424	A	0.3247	0.3033	G	0.4482	1.105
14	rs1188184	56442412	G	0.4768	0.4992	A	0.4483	0.9144
15	rs16972193	42154738	A	0.482	0.5042	G	0.4483	0.9149
2	rs10169988	106042958	A	0.4794	0.5017	G	0.4484	0.9147
7	rs181113	88075010	G	0.4794	0.5017	A	0.4484	0.9147
10	rs11003863	55568972	A	0.2191	0.2392	C	0.4484	0.8924
1	rs2274253	117603156	A	0.1521	0.1358	C	0.4485	1.141
3	rs6438208	114170272	A	0.482	0.5042	G	0.4485	0.915
7	rs855680	149479335	C	0.1521	0.1358	G	0.4485	1.141
7	rs77510079	149483237	A	0.1521	0.1358	G	0.4485	1.141
12	rs2240311	120533965	A	0.482	0.5042	G	0.4485	0.915
16	rs116954456	89712380	G	0.482	0.5042	A	0.4485	0.915
2	rs4973417	232349636	C	0.25	0.23	A	0.4486	1.116
7	rs4607517	44235668	A	0.25	0.23	G	0.4486	1.116
10	rs1801232	16870912	A	0.116	0.1025	C	0.4486	1.149
11	rs58178367	83642842	A	0.25	0.23	G	0.4486	1.116
12	rs62636520	49690798	A	0.03093	0.04083	G	0.4486	0.7497
16	rs9927389	814058	A	0.03093	0.04083	G	0.4486	0.7497
16	rs276639	13179016	A	0.116	0.1025	G	0.4486	1.149
16	rs34504526	81201625	A	0.03093	0.04083	G	0.4486	0.7497
19	rs61751875	9236969	A	0.03093	0.04083	G	0.4486	0.7497
19	rs118114660	9237457	A	0.03093	0.04083	C	0.4486	0.7497
19	rs115923170	9237622	G	0.03093	0.04083	A	0.4486	0.7497
22	rs112636081	19209603	A	0.03093	0.04083	G	0.4486	0.7497
17	rs9895032	3951946	A	0.2938	0.3155	G	0.4488	0.9026
14	rs6575793	101032217	A	0.2938	0.3158	G	0.4489	0.9013
12	rs7297737	43826204	A	0.08763	0.07583	G	0.449	1.17
4	rs62331900	155312411	A	0.2938	0.315	G	0.4491	0.9048
4	rs2276924	187078785	A	0.1933	0.1767	G	0.4491	1.117
15	rs1058260	101922323	G	0.1933	0.1767	A	0.4491	1.117
19	rs891203	17337882	C	0.1933	0.1767	G	0.4491	1.117
7	rs13233916	138874416	C	0.3273	0.3058	G	0.4492	1.104
2	rs151173463	233321916	G	0.01804	0.02588	A	0.4493	0.6916
2	rs137963675	152319458	A	0.01804	0.02583	G	0.4494	0.6928
3	rs12635698	16408489	G	0.01804	0.02583	A	0.4494	0.6928
5	rs116872912	26902832	A	0.01804	0.02583	G	0.4494	0.6928
5	rs7714708	58295014	G	0.25	0.2308	A	0.4494	1.111
5	rs17844411	140502733	A	0.01804	0.02583	G	0.4494	0.6928
5	rs148422543	156593121	A	0.01804	0.02583	G	0.4494	0.6928
11	rs117979626	18253137	A	0.01804	0.02583	G	0.4494	0.6928

11	rs2236654	68704028	A	0.01804	0.02583	G	0.4494	0.6928
14	rs7144523	52495481	A	0.01804	0.02583	G	0.4494	0.6928
14	rs2273430	52496387	C	0.01804	0.02583	A	0.4494	0.6928
14	rs2273431	52496407	G	0.01804	0.02583	A	0.4494	0.6928
14	rs117942237	56585441	C	0.01804	0.02583	G	0.4494	0.6928
1	rs10494217	119469188	A	0.06443	0.05417	C	0.4496	1.203
6	rs9296059	32893102	C	0.06443	0.05417	A	0.4496	1.203
8	rs9657079	49753613	C	0.06443	0.05417	A	0.4496	1.203
10	rs17173752	127484721	C	0.06443	0.05417	A	0.4496	1.203
14	rs73312836	88459795	A	0.06443	0.05417	G	0.4496	1.203
18	rs1788799	21124945	C	0.06443	0.05417	G	0.4496	1.203
6	rs7453944	142396790	A	0.09536	0.11	C	0.4497	0.8529
3	rs2303909	158366900	A	0.3273	0.3067	G	0.4498	1.1
3	rs8455	158388780	G	0.3273	0.3067	A	0.4498	1.1
5	rs13190616	103425505	A	0.3273	0.3067	G	0.4498	1.1
12	rs7966619	52532208	A	0.09536	0.1108	G	0.4499	0.8457
17	rs11655578	7230845	A	0.09536	0.1108	C	0.4499	0.8457
10	rs3829856	98742750	C	0.2216	0.2417	A	0.4501	0.8936
1	rs74467253	28282309	A	0.01804	0.02667	G	0.4505	0.6706
1	rs60854092	120465007	A	0.03093	0.04167	T	0.4505	0.734
3	rs144270136	38835234	A	0.03093	0.04167	G	0.4505	0.734
3	rs3749191	45134822	A	0.01804	0.02667	G	0.4505	0.6706
3	rs6784615	52506426	G	0.01804	0.02667	A	0.4505	0.6706
4	rs1801612	88967828	G	0.03093	0.04167	A	0.4505	0.734
6	rs11544160	116784797	A	0.01804	0.02667	G	0.4505	0.6706
7	rs34603556	20691047	G	0.01804	0.02667	A	0.4505	0.6706
7	rs79606766	149558167	A	0.01804	0.02667	G	0.4505	0.6706
9	rs117880987	130476383	A	0.03093	0.04167	G	0.4505	0.734
11	rs78859654	124789828	A	0.01804	0.02667	T	0.4505	0.6706
12	rs79065113	43846199	A	0.03093	0.04167	C	0.4505	0.734
13	rs10492585	105386176	G	0.03093	0.04167	A	0.4505	0.734
14	rs116929575	95090119	G	0.01804	0.02667	A	0.4505	0.6706
15	rs3803339	43724532	G	0.03093	0.04167	A	0.4505	0.734
16	rs75025609	58030741	A	0.01804	0.02667	G	0.4505	0.6706
17	rs75616699	1183302	A	0.01804	0.02667	G	0.4505	0.6706
17	rs71366574	3661052	A	0.01804	0.02667	G	0.4505	0.6706
17	rs186277389	21217508	A	0.01804	0.02671	G	0.4505	0.6695
19	rs142910242	12157571	A	0.01804	0.02667	G	0.4505	0.6706
19	rs117329175	33140652	A	0.01804	0.02667	G	0.4505	0.6706
16	rs34474910	1129620	G	0.03093	0.04174	A	0.4506	0.7328
6	rs419957	29505769	G	0.299	0.3208	C	0.4509	0.9028
6	rs414282	29506260	G	0.299	0.3208	A	0.4509	0.9028
10	rs10885117	113022555	G	0.06959	0.0825	A	0.4509	0.8318

10	rs2255141	113933886	A	0.299	0.3208	G	0.4509	0.9028
10	rs2792751	113940329	A	0.299	0.3208	G	0.4509	0.9028
12	rs12820008	103152029	A	0.299	0.3208	C	0.4509	0.9028
19	exm1426406	10692051	A	0.06959	0.0825	G	0.4509	0.8318
19	rs76685516	10692051	A	0.06959	0.0825	G	0.4509	0.8318
9	rs10819291	130186700	A	0.2526	0.2333	G	0.451	1.11
4	rs1062677	72433545	C	0.06959	0.08333	A	0.4511	0.8227
8	rs6984526	61149731	A	0.3325	0.3108	G	0.4511	1.104
8	rs261572	97022165	C	0.1186	0.1042	A	0.4511	1.157
11	rs2846412	108384207	G	0.06959	0.08333	A	0.4511	0.8227
16	rs75279195	815549	G	0.1186	0.1042	A	0.4511	1.157
20	rs6142884	60503350	A	0.1186	0.1042	G	0.4511	1.157
1	rs6670655	164739171	A	0.299	0.32	G	0.4512	0.9062
4	rs6858643	133504375	C	0.1289	0.145	A	0.4513	0.8723
19	rs34187696	56733425	A	0.1701	0.1883	C	0.4514	0.8834
11	rs78813996	9305051	G	0.1959	0.1792	A	0.4515	1.116
15	rs2567426	96332861	A	0.1701	0.1892	G	0.4515	0.8786
7	rs10259085	7268431	G	0.3015	0.3233	A	0.4519	0.9035
7	rs2965404	21749502	A	0.2526	0.2342	G	0.4522	1.105
2	rs13011768	120438523	A	0.1985	0.1808	G	0.4523	1.122
2	rs1983406	120439230	C	0.1985	0.1808	G	0.4523	1.122
9	rs76077278	79930225	A	0.1562	0.14	G	0.4523	1.138
6	rs7775698	135418635	A	0.3351	0.3142	G	0.4526	1.1
6	rs7776054	135418916	G	0.3351	0.3142	A	0.4526	1.1
14	rs116769859	81380682	A	0.09021	0.0775	G	0.4526	1.18
16	rs7199343	73009024	T	0.09021	0.0775	A	0.4526	1.18
1	rs10489579	185143721	G	0.3376	0.3158	A	0.453	1.104
12	rs2657879	56865338	G	0.1186	0.105	A	0.4531	1.146
1	rs12027024	149998494	A	0.3041	0.325	G	0.4532	0.9077
6	rs116955726	142688811	A	0.09563	0.08206	G	0.4532	1.183
2	rs13426642	3729254	A	0.1546	0.1392	G	0.4533	1.132
4	rs959903	25810096	A	0.1546	0.1392	G	0.4533	1.132
17	exm-IND17-3083	33806842	D	0.1546	0.1392	I	0.4533	1.132
1	rs200874938	11721315	A	0.01804	0.0125	G	0.4534	1.451
1	rs191448293	85116142	C	0.01804	0.0125	G	0.4534	1.451
1	rs141841342	170964609	A	0.01804	0.0125	G	0.4534	1.451
1	rs77197094	203708945	A	0.01804	0.0125	G	0.4534	1.451
2	rs13306206	21242731	A	0.01804	0.0125	G	0.4534	1.451
2	rs34727427	177016728	G	0.01804	0.0125	A	0.4534	1.451
2	rs72646861	179449186	A	0.01804	0.0125	G	0.4534	1.451
2	rs77239104	216191586	C	0.01804	0.0125	A	0.4534	1.451
3	rs148995934	188426077	A	0.01804	0.0125	G	0.4534	1.451
4	rs139635179	77677618	G	0.01804	0.0125	A	0.4534	1.451

5	rs869955	635508	A	0.01804	0.0125	G	0.4534	1.451
5	rs147650341	5239862	G	0.01804	0.0125	A	0.4534	1.451
6	rs117191676	27879448	A	0.01804	0.0125	G	0.4534	1.451
6	rs200667899	127836033	C	0.01804	0.0125	G	0.4534	1.451
7	rs148800414	21818641	C	0.01804	0.0125	G	0.4534	1.451
7	rs117705799	36489329	A	0.01804	0.0125	G	0.4534	1.451
7	rs11556986	105177041	T	0.01804	0.0125	A	0.4534	1.451
8	rs34725565	21965372	A	0.01804	0.0125	G	0.4534	1.451
12	rs34410987	40677699	A	0.01804	0.0125	G	0.4534	1.451
15	rs77977805	25969049	G	0.01804	0.0125	A	0.4534	1.451
15	rs61754856	84558929	A	0.01804	0.0125	G	0.4534	1.451
15	rs150261781	86697675	A	0.01804	0.0125	G	0.4534	1.451
16	rs145434552	15155630	A	0.01804	0.0125	G	0.4534	1.451
17	rs191314989	37101367	T	0.01804	0.0125	A	0.4534	1.451
17	rs35896669	56272476	A	0.01804	0.0125	C	0.4534	1.451
19	rs143377606	10114362	A	0.01804	0.0125	G	0.4534	1.451
19	rs149297688	11505146	A	0.01804	0.0125	G	0.4534	1.451
22	rs12484731	23627238	A	0.01804	0.0125	C	0.4534	1.451
4	rs67383011	184612553	G	0.04897	0.06	C	0.4535	0.8067
9	rs7865808	2913620	A	0.04897	0.06	G	0.4535	0.8067
11	rs12364102	56949691	A	0.04897	0.06083	G	0.4535	0.7949
18	rs11665084	22056766	A	0.04897	0.06	G	0.4535	0.8067
18	rs11662595	22056970	G	0.04897	0.06	A	0.4535	0.8067
19	rs75323205	15198720	A	0.09211	0.0793	T	0.4535	1.178
20	rs117101542	6022813	A	0.04897	0.06	G	0.4535	0.8067
20	rs1160312	22050503	A	0.3376	0.3167	G	0.4535	1.1
3	rs2869782	117084425	A	0.04897	0.06104	G	0.4536	0.7921
4	rs6845865	148974602	G	0.1985	0.1817	A	0.4537	1.115
1	rs6696981	22702858	A	0.1727	0.1917	C	0.4539	0.8803
3	rs240033	38357961	C	0.3402	0.3183	G	0.4539	1.104
22	rs2071421	51064416	G	0.1727	0.1917	A	0.4539	0.8803
12	rs2272495	105583877	A	0.2577	0.2383	G	0.4541	1.11
6	rs3130649	30803254	T	0.3402	0.3192	A	0.4544	1.1
3	rs345013	145173788	G	0.09794	0.1125	A	0.4545	0.8565
17	rs75117355	37224211	A	0.09794	0.1125	G	0.4545	0.8565
19	rs3786734	49376584	A	0.1314	0.1477	G	0.4545	0.873
4	rs55633891	40356041	A	0.1314	0.1483	G	0.4546	0.8689
16	rs9926580	20410547	A	0.1314	0.1483	G	0.4546	0.8689
21	rs2824700	19562494	A	0.1314	0.1483	G	0.4546	0.8689
2	rs77940364	242674803	A	0.1314	0.1475	G	0.4547	0.8747
3	rs3732619	153202348	A	0.201	0.1833	G	0.4547	1.121
5	rs3813474	33576602	G	0.04639	0.0375	A	0.4547	1.249
6	rs34902660	25851102	A	0.04639	0.0375	C	0.4547	1.249

8	rs140161612	19362969	A	0.04639	0.0375	G	0.4547	1.249
11	rs2282532	66100043	A	0.04639	0.0375	G	0.4547	1.249
12	rs17853860	14976021	G	0.04639	0.0375	A	0.4547	1.249
11	rs556056	56508525	A	0.3093	0.3317	G	0.4548	0.9023
11	rs514076	64990041	C	0.3093	0.3308	G	0.4548	0.9057
5	rs3811987	74324548	A	0.2294	0.2492	G	0.4551	0.897
7	rs17712299	48313881	G	0.2294	0.2492	A	0.4551	0.897
19	rs2108622	15990431	A	0.3093	0.33	G	0.4552	0.9091
19	rs7248508	17316782	A	0.2577	0.2392	G	0.4552	1.105
3	rs117164680	52526372	A	0.03093	0.02333	C	0.4553	1.336
4	rs145979886	13601977	A	0.03093	0.02333	C	0.4553	1.336
6	exm-IND6-25205	25097412	I	0.03093	0.02333	D	0.4553	1.336
6	rs2233976	31079994	A	0.06701	0.05583	G	0.4553	1.215
6	rs12526094	167738668	A	0.03093	0.02333	G	0.4553	1.336
7	rs72657339	21737718	G	0.03093	0.02333	A	0.4553	1.336
7	rs2293177	140244560	A	0.09021	0.07833	G	0.4553	1.167
8	rs34086286	27516969	A	0.03093	0.02333	G	0.4553	1.336
10	rs77930344	5498752	A	0.09021	0.07833	G	0.4553	1.167
13	rs139794584	42772730	A	0.03093	0.02333	G	0.4553	1.336
15	rs12708402	42171462	A	0.06701	0.05583	G	0.4553	1.215
17	rs148602477	7948638	A	0.03093	0.02333	G	0.4553	1.336
19	rs75152309	41382476	T	0.03093	0.02333	A	0.4553	1.336
20	rs34099160	18295730	A	0.1211	0.1067	G	0.4556	1.154
20	rs2228273	18296076	A	0.1211	0.1067	G	0.4556	1.154
12	rs7965624	95301653	G	0.3119	0.3333	A	0.4558	0.9064
13	rs9805818	110130628	A	0.3119	0.3333	G	0.4558	0.9064
18	rs1811358	50768168	G	0.3119	0.3333	A	0.4558	0.9064
2	rs16866378	179393111	G	0.201	0.1842	A	0.456	1.115
5	rs6555699	165995262	A	0.201	0.1842	C	0.456	1.115
6	rs2269709	30540890	G	0.3454	0.3242	A	0.4561	1.1
20	rs6046396	19852503	G	0.3428	0.3225	A	0.4561	1.096
7	rs1285407	9266388	G	0.1753	0.1942	A	0.4563	0.8819
19	rs2609881	44831989	C	0.1572	0.1417	A	0.4565	1.13
2	rs6433573	177076041	A	0.3144	0.3367	G	0.4566	0.9037
14	rs2295320	24682767	A	0.3144	0.3367	G	0.4566	0.9037
2	rs10439493	68261015	C	0.232	0.2525	A	0.4567	0.8941
2	rs3771371	71627539	A	0.3144	0.3358	G	0.4567	0.9071
2	rs6714975	71633389	A	0.3144	0.3358	G	0.4567	0.9071
2	rs1804020	71654175	G	0.3144	0.3358	A	0.4567	0.9071
4	rs897945	83838262	A	0.232	0.2517	C	0.4567	0.898
6	rs4711185	29356046	A	0.232	0.2517	G	0.4567	0.898
6	rs1064627	30698541	G	0.232	0.2525	A	0.4567	0.8941
6	rs10947789	39174922	G	0.232	0.2517	A	0.4567	0.898

7	rs12536873	149517978	A	0.2603	0.2417	C	0.4567	1.104
9	rs10816196	9829254	C	0.2603	0.2417	A	0.4567	1.104
17	rs67129431	25594990	G	0.232	0.2525	A	0.4567	0.8941
3	rs2228468	42907112	A	0.3454	0.325	C	0.4569	1.096
3	rs11719562	172462291	G	0.3454	0.325	A	0.4569	1.096
6	rs2269710	30539952	G	0.3454	0.325	A	0.4569	1.096
6	rs1264440	30551286	A	0.3454	0.325	G	0.4569	1.096
16	rs7499390	54780370	G	0.3454	0.325	A	0.4569	1.096
16	rs61744665	1484528	A	0.07216	0.085	G	0.4574	0.8373
21	rs9984476	46101939	A	0.07216	0.085	G	0.4574	0.8373
6	rs3747742	41162518	G	0.317	0.3392	A	0.4575	0.9044
8	rs7832284	38915604	C	0.317	0.3392	A	0.4575	0.9044
2	rs16844275	160604379	A	0.1211	0.1075	G	0.4576	1.144
2	rs1813849	200142847	A	0.07216	0.08583	G	0.4576	0.8284
6	rs16893526	82515315	A	0.1211	0.1075	G	0.4576	1.144
10	rs1129777	91468925	C	0.317	0.3383	G	0.4576	0.9077
10	rs3758388	91488979	A	0.317	0.3383	T	0.4576	0.9077
12	rs1727307	123575742	A	0.317	0.3383	G	0.4576	0.9077
14	rs1263872	21511458	C	0.1211	0.1075	G	0.4576	1.144
14	rs1243469	21511497	G	0.1211	0.1075	A	0.4576	1.144
17	rs2812	62401118	A	0.317	0.3383	G	0.4576	0.9077
7	rs10247980	100366284	G	0.3505	0.3292	A	0.4578	1.1
14	rs6573560	65031534	G	0.3505	0.3292	A	0.4578	1.1
6	rs4959041	30077967	G	0.2629	0.2442	A	0.4581	1.104
6	rs9261438	30089280	A	0.2629	0.2442	G	0.4581	1.104
11	rs6485587	12245343	A	0.134	0.15	G	0.4581	0.877
11	rs16921260	94759494	G	0.06736	0.05686	A	0.4581	1.198
1	rs663824	43649508	G	0.2345	0.2542	A	0.4583	0.8991
3	rs7651349	11662320	A	0.2345	0.255	G	0.4583	0.8952
17	rs17645023	64917033	T	0.2345	0.2542	A	0.4583	0.8991
22	rs8142147	45038965	G	0.1778	0.1965	A	0.4583	0.8845
13	rs7332115	33147548	C	0.1598	0.1433	A	0.4584	1.137
6	rs2046210	151948366	A	0.3196	0.3408	G	0.4585	0.9084
7	rs1799884	44229068	A	0.1778	0.1958	G	0.4585	0.8882
10	rs2393967	65133156	C	0.3196	0.3408	A	0.4585	0.9084
11	rs2993051	29994465	G	0.3505	0.33	A	0.4585	1.096
14	rs7153027	92427222	C	0.3196	0.3408	A	0.4585	0.9084
1	rs35760408	20072082	A	0.06701	0.05667	G	0.4586	1.196
20	rs2255255	20032998	A	0.3531	0.3317	G	0.4586	1.1
1	rs3766465	54606804	G	0.1005	0.1154	A	0.4588	0.8567
9	rs10760500	130536717	G	0.09278	0.08	A	0.4589	1.176
14	rs3742578	57672715	A	0.09278	0.08	G	0.4589	1.176
14	rs10150771	57675185	A	0.09278	0.08	G	0.4589	1.176

14	rs9658667	93399050	A	0.09278	0.08	G	0.4589	1.176
1	rs2015352	16371067	C	0.3549	0.3344	A	0.459	1.095
22	rs5752330	26859942	G	0.2655	0.2462	A	0.459	1.106
6	rs9357271	38365873	A	0.1005	0.1158	G	0.4591	0.853
7	rs11769469	2705266	G	0.1005	0.115	A	0.4591	0.86
12	rs3827522	42853871	A	0.1005	0.115	G	0.4591	0.86
19	rs2229228	18329784	A	0.1005	0.115	G	0.4591	0.86
1	rs2275254	111861974	G	0.3531	0.3325	A	0.4592	1.096
18	rs754093	77246406	C	0.3222	0.3433	A	0.4593	0.909
17	rs8065080	3480447	A	0.3557	0.3342	G	0.4594	1.1
1	rs744487	234726012	C	0.2655	0.2467	A	0.4596	1.104
10	rs12357206	61790383	A	0.1598	0.1442	G	0.4596	1.129
16	rs7201030	76523792	A	0.2655	0.2467	G	0.4596	1.104
22	rs713998	26862212	A	0.2655	0.2467	G	0.4596	1.104
1	rs12751325	38289383	G	0.3247	0.3467	A	0.46	0.9063
6	rs7759127	31240988	C	0.1237	0.1092	A	0.46	1.152
6	rs4716346	170176648	G	0.3247	0.3467	A	0.46	0.9063
7	rs3800791	150873246	A	0.1237	0.1092	G	0.46	1.152
14	rs8006467	99400269	G	0.1237	0.1092	A	0.46	1.152
22	rs9610875	38323507	A	0.1237	0.1092	C	0.46	1.152
6	rs6936428	32739174	A	0.3582	0.3367	T	0.4601	1.1
8	rs28378047	121764843	A	0.268	0.2483	G	0.4601	1.108
11	rs226124	110710408	G	0.3582	0.3375	A	0.4607	1.096
18	rs1893566	44126909	A	0.3582	0.3375	G	0.4607	1.096
3	rs9283633	164783165	A	0.1804	0.1992	G	0.4608	0.8851
5	rs10043097	23492320	A	0.1804	0.1992	G	0.4608	0.8851
11	rs998544	56184888	G	0.3273	0.3492	A	0.4608	0.907
1	rs4661746	16736132	G	0.1804	0.1983	A	0.4609	0.8898
3	rs922948	69442637	G	0.1804	0.1983	A	0.4609	0.8898
6	rs1738074	159465977	G	0.3608	0.3392	A	0.4609	1.1
7	rs6950826	55223176	A	0.3608	0.3392	G	0.4609	1.1
12	rs2245203	52777513	G	0.1804	0.1983	A	0.4609	0.8898
12	rs1613929	52777580	C	0.1804	0.1983	A	0.4609	0.8898
6	rs200396521	31084941	T	0.03125	0.02417	A	0.461	1.303
11	rs3829224	1276327	A	0.268	0.2492	G	0.461	1.103
3	rs17404153	132163200	A	0.1366	0.1533	C	0.4611	0.8736
4	rs10007352	10447679	A	0.1366	0.1533	G	0.4611	0.8736
15	rs8040009	93044339	G	0.1366	0.1533	A	0.4611	0.8736
19	rs10407445	57649962	A	0.1366	0.1533	G	0.4611	0.8736
4	rs6832052	182066471	G	0.04639	0.03833	A	0.4612	1.22
5	rs1045706	108714298	G	0.04639	0.03833	A	0.4612	1.22
7	rs2301635	107416913	A	0.04639	0.03833	G	0.4612	1.22
12	rs3213832	9321501	A	0.04639	0.03833	G	0.4612	1.22

19	rs10420364	9868278	G	0.04639	0.03833	A	0.4612	1.22
19	rs10414485	9868404	A	0.04639	0.03833	G	0.4612	1.22
1	rs12142671	78184325	G	0.2397	0.26	A	0.4613	0.8973
2	rs1918172	156888500	G	0.2088	0.1908	C	0.4613	1.119
8	rs4732748	27634064	A	0.2088	0.1908	G	0.4613	1.119
12	rs11146963	133181399	A	0.2088	0.1908	G	0.4613	1.119
1	rs7513079	12785494	C	0.1366	0.1525	A	0.4614	0.8792
5	rs117041256	96224904	C	0.09278	0.08083	A	0.4614	1.163
11	rs201491013	72423607	A	0.007772	0.005	G	0.4614	1.559
6	rs885915	30202715	A	0.3608	0.34	G	0.4615	1.096
6	rs2844780	30204526	A	0.3608	0.34	C	0.4615	1.096
8	rs2514689	101157227	G	0.3359	0.3579	A	0.4615	0.9078
13	rs4943302	35986558	G	0.3608	0.34	A	0.4615	1.096
19	rs11085824	13001547	G	0.1624	0.1458	A	0.4615	1.135
6	rs2535335	30812497	G	0.2706	0.2508	A	0.4616	1.108
6	rs2535326	30832063	A	0.2706	0.2508	G	0.4616	1.108
12	rs73118352	64746833	A	0.2706	0.2508	G	0.4616	1.108
5	rs31594	108979718	A	0.3634	0.3417	G	0.4617	1.1
1	rs79306726	39847730	A	0.1237	0.11	G	0.4618	1.142
16	rs117105357	820938	A	0.1237	0.11	G	0.4618	1.142
4	rs17007017	142142729	A	0.3634	0.3425	G	0.4622	1.096
9	rs751543	119122342	G	0.2098	0.1925	A	0.4622	1.114
15	rs2305710	51975608	A	0.03351	0.04341	G	0.4625	0.764
1	rs34770879	32149760	A	0.03351	0.04333	G	0.4626	0.7653
2	rs11894171	21881282	A	0.03351	0.04333	G	0.4626	0.7653
2	rs7594497	55872538	G	0.03351	0.04333	A	0.4626	0.7653
6	rs2233637	34512195	C	0.1624	0.1467	G	0.4626	1.128
6	rs6569648	130349119	G	0.03351	0.04333	A	0.4626	0.7653
7	rs141576983	48452181	A	0.03351	0.04333	C	0.4626	0.7653
11	rs116926312	69063476	A	0.03351	0.04333	G	0.4626	0.7653
19	rs3745945	38673298	C	0.03351	0.04333	G	0.4626	0.7653
1	rs880315	10796866	A	0.3325	0.3533	G	0.4627	0.9116
2	rs2042996	179451420	G	0.3325	0.3533	A	0.4627	0.9116
3	rs1274972	39161456	A	0.2423	0.2625	G	0.4627	0.8983
3	rs1274971	39161464	A	0.2423	0.2625	G	0.4627	0.8983
10	rs16912145	60082668	A	0.2423	0.2625	G	0.4627	0.8983
13	rs238270	42932925	G	0.3325	0.3533	A	0.4627	0.9116
1	rs813004	72954611	C	0.05155	0.0625	A	0.4628	0.8152
7	rs78833972	47913560	A	0.05155	0.0625	C	0.4628	0.8152
9	rs16927037	990653	A	0.05155	0.0625	C	0.4628	0.8152
7	rs10755864	94463888	G	0.366	0.345	A	0.4629	1.096
3	rs3844283	10264480	C	0.1624	0.1469	G	0.463	1.126
8	rs1160133	59305290	A	0.183	0.2017	G	0.463	0.8866

16	rs11863694	12653862	G	0.183	0.2017	A	0.463	0.8866
8	rs16901979	128124916	A	0.183	0.2008	C	0.4631	0.8913
1	rs202157618	24384031	A	0.007732	0.005	C	0.4632	1.551
1	rs12748152	27138393	A	0.007732	0.005	G	0.4632	1.551
1	rs16837197	35578909	G	0.007732	0.005	A	0.4632	1.551
1	rs182877101	45446819	A	0.007732	0.005	T	0.4632	1.551
1	rs149399157	78194235	G	0.007732	0.005	A	0.4632	1.551
1	rs201707267	94476477	A	0.007732	0.005	G	0.4632	1.551
1	rs142732858	114442672	A	0.007732	0.005	G	0.4632	1.551
1	rs202171359	165652272	A	0.007732	0.005	G	0.4632	1.551
1	rs142367511	204228488	A	0.007732	0.005	G	0.4632	1.551
1	rs114563071	204412570	A	0.007732	0.005	G	0.4632	1.551
1	rs148113527	222750877	A	0.007732	0.005	G	0.4632	1.551
1	rs75568712	225211422	G	0.007732	0.005	A	0.4632	1.551
1	rs3210731	232564297	A	0.007732	0.005	T	0.4632	1.551
2	rs141291583	31572927	A	0.007732	0.005	G	0.4632	1.551
2	rs184802645	37469792	G	0.007732	0.005	C	0.4632	1.551
2	rs189732445	47602405	C	0.007732	0.005	G	0.4632	1.551
2	rs183156417	71650647	A	0.007732	0.005	G	0.4632	1.551
2	rs10497410	175263063	A	0.007732	0.005	G	0.4632	1.551
3	rs146575088	9867484	A	0.007732	0.005	G	0.4632	1.551
3	rs74773293	12857880	G	0.007732	0.005	C	0.4632	1.551
3	rs9834623	12857920	G	0.007732	0.005	A	0.4632	1.551
3	rs9838943	12858395	G	0.007732	0.005	A	0.4632	1.551
3	rs117098616	32483474	A	0.007732	0.005	G	0.4632	1.551
3	rs7634545	36545903	G	0.007732	0.005	A	0.4632	1.551
3	rs201569061	42448377	A	0.007732	0.005	G	0.4632	1.551
3	rs148573516	48658943	A	0.007732	0.005	G	0.4632	1.551
3	exm2256098	53804489	A	0.007732	0.005	G	0.4632	1.551
3	rs17053501	53804489	A	0.007732	0.005	G	0.4632	1.551
3	rs10933819	105253645	A	0.007732	0.005	G	0.4632	1.551
3	rs146718769	160156092	G	0.007732	0.005	A	0.4632	1.551
3	rs116484646	182872068	C	0.007732	0.005	A	0.4632	1.551
3	rs200352562	190326957	G	0.007732	0.005	A	0.4632	1.551
3	rs142320589	194134567	G	0.007732	0.005	A	0.4632	1.551
4	rs201359022	674892	G	0.007732	0.005	A	0.4632	1.551
4	rs75964850	39216240	A	0.007732	0.005	G	0.4632	1.551
4	rs151039635	120214277	G	0.007732	0.005	A	0.4632	1.551
4	rs199749473	146576332	A	0.007732	0.005	T	0.4632	1.551
5	rs78627784	138160400	G	0.007732	0.005	A	0.4632	1.551
5	rs182469617	149375017	A	0.007732	0.005	C	0.4632	1.551
5	rs192446755	169310757	A	0.007732	0.005	G	0.4632	1.551
5	rs193290006	176638587	G	0.007732	0.005	A	0.4632	1.551

6	rs79078661	25813093	C	0.007732	0.005	A	0.4632	1.551
6	rs114145988	32726753	A	0.007732	0.005	G	0.4632	1.551
6	rs183559588	34214514	G	0.007732	0.005	A	0.4632	1.551
6	rs75383784	34825664	C	0.007732	0.005	G	0.4632	1.551
6	rs200749169	35715420	A	0.007732	0.005	G	0.4632	1.551
6	rs149832668	75884984	A	0.007732	0.005	G	0.4632	1.551
6	rs199668467	152686022	G	0.007732	0.005	A	0.4632	1.551
6	rs114979735	155574094	A	0.007732	0.005	G	0.4632	1.551
6	rs199987597	158923669	A	0.007732	0.005	G	0.4632	1.551
7	rs139444451	1197365	G	0.007732	0.005	A	0.4632	1.551
7	rs75403455	2968245	A	0.007732	0.005	G	0.4632	1.551
7	rs200795180	20698693	A	0.007732	0.005	G	0.4632	1.551
7	rs200894470	21747364	A	0.007732	0.005	G	0.4632	1.551
7	rs8940	116146074	C	0.007732	0.005	G	0.4632	1.551
7	rs4236601	116162729	A	0.007732	0.005	G	0.4632	1.551
8	rs201403389	6296482	A	0.007732	0.005	G	0.4632	1.551
8	rs141414987	10396002	A	0.007732	0.005	C	0.4632	1.551
8	rs4151643	57228723	A	0.007732	0.005	G	0.4632	1.551
8	rs189481159	71069365	C	0.007732	0.005	A	0.4632	1.551
8	rs3739274	133900382	A	0.007732	0.005	G	0.4632	1.551
8	rs201657125	144998576	A	0.007732	0.005	G	0.4632	1.551
9	rs138080230	35043969	A	0.007732	0.005	C	0.4632	1.551
9	rs1059003	100689693	G	0.007732	0.005	A	0.4632	1.551
9	rs3747834	119460791	G	0.007732	0.005	C	0.4632	1.551
9	rs4531	136509370	A	0.007732	0.005	C	0.4632	1.551
9	rs191274326	136633707	A	0.007732	0.005	G	0.4632	1.551
9	rs55884352	138415740	A	0.007732	0.005	C	0.4632	1.551
10	rs140272734	5772716	C	0.007732	0.005	A	0.4632	1.551
10	rs180868035	33559615	C	0.007732	0.005	A	0.4632	1.551
10	rs184714916	98127968	C	0.007732	0.005	A	0.4632	1.551
10	rs58548334	100202987	G	0.007732	0.005	A	0.4632	1.551
11	rs72878024	199492	A	0.007732	0.005	G	0.4632	1.551
11	rs139424841	3128540	A	0.007732	0.005	G	0.4632	1.551
11	rs35666095	4945236	G	0.007732	0.005	A	0.4632	1.551
11	rs199990022	6807041	G	0.007732	0.005	A	0.4632	1.551
11	rs200893986	7718014	G	0.007732	0.005	A	0.4632	1.551
11	rs184345272	76255454	C	0.007732	0.005	A	0.4632	1.551
11	rs202199158	121028559	A	0.007732	0.005	C	0.4632	1.551
11	rs79071390	122827236	A	0.007732	0.005	C	0.4632	1.551
12	rs200902818	4870241	A	0.007732	0.005	G	0.4632	1.551
12	rs202221764	9885556	G	0.007732	0.005	A	0.4632	1.551
12	rs137971738	46320876	A	0.007732	0.005	C	0.4632	1.551
12	rs7134599	68500075	A	0.007732	0.005	G	0.4632	1.551

12	rs185311452	89864264	A	0.007732	0.005	G	0.4632	1.551
13	rs79836043	25000682	A	0.007732	0.005	T	0.4632	1.551
14	rs148588675	33243091	G	0.007732	0.005	A	0.4632	1.551
14	rs115865530	73673157	G	0.007732	0.005	A	0.4632	1.551
14	rs187049858	105349504	A	0.007732	0.005	G	0.4632	1.551
15	rs201822096	54307601	A	0.007732	0.005	G	0.4632	1.551
15	rs114740641	62305254	A	0.007732	0.005	G	0.4632	1.551
15	rs183862467	65223029	G	0.007732	0.005	A	0.4632	1.551
15	rs184558337	65623929	A	0.007732	0.005	G	0.4632	1.551
15	rs149121639	91548307	G	0.007732	0.005	A	0.4632	1.551
15	rs3829706	101605845	G	0.007732	0.005	A	0.4632	1.551
16	rs146563741	3274356	A	0.007732	0.005	G	0.4632	1.551
16	rs147843700	4949261	A	0.007732	0.005	G	0.4632	1.551
16	rs79129097	15808856	G	0.007732	0.005	A	0.4632	1.551
16	rs199929675	20043061	A	0.007732	0.005	G	0.4632	1.551
16	rs78964414	29912329	A	0.007732	0.005	G	0.4632	1.551
16	rs145397395	55899922	A	0.007732	0.005	G	0.4632	1.551
16	rs141266321	56659783	A	0.007732	0.005	G	0.4632	1.551
16	rs146881905	66420962	A	0.007732	0.005	G	0.4632	1.551
16	rs150644843	88781019	C	0.007732	0.005	A	0.4632	1.551
16	rs75806597	89603268	A	0.007732	0.005	G	0.4632	1.551
17	rs34067256	7592373	G	0.007732	0.005	C	0.4632	1.551
17	rs187438258	10429123	A	0.007732	0.005	G	0.4632	1.551
17	rs142387062	33510470	C	0.007732	0.005	G	0.4632	1.551
17	rs140618662	37814086	A	0.007732	0.005	G	0.4632	1.551
17	rs180974014	42987563	A	0.007732	0.005	G	0.4632	1.551
17	rs199641093	67250466	A	0.007732	0.005	G	0.4632	1.551
17	rs145463528	72341014	A	0.007732	0.005	G	0.4632	1.551
17	rs145631229	72691347	G	0.007732	0.005	A	0.4632	1.551
19	rs200206860	2405495	A	0.007732	0.005	G	0.4632	1.551
19	rs116083435	17836969	G	0.007732	0.005	A	0.4632	1.551
19	rs141241974	19653211	A	0.007732	0.005	G	0.4632	1.551
20	rs200170732	896790	A	0.007732	0.005	T	0.4632	1.551
20	rs192391500	3683889	A	0.007732	0.005	G	0.4632	1.551
20	rs199783968	3687330	G	0.007732	0.005	A	0.4632	1.551
20	rs6076550	3726565	A	0.007732	0.005	G	0.4632	1.551
20	rs117301601	47852688	A	0.007732	0.005	G	0.4632	1.551
20	rs200049003	61938856	A	0.007732	0.005	G	0.4632	1.551
20	rs200588095	62194245	A	0.007732	0.005	G	0.4632	1.551
21	rs3746867	37617929	A	0.007732	0.005	C	0.4632	1.551
21	rs4818842	47858248	G	0.007732	0.005	A	0.4632	1.551
22	rs141942830	24837041	A	0.007732	0.005	G	0.4632	1.551
22	rs112890946	43610141	A	0.007732	0.005	G	0.4632	1.551

2	rs4356603	211508287	A	0.1031	0.1183	G	0.4635	0.8564
5	rs10036748	150458146	G	0.2113	0.1933	A	0.4635	1.118
6	rs3131036	30728290	A	0.1031	0.1183	G	0.4635	0.8564
10	rs11546426	5541230	G	0.2113	0.1933	A	0.4635	1.118
12	rs3802998	56296075	C	0.03385	0.04417	G	0.4635	0.7583
17	rs222749	3495374	A	0.2113	0.1933	G	0.4635	1.118
19	rs3752141	8982271	A	0.1031	0.1183	G	0.4635	0.8564
1	rs33953680	43296195	A	0.03093	0.02417	G	0.4636	1.289
1	rs947374	201299263	A	0.03093	0.02417	G	0.4636	1.289
2	rs7419281	181001694	A	0.3686	0.3475	G	0.4636	1.096
2	rs1365776	231072709	G	0.1031	0.1175	A	0.4636	0.8633
3	rs17040196	14745857	A	0.3686	0.3475	G	0.4636	1.096
3	rs4687718	53282303	A	0.03093	0.02417	G	0.4636	1.289
6	rs144038841	31084936	C	0.03093	0.02417	G	0.4636	1.289
10	rs182512508	17127755	G	0.03093	0.02417	C	0.4636	1.289
11	rs1805002	6291120	A	0.03093	0.02417	G	0.4636	1.289
11	rs147977667	122795677	A	0.03093	0.02417	G	0.4636	1.289
16	rs142345677	84209590	A	0.03093	0.02417	G	0.4636	1.289
17	rs74526564	73662633	A	0.03093	0.02417	G	0.4636	1.289
21	rs117132686	37584306	A	0.03093	0.02417	C	0.4636	1.289
1	rs201100122	120484314	A	0.007732	0.005008	G	0.4637	1.548
2	rs184604613	54131247	A	0.007732	0.005008	T	0.4637	1.548
7	rs186591323	89984511	G	0.007732	0.005008	A	0.4637	1.548
9	rs649891	10430602	A	0.2732	0.2542	G	0.4637	1.103
19	rs139180721	7697662	A	0.007732	0.005008	G	0.4637	1.548
20	rs138612368	61444442	G	0.007732	0.005008	A	0.4637	1.548
21	rs61735841	47558552	A	0.007732	0.005008	G	0.4637	1.548
7	rs7792020	32449568	A	0.07474	0.0875	G	0.4638	0.8424
8	rs7844723	122908503	A	0.07474	0.0875	G	0.4638	0.8424
10	rs2274110	13239651	G	0.07474	0.08833	A	0.4638	0.8337
12	rs80210207	70970293	G	0.07474	0.08833	A	0.4638	0.8337
1	rs10924173	245490640	A	0.3711	0.3492	G	0.4639	1.1
3	rs4602367	17053499	A	0.3376	0.3592	G	0.4639	0.9095
11	rs7941190	59132798	C	0.3376	0.3592	G	0.4639	0.9095
11	rs117954374	55136125	A	0.03093	0.02421	G	0.464	1.287
1	rs41269473	42047300	A	0.03351	0.04417	T	0.4641	0.7502
1	rs10494067	107830858	C	0.06959	0.05833	A	0.4641	1.207
1	rs2072648	155217643	A	0.06959	0.05833	G	0.4641	1.207
3	rs75387493	12872995	A	0.06959	0.05833	G	0.4641	1.207
5	rs299295	162909716	A	0.03351	0.04417	G	0.4641	0.7502
16	rs61753378	3726075	A	0.03351	0.04417	G	0.4641	0.7502
16	rs61733127	71683718	G	0.03351	0.04417	A	0.4641	0.7502
2	rs6732185	240981375	A	0.2448	0.265	T	0.4642	0.8993

3	rs1601875	3658840	G	0.2448	0.265	A	0.4642	0.8993
17	rs9912347	5017772	G	0.1392	0.1561	A	0.4642	0.8741
19	rs80009430	56249615	G	0.1392	0.1558	C	0.4642	0.8758
7	rs6955473	90866589	A	0.3711	0.35	G	0.4643	1.096
8	rs12680114	35184361	A	0.3376	0.3583	G	0.4643	0.9128
15	rs200134036	42145167	A	0.007732	0.005017	G	0.4643	1.545
16	rs16971384	72931085	G	0.3711	0.35	A	0.4643	1.096
22	rs8136495	50943232	A	0.03351	0.04431	G	0.4643	0.7476
15	rs12917189	43023482	G	0.2448	0.2642	A	0.4644	0.9031
2	rs16853309	168103304	A	0.1624	0.1475	G	0.4645	1.12
2	rs7607246	168103925	G	0.1624	0.1475	A	0.4645	1.12
2	rs61750760	168104627	A	0.1624	0.1475	G	0.4645	1.12
2	rs59889092	168104988	A	0.1624	0.1475	C	0.4645	1.12
2	rs16853328	168106085	A	0.1624	0.1475	G	0.4645	1.12
2	rs16853331	168107155	A	0.1624	0.1475	G	0.4645	1.12
2	rs3749004	168107491	G	0.1624	0.1475	A	0.4645	1.12
6	rs2233639	34512064	A	0.1624	0.1475	G	0.4645	1.12
7	rs10951942	48081095	A	0.3402	0.3617	C	0.4647	0.9101
7	rs13237362	11349162	A	0.3737	0.3525	G	0.4649	1.096
5	rs37369	35037115	G	0.3711	0.3508	A	0.465	1.092
3	rs35000036	123190731	A	0.2758	0.2567	G	0.4651	1.103
1	rs17118103	59148118	A	0.1856	0.2042	T	0.4652	0.8881
1	rs1805049	216348764	G	0.1856	0.2042	A	0.4652	0.8881
16	rs4843526	87148749	G	0.3428	0.365	A	0.4653	0.9074
3	rs6765537	14755572	G	0.3763	0.355	A	0.4656	1.096
3	rs6790129	14755617	C	0.3763	0.355	G	0.4656	1.096
13	rs77685055	76055602	A	0.2474	0.2675	G	0.4656	0.9003
3	rs446644	137787192	A	0.3737	0.3533	G	0.4657	1.092
7	rs6465633	77043863	G	0.3737	0.3533	A	0.4657	1.092
11	rs17686210	123848121	G	0.1263	0.1125	A	0.466	1.14
11	rs17759513	123848212	A	0.1263	0.1125	G	0.466	1.14
12	rs7953742	90390724	A	0.3454	0.3675	G	0.466	0.908
18	rs2303507	34398883	A	0.1263	0.1125	G	0.466	1.14
15	rs11071657	62433962	G	0.3454	0.3667	A	0.4661	0.9112
3	rs7624750	193334991	A	0.3789	0.3575	G	0.4662	1.096
8	rs7843479	21820813	A	0.3789	0.3575	C	0.4662	1.096
5	rs30168	13719089	A	0.3763	0.3558	G	0.4663	1.092
15	rs4380028	79111093	A	0.3763	0.3558	G	0.4663	1.092
21	rs2839077	47417303	A	0.3763	0.3558	G	0.4663	1.092
15	rs79562667	74337273	A	0.002577	0.007563	G	0.4665	0.3391
1	rs61747667	14105049	A	0.002577	0.0075	C	0.4666	0.3419
1	rs17350795	14105639	A	0.002577	0.0075	G	0.4666	0.3419
1	rs116238585	14107135	G	0.002577	0.007513	C	0.4666	0.3414

1	rs77393068	34190185	G	0.002577	0.0075	A	0.4666	0.3419
1	rs199962081	34190962	A	0.002577	0.0075	G	0.4666	0.3419
1	rs114043049	60103965	A	0.002577	0.0075	G	0.4666	0.3419
1	rs191269282	85484931	A	0.002577	0.0075	C	0.4666	0.3419
1	rs155906	189574444	G	0.002577	0.0075	A	0.4666	0.3419
1	rs201516065	204226775	A	0.002577	0.0075	C	0.4666	0.3419
1	rs59221415	213186591	C	0.002577	0.0075	A	0.4666	0.3419
1	rs143240767	216052322	A	0.002577	0.0075	G	0.4666	0.3419
1	rs3737978	218480958	G	0.002577	0.0075	A	0.4666	0.3419
1	rs201394768	223438018	C	0.002577	0.0075	A	0.4666	0.3419
1	rs141400629	234594981	A	0.002577	0.0075	C	0.4666	0.3419
2	rs201436177	141299349	A	0.002577	0.0075	G	0.4666	0.3419
2	rs148250645	168726199	A	0.002577	0.0075	G	0.4666	0.3419
2	rs34218846	209108317	A	0.002577	0.0075	G	0.4666	0.3419
2	rs149295795	228883868	A	0.002577	0.0075	G	0.4666	0.3419
3	rs147042905	44635903	A	0.002577	0.0075	G	0.4666	0.3419
3	rs148445016	46005882	A	0.002577	0.0075	G	0.4666	0.3419
3	rs146998598	62860387	C	0.002577	0.0075	G	0.4666	0.3419
3	rs145518557	122354759	A	0.002577	0.0075	G	0.4666	0.3419
3	rs138684820	127336236	A	0.002577	0.0075	G	0.4666	0.3419
3	rs118182811	152553672	A	0.002577	0.0075	G	0.4666	0.3419
4	rs17846992	26483276	A	0.002577	0.0075	G	0.4666	0.3419
4	rs202058615	154649360	A	0.002577	0.0075	G	0.4666	0.3419
4	rs201741692	187540634	A	0.002577	0.0075	G	0.4666	0.3419
5	rs2303986	10450146	A	0.002577	0.0075	G	0.4666	0.3419
5	rs146628734	41015521	A	0.002577	0.0075	G	0.4666	0.3419
5	rs76289268	140730210	A	0.002577	0.0075	G	0.4666	0.3419
5	rs201613360	141242750	A	0.002577	0.0075	G	0.4666	0.3419
5	rs138316493	171777382	A	0.002577	0.0075	G	0.4666	0.3419
6	rs138329459	7585950	C	0.002577	0.0075	A	0.4666	0.3419
6	rs2523942	29925086	C	0.002577	0.0075	A	0.4666	0.3419
6	rs187982887	30865148	G	0.002577	0.0075	A	0.4666	0.3419
6	rs17509489	33036549	G	0.002577	0.0075	A	0.4666	0.3419
6	rs115722167	33037060	A	0.002577	0.0075	G	0.4666	0.3419
6	rs41550812	33037454	C	0.002577	0.0075	A	0.4666	0.3419
6	rs41559316	33037456	A	0.002577	0.0075	G	0.4666	0.3419
6	rs2308907	33037619	A	0.002577	0.0075	C	0.4666	0.3419
6	rs41558014	33043865	A	0.002577	0.0075	G	0.4666	0.3419
6	rs61743530	43191017	A	0.002577	0.0075	G	0.4666	0.3419
6	rs188770881	46659565	G	0.002577	0.0075	A	0.4666	0.3419
6	rs141423190	89977789	C	0.002577	0.0075	A	0.4666	0.3419
6	rs191116854	138763236	A	0.002577	0.0075	G	0.4666	0.3419
6	rs80265744	152614868	A	0.002577	0.0075	G	0.4666	0.3419

8	rs11782386	9201787	A	0.002577	0.0075	G	0.4666	0.3419
8	rs192236678	90965508	A	0.002577	0.0075	C	0.4666	0.3419
8	rs201405525	91057221	A	0.002577	0.0075	G	0.4666	0.3419
8	rs201845067	106813325	A	0.002577	0.0075	G	0.4666	0.3419
9	rs67710536	19376255	C	0.002577	0.0075	A	0.4666	0.3419
9	rs201407189	97887391	A	0.002577	0.0075	G	0.4666	0.3419
10	rs187831118	27508709	A	0.002577	0.0075	G	0.4666	0.3419
10	rs147098184	44052431	A	0.002577	0.0075	G	0.4666	0.3419
10	rs118189413	50374953	G	0.002577	0.0075	C	0.4666	0.3419
10	rs202152796	102054309	A	0.002577	0.0075	G	0.4666	0.3419
11	rs201497459	299405	A	0.002577	0.0075	G	0.4666	0.3419
11	rs146006146	4944755	A	0.002577	0.0075	G	0.4666	0.3419
11	rs141030184	6007469	A	0.002577	0.007513	G	0.4666	0.3414
11	rs201306863	26725160	G	0.002577	0.0075	A	0.4666	0.3419
11	rs140162276	48238704	A	0.002577	0.0075	G	0.4666	0.3419
11	rs145476036	55927736	A	0.002577	0.0075	G	0.4666	0.3419
11	rs79893604	74408274	A	0.002577	0.0075	G	0.4666	0.3419
11	rs76853537	77961419	A	0.002577	0.0075	C	0.4666	0.3419
11	rs199670970	102592381	A	0.002577	0.0075	C	0.4666	0.3419
11	rs114144322	108384273	G	0.002577	0.0075	A	0.4666	0.3419
12	rs142841462	7522171	A	0.002577	0.0075	C	0.4666	0.3419
12	rs17109924	71977787	G	0.002577	0.0075	A	0.4666	0.3419
12	rs11541286	72008421	G	0.002577	0.0075	A	0.4666	0.3419
12	rs15895	113448288	A	0.002577	0.0075	G	0.4666	0.3419
13	rs80358457	32907359	C	0.002577	0.0075	A	0.4666	0.3419
13	rs201754085	37393924	A	0.002577	0.0075	G	0.4666	0.3419
13	rs200588795	45978486	C	0.002577	0.0075	A	0.4666	0.3419
14	rs78610973	70989851	A	0.002577	0.0075	G	0.4666	0.3419
14	rs201591982	74989495	A	0.002577	0.0075	C	0.4666	0.3419
15	rs2280279	63029160	A	0.002577	0.0075	C	0.4666	0.3419
15	rs3803381	68497633	A	0.002577	0.0075	G	0.4666	0.3419
16	rs199863486	1544520	A	0.002577	0.007513	C	0.4666	0.3414
16	rs139187014	2017335	A	0.002577	0.0075	G	0.4666	0.3419
16	rs78644690	2550849	A	0.002577	0.0075	G	0.4666	0.3419
16	rs150352299	12061606	A	0.002577	0.0075	G	0.4666	0.3419
16	rs147978363	22268677	A	0.002577	0.0075	G	0.4666	0.3419
16	rs183489969	23632742	C	0.002577	0.0075	G	0.4666	0.3419
17	rs145812991	3195810	A	0.002577	0.0075	C	0.4666	0.3419
17	rs77857768	17425684	A	0.002577	0.0075	G	0.4666	0.3419
17	rs141146547	36482654	C	0.002577	0.0075	A	0.4666	0.3419
17	rs145659794	58503541	A	0.002577	0.0075	G	0.4666	0.3419
17	rs201425153	78444672	G	0.002577	0.0075	A	0.4666	0.3419
17	rs199573019	79220258	A	0.002577	0.00755	G	0.4666	0.3396

19	rs144463824	40827881	A	0.002577	0.0075	G	0.4666	0.3419
19	rs146772061	44661040	A	0.002577	0.0075	G	0.4666	0.3419
19	rs150036211	48734214	A	0.002577	0.0075	G	0.4666	0.3419
19	rs200973162	50436269	A	0.002577	0.0075	G	0.4666	0.3419
19	rs16986050	55401170	G	0.002577	0.0075	A	0.4666	0.3419
20	rs117773313	1286349	A	0.002577	0.0075	T	0.4666	0.3419
20	rs199674138	10639284	A	0.002577	0.0075	G	0.4666	0.3419
20	rs113976949	50238625	A	0.002577	0.0075	G	0.4666	0.3419
20	rs201750561	62051020	A	0.002577	0.0075	C	0.4666	0.3419
22	rs2285178	38205989	G	0.3479	0.37	A	0.4667	0.9086
14	rs1952153	87775721	C	0.3479	0.3692	A	0.4668	0.9118
14	rs150688	60582053	G	0.3814	0.36	A	0.4669	1.096
14	rs308998	60585131	G	0.3814	0.36	A	0.4669	1.096
14	rs167437	60591887	A	0.3814	0.36	G	0.4669	1.096
17	rs3091326	32601183	A	0.3789	0.3583	G	0.4669	1.092
3	rs10049246	185686741	A	0.25	0.27	G	0.467	0.9012
20	rs4911274	29968863	G	0.25	0.27	A	0.467	0.9012
2	rs75802875	168101570	G	0.06959	0.05917	A	0.4671	1.189
2	rs77219745	168103418	A	0.06959	0.05917	G	0.4671	1.189
6	rs6926723	30882803	A	0.06959	0.05917	G	0.4671	1.189
7	rs2234000	141478509	A	0.06959	0.05917	G	0.4671	1.189
4	rs17589290	112923237	C	0.1418	0.1583	G	0.4673	0.878
22	rs8418	44379838	A	0.1418	0.1583	G	0.4673	0.878
4	rs61659343	139385949	C	0.25	0.2692	A	0.4674	0.9051
15	rs3803357	40751555	C	0.1649	0.15	A	0.4674	1.119
3	rs9824340	43902170	A	0.3505	0.3717	G	0.4675	0.9124
4	rs12639834	126426695	A	0.384	0.3625	G	0.4675	1.096
6	rs7750165	163697439	G	0.384	0.3625	A	0.4675	1.096
20	rs203534	1214814	C	0.1881	0.2058	A	0.4675	0.8941
4	rs727153	155654421	A	0.1418	0.1592	G	0.4676	0.8725
6	rs2022059	32156489	G	0.1418	0.1592	C	0.4676	0.8725
10	rs4838554	50916542	A	0.1418	0.1592	G	0.4676	0.8725
19	rs2280401	50000009	A	0.1418	0.1592	G	0.4676	0.8725
1	rs1625579	98502934	C	0.04897	0.04	A	0.4678	1.236
2	rs2360090	195344783	G	0.1057	0.1208	A	0.4678	0.8597
2	rs4674941	225670901	C	0.04897	0.04	G	0.4678	1.236
4	rs11559290	159601676	G	0.04897	0.04	A	0.4678	1.236
6	rs9277932	33141253	A	0.1057	0.1208	C	0.4678	0.8597
8	rs117084443	1905354	G	0.04897	0.04	C	0.4678	1.236
9	rs3824421	712757	A	0.04897	0.04	G	0.4678	1.236
13	rs7988277	20411701	A	0.04897	0.04	G	0.4678	1.236
13	rs41292167	20413021	G	0.04897	0.04	A	0.4678	1.236
13	rs9579718	20425948	G	0.04897	0.04	A	0.4678	1.236

22	rs20551	41548008	G	0.04897	0.04	A	0.4678	1.236
12	rs79087668	42863266	A	0.1057	0.12	G	0.468	0.8665
10	rs7895853	117282016	G	0.3866	0.365	A	0.4681	1.096
20	rs4812381	37987560	G	0.384	0.3633	A	0.4681	1.092
9	rs7030192	113169631	A	0.3531	0.3742	G	0.4682	0.9129
15	rs8035524	22816713	A	0.3531	0.3742	C	0.4682	0.9129
5	rs1862136	79616083	C	0.04897	0.04013	G	0.4684	1.231
11	rs10840031	8410621	G	0.3557	0.3775	A	0.4686	0.9103
13	rs2391191	106119446	G	0.3866	0.3658	A	0.4686	1.093
15	rs2241423	68086838	G	0.3866	0.3658	A	0.4686	1.093
5	rs1159930	77768943	A	0.3892	0.3675	G	0.4687	1.097
1	rs6673156	14579733	A	0.3557	0.3767	G	0.4689	0.9135
16	rs813913	6554253	C	0.3665	0.3875	A	0.469	0.9144
19	rs12982744	2177193	C	0.2835	0.2642	G	0.469	1.102
2	rs11681642	71163086	G	0.3582	0.38	A	0.4692	0.9108
12	rs328744	72181905	G	0.1907	0.2092	A	0.4694	0.891
5	rs668732	41574561	A	0.3582	0.3792	C	0.4696	0.914
6	rs6902018	155206501	G	0.3582	0.3792	A	0.4696	0.914
6	rs1048197	109762332	A	0.1907	0.2083	G	0.4697	0.8955
9	rs4743034	109632353	A	0.2191	0.2008	G	0.4697	1.116
12	rs17126180	52263956	G	0.1907	0.2083	C	0.4697	0.8955
19	rs501257	11536575	G	0.1907	0.2083	A	0.4697	0.8955
1	rs1031191	188835473	C	0.07732	0.09098	A	0.4698	0.8372
3	rs9828502	111996554	G	0.3918	0.3708	A	0.4698	1.093
3	rs6787064	121039333	G	0.2552	0.275	A	0.4698	0.9031
6	rs2876098	7374207	C	0.07732	0.09083	A	0.4698	0.8388
4	rs17071906	182537084	A	0.3608	0.3825	C	0.4699	0.9113
5	rs868649	640705	G	0.3608	0.3825	A	0.4699	0.9113
6	rs2735014	29805809	A	0.3608	0.3825	C	0.4699	0.9113
6	rs2269703	30658385	A	0.07732	0.09	G	0.4699	0.8473
2	rs12477314	239877148	A	0.2835	0.265	G	0.47	1.097
10	rs6480771	76861680	A	0.07853	0.09244	G	0.47	0.8368
7	rs61732373	6550540	A	0.01823	0.01345	G	0.4701	1.362
5	rs1129495	115428381	C	0.1675	0.1525	A	0.4702	1.118
1	rs4656077	89479063	A	0.2861	0.2667	G	0.4703	1.102
2	rs3213787	45646824	G	0.1443	0.1608	A	0.4703	0.8801
5	rs9285647	152279502	A	0.3943	0.3733	G	0.4703	1.093
19	rs1688005	35648365	A	0.1443	0.1608	C	0.4703	0.8801
6	rs9402515	133860345	G	0.3634	0.385	A	0.4705	0.9119
9	rs7018507	75877116	G	0.3634	0.385	A	0.4705	0.9119
14	rs2274068	36153177	A	0.3969	0.375	G	0.4705	1.097
17	rs3816995	71335260	A	0.1443	0.1617	G	0.4705	0.8747
18	rs4369779	20735408	A	0.2191	0.2017	G	0.4705	1.111

22	rs2294918	44342116	A	0.1443	0.1617	G	0.4705	0.8747
2	rs3731881	219920412	G	0.2861	0.2671	A	0.4706	1.099
1	rs140764403	980552	A	0.01804	0.01333	G	0.4707	1.36
2	rs61746568	26750782	A	0.01804	0.01333	G	0.4707	1.36
2	rs201206447	32770913	A	0.01804	0.01333	G	0.4707	1.36
2	rs117488425	158272220	A	0.01804	0.01333	G	0.4707	1.36
3	rs76948084	35763323	A	0.01804	0.01333	G	0.4707	1.36
3	rs188353907	52557921	A	0.01804	0.01333	G	0.4707	1.36
3	rs17740066	121100283	A	0.01804	0.01333	G	0.4707	1.36
3	rs181701225	127295510	A	0.01804	0.01333	G	0.4707	1.36
5	rs145291543	5239375	A	0.01804	0.01333	G	0.4707	1.36
5	rs143806879	79734201	G	0.01804	0.01333	A	0.4707	1.36
5	rs74844425	115177207	G	0.01804	0.01333	A	0.4707	1.36
5	rs17844262	140182777	A	0.01804	0.01333	C	0.4707	1.36
5	rs117169793	175959412	A	0.01804	0.01333	G	0.4707	1.36
6	rs141384205	51921514	A	0.01804	0.01333	G	0.4707	1.36
7	rs185010988	11457127	A	0.01804	0.01333	C	0.4707	1.36
7	rs78722842	30890161	A	0.01804	0.01333	G	0.4707	1.36
7	rs150090274	91747869	G	0.01804	0.01333	A	0.4707	1.36
8	rs184845115	3165342	A	0.01804	0.01333	G	0.4707	1.36
8	rs147488061	42711517	G	0.01804	0.01333	A	0.4707	1.36
10	rs2231682	101483799	A	0.01804	0.01333	G	0.4707	1.36
11	rs2298808	73071552	A	0.01804	0.01333	C	0.4707	1.36
12	rs11616188	6502742	A	0.01804	0.01333	G	0.4707	1.36
12	rs16940680	110352361	A	0.01804	0.01333	G	0.4707	1.36
12	rs184510019	121471285	A	0.01804	0.01333	C	0.4707	1.36
14	rs75047901	65035067	A	0.01804	0.01333	C	0.4707	1.36
17	rs140932272	1384031	A	0.01804	0.01333	G	0.4707	1.36
17	rs143366707	9683193	A	0.01804	0.01333	C	0.4707	1.36
19	rs142714688	17132869	A	0.01804	0.01333	G	0.4707	1.36
19	rs138799785	34263930	G	0.01804	0.01333	A	0.4707	1.36
21	rs75396112	45681109	A	0.01804	0.01333	G	0.4707	1.36
22	rs185670461	31500991	A	0.01804	0.01333	G	0.4707	1.36
2	rs1250229	216304384	A	0.09794	0.085	G	0.4708	1.169
6	rs2233977	31079894	G	0.3969	0.3758	A	0.4708	1.093
15	rs28393461	101837572	A	0.09794	0.085	G	0.4708	1.169
19	rs78536254	5772897	A	0.3969	0.3758	G	0.4708	1.093
14	rs6574179	74891005	A	0.3634	0.3842	G	0.4709	0.9151
17	rs7502216	36612948	A	0.3634	0.3842	C	0.4709	0.9151
17	rs16965350	36614563	A	0.3634	0.3842	G	0.4709	0.9151
3	rs28516377	111918215	A	0.3995	0.3775	G	0.471	1.097
3	rs9288938	111921116	A	0.3995	0.3775	C	0.471	1.097
1	rs4656078	89479074	A	0.2861	0.2676	G	0.4711	1.097

13	rs3825491	109661359	C	0.366	0.3875	G	0.4711	0.9124
5	rs139422809	146030225	A	0.01804	0.01336	G	0.4712	1.357
22	rs185039637	43623418	G	0.01804	0.01336	C	0.4712	1.357
3	rs9818117	132294075	G	0.05412	0.06583	A	0.4713	0.812
6	rs365053	32195988	C	0.3995	0.3783	A	0.4713	1.093
17	rs60430206	9604928	A	0.05412	0.06583	G	0.4713	0.812
18	rs302043	54117145	G	0.3995	0.3783	A	0.4713	1.093
9	rs2039381	21481483	A	0.1701	0.1542	G	0.4714	1.125
2	rs192608	122392439	A	0.2887	0.2692	G	0.4715	1.102
9	rs10757740	28415970	G	0.2887	0.2692	A	0.4715	1.102
14	rs7142425	82291042	G	0.366	0.3867	A	0.4715	0.9156
2	rs76754479	170465182	A	0.02062	0.02833	G	0.4716	0.722
3	rs9836421	78280658	G	0.3963	0.4182	A	0.4716	0.9132
3	rs11924705	162161616	G	0.05412	0.065	A	0.4716	0.8231
5	rs76872483	7835545	A	0.05412	0.065	G	0.4716	0.8231
6	rs9396946	170033086	G	0.3686	0.39	A	0.4716	0.9129
7	rs190543052	120776099	A	0.02062	0.02833	C	0.4716	0.722
8	rs28455997	3076959	G	0.05412	0.065	A	0.4716	0.8231
8	rs13277736	68277883	G	0.3686	0.3908	A	0.4716	0.9097
9	rs643531	15296034	C	0.02062	0.02833	A	0.4716	0.722
9	rs77358650	131343207	A	0.02062	0.02833	G	0.4716	0.722
10	rs77502204	15138762	A	0.05412	0.065	C	0.4716	0.8231
10	rs1484170	82699491	G	0.02062	0.02833	A	0.4716	0.722
13	rs3742192	111279844	A	0.05412	0.065	G	0.4716	0.8231
13	rs6046	113773159	A	0.05412	0.065	G	0.4716	0.8231
15	rs16972583	81234287	G	0.02062	0.02833	C	0.4716	0.722
16	rs147672861	72828867	G	0.02062	0.02833	A	0.4716	0.722
17	rs62000365	48218672	A	0.02062	0.02833	G	0.4716	0.722
20	rs11570255	57875916	A	0.02062	0.02833	G	0.4716	0.722
7	rs3800569	138411425	G	0.1933	0.2108	A	0.4718	0.8969
12	rs11108222	96124901	G	0.1933	0.2108	A	0.4718	0.8969
17	rs60994383	56247101	A	0.1933	0.2108	C	0.4718	0.8969
15	rs11071896	66821250	G	0.1082	0.1233	A	0.472	0.8628
16	rs11646374	89857935	A	0.1082	0.1233	G	0.472	0.8628
1	rs140243550	238050750	A	0.02062	0.02917	C	0.4721	0.7008
2	rs36096257	122038783	A	0.02062	0.02917	G	0.4721	0.7008
3	rs9872691	111985107	G	0.3995	0.3792	A	0.4721	1.089
9	rs180968644	95077126	A	0.02062	0.02917	G	0.4721	0.7008
10	rs138712415	118385567	G	0.02062	0.02917	A	0.4721	0.7008
11	rs11600179	6578004	A	0.02062	0.02917	G	0.4721	0.7008
19	rs74530614	35843347	A	0.02062	0.02917	C	0.4721	0.7008
19	rs1051303	41118056	G	0.3711	0.3933	A	0.4721	0.9103
19	rs8107014	41127465	A	0.3711	0.3933	G	0.4721	0.9103

19	rs12460394	49344521	A	0.02062	0.02917	G	0.4721	0.7008
20	rs148237195	39983322	A	0.02062	0.02917	G	0.4721	0.7008
3	rs6809854	18784423	G	0.1088	0.1233	A	0.4722	0.8679
6	rs2395110	32215876	G	0.3711	0.3925	A	0.4722	0.9134
10	rs2421013	124089036	G	0.1082	0.1225	A	0.4722	0.8695
20	rs7269320	3846397	A	0.1082	0.1225	G	0.4722	0.8695
11	rs3862618	124742365	A	0.2887	0.27	G	0.4724	1.097
12	rs4760168	58087737	C	0.2603	0.2808	A	0.4724	0.9012
1	rs3176443	177247854	C	0.2216	0.2042	G	0.4725	1.11
1	rs4915154	198671653	G	0.2216	0.2042	A	0.4725	1.11
6	rs727979	149593920	A	0.2216	0.2042	G	0.4725	1.11
15	rs77918503	44967847	G	0.07216	0.06083	A	0.4725	1.201
12	rs10747756	55863381	A	0.1314	0.1167	G	0.4726	1.146
4	rs6821574	164801854	A	0.2912	0.2717	G	0.4727	1.102
3	rs9838815	40129206	A	0.3737	0.395	G	0.4728	0.9139
6	rs409359	159944996	G	0.3737	0.395	A	0.4728	0.9139
7	rs12669573	145193978	G	0.3737	0.395	A	0.4728	0.9139
22	rs6518682	30612949	G	0.4072	0.3858	A	0.4728	1.093
2	rs6435957	217878209	A	0.09794	0.08583	G	0.4729	1.156
17	rs7218767	74014634	A	0.09794	0.08583	C	0.4729	1.156
1	rs12564525	47607281	G	0.4215	0.4	A	0.473	1.093
1	rs2304305	55062960	A	0.1701	0.155	G	0.473	1.117
5	rs4701151	178886655	A	0.2912	0.2721	G	0.4731	1.099
8	rs10958359	54340446	C	0.3763	0.3975	A	0.4733	0.9144
13	rs10047727	22317190	G	0.4098	0.3883	A	0.4733	1.094
20	rs6055065	7482971	A	0.4098	0.3883	G	0.4733	1.094
5	rs7712137	76381287	G	0.4072	0.3867	A	0.4735	1.09
11	rs10792156	58034543	G	0.4072	0.3867	A	0.4735	1.09
1	rs11203366	17657534	G	0.3789	0.4008	A	0.4736	0.9118
3	rs138977513	32031852	A	0.04897	0.04083	G	0.4736	1.21
6	rs1748168	114848711	A	0.2629	0.2833	G	0.4736	0.9021
6	rs10499138	128492182	T	0.04897	0.04083	A	0.4736	1.21
7	rs714438	77982305	G	0.2629	0.2833	A	0.4736	0.9021
12	rs7134375	20473758	A	0.2629	0.2833	C	0.4736	0.9021
19	rs79729613	46973338	A	0.04897	0.04083	G	0.4736	1.21
20	rs112731808	31812206	G	0.04897	0.04083	A	0.4736	1.21
4	rs7041	72618334	C	0.2629	0.2825	A	0.4737	0.9058
7	rs12536928	21765452	A	0.2216	0.205	G	0.4738	1.104
14	rs11848280	94358100	A	0.2216	0.205	G	0.4738	1.104
1	rs10794531	26694245	A	0.4098	0.3892	G	0.4739	1.09
1	rs10493485	72071129	A	0.3789	0.4	T	0.4739	0.9149
2	rs11901486	206830563	A	0.3789	0.4	C	0.4739	0.9149
3	rs1127732	33905566	A	0.3789	0.4	G	0.4739	0.9149

9	rs2905072	135845035	G	0.3789	0.4	A	0.4739	0.9149
16	rs11645831	60241024	A	0.4098	0.3892	G	0.4739	1.09
16	rs1862737	75281964	A	0.3789	0.4	C	0.4739	0.9149
15	rs3743137	40564576	A	0.1314	0.1175	G	0.474	1.137
17	rs3744032	73969835	A	0.1314	0.1175	G	0.474	1.137
7	rs6950676	140856581	A	0.3814	0.4033	G	0.4741	0.9123
7	rs891511	150704843	G	0.4149	0.3932	A	0.4741	1.095
7	rs3800939	102574715	G	0.1727	0.1567	A	0.4742	1.124
9	rs78403475	139235606	C	0.1727	0.1567	G	0.4742	1.124
1	rs4478844	248129240	A	0.4124	0.3917	G	0.4743	1.09
3	rs1027242	167578227	G	0.4149	0.3933	A	0.4743	1.094
7	rs6977660	19805480	G	0.3814	0.4025	A	0.4744	0.9154
11	rs1453542	59224885	C	0.2242	0.2067	G	0.4744	1.11
11	rs308326	67772869	G	0.3814	0.4025	A	0.4744	0.9154
11	rs12282288	93129484	G	0.3814	0.4023	A	0.4744	0.916
7	rs3807125	128354937	G	0.384	0.4058	A	0.4746	0.9127
19	rs231237	36249521	A	0.4149	0.3942	G	0.4747	1.09
1	rs3768080	236179869	A	0.2655	0.2858	G	0.4748	0.903
6	rs3800298	43252029	G	0.2655	0.2858	A	0.4748	0.903
11	rs17856376	69063572	G	0.2938	0.275	A	0.4748	1.097
14	rs1121900	42731966	G	0.2655	0.2858	A	0.4748	0.903
10	rs10904134	3948928	A	0.384	0.405	G	0.4749	0.9159
6	rs10484246	9451197	T	0.4201	0.3982	A	0.475	1.095
6	rs953549	128709493	A	0.2655	0.285	G	0.475	0.9067
1	rs3124314	152037515	A	0.3866	0.4083	G	0.4751	0.9132
7	rs2024408	38432937	G	0.4201	0.3983	A	0.4751	1.094
7	rs4917014	50305863	C	0.3866	0.4083	A	0.4751	0.9132
10	rs4752384	121871353	G	0.4201	0.3983	A	0.4751	1.094
1	rs629301	109818306	C	0.07216	0.06167	A	0.4753	1.183
2	rs117048449	152534545	G	0.07216	0.06167	A	0.4753	1.183
11	rs3750913	71726168	C	0.07216	0.06167	G	0.4753	1.183
2	rs4973044	228843948	G	0.3866	0.4075	A	0.4754	0.9164
15	rs744910	67446785	A	0.3866	0.4075	G	0.4754	0.9164
4	rs6812193	77198986	A	0.0799	0.09333	G	0.4755	0.8435
5	rs13359925	106005425	A	0.0799	0.09333	G	0.4755	0.8435
5	rs61739424	177683905	A	0.0799	0.09333	G	0.4755	0.8435
6	rs4713668	33690796	G	0.1985	0.2167	A	0.4755	0.8951
8	rs79983029	25364811	G	0.03608	0.04583	A	0.4755	0.7793
15	rs55799438	40544493	G	0.03608	0.04583	A	0.4755	0.7793
15	rs55974545	40544520	G	0.03608	0.04583	C	0.4755	0.7793
15	rs55863440	40544617	A	0.03608	0.04583	G	0.4755	0.7793
15	rs56218866	78922229	G	0.03608	0.04583	A	0.4755	0.7793
18	rs2282632	31320229	A	0.0799	0.09333	G	0.4755	0.8435

19	rs45574836	1806667	A	0.0799	0.09333	G	0.4755	0.8435
2	rs11124406	35190105	A	0.3892	0.4108	G	0.4756	0.9137
3	rs6801307	176674598	G	0.4201	0.3992	A	0.4756	1.09
4	rs278981	40428010	A	0.4201	0.3992	G	0.4756	1.09
5	rs11134371	9792158	G	0.4201	0.3992	A	0.4756	1.09
11	rs1042780	86161388	G	0.1985	0.2175	C	0.4756	0.8907
17	rs936656	33433820	A	0.4227	0.4008	G	0.4756	1.094
2	rs815804	47382342	A	0.0799	0.0925	C	0.4757	0.8519
2	rs75295519	152235888	A	0.0799	0.0925	G	0.4757	0.8519
11	rs10768634	5193410	G	0.1727	0.1575	A	0.4757	1.117
18	rs62081533	6993673	G	0.0799	0.0925	A	0.4757	0.8519
4	rs2610998	180487431	A	0.3892	0.41	C	0.4759	0.9168
1	rs35196193	16577794	A	0.03351	0.02583	G	0.476	1.307
2	rs36021078	68794483	A	0.03351	0.02583	C	0.476	1.307
2	rs75048316	201437817	G	0.03351	0.02583	A	0.476	1.307
3	rs2291465	112732840	A	0.03351	0.02583	G	0.476	1.307
7	rs77471815	150935157	A	0.03351	0.02583	G	0.476	1.307
8	rs4871385	124553222	C	0.03351	0.02583	G	0.476	1.307
8	rs35844464	145154099	C	0.03351	0.02583	G	0.476	1.307
12	rs117402032	7241211	C	0.03351	0.02583	A	0.476	1.307
12	rs74357548	121618235	A	0.03351	0.02583	G	0.476	1.307
14	rs2239633	23589057	A	0.3918	0.4133	G	0.476	0.9142
17	rs9901675	7484812	A	0.03351	0.02583	G	0.476	1.307
17	rs8066889	79650828	C	0.3005	0.2805	A	0.476	1.102
18	rs9951065	40182141	G	0.3918	0.4133	A	0.476	0.9142
18	rs7241918	47160953	C	0.1108	0.1258	A	0.476	0.8659
20	rs2281534	62492922	T	0.1108	0.1258	A	0.476	0.8659
8	rs12546220	70373728	G	0.268	0.2883	A	0.4761	0.9039
8	rs1016343	128093297	A	0.268	0.2883	G	0.4761	0.9039
9	rs7850542	90500405	A	0.1495	0.1658	C	0.4761	0.8841
15	rs8023621	75970070	A	0.1495	0.1658	G	0.4761	0.8841
10	rs1209750	37488689	C	0.268	0.2875	G	0.4762	0.9075
14	rs34198557	100375707	A	0.4375	0.4162	G	0.4762	1.091
12	rs3741913	6457196	A	0.00266	0.008361	C	0.4763	0.3163
1	rs11206801	56850686	A	0.1005	0.0875	C	0.4764	1.165
6	rs2076540	32317276	G	0.4278	0.4058	A	0.4764	1.095
2	rs79616802	98277090	G	0.03608	0.04667	A	0.4766	0.7647
6	rs2269705	30656847	G	0.03608	0.04667	A	0.4766	0.7647
11	rs4245219	57996312	G	0.03608	0.04667	C	0.4766	0.7647
11	rs56302613	58034597	C	0.03608	0.04667	G	0.4766	0.7647
11	rs7111538	58034651	A	0.03608	0.04667	G	0.4766	0.7647
1	rs2220948	97110940	A	0.4278	0.4067	G	0.4767	1.091
4	rs4697308	22491705	A	0.4278	0.4067	G	0.4767	1.091

10	rs10887741	89443310	G	0.4278	0.4067	A	0.4767	1.091
11	rs2280134	3681519	G	0.3969	0.4192	A	0.4768	0.9119
1	rs113490655	89478868	A	0.1753	0.1592	G	0.4769	1.123
5	rs636926	72419617	C	0.3943	0.415	A	0.4769	0.9178
7	rs4729624	100529261	G	0.1753	0.1592	A	0.4769	1.123
8	rs10955282	103176946	A	0.3943	0.415	G	0.4769	0.9178
11	rs589691	64525216	G	0.3943	0.415	A	0.4769	0.9178
5	rs3777204	95234350	G	0.4304	0.4092	A	0.4771	1.091
6	rs437179	31929014	A	0.3005	0.2817	C	0.4771	1.096
3	rs3192149	133368362	A	0.3995	0.4217	C	0.4772	0.9124
11	rs15783	26586801	A	0.3995	0.4217	G	0.4772	0.9124
1	rs116013256	226048680	G	0.01031	0.01667	A	0.4774	0.6146
2	rs61756468	47702290	G	0.01031	0.01667	A	0.4774	0.6146
2	rs6743554	99005601	A	0.3969	0.4175	G	0.4774	0.9182
3	rs72986148	139859628	G	0.01031	0.01667	A	0.4774	0.6146
3	rs148717177	140695332	A	0.01031	0.01667	G	0.4774	0.6146
4	rs117157204	77084391	A	0.01031	0.01667	G	0.4774	0.6146
5	rs17061795	162940999	G	0.01031	0.01667	A	0.4774	0.6146
6	rs7770412	33373341	A	0.01031	0.01667	G	0.4774	0.6146
7	rs201791522	2565976	A	0.01031	0.01667	G	0.4774	0.6146
7	rs72657320	21659626	G	0.01031	0.01667	A	0.4774	0.6146
9	rs11139177	83986875	C	0.2268	0.21	A	0.4774	1.103
9	rs138942689	140441299	A	0.01031	0.01667	G	0.4774	0.6146
10	rs116942055	94397295	A	0.01031	0.01667	T	0.4774	0.6146
10	rs56220353	101578641	A	0.01031	0.01667	G	0.4774	0.6146
10	rs145121731	129900859	A	0.01031	0.01667	G	0.4774	0.6146
12	rs141006210	7047842	A	0.01031	0.01667	G	0.4774	0.6146
12	rs144462453	9098149	G	0.01031	0.01667	A	0.4774	0.6146
16	rs199678770	56870627	C	0.01031	0.01667	A	0.4774	0.6146
18	rs138392588	3102554	A	0.01031	0.01667	G	0.4774	0.6146
18	rs148840672	21914213	A	0.01031	0.01667	G	0.4774	0.6146
19	rs147184532	12089538	G	0.01031	0.01667	A	0.4774	0.6146
19	rs2278420	52471872	G	0.201	0.2192	A	0.4774	0.8964
20	rs147336539	25004229	A	0.01031	0.01667	G	0.4774	0.6146
22	rs34920087	29533572	C	0.01031	0.01667	G	0.4774	0.6146
12	rs12425791	783484	A	0.3015	0.2817	G	0.4775	1.101
14	rs2270420	77786863	A	0.201	0.22	C	0.4775	0.8921
18	rs11267036	12308793	G	0.201	0.22	C	0.4775	0.8921
1	rs1147673	214402313	G	0.4021	0.4242	A	0.4776	0.9128
15	rs8043207	39687127	A	0.4021	0.4242	G	0.4776	0.9128
5	rs347344	83499643	A	0.4021	0.4232	G	0.4777	0.9164
7	rs851821	147242624	G	0.4304	0.41	A	0.4777	1.087
6	rs117770313	137814509	G	0.134	0.12	A	0.4778	1.135

8	rs2439312	32412359	A	0.134	0.12	G	0.4778	1.135
10	rs34750407	129899922	G	0.134	0.12	A	0.4778	1.135
17	rs2586514	33749546	G	0.4021	0.4233	A	0.4778	0.916
19	rs349053	44302624	G	0.134	0.12	A	0.4778	1.135
21	rs7279441	24198815	G	0.134	0.12	A	0.4778	1.135
21	rs1056892	37518706	A	0.4356	0.4142	G	0.4778	1.092
2	rs7426114	152527572	A	0.4046	0.4267	G	0.478	0.9133
6	rs7764091	138584685	C	0.433	0.4125	A	0.478	1.088
8	rs2444306	107524985	G	0.4046	0.4267	A	0.478	0.9133
6	rs343705	80624530	A	0.2294	0.2117	T	0.4781	1.109
6	rs3812153	80626375	G	0.2294	0.2117	A	0.4781	1.109
11	rs11552421	118430519	A	0.4356	0.4149	G	0.4781	1.088
19	rs2547064	9063678	C	0.2294	0.2117	A	0.4781	1.109
19	rs1867691	9065632	G	0.2294	0.2117	A	0.4781	1.109
19	rs2121133	9072313	G	0.2294	0.2117	A	0.4781	1.109
19	rs2591590	9072742	G	0.2294	0.2117	A	0.4781	1.109
19	rs2591593	9075969	A	0.2294	0.2117	G	0.4781	1.109
19	rs2547072	9076083	A	0.2294	0.2117	G	0.4781	1.109
19	rs2591594	9076728	A	0.2294	0.2117	G	0.4781	1.109
19	rs2547076	9076950	A	0.2294	0.2117	C	0.4781	1.109
19	rs1862462	9077436	A	0.2294	0.2117	G	0.4781	1.109
19	rs2591597	9077803	G	0.2294	0.2117	A	0.4781	1.109
11	rs7112925	66826160	A	0.4046	0.4258	G	0.4782	0.9164
13	rs4771450	103969491	A	0.4072	0.4292	G	0.4783	0.9137
1	rs10916415	224360892	A	0.4407	0.4192	G	0.4784	1.092
8	rs6988560	58819650	A	0.4407	0.4192	G	0.4784	1.092
13	rs1239947	51106555	G	0.1005	0.08833	A	0.4784	1.153
17	rs75319568	3422073	A	0.2732	0.293	G	0.4784	0.9071
17	rs3744549	7293715	A	0.2732	0.293	G	0.4784	0.9071
19	rs7255596	58879976	G	0.1005	0.08833	A	0.4784	1.153
21	rs2830840	28665347	G	0.4407	0.4192	C	0.4784	1.092
1	rs4243823	7088855	A	0.4072	0.4283	G	0.4786	0.9168
20	rs2295035	30702144	A	0.3041	0.2842	G	0.4786	1.101
2	rs13397180	50565462	A	0.4098	0.4317	G	0.4787	0.9141
5	rs13354339	46349193	A	0.4098	0.4317	G	0.4787	0.9141
7	rs2108349	50786663	G	0.2732	0.2925	A	0.4787	0.9092
12	rs12146709	30888001	G	0.2732	0.2925	A	0.4787	0.9092
12	rs4587807	33510115	A	0.4098	0.4317	G	0.4787	0.9141
20	rs6127921	55638750	C	0.4098	0.4317	A	0.4787	0.9141
1	rs150612979	6638995	A	0.01031	0.005833	G	0.4788	1.775
1	rs141559019	16332749	A	0.01031	0.005833	G	0.4788	1.775
1	rs57227966	19071356	A	0.01031	0.005833	G	0.4788	1.775
1	rs142427813	33960568	G	0.01031	0.005833	A	0.4788	1.775

1	rs199922486	89734522	G	0.01031	0.005833	A	0.4788	1.775
1	rs114325298	114512582	A	0.01031	0.005833	G	0.4788	1.775
1	rs200351059	156779048	C	0.01031	0.005833	G	0.4788	1.775
1	rs201173574	179063231	A	0.01031	0.005833	T	0.4788	1.775
1	rs56020408	231829851	A	0.01031	0.005833	G	0.4788	1.775
2	rs1715999	50511020	C	0.4433	0.4217	A	0.4788	1.092
2	rs148129550	97427170	G	0.01031	0.005833	A	0.4788	1.775
2	rs33993717	103335441	G	0.01031	0.005833	A	0.4788	1.775
2	rs115347862	231035359	A	0.01031	0.005833	G	0.4788	1.775
3	rs394558	10302172	G	0.01031	0.005833	A	0.4788	1.775
3	rs150329640	42916283	A	0.01031	0.005833	G	0.4788	1.775
3	rs117873755	52420333	A	0.01031	0.005833	G	0.4788	1.775
3	rs143655900	52727217	G	0.01031	0.005833	A	0.4788	1.775
3	rs199894224	137787086	G	0.01031	0.005833	A	0.4788	1.775
3	rs76354796	164404292	G	0.4433	0.4217	A	0.4788	1.092
4	rs79520450	6863251	A	0.01031	0.005833	G	0.4788	1.775
4	rs708547	57821309	G	0.01031	0.005833	A	0.4788	1.775
4	rs184514058	114276891	G	0.01031	0.005833	A	0.4788	1.775
4	rs199773919	159549831	A	0.01031	0.005833	G	0.4788	1.775
5	exm2257403	94230426	G	0.01031	0.005833	A	0.4788	1.775
5	rs140944775	94230426	G	0.01031	0.005833	A	0.4788	1.775
5	rs201027777	140682655	A	0.01031	0.005833	C	0.4788	1.775
5	rs139688732	150660628	A	0.01031	0.005833	C	0.4788	1.775
5	rs145273801	151784206	A	0.01031	0.005833	G	0.4788	1.775
6	rs142679463	13307904	A	0.01031	0.005833	G	0.4788	1.775
6	rs78324544	18121747	A	0.01031	0.005833	G	0.4788	1.775
6	rs200975504	28297360	C	0.01031	0.005833	G	0.4788	1.775
6	rs180943940	39311571	A	0.01031	0.005833	G	0.4788	1.775
6	rs41276060	54173584	A	0.01031	0.005833	G	0.4788	1.775
6	rs9479657	153928396	A	0.01031	0.005833	G	0.4788	1.775
6	rs62432291	159621087	A	0.01031	0.005833	G	0.4788	1.775
7	rs199808587	2749278	A	0.01031	0.005833	G	0.4788	1.775
7	rs146160185	132169649	A	0.01031	0.005833	C	0.4788	1.775
8	rs118031911	55542239	A	0.01031	0.005833	G	0.4788	1.775
8	rs146506752	116599487	A	0.01031	0.005833	C	0.4788	1.775
8	rs145503551	140743395	A	0.01031	0.005833	G	0.4788	1.775
9	rs35066989	34660537	A	0.01031	0.005833	G	0.4788	1.775
10	rs200422852	91487648	G	0.01031	0.005833	A	0.4788	1.775
10	rs144586382	95660784	A	0.01031	0.005833	G	0.4788	1.775
11	rs11024773	18750497	G	0.01031	0.005833	A	0.4788	1.775
11	rs202176179	61533449	A	0.01031	0.005833	G	0.4788	1.775
12	rs139848270	438072	A	0.01031	0.005833	G	0.4788	1.775
12	rs201499879	20774295	G	0.01031	0.005833	A	0.4788	1.775

12	rs140679994	63541427	C	0.01031	0.005833	A	0.4788	1.775
12	rs45502896	88523494	C	0.01031	0.005833	G	0.4788	1.775
12	rs34623199	88589256	A	0.01031	0.005833	G	0.4788	1.775
12	rs1982528	132237848	A	0.01031	0.005833	G	0.4788	1.775
14	rs140610133	21526415	A	0.01031	0.005833	G	0.4788	1.775
15	rs74245641	49048641	G	0.01031	0.005833	A	0.4788	1.775
16	rs149556424	57100453	A	0.01031	0.005833	G	0.4788	1.775
17	rs149170701	6673996	G	0.01031	0.005833	A	0.4788	1.775
17	rs184570389	15341322	A	0.01031	0.005833	G	0.4788	1.775
17	rs138101458	28512323	C	0.01031	0.005833	A	0.4788	1.775
17	rs117679927	72943556	A	0.01031	0.005833	G	0.4788	1.775
19	rs139003158	371213	G	0.01031	0.005833	A	0.4788	1.775
19	rs201122804	15565474	A	0.01031	0.005833	G	0.4788	1.775
19	rs115964828	19309012	A	0.01031	0.005833	G	0.4788	1.775
19	rs13306402	46177353	A	0.01031	0.005833	G	0.4788	1.775
20	rs118095359	23584368	A	0.01031	0.005833	G	0.4788	1.775
20	rs140461905	60892011	A	0.01031	0.005833	G	0.4788	1.775
21	rs200876589	41033345	G	0.01031	0.005833	A	0.4788	1.775
22	rs117137547	32000307	A	0.01031	0.005833	G	0.4788	1.775
5	rs13165073	46162561	A	0.4098	0.4308	G	0.479	0.9173
6	rs2294472	33099210	A	0.1521	0.1692	G	0.479	0.8808
6	rs9377343	96512220	G	0.4098	0.4308	A	0.479	0.9173
7	rs4722933	29612042	A	0.4098	0.4308	G	0.479	0.9173
7	rs183046097	36656027	G	0.01031	0.005843	C	0.479	1.772
10	rs508001	99754973	C	0.1521	0.1692	A	0.479	0.8808
13	rs7984952	31231806	G	0.1521	0.1692	A	0.479	0.8808
13	rs3742302	31233063	A	0.1521	0.1692	G	0.479	0.8808
13	rs7319311	111030578	G	0.4098	0.4308	A	0.479	0.9173
19	rs190013803	1065097	C	0.01031	0.005843	A	0.479	1.772
19	rs9304763	55715319	G	0.4124	0.4342	C	0.479	0.9146
21	rs9981861	41415044	G	0.1521	0.1683	A	0.479	0.886
10	rs2243639	81701722	A	0.2036	0.2217	G	0.4793	0.8977
11	rs10742809	5862532	C	0.4124	0.4333	A	0.4793	0.9177
11	rs4357719	5862653	G	0.4124	0.4333	A	0.4793	0.9177
11	rs4592451	5862845	G	0.4124	0.4333	A	0.4793	0.9177
11	rs4362173	5863013	G	0.4124	0.4333	A	0.4793	0.9177
14	rs8005905	102568367	T	0.2036	0.2217	A	0.4793	0.8977
1	rs2796365	203898362	A	0.4149	0.4367	C	0.4794	0.915
1	rs3748666	222713631	G	0.4149	0.4367	C	0.4794	0.915
4	rs2276932	148984321	G	0.07216	0.0625	A	0.4794	1.167
5	rs7729724	28376355	G	0.07216	0.0625	A	0.4794	1.167
7	rs35499907	23300255	A	0.07216	0.0625	G	0.4794	1.167
7	rs1179640	75238617	G	0.4485	0.4267	A	0.4794	1.093

14	rs7157785	64235556	A	0.07216	0.0625	C	0.4794	1.167
14	rs2286913	75386576	A	0.4485	0.4267	G	0.4794	1.093
16	rs4774	11000848	G	0.2036	0.2225	C	0.4794	0.8934
19	rs2303146	7547074	C	0.4149	0.4367	A	0.4794	0.915
21	rs2834167	34640788	A	0.4485	0.4267	G	0.4794	1.093
8	rs28488529	6794399	A	0.0567	0.06833	G	0.4795	0.8195
11	rs7129085	61913196	A	0.0567	0.06833	C	0.4795	0.8195
12	rs883079	114793240	A	0.4459	0.425	G	0.4795	1.089
12	rs11057353	124265687	A	0.0567	0.06833	G	0.4795	0.8195
15	rs8043515	42568547	G	0.0567	0.06833	C	0.4795	0.8195
2	rs6432854	166810570	A	0.2758	0.2958	C	0.4796	0.9064
6	rs4413654	31441349	A	0.2772	0.2967	G	0.4796	0.9092
15	rs4646626	58256127	A	0.2758	0.2958	G	0.4796	0.9064
1	rs744224	156518379	G	0.451	0.4292	A	0.4797	1.093
5	rs6864131	20499120	C	0.4149	0.4358	A	0.4797	0.9181
17	rs9890664	72937851	G	0.3067	0.2867	A	0.4797	1.101
18	rs9959666	1990397	G	0.451	0.4292	A	0.4797	1.093
8	rs3739327	59496052	G	0.4485	0.4275	A	0.4798	1.089
17	rs2959953	43507008	C	0.4485	0.4275	G	0.4798	1.089
1	rs2276403	158817599	A	0.05155	0.0425	G	0.4799	1.224
2	rs2042831	235857114	A	0.4536	0.4317	G	0.4799	1.093
6	rs7749306	10687746	A	0.05155	0.0425	C	0.4799	1.224
7	rs76794740	133868568	A	0.05155	0.0425	G	0.4799	1.224
8	rs2976190	87235150	A	0.4536	0.4317	G	0.4799	1.093
9	rs10758256	3446277	C	0.2758	0.295	A	0.4799	0.91
10	rs1946391	90042906	G	0.4536	0.4317	A	0.4799	1.093
13	rs77919685	21563311	A	0.05155	0.0425	G	0.4799	1.224
14	rs6571923	39818591	G	0.4536	0.4317	A	0.4799	1.093
17	rs148731719	78350110	A	0.05155	0.0425	G	0.4799	1.224
22	rs139293	39496336	A	0.1134	0.129	C	0.4799	0.8638
1	rs2273779	186273994	A	0.134	0.1208	G	0.48	1.126
5	rs2303138	96350710	A	0.4201	0.4417	G	0.48	0.9158
10	rs11016072	129901570	A	0.134	0.1208	G	0.48	1.126
10	rs7083622	129903094	A	0.134	0.1208	C	0.48	1.126
15	rs4906666	26402601	A	0.451	0.43	G	0.48	1.089
16	rs78108426	11001421	A	0.1134	0.1283	C	0.48	0.8688
16	rs2230433	30518041	C	0.134	0.1208	G	0.48	1.126
5	rs13183818	66780590	G	0.4175	0.4383	A	0.4801	0.9185
17	rs7503373	73498483	G	0.4175	0.4383	A	0.4801	0.9185
5	rs34927	111358740	G	0.4562	0.4342	A	0.4802	1.093
8	rs1390963	28304769	G	0.4562	0.4342	A	0.4802	1.093
10	rs4748713	21048682	A	0.4536	0.4325	G	0.4803	1.089
15	rs12101874	75930199	A	0.4536	0.4325	G	0.4803	1.089

17	rs2873624	3563963	G	0.4227	0.4442	C	0.4803	0.9162
4	rs4339259	190151383	C	0.4201	0.4408	A	0.4804	0.9189
6	rs6913900	103403853	G	0.4201	0.4408	A	0.4804	0.9189
7	rs73128772	63986829	G	0.1134	0.1292	C	0.4804	0.8623
9	rs1805313	116151191	G	0.4201	0.4408	A	0.4804	0.9189
17	rs2886959	69347242	C	0.4529	0.4742	A	0.4804	0.9179
4	rs144811522	71024530	C	0.01036	0.0175	G	0.4805	0.5879
8	rs55642448	10464755	G	0.4562	0.435	A	0.4805	1.09
10	rs9664945	116008497	A	0.0567	0.06917	G	0.4805	0.8089
19	rs736289	33757062	G	0.4562	0.435	A	0.4805	1.09
19	rs2043211	48737706	T	0.4562	0.435	A	0.4805	1.09
1	rs264011	45460832	G	0.4588	0.4375	A	0.4807	1.09
8	rs2223059	117129410	A	0.4278	0.45	G	0.4807	0.9139
18	rs985492	29311034	G	0.4588	0.4375	A	0.4807	1.09
1	rs180784616	154067577	G	0.01031	0.0175	A	0.4808	0.5848
3	rs147559897	119211265	T	0.01031	0.0175	A	0.4808	0.5848
3	rs79087781	133191385	A	0.01031	0.0175	C	0.4808	0.5848
4	rs76702927	139104414	G	0.01031	0.0175	A	0.4808	0.5848
6	rs150142878	24847885	A	0.01031	0.0175	G	0.4808	0.5848
6	rs12214934	32836500	C	0.01031	0.0175	G	0.4808	0.5848
6	rs11969522	32859825	C	0.01031	0.0175	G	0.4808	0.5848
6	rs194679	32891338	A	0.01031	0.0175	G	0.4808	0.5848
6	rs2895618	106266373	A	0.4227	0.4433	G	0.4808	0.9193
7	rs117543061	33427727	A	0.01031	0.0175	G	0.4808	0.5848
7	rs138102615	40234653	C	0.01031	0.0175	A	0.4808	0.5848
10	rs12267385	46122199	A	0.01031	0.0175	G	0.4808	0.5848
10	rs142532761	127456130	G	0.01031	0.0175	A	0.4808	0.5848
11	rs192093275	11924337	G	0.01031	0.0175	A	0.4808	0.5848
11	rs3816605	47857253	G	0.2784	0.2983	A	0.4808	0.9072
14	rs200554860	20388973	A	0.01031	0.0175	C	0.4808	0.5848
17	rs144171786	43246901	G	0.01031	0.0175	A	0.4808	0.5848
19	rs45591734	52618506	A	0.01031	0.0175	G	0.4808	0.5848
19	rs192117499	56370051	A	0.01031	0.0175	G	0.4808	0.5848
20	rs6035292	19291116	G	0.2784	0.2983	A	0.4808	0.9072
1	rs1410996	196696933	A	0.4613	0.44	G	0.4809	1.09
4	rs2167494	39216221	A	0.1778	0.1625	G	0.4809	1.115
6	rs3893464	29935250	G	0.4613	0.44	A	0.4809	1.09
13	rs17646069	42803263	G	0.1778	0.1625	A	0.4809	1.115
15	rs306197	85401259	G	0.1778	0.1625	A	0.4809	1.115
1	rs1812242	12835868	A	0.232	0.215	G	0.481	1.103
16	rs4785874	65928770	G	0.232	0.215	A	0.481	1.103
19	rs953551	9076278	A	0.232	0.215	G	0.481	1.103
1	rs1689800	182168885	G	0.2784	0.2975	A	0.4811	0.9108

20	rs2207800	38149274	G	0.4253	0.4458	A	0.4811	0.9197
6	rs404860	32184345	G	0.4304	0.4517	A	0.4812	0.9174
6	rs495335	49701523	A	0.4304	0.4517	G	0.4812	0.9174
10	rs11017252	132231596	A	0.4588	0.4382	C	0.4812	1.087
19	rs78580402	900951	G	0.2062	0.225	A	0.4812	0.8947
2	rs13027171	112686988	A	0.1005	0.08917	G	0.4814	1.141
6	rs12000	28227436	G	0.4665	0.445	A	0.4814	1.091
6	rs2534814	30480877	G	0.3093	0.29	C	0.4814	1.096
6	rs2524226	30497490	A	0.3093	0.29	G	0.4814	1.096
6	rs1058318	30512163	A	0.3093	0.29	G	0.4814	1.096
10	rs1200876	37505141	A	0.1005	0.08917	G	0.4814	1.141
10	rs1227756	71588504	A	0.3093	0.29	G	0.4814	1.096
16	rs1127390	81010073	A	0.4665	0.445	G	0.4814	1.091
19	rs7246376	8203328	A	0.4665	0.445	G	0.4814	1.091
20	rs1555322	33849179	A	0.1005	0.08917	G	0.4814	1.141
4	rs437943	35372098	G	0.433	0.4542	A	0.4815	0.9178
6	rs2499486	51873092	A	0.4356	0.4575	G	0.4815	0.9151
20	rs4911163	33470694	A	0.433	0.4542	G	0.4815	0.9178
20	rs6120757	33488771	A	0.433	0.4542	G	0.4815	0.9178
2	rs1000053	12790328	A	0.1366	0.1225	G	0.4816	1.133
14	rs1051860	58838668	G	0.4691	0.4475	A	0.4816	1.091
16	rs7203944	54438254	G	0.4691	0.4475	A	0.4816	1.091
1	rs1479640	163948373	C	0.4381	0.46	A	0.4817	0.9154
4	rs7667706	96597286	A	0.4381	0.46	G	0.4817	0.9154
5	rs1992906	173182811	A	0.1546	0.1708	G	0.4817	0.8879
7	rs10260011	84709356	G	0.4356	0.4567	A	0.4817	0.9181
9	rs10811345	20327360	A	0.2345	0.2167	C	0.4817	1.108
9	rs34788368	72131577	C	0.2345	0.2167	A	0.4817	1.108
13	rs3742318	33017043	G	0.1546	0.1708	A	0.4817	0.8879
13	rs10492681	40807483	C	0.4356	0.4567	G	0.4817	0.9181
14	rs8005962	96027153	A	0.2345	0.2167	G	0.4817	1.108
15	rs8028182	75718669	A	0.2345	0.2167	C	0.4817	1.108
15	rs8041080	100843884	A	0.4508	0.4725	G	0.4817	0.9163
20	rs4812641	35590249	C	0.4381	0.46	A	0.4817	0.9154
1	rs3219145	226550829	G	0.03368	0.02667	A	0.4818	1.272
1	rs2020863	171174531	G	0.2809	0.3008	A	0.4819	0.908
1	rs4844649	208337392	G	0.4407	0.4625	A	0.4819	0.9158
3	rs3736563	33458266	A	0.4742	0.4525	G	0.4819	1.091
3	rs6765153	33461796	A	0.4742	0.4525	C	0.4819	1.091
6	rs10946398	20661034	C	0.4742	0.4525	A	0.4819	1.091
12	rs10859974	96288860	G	0.4665	0.4458	A	0.4819	1.087
14	rs7140150	52010799	G	0.4742	0.4525	A	0.4819	1.091
14	rs2236267	88632321	A	0.2809	0.3017	G	0.4819	0.9044

17	rs712039	35850553	G	0.4742	0.4525	A	0.4819	1.091
4	rs4642195	164200171	A	0.4691	0.4483	G	0.482	1.087
9	rs1463984	108427062	A	0.4381	0.4592	G	0.482	0.9185
1	rs10920566	190088325	G	0.4768	0.455	A	0.4821	1.092
5	rs4704566	78889624	G	0.4433	0.465	A	0.4821	0.9162
19	rs867168	2833802	G	0.4768	0.455	A	0.4821	1.092
19	rs867169	2833864	A	0.4768	0.455	G	0.4821	1.092
1	rs7524853	116716281	A	0.4407	0.4617	G	0.4822	0.9189
5	rs2247114	475104	G	0.4716	0.4508	A	0.4822	1.087
5	rs57272473	178381722	A	0.4716	0.4508	G	0.4822	1.087
6	rs1233388	29553727	G	0.4716	0.4508	A	0.4822	1.087
7	rs4724951	7187522	G	0.4716	0.4508	A	0.4822	1.087
1	rs532905	189620278	G	0.4637	0.4858	A	0.4823	0.9152
2	rs6740023	129654217	A	0.4459	0.4675	G	0.4823	0.9165
2	rs12694624	224837600	G	0.4459	0.4675	A	0.4823	0.9165
3	rs9311014	33468329	C	0.4742	0.4533	A	0.4823	1.088
3	rs3796295	171087469	A	0.4459	0.4675	G	0.4823	0.9165
4	rs9307576	126654773	C	0.4794	0.4575	A	0.4823	1.092
15	rs1992730	102042282	A	0.4794	0.4575	G	0.4823	1.092
6	rs9272105	32599999	G	0.482	0.46	A	0.4824	1.092
22	rs5747211	17990852	A	0.4433	0.4642	G	0.4825	0.9192
10	rs4919060	98699136	C	0.482	0.4608	A	0.4826	1.089
1	rs11265165	159283746	G	0.4871	0.465	A	0.4827	1.093
7	rs2252521	29041190	A	0.4845	0.4633	G	0.4828	1.089
8	rs919951	103553703	G	0.4845	0.4633	A	0.4828	1.089
8	rs279661	122805639	A	0.4845	0.4633	G	0.4828	1.089
9	rs942519	117169033	A	0.4536	0.475	G	0.4828	0.9176
11	rs1144507	123600475	A	0.4845	0.4633	G	0.4828	1.089
15	rs1442801	98768182	G	0.4562	0.4783	A	0.4828	0.9149
1	rs1233817	198055493	G	0.4485	0.4692	A	0.4829	0.92
5	rs8188245	46174864	A	0.4871	0.4658	G	0.4829	1.089
13	rs1161463	29431338	G	0.4485	0.4692	A	0.4829	0.92
13	rs7982911	45321621	G	0.4485	0.4692	A	0.4829	0.92
18	rs7236632	55434202	G	0.4485	0.4692	A	0.4829	0.92
2	rs1465618	43553949	G	0.2835	0.3042	A	0.483	0.9052
5	rs79988174	125696118	G	0.07474	0.06417	C	0.483	1.178
6	rs4351302	31176921	A	0.07474	0.06417	G	0.483	1.178
10	rs284860	104572963	G	0.4562	0.4775	A	0.483	0.9179
11	rs10832452	15518925	A	0.4897	0.4683	G	0.483	1.089
13	rs2585897	21398979	G	0.2088	0.2275	A	0.483	0.8959
14	rs2149830	34124784	A	0.4562	0.4775	G	0.483	0.9179
14	exm1134221	105518336	A	0.2835	0.3042	C	0.483	0.9052
14	rs3803322	105518336	A	0.2835	0.3042	C	0.483	0.9052

15	rs3803430	101445815	G	0.07474	0.06417	A	0.483	1.178
17	rs2066964	78171944	C	0.4562	0.4775	G	0.483	0.9179
1	rs6669563	32279629	A	0.451	0.4717	G	0.4831	0.9203
1	rs1857353	75913826	A	0.03351	0.02667	G	0.4831	1.265
2	rs13012765	101498174	G	0.2088	0.2267	A	0.4831	0.9002
4	rs7665090	103551603	G	0.4923	0.4708	A	0.4831	1.09
5	rs10057851	64565261	A	0.4948	0.4733	G	0.4831	1.09
5	rs16877135	79028586	G	0.4639	0.4858	A	0.4831	0.9159
5	rs79381504	169310184	A	0.03351	0.02667	G	0.4831	1.265
6	rs146736135	46726464	C	0.03351	0.02667	G	0.4831	1.265
9	rs144862865	15874593	A	0.03351	0.02667	G	0.4831	1.265
9	rs6478109	117568766	A	0.4948	0.4733	G	0.4831	1.09
10	rs2949369	126719704	G	0.4948	0.4733	A	0.4831	1.09
11	rs141809322	14284477	A	0.03351	0.02667	G	0.4831	1.265
17	rs61744697	11672607	A	0.03351	0.02667	C	0.4831	1.265
19	rs6510130	58772579	G	0.03351	0.02667	C	0.4831	1.265
20	rs78807201	23424683	G	0.03351	0.02667	A	0.4831	1.265
20	rs2230782	46264888	G	0.03351	0.02667	C	0.4831	1.265
21	rs2830051	27465355	G	0.451	0.4717	A	0.4831	0.9203
2	rs6433294	172369069	G	0.4665	0.4883	A	0.4832	0.9162
3	rs7374394	184945849	A	0.4665	0.4883	G	0.4832	0.9162
8	rs59606339	59571856	A	0.4974	0.4758	C	0.4832	1.09
12	rs11175883	66112855	G	0.4665	0.4883	A	0.4832	0.9162
5	rs16877109	79024779	A	0.4639	0.485	G	0.4833	0.919
5	rs1366271	79025634	G	0.4639	0.485	A	0.4833	0.919
5	rs62621915	79027700	A	0.4639	0.485	G	0.4833	0.919
5	rs16877133	79028513	A	0.4639	0.485	G	0.4833	0.919
5	rs16877150	79030338	A	0.4639	0.485	G	0.4833	0.919
5	rs27044	96118852	G	0.4613	0.4825	C	0.4833	0.9186
5	rs702098	171274885	A	0.4923	0.4716	C	0.4833	1.086
6	rs9295323	166570346	G	0.4613	0.4825	A	0.4833	0.9186
13	rs9572094	35252882	A	0.4613	0.4825	G	0.4833	0.9186
14	rs11160805	104866687	G	0.5026	0.4808	A	0.4833	1.091
1	rs10797449	233490138	G	0.5155	0.4933	A	0.4834	1.093
4	rs7670069	149063523	G	0.3144	0.295	A	0.4834	1.096
5	rs57544556	79026539	A	0.4639	0.485	C	0.4834	0.9189
5	rs6893869	79027605	A	0.4639	0.485	G	0.4834	0.9189
5	rs1428223	79029288	C	0.4639	0.485	A	0.4834	0.9189
5	rs1428224	79029383	A	0.4639	0.485	C	0.4834	0.9189
5	rs16877141	79029726	A	0.4639	0.485	T	0.4834	0.9189
5	rs1428225	79029749	A	0.4639	0.485	G	0.4834	0.9189
5	rs16877147	79030212	G	0.4639	0.485	A	0.4834	0.9189
5	rs16877151	79030346	A	0.4639	0.485	G	0.4834	0.9189

12	rs2700152	54767443	G	0.2371	0.2192	A	0.4834	1.107
18	rs928980	908777	A	0.5155	0.4933	G	0.4834	1.093
7	rs35940771	127430974	A	0.1804	0.165	C	0.4835	1.114
8	rs2255317	99044528	A	0.1804	0.165	G	0.4835	1.114
10	rs6585236	84944836	A	0.4768	0.4983	G	0.4835	0.9174
12	rs11055450	13603787	A	0.1804	0.165	G	0.4835	1.114
14	rs8017423	90679910	G	0.4897	0.4692	A	0.4835	1.086
16	rs8045933	73140753	G	0.4742	0.4958	A	0.4835	0.9171
5	rs2290595	54603822	G	0.4923	0.4717	A	0.4836	1.086
5	rs10062026	90052289	G	0.4948	0.4742	A	0.4836	1.086
5	rs2961944	159835658	G	0.5155	0.4942	A	0.4836	1.089
6	rs9406183	8639771	C	0.4794	0.5008	A	0.4836	0.9177
6	rs506268	149685617	G	0.4923	0.4717	A	0.4836	1.086
7	rs4723793	38845861	G	0.4794	0.5008	A	0.4836	0.9177
9	rs3780548	99525487	G	0.1031	0.09083	A	0.4836	1.15
9	rs34787999	108366734	A	0.1031	0.09083	G	0.4836	1.15
9	rs4263839	117566440	A	0.4948	0.4742	G	0.4836	1.086
10	rs10795415	16718607	G	0.5155	0.4942	A	0.4836	1.089
10	rs11001819	78315224	A	0.1366	0.1233	G	0.4836	1.125
10	rs2946994	126714714	G	0.4948	0.4742	C	0.4836	1.086
10	rs3012075	126714966	A	0.4948	0.4742	G	0.4836	1.086
10	rs869801	129245684	A	0.1366	0.1233	G	0.4836	1.125
18	rs10460009	2948029	G	0.5155	0.4942	A	0.4836	1.089
18	rs3737353	5956238	G	0.5155	0.4942	A	0.4836	1.089
18	rs1058396	43319519	G	0.4948	0.4742	A	0.4836	1.086
22	rs4820268	37469591	G	0.4794	0.5008	A	0.4836	0.9177
22	rs2413450	37470224	A	0.4794	0.5008	G	0.4836	0.9177
1	rs11240702	204198882	A	0.5129	0.4917	C	0.4837	1.089
1	rs10900562	204199614	A	0.5129	0.4917	G	0.4837	1.089
2	rs10177248	69049941	A	0.4974	0.4767	G	0.4837	1.087
3	rs2293377	191114266	G	0.5077	0.4867	A	0.4837	1.088
6	rs9353806	65149185	T	0.4974	0.4767	A	0.4837	1.087
7	rs34639489	64452830	A	0.5052	0.4842	G	0.4837	1.088
8	rs7005417	89917982	G	0.5026	0.4817	A	0.4837	1.087
9	rs11145017	79324955	A	0.5077	0.4867	G	0.4837	1.088
10	rs34970111	106078937	G	0.5129	0.4917	A	0.4837	1.089
1	rs2076901	115658497	G	0.4716	0.4925	A	0.4838	0.9199
2	rs72650066	179515483	C	0.116	0.1308	G	0.4838	0.8716
4	rs10001580	37851856	C	0.116	0.1308	A	0.4838	0.8716
15	rs2301831	91017718	A	0.116	0.1308	G	0.4838	0.8716
16	rs881803	15802334	G	0.4742	0.495	A	0.4839	0.9202
5	rs2303064	147480080	A	0.4768	0.4975	G	0.484	0.9205
5	rs299290	162902516	G	0.482	0.5025	A	0.484	0.9211

6	rs3129207	33125312	C	0.4768	0.4975	G	0.484	0.9205
12	rs4631964	84369765	C	0.4768	0.4975	A	0.484	0.9205
2	rs300774	112496	A	0.116	0.1317	C	0.4841	0.8652
6	rs9383750	155608455	G	0.2861	0.3058	A	0.4841	0.9095
10	rs2075641	75562108	A	0.116	0.1317	G	0.4841	0.8652
14	rs1956467	30691706	A	0.4794	0.5	G	0.4841	0.9208
17	rs62065084	63822357	G	0.116	0.1317	A	0.4841	0.8652
8	rs7017199	58065790	G	0.1572	0.1742	A	0.4843	0.8845
11	rs11217785	120152591	G	0.1572	0.1742	A	0.4843	0.8845
12	rs10744557	332860	A	0.3144	0.2958	G	0.4843	1.092
2	rs2277912	207634236	C	0.1572	0.1733	A	0.4844	0.8897
6	rs6901541	32442261	G	0.2371	0.22	A	0.4844	1.102
12	rs2403106	84008275	G	0.1572	0.1733	A	0.4844	0.8897
22	rs2236005	26422980	G	0.2371	0.22	A	0.4844	1.102
1	rs2273872	234573025	G	0.183	0.1667	A	0.4848	1.12
1	rs1339847	248039294	A	0.183	0.1667	G	0.4848	1.12
6	rs2306914	152640110	A	0.183	0.1667	G	0.4848	1.12
1	rs2229712	26883511	C	0.2397	0.2217	A	0.4851	1.107
5	rs55900671	14508347	T	0.05155	0.04333	A	0.4851	1.2
7	rs17661364	48315360	G	0.05155	0.04333	A	0.4851	1.2
11	rs2304935	60102384	G	0.2887	0.3092	A	0.4851	0.9068
11	rs2304934	60102396	G	0.2887	0.3092	A	0.4851	0.9068
11	rs2304933	60102507	A	0.2887	0.3092	C	0.4851	0.9068
17	rs7224888	47246163	G	0.05155	0.04333	A	0.4851	1.2
20	rs6142471	34572606	G	0.05155	0.04333	A	0.4851	1.2
1	rs641712	86488232	A	0.1392	0.125	G	0.4852	1.132
5	rs57195665	140803241	A	0.1392	0.125	C	0.4852	1.132
7	rs37423	15788241	G	0.2887	0.3083	A	0.4852	0.9103
19	rs59102460	15233732	A	0.1392	0.125	G	0.4852	1.132
5	rs4921291	159920295	G	0.183	0.1675	A	0.486	1.113
6	rs6914079	14596365	A	0.2397	0.2225	G	0.486	1.102
3	rs13433693	167062902	A	0.2912	0.3117	G	0.4861	0.9075
10	rs1249005	29031485	A	0.2912	0.3117	C	0.4861	0.9075
12	rs2072579	108954862	G	0.2912	0.3117	C	0.4861	0.9075
5	rs6883840	40286410	A	0.2912	0.3108	G	0.4862	0.9111
6	rs2076173	35442397	G	0.3196	0.3008	A	0.4862	1.092
6	rs41265385	170068086	A	0.2912	0.3108	G	0.4862	0.9111
14	rs4243553	48801744	A	0.3196	0.3008	G	0.4862	1.092
5	rs1974777	154291409	G	0.1031	0.09167	A	0.4864	1.139
7	rs13242977	62140786	A	0.3222	0.3025	G	0.4864	1.096
14	rs17646495	86089508	A	0.08505	0.09833	C	0.4864	0.8524
14	rs78711719	48143852	A	0.2139	0.2325	G	0.4865	0.8983
3	rs6445975	58370177	C	0.2139	0.2317	A	0.4867	0.9025

5	rs2230520	66480374	G	0.2139	0.2317	C	0.4867	0.9025
6	rs2757594	7590624	A	0.07474	0.065	G	0.4868	1.162
6	rs2076528	32364194	C	0.08549	0.09933	A	0.4868	0.8476
6	rs3763315	32376654	A	0.08549	0.09933	C	0.4868	0.8476
8	rs6471165	134901186	A	0.07474	0.065	G	0.4868	1.162
11	rs3741005	86123556	G	0.07474	0.065	A	0.4868	1.162
3	rs4685171	14555216	A	0.1598	0.1767	G	0.4869	0.8863
5	rs778821	152972201	A	0.1598	0.1767	G	0.4869	0.8863
10	rs10823148	69926334	G	0.1598	0.1767	C	0.4869	0.8863
19	rs919364	49867913	A	0.1598	0.1767	G	0.4869	0.8863
1	rs4307514	54060248	G	0.1392	0.1258	A	0.4871	1.123
2	rs2163204	21231387	C	0.08505	0.09917	A	0.4871	0.8444
3	rs4613440	14555811	A	0.1606	0.1767	G	0.4871	0.8918
5	rs17097231	140739779	G	0.1392	0.1258	C	0.4871	1.123
6	rs6925151	150210723	A	0.3222	0.3033	G	0.4871	1.092
6	rs3798763	150211100	G	0.3222	0.3033	A	0.4871	1.092
12	rs79443892	71533622	A	0.1598	0.1758	G	0.4871	0.8914
14	rs1890908	64519035	A	0.08505	0.09917	G	0.4871	0.8444
3	rs2872603	183091561	A	0.1856	0.1692	G	0.4873	1.119
7	rs2272193	115890510	A	0.05928	0.07083	G	0.4873	0.8266
11	rs1130290	43940602	C	0.05928	0.07083	G	0.4873	0.8266
18	rs2298645	77728135	C	0.05928	0.07083	A	0.4873	0.8266
19	rs2291473	10415781	G	0.1856	0.1692	A	0.4873	1.119
21	rs2824790	19756040	G	0.05928	0.07083	C	0.4873	0.8266
1	rs75423398	120471712	A	0.03866	0.04833	G	0.4875	0.7918
1	rs150412216	248569960	A	0.03866	0.04833	G	0.4875	0.7918
3	rs6776293	154405414	A	0.03866	0.04833	G	0.4875	0.7918
6	rs6930052	32990481	T	0.03866	0.04833	A	0.4875	0.7918
6	rs2279381	123101544	A	0.03866	0.04833	G	0.4875	0.7918
8	rs1562430	128387852	G	0.1186	0.1333	A	0.4875	0.8743
10	rs4423101	3008619	G	0.03866	0.04833	A	0.4875	0.7918
19	rs8105198	48543862	G	0.1186	0.1333	A	0.4875	0.8743
20	rs6024905	36957837	A	0.1186	0.1333	G	0.4875	0.8743
22	rs7287672	17640045	A	0.03866	0.04833	G	0.4875	0.7918
22	rs5905	21133619	A	0.03866	0.04833	G	0.4875	0.7918
5	rs30386	179290845	A	0.2423	0.225	C	0.4877	1.101
5	rs10942901	79025157	G	0.1186	0.1342	A	0.4878	0.868
6	rs10223421	31390055	A	0.2979	0.3183	C	0.4879	0.9087
2	rs11695248	220402680	A	0.2964	0.3164	G	0.488	0.9103
3	rs56047676	124418879	G	0.05928	0.07167	A	0.4882	0.8162
4	rs35765784	114376933	A	0.05928	0.07167	G	0.4882	0.8162
11	rs325687	6179462	G	0.05928	0.07167	A	0.4882	0.8162
15	rs1834640	48392165	A	0.05928	0.07167	G	0.4882	0.8162

2	rs11901612	19521472	G	0.3273	0.3075	A	0.4883	1.096
3	rs1378796	157131788	C	0.2964	0.3158	A	0.4883	0.9125
2	rs918959	181513729	A	0.2165	0.2342	G	0.4884	0.9037
2	rs17838596	196877557	G	0.03866	0.04917	A	0.4884	0.7777
2	rs73987681	196912179	C	0.03866	0.04917	G	0.4884	0.7777
2	rs80292002	238419321	G	0.03866	0.04917	A	0.4884	0.7777
5	rs183643295	176831083	G	0.03866	0.04917	C	0.4884	0.7777
7	rs61696422	50132717	C	0.03866	0.04917	G	0.4884	0.7777
9	rs55888789	139849843	A	0.03866	0.04917	G	0.4884	0.7777
20	rs16987188	36856616	A	0.03866	0.04917	G	0.4884	0.7777
15	rs11858113	40914177	G	0.2448	0.2267	A	0.4885	1.106
11	rs1453544	59224608	G	0.1418	0.1275	A	0.4888	1.13
14	rs201825906	20216010	G	0.02073	0.015	A	0.4888	1.39
7	rs2159688	31756362	A	0.3273	0.3083	G	0.4889	1.092
1	rs1325266	67160602	A	0.299	0.3192	G	0.4891	0.9097
3	rs2239752	50645413	A	0.299	0.3192	G	0.4891	0.9097
1	rs10920333	202081212	C	0.299	0.3183	A	0.4893	0.9132
8	rs12541381	135649848	A	0.3299	0.31	G	0.4893	1.096
22	rs9610342	36130577	G	0.3299	0.31	A	0.4893	1.096
15	rs1288775	45661678	A	0.1624	0.1792	T	0.4894	0.8881
4	rs3733526	120528327	G	0.1073	0.095	A	0.4896	1.145
1	rs1869780	22222455	A	0.02062	0.015	C	0.4897	1.382
2	rs200498323	242007212	G	0.02062	0.015	A	0.4897	1.382
4	rs59932476	3491504	A	0.02062	0.015	G	0.4897	1.382
5	rs2303018	150441738	A	0.02062	0.015	G	0.4897	1.382
6	rs184385862	17834269	A	0.02062	0.015	G	0.4897	1.382
6	rs117796773	52103527	A	0.02062	0.015	C	0.4897	1.382
12	rs201387635	124103253	A	0.02062	0.015	G	0.4897	1.382
15	rs80028569	72143643	A	0.02062	0.015	T	0.4897	1.382
16	rs181629545	31519547	A	0.02062	0.015	G	0.4897	1.382
16	rs247616	56989590	A	0.1624	0.1783	G	0.4897	0.8931
19	rs74351326	9524997	A	0.02062	0.015	G	0.4897	1.382
20	rs147312767	9440331	G	0.02062	0.015	A	0.4897	1.382
20	rs141204447	37394884	A	0.02062	0.015	G	0.4897	1.382
4	rs4129090	84941806	A	0.3299	0.3108	G	0.4898	1.092
5	rs11249661	179277874	G	0.1881	0.1717	A	0.4898	1.118
17	rs4790200	4424159	A	0.3299	0.3108	C	0.4898	1.092
13	rs588890	63967568	C	0.2191	0.2375	A	0.4899	0.9006
1	rs1800871	206946634	G	0.3015	0.3217	A	0.49	0.9104
9	rs10974657	4622453	G	0.3015	0.3217	A	0.49	0.9104
11	rs7947780	82643760	A	0.3015	0.3217	C	0.49	0.9104
16	rs316371	9735857	A	0.3325	0.3125	G	0.4902	1.096
19	rs4804063	8176945	G	0.3325	0.3125	A	0.4902	1.096

3	rs2228389	81627175	G	0.07732	0.06667	A	0.4904	1.173
12	rs11047443	8976780	G	0.07732	0.06667	A	0.4904	1.173
4	rs10517195	47682174	G	0.1418	0.1283	A	0.4906	1.122
11	rs10895986	64902002	A	0.1418	0.1283	C	0.4906	1.122
19	rs2562456	21666210	G	0.3325	0.3133	A	0.4907	1.092
2	rs10192053	7090819	C	0.2474	0.23	A	0.4909	1.101
6	rs9376090	135411228	G	0.3351	0.315	A	0.4911	1.096
1	rs140076587	15428057	A	0.0232	0.03083	C	0.4912	0.7464
6	rs16879902	17688693	A	0.05412	0.045	G	0.4912	1.214
6	rs1799945	26091179	G	0.05412	0.045	C	0.4912	1.214
7	rs59861703	100383750	G	0.05412	0.045	A	0.4912	1.214
9	rs10961700	14776140	A	0.05412	0.045	G	0.4912	1.214
10	rs117778687	99139505	A	0.0232	0.03083	T	0.4912	0.7464
15	rs2280217	68489810	A	0.0232	0.03083	C	0.4912	0.7464
6	rs9968	29501037	C	0.3041	0.3233	G	0.4913	0.9146
6	rs58508214	30997767	A	0.0232	0.03167	T	0.4913	0.7261
6	rs9471966	42891022	A	0.0232	0.03167	G	0.4913	0.7261
6	rs16874954	46677098	A	0.1211	0.1367	C	0.4913	0.8707
13	rs9551080	24777773	A	0.3041	0.3233	G	0.4913	0.9146
14	rs10138395	55819219	A	0.1211	0.1367	C	0.4913	0.8707
14	rs74810099	62229285	C	0.0232	0.03167	A	0.4913	0.7261
14	rs77756572	70530566	A	0.0232	0.03167	G	0.4913	0.7261
15	rs7169431	56340896	A	0.1057	0.09417	G	0.4913	1.137
15	exm1184899	85401419	A	0.0232	0.03167	G	0.4913	0.7261
15	rs167379	85401419	A	0.02332	0.03172	G	0.4913	0.7287
16	rs149645127	72832285	G	0.0232	0.03167	A	0.4913	0.7261
20	rs79984831	30818637	G	0.0232	0.03167	A	0.4913	0.7261
1	rs2504779	27709020	G	0.3351	0.3158	A	0.4916	1.092
11	rs665278	77825320	G	0.08763	0.1008	A	0.4916	0.8565
17	rs1885987	2203025	C	0.1649	0.1814	A	0.4917	0.8912
5	rs9327603	130558963	C	0.2216	0.2392	A	0.4919	0.9059
8	rs34995506	12879198	A	0.2216	0.2392	T	0.4919	0.9059
14	rs1885137	94912896	C	0.1649	0.1817	A	0.4919	0.8898
8	rs7011934	98059046	C	0.08763	0.1017	A	0.4921	0.8487
1	rs1289658	117656088	G	0.1907	0.1742	A	0.4922	1.117
14	rs3829767	64519455	A	0.1649	0.1808	G	0.4922	0.8948
14	rs8010699	64522843	A	0.1649	0.1808	G	0.4922	0.8948
4	rs11726779	122703201	G	0.3067	0.3258	A	0.4923	0.9153
22	rs139302	39498038	C	0.3067	0.3258	G	0.4923	0.9153
6	rs1058026	31321685	C	0.25	0.2325	A	0.4924	1.1
12	rs10861661	107174646	C	0.3376	0.3183	A	0.4924	1.092
20	rs3746803	744382	A	0.1082	0.09532	G	0.4924	1.152
15	rs1801376	40477831	A	0.3093	0.3292	G	0.4928	0.9125

15	rs12898397	75130093	G	0.3402	0.32	A	0.4928	1.096
15	rs8030485	79408932	A	0.3402	0.32	G	0.4928	1.096
1	exm162297	236925844	A	0.1907	0.175	G	0.4931	1.111
1	rs12063382	236925844	A	0.1907	0.175	G	0.4931	1.111
6	rs2076472	41029342	G	0.2242	0.2425	A	0.4932	0.9029
21	rs3819142	43557698	C	0.2242	0.2425	A	0.4932	0.9029
1	rs5876	100547994	A	0.3402	0.3208	G	0.4933	1.092
1	rs1890645	195531423	G	0.3402	0.3208	A	0.4933	1.092
6	rs3117299	29653957	C	0.3402	0.3208	A	0.4933	1.092
1	rs2791552	219652033	A	0.2242	0.2417	C	0.4936	0.907
9	rs4977826	23127562	A	0.1082	0.09583	G	0.4936	1.145
21	rs234720	44445292	A	0.2242	0.2417	G	0.4936	0.907
4	rs10516541	108115222	A	0.3119	0.3317	G	0.4937	0.9132
5	rs3822568	78670802	A	0.3119	0.3325	G	0.4937	0.9098
1	rs3790549	119575818	G	0.1443	0.1308	C	0.4939	1.121
3	rs10470320	125983396	C	0.2526	0.235	A	0.4939	1.1
6	rs3135365	32389255	C	0.1082	0.09599	A	0.4939	1.143
6	rs61751042	167435928	A	0.07732	0.0675	G	0.4939	1.158
6	rs17856382	167438276	A	0.07732	0.0675	C	0.4939	1.158
15	rs3924426	92445873	G	0.07732	0.0675	A	0.4939	1.158
17	rs769427	3119767	A	0.1443	0.1308	G	0.4939	1.121
17	rs78260319	7366348	C	0.1443	0.1308	A	0.4939	1.121
6	rs3094724	29674295	G	0.3402	0.3217	A	0.4941	1.087
9	rs1407856	7046901	C	0.1675	0.1842	G	0.4943	0.8915
11	rs3741138	73620537	G	0.1675	0.1848	C	0.4943	0.8878
13	rs9574565	80668874	A	0.1675	0.1842	G	0.4943	0.8915
5	rs12515587	141248963	C	0.03608	0.02833	A	0.4945	1.284
13	rs117237504	29598842	A	0.03608	0.02833	G	0.4945	1.284
19	rs117324081	49344428	A	0.03608	0.02833	G	0.4945	1.284
20	rs74949991	37650519	A	0.03608	0.02833	G	0.4945	1.284
1	rs2229579	24201162	A	0.1907	0.1758	G	0.4946	1.105
4	rs17625943	177098285	A	0.3144	0.335	G	0.4946	0.9104
17	rs8080100	65212042	A	0.1675	0.185	G	0.4946	0.8865
2	rs3184781	85549527	A	0.1237	0.1383	G	0.4947	0.8794
2	rs75091099	102851470	A	0.06186	0.07333	G	0.4947	0.8332
9	rs3852401	97333275	G	0.06186	0.07333	A	0.4947	0.8332
2	rs2300478	66781453	C	0.2268	0.245	A	0.4948	0.9039
2	rs61750041	182468803	C	0.2268	0.245	A	0.4948	0.9039
3	rs9883988	130162395	A	0.1237	0.1392	G	0.4948	0.8733
20	rs2227275	43926573	A	0.1237	0.1392	G	0.4948	0.8733
6	rs2213568	32711576	A	0.3428	0.3242	C	0.4949	1.087
6	rs2213567	32711655	C	0.3428	0.3242	G	0.4949	1.087
10	rs10887650	88461699	A	0.3454	0.3258	G	0.4949	1.092

2	rs1473636	212882190	A	0.06186	0.07417	G	0.4954	0.8231
5	rs12153513	136294407	A	0.2552	0.2375	G	0.4954	1.1
6	rs3095318	31088145	A	0.06186	0.07417	T	0.4954	0.8231
7	rs7800072	84628989	C	0.2552	0.2375	A	0.4954	1.1
13	rs1021188	43116133	G	0.317	0.3375	A	0.4954	0.9111
15	rs16959028	34648635	A	0.03608	0.02852	G	0.4954	1.275
20	rs6045676	1941171	C	0.2552	0.2375	G	0.4954	1.1
4	rs16840892	7716061	A	0.317	0.3361	G	0.4956	0.9168
7	rs801840	88964196	A	0.3454	0.3267	T	0.4956	1.087
9	rs10781380	79408144	G	0.3454	0.3267	A	0.4956	1.087
12	rs1520832	43253415	A	0.1469	0.1325	C	0.4956	1.127
6	rs1143684	3010390	G	0.3479	0.3283	A	0.4957	1.092
10	rs3750823	88717154	G	0.3479	0.3283	A	0.4957	1.092
1	rs5174	53712727	A	0.05412	0.04583	G	0.4959	1.191
9	rs6478322	120490389	A	0.05412	0.04583	G	0.4959	1.191
16	rs61733129	72057421	A	0.05412	0.04583	G	0.4959	1.191
20	rs73598378	44806628	A	0.05412	0.04583	G	0.4959	1.191
17	rs12936587	17543722	A	0.1082	0.09667	G	0.4961	1.134
3	rs3764779	118945796	C	0.3196	0.34	G	0.4962	0.9118
1	rs506832	57610673	C	0.2294	0.2475	A	0.4964	0.905
1	rs3795381	153004848	A	0.3479	0.3292	G	0.4964	1.087
3	rs4371530	174951756	A	0.3196	0.3392	G	0.4964	0.9152
5	rs6878704	176030090	G	0.3479	0.3292	A	0.4964	1.087
9	rs2479106	126525212	G	0.2294	0.2475	A	0.4964	0.905
14	rs177389	73727509	A	0.2294	0.2475	C	0.4964	0.905
15	rs2306575	72172883	G	0.2294	0.2475	C	0.4964	0.905
19	rs1017219	49573438	G	0.3479	0.3292	A	0.4964	1.087
19	rs1611775	49574125	G	0.3479	0.3292	C	0.4964	1.087
4	rs12512164	79300993	A	0.2552	0.2383	G	0.4965	1.095
6	rs599121	75056542	A	0.3505	0.3308	G	0.4965	1.092
8	rs2280840	145581557	G	0.3505	0.3308	A	0.4965	1.092
7	rs79534220	97875443	C	0.09021	0.1033	G	0.4966	0.8604
16	rs4673	88713236	A	0.09021	0.1033	G	0.4966	0.8604
19	rs11667393	49662027	A	0.09021	0.1033	G	0.4966	0.8604
4	rs337277	130030652	A	0.1701	0.1867	G	0.4967	0.8931
4	rs11466655	38776070	A	0.1933	0.1783	G	0.4969	1.104
5	rs7737765	16794780	A	0.1701	0.1875	G	0.4969	0.8882
7	rs2272263	55433884	C	0.1701	0.1875	A	0.4969	0.8882
9	rs3739741	107533175	C	0.2577	0.24	G	0.4969	1.1
17	rs792376	55438556	A	0.1701	0.1875	G	0.4969	0.8882
19	rs3752179	57088558	G	0.1701	0.1875	A	0.4969	0.8882
4	rs17429619	38933859	A	0.09021	0.1042	G	0.497	0.8527
10	rs625223	21414892	C	0.3222	0.3425	G	0.497	0.9124

15	rs3759873	44943797	G	0.09021	0.1042	A	0.497	0.8527
22	rs5748648	17280822	A	0.09021	0.1042	G	0.497	0.8527
2	rs62109368	5390055	C	0.3505	0.3317	A	0.4971	1.088
2	rs4670838	38482924	A	0.3505	0.3317	C	0.4971	1.088
3	rs3762648	109052732	A	0.3505	0.3317	G	0.4971	1.088
22	rs5752014	25287889	A	0.3505	0.3317	C	0.4971	1.088
3	rs6443987	184406997	A	0.3222	0.3417	C	0.4972	0.9158
8	rs4739003	63659635	A	0.3531	0.3333	G	0.4972	1.092
12	rs10879274	71633978	G	0.3222	0.3417	A	0.4972	0.9158
11	rs12788990	56143424	G	0.0799	0.06917	A	0.4974	1.169
11	rs12785840	56185224	G	0.0799	0.06917	A	0.4974	1.169
16	rs1503949	22958684	G	0.0799	0.06917	A	0.4974	1.169
17	rs711177	3926110	C	0.0799	0.06917	G	0.4974	1.169
1	rs2251108	117448282	G	0.1959	0.18	A	0.4977	1.11
4	rs10489087	13794416	A	0.1959	0.18	G	0.4977	1.11
22	rs5998135	32205632	C	0.03911	0.03083	A	0.4978	1.279
4	rs925642	187678866	A	0.3531	0.3342	G	0.4979	1.088
12	rs1042725	66358347	G	0.232	0.2508	A	0.4979	0.902
17	rs6561	25628820	G	0.232	0.25	A	0.4979	0.906
8	rs35022521	2000428	A	0.1263	0.1417	C	0.4982	0.8758
12	rs11047543	24788339	A	0.1263	0.1417	G	0.4982	0.8758
13	rs199891204	112723111	A	0.1263	0.1417	G	0.4982	0.8758
1	rs221057	16534255	G	0.1108	0.09833	C	0.4984	1.143
1	rs221058	16534646	C	0.1108	0.09833	G	0.4984	1.143
1	rs9508	28564279	G	0.1108	0.09833	A	0.4984	1.143
1	rs12732675	38135687	A	0.2603	0.2425	G	0.4984	1.099
3	rs17070967	64527275	G	0.1108	0.09833	A	0.4984	1.143
3	rs1534166	133537067	G	0.2603	0.2425	A	0.4984	1.099
6	rs12665339	30601232	G	0.2603	0.2425	A	0.4984	1.099
9	rs10761084	107531152	C	0.2603	0.2425	G	0.4984	1.099
20	rs1042579	23028724	A	0.2603	0.2425	G	0.4984	1.099
21	rs2297270	41725630	G	0.2603	0.2425	C	0.4984	1.099
3	rs9830734	184550501	A	0.3273	0.3475	G	0.4986	0.9137
8	rs3896980	8234714	G	0.3557	0.3367	A	0.4986	1.088
11	rs2075853	1247458	A	0.3557	0.3367	G	0.4986	1.088
6	rs80078646	132910485	A	0.04124	0.05083	T	0.4987	0.8031
12	rs11068531	117969507	A	0.04124	0.05083	G	0.4987	0.8031
1	rs10864219	216172380	A	0.1495	0.135	G	0.4989	1.126
3	rs6764363	312349	G	0.1495	0.135	A	0.4989	1.126
3	rs7618878	83089596	G	0.3273	0.3467	A	0.4989	0.917
3	rs1868531	165761918	A	0.3316	0.352	G	0.4989	0.9133
18	rs59044879	14660464	A	0.3582	0.3389	G	0.499	1.089
11	rs7927997	76301375	A	0.1727	0.1892	G	0.4991	0.8947

7	rs709060	149485059	G	0.1495	0.1352	A	0.4992	1.124
19	rs2288920	50091798	A	0.1727	0.19	C	0.4992	0.8898
1	rs79435376	74506942	A	0.04124	0.05167	G	0.4993	0.7895
1	rs17094777	74507315	A	0.04124	0.05167	G	0.4993	0.7895
6	rs3134604	32122386	C	0.04124	0.05167	G	0.4993	0.7895
6	rs17827619	119327632	A	0.04124	0.05167	G	0.4993	0.7895
17	rs7640	7606722	C	0.3299	0.3497	G	0.4993	0.9153
6	rs3094127	30697447	G	0.2345	0.2533	A	0.4994	0.9031
10	rs780668	73111408	A	0.3299	0.35	G	0.4994	0.9143
4	rs6552223	167694282	C	0.2345	0.2525	A	0.4995	0.9071
9	rs41316976	125316157	A	0.3608	0.3408	G	0.4995	1.092
3	rs9861033	56861222	G	0.3299	0.3489	A	0.4997	0.9187
17	rs17762452	2883588	A	0.3608	0.3414	C	0.4997	1.089
3	rs9840571	158429009	G	0.3608	0.3417	A	0.5	1.088
6	rs3130389	30188199	A	0.3608	0.3417	G	0.5	1.088
9	rs61740213	126134542	A	0.3608	0.3417	G	0.5	1.088
13	rs9541177	68183928	A	0.3608	0.3417	C	0.5	1.088
2	rs384526	193882	A	0.3325	0.3525	G	0.5001	0.9149
6	rs1233421	29501514	A	0.3325	0.3525	C	0.5001	0.9149
9	rs4742380	7581533	C	0.3325	0.3525	A	0.5001	0.9149
15	rs924311	67207563	G	0.3325	0.3525	A	0.5001	0.9149
15	rs6496932	85825567	A	0.3634	0.3433	C	0.5002	1.092
20	rs237655	59523840	A	0.1495	0.1358	G	0.5004	1.118
1	rs78555989	86249192	A	0.01036	0.006667	C	0.5005	1.56
3	rs1799977	37053568	G	0.03608	0.02917	A	0.5005	1.246
4	rs139828265	186263218	A	0.01036	0.006667	G	0.5005	1.56
5	rs75589774	37182902	A	0.03608	0.02917	G	0.5005	1.246
6	rs654128	117086378	A	0.03608	0.02917	C	0.5005	1.246
7	rs10265216	130900121	A	0.3325	0.3517	T	0.5005	0.9182
8	rs61729535	77768512	A	0.03608	0.02917	C	0.5005	1.246
11	rs34552201	118656777	G	0.03608	0.02917	A	0.5005	1.246
15	rs75532475	66832466	G	0.03608	0.02917	A	0.5005	1.246
17	rs147630160	4638474	A	0.01036	0.006667	G	0.5005	1.56
22	rs147609981	45684998	A	0.03608	0.02917	G	0.5005	1.246
1	rs117242156	12081895	A	0.0799	0.07	G	0.5007	1.154
1	rs2274955	179883190	A	0.0799	0.07	G	0.5007	1.154
2	rs66677602	179611711	A	0.2629	0.2458	C	0.5007	1.094
6	rs2073720	31112823	G	0.1108	0.09917	A	0.5007	1.132
8	rs17665859	445601	G	0.0799	0.07	A	0.5007	1.154
10	rs111033289	73560497	A	0.0799	0.07	G	0.5007	1.154
11	rs56067375	56431834	G	0.0799	0.07	A	0.5007	1.154
13	rs9568878	33704290	A	0.0799	0.07	G	0.5007	1.154
17	rs781831	3947644	G	0.3351	0.3556	A	0.5008	0.9131

5	rs2073643	131723288	G	0.2371	0.2558	A	0.5009	0.9041
12	rs11564148	40713901	A	0.2371	0.2558	T	0.5009	0.9041
10	rs17582416	35287650	C	0.2371	0.255	A	0.501	0.9081
2	rs74820922	242002306	A	0.1985	0.1833	G	0.5012	1.103
3	rs2272007	41996136	A	0.1985	0.1833	G	0.5012	1.103
3	rs6794047	160523531	A	0.3351	0.3542	G	0.5012	0.9188
6	rs2228213	12124855	A	0.2655	0.2475	G	0.5012	1.099
20	rs6034866	17603728	A	0.0799	0.07012	G	0.5012	1.152
4	rs306384	131711532	A	0.3394	0.3592	G	0.5013	0.9166
11	rs11217816	120190997	A	0.366	0.3467	G	0.5013	1.088
19	rs735911	2422177	A	0.366	0.3467	G	0.5013	1.088
6	rs6908425	20728731	A	0.1753	0.1925	G	0.5014	0.8914
8	rs7813902	29996256	A	0.1753	0.1917	G	0.5014	0.8962
17	rs4794160	45880748	A	0.1753	0.1917	G	0.5014	0.8962
17	rs2247856	74381555	A	0.1753	0.1925	G	0.5014	0.8914
6	rs9374080	109616420	G	0.09278	0.1058	A	0.5015	0.8641
10	rs2497306	94485211	C	0.1289	0.1442	A	0.5015	0.8782
11	rs2259633	93141580	C	0.09278	0.1058	A	0.5015	0.8641
17	rs3764897	4722876	A	0.09278	0.1058	G	0.5015	0.8641
21	rs458685	31177511	G	0.1289	0.1433	A	0.5015	0.8841
22	rs138708	39138332	A	0.09278	0.1058	G	0.5015	0.8641
1	rs7511651	16070871	A	0.01031	0.006667	G	0.5016	1.552
1	rs77585854	29189601	A	0.01031	0.006667	G	0.5016	1.552
1	rs55796784	38197170	A	0.01031	0.006667	G	0.5016	1.552
1	rs186213541	85598596	A	0.01031	0.006667	G	0.5016	1.552
1	rs140308307	100715340	G	0.01031	0.006667	A	0.5016	1.552
1	rs138326188	102296248	A	0.01031	0.006667	G	0.5016	1.552
1	rs150052375	112998890	A	0.01031	0.006667	G	0.5016	1.552
1	rs117378923	161681870	A	0.01031	0.006667	G	0.5016	1.552
1	rs186763441	171076835	G	0.01031	0.006667	A	0.5016	1.552
1	rs199655500	201869461	A	0.01031	0.006667	G	0.5016	1.552
1	rs76767146	225452983	G	0.01031	0.006667	A	0.5016	1.552
1	rs139232092	226576415	A	0.01031	0.006667	G	0.5016	1.552
2	rs56330730	39406336	A	0.01031	0.006667	G	0.5016	1.552
2	rs200468391	152520260	A	0.01031	0.006667	C	0.5016	1.552
2	rs146121793	158300469	C	0.01031	0.006667	G	0.5016	1.552
2	rs138036688	197708670	G	0.01031	0.006667	C	0.5016	1.552
2	rs115297652	238283529	A	0.01031	0.006667	G	0.5016	1.552
3	rs187487302	9854630	C	0.01031	0.006667	A	0.5016	1.552
3	rs138588985	108572524	A	0.01031	0.006667	C	0.5016	1.552
3	rs143663000	118865224	A	0.01031	0.006667	G	0.5016	1.552
3	rs201541571	125726016	G	0.01031	0.006667	A	0.5016	1.552
3	rs187565189	133302985	A	0.01031	0.006667	G	0.5016	1.552

3	rs79043251	139097997	A	0.01031	0.006667	G	0.5016	1.552
4	rs145691438	71024132	A	0.01031	0.006667	C	0.5016	1.552
5	rs56228802	56177692	G	0.01031	0.006667	C	0.5016	1.552
5	rs146871001	77477331	A	0.01031	0.006667	G	0.5016	1.552
5	rs181902283	126253720	A	0.01031	0.006667	G	0.5016	1.552
5	rs185683917	150425501	A	0.01031	0.006667	G	0.5016	1.552
6	rs139386667	26043876	A	0.01031	0.006667	G	0.5016	1.552
6	rs7742662	74491002	A	0.01031	0.006667	C	0.5016	1.552
6	rs139704194	76731849	A	0.01031	0.006667	G	0.5016	1.552
7	rs117545587	22985435	A	0.01031	0.006667	G	0.5016	1.552
7	rs196594	36327317	A	0.01031	0.006667	G	0.5016	1.552
8	rs142080208	25317913	C	0.01031	0.006667	G	0.5016	1.552
8	rs200412403	81899679	A	0.01031	0.006667	G	0.5016	1.552
8	rs180963656	139767402	A	0.01031	0.006667	G	0.5016	1.552
8	rs150818450	145057110	A	0.01031	0.006667	G	0.5016	1.552
9	rs11140503	86914503	A	0.01031	0.006667	C	0.5016	1.552
10	rs118055903	108923782	A	0.01031	0.006667	G	0.5016	1.552
10	rs149706492	129904036	A	0.01031	0.006667	G	0.5016	1.552
11	rs139943633	5153800	C	0.01031	0.006667	G	0.5016	1.552
11	rs140471717	12237861	G	0.01031	0.006667	A	0.5016	1.552
11	rs187630793	44076808	A	0.01031	0.006667	C	0.5016	1.552
11	rs139773791	55945018	A	0.01031	0.006667	G	0.5016	1.552
11	rs199651989	62292863	A	0.01031	0.006667	G	0.5016	1.552
11	rs542998	63487386	A	0.01031	0.006667	G	0.5016	1.552
11	rs141303580	66359101	A	0.01031	0.006667	G	0.5016	1.552
11	rs187027607	70202278	A	0.01031	0.006667	G	0.5016	1.552
12	rs76064726	55420426	C	0.01031	0.006667	A	0.5016	1.552
12	rs138682950	122669256	A	0.01031	0.006667	G	0.5016	1.552
12	rs150627547	133430146	A	0.01031	0.006667	G	0.5016	1.552
13	rs41288291	42301395	A	0.01031	0.006667	G	0.5016	1.552
14	rs114970740	24806525	G	0.01031	0.006667	A	0.5016	1.552
14	rs74345106	25100391	A	0.01031	0.006667	C	0.5016	1.552
14	rs35162541	69257119	A	0.01031	0.006667	C	0.5016	1.552
15	rs190958876	82564081	G	0.01031	0.006667	A	0.5016	1.552
16	rs75894599	2814461	A	0.01031	0.006667	G	0.5016	1.552
16	rs201203260	3063619	G	0.01031	0.006667	A	0.5016	1.552
16	rs144187091	71318257	G	0.01031	0.006667	A	0.5016	1.552
16	rs138147953	72164125	G	0.01031	0.006667	C	0.5016	1.552
16	rs185608121	90106814	A	0.01031	0.006667	G	0.5016	1.552
17	rs199821422	8702369	A	0.01031	0.006667	G	0.5016	1.552
17	rs2955365	18023897	G	0.3686	0.3483	A	0.5016	1.092
17	rs114442310	35345963	A	0.01031	0.006667	G	0.5016	1.552
17	rs188217621	38447337	G	0.01031	0.006667	A	0.5016	1.552

17	rs12544	64222212	G	0.01031	0.006667	A	0.5016	1.552
19	rs117390715	1051515	G	0.01031	0.006667	C	0.5016	1.552
19	rs202166136	1800152	A	0.01031	0.006667	G	0.5016	1.552
19	rs6511267	10031745	G	0.01031	0.006667	A	0.5016	1.552
19	rs186551702	50165760	G	0.01031	0.006667	A	0.5016	1.552
19	rs199837060	50965234	A	0.01031	0.006667	G	0.5016	1.552
20	rs35327491	3180666	A	0.01031	0.006667	C	0.5016	1.552
21	rs139360624	31538769	A	0.01031	0.006667	G	0.5016	1.552
2	rs6734238	113841030	G	0.0567	0.0475	A	0.5017	1.205
3	rs79093065	167293773	G	0.0567	0.0475	C	0.5017	1.205
10	rs77382849	120810098	A	0.0567	0.0475	G	0.5017	1.205
5	rs12514417	125887715	C	0.09278	0.1067	A	0.5018	0.8565
5	rs117873033	150097846	G	0.01031	0.006678	A	0.5019	1.549
7	rs115211979	7459554	A	0.01031	0.006678	C	0.5019	1.549
18	rs200207441	77246527	A	0.01031	0.006678	G	0.5019	1.549
4	rs13144371	88732746	A	0.3686	0.3492	G	0.502	1.088
6	rs2228209	12120588	A	0.2655	0.2483	G	0.502	1.094
11	rs3741231	1857751	G	0.3376	0.3567	C	0.502	0.9194
16	rs13331769	77246218	A	0.3686	0.3492	G	0.502	1.088
2	rs3731752	179398509	A	0.201	0.185	C	0.5021	1.108
3	rs9284879	44284584	G	0.201	0.185	A	0.5021	1.108
6	rs2071541	32812858	G	0.1521	0.1375	A	0.5021	1.125
7	rs3800962	18066569	A	0.201	0.185	C	0.5021	1.108
22	rs5748622	17264565	C	0.1521	0.1375	A	0.5021	1.125
2	rs13000389	145871379	A	0.3402	0.3608	G	0.5023	0.9134
16	rs56215266	77331028	G	0.3402	0.36	A	0.5023	0.9167
20	rs11780	43566787	G	0.3402	0.3608	A	0.5023	0.9134
2	rs13010713	181996045	A	0.2397	0.2575	G	0.5024	0.909
7	rs671694	4213975	A	0.2397	0.2575	G	0.5024	0.909
11	rs1452928	84822321	C	0.06443	0.07667	G	0.5024	0.8294
17	rs1043515	36922196	G	0.2397	0.2575	A	0.5024	0.909
20	rs7361799	23421079	G	0.06443	0.07667	A	0.5024	0.8294
22	rs11107	32875190	G	0.2397	0.2575	A	0.5024	0.909
6	rs7748681	32773858	A	0.3731	0.3539	C	0.5025	1.086
8	rs11778341	10465344	A	0.3446	0.3648	C	0.5025	0.9154
17	rs187319098	1989027	G	0.01031	0.0067	C	0.5026	1.544
2	rs12468894	141790694	A	0.3686	0.35	G	0.5027	1.084
15	rs2292305	39880822	G	0.3686	0.35	A	0.5027	1.084
1	rs74642512	55307332	A	0.1134	0.1008	C	0.503	1.141
5	rs2241698	149390148	A	0.1134	0.1008	G	0.503	1.141
11	rs417425	5510284	A	0.1134	0.1008	G	0.503	1.141
17	rs2084881	46357120	A	0.1134	0.1008	G	0.503	1.141
17	rs9303542	46411500	G	0.1134	0.1008	A	0.503	1.141

1	rs143778703	78194300	A	0.02062	0.01583	G	0.5032	1.309
1	rs17121403	100335977	G	0.02062	0.01583	A	0.5032	1.309
1	rs79923436	214819818	A	0.02062	0.01583	G	0.5032	1.309
2	rs12691673	144092133	A	0.02062	0.01583	C	0.5032	1.309
3	rs150482056	37559040	G	0.02062	0.01583	A	0.5032	1.309
3	rs117375173	122002576	G	0.02062	0.01583	A	0.5032	1.309
4	rs138275098	126373858	A	0.02062	0.01583	G	0.5032	1.309
6	rs61730330	29641329	A	0.02062	0.01583	G	0.5032	1.309
6	rs117184249	152722413	A	0.02062	0.01583	G	0.5032	1.309
6	rs117546892	159646624	A	0.02062	0.01583	C	0.5032	1.309
7	rs3735051	129944402	A	0.02062	0.01583	C	0.5032	1.309
10	rs2306322	73822855	A	0.02062	0.01583	G	0.5032	1.309
11	rs2271796	113639564	G	0.02062	0.01583	A	0.5032	1.309
12	rs77514395	11461223	A	0.02062	0.01583	G	0.5032	1.309
12	rs861204	51237816	A	0.02062	0.01583	G	0.5032	1.309
14	rs117775572	20851708	G	0.02062	0.01583	A	0.5032	1.309
15	rs140240186	79586159	G	0.02062	0.01583	A	0.5032	1.309
15	rs78671682	79586669	A	0.02062	0.01583	C	0.5032	1.309
19	rs117193413	18710461	G	0.02062	0.01583	A	0.5032	1.309
19	rs61744158	30935704	A	0.02062	0.01583	C	0.5032	1.309
19	rs151324898	58084772	A	0.02062	0.01583	G	0.5032	1.309
22	rs35537221	23632547	G	0.02062	0.01583	A	0.5032	1.309
6	rs7381376	32767673	G	0.3737	0.3542	C	0.5033	1.088
9	rs10491539	17708814	C	0.201	0.1858	A	0.5033	1.102
20	rs6049375	24058407	A	0.201	0.1858	G	0.5033	1.102
12	rs2306283	21329738	A	0.268	0.2508	G	0.5034	1.094
7	rs13223723	99552892	G	0.1521	0.1383	A	0.5035	1.117
11	rs1815811	119059404	G	0.1521	0.1383	A	0.5035	1.117
12	rs17119344	57422934	A	0.1521	0.1383	G	0.5035	1.117
3	rs2290532	31789582	A	0.1778	0.1942	G	0.5036	0.8977
3	rs4343667	77103671	A	0.1778	0.1942	G	0.5036	0.8977
14	rs4904254	85992271	A	0.3454	0.3658	G	0.5036	0.9145
21	rs61748614	30342933	A	0.1778	0.195	C	0.5036	0.8929
1	rs4847399	93584606	G	0.3454	0.365	A	0.5037	0.9178
1	rs1060622	93620393	G	0.3454	0.365	A	0.5037	0.9178
2	rs12623288	46634645	G	0.3763	0.3564	A	0.5037	1.089
6	rs1233427	29496664	A	0.3454	0.365	G	0.5037	0.9178
6	rs1233426	29498102	A	0.3454	0.365	G	0.5037	0.9178
6	rs362546	29499416	G	0.3454	0.365	A	0.5037	0.9178
10	rs2486864	7011967	G	0.3454	0.365	A	0.5037	0.9178
11	rs1442449	100165790	A	0.2423	0.2608	G	0.5037	0.9061
8	rs4715	22021037	A	0.2423	0.26	C	0.5039	0.91
5	rs11739136	169810796	A	0.08247	0.07167	G	0.5041	1.164

6	rs11547228	79692751	A	0.08247	0.07167	G	0.5041	1.164
15	rs11070796	31376365	A	0.2036	0.1875	G	0.5042	1.108
11	rs10766542	19352288	A	0.3479	0.3683	G	0.5043	0.9151
12	rs7137188	854331	G	0.3479	0.3683	A	0.5043	0.9151
17	rs732928	21144074	G	0.3479	0.3683	A	0.5043	0.9151
6	rs206765	32964696	T	0.3479	0.3675	A	0.5044	0.9184
8	rs4831837	12711863	G	0.3479	0.3675	A	0.5044	0.9184
13	rs9545295	80580456	G	0.3763	0.3575	A	0.5045	1.084
14	rs17114908	44836538	A	0.3789	0.3592	G	0.5045	1.088
12	rs4964883	132323250	G	0.1321	0.1472	C	0.5046	0.8823
5	rs13174484	41069852	G	0.1314	0.1467	A	0.5047	0.8805
11	rs3737336	125830697	G	0.2706	0.2533	A	0.5047	1.094
2	rs834514	17696573	G	0.1314	0.1458	A	0.5048	0.8864
18	rs471713	34269346	G	0.3505	0.3708	A	0.5049	0.9156
2	rs4381746	29779019	G	0.3814	0.3617	A	0.5051	1.088
15	rs11636311	48657423	A	0.3505	0.37	C	0.5051	0.9189
15	rs80215855	76019567	T	0.1134	0.1017	A	0.5051	1.13
15	rs8040248	84728405	G	0.3789	0.36	A	0.5051	1.084
2	rs1049817	27550967	G	0.1546	0.14	A	0.5053	1.124
3	rs7647307	69623188	C	0.2448	0.2625	A	0.5053	0.9109
5	rs1135093	149008521	A	0.1546	0.14	G	0.5053	1.124
15	rs1126823	89417238	G	0.2732	0.255	A	0.5053	1.098
16	rs61734410	1252369	G	0.1546	0.1402	A	0.5055	1.122
4	rs1030757	93697153	A	0.3531	0.3733	C	0.5056	0.9162
6	rs4959037	29936307	A	0.384	0.3642	T	0.5057	1.089
6	rs9444825	91779382	A	0.384	0.3642	G	0.5057	1.089
11	rs11040923	6585007	G	0.3814	0.3625	A	0.5057	1.084
12	rs1613931	52777578	A	0.1804	0.197	G	0.5057	0.8973
18	rs16958816	32815683	G	0.3814	0.3625	A	0.5057	1.084
1	rs2292822	201777568	C	0.1804	0.1967	G	0.5058	0.8992
6	rs2234079	36339136	A	0.1804	0.1967	G	0.5058	0.8992
18	rs1943816	71058622	G	0.1804	0.1975	A	0.5058	0.8944
9	rs2248136	17502205	A	0.0567	0.04833	C	0.506	1.184
12	rs10876838	56030938	A	0.2732	0.2558	G	0.506	1.093
12	rs7970885	56031273	A	0.2732	0.2558	G	0.506	1.093
17	rs6504340	46617019	A	0.0567	0.04833	G	0.506	1.184
11	rs35652107	46339011	A	0.09536	0.1083	G	0.5061	0.8676
7	rs4728090	127830114	G	0.3557	0.3758	A	0.5062	0.9167
16	rs60922721	10359780	A	0.3557	0.3758	G	0.5062	0.9167
19	rs351111	844020	A	0.384	0.365	G	0.5062	1.085
7	rs10229731	100675376	C	0.2062	0.19	A	0.5063	1.107
1	rs584367	20442054	A	0.2474	0.2658	G	0.5065	0.908
14	rs1471356	98303331	G	0.1546	0.1408	A	0.5065	1.116

4	rs16892095	15518357	C	0.2758	0.2575	A	0.5066	1.098
9	rs2779529	101350077	A	0.2758	0.2575	C	0.5066	1.098
3	rs2981482	124802888	G	0.3582	0.3783	A	0.5068	0.9173
4	rs6844558	185981436	G	0.3866	0.3675	A	0.5068	1.085
8	rs7016250	65493429	A	0.3866	0.3675	C	0.5068	1.085
16	rs2229317	10989219	C	0.3582	0.3783	G	0.5068	0.9173
20	rs4815853	5762821	G	0.3582	0.3783	A	0.5068	0.9173
16	rs9930484	12657321	A	0.3627	0.3825	G	0.507	0.9188
5	rs2910313	140553581	G	0.3582	0.3775	C	0.5071	0.9205
5	rs1549196	151815911	C	0.3582	0.3775	A	0.5071	0.9205
1	rs880303	39340282	A	0.2758	0.2583	G	0.5073	1.093
1	rs2147914	39352271	A	0.2758	0.2583	C	0.5073	1.093
1	rs6689850	223567401	G	0.116	0.1033	A	0.5074	1.138
1	rs6682552	223567808	A	0.116	0.1033	G	0.5074	1.138
2	rs61750360	131688783	A	0.116	0.1033	G	0.5074	1.138
2	rs2233385	233897503	A	0.116	0.1033	G	0.5074	1.138
3	rs55748151	121251868	C	0.116	0.1033	A	0.5074	1.138
6	rs9367532	53310839	G	0.3608	0.3808	A	0.5074	0.9178
8	rs9314565	5230140	A	0.3608	0.3808	G	0.5074	0.9178
8	rs2069556	133920518	G	0.3918	0.3717	A	0.5074	1.089
9	rs7847871	85941646	G	0.3608	0.3808	A	0.5074	0.9178
22	rs3213445	51015838	G	0.3918	0.3717	A	0.5074	1.089
19	rs8104812	52887437	C	0.2513	0.2696	A	0.5076	0.9092
6	rs884366	109574095	A	0.3608	0.38	G	0.5077	0.9211
11	rs2291334	44940828	G	0.3608	0.38	A	0.5077	0.9211
2	rs7575423	4301257	C	0.25	0.2683	A	0.5078	0.9089
5	rs1366594	88376061	A	0.3918	0.3725	C	0.5078	1.085
6	rs9369905	49489397	A	0.134	0.1492	C	0.5078	0.8827
9	rs320209	104944755	A	0.134	0.1492	T	0.5078	0.8827
11	rs590162	122195989	G	0.3918	0.3725	A	0.5078	1.085
12	rs1800377	6173433	A	0.134	0.1492	G	0.5078	0.8827
18	rs12608143	36492188	G	0.3918	0.3725	A	0.5078	1.085
4	rs6831256	3473139	G	0.3634	0.3831	A	0.5079	0.9191
5	rs26618	96130836	G	0.2784	0.26	A	0.5079	1.098
7	rs2462603	145813854	G	0.3634	0.3833	A	0.508	0.9183
13	exm1080722	113479813	A	0.134	0.1483	G	0.508	0.8886
13	rs9549564	113479813	A	0.134	0.1483	G	0.508	0.8886
16	rs416603	11364079	T	0.134	0.1483	A	0.508	0.8886
16	rs8047022	87836659	G	0.134	0.1483	A	0.508	0.8886
19	rs13343641	52825032	A	0.183	0.1992	G	0.508	0.9006
11	rs2305535	66272237	A	0.25	0.2675	G	0.5081	0.9128
14	rs327463	81251255	G	0.25	0.2675	A	0.5081	0.9128
6	rs1061808	32136547	A	0.3634	0.3825	C	0.5083	0.9216

5	rs2934803	63490739	C	0.2088	0.1925	A	0.5084	1.107
1	rs12407007	30890532	A	0.2784	0.2608	G	0.5085	1.093
1	rs41309181	52703169	G	0.02577	0.03417	A	0.5085	0.7478
1	rs76320728	52823546	G	0.02577	0.03417	C	0.5085	0.7478
1	rs76182569	197643277	T	0.02577	0.03417	A	0.5085	0.7478
3	rs17052097	52396410	A	0.02577	0.03417	G	0.5085	0.7478
3	rs114223323	57489835	G	0.02577	0.03417	A	0.5085	0.7478
3	rs76555381	190106074	G	0.02577	0.03417	C	0.5085	0.7478
4	rs941758	74718941	C	0.366	0.3858	A	0.5085	0.9188
6	rs2744120	22281258	A	0.3969	0.3767	G	0.5085	1.089
13	rs9572423	70846203	G	0.3969	0.3767	A	0.5085	1.089
13	rs2078588	72301632	A	0.2784	0.2608	C	0.5085	1.093
14	rs2273837	23342582	A	0.02577	0.03417	G	0.5085	0.7478
14	rs1514928	62678303	A	0.02577	0.03417	C	0.5085	0.7478
15	rs45611935	99467189	G	0.02577	0.03417	A	0.5085	0.7478
18	rs2957128	60060735	G	0.2784	0.2608	A	0.5085	1.093
11	rs3847531	92534442	A	0.06701	0.07833	G	0.5086	0.8451
11	rs12718465	116707736	A	0.02577	0.03333	G	0.5087	0.7672
12	rs2229300	57865558	A	0.02577	0.03333	C	0.5087	0.7672
15	rs142108245	34134195	G	0.02577	0.03333	A	0.5087	0.7672
17	rs62067380	46000406	G	0.02577	0.03333	A	0.5087	0.7672
20	rs35972756	44511257	A	0.02577	0.03333	G	0.5087	0.7672
20	rs45447691	44512082	A	0.02577	0.03333	G	0.5087	0.7672
22	rs16989528	32217539	A	0.02577	0.03333	G	0.5087	0.7672
5	rs1173727	32830521	A	0.399	0.38	G	0.5088	1.083
17	rs1056534	80708601	G	0.3969	0.3775	C	0.5088	1.085
14	rs10131813	23745533	A	0.366	0.385	G	0.5089	0.9221
3	rs10935268	137447485	A	0.06701	0.07917	G	0.509	0.8354
16	rs4268750	25753251	G	0.3686	0.3892	A	0.509	0.9161
19	rs4804715	12641232	A	0.3686	0.3892	G	0.509	0.9161
20	rs139701837	6022529	G	0.06701	0.07917	C	0.509	0.8354
1	rs5355	169695870	A	0.04381	0.05333	G	0.5091	0.8133
4	rs7675918	134169341	A	0.3686	0.3883	G	0.5091	0.9193
5	rs6872317	1144466	G	0.3686	0.3883	A	0.5091	0.9193
5	rs2434322	141334921	A	0.2526	0.2708	G	0.5091	0.9098
5	rs1744356	157551411	A	0.2526	0.2708	C	0.5091	0.9098
8	rs2640726	27405809	G	0.3686	0.3883	A	0.5091	0.9193
11	rs4963128	589564	A	0.04381	0.05333	G	0.5091	0.8133
13	rs553717	92345579	A	0.2526	0.2708	G	0.5091	0.9098
14	rs1053419	104381502	G	0.04381	0.05333	A	0.5091	0.8133
22	rs854936	20260814	C	0.04381	0.05333	A	0.5091	0.8133
22	rs75680863	31007023	T	0.04381	0.05333	A	0.5091	0.8133
6	rs3115673	31810085	A	0.2526	0.2717	C	0.5092	0.906

2	rs4241344	69049939	A	0.3995	0.38	C	0.5093	1.085
5	rs12653946	1895829	A	0.3995	0.38	G	0.5093	1.085
2	rs10189160	157012321	G	0.116	0.1042	A	0.5094	1.128
7	rs6462411	3915564	G	0.116	0.1042	A	0.5094	1.128
7	rs2282978	92264410	G	0.116	0.1042	A	0.5094	1.128
16	rs12447780	31860399	A	0.3711	0.3913	G	0.5094	0.918
1	rs7483	110279701	G	0.2526	0.27	A	0.5095	0.9137
2	rs61750773	84932720	G	0.04381	0.05417	A	0.5095	0.8001
2	rs61744273	85059227	A	0.04381	0.05417	G	0.5095	0.8001
2	rs7426112	236792769	A	0.3686	0.3875	C	0.5095	0.9226
4	rs3733251	77192838	C	0.04381	0.05417	A	0.5095	0.8001
6	rs1003581	29540204	A	0.3686	0.3875	G	0.5095	0.9226
6	rs10447346	66318585	A	0.2526	0.27	G	0.5095	0.9137
8	rs16939380	77768390	C	0.1572	0.1433	A	0.5095	1.115
11	rs1925368	34378381	G	0.3686	0.3875	C	0.5095	0.9226
14	rs4934	95080803	G	0.3686	0.3875	A	0.5095	0.9226
17	rs2304967	33805180	C	0.1572	0.1433	G	0.5095	1.115
17	rs9895154	42267949	A	0.04381	0.05417	G	0.5095	0.8001
7	rs2249070	138872345	A	0.3711	0.3917	C	0.5096	0.9166
10	rs3824699	26355992	A	0.3711	0.3908	G	0.5096	0.9199
10	rs3817420	26357748	G	0.3711	0.3908	A	0.5096	0.9199
10	rs1819578	82890180	A	0.3711	0.3917	G	0.5096	0.9166
13	rs7334068	86632597	G	0.04381	0.05435	A	0.5096	0.7973
20	rs76270899	19665822	A	0.04381	0.05435	G	0.5096	0.7973
7	rs1464890	129664312	A	0.2809	0.2633	G	0.5098	1.093
1	rs2274327	9009406	A	0.1856	0.2025	G	0.51	0.8973
2	rs2082871	20712300	A	0.1856	0.2025	G	0.51	0.8973
3	rs11711441	182821275	A	0.1856	0.2025	G	0.51	0.8973
11	rs4944092	75909619	G	0.1856	0.2025	A	0.51	0.8973
5	rs28019	115733327	C	0.1856	0.2017	A	0.5101	0.902
6	rs9390307	145677803	G	0.3737	0.3942	A	0.5101	0.9171
7	rs7799478	98681222	G	0.3737	0.3942	A	0.5101	0.9171
2	rs56378630	181710499	G	0.3737	0.3933	A	0.5102	0.9203
8	rs2741669	6802073	A	0.4046	0.385	G	0.5103	1.086
1	rs200736807	11115844	C	0.01289	0.01917	G	0.5104	0.6681
2	rs10204405	165047746	A	0.4021	0.3833	G	0.5104	1.082
2	rs2233391	233899126	A	0.01289	0.01917	C	0.5104	0.6681
3	rs76438582	196675533	A	0.01289	0.01917	G	0.5104	0.6681
6	rs114452424	31593369	A	0.01289	0.01917	G	0.5104	0.6681
7	rs6951399	15496808	A	0.4021	0.3833	C	0.5104	1.082
8	rs77825059	72211326	A	0.01289	0.01917	G	0.5104	0.6681
11	rs150220405	124294006	A	0.01289	0.01917	G	0.5104	0.6681
13	rs9508907	20398669	A	0.01289	0.01917	G	0.5104	0.6681

13	rs75008861	25478083	G	0.01289	0.01917	A	0.5104	0.6681
20	rs182585703	44506534	A	0.01289	0.01917	G	0.5104	0.6681
1	rs1561173	216193693	A	0.2552	0.2742	C	0.5105	0.9069
4	rs13846	110605744	T	0.2552	0.2742	A	0.5105	0.9069
1	rs1722710	48672401	G	0.3763	0.3967	A	0.5106	0.9176
4	rs17081935	57823476	A	0.3763	0.3967	G	0.5106	0.9176
6	rs3734447	127771452	C	0.08505	0.07417	A	0.5106	1.16
6	rs2881194	167267919	C	0.3763	0.3967	A	0.5106	0.9176
7	rs13306698	94940782	G	0.08505	0.07417	A	0.5106	1.16
11	rs7117404	47127153	G	0.08505	0.07417	A	0.5106	1.16
16	rs17763066	74950469	A	0.3763	0.3967	G	0.5106	0.9176
17	rs3744795	76803659	G	0.08505	0.07417	A	0.5106	1.16
18	rs73973908	72238472	A	0.2113	0.1953	C	0.5106	1.104
5	rs4957233	40049919	G	0.3763	0.3958	A	0.5107	0.9208
5	rs9313625	172690224	C	0.3763	0.3958	A	0.5107	0.9208
6	rs2743961	165493183	G	0.3763	0.3958	A	0.5107	0.9208
7	rs28505524	150447027	C	0.2809	0.2642	A	0.5107	1.088
11	rs7105881	112456221	A	0.3763	0.3958	G	0.5107	0.9208
14	rs1959379	44974966	A	0.3763	0.3957	G	0.5107	0.9215
17	rs7225329	43101382	A	0.3763	0.3958	G	0.5107	0.9208
19	rs8106495	36394678	G	0.09794	0.1108	A	0.5107	0.871
3	rs79808533	183508590	G	0.09794	0.1117	A	0.5108	0.8637
3	rs74496163	183508714	A	0.09794	0.1117	G	0.5108	0.8637
8	rs4736872	40451701	A	0.4072	0.3875	G	0.5108	1.086
9	rs2271877	111853412	A	0.09794	0.1117	G	0.5108	0.8637
10	rs4986893	96540410	A	0.09794	0.1117	G	0.5108	0.8637
10	rs871026	131293145	G	0.4072	0.3875	A	0.5108	1.086
12	rs233716	113039943	A	0.4072	0.3875	G	0.5108	1.086
16	rs1366660	77940068	G	0.4072	0.3875	A	0.5108	1.086
14	rs4293296	54857286	A	0.1366	0.1517	G	0.5109	0.8849
18	rs3737512	71816278	A	0.1366	0.1517	G	0.5109	0.8849
19	rs2032887	8121360	G	0.1366	0.1517	A	0.5109	0.8849
22	rs738786	24034288	A	0.4046	0.3858	G	0.5109	1.082
2	rs59332477	220417266	A	0.3789	0.3992	G	0.511	0.9181
6	rs2621421	32740671	C	0.2835	0.2658	G	0.511	1.093
6	rs2341551	149814762	A	0.2835	0.2658	G	0.511	1.093
2	rs6744817	11725908	A	0.03866	0.03083	G	0.5111	1.264
2	rs36030386	11736305	A	0.03866	0.03083	G	0.5111	1.264
4	rs35389530	113352682	A	0.03866	0.03083	G	0.5111	1.264
6	rs76451285	6197488	A	0.03866	0.03083	G	0.5111	1.264
8	rs12549394	104343607	A	0.03866	0.03083	C	0.5111	1.264
16	rs74001006	819441	G	0.03866	0.03083	C	0.5111	1.264
21	rs2836823	40380249	A	0.03866	0.03083	G	0.5111	1.264

4	rs6839672	80993133	G	0.4098	0.39	A	0.5112	1.086
9	rs2796441	84308948	G	0.4072	0.3883	A	0.5113	1.082
11	rs17859808	1074927	A	0.03866	0.03094	G	0.5115	1.26
12	rs1922117	63789836	A	0.3814	0.4017	C	0.5115	0.9186
14	rs2270074	74318645	G	0.3814	0.4017	C	0.5115	0.9186
14	rs2270073	74318754	C	0.3814	0.4017	A	0.5115	0.9186
2	rs146192357	107459861	A	0.05928	0.05	C	0.5116	1.197
4	rs117033348	38800022	G	0.05928	0.05	A	0.5116	1.197
6	rs540825	154414446	A	0.05928	0.05	T	0.5116	1.197
15	rs35875311	89838318	A	0.05928	0.05	T	0.5116	1.197
16	rs17358402	71967927	A	0.05928	0.05	G	0.5116	1.197
5	rs2280170	126993282	A	0.2577	0.2758	G	0.5117	0.9116
6	rs362525	29543646	C	0.2577	0.2767	A	0.5117	0.9078
6	rs3131886	29664452	G	0.4124	0.3925	A	0.5117	1.086
7	rs6973628	27413263	A	0.1186	0.1058	G	0.5118	1.136
9	rs871461	135077361	A	0.1186	0.1058	C	0.5118	1.136
15	rs7177192	40898643	C	0.2835	0.2667	G	0.5119	1.088
15	rs8040502	40915190	G	0.2835	0.2667	A	0.5119	1.088
6	exm2257738	31084048	G	0.1881	0.205	A	0.512	0.8987
6	rs3094216	31084048	G	0.1881	0.205	A	0.512	0.8987
17	rs10278	46939658	G	0.1881	0.205	C	0.512	0.8987
4	rs1037926	91227681	G	0.4149	0.395	A	0.5121	1.086
8	rs3802177	118185025	A	0.4149	0.395	G	0.5121	1.086
12	rs10783486	52362786	A	0.4149	0.395	G	0.5121	1.086
16	rs3096277	83764204	G	0.384	0.4033	A	0.5123	0.9223
1	rs1738023	19611241	A	0.2139	0.1975	G	0.5124	1.106
6	rs2071555	32905086	A	0.2139	0.1975	C	0.5124	1.106
10	rs10999147	71880858	G	0.1598	0.1458	A	0.5124	1.114
14	rs8007716	100784970	A	0.2139	0.1975	G	0.5124	1.106
1	rs12142968	234694041	C	0.4175	0.3975	A	0.5125	1.086
11	rs297494	121291294	C	0.3866	0.4067	A	0.5125	0.9195
12	rs6246	28122893	G	0.4149	0.3958	A	0.5125	1.083
19	rs1035451	54962467	A	0.4149	0.3958	G	0.5125	1.083
3	rs9831488	160755062	A	0.01289	0.02	G	0.5127	0.6397
4	rs4861066	40810663	G	0.01289	0.02	A	0.5127	0.6397
4	rs148277206	187153290	A	0.01289	0.02	G	0.5127	0.6397
5	rs323562	40853160	G	0.01289	0.02	A	0.5127	0.6397
5	rs1036199	156531736	C	0.01289	0.02	A	0.5127	0.6397
7	rs6464749	146020936	G	0.01289	0.02	A	0.5127	0.6397
8	rs78404930	27528765	A	0.01289	0.02	C	0.5127	0.6397
9	rs961831	22362104	C	0.01289	0.02	A	0.5127	0.6397
12	rs2069413	56365381	G	0.01289	0.02	C	0.5127	0.6397
14	rs34323725	81743718	A	0.01289	0.02	G	0.5127	0.6397

15	rs78680460	75913260	A	0.01289	0.02	G	0.5127	0.6397
16	rs75401081	1997206	A	0.01289	0.02	G	0.5127	0.6397
17	rs9894448	14514409	G	0.4201	0.3998	A	0.5128	1.087
1	rs2274333	9017204	A	0.4201	0.4	G	0.5129	1.087
7	rs1175000	22024040	G	0.3892	0.4092	A	0.5129	0.92
11	rs10741657	14914878	A	0.4175	0.3983	G	0.5129	1.083
11	rs2060793	14915310	A	0.4175	0.3983	G	0.5129	1.083
17	rs7214723	3775848	G	0.3892	0.4092	A	0.5129	0.92
1	rs2749146	23582880	G	0.2861	0.2692	A	0.513	1.088
10	rs1764437	31475114	A	0.2603	0.2783	G	0.513	0.9125
15	rs4533251	97262427	A	0.2861	0.2692	C	0.513	1.088
12	rs2617170	10560957	A	0.3892	0.4083	G	0.5132	0.9232
13	rs1885894	22647183	A	0.3892	0.4083	G	0.5132	0.9232
3	rs1605524	21376648	A	0.4227	0.4025	G	0.5133	1.087
5	rs870009	119600902	G	0.3918	0.4117	A	0.5133	0.9205
6	rs3815081	30114074	G	0.2887	0.2708	A	0.5133	1.093
6	rs2814899	38389301	G	0.4227	0.4025	A	0.5133	1.087
5	rs61749618	98192167	A	0.08505	0.075	G	0.5134	1.146
15	rs17802279	65108504	A	0.08505	0.075	T	0.5134	1.146
6	rs10484565	32795032	A	0.1186	0.1067	G	0.5136	1.126
15	rs34348991	43044193	A	0.1186	0.1067	G	0.5136	1.126
6	rs395180	4768691	G	0.1392	0.1542	A	0.5139	0.887
15	rs187316	85405995	G	0.1392	0.1536	A	0.5139	0.891
2	rs9653442	100825367	G	0.4253	0.4058	A	0.514	1.083
11	rs10893053	123480981	G	0.1907	0.2075	A	0.514	0.9001
3	rs756644	169084827	C	0.4278	0.4075	A	0.5141	1.087
19	rs8108269	46158513	A	0.4278	0.4075	C	0.5141	1.087
3	rs4141499	127641968	C	0.3943	0.4133	A	0.5142	0.9241
3	rs6764714	186917751	C	0.2887	0.2717	G	0.5142	1.088
10	rs11101394	49805216	A	0.1598	0.1467	G	0.5142	1.107
12	rs298109	52827818	A	0.2629	0.2817	G	0.5142	0.9095
12	rs7962801	55346235	G	0.1598	0.1467	A	0.5142	1.107
13	rs732774	52523808	A	0.3969	0.4167	G	0.5142	0.9214
13	rs1061472	52524488	G	0.3943	0.4133	A	0.5142	0.9241
19	rs10414398	45021210	A	0.1392	0.155	G	0.5142	0.8814
1	rs232777	59125683	A	0.1907	0.2067	G	0.5143	0.9047
2	rs2385405	220197321	A	0.1907	0.2067	G	0.5143	0.9047
2	rs2385404	220197388	A	0.1907	0.2067	C	0.5143	0.9047
5	rs1465567	38370537	G	0.1907	0.2067	A	0.5143	0.9047
5	rs10068403	129520679	A	0.2165	0.2	G	0.5143	1.105
15	rs34895054	49285001	C	0.2165	0.2	G	0.5143	1.105
2	rs2552010	36478634	G	0.4278	0.4083	A	0.5144	1.083
6	rs2187818	32395568	A	0.4278	0.4083	C	0.5144	1.083

19	rs812847	16855265	G	0.4278	0.4083	A	0.5144	1.083
20	rs6080991	18113486	A	0.4476	0.4275	G	0.5144	1.085
4	rs61748885	673772	A	0.2912	0.2733	G	0.5145	1.092
3	rs2727943	1897973	G	0.3969	0.4158	A	0.5146	0.9245
6	rs1150678	28142276	A	0.3995	0.4192	G	0.5146	0.9218
1	rs696390	195053488	G	0.4304	0.4108	A	0.5147	1.084
3	rs2028494	100004117	G	0.4304	0.4108	A	0.5147	1.084
8	rs12543404	95689312	A	0.4021	0.4225	G	0.5149	0.9191
6	rs3094573	29446632	C	0.4021	0.4217	A	0.515	0.9222
6	rs3128844	29459260	G	0.4021	0.4217	C	0.515	0.9222
7	rs10950821	20647015	C	0.4021	0.4217	G	0.515	0.9222
20	rs2073233	12874585	G	0.4021	0.4215	A	0.515	0.9227
2	rs4234047	224069989	A	0.433	0.4133	G	0.5151	1.084
3	rs35770315	42680000	A	0.1005	0.1133	G	0.5151	0.8743
4	rs6852952	21864975	A	0.1005	0.1142	G	0.5151	0.8671
10	rs12781609	134748331	A	0.433	0.4133	G	0.5151	1.084
13	rs1436708	55818627	A	0.4046	0.4249	G	0.5151	0.92
15	rs62623565	52521383	A	0.06959	0.08083	G	0.5151	0.8505
19	rs3745521	51170706	G	0.433	0.4133	A	0.5151	1.084
22	rs132549	29318724	A	0.1005	0.1133	G	0.5151	0.8743
2	rs11124220	240618118	A	0.1624	0.1483	G	0.5152	1.113
9	rs7023329	21816528	G	0.4046	0.425	A	0.5152	0.9195
15	rs3803466	75648650	A	0.1624	0.1483	G	0.5152	1.113
1	rs3795498	36807481	A	0.06959	0.08167	G	0.5153	0.841
2	rs10865035	100835734	A	0.4304	0.4117	G	0.5153	1.08
4	rs28404221	70505162	A	0.06959	0.08167	G	0.5153	0.841
4	rs147645578	113436546	A	0.06959	0.08167	G	0.5153	0.841
8	rs2272641	23294761	G	0.2655	0.2842	A	0.5153	0.9104
9	rs10760523	130776897	G	0.4304	0.4117	A	0.5153	1.08
13	rs1359790	80717156	A	0.2655	0.2842	G	0.5153	0.9104
18	rs79992793	10699087	C	0.06959	0.08167	G	0.5153	0.841
19	rs11669592	3318510	G	0.2912	0.2742	A	0.5153	1.088
4	rs9715724	8641696	G	0.125	0.1129	A	0.5154	1.123
6	rs7748069	112942161	A	0.4356	0.4158	C	0.5154	1.084
11	rs1387153	92673828	A	0.4356	0.4158	G	0.5154	1.084
11	rs10830956	92681013	A	0.4356	0.4158	G	0.5154	1.084
17	rs12939811	46607958	A	0.05928	0.05083	T	0.5154	1.177
4	rs7664889	77087704	A	0.2655	0.2833	C	0.5155	0.9141
22	rs5748014	19148721	C	0.2655	0.2833	A	0.5155	0.9141
4	rs10033556	32962030	A	0.433	0.4142	G	0.5156	1.08
5	rs17672542	75001582	G	0.4072	0.4275	A	0.5156	0.92
5	rs6899159	89469753	G	0.1624	0.1486	A	0.5156	1.111
19	rs7257948	8138054	A	0.433	0.4142	C	0.5156	1.08

4	rs442177	88030261	C	0.4381	0.4183	A	0.5157	1.084
5	rs34359734	120958334	G	0.4356	0.4165	A	0.5157	1.081
12	rs1468556	4873163	A	0.4381	0.4183	C	0.5157	1.084
4	rs1346145	79625240	G	0.4072	0.4267	A	0.5158	0.9231
5	rs814102	180199307	G	0.4072	0.4267	A	0.5158	0.9231
9	rs4879686	33343397	C	0.4072	0.4267	A	0.5158	0.9231
1	rs800292	196642233	A	0.4356	0.4167	G	0.5159	1.08
6	rs8589	49403282	G	0.4098	0.43	A	0.5159	0.9204
13	rs4000216	111341751	G	0.4098	0.43	A	0.5159	0.9204
6	rs4712556	20856703	A	0.4407	0.4208	G	0.516	1.085
6	rs2524050	31255541	G	0.1211	0.1083	A	0.516	1.134
6	rs2853925	31264922	G	0.1211	0.1083	A	0.516	1.134
6	rs364415	31273224	A	0.1211	0.1083	G	0.516	1.134
9	rs2236405	98211572	A	0.1211	0.1083	T	0.516	1.134
13	rs17079773	24598384	A	0.4407	0.4208	G	0.516	1.085
4	rs1391099	143373910	A	0.4381	0.4192	G	0.5162	1.081
8	rs1825512	119999531	A	0.4381	0.4192	G	0.5162	1.081
16	rs12325410	9675246	A	0.4381	0.4192	C	0.5162	1.081
18	rs4939827	46453463	A	0.2191	0.2025	G	0.5162	1.105
1	rs610100	181778329	A	0.4433	0.4233	C	0.5163	1.085
1	rs76249490	227152761	A	0.03866	0.03167	G	0.5163	1.23
1	rs2297411	227152778	C	0.03866	0.03167	A	0.5163	1.23
2	rs28382942	100029254	A	0.03866	0.03167	G	0.5163	1.23
3	rs144670843	42567463	G	0.03866	0.03167	A	0.5163	1.23
6	rs7762253	29676823	A	0.4433	0.4233	G	0.5163	1.085
6	rs3752579	112493889	T	0.03866	0.03167	A	0.5163	1.23
8	rs3210518	92097062	A	0.03866	0.03167	G	0.5163	1.23
9	rs76761697	107516849	A	0.03866	0.03167	T	0.5163	1.23
10	rs76895268	64416220	A	0.03866	0.03167	G	0.5163	1.23
17	rs184746576	8656662	A	0.03866	0.03167	G	0.5163	1.23
19	exm-IND19-5677	52084691	I	0.03866	0.03167	D	0.5163	1.23
21	rs1051393	34614255	A	0.4433	0.4233	C	0.5163	1.085
8	rs10106960	3333127	A	0.2938	0.2767	G	0.5164	1.088
8	rs11997548	64708102	C	0.2938	0.2767	A	0.5164	1.088
3	rs2700316	163617301	G	0.4124	0.4317	A	0.5165	0.9239
6	rs6926872	33843759	A	0.4407	0.4217	G	0.5165	1.081
5	rs1687503	68003767	A	0.4149	0.435	G	0.5166	0.9212
13	rs3742301	53422553	A	0.4459	0.4258	G	0.5166	1.085
2	rs13412879	191399378	C	0.1418	0.1567	G	0.5168	0.8891
13	rs77597251	97639880	D	0.08763	0.07667	I	0.5168	1.157
19	rs7245429	8159362	C	0.2964	0.2783	A	0.5168	1.092
21	rs3746843	30464866	G	0.08763	0.07667	A	0.5168	1.157
7	rs4717763	72825125	G	0.4149	0.4339	A	0.5169	0.9252

9	rs9299035	680540	A	0.4175	0.4375	C	0.5169	0.9216
19	rs3764656	362283	A	0.1624	0.1492	G	0.5169	1.106
12	rs141932661	124097763	A	0.03866	0.03177	G	0.517	1.225
1	rs368460	79709724	G	0.451	0.4308	A	0.5172	1.085
1	rs12046366	189022599	C	0.4485	0.4292	A	0.5172	1.081
10	rs4751015	130665155	G	0.4175	0.4367	A	0.5172	0.9247
11	rs7933807	7949791	A	0.4201	0.44	C	0.5172	0.922
11	rs7928451	7949860	G	0.4201	0.44	A	0.5172	0.922
11	rs10895547	103808152	A	0.451	0.4308	G	0.5172	1.085
18	rs13381941	19154143	A	0.4175	0.4367	G	0.5172	0.9247
22	rs1005640	20789074	A	0.4201	0.44	G	0.5172	0.922
1	rs1805110	92327045	A	0.451	0.4317	G	0.5175	1.082
8	rs17210452	74042887	G	0.2964	0.2792	A	0.5175	1.088
9	rs943926	31873011	A	0.4201	0.4392	G	0.5176	0.9251
3	rs1043261	53899276	A	0.1211	0.1092	G	0.5177	1.125
5	rs6895566	75306838	G	0.2706	0.2892	A	0.5177	0.9121
10	rs2154203	98212575	A	0.4536	0.4342	G	0.5177	1.082
2	rs6714273	4188698	G	0.4562	0.4367	A	0.5179	1.082
6	rs1925153	56102780	A	0.4227	0.4417	G	0.5179	0.9255
7	rs41859	69006876	A	0.2706	0.2883	G	0.5179	0.9158
10	rs7906685	36379503	C	0.4562	0.4367	A	0.5179	1.082
10	rs7088966	111261278	A	0.4227	0.4417	G	0.5179	0.9255
17	rs11546280	79671714	G	0.299	0.2808	A	0.5179	1.092
18	rs585187	58177124	A	0.299	0.2808	C	0.5179	1.092
20	rs6126559	36557276	A	0.4227	0.4417	G	0.5179	0.9255
11	rs79062509	65793121	A	0.1649	0.1508	G	0.518	1.112
3	rs7636435	125182246	A	0.4588	0.4392	G	0.5181	1.082
6	rs3757340	30921882	G	0.4588	0.4392	A	0.5181	1.082
6	rs12212418	30924024	A	0.4588	0.4392	G	0.5181	1.082
14	rs3784039	74489735	G	0.4588	0.4392	A	0.5181	1.082
17	rs17854013	8701799	A	0.4278	0.4475	C	0.5181	0.9232
2	rs10427255	146125523	G	0.4304	0.4508	A	0.5182	0.9205
8	rs2272662	145639726	A	0.4715	0.4516	G	0.5182	1.083
11	rs17121881	118081345	T	0.4304	0.4508	A	0.5182	0.9205
11	rs10750523	132886373	G	0.4613	0.4417	A	0.5184	1.083
14	rs11550452	24653523	C	0.4613	0.4417	G	0.5184	1.083
6	rs440454	31927342	A	0.299	0.2817	G	0.5185	1.088
6	rs419788	31928799	A	0.299	0.2817	G	0.5185	1.088
8	rs4734130	107096458	A	0.4562	0.4375	G	0.5185	1.079
16	rs2008344	3741562	G	0.4562	0.4375	A	0.5185	1.079
1	rs798042	117304100	A	0.4639	0.4442	G	0.5186	1.083
7	rs1602105	137902633	A	0.433	0.4525	G	0.5186	0.924
10	rs4979906	79444753	A	0.4588	0.44	G	0.5187	1.079

4	rs4956211	109723126	A	0.2732	0.2917	G	0.5188	0.9129
5	rs1867732	60936935	A	0.2732	0.2917	C	0.5188	0.9129
5	rs2116714	148191398	A	0.2216	0.2058	G	0.5188	1.099
6	rs878593	85795657	A	0.2732	0.2917	G	0.5188	0.9129
11	rs2132517	10791983	A	0.2216	0.2058	G	0.5188	1.099
2	rs11901499	142172028	A	0.04639	0.05583	G	0.5189	0.8227
7	rs6979998	70097525	G	0.4613	0.4425	A	0.5189	1.079
8	rs13281615	128355618	A	0.4691	0.4492	G	0.5189	1.083
9	rs4629935	77848630	A	0.4356	0.455	G	0.5189	0.9243
9	rs615189	87697154	G	0.4356	0.455	A	0.5189	0.9243
11	rs490458	117436190	G	0.4691	0.4492	A	0.5189	1.083
3	rs6444127	186143744	G	0.3015	0.2833	A	0.519	1.092
6	rs6907125	47646842	G	0.2732	0.2908	A	0.519	0.9166
6	rs17541107	47649265	A	0.2732	0.2908	T	0.519	0.9166
6	rs10807371	47649573	A	0.2732	0.2908	G	0.519	0.9166
6	rs10807372	47649574	G	0.2732	0.2908	A	0.519	0.9166
6	rs9381594	47649694	G	0.2732	0.2908	A	0.519	0.9166
8	rs16894311	90335076	C	0.4716	0.4516	A	0.519	1.084
9	rs3739510	133936571	C	0.4767	0.4575	G	0.519	1.08
1	rs3820198	53792651	C	0.4381	0.4575	A	0.5191	0.9247
3	rs1044240	105258861	G	0.04639	0.05667	A	0.5191	0.8099
3	rs114656923	128722685	A	0.04639	0.05667	G	0.5191	0.8099
3	rs3733008	186387779	G	0.04639	0.05667	A	0.5191	0.8099
6	rs1233397	29545715	G	0.4716	0.4517	A	0.5191	1.084
11	rs75758940	5758051	A	0.04639	0.05667	G	0.5191	0.8099
11	rs2243411	20124914	A	0.04639	0.05667	C	0.5191	0.8099
11	rs7124275	48162305	G	0.4381	0.4575	A	0.5191	0.9247
22	rs743920	29621128	G	0.4407	0.4608	C	0.5191	0.922
22	rs5996309	43714716	G	0.4407	0.4608	A	0.5191	0.922
1	rs2024764	184653521	A	0.4665	0.4475	G	0.5192	1.08
2	rs2175149	231877207	A	0.4665	0.4475	G	0.5192	1.08
6	rs2856997	32781776	C	0.4691	0.4499	A	0.5192	1.08
16	rs11645643	10524165	A	0.4665	0.4475	G	0.5192	1.08
16	rs13331233	80696071	G	0.4665	0.4475	A	0.5192	1.08
1	rs16841001	158725080	A	0.1031	0.1158	G	0.5193	0.8774
4	rs3828539	113505203	G	0.1031	0.1167	A	0.5193	0.8703
11	rs5006888	5373114	G	0.4742	0.4542	A	0.5193	1.084
12	rs4767535	117804875	A	0.4433	0.4633	G	0.5193	0.9223
16	rs78143373	109103	G	0.08763	0.0775	A	0.5193	1.143
20	rs4814423	15973949	A	0.4433	0.4633	G	0.5193	0.9223
2	rs1370229	128699833	A	0.4459	0.4658	G	0.5194	0.9227
6	rs6903939	126600533	A	0.4768	0.4567	C	0.5194	1.084
1	rs45487196	22974097	A	0.0232	0.0175	G	0.5195	1.333

1	rs146536550	78034049	G	0.0232	0.0175	C	0.5195	1.333
1	rs138426509	249144933	G	0.0232	0.0175	A	0.5195	1.333
3	rs191732065	77607147	A	0.0232	0.0175	C	0.5195	1.333
3	rs199933702	130300462	C	0.0232	0.0175	G	0.5195	1.333
4	rs11938093	75675841	A	0.0232	0.0175	T	0.5195	1.333
5	rs17648108	177831556	A	0.0232	0.0175	G	0.5195	1.333
6	rs29272	29618366	A	0.0232	0.0175	C	0.5195	1.333
6	rs29234	29624112	C	0.0232	0.0175	A	0.5195	1.333
9	rs1052690	86258685	C	0.0232	0.0175	A	0.5195	1.333
10	rs10999406	72326400	A	0.0232	0.0175	G	0.5195	1.333
11	rs4148619	17452500	A	0.0232	0.0175	G	0.5195	1.333
13	rs78829338	111121570	G	0.0232	0.0175	A	0.5195	1.333
14	exm1126423	99924868	A	0.0232	0.0175	G	0.5195	1.333
14	rs145982994	99924868	A	0.0232	0.0175	G	0.5195	1.333
14	rs2273801	100847437	G	0.0232	0.0175	A	0.5195	1.333
14	rs34331240	100847617	A	0.0232	0.0175	G	0.5195	1.333
17	rs150859200	76481692	A	0.0232	0.0175	G	0.5195	1.333
2	rs6544711	44000495	A	0.1649	0.1517	G	0.5196	1.105
4	rs1377210	109674116	G	0.4663	0.4867	A	0.5196	0.9217
5	rs3776331	142441794	A	0.3015	0.2842	G	0.5196	1.088
9	rs3750425	77377410	A	0.1649	0.1517	G	0.5196	1.105
13	rs9574371	36258899	G	0.4794	0.4592	A	0.5196	1.085
16	rs2288041	74921446	A	0.4433	0.4625	G	0.5196	0.9254
7	rs2110871	78080548	A	0.482	0.4617	G	0.5197	1.085
12	rs7305618	121402932	A	0.451	0.4708	G	0.5197	0.9236
16	rs4785755	90037828	G	0.1443	0.1592	A	0.5197	0.8911
5	rs369639	140230371	C	0.4459	0.4649	A	0.5198	0.926
6	rs2734574	31493888	T	0.451	0.4708	A	0.5198	0.9234
7	rs754554	24758818	A	0.4845	0.4642	C	0.5198	1.085
12	rs79705698	88508258	G	0.1985	0.215	A	0.5198	0.904
12	rs7953249	121403724	A	0.451	0.4708	G	0.5198	0.9234
9	rs4977749	21927327	G	0.4794	0.46	A	0.5199	1.081
11	rs689369	78437182	A	0.1443	0.16	G	0.5199	0.8855
11	rs1619714	115552918	A	0.4896	0.4708	G	0.5199	1.078
15	rs2616881	54212245	G	0.4794	0.46	A	0.5199	1.081
22	rs28372448	50350971	A	0.1443	0.16	G	0.5199	0.8855
6	rs2274305	24291203	A	0.2216	0.2067	G	0.52	1.093
10	rs1223306	95380790	G	0.4562	0.4767	A	0.52	0.921
11	rs4757879	20020097	A	0.4485	0.4675	C	0.52	0.9261
19	rs1344572	9070194	A	0.1237	0.1108	G	0.52	1.133
19	rs2287922	49232226	A	0.0232	0.01756	G	0.52	1.329
1	rs17596	223905532	G	0.4588	0.4792	A	0.5201	0.9213
5	rs4469198	18919848	A	0.4562	0.4758	G	0.5201	0.9241

8	rs12680659	77402921	A	0.4588	0.4792	G	0.5201	0.9213
9	rs2025175	35905966	A	0.4562	0.4758	G	0.5201	0.9241
17	rs399605	3440229	A	0.4897	0.4699	C	0.5201	1.082
18	rs9807633	14543057	C	0.4588	0.4792	A	0.5201	0.9213
19	rs2287679	33600764	A	0.4897	0.4699	G	0.5201	1.082
1	rs2056900	47607785	G	0.4871	0.4675	A	0.5202	1.082
3	rs11721025	170171738	G	0.2758	0.2933	A	0.5202	0.9173
4	rs2866413	103557077	A	0.4897	0.47	G	0.5202	1.082
6	rs602903	137941227	A	0.2758	0.2933	G	0.5202	0.9173
8	rs4737753	54701811	A	0.451	0.47	G	0.5202	0.9265
9	rs10812505	26978170	A	0.4613	0.4817	G	0.5202	0.9217
9	rs3814538	109691676	G	0.451	0.47	A	0.5202	0.9265
17	rs6503018	7292107	G	0.3041	0.2863	A	0.5202	1.089
8	rs34589581	99364717	G	0.4639	0.4842	A	0.5203	0.922
9	rs2251760	86811147	A	0.4923	0.4725	G	0.5203	1.082
10	rs7079002	34714686	G	0.4639	0.4842	A	0.5203	0.922
13	rs946837	101287340	G	0.4923	0.4725	A	0.5203	1.082
15	rs3809482	43661802	A	0.4923	0.4725	G	0.5203	1.082
2	rs13045	88895123	A	0.4536	0.4725	G	0.5204	0.9268
4	rs6814124	150554591	A	0.4974	0.4775	G	0.5204	1.083
6	rs1570534	1901495	A	0.4665	0.4867	G	0.5204	0.9223
6	rs614549	31840625	G	0.4665	0.4867	A	0.5204	0.9223
9	rs4745062	73784264	A	0.4974	0.4775	G	0.5204	1.083
9	rs6478108	117558703	G	0.4974	0.4775	A	0.5204	1.083
13	rs4883918	73350079	A	0.5026	0.4824	G	0.5204	1.084
15	rs56170748	50932357	G	0.4948	0.475	A	0.5204	1.083
16	rs7188213	26519394	A	0.4948	0.475	G	0.5204	1.083
18	rs35739383	8783835	A	0.4613	0.4808	G	0.5204	0.9247
18	rs10502736	37970495	A	0.4665	0.4867	C	0.5204	0.9223
18	rs12457157	53296927	G	0.4665	0.4867	A	0.5204	0.9223
19	rs36686	17922795	A	0.5	0.48	G	0.5204	1.083
21	rs999106	32968981	A	0.4948	0.475	C	0.5204	1.083
1	rs12034383	207803595	A	0.4691	0.4892	G	0.5205	0.9226
3	rs11928487	106404147	A	0.5	0.48	C	0.5205	1.083
3	rs1867504	133410661	A	0.5	0.48	G	0.5205	1.083
3	rs1523288	165280209	G	0.4691	0.4892	A	0.5205	0.9226
4	rs10030708	68995529	A	0.4691	0.4892	G	0.5205	0.9226
6	rs2844762	30236754	A	0.5	0.48	G	0.5205	1.083
6	rs1158763	30240074	A	0.5	0.48	G	0.5205	1.083
6	rs968909	30244579	G	0.5	0.48	A	0.5205	1.083
6	rs261950	30269561	T	0.5	0.48	A	0.5205	1.083
6	rs9389946	142022955	A	0.4716	0.4917	G	0.5205	0.9229
7	rs4731907	132526350	G	0.5	0.48	A	0.5205	1.083

9	rs7865059	105412545	A	0.4691	0.4892	G	0.5205	0.9226
11	rs340960	22444436	G	0.4716	0.4917	A	0.5205	0.9229
12	rs10880835	46079245	A	0.4639	0.4833	C	0.5205	0.9251
12	rs7962918	129288534	A	0.4691	0.4892	G	0.5205	0.9226
14	rs2241621	81737076	C	0.5	0.48	A	0.5205	1.083
15	rs2043085	58680954	G	0.4639	0.4833	A	0.5205	0.9251
18	rs11660043	43425494	G	0.4742	0.4942	A	0.5205	0.9233
21	rs468192	31224266	A	0.5	0.48	G	0.5205	1.083
21	rs468413	31233994	G	0.5	0.48	A	0.5205	1.083
3	rs3774729	63982082	A	0.4845	0.505	G	0.5206	0.9214
4	rs6844368	79439935	A	0.5052	0.485	C	0.5206	1.084
4	rs931606	79443850	G	0.5052	0.485	A	0.5206	1.084
7	rs11975146	25563961	A	0.4845	0.505	G	0.5206	0.9214
7	rs7803014	57348834	G	0.5052	0.485	A	0.5206	1.084
7	rs7783895	79159240	G	0.2242	0.2083	A	0.5206	1.098
8	rs75381567	18207413	C	0.3041	0.2867	A	0.5206	1.088
8	rs13257322	65242166	A	0.4562	0.475	G	0.5206	0.9272
11	rs1004446	2170143	A	0.3041	0.2867	G	0.5206	1.088
18	rs3809929	77634361	G	0.4897	0.4708	A	0.5206	1.079
5	rs479632	134364518	C	0.4871	0.4683	G	0.5207	1.078
5	rs31198	134372685	A	0.4871	0.4683	G	0.5207	1.078
6	rs9468344	28309569	C	0.4897	0.4708	A	0.5207	1.078
10	rs1926736	17891705	A	0.4897	0.4708	G	0.5207	1.078
10	rs4918948	97316040	A	0.4794	0.4992	G	0.5207	0.9239
12	rs7966651	115371958	G	0.4665	0.4858	A	0.5207	0.9254
14	rs7143633	21549893	C	0.4768	0.4967	G	0.5207	0.9236
16	rs2071950	774692	A	0.1675	0.1533	G	0.5207	1.111
19	rs35352238	15131330	G	0.4897	0.4708	A	0.5207	1.078
22	rs5756506	37467392	C	0.4794	0.4992	G	0.5207	0.9239
1	rs873233	153226983	G	0.4948	0.4758	A	0.5208	1.079
1	rs7536825	245703284	A	0.4923	0.4733	G	0.5208	1.079
2	rs6733871	80529956	A	0.4923	0.4733	G	0.5208	1.079
2	rs6732434	182901257	G	0.5129	0.4933	A	0.5208	1.081
5	rs725127	64099769	G	0.4691	0.4883	A	0.5208	0.9257
5	rs30187	96124330	G	0.5129	0.4933	A	0.5208	1.081
5	rs27434	96129512	G	0.5129	0.4933	A	0.5208	1.081
7	rs10487524	144841531	A	0.4974	0.4783	G	0.5208	1.079
8	rs7017212	10022938	A	0.4948	0.4758	C	0.5208	1.079
16	rs28631056	8088550	C	0.4691	0.4883	A	0.5208	0.9257
1	rs151336893	43308451	G	0.06186	0.0525	A	0.5209	1.19
3	rs2272118	10049287	G	0.2242	0.2087	A	0.5209	1.096
7	rs10248935	51650669	C	0.5103	0.4908	A	0.5209	1.081
9	rs1331220	90648222	A	0.4716	0.4908	G	0.5209	0.926

11	rs11221912	129830796	A	0.5026	0.4833	G	0.5209	1.08
14	rs421262	88401213	A	0.06186	0.0525	G	0.5209	1.19
15	rs1801591	76578762	A	0.06186	0.0525	G	0.5209	1.19
16	rs3190321	11773662	C	0.4845	0.5034	G	0.5209	0.9274
8	rs3802264	123966085	A	0.2784	0.2967	G	0.521	0.9144
10	rs3740370	95259979	C	0.2784	0.2967	G	0.521	0.9144
16	rs9940346	64716851	G	0.4742	0.4933	A	0.521	0.9263
22	rs738241	46961271	G	0.2784	0.2967	A	0.521	0.9144
7	rs38523	28039797	A	0.4768	0.4958	G	0.5211	0.9266
1	rs12734925	157367179	A	0.4794	0.4983	G	0.5212	0.927
3	rs1042636	122003769	A	0.482	0.5008	G	0.5212	0.9273
6	rs241438	32797620	A	0.482	0.5008	G	0.5212	0.9273
6	rs1980493	32363215	G	0.07216	0.08333	A	0.5213	0.8556
7	rs10226709	14797634	A	0.4845	0.5033	C	0.5213	0.9275
15	rs16961728	49148192	C	0.07216	0.08333	A	0.5213	0.8556
6	rs3800544	170058374	A	0.1237	0.1117	G	0.5216	1.123
11	rs548181	125461709	A	0.1237	0.1117	G	0.5216	1.123
1	rs10127622	151468060	A	0.201	0.2175	C	0.5217	0.9052
2	rs1367248	124998364	A	0.201	0.2175	C	0.5217	0.9052
8	rs7386782	133053309	A	0.2242	0.2092	G	0.5217	1.093
8	rs7386783	133053318	A	0.2242	0.2092	G	0.5217	1.093
21	rs2836878	40465534	A	0.201	0.2175	G	0.5217	0.9052
1	rs3748569	156351699	G	0.3093	0.2908	A	0.5221	1.092
17	rs8066857	70696103	A	0.3093	0.2908	G	0.5221	1.092
7	rs35508364	5396715	G	0.1477	0.1622	A	0.5222	0.895
7	rs10227141	76019590	A	0.1675	0.1542	G	0.5222	1.104
1	rs17027633	111962256	A	0.2268	0.2108	C	0.5223	1.098
3	rs9859433	149362592	G	0.2809	0.2983	A	0.5224	0.9189
3	rs9846480	138025396	A	0.1469	0.1617	G	0.5225	0.893
17	rs228757	42164885	C	0.1469	0.1617	G	0.5225	0.893
1	rs1217382	114423653	A	0.09021	0.07917	G	0.5227	1.153
8	rs1601159	24003241	G	0.09021	0.07917	A	0.5227	1.153
16	rs77595156	28488943	G	0.09021	0.07917	A	0.5227	1.153
19	rs7249094	8672000	G	0.09021	0.07917	A	0.5227	1.153
10	rs7089424	63752159	C	0.2835	0.3025	A	0.5232	0.9124
10	rs12917	131506283	A	0.1057	0.1192	G	0.5233	0.8734
3	rs34550074	133666209	A	0.2268	0.2117	G	0.5234	1.092
6	rs1264432	30562021	G	0.3093	0.2925	A	0.5235	1.083
8	rs11780023	141992778	G	0.3093	0.2925	A	0.5235	1.083
10	rs7092999	102375258	G	0.3093	0.2925	A	0.5235	1.083
11	rs2234451	5529920	G	0.2036	0.2208	C	0.5235	0.9021
11	rs2466620	124189306	A	0.2036	0.2208	G	0.5235	0.9021
11	rs2512219	124189729	A	0.2036	0.2208	G	0.5235	0.9021

12	rs72640150	29648364	A	0.2036	0.2208	G	0.5235	0.9021
20	rs2075960	43547677	C	0.2036	0.2208	A	0.5235	0.9021
1	rs2297911	200959302	A	0.3119	0.2942	G	0.5236	1.087
12	rs11179745	74089396	A	0.3119	0.2942	G	0.5236	1.087
3	rs79845192	130174433	G	0.03866	0.0325	A	0.5239	1.197
4	rs991316	100322445	G	0.03866	0.0325	A	0.5239	1.197
14	rs116261083	23519131	A	0.03866	0.0325	G	0.5239	1.197
20	rs2273075	61386232	A	0.03866	0.0325	G	0.5239	1.197
1	rs2272792	166829522	A	0.1263	0.1133	G	0.524	1.131
2	rs1542087	88387557	C	0.02835	0.03667	A	0.524	0.7666
5	rs2287932	75969360	A	0.02835	0.03667	C	0.524	0.7666
6	rs9471969	42906384	A	0.02835	0.03667	C	0.524	0.7666
7	rs73402889	100678527	A	0.02835	0.03667	C	0.524	0.7666
8	rs3735726	28321247	A	0.02835	0.03667	G	0.524	0.7666
10	rs17108378	99502897	A	0.02835	0.03667	G	0.524	0.7666
17	rs17843021	39116728	A	0.1263	0.1133	G	0.524	1.131
17	rs9891620	72351024	G	0.02835	0.03673	A	0.524	0.7653
21	rs2230033	39671476	A	0.2876	0.3055	G	0.524	0.9176
22	rs61995890	43936219	A	0.02835	0.03667	G	0.524	0.7666
4	rs2725264	89026109	G	0.2294	0.2133	A	0.5241	1.098
13	rs3742262	42442546	G	0.2294	0.2133	A	0.5241	1.098
6	rs28362680	32370816	A	0.2861	0.305	G	0.5242	0.9131
6	rs10947262	32373312	A	0.2861	0.305	G	0.5242	0.9131
6	rs1230345	112382313	A	0.2861	0.3042	C	0.5242	0.9167
7	rs11762014	97852399	A	0.2861	0.3042	G	0.5242	0.9167
18	rs892586	44560429	C	0.3144	0.2963	A	0.5243	1.089
2	rs1863703	219544388	G	0.06186	0.05333	A	0.5244	1.17
2	rs16859180	219553468	A	0.06186	0.05333	G	0.5244	1.17
2	rs12993599	219563602	A	0.06186	0.05333	G	0.5244	1.17
5	rs1486939	1916139	A	0.06186	0.05333	C	0.5244	1.17
17	rs2907373	66533655	A	0.06186	0.05333	G	0.5244	1.17
6	rs1592410	29483968	A	0.3144	0.2967	G	0.5245	1.087
18	rs2571028	44561100	C	0.3144	0.2967	G	0.5245	1.087
19	rs2072490	18257750	G	0.3144	0.2967	A	0.5245	1.087
1	rs1217401	114438951	G	0.09021	0.08	A	0.5251	1.14
1	rs1217397	114447565	A	0.09021	0.08	G	0.5251	1.14
10	rs76052898	5435823	G	0.09021	0.08	A	0.5251	1.14
11	rs503341	63587313	G	0.2294	0.2142	A	0.5251	1.092
7	rs4729759	101536886	G	0.1495	0.1642	A	0.5252	0.8948
14	rs729184	103576415	A	0.2887	0.3075	G	0.5252	0.9139
20	rs6140742	8819112	C	0.2887	0.3067	G	0.5252	0.9175
20	rs6101991	39082308	G	0.1495	0.1642	A	0.5252	0.8948
3	rs3821396	21706369	A	0.2062	0.2225	G	0.5253	0.9076

5	rs2544809	176885499	G	0.2062	0.2225	A	0.5253	0.9076
7	rs2251220	138601826	A	0.2062	0.2225	G	0.5253	0.9076
7	rs2774960	138602417	A	0.2062	0.2225	G	0.5253	0.9076
7	rs2774962	138602523	G	0.2062	0.2225	A	0.5253	0.9076
14	rs2150214	45822243	A	0.1495	0.165	G	0.5253	0.8894
19	rs61736479	52089982	G	0.1495	0.165	C	0.5253	0.8894
19	rs527025	56703248	A	0.3144	0.2975	G	0.5253	1.083
1	rs859398	175375802	A	0.1263	0.1142	G	0.5255	1.122
9	rs1180116	72897434	G	0.1263	0.1142	A	0.5255	1.122
15	rs79477685	102389423	G	0.1263	0.1142	C	0.5255	1.122
18	rs3764507	72114490	A	0.1263	0.1142	G	0.5255	1.122
2	rs842636	61091950	A	0.1727	0.1583	G	0.526	1.11
4	rs79383654	113565835	A	0.1727	0.1583	G	0.526	1.11
17	exm1364606	78341776	A	0.1727	0.1583	G	0.526	1.11
17	rs77506504	78341776	A	0.1727	0.1583	G	0.526	1.11
11	rs11223168	100061865	G	0.02835	0.0375	A	0.5261	0.7489
14	rs74825213	61517256	A	0.02835	0.0375	G	0.5261	0.7489
6	rs3763313	32376471	C	0.2088	0.2258	A	0.527	0.9045
12	rs1669412	11338669	A	0.2088	0.2258	G	0.527	0.9045
12	rs1451772	11338750	G	0.2088	0.2258	A	0.527	0.9045
12	rs10879571	73551552	G	0.3196	0.3025	A	0.5271	1.083
21	rs2223043	16438793	A	0.2938	0.3125	G	0.5271	0.9153
3	rs3772165	43602803	G	0.1082	0.1217	A	0.5272	0.8763
13	rs4907604	113942301	G	0.1082	0.1217	A	0.5272	0.8763
16	rs7190199	57101340	G	0.1082	0.1217	A	0.5272	0.8763
16	rs7185320	57101373	G	0.1082	0.1217	A	0.5272	0.8763
1	rs1799822	53679229	G	0.07474	0.08583	A	0.5273	0.8603
2	rs702873	61081542	A	0.1727	0.1592	G	0.5273	1.103
4	rs62317770	113568544	A	0.1727	0.1592	G	0.5273	1.103
6	rs2269704	30656953	A	0.07474	0.08667	G	0.5273	0.8513
6	rs75355880	30671760	A	0.07474	0.08667	G	0.5273	0.8513
6	rs80087328	30671969	G	0.07474	0.08667	C	0.5273	0.8513
6	rs11759988	31432861	G	0.07513	0.08667	A	0.5273	0.8561
11	rs11545725	1902793	A	0.07474	0.08667	T	0.5273	0.8513
19	rs199822445	8398961	C	0.2938	0.3114	A	0.5273	0.9202
21	rs12483205	38740824	G	0.07474	0.08667	A	0.5273	0.8513
1	rs4646491	47280884	A	0.3222	0.3042	G	0.5274	1.087
18	rs3018362	60082093	G	0.3222	0.3042	A	0.5274	1.087
11	rs16918105	92533057	G	0.1082	0.1208	A	0.5275	0.8832
1	rs12084264	186363119	C	0.1289	0.1158	G	0.5278	1.129
4	rs2271570	130030644	A	0.1289	0.1158	G	0.5278	1.129
6	rs2395163	32387809	G	0.1521	0.1667	A	0.5279	0.8967
6	rs1049056	32634369	A	0.1521	0.1667	C	0.5279	0.8967

3	rs6280	113890815	G	0.2964	0.315	A	0.528	0.916
4	rs4253301	187173012	C	0.04897	0.05833	A	0.528	0.8312
8	rs141555196	101237471	A	0.04897	0.05833	G	0.528	0.8312
12	rs7969151	54159277	A	0.04897	0.05833	G	0.528	0.8312
15	rs1353576	86892638	G	0.2964	0.315	A	0.528	0.916
5	rs35983033	140048707	G	0.04897	0.05917	A	0.5281	0.8188
7	rs1052200	29070320	A	0.2964	0.3142	G	0.5282	0.9196
9	rs1953126	123640500	A	0.2964	0.3142	G	0.5282	0.9196
3	rs11718898	12848822	G	0.2345	0.2192	A	0.5283	1.092
3	rs6798365	186177610	A	0.3247	0.3067	G	0.5283	1.087
8	rs4876717	118320851	G	0.3247	0.3067	A	0.5283	1.087
9	rs10868791	91154126	A	0.3247	0.3067	G	0.5283	1.087
17	rs11078738	8167600	G	0.2964	0.3139	A	0.5283	0.9209
6	rs3892710	32682862	A	0.09278	0.08167	G	0.5284	1.15
16	rs7140	28995757	C	0.09278	0.08167	A	0.5284	1.15
5	rs2303650	94826655	A	0.1753	0.1608	C	0.5285	1.109
14	rs2275554	96871104	A	0.1753	0.1608	G	0.5285	1.109
16	rs3810818	4432029	A	0.1753	0.1608	C	0.5285	1.109
5	rs10066063	32090294	A	0.2113	0.2275	G	0.5288	0.9099
6	rs2023472	30075864	A	0.2113	0.2275	G	0.5288	0.9099
12	rs7135018	11150240	G	0.2113	0.2275	A	0.5288	0.9099
3	rs4875	158320597	G	0.3247	0.3075	A	0.5289	1.083
9	rs12347453	78724005	A	0.299	0.3175	C	0.529	0.9167
16	rs9806942	320759	A	0.299	0.3175	G	0.529	0.9167
3	rs1052618	136574501	A	0.1289	0.1167	G	0.5292	1.12
4	rs57655409	42895423	A	0.299	0.3167	G	0.5292	0.9203
7	rs8188510	62177444	A	0.3273	0.3092	G	0.5292	1.087
9	rs10858293	137809685	A	0.1289	0.1167	C	0.5292	1.12
10	exm2249015	105363289	G	0.1289	0.1167	A	0.5292	1.12
10	rs3740471	105363289	G	0.1289	0.1167	A	0.5292	1.12
12	rs10748128	69827658	C	0.299	0.3167	A	0.5292	0.9203
14	rs10143866	52884355	G	0.1289	0.1167	A	0.5292	1.12
16	rs1231123	3293922	T	0.3273	0.3092	A	0.5292	1.087
22	rs8190315	18226764	G	0.1289	0.1167	A	0.5292	1.12
6	rs6455484	168506045	C	0.06443	0.055	A	0.5297	1.183
8	rs7833909	52366300	G	0.06443	0.055	A	0.5297	1.183
18	rs141224997	33234684	C	0.06443	0.055	A	0.5297	1.183
19	rs706761	17025292	G	0.3273	0.31	A	0.5297	1.083
2	rs2072525	37035945	A	0.1753	0.1617	C	0.5298	1.102
9	rs4741453	15085778	A	0.1753	0.1617	G	0.5298	1.102
9	rs1537415	138529722	C	0.1753	0.1617	G	0.5298	1.102
13	rs2260159	114154419	A	0.1753	0.1617	G	0.5298	1.102
1	rs11162867	80386090	A	0.3015	0.32	G	0.5299	0.9174

9	rs2782931	114850190	A	0.2371	0.2217	G	0.5299	1.091
13	rs7140044	25029295	A	0.3015	0.32	G	0.5299	0.9174
22	rs1557553	44760984	A	0.3015	0.32	G	0.5299	0.9174
2	rs16984239	18234446	A	0.3015	0.3192	C	0.5301	0.921
2	rs2310173	102663628	A	0.3015	0.3192	C	0.5301	0.921
8	rs1133076	134125682	A	0.3015	0.3192	G	0.5301	0.921
5	rs34899	95091201	A	0.2139	0.2308	G	0.5303	0.9068
1	rs147131452	26142081	A	0.0232	0.01833	G	0.5304	1.272
1	rs79085598	26212261	A	0.0232	0.01833	G	0.5304	1.272
2	rs2305462	173895285	A	0.1546	0.17	G	0.5304	0.8931
2	rs117603872	241463461	G	0.0232	0.01833	A	0.5304	1.272
3	rs2650951	122368347	A	0.0232	0.01833	G	0.5304	1.272
5	rs17839323	21842436	G	0.0232	0.01833	A	0.5304	1.272
6	rs148650984	43306602	G	0.0232	0.01833	A	0.5304	1.272
6	rs62622418	46801219	A	0.0232	0.01833	G	0.5304	1.272
8	rs11984691	2800096	A	0.0232	0.01833	G	0.5304	1.272
8	rs191614050	145094836	C	0.0232	0.01833	G	0.5304	1.272
10	rs182928199	31137496	G	0.0232	0.01833	A	0.5304	1.272
10	rs151015331	124753489	A	0.0232	0.01833	G	0.5304	1.272
10	rs3902963	126031899	A	0.1546	0.17	G	0.5304	0.8931
11	rs3729989	47370041	G	0.0232	0.01833	A	0.5304	1.272
12	rs56207115	52994896	G	0.0232	0.01833	A	0.5304	1.272
17	rs201631215	2279155	A	0.0232	0.01833	G	0.5304	1.272
17	rs142596676	37829778	G	0.0232	0.01833	A	0.5304	1.272
17	rs16948048	47440466	G	0.1546	0.17	A	0.5304	0.8931
19	rs117238101	47844955	A	0.0232	0.01833	G	0.5304	1.272
19	rs141628956	49878082	A	0.0232	0.01833	G	0.5304	1.272
19	rs2240040	57956186	G	0.0232	0.01833	A	0.5304	1.272
20	rs117387390	18395153	A	0.0232	0.01833	G	0.5304	1.272
20	rs2427284	60900481	A	0.0232	0.01833	G	0.5304	1.272
22	rs34214100	41077864	A	0.0232	0.01833	G	0.5304	1.272
22	rs4823561	46929692	A	0.0232	0.01833	G	0.5304	1.272
1	rs12130792	161993192	G	0.1546	0.1692	A	0.5305	0.8984
2	rs7561528	127889637	A	0.09278	0.0825	G	0.5305	1.137
3	rs77803411	52551973	A	0.09278	0.0825	G	0.5305	1.137
5	rs459552	112176756	A	0.09278	0.0825	T	0.5305	1.137
12	rs2658658	52788928	A	0.09278	0.0825	G	0.5305	1.137
12	rs3782886	112110489	G	0.1546	0.1692	A	0.5305	0.8984
1	rs149869952	160917792	A	0.04124	0.03417	G	0.5306	1.216
4	rs2302469	15688604	G	0.04124	0.03417	C	0.5306	1.216
4	rs2302465	15709192	A	0.04124	0.03417	G	0.5306	1.216
11	rs12417164	4870269	A	0.04124	0.03417	T	0.5306	1.216
11	rs76688946	57886435	A	0.04124	0.03417	C	0.5306	1.216

15	rs950169	84706461	A	0.04124	0.03417	G	0.5306	1.216
14	rs137914270	92454649	G	0.0232	0.01836	A	0.5308	1.269
16	rs7201282	51939435	G	0.3325	0.3142	A	0.5309	1.087
19	rs11086080	17927041	A	0.3325	0.3142	G	0.5309	1.087
1	rs2048156	69441920	A	0.1778	0.1633	C	0.531	1.108
1	rs11118448	220052720	A	0.1778	0.1633	G	0.531	1.108
10	rs11599750	101805442	A	0.1778	0.1633	G	0.531	1.108
11	rs1939927	123753937	A	0.1797	0.1653	G	0.531	1.106
13	rs9577229	113973832	A	0.1108	0.1242	G	0.531	0.8792
1	rs1518111	206944645	G	0.3041	0.3217	A	0.5311	0.9216
5	rs246105	108672946	G	0.3325	0.3147	A	0.5311	1.085
6	rs4897193	127208390	G	0.3041	0.3217	A	0.5311	0.9216
11	rs10839658	6942476	A	0.3041	0.3217	C	0.5311	0.9216
14	rs1950588	27348573	A	0.3041	0.3217	C	0.5311	0.9216
17	rs144588703	80193928	A	0.0232	0.01839	G	0.5312	1.267
7	rs2302339	30795288	G	0.1289	0.1175	A	0.5313	1.111
1	rs9887913	55462380	G	0.3325	0.315	A	0.5314	1.083
1	rs11120301	214510115	C	0.3325	0.315	A	0.5314	1.083
17	rs2233369	47293906	A	0.1108	0.125	G	0.5315	0.8725
3	rs12491503	165706855	A	0.3067	0.325	G	0.5317	0.9188
6	rs1075496	30658239	A	0.3067	0.325	C	0.5317	0.9188
10	rs515910	105966404	G	0.3351	0.3167	A	0.5317	1.087
12	rs1284879	113553481	G	0.2176	0.2336	A	0.5317	0.9125
1	rs41268336	16069525	A	0.01031	0.0075	G	0.5319	1.378
1	rs140780438	24485564	A	0.01031	0.0075	G	0.5319	1.378
1	rs199752860	171621718	C	0.01031	0.0075	G	0.5319	1.378
1	rs201071031	176863877	A	0.01031	0.0075	G	0.5319	1.378
1	rs201522628	200017566	A	0.01031	0.0075	G	0.5319	1.378
1	rs117940365	222717027	A	0.01031	0.0075	G	0.5319	1.378
2	rs72797147	31420143	A	0.01031	0.0075	G	0.5319	1.378
2	rs150401869	167055164	G	0.01031	0.0075	A	0.5319	1.378
2	rs117360413	167759994	G	0.01031	0.0075	A	0.5319	1.378
2	rs72648939	179597259	G	0.01031	0.0075	A	0.5319	1.378
2	rs117267701	201437335	A	0.01031	0.0075	G	0.5319	1.378
2	rs2066527	217315787	G	0.01031	0.0075	A	0.5319	1.378
3	rs188887061	47163843	A	0.01031	0.0075	G	0.5319	1.378
3	rs61729450	52360318	G	0.01031	0.0075	A	0.5319	1.378
3	rs61729402	52366368	G	0.01031	0.0075	A	0.5319	1.378
3	rs146811566	150931197	C	0.01031	0.0075	G	0.5319	1.378
4	rs141737199	2951669	G	0.01031	0.0075	C	0.5319	1.378
4	rs2581423	42020142	A	0.01031	0.0075	G	0.5319	1.378
4	rs142137451	114278014	A	0.01031	0.0075	G	0.5319	1.378
4	rs147508531	121957565	A	0.01031	0.0075	C	0.5319	1.378

4	rs726575	187539323	A	0.01031	0.0075	G	0.5319	1.378
5	rs77464125	38942984	A	0.01031	0.0075	G	0.5319	1.378
5	rs3812031	40852711	T	0.01031	0.0075	A	0.5319	1.378
5	rs35548075	110782456	A	0.01031	0.0075	G	0.5319	1.378
5	rs185149793	148427475	A	0.01031	0.0075	G	0.5319	1.378
5	rs61734664	159992585	A	0.01031	0.0075	G	0.5319	1.378
6	rs116120266	31592275	A	0.01031	0.0075	G	0.5319	1.378
6	rs181701991	39847198	A	0.01031	0.0075	G	0.5319	1.378
6	rs117777526	88768503	A	0.01031	0.0075	G	0.5319	1.378
6	rs35341741	151670690	A	0.01031	0.0075	G	0.5319	1.378
6	rs13212161	151672285	G	0.01031	0.0075	A	0.5319	1.378
6	rs12201388	151673589	A	0.01031	0.0075	G	0.5319	1.378
6	rs34713284	151674270	A	0.01031	0.0075	G	0.5319	1.378
6	rs6917747	160402705	A	0.01031	0.0075	G	0.5319	1.378
7	rs192989152	21599322	A	0.01031	0.0075	G	0.5319	1.378
7	rs200954020	87479214	A	0.01031	0.0075	G	0.5319	1.378
7	rs4987682	142569596	G	0.01031	0.0075	A	0.5319	1.378
7	rs4987667	142572908	G	0.01031	0.0075	A	0.5319	1.378
7	rs147792476	143054020	A	0.01031	0.0075	G	0.5319	1.378
7	rs3737258	158581070	A	0.01031	0.0075	G	0.5319	1.378
8	rs142315341	30700014	G	0.01031	0.0075	A	0.5319	1.378
8	rs149104030	70674017	G	0.01031	0.0075	A	0.5319	1.378
8	rs7004867	145114844	A	0.01031	0.0075	G	0.5319	1.378
9	rs28374506	711396	G	0.01031	0.0075	C	0.5319	1.378
9	rs141581125	18889583	A	0.01031	0.0075	G	0.5319	1.378
9	rs25695	74349846	A	0.01031	0.0075	T	0.5319	1.378
9	rs25688	74360234	A	0.01031	0.0075	G	0.5319	1.378
9	rs137871340	114090184	A	0.01031	0.0075	G	0.5319	1.378
9	rs143274946	136199421	A	0.01031	0.0075	G	0.5319	1.378
10	rs141897664	124458854	G	0.01031	0.0075	A	0.5319	1.378
11	rs147713756	5536386	G	0.01031	0.0075	A	0.5319	1.378
11	rs141059768	55798421	G	0.01031	0.0075	A	0.5319	1.378
11	rs202156360	56043790	A	0.01031	0.0075	C	0.5319	1.378
11	rs192749813	56258737	A	0.01031	0.0075	G	0.5319	1.378
11	rs200756719	120139928	A	0.01031	0.0075	G	0.5319	1.378
12	rs28919870	2968078	A	0.01031	0.0075	G	0.5319	1.378
12	rs200900846	4714247	A	0.01031	0.0075	T	0.5319	1.378
12	rs150585672	22796739	A	0.01031	0.0075	G	0.5319	1.378
12	rs143885257	30887977	G	0.01031	0.0075	A	0.5319	1.378
12	rs10878307	40671989	G	0.01031	0.0075	A	0.5319	1.378
12	rs77018758	40702269	A	0.01031	0.0075	C	0.5319	1.378
12	rs78315631	55688817	G	0.01031	0.0075	A	0.5319	1.378
13	rs79538375	32914277	G	0.01031	0.0075	A	0.5319	1.378

13	rs11619501	100623882	A	0.01031	0.0075	G	0.5319	1.378
13	rs201426733	111158815	A	0.01031	0.0075	G	0.5319	1.378
14	rs148485154	24607842	A	0.01031	0.0075	G	0.5319	1.378
14	rs117295933	45403699	A	0.01031	0.0075	C	0.5319	1.378
14	rs78246426	77914853	A	0.01031	0.0075	G	0.5319	1.378
15	rs77122016	34546655	A	0.01031	0.0075	G	0.5319	1.378
15	rs143973489	43574114	G	0.01031	0.0075	A	0.5319	1.378
15	rs117612527	51293344	A	0.01031	0.0075	C	0.5319	1.378
15	rs145117610	82575312	C	0.01031	0.0075	A	0.5319	1.378
15	rs143117049	89453152	G	0.01031	0.0075	A	0.5319	1.378
16	rs190231056	70712285	G	0.01031	0.0075	A	0.5319	1.378
17	rs142067676	4646747	A	0.01031	0.0075	G	0.5319	1.378
17	rs141917632	17907714	G	0.01031	0.0075	A	0.5319	1.378
17	rs146051850	29552200	G	0.01031	0.0075	A	0.5319	1.378
17	rs199908433	47247085	A	0.01031	0.0075	G	0.5319	1.378
17	rs148801948	71244668	A	0.01031	0.0075	G	0.5319	1.378
19	rs184916429	3760241	A	0.01031	0.0075	G	0.5319	1.378
19	rs202215753	10444149	A	0.01031	0.0075	C	0.5319	1.378
19	rs143071621	11917484	A	0.01031	0.0075	G	0.5319	1.378
19	rs142064826	40902890	A	0.01031	0.0075	G	0.5319	1.378
19	rs1650983	53554075	G	0.01031	0.0075	A	0.5319	1.378
20	rs146114915	23433244	A	0.01031	0.0075	G	0.5319	1.378
20	rs201121458	52645265	A	0.01031	0.0075	G	0.5319	1.378
21	rs200025631	43161057	C	0.01031	0.0075	G	0.5319	1.378
22	rs146269725	36876729	G	0.01031	0.0075	A	0.5319	1.378
4	rs6829588	165221971	A	0.2165	0.2333	C	0.532	0.9079
11	rs294364	30709418	G	0.3067	0.3242	A	0.532	0.9223
22	rs5756349	37196680	G	0.3067	0.3242	A	0.532	0.9223
3	rs877859	107714075	G	0.2165	0.2325	A	0.5321	0.9121
1	rs7533274	62673225	A	0.2423	0.2258	G	0.5322	1.096
1	rs4846567	219750717	A	0.3351	0.3175	C	0.5322	1.083
2	rs74673035	242513552	G	0.1778	0.1642	A	0.5322	1.101
3	rs2075352	10311939	A	0.2423	0.2258	G	0.5322	1.096
12	rs1274726	49501398	A	0.1778	0.1642	G	0.5322	1.101
5	rs189683566	177548240	A	0.01031	0.007513	G	0.5324	1.376
8	rs3935209	145113241	C	0.01031	0.007513	A	0.5324	1.376
15	rs2414908	66870968	A	0.3376	0.3192	G	0.5326	1.087
2	rs2043449	204116690	G	0.06443	0.05583	A	0.5328	1.165
7	rs13227951	66213108	A	0.1314	0.1192	G	0.5328	1.119
10	rs2657291	76926703	A	0.07732	0.08917	G	0.5329	0.856
11	rs10437653	46297631	C	0.07732	0.08917	A	0.5329	0.856
11	rs12280680	46300722	A	0.07732	0.08917	G	0.5329	0.856
14	rs74849041	24029037	A	0.07732	0.08917	G	0.5329	0.856

16	rs148781922	3900336	A	0.01031	0.007525	G	0.5329	1.374
19	rs11084753	34322137	G	0.3093	0.3267	A	0.5329	0.9229
19	rs3745751	48519241	C	0.07732	0.08917	G	0.5329	0.856
1	rs1934299	31943343	A	0.2423	0.2267	G	0.533	1.091
1	rs10913469	177913519	G	0.2423	0.2267	A	0.533	1.091
2	rs3210652	220046840	A	0.1572	0.1717	G	0.533	0.9001
10	rs1398024	23665438	A	0.07732	0.08833	C	0.533	0.8649
14	rs74036628	24877699	G	0.07732	0.08833	A	0.533	0.8649
15	rs16964211	51530495	A	0.2273	0.245	G	0.5331	0.9064
4	rs3843891	138609462	A	0.3119	0.33	C	0.5334	0.9201
5	rs10515148	71716928	C	0.3119	0.3308	G	0.5334	0.9166
6	rs2232430	28359186	A	0.1804	0.1658	G	0.5334	1.107
8	rs3779620	27679981	G	0.3119	0.3308	A	0.5334	0.9166
10	rs10997469	68746285	A	0.3119	0.3308	G	0.5334	0.9166
11	rs7130899	82644904	A	0.3119	0.33	G	0.5334	0.9201
13	rs1140605	102220193	A	0.1804	0.1658	C	0.5334	1.107
17	rs78686509	37107903	A	0.01031	0.007538	G	0.5334	1.372
7	rs200067879	75915006	A	0.002941	0.001701	G	0.5335	1.732
3	rs2660753	87110674	A	0.3376	0.3208	G	0.5338	1.079
4	rs1534140	186117162	A	0.3376	0.3208	G	0.5338	1.079
7	rs6948404	36610493	G	0.09536	0.08417	A	0.5338	1.147
11	rs11024074	16917219	G	0.2448	0.2283	A	0.5338	1.096
7	rs4987657	142574913	G	0.01031	0.00755	A	0.5339	1.369
10	rs1133400	134459388	G	0.3144	0.3333	A	0.5342	0.9173
8	rs4921914	18272438	A	0.3144	0.3325	G	0.5343	0.9207
8	rs1495741	18272881	A	0.3144	0.3325	G	0.5343	0.9207
16	rs56041036	88599023	G	0.3144	0.3325	A	0.5343	0.9207
1	rs1210110	14096821	G	0.2448	0.2292	A	0.5345	1.091
1	rs3766680	175296448	A	0.1804	0.1667	G	0.5345	1.101
1	rs12737248	225555602	C	0.2448	0.2292	A	0.5345	1.091
5	rs6895902	179201847	A	0.1804	0.1667	G	0.5345	1.101
12	rs11146955	133158097	G	0.1804	0.1667	A	0.5345	1.101
19	rs6508750	38379446	G	0.1804	0.1667	A	0.5345	1.101
1	rs74157349	249211096	A	0.1134	0.1267	G	0.5348	0.8819
1	rs77124333	249211619	A	0.1134	0.1267	G	0.5348	0.8819
2	rs1550116	25022598	G	0.2216	0.2379	A	0.535	0.9125
1	rs4908343	27931698	G	0.317	0.335	A	0.5351	0.9214
7	rs6461378	881668	G	0.317	0.335	A	0.5351	0.9214
12	rs2857671	52708420	G	0.317	0.335	A	0.5351	0.9214
16	rs4782300	88498221	G	0.1134	0.1275	A	0.5352	0.8753
5	rs248427	150565008	A	0.3428	0.3258	G	0.5353	1.079
11	rs6591536	59211188	G	0.3428	0.3258	A	0.5353	1.079
9	rs6560142	73150984	G	0.1598	0.1742	A	0.5355	0.9018

1	rs10889353	63118196	C	0.183	0.1683	A	0.5358	1.107
3	rs7624575	95353835	A	0.09536	0.085	C	0.5358	1.135
6	rs73397100	31111125	A	0.09536	0.085	G	0.5358	1.135
6	rs116969494	31111148	T	0.09536	0.085	A	0.5358	1.135
3	rs1163447	109056751	A	0.3479	0.33	C	0.5361	1.083
4	rs7659062	189587079	A	0.134	0.1217	G	0.5363	1.117
1	rs7550857	209956542	A	0.05155	0.06167	C	0.5366	0.827
1	rs12135684	248458760	A	0.3222	0.3408	C	0.5366	0.9192
2	rs1649292	85051122	A	0.134	0.1219	G	0.5366	1.115
2	rs1464168	221544964	A	0.3222	0.3408	G	0.5366	0.9192
6	rs3800326	28264717	A	0.05155	0.06167	G	0.5366	0.827
17	rs635874	76464916	A	0.05155	0.06167	G	0.5366	0.827
2	rs2229668	85628983	A	0.05155	0.06083	G	0.5367	0.839
3	rs3732813	112724661	G	0.2242	0.2408	A	0.5367	0.9111
3	rs2306858	112727199	A	0.2242	0.2408	G	0.5367	0.9111
5	rs702689	56177443	A	0.2242	0.2408	G	0.5367	0.9111
6	rs1936808	127477441	G	0.05155	0.06083	A	0.5367	0.839
20	rs6051545	3025107	A	0.3479	0.3308	G	0.5367	1.079
21	rs2833607	33381040	A	0.2242	0.2408	G	0.5367	0.9111
16	rs1991020	22161136	A	0.183	0.1692	G	0.5368	1.1
21	rs915770	45220483	G	0.25	0.2333	A	0.5368	1.095
19	rs33435	30943455	A	0.2242	0.24	G	0.537	0.9153
1	rs1126656	111957583	G	0.1402	0.127	A	0.5372	1.121
1	rs11553925	12082334	T	0.04124	0.035	A	0.5373	1.186
1	rs11588712	12082461	A	0.04124	0.035	G	0.5373	1.186
1	rs2251746	159272060	G	0.04124	0.035	A	0.5373	1.186
2	rs3749117	160885442	G	0.3531	0.3347	A	0.5373	1.085
4	rs116150154	15004499	A	0.04124	0.035	G	0.5373	1.186
5	rs7724655	107032708	A	0.3247	0.3433	C	0.5373	0.9198
7	rs10486607	29184489	A	0.04124	0.035	G	0.5373	1.186
8	rs17092104	20005554	C	0.04124	0.035	G	0.5373	1.186
9	rs7026705	107331504	C	0.04124	0.035	A	0.5373	1.186
18	rs489693	57882787	A	0.25	0.2342	C	0.5374	1.09
11	rs10896563	56756399	A	0.3531	0.335	C	0.5375	1.083
5	rs149391669	178562922	A	0.01546	0.0217	G	0.5377	0.708
13	rs145018661	110828986	C	0.01546	0.0217	G	0.5377	0.708
1	rs78042623	3412567	A	0.005155	0.01007	G	0.5378	0.5095
1	rs117437844	247151467	A	0.01546	0.02167	G	0.5378	0.7092
5	rs837105	40746916	A	0.01546	0.02167	T	0.5378	0.7092
5	rs16872235	74037386	T	0.01546	0.02167	A	0.5378	0.7092
5	rs957680	74055209	G	0.01546	0.02167	A	0.5378	0.7092
5	rs17844265	140183237	G	0.01546	0.02167	A	0.5378	0.7092
5	rs4242182	174156168	A	0.01546	0.02167	G	0.5378	0.7092

6	rs9257770	29323838	C	0.01546	0.02167	A	0.5378	0.7092
6	rs3749971	29342775	A	0.01546	0.02167	G	0.5378	0.7092
9	rs143061509	125437611	G	0.01546	0.02167	A	0.5378	0.7092
10	rs144121793	17191129	G	0.01546	0.02167	C	0.5378	0.7092
11	rs78511352	5322776	A	0.01546	0.02167	G	0.5378	0.7092
13	rs75005059	25831336	G	0.01546	0.02167	A	0.5378	0.7092
14	rs74039343	23992753	A	0.01546	0.02167	C	0.5378	0.7092
15	rs4646422	75015305	A	0.1624	0.1775	G	0.5378	0.8982
15	rs2472304	75044238	A	0.1624	0.1775	G	0.5378	0.8982
16	rs17822931	48258198	G	0.01546	0.02167	A	0.5378	0.7092
16	rs3764261	56993324	A	0.1624	0.1775	C	0.5378	0.8982
16	rs187620725	67865940	C	0.01546	0.02167	A	0.5378	0.7092
17	rs145355767	10265714	G	0.01546	0.02167	A	0.5378	0.7092
1	rs34471231	16356522	G	0.005155	0.01	A	0.5379	0.513
1	rs117626025	32280367	A	0.005155	0.01	G	0.5379	0.513
1	rs212989	48695081	A	0.005155	0.01	G	0.5379	0.513
1	rs850763	48708228	A	0.005155	0.01	C	0.5379	0.513
1	rs139275808	152975936	C	0.06701	0.0575	G	0.5379	1.177
1	rs36022742	153520954	A	0.005155	0.01	G	0.5379	0.513
1	rs145906888	155221353	G	0.005155	0.01002	C	0.5379	0.5121
1	rs141906655	171688351	G	0.005155	0.01002	A	0.5379	0.5121
1	rs33990840	206225011	C	0.005155	0.01	G	0.5379	0.513
1	rs202210586	245849483	C	0.005155	0.01	G	0.5379	0.513
2	rs201462244	10803104	G	0.005155	0.01	A	0.5379	0.513
2	rs34892520	86269163	A	0.005155	0.01	G	0.5379	0.513
2	rs75320001	102958715	A	0.005155	0.01	G	0.5379	0.513
2	rs139274808	196762444	G	0.005155	0.01	A	0.5379	0.513
2	rs2289170	209215633	A	0.005155	0.01	G	0.5379	0.513
3	rs201896056	50645911	T	0.005155	0.01	A	0.5379	0.513
3	rs182902370	54952918	A	0.005155	0.01	G	0.5379	0.513
3	rs144519399	121713043	A	0.005155	0.01	G	0.5379	0.513
3	rs144835358	149245654	C	0.005155	0.01	A	0.5379	0.513
3	rs118079207	173998768	A	0.005155	0.01	G	0.5379	0.513
3	rs140058809	193042718	G	0.005155	0.01	A	0.5379	0.513
3	rs145013670	194061922	A	0.005155	0.01	G	0.5379	0.513
4	rs41264705	7780582	A	0.06701	0.0575	G	0.5379	1.177
4	rs118048800	47514778	G	0.005155	0.01	A	0.5379	0.513
4	rs150327134	56436020	A	0.005155	0.01	G	0.5379	0.513
4	rs3811765	80247016	A	0.005155	0.01	G	0.5379	0.513
4	rs55735703	100057727	A	0.005155	0.01	C	0.5379	0.513
5	rs3763118	131325201	A	0.005155	0.01	G	0.5379	0.513
5	rs10036567	141307833	G	0.005155	0.01	A	0.5379	0.513
5	rs78819112	149006727	A	0.005155	0.01	C	0.5379	0.513

5	rs77711671	154278131	A	0.005155	0.01	G	0.5379	0.513
6	rs3823184	7606299	A	0.005155	0.01	G	0.5379	0.513
6	rs111652998	24447177	A	0.005155	0.01	G	0.5379	0.513
6	rs17342717	25821770	A	0.005155	0.01	G	0.5379	0.513
6	rs57312007	65300516	C	0.005155	0.01	A	0.5379	0.513
6	rs1998813	109900765	A	0.005155	0.01	C	0.5379	0.513
6	rs3218606	111689082	A	0.005155	0.01	G	0.5379	0.513
7	rs144372265	31009485	A	0.005155	0.01	G	0.5379	0.513
7	rs189837730	122342453	A	0.005155	0.01	G	0.5379	0.513
7	rs117366477	128485276	A	0.005155	0.01	G	0.5379	0.513
7	rs1046515	140394587	A	0.06701	0.0575	G	0.5379	1.177
7	rs147597725	150035766	A	0.005155	0.01	G	0.5379	0.513
8	rs142253103	12943382	A	0.005155	0.01	G	0.5379	0.513
8	rs115507207	71646084	A	0.005155	0.01	G	0.5379	0.513
8	rs191140809	125115409	G	0.005155	0.01	C	0.5379	0.513
9	rs56023271	98209213	A	0.005155	0.01	G	0.5379	0.513
10	rs2279219	3181157	G	0.005155	0.01	C	0.5379	0.513
10	rs12774134	4963327	A	0.06701	0.0575	G	0.5379	1.177
10	rs139953205	22022704	A	0.005155	0.01	G	0.5379	0.513
11	rs2232919	10876353	G	0.005155	0.01	A	0.5379	0.513
11	rs2232918	10876443	A	0.005155	0.01	G	0.5379	0.513
11	rs2761591	31329373	A	0.005155	0.01	G	0.5379	0.513
11	rs143538754	32953516	A	0.005155	0.01	G	0.5379	0.513
11	rs145772634	74977269	G	0.005155	0.01	A	0.5379	0.513
11	rs189589528	101762206	A	0.005155	0.01	G	0.5379	0.513
11	rs187990506	108060001	G	0.005155	0.01	A	0.5379	0.513
11	rs77432041	133807733	A	0.005155	0.01	G	0.5379	0.513
12	rs200479270	99020502	C	0.005155	0.01	A	0.5379	0.513
13	rs146504568	111279829	A	0.005155	0.01	G	0.5379	0.513
14	rs142700275	39649781	G	0.005155	0.01	A	0.5379	0.513
14	rs150464227	64465019	A	0.005155	0.01	G	0.5379	0.513
15	rs145061993	45396563	A	0.005155	0.01	G	0.5379	0.513
15	rs150262564	50931700	G	0.005155	0.01	C	0.5379	0.513
15	rs144807493	74467625	G	0.005155	0.01	C	0.5379	0.513
15	rs11073619	85188839	A	0.005155	0.01	G	0.5379	0.513
15	rs138352975	90984869	G	0.005155	0.01	A	0.5379	0.513
16	rs202137785	1821166	A	0.005155	0.01	G	0.5379	0.513
16	rs115073082	23404584	A	0.005155	0.01	G	0.5379	0.513
16	rs34214571	49671177	G	0.005155	0.01	A	0.5379	0.513
16	rs2290178	57719586	A	0.005155	0.01	G	0.5379	0.513
17	rs181049067	40001660	G	0.005155	0.01	A	0.5379	0.513
17	rs58471799	41606044	A	0.005155	0.01	G	0.5379	0.513
17	rs28725418	72306291	A	0.06701	0.0575	G	0.5379	1.177

18	rs3752097	74155000	G	0.005155	0.01	A	0.5379	0.513
19	rs200110632	3150130	A	0.005155	0.01	T	0.5379	0.513
19	rs201322610	4327381	G	0.005155	0.01	A	0.5379	0.513
19	rs12710265	9059232	G	0.005155	0.01	A	0.5379	0.513
19	rs12977368	9060059	C	0.005155	0.01	A	0.5379	0.513
19	rs117617972	44636575	G	0.005155	0.01	A	0.5379	0.513
20	rs78127944	33874600	A	0.005155	0.01	G	0.5379	0.513
20	rs146315738	35826857	G	0.005155	0.01	A	0.5379	0.513
20	rs181280610	44003998	A	0.005155	0.01	G	0.5379	0.513
20	rs201785583	44469698	A	0.005155	0.01	G	0.5379	0.513
20	rs142580477	51870163	G	0.005155	0.01	A	0.5379	0.513
21	rs141637413	37734516	G	0.005155	0.01	A	0.5379	0.513
21	rs146657926	40578068	G	0.005155	0.01	A	0.5379	0.513
21	rs14378	47581423	A	0.005155	0.01	G	0.5379	0.513
21	rs113146399	47581949	A	0.005155	0.01	G	0.5379	0.513
18	rs35470719	55336626	G	0.03093	0.03859	A	0.538	0.7951
1	rs191693014	156776968	A	0.03093	0.03917	G	0.5381	0.7829
2	rs2255991	37579971	A	0.3273	0.3458	G	0.5381	0.9204
2	rs35771982	160885418	G	0.3531	0.3358	C	0.5381	1.079
7	rs3807153	138417791	G	0.03093	0.03917	A	0.5381	0.7829
9	rs10756576	14556659	A	0.3273	0.3458	G	0.5381	0.9204
10	rs118083279	105967431	A	0.03093	0.03917	G	0.5381	0.7829
11	rs3747548	45869013	A	0.03093	0.03917	C	0.5381	0.7829
13	rs76838169	20763113	G	0.03093	0.03917	A	0.5381	0.7829
14	rs858888	46728896	A	0.3531	0.3358	G	0.5381	1.079
17	rs2305556	39671724	G	0.03093	0.03917	A	0.5381	0.7829
17	rs55781437	40939855	A	0.03093	0.03917	C	0.5381	0.7829
17	rs2290041	40947320	A	0.03093	0.03917	G	0.5381	0.7829
17	rs61995868	40957849	A	0.03093	0.03917	G	0.5381	0.7829
22	rs61735519	45749966	G	0.03093	0.03917	C	0.5381	0.7829
2	rs36075906	234436069	A	0.2268	0.2433	G	0.5382	0.9121
8	rs4840955	8234219	G	0.3557	0.3375	A	0.5382	1.084
10	rs1541010	13755544	A	0.1856	0.1708	G	0.5382	1.106
13	rs12429889	74742322	G	0.134	0.1225	A	0.5382	1.109
18	rs2848782	39152046	A	0.3557	0.3375	G	0.5382	1.084
5	rs7710874	29339392	G	0.3273	0.345	A	0.5383	0.9238
5	rs2229086	108281909	G	0.0799	0.09167	C	0.5383	0.8605
14	rs1041316	57099859	G	0.3273	0.345	A	0.5383	0.9238
3	rs11708189	140175360	A	0.2539	0.2375	G	0.5384	1.092
1	rs2901964	15792426	G	0.0799	0.09083	C	0.5385	0.8691
2	rs1801251	233633460	A	0.25	0.235	G	0.5385	1.085
6	rs6934690	54054686	T	0.25	0.235	A	0.5385	1.085
8	rs3779617	102631911	A	0.0799	0.09083	G	0.5385	0.8691

1	rs10908356	38038388	G	0.3557	0.3383	A	0.5387	1.08
7	rs6944414	71152622	G	0.3557	0.3383	A	0.5387	1.08
8	rs4840953	8234192	C	0.3557	0.3383	G	0.5387	1.08
6	rs3094572	29480993	A	0.3299	0.3483	G	0.5388	0.921
6	rs3094564	29486345	G	0.3299	0.3483	A	0.5388	0.921
10	rs7478291	24489659	A	0.3299	0.3483	G	0.5388	0.921
2	rs2945572	141096291	A	0.3299	0.3475	C	0.5391	0.9244
3	rs864265	186554292	A	0.09794	0.08667	C	0.5391	1.144
6	rs3130955	31054511	A	0.3299	0.3475	C	0.5391	0.9244
7	rs10486715	41478608	A	0.1856	0.1717	G	0.5391	1.099
16	rs11643718	56933519	A	0.09794	0.08667	G	0.5391	1.144
17	rs79803528	19861599	G	0.09794	0.08667	C	0.5391	1.144
17	exm1303644	19871740	G	0.09794	0.08667	C	0.5391	1.144
17	rs76345077	19871740	G	0.09794	0.08667	C	0.5391	1.144
1	rs143770846	24840882	G	0.01546	0.0225	A	0.5392	0.6824
2	rs34694228	141457962	A	0.01546	0.0225	G	0.5392	0.6824
4	rs140107488	154279752	T	0.01546	0.0225	A	0.5392	0.6824
6	rs61744969	29555738	A	0.01546	0.0225	C	0.5392	0.6824
6	rs117938048	73905122	A	0.01546	0.0225	T	0.5392	0.6824
8	rs2230229	23049292	G	0.01546	0.0225	A	0.5392	0.6824
9	rs148377508	133927922	G	0.01546	0.0225	A	0.5392	0.6824
11	rs149483638	2161530	A	0.01546	0.0225	G	0.5392	0.6824
11	rs140687140	118531335	G	0.01546	0.0225	A	0.5392	0.6824
12	rs303819	52215102	A	0.01546	0.0225	C	0.5392	0.6824
13	rs76366906	60545212	A	0.01546	0.0225	G	0.5392	0.6824
13	rs80037301	60590107	T	0.01546	0.0225	A	0.5392	0.6824
18	rs184924723	76856565	G	0.01546	0.0225	A	0.5392	0.6824
22	rs12170939	38046695	A	0.01546	0.0225	C	0.5392	0.6824
6	rs3734311	76660551	C	0.3582	0.3408	G	0.5394	1.08
1	rs7533863	113822284	A	0.3325	0.3508	G	0.5395	0.9216
13	rs9315923	43070894	G	0.3325	0.3508	A	0.5395	0.9216
12	rs1860045	2154876	G	0.3608	0.3425	A	0.5396	1.084
14	rs1957358	55222475	G	0.3608	0.3425	A	0.5396	1.084
6	rs1800454	32800412	A	0.1366	0.1242	G	0.5397	1.116
15	rs2415129	72191266	G	0.2294	0.2467	A	0.5397	0.9091
19	rs56854837	38876352	A	0.2294	0.2467	G	0.5397	0.9091
2	rs6710518	166583244	A	0.3325	0.35	G	0.5398	0.925
9	rs12685968	117093939	C	0.03093	0.04	G	0.5398	0.766
10	rs41283303	50178246	A	0.03093	0.04	G	0.5398	0.766
11	rs115333544	5373309	A	0.03093	0.04	C	0.5398	0.766
17	rs77725092	40849573	A	0.03093	0.04	G	0.5398	0.766
2	rs6707760	45123765	A	0.1649	0.18	C	0.5401	0.8999
6	rs3130391	30190640	A	0.3608	0.3433	G	0.5401	1.08

8	rs3824276	41906095	G	0.2552	0.2392	A	0.5402	1.09
15	rs6416452	53994493	A	0.2552	0.2392	G	0.5402	1.09
10	rs2224865	85422854	C	0.3351	0.3533	A	0.5403	0.9222
1	rs6683071	222923351	A	0.1881	0.1733	G	0.5404	1.105
5	rs62621912	79031558	A	0.1881	0.1733	G	0.5404	1.105
5	rs7701116	138406078	A	0.1881	0.1733	G	0.5404	1.105
8	rs9297447	112515260	A	0.1649	0.1792	G	0.5404	0.905
17	rs61746217	79993919	A	0.1649	0.1792	G	0.5404	0.905
19	rs2279517	46914547	C	0.1881	0.1733	G	0.5404	1.105
19	rs75175362	46914927	A	0.1881	0.1733	G	0.5404	1.105
2	rs1489479	179708677	A	0.3351	0.3525	C	0.5406	0.9256
11	rs6591090	104532836	A	0.3351	0.3525	G	0.5406	0.9256
15	rs285705	93118504	A	0.3351	0.3525	G	0.5406	0.9256
8	rs6472966	77258843	G	0.06701	0.05833	A	0.5407	1.159
11	rs2029285	104418194	G	0.3634	0.3458	A	0.5407	1.08
14	rs1770984	104559919	G	0.3394	0.3575	A	0.5407	0.9233
22	rs35113423	21989142	A	0.06701	0.05833	G	0.5407	1.159
5	rs4635969	1308552	A	0.09794	0.0875	G	0.5409	1.132
3	rs2166801	77526031	A	0.3376	0.3558	C	0.541	0.9228
3	rs12638324	122607098	G	0.366	0.3475	A	0.541	1.084
9	rs58226717	125288893	A	0.366	0.3475	G	0.541	1.084
10	rs7086845	91743629	G	0.366	0.3475	A	0.541	1.084
10	rs807023	102770082	A	0.3376	0.3558	C	0.541	0.9228
15	rs2924835	101606889	A	0.3376	0.3558	G	0.541	0.9228
3	rs2378269	187223226	A	0.2552	0.24	C	0.5412	1.085
2	rs993648	182455994	A	0.3376	0.355	G	0.5413	0.9261
11	rs12812	1977552	A	0.1881	0.1742	G	0.5413	1.099
7	rs4598195	107503441	C	0.3402	0.3583	A	0.5416	0.9233
9	rs1883025	107664301	A	0.2577	0.2417	G	0.5416	1.09
19	rs1615419	3030118	G	0.3402	0.3583	A	0.5416	0.9233
19	rs266849	51349090	G	0.3679	0.3508	A	0.5416	1.077
7	rs1981529	87913361	G	0.1186	0.1317	A	0.5419	0.887
8	rs3829036	125568623	G	0.1186	0.1317	A	0.5419	0.887
12	rs3759302	32135186	A	0.1186	0.1317	T	0.5419	0.887
15	rs12593813	68036852	G	0.3686	0.3508	A	0.5419	1.08
6	rs3130171	32998729	G	0.3402	0.3575	A	0.542	0.9267
14	rs78211950	45650900	G	0.1366	0.1252	A	0.542	1.105
6	rs1998206	117725448	C	0.1186	0.1325	A	0.5421	0.8806
7	rs10229800	53103425	A	0.1186	0.1325	C	0.5421	0.8806
5	rs2922426	107563468	G	0.3428	0.3614	A	0.5422	0.9215
10	rs11191580	104906211	G	0.2577	0.2425	A	0.5425	1.085
10	rs10795668	8701219	A	0.3686	0.3517	G	0.5426	1.076
10	rs9664222	89338633	A	0.2345	0.2517	C	0.5426	0.9111

2	rs882300	136976255	A	0.1675	0.1833	G	0.5427	0.8964
10	rs3998860	70405855	A	0.1675	0.1833	G	0.5427	0.8964
6	rs9367359	49494241	G	0.3497	0.3685	A	0.5429	0.9217
11	rs10830602	90646272	A	0.3711	0.3539	G	0.5429	1.077
4	rs6840372	122978844	A	0.2603	0.2442	G	0.543	1.089
6	rs3131019	29470985	C	0.3454	0.3633	A	0.543	0.9244
7	rs3735007	138732497	A	0.3454	0.3633	G	0.543	0.9244
11	rs716274	103418158	G	0.2603	0.2442	A	0.543	1.089
16	rs9302752	50719103	G	0.3454	0.3633	A	0.543	0.9244
1	rs6702859	21031983	G	0.1392	0.1267	A	0.5431	1.115
14	rs3736772	45665468	G	0.1392	0.1267	C	0.5431	1.115
1	rs2070955	24194773	A	0.1907	0.1767	G	0.5435	1.098
2	rs7580625	144146850	A	0.3479	0.3667	G	0.5435	0.9217
8	rs7834527	20787381	A	0.3479	0.3667	G	0.5435	0.9217
10	rs10786706	104500659	G	0.3479	0.3667	A	0.5435	0.9217
13	rs2031532	50080847	A	0.08247	0.09417	G	0.5435	0.8647
5	rs1000113	150240076	A	0.3763	0.3581	G	0.5436	1.081
1	rs7543130	100049785	A	0.04381	0.03667	C	0.5437	1.204
4	rs4318720	118332255	A	0.3763	0.3583	G	0.5437	1.08
5	rs31517	135287029	A	0.3763	0.3583	G	0.5437	1.08
8	rs1036689	2112130	A	0.3763	0.3583	G	0.5437	1.08
12	rs117889746	9333626	A	0.09794	0.08833	G	0.5437	1.121
14	rs148871932	45644706	G	0.04381	0.03667	A	0.5437	1.204
17	rs139928505	58678037	A	0.04381	0.03667	G	0.5437	1.204
20	rs140056672	31757102	C	0.04381	0.03667	G	0.5437	1.204
20	rs61734343	31761885	A	0.04381	0.03667	G	0.5437	1.204
20	rs1075435	31767470	G	0.04381	0.03667	C	0.5437	1.204
6	rs208696	148865257	G	0.3737	0.3567	A	0.5438	1.076
16	rs4843880	86008641	A	0.3505	0.3692	G	0.5441	0.9222
1	rs2289189	220331205	C	0.08247	0.095	G	0.5442	0.8563
11	rs17884789	102398329	A	0.08247	0.095	G	0.5442	0.8563
1	rs1046592	185069561	G	0.3763	0.3592	A	0.5443	1.076
3	rs6768523	111962851	G	0.3763	0.3592	A	0.5443	1.076
18	rs1071600	3126811	G	0.3789	0.3608	A	0.5443	1.08
18	rs9945724	46440489	A	0.2629	0.2467	G	0.5443	1.089
22	rs1894704	26853905	C	0.2629	0.2467	A	0.5443	1.089
22	rs1894706	26854441	G	0.2629	0.2467	A	0.5443	1.089
2	rs13031237	61136129	A	0.02577	0.02	C	0.5446	1.296
2	rs2194508	186317662	G	0.05412	0.06417	A	0.5446	0.8345
3	rs2305868	148786085	G	0.02577	0.02	A	0.5446	1.296
6	rs205944	118496796	A	0.02577	0.02	G	0.5446	1.296
9	rs12339582	35398900	A	0.02577	0.02	C	0.5446	1.296
10	rs9665610	116008464	T	0.05412	0.06417	A	0.5446	0.8345

11	rs369819	16863130	G	0.05412	0.06417	A	0.5446	0.8345
12	rs638043	53044267	A	0.02577	0.02	G	0.5446	1.296
12	rs78504538	62861043	A	0.02577	0.02	G	0.5446	1.296
13	rs2765086	24504955	A	0.02577	0.02	G	0.5446	1.296
14	rs1959607	20452460	G	0.02577	0.02	A	0.5446	1.296
14	rs199596610	55878516	A	0.02577	0.02	C	0.5446	1.296
15	rs77096807	42982124	G	0.02577	0.02	A	0.5446	1.296
16	rs61743495	1484455	G	0.02577	0.02	A	0.5446	1.296
17	rs117575112	47210469	A	0.05412	0.06427	G	0.5446	0.833
1	rs2136093	90875888	G	0.1701	0.185	A	0.5447	0.903
6	rs1931983	142565531	A	0.3531	0.3717	G	0.5447	0.9227
16	rs8047222	50660962	G	0.3531	0.3717	A	0.5447	0.9227
1	rs12048768	15441090	A	0.1392	0.1275	G	0.5448	1.106
1	rs61744357	16134072	A	0.005155	0.01083	G	0.5448	0.4731
1	rs114404250	36056256	A	0.005155	0.01083	G	0.5448	0.4731
1	rs140498292	151502400	A	0.005155	0.01083	G	0.5448	0.4731
1	rs2234927	171607843	C	0.005155	0.01083	G	0.5448	0.4731
1	rs145634709	177908893	G	0.005155	0.01083	A	0.5448	0.4731
1	rs200038092	216138711	C	0.005155	0.01083	A	0.5448	0.4731
2	rs192467161	15523409	A	0.005155	0.01083	G	0.5448	0.4731
2	rs147302249	44116923	A	0.005155	0.01083	G	0.5448	0.4731
2	rs201556073	71190308	G	0.005155	0.01083	A	0.5448	0.4731
2	rs139314354	97508140	A	0.005155	0.01083	G	0.5448	0.4731
3	rs2256734	52852488	C	0.005155	0.01083	G	0.5448	0.4731
3	rs2276814	52853480	A	0.005155	0.01083	T	0.5448	0.4731
3	exm2256090	52863155	A	0.005155	0.01083	G	0.5448	0.4731
3	rs150181495	52863155	A	0.005155	0.01083	G	0.5448	0.4731
3	rs10513680	169644710	C	0.1392	0.1275	G	0.5448	1.106
4	rs191118242	151792589	C	0.005155	0.01083	A	0.5448	0.4731
4	rs151170713	164436465	A	0.005155	0.01083	C	0.5448	0.4731
6	rs200847762	32097148	A	0.005155	0.01083	G	0.5448	0.4731
6	rs184343332	46656357	A	0.005155	0.01083	C	0.5448	0.4731
6	rs139146419	112512915	A	0.005155	0.01083	G	0.5448	0.4731
6	rs8191859	160485490	A	0.005155	0.01083	G	0.5448	0.4731
6	rs145450955	160671651	A	0.005155	0.01083	G	0.5448	0.4731
8	rs75741316	6299654	G	0.005155	0.01083	A	0.5448	0.4731
9	rs1926749	26579248	A	0.005155	0.01083	G	0.5448	0.4731
10	rs202182563	100021916	G	0.005155	0.01083	A	0.5448	0.4731
11	rs138243875	810318	G	0.005155	0.01083	C	0.5448	0.4731
11	rs77344022	6190850	A	0.005155	0.01083	G	0.5448	0.4731
11	rs4909945	10673739	A	0.005155	0.01083	G	0.5448	0.4731
11	rs192676304	55322140	G	0.005155	0.01083	A	0.5448	0.4731
12	rs185375655	94072680	C	0.005155	0.01083	A	0.5448	0.4731

12	rs140450523	122091018	G	0.005155	0.01083	A	0.5448	0.4731
12	rs73417486	125398319	A	0.005155	0.01083	G	0.5448	0.4731
14	rs192457260	55621364	C	0.005155	0.01083	G	0.5448	0.4731
14	rs142539336	91763720	A	0.005155	0.01083	G	0.5448	0.4731
14	rs148548158	93650172	A	0.005155	0.01083	G	0.5448	0.4731
16	rs76162903	81174999	G	0.1907	0.1775	A	0.5448	1.092
19	rs62621087	10259654	A	0.005155	0.01083	G	0.5448	0.4731
19	rs150063338	38682946	A	0.005155	0.01083	G	0.5448	0.4731
19	rs2290772	50393130	A	0.005155	0.01083	C	0.5448	0.4731
20	rs41400249	23028934	A	0.005155	0.01083	G	0.5448	0.4731
20	rs61749691	56136510	A	0.005155	0.01083	C	0.5448	0.4731
21	rs200493501	27117541	A	0.005155	0.01083	G	0.5448	0.4731
1	rs12029454	162133117	A	0.3531	0.3708	G	0.5449	0.926
4	rs16880691	28473048	C	0.3789	0.3617	A	0.5449	1.077
6	rs858345	132163617	G	0.3789	0.3617	A	0.5449	1.077
8	rs12677791	89342944	G	0.3789	0.3617	A	0.5449	1.077
11	rs3135507	116661488	A	0.1701	0.1858	G	0.5449	0.898
13	rs2241913	76382100	A	0.1701	0.1858	G	0.5449	0.898
16	rs7193541	74664743	G	0.3789	0.3617	A	0.5449	1.077
3	rs1395769	96442435	A	0.2629	0.2475	G	0.5451	1.084
15	rs11631813	82555242	G	0.2629	0.2475	A	0.5451	1.084
5	rs60708187	145215872	G	0.1211	0.1344	A	0.5452	0.8878
2	rs28365986	69240651	A	0.1211	0.1342	G	0.5453	0.8895
2	rs12474603	100250011	A	0.3557	0.3742	G	0.5453	0.9233
11	rs2303975	14276999	A	0.1211	0.1342	G	0.5453	0.8895
20	rs4811309	50817740	A	0.3557	0.3742	G	0.5453	0.9233
1	rs139902189	1430985	A	0.1211	0.135	G	0.5454	0.8831
3	rs2724693	137807259	G	0.3814	0.3642	A	0.5454	1.077
5	rs409045	34628627	G	0.1211	0.135	A	0.5454	0.8831
9	rs2250242	117103973	A	0.3814	0.3642	G	0.5454	1.077
11	rs10749920	108383676	G	0.2397	0.2558	A	0.5454	0.917
18	rs1941184	29038123	C	0.2397	0.2558	A	0.5454	0.917
19	rs439401	45414451	G	0.384	0.3658	A	0.5454	1.081
15	rs4776396	68640452	G	0.3557	0.3733	A	0.5455	0.9266
2	rs2244438	202252539	A	0.2655	0.2492	G	0.5456	1.089
6	rs214976	152772264	A	0.2655	0.2492	G	0.5456	1.089
6	rs70018	152774033	G	0.2655	0.2492	A	0.5456	1.089
2	rs6714832	154808697	G	0.06959	0.06	A	0.5457	1.172
2	rs1470778	165196500	A	0.06959	0.06	C	0.5457	1.172
6	rs6926224	30882689	A	0.06959	0.06	G	0.5457	1.172
6	rs4236176	170070723	A	0.06959	0.06	G	0.5457	1.172
11	rs77759828	124792667	G	0.06959	0.06	A	0.5457	1.172
18	rs1395063	5145609	A	0.06959	0.06	C	0.5457	1.172

1	rs6701037	175120079	C	0.1005	0.09	A	0.5458	1.13
4	rs2270655	146576418	G	0.1005	0.09	C	0.5458	1.13
5	rs285599	73339114	G	0.384	0.3667	A	0.5459	1.077
5	rs1048719	150632832	A	0.1933	0.1795	G	0.5459	1.096
12	rs7956679	48866585	A	0.384	0.3671	C	0.5461	1.075
20	rs6074148	1098486	A	0.3582	0.3758	G	0.5461	0.9271
2	rs1106609	221238189	G	0.3866	0.3692	A	0.5464	1.077
10	rs11009235	33421548	A	0.3866	0.3692	G	0.5464	1.077
14	rs1263810	21991626	C	0.2655	0.25	G	0.5464	1.084
19	rs11615	45923653	A	0.2655	0.25	G	0.5464	1.084
6	rs7759740	79196788	G	0.3608	0.3792	A	0.5465	0.9243
1	rs10913300	177193408	G	0.3608	0.3783	A	0.5467	0.9276
20	rs6090685	46114027	G	0.3608	0.3783	A	0.5467	0.9276
13	rs4884021	76397731	A	0.2423	0.2583	G	0.5468	0.9179
17	rs11651270	5425077	G	0.2655	0.2504	A	0.5468	1.082
19	rs34226397	14758168	A	0.2423	0.2583	G	0.5468	0.9179
1	rs7553686	185364104	G	0.3892	0.3717	A	0.5469	1.077
6	rs1264309	30875899	G	0.268	0.2517	A	0.5469	1.089
6	rs1264303	30882513	G	0.268	0.2517	A	0.5469	1.089
6	rs7738138	30887344	G	0.268	0.2517	A	0.5469	1.089
6	rs2074506	30890483	A	0.268	0.2517	C	0.5469	1.089
6	rs2252760	30892377	C	0.268	0.2517	G	0.5469	1.089
11	rs2242182	562421	A	0.1933	0.18	G	0.5469	1.092
11	rs3851179	85868640	A	0.3892	0.3717	G	0.5469	1.077
15	rs600753	55759193	A	0.268	0.2517	G	0.5469	1.089
1	rs12043423	36550658	A	0.1727	0.1883	G	0.547	0.8995
1	rs2300747	117104215	A	0.3918	0.3733	G	0.547	1.081
1	rs876537	159674933	G	0.3918	0.3733	A	0.547	1.081
5	rs351855	176520243	A	0.3918	0.3733	G	0.547	1.081
6	rs2107202	30105743	A	0.1727	0.1883	G	0.547	0.8995
6	rs2524182	30425943	T	0.3918	0.3733	A	0.547	1.081
7	rs1109806	21778449	A	0.1727	0.1883	G	0.547	0.8995
8	rs1793709	118296603	G	0.3634	0.3817	A	0.547	0.9248
8	rs1876201	139982997	G	0.1727	0.1883	A	0.547	0.8995
19	rs2633350	16808183	G	0.3634	0.3817	A	0.547	0.9248
21	rs1452093	28744356	G	0.1727	0.1883	A	0.547	0.8995
19	rs73032311	41386033	G	0.1959	0.1811	A	0.5471	1.101
5	rs3195676	34008100	A	0.3634	0.3808	G	0.5473	0.9281
7	rs11763044	47481602	A	0.3634	0.3808	G	0.5473	0.9281
6	rs2233978	31079703	G	0.3943	0.3758	C	0.5475	1.081
1	rs2281959	167097739	C	0.268	0.2525	A	0.5476	1.084
2	rs2241797	105885961	G	0.268	0.2525	A	0.5476	1.084
6	rs2844702	30912481	A	0.268	0.2525	G	0.5476	1.084

11	rs1077126	5602275	A	0.268	0.2525	G	0.5476	1.084
11	rs2341433	5602605	G	0.268	0.2525	A	0.5476	1.084
5	rs217272	137486426	G	0.3943	0.3765	A	0.5477	1.078
12	rs3741903	6030405	G	0.1959	0.1817	A	0.5477	1.097
7	rs12540874	50664922	G	0.366	0.3833	A	0.5479	0.9286
7	rs7796960	64767457	A	0.3943	0.3767	G	0.5479	1.077
12	rs11829037	121151093	A	0.1418	0.13	G	0.5479	1.105
15	rs7164838	34967574	A	0.3943	0.3767	G	0.5479	1.077
19	rs2305780	14083761	G	0.366	0.3833	A	0.5479	0.9286
6	rs2242416	43273604	A	0.2448	0.2617	G	0.548	0.9149
3	rs7614232	184452676	A	0.3686	0.3867	G	0.5481	0.9258
14	rs810087	62021879	G	0.3686	0.3867	A	0.5481	0.9258
5	rs2619844	5460619	C	0.2706	0.2542	G	0.5482	1.089
10	rs156697	106039185	G	0.2448	0.2608	A	0.5482	0.9188
15	rs4417518	81624768	G	0.2448	0.2608	A	0.5482	0.9188
2	rs13398848	84245168	G	0.06959	0.06083	A	0.5483	1.155
19	rs117767265	2405456	A	0.06959	0.06083	G	0.5483	1.155
19	rs3752187	14862278	A	0.06959	0.06083	G	0.5483	1.155
1	rs3737786	161049662	G	0.08505	0.09667	A	0.5485	0.8687
6	rs16880441	88975977	C	0.08505	0.09667	G	0.5485	0.8687
7	rs6949654	77035392	A	0.1005	0.09083	G	0.5485	1.119
7	rs79471015	103474041	G	0.1005	0.09083	C	0.5485	1.119
10	rs9414827	52897074	A	0.08505	0.09667	G	0.5485	0.8687
4	rs971074	100341861	A	0.1237	0.1367	G	0.5486	0.8918
17	rs73232344	7674784	A	0.1237	0.1375	G	0.5487	0.8856
9	rs10820100	104989394	A	0.3995	0.3817	G	0.5488	1.078
11	rs2227973	36597313	A	0.3995	0.3817	G	0.5488	1.078
1	rs6693255	63999826	A	0.1959	0.1825	G	0.5489	1.091
1	rs2273367	64021096	A	0.1959	0.1825	G	0.5489	1.091
3	rs3846041	55449471	A	0.1753	0.19	G	0.549	0.9059
3	rs1382269	137108482	G	0.3737	0.3925	A	0.549	0.9236
5	rs3733698	140502343	A	0.08505	0.0975	G	0.549	0.8605
5	rs3733697	140502344	A	0.08505	0.0975	G	0.549	0.8605
20	rs2245231	37396120	G	0.3969	0.38	A	0.549	1.074
20	rs2301366	43836173	A	0.08505	0.0975	T	0.549	0.8605
2	rs7579944	30445026	G	0.3737	0.3917	A	0.5491	0.9268
6	rs3095341	30726748	A	0.1753	0.1908	G	0.5491	0.901
9	rs3935601	94508700	G	0.3737	0.3917	A	0.5491	0.9268
10	rs45575338	5784151	G	0.1753	0.1908	A	0.5491	0.901
3	rs1716975	41960006	A	0.1985	0.1836	G	0.5492	1.101
10	rs2297040	30663275	A	0.4021	0.3842	G	0.5493	1.078
16	rs12447690	88298124	G	0.2474	0.2642	A	0.5493	0.9158
2	rs2044770	175183905	A	0.3995	0.3825	G	0.5494	1.074

8	rs2069561	133975283	A	0.3995	0.3825	G	0.5494	1.074
1	rs12074053	177974569	G	0.3763	0.395	A	0.5495	0.9241
3	rs9834596	65034421	G	0.1443	0.1317	A	0.5495	1.112
11	rs1508102	116379889	A	0.2474	0.2633	G	0.5495	0.9197
15	rs7359257	67702907	C	0.1443	0.1317	A	0.5495	1.112
19	rs17000886	9083457	A	0.3763	0.395	G	0.5495	0.9241
19	rs11085805	9085004	G	0.3763	0.395	A	0.5495	0.9241
19	rs1574479	9085643	G	0.3763	0.395	A	0.5495	0.9241
19	rs4520945	9086318	A	0.3763	0.395	G	0.5495	0.9241
19	rs12611293	9087758	A	0.3763	0.395	G	0.5495	0.9241
19	rs17000947	9088330	A	0.3763	0.395	G	0.5495	0.9241
19	rs17000950	9088772	G	0.3763	0.395	A	0.5495	0.9241
1	rs28372941	151288698	C	0.04381	0.0375	A	0.5496	1.176
5	rs2229232	35876449	A	0.04381	0.0375	G	0.5496	1.176
15	rs34958422	55652719	G	0.04381	0.0375	A	0.5496	1.176
16	rs75318258	31374256	C	0.04381	0.0375	A	0.5496	1.176
17	rs7213256	39114962	A	0.04381	0.0375	G	0.5496	1.176
20	rs117858854	42939673	G	0.04381	0.0375	A	0.5496	1.176
2	rs16866538	179659912	G	0.3763	0.3942	A	0.5497	0.9273
3	rs3774372	41877414	G	0.1985	0.1842	A	0.5497	1.097
3	rs9815354	41912651	A	0.1985	0.1842	G	0.5497	1.097
3	rs1052501	41925398	G	0.1985	0.1842	A	0.5497	1.097
12	rs10773123	125427751	G	0.4046	0.3867	A	0.5497	1.078
2	rs3817362	238449007	G	0.4021	0.385	A	0.5498	1.074
7	rs10268865	143716265	A	0.4021	0.385	G	0.5498	1.074
6	rs2534804	30486456	G	0.2732	0.2575	A	0.55	1.084
12	rs7958340	20097565	G	0.3789	0.3975	A	0.55	0.9245
12	rs4341610	96149288	A	0.2732	0.2575	C	0.55	1.084
16	rs758747	3627358	A	0.3789	0.3975	G	0.55	0.9245
1	rs3795634	200635550	G	0.3834	0.4017	A	0.5501	0.9263
17	rs78111408	48618360	A	0.04381	0.03756	G	0.5501	1.174
18	rs763361	67531642	A	0.4072	0.3892	G	0.5501	1.078
5	rs11134354	9421794	G	0.4046	0.3875	A	0.5502	1.074
7	rs1005261	139884494	G	0.3789	0.3967	A	0.5502	0.9278
13	rs4526902	65714783	G	0.3789	0.3967	A	0.5502	0.9278
11	rs4391795	3249552	A	0.3814	0.4	G	0.5504	0.925
4	rs10008898	60739796	G	0.4098	0.3917	A	0.5506	1.078
13	rs7992643	100555038	C	0.4072	0.39	G	0.5506	1.074
15	rs200699205	40268998	C	0.1031	0.0925	G	0.5506	1.128
22	rs5771320	50482126	A	0.4072	0.39	C	0.5506	1.074
1	rs9782980	55273580	A	0.2758	0.2592	C	0.5507	1.088
6	rs2256543	29937833	A	0.2758	0.2592	G	0.5507	1.088
11	rs3740912	125891269	A	0.2758	0.2592	G	0.5507	1.088

14	rs13379043	74250126	G	0.2758	0.2592	A	0.5507	1.088
3	rs9826392	146874012	A	0.25	0.2658	G	0.5508	0.9206
11	rs1387144	27635319	A	0.384	0.4025	C	0.5509	0.9255
20	rs2281808	1610551	A	0.1985	0.185	G	0.5509	1.091
2	rs2030259	86683642	A	0.03351	0.04167	G	0.551	0.7973
9	rs1980889	91899245	G	0.4124	0.3942	A	0.551	1.079
11	rs150895565	124253124	G	0.03351	0.04167	A	0.551	0.7973
11	rs143831572	124267132	G	0.03351	0.04167	A	0.551	0.7973
17	rs4796603	39890715	T	0.1443	0.1325	A	0.551	1.104
19	rs2735839	51364623	A	0.4098	0.3925	G	0.551	1.075
22	rs1983609	44489868	A	0.03351	0.04167	G	0.551	0.7973
4	rs3755955	994414	A	0.1778	0.1933	G	0.5512	0.9025
4	rs6532197	90797301	G	0.2758	0.26	A	0.5512	1.084
5	rs17860502	147466001	A	0.1778	0.1933	G	0.5512	0.9025
13	rs9317527	66230607	C	0.1778	0.1933	A	0.5512	0.9025
6	rs2071556	32904601	A	0.3866	0.405	C	0.5513	0.9259
1	rs3766117	169527856	A	0.4124	0.395	G	0.5514	1.075
2	rs4927611	1460004	A	0.201	0.1865	C	0.5514	1.098
13	rs4886217	60789907	A	0.4124	0.395	G	0.5514	1.075
1	rs213060	21629447	C	0.3866	0.4042	A	0.5516	0.9291
4	rs1670534	1087265	A	0.4395	0.4207	G	0.5516	1.08
4	rs10025618	31627136	A	0.3866	0.4042	G	0.5516	0.9291
6	rs1265086	31109882	A	0.4175	0.3992	C	0.5518	1.079
6	rs9460083	170539872	G	0.4175	0.3992	A	0.5518	1.079
7	rs2074599	11676065	G	0.1263	0.1392	C	0.5518	0.8941
11	rs2114084	125617610	G	0.4149	0.3975	A	0.5518	1.075
20	rs7262903	3843027	A	0.1263	0.1392	C	0.5518	0.8941
2	rs1344814	221925929	A	0.3892	0.4067	G	0.552	0.9296
17	rs11867479	68090207	G	0.3892	0.4067	A	0.552	0.9296
1	rs10889205	40705726	T	0.06959	0.06167	A	0.5521	1.138
2	rs117271684	152521983	G	0.06959	0.06167	A	0.5521	1.138
3	rs28497577	123512627	A	0.06959	0.06167	C	0.5521	1.138
12	rs7302981	50537815	A	0.06959	0.06167	G	0.5521	1.138
17	rs3760423	10243717	G	0.06959	0.06167	A	0.5521	1.138
20	rs16991866	6093177	G	0.06959	0.06167	A	0.5521	1.138
6	rs7451713	46107752	G	0.4201	0.4017	C	0.5522	1.079
6	rs6926570	46135489	G	0.4175	0.4	A	0.5522	1.075
7	rs79931924	88964226	A	0.0567	0.06667	C	0.5522	0.8415
9	rs353785	89099362	A	0.0567	0.06667	G	0.5522	0.8415
11	rs59648931	124767067	G	0.4201	0.4017	A	0.5522	1.079
12	rs1991192	9500499	A	0.4201	0.4017	G	0.5522	1.079
17	rs9894480	65289798	A	0.4201	0.4017	G	0.5522	1.079
2	rs150878701	219374744	C	0.03351	0.0425	G	0.5523	0.781

3	rs7635311	16454480	A	0.4201	0.4023	G	0.5523	1.076
6	rs3117103	32349557	A	0.03351	0.0425	T	0.5523	0.781
8	rs78620801	133584612	G	0.03351	0.0425	A	0.5523	0.781
11	rs4910755	5372751	C	0.03351	0.0425	A	0.5523	0.781
11	rs7483122	5373006	G	0.03351	0.0425	A	0.5523	0.781
11	rs5006889	5373104	G	0.03351	0.0425	A	0.5523	0.781
11	rs7479477	5373111	A	0.03351	0.0425	G	0.5523	0.781
11	rs5006887	5373129	A	0.03351	0.0425	G	0.5523	0.781
11	rs5006886	5373170	G	0.03351	0.0425	C	0.5523	0.781
11	rs5006885	5373242	C	0.03351	0.0425	A	0.5523	0.781
11	rs5006884	5373251	A	0.03351	0.0425	G	0.5523	0.781
11	rs5006883	5373311	G	0.03351	0.0425	A	0.5523	0.781
11	rs5024042	5373562	A	0.03351	0.0425	C	0.5523	0.781
15	rs56079860	77407516	A	0.03351	0.0425	C	0.5523	0.781
15	rs35335169	77472951	G	0.03351	0.0425	A	0.5523	0.781
16	rs61729118	2030127	A	0.03351	0.0425	G	0.5523	0.781
19	rs149805314	9722005	A	0.03351	0.0425	G	0.5523	0.781
6	rs2844802	29948812	A	0.2784	0.2625	T	0.5524	1.084
6	rs2735067	29951106	G	0.2784	0.2625	A	0.5524	1.084
17	rs2071587	26851602	A	0.2784	0.2625	G	0.5524	1.084
1	exm2254132	99225627	G	0.4201	0.4025	A	0.5525	1.075
1	rs2019213	99225627	G	0.4201	0.4025	A	0.5525	1.075
6	rs3132682	30044388	C	0.4201	0.4025	G	0.5525	1.075
10	rs10899795	43869097	A	0.4201	0.4025	C	0.5525	1.075
11	rs2512276	124115370	G	0.4227	0.4042	C	0.5525	1.079
11	rs4268525	124121199	G	0.4227	0.4042	C	0.5525	1.079
11	rs2512167	124135438	G	0.4227	0.4042	A	0.5525	1.079
12	rs4363657	21368722	G	0.3918	0.4092	A	0.5525	0.93
20	rs6015510	58071371	A	0.3918	0.4092	G	0.5525	0.93
1	rs6702823	246755124	A	0.1469	0.1342	G	0.5526	1.111
6	rs75746803	41773726	G	0.04639	0.03853	C	0.5527	1.214
19	rs2195310	53645291	A	0.201	0.1875	G	0.5528	1.09
1	rs7530895	203260756	G	0.3943	0.4117	A	0.5529	0.9305
3	rs1464510	188112554	C	0.4227	0.405	A	0.5529	1.076
5	rs867822	56626359	A	0.4227	0.405	G	0.5529	1.076
6	rs2621326	32783896	G	0.4253	0.4067	A	0.5529	1.08
11	rs1007738	46849360	A	0.4253	0.4067	G	0.5529	1.08
12	rs36127550	133780309	A	0.1031	0.09333	C	0.553	1.117
6	rs11751198	31753526	A	0.0567	0.0675	G	0.5531	0.8304
16	rs4293393	20364588	G	0.0567	0.0675	A	0.5531	0.8304
16	rs13333226	20365654	G	0.0567	0.0675	A	0.5531	0.8304
17	rs1050784	39674641	A	0.0567	0.0675	G	0.5531	0.8304
19	rs1135062	45322744	G	0.0567	0.0675	A	0.5531	0.8304

5	rs4145748	52201722	A	0.1804	0.195	G	0.5532	0.9087
11	rs3816614	46890165	A	0.4253	0.4075	G	0.5532	1.076
11	rs2306033	46897446	G	0.4253	0.4075	A	0.5532	1.076
12	rs61764061	53238387	A	0.1804	0.195	G	0.5532	0.9087
12	rs10876360	53240025	A	0.1804	0.195	G	0.5532	0.9087
12	rs11170289	53242641	A	0.1804	0.195	G	0.5532	0.9087
4	rs34082815	156294333	G	0.08763	0.09917	A	0.5533	0.8725
7	rs145750007	299872	A	0.08763	0.09917	G	0.5533	0.8725
17	rs8075276	73257986	G	0.08763	0.09917	A	0.5533	0.8725
7	rs713598	141673345	C	0.3995	0.4175	G	0.5534	0.9281
1	rs74896528	28598287	A	0.02577	0.02083	G	0.5535	1.243
1	rs141722381	154931757	T	0.02577	0.02083	A	0.5535	1.243
1	rs12118933	176833497	G	0.02577	0.02083	C	0.5535	1.243
5	rs3734116	137517132	A	0.02577	0.02083	G	0.5535	1.243
6	rs61753605	57499035	G	0.02577	0.02083	A	0.5535	1.243
7	rs145069572	42005073	C	0.02577	0.02083	G	0.5535	1.243
8	rs6990223	73963866	A	0.02577	0.02083	C	0.5535	1.243
9	rs146932577	34627983	A	0.02577	0.02083	G	0.5535	1.243
10	rs149116714	102739993	A	0.02577	0.02083	C	0.5535	1.243
11	rs1550976	131320070	A	0.02577	0.02083	G	0.5535	1.243
12	rs3741923	6752776	G	0.02577	0.02083	A	0.5535	1.243
13	rs117237810	113333793	C	0.02577	0.02083	A	0.5535	1.243
14	rs17619	23237212	A	0.02577	0.02083	G	0.5535	1.243
15	rs4779816	31369123	A	0.02577	0.02083	G	0.5535	1.243
16	rs148423881	68105011	A	0.02577	0.02083	G	0.5535	1.243
19	rs7245949	9083143	A	0.02577	0.02083	G	0.5535	1.243
19	rs7245960	9083174	A	0.02577	0.02083	G	0.5535	1.243
19	rs1609459	9084216	G	0.02577	0.02083	A	0.5535	1.243
19	rs10402812	9086123	C	0.02577	0.02083	A	0.5535	1.243
19	rs3729841	55665410	A	0.02577	0.02083	G	0.5535	1.243
1	rs4579760	152141674	G	0.4021	0.4208	A	0.5536	0.9254
8	rs7842055	121244089	A	0.4021	0.4208	C	0.5536	0.9254
20	rs3810481	62193445	G	0.4021	0.4208	A	0.5536	0.9254
4	rs2286866	25759334	C	0.08763	0.1	G	0.5537	0.8644
6	rs28362675	32362521	A	0.08763	0.1	C	0.5537	0.8644
6	rs41441651	32363888	A	0.08763	0.1	G	0.5537	0.8644
6	rs41417449	32364011	G	0.08763	0.1	A	0.5537	0.8644
6	rs34423804	32364046	A	0.08763	0.1	T	0.5537	0.8644
6	rs78587369	32370927	A	0.08763	0.1	G	0.5537	0.8644
6	rs4746	38650628	C	0.08763	0.1	A	0.5537	0.8644
10	rs2296136	5929962	C	0.08763	0.1	G	0.5537	0.8644
1	rs12751100	1220954	G	0.4253	0.4083	A	0.5538	1.072
1	rs1131471	152079989	A	0.4021	0.42	C	0.5538	0.9286

1	rs6662450	152191709	G	0.4021	0.42	C	0.5538	0.9286
1	rs11204937	152193851	A	0.4021	0.42	G	0.5538	0.9286
2	rs2280289	238483729	G	0.4304	0.4125	A	0.5538	1.076
4	rs6553238	189759697	G	0.4021	0.42	A	0.5538	0.9286
6	rs6919202	99958818	A	0.4021	0.42	G	0.5538	0.9286
17	rs11871606	45732774	C	0.4253	0.4083	A	0.5538	1.072
11	rs12805648	7509566	A	0.1469	0.135	T	0.5539	1.103
12	rs2290788	114377835	G	0.1601	0.1467	A	0.5539	1.109
6	rs2107191	29434295	G	0.4046	0.4233	A	0.554	0.9258
11	rs3740809	89892250	C	0.4046	0.4233	A	0.554	0.9258
20	rs756529	48011008	G	0.4046	0.4233	A	0.554	0.9258
9	rs7023954	21816758	A	0.4046	0.4225	G	0.5541	0.929
18	rs1013696	31420010	G	0.4278	0.4108	A	0.5541	1.072
1	rs1738475	23536891	C	0.4072	0.4258	G	0.5543	0.9262
1	rs627304	23537555	G	0.4072	0.4258	A	0.5543	0.9262
2	rs6713162	152496526	A	0.4072	0.4258	G	0.5543	0.9262
2	rs6733811	152546145	G	0.4072	0.4258	A	0.5543	0.9262
3	rs7648947	43174229	G	0.4072	0.4258	A	0.5543	0.9262
4	rs6814407	81058566	A	0.4072	0.4258	C	0.5543	0.9262
5	rs2286394	140048544	A	0.2577	0.275	G	0.5543	0.9154
6	rs574914	31819376	A	0.2577	0.275	G	0.5543	0.9154
12	rs1801046	7242299	A	0.4072	0.4258	G	0.5543	0.9262
5	rs1345981	51655244	G	0.4304	0.4133	A	0.5544	1.073
12	rs11173699	61403454	C	0.4304	0.4133	A	0.5544	1.073
17	rs2257020	74076415	A	0.4304	0.4133	G	0.5544	1.073
3	rs6797429	193080414	G	0.4356	0.4175	C	0.5545	1.077
6	rs10498810	56126903	C	0.4072	0.425	A	0.5545	0.9294
11	rs324299	29680576	A	0.4356	0.4175	G	0.5545	1.077
9	rs7863519	113208250	C	0.4098	0.4283	A	0.5546	0.9267
2	rs3821236	191902758	A	0.433	0.4158	G	0.5547	1.073
4	rs236985	88008782	A	0.4381	0.42	G	0.5547	1.077
5	rs2354124	1721485	G	0.2835	0.2675	A	0.5547	1.084
9	rs8176720	136132873	G	0.433	0.4158	A	0.5547	1.073
14	rs3748361	21796784	G	0.433	0.4158	C	0.5547	1.073
15	rs10518733	53940307	C	0.433	0.4158	A	0.5547	1.073
16	rs8053800	29043450	A	0.433	0.4158	C	0.5547	1.073
18	rs1217562	55091293	A	0.2835	0.2675	G	0.5547	1.084
20	rs4813048	11169603	A	0.433	0.4158	G	0.5547	1.073
2	rs12693199	152859992	G	0.4098	0.4275	A	0.5549	0.9298
3	rs2132240	184816323	G	0.4098	0.4275	A	0.5549	0.9298
5	rs10941694	45197779	A	0.1289	0.1425	G	0.5549	0.8902
19	rs10104	13885484	G	0.4356	0.4183	A	0.5549	1.073
19	rs3746297	43772302	A	0.4407	0.4224	G	0.5549	1.078

1	rs11118346	219743719	G	0.4407	0.4225	A	0.555	1.077
8	rs13263959	142632315	A	0.4124	0.4308	G	0.555	0.9271
9	rs10818708	125289521	G	0.1057	0.095	A	0.5551	1.126
12	rs17554249	59557430	A	0.1057	0.095	G	0.5551	1.126
1	rs1988163	209581483	A	0.4381	0.4208	C	0.5552	1.073
2	rs2229621	75099477	A	0.183	0.1983	T	0.5552	0.9053
6	rs3734905	169958982	A	0.183	0.1983	G	0.5552	0.9053
1	rs7536561	180243524	A	0.4433	0.425	G	0.5553	1.077
3	rs231983	172236440	A	0.183	0.1975	C	0.5553	0.9101
13	rs1927404	77947207	A	0.4433	0.425	G	0.5553	1.077
1	rs12037217	85742023	A	0.07216	0.06333	C	0.5554	1.15
2	rs2631941	29981286	G	0.2603	0.2767	A	0.5554	0.9201
2	rs3732240	71366957	A	0.4407	0.4233	C	0.5554	1.073
6	rs2270172	30592414	G	0.07216	0.06333	A	0.5554	1.15
7	rs7784776	46620145	A	0.2603	0.2767	G	0.5554	0.9201
8	rs1532278	27466315	A	0.2603	0.2767	G	0.5554	0.9201
11	rs3740881	130010313	A	0.07216	0.06333	G	0.5554	1.15
12	rs12313946	68961584	C	0.4407	0.4233	A	0.5554	1.073
17	rs78806449	3981290	G	0.07216	0.06333	A	0.5554	1.15
19	rs10422961	35422808	A	0.2603	0.2767	G	0.5554	0.9201
1	rs28375469	46195375	G	0.2835	0.2683	A	0.5555	1.079
4	rs2877558	171743216	A	0.2835	0.2683	G	0.5555	1.079
6	rs11244	32780724	A	0.2835	0.2683	G	0.5555	1.079
10	rs7097078	71084361	G	0.2835	0.2683	A	0.5555	1.079
12	rs1503767	118889488	A	0.4459	0.4274	C	0.5555	1.078
1	rs3762271	197070442	A	0.1495	0.1367	C	0.5556	1.11
1	rs41310927	197070697	G	0.1495	0.1367	A	0.5556	1.11
2	rs1045881	50148972	A	0.1495	0.1367	G	0.5556	1.11
6	rs9342825	71962755	G	0.1495	0.1367	A	0.5556	1.11
6	rs1539312	158743188	A	0.4175	0.4358	G	0.5556	0.9279
12	rs11169172	50232169	A	0.4149	0.4325	T	0.5556	0.9306
12	rs735396	121438844	A	0.4175	0.4358	G	0.5556	0.9279
12	rs1169310	121439433	G	0.4175	0.4358	A	0.5556	0.9279
16	rs2303262	82203758	G	0.1495	0.1367	A	0.5556	1.11
17	rs8075017	39881289	C	0.1495	0.1367	G	0.5556	1.11
19	rs4804108	9555855	A	0.4459	0.4275	G	0.5556	1.078
6	rs9275596	32681631	G	0.2062	0.1917	A	0.5557	1.095
8	rs3812475	125463250	G	0.4433	0.4258	A	0.5557	1.074
14	rs17094077	74401038	G	0.04639	0.03917	A	0.5557	1.193
15	rs17565841	27997247	A	0.04639	0.03917	G	0.5557	1.193
21	rs11203203	43836186	A	0.04639	0.03917	G	0.5557	1.193
11	rs7106927	5491183	C	0.4485	0.43	A	0.5558	1.078
18	rs1398217	44752238	C	0.4485	0.43	G	0.5558	1.078

16	rs11076507	49927513	G	0.4175	0.435	A	0.5559	0.931
18	rs12605558	26719357	G	0.4227	0.4408	A	0.5561	0.9287
6	rs1246183	156969532	G	0.4201	0.4375	A	0.5562	0.9314
1	rs2232842	202935911	G	0.4536	0.435	A	0.5563	1.078
17	rs3744581	11651057	G	0.4253	0.4441	A	0.5563	0.9263
1	rs4654168	246078476	G	0.451	0.4333	A	0.5564	1.074
5	rs2172058	68366457	A	0.451	0.4333	G	0.5564	1.074
12	rs904654	120189888	A	0.4253	0.4433	G	0.5564	0.9291
7	rs258684	81735015	G	0.4227	0.44	A	0.5565	0.9318
6	rs2240058	30310500	G	0.2861	0.2708	A	0.5566	1.079
9	rs3001115	113403846	G	0.4536	0.4358	A	0.5566	1.075
10	rs3013236	124330427	G	0.2062	0.1925	A	0.5566	1.09
11	rs592644	74053547	G	0.2861	0.2708	A	0.5566	1.079
21	rs2830088	27514740	G	0.2861	0.2708	A	0.5566	1.079
5	rs2544773	96513471	C	0.4278	0.4457	G	0.5567	0.9298
7	rs12669881	116645880	G	0.4278	0.4458	A	0.5567	0.9294
10	rs10906115	12314997	G	0.4278	0.4458	A	0.5567	0.9294
10	rs11101114	50569286	G	0.4278	0.4458	A	0.5567	0.9294
21	rs2832657	31588676	C	0.4663	0.4482	A	0.5567	1.076
1	rs201425552	12837720	A	0.07216	0.06376	G	0.5568	1.142
3	rs3204849	46450070	A	0.4253	0.4425	T	0.5568	0.9322
5	rs682748	17148911	G	0.4304	0.4492	A	0.5568	0.9267
12	rs9651976	86031486	A	0.4304	0.4492	G	0.5568	0.9267
18	rs1790706	28683238	A	0.4304	0.4492	G	0.5568	0.9267
1	rs17409304	86900372	G	0.1495	0.1375	C	0.5569	1.102
1	rs4915221	201166383	G	0.1495	0.1375	A	0.5569	1.102
21	rs9978843	20984026	G	0.4588	0.4407	A	0.5569	1.076
13	rs308592	62664851	A	0.433	0.4517	G	0.557	0.9271
15	rs7168365	53805825	A	0.433	0.4517	C	0.557	0.9271
1	rs12118628	159409884	A	0.1856	0.2008	G	0.5572	0.9067
2	rs1445653	155661432	G	0.4562	0.4391	A	0.5572	1.072
4	rs6840796	144617397	T	0.1856	0.2008	A	0.5572	0.9067
6	rs2071552	32806461	G	0.4613	0.4433	A	0.5572	1.075
18	rs1785934	33718326	C	0.1856	0.2008	A	0.5572	0.9067
6	rs9272219	32602269	A	0.1856	0.2	C	0.5573	0.9114
7	rs13234724	150325175	A	0.1856	0.2	G	0.5573	0.9114
11	rs2298475	126278203	G	0.1856	0.2	A	0.5573	0.9114
12	rs3825195	54972992	G	0.4562	0.4392	A	0.5573	1.071
21	rs61743204	34926043	A	0.1856	0.2	G	0.5573	0.9114
2	rs10175880	118924569	A	0.4639	0.4458	G	0.5574	1.076
5	rs638333	72419267	G	0.1057	0.09583	A	0.5574	1.115
2	rs7571816	233077064	A	0.4356	0.4533	G	0.5575	0.9306
12	rs1129593	105380152	A	0.4381	0.4567	C	0.5575	0.9278

17	rs56152251	64280153	G	0.4665	0.4483	A	0.5575	1.076
1	rs3903683	32672908	C	0.2088	0.1942	A	0.5576	1.095
4	rs2407221	152212603	A	0.2088	0.1942	C	0.5576	1.095
11	rs2500017	5068137	A	0.2887	0.2733	G	0.5576	1.079
11	rs2500018	5068177	A	0.2887	0.2733	T	0.5576	1.079
15	rs4932217	89902032	C	0.2887	0.2733	A	0.5576	1.079
9	rs7865489	109312922	A	0.4407	0.4592	C	0.5577	0.9282
11	rs11235819	73318285	C	0.4691	0.4508	A	0.5577	1.076
12	rs7958311	121605355	A	0.4613	0.4442	G	0.5577	1.072
12	rs3751143	121622304	C	0.2655	0.2825	A	0.5577	0.9179
14	rs4903096	40329432	C	0.4381	0.4558	A	0.5577	0.9309
1	rs3820071	15808767	G	0.4433	0.4617	A	0.5578	0.9285
8	rs13280933	60308317	A	0.2655	0.2817	G	0.5578	0.9217
9	rs963774	97959500	C	0.4639	0.4467	A	0.5578	1.072
1	rs2179763	84295861	A	0.4407	0.4583	G	0.5579	0.9313
3	rs12632177	183056598	A	0.1314	0.145	G	0.5579	0.8924
14	rs45478391	44975511	A	0.09021	0.1017	G	0.5579	0.8761
15	rs1051168	85200520	A	0.1314	0.145	C	0.5579	0.8924
17	rs758642	3786907	A	0.1314	0.145	G	0.5579	0.8924
18	rs2564486	57246293	A	0.1314	0.145	C	0.5579	0.8924
20	rs2273086	48273174	A	0.09021	0.1017	G	0.5579	0.8761
2	rs11550699	233537125	A	0.4459	0.4642	G	0.558	0.9289
3	rs779506	191412597	A	0.4742	0.4558	G	0.558	1.077
7	rs13928	44153780	G	0.2912	0.275	A	0.558	1.083
8	rs2272736	42177163	A	0.1314	0.1442	G	0.558	0.8984
14	rs1951082	27260043	A	0.1314	0.1442	T	0.558	0.8984
19	rs10410943	8808900	A	0.1314	0.1442	G	0.558	0.8984
19	rs2340550	8808942	A	0.1314	0.1442	G	0.558	0.8984
19	rs10420543	35897918	A	0.4665	0.4492	G	0.558	1.072
7	rs2240026	94903420	A	0.4716	0.4542	G	0.5582	1.073
8	rs12679254	74274191	A	0.4768	0.4583	G	0.5582	1.077
10	rs10748927	108543337	G	0.4768	0.4583	A	0.5582	1.077
22	rs13815	43529314	G	0.4716	0.4542	C	0.5582	1.073
1	rs2274254	117568200	A	0.09021	0.1025	G	0.5583	0.8682
1	rs2061690	154919080	G	0.09021	0.1025	A	0.5583	0.8682
1	rs1009668	226040404	A	0.09021	0.1025	G	0.5583	0.8682
4	rs1845344	120962385	A	0.09021	0.1025	C	0.5583	0.8682
6	rs9443804	81315597	G	0.4794	0.4608	A	0.5583	1.077
19	rs8103697	9354461	A	0.4794	0.4608	C	0.5583	1.077
19	rs7259660	30568421	G	0.4794	0.4608	A	0.5583	1.077
5	rs2577539	128301211	G	0.09021	0.1027	A	0.5584	0.8666
8	rs2270637	20036827	C	0.2088	0.195	G	0.5584	1.089
8	rs10435683	143413136	G	0.4459	0.4633	A	0.5584	0.932

10	rs2184898	119418104	A	0.2088	0.195	G	0.5584	1.089
11	rs2186564	77583266	A	0.2088	0.195	G	0.5584	1.089
17	rs7225835	8431453	G	0.4459	0.4633	A	0.5584	0.932
17	rs1053739	43183108	A	0.5104	0.4917	G	0.5584	1.078
2	rs13009279	29222070	G	0.4536	0.4717	A	0.5585	0.9299
12	rs4516060	125438516	A	0.1521	0.1392	G	0.5585	1.109
4	rs949807	36398436	A	0.4485	0.4658	G	0.5586	0.9324
6	rs9347144	167079111	G	0.4485	0.4658	A	0.5586	0.9324
7	rs10241412	7873385	C	0.4485	0.4658	A	0.5586	0.9324
8	rs12550664	14879087	A	0.4794	0.4617	C	0.5586	1.074
8	rs4316158	123464305	A	0.4485	0.4658	G	0.5586	0.9324
14	rs11627901	92013263	A	0.4845	0.4658	C	0.5586	1.078
3	rs3762735	101283792	G	0.1528	0.14	C	0.5587	1.108
6	rs2327429	134209837	G	0.4588	0.4775	A	0.5587	0.9275
11	rs11036212	5221825	A	0.4562	0.4742	G	0.5587	0.9303
21	rs643608	44768562	G	0.482	0.4642	A	0.5587	1.074
22	rs200488681	24226213	G	0.01295	0.009182	A	0.5587	1.416
1	rs10873862	88112646	A	0.268	0.285	C	0.5588	0.9187
7	rs6943837	3147020	G	0.4588	0.4767	A	0.5588	0.9306
8	rs416829	94167259	A	0.4613	0.48	G	0.5588	0.9278
20	rs6068020	50559652	A	0.4613	0.48	G	0.5588	0.9278
1	rs9286879	172862234	A	0.07216	0.06417	G	0.5589	1.134
2	rs13394619	11727507	G	0.4639	0.4825	A	0.5589	0.9282
7	rs11768079	4825314	A	0.07216	0.06417	G	0.5589	1.134
17	rs35819807	61623052	A	0.07216	0.06417	G	0.5589	1.134
18	rs16975293	39346601	G	0.07216	0.06417	A	0.5589	1.134
19	rs737337	11347493	G	0.268	0.2842	A	0.5589	0.9225
19	rs1806931	15839365	G	0.4639	0.4825	A	0.5589	0.9282
19	rs2240227	15852242	C	0.4639	0.4825	A	0.5589	0.9282
4	rs2437323	40778162	A	0.4613	0.4792	C	0.559	0.9309
5	rs1588265	59369794	A	0.2938	0.2775	G	0.559	1.083
11	rs12288731	91260969	A	0.4923	0.4742	G	0.559	1.075
1	rs15702	212911836	A	0.4716	0.49	G	0.5591	0.9291
1	rs7531705	220565331	G	0.5	0.4817	A	0.5591	1.076
2	rs13008689	8530256	A	0.4562	0.4733	G	0.5591	0.9334
2	rs713586	25158008	G	0.4639	0.4817	A	0.5591	0.9313
2	rs6758435	71246439	G	0.4974	0.4792	A	0.5591	1.076
4	rs6825176	150990695	G	0.5	0.4817	A	0.5591	1.076
5	rs11133784	14239606	C	0.4691	0.4875	A	0.5591	0.9288
7	rs10265222	35332117	A	0.4562	0.4733	G	0.5591	0.9334
7	rs976714	82581859	G	0.4562	0.4733	A	0.5591	0.9334
9	rs10118083	12961631	A	0.5	0.4817	C	0.5591	1.076
10	rs2503052	36727213	G	0.4716	0.49	A	0.5591	0.9291

10	rs4933800	83418056	G	0.4639	0.4817	A	0.5591	0.9313
11	rs1592586	50630255	C	0.5	0.4817	A	0.5591	1.076
11	rs634552	75282052	A	0.4691	0.4875	C	0.5591	0.9288
11	rs55969429	134889485	G	0.5	0.4817	A	0.5591	1.076
12	rs10774171	3868309	A	0.4562	0.4733	G	0.5591	0.9334
12	rs25644	121666646	G	0.1881	0.2033	A	0.5591	0.908
18	rs35012773	3262784	G	0.4562	0.4733	A	0.5591	0.9334
21	rs2826962	23083278	A	0.4691	0.4875	G	0.5591	0.9288
1	rs2864884	23945443	A	0.5077	0.4892	G	0.5592	1.077
4	rs6826089	7266856	C	0.5103	0.4917	A	0.5592	1.077
4	rs1433640	66773365	G	0.4742	0.4925	A	0.5592	0.9294
5	rs1019747	5146377	G	0.4665	0.4842	A	0.5592	0.9316
5	rs840398	34016023	G	0.5129	0.4942	A	0.5592	1.078
5	rs3805495	40755568	G	0.4742	0.4925	A	0.5592	0.9294
5	rs1448044	44296986	G	0.5129	0.4942	A	0.5592	1.078
6	rs1570843	84520520	A	0.4742	0.4925	G	0.5592	0.9294
9	rs7854450	24998417	G	0.4742	0.4925	A	0.5592	0.9294
10	rs35441642	14976727	C	0.1881	0.2025	G	0.5592	0.9127
12	rs16942745	113758166	A	0.1881	0.2025	G	0.5592	0.9127
17	rs12103753	12171195	C	0.5129	0.4942	A	0.5592	1.078
19	rs4806015	34865551	A	0.1881	0.2025	G	0.5592	0.9127
1	rs142743253	36552858	A	0.01289	0.009167	C	0.5593	1.411
1	rs6692611	43919413	A	0.4691	0.4867	G	0.5593	0.9319
1	rs147691168	46073517	A	0.01289	0.009167	G	0.5593	1.411
1	exm2252187	55060288	A	0.01289	0.009167	G	0.5593	1.411
1	rs300283	55060288	A	0.01289	0.009167	G	0.5593	1.411
1	rs201589656	84415640	G	0.01289	0.009167	A	0.5593	1.411
1	rs843971	153277423	G	0.4794	0.4975	A	0.5593	0.93
1	rs147929171	158746830	A	0.01289	0.009167	T	0.5593	1.411
1	rs12720691	186948448	C	0.01289	0.009167	A	0.5593	1.411
1	rs142206068	203670013	A	0.01289	0.009167	C	0.5593	1.411
1	rs148860285	206711608	A	0.01289	0.009167	G	0.5593	1.411
1	rs146082990	223285901	A	0.01289	0.009167	G	0.5593	1.411
1	rs201294185	228612837	A	0.01289	0.009167	G	0.5593	1.411
2	rs34996177	32641040	A	0.01289	0.009167	G	0.5593	1.411
2	rs9309464	72533963	G	0.01289	0.009167	A	0.5593	1.411
2	exm201705	72707874	G	0.01289	0.009167	A	0.5593	1.411
2	rs653220	72707874	G	0.01289	0.009167	A	0.5593	1.411
2	rs78461888	220472831	A	0.01289	0.009167	G	0.5593	1.411
2	rs34946978	234676872	A	0.01289	0.009167	G	0.5593	1.411
3	rs142953386	33877696	G	0.01289	0.009167	A	0.5593	1.411
3	rs144341991	37323711	A	0.01289	0.009167	G	0.5593	1.411
3	rs6764543	55281622	G	0.4845	0.4675	A	0.5593	1.071

3	rs201905772	100964807	G	0.01289	0.009167	C	0.5593	1.411
3	rs76363649	113697179	A	0.01289	0.009167	C	0.5593	1.411
4	rs200075138	3257667	A	0.01289	0.009167	C	0.5593	1.411
4	rs140011683	68488583	A	0.01289	0.009167	G	0.5593	1.411
4	rs11931074	90639515	C	0.4768	0.495	A	0.5593	0.9297
5	rs145293565	44809408	T	0.01289	0.009167	A	0.5593	1.411
6	rs1480380	32913246	A	0.01289	0.009167	G	0.5593	1.411
6	rs41555121	32917411	A	0.01289	0.009167	G	0.5593	1.411
6	rs6926628	32917498	G	0.01289	0.009167	C	0.5593	1.411
6	rs9276934	32933222	G	0.01289	0.009167	A	0.5593	1.411
6	rs10484568	32935751	G	0.01289	0.009167	A	0.5593	1.411
6	rs3918143	32945625	A	0.01289	0.009167	G	0.5593	1.411
6	rs192410865	155757671	A	0.01289	0.009167	G	0.5593	1.411
7	rs199820945	50799974	G	0.01289	0.009167	A	0.5593	1.411
8	rs118016633	70744167	A	0.01289	0.009167	G	0.5593	1.411
8	rs139157188	100974669	G	0.01289	0.009167	A	0.5593	1.411
9	rs117748903	18928508	A	0.01289	0.009167	G	0.5593	1.411
9	rs143449578	79936426	A	0.01289	0.009167	G	0.5593	1.411
10	rs201331459	13043224	G	0.01289	0.009167	A	0.5593	1.411
10	rs187685058	13251117	A	0.01289	0.009167	G	0.5593	1.411
10	rs2306236	27048077	G	0.01289	0.009167	C	0.5593	1.411
10	rs2273903	70098948	G	0.01289	0.009167	A	0.5593	1.411
10	rs7914558	104775908	G	0.4794	0.4975	A	0.5593	0.93
10	rs1891110	124610027	A	0.4691	0.4867	G	0.5593	0.9319
11	rs138740326	28119403	G	0.01289	0.009167	A	0.5593	1.411
11	rs144349650	55432976	C	0.01289	0.009167	G	0.5593	1.411
11	rs145672238	57080882	A	0.01289	0.009167	G	0.5593	1.411
12	rs139938131	6571250	G	0.01289	0.009167	A	0.5593	1.411
13	rs2812234	51287814	G	0.4845	0.4675	A	0.5593	1.071
13	rs3742298	53307362	G	0.01289	0.009167	A	0.5593	1.411
13	rs1415160	54175298	C	0.4768	0.495	A	0.5593	0.9297
14	rs201144028	23344609	A	0.01289	0.009167	G	0.5593	1.411
15	rs2278857	43632549	A	0.4871	0.47	G	0.5593	1.071
16	rs150079802	4410985	A	0.01289	0.009167	C	0.5593	1.411
16	rs141592630	30594109	A	0.01289	0.009167	C	0.5593	1.411
16	rs12933808	76532583	A	0.01289	0.009167	G	0.5593	1.411
17	rs200558188	56384217	A	0.01289	0.009167	G	0.5593	1.411
17	rs79861199	77080700	A	0.01289	0.009167	G	0.5593	1.411
19	rs201858159	15905357	A	0.01289	0.009167	G	0.5593	1.411
19	rs61745313	15918349	A	0.01289	0.009167	G	0.5593	1.411
19	rs9524	51517731	A	0.4794	0.4975	G	0.5593	0.93
19	rs56353414	56047540	A	0.01289	0.009167	G	0.5593	1.411
19	rs79063277	58945844	A	0.01289	0.009167	C	0.5593	1.411

20	rs181950038	34618331	A	0.01289	0.009167	G	0.5593	1.411
22	rs139809906	37770812	A	0.01289	0.009167	C	0.5593	1.411
22	rs139686326	51159778	A	0.01289	0.009167	G	0.5593	1.411
1	rs2235937	29631909	A	0.4845	0.5025	G	0.5594	0.9306
1	rs4846641	216856115	A	0.4948	0.4775	G	0.5594	1.072
2	rs1131296	238243292	G	0.4845	0.5025	A	0.5594	0.9306
7	rs3812265	135048804	G	0.4974	0.48	A	0.5594	1.072
7	rs6459804	157510195	A	0.4948	0.4775	G	0.5594	1.072
10	rs10823607	72500763	A	0.05928	0.06917	G	0.5594	0.848
12	rs150136669	46246206	A	0.05928	0.06917	C	0.5594	0.848
13	rs9508801	31135492	A	0.4948	0.4775	G	0.5594	1.072
14	rs11626701	94954368	A	0.5103	0.4925	G	0.5594	1.074
15	rs7181789	23043896	A	0.4974	0.48	G	0.5594	1.072
16	rs8047557	3261955	A	0.4897	0.4725	G	0.5594	1.071
17	rs2108978	19861458	A	0.2113	0.1967	G	0.5594	1.095
19	rs762562	45912343	G	0.5103	0.4925	A	0.5594	1.074
19	rs2336219	45912406	A	0.5103	0.4925	G	0.5594	1.074
2	rs13022344	202264156	A	0.5	0.4825	G	0.5595	1.073
8	rs6469084	107908032	A	0.5077	0.49	G	0.5595	1.074
9	rs3739524	79320095	C	0.5077	0.49	A	0.5595	1.074
10	rs10904854	16999286	A	0.5052	0.4875	G	0.5595	1.073
12	rs3842936	58801027	A	0.5026	0.485	G	0.5595	1.073
12	rs704124	90826178	G	0.5	0.4825	A	0.5595	1.073
14	rs12435266	61652860	G	0.5052	0.4875	A	0.5595	1.073
18	rs273695	23178593	A	0.4742	0.4917	C	0.5595	0.9325
20	rs926392	37690464	A	0.4742	0.4917	G	0.5595	0.9325
2	rs6712141	6990033	G	0.1082	0.0975	A	0.5596	1.124
3	rs12106890	141519874	A	0.1082	0.0975	G	0.5596	1.124
4	rs356220	90641340	G	0.4768	0.4942	A	0.5596	0.9328
5	rs9313307	165553143	A	0.1082	0.0975	C	0.5596	1.124
6	rs6910071	32282854	G	0.1082	0.0975	A	0.5596	1.124
7	rs2465185	68225416	A	0.4768	0.4942	G	0.5596	0.9328
9	rs55764262	136131539	G	0.01289	0.009182	A	0.5596	1.409
10	rs11812567	60548276	A	0.4768	0.4942	G	0.5596	0.9328
12	rs60683621	55863224	C	0.4768	0.4942	G	0.5596	0.9328
4	rs13109457	155514879	G	0.4794	0.4967	A	0.5597	0.9332
6	rs3734313	76640781	A	0.2938	0.2783	G	0.5597	1.079
12	rs4447263	125434580	G	0.1521	0.14	A	0.5597	1.102
19	rs11666267	17660300	A	0.2938	0.2783	G	0.5597	1.079
1	rs6677074	116278503	A	0.482	0.4992	C	0.5598	0.9335
2	rs12711538	121747406	A	0.4845	0.5017	G	0.5599	0.9338
4	rs7681423	155542248	G	0.4845	0.5017	A	0.5599	0.9338
17	rs9915645	812534	A	0.4845	0.5017	G	0.5599	0.9338

1	rs150014958	152058192	A	0.05928	0.07	G	0.5601	0.8372
10	rs17112076	98469693	A	0.2964	0.28	G	0.5601	1.083
15	rs2306024	68628049	C	0.05928	0.07	A	0.5601	0.8372
14	rs7157599	100625902	G	0.2706	0.2863	A	0.5602	0.9249
19	rs527221	46275976	G	0.2113	0.1975	C	0.5602	1.089
10	rs703479	106865955	G	0.2964	0.2808	A	0.5607	1.079
1	rs11210969	44595047	A	0.1907	0.2058	T	0.5609	0.9093
1	rs16833953	236740135	G	0.01804	0.02417	A	0.5609	0.7419
3	rs2271207	9055458	G	0.01804	0.02417	A	0.5609	0.7419
6	rs1341239	22304204	A	0.01804	0.02417	C	0.5609	0.7419
7	rs74734392	34085921	A	0.134	0.1475	G	0.5609	0.8945
8	rs12549018	133918963	A	0.01804	0.02417	G	0.5609	0.7419
9	rs12352254	2729686	C	0.01804	0.02417	G	0.5609	0.7419
10	rs117503332	8019223	A	0.01804	0.02417	G	0.5609	0.7419
10	rs118153230	34630570	A	0.01804	0.02417	G	0.5609	0.7419
10	rs5015480	94465559	G	0.1907	0.2058	A	0.5609	0.9093
11	rs7944584	47336320	T	0.01804	0.02417	A	0.5609	0.7419
12	rs1860967	9013755	G	0.1907	0.2058	A	0.5609	0.9093
14	rs58154675	64691744	A	0.01804	0.02417	G	0.5609	0.7419
15	rs16977594	57839587	G	0.01804	0.02417	A	0.5609	0.7419
17	rs690844	76497920	C	0.01804	0.02417	A	0.5609	0.7419
19	rs1133893	8486884	A	0.1907	0.2058	G	0.5609	0.9093
19	rs78052894	10225370	A	0.01804	0.02417	G	0.5609	0.7419
19	rs79279971	36674347	G	0.01804	0.02417	A	0.5609	0.7419
1	rs12073329	6272078	A	0.04639	0.04	G	0.561	1.168
2	rs3749002	168106610	A	0.04639	0.04	G	0.561	1.168
2	rs143084183	168107035	G	0.04639	0.04	A	0.561	1.168
3	rs9869263	190030680	A	0.134	0.1467	G	0.561	0.9004
11	rs2896526	18419806	G	0.04639	0.04	A	0.561	1.168
17	rs79657649	505105	G	0.04639	0.04	A	0.561	1.168
19	rs11557488	11558275	A	0.04639	0.04	G	0.561	1.168
22	rs80055673	31494813	G	0.04639	0.04	C	0.561	1.168
1	rs397260	49133886	A	0.299	0.2825	G	0.5611	1.083
1	rs4132509	243943084	A	0.2732	0.2892	C	0.5611	0.924
2	rs2116665	157406249	G	0.2139	0.1992	A	0.5612	1.094
6	rs3131635	31476134	A	0.1546	0.1418	G	0.5612	1.107
10	rs1800450	54531235	A	0.2139	0.1992	G	0.5612	1.094
11	rs4014195	65506822	C	0.1907	0.205	G	0.5612	0.9139
5	rs1864182	81549240	C	0.04639	0.04007	A	0.5614	1.166
5	rs118093952	141022569	A	0.04639	0.04007	G	0.5614	1.166
9	rs74696187	134350851	G	0.04639	0.04007	A	0.5614	1.166
6	rs118070529	146243916	G	0.1082	0.09833	A	0.5617	1.113
20	rs2762934	52771261	A	0.1082	0.09833	G	0.5617	1.113

20	rs259956	57829301	A	0.1082	0.09833	G	0.5617	1.113
1	rs61823162	159785413	A	0.01804	0.025	G	0.5618	0.7165
1	rs139593986	177911130	A	0.01804	0.025	G	0.5618	0.7165
2	rs60240337	242140745	A	0.01804	0.025	T	0.5618	0.7165
4	rs138306746	2940921	G	0.01804	0.025	A	0.5618	0.7165
4	rs144142828	48549607	G	0.01804	0.025	A	0.5618	0.7165
6	rs117951780	31084034	A	0.01804	0.025	G	0.5618	0.7165
7	rs35683659	141429371	C	0.01804	0.025	A	0.5618	0.7165
7	rs140118386	149504070	A	0.01804	0.025	G	0.5618	0.7165
8	rs75319208	125115444	A	0.01804	0.025	G	0.5618	0.7165
9	rs75251304	33986805	G	0.01804	0.025	A	0.5618	0.7165
12	rs145639855	51354842	A	0.01804	0.025	G	0.5618	0.7165
12	rs11065611	110704487	A	0.01804	0.025	G	0.5618	0.7165
16	rs144692165	90142260	A	0.01804	0.025	G	0.5618	0.7165
17	rs2271233	6704121	A	0.01804	0.025	G	0.5618	0.7165
17	rs141472706	78196475	C	0.01804	0.025	A	0.5618	0.7165
19	rs117216773	53958503	A	0.01804	0.025	C	0.5618	0.7165
2	rs11686946	128394955	G	0.2139	0.2	A	0.562	1.089
2	rs76452346	235951752	G	0.01804	0.02517	A	0.562	0.7116
4	rs3736265	23814707	A	0.2139	0.2	G	0.562	1.089
2	rs7567288	134434824	G	0.07474	0.06583	A	0.5622	1.146
5	rs41271083	43653243	A	0.09278	0.1042	G	0.5624	0.8795
14	rs61742642	76964655	A	0.09278	0.1042	G	0.5624	0.8795
3	rs36052974	118621664	A	0.1546	0.1425	C	0.5625	1.101
6	rs2523408	29778109	C	0.1546	0.1425	A	0.5625	1.101
2	rs4973318	231222675	G	0.09278	0.105	A	0.5627	0.8718
4	rs72613147	87809020	C	0.1933	0.2087	G	0.5627	0.9086
6	rs3778638	31092124	A	0.09278	0.105	G	0.5627	0.8718
6	rs11155242	142691549	C	0.09278	0.105	A	0.5627	0.8718
8	rs7460258	32959945	A	0.3015	0.2858	G	0.5627	1.079
20	rs7508949	20033367	C	0.09278	0.105	G	0.5627	0.8718
2	rs1042031	21225753	A	0.03608	0.04417	G	0.5628	0.8101
2	rs1432273	166797646	G	0.1933	0.2083	A	0.5628	0.9105
3	rs3749383	4738837	G	0.03608	0.04417	A	0.5628	0.8101
4	rs1047626	42003671	A	0.03608	0.04417	G	0.5628	0.8101
9	rs2841498	87930045	A	0.03608	0.04417	C	0.5628	0.8101
9	rs10991231	107112483	G	0.1933	0.2083	A	0.5628	0.9105
14	rs1544317	107281980	G	0.1933	0.2083	A	0.5628	0.9105
18	rs62621182	72244208	A	0.03608	0.04417	G	0.5628	0.8101
19	rs58239286	53344919	C	0.03608	0.04417	A	0.5628	0.8101
19	rs565791	35719106	G	0.1933	0.2075	A	0.563	0.9152
6	rs2244546	31435833	G	0.09326	0.1058	C	0.5631	0.869
21	rs7278930	37450210	G	0.3041	0.2875	A	0.5631	1.083

3	rs1152846	188420897	G	0.2784	0.2942	A	0.5633	0.9255
6	rs3749903	42992825	C	0.2784	0.2942	G	0.5633	0.9255
8	rs10492294	129246415	G	0.1366	0.15	A	0.5637	0.8965
1	rs2154319	41745770	G	0.03608	0.045	A	0.5638	0.7944
1	rs4839469	116206423	A	0.1108	0.1	G	0.5638	1.122
1	rs12143301	169823790	C	0.03608	0.045	G	0.5638	0.7944
1	rs12135726	169824104	A	0.03608	0.045	G	0.5638	0.7944
2	rs6733379	34480075	C	0.03608	0.045	A	0.5638	0.7944
2	rs2293079	220100787	A	0.03608	0.045	G	0.5638	0.7944
3	exm2255698	132409455	A	0.03608	0.045	G	0.5638	0.7944
3	rs16839515	132409455	A	0.03608	0.045	G	0.5638	0.7944
5	rs34879141	55396074	A	0.002618	0.001667	G	0.5638	1.572
6	rs117160266	32097475	G	0.03608	0.045	A	0.5638	0.7944
8	rs79332351	133045399	G	0.1108	0.1	A	0.5638	1.122
12	rs146999091	128899748	A	0.03608	0.045	G	0.5638	0.7944
14	rs45438398	94936110	A	0.03608	0.045	G	0.5638	0.7944
19	rs16973890	38055553	G	0.03608	0.045	A	0.5638	0.7944
14	rs12887189	59988324	A	0.1366	0.1492	G	0.564	0.9024
17	rs6587175	21503147	A	0.1366	0.1492	C	0.564	0.9024
1	rs6687758	222164948	G	0.3067	0.29	A	0.5641	1.083
2	rs1364054	8178735	G	0.3067	0.29	A	0.5641	1.083
6	rs1736951	29785776	A	0.1572	0.1442	G	0.5641	1.107
10	rs56391924	32745248	C	0.1572	0.1442	A	0.5641	1.107
10	rs12268559	32856746	C	0.1572	0.1442	A	0.5641	1.107
17	rs17609240	38110689	A	0.2809	0.2967	C	0.5643	0.9262
13	rs3764056	41517985	G	0.1959	0.2108	A	0.5646	0.9118
9	rs3750436	35753170	A	0.1959	0.21	C	0.5649	0.9164
6	rs12195173	47623292	A	0.2835	0.3008	G	0.5651	0.9196
7	rs4407763	67018692	A	0.2835	0.3008	G	0.5651	0.9196
15	rs1866928	90168693	A	0.2835	0.3008	T	0.5651	0.9196
15	rs3743372	90170237	A	0.2835	0.3008	G	0.5651	0.9196
1	rs1933631	230914729	C	0.1572	0.145	A	0.5652	1.1
2	rs1054765	8811354	C	0.1572	0.145	A	0.5652	1.1
4	rs11099592	84230619	A	0.1572	0.145	G	0.5652	1.1
6	rs744436	83588229	A	0.1572	0.145	G	0.5652	1.1
12	rs2280067	121768248	G	0.1572	0.145	A	0.5652	1.1
5	rs2303078	162901156	A	0.07474	0.06667	G	0.5654	1.131
6	rs10485165	89112817	A	0.3067	0.2917	G	0.5654	1.074
4	rs79868722	57176832	G	0.2191	0.205	A	0.5655	1.088
6	rs2074503	30530496	A	0.3093	0.2933	G	0.5655	1.079
10	rs3747869	73520632	A	0.2191	0.205	C	0.5655	1.088
6	rs17879981	32552121	T	0.0285	0.02254	A	0.5656	1.272
1	rs10733022	228660501	A	0.2861	0.3025	C	0.566	0.924

4	rs2389202	117254289	A	0.2861	0.3025	T	0.566	0.924
2	rs6716066	55407754	G	0.02835	0.0225	A	0.5661	1.268
2	rs117627894	179188979	A	0.02835	0.0225	G	0.5661	1.268
2	rs116869006	179188986	A	0.02835	0.0225	G	0.5661	1.268
3	rs144650170	151535235	G	0.02835	0.0225	A	0.5661	1.268
6	rs35259282	87994504	A	0.02835	0.0225	G	0.5661	1.268
7	rs3816887	128441545	G	0.02835	0.0225	C	0.5661	1.268
7	rs61730174	128454866	A	0.02835	0.0225	G	0.5661	1.268
8	rs10088218	129543949	A	0.02835	0.0225	G	0.5661	1.268
11	rs77080347	111746056	G	0.02835	0.0225	A	0.5661	1.268
12	rs113469395	53687113	A	0.02835	0.0225	G	0.5661	1.268
15	rs35128996	101606145	A	0.02835	0.0225	G	0.5661	1.268
16	rs35438466	15715636	G	0.02835	0.0225	C	0.5661	1.268
17	rs115146121	47583681	A	0.02835	0.0225	G	0.5661	1.268
2	rs2015161	225867091	C	0.06186	0.07167	A	0.5662	0.8541
2	rs11681826	28814033	G	0.3093	0.2942	A	0.5663	1.074
19	rs1052692	1619350	A	0.3093	0.2942	G	0.5663	1.074
5	rs1366363	29949620	A	0.1985	0.2133	G	0.5664	0.913
8	rs264	19813180	A	0.2191	0.2058	G	0.5666	1.082
9	rs4144636	119762707	A	0.2191	0.2058	C	0.5666	1.082
17	rs3744307	66267650	C	0.2191	0.2058	A	0.5666	1.082
8	rs17232789	122622500	A	0.09536	0.1067	G	0.5667	0.8828
1	rs117678693	203192658	A	0.04897	0.04167	C	0.5668	1.184
4	rs78421449	36212049	A	0.06186	0.0725	C	0.5668	0.8435
8	rs7827446	52233408	A	0.1392	0.1517	G	0.5668	0.9043
8	rs142301665	142185385	G	0.04897	0.04167	C	0.5668	1.184
9	rs7045881	26935996	A	0.1392	0.1517	T	0.5668	0.9043
15	rs16958445	74176557	A	0.1392	0.1517	G	0.5668	0.9043
16	rs73491269	825255	A	0.04897	0.04167	G	0.5668	1.184
1	rs17185492	118644524	A	0.09536	0.1075	T	0.5669	0.8752
13	rs3851167	50409275	A	0.2887	0.305	G	0.567	0.9247
15	rs17803620	89804043	A	0.2887	0.305	G	0.567	0.9247
1	rs10494112	110352477	G	0.002591	0.001667	A	0.5671	1.556
2	rs6433782	180224834	G	0.3119	0.2967	A	0.5671	1.074
2	rs2251692	227589780	G	0.3119	0.2967	A	0.5671	1.074
3	rs139652708	50000117	A	0.002591	0.001667	G	0.5671	1.556
6	rs2734990	29812505	A	0.3119	0.2967	T	0.5671	1.074
6	rs41548215	31322905	G	0.002591	0.001667	A	0.5671	1.556
7	rs1012803	26688808	A	0.3119	0.2967	G	0.5671	1.074
14	rs148241854	50778861	A	0.002591	0.001667	G	0.5671	1.556
17	rs2304968	33805150	A	0.3119	0.2967	G	0.5671	1.074
19	rs61732447	16060523	A	0.002591	0.001667	G	0.5671	1.556
1	rs606149	193921548	G	0.3144	0.2983	A	0.5673	1.079

17	rs61644407	505129	C	0.2216	0.2078	A	0.5674	1.085
19	rs149735408	56538513	G	0.002591	0.001669	A	0.5676	1.553
3	rs75907560	41942255	G	0.1598	0.1475	A	0.5678	1.099
1	rs1045105	26515956	A	0.1134	0.1025	C	0.5679	1.12
1	rs75945379	223566876	A	0.1134	0.1025	G	0.5679	1.12
2	rs17235409	219259732	A	0.1134	0.1025	G	0.5679	1.12
19	rs9973235	4847713	A	0.1134	0.1025	G	0.5679	1.12
20	rs6137081	20599985	A	0.1134	0.1025	G	0.5679	1.12
3	rs7638504	105499373	G	0.2216	0.2083	A	0.5682	1.082
5	rs3776070	149361221	A	0.1134	0.1027	T	0.5682	1.118
6	rs2743941	29755451	A	0.201	0.2167	G	0.5682	0.9097
12	rs7960290	33902150	A	0.201	0.2167	C	0.5682	0.9097
1	rs35351292	31208042	A	0.07732	0.06833	G	0.5687	1.143
1	rs6429644	44487280	G	0.07732	0.06833	A	0.5687	1.143
6	rs35880970	41753136	A	0.002604	0.001684	G	0.5687	1.548
21	rs55870334	39579012	G	0.07732	0.06833	A	0.5687	1.143
1	rs201706077	1848287	A	0.002577	0.001667	G	0.5688	1.548
1	rs201836187	2252973	G	0.002577	0.001667	A	0.5688	1.548
1	rs121909136	16382003	A	0.002577	0.001667	G	0.5688	1.548
1	rs150336992	18554424	A	0.002577	0.001667	C	0.5688	1.548
1	rs188986875	21551809	A	0.002577	0.001667	G	0.5688	1.548
1	rs146305109	22172735	A	0.002577	0.001667	G	0.5688	1.548
1	rs138359307	26524246	G	0.002577	0.001667	A	0.5688	1.548
1	rs187631645	27105700	A	0.002577	0.001667	G	0.5688	1.548
1	rs200249506	27679805	A	0.002577	0.001667	G	0.5688	1.548
1	rs182542991	29344869	A	0.002577	0.001667	G	0.5688	1.548
1	rs149882646	36055616	A	0.002577	0.001667	G	0.5688	1.548
1	rs148608573	36638206	A	0.002577	0.001667	G	0.5688	1.548
1	rs59084124	39816512	A	0.002577	0.001667	T	0.5688	1.548
1	rs585655	43597014	A	0.002577	0.001667	G	0.5688	1.548
1	rs182040367	75065377	A	0.002577	0.001667	G	0.5688	1.548
1	rs184606223	103540265	A	0.002577	0.001667	G	0.5688	1.548
1	rs183034705	114445447	C	0.002577	0.001667	A	0.5688	1.548
1	rs139692289	117559721	G	0.002577	0.001667	A	0.5688	1.548
1	rs201890460	146672890	A	0.002577	0.001667	G	0.5688	1.548
1	rs190000383	152595565	G	0.002577	0.001667	C	0.5688	1.548
1	rs183290832	152732299	G	0.002577	0.001667	A	0.5688	1.548
1	rs201808585	153940996	C	0.002577	0.001667	A	0.5688	1.548
1	rs144919505	154514541	A	0.002577	0.001667	G	0.5688	1.548
1	rs191738898	154942861	A	0.002577	0.001667	G	0.5688	1.548
1	rs140447805	155746214	A	0.002577	0.001667	G	0.5688	1.548
1	rs185517726	156287027	A	0.002577	0.001667	G	0.5688	1.548
1	rs185598690	156532436	G	0.002577	0.001667	A	0.5688	1.548

1	rs143673331	156641353	G	0.002577	0.001667	A	0.5688	1.548
1	rs201192875	156838353	A	0.002577	0.001667	G	0.5688	1.548
1	rs188827319	159023509	C	0.002577	0.001667	A	0.5688	1.548
1	rs35759983	160769690	A	0.002577	0.001667	G	0.5688	1.548
1	rs3737787	161009523	A	0.2242	0.21	G	0.5688	1.087
1	rs185368151	161335344	G	0.002577	0.001667	A	0.5688	1.548
1	rs144937515	169528493	A	0.002577	0.001667	C	0.5688	1.548
1	rs148309294	181058084	A	0.002577	0.001667	G	0.5688	1.548
1	rs78436705	183854067	A	0.002577	0.001667	G	0.5688	1.548
1	rs182806418	203154512	A	0.002577	0.001667	G	0.5688	1.548
1	rs184474693	205767816	A	0.002577	0.001667	C	0.5688	1.548
1	rs116800126	207930891	A	0.002577	0.001667	G	0.5688	1.548
1	rs79500044	208061148	A	0.002577	0.001667	C	0.5688	1.548
1	rs201889146	208391023	A	0.002577	0.001667	G	0.5688	1.548
1	rs2229466	209797253	G	0.2242	0.21	A	0.5688	1.087
1	exm154269	227219067	G	0.002577	0.001667	A	0.5688	1.548
1	rs201777691	230846449	T	0.002577	0.001667	A	0.5688	1.548
1	rs35565926	236143189	A	0.002577	0.001667	G	0.5688	1.548
1	rs55662927	241755414	A	0.002577	0.001667	G	0.5688	1.548
1	rs118064970	243507569	G	0.002577	0.001667	A	0.5688	1.548
1	rs12070953	247614386	G	0.002577	0.001667	A	0.5688	1.548
1	rs12065526	247614407	A	0.002577	0.001667	G	0.5688	1.548
1	rs202099060	248005189	C	0.002577	0.001667	G	0.5688	1.548
2	rs200237910	675618	A	0.002577	0.001667	T	0.5688	1.548
2	rs142736664	20974570	A	0.002577	0.001667	G	0.5688	1.548
2	rs78875649	21238034	G	0.002577	0.001667	A	0.5688	1.548
2	rs78882128	27478277	G	0.002577	0.001667	A	0.5688	1.548
2	rs146285804	27741783	A	0.002577	0.001667	C	0.5688	1.548
2	rs145379829	28748165	G	0.002577	0.001667	A	0.5688	1.548
2	rs145252041	28865884	A	0.002577	0.001667	G	0.5688	1.548
2	rs201933656	29287898	A	0.002577	0.001667	G	0.5688	1.548
2	rs148904866	31588939	A	0.002577	0.001667	G	0.5688	1.548
2	rs201792967	31596756	A	0.002577	0.001667	G	0.5688	1.548
2	rs145381023	33172816	G	0.002577	0.001667	A	0.5688	1.548
2	rs80243841	37111123	A	0.002577	0.001667	G	0.5688	1.548
2	rs201933119	39499683	A	0.002577	0.001667	G	0.5688	1.548
2	rs151199680	42522365	A	0.002577	0.001667	G	0.5688	1.548
2	rs199706677	44145469	A	0.002577	0.001667	G	0.5688	1.548
2	rs140165185	44556203	G	0.002577	0.001667	C	0.5688	1.548
2	rs183615774	62067223	A	0.002577	0.001667	G	0.5688	1.548
2	rs61734468	63401820	G	0.002577	0.001667	A	0.5688	1.548
2	rs17009792	74489323	A	0.002577	0.001667	G	0.5688	1.548
2	rs116858801	96906359	G	0.002577	0.001667	C	0.5688	1.548

2	rs180808618	99227330	A	0.002577	0.001667	G	0.5688	1.548
2	rs11541353	101594191	A	0.002577	0.001667	G	0.5688	1.548
2	rs1062062	101627925	A	0.002577	0.001667	G	0.5688	1.548
2	rs150869326	101881399	A	0.002577	0.001667	G	0.5688	1.548
2	rs11465716	103061776	A	0.002577	0.001667	G	0.5688	1.548
2	rs200042988	109086737	G	0.002577	0.001667	A	0.5688	1.548
2	rs112971525	109099531	G	0.002577	0.001667	C	0.5688	1.548
2	rs141907218	110920684	A	0.002577	0.001667	G	0.5688	1.548
2	rs200590124	119752091	A	0.002577	0.001667	G	0.5688	1.548
2	rs4150522	128016886	G	0.002577	0.001667	A	0.5688	1.548
2	rs115348342	160663522	G	0.002577	0.001667	A	0.5688	1.548
2	rs56075631	162091931	G	0.002577	0.001667	C	0.5688	1.548
2	rs201428751	174086160	G	0.002577	0.001667	A	0.5688	1.548
2	rs201653851	179584152	A	0.002577	0.001667	G	0.5688	1.548
2	rs72647876	179642589	G	0.002577	0.001667	C	0.5688	1.548
2	rs35683768	179666982	A	0.002577	0.001667	C	0.5688	1.548
2	rs150543864	189861158	A	0.002577	0.001667	G	0.5688	1.548
2	rs55859858	190732599	C	0.002577	0.001667	G	0.5688	1.548
2	rs7562391	201736166	C	0.002577	0.001667	A	0.5688	1.548
2	rs200815994	219209268	A	0.002577	0.001667	G	0.5688	1.548
2	rs115609218	219528775	A	0.002577	0.001667	G	0.5688	1.548
2	rs201615111	227875130	A	0.002577	0.001667	G	0.5688	1.548
3	rs200823423	4355201	C	0.002577	0.001667	G	0.5688	1.548
3	rs201921926	9739483	A	0.002577	0.001667	G	0.5688	1.548
3	rs139384572	10219689	A	0.002577	0.001667	G	0.5688	1.548
3	rs2305296	12610453	G	0.002577	0.001667	A	0.5688	1.548
3	rs180950245	16364898	C	0.002577	0.001667	G	0.5688	1.548
3	rs77348972	39174721	G	0.002577	0.001667	C	0.5688	1.548
3	rs201134509	44490011	G	0.002577	0.001667	A	0.5688	1.548
3	rs201137952	44816783	A	0.002577	0.001667	G	0.5688	1.548
3	rs201126407	44852434	A	0.002577	0.001667	T	0.5688	1.548
3	rs201058230	50380438	A	0.002577	0.001667	G	0.5688	1.548
3	rs75800791	51863322	A	0.002577	0.001667	G	0.5688	1.548
3	rs35338461	52233295	A	0.002577	0.001667	G	0.5688	1.548
3	rs5743845	52255744	A	0.002577	0.001667	G	0.5688	1.548
3	rs188699424	52380590	C	0.002577	0.001667	A	0.5688	1.548
3	rs61736838	52522591	A	0.002577	0.001667	G	0.5688	1.548
3	rs116426983	52740889	G	0.002577	0.001667	A	0.5688	1.548
3	rs78172374	57448435	G	0.002577	0.001667	A	0.5688	1.548
3	rs56384862	58395863	G	0.002577	0.001667	A	0.5688	1.548
3	rs34579268	58410554	A	0.002577	0.001667	G	0.5688	1.548
3	rs3736422	69087821	G	0.002577	0.001667	A	0.5688	1.548
3	rs201166758	101451483	G	0.002577	0.001667	A	0.5688	1.548

3	rs6779094	108081277	A	0.002577	0.001667	C	0.5688	1.548
3	rs140727933	111261127	G	0.002577	0.001667	A	0.5688	1.548
3	rs200569169	113595063	A	0.002577	0.001667	G	0.5688	1.548
3	rs150353258	119459516	A	0.002577	0.001667	G	0.5688	1.548
3	rs17249244	120976051	G	0.002577	0.001667	C	0.5688	1.548
3	rs138311983	122123138	A	0.002577	0.001667	G	0.5688	1.548
3	rs144149458	130742901	G	0.002577	0.001667	A	0.5688	1.548
3	rs148550603	132068845	A	0.002577	0.001667	G	0.5688	1.548
3	rs140029062	134078284	A	0.002577	0.001667	G	0.5688	1.548
3	rs138115393	141272706	A	0.002577	0.001667	G	0.5688	1.548
3	rs200226362	148905929	A	0.002577	0.001667	G	0.5688	1.548
3	rs143116281	152058351	A	0.002577	0.001667	G	0.5688	1.548
3	rs200789237	155560337	A	0.002577	0.001667	G	0.5688	1.548
3	rs1170672	178968660	G	0.002577	0.001667	A	0.5688	1.548
3	rs188036977	195925709	A	0.002577	0.001667	G	0.5688	1.548
3	rs61743568	197432036	A	0.002577	0.001667	G	0.5688	1.548
4	rs202121184	3768805	A	0.002577	0.001667	G	0.5688	1.548
4	rs181146375	4322607	A	0.002577	0.001667	G	0.5688	1.548
4	rs41269549	5733317	A	0.002577	0.001667	G	0.5688	1.548
4	rs149857509	13603003	A	0.002577	0.001667	G	0.5688	1.548
4	rs186265781	36345346	A	0.002577	0.001667	G	0.5688	1.548
4	rs34137542	38879818	A	0.002577	0.001667	G	0.5688	1.548
4	rs142570098	40104609	A	0.002577	0.001667	G	0.5688	1.548
4	rs141526764	44688041	A	0.002577	0.001667	G	0.5688	1.548
4	rs183408354	52862250	G	0.002577	0.001667	A	0.5688	1.548
4	rs141789930	52938139	A	0.002577	0.001667	G	0.5688	1.548
4	rs147697562	56819340	G	0.002577	0.001667	A	0.5688	1.548
4	rs201450284	74853703	C	0.002577	0.001667	G	0.5688	1.548
4	rs184735609	76442163	G	0.002577	0.001667	A	0.5688	1.548
4	rs180770321	83838420	C	0.002577	0.001667	A	0.5688	1.548
4	rs187506570	85762338	A	0.002577	0.001667	G	0.5688	1.548
4	rs61730639	87692523	A	0.002577	0.001667	C	0.5688	1.548
4	rs149531474	87812672	G	0.002577	0.001667	A	0.5688	1.548
4	rs140898788	88091339	A	0.002577	0.001667	G	0.5688	1.548
4	rs140725424	101109088	A	0.002577	0.001667	G	0.5688	1.548
4	rs143985505	104012371	A	0.002577	0.001667	G	0.5688	1.548
4	rs202225266	123664203	A	0.002577	0.001667	G	0.5688	1.548
4	rs199885169	134073527	A	0.002577	0.001667	C	0.5688	1.548
4	rs147575266	162376178	G	0.002577	0.001667	A	0.5688	1.548
4	rs201598032	187084634	A	0.002577	0.001667	G	0.5688	1.548
5	rs61750331	169589	G	0.002577	0.001667	A	0.5688	1.548
5	rs200263887	917213	G	0.002577	0.001667	A	0.5688	1.548
5	rs9313105	5239880	A	0.002577	0.001667	G	0.5688	1.548

5	rs201921000	35035360	A	0.002577	0.001667	G	0.5688	1.548
5	rs2291703	36976160	G	0.002577	0.001667	A	0.5688	1.548
5	rs201688514	50057670	G	0.002577	0.001667	A	0.5688	1.548
5	rs6859219	55438580	A	0.002577	0.001667	C	0.5688	1.548
5	rs150480852	79026866	A	0.002577	0.001667	G	0.5688	1.548
5	rs2229396	79372721	A	0.002577	0.001667	G	0.5688	1.548
5	rs200670494	79616117	G	0.002577	0.001667	A	0.5688	1.548
5	rs41311343	90040890	G	0.002577	0.001667	A	0.5688	1.548
5	rs201420881	90281227	G	0.002577	0.001667	A	0.5688	1.548
5	rs7719346	111530284	A	0.002577	0.001667	G	0.5688	1.548
5	rs185385178	132409020	G	0.002577	0.001667	A	0.5688	1.548
5	rs2231471	137548976	A	0.002577	0.001667	G	0.5688	1.548
5	rs199748187	149264115	A	0.002577	0.001667	G	0.5688	1.548
5	rs150700679	150924968	G	0.002577	0.001667	A	0.5688	1.548
5	rs192241783	154188115	G	0.002577	0.001667	A	0.5688	1.548
5	rs144891956	167924324	A	0.002577	0.001667	G	0.5688	1.548
5	rs149199617	178413676	A	0.002577	0.001667	G	0.5688	1.548
5	rs200565045	180662357	A	0.002577	0.001667	G	0.5688	1.548
6	rs78806162	16295278	A	0.002577	0.001667	T	0.5688	1.548
6	rs1772256	24437445	G	0.002577	0.001667	A	0.5688	1.548
6	rs117409123	26017836	A	0.002577	0.001667	G	0.5688	1.548
6	rs199702760	26234554	A	0.002577	0.001667	G	0.5688	1.548
6	rs145278803	27834857	A	0.002577	0.001667	C	0.5688	1.548
6	rs147890226	29429617	C	0.002577	0.001667	G	0.5688	1.548
6	rs16897204	30154049	A	0.002577	0.001667	G	0.5688	1.548
6	rs10456378	30206186	A	0.002577	0.001667	G	0.5688	1.548
6	rs928824	30224889	A	0.002577	0.001667	G	0.5688	1.548
6	rs928823	30257846	T	0.002577	0.001667	A	0.5688	1.548
6	rs1264608	30278112	A	0.002577	0.001667	G	0.5688	1.548
6	rs1264583	30293483	A	0.002577	0.001667	G	0.5688	1.548
6	rs9262138	30627867	G	0.002577	0.001667	A	0.5688	1.548
6	rs145449397	32714134	A	0.002577	0.001667	G	0.5688	1.548
6	rs201507218	33382581	C	0.002577	0.001667	G	0.5688	1.548
6	rs35080860	35443835	A	0.002577	0.001667	G	0.5688	1.548
6	rs142348124	35928624	A	0.002577	0.001667	G	0.5688	1.548
6	rs145146911	37614135	A	0.002577	0.001667	G	0.5688	1.548
6	rs2143678	41623033	A	0.002577	0.001667	C	0.5688	1.548
6	rs111948785	41774340	A	0.002577	0.001667	C	0.5688	1.548
6	rs142676892	41889553	A	0.002577	0.001667	G	0.5688	1.548
6	rs143003258	42833003	A	0.002577	0.001667	G	0.5688	1.548
6	rs147775109	43016266	A	0.002577	0.001667	G	0.5688	1.548
6	rs9357410	43322464	C	0.002577	0.001667	G	0.5688	1.548
6	rs190204308	43480045	A	0.002577	0.001667	C	0.5688	1.548

6	rs9472463	45290634	A	0.002577	0.001667	G	0.5688	1.548
6	rs17240063	52137158	C	0.002577	0.001667	A	0.5688	1.548
6	rs145027088	55142358	G	0.002577	0.001667	A	0.5688	1.548
6	rs192304860	109771583	A	0.002577	0.001667	G	0.5688	1.548
6	rs3218600	111695899	A	0.002577	0.001667	G	0.5688	1.548
6	rs9489444	118887215	A	0.002577	0.001667	T	0.5688	1.548
6	rs61756668	128404958	A	0.002577	0.001667	G	0.5688	1.548
6	rs77081633	136589425	A	0.002577	0.001667	C	0.5688	1.548
6	rs116282282	144869823	G	0.002577	0.001667	A	0.5688	1.548
6	rs182644629	146214448	C	0.002577	0.001667	A	0.5688	1.548
6	rs201123268	150210544	A	0.002577	0.001667	G	0.5688	1.548
6	rs143269665	151334947	G	0.002577	0.001667	A	0.5688	1.548
6	rs200825287	152652867	A	0.002577	0.001667	G	0.5688	1.548
6	rs142294952	154732169	A	0.002577	0.001667	G	0.5688	1.548
6	rs138618542	158924587	A	0.002577	0.001667	G	0.5688	1.548
6	rs61750777	159420548	A	0.002577	0.001667	G	0.5688	1.548
6	rs111656694	170036466	A	0.002577	0.001667	C	0.5688	1.548
6	rs138374105	170089044	A	0.002577	0.001667	T	0.5688	1.548
7	rs41493047	2962848	A	0.002577	0.001667	G	0.5688	1.548
7	rs188234794	4855862	G	0.002577	0.001667	A	0.5688	1.548
7	rs140264038	5780818	A	0.002577	0.001667	G	0.5688	1.548
7	rs191930676	6189487	A	0.002577	0.001667	C	0.5688	1.548
7	rs79671818	30693204	A	0.002577	0.001667	G	0.5688	1.548
7	rs201901788	31617627	A	0.002577	0.001667	G	0.5688	1.548
7	rs186094669	91504058	G	0.002577	0.001667	A	0.5688	1.548
7	rs115375965	98246958	A	0.002577	0.001667	G	0.5688	1.548
7	rs52833865	100176362	A	0.002577	0.001667	G	0.5688	1.548
7	rs200810028	100355879	A	0.002577	0.001667	G	0.5688	1.548
7	rs35776303	107414570	G	0.002577	0.001667	A	0.5688	1.548
7	rs886774	107495434	G	0.2242	0.21	A	0.5688	1.087
7	rs17155094	107800936	A	0.002577	0.001667	G	0.5688	1.548
7	rs139800212	137148323	A	0.002577	0.001667	G	0.5688	1.548
7	exm2245052	138522671	A	0.002577	0.001667	G	0.5688	1.548
7	rs8192864	139717608	A	0.002577	0.001667	G	0.5688	1.548
7	rs186801504	149557871	A	0.002577	0.001667	G	0.5688	1.548
7	rs138029142	150325279	A	0.002577	0.001667	G	0.5688	1.548
7	rs1049742	150554553	A	0.002577	0.001667	G	0.5688	1.548
7	rs141106704	151842355	G	0.002577	0.001667	A	0.5688	1.548
7	rs183641482	155295780	C	0.002577	0.001667	A	0.5688	1.548
8	rs199717191	6690345	A	0.002577	0.001667	G	0.5688	1.548
8	rs199806000	17739687	A	0.002577	0.001667	G	0.5688	1.548
8	rs200942511	22472960	G	0.002577	0.001667	A	0.5688	1.548
8	rs149267963	22472985	A	0.002577	0.001667	G	0.5688	1.548

8	rs117655902	23294491	G	0.002577	0.001667	A	0.5688	1.548
8	rs150677263	67066511	A	0.002577	0.001667	G	0.5688	1.548
8	exm2258945	70617424	A	0.002577	0.001667	G	0.5688	1.548
8	rs200618319	70617424	A	0.002577	0.001667	G	0.5688	1.548
8	rs139337746	87591364	G	0.002577	0.001667	A	0.5688	1.548
8	rs200661878	95523796	G	0.002577	0.001667	A	0.5688	1.548
8	rs201210090	98831434	A	0.002577	0.001667	C	0.5688	1.548
8	rs121908603	106814417	C	0.002577	0.001667	A	0.5688	1.548
8	rs202241413	110463357	A	0.002577	0.001667	G	0.5688	1.548
8	rs186951934	110510904	C	0.002577	0.001667	A	0.5688	1.548
8	rs145027071	113299353	G	0.002577	0.001667	A	0.5688	1.548
8	rs191199415	131127924	C	0.002577	0.001667	A	0.5688	1.548
8	rs201424010	145138351	A	0.002577	0.001667	G	0.5688	1.548
8	rs137926644	145626438	A	0.002577	0.001667	G	0.5688	1.548
9	rs73370597	215296	G	0.002577	0.001667	A	0.5688	1.548
9	rs114784029	711962	A	0.002577	0.001667	G	0.5688	1.548
9	rs150766325	2812337	G	0.002577	0.001667	A	0.5688	1.548
9	rs184910238	12704582	A	0.002577	0.001667	C	0.5688	1.548
9	rs192785156	13175774	G	0.002577	0.001667	A	0.5688	1.548
9	rs4741289	13190163	A	0.002577	0.001667	G	0.5688	1.548
9	rs144658832	18721642	G	0.002577	0.001667	A	0.5688	1.548
9	rs186749928	35957877	A	0.002577	0.001667	C	0.5688	1.548
9	rs78749972	36105196	A	0.002577	0.001667	G	0.5688	1.548
9	rs141986091	36674904	C	0.002577	0.001667	G	0.5688	1.548
9	rs141858334	71661314	A	0.002577	0.001667	G	0.5688	1.548
9	rs148028742	88650531	G	0.002577	0.001667	A	0.5688	1.548
9	rs200389880	95030556	A	0.002577	0.001667	G	0.5688	1.548
9	rs34607425	95179071	A	0.002577	0.001667	G	0.5688	1.548
9	rs201433639	95432254	G	0.002577	0.001667	A	0.5688	1.548
9	rs182045135	98211463	G	0.002577	0.001667	A	0.5688	1.548
9	rs140955159	104335535	A	0.002577	0.001667	G	0.5688	1.548
9	rs79044641	107331618	A	0.002577	0.001667	G	0.5688	1.548
9	rs5030718	120475826	A	0.002577	0.001667	G	0.5688	1.548
9	rs139868007	125391391	A	0.002577	0.001667	G	0.5688	1.548
9	rs200222599	130492981	A	0.002577	0.001667	G	0.5688	1.548
9	rs186499200	131584804	G	0.002577	0.001667	A	0.5688	1.548
9	rs201749338	133573588	A	0.002577	0.001667	C	0.5688	1.548
9	rs74872755	140140111	A	0.002577	0.001667	G	0.5688	1.548
10	rs202132062	3206008	A	0.002577	0.001667	C	0.5688	1.548
10	rs143074510	5415798	A	0.002577	0.001667	G	0.5688	1.548
10	rs191866446	5791298	C	0.002577	0.001667	G	0.5688	1.548
10	rs75614526	13237181	A	0.002577	0.001667	G	0.5688	1.548
10	rs10466081	22498450	C	0.002577	0.001667	A	0.5688	1.548

10	rs147531808	25279456	G	0.002577	0.001667	A	0.5688	1.548
10	rs148063421	38241920	A	0.002577	0.001667	G	0.5688	1.548
10	rs139656912	43292337	A	0.002577	0.001667	G	0.5688	1.548
10	rs144825016	52575814	C	0.002577	0.001667	A	0.5688	1.548
10	rs182040233	59956304	A	0.002577	0.001667	C	0.5688	1.548
10	rs147472176	72061211	A	0.002577	0.001667	G	0.5688	1.548
10	rs201891685	76788574	G	0.002577	0.001667	C	0.5688	1.548
10	rs190565839	79614079	A	0.002577	0.001667	G	0.5688	1.548
10	rs142449029	95123803	A	0.002577	0.001667	G	0.5688	1.548
10	rs200557763	99661261	A	0.002577	0.001667	G	0.5688	1.548
10	rs116266095	102088968	A	0.002577	0.001667	G	0.5688	1.548
10	rs201771386	104445670	A	0.002577	0.001667	G	0.5688	1.548
10	rs192144932	105215119	A	0.002577	0.001667	C	0.5688	1.548
10	rs201065560	105361800	A	0.002577	0.001667	G	0.5688	1.548
10	rs75922594	105361941	A	0.002577	0.001667	G	0.5688	1.548
10	rs146267259	105836074	A	0.002577	0.001667	G	0.5688	1.548
10	rs143884799	118215261	A	0.002577	0.001667	C	0.5688	1.548
10	rs139830072	118969380	G	0.002577	0.001667	A	0.5688	1.548
11	rs147162160	373828	A	0.002577	0.001667	G	0.5688	1.548
11	rs199886265	865561	A	0.002577	0.001667	G	0.5688	1.548
11	rs118104064	2991124	A	0.002577	0.001667	G	0.5688	1.548
11	rs184336225	5020511	G	0.002577	0.001667	A	0.5688	1.548
11	rs186096167	5461951	A	0.002577	0.001667	G	0.5688	1.548
11	rs185967589	6191528	G	0.002577	0.001667	A	0.5688	1.548
11	rs146000679	6622241	A	0.002577	0.001667	G	0.5688	1.548
11	rs149678438	47267375	A	0.002577	0.001667	G	0.5688	1.548
11	rs505370	51391587	A	0.002577	0.001667	G	0.5688	1.548
11	rs191576850	56043172	A	0.002577	0.001667	G	0.5688	1.548
11	rs71484443	57103350	G	0.002577	0.001667	A	0.5688	1.548
11	rs116129637	61254514	A	0.002577	0.001667	G	0.5688	1.548
11	rs115526077	62284845	A	0.002577	0.001667	C	0.5688	1.548
11	rs137909090	62398640	G	0.002577	0.001667	A	0.5688	1.548
11	rs200671505	64815802	A	0.002577	0.001667	T	0.5688	1.548
11	rs138744839	66281952	A	0.002577	0.001667	G	0.5688	1.548
11	rs190544690	67790188	A	0.002577	0.001667	G	0.5688	1.548
11	rs75148876	69950126	A	0.002577	0.001667	G	0.5688	1.548
11	rs138969547	71191891	A	0.002577	0.001667	G	0.5688	1.548
11	rs143730967	71718294	A	0.002577	0.001667	G	0.5688	1.548
11	rs10830430	89902154	G	0.002577	0.001667	A	0.5688	1.548
11	rs192763656	93433273	G	0.002577	0.001667	A	0.5688	1.548
11	rs185568824	94040334	A	0.002577	0.001667	C	0.5688	1.548
11	rs150946177	107300108	A	0.002577	0.001667	G	0.5688	1.548
11	rs79045562	112064369	C	0.002577	0.001667	A	0.5688	1.548

11	rs180917395	116693862	A	0.002577	0.001667	G	0.5688	1.548
11	rs201598684	119230277	A	0.002577	0.001667	G	0.5688	1.548
11	rs147722450	119243748	A	0.002577	0.001667	G	0.5688	1.548
11	rs201924719	129812490	A	0.002577	0.001667	T	0.5688	1.548
11	rs139630658	130131026	A	0.002577	0.001667	G	0.5688	1.548
11	rs191108994	134241337	G	0.002577	0.001667	A	0.5688	1.548
12	rs200802666	2937504	A	0.002577	0.001667	G	0.5688	1.548
12	rs141838188	6571287	A	0.002577	0.001667	G	0.5688	1.548
12	rs139364109	7361765	G	0.002577	0.001667	A	0.5688	1.548
12	rs200058002	9318699	A	0.002577	0.001667	G	0.5688	1.548
12	rs192853755	13233561	C	0.002577	0.001667	G	0.5688	1.548
12	rs79044647	21467482	A	0.002577	0.001667	G	0.5688	1.548
12	rs61733199	21695439	G	0.002577	0.001667	A	0.5688	1.548
12	rs186772900	32135174	G	0.002577	0.001667	A	0.5688	1.548
12	rs148686457	32886737	G	0.002577	0.001667	A	0.5688	1.548
12	rs150158667	41949530	A	0.002577	0.001667	G	0.5688	1.548
12	rs147285081	54757475	A	0.002577	0.001667	G	0.5688	1.548
12	rs117778870	56396395	A	0.002577	0.001667	G	0.5688	1.548
12	rs187173108	57873135	A	0.002577	0.001667	G	0.5688	1.548
12	rs190598622	57883087	A	0.002577	0.001667	G	0.5688	1.548
12	rs139369627	58126761	A	0.002577	0.001667	G	0.5688	1.548
12	rs75770066	66704225	G	0.002577	0.001667	A	0.5688	1.548
12	rs201102571	66724940	A	0.002577	0.001667	G	0.5688	1.548
12	rs140348782	96288809	G	0.002577	0.001667	A	0.5688	1.548
12	rs201623880	101520764	A	0.002577	0.001667	G	0.5688	1.548
12	rs146403821	108169494	A	0.002577	0.001667	C	0.5688	1.548
12	rs149061964	120650365	A	0.002577	0.001667	G	0.5688	1.548
12	rs145763887	121660756	C	0.002577	0.001667	G	0.5688	1.548
12	rs61734124	122248083	A	0.002577	0.001667	G	0.5688	1.548
12	rs11057405	122781897	A	0.002577	0.001667	G	0.5688	1.548
12	rs199553878	123645738	A	0.002577	0.001667	G	0.5688	1.548
12	rs142255135	124403451	C	0.002577	0.001667	A	0.5688	1.548
12	rs147262074	130907078	A	0.002577	0.001667	G	0.5688	1.548
12	rs199942065	133354366	A	0.002577	0.001667	G	0.5688	1.548
13	rs201899988	23908732	C	0.002577	0.001667	G	0.5688	1.548
13	rs200517685	23915372	G	0.002577	0.001667	C	0.5688	1.548
13	rs10676	52343391	A	0.002577	0.001667	G	0.5688	1.548
13	rs139289704	52544745	A	0.002577	0.001667	G	0.5688	1.548
13	rs140075542	52972050	A	0.002577	0.001667	G	0.5688	1.548
13	rs201686630	74387355	G	0.002577	0.001667	A	0.5688	1.548
13	rs75343650	79932435	A	0.002577	0.001667	C	0.5688	1.548
13	rs17080147	86369403	G	0.002577	0.001667	A	0.5688	1.548
13	rs139687710	113052447	A	0.002577	0.001667	G	0.5688	1.548

14	rs146537080	20586368	A	0.002577	0.001667	G	0.5688	1.548
14	rs182796454	20763881	A	0.002577	0.001667	G	0.5688	1.548
14	rs201605296	21993215	A	0.002577	0.001667	G	0.5688	1.548
14	rs74642009	25077537	A	0.002577	0.001667	G	0.5688	1.548
14	rs151228858	57103316	A	0.002577	0.001667	G	0.5688	1.548
14	rs142179458	62203623	A	0.002577	0.001667	G	0.5688	1.548
14	rs61753549	70989326	A	0.002577	0.001667	G	0.5688	1.548
14	rs192013355	71444041	C	0.002577	0.001667	G	0.5688	1.548
14	rs140067086	71444577	A	0.002577	0.001667	G	0.5688	1.548
14	rs199797145	72138194	G	0.002577	0.001667	A	0.5688	1.548
14	rs202241964	77697969	A	0.002577	0.001667	G	0.5688	1.548
14	rs200834921	78170792	G	0.002577	0.001667	C	0.5688	1.548
14	rs139286618	81609331	A	0.002577	0.001667	G	0.5688	1.548
14	rs183000861	90755027	A	0.002577	0.001667	G	0.5688	1.548
14	rs183666503	93650339	A	0.002577	0.001667	G	0.5688	1.548
14	rs200171587	102793124	A	0.002577	0.001667	G	0.5688	1.548
14	rs199738023	102904413	A	0.002577	0.001667	G	0.5688	1.548
14	rs201533315	105359441	A	0.002577	0.001667	G	0.5688	1.548
15	rs200297626	25924967	A	0.002577	0.001667	C	0.5688	1.548
15	rs150499946	25926003	A	0.002577	0.001667	G	0.5688	1.548
15	rs117449906	41194872	A	0.002577	0.001667	G	0.5688	1.548
15	rs148814029	41797228	A	0.002577	0.001667	G	0.5688	1.548
15	rs11637235	48633153	A	0.002577	0.001667	G	0.5688	1.548
15	rs2306785	59406778	A	0.002577	0.001667	G	0.5688	1.548
15	rs8037614	64275876	A	0.002577	0.001667	G	0.5688	1.548
15	rs145494146	85341891	A	0.002577	0.001667	G	0.5688	1.548
15	rs142416384	91525048	C	0.002577	0.001667	G	0.5688	1.548
15	rs45482895	101858536	A	0.002577	0.001667	G	0.5688	1.548
15	rs1143136	102264476	A	0.002577	0.001667	C	0.5688	1.548
16	rs200846343	532645	A	0.002577	0.001667	G	0.5688	1.548
16	rs202049960	1962123	A	0.002577	0.001667	G	0.5688	1.548
16	rs193284245	3274511	A	0.002577	0.001667	G	0.5688	1.548
16	rs201533738	3658893	A	0.002577	0.001667	G	0.5688	1.548
16	rs111588143	15802687	C	0.002577	0.001667	G	0.5688	1.548
16	rs199773531	16208739	A	0.002577	0.001667	G	0.5688	1.548
16	rs200221988	20833162	A	0.002577	0.001667	T	0.5688	1.548
16	rs139118151	21132148	G	0.002577	0.001667	A	0.5688	1.548
16	rs201118038	28167623	A	0.002577	0.001667	G	0.5688	1.548
16	rs61744449	30980962	G	0.002577	0.001667	C	0.5688	1.548
16	rs17859943	55527073	A	0.002577	0.001667	G	0.5688	1.548
16	rs142731757	67203533	A	0.002577	0.001667	C	0.5688	1.548
16	rs149278976	67325566	A	0.002577	0.001667	G	0.5688	1.548
16	rs1559401	72011181	C	0.2938	0.3108	A	0.5688	0.9225

16	rs7185272	72013797	G	0.2938	0.3108	C	0.5688	0.9225
16	rs147897268	81076959	C	0.002577	0.001667	A	0.5688	1.548
16	rs149659582	84209903	A	0.002577	0.001667	G	0.5688	1.548
16	rs61753438	84212597	A	0.002577	0.001667	G	0.5688	1.548
17	rs34519093	649164	A	0.002577	0.001667	G	0.5688	1.548
17	rs139345811	1386304	A	0.002577	0.001667	G	0.5688	1.548
17	rs146939364	1679952	G	0.002577	0.001667	C	0.5688	1.548
17	rs202089427	4434030	A	0.002577	0.001667	G	0.5688	1.548
17	rs76904479	4575749	A	0.002577	0.001667	G	0.5688	1.548
17	rs199641819	4837803	A	0.002577	0.001667	G	0.5688	1.548
17	rs201982608	5126630	A	0.002577	0.001667	G	0.5688	1.548
17	rs117553236	7224736	C	0.002577	0.001667	A	0.5688	1.548
17	rs140185249	7951780	A	0.002577	0.001667	G	0.5688	1.548
17	rs143323346	8000048	A	0.002577	0.001667	G	0.5688	1.548
17	rs114802786	9737196	A	0.002577	0.001667	G	0.5688	1.548
17	rs182969012	10215956	A	0.002577	0.001667	G	0.5688	1.548
17	rs75477725	10304713	G	0.002577	0.001667	C	0.5688	1.548
17	rs118160421	11650904	A	0.002577	0.001667	G	0.5688	1.548
17	rs201355502	26692205	A	0.002577	0.001667	G	0.5688	1.548
17	rs200057429	27030691	A	0.002577	0.001667	G	0.5688	1.548
17	rs151310824	27613663	C	0.002577	0.001667	G	0.5688	1.548
17	rs190013343	29645838	A	0.002577	0.001667	G	0.5688	1.548
17	rs115279154	33269517	A	0.002577	0.001667	G	0.5688	1.548
17	rs78223890	39659637	A	0.002577	0.001667	G	0.5688	1.548
17	rs138271368	39673185	A	0.002577	0.001667	G	0.5688	1.548
17	rs192490174	40129324	G	0.002577	0.001667	A	0.5688	1.548
17	rs201815037	40175689	G	0.002577	0.001667	A	0.5688	1.548
17	rs146056067	40726131	A	0.002577	0.001667	G	0.5688	1.548
17	rs137982413	40841014	A	0.002577	0.001667	G	0.5688	1.548
17	rs199824275	43190368	A	0.002577	0.001667	G	0.5688	1.548
17	rs146126922	48165148	G	0.002577	0.001667	C	0.5688	1.548
17	rs142759175	53155537	C	0.002577	0.001667	G	0.5688	1.548
17	rs201939520	73758933	A	0.002577	0.001667	G	0.5688	1.548
17	rs140726801	74003318	A	0.002577	0.001667	G	0.5688	1.548
17	rs143694911	76490695	A	0.002577	0.001667	G	0.5688	1.548
17	rs147759020	78219960	A	0.002577	0.001667	G	0.5688	1.548
17	rs193236167	79683879	A	0.002577	0.001667	G	0.5688	1.548
17	rs142049212	79857229	A	0.002577	0.001667	G	0.5688	1.548
18	rs182642815	9256189	A	0.002577	0.001667	C	0.5688	1.548
18	rs184265318	19995775	A	0.002577	0.001667	G	0.5688	1.548
18	rs200907971	47367799	A	0.002577	0.001667	G	0.5688	1.548
18	rs145598498	47379895	A	0.002577	0.001667	G	0.5688	1.548
18	rs202206682	72228097	A	0.002577	0.001667	G	0.5688	1.548

18	rs199751262	77895078	G	0.002577	0.001667	A	0.5688	1.548
19	rs138633729	652838	A	0.002577	0.001667	G	0.5688	1.548
19	rs201171846	2234156	A	0.002577	0.001667	G	0.5688	1.548
19	rs61033781	2877597	A	0.002577	0.001667	G	0.5688	1.548
19	rs151065401	5867247	A	0.002577	0.001667	G	0.5688	1.548
19	rs202245509	7977267	A	0.002577	0.001667	G	0.5688	1.548
19	rs72999925	8962389	A	0.002577	0.001667	G	0.5688	1.548
19	rs6511720	11202306	A	0.002577	0.001667	C	0.5688	1.548
19	rs199580884	11834030	A	0.002577	0.001667	G	0.5688	1.548
19	rs182952954	11918051	G	0.002577	0.001667	A	0.5688	1.548
19	rs201711242	12812200	A	0.002577	0.001667	G	0.5688	1.548
19	rs201300850	14157021	A	0.002577	0.001667	G	0.5688	1.548
19	rs202194661	14826256	A	0.002577	0.001667	G	0.5688	1.548
19	rs115375345	15852908	A	0.002577	0.001667	G	0.5688	1.548
19	rs151104169	17014658	A	0.002577	0.001667	G	0.5688	1.548
19	rs189833366	17886295	T	0.002577	0.001667	A	0.5688	1.548
19	rs114464160	19612826	A	0.002577	0.001667	G	0.5688	1.548
19	rs200856432	33098729	G	0.002577	0.001667	A	0.5688	1.548
19	rs187942647	34843633	A	0.002577	0.001667	G	0.5688	1.548
19	rs202032887	36105948	A	0.002577	0.001667	G	0.5688	1.548
19	rs144951904	36494376	A	0.002577	0.001667	C	0.5688	1.548
19	rs141608600	44590505	A	0.002577	0.001667	T	0.5688	1.548
19	rs140681283	44611245	A	0.002577	0.001667	G	0.5688	1.548
19	rs143297439	45574894	A	0.002577	0.001667	G	0.5688	1.548
19	rs141966158	46914974	C	0.002577	0.001667	A	0.5688	1.548
19	rs150805394	47285707	A	0.002577	0.001667	G	0.5688	1.548
19	rs7462	49949652	A	0.002577	0.001667	G	0.5688	1.548
19	rs145883954	49962986	G	0.002577	0.001667	C	0.5688	1.548
19	exm1490818	49967981	C	0.002577	0.001667	A	0.5688	1.548
19	rs11558188	49967981	C	0.002577	0.001667	A	0.5688	1.548
19	rs199696801	50755984	A	0.002577	0.001667	G	0.5688	1.548
19	rs147229320	50985427	A	0.002577	0.001667	G	0.5688	1.548
19	rs148298627	51957528	A	0.002577	0.001667	G	0.5688	1.548
19	rs138080428	55707952	A	0.002577	0.001667	G	0.5688	1.548
19	rs138035807	55832347	A	0.002577	0.001667	G	0.5688	1.548
19	rs199686405	58574832	A	0.002577	0.001667	G	0.5688	1.548
20	rs35051116	372097	A	0.002577	0.001667	G	0.5688	1.548
20	rs3886999	3577062	A	0.002577	0.001667	G	0.5688	1.548
20	rs199981853	10030595	G	0.002577	0.001667	A	0.5688	1.548
20	rs148869724	13610664	A	0.002577	0.001667	G	0.5688	1.548
20	rs34537635	17622495	G	0.002577	0.001667	A	0.5688	1.548
20	rs200777037	32000565	G	0.002577	0.001667	A	0.5688	1.548
20	rs201573745	33567464	A	0.002577	0.001667	G	0.5688	1.548

20	rs185219770	34599150	A	0.002577	0.001667	C	0.5688	1.548
20	rs78442416	34797675	A	0.002577	0.001667	G	0.5688	1.548
20	rs139749355	36572456	A	0.002577	0.001667	G	0.5688	1.548
20	rs149184595	39992391	A	0.002577	0.001667	G	0.5688	1.548
20	rs148839313	56190012	G	0.002577	0.001667	A	0.5688	1.548
20	rs144781761	60892479	A	0.002577	0.001667	G	0.5688	1.548
20	exm2234723	61513248	A	0.002577	0.001667	C	0.5688	1.548
20	rs199612485	62594455	A	0.002577	0.001667	G	0.5688	1.548
21	rs182431978	19756012	A	0.002577	0.001667	C	0.5688	1.548
21	rs199887707	27284152	A	0.002577	0.001667	G	0.5688	1.548
21	rs200510636	35183493	A	0.002577	0.001667	G	0.5688	1.548
21	rs144653613	42778854	A	0.002577	0.001667	G	0.5688	1.548
21	rs61735791	42866439	A	0.002577	0.001667	G	0.5688	1.548
21	rs142112499	46308755	A	0.002577	0.001667	C	0.5688	1.548
21	rs35044802	47808772	A	0.002577	0.001667	G	0.5688	1.548
21	rs112853262	47831601	A	0.002577	0.001667	G	0.5688	1.548
22	rs149739505	17990861	A	0.002577	0.001667	G	0.5688	1.548
22	rs192602831	18274050	A	0.002577	0.001667	C	0.5688	1.548
22	rs204710	24717850	A	0.002577	0.001667	G	0.5688	1.548
22	rs113203306	26902835	A	0.002577	0.001667	G	0.5688	1.548
22	rs17812681	37578388	A	0.002577	0.001667	G	0.5688	1.548
22	rs143343534	38130910	A	0.002577	0.001667	G	0.5688	1.548
22	rs201348583	39497389	G	0.002577	0.001667	C	0.5688	1.548
22	rs201314224	46679924	C	0.002577	0.001667	G	0.5688	1.548
22	rs35503220	46708152	A	0.002577	0.001667	G	0.5688	1.548
22	rs200478512	46724650	C	0.002577	0.001667	G	0.5688	1.548
22	rs200487125	47085983	G	0.002577	0.001667	A	0.5688	1.548
22	rs148346577	47086007	A	0.002577	0.001667	C	0.5688	1.548
22	rs201033281	50307162	A	0.002577	0.001667	G	0.5688	1.548
22	rs144936442	51012057	A	0.002577	0.001667	G	0.5688	1.548
12	rs1797517	10041364	G	0.2938	0.31	A	0.5689	0.9261
3	rs4973760	27349047	A	0.1418	0.155	G	0.5692	0.9004
1	rs145442390	865665	A	0.002577	0.001669	G	0.5693	1.545
1	rs145687082	22149967	A	0.002577	0.001669	G	0.5693	1.545
1	rs149485004	236751262	G	0.002577	0.001669	A	0.5693	1.545
7	rs148058026	591062	A	0.002577	0.001669	G	0.5693	1.545
9	rs201007445	139848309	A	0.002577	0.001669	G	0.5693	1.545
11	rs202232848	4409718	C	0.002577	0.001669	G	0.5693	1.545
11	rs199515481	63525778	A	0.002577	0.001669	G	0.5693	1.545
11	rs145592178	119207100	A	0.002577	0.001669	G	0.5693	1.545
16	rs140254693	682738	A	0.002577	0.001669	G	0.5693	1.545
17	rs147736312	8215869	A	0.002577	0.001669	G	0.5693	1.545
17	rs150298204	18167897	C	0.002577	0.001669	A	0.5693	1.545

20	rs148274683	23432541	C	0.002577	0.001669	A	0.5693	1.545
4	rs9410	159631991	A	0.1598	0.1483	C	0.5694	1.092
12	rs2120991	54270228	A	0.1598	0.1483	C	0.5694	1.092
4	rs1560489	90198463	G	0.2964	0.3133	A	0.5697	0.9232
1	rs200490325	16464614	A	0.002577	0.001672	G	0.5698	1.543
1	rs7825	111957097	C	0.2242	0.2108	G	0.5698	1.082
3	rs60527165	129023630	A	0.1134	0.1033	G	0.5698	1.11
3	rs1047584	187088656	A	0.1134	0.1033	G	0.5698	1.11
13	rs78824256	114537621	A	0.002577	0.001672	G	0.5698	1.543
16	rs12443954	89741496	A	0.2036	0.2183	G	0.5698	0.9153
17	rs201763838	40737170	C	0.002577	0.001672	A	0.5698	1.543
19	rs7246479	55824332	C	0.2242	0.2108	A	0.5698	1.082
7	rs6959964	68905738	A	0.3222	0.3058	G	0.5699	1.079
21	rs80040922	43547165	A	0.2036	0.2192	G	0.5699	0.9109
22	rs4822502	24881781	G	0.3222	0.3058	A	0.5699	1.079
1	rs4926472	9670834	G	0.3222	0.3065	A	0.5701	1.075
3	rs142260634	136287658	G	0.002577	0.001675	A	0.5703	1.54
7	rs201766269	107684230	A	0.002577	0.001675	G	0.5703	1.54
8	rs201457448	27288501	G	0.002577	0.001675	A	0.5703	1.54
19	rs188212047	7706944	A	0.002591	0.001684	C	0.5703	1.54
1	rs1736557	171080080	A	0.2268	0.2125	G	0.5704	1.087
5	rs6893216	74442964	G	0.1624	0.15	A	0.5704	1.098
8	rs10106921	51543713	G	0.1624	0.15	A	0.5704	1.098
1	rs2284746	17306675	G	0.299	0.315	C	0.5707	0.9274
4	rs6449362	18194063	C	0.299	0.315	A	0.5707	0.9274
4	rs2869950	89742764	G	0.299	0.315	A	0.5707	0.9274
5	rs4867100	32007713	A	0.09794	0.1092	G	0.5709	0.886
8	rs180791960	22145047	C	0.002577	0.001678	A	0.5709	1.537
11	rs527709	106251514	A	0.09794	0.1092	G	0.5709	0.886
19	rs1465723	45017249	G	0.09794	0.1092	A	0.5709	0.886
9	rs2297538	139564668	A	0.09794	0.11	G	0.571	0.8784
22	rs16999173	39034786	A	0.09794	0.11	G	0.571	0.8784
1	rs7528405	22307538	G	0.3015	0.3183	A	0.5714	0.9245
10	rs11194886	111592608	A	0.3015	0.3183	G	0.5714	0.9245
13	rs10492664	108816225	A	0.3015	0.3177	G	0.5714	0.9271
14	rs191944410	74967636	A	0.002577	0.001681	G	0.5714	1.535
16	rs12598966	15961356	A	0.2268	0.2133	G	0.5714	1.082
3	rs76739135	36897630	A	0.04897	0.0425	C	0.5715	1.16
10	rs28943579	5141058	A	0.04897	0.0425	G	0.5715	1.16
12	rs11172113	57527283	G	0.2062	0.2217	A	0.5715	0.912
20	rs2232618	37001761	G	0.04897	0.0425	A	0.5715	1.16
1	rs76611705	223177974	A	0.07732	0.06917	G	0.5716	1.128
9	rs12380601	74648631	A	0.07732	0.06917	G	0.5716	1.128

20	rs6085920	7180056	T	0.07732	0.06917	A	0.5716	1.128
3	rs1803274	165491280	A	0.1624	0.1508	G	0.5719	1.091
6	rs17194474	30131711	G	0.002577	0.001684	C	0.5719	1.532
8	rs4242384	128518554	C	0.1624	0.1508	A	0.5719	1.091
13	rs9573974	77575137	G	0.116	0.105	A	0.5719	1.118
14	rs1970911	91773494	A	0.2294	0.215	G	0.572	1.087
1	rs1934625	210902915	A	0.3273	0.3117	G	0.5721	1.075
8	rs2879537	93698134	G	0.3273	0.3117	A	0.5721	1.075
5	rs7719411	65829867	G	0.3041	0.32	A	0.5725	0.9287
6	rs2523868	31016114	A	0.06443	0.07417	G	0.5727	0.8597
11	rs7121106	71191817	A	0.06443	0.07417	G	0.5727	0.8597
17	rs3744692	19643672	A	0.06443	0.07417	G	0.5727	0.8597
5	rs164515	141335498	A	0.1649	0.1525	G	0.5729	1.098
9	rs877809	77357968	A	0.1649	0.1525	G	0.5729	1.098
20	rs4911241	31140165	G	0.1649	0.1525	A	0.5729	1.098
6	rs3131115	30468791	A	0.3067	0.3233	G	0.5731	0.9258
16	rs9924951	7504854	A	0.2088	0.2242	G	0.5731	0.9132
5	rs2630767	176870700	G	0.2088	0.2233	A	0.5732	0.9175
6	rs1737046	29735693	A	0.3325	0.3158	G	0.5732	1.079
9	rs2282683	79117751	G	0.2088	0.2233	A	0.5732	0.9175
10	rs3802711	73544086	A	0.2088	0.2233	G	0.5732	0.9175
11	rs3765620	102595492	G	0.3325	0.3158	A	0.5732	1.079
13	rs7323310	87590833	C	0.3325	0.3158	A	0.5732	1.079
14	rs10140652	20691888	A	0.06443	0.075	C	0.5732	0.8494
14	rs9323693	20691962	C	0.06443	0.075	G	0.5732	0.8494
15	rs12912233	61267096	A	0.2088	0.2229	G	0.5733	0.92
2	rs146598485	198359384	G	0.02835	0.02333	A	0.5734	1.221
10	rs11013746	24133028	A	0.02835	0.02333	G	0.5734	1.221
11	rs11229281	57971478	G	0.02835	0.02333	A	0.5734	1.221
11	rs12787462	59573980	A	0.02835	0.02333	G	0.5734	1.221
12	rs2304821	70963619	A	0.02835	0.02333	G	0.5734	1.221
15	rs62621375	75111530	A	0.02835	0.02333	G	0.5734	1.221
15	rs147808783	75116023	A	0.02835	0.02333	G	0.5734	1.221
19	rs3746072	3179884	A	0.02835	0.02333	C	0.5734	1.221
6	rs17587	32825090	A	0.232	0.2175	G	0.5735	1.087
22	rs4077129	50356693	A	0.232	0.2175	G	0.5735	1.087
1	rs966365	236557771	G	0.03866	0.04667	A	0.5737	0.8215
3	rs17040154	14724345	A	0.116	0.1058	G	0.5737	1.108
6	rs9369640	12901441	C	0.03866	0.04667	A	0.5737	0.8215
6	rs2858881	32703845	G	0.03866	0.04667	A	0.5737	0.8215
6	rs9360921	76265642	C	0.03866	0.04667	A	0.5737	0.8215
14	rs8014119	23354066	C	0.03866	0.04667	G	0.5737	0.8215
14	rs3783697	58958923	G	0.116	0.1058	A	0.5737	1.108

6	rs3910312	30008746	C	0.3351	0.3183	A	0.574	1.079
11	rs2957778	24029671	A	0.3351	0.3183	G	0.574	1.079
22	rs5748919	17644131	G	0.3093	0.325	A	0.5742	0.93
2	rs231735	204693876	C	0.1649	0.1533	A	0.5743	1.091
19	rs2287803	10112346	A	0.1649	0.1533	G	0.5743	1.091
20	rs16989303	4169079	A	0.1649	0.1533	G	0.5743	1.091
5	rs10805890	73992881	G	0.232	0.2183	A	0.5744	1.081
10	rs7100408	8508228	G	0.232	0.2183	A	0.5744	1.081
12	rs4430553	66698895	G	0.3351	0.3192	A	0.5744	1.075
13	rs912969	103867104	A	0.232	0.2183	G	0.5744	1.081
19	rs1132899	45448036	A	0.3351	0.3192	G	0.5744	1.075
1	rs76065876	4772535	A	0.03866	0.0475	G	0.5745	0.8064
5	rs40497	56209735	G	0.03866	0.0475	A	0.5745	0.8064
10	rs78340846	38343858	A	0.03866	0.0475	G	0.5745	0.8064
14	rs12587478	20898347	A	0.03866	0.0475	G	0.5745	0.8064
17	rs981684	38935812	A	0.1469	0.16	G	0.5745	0.9041
17	rs77875845	79205783	A	0.03866	0.0475	G	0.5745	0.8064
8	rs2060145	129807905	G	0.1469	0.1608	A	0.5746	0.8985
16	rs11641035	8857749	A	0.1469	0.1608	G	0.5746	0.8985
2	rs28497373	239417840	G	0.2113	0.2267	A	0.5747	0.9143
3	rs6799969	8895531	G	0.3119	0.3283	A	0.5747	0.9271
3	rs2246901	195489009	C	0.2113	0.2267	A	0.5747	0.9143
12	rs11113995	108970462	A	0.2113	0.2267	G	0.5747	0.9143
1	rs28359608	230895340	A	0.0799	0.07083	C	0.5748	1.139
6	rs11155133	141169825	G	0.0799	0.07083	A	0.5748	1.139
9	rs2515629	107594364	G	0.0799	0.07083	A	0.5748	1.139
15	rs202148289	98504236	A	0.0799	0.07083	G	0.5748	1.139
17	rs61031471	7751138	A	0.2113	0.2258	C	0.5748	0.9186
19	rs57088011	53454387	G	0.0799	0.07083	C	0.5748	1.139
1	rs4660293	40028180	G	0.1005	0.1117	A	0.575	0.889
1	rs16842789	159854251	G	0.1005	0.1117	A	0.575	0.889
6	rs324368	155642564	A	0.1005	0.1117	G	0.575	0.889
8	exm2258573	10480211	G	0.1005	0.1125	A	0.575	0.8816
8	rs79329877	10480211	G	0.1005	0.1125	A	0.575	0.8816
11	rs58664826	5068297	A	0.1005	0.1125	G	0.575	0.8816
11	rs57026471	5068662	A	0.1005	0.1125	G	0.575	0.8816
11	rs61746343	5080337	G	0.1005	0.1117	A	0.575	0.889
17	rs61731746	7011225	C	0.1005	0.1117	A	0.575	0.889
20	rs1321936	12826999	G	0.0799	0.07095	A	0.5751	1.137
21	rs492338	43701977	G	0.3376	0.3217	A	0.5751	1.075
22	rs11479	50964236	A	0.2345	0.22	G	0.5751	1.086
8	rs2897333	35688841	A	0.1675	0.155	C	0.5754	1.097
1	rs3811466	222717132	A	0.3144	0.3317	G	0.5755	0.9242

6	rs793834	24234933	A	0.3144	0.3317	G	0.5755	0.9242
6	rs7762160	166015431	G	0.3144	0.3308	A	0.5755	0.9277
16	rs4786150	7442293	G	0.3144	0.3317	A	0.5755	0.9242
21	rs2832191	30489300	A	0.3144	0.3308	C	0.5755	0.9277
1	rs1998027	231326086	G	0.3402	0.3242	A	0.5758	1.075
15	rs78069432	75893532	A	0.1186	0.1075	G	0.5758	1.117
7	rs6952195	7514235	G	0.2345	0.2208	C	0.5759	1.081
3	rs358806	55313400	A	0.2139	0.2292	C	0.5762	0.9154
3	rs60460891	160168769	G	0.2139	0.2292	A	0.5762	0.9154
5	rs2032794	86432617	G	0.2139	0.2292	A	0.5762	0.9154
11	rs10793379	70731148	G	0.2139	0.2292	A	0.5762	0.9154
4	rs3748034	3446091	A	0.317	0.3342	C	0.5763	0.9248
10	rs10999516	72517830	A	0.317	0.3333	G	0.5763	0.9283
16	rs863980	4751045	G	0.317	0.3331	A	0.5763	0.9295
13	rs7335046	100041738	G	0.3402	0.325	C	0.5765	1.071
14	rs13021	39650924	G	0.3402	0.325	A	0.5765	1.071
1	rs2242637	44401384	A	0.2371	0.2225	G	0.5766	1.086
12	rs1064319	119866533	G	0.2371	0.2225	A	0.5766	1.086
14	rs12431733	54290830	G	0.2371	0.2225	A	0.5766	1.086
22	rs140524	50960682	A	0.2371	0.2225	G	0.5766	1.086
3	rs12629106	177095072	A	0.1675	0.1558	G	0.5767	1.09
11	rs2282580	126081403	A	0.1675	0.1558	G	0.5767	1.09
15	rs3100139	45047573	G	0.1675	0.1558	A	0.5767	1.09
2	rs2228331	241405528	G	0.3196	0.3367	A	0.577	0.9254
3	rs56084453	44762830	G	0.05155	0.04417	A	0.577	1.176
3	rs60516581	125702113	C	0.05155	0.04417	A	0.577	1.176
7	rs79848686	138329572	A	0.05155	0.04417	G	0.577	1.176
11	rs11018879	89880604	A	0.05155	0.04417	G	0.577	1.176
19	rs541169	35719020	A	0.1495	0.1625	G	0.577	0.9058
1	rs12042938	231816842	G	0.1495	0.1633	A	0.5771	0.9003
11	rs7116662	105393840	A	0.3196	0.3358	G	0.5771	0.9289
2	rs887992	103524931	A	0.3454	0.3292	C	0.5772	1.075
4	rs1134921	843508	A	0.1675	0.1561	G	0.5772	1.088
5	rs1366589	84886843	A	0.3454	0.3292	G	0.5772	1.075
6	rs6456746	10894381	A	0.1675	0.1561	G	0.5772	1.088
7	rs2293767	100361675	A	0.3454	0.3292	G	0.5772	1.075
1	rs2273346	11090897	G	0.2371	0.2233	A	0.5773	1.081
4	rs2685820	190758884	G	0.2371	0.2233	A	0.5773	1.081
13	rs9554897	103383417	G	0.2371	0.2233	A	0.5773	1.081
13	rs9300756	103384824	G	0.2371	0.2233	A	0.5773	1.081
1	rs849961	37577214	A	0.1186	0.1083	G	0.5774	1.107
2	rs1063479	68622914	G	0.1186	0.1083	A	0.5774	1.107
13	rs11070136	96239805	A	0.1186	0.1083	G	0.5774	1.107

22	rs34422484	50696678	A	0.1186	0.1083	G	0.5774	1.107
19	rs28362459	5844792	C	0.3454	0.3297	A	0.5775	1.072
6	rs7774197	32046275	C	0.0799	0.07167	A	0.5776	1.125
1	rs192791486	1154157	A	0	0.003333	G	0.5777	0
1	rs114192897	3551783	G	0	0.003333	A	0.5777	0
1	rs143443665	7863826	A	0	0.003333	T	0.5777	0
1	exm20768	16269040	C	0	0.003333	G	0.5777	0
1	rs201524531	16269040	C	0	0.003333	G	0.5777	0
1	rs146573703	17592187	A	0	0.003333	G	0.5777	0
1	rs138522903	17596797	A	0	0.003333	G	0.5777	0
1	rs202006379	31899521	A	0	0.003333	G	0.5777	0
1	rs200994339	36553686	G	0	0.003333	A	0.5777	0
1	rs79051491	52821952	A	0	0.003333	G	0.5777	0
1	rs141759859	54474738	A	0	0.003333	G	0.5777	0
1	rs10157422	67518534	A	0	0.003333	G	0.5777	0
1	rs114177761	78267157	C	0	0.003333	A	0.5777	0
1	rs145826692	78279446	A	0	0.003333	G	0.5777	0
1	rs193167060	91403464	G	0	0.003333	A	0.5777	0
1	rs149597385	91405998	A	0	0.003333	G	0.5777	0
1	rs148460146	94471025	A	0	0.003333	G	0.5777	0
1	rs183410431	94685903	A	0	0.003333	G	0.5777	0
1	rs201239079	95330427	G	0	0.003333	A	0.5777	0
1	rs148639495	100964808	A	0	0.003333	G	0.5777	0
1	rs200684981	110882221	C	0	0.003333	G	0.5777	0
1	rs142648390	113246274	A	0	0.003333	G	0.5777	0
1	rs79050956	114437707	G	0	0.003333	A	0.5777	0
1	rs183201500	115167999	A	0	0.003333	T	0.5777	0
1	rs148217136	118426147	A	0	0.003333	G	0.5777	0
1	rs139990606	150526051	A	0	0.003333	G	0.5777	0
1	rs138102093	150960423	A	0	0.003333	G	0.5777	0
1	rs201517519	153279615	A	0	0.003333	G	0.5777	0
1	rs142724511	156206205	A	0	0.003333	G	0.5777	0
1	rs184429811	158301161	A	0	0.003333	G	0.5777	0
1	rs201020976	159888645	A	0	0.003333	G	0.5777	0
1	rs199501005	161683096	A	0	0.003333	G	0.5777	0
1	rs200927034	169394083	C	0	0.003333	A	0.5777	0
1	rs6125	169582317	A	0	0.003333	G	0.5777	0
1	rs142430695	180063248	G	0	0.003333	A	0.5777	0
1	rs200327975	186038870	A	0	0.003333	G	0.5777	0
1	rs184969664	197102571	A	0	0.003333	T	0.5777	0
1	rs199754074	203677138	A	0	0.003333	G	0.5777	0
1	rs34660159	207196639	C	0	0.003333	A	0.5777	0
1	rs77877052	207196760	G	0	0.003333	A	0.5777	0

1	rs35299018	207196790	G	0	0.003333	A	0.5777		0
1	rs200399460	226923911	A	0	0.003344	G	0.5777		0
1	rs117609949	235952111	A	0	0.003333	T	0.5777		0
1	rs117720055	241767854	G	0	0.003333	A	0.5777		0
1	rs141495513	247201569	A	0	0.003333	C	0.5777		0
1	rs147056410	247835749	A	0	0.003333	G	0.5777		0
1	rs150445512	248309326	A	0	0.003333	G	0.5777		0
2	rs143269114	21230249	A	0	0.003333	G	0.5777		0
2	rs151243708	24521652	A	0	0.003333	G	0.5777		0
2	rs148181027	26537311	A	0	0.003333	G	0.5777		0
2	rs138901368	29366627	A	0	0.003333	G	0.5777		0
2	rs149968229	29754824	A	0	0.003333	G	0.5777		0
2	rs186220147	32654266	C	0	0.003333	A	0.5777		0
2	rs61757604	39095403	A	0	0.003333	G	0.5777		0
2	rs202033109	44139587	G	0	0.003333	A	0.5777		0
2	rs62133344	69177269	A	0	0.003333	C	0.5777		0
2	rs143087335	88485535	A	0	0.003333	G	0.5777		0
2	rs146972264	97638311	A	0	0.003333	G	0.5777		0
2	rs137868721	106498333	A	0	0.003333	G	0.5777		0
2	rs148715226	128370111	A	0	0.003333	G	0.5777		0
2	rs181892289	128412409	A	0	0.003333	C	0.5777		0
2	rs151301223	136029457	G	0	0.003333	A	0.5777		0
2	rs201048662	160303360	A	0	0.003333	G	0.5777		0
2	rs199938643	168101944	A	0	0.003333	G	0.5777		0
2	rs187005870	202712127	A	0	0.003333	T	0.5777		0
2	rs17385675	202807196	A	0	0.003333	G	0.5777		0
2	rs200677791	204313498	A	0	0.003333	G	0.5777		0
2	rs145761335	204599564	A	0	0.003333	G	0.5777		0
2	rs13022398	216577567	A	0	0.003333	C	0.5777		0
2	rs201853142	219299361	G	0	0.003333	A	0.5777		0
2	rs143759265	219614067	A	0	0.003333	G	0.5777		0
2	rs138484554	223504326	G	0	0.003333	A	0.5777		0
2	rs140493973	228205039	A	0	0.003333	G	0.5777		0
2	rs115729513	238296675	A	0	0.003333	G	0.5777		0
2	rs80234134	242407741	A	0	0.003333	G	0.5777		0
3	rs116261368	361547	C	0	0.003333	A	0.5777		0
3	rs150478813	3072635	A	0	0.003333	C	0.5777		0
3	rs139321972	8983405	G	0	0.003333	A	0.5777		0
3	rs145374241	9965642	A	0	0.003333	G	0.5777		0
3	rs9845475	32842101	C	0	0.003333	A	0.5777		0
3	rs79233786	38438638	A	0	0.003333	G	0.5777		0
3	rs150501065	38770349	A	0	0.003333	G	0.5777		0
3	rs76132393	47079245	G	0	0.003333	A	0.5777		0

3	rs200256458	48215844	A	0	0.003333	G	0.5777	0
3	rs148970948	50413290	A	0	0.003333	G	0.5777	0
3	rs146731697	52834608	A	0	0.003333	G	0.5777	0
3	rs201711185	66023754	A	0	0.003333	G	0.5777	0
3	rs117931398	66455701	G	0	0.003333	C	0.5777	0
3	rs200227143	107886661	C	0	0.003333	A	0.5777	0
3	rs140154344	108288408	G	0	0.003333	A	0.5777	0
3	rs201448153	109049430	C	0	0.003333	G	0.5777	0
3	rs189157672	112358343	A	0	0.003333	G	0.5777	0
3	rs146278925	119120980	A	0	0.003333	C	0.5777	0
3	rs3218636	121208894	G	0	0.003333	A	0.5777	0
3	rs147708058	121500607	G	0	0.003333	A	0.5777	0
3	rs181369854	123633951	A	0	0.003333	G	0.5777	0
3	rs150679929	133474225	A	0	0.003333	G	0.5777	0
3	rs187345906	134322630	A	0	0.003333	G	0.5777	0
3	rs191779932	151458518	G	0	0.003333	A	0.5777	0
3	rs144969124	155198866	A	0	0.003333	G	0.5777	0
3	rs34273285	169485588	C	0	0.003333	G	0.5777	0
3	rs139790884	170585827	A	0	0.003333	G	0.5777	0
3	rs145038110	172504364	A	0	0.003333	G	0.5777	0
3	rs79428658	195477779	A	0	0.003333	G	0.5777	0
4	rs2234573	1985717	A	0	0.00335	G	0.5777	0
4	rs200793789	6578378	A	0	0.003333	G	0.5777	0
4	rs146608974	7062894	A	0	0.003333	G	0.5777	0
4	rs11941659	8588986	A	0	0.003333	G	0.5777	0
4	rs75897705	10447628	A	0	0.003333	G	0.5777	0
4	rs187778411	13600728	A	0	0.003333	G	0.5777	0
4	rs144018108	16760830	A	0	0.003333	G	0.5777	0
4	rs201074166	36093553	A	0	0.003333	G	0.5777	0
4	rs146872442	38907236	G	0	0.003333	C	0.5777	0
4	rs201590132	76708226	G	0	0.003333	A	0.5777	0
4	rs77937508	106890077	A	0	0.003333	G	0.5777	0
4	rs192244114	110638865	G	0	0.003333	A	0.5777	0
4	rs185081816	110638871	A	0	0.003333	T	0.5777	0
4	rs192190120	148407089	G	0	0.003333	A	0.5777	0
4	rs137869187	156269027	A	0	0.003333	G	0.5777	0
4	rs115262512	164393508	G	0	0.003333	A	0.5777	0
4	rs149684063	187117196	G	0	0.003333	A	0.5777	0
5	rs6345	1422073	A	0	0.003333	G	0.5777	0
5	rs139951158	32010445	A	0	0.003333	G	0.5777	0
5	rs141143848	35955811	A	0	0.003333	G	0.5777	0
5	rs199877909	54585248	G	0	0.003333	A	0.5777	0
5	rs200385766	94865856	A	0	0.003333	G	0.5777	0

5	rs142276540	132652318	A	0	0.003333	G	0.5777	0
5	rs200482355	151784557	A	0	0.003333	G	0.5777	0
5	rs398829	178634672	G	0.3222	0.3392	A	0.5777	0.926
5	rs199854262	179263586	A	0	0.003333	G	0.5777	0
6	rs184429527	2890543	A	0	0.003333	C	0.5777	0
6	rs34964470	7246834	A	0	0.003333	G	0.5777	0
6	rs77460377	15638035	G	0	0.003344	C	0.5777	0
6	rs62405964	29142020	G	0	0.003333	A	0.5777	0
6	rs143020056	30080405	A	0	0.003333	G	0.5777	0
6	rs11754099	30329101	A	0	0.003333	G	0.5777	0
6	rs145717144	31670982	A	0	0.003333	G	0.5777	0
6	rs55767910	32818242	G	0	0.003333	A	0.5777	0
6	rs202178128	35773782	G	0	0.003333	A	0.5777	0
6	rs199508851	39159142	A	0	0.003333	G	0.5777	0
6	rs139617182	41197268	A	0	0.003333	G	0.5777	0
6	rs2297980	54173413	G	0	0.003333	A	0.5777	0
6	rs3811069	96651740	A	0	0.003333	G	0.5777	0
6	rs189889136	97247079	G	0	0.003333	A	0.5777	0
6	rs200372976	99323548	A	0	0.003333	G	0.5777	0
6	rs184127828	129824266	C	0	0.003333	A	0.5777	0
6	rs148941985	134583213	A	0	0.003333	G	0.5777	0
6	rs149799182	136599627	A	0	0.003333	G	0.5777	0
6	rs201015207	144183246	G	0	0.003333	A	0.5777	0
6	rs190201915	147123086	G	0	0.003333	A	0.5777	0
6	rs146113958	151673577	G	0	0.003333	A	0.5777	0
6	rs138740409	155450580	A	0	0.003333	G	0.5777	0
7	rs12534093	23502974	A	0	0.003333	T	0.5777	0
7	rs2228078	31018852	G	0	0.003333	A	0.5777	0
7	exm617952	44573130	A	0	0.003344	G	0.5777	0
7	rs200121935	47947839	G	0	0.003333	A	0.5777	0
7	rs139969494	70597970	G	0	0.003333	A	0.5777	0
7	rs139636296	73646449	A	0	0.003333	G	0.5777	0
7	rs74839397	90747440	A	0	0.003333	G	0.5777	0
7	rs150412190	105429058	A	0	0.003333	G	0.5777	0
7	rs199685282	123349183	G	0	0.003333	A	0.5777	0
7	rs114216747	150749260	A	0	0.003333	G	0.5777	0
7	rs199876601	151071225	A	0	0.003333	G	0.5777	0
7	rs139770288	151874709	A	0	0.003333	G	0.5777	0
7	rs199630455	158827277	G	0	0.003333	A	0.5777	0
8	rs505480	12878843	A	0	0.003333	C	0.5777	0
8	rs1805158	18257703	A	0	0.003333	G	0.5777	0
8	rs116429520	25319678	G	0	0.003333	A	0.5777	0
8	rs140371319	26721750	A	0	0.003333	G	0.5777	0

8	rs201292574	38677393	A	0	0.003333	G	0.5777	0
8	rs200135800	55539393	G	0	0.003333	A	0.5777	0
8	rs200631708	107773278	A	0	0.003333	G	0.5777	0
8	rs7003306	142459814	G	0	0.003339	A	0.5777	0
8	rs200239582	145141021	A	0	0.003333	G	0.5777	0
8	rs201857433	145698765	A	0	0.003333	G	0.5777	0
9	rs202112833	13119542	A	0	0.003333	G	0.5777	0
9	rs187388944	14805021	G	0	0.003333	A	0.5777	0
9	rs201225408	18635986	A	0	0.003333	G	0.5777	0
9	rs1334811	27190655	A	0	0.003333	G	0.5777	0
9	rs201283769	33944445	G	0	0.003333	A	0.5777	0
9	rs192125496	109691399	G	0	0.003333	A	0.5777	0
9	rs142466437	111741679	A	0	0.003333	G	0.5777	0
9	rs200540250	130494493	A	0	0.003333	G	0.5777	0
9	rs142316378	132897380	A	0	0.003333	G	0.5777	0
9	rs34634745	133759794	A	0	0.003333	C	0.5777	0
9	rs199710577	135277472	A	0	0.003333	G	0.5777	0
9	rs202068817	136270517	A	0	0.003333	C	0.5777	0
9	rs142060916	136323080	A	0	0.003333	G	0.5777	0
9	rs144997410	139749302	A	0	0.003333	G	0.5777	0
9	rs117093136	139751164	A	0	0.003333	G	0.5777	0
9	rs74415508	140166982	A	0	0.003333	G	0.5777	0
9	rs28671254	140242564	A	0	0.003333	G	0.5777	0
10	rs76810894	63976914	A	0	0.003333	G	0.5777	0
10	rs188248522	67726392	A	0	0.003333	G	0.5777	0
10	rs199873479	83635785	A	0	0.003333	G	0.5777	0
10	rs112208466	90699265	A	0	0.003333	G	0.5777	0
10	rs201576692	97987252	A	0	0.003333	C	0.5777	0
10	rs182758486	98742919	G	0	0.003333	C	0.5777	0
10	rs29001314	99226656	C	0	0.003333	G	0.5777	0
10	rs11189525	100017553	A	0	0.003333	C	0.5777	0
10	rs185283450	103589693	G	0	0.003333	C	0.5777	0
10	rs74661743	105361884	A	0	0.003333	G	0.5777	0
10	rs200938753	105362610	A	0	0.003333	G	0.5777	0
10	rs12243326	114788815	G	0	0.003333	A	0.5777	0
10	rs187990373	115380430	A	0	0.003333	T	0.5777	0
10	rs145424503	116038571	A	0	0.003333	G	0.5777	0
10	rs137929196	121586520	A	0	0.003333	G	0.5777	0
10	rs187753368	121612703	T	0	0.003333	A	0.5777	0
10	rs143736049	124802605	A	0	0.003333	G	0.5777	0
10	rs199590785	126370390	A	0	0.003333	G	0.5777	0
10	rs201458514	134523914	A	0	0.003333	G	0.5777	0
11	rs199936023	5080661	A	0	0.003333	C	0.5777	0

11	rs188458277	5530504	A	0	0.003333	C	0.5777	0
11	rs186456618	5602798	G	0	0.003333	A	0.5777	0
11	rs200029552	8729362	A	0	0.003333	G	0.5777	0
11	rs66830472	16007446	A	0.3222	0.3392	G	0.5777	0.926
11	rs2234584	32450067	A	0	0.003333	G	0.5777	0
11	rs182257970	44069816	A	0	0.003333	G	0.5777	0
11	rs147787174	45307634	A	0	0.003333	G	0.5777	0
11	rs35690719	47364286	A	0	0.003333	G	0.5777	0
11	rs199726652	55587812	A	0	0.003333	G	0.5777	0
11	rs140538227	60617465	A	0	0.003333	G	0.5777	0
11	rs188010920	60709508	A	0	0.003333	G	0.5777	0
11	rs200277476	61724418	A	0	0.003333	G	0.5777	0
11	rs200860039	62384115	A	0	0.003333	G	0.5777	0
11	rs202213951	64675078	A	0	0.003333	G	0.5777	0
11	rs139176991	64698961	A	0	0.003333	G	0.5777	0
11	rs143145862	65630970	A	0	0.003339	G	0.5777	0
11	rs61740616	85420401	A	0	0.003333	G	0.5777	0
11	rs35751209	85445734	G	0	0.003333	A	0.5777	0
11	rs139330011	101323712	A	0	0.003333	G	0.5777	0
11	rs34417210	110451721	C	0	0.003333	A	0.5777	0
11	rs200170353	118133741	A	0	0.003333	G	0.5777	0
11	rs147205617	123513271	A	0	0.003333	G	0.5777	0
12	rs147388541	331781	A	0	0.003333	G	0.5777	0
12	rs41276696	1940514	A	0	0.003333	G	0.5777	0
12	rs202182125	6567847	A	0	0.003333	G	0.5777	0
12	rs200427740	10342716	A	0	0.003333	G	0.5777	0
12	rs200573264	12630641	A	0	0.003333	G	0.5777	0
12	rs143183040	14695176	A	0	0.003333	C	0.5777	0
12	rs140390981	15654555	A	0	0.003333	G	0.5777	0
12	rs138191568	32134413	A	0	0.003333	G	0.5777	0
12	rs190043383	32906865	G	0	0.003333	C	0.5777	0
12	rs145966332	38714159	G	0	0.003333	A	0.5777	0
12	rs117472187	49317589	A	0	0.003333	G	0.5777	0
12	rs186696516	49427347	A	0	0.003333	G	0.5777	0
12	rs201581582	49434507	A	0	0.003333	G	0.5777	0
12	rs201327431	49743206	A	0	0.003333	G	0.5777	0
12	rs186697175	50189803	G	0	0.003333	C	0.5777	0
12	rs17688672	53223865	G	0	0.003333	A	0.5777	0
12	exm-IND12-5436	56075648	D	0	0.003333	I	0.5777	0
12	rs151238555	56818610	A	0	0.003333	G	0.5777	0
12	rs199752894	57556673	G	0	0.003333	C	0.5777	0
12	rs782097	58814968	A	0.3222	0.3392	G	0.5777	0.926
12	rs189155628	78225384	C	0	0.003333	A	0.5777	0

12	rs142393083	109677729	A	0	0.003333	G	0.5777	0
12	rs143862876	112184865	G	0	0.003333	A	0.5777	0
12	rs144613390	113730816	A	0	0.003333	G	0.5777	0
12	rs145677929	113822717	A	0	0.003333	G	0.5777	0
12	rs1546115	128209061	A	0	0.003333	G	0.5777	0
12	rs142639224	130927022	A	0	0.003339	G	0.5777	0
13	rs202087877	41515418	G	0	0.003333	A	0.5777	0
13	rs201847271	67477707	A	0	0.003333	G	0.5777	0
13	rs187677432	96599295	G	0	0.003333	A	0.5777	0
13	rs189869941	99337039	G	0	0.003333	A	0.5777	0
13	rs200491395	111294750	A	0	0.003333	G	0.5777	0
14	rs186131311	23354213	A	0	0.003333	G	0.5777	0
14	rs142449845	24545458	A	0	0.003333	G	0.5777	0
14	rs201340475	24572402	A	0	0.003333	G	0.5777	0
14	rs111723834	24572932	A	0	0.003333	G	0.5777	0
14	rs200803889	24883964	A	0	0.003333	C	0.5777	0
14	rs854384	25271520	A	0	0.003333	G	0.5777	0
14	rs202213974	70246047	A	0	0.003333	G	0.5777	0
14	rs138684775	76548974	A	0	0.003333	G	0.5777	0
14	rs78037497	91779567	A	0	0.003333	G	0.5777	0
14	rs2296687	94121728	G	0	0.003333	A	0.5777	0
15	rs201521227	40265116	G	0	0.003333	A	0.5777	0
15	rs55683312	41800366	G	0	0.003333	A	0.5777	0
15	rs200897167	42192923	A	0	0.003333	G	0.5777	0
15	rs10643	45981317	A	0	0.003333	G	0.5777	0
15	rs114277501	62209734	A	0	0.003333	G	0.5777	0
15	rs192138453	62256948	C	0	0.003333	A	0.5777	0
15	rs75413920	63102176	A	0	0.003333	G	0.5777	0
15	rs185487514	65110254	C	0	0.003333	A	0.5777	0
15	rs118062397	65113687	G	0	0.003333	A	0.5777	0
15	rs142546832	72170478	G	0	0.003333	A	0.5777	0
15	rs201316363	72457714	G	0	0.003333	C	0.5777	0
15	rs185797496	72645431	T	0	0.003333	A	0.5777	0
15	rs5742915	74336633	G	0	0.003333	A	0.5777	0
15	rs200430821	74865203	G	0	0.003333	A	0.5777	0
15	rs150525282	75188640	G	0	0.003333	A	0.5777	0
16	rs200353362	624694	A	0	0.003344	G	0.5777	0
16	rs190614894	1412297	A	0	0.003333	G	0.5777	0
16	rs45509392	2121610	A	0	0.003333	G	0.5777	0
16	rs201897065	4513722	A	0	0.003333	G	0.5777	0
16	rs188503332	10534276	G	0	0.003333	A	0.5777	0
16	rs63750428	16248624	A	0	0.003333	G	0.5777	0
16	rs190557767	16263649	A	0	0.003333	G	0.5777	0

16	rs59002125	16276741	A	0	0.003333	G	0.5777	0
16	rs150866831	16281065	A	0	0.003333	G	0.5777	0
16	rs139454085	20975854	A	0	0.003333	G	0.5777	0
16	rs58508496	21157406	A	0	0.003333	G	0.5777	0
16	rs183932619	22343448	A	0	0.003333	G	0.5777	0
16	rs139014248	24580647	A	0	0.003333	G	0.5777	0
16	rs61746620	25255366	A	0	0.003333	G	0.5777	0
16	rs201680708	28995189	A	0	0.003333	G	0.5777	0
16	rs138824667	30081477	A	0	0.003333	G	0.5777	0
16	rs112002818	57935442	A	0	0.003333	G	0.5777	0
16	rs187504367	81030993	A	0	0.003333	G	0.5777	0
16	rs187454354	81953195	A	0	0.003333	G	0.5777	0
16	rs199593264	87678548	A	0	0.003333	G	0.5777	0
16	rs143284523	88872202	A	0	0.003333	G	0.5777	0
16	rs200146632	89211706	A	0	0.003333	G	0.5777	0
16	rs190143218	89357538	C	0	0.003333	G	0.5777	0
17	rs201160838	465956	G	0	0.003333	A	0.5777	0
17	rs61733096	2276707	A	0	0.003333	G	0.5777	0
17	rs200425446	4586159	A	0	0.003333	G	0.5777	0
17	rs200036545	4794961	G	0	0.003333	A	0.5777	0
17	rs199949982	4855170	G	0	0.003333	C	0.5777	0
17	rs202145312	5347793	A	0	0.003333	G	0.5777	0
17	rs183966301	8132702	A	0	0.003333	G	0.5777	0
17	rs200137992	8141754	A	0	0.003333	G	0.5777	0
17	rs191837780	10554922	A	0	0.003333	G	0.5777	0
17	rs201073816	18054497	A	0	0.003333	G	0.5777	0
17	rs200802399	34344904	A	0	0.003333	G	0.5777	0
17	rs187943557	34956121	A	0	0.003333	G	0.5777	0
17	rs201477948	39537456	A	0	0.003333	G	0.5777	0
17	rs199599875	39998487	A	0	0.003333	G	0.5777	0
17	rs138695961	40695346	A	0	0.003333	G	0.5777	0
17	rs199604208	42161214	G	0	0.003333	A	0.5777	0
17	rs200841211	57335131	A	0	0.003333	G	0.5777	0
17	rs201285735	72926794	A	0	0.003333	G	0.5777	0
17	rs199937273	73627666	G	0	0.003333	A	0.5777	0
17	rs200154594	74005880	A	0	0.003333	C	0.5777	0
17	rs3744064	75211208	A	0	0.003333	G	0.5777	0
17	rs143004059	76831490	A	0	0.003333	G	0.5777	0
17	rs137969068	77987325	A	0	0.003333	G	0.5777	0
17	rs79563828	79638824	A	0	0.003333	G	0.5777	0
17	rs143981268	80273291	A	0	0.003333	G	0.5777	0
18	rs190733334	6859874	A	0	0.003333	G	0.5777	0
18	rs148593258	7016490	A	0	0.003333	G	0.5777	0

18	rs116551011	28574152	G	0	0.003333	A	0.5777	0
18	rs148885679	61256025	A	0	0.003333	G	0.5777	0
19	rs142624165	881560	A	0	0.003333	G	0.5777	0
19	rs200897976	1453017	A	0	0.003333	G	0.5777	0
19	rs114206435	2853364	G	0	0.003333	A	0.5777	0
19	rs202172544	3543908	A	0	0.00335	G	0.5777	0
19	rs150606217	4538608	A	0	0.003333	G	0.5777	0
19	rs186706068	5751781	A	0	0.003333	G	0.5777	0
19	rs200967589	6711204	A	0	0.003339	G	0.5777	0
19	rs28599881	9225694	A	0	0.003333	G	0.5777	0
19	rs150624810	9721564	A	0	0.003333	G	0.5777	0
19	rs3745268	10335403	A	0	0.003333	G	0.5777	0
19	rs140594440	10472598	A	0	0.003333	G	0.5777	0
19	rs201089328	10883303	A	0	0.003333	G	0.5777	0
19	rs181639639	11322772	A	0	0.00335	G	0.5777	0
19	rs117204726	14270289	A	0	0.003339	G	0.5777	0
19	rs145982795	14273649	A	0	0.003333	G	0.5777	0
19	rs192329346	14755036	A	0	0.003333	C	0.5777	0
19	rs2285981	15302649	A	0	0.003333	G	0.5777	0
19	rs115517770	16008283	A	0	0.003339	G	0.5777	0
19	rs3826726	16620328	A	0	0.003333	G	0.5777	0
19	rs16982285	18376509	A	0	0.003333	G	0.5777	0
19	rs12462974	18376780	G	0	0.003333	A	0.5777	0
19	rs144034570	33439219	G	0	0.003333	A	0.5777	0
19	rs35021937	38979891	G	0	0.003333	A	0.5777	0
19	rs140514392	40225044	A	0	0.003333	C	0.5777	0
19	rs151110565	40902977	A	0	0.003333	G	0.5777	0
19	rs117101875	42820695	A	0	0.003333	G	0.5777	0
19	rs182789975	44470012	G	0	0.003333	A	0.5777	0
19	rs144515600	44738879	A	0	0.003333	T	0.5777	0
19	rs139979975	44740441	A	0	0.003333	G	0.5777	0
19	rs141091843	46021238	A	0	0.003333	G	0.5777	0
19	rs201002877	47124591	A	0	0.003333	C	0.5777	0
19	rs148184205	49123796	A	0	0.003333	G	0.5777	0
19	rs202060707	49142250	A	0	0.003333	G	0.5777	0
19	rs35209148	50940938	A	0	0.003333	C	0.5777	0
19	rs201524467	51453210	A	0	0.003333	G	0.5777	0
19	rs202164468	51483558	A	0	0.003333	G	0.5777	0
19	rs187983315	53385146	A	0	0.003333	G	0.5777	0
19	rs146786265	54297343	A	0	0.003333	G	0.5777	0
19	rs201722303	55653283	A	0	0.003333	G	0.5777	0
19	rs200414554	55967789	A	0	0.003333	T	0.5777	0
19	rs115243845	56320376	A	0	0.003333	G	0.5777	0

20	rs148817519	2796341	C	0	0.003333	G	0.5777	0
20	rs201219750	6022222	G	0	0.003333	A	0.5777	0
20	rs61750849	30584710	A	0	0.003333	G	0.5777	0
20	rs75863437	33519830	A	0	0.003333	G	0.5777	0
20	rs201071715	37203537	A	0	0.003333	G	0.5777	0
20	rs147370500	40054766	A	0	0.003333	G	0.5777	0
20	rs34405647	44983713	A	0	0.003333	G	0.5777	0
20	rs138354653	45194945	A	0	0.003333	G	0.5777	0
20	rs200221009	48273186	A	0	0.003333	G	0.5777	0
20	rs3736832	55908269	G	0	0.003333	A	0.5777	0
20	rs148044740	61909542	A	0	0.003333	G	0.5777	0
20	rs2273499	61910270	A	0	0.003333	G	0.5777	0
21	rs7280643	15599466	G	0	0.003333	A	0.5777	0
21	rs457947	28338298	G	0	0.003333	C	0.5777	0
21	rs999597	31803054	A	0	0.003333	G	0.5777	0
21	rs437484	43531494	A	0	0.003333	G	0.5777	0
21	rs185842266	45750112	A	0	0.003333	G	0.5777	0
21	rs144412484	45810817	G	0	0.003333	A	0.5777	0
21	rs200346541	45837892	A	0	0.003333	G	0.5777	0
21	rs138703739	46311816	C	0	0.003333	G	0.5777	0
22	rs113123350	19163991	G	0	0.003333	A	0.5777	0
22	rs200317892	19221123	A	0	0.003333	G	0.5777	0
22	rs202190981	22892362	A	0	0.003339	G	0.5777	0
22	rs201618130	24726329	A	0	0.003333	G	0.5777	0
22	rs200544443	25115747	G	0	0.003333	A	0.5777	0
22	rs184759146	26838430	C	0	0.003339	A	0.5777	0
22	rs115278158	30864655	G	0	0.003333	A	0.5777	0
22	rs114438349	30866053	A	0	0.003333	G	0.5777	0
22	rs61744139	30888492	C	0	0.003333	G	0.5777	0
22	rs55674628	30953280	A	0	0.003333	G	0.5777	0
22	rs9621049	31013419	A	0	0.003333	G	0.5777	0
22	rs55984643	31536074	A	0	0.003333	T	0.5777	0
22	rs61731667	32218727	A	0	0.003333	C	0.5777	0
22	rs61731662	32266603	G	0	0.003333	A	0.5777	0
22	rs146449468	32302045	A	0	0.003333	G	0.5777	0
22	rs5754014	32756793	A	0	0.003333	G	0.5777	0
22	rs200003579	37420713	A	0	0.003333	G	0.5777	0
22	rs149941944	38474490	A	0	0.003333	G	0.5777	0
22	rs200907864	40661105	A	0	0.003333	G	0.5777	0
22	rs150028621	43924749	A	0	0.003333	G	0.5777	0
22	rs200062058	45255643	A	0	0.003333	G	0.5777	0
22	rs71766169	45255714	A	0	0.003333	G	0.5777	0
22	rs151269819	50596612	A	0	0.003333	G	0.5777	0

22	rs141762500	50721495	A	0	0.003333	G	0.5777	0
1	rs190578331	9795602	A	0	0.003333	G	0.5778	0
1	rs1568103	244091316	G	0.3222	0.3383	A	0.5778	0.9295
3	rs11129280	27326097	A	0.1186	0.1085	C	0.5778	1.105
3	rs55691228	159713243	A	0	0.003333	G	0.5778	0
4	rs4220	155491759	A	0.1701	0.1575	G	0.5778	1.096
10	rs180837482	97787021	C	0	0.003333	A	0.5778	0
11	rs2239734	7022360	C	0.1701	0.1575	A	0.5778	1.096
11	rs11225434	102691482	G	0.3222	0.3383	A	0.5778	0.9295
11	rs202027996	120175748	A	0	0.003333	G	0.5778	0
12	rs117492949	3747472	A	0	0.003333	G	0.5778	0
12	rs201087250	42839890	A	0	0.003339	G	0.5778	0
4	rs1395479	178318191	A	0.2165	0.2308	C	0.5779	0.9207
7	rs1722232	101288516	A	0.3454	0.33	G	0.5779	1.071
15	rs8043440	26973086	G	0.3454	0.33	A	0.5779	1.071
1	rs1184626	77093180	A	0.2397	0.225	C	0.578	1.086
1	rs3738401	231830295	A	0.2397	0.225	G	0.578	1.086
7	rs55745506	7495735	G	0.2397	0.225	A	0.578	1.086
8	rs2272754	144522387	A	0.2397	0.225	C	0.578	1.086
11	rs201894536	823742	A	0	0.003333	G	0.578	0
11	rs1045861	89935586	C	0.25	0.2657	A	0.5781	0.9214
6	rs200220211	33643512	G	0	0.003333	A	0.5782	0
4	rs11721627	15127630	A	0.3247	0.3417	G	0.5784	0.9266
9	rs911491	4242464	G	0.3247	0.3417	A	0.5784	0.9266
17	rs1292053	57963537	A	0.3247	0.3417	G	0.5784	0.9266
6	rs9296669	51890823	A	0.3247	0.3408	G	0.5786	0.9301
16	rs2287790	27789278	A	0.1031	0.115	G	0.5788	0.8846
4	rs13119659	76720885	A	0.06701	0.07667	G	0.5789	0.865
5	rs17577085	141863604	C	0.06701	0.07667	A	0.5789	0.865
12	rs12818575	52984620	A	0.06701	0.07667	G	0.5789	0.865
21	rs35531957	31538692	A	0.1031	0.1142	G	0.5789	0.8919
22	rs139553	42187199	A	0.06701	0.07667	G	0.5789	0.865
22	rs11541159	43089849	G	0.1701	0.1583	A	0.579	1.09
2	rs4852831	72236597	A	0.3273	0.3442	G	0.5791	0.9272
4	rs62333891	169606649	A	0.06701	0.0775	C	0.5792	0.8549
13	rs17066954	46170621	A	0.3505	0.335	T	0.5792	1.071
15	rs12593255	56208534	A	0.06701	0.0775	G	0.5792	0.8549
15	rs3825859	74002006	A	0.06701	0.0775	G	0.5792	0.8549
21	rs2277809	45751822	A	0.06701	0.0775	G	0.5792	0.8549
4	rs1565908	84347423	G	0.3273	0.3433	A	0.5793	0.9307
11	rs659777	107641505	C	0.3273	0.3433	A	0.5793	0.9307
16	rs3743930	3304626	G	0.2191	0.2342	C	0.5793	0.9175
16	rs12925771	81199544	G	0.3273	0.3433	A	0.5793	0.9307

16	rs12597040	81199555	A	0.3273	0.3433	G	0.5793	0.9307
22	rs5762809	29196494	A	0.1521	0.1653	G	0.5794	0.9057
1	rs79973962	158669782	A	0.1211	0.11	G	0.5795	1.115
1	rs2266782	171076966	A	0.1521	0.1658	G	0.5795	0.9021
1	rs708775	226923264	A	0.2423	0.2275	G	0.5795	1.086
4	rs2010907	189540683	A	0.1521	0.165	G	0.5795	0.9075
8	rs4289770	10487222	A	0.1521	0.1658	G	0.5795	0.9021
11	rs11041372	7441737	G	0.1211	0.11	A	0.5795	1.115
11	rs10770136	10715124	A	0.1521	0.1658	G	0.5795	0.9021
12	rs11833905	92214658	A	0.2423	0.2275	C	0.5795	1.086
15	rs2277603	74622533	G	0.2423	0.2275	A	0.5795	1.086
19	rs2075090	20117685	A	0.1521	0.165	G	0.5795	0.9075
20	rs4141463	14747471	G	0.1211	0.11	A	0.5795	1.115
6	rs1049059	32634313	C	0.1521	0.1661	G	0.5796	0.9003
1	rs10903129	25768937	G	0.3531	0.3375	A	0.5798	1.071
1	rs12027135	25775733	A	0.3531	0.3375	T	0.5798	1.071
1	rs74718983	53535477	A	0.01289	0.01	G	0.5798	1.292
1	rs141604312	154962945	G	0.01289	0.01	A	0.5798	1.292
1	rs145168896	159505762	A	0.01289	0.01	C	0.5798	1.292
2	rs188572845	61149108	A	0.01289	0.01	G	0.5798	1.292
2	rs13404469	68765205	A	0.01289	0.01	G	0.5798	1.292
2	rs143467854	74466475	G	0.01289	0.01	A	0.5798	1.292
2	rs1427593	137555224	A	0.01289	0.01	G	0.5798	1.292
2	rs141748745	166003318	C	0.01289	0.01	A	0.5798	1.292
2	rs148697217	197537115	C	0.01289	0.01	A	0.5798	1.292
2	rs17275498	231420401	C	0.01289	0.01	A	0.5798	1.292
3	rs139324337	130345381	A	0.01289	0.01	G	0.5798	1.292
4	rs148840944	48583537	A	0.01289	0.01	G	0.5798	1.292
4	rs148334494	152048801	A	0.01289	0.01	G	0.5798	1.292
5	rs140416835	637668	G	0.01289	0.01	A	0.5798	1.292
5	rs150376362	637673	A	0.01289	0.01	G	0.5798	1.292
5	rs2232376	1880891	A	0.01289	0.01	G	0.5798	1.292
5	rs141427696	32090312	G	0.01289	0.01	A	0.5798	1.292
5	rs17857089	140563203	A	0.01289	0.01	G	0.5798	1.292
6	rs79512125	36442659	A	0.01289	0.01	G	0.5798	1.292
6	rs149112329	43098060	A	0.01289	0.01	G	0.5798	1.292
6	rs586024	46834685	G	0.01289	0.01	A	0.5798	1.292
6	rs61748896	147038089	A	0.01289	0.01	C	0.5798	1.292
7	rs72655982	21609691	A	0.01289	0.01	G	0.5798	1.292
7	rs72655988	21621631	A	0.01289	0.01	G	0.5798	1.292
7	rs76244589	43664290	G	0.01289	0.01	A	0.5798	1.292
7	rs142005939	45717651	A	0.01289	0.01	G	0.5798	1.292
8	rs16916632	87680282	A	0.01289	0.01	G	0.5798	1.292

9	rs138655830	101787280	A	0.01289	0.01	C	0.5798	1.292
9	rs150803564	116930998	A	0.01289	0.01	G	0.5798	1.292
9	rs6478854	131588888	G	0.3299	0.3467	C	0.5798	0.9278
10	rs138695721	5691034	C	0.01289	0.01	A	0.5798	1.292
11	rs148640037	1031856	A	0.01289	0.01	G	0.5798	1.292
11	rs61736238	119215586	A	0.01289	0.01	G	0.5798	1.292
11	rs79770470	124293857	C	0.01289	0.01	A	0.5798	1.292
15	rs4246302	100687967	G	0.3299	0.3467	A	0.5798	0.9278
16	rs138012549	30006914	G	0.01289	0.01	A	0.5798	1.292
16	rs76070113	68112812	C	0.01289	0.01	G	0.5798	1.292
17	rs79220301	7352012	A	0.01289	0.01	G	0.5798	1.292
18	rs1805082	21120444	A	0.3299	0.3467	G	0.5798	0.9278
18	rs200647674	56936667	A	0.01289	0.01	C	0.5798	1.292
18	rs116195130	77703341	A	0.01289	0.01	G	0.5798	1.292
19	rs41276898	14269279	C	0.01289	0.01	A	0.5798	1.292
19	rs201971377	39589715	A	0.01289	0.01	C	0.5798	1.292
21	rs80166001	47768933	G	0.01289	0.01	A	0.5798	1.292
22	rs146242251	41527628	G	0.01289	0.01	A	0.5798	1.292
2	rs4663990	234862040	A	0.3557	0.3392	G	0.5799	1.076
1	rs7542665	62673037	G	0.2423	0.2283	A	0.5801	1.081
1	rs10779617	214668462	G	0.3299	0.3458	A	0.5801	0.9312
1	rs35539373	223813586	C	0.3299	0.3458	A	0.5801	0.9312
5	rs4705952	131839618	A	0.3299	0.3456	G	0.5801	0.9323
9	rs7861943	4836811	G	0.3299	0.3458	A	0.5801	0.9312
12	rs2358944	66117558	A	0.3299	0.3458	G	0.5801	0.9312
11	rs12272419	28110151	G	0.3557	0.34	A	0.5804	1.072
10	rs2901235	122152784	A	0.3582	0.3417	G	0.5806	1.076
1	rs541728	90992380	G	0.2216	0.2367	A	0.5807	0.9185
2	rs10497191	158667217	A	0.08247	0.07333	G	0.5807	1.136
4	rs3814422	184203886	G	0.2216	0.2367	C	0.5807	0.9185
9	rs4740164	134386781	A	0.08247	0.07333	G	0.5807	1.136
12	rs830124	122395777	A	0.2216	0.2367	G	0.5807	0.9185
19	rs77370625	57839506	G	0.08247	0.07333	C	0.5807	1.136
17	rs12452947	17193215	G	0.3325	0.3483	A	0.5808	0.9318
22	rs5761711	27129747	A	0.3325	0.3483	G	0.5808	0.9318
2	rs143730934	209345862	G	0.02062	0.02667	A	0.5809	0.7684
3	rs17855824	93779860	A	0.08247	0.07346	G	0.5809	1.134
3	rs17348572	140178381	G	0.02062	0.02667	A	0.5809	0.7684
5	rs3756672	13719018	A	0.02062	0.02667	G	0.5809	0.7684
6	rs74607434	41557567	A	0.02062	0.02667	G	0.5809	0.7684
11	rs139640491	6630007	C	0.02062	0.02667	A	0.5809	0.7684
11	rs11041525	7694013	C	0.02062	0.02667	A	0.5809	0.7684
11	rs3092856	108159732	A	0.02062	0.02667	G	0.5809	0.7684

16	rs7198494	81095091	G	0.02062	0.02667	A	0.5809	0.7684
3	rs79825658	57494263	A	0.1211	0.1108	C	0.581	1.106
3	rs645040	135926622	C	0.1211	0.1108	A	0.581	1.106
4	rs2008242	5221538	C	0.3582	0.3429	G	0.581	1.07
6	rs3923809	38440970	A	0.3582	0.3425	G	0.581	1.072
16	rs61744399	67037054	C	0.2216	0.2358	A	0.581	0.9227
21	rs10854485	48063476	G	0.1211	0.1108	C	0.581	1.106
2	rs1430243	222183997	A	0.3608	0.3442	G	0.5812	1.076
5	rs4467698	151270122	A	0.3351	0.3517	G	0.5812	0.9289
7	rs3847059	100389638	A	0.05155	0.045	G	0.5812	1.153
19	rs17856650	6187800	A	0.05155	0.045	G	0.5812	1.153
19	rs17856651	6187805	G	0.05155	0.045	C	0.5812	1.153
19	rs35002951	50312801	A	0.05155	0.045	G	0.5812	1.153
1	rs35359362	208073205	A	0.02062	0.0275	C	0.5813	0.7445
5	rs17263496	13737444	A	0.02062	0.0275	G	0.5813	0.7445
8	rs143646514	2088717	A	0.02062	0.0275	C	0.5813	0.7445
8	rs117478253	2089096	G	0.02062	0.0275	A	0.5813	0.7445
10	rs1503256	111222113	G	0.02062	0.0275	A	0.5813	0.7445
11	rs142956576	33054958	G	0.02062	0.0275	A	0.5813	0.7445
13	rs145797698	20233141	A	0.02062	0.0275	G	0.5813	0.7445
14	rs17108533	71249802	A	0.02062	0.0275	G	0.5813	0.7445
15	rs16969968	78882925	A	0.02062	0.0275	G	0.5813	0.7445
16	rs138147609	28603710	A	0.02062	0.0275	C	0.5813	0.7445
20	rs2250860	44184375	G	0.02062	0.0275	A	0.5813	0.7445
3	rs1708504	164182926	G	0.3351	0.3508	A	0.5815	0.9324
6	rs119408	5159064	C	0.3608	0.345	A	0.5816	1.072
16	rs7193343	73029160	G	0.3608	0.345	A	0.5816	1.072
21	rs2269187	37761364	A	0.3376	0.3542	G	0.5818	0.9295
6	rs2844496	31480398	A	0.1546	0.1675	G	0.5819	0.9092
6	rs2516399	31481299	G	0.1546	0.1675	A	0.5819	0.9092
6	rs1853665	150298842	A	0.3634	0.3467	G	0.5819	1.076
7	rs1486139	46275728	A	0.3634	0.3467	G	0.5819	1.076
9	rs2297603	101778265	A	0.1546	0.1683	C	0.5819	0.9038
12	rs2071072	7015075	C	0.1546	0.1675	A	0.5819	0.9092
6	rs11531	31750509	A	0.05155	0.04515	G	0.582	1.149
2	rs1420757	83146534	C	0.2242	0.2392	A	0.5822	0.9195
2	rs2679722	86135154	A	0.3376	0.3533	G	0.5822	0.9329
2	rs12992066	95947085	A	0.3376	0.3533	G	0.5822	0.9329
5	rs2548003	28747323	C	0.2242	0.2392	G	0.5822	0.9195
7	rs12536936	3720117	G	0.3634	0.3475	A	0.5822	1.072
21	rs10775648	33371123	A	0.3634	0.3475	G	0.5822	1.072
7	rs2240089	51096036	C	0.2474	0.2325	G	0.5823	1.085
3	rs17272796	17077268	G	0.3402	0.3567	A	0.5825	0.9301

3	rs7648640	64332101	G	0.1753	0.1625	A	0.5825	1.095
5	rs17084873	94861118	G	0.1753	0.1625	C	0.5825	1.095
6	rs9379084	7231843	A	0.1753	0.1625	G	0.5825	1.095
6	rs757257	30142458	A	0.366	0.3492	G	0.5825	1.076
6	rs12198275	123395743	A	0.1057	0.1175	G	0.5825	0.8874
14	rs2074930	74428000	A	0.366	0.3492	T	0.5825	1.076
17	rs11078672	7215142	A	0.1057	0.1175	G	0.5825	0.8874
1	rs4420065	66161461	G	0.1057	0.1167	A	0.5827	0.8946
4	rs72731014	155410650	G	0.1057	0.1167	A	0.5827	0.8946
4	rs55810732	155410667	A	0.1057	0.1167	G	0.5827	0.8946
4	rs17373874	155411939	C	0.1057	0.1167	A	0.5827	0.8946
4	rs13149269	155412464	A	0.1057	0.1167	G	0.5827	0.8946
9	rs3747539	37692654	C	0.1057	0.1167	A	0.5827	0.8946
11	rs2568076	8948615	A	0.1057	0.1167	G	0.5827	0.8946
1	rs2274570	100376448	G	0.366	0.35	A	0.5828	1.072
5	rs12186491	147593497	C	0.3402	0.3558	A	0.5828	0.9334
14	rs8015016	96122408	G	0.2474	0.2333	A	0.5829	1.08
5	rs12523625	127294777	A	0.3428	0.3598	G	0.583	0.9282
4	rs6852374	61370159	A	0.3428	0.3592	G	0.5831	0.9306
6	rs6906499	17665479	G	0.3428	0.3592	C	0.5831	0.9306
6	rs12199222	17699322	A	0.3428	0.36	C	0.5831	0.9272
19	rs12978469	4210356	G	0.3428	0.3592	A	0.5831	0.9306
6	rs1165165	25862466	A	0.1237	0.1125	G	0.5832	1.114
12	rs11111245	79139541	G	0.08247	0.07417	A	0.5832	1.122
18	rs12457323	21424986	G	0.08247	0.07417	A	0.5832	1.122
6	rs9466056	21384613	A	0.3686	0.3525	G	0.5834	1.072
5	rs17084927	94939193	G	0.1753	0.1633	A	0.5835	1.089
6	rs1057141	32818774	G	0.1753	0.1633	A	0.5835	1.089
16	rs9941349	53825488	A	0.1753	0.1633	G	0.5835	1.089
18	rs1805034	60027241	G	0.366	0.3508	A	0.5835	1.068
4	rs1039808	126239986	A	0.2268	0.2417	G	0.5836	0.9205
9	rs1409955	19790795	A	0.2268	0.2417	G	0.5836	0.9205
3	rs6438804	123331143	A	0.3454	0.3625	G	0.5837	0.9278
4	rs13146272	187120211	A	0.3454	0.3625	C	0.5837	0.9278
13	rs144509002	109475602	G	0.04124	0.04925	A	0.5837	0.8303
17	rs4796561	6715722	G	0.3686	0.3531	A	0.5837	1.069
3	rs33990195	113320477	A	0.04124	0.04917	G	0.5838	0.8318
3	rs11546878	183976103	A	0.04124	0.04917	G	0.5838	0.8318
6	rs2228375	17675246	G	0.04124	0.04917	A	0.5838	0.8318
6	rs4713340	30599702	A	0.04124	0.04917	G	0.5838	0.8318
6	rs2855437	33138955	G	0.04124	0.04917	A	0.5838	0.8318
1	rs2199074	234166129	G	0.2474	0.2342	A	0.5839	1.075
11	rs11246212	610277	A	0.2474	0.2342	G	0.5839	1.075

14	rs915071	32433858	G	0.1572	0.1708	A	0.5842	0.9054
14	rs17104630	37000049	G	0.1572	0.1708	A	0.5842	0.9054
16	rs75331747	29899123	A	0.25	0.2358	C	0.5842	1.08
3	rs9845816	14551444	A	0.1572	0.17	G	0.5843	0.9108
3	exm-IND3-11324	111766749	D	0.04124	0.05	I	0.5843	0.8172
4	rs4317244	186320906	G	0.04124	0.05	C	0.5843	0.8172
6	rs6925614	168709385	G	0.3737	0.3573	A	0.5843	1.074
10	rs10748861	105927104	A	0.1572	0.17	G	0.5843	0.9108
20	rs2273526	18523002	G	0.1572	0.17	C	0.5843	0.9108
10	rs7915707	49810977	C	0.3479	0.3642	A	0.5844	0.9317
22	rs743749	37398195	G	0.3479	0.3642	A	0.5844	0.9317
5	rs6870158	16537473	C	0.3711	0.3558	A	0.5845	1.068
10	rs7918487	23482635	A	0.1237	0.1133	G	0.5845	1.104
10	rs721917	81706324	A	0.3737	0.3575	G	0.5845	1.072
15	rs12906938	90191355	G	0.3711	0.3558	A	0.5845	1.068
1	rs2271458	211534056	C	0.03093	0.025	A	0.5847	1.245
6	rs144581330	159210399	G	0.03093	0.025	A	0.5847	1.245
10	rs117042762	121286832	A	0.03093	0.025	G	0.5847	1.245
11	rs73411617	8159843	A	0.03093	0.025	G	0.5847	1.245
11	rs17824933	60760612	C	0.03093	0.025	G	0.5847	1.245
11	rs12575909	111680496	C	0.03093	0.025	A	0.5847	1.245
19	rs7256147	6921868	A	0.1778	0.165	G	0.5847	1.095
2	rs7589485	28645120	C	0.3505	0.3675	A	0.5848	0.9288
2	rs980545	75460060	C	0.06959	0.07917	A	0.5848	0.87
3	rs6779230	173488137	A	0.3505	0.3675	C	0.5848	0.9288
1	rs2236359	227935444	G	0.3505	0.3667	A	0.585	0.9322
2	rs14234	70524142	A	0.3763	0.36	G	0.585	1.073
4	rs75060923	4322570	A	0.3763	0.36	G	0.585	1.073
10	rs3849969	75525999	G	0.2294	0.2442	A	0.585	0.9214
15	rs1994016	79080234	A	0.06959	0.08	G	0.585	0.8601
17	rs3744741	649232	A	0.2294	0.245	G	0.585	0.9173
17	rs11867457	29111368	G	0.06959	0.08	A	0.585	0.8601
17	rs4523977	39883350	A	0.2294	0.2442	G	0.585	0.9214
17	rs78265875	4449228	A	0.06959	0.08013	G	0.5851	0.8586
1	rs1332816	79591801	G	0.3789	0.3625	A	0.5856	1.073
3	rs1109643	39139776	A	0.1778	0.1658	G	0.5857	1.088
7	rs1130329	5112554	C	0.3557	0.3725	A	0.5859	0.9299
5	rs2303007	179740827	G	0.3789	0.3633	A	0.586	1.069
6	rs4946846	107990428	A	0.1082	0.1196	C	0.586	0.8939
13	rs9568968	54592856	G	0.3789	0.3633	A	0.586	1.069
17	rs2707038	77137918	G	0.3789	0.3633	A	0.586	1.069
9	rs7027958	90218347	G	0.3557	0.3717	A	0.5861	0.9332
16	rs9931465	88499539	C	0.1082	0.12	G	0.5861	0.8902

1	rs112752591	32279890	G	0.007732	0.01254	A	0.5863	0.6135
5	rs331558	94478881	A	0.3814	0.3656	G	0.5863	1.07
5	rs11167743	140567608	A	0.3582	0.3752	G	0.5863	0.9296
8	rs200577841	145057916	A	0.007732	0.01256	C	0.5863	0.6125
10	rs74145660	73544093	G	0.1082	0.1192	C	0.5863	0.8972
11	rs7117260	4869906	C	0.08505	0.07583	A	0.5863	1.133
17	rs79813370	56693677	A	0.08505	0.07583	C	0.5863	1.133
19	rs143873873	18271764	A	0.007732	0.01254	G	0.5863	0.6135
1	rs149332265	2289030	A	0.007732	0.0125	G	0.5864	0.6156
1	rs112651926	32279871	G	0.007732	0.0125	A	0.5864	0.6156
1	rs112754928	32280724	A	0.007732	0.0125	G	0.5864	0.6156
1	rs117385606	35250943	A	0.007732	0.0125	G	0.5864	0.6156
1	rs190476675	86920912	A	0.007732	0.0125	G	0.5864	0.6156
1	rs12021720	100672060	A	0.007732	0.0125	G	0.5864	0.6156
1	rs77912414	145541806	G	0.007732	0.0125	A	0.5864	0.6156
1	rs34249690	159802845	A	0.007732	0.0125	G	0.5864	0.6156
2	rs6714546	33361425	A	0.007732	0.0125	G	0.5864	0.6156
2	rs189000383	64808387	A	0.007732	0.0125	G	0.5864	0.6156
2	rs3749131	69053166	G	0.007732	0.0125	A	0.5864	0.6156
2	rs79840632	201756740	A	0.007732	0.0125	G	0.5864	0.6156
3	rs3921595	161221705	C	0.232	0.2467	A	0.5864	0.9224
4	rs75299065	119754766	G	0.007732	0.0125	A	0.5864	0.6156
4	rs2070951	149358014	C	0.232	0.2467	G	0.5864	0.9224
5	rs188983008	112849608	T	0.007732	0.0125	A	0.5864	0.6156
7	rs117477399	20682989	A	0.007732	0.0125	G	0.5864	0.6156
7	rs180834558	128497224	A	0.007732	0.01252	C	0.5864	0.6145
8	rs1592624	113241088	A	0.232	0.2467	C	0.5864	0.9224
8	rs35971959	145736086	A	0.007732	0.0125	C	0.5864	0.6156
8	rs200070447	146278473	A	0.007732	0.0125	G	0.5864	0.6156
9	rs138788156	5920283	A	0.007732	0.0125	G	0.5864	0.6156
9	rs150198392	139350125	A	0.007732	0.0125	G	0.5864	0.6156
10	rs147259181	115529558	G	0.007732	0.0125	C	0.5864	0.6156
10	rs4962416	126696872	G	0.007732	0.0125	A	0.5864	0.6156
11	rs140994058	8938895	C	0.007732	0.01252	G	0.5864	0.6145
11	rs115125023	11362458	A	0.007732	0.0125	G	0.5864	0.6156
11	rs201642255	17035678	A	0.007732	0.0125	G	0.5864	0.6156
11	rs115991011	65651040	A	0.007732	0.0125	C	0.5864	0.6156
11	rs188530929	107299811	A	0.007732	0.0125	G	0.5864	0.6156
11	rs147554220	117352857	A	0.007732	0.0125	G	0.5864	0.6156
11	rs138250193	118461172	A	0.007732	0.0125	G	0.5864	0.6156
12	rs202132447	7283255	T	0.007732	0.0125	A	0.5864	0.6156
12	rs79083300	56532718	G	0.007732	0.0125	C	0.5864	0.6156
14	rs751837	103484825	G	0.007732	0.0125	A	0.5864	0.6156

15	rs149721349	44048870	A	0.007732	0.0125	G	0.5864	0.6156
15	rs144836310	59911556	G	0.007732	0.0125	C	0.5864	0.6156
15	rs34874770	101606215	A	0.007732	0.01252	G	0.5864	0.6145
16	rs117887370	55905536	A	0.007732	0.0125	G	0.5864	0.6156
19	rs192103497	40433893	G	0.007732	0.0125	C	0.5864	0.6156
19	rs16976937	44470147	A	0.007732	0.0125	G	0.5864	0.6156
19	rs139528439	45153329	A	0.007732	0.0125	G	0.5864	0.6156
20	rs114151707	60736519	A	0.007732	0.0125	G	0.5864	0.6156
21	rs147211854	40570769	A	0.007732	0.0125	T	0.5864	0.6156
1	rs6695567	53629585	G	0.3582	0.375	A	0.5865	0.9304
1	exm2252724	67327894	G	0.05412	0.04667	A	0.5865	1.169
1	rs34182923	67327894	G	0.05412	0.04667	A	0.5865	1.169
1	rs6693882	96145968	G	0.1598	0.1733	A	0.5865	0.907
1	rs2816949	199997778	G	0.05412	0.04667	A	0.5865	1.169
4	rs4331786	38769408	G	0.3814	0.3658	A	0.5865	1.069
4	rs3792615	164532801	G	0.1598	0.1733	A	0.5865	0.907
5	rs77793555	34850389	A	0.05412	0.04667	G	0.5865	1.169
6	rs9472155	43897727	A	0.1598	0.1733	G	0.5865	0.907
8	rs7000642	64428514	A	0.3582	0.375	G	0.5865	0.9304
10	rs11553577	97453464	C	0.05412	0.04667	A	0.5865	1.169
11	rs7110845	120175749	A	0.05412	0.04667	G	0.5865	1.169
16	rs152451	23646191	G	0.1598	0.1733	A	0.5865	0.907
19	rs34186470	46914921	A	0.3814	0.3658	G	0.5865	1.069
20	rs967417	6620893	G	0.1598	0.1733	A	0.5865	0.907
1	rs7553864	87613358	A	0.1598	0.1725	G	0.5866	0.9123
2	rs3811047	113671410	A	0.1598	0.1725	G	0.5866	0.9123
3	rs17238798	42799765	A	0.1598	0.1725	G	0.5866	0.9123
5	rs17101107	143225966	A	0.3582	0.3742	G	0.5867	0.9337
1	rs848210	16259813	G	0.384	0.3683	A	0.587	1.069
2	rs10469562	144463315	G	0.384	0.3683	A	0.587	1.069
5	rs2546423	180582604	A	0.3608	0.3775	G	0.587	0.9309
11	exm2250129	64496357	A	0.3608	0.3775	C	0.587	0.9309
11	rs2230414	64496357	A	0.3608	0.3775	C	0.587	0.9309
11	rs531184	117112526	C	0.3866	0.3698	A	0.587	1.074
5	rs161704	55206444	A	0.3866	0.37	G	0.5871	1.073
6	rs9380345	33080359	A	0.3866	0.3706	G	0.5873	1.07
6	rs339331	117210052	G	0.3608	0.3767	A	0.5873	0.9342
7	rs10215180	57238425	G	0.3608	0.3765	A	0.5873	0.935
6	rs9380343	33079166	A	0.3866	0.3708	G	0.5875	1.069
17	rs11078078	12471886	A	0.3634	0.3806	G	0.5875	0.9289
2	rs744426	179436020	A	0.3634	0.38	G	0.5876	0.9314
4	rs7690296	95561459	G	0.3634	0.38	A	0.5876	0.9314
15	rs969348	81847279	G	0.3892	0.3725	A	0.5876	1.073

17	rs1043149	74077797	A	0.3892	0.3725	G	0.5876	1.073
18	rs507163	33907628	G	0.3892	0.3725	A	0.5876	1.073
19	rs72480733	40196627	A	0.3892	0.3725	G	0.5876	1.073
20	rs127430	57164855	A	0.3634	0.38	G	0.5876	0.9314
5	rs10063647	127371588	A	0.2345	0.2492	G	0.5877	0.9233
5	rs1432817	155253756	A	0.3892	0.3731	C	0.5877	1.07
11	rs3812762	8751640	C	0.2345	0.2492	G	0.5877	0.9233
5	rs4530754	122855416	A	0.3634	0.3792	G	0.5878	0.9347
16	rs885479	89986154	G	0.2345	0.2487	A	0.5878	0.9254
17	rs4968415	60264240	C	0.3634	0.3792	A	0.5878	0.9347
3	rs750422	10595564	A	0.266	0.2517	G	0.5879	1.077
4	rs6840093	66366103	G	0.3892	0.3733	A	0.5879	1.069
14	rs468167	59068964	G	0.1804	0.1683	A	0.5879	1.088
2	rs4853018	74466594	A	0.1263	0.1158	G	0.588	1.103
8	rs9792269	129264589	G	0.1263	0.1158	A	0.588	1.103
14	rs6572635	50472405	A	0.1263	0.1158	G	0.588	1.103
19	rs12609976	55587822	A	0.1263	0.1158	G	0.588	1.103
7	rs2040366	142196434	A	0.366	0.3825	G	0.5881	0.9319
10	rs12413409	104719096	A	0.2577	0.2433	G	0.5881	1.08
1	rs11586833	156347834	T	0.3918	0.3758	A	0.5884	1.07
1	rs1335532	117100957	A	0.3943	0.3775	G	0.5885	1.074
1	rs3813954	207134226	A	0.08505	0.07667	G	0.5886	1.12
1	rs3813953	207134410	C	0.08505	0.07667	A	0.5886	1.12
2	rs2166898	121612659	A	0.08505	0.07667	G	0.5886	1.12
2	rs3749003	168107351	G	0.08505	0.07667	A	0.5886	1.12
6	rs2275215	129861392	G	0.3686	0.385	A	0.5886	0.9324
6	rs17075385	146126397	G	0.08505	0.07667	C	0.5886	1.12
1	rs3737983	53716416	G	0.2577	0.2442	A	0.5889	1.075
1	rs683968	218333601	G	0.3686	0.3842	A	0.5889	0.9357
3	rs1042787	134898742	G	0.2577	0.2442	A	0.5889	1.075
5	rs2918284	148588846	G	0.3686	0.3842	A	0.5889	0.9357
7	rs74974199	100686491	C	0.1624	0.175	G	0.5889	0.9138
10	rs11191548	104846178	G	0.2577	0.2442	A	0.5889	1.075
15	rs551225	54003091	A	0.2577	0.2442	G	0.5889	1.075
5	rs192167	133439635	G	0.2371	0.2517	A	0.589	0.9242
6	rs2857130	32776414	A	0.2617	0.2467	T	0.589	1.082
2	rs6437353	241558397	G	0.3711	0.3875	A	0.5891	0.9328
11	rs2468623	23079457	G	0.3711	0.3875	A	0.5891	0.9328
6	rs7166	4116262	A	0.3711	0.3867	G	0.5894	0.9361
6	rs643232	4122249	A	0.3711	0.3867	C	0.5894	0.9361
11	rs12296050	2489342	A	0.3737	0.3908	G	0.5895	0.93
16	rs6565110	83102477	A	0.3995	0.3831	C	0.5895	1.071
4	rs4082504	106448900	C	0.3995	0.3833	A	0.5897	1.07

6	rs415890	167406633	G	0.3995	0.3833	C	0.5897	1.07
7	rs292592	134889141	G	0.3763	0.3933	A	0.5899	0.9305
14	rs1957309	60312064	A	0.3763	0.3933	G	0.5899	0.9305
3	rs9810311	31481326	G	0.4021	0.3858	A	0.5901	1.07
6	rs2857150	32763821	A	0.2603	0.2467	G	0.5901	1.075
6	rs2295333	43968814	G	0.4021	0.3858	A	0.5901	1.07
8	rs3753115	17930772	A	0.4021	0.3858	G	0.5901	1.07
8	rs13266634	118184783	A	0.4021	0.3858	G	0.5901	1.07
10	rs4748636	5782022	A	0.1108	0.1233	C	0.5901	0.8859
14	rs4140998	72895359	G	0.1108	0.1233	A	0.5901	0.8859
17	rs2290505	18137141	A	0.2603	0.2467	G	0.5901	1.075
4	rs2286455	16020162	A	0.2397	0.255	G	0.5902	0.921
5	rs7727588	171468278	G	0.05412	0.0475	A	0.5903	1.147
8	rs28366046	19171316	A	0.05412	0.0475	G	0.5903	1.147
10	rs41291604	97919011	G	0.05412	0.0475	A	0.5903	1.147
10	rs55812591	134021576	A	0.05412	0.0475	G	0.5903	1.147
20	rs75122556	2384236	A	0.007812	0.0134	G	0.5903	0.5797
20	rs143118786	25457690	A	0.05412	0.0475	G	0.5903	1.147
2	rs935172	26804247	A	0.3789	0.3958	G	0.5904	0.931
2	rs2234671	219029108	C	0.08549	0.0775	G	0.5904	1.113
4	rs6449134	15380517	A	0.07216	0.08167	C	0.5904	0.8746
4	rs78397848	152069090	G	0.07216	0.08167	A	0.5904	0.8746
6	rs2394450	30817501	A	0.3789	0.3958	G	0.5904	0.931
6	rs14398	33254665	G	0.07216	0.08167	A	0.5904	0.8746
9	rs76922475	135285840	A	0.07216	0.08167	G	0.5904	0.8746
19	rs148299276	815254	A	0.07216	0.08167	G	0.5904	0.8746
19	rs55742440	35524824	G	0.2629	0.2483	A	0.5905	1.08
2	rs2044693	68385097	A	0.4021	0.3867	G	0.5906	1.067
15	rs4886707	75755467	A	0.4021	0.3867	G	0.5906	1.067
1	rs201172117	36786568	A	0.007732	0.01333	G	0.5907	0.5766
1	rs115445569	55085608	A	0.007732	0.01333	G	0.5907	0.5766
1	rs145762716	92737184	C	0.007732	0.01333	G	0.5907	0.5766
1	rs145312899	156526467	A	0.007732	0.01333	G	0.5907	0.5766
1	rs117790439	182550360	A	0.007732	0.01333	G	0.5907	0.5766
2	rs144676974	21001160	A	0.007732	0.01333	G	0.5907	0.5766
2	rs3749130	69034515	A	0.007732	0.01333	G	0.5907	0.5766
2	rs138190910	223553186	A	0.007732	0.01333	C	0.5907	0.5766
3	rs184153168	10050103	A	0.007732	0.01333	G	0.5907	0.5766
3	rs34936017	10081411	G	0.007732	0.01333	A	0.5907	0.5766
3	rs185125160	52727467	A	0.007732	0.01333	C	0.5907	0.5766
3	rs142027515	148876472	A	0.007732	0.01333	G	0.5907	0.5766
3	rs145159791	192981010	A	0.007732	0.01333	G	0.5907	0.5766
5	rs75028043	42689061	A	0.007732	0.01333	G	0.5907	0.5766

5	rs2233215	52397337	G	0.007732	0.01333	A	0.5907	0.5766
5	rs139319889	71494694	G	0.007732	0.01333	A	0.5907	0.5766
5	rs76657982	129072868	A	0.007732	0.01333	G	0.5907	0.5766
5	rs10037485	156770133	A	0.007732	0.01333	G	0.5907	0.5766
5	rs2270898	169508893	A	0.007732	0.01333	T	0.5907	0.5766
6	rs9477599	18055908	G	0.007732	0.01333	A	0.5907	0.5766
6	rs139446902	38917235	A	0.007732	0.01333	G	0.5907	0.5766
6	rs190759	50987768	A	0.007732	0.01333	G	0.5907	0.5766
6	rs117850701	90575924	A	0.007732	0.01333	G	0.5907	0.5766
6	rs9388489	126698719	A	0.007732	0.01333	G	0.5907	0.5766
6	rs1361108	126767600	G	0.007732	0.01333	A	0.5907	0.5766
6	rs1490388	126835655	G	0.007732	0.01333	A	0.5907	0.5766
6	rs1490384	126851160	G	0.007732	0.01333	A	0.5907	0.5766
6	rs4549631	126966308	A	0.007732	0.01333	G	0.5907	0.5766
7	rs17864092	126639604	G	0.007732	0.01333	A	0.5907	0.5766
8	rs200741431	20003282	A	0.007732	0.01333	G	0.5907	0.5766
8	rs114216685	95403868	G	0.007732	0.01333	A	0.5907	0.5766
10	rs117111102	96447920	A	0.007732	0.01333	G	0.5907	0.5766
11	rs146600946	55322639	A	0.007732	0.01333	G	0.5907	0.5766
11	rs7941523	124507019	A	0.007732	0.01333	G	0.5907	0.5766
12	rs2271188	56495023	A	0.007732	0.01333	G	0.5907	0.5766
13	rs116615412	20425539	A	0.007732	0.01333	G	0.5907	0.5766
13	rs138532095	113487196	A	0.007732	0.01333	G	0.5907	0.5766
15	rs141981389	65623011	A	0.007732	0.01333	T	0.5907	0.5766
16	rs16956174	82033612	G	0.007732	0.01333	A	0.5907	0.5766
16	rs6564956	82034435	C	0.007732	0.01333	G	0.5907	0.5766
17	rs149278711	1551656	A	0.007732	0.01333	G	0.5907	0.5766
17	rs139116684	39137090	A	0.007732	0.01333	C	0.5907	0.5766
19	rs2242519	36212009	C	0.007732	0.01333	G	0.5907	0.5766
19	rs142913953	52497070	A	0.007732	0.01333	G	0.5907	0.5766
19	rs185737879	53057794	A	0.007732	0.01333	G	0.5907	0.5766
20	rs187224668	210144	A	0.007732	0.01333	C	0.5907	0.5766
20	rs34924243	3682126	A	0.007732	0.01333	G	0.5907	0.5766
20	rs1050475	18296473	A	0.007732	0.01333	G	0.5907	0.5766
20	rs112806196	47271866	A	0.007732	0.01333	G	0.5907	0.5766
22	rs186541010	17583085	A	0.007732	0.01333	C	0.5907	0.5766
22	rs138578557	20103915	A	0.007732	0.01333	G	0.5907	0.5766
22	rs142797829	50960235	A	0.007732	0.01333	G	0.5907	0.5766
5	rs145215828	150945824	G	0.007732	0.01336	A	0.5908	0.5756
17	rs76011501	37296026	A	0.007732	0.01336	G	0.5908	0.5756
1	rs78072249	203054852	A	0.03093	0.02583	G	0.5909	1.204
1	rs7527798	207872290	G	0.03093	0.02583	A	0.5909	1.204
5	rs17078877	178410213	G	0.1649	0.1783	A	0.5909	0.9101

6	rs115653647	32370993	A	0.03093	0.02583	G	0.5909	1.204
7	rs73712043	100680521	A	0.03093	0.02583	G	0.5909	1.204
11	rs144440324	58125740	C	0.03093	0.02583	G	0.5909	1.204
11	rs117109892	65766041	A	0.03093	0.02583	G	0.5909	1.204
12	rs76655496	132416813	A	0.03093	0.02583	G	0.5909	1.204
13	rs74628343	113960845	A	0.03093	0.02583	G	0.5909	1.204
16	rs11150606	31099011	A	0.1649	0.1783	G	0.5909	0.9101
17	rs200753630	79254485	A	0.03093	0.02583	G	0.5909	1.204
18	rs2276211	74580780	A	0.03093	0.02583	G	0.5909	1.204
4	rs35846220	42221342	A	0.3814	0.3975	G	0.591	0.9347
11	rs606458	64546391	G	0.3814	0.3975	A	0.591	0.9347
13	rs9316077	44808665	A	0.4046	0.3892	C	0.591	1.067
12	rs956868	990912	A	0.1649	0.1775	C	0.5911	0.9153
1	rs1033325	233398713	A	0.1856	0.1725	G	0.5912	1.093
3	rs9825372	1498979	G	0.384	0.4008	A	0.5912	0.9319
10	rs2296545	90342837	C	0.384	0.4008	G	0.5912	0.9319
19	rs2302061	2226772	G	0.2629	0.2492	C	0.5912	1.075
2	rs4635554	21389659	C	0.4098	0.3933	A	0.5913	1.071
7	rs741664	132070054	G	0.1289	0.1183	A	0.5913	1.102
12	rs12322164	44413166	A	0.1289	0.1183	G	0.5913	1.102
16	rs6499640	53769677	A	0.1289	0.1183	G	0.5913	1.102
17	rs346822	4937575	A	0.1856	0.1728	G	0.5913	1.091
1	rs522496	21024901	G	0.4072	0.3917	A	0.5914	1.067
9	rs1543527	5762894	A	0.4072	0.3917	G	0.5914	1.067
10	rs1337322	23936020	A	0.384	0.4	G	0.5914	0.9351
10	rs3736582	124742895	G	0.384	0.4	C	0.5914	0.9351
11	rs7122644	6816875	A	0.384	0.4	G	0.5914	0.9351
17	rs12452184	79664426	A	0.4072	0.3917	G	0.5914	1.067
22	rs5751114	41880770	G	0.3866	0.4033	A	0.5916	0.9324
3	rs6773188	51568137	A	0.4098	0.3942	G	0.5917	1.067
9	rs1572983	104133628	G	0.4124	0.3958	A	0.5917	1.071
14	rs10133111	103377321	A	0.4098	0.3942	G	0.5917	1.067
22	rs2228313	42293140	G	0.08763	0.07833	C	0.5917	1.13
2	rs55952639	65299766	G	0.2655	0.2508	A	0.5918	1.079
19	rs773902	17000632	A	0.2655	0.2508	G	0.5918	1.079
2	rs9917151	203033509	A	0.3866	0.4025	G	0.5919	0.9356
5	rs11744729	33176567	A	0.3892	0.4058	G	0.592	0.9328
6	rs762324	30455886	A	0.1856	0.1733	G	0.592	1.087
10	rs1779876	36648040	C	0.1856	0.1733	A	0.592	1.087
4	rs17041860	162912081	A	0.4124	0.3967	G	0.5921	1.067
8	rs61695204	144101037	A	0.4149	0.3983	G	0.5921	1.071
4	rs13112358	178274750	A	0.3892	0.405	G	0.5923	0.936
8	rs1016733	26592410	A	0.3892	0.405	G	0.5923	0.936

9	rs755383	863635	G	0.3892	0.405	A	0.5923	0.936
16	rs4984746	1049139	A	0.3892	0.405	G	0.5923	0.936
4	rs11944688	8116782	A	0.4175	0.4008	G	0.5924	1.071
15	rs809736	61329788	G	0.2655	0.2517	A	0.5924	1.075
13	rs2245300	107721740	A	0.3918	0.4075	G	0.5927	0.9365
8	rs2981133	39921477	A	0.4201	0.4033	G	0.5928	1.072
8	rs4735536	99198040	A	0.4201	0.4033	G	0.5928	1.072
8	rs11558471	118185733	G	0.4201	0.4033	A	0.5928	1.072
11	rs4268468	89886284	A	0.3943	0.4108	G	0.5928	0.9337
21	rs2847231	45735995	G	0.4175	0.4017	A	0.5928	1.068
1	rs35837853	89844035	G	0.268	0.2533	A	0.5929	1.079
1	rs3795605	167095881	G	0.268	0.2533	A	0.5929	1.079
2	rs7577894	56008904	A	0.1134	0.125	G	0.593	0.8953
10	rs3793733	1421326	A	0.1134	0.125	G	0.593	0.8953
13	rs9534264	46541673	T	0.1134	0.125	A	0.593	0.8953
13	rs1926447	46629944	A	0.1134	0.125	G	0.593	0.8953
13	rs3783185	100518580	A	0.1134	0.125	G	0.593	0.8953
1	rs75991575	154918939	T	0.04381	0.05167	A	0.5931	0.8411
2	rs111756480	26534813	G	0.04381	0.05167	A	0.5931	0.8411
2	rs79413730	26534872	A	0.04381	0.05167	G	0.5931	0.8411
3	rs7622799	169098992	A	0.1289	0.1192	G	0.5931	1.093
5	rs2924461	8012069	G	0.4227	0.4058	A	0.5931	1.072
5	rs4546405	167095559	C	0.04381	0.05167	A	0.5931	0.8411
6	rs65685	119136759	C	0.04381	0.05167	A	0.5931	0.8411
7	rs4729260	96117918	C	0.1289	0.1192	G	0.5931	1.093
9	rs4742269	6849317	A	0.1289	0.1192	G	0.5931	1.093
10	rs12762188	833528	A	0.4227	0.4058	C	0.5931	1.072
15	rs6494340	62954948	C	0.04381	0.05167	A	0.5931	0.8411
1	rs1335718	91279915	A	0.3969	0.4133	G	0.5932	0.9341
18	rs11662888	29300708	G	0.3969	0.4133	A	0.5932	0.9341
16	rs201731118	70883694	A	0.1881	0.175	G	0.5933	1.093
4	rs3733227	83582211	G	0.4227	0.4067	C	0.5934	1.068
5	rs6867460	140778256	A	0.1881	0.1753	G	0.5934	1.09
5	rs609412	146225244	A	0.4227	0.4067	G	0.5934	1.068
12	rs2041292	4735968	C	0.1134	0.1258	A	0.5934	0.8886
12	rs75777591	4735970	G	0.1134	0.1258	A	0.5934	0.8886
1	rs3790525	52704330	C	0.04381	0.0525	A	0.5935	0.827
3	rs12489516	148597612	G	0.04381	0.0525	A	0.5935	0.827
5	rs389286	116968077	A	0.04381	0.0525	C	0.5935	0.827
8	rs3213606	17942220	G	0.04381	0.0525	A	0.5935	0.827
10	rs10906189	12675061	A	0.3995	0.4167	G	0.5935	0.9313
10	rs17112457	90512485	G	0.04381	0.0525	A	0.5935	0.827
13	rs1801249	52515354	G	0.3995	0.4167	A	0.5935	0.9313

14	rs10140554	101036074	G	0.3995	0.4167	C	0.5935	0.9313
15	rs77964295	72030194	A	0.04381	0.0525	G	0.5935	0.827
17	rs2289532	78024067	A	0.04381	0.0525	G	0.5935	0.827
18	rs61751994	61233907	A	0.268	0.2542	G	0.5935	1.075
22	rs3761472	44368122	G	0.3995	0.4167	A	0.5935	0.9313
3	rs4686683	185307363	A	0.4253	0.4092	C	0.5937	1.068
5	rs347720	19513668	A	0.4253	0.4092	C	0.5937	1.068
11	rs11038628	5688940	A	0.4253	0.4092	G	0.5937	1.068
1	rs3818822	111857208	A	0.08763	0.07917	G	0.5938	1.117
2	rs57302492	71058230	A	0.08763	0.07917	T	0.5938	1.117
6	rs12527253	89293745	G	0.08763	0.07917	A	0.5938	1.117
17	rs4299203	17878159	A	0.08763	0.07917	C	0.5938	1.117
22	rs76013375	30163526	G	0.08763	0.07917	A	0.5938	1.117
5	rs75451667	87493525	A	0.2474	0.2621	G	0.5939	0.9256
6	rs2295852	35923246	G	0.2474	0.2625	A	0.5939	0.9237
7	rs10246939	141672604	A	0.3995	0.415	G	0.5939	0.9377
7	rs1726866	141672705	A	0.3995	0.415	G	0.5939	0.9377
10	rs12242110	35535695	G	0.2474	0.2625	A	0.5939	0.9237
10	rs1051338	91007360	C	0.2474	0.2625	A	0.5939	0.9237
11	rs2509843	98125404	G	0.4021	0.4183	A	0.5939	0.9349
12	rs7315438	115891403	A	0.4021	0.4183	G	0.5939	0.9349
16	rs3995818	57068106	A	0.4021	0.4183	G	0.5939	0.9349
17	rs9911502	73518203	C	0.4021	0.4183	G	0.5939	0.9349
5	rs2240700	140773388	A	0.1881	0.1758	C	0.594	1.086
2	rs6547014	75853788	A	0.4046	0.4217	G	0.5941	0.9322
13	rs9512752	28150381	A	0.4046	0.4217	G	0.5941	0.9322
14	rs3825584	24587639	A	0.4046	0.4217	G	0.5941	0.9322
15	rs7178424	62380259	A	0.4046	0.4217	G	0.5941	0.9322
1	rs2184953	152280782	A	0.4046	0.4208	G	0.5943	0.9354
4	rs4234972	165445733	C	0.4046	0.4208	A	0.5943	0.9354
6	rs3812204	16698022	G	0.4046	0.4208	A	0.5943	0.9354
8	rs2044895	40523282	G	0.4046	0.4208	A	0.5943	0.9354
16	rs10514604	84446384	C	0.4304	0.4142	G	0.5943	1.069
17	rs8065506	16527795	C	0.4046	0.4208	A	0.5943	0.9354
17	rs12950671	30612520	G	0.4046	0.4208	A	0.5943	0.9354
7	rs1682731	95287743	G	0.4072	0.4242	A	0.5944	0.9326
4	rs1216440	129543559	G	0.4072	0.4233	A	0.5946	0.9358
5	rs4957858	109417383	A	0.4304	0.415	G	0.5948	1.065
5	rs957998	169661994	C	0.4098	0.4267	G	0.5948	0.933
7	rs2072221	21775443	G	0.4304	0.415	A	0.5948	1.065
9	rs10812428	26614847	A	0.4304	0.415	G	0.5948	1.065
15	rs17730281	53907948	A	0.4304	0.415	G	0.5948	1.065
17	rs8080078	28195773	G	0.4098	0.4267	A	0.5948	0.933

7	rs2029623	128507297	G	0.4098	0.4258	A	0.5949	0.9362
11	rs2166706	92691532	G	0.4356	0.4192	A	0.5949	1.069
5	rs3777134	147498019	A	0.433	0.4175	G	0.595	1.065
5	rs10041981	147507420	G	0.433	0.4175	A	0.595	1.065
15	rs8040061	75393720	C	0.433	0.4175	A	0.595	1.065
2	rs921465	200015428	C	0.25	0.265	A	0.5951	0.9245
7	rs3735080	150217309	A	0.1701	0.1833	G	0.5951	0.913
17	rs874889	38857446	C	0.4124	0.4292	A	0.5951	0.9334
6	rs12199003	55196587	A	0.2732	0.2583	G	0.5952	1.079
1	rs4657451	165492327	G	0.1701	0.1842	A	0.5953	0.908
2	rs364891	7137067	A	0.1701	0.1842	G	0.5953	0.908
6	rs3129305	32959180	G	0.1701	0.1842	C	0.5953	0.908
17	rs2469825	38841662	C	0.4124	0.4283	A	0.5953	0.9366
17	rs2462961	38855772	A	0.4124	0.4283	G	0.5953	0.9366
2	rs7568054	190497896	G	0.0567	0.04917	A	0.5954	1.162
3	rs7628371	180433381	G	0.4407	0.4242	A	0.5954	1.07
6	rs1610677	29789171	A	0.4149	0.4317	G	0.5954	0.9338
11	rs4758540	3174177	A	0.4149	0.4317	G	0.5954	0.9338
11	rs4131241	114916450	G	0.4149	0.4317	A	0.5954	0.9338
2	rs13019430	171219055	A	0.4149	0.4308	G	0.5956	0.937
12	rs3813526	50189602	A	0.4175	0.4342	G	0.5956	0.9342
17	rs9913890	15298776	G	0.4175	0.4342	A	0.5956	0.9342
20	rs211835	38478566	A	0.4149	0.4308	G	0.5956	0.937
5	rs13162502	53815560	C	0.07474	0.08417	A	0.5958	0.879
1	rs11264498	156526387	G	0.4459	0.4292	C	0.5959	1.07
11	rs4617603	122868205	G	0.4201	0.4367	A	0.5959	0.9346
2	rs3820928	227773466	G	0.4433	0.4275	A	0.596	1.066
10	rs805722	105810400	A	0.1907	0.1783	G	0.596	1.086
17	rs11567842	26824156	G	0.4433	0.4275	A	0.596	1.066
3	rs9813474	130545096	G	0.4485	0.4317	A	0.5962	1.071
4	rs2736990	90678541	A	0.4227	0.4392	G	0.5962	0.935
6	rs831510	36976637	C	0.4227	0.4392	G	0.5962	0.935
7	rs6462770	4392176	A	0.4201	0.4358	G	0.5962	0.9378
9	rs4366181	100130345	G	0.4459	0.43	A	0.5962	1.067
12	rs4768109	46658059	A	0.4227	0.4392	G	0.5962	0.935
13	rs4772372	102033686	A	0.4227	0.4392	G	0.5962	0.935
19	rs1644731	10131999	A	0.4227	0.4392	G	0.5962	0.935
20	rs2208451	58711935	G	0.4485	0.4317	A	0.5962	1.071
5	rs56080630	154395682	A	0.116	0.1275	G	0.5963	0.8978
5	rs60928118	154396505	A	0.116	0.1275	G	0.5963	0.8978
6	rs12614	31914179	A	0.116	0.1275	G	0.5963	0.8978
13	rs2282133	99449717	A	0.116	0.1275	G	0.5963	0.8978
9	rs10756070	10626531	C	0.451	0.4342	A	0.5964	1.071

16	rs238551	23375921	G	0.451	0.4342	A	0.5964	1.071
11	rs1792509	56670810	G	0.4227	0.4383	A	0.5965	0.9381
12	rs10843843	30979116	G	0.4227	0.4383	A	0.5965	0.9381
19	rs3745936	42092815	T	0.4227	0.4383	A	0.5965	0.9381
4	rs734312	6303354	G	0.116	0.1283	A	0.5966	0.8911
7	rs10156191	150553605	A	0.116	0.1283	G	0.5966	0.8911
19	rs4072910	8644031	G	0.2526	0.2667	C	0.5966	0.9293
19	rs7252127	40427903	C	0.451	0.435	A	0.5966	1.067
6	rs6926980	56917538	A	0.09021	0.08083	G	0.5968	1.127
8	rs59511580	22473725	G	0.09021	0.08083	C	0.5968	1.127
2	rs10804117	202410300	T	0.2758	0.2617	A	0.5969	1.074
6	rs2442719	31320538	A	0.2758	0.2617	G	0.5969	1.074
14	rs17175506	80408653	A	0.4304	0.4467	G	0.5969	0.9361
1	rs2273682	67581127	G	0.1727	0.1858	A	0.5971	0.9144
2	rs4399693	8694850	G	0.4278	0.4433	A	0.5971	0.9389
14	rs8660	59939727	G	0.1727	0.1858	A	0.5971	0.9144
14	rs9972231	105615648	A	0.1727	0.1858	G	0.5971	0.9144
19	rs12976472	14877799	G	0.4844	0.4674	C	0.5971	1.07
2	rs7557290	77512571	A	0.1907	0.1792	G	0.5972	1.08
3	rs7636910	183699516	G	0.4356	0.4525	A	0.5972	0.9337
6	rs3734354	100868779	A	0.4583	0.475	C	0.5972	0.9352
10	rs10995271	64438486	C	0.433	0.4492	G	0.5972	0.9365
11	rs921561	97319496	A	0.4356	0.4525	G	0.5972	0.9337
12	rs12833456	52986187	A	0.4588	0.4425	G	0.5972	1.068
12	rs11170187	52992812	A	0.4588	0.4425	G	0.5972	1.068
13	rs3783082	25428002	A	0.1907	0.1792	C	0.5972	1.08
7	rs2707466	120979089	A	0.1727	0.1867	G	0.5973	0.9094
9	rs1333111	9416962	G	0.1727	0.1867	A	0.5973	0.9094
10	rs151595	115719765	G	0.4613	0.445	A	0.5974	1.068
10	rs746832	122348065	A	0.4356	0.4517	G	0.5974	0.9368
11	rs6486182	14233522	G	0.4356	0.4517	A	0.5974	0.9368
15	rs1549318	71109147	A	0.4356	0.4517	G	0.5974	0.9368
17	rs2291189	33592621	A	0.2552	0.27	G	0.5974	0.9262
3	rs9290936	190252635	A	0.4639	0.4475	C	0.5975	1.068
9	rs28624420	91360612	A	0.4562	0.4408	G	0.5975	1.064
9	rs17343890	123349699	G	0.4639	0.4475	A	0.5975	1.068
12	rs10771012	23788075	G	0.2552	0.2708	A	0.5975	0.9223
2	rs4852681	82432988	G	0.4381	0.4541	A	0.5976	0.9375
6	rs10440833	20688121	A	0.4588	0.4433	T	0.5976	1.064
13	rs11619319	28487599	G	0.4407	0.4575	A	0.5976	0.9344
14	rs1202667	55037156	G	0.4588	0.4433	A	0.5976	1.064
2	rs3112256	101321822	G	0.2758	0.2625	A	0.5977	1.07
4	rs1450432	139443162	G	0.2758	0.2625	A	0.5977	1.07

15	rs1568657	83726179	A	0.4534	0.47	G	0.5977	0.9353
1	rs7535533	182496829	G	0.4613	0.4458	A	0.5978	1.065
1	rs2378607	222919895	A	0.1933	0.1808	C	0.5979	1.085
2	rs2357266	9924387	A	0.4639	0.4483	G	0.5979	1.065
5	rs820875	73986648	A	0.4639	0.4483	G	0.5979	1.065
5	rs264879	169078934	G	0.4639	0.4483	A	0.5979	1.065
7	rs2032582	87160618	C	0.4407	0.4567	A	0.5979	0.9376
12	rs4883263	7649484	A	0.2784	0.2642	G	0.5979	1.074
3	rs3846087	101211487	A	0.4665	0.4508	G	0.5981	1.065
16	rs5882	57016092	G	0.4485	0.465	A	0.5981	0.9355
20	rs3810510	42747247	A	0.4665	0.4508	G	0.5981	1.065
1	rs7539409	84254735	G	0.0232	0.02917	A	0.5982	0.7904
1	rs55807605	196706049	A	0.0232	0.02917	G	0.5982	0.7904
2	rs1796049	97645785	A	0.4691	0.4533	G	0.5982	1.065
2	rs17244632	165551404	A	0.0232	0.02917	G	0.5982	0.7904
3	rs117655885	40573556	G	0.0232	0.02917	A	0.5982	0.7904
3	rs116932390	48474258	G	0.0232	0.02917	A	0.5982	0.7904
6	rs79802085	128411109	A	0.0232	0.02917	G	0.5982	0.7904
11	rs2672785	1246941	G	0.4691	0.4533	A	0.5982	1.065
11	rs61063081	7059981	A	0.0232	0.02917	G	0.5982	0.7904
11	rs116286425	77937768	A	0.0232	0.02917	G	0.5982	0.7904
12	rs28919570	6925407	A	0.0232	0.02917	G	0.5982	0.7904
16	rs1126933	68721533	G	0.4691	0.4533	C	0.5982	1.065
17	rs139293791	8726792	A	0.0232	0.02917	G	0.5982	0.7904
18	rs80252896	22048821	C	0.0232	0.02917	A	0.5982	0.7904
19	rs117581914	9236724	C	0.0232	0.02917	G	0.5982	0.7904
19	rs11575935	18174731	A	0.0232	0.02917	G	0.5982	0.7904
1	rs10489962	15639581	G	0.0232	0.03	A	0.5983	0.7678
1	rs117798340	86822204	A	0.0232	0.03	G	0.5983	0.7678
2	rs35195207	17897488	G	0.0232	0.03	A	0.5983	0.7678
2	rs34069439	17998025	T	0.0232	0.03	A	0.5983	0.7678
2	rs117864472	228560645	G	0.0232	0.03	A	0.5983	0.7678
2	rs34507036	228560728	G	0.0232	0.03	A	0.5983	0.7678
3	rs13073817	18706858	A	0.0232	0.03	G	0.5983	0.7678
4	rs2070011	155511897	G	0.451	0.4675	A	0.5983	0.9358
5	rs2240698	140763100	A	0.1347	0.1244	G	0.5983	1.096
6	rs9345612	66083780	A	0.4459	0.4617	G	0.5983	0.9383
6	rs17847665	160524825	G	0.0232	0.03	A	0.5983	0.7678
7	rs77298949	4876207	A	0.0232	0.03	G	0.5983	0.7678
7	rs62441683	21628242	G	0.0232	0.03	C	0.5983	0.7678
9	rs1076160	135776034	A	0.451	0.4675	G	0.5983	0.9358
10	rs9663814	77738382	A	0.4716	0.4558	G	0.5983	1.066
16	rs62038760	11887629	G	0.451	0.4675	A	0.5983	0.9358

22	rs117220882	24717637	A	0.0232	0.03	G	0.5983	0.7678
10	rs1775091	43420922	A	0.4794	0.4625	G	0.5984	1.07
15	rs937732	76496232	G	0.4794	0.4625	A	0.5984	1.07
2	rs956968	16138879	A	0.482	0.465	G	0.5985	1.07
5	rs11241713	123119565	A	0.4896	0.4741	C	0.5985	1.064
5	rs10052344	173297883	A	0.4536	0.47	G	0.5985	0.9362
6	rs6910666	109049607	G	0.4536	0.47	A	0.5985	0.9362
10	rs10781587	134629053	G	0.482	0.465	A	0.5985	1.07
14	rs9624	21500121	A	0.2577	0.2725	C	0.5985	0.927
14	rs61991867	68191368	G	0.482	0.465	A	0.5985	1.07
17	rs61562914	35272108	A	0.4485	0.4642	G	0.5985	0.9386
18	rs4602126	43948582	G	0.4485	0.4642	A	0.5985	0.9386
19	rs4802605	49930721	G	0.482	0.465	A	0.5985	1.07
3	rs2895315	106393843	C	0.4588	0.4758	A	0.5986	0.9337
5	rs1494555	35871190	A	0.4588	0.4758	G	0.5986	0.9337
6	rs241437	32797684	A	0.4794	0.4633	G	0.5986	1.067
7	rs2293404	2644519	G	0.4794	0.4633	A	0.5986	1.067
8	rs7831261	135346972	A	0.2577	0.2733	C	0.5986	0.9231
15	rs1044032	45968435	G	0.4794	0.4633	A	0.5986	1.067
15	rs17374222	69995344	C	0.2577	0.2733	A	0.5986	0.9231
1	rs734999	2513216	A	0.482	0.4658	G	0.5987	1.067
1	rs2022858	102608577	G	0.4613	0.4783	A	0.5987	0.934
6	rs2621323	32788707	G	0.4613	0.4783	A	0.5987	0.934
1	rs2791494	86959173	A	0.0567	0.05	G	0.5988	1.142
2	rs6753101	8104849	A	0.4871	0.4708	C	0.5988	1.067
3	rs3856580	73484914	C	0.09021	0.08167	A	0.5988	1.115
3	rs13083186	118356071	G	0.4639	0.4808	A	0.5988	0.9344
7	rs12673041	100371417	A	0.0567	0.05	G	0.5988	1.142
8	rs141504768	68999963	A	0.09021	0.08167	C	0.5988	1.115
10	rs4253211	50678317	G	0.0567	0.05	C	0.5988	1.142
12	rs7305599	51901390	G	0.4871	0.4708	A	0.5988	1.067
12	rs12580100	56439209	G	0.2784	0.265	A	0.5988	1.07
12	rs7955450	96337183	A	0.09021	0.08167	G	0.5988	1.115
13	rs1329667	34491838	A	0.4845	0.4683	G	0.5988	1.067
13	rs61756566	42876103	A	0.0567	0.05	G	0.5988	1.142
17	rs2302319	4797305	A	0.4536	0.4691	G	0.5988	0.9395
17	rs7208422	76130575	T	0.4871	0.4708	A	0.5988	1.067
18	rs11874468	8813046	C	0.0567	0.05	A	0.5988	1.142
19	rs1042229	52249672	C	0.4845	0.4683	A	0.5988	1.067
21	rs12329924	21639279	C	0.4845	0.4683	A	0.5988	1.067
3	rs7642894	39957493	G	0.4897	0.4733	A	0.5989	1.068
13	rs4769841	31108727	G	0.4613	0.4775	A	0.5989	0.9372
14	rs2402341	94287048	A	0.4665	0.4833	G	0.5989	0.9347

16	rs8058694	16278863	A	0.1943	0.1825	C	0.5989	1.08
1	rs35077499	31661921	A	0.4639	0.48	G	0.599	0.9375
1	rs17030613	113190807	A	0.4716	0.4883	C	0.599	0.9353
3	rs7633596	59678338	C	0.4948	0.4783	A	0.599	1.068
3	rs6780568	95519536	C	0.4742	0.4908	A	0.599	0.9357
5	rs3828609	149432863	G	0.1933	0.1817	A	0.599	1.079
5	rs3733875	176637240	A	0.4639	0.48	C	0.599	0.9375
5	rs28932178	176637576	G	0.4639	0.48	A	0.599	0.9375
14	rs6572579	49904851	G	0.4948	0.4783	A	0.599	1.068
14	rs3737035	90745422	A	0.4948	0.4783	G	0.599	1.068
15	rs12911738	40903684	G	0.2809	0.2667	A	0.599	1.074
1	rs873917	40132795	A	0.4665	0.4825	C	0.5991	0.9378
6	rs6900042	30289271	G	0.5	0.4833	A	0.5991	1.069
8	rs7842128	26518780	G	0.4665	0.4825	A	0.5991	0.9378
9	rs4742762	101855570	A	0.4974	0.4808	G	0.5991	1.069
10	rs9325890	48494099	A	0.4974	0.4808	C	0.5991	1.069
18	rs6566532	69051326	G	0.4974	0.4808	A	0.5991	1.069
19	rs370094	6904137	A	0.4742	0.4908	G	0.5991	0.9357
1	rs12028397	4037665	A	0.4588	0.4742	G	0.5992	0.94
1	rs1329428	196702810	A	0.4845	0.4692	G	0.5992	1.064
2	rs4522556	160957846	A	0.4691	0.485	G	0.5992	0.9381
3	rs6805421	42728144	A	0.1753	0.1892	G	0.5992	0.9108
4	rs10516676	83462759	A	0.1753	0.1892	G	0.5992	0.9108
5	rs3749817	132535046	G	0.4691	0.485	A	0.5992	0.9382
6	rs2269346	33158898	G	0.4871	0.5042	A	0.5992	0.9341
6	rs1405069	36922684	C	0.5077	0.4908	A	0.5992	1.07
6	rs1687128	47094118	C	0.4871	0.5042	A	0.5992	0.9341
7	rs2244639	82893679	G	0.4588	0.4742	A	0.5992	0.94
9	rs10908907	92249584	G	0.5077	0.4908	A	0.5992	1.07
9	rs10820412	106058590	G	0.4871	0.5042	A	0.5992	0.9341
11	rs3802799	20065673	A	0.4794	0.4958	C	0.5992	0.9363
11	rs11232816	81441351	G	0.5103	0.4933	A	0.5992	1.07
14	rs17719756	57454439	G	0.5077	0.4908	A	0.5992	1.07
14	rs4902264	64491695	A	0.1753	0.1892	G	0.5992	0.9108
15	rs1532085	58683366	G	0.4768	0.4933	A	0.5992	0.936
15	rs16948824	86707722	C	0.4871	0.5042	A	0.5992	0.9341
20	rs6052699	4611877	G	0.4794	0.4958	A	0.5992	0.9363
4	rs544296	150217461	C	0.4871	0.4717	A	0.5993	1.064
5	rs7702283	114974122	G	0.4871	0.5033	A	0.5993	0.9372
6	rs12661281	31842598	T	0.134	0.1242	A	0.5993	1.092
6	rs937055	47086952	A	0.4845	0.5008	G	0.5993	0.9369
6	rs984684	57133333	G	0.1959	0.1828	A	0.5993	1.089
8	rs4455823	34524479	G	0.4871	0.5033	A	0.5993	0.9372

8	rs2875967	61428280	C	0.4923	0.4767	A	0.5993	1.064
11	rs12801636	65391317	A	0.4845	0.5008	G	0.5993	0.9369
11	rs17122021	118145686	A	0.4845	0.5008	G	0.5993	0.9369
12	rs9651971	83239281	A	0.134	0.1242	G	0.5993	1.092
13	rs7319493	114175038	A	0.134	0.1242	G	0.5993	1.092
14	rs3813563	73958812	A	0.4897	0.4742	G	0.5993	1.064
20	rs5741809	36956026	G	0.4923	0.4767	A	0.5993	1.064
3	rs842274	16327855	A	0.5077	0.4917	C	0.5994	1.066
4	rs6841520	183553379	G	0.4742	0.49	A	0.5994	0.9388
6	rs213210	33175824	G	0.5103	0.4942	A	0.5994	1.067
8	rs2958710	106772453	C	0.4948	0.4792	A	0.5994	1.065
10	rs3789940	98085338	A	0.5	0.4842	G	0.5994	1.065
13	rs660622	101795160	A	0.4948	0.4792	C	0.5994	1.065
17	rs12601317	79424516	G	0.5103	0.4942	A	0.5994	1.067
18	rs9949946	3797659	G	0.5026	0.4867	A	0.5994	1.066
20	rs6118279	8626282	A	0.5103	0.4942	G	0.5994	1.067
21	rs2833610	33385186	G	0.4742	0.49	A	0.5994	0.9388
3	rs3732487	123419573	C	0.4768	0.4925	A	0.5995	0.9391
7	rs2718021	36079709	A	0.4768	0.4925	G	0.5995	0.9391
19	rs897738	6901891	A	0.4768	0.4925	G	0.5995	0.9391
15	rs674155	33872177	G	0.4794	0.495	A	0.5996	0.9394
2	rs3738880	121746956	A	0.4845	0.5	C	0.5997	0.94
6	rs916570	30066031	G	0.2603	0.275	A	0.5997	0.9278
7	rs218979	10975011	G	0.2603	0.275	A	0.5997	0.9278
8	rs11136000	27464519	A	0.2603	0.2758	G	0.5997	0.9239
12	rs7315731	46321514	T	0.4845	0.5	A	0.5997	0.94
3	rs35217513	40557443	A	0.1186	0.1308	C	0.5998	0.8935
3	rs2271087	44929287	G	0.1959	0.1833	C	0.5998	1.085
4	rs4862650	187074833	A	0.1959	0.1833	G	0.5998	1.085
7	rs10245760	93746499	A	0.1186	0.1308	G	0.5998	0.8935
8	rs16936922	71239363	A	0.2809	0.2675	G	0.5998	1.07
10	rs363315	118969346	G	0.1186	0.1308	A	0.5998	0.8935
15	rs11636613	77335902	G	0.4871	0.5025	A	0.5998	0.9403
15	rs1455782	93050966	A	0.1186	0.1308	G	0.5998	0.8935
19	rs1056185	53117531	G	0.2809	0.2675	A	0.5998	1.07
20	rs35031530	55033420	A	0.2809	0.2675	G	0.5998	1.07
6	rs281868	118574061	A	0.2835	0.2692	G	0.6	1.074
6	rs89107	118578043	A	0.2835	0.2692	G	0.6	1.074
12	rs200489873	106461103	A	0.005208	0.0025	G	0.6002	2.089
19	rs12971926	21837430	G	0.2835	0.2697	A	0.6002	1.072
9	rs10961689	14737506	A	0.2642	0.2792	C	0.6006	0.9274
1	rs61744700	6634764	G	0.01546	0.01167	A	0.6007	1.331
1	rs12721465	20305016	A	0.01546	0.01167	G	0.6007	1.331

2	rs2304602	136537941	C	0.01546	0.01167	G	0.6007	1.331
3	rs190867803	12977856	A	0.01546	0.01167	G	0.6007	1.331
4	rs76345011	79372992	G	0.01546	0.01167	A	0.6007	1.331
4	rs149931440	157891963	A	0.01546	0.01167	C	0.6007	1.331
5	rs11739663	594083	G	0.01546	0.01167	A	0.6007	1.331
5	rs12522955	639231	A	0.01546	0.01167	C	0.6007	1.331
5	rs55638729	66458615	C	0.01546	0.01167	A	0.6007	1.331
5	rs201107418	134785326	A	0.01546	0.01167	G	0.6007	1.331
5	rs7706614	137754808	A	0.01546	0.01167	G	0.6007	1.331
5	rs61749634	137766066	A	0.01546	0.01167	G	0.6007	1.331
5	exm2091824	149225335	A	0.01546	0.01167	G	0.6007	1.331
5	rs143268818	149225335	A	0.01546	0.01167	G	0.6007	1.331
6	rs149106445	33372831	G	0.01546	0.01167	A	0.6007	1.331
6	rs35972078	151789868	A	0.01546	0.01167	G	0.6007	1.331
6	rs114696942	155577671	A	0.01546	0.01167	G	0.6007	1.331
6	rs139253533	166755933	G	0.01546	0.01167	C	0.6007	1.331
7	rs138397380	1512808	G	0.01546	0.01167	C	0.6007	1.331
7	rs3778922	151802332	A	0.01546	0.01167	C	0.6007	1.331
7	rs980442	154876342	A	0.1366	0.1258	C	0.6007	1.099
8	rs181641183	145051711	A	0.01546	0.01167	C	0.6007	1.331
9	rs145731729	2717819	A	0.01546	0.01167	G	0.6007	1.331
10	rs12774726	67199027	A	0.2629	0.2783	G	0.6007	0.9247
10	rs115775127	72541595	A	0.01546	0.01167	G	0.6007	1.331
10	rs3814211	85991784	G	0.01546	0.01167	A	0.6007	1.331
10	rs363294	119043554	A	0.01546	0.01167	G	0.6007	1.331
10	rs185020036	120095773	G	0.005181	0.0025	A	0.6007	2.078
11	rs2276093	102826412	A	0.01546	0.01167	G	0.6007	1.331
12	rs141642623	96275306	G	0.01546	0.01167	A	0.6007	1.331
14	rs151207390	61180673	C	0.01546	0.01167	A	0.6007	1.331
16	rs200670079	31098107	A	0.01546	0.01167	C	0.6007	1.331
16	rs2639889	61123147	G	0.2629	0.2783	A	0.6007	0.9247
16	rs140084649	70500084	A	0.01546	0.01167	G	0.6007	1.331
16	rs9888988	73495661	G	0.2629	0.2783	A	0.6007	0.9247
16	rs138454126	81056395	C	0.01546	0.01167	A	0.6007	1.331
17	rs143246503	8000049	A	0.01546	0.01167	G	0.6007	1.331
17	rs2301626	48156557	A	0.01546	0.01167	G	0.6007	1.331
17	rs201764380	67129981	G	0.01546	0.01167	C	0.6007	1.331
18	rs117084287	29784171	A	0.01546	0.01167	G	0.6007	1.331
18	rs77760128	67863745	G	0.01546	0.01167	A	0.6007	1.331
19	rs62620757	13936385	A	0.1366	0.1258	G	0.6007	1.099
19	rs78635115	49964111	G	0.01546	0.01167	A	0.6007	1.331
19	rs61743893	52568422	C	0.01546	0.01167	A	0.6007	1.331
22	rs149914294	50435827	A	0.01546	0.01167	G	0.6007	1.331

22	rs142430606	50436488	A	0.01546	0.01167	G	0.6007	1.331
22	rs200958270	50438279	G	0.01546	0.01167	A	0.6007	1.331
22	rs150229652	50438379	C	0.01546	0.01167	A	0.6007	1.331
5	rs200819997	177022364	A	0.005181	0.002504	G	0.6008	2.075
12	rs7970273	39046064	G	0.2629	0.2775	A	0.6008	0.9286
1	rs2481694	62675892	G	0.1959	0.1842	A	0.6009	1.079
3	rs61744388	41756965	A	0.1959	0.1842	G	0.6009	1.079
3	rs61744385	41756986	T	0.1959	0.1842	A	0.6009	1.079
12	rs4758826	61924685	C	0.1959	0.1842	A	0.6009	1.079
16	rs77488103	846811	G	0.01546	0.01169	C	0.6009	1.328
16	rs3747579	4445327	G	0.1959	0.1842	A	0.6009	1.079
1	rs77261230	10659333	C	0.03351	0.0275	A	0.6011	1.226
1	rs202164592	17257819	A	0.005155	0.0025	G	0.6011	2.067
1	rs189027200	22141269	A	0.005155	0.0025	C	0.6011	2.067
1	rs138831719	27699644	A	0.005155	0.0025	G	0.6011	2.067
1	rs183415309	38265258	G	0.005155	0.0025	A	0.6011	2.067
1	rs201919334	39797583	G	0.005155	0.0025	A	0.6011	2.067
1	rs55654497	45271238	A	0.005155	0.0025	G	0.6011	2.067
1	rs145825874	53108545	A	0.005155	0.0025	G	0.6011	2.067
1	rs147227819	55224552	A	0.005155	0.0025	G	0.6011	2.067
1	rs187897102	57431601	A	0.005155	0.0025	C	0.6011	2.067
1	rs185093512	94487460	A	0.005155	0.0025	G	0.6011	2.067
1	rs145677992	110293388	A	0.005155	0.0025	G	0.6011	2.067
1	rs150129126	110604142	A	0.005155	0.0025	G	0.6011	2.067
1	rs138975338	112031476	A	0.005155	0.0025	G	0.6011	2.067
1	rs144508898	117699283	G	0.005155	0.0025	C	0.6011	2.067
1	rs148749865	151016188	A	0.005155	0.0025	G	0.6011	2.067
1	rs148623396	151076116	A	0.005155	0.0025	C	0.6011	2.067
1	rs141800928	160786549	G	0.005155	0.0025	A	0.6011	2.067
1	rs202017285	167096275	A	0.005155	0.0025	G	0.6011	2.067
1	rs202187751	177936996	C	0.005155	0.0025	G	0.6011	2.067
1	rs77981586	180886063	A	0.03351	0.0275	G	0.6011	1.226
1	rs145064363	181018422	A	0.005155	0.0025	G	0.6011	2.067
1	rs200558564	223905492	G	0.005155	0.0025	A	0.6011	2.067
1	rs202108267	233514836	A	0.005155	0.0025	G	0.6011	2.067
2	rs186289991	7023549	A	0.005155	0.0025	G	0.6011	2.067
2	rs4592896	31399988	A	0.1778	0.1917	G	0.6011	0.9122
2	rs138349062	53921138	G	0.005155	0.0025	A	0.6011	2.067
2	rs199564543	58392928	A	0.005155	0.0025	G	0.6011	2.067
2	rs7570797	68873071	G	0.03351	0.0275	A	0.6011	1.226
2	rs4983	70900420	A	0.005155	0.0025	G	0.6011	2.067
2	rs148044176	73799821	A	0.005155	0.0025	G	0.6011	2.067
2	rs200184935	100052398	C	0.005155	0.0025	A	0.6011	2.067

2	rs201748115	120858314	C	0.005155	0.0025	A	0.6011	2.067
2	rs201842772	125204345	A	0.005155	0.0025	G	0.6011	2.067
2	rs111765932	128366416	G	0.005155	0.0025	A	0.6011	2.067
2	rs116182492	200820862	A	0.005155	0.0025	C	0.6011	2.067
2	rs3769824	202122956	G	0.005155	0.0025	A	0.6011	2.067
3	rs144084212	15686270	A	0.005155	0.0025	G	0.6011	2.067
3	rs188839847	37340833	G	0.005155	0.0025	A	0.6011	2.067
3	rs2270569	47316842	A	0.005155	0.0025	G	0.6011	2.067
3	rs201916805	48630293	A	0.005155	0.0025	G	0.6011	2.067
3	rs186089632	62317022	A	0.005155	0.0025	C	0.6011	2.067
3	rs3733124	63981215	G	0.03351	0.0275	A	0.6011	1.226
3	rs149389279	77614161	G	0.005155	0.0025	A	0.6011	2.067
3	rs202109497	113697072	A	0.005155	0.0025	G	0.6011	2.067
3	rs200871576	119379078	A	0.005155	0.0025	G	0.6011	2.067
3	rs117121840	123022002	G	0.005155	0.0025	A	0.6011	2.067
3	rs35306038	142285065	G	0.005155	0.0025	A	0.6011	2.067
3	rs186155706	179095227	G	0.005155	0.0025	A	0.6011	2.067
3	rs73057608	187930047	G	0.005155	0.0025	A	0.6011	2.067
3	rs201921184	194337905	A	0.005155	0.0025	G	0.6011	2.067
4	rs79826315	619560	A	0.03351	0.0275	C	0.6011	1.226
4	rs200183497	951622	A	0.005155	0.0025	G	0.6011	2.067
4	rs138327840	1019094	A	0.005155	0.0025	G	0.6011	2.067
4	rs147834269	6303731	A	0.03351	0.0275	G	0.6011	1.226
4	rs145396953	44724158	A	0.005155	0.0025	G	0.6011	2.067
4	rs200400344	87622856	A	0.005155	0.0025	G	0.6011	2.067
4	rs146552179	88902704	A	0.005155	0.0025	C	0.6011	2.067
4	rs149924953	90816448	A	0.005155	0.0025	T	0.6011	2.067
4	rs182933054	94411912	A	0.005155	0.0025	T	0.6011	2.067
4	rs140012318	153889208	A	0.005155	0.0025	G	0.6011	2.067
4	rs79967711	174169569	A	0.005155	0.0025	G	0.6011	2.067
4	rs144526682	187584696	A	0.005155	0.0025	G	0.6011	2.067
5	rs1433271	4456841	G	0.005155	0.0025	A	0.6011	2.067
5	rs182494860	10415633	A	0.005155	0.0025	G	0.6011	2.067
5	rs199816039	56171125	A	0.005155	0.0025	G	0.6011	2.067
5	rs114301971	68504097	G	0.005155	0.0025	A	0.6011	2.067
5	rs118124886	78587100	A	0.005155	0.0025	G	0.6011	2.067
5	rs183984308	82843864	C	0.005155	0.0025	G	0.6011	2.067
5	rs13157174	117912524	C	0.1778	0.1917	A	0.6011	0.9122
5	rs199679276	141233707	A	0.005155	0.0025	G	0.6011	2.067
5	rs13169495	144625079	G	0.03351	0.0275	A	0.6011	1.226
5	rs112438191	156378556	A	0.005155	0.0025	G	0.6011	2.067
5	rs201396068	178413718	A	0.005155	0.0025	G	0.6011	2.067
5	rs41285557	179195867	A	0.005155	0.0025	G	0.6011	2.067

6	rs117415551	42995126	A	0.005155	0.0025	G	0.6011	2.067
6	rs150329930	46684169	G	0.005155	0.0025	A	0.6011	2.067
6	rs117107973	71567717	C	0.005155	0.0025	G	0.6011	2.067
6	rs142498397	74502394	A	0.005155	0.0025	G	0.6011	2.067
6	rs146461956	76624695	G	0.005155	0.0025	A	0.6011	2.067
6	rs200381959	83888401	C	0.005155	0.0025	A	0.6011	2.067
6	rs201325694	116600621	A	0.005155	0.0025	C	0.6011	2.067
6	rs141521127	129588330	A	0.005155	0.0025	G	0.6011	2.067
7	rs140752909	16505144	A	0.005155	0.0025	C	0.6011	2.067
7	rs145151450	76112299	G	0.03351	0.0275	A	0.6011	1.226
7	rs17847377	113519666	A	0.005155	0.0025	G	0.6011	2.067
7	rs2908004	120969769	A	0.1778	0.1917	G	0.6011	0.9122
7	rs11980387	121513570	A	0.005155	0.0025	C	0.6011	2.067
7	rs150002869	129095138	G	0.005155	0.0025	A	0.6011	2.067
7	rs115547267	138849974	A	0.005155	0.0025	G	0.6011	2.067
7	rs9655672	143792867	A	0.005155	0.0025	G	0.6011	2.067
8	rs148981308	17500131	A	0.005155	0.0025	G	0.6011	2.067
8	rs141857173	17611492	C	0.005155	0.0025	A	0.6011	2.067
8	rs13306060	110100245	C	0.005155	0.0025	A	0.6011	2.067
8	rs148571616	113516174	G	0.005155	0.0025	A	0.6011	2.067
8	rs189112958	125047645	A	0.005155	0.0025	G	0.6011	2.067
8	rs200524049	145640632	A	0.005155	0.0025	G	0.6011	2.067
8	rs3735784	146157860	A	0.005155	0.0025	G	0.6011	2.067
9	rs77236826	71863235	G	0.005155	0.0025	A	0.6011	2.067
9	rs202147412	106896757	A	0.005155	0.0025	G	0.6011	2.067
9	rs78046206	118138995	A	0.005155	0.0025	C	0.6011	2.067
9	rs115678685	131475665	A	0.005155	0.0025	G	0.6011	2.067
9	rs201286389	132630582	A	0.005155	0.0025	G	0.6011	2.067
9	rs199992644	139889895	A	0.005155	0.0025	G	0.6011	2.067
10	rs2854482	5043821	A	0.005155	0.0025	T	0.6011	2.067
10	rs17134592	5260682	C	0.09459	0.08612	G	0.6011	1.109
10	rs34630110	13214753	C	0.03351	0.0275	G	0.6011	1.226
10	rs201839212	99995805	A	0.005155	0.0025	G	0.6011	2.067
10	rs142860219	101165607	G	0.005155	0.0025	A	0.6011	2.067
10	rs116720164	134155774	A	0.005155	0.0025	C	0.6011	2.067
10	rs148597749	135102440	A	0.005155	0.0025	G	0.6011	2.067
11	rs200807624	403711	A	0.005155	0.0025	G	0.6011	2.067
11	rs80298911	1256409	A	0.005155	0.0025	G	0.6011	2.067
11	rs73403015	4825022	G	0.005155	0.0025	A	0.6011	2.067
11	rs142485585	7064876	A	0.005155	0.0025	G	0.6011	2.067
11	rs199708782	44105284	A	0.005155	0.0025	T	0.6011	2.067
11	rs17153770	59224738	G	0.03351	0.0275	A	0.6011	1.226
11	rs188599658	62519876	A	0.005155	0.0025	G	0.6011	2.067

11	rs191746035	65357978	A	0.005155	0.0025	C	0.6011	2.067
11	rs142808899	71148914	A	0.005155	0.0025	G	0.6011	2.067
11	rs189548101	102663449	A	0.005155	0.0025	G	0.6011	2.067
11	rs148591515	118403682	C	0.005155	0.0025	G	0.6011	2.067
11	rs78778622	124530664	G	0.005155	0.0025	A	0.6011	2.067
12	rs139185675	6762500	G	0.005155	0.0025	A	0.6011	2.067
12	rs199878433	9304243	A	0.005155	0.0025	G	0.6011	2.067
12	rs188078627	9334698	A	0.005155	0.0025	G	0.6011	2.067
12	rs150287042	42853913	A	0.005155	0.0025	G	0.6011	2.067
12	rs182987633	48458912	G	0.005155	0.0025	A	0.6011	2.067
12	rs200921207	50483727	A	0.005155	0.0025	G	0.6011	2.067
12	rs77545847	55524135	A	0.005155	0.0025	G	0.6011	2.067
12	rs143080244	55726373	A	0.005155	0.0025	G	0.6011	2.067
12	rs199837509	95451388	G	0.005155	0.0025	A	0.6011	2.067
12	rs201585823	120575553	G	0.005155	0.0025	C	0.6011	2.067
12	rs3741447	122825413	A	0.005155	0.0025	G	0.6011	2.067
12	rs200898397	133373125	A	0.005155	0.0025	G	0.6011	2.067
13	rs55974987	29012441	A	0.005155	0.0025	G	0.6011	2.067
13	rs76367683	33252994	G	0.005155	0.0025	A	0.6011	2.067
13	rs114171443	39338456	A	0.005155	0.0025	G	0.6011	2.067
13	rs149509211	42876861	G	0.005155	0.0025	A	0.6011	2.067
13	rs79697600	44442874	A	0.005155	0.0025	G	0.6011	2.067
13	rs77339766	52604652	C	0.005155	0.0025	G	0.6011	2.067
13	rs41300600	98828892	G	0.005155	0.0025	A	0.6011	2.067
14	rs201049979	20388910	A	0.005155	0.0025	G	0.6011	2.067
14	rs115122004	21250144	C	0.005155	0.0025	A	0.6011	2.067
14	rs17111920	27406892	A	0.005155	0.0025	C	0.6011	2.067
14	rs116855254	52477613	A	0.005155	0.0025	G	0.6011	2.067
14	rs147119902	58896083	A	0.005155	0.0025	T	0.6011	2.067
14	rs1748986	58937416	A	0.005155	0.0025	G	0.6011	2.067
14	rs139493302	58941425	A	0.005155	0.0025	G	0.6011	2.067
14	rs149276487	68249803	C	0.005155	0.0025	A	0.6011	2.067
14	rs201453315	75388001	A	0.005155	0.0025	G	0.6011	2.067
14	rs139731348	77274292	A	0.005155	0.0025	G	0.6011	2.067
14	rs138297457	91666127	A	0.005155	0.0025	G	0.6011	2.067
14	rs183646530	91766410	A	0.005155	0.0025	G	0.6011	2.067
14	rs142288638	94594327	A	0.005155	0.0025	C	0.6011	2.067
14	rs2234146	103600056	A	0.005155	0.0025	G	0.6011	2.067
15	rs190048306	25959129	A	0.005155	0.0025	G	0.6011	2.067
15	rs181009746	41071757	A	0.005155	0.0025	G	0.6011	2.067
15	rs76010310	41099819	A	0.005155	0.0025	G	0.6011	2.067
15	rs116166434	41100001	C	0.005155	0.0025	G	0.6011	2.067
15	rs201139245	42374548	A	0.005155	0.0025	G	0.6011	2.067

15	rs189743179	42375445	A	0.005155	0.0025	G	0.6011	2.067
15	rs138839403	43021813	A	0.005155	0.0025	G	0.6011	2.067
15	rs201203376	65804899	G	0.005155	0.0025	A	0.6011	2.067
15	rs150013138	70960058	A	0.005155	0.0025	G	0.6011	2.067
15	rs201281548	74467345	A	0.005155	0.0025	G	0.6011	2.067
15	rs150691873	82574661	A	0.005155	0.0025	G	0.6011	2.067
15	rs200891085	90171718	G	0.005155	0.0025	A	0.6011	2.067
16	rs200722856	3254964	G	0.005155	0.0025	A	0.6011	2.067
16	rs117175899	31429420	A	0.005155	0.0025	G	0.6011	2.067
16	rs139481929	67222740	A	0.005155	0.0025	G	0.6011	2.067
16	rs200864375	67969333	C	0.005155	0.0025	G	0.6011	2.067
16	rs142001942	68012207	A	0.005155	0.0025	G	0.6011	2.067
16	rs200128996	68056661	A	0.005155	0.0025	C	0.6011	2.067
16	rs199904028	71319339	A	0.005155	0.0025	G	0.6011	2.067
16	rs202085761	87446500	C	0.005155	0.0025	G	0.6011	2.067
16	rs74033443	89294044	G	0.005155	0.0025	A	0.6011	2.067
17	rs181444163	1840677	A	0.005155	0.0025	G	0.6011	2.067
17	rs151091483	10312678	A	0.005155	0.0025	T	0.6011	2.067
17	rs4135013	38450261	A	0.005155	0.0025	G	0.6011	2.067
17	rs200840933	40958806	G	0.005155	0.0025	A	0.6011	2.067
17	rs199556388	42255661	A	0.005155	0.0025	G	0.6011	2.067
17	rs16939879	43216472	G	0.005155	0.0025	A	0.6011	2.067
17	rs182118556	81050940	A	0.005155	0.0025	C	0.6011	2.067
18	rs188722806	21512212	A	0.005155	0.0025	G	0.6011	2.067
18	rs149191001	28934293	A	0.005155	0.0025	G	0.6011	2.067
18	rs78622804	29432443	G	0.005155	0.0025	A	0.6011	2.067
18	rs189786599	31319293	A	0.005155	0.0025	G	0.6011	2.067
19	rs144396442	758121	A	0.03351	0.0275	G	0.6011	1.226
19	rs147230168	2431625	A	0.005155	0.0025	G	0.6011	2.067
19	rs140448414	8175953	A	0.005155	0.0025	G	0.6011	2.067
19	rs201343534	8320563	A	0.005155	0.0025	G	0.6011	2.067
19	rs145691237	11892137	A	0.005155	0.0025	G	0.6011	2.067
19	rs200184007	12089307	A	0.005155	0.0025	G	0.6011	2.067
19	rs138678978	14049013	A	0.005155	0.0025	G	0.6011	2.067
19	rs199764331	15533921	A	0.005155	0.0025	G	0.6011	2.067
19	rs201644487	19120943	C	0.005155	0.0025	G	0.6011	2.067
19	rs148497147	42583500	A	0.005155	0.0025	C	0.6011	2.067
19	rs199584109	46119772	G	0.005155	0.0025	C	0.6011	2.067
19	rs192952321	47909743	C	0.005155	0.0025	A	0.6011	2.067
19	rs61747437	53116514	A	0.005155	0.0025	G	0.6011	2.067
20	rs201864662	5528332	A	0.005155	0.0025	G	0.6011	2.067
20	rs201859601	7915210	A	0.005155	0.0025	C	0.6011	2.067
20	rs150412422	13279717	A	0.005155	0.0025	G	0.6011	2.067

20	rs143661747	25261594	A	0.005155	0.0025	G	0.6011	2.067
20	rs201729823	25457712	A	0.005155	0.0025	G	0.6011	2.067
20	rs190534425	30458965	A	0.005155	0.0025	G	0.6011	2.067
20	rs5743500	36937342	A	0.005155	0.0025	G	0.6011	2.067
20	rs199523729	39977500	A	0.005155	0.0025	G	0.6011	2.067
20	rs2273148	47872421	G	0.005155	0.0025	A	0.6011	2.067
20	rs34969822	62173817	A	0.005155	0.0025	G	0.6011	2.067
20	rs149145821	62326538	A	0.005155	0.0025	G	0.6011	2.067
21	rs62229404	37759957	A	0.005155	0.0025	G	0.6011	2.067
21	rs199936080	38126664	A	0.005155	0.0025	C	0.6011	2.067
21	rs200153989	43867288	C	0.005155	0.0025	G	0.6011	2.067
22	rs200793347	29726624	A	0.005155	0.0025	G	0.6011	2.067
22	rs115617635	30863040	A	0.005155	0.0025	G	0.6011	2.067
22	rs2307340	35802661	G	0.005155	0.0025	C	0.6011	2.067
22	rs201526262	38336799	A	0.005155	0.0025	G	0.6011	2.067
22	rs187455529	38474406	A	0.005155	0.0025	G	0.6011	2.067
1	rs142914187	11008864	A	0.005155	0.002504	G	0.6013	2.064
1	rs143930965	203143721	A	0.005155	0.002504	G	0.6013	2.064
2	rs200944866	55872526	C	0.005155	0.002504	A	0.6013	2.064
2	rs192754138	58386773	A	0.005155	0.002504	G	0.6013	2.064
11	rs142759842	113629371	A	0.005155	0.002504	G	0.6013	2.064
11	rs61756691	124440241	A	0.005155	0.002504	G	0.6013	2.064
14	rs142112144	56106698	G	0.005181	0.002517	A	0.6013	2.064
15	rs140262572	44066864	C	0.005155	0.002504	G	0.6013	2.064
15	rs192430485	65992924	A	0.005155	0.002504	G	0.6013	2.064
22	rs117258406	29885554	A	0.005155	0.002508	G	0.6014	2.06
9	rs201772265	72967219	A	0.005155	0.002513	G	0.6016	2.057
10	rs3188055	121586882	G	0.1985	0.1858	A	0.6016	1.085
22	rs143227882	50942862	C	0.005155	0.002513	G	0.6016	2.057
5	rs4956987	174106	A	0.2861	0.2725	G	0.6017	1.07
20	rs1884136	10850464	A	0.2861	0.2725	C	0.6017	1.07
1	rs117820608	1601104	A	0.04639	0.05417	G	0.6018	0.8495
1	rs12745968	93401837	G	0.04639	0.05417	A	0.6018	0.8495
1	rs3754112	117554421	G	0.09278	0.08333	A	0.6018	1.125
1	rs9426581	228033197	A	0.2655	0.28	G	0.6018	0.9293
2	rs6746030	167099158	A	0.04639	0.05417	G	0.6018	0.8495
2	rs920525	213986695	G	0.09278	0.08333	A	0.6018	1.125
3	rs2276812	52729960	A	0.09278	0.08333	G	0.6018	1.125
4	rs769242	122742217	A	0.04639	0.05417	G	0.6018	0.8495
5	rs10076911	37173930	C	0.04639	0.05417	A	0.6018	0.8495
8	exm2258811	27315936	G	0.04639	0.05417	A	0.6018	0.8495
8	rs55875819	27315936	G	0.04639	0.05417	A	0.6018	0.8495
9	rs3739602	95609564	A	0.2655	0.2808	G	0.6018	0.9255

10	rs3814171	15145569	G	0.04639	0.05417	A	0.6018	0.8495
13	rs1535692	95034749	A	0.2655	0.2808	G	0.6018	0.9255
14	rs17277522	21109141	G	0.2655	0.28	A	0.6018	0.9293
14	rs11622969	21109385	A	0.2655	0.28	G	0.6018	0.9293
2	rs10194138	29444656	G	0.2887	0.2742	A	0.602	1.074
3	rs9842389	173276758	G	0.2887	0.2742	A	0.602	1.074
5	rs10045328	16969392	A	0.04639	0.055	G	0.602	0.8359
5	rs979233	42375243	A	0.04639	0.055	C	0.602	0.8359
9	rs2229817	137726950	A	0.04639	0.055	G	0.602	0.8359
10	rs2271904	74894375	G	0.2887	0.275	A	0.6027	1.07
11	rs494606	116714118	C	0.1211	0.1325	A	0.6027	0.9024
15	rs4775031	58635583	A	0.1985	0.1867	C	0.6027	1.079
19	rs10405238	37488055	C	0.1985	0.1867	A	0.6027	1.079
21	rs77503870	33719343	G	0.1211	0.1325	C	0.6027	0.9024
4	rs7666425	517461	G	0.268	0.2833	A	0.6028	0.9263
4	rs34623004	527677	G	0.268	0.2833	A	0.6028	0.9263
4	rs1127410	533001	G	0.268	0.2833	A	0.6028	0.9263
12	rs16944514	115041895	A	0.268	0.2833	G	0.6028	0.9263
6	rs12193967	66204945	A	0.268	0.2825	G	0.6029	0.9301
15	rs7173947	95270467	G	0.268	0.2825	A	0.6029	0.9301
1	rs2289015	55075062	A	0.1804	0.1942	G	0.603	0.9136
6	rs2714337	7240577	T	0.1804	0.1942	A	0.603	0.9136
11	rs1059440	63991801	G	0.201	0.1883	A	0.6035	1.084
9	rs2061634	100105782	G	0.1392	0.1283	C	0.6036	1.098
9	rs4489420	139418260	A	0.1392	0.1283	G	0.6036	1.098
10	rs663263	27679816	A	0.2912	0.2775	C	0.6036	1.07
14	rs1952896	32021438	G	0.2912	0.2775	A	0.6036	1.07
16	rs7186852	30635659	G	0.09278	0.08417	A	0.6036	1.113
16	rs7197475	30642867	A	0.09278	0.08417	G	0.6036	1.113
16	rs1424114	69224615	A	0.2912	0.2775	G	0.6036	1.07
18	rs11151964	72201918	A	0.09278	0.08417	G	0.6036	1.113
19	rs16986718	56369314	A	0.2912	0.2775	G	0.6036	1.07
21	rs2823962	18046232	A	0.2912	0.2775	G	0.6036	1.07
1	rs11577579	179414105	C	0.05928	0.05167	A	0.6037	1.157
6	rs807534	24551664	G	0.05928	0.05167	A	0.6037	1.157
11	rs3741379	65408708	A	0.05928	0.05167	C	0.6037	1.157
15	rs2290559	42168400	A	0.05928	0.05167	G	0.6037	1.157
10	rs76806238	46999008	A	0.05928	0.05175	G	0.6039	1.155
22	rs2272829	38323700	A	0.05928	0.05175	G	0.6039	1.155
1	rs11587438	6631431	G	0.201	0.1892	A	0.6044	1.078
15	rs10520589	86010345	C	0.201	0.1892	A	0.6044	1.078
12	rs7312857	51707626	A	0.2732	0.2883	G	0.6048	0.9278
4	rs2624038	53165940	A	0.2964	0.2817	G	0.605	1.074

6	rs198823	26122933	A	0.2964	0.2817	C	0.605	1.074
7	rs11762428	35650563	A	0.2732	0.2875	G	0.605	0.9315
7	rs10268637	114074257	A	0.2732	0.2875	G	0.605	0.9315
16	rs6499886	57613658	A	0.2964	0.2817	G	0.605	1.074
18	rs11081251	674440	A	0.2732	0.2875	C	0.605	0.9315
20	rs6139030	3187733	G	0.1392	0.1292	A	0.6051	1.09
1	rs1287637	5935162	T	0.2036	0.1908	A	0.6052	1.084
17	rs9899177	5058808	A	0.1237	0.1359	G	0.6055	0.8976
5	rs3806873	5462607	G	0.2758	0.2908	A	0.6057	0.9285
5	rs3806874	5462620	A	0.2758	0.2908	G	0.6057	0.9285
6	rs7764472	35479574	C	0.1237	0.135	G	0.6057	0.9046
7	rs41275227	102098010	A	0.1237	0.135	G	0.6057	0.9046
8	rs4265150	129384895	C	0.2758	0.2908	A	0.6057	0.9285
14	rs2275462	51446216	A	0.2758	0.2908	C	0.6057	0.9285
14	rs72629402	92159558	G	0.1237	0.135	A	0.6057	0.9046
17	rs3744550	10206595	G	0.2758	0.2913	A	0.6057	0.9263
6	rs499818	13332467	A	0.1237	0.1358	G	0.6058	0.8982
6	rs2859448	31436273	G	0.08031	0.09	A	0.6059	0.8829
3	rs2272487	126451937	A	0.2758	0.29	C	0.606	0.9323
3	rs1008369	44105159	A	0.2036	0.1917	G	0.6061	1.078
5	rs2287780	7889304	A	0.2036	0.1917	G	0.6061	1.078
2	rs117569106	160239116	A	0.03351	0.02833	T	0.6063	1.189
3	rs137972331	171405373	A	0.03351	0.02833	G	0.6063	1.189
5	rs1866374	71490962	A	0.03351	0.02833	G	0.6063	1.189
6	rs41276062	54191686	A	0.03351	0.02833	G	0.6063	1.189
7	rs117722738	116146127	A	0.03351	0.02833	G	0.6063	1.189
11	rs10839531	6221169	A	0.03351	0.02833	G	0.6063	1.189
11	rs118081870	63067061	A	0.03351	0.02833	G	0.6063	1.189
6	rs3131024	29385237	A	0.299	0.285	G	0.6064	1.07
6	rs1633034	29743563	G	0.299	0.285	A	0.6064	1.07
6	rs1632983	29773509	A	0.299	0.285	G	0.6064	1.07
8	rs2116078	73363989	C	0.299	0.285	A	0.6064	1.07
1	rs2274616	145536082	A	0.09536	0.08583	G	0.6065	1.123
1	rs17615	207646462	A	0.1418	0.1308	G	0.6065	1.097
1	rs4308977	207646898	G	0.1418	0.1308	A	0.6065	1.097
7	rs1799806	100488658	G	0.1418	0.1308	C	0.6065	1.097
10	rs11005328	58120988	A	0.09536	0.08583	C	0.6065	1.123
11	rs4910742	5306509	G	0.09536	0.08583	A	0.6065	1.123
16	rs151181	28490517	G	0.1418	0.1308	A	0.6065	1.097
19	rs7255436	8433196	A	0.09536	0.08583	C	0.6065	1.123
1	rs12138950	19839115	C	0.1856	0.1992	A	0.6066	0.9162
10	exm812875	17869577	A	0.1856	0.1992	G	0.6066	0.9162
10	rs201810168	18116531	A	0.1856	0.1992	G	0.6066	0.9162

3	rs34769632	141497469	A	0.05928	0.0525	T	0.6067	1.137
11	rs1032189	98794453	G	0.2784	0.2933	A	0.6067	0.9292
7	rs1035042	52452724	A	0.3015	0.2867	G	0.6068	1.074
22	rs2074735	31535872	C	0.3015	0.2867	G	0.6068	1.074
14	rs4905366	96103099	G	0.2062	0.1933	A	0.607	1.084
21	rs79021510	47558528	A	0.03351	0.02848	G	0.6073	1.183
11	rs4945197	77378388	G	0.2062	0.1942	A	0.6078	1.078
1	rs17616	207646923	A	0.1418	0.1317	G	0.6079	1.089
1	rs10779513	209939599	A	0.2809	0.295	G	0.6079	0.9337
12	rs10878102	64434014	A	0.2809	0.295	G	0.6079	0.9337
15	rs6496589	90213229	C	0.2809	0.295	G	0.6079	0.9337
22	rs1018448	43206950	A	0.2809	0.295	C	0.6079	0.9337
10	rs2031577	4050003	A	0.3041	0.29	G	0.6082	1.07
12	rs7975356	29630081	G	0.09536	0.08667	A	0.6082	1.111
7	rs7493	95034775	G	0.1881	0.2008	C	0.6085	0.9222
7	rs12026	95041016	C	0.1881	0.2008	G	0.6085	0.9222
5	rs10380	7897191	A	0.1263	0.1375	G	0.6086	0.9067
8	rs2218488	72263956	G	0.1263	0.1375	A	0.6086	0.9067
9	rs16929332	12671693	G	0.2835	0.2983	A	0.6086	0.9306
9	rs1412829	22043926	G	0.1263	0.1375	A	0.6086	0.9067
10	rs10821936	63723577	G	0.2835	0.2992	A	0.6086	0.9269
21	rs2836048	39284298	G	0.2835	0.2983	A	0.6086	0.9306
8	rs2748416	142490120	C	0.2088	0.1958	A	0.6087	1.083
3	rs16833118	182132383	G	0.2835	0.2975	A	0.6089	0.9343
11	rs10893172	124095414	G	0.3067	0.2925	C	0.609	1.07
8	rs4585714	94915411	A	0.1443	0.1333	G	0.6093	1.096
10	rs3127679	46999190	A	0.04897	0.05734	G	0.6094	0.8466
1	rs3889814	23731819	G	0.2861	0.3017	A	0.6095	0.9276
3	rs6790878	193766273	G	0.2861	0.3008	A	0.6095	0.9313
4	rs76527646	169195109	A	0.2088	0.1967	C	0.6095	1.078
4	rs550625	169197297	A	0.2088	0.1967	G	0.6095	1.078
6	rs10498712	22743403	G	0.2861	0.3008	A	0.6095	0.9313
17	rs203462	19812541	G	0.2088	0.1967	A	0.6095	1.078
17	rs3760128	73886888	G	0.2861	0.3005	A	0.6095	0.9328
9	rs10989563	104385711	A	0.05959	0.05333	G	0.6096	1.125
1	rs10917051	22141206	A	0.3067	0.2933	C	0.6098	1.066
1	rs1128750	161069544	A	0.04897	0.05667	G	0.61	0.8572
2	rs7566476	71012734	C	0.04897	0.05667	G	0.61	0.8572
2	rs7576532	186670104	A	0.04897	0.0575	C	0.61	0.844
2	rs1468999	186697876	A	0.04897	0.0575	G	0.61	0.844
4	rs1550931	87809387	C	0.04897	0.0575	A	0.61	0.844
5	rs1422450	128040308	A	0.04897	0.05667	G	0.61	0.8572
8	rs3739308	12878982	G	0.04897	0.0575	A	0.61	0.844

8	rs16937976	72984041	C	0.04897	0.0575	G	0.61	0.844
9	rs76424583	115598610	G	0.04897	0.0575	A	0.61	0.844
10	rs990310	129347785	A	0.04897	0.0575	G	0.61	0.844
12	rs12366671	4736569	G	0.04897	0.05667	A	0.61	0.8572
12	rs79989395	15070187	G	0.04897	0.05667	A	0.61	0.8572
2	rs3754539	63626173	A	0.2887	0.3033	G	0.6104	0.932
3	rs36115950	64672474	A	0.08247	0.0925	T	0.6104	0.8819
5	rs10067427	99342047	G	0.2887	0.3042	A	0.6104	0.9283
6	rs2071593	31512799	A	0.08247	0.0925	G	0.6104	0.8819
7	rs4722064	21893993	A	0.2887	0.3042	C	0.6104	0.9283
7	rs11525060	148801091	G	0.08247	0.0925	A	0.6104	0.8819
12	rs11109058	97873725	C	0.08247	0.0925	A	0.6104	0.8819
13	rs2057413	50057097	G	0.08247	0.0925	A	0.6104	0.8819
15	rs4778138	28335820	A	0.2887	0.3033	G	0.6104	0.932
15	rs12915007	101110096	G	0.2113	0.1983	A	0.6104	1.083
17	rs61753060	650510	G	0.08247	0.0925	A	0.6104	0.8819
18	rs608433	74611127	A	0.08247	0.0925	G	0.6104	0.8819
5	rs10035612	442586	C	0.08247	0.09182	G	0.6105	0.8891
1	rs60891279	86512536	A	0.3093	0.2958	G	0.6106	1.066
6	rs1894411	32792973	G	0.1443	0.1342	A	0.6106	1.089
12	rs35577124	41900463	A	0.1443	0.1342	G	0.6106	1.089
4	rs2685471	81981592	G	0.3119	0.2975	A	0.6107	1.07
5	rs10035653	442790	A	0.08247	0.09167	G	0.6107	0.8907
6	rs3130379	30325531	A	0.08247	0.09167	G	0.6107	0.8907
4	rs10033464	111720761	A	0.2113	0.1992	C	0.6111	1.078
12	rs7972986	42481732	A	0.2113	0.1992	G	0.6111	1.078
1	rs78657023	74507450	C	0.06186	0.05417	G	0.6114	1.151
4	rs17316633	110825634	A	0.06186	0.05417	G	0.6114	1.151
19	rs45467802	19230816	G	0.06186	0.05417	A	0.6114	1.151
19	rs61743049	37005998	G	0.06186	0.05417	A	0.6114	1.151
8	rs7821565	52216762	G	0.1289	0.1408	A	0.6115	0.9025
13	rs41308564	109472733	A	0.1289	0.14	G	0.6115	0.9087
18	rs991014	42439886	A	0.3144	0.3	G	0.6115	1.07
1	rs41266136	152193162	A	0.06186	0.05426	G	0.6116	1.149
9	rs2240961	131019738	G	0.2135	0.2018	A	0.6116	1.074
2	rs11900586	67513152	A	0.09845	0.08917	G	0.6117	1.115
5	rs1800449	121413208	A	0.1933	0.2067	G	0.6118	0.9198
3	rs2121752	186296304	A	0.2139	0.2008	G	0.612	1.083
10	rs10786783	105882765	G	0.2139	0.2008	A	0.612	1.083
13	rs9582807	104780173	C	0.1933	0.2058	A	0.612	0.9245
17	rs11538927	26686416	A	0.1933	0.2058	G	0.612	0.9245
4	rs58225850	128949799	A	0.3144	0.3008	G	0.6121	1.066
4	rs35763794	128949850	G	0.3144	0.3008	A	0.6121	1.066

4	rs60505265	128949929	A	0.3144	0.3008	C	0.6121	1.066
6	rs443623	32996350	A	0.2938	0.3092	T	0.6121	0.9297
8	rs1481792	72089631	A	0.1469	0.1358	G	0.6121	1.096
12	rs4768556	44932106	A	0.3144	0.3008	G	0.6121	1.066
2	rs3811040	113498566	A	0.2938	0.3083	G	0.6122	0.9333
5	rs62370437	101575091	A	0.2938	0.308	G	0.6122	0.9347
15	rs4383118	92139237	A	0.317	0.3025	G	0.6123	1.07
5	rs1042714	148206473	C	0.09794	0.08917	G	0.6126	1.109
9	rs16935508	34311195	G	0.09794	0.08917	C	0.6126	1.109
10	rs1200875	37505192	A	0.09794	0.08917	G	0.6126	1.109
9	rs8176746	136131322	A	0.2139	0.2017	C	0.6127	1.077
18	rs12957347	57751014	G	0.2139	0.2017	A	0.6127	1.077
17	rs1443417	6493198	A	0.2964	0.3114	G	0.6128	0.9317
17	exm1284757	6511781	A	0.2964	0.3114	G	0.6128	0.9317
17	rs4796519	6511781	A	0.2964	0.3114	G	0.6128	0.9317
17	rs2289642	6515387	A	0.2964	0.3114	G	0.6128	0.9317
17	rs2289643	6515454	G	0.2964	0.3114	A	0.6128	0.9317
2	rs7562838	80698371	G	0.2964	0.3117	A	0.6129	0.9303
6	rs7760988	31273030	A	0.317	0.3033	G	0.6129	1.066
6	rs3129871	32406342	A	0.317	0.3033	C	0.6129	1.066
9	rs2066714	107586753	A	0.2964	0.3108	G	0.613	0.934
6	rs2239800	32713267	G	0.3196	0.305	A	0.6131	1.07
9	rs481905	215057	A	0.2964	0.3105	G	0.6131	0.9353
1	rs7555668	112856570	A	0.1469	0.1367	G	0.6132	1.088
5	rs56174528	74400516	G	0.1469	0.1367	C	0.6132	1.088
7	rs1076277	149489491	A	0.1469	0.1367	G	0.6132	1.088
9	rs147367410	113637741	G	0.02577	0.03177	A	0.6132	0.8062
15	rs1867380	58476281	A	0.1469	0.1367	G	0.6132	1.088
19	rs1549697	376008	A	0.1469	0.1367	G	0.6132	1.088
14	rs11159647	84775209	A	0.1969	0.2107	G	0.6133	0.9184
1	rs144390511	180904367	A	0.02577	0.0325	G	0.6134	0.7875
1	rs2243191	207015957	G	0.1959	0.2092	A	0.6134	0.921
3	rs3796191	125872384	G	0.02577	0.0325	A	0.6134	0.7875
3	rs117574050	137881275	G	0.02577	0.0325	A	0.6134	0.7875
4	rs9991339	187088248	A	0.02577	0.0325	G	0.6134	0.7875
14	rs10146906	24533539	A	0.02577	0.0325	C	0.6134	0.7875
16	rs6564851	81264597	A	0.1959	0.2092	C	0.6134	0.921
17	rs545652	72588806	A	0.02577	0.0325	C	0.6134	0.7875
22	rs16989495	32194581	G	0.02577	0.0325	A	0.6134	0.7875
22	rs117014418	36587404	A	0.02577	0.0325	G	0.6134	0.7875
9	rs75739279	117138846	A	0.02577	0.03167	C	0.6135	0.809
12	rs140234136	404774	A	0.02577	0.03167	C	0.6135	0.809
13	rs150932292	25484030	G	0.02577	0.03167	A	0.6135	0.809

16	rs78637028	3640784	A	0.02577	0.03167	G	0.6135	0.809
22	rs3213549	42950033	A	0.02577	0.03167	G	0.6135	0.809
10	rs7904946	120727619	G	0.2165	0.2033	A	0.6136	1.083
3	rs61268888	127642814	G	0.1959	0.2083	C	0.6137	0.9256
5	rs6890457	42949962	A	0.3222	0.3072	C	0.6137	1.072
6	rs2267637	30578335	A	0.299	0.3142	G	0.6137	0.931
9	rs886089	136199466	A	0.3196	0.3058	G	0.6137	1.066
10	rs716595	112006486	A	0.299	0.3142	G	0.6137	0.931
12	rs4980895	520947	C	0.299	0.3142	A	0.6137	0.931
14	rs4983413	105930406	G	0.299	0.3142	A	0.6137	0.931
7	rs6950127	28128410	A	0.3222	0.3075	G	0.6139	1.07
15	rs2470911	45047134	G	0.3222	0.3075	A	0.6139	1.07
18	rs8098398	26223745	G	0.299	0.3133	A	0.6139	0.9346
20	rs3746638	3675333	G	0.299	0.3133	A	0.6139	0.9346
1	rs12741518	152193605	A	0.06186	0.055	G	0.6142	1.133
1	rs62623409	152281745	A	0.06186	0.055	C	0.6142	1.133
3	rs146940902	39433037	G	0.06186	0.055	A	0.6142	1.133
9	rs12002901	90359962	G	0.1314	0.1433	A	0.6142	0.9045
10	rs6597801	126172863	A	0.06186	0.055	G	0.6142	1.133
17	rs34303409	72691263	A	0.06186	0.055	C	0.6142	1.133
20	rs2227946	47685320	G	0.06186	0.055	C	0.6142	1.133
7	rs542137	100373077	G	0.1314	0.1425	C	0.6143	0.9107
7	rs539445	100373367	G	0.1314	0.1425	C	0.6143	0.9107
12	rs34594498	40646786	A	0.01554	0.0125	G	0.6143	1.247
12	rs2239187	48117707	G	0.1314	0.1425	A	0.6143	0.9107
13	rs1805388	108863591	A	0.1314	0.1425	G	0.6143	0.9107
17	rs61736066	70645032	A	0.2165	0.2042	G	0.6143	1.077
22	rs140174	23922983	A	0.2165	0.2042	G	0.6143	1.077
13	rs9517320	99126303	C	0.3015	0.3167	A	0.6145	0.9316
4	rs1046994	90874879	A	0.1495	0.1383	G	0.6147	1.095
7	rs2080161	13331150	C	0.3015	0.3158	A	0.6147	0.9352
12	rs35493121	57390038	G	0.1495	0.1383	A	0.6147	1.095
13	rs1561859	88623302	A	0.3015	0.3158	G	0.6147	0.9352
16	rs3751803	86565826	C	0.1495	0.1383	G	0.6147	1.095
1	rs2798851	144859929	A	0.08505	0.09448	G	0.6149	0.8909
15	rs113463533	43552360	A	0.09794	0.09	G	0.6149	1.098
7	rs1007358	46201355	G	0.1985	0.2117	A	0.6151	0.9221
7	rs6949056	128359133	A	0.3247	0.3108	G	0.6151	1.066
17	rs4789659	72398336	G	0.1985	0.2117	A	0.6151	0.9221
3	rs6779254	108072298	G	0.1985	0.2125	A	0.6152	0.9175
18	rs12970134	57884750	A	0.2165	0.205	G	0.6153	1.072
1	rs2235541	24077451	A	0.08505	0.09583	G	0.6154	0.877
2	rs1196705	150219222	G	0.08505	0.09583	A	0.6154	0.877

4	rs357652	101353122	A	0.08505	0.09583	G	0.6154	0.877
6	rs7767310	77599333	A	0.3273	0.3125	C	0.6154	1.07
1	rs140554876	32264191	C	0.01546	0.0125	A	0.6155	1.241
1	rs34943764	227268705	G	0.01546	0.0125	A	0.6155	1.241
1	rs118122259	248202295	A	0.01546	0.0125	C	0.6155	1.241
2	rs137922460	209215654	A	0.01546	0.0125	G	0.6155	1.241
2	rs139713392	220424192	A	0.01546	0.0125	G	0.6155	1.241
2	rs150279473	220473882	A	0.01546	0.0125	G	0.6155	1.241
3	rs9872121	46140002	G	0.01546	0.0125	A	0.6155	1.241
3	rs146948408	167506993	G	0.01546	0.0125	A	0.6155	1.241
3	rs141885014	179096187	A	0.01546	0.0125	T	0.6155	1.241
5	rs202179572	35763699	C	0.01546	0.0125	G	0.6155	1.241
5	rs11750814	137500665	A	0.01546	0.0125	G	0.6155	1.241
6	rs77429005	24588899	G	0.01546	0.0125	A	0.6155	1.241
6	rs495089	32197463	G	0.3041	0.3183	A	0.6155	0.9359
6	rs147468160	148840728	C	0.01546	0.0125	G	0.6155	1.241
7	rs2385168	104078657	A	0.3041	0.3183	C	0.6155	0.9359
8	rs1631430	23786333	A	0.3041	0.3183	G	0.6155	0.9359
8	exm-IND8-10167	101608882	D	0.01546	0.0125	I	0.6155	1.241
11	rs78950816	4594535	G	0.01546	0.0125	C	0.6155	1.241
12	rs138005116	56230848	A	0.01546	0.0125	G	0.6155	1.241
12	rs117200049	124097777	A	0.01546	0.0125	G	0.6155	1.241
14	rs3814874	71199811	A	0.01546	0.0125	G	0.6155	1.241
14	rs200512221	94405819	A	0.01546	0.0125	G	0.6155	1.241
15	rs145012038	72637878	A	0.01546	0.0125	G	0.6155	1.241
16	rs79121865	55361727	C	0.01546	0.0125	A	0.6155	1.241
21	rs142956391	45108038	C	0.01546	0.0125	A	0.6155	1.241
21	rs78362277	45877078	A	0.01546	0.0125	G	0.6155	1.241
22	rs199559753	18300641	A	0.01546	0.0125	G	0.6155	1.241
22	rs1007298	24038847	A	0.3041	0.3183	G	0.6155	0.9359
22	rs9615362	46795141	A	0.01546	0.0125	C	0.6155	1.241
22	rs139023197	50969647	G	0.01546	0.0125	C	0.6155	1.241
2	rs55677134	179439490	A	0.03608	0.03	G	0.6156	1.21
2	rs111231885	238277211	A	0.03608	0.03	G	0.6156	1.21
4	rs146683175	107845819	A	0.03608	0.03	G	0.6156	1.21
6	rs3815101	30514810	A	0.03608	0.03	G	0.6156	1.21
6	rs3888778	30529622	A	0.03608	0.03	G	0.6156	1.21
6	rs7742650	30573334	G	0.03608	0.03	A	0.6156	1.21
6	rs2302347	30587613	A	0.03608	0.03	G	0.6156	1.21
6	rs34315095	30593485	G	0.03608	0.03	C	0.6156	1.21
6	rs6904236	30596135	G	0.03608	0.03	A	0.6156	1.21
6	rs2428514	31027516	A	0.03608	0.03	G	0.6156	1.21
7	rs79397821	150171165	A	0.03608	0.03	G	0.6156	1.21

8	rs2229126	26627672	T	0.03608	0.03	A	0.6156	1.21
11	rs78466420	6524126	G	0.03608	0.03	A	0.6156	1.21
12	rs147472788	62946739	C	0.03608	0.03	A	0.6156	1.21
14	rs77115877	61180693	T	0.03608	0.03	A	0.6156	1.21
15	rs2233070	23889739	A	0.03608	0.03	C	0.6156	1.21
15	rs3784543	79606186	A	0.03608	0.03	G	0.6156	1.21
20	rs144884167	16385494	A	0.03608	0.03	G	0.6156	1.21
14	rs142609376	94546058	G	0.03608	0.03005	A	0.6157	1.208
6	rs137902889	31895903	A	0.01546	0.01252	G	0.6159	1.239
9	rs886090	136199503	A	0.3299	0.315	G	0.6162	1.071
8	rs4244612	145741702	C	0.3067	0.3208	G	0.6163	0.9365
6	rs1737043	29736047	A	0.3299	0.3158	G	0.6166	1.066
19	rs1052450	50479999	G	0.3299	0.3158	A	0.6166	1.066
12	rs10846934	126126340	G	0.201	0.2142	A	0.6167	0.9232
19	rs377993	45818628	G	0.201	0.2142	A	0.6167	0.9232
1	rs151295225	241964468	G	0.1005	0.09167	A	0.6168	1.107
7	exm2137387	117865028	A	0.1005	0.09167	C	0.6168	1.107
7	rs10487391	117865028	A	0.1005	0.09167	G	0.6168	1.107
16	rs4843747	87991051	A	0.201	0.215	C	0.6168	0.9187
19	exm-IND19-6284	58151339	D	0.201	0.215	I	0.6168	0.9187
1	rs1060575	67236097	T	0.134	0.1458	A	0.6169	0.9065
1	rs3816989	67242087	A	0.134	0.1458	G	0.6169	0.9065
7	rs7385804	100235970	C	0.134	0.1458	A	0.6169	0.9065
7	rs3931814	48349647	A	0.134	0.145	G	0.617	0.9126
5	rs321776	55407542	A	0.3093	0.3233	G	0.6171	0.9371
11	rs2511841	119206375	G	0.3093	0.3233	A	0.6171	0.9371
1	rs7550295	9804693	A	0.1521	0.1408	G	0.6173	1.094
2	rs2241883	88424066	G	0.2216	0.2092	A	0.6173	1.077
4	rs6818066	14848030	A	0.3325	0.3183	C	0.6173	1.067
9	rs8176742	136131437	A	0.2216	0.2092	G	0.6173	1.077
9	rs8176740	136131472	T	0.2216	0.2092	A	0.6173	1.077
20	rs2273506	61990939	A	0.1521	0.1408	G	0.6173	1.094
21	rs117931394	47552386	A	0.1521	0.1408	G	0.6173	1.094
5	rs79702707	74400151	C	0.05155	0.06	A	0.6175	0.8514
5	rs78756596	74400400	G	0.05155	0.06	A	0.6175	0.8514
5	rs11557637	167924260	A	0.05155	0.0601	C	0.6175	0.8499
7	rs57109564	47925525	A	0.05155	0.06	C	0.6175	0.8514
11	rs7103246	50367359	G	0.1521	0.1411	A	0.6175	1.092
12	rs117866676	123342706	A	0.05155	0.06	G	0.6175	0.8514
22	rs12484074	41752747	A	0.05155	0.06	G	0.6175	0.8514
1	rs76011927	47603188	A	0.05155	0.05917	G	0.6176	0.8642
6	rs886398	29839268	A	0.05155	0.05917	G	0.6176	0.8642
6	rs2523807	29850274	G	0.05155	0.05917	A	0.6176	0.8642

6	rs2234028	42713274	A	0.05155	0.05917	G	0.6176	0.8642
6	rs2234027	42713307	A	0.05155	0.05917	G	0.6176	0.8642
7	rs2968863	150623137	A	0.05155	0.05917	G	0.6176	0.8642
8	rs16933182	68074137	A	0.05155	0.05917	G	0.6176	0.8642
15	rs79042205	72455757	C	0.05155	0.05917	G	0.6176	0.8642
16	rs841227	4734513	G	0.3119	0.3267	A	0.6176	0.9341
16	rs76327478	76461474	A	0.05155	0.05917	G	0.6176	0.8642
22	rs80068543	19969043	A	0.05263	0.06083	G	0.6181	0.8577
1	rs41269993	160968609	A	0.2216	0.21	G	0.6182	1.071
5	rs16903965	38412674	A	0.2036	0.2167	G	0.6182	0.9243
9	rs76003300	90503313	C	0.2216	0.21	G	0.6182	1.071
9	rs8176745	136131347	A	0.2216	0.21	G	0.6182	1.071
3	rs75410160	129130087	A	0.1521	0.1417	G	0.6183	1.087
4	rs9328764	2176454	A	0.3144	0.33	G	0.6183	0.9312
4	rs10011549	2194946	A	0.3144	0.33	G	0.6183	0.9312
4	rs10018786	2195024	C	0.3144	0.33	A	0.6183	0.9312
17	rs4312356	36493203	A	0.1521	0.1417	G	0.6183	1.087
1	rs6671466	230656763	C	0.3351	0.3217	A	0.6186	1.063
5	rs116364	6699224	A	0.3351	0.3217	G	0.6186	1.063
3	rs189015907	51977419	A	0.06443	0.05667	G	0.6187	1.146
5	rs6883562	7835555	G	0.06443	0.05667	C	0.6187	1.146
5	rs2974617	114462355	G	0.06443	0.05667	A	0.6187	1.146
6	rs13202830	154578345	A	0.06443	0.05667	G	0.6187	1.146
11	rs11604671	113268059	A	0.06443	0.05667	G	0.6187	1.146
11	rs2734849	113270160	G	0.06443	0.05667	A	0.6187	1.146
19	rs4644955	55738634	A	0.06443	0.05667	G	0.6187	1.146
1	rs10158309	211813550	A	0.1005	0.0925	G	0.619	1.096
4	rs1381657	104061993	C	0.1005	0.0925	G	0.619	1.096
6	rs2395175	32405026	A	0.1005	0.0925	G	0.619	1.096
12	rs2072374	6637845	A	0.317	0.3325	G	0.619	0.9318
14	rs12894832	50855257	G	0.317	0.3317	A	0.6191	0.9353
5	rs12515688	140480852	A	0.08763	0.0975	G	0.6192	0.889
10	rs11000217	73964243	C	0.08763	0.0975	G	0.6192	0.889
3	rs2303983	122630346	G	0.3376	0.3242	A	0.6193	1.063
11	rs1940475	102593248	A	0.3376	0.3242	G	0.6193	1.063
2	rs16831235	135745129	A	0.1366	0.1483	G	0.6195	0.9084
15	rs938609	89398631	T	0.1366	0.1483	A	0.6195	0.9084
2	rs2241985	239049540	A	0.3402	0.3264	G	0.6196	1.064
3	rs293813	191097966	A	0.3196	0.335	G	0.6197	0.9324
10	rs11558719	73972957	A	0.08763	0.09833	G	0.6197	0.8807
11	rs2273549	33079580	G	0.08763	0.09833	A	0.6197	0.8807
12	rs1463605	30113883	G	0.1366	0.1475	C	0.6197	0.9144
12	rs4272850	129153986	A	0.2242	0.2125	G	0.6197	1.071

17	rs377963	42158310	A	0.08763	0.09833	C	0.6197	0.8807
19	rs3803915	2160529	A	0.2242	0.2125	C	0.6197	1.071
2	rs1367158	16590965	G	0.2062	0.2192	A	0.6198	0.9254
2	rs3183099	220427395	A	0.1546	0.1433	G	0.6198	1.093
6	rs3873283	29933000	G	0.1546	0.1433	A	0.6198	1.093
6	rs3130320	32223258	A	0.2062	0.2192	G	0.6198	0.9254
8	rs7843884	122318550	C	0.1546	0.1433	A	0.6198	1.093
20	rs2427283	60899206	G	0.2062	0.2192	A	0.6198	0.9254
21	rs15736	44273858	A	0.1546	0.1433	G	0.6198	1.093
16	rs13337278	700644	C	0.3402	0.3267	G	0.6199	1.063
1	rs75881102	6615469	C	0.03608	0.03083	G	0.62	1.177
1	rs150674974	112019467	A	0.03608	0.03083	G	0.62	1.177
2	rs1801702	21225485	C	0.03608	0.03083	G	0.62	1.177
2	rs74589926	43903262	A	0.03608	0.03083	C	0.62	1.177
2	rs62840660	45171842	A	0.03608	0.03083	G	0.62	1.177
7	rs73407505	91659259	G	0.03608	0.03083	A	0.62	1.177
8	rs1037699	103250930	A	0.03608	0.03083	G	0.62	1.177
12	rs11836060	80750659	A	0.03608	0.03083	G	0.62	1.177
12	rs11114416	80761411	A	0.03608	0.03083	C	0.62	1.177
13	rs17075482	51826044	G	0.03608	0.03083	C	0.62	1.177
15	rs201340864	40655827	G	0.03608	0.03083	A	0.62	1.177
5	rs12153202	23533746	G	0.3222	0.3375	A	0.6204	0.933
2	rs6710409	122608702	A	0.3428	0.3292	C	0.6205	1.063
2	rs6734978	213858069	G	0.3428	0.3292	A	0.6205	1.063
3	rs9872799	184428903	C	0.3428	0.3292	A	0.6205	1.063
6	rs1736981	29770322	A	0.3222	0.3367	G	0.6205	0.9365
8	rs4840502	10468172	G	0.3428	0.3292	A	0.6205	1.063
8	rs1835740	98166913	G	0.3222	0.3367	A	0.6205	0.9365
1	rs6695965	147060091	G	0.3454	0.3308	A	0.6206	1.067
9	rs2236547	214864	A	0.3454	0.3308	G	0.6206	1.067
14	rs3783941	81575005	A	0.3264	0.3414	C	0.6207	0.9349
6	rs3132468	31475486	G	0.1546	0.1442	A	0.6208	1.086
9	rs2807580	126926107	G	0.1546	0.1442	A	0.6208	1.086
2	rs6735620	38677471	C	0.3247	0.34	A	0.621	0.9335
2	rs1130609	10262920	C	0.3454	0.3317	A	0.6211	1.063
5	rs17137481	114469724	G	0.3454	0.3317	A	0.6211	1.063
14	rs17832998	59112732	A	0.2268	0.215	G	0.6211	1.071
2	rs61744963	207615724	A	0.06443	0.0575	G	0.6212	1.129
3	rs2249551	192125768	A	0.3479	0.3333	G	0.6212	1.067
16	rs8052157	21154385	G	0.3479	0.3336	A	0.6212	1.066
21	rs12053674	32119495	C	0.3247	0.3392	G	0.6212	0.937
6	rs3800229	108996963	C	0.2088	0.2225	A	0.6213	0.922
20	rs910397	32295541	G	0.2088	0.2225	A	0.6213	0.922

8	rs11136300	144124609	A	0.2294	0.2166	G	0.6214	1.077
1	rs3738814	17331676	A	0.3273	0.3425	G	0.6217	0.9341
4	rs11100128	158620303	G	0.2294	0.2167	A	0.6217	1.076
14	rs11159491	81572939	G	0.3273	0.3417	A	0.6218	0.9376
14	exm1118722	81574959	G	0.3273	0.3417	A	0.6218	0.9376
14	rs3783942	81574959	G	0.3273	0.3417	A	0.6218	0.9376
15	rs8042868	43939642	A	0.3273	0.3417	G	0.6218	0.9376
16	rs12929500	10692832	C	0.3505	0.3358	A	0.6218	1.067
1	rs11120616	215916563	A	0.1392	0.1508	G	0.622	0.9102
7	rs2715094	50730452	G	0.1392	0.1508	A	0.622	0.9102
15	rs16977195	86984240	G	0.1392	0.1508	A	0.622	0.9102
1	rs144340720	15953261	G	0.01031	0.015	A	0.6221	0.684
1	rs200985789	16065144	G	0.01031	0.015	A	0.6221	0.684
1	rs117344991	16259454	A	0.01031	0.015	G	0.6221	0.684
1	rs148103384	48703362	C	0.01031	0.015	A	0.6221	0.684
1	rs147324255	95305504	A	0.01031	0.015	C	0.6221	0.684
1	rs201975846	203467929	A	0.01031	0.015	C	0.6221	0.684
1	rs77789819	203690406	A	0.01031	0.015	G	0.6221	0.684
1	rs1614866	211700658	A	0.01031	0.015	G	0.6221	0.684
1	rs62617126	231488952	T	0.01031	0.015	A	0.6221	0.684
1	rs201216518	247267413	A	0.01031	0.015	C	0.6221	0.684
1	rs117287351	247582310	A	0.01031	0.015	G	0.6221	0.684
2	rs182081324	30974108	A	0.01031	0.015	G	0.6221	0.684
3	rs145045076	1414127	G	0.01031	0.015	A	0.6221	0.684
3	rs148953234	48465168	G	0.01031	0.015	A	0.6221	0.684
3	rs80279768	66463340	G	0.01031	0.015	A	0.6221	0.684
3	rs76877671	93598157	G	0.01031	0.01503	A	0.6221	0.6829
3	rs76749982	97806305	C	0.01031	0.015	G	0.6221	0.684
3	rs142744559	164906269	A	0.01031	0.015	G	0.6221	0.684
5	rs115565444	140867087	A	0.01031	0.015	G	0.6221	0.684
6	rs2814993	34618893	A	0.01031	0.015	G	0.6221	0.684
7	rs145787430	73279405	G	0.01031	0.015	A	0.6221	0.684
7	rs186148498	91731945	A	0.01031	0.015	G	0.6221	0.684
10	rs45596231	5791578	A	0.01031	0.015	C	0.6221	0.684
11	rs76718681	69933891	A	0.01031	0.015	G	0.6221	0.684
12	rs12305672	29614807	A	0.01031	0.015	G	0.6221	0.684
12	rs187986863	29649557	A	0.01031	0.015	G	0.6221	0.684
14	rs10139784	88904567	A	0.01031	0.015	G	0.6221	0.684
15	exm1145538	33855175	A	0.01031	0.015	G	0.6221	0.684
15	rs16972317	33855175	A	0.01031	0.015	G	0.6221	0.684
15	rs189323864	41228746	A	0.01031	0.015	G	0.6221	0.684
15	rs199924130	77407280	A	0.01031	0.015	G	0.6221	0.684
16	rs117969741	2979984	A	0.01031	0.015	G	0.6221	0.684

16	rs77056932	22130242	G	0.01031	0.015	A	0.6221	0.684
16	rs76023376	89799804	C	0.01031	0.015	A	0.6221	0.684
17	rs116885661	5329392	A	0.01031	0.015	C	0.6221	0.684
17	rs78848036	42239292	G	0.01031	0.015	A	0.6221	0.684
20	rs138855546	39974573	A	0.01031	0.015	G	0.6221	0.684
22	rs144291562	50315361	A	0.01031	0.015	G	0.6221	0.684
4	rs10009228	40356422	A	0.1392	0.15	G	0.6222	0.9162
1	rs943762	177908852	A	0.3299	0.345	G	0.6223	0.9347
3	rs820470	123362856	A	0.3299	0.345	G	0.6223	0.9347
9	rs1536929	125391369	A	0.3299	0.345	G	0.6223	0.9347
14	rs398607	88407888	G	0.1572	0.1458	A	0.6223	1.093
14	rs17097718	99182535	A	0.1572	0.1458	G	0.6223	1.093
17	rs3744372	33802156	C	0.1572	0.1458	A	0.6223	1.093
1	rs4650754	188071966	G	0.3531	0.3383	A	0.6224	1.067
6	rs2477307	88541622	A	0.3531	0.3383	G	0.6224	1.067
2	rs201874641	211421452	A	0.3299	0.3442	T	0.6225	0.9381
16	rs2278023	81077016	G	0.06443	0.05789	A	0.6225	1.121
17	rs2869836	63333704	G	0.3299	0.3442	A	0.6225	0.9381
19	rs3745601	10224548	A	0.3299	0.3442	G	0.6225	0.9381
1	rs8191981	154941263	A	0.1031	0.095	G	0.623	1.095
2	rs2540060	201560331	G	0.1031	0.095	A	0.623	1.095
5	rs2472647	140710705	G	0.1572	0.1467	A	0.6232	1.085
7	rs7794503	31048898	G	0.09021	0.1	A	0.6234	0.8924
15	rs2303578	44849698	A	0.09021	0.1	G	0.6234	0.8924
15	rs8028537	89345947	A	0.09021	0.1	G	0.6234	0.8924
3	rs1909521	25063595	A	0.3582	0.3433	G	0.6236	1.068
3	rs6782299	180550702	C	0.09021	0.1008	A	0.6237	0.8842
7	rs2291569	128488734	A	0.09021	0.1008	G	0.6237	0.8842
11	rs17099008	102482504	C	0.09021	0.1008	A	0.6237	0.8842
1	rs6704425	38875991	A	0.232	0.22	G	0.6238	1.071
3	rs2255543	120389316	A	0.3351	0.3492	T	0.6238	0.9392
11	rs1047730	10536298	G	0.232	0.22	A	0.6238	1.071
16	rs455527	89644001	G	0.3351	0.3492	A	0.6238	0.9392
19	rs3745193	17451981	A	0.3582	0.3442	G	0.624	1.064
13	rs9561921	96293631	A	0.2139	0.2275	G	0.6241	0.924
18	rs8099021	3176040	G	0.2139	0.2275	C	0.6241	0.924
5	rs1530498	13902220	A	0.2139	0.2267	G	0.6242	0.9284
17	rs4796089	33814758	G	0.2139	0.2267	A	0.6242	0.9284
15	rs2279482	29416901	G	0.2345	0.2217	A	0.6244	1.076
3	rs4279104	69422051	G	0.3376	0.3517	A	0.6245	0.9397
17	rs166613	47943461	C	0.1418	0.1533	A	0.6245	0.912
1	rs7538876	17722363	A	0.1572	0.1475	G	0.6246	1.078
1	rs1025806	47139103	A	0.3608	0.3467	G	0.6246	1.064

1	rs10789501	47609489	A	0.05412	0.0625	G	0.6246	0.8583
1	rs1298954	145730160	G	0.3608	0.3467	A	0.6246	1.064
11	rs3794084	12257789	G	0.05412	0.0625	C	0.6246	0.8583
14	rs13306254	24976657	A	0.01036	0.01583	C	0.6246	0.6509
16	rs1948632	75991446	G	0.1572	0.1475	A	0.6246	1.078
20	rs214803	2290333	C	0.05412	0.0625	A	0.6246	0.8583
7	rs41281692	120776097	G	0.1418	0.1542	C	0.6247	0.9062
16	rs217180	72184629	A	0.1418	0.1542	G	0.6247	0.9062
1	rs144498730	12198362	A	0.01031	0.01583	C	0.6248	0.6475
1	rs61747285	210004332	A	0.05412	0.06167	G	0.6248	0.8707
2	rs3762578	11751118	A	0.01031	0.01583	G	0.6248	0.6475
2	rs2163649	166781158	A	0.05412	0.06167	G	0.6248	0.8707
2	rs116342827	192700912	A	0.01031	0.01583	G	0.6248	0.6475
2	rs117420067	202352433	A	0.01031	0.01583	G	0.6248	0.6475
2	rs114596320	238243350	A	0.01031	0.01583	G	0.6248	0.6475
3	rs76884309	101576143	G	0.01031	0.01583	A	0.6248	0.6475
3	rs1801726	122003832	C	0.01031	0.01583	G	0.6248	0.6475
3	rs9818870	138122122	A	0.01031	0.01583	G	0.6248	0.6475
4	rs4634233	37445315	A	0.05412	0.06167	C	0.6248	0.8707
4	rs76674380	57261656	A	0.01031	0.01583	C	0.6248	0.6475
5	rs12110273	4288545	G	0.3402	0.355	A	0.6248	0.9368
5	rs116939630	60050522	A	0.01031	0.01583	G	0.6248	0.6475
6	rs2814944	34552797	A	0.01031	0.01583	G	0.6248	0.6475
6	rs78849281	38980087	A	0.01031	0.01583	G	0.6248	0.6475
7	rs4720951	810219	G	0.3402	0.355	A	0.6248	0.9368
7	rs143118835	80141152	A	0.01031	0.01583	G	0.6248	0.6475
7	rs144484895	92019330	G	0.01031	0.01583	A	0.6248	0.6475
7	rs10224002	151415041	G	0.05412	0.06167	A	0.6248	0.8707
8	rs78267388	17917088	G	0.01031	0.01583	C	0.6248	0.6475
8	rs77969734	30949398	C	0.01031	0.01583	G	0.6248	0.6475
8	rs143808869	144875154	A	0.01031	0.01583	G	0.6248	0.6475
9	rs41277120	125273435	A	0.3634	0.3483	G	0.6248	1.068
9	rs201379007	132630668	C	0.01031	0.01583	G	0.6248	0.6475
10	rs10995190	64278682	A	0.01031	0.01583	G	0.6248	0.6475
10	rs149833441	64968251	G	0.01031	0.01583	A	0.6248	0.6475
10	rs76850691	101157438	C	0.01031	0.01583	G	0.6248	0.6475
11	rs186242535	20982082	C	0.01031	0.01583	G	0.6248	0.6475
11	rs143381179	55681201	A	0.01031	0.01583	C	0.6248	0.6475
12	rs61745251	81528625	A	0.05412	0.06167	G	0.6248	0.8707
14	rs114591848	21550212	A	0.01031	0.01583	G	0.6248	0.6475
14	rs76804017	73719407	A	0.01031	0.01583	G	0.6248	0.6475
16	rs2233531	10837913	G	0.01031	0.01583	C	0.6248	0.6475
17	rs406446	10614442	G	0.3402	0.3548	A	0.6248	0.9378

17	rs10853110	47318414	G	0.3402	0.355	A	0.6248	0.9368
19	rs190473107	648401	A	0.01031	0.01583	G	0.6248	0.6475
19	rs201540389	2807749	A	0.01031	0.01583	G	0.6248	0.6475
19	rs148018996	4695485	G	0.01031	0.01583	C	0.6248	0.6475
19	rs111476305	11287353	A	0.01031	0.01583	G	0.6248	0.6475
19	rs140193183	13035522	A	0.01031	0.01583	C	0.6248	0.6475
19	rs3760791	48846608	A	0.01031	0.01583	C	0.6248	0.6475
19	rs564196	49376582	A	0.05412	0.06167	G	0.6248	0.8707
20	rs35655964	746197	C	0.01031	0.01583	G	0.6248	0.6475
20	rs1800961	43042364	A	0.01031	0.01583	G	0.6248	0.6475
20	rs80097864	60903041	A	0.01031	0.01583	G	0.6248	0.6475
3	rs9866968	85683470	G	0.3634	0.3489	A	0.6249	1.065
7	rs17151689	76950686	A	0.1057	0.09667	C	0.6249	1.104
3	rs35070271	182897920	G	0.3402	0.3542	A	0.6251	0.9403
13	rs2275660	25029218	G	0.3634	0.3492	A	0.6251	1.064
18	rs4797155	4261884	A	0.2345	0.2225	G	0.6251	1.071
18	rs618662	40972892	A	0.3634	0.3492	C	0.6251	1.064
5	rs27047	1412251	A	0.3428	0.3583	G	0.6253	0.934
6	rs970547	75797302	G	0.3428	0.3575	A	0.6254	0.9374
6	rs6919947	126210395	A	0.3428	0.3575	C	0.6254	0.9374
12	rs4244857	27940158	A	0.3428	0.3575	G	0.6254	0.9374
17	rs7813	648186	G	0.3634	0.3497	A	0.6254	1.061
1	rs4648	201934578	A	0.06701	0.05917	G	0.6256	1.142
4	rs12648093	123838758	A	0.06701	0.05917	G	0.6256	1.142
6	rs6904029	29943067	A	0.366	0.3517	G	0.6256	1.064
8	rs62000450	143396446	A	0.06701	0.05917	G	0.6256	1.142
11	rs2234301	2334893	A	0.06701	0.05917	G	0.6256	1.142
11	rs1995158	5566030	A	0.06701	0.05917	G	0.6256	1.142
11	rs1566275	5566365	A	0.06701	0.05917	G	0.6256	1.142
19	rs116953419	19121086	A	0.06701	0.05917	G	0.6256	1.142
20	rs237743	47903019	A	0.06701	0.05917	G	0.6256	1.142
22	rs11705259	21990823	A	0.06701	0.05917	G	0.6256	1.142
3	rs13095016	195515594	C	0.2165	0.2292	G	0.6257	0.9294
6	rs3765467	39033595	A	0.2383	0.2257	G	0.6257	1.074
10	rs11254413	17204187	A	0.2165	0.2292	G	0.6257	0.9294
12	rs2240871	6639981	G	0.3428	0.3567	C	0.6257	0.9408
22	rs3747151	31333631	A	0.3428	0.3567	G	0.6257	0.9408
2	rs1250914	224228654	G	0.2371	0.2242	A	0.6258	1.076
7	rs2302445	156761818	A	0.2371	0.2242	G	0.6258	1.076
7	rs12919	156762248	A	0.2371	0.2242	G	0.6258	1.076
3	rs4328821	128316435	G	0.3454	0.3608	A	0.6259	0.9345
11	rs1441519	25929959	A	0.3454	0.3608	C	0.6259	0.9345
19	rs3813135	15587345	G	0.3454	0.3608	A	0.6259	0.9345

4	rs13110927	169299528	A	0.3454	0.36	G	0.626	0.9379
7	rs11770052	73275565	T	0.3454	0.36	A	0.626	0.9379
11	rs4936881	123887164	C	0.3832	0.3983	A	0.626	0.9382
6	rs927332	6331873	A	0.3686	0.3542	G	0.6261	1.064
12	rs10735979	74705055	A	0.3686	0.3542	G	0.6261	1.064
3	rs1996483	166124934	G	0.366	0.3525	A	0.6262	1.06
12	rs829881	98881228	A	0.366	0.3525	C	0.6262	1.06
13	rs568772	75923963	C	0.3479	0.3633	A	0.6264	0.935
20	rs1555286	24118582	G	0.3479	0.3633	A	0.6264	0.935
1	rs13306014	71440052	G	0.03608	0.03167	A	0.6266	1.145
4	rs146378981	77317630	A	0.03608	0.03167	G	0.6266	1.145
5	rs147458082	1201806	A	0.03608	0.03167	G	0.6266	1.145
5	rs74460472	71757266	G	0.03608	0.03167	A	0.6266	1.145
7	rs10277516	22908774	A	0.03608	0.03167	C	0.6266	1.145
12	rs1025016	70091452	G	0.03608	0.03167	A	0.6266	1.145
14	rs2273906	102898363	A	0.03608	0.03167	G	0.6266	1.145
15	rs150003957	69325606	C	0.03608	0.03167	A	0.6266	1.145
19	rs67712849	16387640	G	0.03608	0.03167	A	0.6266	1.145
19	rs9807853	52376172	A	0.03608	0.03167	T	0.6266	1.145
19	rs9807842	52376207	A	0.03608	0.03167	G	0.6266	1.145
19	rs34295271	52376434	G	0.03608	0.03167	A	0.6266	1.145
6	rs7773075	110680744	A	0.3711	0.3567	C	0.6267	1.065
7	rs13235516	130923681	G	0.3711	0.3567	A	0.6267	1.065
2	rs13003464	61186829	G	0.02835	0.035	A	0.6268	0.8045
7	rs10245778	142750147	A	0.02835	0.035	G	0.6268	0.8045
9	rs139272916	134371163	C	0.02835	0.035	G	0.6268	0.8045
10	rs7474896	37982097	A	0.1057	0.0975	G	0.6268	1.094
16	rs4782812	83563611	C	0.1057	0.0975	A	0.6268	1.094
17	rs13290	7329632	C	0.02835	0.035	A	0.6268	0.8045
17	rs13306788	42338136	A	0.02835	0.035	C	0.6268	0.8045
17	rs2011951	42398052	A	0.1057	0.0975	G	0.6268	1.094
20	rs75442118	126149	G	0.1057	0.0975	A	0.6268	1.094
22	rs2269619	41257877	C	0.02835	0.035	A	0.6268	0.8045
1	rs2486668	24658063	G	0.1443	0.1558	C	0.6269	0.9137
3	rs155409	1355266	A	0.1443	0.1558	G	0.6269	0.9137
3	rs75218075	167320010	G	0.1598	0.15	A	0.6269	1.078
11	rs1050239	6415463	A	0.1443	0.1558	G	0.6269	0.9137
13	rs17076657	32371361	G	0.2191	0.2325	A	0.6269	0.926
22	rs6972	43558972	A	0.2191	0.2325	G	0.6269	0.926
1	rs78237904	109365991	A	0.03608	0.03172	G	0.627	1.143
6	rs1632880	29917610	C	0.06736	0.06	G	0.627	1.131
11	rs2512344	86835374	A	0.3505	0.3658	G	0.627	0.9355
2	rs12233132	25328703	G	0.2191	0.2317	A	0.6271	0.9304

3	rs6793735	142408606	A	0.3505	0.365	G	0.6271	0.9389
10	rs3818876	99498234	A	0.3505	0.365	G	0.6271	0.9389
12	rs10842753	26697411	G	0.2397	0.2267	A	0.6271	1.076
12	rs11540407	75900382	A	0.1443	0.1567	G	0.6271	0.908
15	rs938608	89398605	A	0.1443	0.1567	C	0.6271	0.908
22	rs3827318	23974200	G	0.2397	0.2267	A	0.6271	1.076
3	rs36092077	183753777	A	0.3711	0.3575	G	0.6272	1.061
17	rs17558560	38936659	G	0.3737	0.3592	A	0.6272	1.065
2	rs2298771	166892788	G	0.09278	0.1025	A	0.6274	0.8955
3	rs4450776	111423129	G	0.09278	0.1025	C	0.6274	0.8955
4	rs10516487	102751076	A	0.09278	0.1025	G	0.6274	0.8955
16	rs71389465	30380872	C	0.09278	0.1025	G	0.6274	0.8955
3	rs9873067	122649522	G	0.3737	0.36	A	0.6276	1.061
5	rs3815768	95236459	A	0.3737	0.36	G	0.6276	1.061
8	rs967851	126984970	C	0.3531	0.3675	A	0.6276	0.9394
10	rs12722489	6102012	A	0.09278	0.1033	G	0.6276	0.8875
10	rs7909329	58077694	G	0.3763	0.3617	A	0.6276	1.065
16	rs8059048	88781040	A	0.3763	0.3617	G	0.6276	1.065
17	rs3803884	48919039	A	0.3737	0.36	G	0.6276	1.061
1	rs291353	235720898	G	0.1624	0.1517	A	0.6278	1.084
6	rs79792575	30920086	A	0.06701	0.06	G	0.6278	1.125
15	rs3743266	60781513	G	0.1624	0.1517	A	0.6278	1.084
19	rs77267061	9449888	G	0.06701	0.06	A	0.6278	1.125
21	rs3827183	37661405	A	0.1624	0.1517	G	0.6278	1.084
3	rs1304002	26562597	G	0.3557	0.3708	A	0.628	0.9365
6	rs3132454	31489644	G	0.3557	0.3708	A	0.628	0.9365
1	rs5253	16380196	A	0.2216	0.2345	G	0.6281	0.9296
3	rs442800	137786442	A	0.3789	0.3642	G	0.6281	1.065
3	rs2724697	137798155	G	0.3789	0.3642	A	0.6281	1.065
5	rs11959209	16060510	G	0.3763	0.3625	A	0.6281	1.061
6	rs2517840	29826092	A	0.3763	0.3625	G	0.6281	1.061
6	rs2523822	29828660	G	0.3763	0.3625	A	0.6281	1.061
10	rs756322	72744630	G	0.3789	0.3642	A	0.6281	1.065
1	rs4268394	77295109	A	0.3557	0.37	G	0.6282	0.9399
2	rs1357136	213103721	A	0.2216	0.235	G	0.6282	0.927
5	rs702640	53256679	A	0.3557	0.37	C	0.6282	0.9399
9	rs2066715	107588033	A	0.3557	0.37	G	0.6282	0.9399
19	rs35026308	14153293	G	0.2216	0.235	A	0.6282	0.927
2	rs1918173	156893605	A	0.2423	0.2292	C	0.6284	1.075
9	rs216345	33799370	A	0.2423	0.2292	G	0.6284	1.075
15	rs1667394	28530182	A	0.2423	0.2292	G	0.6284	1.075
17	rs2071214	76219591	G	0.2423	0.2292	A	0.6284	1.075
5	rs35262183	121488506	G	0.3582	0.3733	C	0.6285	0.937

9	rs10757225	21555445	C	0.3582	0.3733	A	0.6285	0.937
17	rs2197159	47477635	A	0.3582	0.3733	G	0.6285	0.937
1	rs4653044	33796081	A	0.3789	0.365	C	0.6286	1.061
2	rs117319532	100199339	G	0.02835	0.03583	A	0.6286	0.7851
4	rs76333976	76447062	G	0.02835	0.03583	C	0.6286	0.7851
6	rs1033423	11822003	G	0.02835	0.03583	A	0.6286	0.7851
7	rs17164867	130008285	G	0.02835	0.03583	A	0.6286	0.7851
9	rs2231406	135762921	A	0.02835	0.03583	G	0.6286	0.7851
10	rs593992	34536049	G	0.02835	0.03583	A	0.6286	0.7851
11	rs61744209	46917812	A	0.02835	0.03583	G	0.6286	0.7851
11	rs360726	111989592	A	0.3814	0.3667	G	0.6286	1.065
11	rs73607036	124007930	G	0.02835	0.03583	A	0.6286	0.7851
12	rs1984564	7090193	G	0.02835	0.03583	A	0.6286	0.7851
14	rs76859844	59931373	A	0.02835	0.03583	G	0.6286	0.7851
16	exm1204945	2087562	A	0.02835	0.03583	G	0.6286	0.7851
16	rs1132368	2087562	A	0.02835	0.03583	G	0.6286	0.7851
17	rs17686569	67290840	G	0.02835	0.03583	A	0.6286	0.7851
19	rs80055666	36278111	A	0.02835	0.03583	C	0.6286	0.7851
4	rs28520304	3881341	A	0.3582	0.3725	C	0.6287	0.9404
20	rs2180439	21853100	G	0.3582	0.3725	A	0.6287	0.9404
1	rs974843	87015179	A	0.2423	0.23	G	0.629	1.07
4	rs7653897	90169925	A	0.3608	0.3758	G	0.629	0.9375
7	rs2292498	4874420	C	0.3814	0.3675	G	0.629	1.061
11	rs7934731	23368595	A	0.3608	0.3758	G	0.629	0.9375
19	rs1079166	18385214	G	0.3608	0.3758	A	0.629	0.9375
20	rs1545	10386013	A	0.2423	0.23	C	0.629	1.07
20	rs2076584	19970705	G	0.2423	0.23	A	0.629	1.07
6	rs6937034	33079766	G	0.384	0.3692	A	0.6291	1.065
10	rs7918199	129905429	A	0.1624	0.1525	G	0.6291	1.077
12	rs671	112241766	A	0.1469	0.1583	G	0.6292	0.9154
13	rs2496425	39264690	G	0.3608	0.375	A	0.6292	0.9409
22	rs8139900	26394724	G	0.1469	0.1583	A	0.6292	0.9154
6	rs9386463	106094057	G	0.1477	0.1592	A	0.6293	0.9152
1	rs16830301	203823256	G	0.384	0.37	A	0.6294	1.062
12	rs11066015	112168009	A	0.1469	0.1592	G	0.6294	0.9097
14	rs8016892	48473724	G	0.384	0.37	A	0.6294	1.062
14	rs8015745	59438034	A	0.384	0.37	C	0.6294	1.062
16	rs9934272	81253745	C	0.384	0.37	G	0.6294	1.062
1	rs2075995	23847464	C	0.3634	0.3783	A	0.6295	0.938
2	rs1866558	105193300	G	0.3634	0.3783	A	0.6295	0.938
1	rs13306731	179320578	G	0.3634	0.3775	A	0.6297	0.9413
3	rs6801425	110940862	G	0.3634	0.3775	A	0.6297	0.9413
3	rs16836266	130992385	G	0.2448	0.2317	A	0.6297	1.075

7	rs10272900	32525631	G	0.3634	0.3775	A	0.6297	0.9413
14	rs8018102	91489448	A	0.3634	0.3775	G	0.6297	0.9413
10	rs224055	64496870	A	0.3866	0.3725	G	0.6299	1.062
1	rs2489337	225178198	A	0.2448	0.2325	G	0.6302	1.07
2	rs2241764	133174764	A	0.2448	0.2325	G	0.6302	1.07
2	rs66523341	135122375	A	0.366	0.38	G	0.6302	0.9418
3	rs59038848	197063068	A	0.366	0.38	G	0.6302	0.9418
7	rs2192828	12376811	A	0.366	0.38	C	0.6302	0.9418
6	rs3131888	29663889	G	0.3892	0.375	A	0.6303	1.062
8	rs1013209	24116304	A	0.3686	0.3833	G	0.6304	0.939
8	rs4737845	68454942	G	0.3918	0.3767	A	0.6304	1.066
16	rs4783227	82594383	G	0.3686	0.3833	A	0.6304	0.939
20	rs45539436	23805953	A	0.1082	0.1	G	0.6305	1.092
1	rs12044355	231844347	C	0.3686	0.3825	A	0.6307	0.9423
11	rs1128334	128328959	A	0.3918	0.3775	G	0.6307	1.062
11	rs6590330	128311059	A	0.3918	0.3779	G	0.6308	1.06
4	rs1344602	37996334	G	0.3711	0.3858	A	0.6309	0.9394
7	rs11238298	54219207	G	0.2268	0.24	A	0.6309	0.9289
7	rs997980	105951509	A	0.3711	0.3858	G	0.6309	0.9394
10	rs1937332	90826779	G	0.2268	0.24	A	0.6309	0.9289
4	rs16851681	47578971	A	0.2268	0.2408	G	0.631	0.9247
2	rs4973342	231532555	G	0.09536	0.1054	A	0.6311	0.8952
5	rs10037512	88354675	A	0.3943	0.38	G	0.6311	1.062
16	rs3743503	81056441	C	0.2268	0.2392	A	0.6311	0.9331
3	rs1381057	121154974	A	0.06701	0.06083	G	0.6312	1.109
3	rs3218649	121208833	G	0.06701	0.06083	C	0.6312	1.109
11	rs7120238	129175013	A	0.09536	0.105	G	0.6312	0.8985
12	rs747902	109431607	G	0.3711	0.385	A	0.6312	0.9427
19	rs34081	11531272	A	0.09536	0.105	C	0.6312	0.8985
19	rs365745	44471209	T	0.1649	0.155	A	0.6312	1.077
2	rs10180451	85926238	C	0.3737	0.3892	A	0.6313	0.9366
7	rs2192341	8261730	G	0.0567	0.065	A	0.6313	0.8646
11	rs6592284	86055901	G	0.3737	0.3892	A	0.6313	0.9366
16	rs141218390	88501386	A	0.0567	0.065	C	0.6313	0.8646
15	rs8028043	87217613	A	0.09536	0.1058	G	0.6314	0.8906
1	rs12085269	85213900	G	0.3969	0.3825	A	0.6315	1.062
4	rs4550960	80571936	A	0.3969	0.3825	G	0.6315	1.062
4	rs13118704	188451707	A	0.1495	0.1608	G	0.6315	0.917
6	rs4713240	29710726	G	0.3969	0.3825	A	0.6315	1.062
6	rs909545	167754661	A	0.2474	0.235	G	0.6315	1.07
10	rs1998596	9399473	A	0.2474	0.235	G	0.6315	1.07
3	rs35964523	898661	G	0.3995	0.3846	A	0.6316	1.064
6	rs2508015	31010200	A	0.1495	0.1617	G	0.6316	0.9114

8	rs78448370	142446937	A	0.1495	0.1617	G	0.6316	0.9114
10	rs4925	106022789	A	0.1495	0.1617	C	0.6316	0.9114
13	rs1927745	107780316	A	0.3943	0.3808	G	0.6316	1.059
14	rs28701519	29980231	C	0.3943	0.3808	A	0.6316	1.059
6	rs619483	4087934	G	0.3763	0.3917	C	0.6317	0.937
7	rs12719961	155151194	G	0.3763	0.3917	A	0.6317	0.937
14	rs11629205	79156785	A	0.3763	0.3917	G	0.6317	0.937
5	rs1395136	8276128	G	0.3763	0.3907	A	0.6318	0.9411
2	rs3844267	174525107	A	0.3969	0.3833	G	0.632	1.059
1	rs61910697	3410410	A	0.0567	0.06583	G	0.6321	0.8529
1	rs139445275	23420650	C	0.01804	0.01417	A	0.6321	1.279
1	rs74953085	169776993	G	0.01804	0.01417	A	0.6321	1.279
1	rs117391770	204197356	G	0.01804	0.01417	A	0.6321	1.279
2	rs138422162	242814983	A	0.01804	0.01417	G	0.6321	1.279
3	rs183126947	33055567	G	0.01804	0.01417	A	0.6321	1.279
3	rs78555408	38798298	C	0.01804	0.01417	A	0.6321	1.279
3	rs78132193	150416659	A	0.0567	0.06583	G	0.6321	0.8529
3	rs33927333	187088612	A	0.01804	0.01417	G	0.6321	1.279
4	rs142988385	1730313	C	0.01804	0.01417	G	0.6321	1.279
5	exm2257016	146030225	A	0.01804	0.01417	G	0.6321	1.279
6	rs117314762	31914306	A	0.01804	0.01417	G	0.6321	1.279
6	rs145115573	38704868	A	0.01804	0.01417	C	0.6321	1.279
6	rs121918027	161159625	A	0.01804	0.01417	G	0.6321	1.279
9	rs75820036	135781005	G	0.01804	0.01417	C	0.6321	1.279
10	rs11191439	104638723	G	0.01804	0.01417	A	0.6321	1.279
10	rs62640957	118401721	A	0.01804	0.01417	G	0.6321	1.279
11	rs186771698	76833711	C	0.01804	0.01417	A	0.6321	1.279
12	rs33962311	109526189	A	0.01804	0.01417	G	0.6321	1.279
12	rs115531779	125432696	A	0.01804	0.01417	C	0.6321	1.279
13	rs74317455	47351667	G	0.01804	0.01417	A	0.6321	1.279
15	rs143908070	23892037	A	0.01804	0.01417	G	0.6321	1.279
15	rs448720	68198911	G	0.2294	0.2425	A	0.6321	0.9298
17	rs201754956	9586221	A	0.01804	0.01417	G	0.6321	1.279
18	rs140494095	43534628	A	0.01804	0.01417	G	0.6321	1.279
19	rs55793080	41211076	A	0.01804	0.01417	C	0.6321	1.279
21	rs3819158	43222889	A	0.01804	0.01417	G	0.6321	1.279
21	rs186701249	47766840	A	0.01804	0.01417	G	0.6321	1.279
10	rs12251307	6123495	A	0.2294	0.2433	G	0.6322	0.9256
14	rs2281680	24033070	A	0.1675	0.1567	G	0.6322	1.083
15	rs7495052	92552029	A	0.2294	0.2433	G	0.6322	0.9256
21	rs437470	18937758	G	0.1675	0.1567	A	0.6322	1.083
1	rs4641	156107534	A	0.2474	0.2358	G	0.6323	1.065
6	rs9358856	25426768	A	0.2474	0.2358	G	0.6323	1.065

6	rs2275769	54095524	A	0.2474	0.2358	G	0.6323	1.065
5	rs17482078	96118866	A	0.03866	0.03333	G	0.6324	1.166
5	rs10050860	96122210	A	0.03866	0.03333	G	0.6324	1.166
5	rs2287987	96129535	G	0.03866	0.03333	A	0.6324	1.166
5	rs78253116	129521440	A	0.01804	0.01419	G	0.6324	1.276
12	rs78162420	72335380	A	0.03866	0.03333	C	0.6324	1.166
17	rs73337533	4451576	G	0.03866	0.03333	A	0.6324	1.166
2	rs10166942	234825093	A	0.3814	0.3958	G	0.6326	0.9412
11	rs2248020	93259965	A	0.3814	0.3958	C	0.6326	0.9412
17	rs2278868	46262171	G	0.25	0.2375	A	0.6326	1.07
18	rs4800452	20727611	G	0.25	0.2375	A	0.6326	1.07
22	rs131779	50988193	A	0.4046	0.39	G	0.6326	1.063
6	rs2051542	26108168	G	0.4021	0.3883	A	0.6327	1.059
9	rs1886264	2196734	A	0.384	0.3992	G	0.6329	0.9384
6	rs2596501	31321211	G	0.4072	0.3925	A	0.633	1.063
6	rs1508247	87145378	A	0.4072	0.3933	G	0.633	1.06
19	rs2547065	9080462	G	0.1786	0.1675	C	0.633	1.08
1	rs7514102	110998854	G	0.4046	0.3908	A	0.6331	1.059
1	rs12021830	119562382	A	0.384	0.3983	G	0.6331	0.9417
2	rs4499362	149568396	A	0.384	0.3983	G	0.6331	0.9417
4	rs7675033	164402006	C	0.384	0.3983	A	0.6331	0.9417
11	rs7950486	80547481	A	0.4046	0.3908	G	0.6331	1.059
13	rs1926657	95874956	A	0.384	0.3983	G	0.6331	0.9417
14	rs221903	71600208	G	0.4046	0.3908	A	0.6331	1.059
16	rs4362393	26091436	A	0.4046	0.3908	C	0.6331	1.059
20	rs6512586	48301146	A	0.384	0.3983	G	0.6331	0.9417
20	rs3827022	62195220	G	0.3866	0.4015	A	0.6332	0.9395
3	rs9819252	80023498	G	0.3866	0.4017	A	0.6333	0.9388
5	rs2973360	89418270	A	0.3866	0.4017	G	0.6333	0.9388
9	rs10818752	125585909	A	0.4098	0.395	G	0.6333	1.063
11	rs2276039	57137371	G	0.4098	0.395	A	0.6333	1.063
17	rs17843023	39116603	A	0.06995	0.0625	C	0.6333	1.128
2	rs2288709	44004010	G	0.1675	0.1575	A	0.6334	1.076
2	rs2580816	232797966	G	0.4072	0.3933	A	0.6334	1.06
19	rs11666579	17590281	A	0.232	0.2458	C	0.6334	0.9265
4	rs13115344	515489	A	0.25	0.2383	G	0.6335	1.065
13	rs1413191	93213476	A	0.25	0.2383	G	0.6335	1.065
17	rs2286672	4712617	A	0.3866	0.4007	G	0.6335	0.9428
1	rs197412	112308953	G	0.4098	0.3958	A	0.6337	1.06
1	rs1188710	228528563	G	0.4124	0.3975	C	0.6337	1.064
5	rs9313396	167499832	C	0.4124	0.3975	A	0.6337	1.064
2	rs2305165	136409574	C	0.1521	0.1642	A	0.6338	0.913
8	rs13255110	144945792	G	0.2526	0.24	A	0.6338	1.07

11	rs6590705	133334522	A	0.1521	0.1633	C	0.6338	0.9186
17	rs1071648	5326089	G	0.1521	0.1642	A	0.6338	0.913
2	rs816884	151319753	G	0.3918	0.4067	A	0.634	0.9397
3	rs2228001	14187449	C	0.4124	0.3983	A	0.634	1.06
3	rs10513762	179306775	A	0.1108	0.1025	G	0.634	1.091
6	rs10751	32902583	A	0.1108	0.1025	G	0.634	1.091
6	rs4896547	125125128	A	0.4149	0.4	G	0.634	1.064
7	rs4520084	147908658	A	0.3918	0.4067	G	0.634	0.9397
12	rs7969937	124330542	A	0.1108	0.1025	C	0.634	1.091
14	rs11623267	101348584	G	0.4149	0.4	C	0.634	1.064
15	rs16939937	58519664	G	0.4149	0.4	A	0.634	1.064
16	rs74439742	57059427	A	0.1553	0.1678	G	0.634	0.9117
18	rs4130047	40678235	G	0.4149	0.4	A	0.634	1.064
5	rs3776099	140502841	A	0.06959	0.0625	G	0.6341	1.122
9	rs200634680	20881887	G	0.005291	0.003333	A	0.6341	1.59
17	rs74744272	4892176	C	0.06959	0.0625	G	0.6341	1.122
20	rs73598376	35438474	A	0.06959	0.0625	G	0.6341	1.122
16	rs276967	86233398	A	0.2592	0.2458	G	0.6342	1.073
2	rs3769769	170675765	A	0.3918	0.4058	G	0.6343	0.943
6	rs2235883	46128688	C	0.4175	0.4023	G	0.6343	1.065
13	rs1408184	46946157	G	0.4149	0.4008	A	0.6343	1.06
17	rs2292751	40725799	A	0.4149	0.4008	G	0.6343	1.06
2	rs7593730	161171454	A	0.1701	0.1592	G	0.6344	1.083
5	rs9313379	166840321	A	0.3943	0.4092	G	0.6344	0.9401
11	rs2289965	18728650	G	0.1701	0.1592	A	0.6344	1.083
11	rs10742805	47454701	A	0.3943	0.4092	G	0.6344	0.9401
19	rs4438413	53884412	G	0.4175	0.4025	A	0.6344	1.064
3	rs11719375	10706597	A	0.3943	0.4083	G	0.6346	0.9434
6	rs4713420	30993567	A	0.2526	0.2408	C	0.6346	1.065
6	rs612421	150716696	A	0.2526	0.2408	G	0.6346	1.065
7	rs1990427	83471330	A	0.2526	0.2408	G	0.6346	1.065
19	rs17855403	51607507	A	0.2526	0.2408	G	0.6346	1.065
5	rs1035480	36269551	G	0.3969	0.4117	C	0.6347	0.9406
6	rs560505	32261771	G	0.4201	0.405	A	0.6347	1.064
7	rs41030	104143381	A	0.4201	0.405	C	0.6347	1.064
5	rs17521570	34757666	A	0.09794	0.1077	G	0.6349	0.8997
6	rs477005	32270500	G	0.4201	0.4058	A	0.6349	1.061
2	rs11127202	29240060	G	0.09794	0.1075	A	0.635	0.9014
2	rs6741148	38277832	C	0.09794	0.1075	A	0.635	0.9014
2	rs7349332	219756383	A	0.2552	0.2425	G	0.635	1.07
2	rs6759892	234601669	C	0.2552	0.2425	A	0.635	1.07
3	rs507856	161453946	A	0.3969	0.4108	G	0.635	0.9438
6	rs1523965	147987530	G	0.4227	0.4075	A	0.635	1.065

8	rs3735887	99205612	G	0.4227	0.4075	A	0.635	1.065
8	rs1870809	141004455	G	0.09794	0.1075	A	0.635	0.9014
9	rs7852962	113196733	C	0.09794	0.1075	A	0.635	0.9014
11	rs1541314	1263523	A	0.09794	0.1075	G	0.635	0.9014
11	rs3021156	1272754	G	0.09794	0.1075	A	0.635	0.9014
11	rs685870	64111928	G	0.3969	0.4108	A	0.635	0.9438
12	rs7978353	122617989	G	0.3969	0.4108	A	0.635	0.9438
16	rs78970023	21689879	A	0.4349	0.4199	T	0.635	1.063
4	rs1782360	151773593	G	0.09794	0.1083	C	0.6351	0.8936
11	rs2943510	1263776	A	0.09794	0.1083	G	0.6351	0.8936
17	rs2072147	56395757	A	0.09794	0.1083	G	0.6351	0.8936
17	rs2072145	56396609	G	0.09794	0.1083	A	0.6351	0.8936
22	rs9610841	38121152	C	0.3995	0.4142	A	0.6351	0.941
9	rs7851008	82596501	A	0.4227	0.4083	G	0.6352	1.061
9	rs3739927	135139826	G	0.4227	0.4083	A	0.6352	1.061
1	rs1317209	20140036	A	0.4021	0.4175	G	0.6353	0.9382
3	rs6809073	74112675	A	0.4021	0.4175	G	0.6353	0.9382
4	rs28425634	121429891	A	0.4021	0.4175	C	0.6353	0.9382
6	rs375555	33557741	G	0.4021	0.4175	A	0.6353	0.9382
12	rs3825214	114795443	G	0.4021	0.4175	A	0.6353	0.9382
17	rs8064529	73519413	A	0.4021	0.4175	G	0.6353	0.9382
1	rs2527614	228126470	G	0.4021	0.4167	A	0.6354	0.9414
2	rs6707813	173923137	A	0.4021	0.4167	G	0.6354	0.9414
3	rs2234358	45989044	A	0.4021	0.4167	C	0.6354	0.9414
5	rs1494558	35861068	G	0.4021	0.4167	A	0.6354	0.9414
8	rs4242382	128517573	A	0.1701	0.16	G	0.6354	1.076
9	rs2298050	105767481	G	0.1701	0.16	A	0.6354	1.076
9	rs2298051	105767536	A	0.1701	0.16	G	0.6354	1.076
15	rs1800588	58723675	A	0.4021	0.4167	G	0.6354	0.9414
18	rs67368442	1631465	C	0.4021	0.4167	A	0.6354	0.9414
18	rs486285	55822713	G	0.4021	0.4167	A	0.6354	0.9414
19	rs3810267	50379053	G	0.3995	0.4133	A	0.6354	0.9442
19	rs3745486	50383636	G	0.3995	0.4133	A	0.6354	0.9442
4	rs4538475	15737937	A	0.4253	0.4108	G	0.6355	1.061
4	rs1372887	12000904	A	0.4046	0.42	G	0.6356	0.9386
6	rs1119080	29536685	A	0.4167	0.4317	G	0.6356	0.9404
1	rs2501432	24201920	A	0.4227	0.4092	G	0.6357	1.057
11	rs11229898	59029485	G	0.4227	0.4092	A	0.6357	1.057
12	rs10842496	25311489	A	0.2552	0.2433	C	0.6357	1.065
17	rs9916491	42987524	G	0.2552	0.2433	A	0.6357	1.065
22	rs573273	27638420	G	0.4227	0.4092	A	0.6357	1.057
6	rs3131622	31420500	C	0.2591	0.2458	A	0.6358	1.073
6	rs2239707	31525319	G	0.2371	0.2508	A	0.6358	0.9283

6	rs2229848	129807629	G	0.4278	0.4133	A	0.6358	1.061
1	rs773564	113824297	A	0.4072	0.4225	G	0.6359	0.939
2	rs3821348	241463453	A	0.1134	0.1042	C	0.6359	1.1
16	rs4359426	57392733	A	0.1546	0.1658	C	0.6359	0.9201
17	rs2249542	685752	A	0.1134	0.1042	G	0.6359	1.1
1	rs11184961	107220341	A	0.4253	0.4117	G	0.636	1.057
1	rs814346	190764733	G	0.4253	0.4117	A	0.636	1.057
8	rs35119879	2595868	G	0.4072	0.4217	A	0.636	0.9422
8	rs2280838	145642002	A	0.4304	0.4158	G	0.636	1.062
11	rs11036913	5364742	A	0.2577	0.245	G	0.6361	1.07
17	rs12449313	18221010	G	0.2577	0.245	A	0.6361	1.07
6	rs6457702	32988049	A	0.4098	0.425	C	0.6362	0.9394
20	rs2076015	7963041	G	0.4098	0.425	A	0.6362	0.9394
1	rs1337248	109806442	C	0.433	0.4183	A	0.6363	1.062
2	rs2304290	47251469	G	0.433	0.4183	C	0.6363	1.062
2	rs9309107	44021826	T	0.1727	0.1617	A	0.6364	1.082
4	rs1800789	155482743	A	0.1727	0.1617	G	0.6364	1.082
4	rs6056	155488821	A	0.1727	0.1617	G	0.6364	1.082
6	rs4357136	13097658	C	0.1727	0.1617	A	0.6364	1.082
12	rs2273987	10150961	A	0.1727	0.1617	G	0.6364	1.082
15	rs61731670	52901283	A	0.1727	0.1617	G	0.6364	1.082
4	rs10518556	130691794	G	0.4304	0.4167	A	0.6365	1.058
14	rs7145636	29063877	A	0.4145	0.4283	G	0.6365	0.9449
2	rs10188142	53958136	G	0.4356	0.4208	A	0.6366	1.062
1	rs2494620	2441358	G	0.433	0.4192	A	0.6367	1.058
2	rs2629046	225047744	A	0.4149	0.43	G	0.6367	0.9402
4	rs10213441	36624280	A	0.4149	0.43	G	0.6367	0.9402
4	rs3912476	96379903	A	0.4149	0.43	G	0.6367	0.9402
4	rs1898854	178028186	A	0.4149	0.43	G	0.6367	0.9402
10	rs7914810	89336834	G	0.4149	0.43	A	0.6367	0.9402
21	rs762173	33832675	A	0.433	0.4192	G	0.6367	1.058
21	rs2838519	45615023	A	0.4149	0.43	G	0.6367	0.9402
2	rs984066	116283668	A	0.4356	0.4217	G	0.6369	1.058
10	rs2031383	11885961	G	0.4356	0.4217	A	0.6369	1.058
2	rs7604613	19989521	A	0.4175	0.4325	G	0.637	0.9406
4	rs2684348	169282355	A	0.2397	0.2533	G	0.637	0.9292
5	rs1864982	146320823	A	0.2397	0.2525	C	0.637	0.9333
5	rs411174	156609000	A	0.2397	0.2533	G	0.637	0.9292
6	rs540785	49288384	G	0.4175	0.4325	A	0.637	0.9406
8	rs12549973	8176554	G	0.4149	0.4292	A	0.637	0.9434
11	rs4757388	16274295	G	0.4149	0.4292	A	0.637	0.9434
12	rs4764393	18487862	A	0.4149	0.4292	G	0.637	0.9434
15	rs3825801	52517714	A	0.2397	0.2533	G	0.637	0.9292

8	rs2229315	145665407	A	0.4381	0.4242	G	0.6371	1.059
11	rs6591778	62987767	A	0.4381	0.4242	G	0.6371	1.059
11	rs1790218	63057925	A	0.4381	0.4242	G	0.6371	1.059
11	rs1201559	63072310	A	0.4381	0.4242	G	0.6371	1.059
1	rs3120714	246730090	C	0.06959	0.06333	G	0.6372	1.106
3	rs62290268	194790799	G	0.06959	0.06333	C	0.6372	1.106
4	rs11096807	33200295	A	0.4175	0.4317	G	0.6372	0.9438
5	rs31853	140474756	A	0.06959	0.06333	G	0.6372	1.106
5	rs31849	140480355	G	0.06959	0.06333	A	0.6372	1.106
6	rs2535260	29628883	C	0.06959	0.06333	G	0.6372	1.106
6	rs3213485	32724719	A	0.2603	0.2475	G	0.6372	1.07
10	rs12412320	128150096	A	0.2603	0.2475	C	0.6372	1.07
15	rs2241647	34159986	G	0.2603	0.2475	A	0.6372	1.07
19	rs61180947	5772950	A	0.4201	0.4349	G	0.6372	0.9414
3	rs2929394	20109901	A	0.4407	0.4267	C	0.6374	1.059
9	rs1831521	93409357	G	0.4407	0.4267	A	0.6374	1.059
2	rs2970895	88417387	A	0.4201	0.4342	G	0.6375	0.9441
5	rs35384751	39202689	A	0.05928	0.0675	C	0.6375	0.8705
5	rs17844350	140250471	A	0.05928	0.06773	C	0.6375	0.8674
6	rs9354308	66565353	G	0.1134	0.105	A	0.6375	1.09
7	rs79519739	47906069	A	0.05928	0.0675	G	0.6375	0.8705
11	rs1203998	65793454	A	0.4459	0.4308	G	0.6375	1.063
11	rs11237599	70666765	A	0.05928	0.0675	G	0.6375	0.8705
11	rs10488698	116633947	A	0.05928	0.0675	G	0.6375	0.8705
12	rs187811112	26784809	C	0.005181	0.003333	A	0.6375	1.557
13	rs80135321	25424583	G	0.1134	0.105	A	0.6375	1.09
15	rs75981505	45412946	A	0.05928	0.0675	C	0.6375	0.8705
16	rs3785250	7604361	G	0.4201	0.4342	A	0.6375	0.9441
17	rs35670089	56349236	A	0.005181	0.003333	G	0.6375	1.557
19	rs12460932	36004171	A	0.4201	0.4342	C	0.6375	0.9441
22	rs5763662	30378703	A	0.05928	0.0675	G	0.6375	0.8705
5	rs2303063	147480027	A	0.4433	0.4292	G	0.6376	1.059
5	rs2303067	147480955	G	0.4433	0.4292	A	0.6376	1.059
9	rs1111782	27212966	A	0.4485	0.4333	G	0.6377	1.063
1	rs10783071	92428495	C	0.4459	0.4317	A	0.6378	1.059
12	rs2228570	48272895	A	0.4459	0.4317	G	0.6378	1.059
12	rs11176433	67209123	A	0.4227	0.4367	G	0.6378	0.9445
18	rs9949570	68844408	G	0.4227	0.4367	A	0.6378	0.9445
22	rs134474	49911222	C	0.4459	0.4317	A	0.6378	1.059
1	rs1023252	11899033	A	0.1572	0.1686	C	0.6379	0.9198
5	rs251018	140902116	C	0.1572	0.1686	A	0.6379	0.9198
6	rs240993	111673714	G	0.4485	0.4342	A	0.6379	1.06
10	rs2295778	102295836	C	0.2603	0.2483	G	0.6379	1.065

14	rs944289	36649246	A	0.451	0.4358	G	0.6379	1.064
14	rs808225	58397916	A	0.2603	0.2483	G	0.6379	1.065
6	rs1015465	30086340	G	0.1572	0.1692	A	0.638	0.9162
6	rs2021723	30103923	A	0.1572	0.1692	G	0.638	0.9162
6	rs2257914	30120563	A	0.1572	0.1692	C	0.638	0.9162
6	rs2049953	135138990	C	0.1572	0.1692	A	0.638	0.9162
10	rs2989404	30164762	A	0.4585	0.4441	G	0.638	1.06
1	rs4601530	25044111	A	0.4253	0.4392	G	0.6381	0.9449
1	rs9803659	167156500	G	0.2423	0.2558	A	0.6381	0.93
2	rs6433379	173620465	G	0.03866	0.03417	A	0.6381	1.137
2	rs56071233	179439952	A	0.03866	0.03417	G	0.6381	1.137
4	rs1435787	30407029	G	0.4304	0.4458	A	0.6381	0.9393
9	rs7027092	95812707	A	0.4304	0.4458	G	0.6381	0.9393
11	rs117729282	71728822	C	0.03866	0.03417	G	0.6381	1.137
11	rs116981680	117100134	A	0.03866	0.03417	C	0.6381	1.137
12	rs11549949	52908872	A	0.03866	0.03417	G	0.6381	1.137
12	rs11549950	52908917	G	0.03866	0.03417	A	0.6381	1.137
12	rs2464196	121435427	G	0.4253	0.4392	A	0.6381	0.9449
15	rs75478132	91456590	G	0.03866	0.03417	A	0.6381	1.137
20	rs995636	7008824	G	0.1572	0.1683	A	0.6381	0.9216
20	rs1053993	17716416	C	0.4253	0.4392	G	0.6381	0.9449
22	rs140079	32110943	A	0.03866	0.03417	G	0.6381	1.137
3	rs816488	39553788	G	0.05928	0.06833	A	0.6382	0.8591
12	rs838905	125291006	A	0.4536	0.4391	G	0.6382	1.061
13	rs36084898	60566644	G	0.05928	0.06833	A	0.6382	0.8591
16	rs9935113	81211496	A	0.05928	0.06833	C	0.6382	0.8591
18	rs35193847	47777244	A	0.05928	0.06833	G	0.6382	0.8591
6	rs2516703	30229922	G	0.4278	0.4417	A	0.6383	0.9453
6	rs261949	30270080	A	0.4278	0.4417	G	0.6383	0.9453
9	rs3750534	116060124	G	0.4536	0.4392	A	0.6383	1.06
9	rs3750533	116060221	A	0.4536	0.4392	G	0.6383	1.06
1	rs200296371	2268280	G	0.005155	0.003333	A	0.6384	1.549
1	rs1281013	11009679	A	0.005155	0.003333	G	0.6384	1.549
1	rs1281012	11009703	A	0.005155	0.003333	G	0.6384	1.549
1	rs2359484	12907701	C	0.005155	0.003333	G	0.6384	1.549
1	rs147414271	17666263	A	0.005155	0.003333	G	0.6384	1.549
1	rs139281890	20639464	A	0.005155	0.003333	G	0.6384	1.549
1	rs183114446	32119590	A	0.005155	0.003333	C	0.6384	1.549
1	rs192016258	43908279	A	0.005155	0.003333	G	0.6384	1.549
1	rs117709229	47689696	G	0.005155	0.003333	A	0.6384	1.549
1	rs141195581	79002230	G	0.005155	0.003333	A	0.6384	1.549
1	rs202079327	89730384	A	0.005155	0.003333	C	0.6384	1.549
1	rs56357060	94496039	A	0.005155	0.003333	G	0.6384	1.549

1	rs112542201	118628623	C	0.005155	0.003333	G	0.6384	1.549
1	rs112264801	144922524	A	0.005155	0.003333	G	0.6384	1.549
1	rs201853284	153362927	A	0.005155	0.003333	G	0.6384	1.549
1	rs149673076	158151902	A	0.005155	0.003333	G	0.6384	1.549
1	rs138480183	158813911	A	0.005155	0.003333	G	0.6384	1.549
1	rs2295617	160722007	A	0.005155	0.003333	G	0.6384	1.549
1	rs189889983	167095278	G	0.005155	0.003333	A	0.6384	1.549
1	rs201292247	169512296	G	0.005155	0.003333	A	0.6384	1.549
1	rs142260750	174274224	A	0.005155	0.003333	G	0.6384	1.549
1	rs149197116	178427099	A	0.005155	0.003333	G	0.6384	1.549
1	rs1318056	179112145	C	0.005155	0.003333	G	0.6384	1.549
1	rs201353530	179815307	A	0.005155	0.003333	T	0.6384	1.549
1	rs7539719	185958737	G	0.005155	0.003333	A	0.6384	1.549
1	rs146010254	203798997	A	0.005155	0.003333	G	0.6384	1.549
1	rs182395266	206811017	G	0.005155	0.003333	A	0.6384	1.549
1	rs34259050	214507580	G	0.005155	0.003333	A	0.6384	1.549
1	rs193045993	217804724	A	0.005155	0.003333	G	0.6384	1.549
1	rs150611181	230513337	A	0.005155	0.003333	G	0.6384	1.549
1	rs189454297	230883283	A	0.005155	0.003333	G	0.6384	1.549
2	rs185330217	8926341	A	0.005155	0.003333	G	0.6384	1.549
2	rs189933923	27501096	G	0.005155	0.003333	A	0.6384	1.549
2	rs200817926	29294325	A	0.005155	0.003333	G	0.6384	1.549
2	rs200566214	39499448	G	0.005155	0.003333	A	0.6384	1.549
2	rs183643261	88828744	A	0.005155	0.003333	G	0.6384	1.549
2	rs143132498	99185068	A	0.005155	0.003333	G	0.6384	1.549
2	rs189229644	113984805	A	0.005155	0.003333	G	0.6384	1.549
2	rs200835233	121999957	G	0.005155	0.003333	A	0.6384	1.549
2	rs138094198	136026633	A	0.005155	0.003333	G	0.6384	1.549
2	rs144134332	160862240	A	0.005155	0.003333	G	0.6384	1.549
2	rs139145525	166626832	A	0.005155	0.003333	C	0.6384	1.549
2	rs121918807	166850785	A	0.005155	0.003333	G	0.6384	1.549
2	rs181539061	168103129	A	0.005155	0.003333	G	0.6384	1.549
2	rs868179	177549497	A	0.005155	0.003333	G	0.6384	1.549
2	rs61306957	178528641	G	0.005155	0.003333	C	0.6384	1.549
2	rs201469715	197184538	A	0.005155	0.003333	G	0.6384	1.549
2	rs115493196	203620403	G	0.005155	0.003333	A	0.6384	1.549
2	rs140000315	204326023	A	0.005155	0.003333	G	0.6384	1.549
2	rs185854595	217293430	G	0.005155	0.003333	A	0.6384	1.549
2	rs145588740	230450693	A	0.005155	0.003333	G	0.6384	1.549
2	rs199778762	239009082	A	0.005155	0.003333	G	0.6384	1.549
3	rs200343387	9918782	G	0.005155	0.003333	A	0.6384	1.549
3	rs80261363	12953129	G	0.005155	0.003333	A	0.6384	1.549
3	rs201069462	28520380	A	0.005155	0.003333	G	0.6384	1.549

3	rs112394380	33453135	G	0.005155	0.003333	A	0.6384	1.549
3	rs3733101	46009684	A	0.005155	0.003333	G	0.6384	1.549
3	rs187964377	48455393	A	0.005155	0.003333	G	0.6384	1.549
3	rs188487818	49167286	A	0.005155	0.003333	G	0.6384	1.549
3	rs202217531	52378506	A	0.005155	0.003333	G	0.6384	1.549
3	rs201630300	58121848	A	0.005155	0.003333	G	0.6384	1.549
3	rs832032	97726747	A	0.005155	0.003333	T	0.6384	1.549
3	rs4434123	111923123	G	0.005155	0.003333	A	0.6384	1.549
3	rs146464395	113955753	A	0.005155	0.003333	G	0.6384	1.549
3	rs117446067	123003465	A	0.005155	0.003333	G	0.6384	1.549
3	rs34985183	124906162	A	0.005155	0.003333	G	0.6384	1.549
3	rs185816798	133119306	C	0.005155	0.003333	G	0.6384	1.549
3	rs61733582	180379699	A	0.005155	0.003333	G	0.6384	1.549
3	rs201628957	183774688	A	0.005155	0.003333	G	0.6384	1.549
3	rs3774266	186937924	A	0.005155	0.003333	G	0.6384	1.549
3	rs201527436	192516315	G	0.005155	0.003333	C	0.6384	1.549
4	rs76052398	675777	C	0.005155	0.003333	G	0.6384	1.549
4	rs117409587	2306896	A	0.005155	0.003333	G	0.6384	1.549
4	rs76941356	56825869	C	0.005155	0.003333	A	0.6384	1.549
4	rs117925738	72635114	A	0.005155	0.003333	G	0.6384	1.549
4	rs148026447	76452357	A	0.005155	0.003333	G	0.6384	1.549
4	rs181194611	77663057	A	0.005155	0.003333	G	0.6384	1.549
4	rs191030781	100356378	A	0.005155	0.003333	C	0.6384	1.549
4	rs142903075	128628066	A	0.005155	0.003333	G	0.6384	1.549
4	rs118037378	151772021	G	0.005155	0.003333	A	0.6384	1.549
4	rs201842678	156775452	A	0.005155	0.003333	G	0.6384	1.549
4	rs186220887	187549883	A	0.005155	0.003333	G	0.6384	1.549
5	rs201565342	635600	A	0.005155	0.003333	G	0.6384	1.549
5	rs78256662	71495805	A	0.005155	0.003333	G	0.6384	1.549
5	rs144833043	71591394	A	0.005155	0.003333	G	0.6384	1.549
5	rs186214606	82815317	A	0.005155	0.003333	G	0.6384	1.549
5	rs148562350	126862430	G	0.005155	0.003333	A	0.6384	1.549
5	rs180811158	145547800	G	0.005155	0.003333	A	0.6384	1.549
5	rs61739724	154394577	G	0.005155	0.003333	A	0.6384	1.549
5	rs139716868	169812421	A	0.005155	0.003333	G	0.6384	1.549
6	rs184154918	7580346	A	0.005155	0.003333	G	0.6384	1.549
6	rs143364138	26020917	A	0.005155	0.003333	G	0.6384	1.549
6	rs36043209	27114910	A	0.005155	0.003333	G	0.6384	1.549
6	rs35273540	33260215	A	0.005155	0.003333	G	0.6384	1.549
6	rs116628998	90399646	G	0.005155	0.003333	A	0.6384	1.549
6	rs115155680	90459333	A	0.005155	0.003333	C	0.6384	1.549
6	rs41285300	130372517	G	0.005155	0.003333	A	0.6384	1.549
6	rs200877330	132054738	A	0.005155	0.003333	G	0.6384	1.549

7	rs6974491	37374510	A	0.005155	0.003333	G	0.6384	1.549
7	rs34741656	87913221	A	0.005155	0.003333	G	0.6384	1.549
7	rs80006813	88964061	C	0.005155	0.003333	A	0.6384	1.549
7	rs74564278	88965150	G	0.005155	0.003333	A	0.6384	1.549
7	rs35996821	92132493	G	0.005155	0.003333	A	0.6384	1.549
7	rs138238924	92764270	A	0.005155	0.003333	C	0.6384	1.549
7	rs1800073	117144344	A	0.005155	0.003333	G	0.6384	1.549
7	rs61729032	142641420	A	0.005155	0.003333	T	0.6384	1.549
8	rs143759433	11710185	G	0.005155	0.003333	A	0.6384	1.549
8	rs4922233	16639069	G	0.433	0.4475	A	0.6384	0.9428
8	rs200920297	59498262	A	0.005155	0.003333	G	0.6384	1.549
8	rs2976189	87235273	A	0.005155	0.003333	G	0.6384	1.549
8	rs137876000	91054991	A	0.005155	0.003333	G	0.6384	1.549
8	rs138285479	134262747	A	0.005155	0.003333	G	0.6384	1.549
8	rs139956930	144411609	G	0.005155	0.003333	C	0.6384	1.549
8	rs200436731	145004104	A	0.005155	0.003333	G	0.6384	1.549
9	rs75352090	328113	A	0.005155	0.003333	G	0.6384	1.549
9	rs201727651	5920528	A	0.005155	0.003333	G	0.6384	1.549
9	rs191470643	7799494	A	0.005155	0.003333	C	0.6384	1.549
9	rs191333137	21333465	A	0.005155	0.003333	G	0.6384	1.549
9	rs199527164	33944624	T	0.005155	0.003333	A	0.6384	1.549
9	rs146155568	114172355	T	0.005155	0.003333	A	0.6384	1.549
9	rs139039113	116958281	A	0.005155	0.003333	G	0.6384	1.549
9	rs144220097	117849420	A	0.005155	0.003333	G	0.6384	1.549
9	rs138956040	119097230	A	0.005155	0.003333	G	0.6384	1.549
9	rs75008792	123886324	A	0.005155	0.003333	G	0.6384	1.549
9	rs72760254	123932039	A	0.005155	0.003333	C	0.6384	1.549
9	rs140009447	139835876	A	0.005155	0.003333	G	0.6384	1.549
10	rs137874246	7605146	G	0.005155	0.003333	A	0.6384	1.549
10	rs189563213	8006547	C	0.005155	0.003333	G	0.6384	1.549
10	rs115687408	27035311	G	0.005155	0.003333	A	0.6384	1.549
10	rs117134003	27459035	A	0.005155	0.003333	G	0.6384	1.549
10	rs202056332	48370683	A	0.005155	0.003333	G	0.6384	1.549
10	rs199786639	55955553	G	0.005155	0.003333	A	0.6384	1.549
10	rs150589189	75566917	C	0.005155	0.003333	G	0.6384	1.549
10	rs141412560	95079675	A	0.005155	0.003333	G	0.6384	1.549
10	rs147842178	96121503	A	0.005155	0.003333	C	0.6384	1.549
10	rs116066839	104858719	G	0.005155	0.003333	A	0.6384	1.549
10	rs189150188	115381756	A	0.005155	0.003333	G	0.6384	1.549
10	rs148679332	115412785	C	0.005155	0.003333	A	0.6384	1.549
10	rs146536805	115885779	A	0.005155	0.003333	G	0.6384	1.549
10	rs144406058	124593214	A	0.005155	0.003333	G	0.6384	1.549
10	rs199957428	126097420	G	0.005155	0.003333	A	0.6384	1.549

10	rs141506421	127548304	C	0.005155	0.003333	A	0.6384	1.549
11	rs192996736	864511	A	0.005155	0.003333	G	0.6384	1.549
11	rs139629765	4944620	A	0.005155	0.003333	G	0.6384	1.549
11	rs147301110	5809455	A	0.005155	0.003333	G	0.6384	1.549
11	rs199915216	6415539	A	0.005155	0.003333	G	0.6384	1.549
11	rs200784979	9809181	A	0.005155	0.003333	G	0.6384	1.549
11	rs11604561	17118687	G	0.005155	0.003333	A	0.6384	1.549
11	rs144811457	17132074	G	0.005155	0.003333	A	0.6384	1.549
11	rs141449584	18048098	A	0.005155	0.003333	G	0.6384	1.549
11	rs139618850	22283684	A	0.005155	0.003333	G	0.6384	1.549
11	rs141209313	31284619	G	0.005155	0.003333	A	0.6384	1.549
11	rs199609729	67376023	A	0.005155	0.003333	C	0.6384	1.549
11	rs58428187	74992129	G	0.005155	0.003333	A	0.6384	1.549
11	rs11826199	82644764	C	0.005155	0.003333	G	0.6384	1.549
11	rs140703637	100221580	C	0.005155	0.003333	A	0.6384	1.549
11	rs146498786	113266930	A	0.005155	0.003333	G	0.6384	1.549
12	rs887304	3757548	A	0.005155	0.003333	G	0.6384	1.549
12	rs200419261	9229390	G	0.005155	0.003333	A	0.6384	1.549
12	rs76943150	32138027	A	0.005155	0.003333	G	0.6384	1.549
12	rs200496180	51685716	A	0.005155	0.003333	G	0.6384	1.549
12	rs139316403	53042808	G	0.005155	0.003333	A	0.6384	1.549
12	rs144134358	53803151	A	0.005155	0.003333	G	0.6384	1.549
12	rs200577458	97338479	A	0.005155	0.003333	C	0.6384	1.549
12	rs11110077	100266113	C	0.005155	0.003333	G	0.6384	1.549
12	rs17851246	100482866	A	0.005155	0.003333	G	0.6384	1.549
12	rs7311096	100775956	A	0.005155	0.003333	G	0.6384	1.549
12	rs139585780	120966809	A	0.005155	0.003333	G	0.6384	1.549
12	rs76063811	124835212	C	0.005155	0.003333	G	0.6384	1.549
12	rs200413984	130839145	A	0.005155	0.003333	G	0.6384	1.549
13	rs199610348	21173662	A	0.005155	0.003333	G	0.6384	1.549
13	rs147390019	48619856	A	0.005155	0.003333	G	0.6384	1.549
13	rs184995173	61141762	A	0.005155	0.003333	G	0.6384	1.549
13	rs139899673	76423351	A	0.005155	0.003333	C	0.6384	1.549
13	rs144929980	76429413	A	0.005155	0.003333	G	0.6384	1.549
13	rs201767993	77574962	G	0.005155	0.003333	A	0.6384	1.549
13	rs200759559	88330315	A	0.005155	0.003333	G	0.6384	1.549
13	rs201647127	111164432	A	0.005155	0.003333	G	0.6384	1.549
14	rs144727961	57052563	A	0.005155	0.003333	G	0.6384	1.549
14	rs201376759	74360573	A	0.005155	0.003333	T	0.6384	1.549
14	rs150395539	78140295	A	0.005155	0.003333	G	0.6384	1.549
15	rs201650867	31327796	G	0.005155	0.003333	A	0.6384	1.549
15	rs199513326	43317088	A	0.005155	0.003333	G	0.6384	1.549
15	rs200710879	74708223	A	0.005155	0.003333	G	0.6384	1.549

15	rs189925962	91333894	G	0.005155	0.003333	A	0.6384	1.549
16	rs200758799	427062	A	0.005155	0.003333	G	0.6384	1.549
16	rs200840629	4935048	A	0.005155	0.003333	G	0.6384	1.549
16	rs199663511	19741811	G	0.005155	0.003333	A	0.6384	1.549
16	rs114649866	23113762	G	0.005155	0.003333	A	0.6384	1.549
16	rs17289102	50342658	A	0.005155	0.003333	G	0.6384	1.549
16	rs199662749	53337907	A	0.005155	0.003333	G	0.6384	1.549
16	rs144494221	58541762	A	0.005155	0.003333	G	0.6384	1.549
16	rs11859363	61461435	G	0.005155	0.003333	A	0.6384	1.549
16	rs139051907	71713301	G	0.005155	0.003333	A	0.6384	1.549
17	rs148156494	602613	A	0.005155	0.003333	G	0.6384	1.549
17	rs190874588	1840547	A	0.005155	0.003333	G	0.6384	1.549
17	rs150471603	7005508	G	0.005155	0.003333	A	0.6384	1.549
17	rs191995793	11648150	A	0.005155	0.003333	G	0.6384	1.549
17	rs139373457	17715971	G	0.005155	0.003333	C	0.6384	1.549
17	rs143815623	18220721	A	0.005155	0.003333	C	0.6384	1.549
17	rs201337554	26875038	A	0.005155	0.003333	G	0.6384	1.549
17	rs114337101	38028598	G	0.005155	0.003333	A	0.6384	1.549
17	rs202225978	39019462	C	0.005155	0.003333	A	0.6384	1.549
17	rs117897485	39974440	A	0.005155	0.003333	G	0.6384	1.549
17	rs201130883	40256863	G	0.005155	0.003333	A	0.6384	1.549
17	rs186669379	48245315	A	0.005155	0.003333	G	0.6384	1.549
17	rs186222560	57763111	G	0.005155	0.003333	A	0.6384	1.549
17	rs76501888	73132140	G	0.005155	0.003333	A	0.6384	1.549
17	rs117610545	74015697	A	0.005155	0.003333	G	0.6384	1.549
18	rs148225763	7010327	A	0.005155	0.003333	G	0.6384	1.549
18	rs113553100	7012099	A	0.005155	0.003333	C	0.6384	1.549
19	rs201378376	5693709	A	0.005155	0.003333	G	0.6384	1.549
19	rs117824875	7533891	A	0.005155	0.003333	G	0.6384	1.549
19	rs201293382	7707702	A	0.005155	0.003333	G	0.6384	1.549
19	rs35061979	8436200	A	0.005155	0.003333	G	0.6384	1.549
19	rs56361194	14165598	A	0.005155	0.003333	G	0.6384	1.549
19	rs199917925	14705363	G	0.005155	0.003333	A	0.6384	1.549
19	rs188702643	15651365	C	0.005155	0.003333	G	0.6384	1.549
19	rs138789000	17476328	A	0.005155	0.003333	G	0.6384	1.549
19	rs199680814	35550869	G	0.005155	0.003333	A	0.6384	1.549
19	rs138262646	40419672	A	0.005155	0.003333	G	0.6384	1.549
19	rs200553377	41627438	A	0.005155	0.003333	G	0.6384	1.549
19	rs200843831	42914568	A	0.005155	0.003333	G	0.6384	1.549
19	rs142498461	44660997	C	0.005155	0.003333	G	0.6384	1.549
19	rs200611066	45368740	A	0.005155	0.003333	G	0.6384	1.549
19	rs190776413	48377926	A	0.005155	0.003333	G	0.6384	1.549
19	rs201547716	49368858	A	0.005155	0.003333	G	0.6384	1.549

19	rs140693786	50983419	A	0.005155	0.003333	G	0.6384	1.549
20	rs200309763	3804858	C	0.005155	0.003333	A	0.6384	1.549
20	rs34102290	9538335	A	0.005155	0.003333	C	0.6384	1.549
20	rs1799841	23860178	G	0.433	0.4475	A	0.6384	0.9428
20	rs201887283	36500369	A	0.005155	0.003333	G	0.6384	1.549
20	rs190461938	47863958	A	0.005155	0.003333	G	0.6384	1.549
22	rs118025402	20073919	A	0.005155	0.003333	G	0.6384	1.549
22	rs199891700	29534644	A	0.005155	0.003333	G	0.6384	1.549
22	rs199860120	32929861	C	0.005155	0.003333	G	0.6384	1.549
22	rs184241759	50523267	A	0.005155	0.003333	G	0.6384	1.549
22	rs147929426	50689427	A	0.005155	0.003333	G	0.6384	1.549
4	rs2711941	39064162	A	0.1753	0.1642	C	0.6385	1.082
4	rs11938367	161508641	A	0.4356	0.4508	G	0.6385	0.94
6	rs9357155	32809848	A	0.1753	0.1642	G	0.6385	1.082
16	rs8060283	5781710	G	0.1753	0.1642	A	0.6385	1.082
1	rs17121745	85589842	G	0.1005	0.1108	A	0.6386	0.8965
1	rs2305930	85594392	G	0.1005	0.1108	A	0.6386	0.8965
3	rs56053222	10251288	C	0.005155	0.003339	G	0.6386	1.547
3	rs149948290	43122560	A	0.005155	0.003339	G	0.6386	1.547
4	rs61743677	155157015	G	0.1005	0.1108	A	0.6386	0.8965
4	rs188510742	164247588	G	0.005155	0.003339	A	0.6386	1.547
8	rs2014286	16907125	G	0.4304	0.4442	A	0.6386	0.9456
8	rs75499773	144620861	A	0.005155	0.003339	G	0.6386	1.547
9	rs138014548	136307797	A	0.005155	0.003339	C	0.6386	1.547
11	rs490262	117222592	A	0.1005	0.1108	G	0.6386	0.8965
14	rs7146310	24653954	A	0.4588	0.4442	G	0.6386	1.061
17	rs34987539	66878099	A	0.005155	0.003339	G	0.6386	1.547
20	rs71647828	3870079	A	0.1005	0.11	T	0.6386	0.9041
3	rs264111	65766233	C	0.4536	0.44	A	0.6388	1.057
11	rs2741804	6913127	A	0.4536	0.44	G	0.6388	1.057
1	rs3737994	33234307	G	0.03093	0.0375	A	0.6389	0.8191
1	rs61739198	232942028	G	0.03093	0.0375	A	0.6389	0.8191
2	rs11125418	52478836	A	0.4407	0.4558	G	0.6389	0.9407
3	rs17036101	12277845	A	0.03093	0.0375	G	0.6389	0.8191
3	rs73866671	148562470	G	0.03093	0.0375	A	0.6389	0.8191
3	rs9853408	155520356	A	0.03093	0.0375	G	0.6389	0.8191
4	rs430979	2814698	A	0.4562	0.4425	C	0.6389	1.057
4	rs3756050	89670155	G	0.4639	0.4492	A	0.6389	1.061
4	rs148182120	106621170	A	0.03109	0.03769	G	0.6389	0.8193
6	rs610473	70911776	A	0.4562	0.4425	G	0.6389	1.057
6	rs2679672	122313558	G	0.4381	0.4525	A	0.6389	0.9435
7	rs7786347	138140578	C	0.4562	0.4425	A	0.6389	1.057
13	rs2274810	42264380	A	0.03093	0.0375	G	0.6389	0.8191

14	rs148072678	73729069	A	0.03093	0.0375	G	0.6389	0.8191
15	rs11072217	71110305	G	0.4381	0.4525	A	0.6389	0.9435
16	rs184877327	84906114	A	0.005155	0.003344	G	0.6389	1.544
19	rs11564538	48601512	A	0.03093	0.0375	G	0.6389	0.8191
1	rs2076599	17395480	A	0.4433	0.4583	G	0.639	0.9411
6	rs1362104	30101656	G	0.4588	0.445	A	0.639	1.057
6	rs1417842	119990080	A	0.4588	0.445	G	0.639	1.057
15	rs11635685	42373810	C	0.4433	0.4583	G	0.639	0.9411
16	rs76898487	68026486	C	0.03866	0.03428	A	0.639	1.133
22	rs11547731	50722134	A	0.4665	0.4516	G	0.639	1.062
8	rs4738757	60056229	A	0.4407	0.455	G	0.6391	0.9439
14	rs7141519	67524525	G	0.4407	0.455	A	0.6391	0.9439
2	rs2346177	46642249	G	0.4459	0.4608	A	0.6392	0.9414
2	rs492594	169764176	G	0.4613	0.4475	C	0.6392	1.057
3	rs12637558	193081122	C	0.4613	0.4475	A	0.6392	1.057
4	rs200612403	74364905	T	0.005155	0.00335	A	0.6392	1.541
5	rs7702187	9332281	A	0.2448	0.2583	T	0.6392	0.9309
17	rs3744793	76817090	G	0.4691	0.4542	A	0.6392	1.062
19	rs200728111	11660569	A	0.005155	0.00335	G	0.6392	1.541
6	rs998584	43757896	C	0.4716	0.4566	A	0.6393	1.062
12	rs11174574	63026358	A	0.4716	0.4567	G	0.6393	1.062
17	rs2241091	3101578	C	0.4485	0.4633	A	0.6393	0.942
17	rs12150427	3101691	C	0.4485	0.4633	A	0.6393	0.942
17	rs6503235	9515777	G	0.4716	0.4566	A	0.6393	1.062
4	rs7437940	7887500	G	0.1753	0.165	A	0.6394	1.075
4	rs1130643	88412806	G	0.116	0.1067	A	0.6394	1.099
6	rs2228210	12122174	G	0.2655	0.2525	A	0.6394	1.07
6	rs16893892	28239873	G	0.1753	0.165	A	0.6394	1.075
6	rs7758229	160840252	A	0.2655	0.2525	C	0.6394	1.07
9	rs10123583	1928115	A	0.4665	0.4525	G	0.6394	1.058
9	rs2275260	131285955	G	0.4665	0.4525	A	0.6394	1.058
16	rs9930333	53799977	C	0.1753	0.165	A	0.6394	1.075
16	rs1121980	53809247	A	0.1753	0.165	G	0.6394	1.075
19	rs6509544	52003331	C	0.4665	0.4525	G	0.6394	1.058
22	rs1547014	29100711	A	0.1753	0.165	G	0.6394	1.075
22	rs28379706	50728062	A	0.4665	0.4525	G	0.6394	1.058
1	rs664090	194066989	G	0.451	0.4658	A	0.6395	0.9421
6	rs3756987	159398700	A	0.4742	0.4592	G	0.6395	1.062
6	rs768994	159399348	G	0.4742	0.4592	A	0.6395	1.062
6	rs34582178	159407446	A	0.4742	0.4592	G	0.6395	1.062
6	rs16889320	159414899	G	0.4742	0.4592	A	0.6395	1.062
10	rs7913907	63849790	G	0.451	0.4658	A	0.6395	0.9421
20	rs1744769	35769592	A	0.451	0.4658	G	0.6395	0.9421

2	rs10174353	112447888	A	0.4485	0.4625	G	0.6396	0.9449
4	rs6823664	87326724	A	0.4536	0.4683	G	0.6396	0.9425
7	rs3735109	2649704	A	0.4768	0.4617	C	0.6396	1.063
12	rs10879513	73259114	A	0.4536	0.4683	C	0.6396	0.9425
19	rs12463334	40169328	G	0.4485	0.4625	A	0.6396	0.9449
5	rs2740588	140567264	C	0.4588	0.4741	A	0.6397	0.9401
6	rs2263316	31421297	G	0.5104	0.495	A	0.6397	1.063
11	rs11245723	50495067	C	0.4794	0.4642	A	0.6397	1.063
11	rs7115578	96000100	G	0.4794	0.4642	A	0.6397	1.063
17	rs12943640	11224534	A	0.4794	0.4642	G	0.6397	1.063
17	rs11869286	37813856	G	0.4742	0.46	C	0.6397	1.059
21	rs2826774	22666976	G	0.4794	0.4642	A	0.6397	1.063
5	rs3792794	150434722	G	0.4768	0.4625	A	0.6398	1.059
6	rs10484750	8378609	G	0.1753	0.1653	A	0.6398	1.073
6	rs3130553	31082285	G	0.4562	0.4708	A	0.6398	0.9428
6	rs6923492	146755324	G	0.482	0.4667	A	0.6398	1.063
7	rs10954696	82582846	G	0.4562	0.4708	A	0.6398	0.9428
7	rs4510766	107492789	A	0.482	0.4667	G	0.6398	1.063
7	rs7795646	120323727	A	0.4562	0.4708	G	0.6398	0.9428
10	rs2250303	79872834	A	0.4768	0.4625	G	0.6398	1.059
10	rs1269867	80849153	A	0.482	0.4667	G	0.6398	1.063
14	rs7141950	43960993	A	0.4768	0.4625	G	0.6398	1.059
16	rs837550	55562466	A	0.4768	0.4625	G	0.6398	1.059
4	rs11131511	64933220	A	0.4794	0.465	C	0.6399	1.059
17	rs3744395	6716242	A	0.4794	0.465	G	0.6399	1.059
1	rs599839	109822166	G	0.07216	0.065	A	0.64	1.119
2	rs2242073	208994045	C	0.07216	0.065	A	0.64	1.119
3	rs9845687	157682536	G	0.482	0.4675	A	0.64	1.06
6	rs9461684	31253444	A	0.07216	0.065	G	0.64	1.119
6	rs80169011	43969839	A	0.07216	0.065	C	0.64	1.119
6	rs9357655	50279484	A	0.4845	0.47	G	0.64	1.06
8	rs2947255	19076995	A	0.4845	0.47	G	0.64	1.06
10	rs11191454	104660004	G	0.2655	0.2533	A	0.64	1.065
11	rs75746929	100792335	A	0.2655	0.2533	G	0.64	1.065
14	rs35179634	65417070	C	0.4845	0.47	A	0.64	1.06
16	rs3743690	20635418	A	0.4639	0.4792	G	0.64	0.9406
4	rs13118279	162713356	A	0.4665	0.4817	C	0.6401	0.941
6	rs926557	151592056	A	0.4871	0.4725	C	0.6401	1.06
7	rs4338053	54318757	A	0.4665	0.4817	G	0.6401	0.941
8	rs3764795	143922620	A	0.4562	0.47	G	0.6401	0.946
9	rs11141018	88164213	C	0.4562	0.47	A	0.6401	0.946
10	rs10887621	88277457	A	0.1598	0.1717	G	0.6401	0.9177
13	rs7336806	27589593	A	0.4691	0.4842	G	0.6401	0.9413

16	rs7206703	57091977	G	0.1598	0.1717	A	0.6401	0.9177
20	rs235739	6778164	G	0.4639	0.4783	A	0.6401	0.9438
10	rs11245008	128193368	A	0.1606	0.1731	G	0.6402	0.9143
11	rs2075292	116732512	A	0.4897	0.475	C	0.6402	1.061
11	rs6589964	122870683	A	0.4923	0.4775	C	0.6402	1.061
14	rs6574512	26077514	A	0.4716	0.4867	G	0.6402	0.9416
1	rs146938681	225155286	A	0.03093	0.03833	G	0.6403	0.8006
2	rs61748231	152320892	A	0.03093	0.03833	G	0.6403	0.8006
3	rs13081389	12289800	G	0.03093	0.03833	A	0.6403	0.8006
3	rs7647305	185834290	A	0.03093	0.03833	G	0.6403	0.8006
4	rs794001	40121562	G	0.03093	0.03833	A	0.6403	0.8006
4	rs1553745	95644727	A	0.5	0.485	G	0.6403	1.062
5	rs3776096	140530529	G	0.4588	0.4725	A	0.6403	0.9463
6	rs2140418	34975415	G	0.4768	0.4917	A	0.6403	0.9422
6	rs16882046	90374283	G	0.03093	0.03833	A	0.6403	0.8006
6	rs41273327	90384274	G	0.03093	0.03833	A	0.6403	0.8006
6	rs9942473	118387296	G	0.5	0.485	A	0.6403	1.062
8	rs16916188	94746049	C	0.03093	0.03833	A	0.6403	0.8006
10	rs147595775	106130801	A	0.03093	0.03833	G	0.6403	0.8006
11	rs2239729	6977334	G	0.03093	0.03833	A	0.6403	0.8006
11	rs11550299	66254085	A	0.2474	0.2608	C	0.6403	0.9317
11	rs1695739	126196175	G	0.03093	0.03833	A	0.6403	0.8006
12	rs3741901	6030301	A	0.03093	0.03833	G	0.6403	0.8006
13	rs2297192	113720476	A	0.4974	0.4825	G	0.6403	1.062
14	rs4409996	48981279	G	0.4948	0.48	A	0.6403	1.061
14	rs35572669	77141290	A	0.4742	0.4892	C	0.6403	0.9419
15	rs33918653	65676726	A	0.4948	0.48	G	0.6403	1.061
18	rs2278792	29122799	A	0.4742	0.4892	G	0.6403	0.9419
1	rs17163774	223467316	A	0.4691	0.4833	C	0.6404	0.9444
5	rs1432865	150951581	G	0.4845	0.4708	A	0.6404	1.056
6	rs241439	32797537	A	0.482	0.4683	C	0.6404	1.056
7	rs2595644	43980540	C	0.4794	0.4942	A	0.6404	0.9425
8	rs6987138	29850729	A	0.482	0.4683	G	0.6404	1.056
18	rs6507387	38593162	G	0.5052	0.49	A	0.6404	1.062
18	rs608713	47379430	A	0.482	0.4967	G	0.6404	0.9428
19	rs330880	6897464	G	0.5052	0.49	C	0.6404	1.062
19	rs1105561	44669960	A	0.5077	0.4925	G	0.6404	1.063
2	rs2724863	477364	A	0.4716	0.4858	G	0.6405	0.9447
2	rs2322659	136555659	A	0.4871	0.5017	G	0.6405	0.9434
5	rs4867353	31603202	A	0.4742	0.4883	C	0.6405	0.9451
6	rs9482017	120583762	G	0.4923	0.4783	A	0.6405	1.057
7	rs6970002	11704583	A	0.4897	0.4758	G	0.6405	1.057
9	rs3810936	117552885	A	0.5103	0.4958	G	0.6405	1.06

11	rs2277302	66240882	G	0.2474	0.26	A	0.6405	0.9357
11	rs10047462	116722041	A	0.4923	0.4783	C	0.6405	1.057
12	rs3751303	133158650	A	0.1778	0.1667	G	0.6405	1.082
13	rs2039622	40740239	A	0.4897	0.4758	G	0.6405	1.057
13	rs4148546	95680285	A	0.4871	0.5017	G	0.6405	0.9434
15	rs11630449	31614741	G	0.2474	0.26	A	0.6405	0.9357
20	rs4468878	59928237	A	0.4742	0.4883	G	0.6405	0.9451
21	rs2222999	41425831	G	0.4716	0.4858	A	0.6405	0.9447
2	rs10496236	80299354	C	0.5052	0.4908	A	0.6406	1.059
2	rs733112	125061838	A	0.5077	0.4933	C	0.6406	1.059
4	rs6815111	44531148	A	0.4974	0.4833	G	0.6406	1.058
4	rs344140	77660162	C	0.5052	0.4908	G	0.6406	1.059
7	rs2551762	135162927	G	0.5	0.4858	A	0.6406	1.058
11	rs1395558	6727468	G	0.5	0.4858	A	0.6406	1.058
11	rs11229158	55111238	C	0.4948	0.4808	A	0.6406	1.058
11	rs10896659	55111584	A	0.4948	0.4808	T	0.6406	1.058
22	rs5747933	18910355	A	0.1778	0.1669	C	0.6406	1.079
3	rs12635131	143360568	A	0.4794	0.4933	C	0.6407	0.9457
10	rs11414	3180316	G	0.482	0.4958	A	0.6408	0.946
14	rs1265879	50877983	A	0.116	0.1075	G	0.6408	1.089
15	rs4966020	99284680	G	0.4845	0.4983	A	0.6408	0.9463
16	rs77260498	815565	G	0.116	0.1075	A	0.6408	1.089
2	rs1527986	12621757	G	0.4871	0.5008	A	0.6409	0.9466
19	rs867228	52249211	A	0.25	0.2633	C	0.6414	0.9325
6	rs1991355	48608329	G	0.2706	0.2575	A	0.6415	1.07
13	rs6561030	42631719	A	0.2706	0.2575	C	0.6415	1.07
14	rs9989177	50092471	G	0.1031	0.1133	A	0.642	0.8993
9	rs17057133	74319677	G	0.1031	0.1125	A	0.6421	0.9068
12	rs1005902	112667675	A	0.1031	0.1125	C	0.6421	0.9068
10	rs12571093	70019371	A	0.1804	0.1692	G	0.6424	1.081
20	rs2236200	60986019	C	0.1804	0.1692	A	0.6424	1.081
1	rs1935881	190066386	G	0.1186	0.1092	A	0.6427	1.098
2	rs76832527	242157241	A	0.1186	0.1092	G	0.6427	1.098
6	rs9362667	90340187	A	0.1186	0.1092	G	0.6427	1.098
2	rs2247197	204306433	G	0.07216	0.06583	A	0.6429	1.104
8	rs17847126	95272598	A	0.07216	0.06583	G	0.6429	1.104
15	rs35985016	101529012	G	0.07216	0.06583	A	0.6429	1.104
16	rs749670	31088625	A	0.07216	0.06583	G	0.6429	1.104
18	rs2856966	907710	G	0.07216	0.06583	A	0.6429	1.104
2	rs139834711	165551128	A	0.01804	0.015	G	0.6434	1.206
4	rs79116599	108953492	A	0.01804	0.015	G	0.6434	1.206
5	rs201469505	13916528	A	0.01804	0.015	C	0.6434	1.206
5	rs41294552	137910932	A	0.01804	0.015	G	0.6434	1.206

5	rs117187367	178540998	A	0.01804	0.015	C	0.6434	1.206
5	rs147342327	179069943	A	0.01804	0.015	C	0.6434	1.206
6	rs3752528	32942264	G	0.01804	0.015	A	0.6434	1.206
7	rs10269582	21640361	G	0.01804	0.015	A	0.6434	1.206
7	rs61735496	36396593	G	0.01804	0.015	A	0.6434	1.206
10	rs189652490	5762725	A	0.01804	0.015	G	0.6434	1.206
10	rs3814196	5789000	A	0.01804	0.015	G	0.6434	1.206
10	rs1727	91066769	C	0.01804	0.015	A	0.6434	1.206
11	rs186087116	77884952	G	0.01804	0.015	A	0.6434	1.206
11	rs138057144	92534497	A	0.01804	0.015	G	0.6434	1.206
12	rs116587731	21427515	G	0.01804	0.015	A	0.6434	1.206
14	rs142255517	105452941	A	0.01804	0.015	G	0.6434	1.206
16	rs117840561	57075962	A	0.01804	0.015	C	0.6434	1.206
17	rs200779134	8167178	A	0.01804	0.015	G	0.6434	1.206
17	rs75425060	18880986	A	0.01804	0.015	G	0.6434	1.206
19	rs2228539	6926378	G	0.01804	0.015	A	0.6434	1.206
20	rs200803813	60704940	A	0.01804	0.015	C	0.6434	1.206
22	rs143525991	42611265	C	0.01804	0.015	G	0.6434	1.206
6	rs72502537	30995894	A	0.06186	0.06951	T	0.6435	0.8826
6	rs2279463	160668389	G	0.06186	0.07	A	0.6435	0.876
9	rs10975061	543631	G	0.2552	0.2683	A	0.6435	0.9341
17	rs236531	68217860	G	0.2552	0.2683	A	0.6435	0.9341
3	rs3816877	49719799	A	0.04124	0.03583	G	0.6436	1.157
9	rs1556189	125390874	A	0.04124	0.03583	C	0.6436	1.157
9	rs3739916	135277007	A	0.04124	0.03583	G	0.6436	1.157
10	rs11591355	21415006	G	0.2552	0.2692	A	0.6436	0.9301
14	rs145716748	45693605	G	0.04124	0.03583	A	0.6436	1.157
14	rs138267500	45711988	A	0.04124	0.03583	C	0.6436	1.157
15	rs1517618	92647645	G	0.04124	0.03583	C	0.6436	1.157
16	rs61734902	27761040	A	0.04124	0.03583	G	0.6436	1.157
17	rs12150220	5485367	A	0.04124	0.03583	T	0.6436	1.157
17	rs3110496	27917771	A	0.2552	0.2692	G	0.6436	0.9301
19	rs17426435	11546269	A	0.04124	0.03583	G	0.6436	1.157
19	rs8104808	52887427	C	0.2552	0.2692	A	0.6436	0.9301
10	rs12261843	35554054	C	0.2552	0.2675	A	0.6438	0.938
20	rs79267778	62194464	A	0.04124	0.03589	G	0.6439	1.155
2	rs10178458	228111435	A	0.1186	0.11	G	0.644	1.088
2	rs6436669	228113175	A	0.1186	0.11	G	0.644	1.088
4	rs6599389	939113	A	0.2758	0.2633	G	0.644	1.065
5	rs2303080	7878424	T	0.1186	0.11	A	0.644	1.088
9	rs4076794	90501514	C	0.2758	0.2633	G	0.644	1.065
10	rs10994336	62179812	A	0.2758	0.2633	G	0.644	1.065
10	rs10994338	62181128	A	0.2758	0.2633	G	0.644	1.065

10	rs2986035	105233110	G	0.1649	0.177	A	0.644	0.9187
1	rs16835244	33562416	A	0.06186	0.07083	C	0.6441	0.8649
1	rs75105221	244723989	A	0.06186	0.07083	C	0.6441	0.8649
1	rs2072671	20915701	C	0.1649	0.1758	A	0.6442	0.9259
8	rs10091038	29360305	A	0.2577	0.2708	C	0.6445	0.9348
2	rs1064213	198950240	A	0.2577	0.2717	G	0.6446	0.9309
10	rs10905900	11143281	G	0.2784	0.2658	A	0.645	1.065
2	rs2278196	179400895	A	0.183	0.1725	G	0.6451	1.074
2	rs72648270	179404628	A	0.183	0.1725	T	0.6451	1.074
7	rs20556	107593989	A	0.183	0.1725	G	0.6451	1.074
11	rs10765565	92573911	A	0.183	0.1725	C	0.6451	1.074
4	rs3088248	6623332	G	0.1057	0.1158	A	0.6453	0.9019
6	rs9455487	169248186	A	0.1057	0.115	G	0.6454	0.9093
12	rs4767364	112521448	G	0.1057	0.115	A	0.6454	0.9093
9	rs706107	33240225	A	0.2603	0.2733	G	0.6455	0.9356
6	rs2323150	81913895	G	0.2603	0.2742	A	0.6456	0.9317
1	rs2236378	19422060	A	0.07474	0.0675	G	0.6457	1.116
1	rs12061304	111237685	G	0.07474	0.0675	A	0.6457	1.116
1	rs3814299	152275453	A	0.07474	0.0675	G	0.6457	1.116
2	rs2278358	43968167	A	0.07474	0.0675	G	0.6457	1.116
6	rs7755364	30981715	A	0.07474	0.0675	C	0.6457	1.116
9	rs3780679	123205912	C	0.07474	0.0675	G	0.6457	1.116
9	rs34523498	123205981	A	0.07474	0.0675	G	0.6457	1.116
13	rs8000245	74730392	A	0.07474	0.0675	G	0.6457	1.116
14	rs1256149	64935720	G	0.07474	0.0675	A	0.6457	1.116
19	rs745961	33370070	G	0.07474	0.0675	A	0.6457	1.116
19	rs745960	33370084	A	0.07474	0.0675	T	0.6457	1.116
3	rs2290972	42738594	A	0.1211	0.1117	G	0.6459	1.096
15	rs715467	27799223	A	0.2809	0.2683	G	0.6459	1.065
16	rs7499892	57006590	A	0.1675	0.1792	G	0.6459	0.922
2	rs1448536	177421669	A	0.1675	0.1786	G	0.646	0.9253
20	rs6056209	8836771	A	0.1675	0.1783	G	0.6462	0.9272
1	rs7554607	237266603	G	0.2629	0.2758	A	0.6465	0.9363
4	rs6817993	24438686	A	0.2629	0.2767	C	0.6465	0.9324
9	rs2274654	98691137	G	0.2629	0.2758	A	0.6465	0.9363
5	rs6874609	74069863	G	0.1856	0.175	A	0.6469	1.074
14	rs1040835	93276654	A	0.1856	0.175	G	0.6469	1.074
1	rs7551421	109369915	A	0.2921	0.2795	C	0.647	1.064
14	rs8014194	95720678	T	0.1211	0.1125	A	0.6471	1.087
19	rs77857083	58718332	A	0.1211	0.1125	G	0.6471	1.087
2	rs231775	204732714	A	0.2835	0.2717	G	0.6475	1.061
3	rs9822683	97462795	A	0.2835	0.2717	G	0.6475	1.061
12	rs7979575	24278821	C	0.2655	0.2783	G	0.6475	0.9371

5	rs2279804	156479204	A	0.2861	0.2733	G	0.6477	1.065
11	rs1382774	79980044	C	0.2861	0.2733	A	0.6477	1.065
8	rs6474412	42550498	G	0.1701	0.1817	A	0.6478	0.9233
17	rs2382644	72346868	A	0.1701	0.1817	G	0.6478	0.9233
6	rs926070	32257566	G	0.1701	0.1825	A	0.648	0.9181
3	rs17079425	47036565	A	0.1881	0.177	G	0.6481	1.078
3	rs76208147	47162886	A	0.1881	0.177	G	0.6481	1.078
6	rs314277	105407662	A	0.07474	0.06833	C	0.6483	1.101
7	rs7807771	85148963	G	0.07474	0.06833	A	0.6483	1.101
7	rs3757645	111426682	A	0.07474	0.06833	G	0.6483	1.101
20	rs6060750	34596371	A	0.2861	0.2742	G	0.6483	1.061
2	rs7584993	223845942	C	0.268	0.2817	A	0.6484	0.9339
20	rs932826	62380516	A	0.268	0.2817	C	0.6484	0.9339
11	rs3888798	88027209	G	0.1082	0.1183	A	0.6485	0.9044
15	rs2624265	39148649	G	0.268	0.2808	A	0.6485	0.9378
17	rs704	26694861	G	0.268	0.2808	A	0.6485	0.9378
18	rs17077540	65285279	G	0.1082	0.1183	A	0.6485	0.9044
1	rs16861990	169135127	C	0.04124	0.03667	A	0.6486	1.13
1	rs3730238	201330429	G	0.04124	0.03667	A	0.6486	1.13
2	rs3087399	100055158	G	0.04124	0.03667	A	0.6486	1.13
2	rs78386861	207171102	A	0.04124	0.03667	G	0.6486	1.13
3	rs2307311	127336910	A	0.04124	0.03667	G	0.6486	1.13
10	rs36152134	74896664	G	0.2887	0.2758	A	0.6486	1.065
14	rs2236235	68029313	A	0.2887	0.2758	G	0.6486	1.065
14	rs7160694	81382845	A	0.04124	0.03667	C	0.6486	1.13
17	rs28641831	73236124	G	0.04124	0.03667	A	0.6486	1.13
4	rs6414624	5743512	A	0.1237	0.1142	G	0.649	1.095
6	rs7745023	121577370	G	0.06443	0.0725	A	0.6491	0.8811
7	rs6963781	88965609	G	0.06443	0.0725	A	0.6491	0.8811
8	rs2977020	52321243	A	0.06443	0.0725	G	0.6491	0.8811
12	rs2388981	32134207	A	0.06443	0.07262	C	0.6491	0.8795
10	rs3736518	74899134	G	0.2887	0.2767	C	0.6492	1.061
10	rs3812619	74923562	A	0.2887	0.2767	G	0.6492	1.061
3	rs854208	45452578	G	0.2912	0.278	A	0.6493	1.067
8	rs3134031	94808165	A	0.2706	0.2833	G	0.6494	0.9385
7	rs6962993	88965141	G	0.06443	0.07333	A	0.6496	0.8703
11	rs117709265	67401660	A	0.01289	0.01759	G	0.6496	0.7292
12	rs1057994	32137565	A	0.06443	0.07333	G	0.6496	0.8703
18	rs79615555	12698983	A	0.06443	0.07333	G	0.6496	0.8703
5	rs11959928	39397132	T	0.1881	0.1783	A	0.6497	1.068
1	rs2297595	98165091	G	0.01289	0.0175	A	0.6498	0.7329
1	rs2274992	154209611	A	0.01289	0.0175	G	0.6498	0.7329
1	rs35448215	179631242	A	0.03351	0.04	G	0.6498	0.832

1	rs150188026	186140508	A	0.01289	0.0175	G	0.6498	0.7329
2	rs3755073	45640374	A	0.1727	0.185	C	0.6498	0.9195
3	rs117013868	38105366	G	0.03351	0.04	C	0.6498	0.832
3	rs2291090	124515646	C	0.01289	0.0175	A	0.6498	0.7329
3	rs148403666	133327432	A	0.01289	0.0175	C	0.6498	0.7329
4	rs16844152	3318726	A	0.01289	0.0175	T	0.6498	0.7329
4	rs7669299	78447144	G	0.03351	0.04	A	0.6498	0.832
5	rs142324076	66480051	G	0.01289	0.0175	A	0.6498	0.7329
6	rs58690463	27368874	G	0.01289	0.0175	A	0.6498	0.7329
6	rs894124	159096121	G	0.03351	0.04	A	0.6498	0.832
7	rs181998278	1484922	A	0.01289	0.0175	G	0.6498	0.7329
7	rs192511474	4874645	A	0.01289	0.0175	G	0.6498	0.7329
8	rs9987289	9183358	A	0.01289	0.0175	G	0.6498	0.7329
8	rs4841132	9183596	A	0.01289	0.0175	G	0.6498	0.7329
8	rs183758075	39550180	A	0.01289	0.0175	G	0.6498	0.7329
8	rs150347257	134239746	A	0.01289	0.0175	G	0.6498	0.7329
8	rs150994390	144621058	A	0.01289	0.01753	G	0.6498	0.7317
9	rs139477472	112705112	A	0.01289	0.0175	G	0.6498	0.7329
11	rs150685279	75902734	A	0.01289	0.0175	G	0.6498	0.7329
14	rs147041732	24654455	C	0.01289	0.0175	G	0.6498	0.7329
16	rs2301426	711905	A	0.01289	0.0175	G	0.6498	0.7329
16	rs75977701	3304158	A	0.01289	0.0175	G	0.6498	0.7329
16	rs78384770	4764117	C	0.01289	0.0175	A	0.6498	0.7329
17	rs112980285	8047081	A	0.01289	0.0175	G	0.6498	0.7329
18	rs117342470	3071878	A	0.01289	0.0175	G	0.6498	0.7329
19	rs185076989	38877754	G	0.01289	0.0175	A	0.6498	0.7329
19	rs77075347	57027673	G	0.01289	0.0175	A	0.6498	0.7329
19	rs138019714	58058561	C	0.01289	0.0175	G	0.6498	0.7329
11	rs10833411	20931291	G	0.2912	0.2792	A	0.6501	1.061
2	rs13391759	108217756	A	0.2732	0.2867	C	0.6502	0.9353
6	rs9347162	167271716	A	0.1237	0.115	G	0.6502	1.086
11	rs34134444	1972143	A	0.1237	0.115	G	0.6502	1.086
15	rs950776	78926018	G	0.1237	0.115	A	0.6502	1.086
3	rs4402960	185511687	A	0.2938	0.2808	C	0.6504	1.065
3	rs7651090	185513392	G	0.2938	0.2808	A	0.6504	1.065
5	rs10062843	160127677	A	0.1907	0.18	G	0.6504	1.074
6	rs886381	29500225	A	0.1907	0.18	G	0.6504	1.074
16	rs3803704	71318577	G	0.2732	0.2858	A	0.6504	0.9392
6	rs2180314	52617731	G	0.2938	0.2813	C	0.6506	1.063
1	rs1329424	196646176	A	0.07732	0.07	C	0.651	1.113
1	rs380390	196701051	G	0.07732	0.07	C	0.651	1.113
2	rs35226268	88876094	A	0.03351	0.04083	G	0.651	0.8143
5	rs79487218	35753763	A	0.03351	0.04083	G	0.651	0.8143

6	rs2229385	49415448	A	0.07732	0.07	G	0.651	1.113
12	rs665470	52946498	A	0.03351	0.04083	G	0.651	0.8143
12	rs665522	52946543	A	0.03351	0.04083	G	0.651	0.8143
19	rs79591246	4818295	A	0.03351	0.04083	G	0.651	0.8143
19	rs117511354	18368181	A	0.03351	0.04083	T	0.651	0.8143
6	rs3134768	31206844	G	0.2995	0.2867	C	0.6511	1.064
19	rs2967614	8458145	A	0.2964	0.2833	G	0.6512	1.066
1	rs671108	55266797	C	0.2758	0.2883	G	0.6513	0.9399
3	rs9843784	149468530	C	0.1753	0.1867	A	0.6514	0.9259
6	rs2817937	121098457	G	0.01295	0.01833	A	0.6514	0.7027
7	rs1568731	4665570	A	0.1907	0.1808	G	0.6514	1.068
12	rs1800693	6440009	G	0.1907	0.1808	A	0.6514	1.068
12	rs1021672	24177550	A	0.2964	0.2838	C	0.6514	1.063
13	rs2274407	95859035	A	0.1907	0.1808	C	0.6514	1.068
11	rs7107487	124908352	G	0.1753	0.1875	A	0.6515	0.9208
12	rs79387839	103696182	C	0.1753	0.1875	A	0.6515	0.9208
14	rs4027402	64496749	G	0.1753	0.1875	A	0.6515	0.9208
1	rs150408858	55252738	A	0.01289	0.01833	G	0.6516	0.699
1	rs137949054	159159647	G	0.01289	0.01833	A	0.6516	0.699
2	rs146172396	80529738	A	0.01289	0.01833	C	0.6516	0.699
3	rs2228561	48628014	A	0.01289	0.01833	G	0.6516	0.699
3	rs181256798	113342281	G	0.01289	0.01833	A	0.6516	0.699
4	rs141092310	38045998	A	0.01289	0.01833	G	0.6516	0.699
5	rs199704720	139938369	A	0.01289	0.01833	G	0.6516	0.699
5	rs117586833	176070216	A	0.01289	0.01833	G	0.6516	0.699
6	rs22662	5183536	G	0.01289	0.01833	A	0.6516	0.699
6	rs61745766	150174278	C	0.01289	0.01833	A	0.6516	0.699
7	rs6977165	99269397	G	0.01289	0.01833	A	0.6516	0.699
8	rs6601299	9184691	A	0.01289	0.01833	G	0.6516	0.699
8	rs2126259	9185146	A	0.01289	0.01833	G	0.6516	0.699
11	rs61730422	5989223	A	0.01289	0.01833	G	0.6516	0.699
11	rs186615197	99932010	T	0.01289	0.01833	A	0.6516	0.699
13	rs9508908	20398742	G	0.01289	0.01833	A	0.6516	0.699
14	rs2234919	81422178	A	0.01289	0.01833	C	0.6516	0.699
19	rs61741212	36157740	A	0.01289	0.01833	G	0.6516	0.699
19	rs200409216	51190068	A	0.01289	0.01833	G	0.6516	0.699
20	rs141475072	826140	A	0.01289	0.01833	C	0.6516	0.699
20	rs200551395	3785604	G	0.01289	0.01833	A	0.6516	0.699
6	rs1611350	29698821	G	0.2964	0.2842	A	0.6517	1.061
21	rs2205204	41137507	G	0.2964	0.2842	C	0.6517	1.061
4	rs11736872	177100644	A	0.1108	0.12	G	0.6518	0.914
17	rs11077969	80085633	G	0.1108	0.12	A	0.6518	0.914
17	rs7406163	80086389	A	0.1108	0.12	G	0.6518	0.914

17	rs4625783	80086395	G	0.1108	0.12	A	0.6518	0.914
17	rs7209474	80136418	G	0.1108	0.12	A	0.6518	0.914
3	rs10490823	41123735	A	0.2784	0.2917	G	0.652	0.9367
6	rs146057838	32713693	C	0.1263	0.1167	A	0.652	1.094
8	rs2843894	36025847	G	0.2784	0.2917	A	0.652	0.9367
19	rs62118272	9024871	A	0.1263	0.1167	G	0.652	1.094
6	rs1736991	29766926	C	0.299	0.2858	G	0.6521	1.066
6	rs1214752	43396325	G	0.299	0.2858	A	0.6521	1.066
8	rs877386	19221664	C	0.1933	0.1825	G	0.6521	1.073
14	rs4906172	102454933	A	0.299	0.2867	C	0.6525	1.061
1	rs10746514	232246027	A	0.2809	0.2942	G	0.6528	0.9374
10	rs1913517	50119054	A	0.2809	0.2942	G	0.6528	0.9374
11	rs611530	81979892	A	0.2809	0.2942	G	0.6528	0.9374
12	rs4765937	2570535	A	0.2809	0.2942	G	0.6528	0.9374
3	rs1995641	44943389	G	0.1933	0.1833	A	0.653	1.067
22	rs1012068	32265903	C	0.1933	0.1833	A	0.653	1.067
5	rs7705355	1225687	G	0.2809	0.2933	A	0.6531	0.9412
5	rs1320021	76219181	G	0.1263	0.1175	A	0.6531	1.086
5	rs286592	154376097	A	0.2809	0.2933	G	0.6531	0.9412
6	rs3016539	162236075	G	0.1263	0.1175	A	0.6531	1.086
7	rs6943029	155762157	A	0.2809	0.2933	G	0.6531	0.9412
13	rs9562353	42385446	G	0.1263	0.1175	A	0.6531	1.086
15	rs35043215	40659476	A	0.1263	0.1175	G	0.6531	1.086
19	rs3826656	51726613	A	0.2809	0.2933	G	0.6531	0.9412
5	rs15251	149776232	A	0.1778	0.1892	G	0.6532	0.9271
7	rs17847825	106509331	A	0.1778	0.1892	C	0.6532	0.9271
15	rs4072111	81578139	A	0.1778	0.1892	G	0.6532	0.9271
2	rs2270669	238243464	C	0.3015	0.2892	G	0.6533	1.061
3	rs34423045	194387253	G	0.07732	0.07083	A	0.6534	1.099
6	rs1342642	137323213	A	0.07732	0.07083	G	0.6534	1.099
11	rs10768448	5020799	A	0.07732	0.07083	G	0.6534	1.099
11	rs10768450	5020832	A	0.07732	0.07083	G	0.6534	1.099
5	rs2569006	145246085	A	0.2835	0.2972	C	0.6536	0.9359
1	rs11749	38023316	A	0.3041	0.2908	G	0.6537	1.066
1	rs10788819	151895093	A	0.3041	0.2908	G	0.6537	1.066
2	rs740393	242065498	G	0.2835	0.2967	A	0.6537	0.9381
5	rs348943	112823998	G	0.3041	0.2908	A	0.6537	1.066
10	rs4494250	96563757	A	0.1959	0.185	G	0.6537	1.073
13	rs1375719	103410782	G	0.2835	0.2967	A	0.6537	0.9381
13	rs12855785	103410914	A	0.2835	0.2967	T	0.6537	0.9381
8	rs76337474	142199229	A	0.04381	0.03833	G	0.6538	1.15
10	rs11253684	15943346	A	0.04381	0.03833	G	0.6538	1.15
14	rs4901869	59334128	G	0.04381	0.03833	A	0.6538	1.15

19	rs17697947	10449514	G	0.07732	0.07095	A	0.6538	1.097
19	rs1042164	13264398	A	0.04381	0.03833	G	0.6538	1.15
2	rs2280334	66665146	G	0.2861	0.3	A	0.6545	0.935
6	rs1159148	106967185	C	0.06701	0.075	A	0.6545	0.8858
14	rs3742884	68251934	A	0.06701	0.075	G	0.6545	0.8858
8	rs1042381	97614661	A	0.1959	0.1858	T	0.6546	1.067
10	rs1049125	118368606	G	0.1134	0.1229	A	0.6546	0.9128
13	rs9577230	113975768	G	0.1134	0.1233	A	0.6546	0.9092
3	rs1127717	125826059	G	0.1263	0.1183	A	0.6547	1.077
3	rs2229032	142178144	A	0.1263	0.1183	G	0.6547	1.077
1	rs2304303	55064980	A	0.1804	0.1915	G	0.6548	0.9295
2	rs78774163	182780126	A	0.06701	0.07583	G	0.6548	0.8753
6	rs2071465	32805470	C	0.3041	0.2925	G	0.6548	1.057
6	rs3799260	53519605	G	0.06701	0.07583	A	0.6548	0.8753
22	rs739134	42089623	A	0.06701	0.07583	G	0.6548	0.8753
12	rs701097	125179253	A	0.3067	0.2942	G	0.6549	1.061
14	rs61755995	51219349	A	0.1804	0.1925	G	0.6549	0.9234
19	rs3752109	59082605	A	0.3067	0.2942	G	0.6549	1.061
10	rs1341667	70641860	A	0.1134	0.1242	G	0.655	0.9022
13	rs9593132	76395620	A	0.1134	0.1242	G	0.655	0.9022
16	rs74412242	55360297	A	0.1134	0.1242	G	0.655	0.9022
16	rs9282681	89805914	G	0.1134	0.1242	A	0.655	0.9022
1	rs61733130	55248120	G	0.0799	0.07215	A	0.6552	1.117
10	rs7088963	92905682	G	0.2887	0.3025	A	0.6553	0.9357
5	rs7709485	145894896	G	0.1985	0.1875	C	0.6554	1.073
2	rs1622455	184298771	A	0.3067	0.295	C	0.6555	1.057
6	rs1610601	29700183	A	0.3093	0.2967	C	0.6556	1.062
8	rs4471028	75294975	C	0.3093	0.2967	A	0.6556	1.062
6	rs10947230	32024395	A	0.0799	0.0725	G	0.6561	1.111
6	rs9501234	32876049	G	0.0799	0.0725	A	0.6561	1.111
6	rs154986	32879233	A	0.0799	0.0725	C	0.6561	1.111
7	rs79809732	48321010	A	0.0799	0.0725	G	0.6561	1.111
5	rs16893687	60982841	G	0.3093	0.2975	A	0.6562	1.057
17	rs854625	34328461	A	0.3093	0.2975	G	0.6562	1.057
6	rs2517911	29684634	G	0.3119	0.2992	A	0.6564	1.062
5	rs75331699	121488636	D	0.183	0.1942	I	0.6566	0.9295
6	rs2571402	29932380	T	0.3119	0.3	A	0.6569	1.057
11	rs220859	115312071	G	0.201	0.19	A	0.6569	1.073
1	rs118113115	115316921	G	0.02062	0.01667	A	0.6571	1.242
1	rs148428979	203452341	A	0.02062	0.01667	G	0.6571	1.242
2	rs12619169	103011084	A	0.02062	0.01667	G	0.6571	1.242
2	rs140192021	165802112	A	0.02062	0.01667	G	0.6571	1.242
2	rs11892031	234565283	C	0.02062	0.01667	A	0.6571	1.242

4	rs140432368	30726226	A	0.02062	0.01667	G	0.6571	1.242
5	rs116774010	133914380	A	0.02062	0.01667	C	0.6571	1.242
6	rs241428	32804070	C	0.02062	0.01667	A	0.6571	1.242
7	rs41279639	44611235	G	0.02062	0.01667	A	0.6571	1.242
8	rs61732273	74893419	C	0.02062	0.01667	G	0.6571	1.242
9	rs111936933	111692143	A	0.02062	0.01667	G	0.6571	1.242
9	rs141843004	139734176	A	0.02062	0.01667	G	0.6571	1.242
10	rs17143175	5790116	A	0.02062	0.01667	G	0.6571	1.242
10	rs138649767	114925441	A	0.02062	0.01667	G	0.6571	1.242
12	rs187818061	56994614	A	0.02062	0.01667	C	0.6571	1.242
15	rs118084625	78487042	A	0.02062	0.01667	G	0.6571	1.242
20	rs1133358	31590686	A	0.02062	0.01667	C	0.6571	1.242
4	rs3796623	983625	A	0.02062	0.01669	G	0.6573	1.24
3	rs4594610	1014666	A	0.2979	0.3114	G	0.6574	0.9386
2	rs17043947	22736987	A	0.1289	0.1208	G	0.6575	1.076
11	rs17350764	5068431	A	0.116	0.1258	G	0.6575	0.9114
14	rs10149146	102901201	G	0.116	0.1258	A	0.6575	0.9114
16	rs181865961	4802423	A	0.02062	0.01672	G	0.6575	1.238
7	rs2734880	100371100	A	0.1166	0.1267	C	0.6576	0.9099
18	rs337718	69774278	A	0.317	0.3038	G	0.6576	1.063
7	rs11766982	151693502	G	0.2964	0.3092	A	0.6577	0.9413
14	rs10139660	35173624	G	0.2964	0.3092	A	0.6577	0.9413
18	rs1871510	19589336	G	0.2964	0.3092	A	0.6577	0.9413
20	rs3810562	3218634	C	0.2964	0.3092	G	0.6577	0.9413
1	rs425277	2069172	A	0.201	0.1908	G	0.6578	1.067
2	rs1429148	216923679	A	0.317	0.3042	G	0.6578	1.062
3	rs74717885	38805069	G	0.116	0.1267	A	0.6578	0.9046
13	rs1324015	43727849	A	0.317	0.3047	G	0.658	1.059
1	rs151133441	7723593	G	0.04381	0.03917	A	0.6582	1.124
1	rs12025126	8759554	A	0.1856	0.1975	G	0.6582	0.9258
1	rs986027	48713394	G	0.1856	0.1967	A	0.6582	0.9307
1	rs11204538	248046272	G	0.1856	0.1975	A	0.6582	0.9258
2	rs13026308	220353532	A	0.04381	0.03917	C	0.6582	1.124
3	rs7617596	71521494	A	0.04381	0.03917	G	0.6582	1.124
3	rs6438884	124938103	A	0.04381	0.03917	G	0.6582	1.124
5	rs3749760	141026182	A	0.04381	0.03917	C	0.6582	1.124
7	rs7791767	149513152	A	0.1856	0.1967	G	0.6582	0.9307
8	rs160441	90656988	A	0.1856	0.1967	G	0.6582	0.9307
10	rs143714645	75188033	A	0.04381	0.03917	G	0.6582	1.124
14	rs1713457	20850156	C	0.04381	0.03917	G	0.6582	1.124
19	rs139528870	15793213	A	0.04381	0.03917	C	0.6582	1.124
19	rs12611254	58965660	G	0.04381	0.03917	A	0.6582	1.124
21	rs61732502	38309459	A	0.04381	0.03917	G	0.6582	1.124

1	rs3737657	204110547	A	0.0799	0.07333	G	0.6583	1.097
19	rs8108738	18255359	G	0.317	0.305	A	0.6583	1.058
8	rs4921617	18782047	G	0.3196	0.3067	A	0.6585	1.062
9	rs8176749	136131188	A	0.2036	0.1925	G	0.6585	1.072
2	rs1919126	27801418	C	0.1314	0.1225	A	0.6587	1.084
5	rs2304024	150908813	A	0.1314	0.1225	G	0.6587	1.084
8	rs516946	41519248	A	0.1314	0.1225	G	0.6587	1.084
9	rs3812553	139256495	A	0.0799	0.07346	C	0.6587	1.095
10	rs869244	112909105	A	0.3196	0.3075	G	0.659	1.058
1	rs1998598	197727642	G	0.2036	0.1933	A	0.6593	1.067
2	rs2288101	31135184	A	0.2036	0.1933	C	0.6593	1.067
5	rs4705411	149431025	A	0.2036	0.1933	G	0.6593	1.067
11	rs7935564	5718517	A	0.2036	0.1933	G	0.6593	1.067
12	rs774047	56815922	G	0.3255	0.3133	A	0.6594	1.058
16	rs4280262	11647492	G	0.06959	0.0775	A	0.6596	0.8903
6	rs2308488	31323184	A	0.03608	0.04257	G	0.6597	0.8419
12	rs11047102	23946146	A	0.03608	0.04257	G	0.6597	0.8419
15	rs1436955	62404382	A	0.3222	0.31	G	0.6597	1.058
16	rs1046502	2035868	A	0.3222	0.31	G	0.6597	1.058
5	rs11168048	147842353	A	0.3041	0.318	G	0.6598	0.9372
6	rs9328202	3887339	A	0.03608	0.0425	C	0.6598	0.8433
7	rs150524780	127729628	A	0.03608	0.0425	G	0.6598	0.8433
12	rs7138562	4229988	A	0.06959	0.07833	G	0.6598	0.88
13	rs2289909	25283596	A	0.03608	0.0425	G	0.6598	0.8433
19	rs75607624	12059699	C	0.03608	0.0425	A	0.6598	0.8433
5	rs737154	1065399	G	0.3247	0.3117	A	0.6599	1.062
12	rs1558744	68504592	A	0.1314	0.1233	G	0.6602	1.076
13	rs3106600	61683209	C	0.3247	0.3125	A	0.6603	1.058
14	rs3742424	102391577	C	0.3247	0.3125	G	0.6603	1.058
1	rs6603855	16460541	G	0.1186	0.1283	A	0.6604	0.9136
18	rs3862712	59780393	A	0.1186	0.1283	T	0.6604	0.9136
6	rs2022544	32321004	A	0.3067	0.32	G	0.6605	0.9401
6	rs7770048	32334754	A	0.3067	0.32	G	0.6605	0.9401
11	rs60847789	128839526	C	0.03627	0.04333	G	0.6605	0.8309
1	rs3085	207857254	G	0.3067	0.3192	A	0.6607	0.9437
5	rs11575948	140724060	C	0.03608	0.04333	A	0.6607	0.8264
5	rs11575949	140735215	A	0.03608	0.04333	G	0.6607	0.8264
5	rs62378417	140741738	G	0.03608	0.04333	C	0.6607	0.8264
12	rs74528155	109877506	A	0.03608	0.04333	G	0.6607	0.8264
15	rs8179065	90196008	A	0.3067	0.3192	G	0.6607	0.9437
15	rs2715423	99511873	A	0.03608	0.04333	G	0.6607	0.8264
17	rs35403175	66871837	A	0.03608	0.04333	G	0.6607	0.8264
1	rs61785974	28800380	A	0.08247	0.075	C	0.661	1.109

3	rs33944211	93768268	G	0.08247	0.075	C	0.661	1.109
7	rs10255061	123152019	A	0.3273	0.315	G	0.661	1.058
16	rs16959641	58054099	C	0.08247	0.075	G	0.661	1.109
18	rs10853392	77708781	A	0.3273	0.315	G	0.661	1.058
19	rs11673029	58491627	G	0.08247	0.075	A	0.661	1.109
10	rs3758490	64159333	C	0.3093	0.3225	A	0.6612	0.9406
11	rs11034037	37256343	G	0.3093	0.3225	A	0.6612	0.9406
19	rs8102086	3752874	A	0.3093	0.3225	G	0.6612	0.9406
7	rs757656	86372884	C	0.1907	0.2025	A	0.6613	0.9281
1	rs6703653	84555908	G	0.3093	0.3217	A	0.6614	0.9442
1	rs2071921	167825485	A	0.3093	0.3217	G	0.6614	0.9442
2	rs59900519	135988127	A	0.1907	0.2017	T	0.6614	0.9329
2	rs935615	135988416	A	0.1907	0.2017	G	0.6614	0.9329
3	rs9876921	44948590	G	0.134	0.125	A	0.6614	1.083
3	rs6791644	60771108	G	0.134	0.125	A	0.6614	1.083
5	rs7729273	7228047	A	0.134	0.125	G	0.6614	1.083
9	rs6415847	101533220	G	0.3093	0.3217	A	0.6614	0.9442
17	rs2586038	55916641	C	0.3093	0.3217	A	0.6614	0.9442
19	rs10775583	36017928	C	0.3093	0.3217	G	0.6614	0.9442
1	rs4233356	159753183	A	0.3299	0.3175	G	0.6616	1.058
10	rs9664184	75409967	G	0.3299	0.3175	A	0.6616	1.058
18	rs1805084	21112206	A	0.2088	0.1978	G	0.6617	1.07
12	rs997173	55355033	G	0.3119	0.325	A	0.6619	0.9412
18	rs1566199	11259601	A	0.3325	0.3192	C	0.6619	1.062
20	rs6037651	3686436	G	0.3119	0.325	A	0.6619	0.9412
1	rs4256842	107163979	G	0.2088	0.1983	A	0.6622	1.066
2	rs3770436	171822576	A	0.3325	0.32	G	0.6622	1.058
3	rs1799852	133475722	A	0.2088	0.1983	G	0.6622	1.066
2	rs6546073	64698397	A	0.3144	0.3283	G	0.6626	0.9382
5	rs1016988	131744574	G	0.3144	0.3272	A	0.6626	0.943
1	rs3748697	214820099	G	0.134	0.1258	A	0.6628	1.075
2	rs1033688	31695655	G	0.3351	0.3225	A	0.6628	1.059
6	rs9348876	31575276	A	0.134	0.1258	G	0.6628	1.075
16	rs9938333	81190598	G	0.3144	0.3267	A	0.6628	0.9454
18	rs4149601	55816791	A	0.134	0.1258	G	0.6628	1.075
1	rs1047840	242042301	A	0.1933	0.2042	G	0.663	0.934
6	rs2228391	32797773	G	0.08247	0.07583	A	0.663	1.095
7	rs2718131	138602916	G	0.08247	0.07583	C	0.663	1.095
18	rs2847281	12821593	G	0.1933	0.2042	A	0.663	0.934
1	rs2274260	74905205	A	0.04639	0.04083	G	0.6631	1.143
5	rs17208397	140798639	G	0.04639	0.04083	C	0.6631	1.143
6	rs35551087	136888782	G	0.04639	0.04083	A	0.6631	1.143
11	rs34337292	57958497	G	0.1211	0.1308	A	0.6631	0.9156

12	rs7297606	119568596	A	0.04639	0.04083	G	0.6631	1.143
15	rs78648980	48559813	A	0.04639	0.04083	G	0.6631	1.143
19	exm-IND19-5413	49447749	D	0.04639	0.04083	I	0.6631	1.143
6	rs4151657	31917540	G	0.317	0.3308	A	0.6632	0.9388
8	rs1047406	22570935	G	0.317	0.3308	A	0.6632	0.9388
9	rs2081670	92887231	A	0.1211	0.1317	G	0.6633	0.909
15	rs10775247	90126121	A	0.3376	0.325	G	0.6634	1.059
17	rs744166	40514201	G	0.3376	0.325	A	0.6634	1.059
17	rs1026916	40529835	A	0.3376	0.325	G	0.6634	1.059
21	rs928302	43810084	A	0.2113	0.2008	G	0.6636	1.066
13	rs2149215	113097722	A	0.3196	0.3333	C	0.6638	0.9394
2	rs6739713	136488978	A	0.3196	0.3325	G	0.6639	0.9429
10	rs1954343	55477195	A	0.3196	0.3325	G	0.6639	0.9429
11	rs610932	59939307	A	0.3196	0.3325	C	0.6639	0.9429
12	rs3782181	88953561	C	0.3196	0.3325	A	0.6639	0.9429
12	rs4474514	88953959	G	0.3196	0.3325	A	0.6639	0.9429
1	rs2070123	79115905	G	0.1366	0.1275	A	0.664	1.083
6	rs1632962	29683808	A	0.3376	0.3258	G	0.664	1.055
19	rs1644634	37441980	G	0.3402	0.3275	A	0.664	1.059
6	rs3117292	29652199	G	0.1959	0.2075	A	0.6643	0.9303
2	rs6715551	68035962	A	0.3222	0.3358	G	0.6644	0.94
8	rs2732014	75792274	A	0.3222	0.3358	G	0.6644	0.94
8	rs1382469	114363347	A	0.3222	0.3358	G	0.6644	0.94
2	rs3731625	24439048	G	0.07216	0.08	A	0.6645	0.8944
3	rs1034405	50597092	G	0.07216	0.08	A	0.6645	0.8944
6	rs74693071	36298059	A	0.07216	0.08	G	0.6645	0.8944
9	rs41265306	14784389	A	0.07216	0.08	G	0.6645	0.8944
9	rs8176741	136131461	A	0.2139	0.2025	G	0.6645	1.072
9	rs7853989	136131592	G	0.2139	0.2025	C	0.6645	1.072
11	rs17150243	56019925	C	0.1959	0.2067	A	0.6645	0.9351
18	rs658995	70207340	A	0.07216	0.08	G	0.6645	0.8944
3	rs2290189	13677946	A	0.07216	0.08083	G	0.6646	0.8844
10	rs7477335	21998445	C	0.3402	0.3283	A	0.6646	1.055
15	rs55712142	52536596	A	0.07216	0.08083	G	0.6646	0.8844
17	rs9972941	38867922	A	0.3428	0.33	C	0.6646	1.059
22	rs80587	36598049	C	0.07216	0.08083	G	0.6646	0.8844
22	rs132736	36598058	A	0.07216	0.08083	G	0.6646	0.8844
6	rs10004	7310259	G	0.2139	0.2033	A	0.665	1.066
17	rs1058046	42030531	G	0.3247	0.3383	C	0.665	0.9405
3	rs4682869	42923964	A	0.3428	0.3308	G	0.6651	1.055
5	rs2306764	176915143	A	0.3247	0.3375	G	0.6651	0.944
7	rs2961135	143771937	G	0.3247	0.3375	C	0.6651	0.944
13	rs279070	51133655	A	0.3247	0.3375	G	0.6651	0.944

14	rs1956533	68911554	G	0.3428	0.3308	A	0.6651	1.055
1	rs6682831	177539967	A	0.3454	0.3325	C	0.6652	1.059
4	rs1476272	72706235	G	0.3454	0.3325	A	0.6652	1.059
15	rs2178004	41991315	T	0.1366	0.1283	A	0.6653	1.075
19	rs471979	56538976	G	0.1366	0.1283	C	0.6653	1.075
1	rs7007	151735576	G	0.08505	0.0775	A	0.6656	1.106
2	rs72989577	133538651	A	0.08505	0.0775	G	0.6656	1.106
2	rs77278822	168074706	A	0.08505	0.0775	G	0.6656	1.106
3	rs76844316	112188609	C	0.08505	0.0775	A	0.6656	1.106
3	rs7427021	163761964	A	0.3273	0.3408	G	0.6656	0.9411
6	rs915664	30794617	G	0.08505	0.0775	A	0.6656	1.106
6	rs117586623	146275983	C	0.08505	0.0775	A	0.6656	1.106
8	rs7000884	15776837	G	0.3273	0.3408	A	0.6656	0.9411
11	rs679620	102713620	A	0.3273	0.3408	G	0.6656	0.9411
19	rs506425	49368843	G	0.08505	0.0775	A	0.6656	1.106
1	rs3789604	114354942	C	0.215	0.205	A	0.6657	1.062
3	rs34692573	32886446	G	0.3273	0.34	A	0.6657	0.9446
12	rs9300212	33701166	G	0.3273	0.34	A	0.6657	0.9446
3	rs9681405	142547417	A	0.3479	0.335	G	0.6658	1.059
6	rs9471577	41303995	G	0.3479	0.335	A	0.6658	1.059
6	rs4896582	142703877	G	0.1985	0.21	A	0.6658	0.9314
10	rs1004467	104594507	G	0.3479	0.335	A	0.6658	1.059
12	rs3887954	53186088	G	0.1237	0.1333	C	0.6658	0.9176
1	rs16830693	43805240	G	0.02062	0.0175	A	0.6659	1.182
1	rs200491558	110882052	A	0.02062	0.0175	G	0.6659	1.182
2	rs17521008	53955850	A	0.1237	0.1342	G	0.6659	0.9111
2	rs16866412	179474668	A	0.1237	0.1342	G	0.6659	0.9111
3	rs11554130	16364954	A	0.02062	0.0175	G	0.6659	1.182
6	rs116325082	25691352	A	0.02062	0.0175	G	0.6659	1.182
6	rs1980600	26508729	G	0.02062	0.0175	A	0.6659	1.182
6	rs62620225	28117331	A	0.02062	0.0175	G	0.6659	1.182
6	rs78556678	30673725	A	0.02062	0.0175	G	0.6659	1.182
6	rs8180688	158074592	A	0.02062	0.0175	G	0.6659	1.182
7	rs2075671	100345106	A	0.1237	0.1342	G	0.6659	0.9111
7	rs188074511	138344720	A	0.02062	0.0175	G	0.6659	1.182
9	rs200166806	20923667	A	0.02062	0.0175	G	0.6659	1.182
10	rs1245541	73849639	A	0.1237	0.1342	G	0.6659	0.9111
11	rs143001920	92901249	C	0.02062	0.0175	G	0.6659	1.182
11	rs141565726	118827987	A	0.02062	0.0175	G	0.6659	1.182
16	rs8177907	81121246	G	0.02062	0.0175	A	0.6659	1.182
16	rs8177877	81124216	G	0.02062	0.0175	A	0.6659	1.182
18	rs3819129	51887025	A	0.02062	0.0175	G	0.6659	1.182
19	rs1802498	19312496	C	0.1985	0.2108	G	0.6659	0.9267

19	rs79259738	39876735	A	0.02062	0.0175	G	0.6659	1.182
20	rs17577	44643111	A	0.1237	0.1342	G	0.6659	0.9111
22	rs5749088	30776095	A	0.1237	0.1342	G	0.6659	0.9111
5	rs185435	72348272	G	0.1985	0.2092	A	0.666	0.9361
6	rs1042127	31084170	C	0.1985	0.2092	A	0.666	0.9361
9	rs3739630	21409584	A	0.02062	0.01753	G	0.6662	1.18
12	rs2046383	30104142	C	0.3299	0.3433	A	0.6662	0.9416
6	rs2076506	22570064	A	0.3505	0.3375	G	0.6663	1.059
9	rs2383614	26068261	C	0.3505	0.3375	A	0.6663	1.059
19	rs1799782	44057574	A	0.3505	0.3375	G	0.6663	1.059
2	rs3738952	225362478	A	0.3299	0.3425	G	0.6664	0.9451
6	rs1799964	31542308	G	0.2165	0.2058	A	0.6664	1.066
19	rs268673	40901496	G	0.1392	0.13	A	0.6665	1.082
21	rs2248490	44293744	C	0.3325	0.3459	G	0.6666	0.9419
18	rs9960464	43246156	A	0.3505	0.3383	G	0.6667	1.055
7	rs13225903	130114227	A	0.3325	0.3458	G	0.6668	0.9421
11	rs7947734	1632180	A	0.3531	0.34	C	0.6669	1.06
2	rs2652423	155720940	A	0.04639	0.04167	G	0.6671	1.119
8	rs17689740	17503316	C	0.04639	0.04167	A	0.6671	1.119
10	rs76221724	85944516	A	0.04639	0.04167	C	0.6671	1.119
11	rs3741395	64597506	G	0.04639	0.04167	A	0.6671	1.119
12	rs1800974	56089357	G	0.04639	0.04167	A	0.6671	1.119
15	rs17807723	90023558	A	0.04639	0.04167	G	0.6671	1.119
17	rs12452285	63189687	A	0.04639	0.04167	G	0.6671	1.119
17	rs12937557	77078069	A	0.04639	0.04167	G	0.6671	1.119
20	rs11906160	33565755	A	0.04639	0.04167	G	0.6671	1.119
9	rs4837768	123170733	C	0.201	0.2125	G	0.6672	0.9324
3	rs7626795	190350461	G	0.2165	0.2067	A	0.6673	1.061
4	rs78280171	175898555	A	0.201	0.2133	G	0.6673	0.9278
5	rs11559	111066697	G	0.2165	0.2067	A	0.6673	1.061
6	rs3115572	32220484	C	0.201	0.2133	G	0.6673	0.9278
6	rs530160	124098943	C	0.2165	0.2067	A	0.6673	1.061
15	rs1130741	75189930	G	0.2165	0.2067	A	0.6673	1.061
17	rs9903350	76182323	A	0.2165	0.2067	C	0.6673	1.061
7	rs10266050	81297342	G	0.08505	0.07833	A	0.6675	1.094
11	rs12577167	5475118	G	0.3351	0.3475	A	0.6675	0.9461
12	rs10858945	90443482	A	0.3351	0.3475	G	0.6675	0.9461
13	rs9510738	24014194	A	0.3351	0.3475	G	0.6675	0.9461
22	rs1015939	19678655	A	0.3351	0.3475	G	0.6675	0.9461
3	rs868891	49949071	G	0.1392	0.1308	A	0.6677	1.074
3	rs6762477	50093209	G	0.1392	0.1308	A	0.6677	1.074
5	rs7728667	1225626	A	0.3557	0.3433	T	0.6677	1.056
16	rs28555129	83984776	A	0.1392	0.1308	C	0.6677	1.074

16	rs10863168	86817240	C	0.3557	0.3433	A	0.6677	1.056
19	rs12976122	9001833	A	0.3557	0.3433	G	0.6677	1.056
2	rs6714710	98345086	C	0.3582	0.345	A	0.6679	1.06
17	rs147005034	2601836	G	0.08505	0.0786	A	0.6681	1.09
20	rs2424405	21741526	A	0.3376	0.35	G	0.6681	0.9466
1	rs10799071	35049252	A	0.3582	0.3458	G	0.6682	1.056
6	rs3132671	30178287	A	0.3582	0.3458	G	0.6682	1.056
9	rs7861108	86826872	C	0.3582	0.3458	A	0.6682	1.056
2	rs6545397	54615526	A	0.3402	0.3533	G	0.6684	0.9437
2	rs7567687	130034326	A	0.1263	0.1358	G	0.6684	0.9196
3	rs200886927	125725272	A	0.1263	0.1358	T	0.6684	0.9196
4	rs7441547	59838120	G	0.3402	0.3533	A	0.6684	0.9437
8	rs7838433	110007151	A	0.3402	0.3533	C	0.6684	0.9437
10	rs6585827	124165615	G	0.3608	0.3475	A	0.6684	1.06
1	rs2296308	95709939	A	0.2036	0.2158	C	0.6686	0.9289
3	rs939885	195955762	G	0.2036	0.215	A	0.6686	0.9335
8	rs9314485	3178721	A	0.2191	0.2092	G	0.6686	1.061
11	rs948445	67414492	G	0.2036	0.215	A	0.6686	0.9335
15	rs2306277	33357262	G	0.2036	0.2158	A	0.6686	0.9289
19	rs484870	7569282	G	0.2191	0.2092	A	0.6686	1.061
19	rs1133378	7573287	A	0.2191	0.2092	G	0.6686	1.061
6	rs2571400	29927722	G	0.3608	0.3483	C	0.6687	1.056
6	rs1796743	89888744	G	0.3608	0.3483	A	0.6687	1.056
6	rs9352000	74161801	C	0.03866	0.045	A	0.6689	0.8534
7	rs3824004	127253551	A	0.03866	0.045	C	0.6689	0.8534
9	rs41274041	34257095	A	0.03866	0.045	G	0.6689	0.8534
10	rs10509680	96734339	A	0.03866	0.045	C	0.6689	0.8534
10	rs1057910	96741053	C	0.03866	0.045	A	0.6689	0.8534
11	rs10893355	125117012	G	0.3634	0.35	A	0.6689	1.06
14	rs11158493	63953522	A	0.3634	0.35	G	0.6689	1.06
8	rs208753	17814915	A	0.3428	0.3558	G	0.669	0.9442
8	rs7827153	19543862	A	0.3428	0.3558	G	0.669	0.9442
17	rs10853136	28406018	A	0.3428	0.3558	G	0.669	0.9442
1	rs2072036	175105996	A	0.2216	0.2108	G	0.6691	1.066
3	rs9871143	97983981	A	0.07474	0.0825	G	0.6691	0.8984
3	rs1515596	174298999	A	0.2216	0.2108	G	0.6691	1.066
4	rs12510688	89311962	G	0.07474	0.0825	A	0.6691	0.8984
12	rs2302685	12301898	G	0.07474	0.0825	A	0.6691	0.8984
5	rs3733905	96219561	A	0.07474	0.08333	G	0.6692	0.8886
6	rs3798220	160961137	G	0.07474	0.08333	A	0.6692	0.8886
7	rs2330425	53662097	G	0.3743	0.3608	A	0.6692	1.06
11	rs61552	86920178	G	0.07474	0.08333	A	0.6692	0.8886
13	rs2987346	29600415	G	0.07474	0.08333	A	0.6692	0.8886

17	rs9897794	28296327	A	0.3428	0.355	C	0.6693	0.9476
7	rs1155597	126613163	C	0.3454	0.3592	A	0.6694	0.9413
8	rs2843744	38025175	A	0.3454	0.3592	G	0.6694	0.9413
11	rs6589444	98254968	A	0.3454	0.3592	G	0.6694	0.9413
16	rs13335375	16018589	A	0.03927	0.04583	G	0.6694	0.8509
2	rs4662742	128350744	A	0.3454	0.3583	C	0.6695	0.9447
1	rs34305100	7913029	G	0.03866	0.04583	A	0.6696	0.8372
1	rs13306061	7913445	A	0.03866	0.04583	G	0.6696	0.8372
2	rs792061	5523127	G	0.03866	0.04583	A	0.6696	0.8372
6	rs9469031	31595795	A	0.03866	0.04583	G	0.6696	0.8372
6	rs9469057	31779728	C	0.03866	0.04583	G	0.6696	0.8372
8	rs57918843	23117740	A	0.03866	0.04583	G	0.6696	0.8372
11	rs79183202	644568	C	0.03866	0.04583	G	0.6696	0.8372
1	rs2246514	61987970	G	0.366	0.3533	A	0.6697	1.056
2	rs355910	165647332	A	0.366	0.3533	G	0.6697	1.056
11	rs3919907	56000600	A	0.366	0.3533	C	0.6697	1.056
21	rs459094	40007704	C	0.366	0.3533	A	0.6697	1.056
2	rs17027258	103091540	G	0.3634	0.3517	A	0.6698	1.052
11	rs211114	18001888	A	0.3634	0.3517	G	0.6698	1.052
18	rs1111207	24125857	A	0.3634	0.3517	G	0.6698	1.052
7	rs40143	111732556	A	0.3479	0.3617	G	0.6699	0.9418
12	rs2041387	6562823	A	0.3479	0.3617	G	0.6699	0.9418
15	rs1564782	74622678	A	0.3479	0.3617	G	0.6699	0.9418
5	rs10065401	116752778	A	0.3479	0.3606	C	0.67	0.9461
8	rs13263632	17942204	A	0.08763	0.08	G	0.67	1.105
12	rs2041385	6562293	A	0.3479	0.3608	G	0.67	0.9452
15	rs3826007	80263217	A	0.2062	0.2183	G	0.67	0.9299
3	rs13080353	140875814	A	0.3686	0.3558	G	0.6701	1.057
11	rs459309	72335834	A	0.3586	0.3717	G	0.6702	0.9453
16	rs36117280	1250389	G	0.04897	0.04293	A	0.6702	1.148
3	rs2197896	30032530	G	0.3505	0.3642	A	0.6704	0.9423
6	rs3828923	29590715	A	0.08854	0.08097	G	0.6704	1.103
9	rs867977	107056847	A	0.2242	0.2133	G	0.6704	1.066
17	rs4796856	79899272	G	0.3505	0.3642	A	0.6704	0.9423
18	rs2298624	47429022	A	0.2242	0.2133	G	0.6704	1.066
3	rs3732675	12858028	G	0.3711	0.3583	A	0.6706	1.057
11	rs11032025	32874926	A	0.3686	0.3567	G	0.6706	1.053
22	rs738322	38569006	G	0.3711	0.3583	A	0.6706	1.057
17	rs4790404	2886642	G	0.3711	0.3589	A	0.6708	1.054
17	rs2242373	8700982	A	0.1295	0.1394	G	0.6708	0.9187
3	rs9859538	151090963	A	0.1289	0.1383	G	0.6709	0.9214
4	rs11248060	964359	A	0.1289	0.1383	G	0.6709	0.9214
6	rs2073260	153296541	C	0.3531	0.3667	G	0.6709	0.9428

7	rs1404918	110236649	G	0.3531	0.3667	A	0.6709	0.9428
15	rs1898882	40655873	G	0.1289	0.1392	C	0.6709	0.915
1	rs7513275	3511934	G	0.3737	0.3608	A	0.671	1.057
2	rs60719900	85299783	C	0.3737	0.3608	A	0.671	1.057
2	rs2108215	159446818	C	0.3531	0.3658	A	0.671	0.9462
1	rs946448	54661150	A	0.2088	0.22	G	0.6713	0.9354
1	rs17121464	100340787	A	0.2088	0.2208	G	0.6713	0.9309
6	rs9503893	4031998	A	0.2088	0.2208	G	0.6713	0.9309
11	rs4936880	123886984	G	0.3783	0.3922	A	0.6713	0.9431
14	rs1950902	64882380	A	0.2088	0.2208	G	0.6713	0.9309
15	rs3743200	79274046	A	0.2088	0.22	G	0.6713	0.9354
17	rs2748427	76121864	G	0.2088	0.2208	A	0.6713	0.9309
10	rs597371	116032519	G	0.3557	0.3692	A	0.6714	0.9433
12	rs2014355	121175524	G	0.1443	0.135	A	0.6714	1.081
12	rs1799958	121176083	A	0.1443	0.135	G	0.6714	1.081
11	rs10834648	3645345	G	0.3737	0.3617	A	0.6715	1.053
12	rs7296288	49479968	C	0.3557	0.3683	A	0.6715	0.9466
1	rs141587551	85666163	A	0.04897	0.04333	C	0.6717	1.137
1	rs653737	236729956	G	0.04897	0.04333	A	0.6717	1.137
13	rs9579717	20425911	A	0.04897	0.04333	C	0.6717	1.137
1	rs6662276	19166893	A	0.08763	0.08083	G	0.6718	1.092
1	rs3811448	154516578	A	0.08763	0.08083	G	0.6718	1.092
7	rs2302427	148525904	C	0.08763	0.08083	G	0.6718	1.092
10	rs4462262	59189178	A	0.08763	0.08083	G	0.6718	1.092
12	rs10783299	49390677	G	0.3582	0.3717	A	0.6718	0.9437
16	rs8060686	67911517	G	0.08763	0.08083	A	0.6718	1.092
20	rs6020856	49543293	A	0.3789	0.3656	G	0.6718	1.058
11	rs11214677	113477081	G	0.3789	0.3658	A	0.6719	1.057
12	rs12230214	9346792	G	0.3789	0.3658	C	0.6719	1.057
1	rs28563947	188755011	A	0.3582	0.3708	G	0.672	0.9471
7	rs117362932	149513141	A	0.08763	0.08097	G	0.672	1.09
1	rs9803851	102159783	A	0.01546	0.02	G	0.6722	0.7696
1	rs139614117	212798251	A	0.01546	0.02	G	0.6722	0.7696
2	rs117201430	79253943	A	0.01546	0.02	T	0.6722	0.7696
5	rs79561592	133997171	A	0.01546	0.02	G	0.6722	0.7696
6	rs141430703	33154514	A	0.01546	0.02	C	0.6722	0.7696
7	rs150148096	138758754	G	0.01546	0.02	A	0.6722	0.7696
8	rs143689645	17132243	A	0.01546	0.02	G	0.6722	0.7696
9	rs77248992	95772666	C	0.01546	0.02	A	0.6722	0.7696
9	rs117068327	95846980	A	0.01546	0.02	G	0.6722	0.7696
9	rs146828540	118997457	A	0.01546	0.02	G	0.6722	0.7696
11	rs149918157	114576683	G	0.01546	0.02	A	0.6722	0.7696
12	rs78527125	88547215	C	0.01546	0.02	G	0.6722	0.7696

15	rs118138806	89834846	C	0.01546	0.02	A	0.6722	0.7696
2	rs11680012	238672425	C	0.04897	0.04355	G	0.6723	1.131
14	rs1480415	85429403	G	0.3814	0.3683	A	0.6723	1.058
17	rs2159132	14005439	A	0.3608	0.3742	G	0.6723	0.9442
19	rs2905426	19478022	C	0.3814	0.3683	A	0.6723	1.058
19	rs575756	56694505	A	0.3814	0.3683	G	0.6723	1.058
1	rs10752838	181116023	G	0.3608	0.3733	A	0.6725	0.9476
5	rs2271704	41008780	A	0.3608	0.3733	G	0.6725	0.9476
6	rs1053924	32120715	A	0.3608	0.3733	G	0.6725	0.9476
11	rs7107309	128272676	G	0.1443	0.1358	A	0.6725	1.073
14	rs3759601	96777468	C	0.2113	0.2233	G	0.6726	0.9319
15	rs2277598	73027478	G	0.2113	0.2225	A	0.6726	0.9364
22	rs34074034	43568512	A	0.2113	0.2233	G	0.6726	0.9319
1	rs11264248	154688197	G	0.3814	0.3692	A	0.6727	1.054
6	rs9460678	21789088	G	0.3814	0.3692	A	0.6727	1.054
17	rs2280777	18041507	G	0.384	0.3706	A	0.6727	1.059
16	rs9924371	81253759	G	0.384	0.3708	A	0.6728	1.058
2	rs10931468	191538562	A	0.384	0.3717	C	0.6731	1.054
2	rs7595229	202799297	G	0.366	0.3798	A	0.6731	0.9426
3	rs1675943	194387300	A	0.366	0.3792	G	0.6731	0.9451
11	rs2405657	97682564	G	0.384	0.3717	A	0.6731	1.054
12	rs10845159	7585971	G	0.366	0.3792	A	0.6731	0.9451
13	rs2297225	19751127	A	0.01562	0.0209	G	0.6731	0.7435
15	rs149457868	63958229	A	0.01554	0.02083	G	0.6731	0.7421
18	rs10503091	62013349	A	0.384	0.3717	G	0.6731	1.054
2	rs6749634	119816228	A	0.3866	0.3733	G	0.6732	1.058
8	rs147319341	11301733	A	0.01554	0.02087	G	0.6732	0.7408
1	rs2295815	235324298	G	0.01546	0.02083	A	0.6733	0.7382
2	rs6547829	28323869	A	0.1314	0.1408	G	0.6733	0.9232
2	rs4670766	37940542	G	0.01546	0.02083	A	0.6733	0.7382
2	rs200406810	219259361	A	0.01546	0.02083	G	0.6733	0.7382
3	rs147838565	5244678	A	0.01546	0.02083	G	0.6733	0.7382
5	rs2233295	150429452	A	0.01546	0.02083	G	0.6733	0.7382
6	rs2524172	30433611	A	0.01546	0.02083	G	0.6733	0.7382
7	rs145970530	141673074	G	0.01546	0.02083	A	0.6733	0.7382
8	rs117235442	22675244	C	0.01546	0.02083	G	0.6733	0.7382
9	rs3824420	712766	A	0.1314	0.1408	G	0.6733	0.9232
12	rs200369965	4461796	A	0.01546	0.02083	G	0.6733	0.7382
12	rs150678135	75900262	G	0.01546	0.02083	A	0.6733	0.7382
14	rs17782124	74203789	A	0.1314	0.1417	G	0.6733	0.9169
17	rs149758866	81050913	A	0.01546	0.02083	G	0.6733	0.7382
19	rs150155929	11943022	G	0.01546	0.02083	A	0.6733	0.7382
19	rs202170563	12989289	A	0.01546	0.02083	G	0.6733	0.7382

20	rs4911320	31914031	A	0.1314	0.1417	C	0.6733	0.9169
2	rs3731749	179406191	A	0.366	0.3783	G	0.6734	0.9485
11	rs476822	55305406	A	0.366	0.3783	C	0.6734	0.9485
19	rs1043996	15295134	G	0.366	0.3783	A	0.6734	0.9485
22	rs12171042	32011225	G	0.366	0.3783	A	0.6734	0.9485
4	rs77293186	148559892	A	0.07732	0.08583	G	0.6735	0.8925
9	rs10818854	126446778	A	0.07732	0.08583	G	0.6735	0.8925
15	rs3784609	60910550	A	0.07732	0.08583	G	0.6735	0.8925
20	rs2233903	43851108	A	0.07732	0.08583	G	0.6735	0.8925
20	rs2071650	43851375	C	0.07732	0.08583	G	0.6735	0.8925
3	rs3732477	194062519	A	0.3892	0.3758	G	0.6736	1.058
4	rs2074380	113353311	A	0.07732	0.085	G	0.6736	0.9021
6	rs1541166	105054947	A	0.3892	0.3758	G	0.6736	1.058
22	rs9610645	22288560	A	0.07732	0.085	G	0.6736	0.9021
6	rs932316	25641200	G	0.1469	0.1375	A	0.6737	1.08
15	rs1135911	101924546	A	0.1469	0.1375	G	0.6737	1.08
3	rs11711157	194061826	A	0.3892	0.3767	G	0.6738	1.054
5	rs745105	20606497	G	0.3686	0.3808	A	0.6738	0.9489
8	rs2591002	69347872	A	0.3686	0.3808	G	0.6738	0.9489
16	rs8060294	81248745	G	0.3892	0.3767	A	0.6738	1.054
20	rs158551	52645166	G	0.3892	0.3767	A	0.6738	1.054
6	rs404890	32198867	A	0.3756	0.3892	C	0.674	0.9444
9	rs6477694	111932342	A	0.3711	0.3842	G	0.674	0.9461
10	rs2487068	73121913	A	0.3711	0.3842	G	0.674	0.9461
11	rs7932813	7708281	G	0.232	0.2208	A	0.6741	1.066
11	rs1939875	95422867	A	0.232	0.2208	G	0.6741	1.066
1	rs3013105	13802325	A	0.3918	0.3792	G	0.6742	1.055
2	rs11885653	34200798	G	0.3918	0.3792	A	0.6742	1.055
4	rs7664927	114058373	A	0.3918	0.3792	G	0.6742	1.055
6	rs375912	33016728	G	0.3918	0.3792	A	0.6742	1.055
7	rs2715148	82450035	A	0.09021	0.0825	C	0.6743	1.103
8	rs750835	5981454	G	0.3737	0.3875	A	0.6743	0.9432
12	rs17375215	12483764	A	0.09021	0.0825	G	0.6743	1.103
12	rs35332676	96641016	A	0.09021	0.0825	G	0.6743	1.103
16	rs7196505	34516075	A	0.3711	0.3833	G	0.6743	0.9494
2	rs3905317	135744007	G	0.3737	0.3867	A	0.6744	0.9465
2	rs1112542	135744416	G	0.3737	0.3867	C	0.6744	0.9465
1	rs1353428	147326222	C	0.3918	0.38	A	0.6747	1.051
1	rs1016610	163781066	A	0.3918	0.38	G	0.6747	1.051
3	rs3828419	164906806	G	0.1469	0.1383	A	0.6747	1.073
6	rs9500864	30693232	A	0.1469	0.1383	G	0.6747	1.073
6	rs4148876	32796793	A	0.1469	0.1383	G	0.6747	1.073
8	rs12541801	39611992	C	0.3763	0.39	A	0.6747	0.9436

14	rs12890229	86268812	C	0.3918	0.38	A	0.6747	1.051
15	rs12594307	91449702	A	0.3918	0.38	T	0.6747	1.051
1	rs2378454	221396325	A	0.232	0.2217	G	0.6748	1.06
2	rs2114582	179287834	A	0.3763	0.3892	G	0.6748	0.9469
5	rs7709552	71674855	G	0.3763	0.389	A	0.6748	0.9477
17	rs1399062	6285864	A	0.3943	0.3825	G	0.675	1.051
18	rs7505747	3111569	C	0.3943	0.3825	A	0.675	1.051
3	rs2641776	195515617	C	0.2165	0.2283	G	0.6751	0.9338
3	rs2177336	195516878	A	0.2165	0.2283	G	0.6751	0.9338
3	rs1104760	195517321	G	0.2165	0.2275	A	0.6752	0.9383
3	rs882605	195517553	A	0.2165	0.2275	C	0.6752	0.9383
4	rs7683365	144920596	A	0.04897	0.04417	G	0.6752	1.114
9	rs1411272	125316350	G	0.3995	0.3867	A	0.6752	1.055
9	rs28756857	140960567	C	0.2165	0.2275	A	0.6752	0.9383
10	rs2281636	101492403	C	0.3789	0.3917	A	0.6752	0.9474
16	rs9927912	56576763	G	0.3995	0.3867	A	0.6752	1.055
19	rs3813148	54965860	A	0.3995	0.3867	G	0.6752	1.055
22	rs3804097	41601385	G	0.04897	0.04417	A	0.6752	1.114
6	rs9352009	63389189	G	0.3969	0.385	A	0.6753	1.051
7	rs848016	12428803	G	0.3969	0.385	A	0.6753	1.051
12	rs17125266	53671005	A	0.2345	0.2233	G	0.6754	1.066
15	rs4321143	96156368	G	0.2345	0.2233	A	0.6754	1.066
5	rs4270726	91093966	A	0.4021	0.389	G	0.6755	1.056
1	rs872222	16344664	G	0.3814	0.3942	A	0.6756	0.9478
6	rs11754288	25776949	A	0.134	0.1442	G	0.6756	0.9187
12	rs6582298	76114872	G	0.134	0.1442	A	0.6756	0.9187
14	rs1254319	60903757	G	0.3814	0.3942	A	0.6756	0.9478
15	rs11630435	46850179	C	0.4021	0.3892	A	0.6756	1.055
7	rs17154155	80234243	C	0.3995	0.3875	A	0.6757	1.052
12	rs4964527	107563247	G	0.3995	0.3875	A	0.6757	1.052
20	rs6024653	54751363	A	0.3995	0.3875	G	0.6757	1.052
6	rs4713243	29720961	C	0.4046	0.3917	G	0.6759	1.056
14	rs3809429	44975606	A	0.384	0.3967	C	0.6759	0.9482
1	rs2230625	12186058	G	0.1495	0.14	A	0.676	1.08
11	rs1129195	64882789	A	0.2345	0.2242	G	0.676	1.06
19	rs12973901	19823270	T	0.2345	0.2242	A	0.676	1.06
4	rs7664076	172388728	A	0.4046	0.3923	G	0.6761	1.053
8	rs1460163	80227448	A	0.3866	0.4	G	0.6761	0.9454
4	rs13328024	16427871	A	0.4072	0.3942	G	0.6762	1.056
11	rs9667193	71524861	G	0.3866	0.399	A	0.6763	0.9493
16	rs714181	3640274	A	0.09021	0.08361	G	0.6764	1.087
1	rs4653328	38227086	T	0.4098	0.3967	A	0.6765	1.056
1	rs3128658	225273248	C	0.2371	0.2258	A	0.6765	1.065

2	rs2273664	32983480	A	0.2191	0.23	G	0.6765	0.9392
6	rs6904669	31192796	G	0.4093	0.3975	A	0.6765	1.05
7	rs7798471	6744957	G	0.2371	0.2258	A	0.6765	1.065
14	rs4981088	20665840	G	0.3892	0.4025	A	0.6765	0.9458
4	rs180730	121801790	A	0.4072	0.395	G	0.6766	1.052
8	rs1049874	17927327	G	0.4072	0.395	A	0.6766	1.052
11	rs7122648	21011821	A	0.3892	0.4017	G	0.6767	0.9491
12	rs7298692	64850050	A	0.3892	0.4017	G	0.6767	0.9491
12	rs1809889	124801226	A	0.3892	0.4017	G	0.6767	0.9491
4	rs2018601	136694463	G	0.3918	0.405	A	0.6768	0.9462
19	rs79186164	53454475	A	0.3918	0.405	G	0.6768	0.9462
6	rs2073214	144081609	A	0.1495	0.1408	G	0.6769	1.072
8	rs2748421	142517222	G	0.4098	0.3975	A	0.6769	1.052
8	rs2613648	142517227	A	0.4098	0.3975	G	0.6769	1.052
14	rs11625112	23596740	A	0.4124	0.3992	G	0.6769	1.056
17	rs3809806	3628362	G	0.4098	0.3975	A	0.6769	1.052
1	rs10789165	65186794	A	0.3918	0.4042	G	0.677	0.9495
10	rs10762420	72813306	G	0.3918	0.4042	A	0.677	0.9495
11	rs757110	17418477	C	0.3918	0.4042	A	0.677	0.9495
2	rs62168710	138716177	G	0.3943	0.4075	A	0.6771	0.9466
6	rs116938859	30994232	G	0.04167	0.04762	A	0.6771	0.8696
11	rs9326246	116611733	G	0.2371	0.2267	C	0.6771	1.06
13	rs7329174	41558110	G	0.2371	0.2267	A	0.6771	1.06
15	rs3759917	92935447	A	0.4124	0.4	C	0.6771	1.053
17	rs605059	40706906	A	0.4149	0.4015	G	0.6771	1.057
2	rs10211596	233699415	G	0.4149	0.4017	A	0.6772	1.057
16	rs7193248	27113695	G	0.4149	0.4017	A	0.6772	1.057
3	rs879634	150128392	A	0.04124	0.0475	G	0.6773	0.8625
12	rs34837068	42863262	G	0.04124	0.0475	A	0.6773	0.8625
15	rs4598860	93616975	A	0.04124	0.0475	G	0.6773	0.8625
6	rs6927706	138539315	G	0.4149	0.4025	A	0.6774	1.053
10	rs10732921	3097212	G	0.4175	0.4042	A	0.6774	1.057
1	rs117146839	12089946	G	0.0232	0.01917	C	0.6775	1.215
1	rs74797667	12090154	A	0.0232	0.01917	G	0.6775	1.215
1	rs3737573	76253229	T	0.0232	0.01917	A	0.6775	1.215
2	rs77932609	131487698	A	0.0232	0.01917	G	0.6775	1.215
2	rs958672	155070567	A	0.0232	0.01917	T	0.6775	1.215
2	rs34883828	179770175	A	0.0232	0.01917	C	0.6775	1.215
2	rs16864976	224463820	G	0.0232	0.01917	A	0.6775	1.215
3	rs143322256	43618547	G	0.0232	0.01917	A	0.6775	1.215
4	rs79272852	3319261	A	0.0232	0.01917	G	0.6775	1.215
5	rs10440635	40490790	A	0.2216	0.2333	G	0.6775	0.9357
5	rs1800041	63257500	A	0.0232	0.01917	G	0.6775	1.215

5	rs139687157	170692818	T	0.0232	0.01917	A	0.6775	1.215
6	rs6926101	133105120	A	0.0232	0.01917	G	0.6775	1.215
10	rs143274747	32575715	G	0.0232	0.01917	A	0.6775	1.215
10	rs61751502	99019287	A	0.0232	0.01917	G	0.6775	1.215
10	rs79461687	115366006	A	0.0232	0.01917	C	0.6775	1.215
12	rs76879660	26218163	A	0.0232	0.01917	G	0.6775	1.215
17	rs185670819	37864776	A	0.0232	0.01917	G	0.6775	1.215
17	rs143784778	80895231	G	0.0232	0.01917	C	0.6775	1.215
19	rs3810110	52004743	A	0.3969	0.41	G	0.6775	0.9471
20	rs3746500	44511533	A	0.0232	0.01917	G	0.6775	1.215
1	rs16832015	160421916	A	0.0799	0.08833	G	0.6777	0.8962
3	rs6599077	40096618	A	0.3969	0.4092	G	0.6777	0.9503
5	rs61998228	79286419	A	0.0799	0.08833	G	0.6777	0.8962
7	rs112305062	6547902	A	0.0799	0.08833	G	0.6777	0.8962
9	rs56350726	86900369	A	0.0799	0.08833	T	0.6777	0.8962
9	rs921122	95063947	A	0.04145	0.04833	G	0.6777	0.8514
9	rs61742574	95077470	T	0.04145	0.04833	A	0.6777	0.8514
14	rs10483251	21671277	A	0.0799	0.08833	C	0.6777	0.8962
14	rs946072	84457393	G	0.4201	0.4067	A	0.6777	1.057
2	rs74531854	47604176	A	0.04124	0.04833	G	0.6778	0.8469
3	rs7631605	37234589	A	0.0799	0.0875	G	0.6778	0.9056
3	rs74970181	40558150	C	0.04124	0.04841	G	0.6778	0.8454
3	rs819085	130174334	G	0.04124	0.04833	A	0.6778	0.8469
4	rs4693909	88837705	G	0.3995	0.4125	A	0.6778	0.9475
5	rs4546421	117239454	G	0.3995	0.4125	A	0.6778	0.9475
5	rs2961646	136608159	C	0.3995	0.4125	A	0.6778	0.9475
8	rs10504740	82427790	A	0.04124	0.04833	G	0.6778	0.8469
8	rs6980476	87519315	C	0.04124	0.04833	A	0.6778	0.8469
11	rs341047	72301503	A	0.0799	0.0875	G	0.6778	0.9056
15	rs3816988	63111739	G	0.0799	0.0875	A	0.6778	0.9056
17	rs73350377	74273299	A	0.04124	0.04833	G	0.6778	0.8469
19	rs78607249	16630994	C	0.0799	0.0875	A	0.6778	0.9056
22	rs76273991	31337950	G	0.04124	0.04833	A	0.6778	0.8469
5	rs245201	127169212	G	0.4021	0.4157	A	0.6779	0.9452
6	rs2735079	29938932	A	0.4021	0.4156	G	0.6779	0.9457
16	rs2290895	88779132	A	0.04124	0.04849	G	0.6779	0.8439
6	rs2437092	129716209	G	0.4021	0.4158	A	0.678	0.9446
3	rs2291861	4842488	A	0.3995	0.4117	C	0.6781	0.9507
11	rs597626	66316557	A	0.4021	0.415	G	0.6781	0.9479
18	rs11660213	42481985	G	0.4021	0.415	A	0.6781	0.9479
1	rs3818831	152331240	A	0.4227	0.41	G	0.6782	1.054
5	rs469727	112242968	A	0.1521	0.1425	G	0.6782	1.079
6	rs4946578	121895061	A	0.4227	0.41	G	0.6782	1.054

6	rs9331348	158041511	C	0.1521	0.1425	A	0.6782	1.079
9	rs869457	133924451	A	0.2397	0.2292	G	0.6782	1.06
21	rs3746887	41032740	A	0.4227	0.41	G	0.6782	1.054
1	rs6681719	112538743	G	0.4046	0.4183	A	0.6783	0.945
3	rs6773957	186573705	G	0.4046	0.4183	A	0.6783	0.945
6	rs3094574	29422092	A	0.4046	0.4183	G	0.6783	0.945
2	rs1198849	10908893	A	0.4046	0.4175	G	0.6784	0.9483
6	rs3128854	29431698	A	0.4046	0.4175	G	0.6784	0.9483
6	rs435766	29939852	A	0.4046	0.4175	G	0.6784	0.9483
8	rs906998	78530715	G	0.4046	0.4175	A	0.6784	0.9483
18	rs17674580	43309911	A	0.09278	0.085	G	0.6784	1.101
20	rs1555300	43430490	G	0.4021	0.4142	A	0.6784	0.9511
1	rs12568784	152323132	A	0.4253	0.4125	C	0.6785	1.054
2	rs1865257	47259467	C	0.4253	0.4125	A	0.6785	1.054
9	rs62579874	139144609	C	0.4278	0.4147	A	0.6785	1.055
6	rs1534443	144869785	A	0.4072	0.4208	G	0.6786	0.9454
4	rs12513325	180802775	A	0.4227	0.4108	C	0.6787	1.05
20	rs2427158	60091406	G	0.4227	0.4108	A	0.6787	1.05
20	rs2039369	62253821	G	0.4072	0.42	A	0.6787	0.9487
1	rs2282303	152329435	A	0.4253	0.4133	G	0.6789	1.05
3	rs2049330	121633904	G	0.2242	0.235	A	0.6789	0.9409
3	rs2257212	121643804	G	0.2242	0.235	A	0.6789	0.9409
3	rs1143671	121647286	G	0.2242	0.235	A	0.6789	0.9409
3	rs1143672	121648168	G	0.2242	0.235	A	0.6789	0.9409
4	rs4639059	27216501	G	0.4098	0.4225	A	0.6789	0.949
[truncated: 464,554 more chars]
